# Genomic insights into mite phylogeny, fitness, development, and reproduction

**DOI:** 10.1186/s12864-019-6281-1

**Published:** 2019-12-09

**Authors:** Yan-Xuan Zhang, Xia Chen, Jie-Ping Wang, Zhi-Qiang Zhang, Hui Wei, Hai-Yan Yu, Hong-Kun Zheng, Yong Chen, Li-Sheng Zhang, Jian-Zhen Lin, Li Sun, Dong-Yuan Liu, Juan Tang, Yan Lei, Xu-Ming Li, Min Liu

**Affiliations:** 1 Research Center of Engineering and Technology of Natural Enemy Resource of Crop Pest in Fujian, Institute of Plant Protection, Fujian Academy of Agricultural Sciences, Fuzhou, 350003 People’s Republic of China; 2 Agricultural Bio-Resources Research Institute, Fujian Academy of Agricultural Sciences, Fuzhou, 350013 People’s Republic of China; 3 Landcare Research, Auckland and School of Biological Sciences, The University of Auckland, Auckland, New Zealand; 4 Biomarker Technologies Corporation, Beijing, 101300 People’s Republic of China; 5 Institute of Plant Protection, Chinese Academy of Agricultural Sciences, Beijing, 100193 People’s Republic of China; 6 Fujian Yanxuan Bio-preventing and Technology Biocontrol Corporation, Fuzhou, People’s Republic of China

**Keywords:** Genome, Acari, Ecology, Development, Feeding, Sex, Evolution

## Abstract

**Background:**

Predatory mites (Acari: Phytoseiidae) are the most important beneficial arthropods used in augmentative biological pest control of protected crops around the world. However, the genomes of mites are far less well understood than those of insects and the evolutionary relationships among mite and other chelicerate orders are contested, with the enigmatic origin of mites at one of the centres in discussion of the evolution of Arachnida.

**Results:**

We here report the 173 Mb nuclear genome (from 51.75 Gb pairs of Illumina reads) of the predatory mite, *Neoseiulus cucumeris*, a biocontrol agent against pests such as mites and thrips worldwide. We identified nearly 20.6 Mb (~ 11.93% of this genome) of repetitive sequences and annotated 18,735 protein-coding genes (a typical gene 2888 bp in size); the total length of protein-coding genes was about 50.55 Mb (29.2% of this assembly). About 37% (6981) of the genes are unique to *N. cucumeris* based on comparison with other arachnid genomes. Our phylogenomic analysis supported the monophyly of Acari, therefore rejecting the biphyletic origin of mites advocated by other studies based on limited gene fragments or few taxa in recent years. Our transcriptomic analyses of different life stages of *N. cucumeris* provide new insights into genes involved in its development. Putative genes involved in vitellogenesis, regulation of oviposition, sex determination, development of legs, signal perception, detoxification and stress-resistance, and innate immune systems are identified.

**Conclusions:**

Our genomics and developmental transcriptomics analyses of *N. cucumeris* provide invaluable resources for further research on the development, reproduction, and fitness of this economically important mite in particular and Arachnida in general.

## Background

Arthropods, with more than 1.3 million named species, are the most successful animals on the planet [1]. Among the two major ancient linages of arthropods, insects dominate the Madibulata, whereas mites or Acari (Arachnida) dominate the Chelicerata [2]. Mites, including predators, animal parasites and hitchhikers, plant-eaters and fungal-feeders, and saprophytes with various modes of reproduction and genetic systems, have invaded all major habitats, including the deep ocean where insects have failed to conquer [3–5]. With nearly 60,000 named species and an estimated total of half a million [5, 6] to 10.2 million species [7], mites are far less well understood than insects. While the phylogeny of insects and allies of the Madibulata are relatively well known [8], the evolutionary relationships of the chelicerate orders are contested, with the enigmatic origin of mites at one of the centers in discussion of the evolution of Arachnida [9, 10].

Mites consist of two major lineages: the Acariformes or Actinotrichida (known from Devonian), and the Parasitiformes or Anactinotrichida (known from Cretaceous) [3, 5, 9, 11]. The Acari has been traditionally recognized as a monophyletic group [4, 12–15], characterized primarily by the presence of a gnathosoma, an idiosoma with reduced segmentation and six-legged larvae. The monophyly of Acari was supported by phylogentic studies [3, 16–19], but diphyletic or even polyphyletic origins of Acari are suggested as early as the 1930s [20, 21], revived by Russian arachnologists Zakhvatkin [22] and Dubinin [23, 24] in the mid-1900s, further developed by van der Hammen [25–27] in the 1970–1980s, and accepted by some general texts on arthropod phylogeny in the late-1900 s [28]. In the last two decades, the diphyly of Acari received increased support from morphological studies [11, 29] and molecular data [10, 30–38]. Recent genomic data also reject the monophyly of Acari, placing Parasitiformes closer to spiders than to other real mites (i.e. Acariformes) [ 39, 40], or placing Acariformes closer to Pseudoscopiones than to Parasitiformes [41], although the number of arachinid taxa included in both analyses is very small (only two, four and five mite species, respectively). However, this result was refuted by another comparative genomic analysis of chemosensory genes, which included only three mite species [42]. A more recent phylogemomic study using targeted ultraconserved genomic elements from 13 species of Acari also showed a non-monophyletic Acari [43].

Predatory mites are the most important beneficial arthropods used in augmentative biological pest control in protected crops around the world [44, 45]. *Neoseiulus cucumeris* (Mesostigmata: Phytoseiidae) has become the most widely used predatory mite due to its use on a large scale in orchards and field crops in China during the last decade [46]. Compared with more oligophagous *Metaseiulus* (= *Galendromus* or *Typhlodromus*) *occidentalis* [39], *N. cucumeris* feeds on a much wider variety of food (various species of mites, thrips, psyllids and pollen) and has been employed in biocontrol against more pest groups in a greater number of crops and climatic zones [40, 46, 47]. It is therefore exposed to manifold stresses, including toxic endogenous compounds and xenobiotics, starvation, and oxidative and thermal stress. Compared with insects, only a few arachnid genomes have been sequenced. Here we present the genomic analyses of the 173 Mb nuclear genome of an important biocontrol agent. We performed a phylogenomic analysis of known arachnid genomic sequences to test the monophyly of Acari. We also conducted transcriptomic analysis of different life stages of *N. cucumeris* to examine the genes involved in its development. We examined the putative genes involved in its development and reproduction to understand their roles in vitellogenesis, regulation of oviposition, sex determination, development of legs, signal perception, detoxification and stress-resistance, and innate immune systems.

## Results

### Assembly, annotation and content of the *N. cucumeris* genome

#### Sequencing

We isolated approximately 40,000 eggs to acquire sufficient genomic DNA for constructing 12 sequencing libraries (3 paired-end libraries with the insert fragment length from 180 bp to 500 bp, and 9 mate-pair libraries from 2 kb to 15 kb) (Additional file 1: Table S1). We used eggs because our initial analysis showed that the genomic DNA isolated from eggs had far higher homozygosity than those from females. The draft genome size of *N. cucumeris* was estimated to be 173 megabases (Mb) using a whole-genome shotgun approach with the sequencing platform Illumina HiSeq2500 (Table 1 and Additional file 1: Table S1). The average sequencing depth and coverage reached 287 X and 98.14%, respectively.

**Table 1:** Summary of the *N. cucumeris* genome assembly statistics

Total length of sequencing reads	51.75 Gb
Average sequencing depth and coverage of the assembly	287 X and 98.14%
Number of scaffolds (> 1000 bp)	1173
Total length of scaffolds (> 1000 bp)	173,051,269 bp
N_50_ scaffold length	1572,811 bp
N_90_ scaffold length	356,753 bp
The longest scaffold length	9,949,812 bp
Number of contigs	3715
Total length of contigs	171,084,239 bp
N_50_ contig length	222,916 bp
N_90_ contig length	29,715 bp
The longest contig length	1,906,184
Number of gaps	2542
Total length of gaps	1,967,030 bp
The longest gap length	16,194 bp
Estimated genome size	173 MB

#### Genome assembly

The combined 51.75 Gbp of Illumina reads were assembled into 3715 contigs with a contig N_50_ of 222.9 kb and then into 1173 scaffolds with a scaffold N_50_ of 1572.8 kb, resulting in 173 Mb of genome sequence, which is slightly smaller than the estimated genome size (176 Mb) (Table 1). To assess the accuracy and completeness of genic region assembly in the draft genome, we examined the Benchmaking Universal Single-Copy Orthologues and two sets of transcripts assembled with RNA-seq data by the BUSCO software (version 2.0.1, RRID:SCR_015008) [48] and BLAST, respectively. The results revealed that 95.22% of the arthropod BUSCOs were present in the assembly (89.96% complete single copy and 5.25% duplicated BUSCOs); moreover, the percentage of the matched transcripts with length of > 500 nt and > 1000 nt reached 98.14 and 99.32%, respectively (Additional file 1: Table S2). Therefore, the *N. cucumeris* genome acquired a significantly complete assembly [39]. Comparison of the genome assemblies of the sequenced species within the Acari showed that: i) in general, the assemblies and the estimated genome sizes of the species belonging to the superorder Parasitiformes are larger than those belonging to the superorder Acariformes, except *Hypochthonius rufulus*; ii) within the Parasitiformes, the *N. cucumeris* genome assembly is slightly larger than those of both *M. occidentalis* [39] and *Rhipicephalus microplus* [49], but smaller than those of *Varroa destructor* [50], *Tropilaelaps mercedesae* [40] and *Ixodes scapularis* [51] assemblies; iii) the genome size of *N. cucumeris* is nearly twice that of its prey *T. urticae* [52] (Additional file 1: Table S3).

#### Genome annotation and assessment

First, a total of 17,514 protein-coding genes were annotated by combining homology-based and ab initio methods, and approximately 84% significant homology to sequences in public databases (such as NR, SWISS-PROT, COG, TrEMBL, GO and KEGG) (Additional file 1: Table S4 and S5). Subsequently, 1221 additional protein-coding genes were annotated through transcriptomic analysis. The gene repertoire of *N. cucumeris* is very similar to those of *M. occidentalis* [39], *T. urticae* [52] and *I. scapularis* [51]. The total length of protein-coding genes was about 50.55 Mb representing 29.2% of this assembly. The gene density of this assembly (~ 101 genes per Mb) is similar to those in *M. occidentalis* (~ 121 genes per Mb) and *D. melanogaster* (~ 92 genes per Mb), but nearly half of that in *T. urticae* (~ 205 genes per Mb). On average, a typical *N. cucumeris* gene was 2888 bp in size, and contained nearly four exons and three introns with an average length of 283 bp and 420 bp, respectively. According to our transcriptome data, 79.34% (14,865/18735) of the identified genes exhibited transcriptional activities across process of development (13,662, 72.92%), under different temperatures (13,797, 73.64%), or feeding on different foods (13,324, 71.12%). Additionally, 176 pseudogenes were predicted to be scattered in this genome, caused by framesift (89 pseudogenes), premature stop codons (55 pseudogenes), and framesift and premature stop codons (32 pseudogenes), respectively (Additional file 1: Table S6).

Following Schoville et al. [53], Blobplot [54] was used to assess the potential contamination of the genome assembly and only 0.71% reads were identified as putative contaminants (Additional file 2: Figure S1)—this level is very low, or about 21.5% of that in the Colorado potato beetle, *Leptinotarsa decemlineata* (3.3% reads as putative contaminants) [53].

#### Transposable elements

Transposable elements (TEs) play an important role in dynamic genome architecture evolution of eukaryotic organisms. We identified nearly 20.6 Mb of repetitive sequences accounting for ~ 11.9% of this genome (Additional file 1: Table S7), which is almost twice as high as those in *M. occidentalis* (6.8%) and *D. melanogaster* (6.6%) [39]. Furthermore, the *N. cucumeris* genome harbours a TE family repertoire: i) The dominant TEs were the class I TEs (RNA-based retrotransposons, ~ 5.66%), followed by the class II TEs (DNA-based transposons, ~ 4.13%) and unclassified TEs (unknown, 2.82%); ii) Like *D. melanogaster* and *T. urticae* but not *M. occidentalis* [39, 52], members of the long terminal repeat (LTR) element superfamily belonging to the class I TEs were the most abundant TEs in the *N. cucumeris* genome, which included the families LTR_Gypsy (1.19%) and LTR_Copia (1.13%), LTR_unclassified (0.55%) and the tiniest LTR family TRIM (terminal-repeat retrotransposon in miniature) [55] (0.01%); iii) The highly abundant non-LTR retrotransposons were LINE (long interspersed nuclear elements, 1.49%) and PLE (Penelope-like elements, 1.18%); the latter is absent in the mite *M. occidentalis* genome and is an ancient trans-kingdom horizontal transfer that can mediate DNA transfer between animals and plants [56]; iv) Members of the superfamily DIRS, encoding tyrosine recombinase frequently involved in site-specific recombination, were found in this genome (483 members, 0.11%) but not in both *D. melanogaster* and *M. occidentalis* genomes [39, 57]; v) The dominant class II TEs were TIR (terminal inverted repeat, 1.96%), MITE (miniature inverted-repeat transposable element, 1.02%), and Helitron (rolling-circle transposon, 0.99%), whereas the Crypton (only 28 members with total length of 3806 bp) and the Mariner-type transposon Maverick (0.07%) were rare; vi) Simple sequence repeats (SSR, or microsatellites) were also rare in this genome (0.21%), being unlike the scabies mite (*S. scabiei*) genome in which SSRs accounted for ~ 3% [58].

In accordance with a high abundance of the TEs in the *N. cucumeris* genome, we found at least 20 genes belonging to 8 transposase families and 11 genes belonging to 2 expanded phage integrase families (Additional file 1: Table S8), and at least 83 reverse transcriptase (RNA-dependent DNA polymerase) genes belonging to 7 reverse transcriptase families and 5 endonuclease-reverse transcriptase families (Additional file 1: Table S9). Additionally, 3 genes of the retrotransposon gag protein family were found. These gene families had all expanded, and the most expanded reverse transcriptase family possesses 24 members. So many copies of them might infer their important roles in activities of the high aboundant transposons, retrotransposons and genome architecture evolution of this predatory mite.

### Phylogeny and evolution of Acari

#### Phylogenetic relationship and origin of Acari

**Fig. 1:**
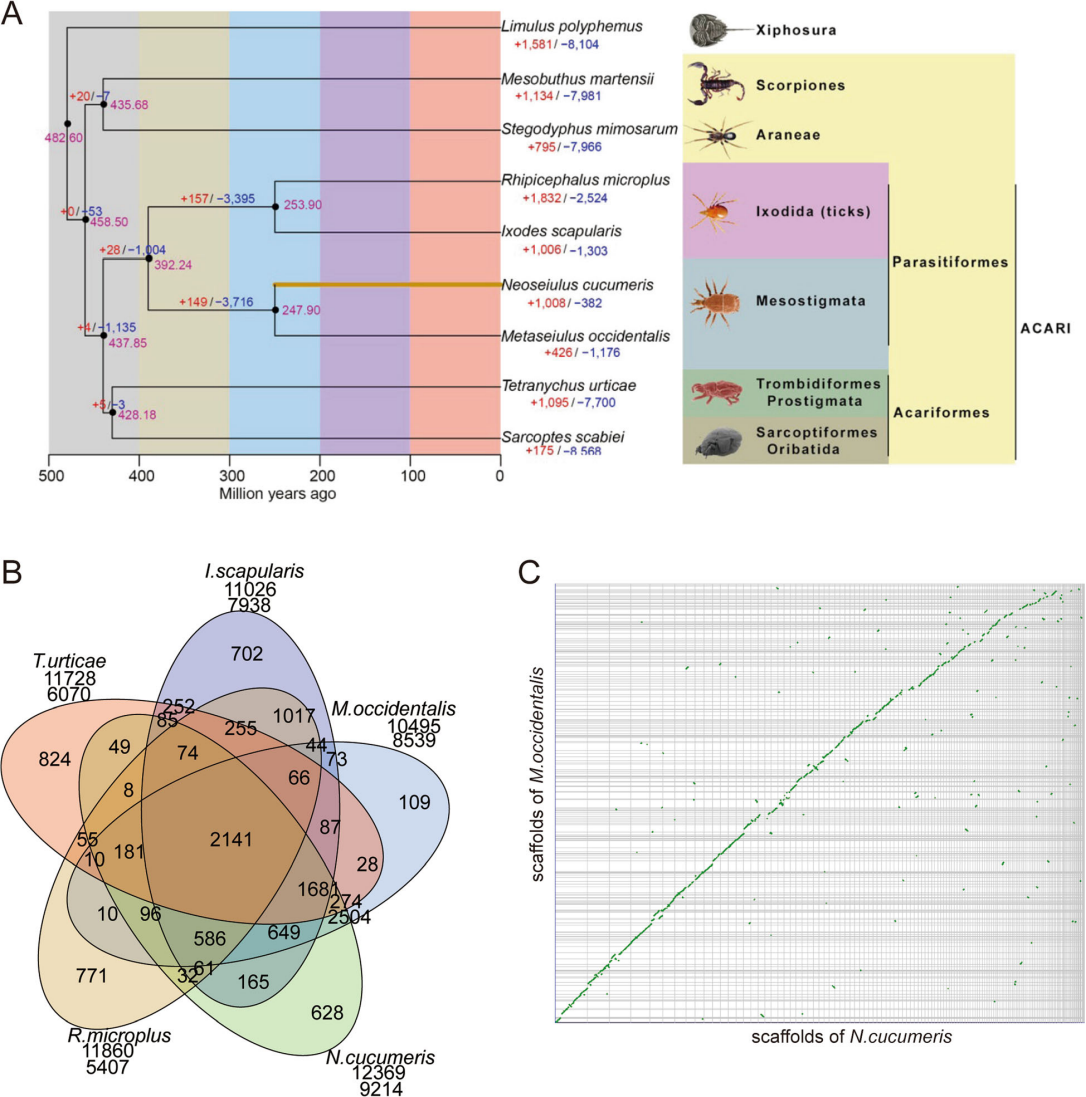
Comparative genomics, phylogenesis, and evolution of the Acari species. **a**. The phylogenomic tree of mites based on predicted protein data with divergence time estimates) and. Genomic data from six species of Acari were included: two tick species (*Ixodes scapularis* and *Rhipicephalus microplus,* order Ixodida), two predatory mite species (*Metaseiulus occidentalis* and *Neoseiulus cucmeris*, order Mesostigmata), and two acariform mites (*Tetranychus urticae*, order Trombidiformes and *Sarcoptes scabiei*, order Sarcoptiformes). Two non-mite arachinids were also included: *Stegodyphus mimosarum* (Scorpiones *Mesobuthus martensii* (Araneae); full genomic data for other orders of Arachnida not available. *Limulus polyphemus* (Xiphosura) was used as an outgroup taxon, with the possible *Limulus polyphemus*-arachnida split 490 (468–520) MYA as one fossil calibration. **b**. Comparison of the gene families of five sequenced species within the Subclass Acari. A total of 2141 gene families were shared by all the species *N. cucumeris* (23.24% of 9214), *M. occidentalis* (25.07% of 8539), *I. scapularis* (28.94% of 7398), *R. microplus* (42.42% of 5047) and *T. urticae* (35.27% of 6070). **c**. The genome microsynteny between two predatory mites: *N. cucumeris* and *M. occidentalis*. 142 N. cucumeris scaffolds (> 10 kb) had strong co-linearity with 224 *M. occidentalis* scaffolds, spanning 137.85 Mb and 123.58 Mb of the N. cucumeris and *M. occidentalis* genomes, respectively

**Fig. 2:**
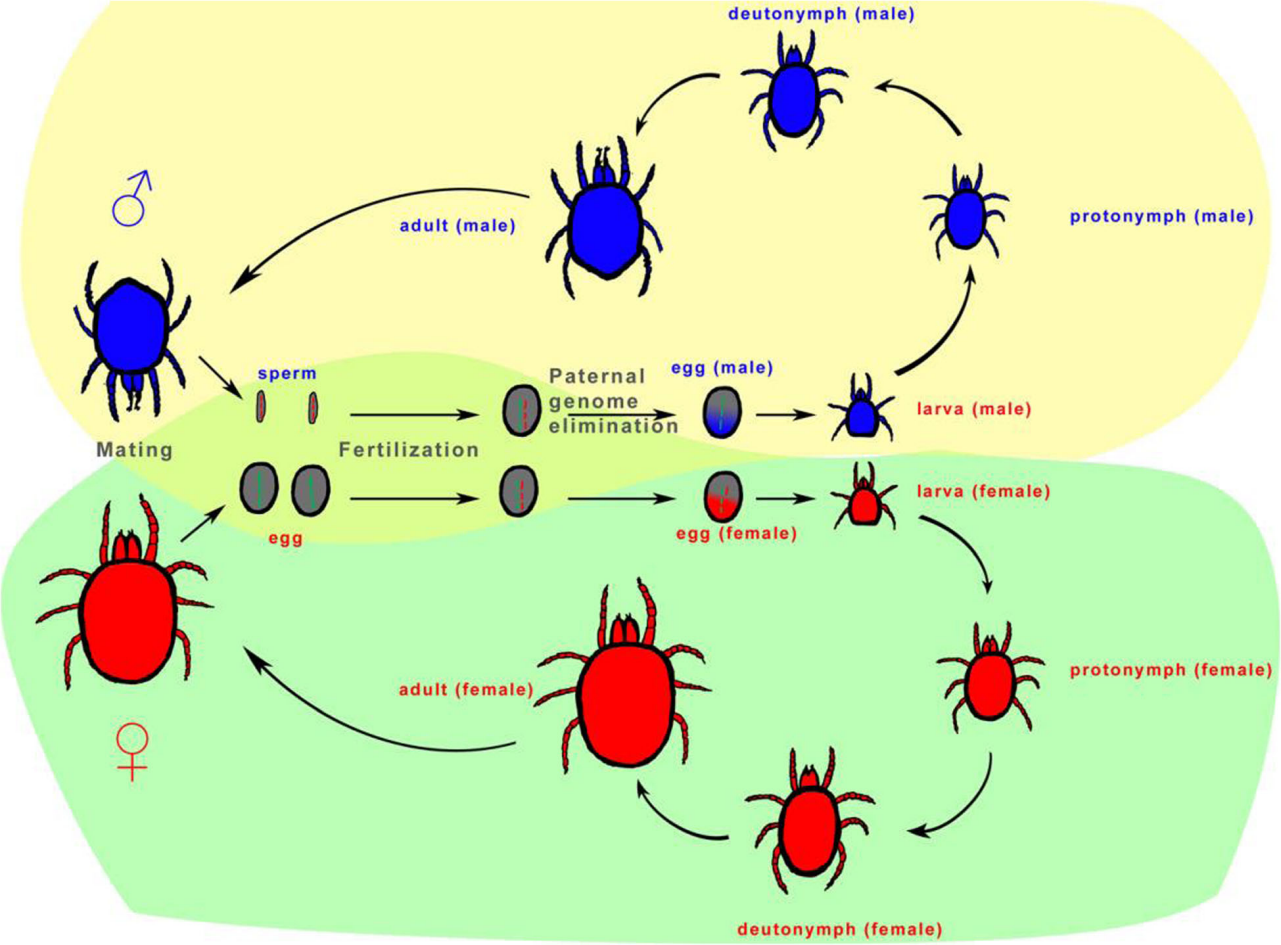
Life cycle, reproduction, and genetic system of the predatory mite *Neoseiulus cucumeris*. Both males and females go through one 6-legged larval stage and two 8-legged nymphal stages (first or protonymph and second or deutonymph) without obvious differences in morphology. Adult males are smaller than females and have more a pointed posterior end. Mating is required for oviposition for female mites, which produce fertilized eggs (2n). In the early embryo, the paternal genome is eliminated in eggs destined to be males in this parahaploid species

A phylogenetic analysis of six species of mites and ticks distributed in four main orders of Acari and two species of other Arachnida based on predicted genomic protein data (Fig. 1a) supported a monophyletic Acari. This maximum likelihood (ML)-based phylogenetic tree was based on 130 universal single-copy orthologues. A further analysis using PhyML4.0 of 1262 single and multi-copy orthologues confirmed the result (Additional file 2: Figure S2a), while another analysis of the same data using the neighbor-joining (NJ)-based MEGA7.0 also revealed a monophyletic Acari (Additional file 2: Figure S2b), with the same internal relationships among orders of Acari as in two other analyses (i.e. Figure 2a and Additional file 2: Figure S2a).

In our analysis, Acari diverged from other arachnids around 437 mya and branched into two mite lineages: Acariformes (428 mya) and Parasitiformes (392 mya). The estimated dates for Acari and Acariformes are within the range of fossil records of Acariformes [9, 59] and in broad agreement with other estimates [37, 60]. The oldest fossil records for Parasitiformes are a tick and an opilioacarid dated back to Upper Cretaceous (Cenomanian, 99 mya) [61, 62]. Our estimated date for Parasitiformes is 355.39 mya, however, within the range of other estimates based on mitochondrial genes (283.40–418.09 mya) [37].

#### Orthology and evolution of Chelicerata

We carried out a clustering of homologous gene families of nine species within the Subphylum Chelicerata, including four Parasitiformes species *N. cucumeris* and *M. occidentalis* (two mites), and *I. scapularis* and *R. microplus* (two ticks); two Acariformes species *S. scabiei* and *T. urticae*; one Scorpiones species *Mesobuthus martensii*; one Araneae species *Stegodyphus mimosarum*; and one Xiphosura species *Limulus polyphemus* (Additional file 1: Table S10). The results indicated that ~ 86.18% (8060) of the 9352 *N. cucumeris* gene families are common to the species *M. occidentalis* within the Suborder Mesostigmata, while only ~ 29.47% (2756) of those are exclusively shared by all the four species belonging to the Superorder Parasitiformes. At the Subclass and Class levels, ~ 20.36% (1904) and ~ 14.88% (1392) of those have homologues in six species within the Subclass Acari and eight species within the Class Arachnida, respectively. Notably, ~ 14.76% (1380) of those are still shared by all the nine species within the Subphylum Chelicerata (Additional file 1: Table S11), suggesting they are conserved Chelicerata-lineage gene families.

According to the homologous gene family clustering of the nine species listed above, 619 (~ 6.62%) gene families were found to be unique to *N. cucumeris*, consisting of 2102 genes (Additional file 1: Table S10). Furthermore, there are 4879 genes without homologues in other lineages (Additional file 1: Table S10). Taking these orphans into consideration, a total of 6981 (37.26%) genes could be identified as *N. cucumeris*-lineage specific genes.

Among the 9352 gene families, ~ 11% of those (consisting of 2639 genes) are significantly (*p* value = 0.01) expanded in *N. cucumeris*. Based on the function annotation against the Pfam database, the most significantly expanded gene families are related to DUF1705 (Domain of unknown function, 45 genes), phage terminase-small subunit (45 genes), ABC transporter transmembrane region (28 genes), reverse transcriptase (RNA-dependent DNA polymerase) (24 genes), and bacterial extracellular solute-binding proteins-family 3 (22 genes). Moreover, the most dominant KEGG pathways enriched by these expanded genes are involved in DNA replication and repair (including DNA replication, 39; Nucleotide excision repair, 37; Mismatch repair, 34; and Homologous recombination, 35), signal transduction (including Wnt signaling pathway, 41; Hedgehog signaling pathway, 31; Neuroactive ligand-receptor interaction and mTOR signaling pathway, each 8; MAPK signaling pathway, JAK-STAT signaling pathway, and Calcium signaling pathway, each 7; and TGF-beta signaling pathway, 6), protein modification and processing (including Lysosome, 17; Protein processing in endoplasmic reticulum, 16; and Ubiquitin mediated proteolysis, 11), reproductive cycle (such as Progesterone- mediated oocyte maturation, 43), and mRNA surveillance pathway (16 genes).

#### Orthology and evolution of Acari

To emphasize orthology and evolution of the Subclass Acari, we further compared the gene families of five sequenced species within the Subclass Acari (Fig. 1b and Additional file 1: Table S12). A total of 2141 gene families were shared by all the species *N. cucumeris* (23.24% of 9214), *M. occidentalis* (25.07% of 8539), *I. scapularis* (28.94% of 7398), *R. microplus* (42.42% of 5047) and *T. urticae* (35.27% of 6070). The relatively low proportion of the conserved core gene families suggests a significant difference in gain and loss property of gene families across the Subclass Acari. *N. cucumeris* shared the highest proportion of homolog gene families with *M. occidentalis* belonging to the same family Phytoseiidae: 8112 gene families with 11,871 genes of *N. cucumeris* had their corresponding homologs in the *M. occidentalis* genome, representing ~ 88.04% and ~ 67.83%, and ~ 95% and ~ 64.73% (total 18,338) of the gene families and genes in the two genomes, respectively. Whereas, 5442 and 3179 gene families of *N. cucumeris* (~ 59.06% and ~ 34.5%) were shared respectively with *I. scapularis* (~ 68.56%) and *R. microplus* (~ 58.79) of the family Ixodidae (order Ixodida) still within the Superorder Parasitiformes. Across the superorder, *N. cucumeris* only shared 4493 (~ 48.76%) homolog gene families (6055 homolog genes, 32.32%) with *T. urticae* (70.02 and 42.9%) of Acariformes. Taken together, these genomic data strongly support the phylogenetic relationship and evolutionary distance between *N. cucumeris* and other Acari species (Fig. 1a).

Besides large proportion of homolog gene families and genes, the genomes of two predatory mites, *N. cucumeris* and *M. occidentalis*, also shared a high degree of microsynteny (Fig. 1c). The mapping results indicated that 142 *N. cucumeris* scaffolds (> 10 kb) had strong co-linearity with 224 *M. occidentalis* scaffolds, spanning 137.85 Mb and 123.58 Mb of the *N. cucumeris* and *M. occidentalis* genomes, respectively. Moreover, a total of 9108 genes (~ 49%) of *N. cucumeris* could be mapped in the 423 microsynteny blocks. However, the degree of microsynteny between the *N. cucumeris* and *T. urticae* genomes was very poor, with only 2 well-recognized microsynteny blocks.

### Development, reproduction and sex determination of the Phytoseiid predatory mite

#### General description of life cycle and reproductive biology

The life cycle of a typical phytoseiid mite includes the egg and four motile stages: larva, protonymph, deutonymph and adult [63]. With the exception of a few thelytokous species, most phytoseids are pseudoarrhenotokous and females must mate before producing fertilized eggs (2n). In early embryonic development, the paternal genome is eliminated in eggs destined to become males in this pseudoarrhenotokous group (Fig. 2, upper part in yellow), whereas diploid eggs develop into females [39, 64] (Fig. 2 lower part in green). The larvae are six-legged and often non-feeding, with a short duration. The fourth pair of legs first appear in protonymphs. Deutonymphs are similar to protonymphs, but slightly bigger. Adult males are smaller than females and have more pointed posterior end. Females have a short pre-oviposition period (2 or 3 days) after mating and can lay one to five eggs per day for a couple of weeks or more.

#### Overview of the developmental transcriptomes

The transcriptomes of the five developmental stages, namely 12 h eggs (early embryonic development), 36 h eggs (late embryonic development), larvae, nymphs and adults, were determined by the RNA-seq technique. According to our transcriptome data, a total of 92.77% (17,380/18,735) of the predicted genes exhibited transcriptional activities; the gene transcription percentage of the five developmental stages was 81.73% (15,313), 82.21% (15,402), 85.26% (15,974), 88.28% (16,539), and 81.53% (15,275), respectively (Additional file 1: Table S13). The results demonstrated that the larvae and nymphs of the mite *N. cucumeris* have relatively higher gene transcriptional activity.

We found that the top 10 KEGG pathways enriched among the expressed genes with high levels in each of the five developmental stages were Spliceosome, Protein processing in endoplasmic reticulum, RNA transport, Ribosome, Carbon metabolism, Ubiquitin mediated proteolysis, Purine metabolism, Pyrimidine metabolism, mRNA surveillance pathway, and Nucleotide excision repair. Notably, except 37 genes of unknown function, the top 100 highly expressed genes in the adults were mainly involved in translation (including 47 ribosomal proteins, ribosome biogenesis protein NSA2, elongation factor 1-alpha 1, peptidyl-prolyl cis-trans isomerase-like, and ubiquitin-like protein FUBI-like), vitellogenesis (including 4 vitellogenin genes), transcription (including ATP-dependent RNA helicase cgh-1 and transcription initiation factor TFIID subunit 10), metabolism (including fatty acid-binding protein and ATP synthase lipid-binding protein, mitochondrial-like), signal transduction (gamma-aminobutyric acid receptor-associated protein-like), and stress resistance (peroxiredoxin 1).

The KEGG pathways that have stage-specific expressions are i) Nucleotide excision repair, DNA replication, Mismatch repair, Homologous recombination, and Arachidonic acid metabolism in the 12 h eggs; ii) Ubiquitin mediated proteolysis, and Tyrosine metabolism in the 36 h eggs; iii) Ubiquitin mediated proteolysis, Nucleotide excision repair, DNA replication, Mismatch repair, Homologous recombination, and Plant hormone signal transduction in the larvae; and iv) Spliceosome, RNA transport, Ubiquitin mediated proteolysis, mRNA surveillance pathway, Nucleotide excision repair, DNA replication, Mismatch repair, Homologous recombination, Aminoacyl-tRNA biosynthesis, Starch and sucrose metabolism, and Porphyrin and chlorophyll metabolism in the nymphs. In addition, the number of genes differentially expressed between two adjoining developmental stages was 2808 between the 12 h eggs and 36 h eggs, 3443 between the 36 h eggs and larvae, and 3353 between the larvae and nymphs, respectively.

#### Vitellogenins and vitellogenesis

Regulation of yolk protein vitellogenin (Vg) synthesis plays a critical role in female reproduction in insects [65]. This is the same in mites, for example, *Vg* mRNA was not detected in diapausing adult females of *T. urticae* [66]. In the *N. cucumeris* genome, both *NcVg1* and *NcVg2* have two copies (Additional file 1: Table S14). Furthermore, we found two genes encoding the vitellogenin receptor (*VgR*), which had been confirmed as being absolutely required for the uptake of Vgs into the eggs in the American dog tick, *Dermacentor variabilis* [67]. It was reported that both *NcVg1* and *NcVg2* reached the maximum expression level during the pre-oviposition period, and there is a positive correlation between the expression of Vgs and fecundity in *N. cucumeris* [68]. In our RNA-seq data, *NcVg1* (Gglean015031 and Gglean014915), *NcVg2* (Gglean009529 and Gglean009616) and *NcVgR* (Gglean006835 and Gglean011739) were all expressed at extremely high levels (RPKM values, from 192.5 to 5671.8) in the adult mites but with extremely low level (RPKM values, from 0.01 to 8.04) in the mite eggs, larvae, and nymphs. Notbaly, the transcriptional level of Gglean009529 (RPKM = 5671.8), Gglean015031 (RPKM = 2715.3), Gglean009616 (RPKM = 2088.1) and Gglean014915 (RPKM = 1083.9) in the adults ranked the second, the thirteenth, the twentieth and the eighty-fifth among all the 18,380 genes, respectively (Additional file 1: Table S13).

In most insects studied so far, juvenile hormone (JH) regulates the synthesis of Vgs that initiate vitellogenesis and female reproduction [69]. In *T. urticae*, the final product of JH is methyl farnesoate (MF) due to the absence of the insect JH epoxide gene *CYP15A1*, which is the same in Crustacea [52, 70]. The role of MF in the spider mite physiology has not been verified, and its role in Crustacea is still debated [70]. According to a hypothesis and a unifying model for the Acari proposed by Cabrera et al. [71], ecdysteroids, instead of JHs, regulate vitellogenesis in both mites and ticks. In Acari, the typical arthropod 20E had been found in the tick *Ornithodoros moubata* [72], but the ecdysteroid 25-deoxy-20-hydroxyecdysone (ponasterone A) had been confirmed to be used as the moulting hormone in the spider mite *T. urticae* [52]. Most known ecdysteroid biosynthesis *CYP450* genes were identified in the *N. cucumeris* (Additional file 1: Table S14, Additional file 2: Figure S3). Surprisingly, we could also not identify the Rieske-like oxygenase gene *Neverland* that converts cholesterol into 7-dehydrocholesterol [73]. The *N. cucumeris* genome contains three ecdysone receptor (*EcR*) genes and the partner ultraspiracle (*Usp*) gene (Additional file 1: Table S14), whose products form a heterodimer to which ecdysteroids bind and which control a variety of downstream processes related to development and reproduction [73]. In addition, at least 29 hormone-related nuclear receptor genes were found in the *N. cucumeris* genome (Additional file 1: Table S14), which could play important roles in reproduction and development of the mite *N. cucumeris*.

#### Regulation of oviposition

Oviposition (egg-laying) consists of ovulation, transfer of a mature egg from the ovary to the uterus where fertilization occurs, and deposition of eggs to an external location with suitable environmental conditions [74]. In insects, the major biogenic amine Octopamine (OA), which functions as a neurotransmitter, neuromodulator and neurohormone, is vital for oviposition, and is biosynthesized from tyrosine by the sequential actions of tyrosine decarboxylase (TDC) and tyramine beta-hydroxylase (TβH) [75, 76]. Furthermore, insect females lacking the vesicular monoamine transporter (VMAT) are sterile. In the *N. cucumeris* genome, we could identify homologs of TDC, TβH and VMAT genes (Additional file 1: Table S14), impling that the mites would use the biogenic amine OA as the neurotransmitter, neuromodulator and neurohormone for oviposition. During vitellogenesis, the oocytes in mites increase in size due to the absorption of cytoplasm, organelles and nutrients including Vgs, for example, an increase of 25-fold and 10-fold for *Varroa jacobsoni* and *T. urticae* oocytes, respectively [71]. In *Drosophila*, Ca^2+^/calmodulin-sensitive protein kinase II (CaMKII) is important for ovulation, and the CaMKII activated by the OA receptor Octβ2R may act on nitric oxide synthase (NOS) to release NO, which diffuses to the muscle for relaxation [74]. The NcCaMKII and NcNOS might play similar roles during the process of *N. cucumeris* ovulation (Additional file 1: Table S14).

In the predatory mite *M. occidentalis*, the clathrin heavy chain gene is important for oviposition, and also for viability, embryogenesis, and systemic RNAi response [77]. In the tick *Haemaphysalis longicornis*, follistatin-related proteins (FRP) were found to be expressed mainly in the ovary and hemolymph, and silencing of FRP by RNAi showed a decrease in tick oviposition [78]. Netrin is a diffusible laminin-like protein and conserved from worms to mammals. In *Drosophila*, the *NetAB* mutants exhibit egg-laying defects due to ovulation defects in females [79]. The clathrin heavy chain gene, *FRP* gene (three copies) and *NetAB* genes could be found in the *N. cucumeris* genome (Additional file 1: Table S14), indicating that they perhaps function similarly in the regulation of oviposition in *N. cucumeris*.

### Sex determination and parahaploidy

#### Sex determination and pseudoarrhenotoky

Haplodiploid reproduction is widespread among animals. Unlike arrhenotoky, in pseudoarrhenotoky both the haploid males and diploid females develop from fertilized eggs in some insects and mites, but in males the paternal chromosomes are eliminated from their germline, namely paternal genome elimination (PGE) [80, 81]. In some phytoseiid mites (Acari: Phytoseiidae) including *N. cucumeris* and *M. occidentalis*, females have six chromosomes but males have only three chromosomes, representing embryonic PGE, in which the paternal genome is lost early during male embryonic development [80, 81]. Although little is known about genetic mechanism of this pseudoarrhenotokous system, there is evidence that low chromosome number evolved prior to haplodiploidy [81]. The ability to control the sex ratio of offspring has been hypothesized to favour arrhenotoky over pseudoarrhenotoky; however, Nagelkerke and Sabelis found that the pseudoarrhenotokous phytoseiid mites can perform precise control of sex allocation [82].

In the pseudoarrhenotokous mealybug *Planococcus citri*, epigenetic marks (such as Me (3) K9H3 and Me (2) K9H3) can serve as the signal to establish the male-specific imprinting on the paternal genome [83]. The *N. cucumeris* genome contains at least 109 methyltransferase genes, including 30 putative histone-lysine N-methyltransferases (HMT) and 8 putative histone-arginine methyltransferases, and 15 histone demethylase genes, including 13 lysine-specific histone demethylase (Additional file 1: Table S15). The transcriptomics data revealed that i) among these 53 histone-methylation modification related genes, 47 genes (88.7%) were all expressed in the mite eggs, larvae, nymphs, and adults, and only one histone-arginine methyltransferase gene (Gglean013613) was not expressed at any of the four developmental stages; ii) the expression level of 33 genes (62.3%) decreased gradually from the mite eggs at 12 h to the nymph stage, suggesting they may have a vital role for early embryo development; iii) 23 genes (43.4%) displayed the highest expression level in the adult stage; and iv) the HMT|SMYD gene Gglean003543 was expressed specifically at the nymph stage (Additional file 1: Table S13). The information suggests that the histone epigenetic regulation of *N. cucumeris* is fully functional, and that it is achieved mainly by methylation/demethylation of histone-lysines. As for DNA methylation, we identified four DNA methyltransferase-like genes including *Dnmt2* without the DNA (cytosine-5)-methyltransferase genes *Dnmt1* and *Dnmt3* (Additional file 1: Table S15), which is the same in the fruit fly *D. melanogaster* and the mite *M. occidentalis* [39]. Although Dnmt2 contains all the signature motifs of a DNA methyltransferase, it actually functions as a (cytosine-5) tRNA methyltransferase in *D. melanogaster* [84]. Indeed, DNA cytosine-5 methylation can be found in the *D. melanogaster* genome [85] and is independent of Dnmt2 activity, implying the presence of novel DNA (cytosine-5)-methyltransferase(s) [86]. Furthermore, very low levels of adenine-6 DNA methylation (m^6^A) have also been described in various eukaryotic genomes [87]. Thus, DNA methylation of the *N. cucumeris* genome might be achieved by both the DNA (adenine-6)-methyltransferases and DNA (cytosine-5)-methyltransferase(s), which needs to be verified in the future. Moreover, the *N. cucumeris* genome contains a histone acetylation and deacetylation system, which includes 14 putative histone acetyltransferase (HAT), and 7 putative histone deacetylase (HDAC) genes and 3 putative NAD-dependent histone deacetylases (Sirt1, Sirt2, and Sirt6) (Table S15). The RNA-seq data indicated that i) 23 HAT and HDAC genes were expressed in the mite eggs, larvae, nymphs and adults besides the HAT gene Gglean005421; ii) the expression level of the HDAC gene *Sirt1* (Gglean008244) was the highest at the all four developmental stages, implying its functional importance; and iii) the *HDAC1* (Gglean004265) and *HAT*|Bromodomain (Gglean007276) genes displayed the highest expression level in the mite eggs at 12 h with a 2~3-fold downregulation at other developmental stages, suggesting they might have a vital role in early embryo development (Additional file 1: Table S13). Therefore, epigenetic regulation of DNA and histone methylation/demethylation and the histone acetylation/deacetylation system could play indispensible roles in the haplodiploid reproduction, sex determination, and development of *N. cucumeris*.

Although genetic systems for sex determination in arthropods show a high diversity in different species, the signal cascade genes of the sex-determining pathway is remarkably well conserved [39, 88], with more conserved at the bottom but more diverse primary signals at the top [89]. Sex determination in *D. melanogaster* is controlled hierarchically by *Sex-lethal* (*Sxl*) > *transformer*/ *transformer*-2 (*tra*/*tra2*) > *doublesex* (*dsx*) and *fruitless* (*fru*) [90]. Moreover, the *doublesex*–*transformer* axis is conserved among most insects studied so far and that *transformer* is the key gene around which variation in sex determining mechanisms has evolved [91]. At the top of the sex-determining signal cascade, the expression of *Sxl* is transcriptionally regulated by several transcription factors such as *sisterless*-*B* (*sisB*), *deadpan* (*dpn*), and the segmentation gene *runt* [92, 93], to respond to the relative number of X chromosomes and sets of autosomes (X:A ratio). A transcriptional co-factor *intersex* (*ix*) is expressed in both sexes of *D. melanogaster*, while its protein product IX interacts with DSX(^F^) but not DSX(^M^), resulting in a female-specific terminal differentiation [94]. Comparative genomics analysis indicated that homologs of all the aforementioned genes were identified in the *N. cucumeris* genome (Additional file 1: Table S16), and the majority of them are present in other Acari genomes such as *M. occidentalis*, *I. scapularis*, *T. urticae* and *S. scabiei* (Additional file 1: Table S17). Notably, these genes were all expressed from the eggs to the adults and most of them exhibited a similar trend with significantly higher level in eggs and juveniles than in the adult mites (Additional file 1: Table S13). The overall expression levels of these genes, however, exhibited great differences. These data might infer that this signal cascade pathway may contribute not only to sex-determination but also sex maturation and development.

Interestingly, we discovered potential gene duplications for both *tra* (3 copies) and *dsx* (3 copies) in the *N. cucumeris* genome (Additional file 1: Table S16; Additional file 2: Figure S4 and Additional file 2: Figure S5). Gene duplications were also found for *tra* (2 copies) in the spider mite *T. urticae* genome and for *tra2* (4 copies) and *dsx* (3 copies) in the Atlantic horseshoe crab *L. polyphemus* genome, but not in the western orchard mite *M. occidentalis*, the tick *I. scapularis*, the scabies mite *S. scabiei* genomes (Additional file 1: Table S17). In a phylogenetic tree, three *Nctra* genes were all clustered together with the *tra* genes from other species in a lineage, which is distinct from the *tra2* cluster (Additional file 2: Figure S4). In insects, gene duplications of *tra* have thus far only been identified in the Order Hymenoptera, including bees, wasps and ants, which are also haplodiploid [89, 95, 96]. Thus, it may be interesting to examine the link between the duplication of *tra* genes and haplodiploidy.

#### Development from three to four pairs of legs

The adult chelicerate body plan is composed of the anterior prosoma bearing the chelicerae, pedipalps, and the four pairs of walking legs and the posterior opisthosoma. In Acari, however, the development of the fourth pair of walking legs is suppressed during embryogenesis and larval stages, with the fully developed fourth pair of walking legs appearing in the nymphal stages [97]. The molecular mechanisms driving the suppression of this appendage are unclear thus far.

In *D. melanogaster*, segmentation occurs by the initial activation of the gap genes, followed by the activation of the pair-rule and segment-polarity genes, and finally by the establishment of the *Hox* gene expression domains within each segment [97, 98]. Despite enormous variation in the arthropod body plan, genes regulating embryonic development are highly conserved. The *N. cucumeris* genome encodes all the four conserved limb gap genes (Additional file 1: Table S18, Additional file 2: Figure S6): *Distal-less* (*Dll*), which is expressed mainly in the distal podomeres; *dachshund* (*dac*), which is expressed mainly in the medial podomeres; and *extradenticle* (*exd*) and *homothorax* (*hth*), which are co-expressed in the proximal podomeres [98]. Interestingly, our transcriptomic data indicated that the transcriptional levels of *exd* and *hth* remain unchanged from embryogenesis (12 h and 36 h) to larval and nymphal stages, while those of *Dll* and *dac* are downregulated almost two fold in the larvae and nymphal stages compared with the embryogenesis stages (Additional file 1: Table S13). These imply that the expression of the limb gap genes is controlled under spatio-temporal regulation.

Moreover, the orthologues of the transcription factors, the pair-rule genes (*hairy*, *even-skipped* (*eve*), *runt* (*run*), *odd-skipped* (*odd*, 2 copies), *fushi tarazu* (*ftz*) and its partner *fushi tarazu transcription factor 1* (*ftz-f1*), *paired* (*prd*) and *sloppy-paired* (*slp*)) and the segment-polarity genes (*engrailed* (*en*), *hedgehog* (*hh*, 2 copies), *wingless* (*wg*)) could all be identified in the *N. cucumeris* genome (Additional file 1: Table S19). Notably, the expression levels of *hairy*, *eve*, *run*, *prd*, *slp*, *en*, and *hh* (Gglean004851) were significantly decreased from the late stage of embryogenesis (36 h) and are extremely low during the larvae and nymphal stages, whereas that of *odd* (Gglean016652) remains unchanged and the other *hh* (Gglean004680) is not expressed throughout all developmental stages (Additional file 1: Table S13).

**Fig. 3:**
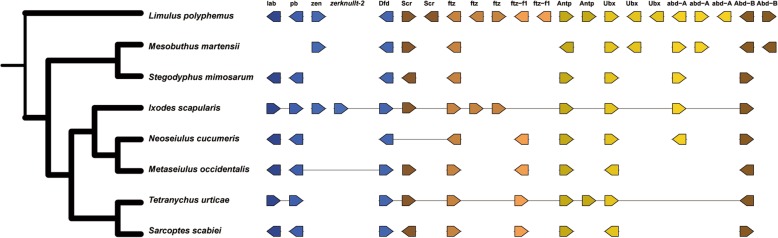
The organization of Hox genes of *Neoseiulus cucumeris* and other chelicerate species. The forward arrows represent the genes on the forward strand while the reverse arrows represent the genes on the reverse strand. The lines indicate the genes on the same scaffold. The length of the lines does not represent the physical length

The structurally and functionally conserved *Hox* genes that encode transcription factors have an ancestral role in all bilaterian animals in specifying segment identities along the antero-posterior axis [98–100]. In the *N. cucumeris* genome, we could identify the orthologues of the *D. melanogaster Hox* genes *labial* (*lab*), *proboscipedia* (*pb*), *Deformed* (*Dfd*), *Antennapedia* (*Antp*), *Ultrabithorax* (*Ubx*), *Abdominal-A* (*Abd-A*) and *Abdominal-B* (*Abd-B*) (Additional file 1: Table S20; Fig. 3). Moreover, the expression level of the *Hox* genes except the gene *pb* was significantly downregulated at the adult stage compared with the immature stages (Additional file 1: Table S13). It is worth mentioning that the *M. occidentalis* Hox genes are completely atomized with each gene on a different scaffold [39], while the *N. cucumeris Hox* genes form a *Hox* gene cluster (from Gglean017502 to Gglean017510), which is similar to those other examined arthropods (Additional file 1: Table S20). Like *M. occidentalis* and *T. urticae*, *N. cucumeris* lacks the genes *bicoid* (*bcd*) and *zerknullt* (*zen*). *I. scapularis*, *M. occidentalis* and *N. cucumeris* all lack the gene *Sex combs reduced* (*Scr*), which is present in *T. urticae* (Fig. 3). It was shown that the *Hox* genes *bcd* and *zen*, also including *ftz*, have taken on non-*Hox*-like functions in *Drosophila* [100], which may explain partly why the *Hox* genes *bcd* and *zen* are absent in the mite genomes. However, the loss of *Scr* in the genomes of *I. scapularis*, *M. occidentalis*, and *N. cucumeris* is interesting as it may be unique to the superorder Parasitoformes.

Zinc finger proteins (ZFP) play important roles in transcription regulation, chromatin dynamics, cell signaling, ecdysone biosynthesis, development, and disease in metazoans by protein–DNA, protein–RNA or protein–protein interaction. At least 163 ZFP genes assigned to 19 subgroups were predicted in the *N. cucumeris* genome (Additional file 1: Table S21). The most expanded subgroup with 65 genes is the Cys_2_-His_2_ zinc finger (C2H2-type), which is the largest class of transcription factors (TFs) within the majority of metazoan genomes and makes up nearly half of all annotated TFs in human [101, 102]. Other predominant subgroups of ZFPs include the MYM-type (24 genes), BED-type (23 genes), and NF-X1-type (11 genes) (Additional file 1: Table S21). Many ZFPs are thought to function as either transcription activators or repressors by modifying local chromatin structure, such as MYM-type and ZZ-type by histone acetylation, GATA-type by histone methylation, and NF-X1-type and RING-type by ubiquitination [103–105]. Some types of ZFPs are involved in RNA processing and/or stability, including AN1-type, C3H1-type, CCHC-type and Matrin-type [106–108].

The Notch signalling pathway is an evolutionarily highly conserved signaling mechanism and participates in a wide variety of developmental processes in invertebrates and vertebrates, including oogenesis, growth of the leg and specifying the leg joints, and sensory organ development [109]. In the *N. cucumeris* genome, we identified the core genes of the Notch pathway, 3 copies of the receptor gene *Notch* (*N*), the ligand genes *Delta* (*Dl*) and *Serrate* (*Ser*); two E3 ubiquitin ligase genes for ubiquitylation of the ligands Dl and Ser, *Neuralized* (*Neur*) or *Mind bomb 1* (*Mib1*); the antagonist genes *Chip* (*Chi*) and *Beadex* (*Bx*), but not the major antagonist *Hairless* (*H*) and its partner *Cyclin G* (*CycG*) of *Drosophila* [110, 111] (Additional file 1: Table S22).

The Hedgehog (Hh) pathway is another major developmental signalling pathway that plays essential roles in embryonic development and adult tissue homeostasis [112]. The majority of the Hh signaling components could be found in the *N. cucumeris* genome, including the ligand *hedgehog* (*hh*) and its receptor *patched* (*ptc*); the G protein-coupled receptor *Smoothened* (*Smo*); the important complex composed of *Suppressor of fused* (*Su (Fu)*) and zinc-finger transcription factor *cubitus interruptus* (*ci*), but without the scaffold *Costal2* (*Cos2*) and the kinase *Fused* (*Fu*); the related phosphorylation regulators *Gprk2*, *Pka* (*C1~3*), *Ck1* (*dco* and *gish*), *SkpA* and GSK3β *shaggy* (*sgg*); the related ubiquitylation regulators *Cul1*and *Cul3*, *Smurf*, and *Usp8* [113] (Additional file 1: Table S23).

The Hippo pathway—with a kinase cascade, transcription coactivators, and DNA-binding partner—can play pivotal roles in the control of adult tissue growth and cell proliferation, differentiation, and migration in developing organs [114]. In the *N. cucumeris* genome, we identified the core Hippo kinase cascades *hippo* (*hpo*), *warts* (*wts*) and *mob as tumor suppressor* (*mats*), except the Hpo adaptor *salvador* (*sav*); the transcription activator *yorkie* (*yki*); the regulatory kinases *Merlin* (*Mer*, 3genes), *misshapen* (*msn*), *happyhour* (*hppy*); the regulator of Yki subcellular localization, *14–3-3zeta* (2 genes); the EGFR-Hippo pathway link *Jub*; the transcription co-activators *teashirt* (*tsh*), *scalloped* (*sd*), and *Mothers against dpp* (*Mad*) (Additional file 1: Table S24). Several intrinsic cell machineries (e.g. cell-cell contact, cell polarity, and actin cytoskeleton) are known to regulate the Hippo pathway, which is also controlled by a variety of signals such as mechanical cues, cellular energy status, as well as hormonal signals acting via G-protein-coupled receptors [114].

### Environmental adaptation and stress-resistance

#### Signal perception and feeding

Previous studies have indicated that photoperiod has a significant effect on development and reproduction of *Neoseiulus barkeri* [115], and harmful/harmless UV wavelength may influence decision making in foraging phytoseiid mites (*Neoseiulus californicus*) [116]. Eyeless phytoseiid mites can perceive and respond to light, including UV radiation [39]. In the *N. cucumeris* genome, we identified some homologs of known regulatory factors involved in eye specification and retinal differentiation, including *eyeless* (*ey*), *homothorax* (*hth*), *sine oculis* (*so*), *teashirt* (*tsh*), *eyes absent* (*eya*), and *atonal* (*ato*) (Additional file 1: Table S25). Moreover, *N. cucumeris* has the gene *dachshund* (dac) (Additional file 2: Figure S6A), but lacks the *twin of eyeless* (*toy*) that is the other homologue of the Paired domain/homeodomain transcription factor Pax6, whose products interact physically with the Ey in *Drosophila* [117]. For the phototransduction cascade related genes in *N. cucumeris*, no homolog was matched to any of the seven *Drosophila* Rhodopsins (Rh1-Rh7) encoding photosensitive G protein-coupled receptors (GPCRs) that initiate the phototransduction cascade [118], but rhodopsin’s chaperone, *neither inactivation nor afterpotential A* (*NinaA*) [119], and *NinaG* involved in biosynthesis of visual pigment chromophore (3-hydroxyretinal) for rhodopsin [120] could be identified. Except for the photoreceptors (rhodopsins), we identified in *N. cucumeris* the encoding genes of other key phototransduction cascade components: G-proteins (including *Gαq*), phospholipases (including No receptor potential *A*, *NorpA*), transient receptor potential channels (*Trpgamma* and *TrpA1*), and G-protein coupled receptors and G protein-coupled receptor kinases (Additional file 1: Table S25).

The predatory mite *N. cucumeris* can adapt to a changing environment and feed on many species of prey mites, insects and pollens. Chemoreception is accomplished by both membrane-bound receptors and soluble binding proteins that commonly function as solubilizers and carriers of odorants and pheromones [121]. In the *N. cucumeris* genome, we identified 24 gustatory receptor (GR) genes that can be clustered into three clans (Additional file 1: Table S26; Additional file 2: Figure S7A) and 65 ionotropic receptor (IR) genes that can be clustered into four clans (Additional file 1: Table S26; Additional file 2: Figure S7B); in comparison, *M. occidentalis* has the same number of IR genes but much more GR genes (64) [39]. As in other noninsect arthropods, the odorant receptors (ORs) are absent in *N. cucumeris* [39, 52, 122]. For the soluble binding proteins (SBPs), two Niemann-Pick type C2 (NPC2) genes were found in the *N. cucumeris* genome, while the odorant-binding proteins (OBPs) and chemosensory proteins (CSPs) are absent as in most species of Acari with *I. scapularis* as an exception, in which one *csp* gene has been identified [122]. In addition, 5 Transient Receptor Potential (TRP) cation channel genes were identified in the *N. cucumeris* genome (Additional file 1: Table S26), which have also been implicated as chemosensory receptors [123]. Moreover, the *N. cucumeris* genome harbors two oo18 RNA-binding protein 2 (ORB2) genes, which have been confirmed to be involved in olfactory long-term memory formation in *Drosophila* [124]. These genes would enable *N. cucumeris* to conduct communication between conspecifics, locate food sources, and detect predators and toxic substances by perceiving a variety of odorant and pheromone cues [123].

#### Detoxification and stress-resistance

The polyphagous predatory mites, such as *N. cucumeris* and *N. barkeri*, are now regarded as successful commercial biological control agents for pest mites and insects. However, they are subjected to manifold stresses including toxic endogenous compounds and xenobiotics, and starvation, oxidative and thermal stress. One route that leads to toxin resistance of mites is the duplication or amplification of the detoxifying enzyme genes, which have now been described for several gene superfamilies: cytochrome P450 (CYP), glutathione-S-transferase (GST), and carboxyl/ cholinesterase (CCE) [52, 125, 126].

**Fig. 4:**
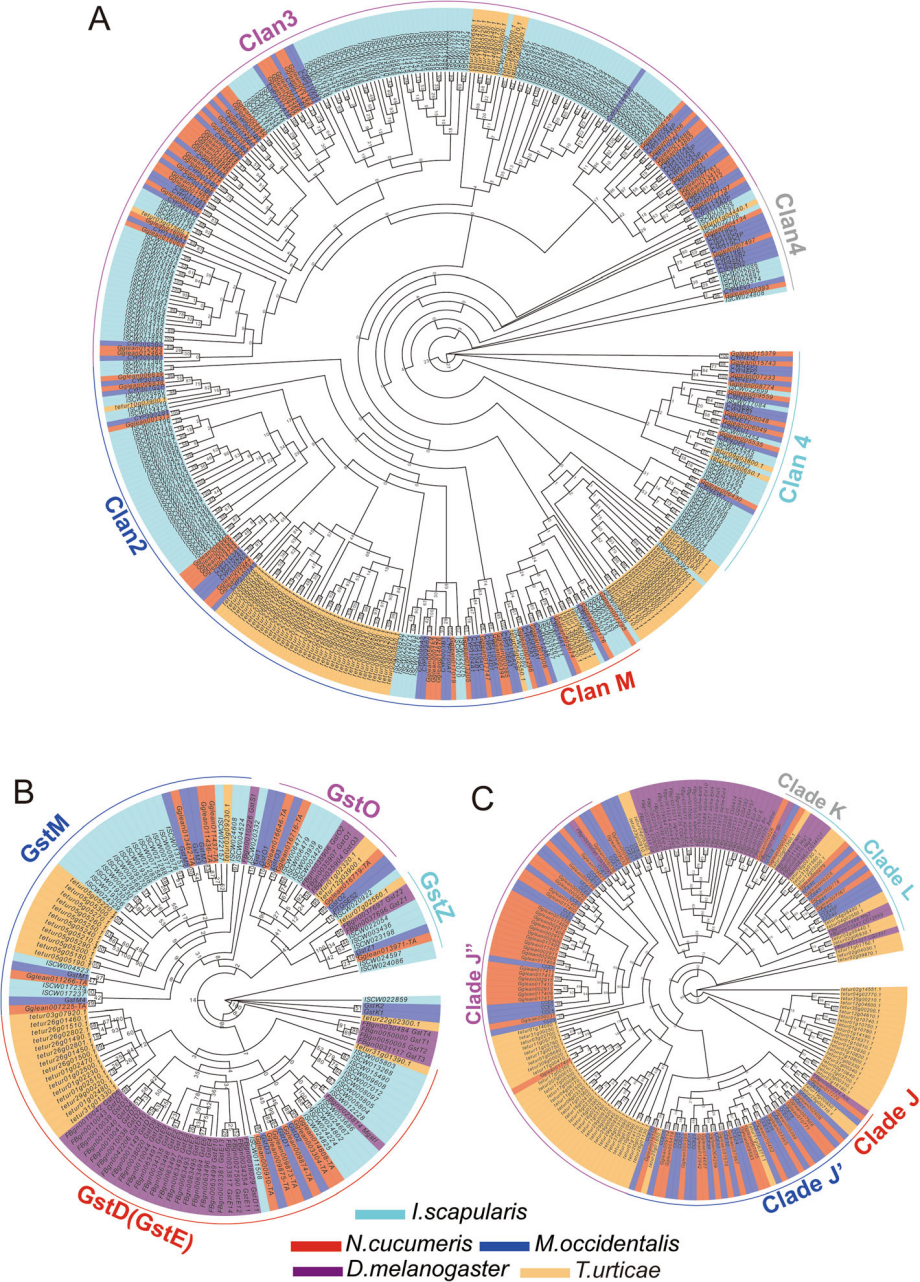
The detoxification and stress-resistance related gene superfamilies identified in *N. cucumeris*, showing phylogenetic relationships (in protein sequences). **a** of cytochrome P450 (CYP450), **b** of glutathione-S-transferase (GST) and C of carboxyl/ cholinesterase (CCE). This is maximum likelihood tree. Numbers at nodes are bootstrap values. The genes with red background are from *N. cucumeris*. The genes with blue background are from *M. occidentalis*. The genes with cyan background are from *I. scapularis*. The genes with purple background are from *D. melanogaster*

Sixty-nine CYP genes were identified in the *N. cucumeris* genome (Additional file 1: Table S27), similar to those in fly *D. melanogaster* (70 genes), fewer than those in spider mite *T. urticae* (86 genes) but more than those in another predator *M. occidentalis* (63 genes). These CYP genes can be assigned to four clans: CYP2 (17 genes), CYP3 (34 genes), CYP4 (12 genes) and Mitochondrial CYP (CYPM, 6 genes) (Fig. 4a). The *N. cucumeris* genome has four classes of GST genes: Delta/Epsilon (6 genes), Mu (5 genes), Omega (3 genes), and Zeta (1 gene) (Additional file 1: Table S28 and Fig. 4b), similar in number to *M. occidentalis* (12 genes) but almost half of *T. urticae* [52, 126]. Fifty-three CCE genes were found in the *N. cucumeris* genome, being more than those in *M. occidentalis* (42 genes) but fewer than *T. urticae* (68 genes) (Table S29). Moreover, these CCE genes can be assigned to five clades: clade J (Acetylcholine esterase, AChE, 2 genes), clade K (Gliotactin, 1 gene), clade L (Neuroligin, 4 genes), clade J’ (17 genes) and clade J” (29 genes) (Fig. 4c). Like most noninsect species, the mite *N. cucumeris* also lacks the dietary/detoxification CCE clades (A, B and C) and the hormone/semiochemical CCE clades (D, E, G and F) of insects, but has an obvious expansion in the specific and ancient clades J’ and J” [126]. Interestingly, the polyphagous predatory mite *N. cucumeris* harbours more GST, CYP and CCE genes than the obligatory predator *M. occidentalis*. Members of the GST, CYP and CCE superfamilies in the two predatory mites are both fewer than those in the phytophagous spider mite *T. urticae* [52, 126]. These differences might reflect a different range of potentially toxic xenobiotics in their diets.

In addition to CCEs, CYPs and GSTs, many other functional gene families can also confer animal resistance to drugs and toxic compounds as well as starvation, oxidative and thermal stress. More than 500 transporter-related genes were predicted in the *N. cucumeris* genome; an expansion occurred within at least 39 gene families which could be assigned to 16 gene superfamilies including the ATP-binding cassette transporter (ABC) and major facilitator superfamilies (Additional file 1: Table S30). These genes could be crucial in supporting mite detoxification, metabolism and development. In addiiton, at least 26 heat shock protein (HSP) genes were predicted in the *N. cucumeris* genome (Additional file 1: Table S31), and it has been confirmed that the expression of some *Hsp* genes (*NcHsp90*, *NcHsp75*, *NcHsp70*, *NcHsp60*, and *NcHsp40*) in *N. cucumeris* can reflect developmental changes, sexual difference, and discriminately induced response to thermal stress [127]. Furthermore, this genome encodes at least 31 antioxidant enzymes: 1 catalase (CAT), 26 peroxidases (POX, including 1 thioredoxin peroxidase, 2 peroxiredoxin-6-like, 3 peroxidase-like, 4 chorion peroxidase-like, 5 phospholipid hydroperoxide/glutathione peroxidase-like, and 11 peroxiredoxin-like), four superoxide dismutases (SOD) (Additional file 1: Table S31). Notably, the high level of the lipid peroxidation (LPO) in *N. cucumeris* under thermal stress can serve as an important signal that the antioxidant enzyme-based defense mechanism is not always adequate to counteract the increased generation of reactive oxygen species (ROS) [128]. The surplus ROS may result in oxidative damage, particularly, DNA damage. Consequently, DNA damage-repair related genes might confer *N. cucumeris* complementary stress resistence [129]. According to the KEGG analysis, the *N. cucumeris* genome harbours at least 108 genes associated with DNA damage-repair such as base/nucleotide excision repairand mismatch repair (Additional file 1: Table S32). In addition to these functional genes, some signal transduction pathways and epigenetic regulation could also play important roles in stress resistence, such as histone deacetylation [130–132].

#### Innate immune systems

Arthropods have a powerful innate immune response via the remarkably well conserved gene families and key immune pathways, which include the Toll, Imd, JAK/STAT, and JNK signaling pathways [133]. Like other mites, *N. cucumeris* has only one peptidoglycan recognition protein (PGRP) gene, without any fungal recognition receptors β-1,3 glucan recognition proteins (βGRPs), which have been found to be entirely absent from all Chelicerates [133]. The cellular immune receptor Dscam (Down syndrome cell adhesion molecule) can bind to bacteria and is thought to play a role in immunity (Armitage et al. 2014). *N. cucumeris* holds 19 Dscam-like genes, being consistent with the fact that Dscam is widespread and has extensive duplications across all the major groups of arthropods [133]. Additionally, the *N. cucumeris* genome encodes one thioester-containing protein (TEP) that can covalently bind to pathogens including fungi for destruction by phagocytosis or melanotic encapsulation [134, 135], one bacterial fibrinogen-binding adhesion protein (FRP) and one Nimrod-like protein Draper that is important in killing bacteria by phagocytosis [136] (Additional file 1: Table S33).

The mite *N. cucumeris* holds a largely intact Toll singaling pathway, including eight Toll-like receptor (*TLR*) genes and two Toll ligand *Spätzle* (*Spz*) genes; one *MyD88* gene, a central player in innate immune signaling; two *Pelle* genes, the functional counterpart of IκB kinase; the nuclear factor-κB (NF-κB) transcription factor *dorsal*, and its inhibitor *cactus*; five *persephone* homologue genes, the CLIP domain serine proteases that trigger a proteolytic cascade and are required for Toll signaling activation (Ming et al., 2014); and other positive or negative regulators (Additional file 1: Table S33).

However, the Imd signaling pathway of *N. cucumeris* is highly reduced, which is common in other species of Chelicerate [39, 133]. Although some components of the Imd signaling pathway could be found (Additional file 1: Table S33), we failed to find any likely key component homologues: *Imd*, *Fadd*, *Dredd* or NF-κB transcription factor *Relish*. In contrast to the absence of many key Imd pathway components in *N. cucumeris*, the highly conserved JNK and JAK/STAT signaling pathways are relatively intact (Additional file 1: Table S33). In the JNK pathway, the genes *Basket* (*JNK*, 2 genes), *hemipterous*, *bendless*, and other regulators are present; in the JAK/STAT pathway, the receptor *Domeless*, the kinase *JAK*, and transcription factor *Stat92E* (8 copies) are found, but without the homologue of the *Drosophila* Janus kinase *Hopscotch* (*Hop*). Hopscotch FBgn0004864 in *Drosophila* fly was used for searching homolog in *M. occidentalis* using BLASTp and GeMoMa. The low sequence similarity (identity: 40.98% and coverage: 22.6%) indicated that hopscotch was also missing from *M. occidentalis*.

For primary antiviral defense, double stranded viral RNA is cleaved into short interfering RNAs (siRNAs) by Dicer, and then loaded into Argonaute (Ago) proteins to direct the degradation of viral RNA [133]. Four *Dicer*-*1* genes and two *Dicer*-*2* genes, and four *Ago1* genes, six *Ago2* genes and five *Ago3* genes were found in the *N. cucumeris* genome (Additional file 1: Table S34). In addition, eleven immune effector Lysozyme (Lys) genes could be found in the *N. cucumeris* genome, but no homologue of the *Drosophila* antimicrobial peptides (AMPs) are present (Additional file 1: Table S33). More importantly, the vast majority of the above-mentioned innate immunity-related genes appear to be transcriptionally active in *N. cucumeris* (Additional file 1: Table S13), suggesting the mite has a powerful and functional innate immune response.

## Discussion

One interesting result of this study is from our phylogenomic analysis which supported the monophyly of Acari. This is especially encouraging given that two earlier phylogenomic analyses associated with full genomic sequencing of Acari [39, 40] rejected the monophyly of Acari.

The monophyly of Acari has been recognized by traditional acarologists [12–15] and was supported by early phylogentic studies [3, 16–19]. A recent comparative genomic analysis of chemosensory genes also supported the monophly of Acari, although it included only three mite species [42]. It should be noted that a monophyletic Acari was rejected by many studies, molecular or morphological, in the last two decades [10, 11, 29–38], including three studies using genomic data which either placed Parasitiformes more closely to spiders (Araneae) than to Acariformes [39, 40], or placing Acariformes closer to Pseudoscopiones than to Parasitiformes [41], although the number of arachnid taxa included in both analyses is small (two, four and five mite species, respectively). The genomic sequencing of *N. cucumeries* enabled this analysis to include six species of mites distributed in four main orders of mites—a much better sampling of taxa than three recent other studies [39–41]. The relationships within Acariformes and Parasitformes are also consistent with conventional hypothesis based on morphology [14].

It should be noted that while this paper was submitted in review, two related papers were published. A recent phylogemomic study using targeted ultraconserved genomic elements from 13 species of Acari showed a non-monophyletic Acari [43]. However, another new study published in mid-2019 [137] increased species sampling to 21 species of Acari (but not including *N. cucumeris* and *M. occidentalis*) and most orders of Arachnida provided support for a monophyletic Acari.

Recent phylogenomic analyses thus indicate that it is premature to reject the monophly of Acari. Further studies (including missing taxa and other lines of evidence) are needed to understand the phylogeny of mites and relatives.

## Conclusion

The polyphagous predatory mite *N. cucumeris* had been used as a biocontrol agent against pests such as mites and thrips worldwide. With combined genomics and developmental transcriptomics we showed: (1) The 173 Mb genome contains ~ 11.9% (20.6 Mb) repetitive sequences and encodes 18,735 protein-coding genes, with approximately 37% (6981) being *N. cucumeris*-lineage specific genes; (2) For the evolutionary relationships of the chelicerate orders, our phylogenomic analysis supported the monophyly of Acari; (3) Our transcriptomic analysis of different life stages of *N. cucumeris* provides new insights into genes involved in its development; (4) The sex determination, development, and reproduction related genes are relatively conserved, but the innate immune system related genes are less conserved; (5) The polyphagous predatory mite *N. cucumeris* holds more detoxification and stress-resistance related genes (including GST, CYP and CCE genes) than the obligatory predator *M. occidentalis*, but fewer than those in the phytophagous spider mite *T. urticae*. Together, our genomics and developmental transcriptomics analyses of *N. cucumeris* provide invaluable resources for further research on the development, reproduction, and fitness of this economically important mite.

## Methods

### Genome sequencing and assembly

#### Specimen collection and genomic DNA preparation and sequencing

##### Specimens used for sequencing *Neoseiulus cucumeris* genome

We used mite eggs to extract genomic DNA for sequencing. Adult females of *N. cucumeris* were picked from our lab culture, which is a susceptible strain maintained on acarid mite *Aleuroglyphus ovatus* in our laboratory for over ten years. *N. cucumeris* was originally imported from Biological Crop Protection Limited, UK. They were placed on a black plastic sheet resting on a round foam plastic (17 cm diameter) soaked with water inside a Petri dish (19 cm diameter). A few small pieces of cinnamon leaves (*Cinnamomum cassia*) added to the area to allow female mites to lay eggs for 24 h before egg collections. Collected eggs were within 1 day old and were stored in liquid nitrogen immediately. We collected a total of 40,000 eggs.

##### Genomic DNA preparation and sequencing

Genomic DNA was extracted from these eggs with the standard CTAB method [138]. First, we performed contamination estimation by aligning 20,000 randomly selected reads to the NT database to know the major contaminated species using BLASTn. Then all the sequencing reads were aligned to the genomes of contaminated species using BWA and properly mapping reads would be removed. Filtered reads were used for assembly. The purified genomic DNA was quantified using Qubit2.0 before whole genome shotgun sequencing. Three paired-end (PE) libraries (with insert size of 180 bp, 220 bp and 500 bp, respectively) and nine mate-pair (MP) libraries (ranging from 2 Kb to 15 Kb) were prepared using Illunima’s DNA library preparation kits following the standard protocols. The libraries were sequenced on Illumina Hiseq 2500 platform (2 × 125 bp).

#### Estimation of genome size by 21-mer analysis

We used the filtered reads from a PE library (220 bp insert-size) to estimate the genome size with algorithm G = K_num/peak_depth, where the K_num is the total number of K-mer, and Peak_depth is the value of K-mer depth [139]. A K-mer typically refers to a sequence of length K. The genome size can be estimated by counting k-mer occurrence. The results showed that the K value was 21 and the main peak depth was observed at 24 as exhibited by 21-mer frequency plot. Therefore, the genome size of *N. cucumeris* was estimated to be 173 Mb.

#### Raw data filtering

To reduce the effect of sequencing error on assembly and facilitate the genome assembly, we filtered the following reads:

i. Identical reads probably deriving from PCR duplication during library construction;
ii. Adaptor contaminated reads with more than 10 bp aligned to the adaptor (allowing at most 3 bp mismatch);
iii. Unknown nucleotide (N)-dominated reads (> 5%);
iv. Overlapped reads with more than 10 bp mapped to read1 and read2 simultaneously;
v. Low quality base containing reads.

The filtering was carried out using scripts developed at Biomarker Technologies Corporation, Beijing, 101,300 China. The high-quality reads were obtained and used for genome assembly.

#### Genome assembly

We developed a de novo assembly pipeline for genome assembly. We used a whole-genome shotgun assembler ALLPATHS-LG (52488) [140], with the default parameters, to assemble into contigs with reads from PE libraries and MP libraries (< 8 Kb). Subsequently, these contigs were used for scaffolding by SSPACE [141] and then gap filling by GapCloser [142].

#### Genome assembly assessment and validation

The RNA-seq assembled transcripts and Benchmarking Universal Single-Copy Orthologues (BUSCO version 2.0.1, RRID:SCR_015008) [48] were used to assess the accuracy and completeness of genic region assembly in the draft genome, respectively.

We assessed the genome assembly for evidence of contamination using a Blobplot [53]. 7.17 Gb next-generation sequencing data (~40x) were mapped to the genome assembly using BWA. Taxonomy id was assigned to each sequence though BLASTn against NCBI’s nt database. Then modules ‘create’ and ‘view’ implemented in BlobTools v1.1 [54] were used to obtain the contamination screen result which was visualized using scripts developed at Biomarker Technologies Corporation, Beijing, 101,300 China. Scaffolds labelled with ‘no-hit’ by BlobTools were not considered as the putative contaminants.

### Genome annotation

#### Prediction of repetitive elements

The repeat sequences of the *N. cucumeris* genome were searched by both de novo search and homology-based search against Repbase 6 [143]. We first used the de novo prediction software, including MITE-Hunter [144], LTR-FINDER version .0.5 [145], RepeatScout version 1.0.5 [146] and domain software for repetitive DNA, PILER-DF [147], to construct a de novo repeat library. Then we used RepeatMasker version 4.0.5 [148] for homologous sequence alignment against Repbase 6.

#### Gene model prediction and functional annotation

Genes were predicted separately by de novo*-*based, transcriptome-based, and homology-based strategies to build optimal gene models.

RNA-seq data were assembled by Trinity [149] and the assembled sequences were aligned against the *N. cucumeris* genome by PASA [150]. Gene models created by PASA were used as training set by de novo prediction software Augustus2.4 [151], GlimmerHMM version 3.0.3 [152] and SNAP version 2006-07-28 [153]. The de novo gene prediction of the repeat-masked genome was performed using Genscan 1.0 [154], Augustus2.4 [151], GlimmerHMM version 3.0.3 [152], GeneID version 1.4 [ 155] and SNAP version 2006-07-28 [153]. Accordingly, the preliminary gene models were built. GeMoMa version 1.3.1 [ 156] was used in homology-based prediction and to predict the exact gene structure of tBlastn hits on genome. Protein sequences of *Drosophila melanogaster* and *Homo sapiens* were aligned to the genome using tBlastn. Gene model evidence from the above three methods were integrated by GLEAN to produce a high confidence gene-set. Gene models only supported by de novo evidence and with ≤150 bp coding sequence were filtered out. We assigned gene functions based on the best hits of the alignments against databases using BLASTN (E-value = 1e-5), including the National Center for Biotechnology Information non-redundant protein (Nr) [157] database and non-redundant nucleotide sequence (Nt) database. Furthermore, putative gene functions were searched against SwissProt Release 2015_01 and TrEMBL Release 2015_01 [158] databases using BLASTP by a cut-off E-value = 1e-5. Gene domains and motifs were identified by InterProScan version 5.14–53 [159] against protein databases, including Pfam version 27, PRINTS, ProSiteProfiles and SMART. Hits of InterPro entries were assigned to each gene with GO terms. All genes were searched against KEGG [160]proteins.

#### Prediction of non-coding RNA

The prediction of rRNA sequences was performed by blastn against Rfam database version 12.0 [161] with E-value = 1e-5. The software tRNAscan-SE [162] with eukaryote parameters was used to identify tRNA positions. The miRNAs and snRNAs were predicted by software Infernal [163] using the default parameters.

### Transcriptome sequencing

The samples of different developmental stages (12 h-eggs, 36 h-eggs, larvae, nymphs and adults) of *N. cucumeris* were collected from lab culture which is a susceptible strain maintained on acarid mite *Aleuroglyphus ovatus* in our laboratory for over ten years. For each developmental stage, more than 3000 individuals were sampled and then immediately frozen in liquid nitrogen. Total RNA was isolated using the Trizol reagent (Invitrogen, USA) following the instruction. RNA integrity and concentration were checked using an Agilent 2100 Bioanalyzer (Agilent Technologies, Inc., Santa Clara, CA, USA). RNA-seq libraries were constructed and then sequenced on Illumina Hiseq 2500 platform (2 × 125 bp). Raw reads were filtered with a base quality cutoff of 20.

Clean reads were aligned onto *N. cucumeris* genome assembly using Tophat 2 [164] to perform reference-guided mapping. Next, the transcripts were assembled using Cufflinks version 2.2.1 [164]. Cuffquan and CuffnormGene modules of Cufflinks were used to quantify expression levels of individual genes and normalized FPKM values (Fragments Per Kilobase of transcript per Million fragments mapped) were used as the measurement given trancriptome volume of each tissue and length of each gene. Genes with an absolute fold-change cutoff of 2 and *P*-value < 0.05 were regarded as differentially expressed genes (DEG). All DEGs were mapped against GO terms and KEGG pathways and gene numbers were obtained for each GO term or KEGG pathway.

### Comparative genomics

#### Data collection

For *Sarcoptes scabiei*, *Stegodyphus mimosarum*, *Metaseiulus occidentalis* and *Limulus polyphemus*, their predicted protein data were collected from NCBI (ftp://ftp.ncbi.nlm.nih.gov/). For *Tetranychus urticae* and *Ixodes scapularis*, their predicted protein data were downloaded from ENSEMBL (ftp://ftp.ensemblgenomes.org/). We downloaded *Mesobuthus martensii* predicted protein data from http://lifecenter.sgst.cn/main/en/scorpion.jsp. We thank Dr. M.I. Bellgard for *Rhipicephalus microplus* predicted protein data.

#### Orthologous clustering

OrthoMCL [165], which implements a Markov Cluster algorithm (MCL), was used to perform clustering of gene families. Firstly, the sequences with quite short length or high stop codon percent were removed from the input protein sequences. All-against-all BLASTP comparisons of proteins were performed with *P*-value cutoff 1e-5. The result gave an overview of sequence similarity which indicates the degree of conservation. Next, we filtered the BLASTP results using thresholds with e-value <1e-5 and percent match length ≥ 50% (50% of all possible pairs within the group that are matched through BLAST). Genes with coding region shorter than 300 bp were removed. With the OrthoMCL pairs program, we found potential inparalog, ortholog and co-ortholog pairs. Last, these pairs were grouped based on their relationships using the MCL program.

#### Constructing phylogenetic tree

Based on the OrthoMCL clustering results, the proteins of each single copies from nine species were aligned using MUSCLE v3.6 [166]. Then the poorly aligned regions were removed. After that, the 130 truncated sequences were concatenated to one super-gene for each species. The super-genes of nine species were used to construct the phylogenetic tree.

The phylogenetic tree was calculated using maximum likelihood (ML) algorithms of PhyML 4.0 [167, 168]. In terms of the substitution model, the JTT amino acid substitution model was selected and the parameter equilibrium frequency was empirical 0.00 and 4 were set as the parameters: the proportion of invariable sites and the number of substitution rate categories, respectively. We used a discrete gamma distribution to handle site evolution rate variation. Pertaining to tree searching, BIONJ served as the starting tree. We chose SPR (subtree pruning and regrafting) to find better tree topology and branch lengths. In regard to branch support, we used SH (Shimodaira–Hasegawa)-like procedure and with 100 bootstrap samples, respectively. We did not perform approximate likelihood ratio test.

Furthermore, a phylogenetic tree with 1000 bootstraps was constructed. To check the robustness of the phylogenetic tree, the tree was reconstructed using two strategies. One strategy is to increase the number of orthologues. Given the low number of single-copy orthologues, both single and multi-copy orthologues were used for reconstructing the tree. For each multi-copy orthologue set, one gene for each species was chosen according to the sequence similarity to the gene in *N. cucumeris*. 1262 orthologues were used for constructing the tree using PhyML4.0 and bootstrap values were added. Another is to construct the phylogenetic tree using RAxML with JTT model.

Each orthologue set were aligned using MUSCLE v3.6 and then concatenated into one super-gene sequence.

#### Estimation of divergence time

Both CODEML and MCMCTREE, implemented in the PAML version 4 package [169], were used to estimate the divergence time.

We used CODEML to estimate substitution rates between the orthologous pairs. The substitution rates of each branch on the tree were set and branch rates were then computed based on protein sequences. We chose the global clock, which means the rates of all branches are same. Meanwhile, the ambiguity characters and alignment gaps were removed.

MCMCTREE was used to estimate the divergence time based on the Bayesian Markov chain Monte Carlo algorithm. Calibration time from the TimeTree database (http://www.timetree.org) was obtained. We chose the relaxed-clock model (clock = 2) to provide a loose upper bound (RootAge), the maximal time constraint. We used *Limulus polyphemus* as an outgroup taxon, and chose the most likely *Limulus polyphemus*-arachnida split 490 MYA as one fossil calibration, the soft upper bound. Spider-scorpion split 397 MYA [60] served as another fossil calibration, the soft lower bound. We set the lower bound ranging from 370.0 to 420.0 MYA. The TimeTree database gives one *Limulus polyphemus*-arachnida split time-490 MYA and four references give 4 different divergence time (437.3 MYA [170], 520.8 MYA [171], 445.0 MYA [30], and 510.5 MYA [172]), so we set the scope of 468.0–520.0 MYA to *Limulus polyphemus*-arachnida split. The most likely split time (estimated by TimeTree database) affects the choice of split range. The bounds are soft, namely, the node age is at 95% confidence interval. The overall average substitution rate is 0.1 per site per 100 MYA, namely, 1e-8 substitutions per site per year. Calibration distribution and the RootAge Constraint as well as the birth-death process (birth rate 1 and death rate 1) were used. The program discarded the first 50,000 iterations (burnin = 50,000), and then perform MCMC for 50 (sampfreq = 50) × 10,000 (nample = 10,000), sampling every 10,000 iterations. Finally, the split time were deduced with the most possible time and a range.

#### Gene family expansion and contraction

We used CAFE version 3.0 [173] to analyze the evolution of gene family size in *N. cucumeris* and its related species with the above branch-length added phylogenetic tree. Using a probabilistic graphic model (*P*-value), the software can infer the ancestral gene family sizes. The smaller the *P*-value of a gene family is, the more dramatic changes the family shows. The *P*-value threshold was set to 0.01. The global lambda was specified as 0.002, which indicates the probability of gene gain and loss per gene per unit time in the phylogenetic tree. Finally, the expanded and contracted gene families were predicted by using the Viterbi algorithm.

### Gene family and pathway analysis

#### Sex determination pathway

The *Dsx*, *dmrt*, *Tra* and *Tra2* gene sequences predicted in the western orchard predatory mite *Metaseiulus occidentalis* [88] were downloaded from the NCBI protein database. TBLASTN searches were performed using these queries. Three *Dsx* and two *dmrt* candidate genes were annotated in the *N. cucumeris* genome by PFAM database, respectively, and then each gene was assigned a PF00751.13 (DM DNA binding domain). Finally, these genes were manually corrected with RNA-seq data.

To test the reliability of the gene predictions, phylogenetic analysis of each gene was performed. The revised protein sequences of *N. cucumeris* and queries were aligned using MUSCLE v3.6 [166]. The poorly aligned regions were eliminated by GBlocks (version 0.91b) [174] with default parameters. ProteinModelSelection.pl of RAxML (version 8.2.9) [175] was used to determine the optimal substitution model. RAxML was used to construct the maximum likelihood phylogenetic tree. If the high divergence of sequences between *N. cucumeris* and homologs in other species (evaluated by percentage of the retained amino acid number after GBlocks elimination, parameters: -t = p -b5 = h) were detected, the positions containing gaps or missing data were retained and a reanalysis of 100 bootstrap replicates was used to test the robustness of phylogenies.

Other key genes in sex determination cascade were identified through TBALSTN search with insect homolog sequences as queries. E-value and identity were used to filter the genes. Domains were annotated with PFAM database. These genes identified in *N. cucumeris* were manually corrected and phylogenetic analysis was performed.

#### Limb gap genes distal-less (Dll), dachshund (dac) extradenticle (exd) and homothorax (hth)

The reported *Dll*, *dac*, *exd* and *hth* gene sequences in Arachnida and Hexapoda were downloaded from NCBI protein database. TBLASTN searches were performed using these queries. The possible loci were manually corrected with RNA-seq data to extract the exact gene structure. Domains of the revised sequences were annotated. Phylogenetic analysis was performed as previously described.

#### Hox gene family analysis

The reported *hox* gene sequences in *Apis mellifera*, *Tribolium castaneum*, *Drosophila melanogaster*, *Ixodes scapularis* and *Tetranychus urticae* (downloaded from NCBI protein database*)* were aligned against the *N. cucumeris* genome using tblastn. GeMOMA [176] was used to identify the possible loci. Since the large evolutionary divergence of HOX sequences between Hexapoda and Arachnida, homology-based prediction may be insufficient to predict possible loci. We considered all loci predicted by the reported hexapoda and chelicerate hox sequences and ranked them by score assigned by tblastn. RNA sequencing reads were aligned to the *N. cucumeris* assembly using Tophat2 and the bam files storing alignment sequence data were thus obtained. The boundaries of exon-intron of these loci were manually checked using transcriptome bam files. The same approach was used to identify and check *hox* genes in other arachnid genomes. Phylogenetic analysis was performed as previously described.

#### Gustatory receptor (GR) and ionotropic receptor (IR) gene family analysis

The reported GR genes in *M. occidentalis* [39], *T. urticae* [ 123], *I. scapularis* (from NCBI protein database) and *D. melanogaster* (from NCBI protein database) were used as queries in tblastn searches against the *N. cucumeris* genome with e-value 1e-2. 7tm_7 (PF08395.7, 7tm Chemosensory receptor) domain is common in arthropod GRs. Either Lig_chan-Glu_bd (PF10613.4, Ligated ion channel L-glutamate- and glycine-binding site) or Lig_chan (PF00060.21, Ligand-gated ion channel) were detectable in IRs. The sequences of GRs and IRs are highly divergent. We combined sequencing similarity analyzed by TBLASTN and domain annotated using PFAM to recognize GRs and IRs. The identified genes in *N. cucumeris* were aligned with above queries and phylogenetic analysis was performed as previously described.

#### Glutathione-S-Transferase (GST), carboxyl/cholinesterase (CCE), cytochrome P450 (CYP) gene family analysis

Identified GST, CCE and CYP sequences in *M. occidentalis* [126], *T. urticae* [52], *D. melanogaster* (from NCBI protein database) and *I. scapularis* (from NCBI protein database) were used to search for GSTs, CCEs and CYPs in *N. cucumeris* with tblastn. Domains were annotated with PFAM database. The identified genes in *N. cucumeris* were aligned with above queries and phylogenetic analysis was performed as previously described.

## Supplementary information

**Additional file 1: Tables S1-S34.**

**Additional file 2: Figures S1-S7.**

## Data Availability

The data generated or analyzed during this study are included in this article and its supplementary information files. The genome assembly has been deposited in the NCBI under accession PRJNA549381. The sequence, aligned sequences and phylogenetic tree text can be found in the TreeBASE from the link http://purl.org/phylo/treebase/phylows/study/TB2:S25342.

## Abbreviations

AMP: antimicrobial peptide
BIONJ: Bio neighbor-joining
BUSCO: Benchmaking Universal Single-Copy Orthologues
BWA: Burrows-Wheeler Aligner in 778
CCE: Carboxyl/Cholinesterase
CEG: Conserved Eukaryotic Genes
COG: Clusters of Orthologous Groups of proteins
CSP: Chemosensory Protein
CYP: Cytochrome P450
DEG: Differentially Expressed Genes
FPKM: Fragments Per Kilobase of transcript per Million fragments mapped
FRP: Fibrinogen-Binding Adhesion Protein
GeMoMa: Gene Model Mapper
GO: Gene Ontology
GR: Gustatory Receptor
GST: Glutathione-S-Transferase
HSP: Heat Shock Protein
IR: Ionotropic Receptor
JAK/STAT: Janus Kinase/Signal Transducers and Activators of Transcription
JJT: Jones–Taylor–Thornton
JNK: c-Jun N-terminal Kinase
KEGG: Kyoto Encyclopedia of Genes and Genomes
MCL: Markov Cluster Algorithm
MEGA: Molecular evolutionary genetics analysis
ML: Maximum Likehood
MYA: Million Years Ago
NR: Non-Redundant proteins
OBP: Odorant-Binding Protein
PASA: Program to Assemble Spliced Alignments
Pfam: protein families database
PGE: Paternal Genome Elimination
PGRP: Peptidoglycan Recognition Protein
RAxML: Randomized Axelerated Maximum Likelihood
Rfam: RNA families
SMART: Simple Modular Architecture Research Tool
TE: Transposable elements
TEP: Thioester-containing Protein
TF: Transcription Factor
TLR: Toll-like Receptor
TrEMBL: Translated EMBL Nucleotide data base
TRP: Transient Receptor Potential
ZFP: Zinc Finger Protein

## References

[1] Zhang ZQ (2013). Animal biodiversity: an update of classification and diversity in 2013. Zootaxa.

[2] Zhang ZQ (2013). Phylum Arthropoda. Zootaxa.

[3] Lindquist EE. Current theories on the evolution of major groups of Acari and on their relationships with other groups of Arachnida, with consequent implications for their classificaton. *In*: Griffiths DA, Bowman CE. editors. Acarology VI. Volume I. Chichester: Ellis-Horwood Ltd.; 1984. p. 28–62.

[4] Krantz GW (1978). A manual of acarology.

[5] Walter DE, Proctor HC (1999). Mites ecology, evolution and behaviour.

[6] Halliday RB, OConnor BM, Baker AS, Raven PH, Williams T (2000). Global diversity of mites. Nature and human society.

[7] Larsen BB, Miller EC, Rhodes MK, Wiens JJ (2017). Inordinate fondness multiplied and redistributed: the number of species on Earth and the new pie of life. Q Rev Biol.

[8] Misof B (2014). Phylogenomics resolves the timing and pattern of insect evolution. Science.

[9] Dunlop JA (2010). Geological history and phylogeny of Chelicerata. Arthropod Struct Dev.

[10] Sharma PP (2014). Phylogenomic interrogation of Arachnida reveals systemic conflicts in phylogenetic signal. Mol Biol Evol.

[11] Dunlop JA, Alberti G (2008). The affinities of mites and ticks: a review. J Zool Syst Evol Res.

[12] Bekker EG. Concerning the Acarina as a normal grouping. Trud Sci Conf Parasito USSR. 1959;46–51 [in Russian].

[13] Krantz GW (1970). A manual of acarology.

[14] Lindquist EE. Origins and phylogenetic relationships. *In*: Krantz GW, Walter DE. (Eds.) A manual of acarology. third ed. Texas Tech University Press Lubbock; 2009. p. 3–4.

[15] Lindquist EE, Krantz GW, Walter DE, Krantz GW, Walter DE (2009). Classification. A manual of acarology.

[16] Shultz JW (1990). Evolutionary morphology and phylogeny of Arachnida. Cladistics.

[17] Wheeler WC, Hayashi CY (1998). The phylogeny of the extant chelicerate orders. Cladistics.

[18] Giribet G (2002). Phylogeny and systematic position of Opiliones: a combined analysis of chelicerate relationships using morphological and molecular data. Cladistics.

[19] Klompen H, Lekveishvili M, Black WC (2007). Phylogeny of parasitiform mites (Acari) based on rRNA. Mol Phylogenet Evol.

[20] Grandjean F (1935). Observations sur les Acariens. Bull Mu Natl Hist Nat.

[21] Andre M, Lamy E (1937). Les Idees actuelles sur la phylogenie des Aacariens.

[22] Zakhvatkin AA (1952). The division of the Acarina into orders and their position in the system of the Chelicerata. Parazitol Sborn.

[23] Dubinin VB (1957). New system of the superclass Chelicerata. Bulletin de la Societedes Naturalistes de Moscow. Biologie.

[24] Dubinin VB, Rodendorf BB (1962). Class Acaromorpha: mites of gnathosomic chelicerate arthropods. Fundamentals of palaeontology.

[25] van der Hammen L (1972). A revised classification of the mites (Arachnidea, Acarida) with diagnoses, a key, and notes on phylogeny. Zool Meded.

[26] van der Hammen L (1977). A new classification of the Chelicerata. Zool Meded.

[27] van der Hammen L (1989). An Introduction to Comparative Arachnology.

[28] Boudreaux HB (1979). Arthropod Phylogeny with Special References to Insects.

[29] Alberti G, Michalik P (2004). Feinstrukturelle Aspekte der Fortpflanzungssysteme von Spinnentieren (Arachnida). Denisia.

[30] Dabert M (2010). Molecular phylogeny of acariform mites (Acari, Arachnida): strong conflict between phylogenetic signal and long-branch attraction artifacts. Mol Phylogenet Evol.

[31] Pepato AR, da Rocha CE, Dunlop JA (2010). Phylogenetic position of the acariform mites: sensitivity to homology assessment under total evidence. BMC Evol Biol.

[32] Arabi J (2012). Nucleotide composition of *CO1* sequences in Chelicerata (Arthropoda): detecting new mitogenomic rearrangements. J Mol Evol.

[33] Rota-Stabelli O, Daley AC, Pisani D (2013). Molecular timetrees reveal a Cambrian colonization of land and a new scenario for ecdysozoan evolution. Curr Biol.

[34] Dunlop J, Borner J, Burmester T, Wägele JW, Bartolomaeus T (2014). Phylogeny of the Chelicerates: morphological and molecular evidence. Deep metazoan phylogeny: the backbone of the tree of life.

[35] Pepato AR, Klimov PB (2015). Origin and higher-level diversification of acariform mites – evidence from nuclear ribosomal genes, extensive taxon sampling, and secondary structure alignment. BMC Evol Biol.

[36] Xue XF (2016). Mitochondrial genome evolution and tRNA truncation in Acariformes mites: new evidence from eriophyoid mites. Sci Rep.

[37] Xue XF (2017). The phylogenetic position of eriophyoid mites (superfamily Eriophyoidea) in Acariformes inferred from the sequences of mitochondrial genomes and nuclear small subunit (18S) rRNA gene. Mol Phylogenet Evol.

[38] Klimov PB, OConnora BM, Chetverikovc PE, Boltond SJ, Pepato AR, Mortazavia AL, Tolstikovb AV, Bauchan GR, Ochoa R (2018). Comprehensive phylogeny of acariform mites (Acariformes) provides insights on the origin of the four-legged mites (Eriophyoidea), a long branch. Mol Phylogenet Evol.

[39] Hoy MA (2016). Genome sequencing of the phytoseiid predatory mite *Metaseiulus occidentalis* reveals completely atomized Hox genes and superdynamic intron evolution. Genome Biol Evol.

[40] Dong X (2017). Draft genome of the honey bee ectoparasitic mite, *Tropilaelaps mercedesae*, is shaped by the parasitic life history. Gigascience.

[41] Ballesteros Jesús A, Sharma Prashant P (2019). A Critical Appraisal of the Placement of Xiphosura (Chelicerata) with Account of Known Sources of Phylogenetic Error. Systematic Biology.

[42] Vizueta J, Rozas J, Sánchez-Gracia A (2018). Comparative genomics reveals thousands of novel chemosensory genes and massive changes in chemoreceptor repertories across Chelicerates. Genome Biol Evol.

[43] Van Dam MH, Trautwein M, Spicer GS, Esposito L (2019). Advancing mite phylogenomics: Designing ultraconserved elements for Acari phylogeny. Mol Ecol Resour.

[44] van Lenteren JC (2012). The state of commercial augmentative biological control: plenty of natural enemies, but a frustrating lack of uptake. Biocontrol.

[45] Knapp M (2018). Use of predatory mites in commercial biocontrol: current status and future prospects. Acarologia.

[46] Palevsky E, Gerson U, Zhang ZQ (2013). Can exotic phytoseiids be considered ‘benevolent invaders’ in perennial cropping systems?. Exp Appl Acarol.

[47] Patel K, Zhang ZQ (2017). Functional and numerical responses of *Amblydromalus limonicus* and *Neoseiulus cucumeris* to eggs and first instar nymph of tomato/potato psyllid (*Bactericera cockerrelli*). Syst Appl Acarol.

[48] Simão FA, Waterhouse RM, Ioannidis P, Kriventseva EV, Zdobnov EM (2015). BUSCO: assessing genome assembly and annotation completeness with single-copy orthologs. Bioinformatics.

[49] Barrero RA (2017). Gene-enriched draft genome of the cattle tick *Rhipicephalus microplus*: assembly by the hybrid Pacific Biosciences/Illumina approach enabled analysis of the highly repetitive genome. Int J Parasitol.

[50] Cornman SR (2010). Genomic survey of the ectoparasitic mite *Varroa destructor*, a major pest of the honey bee *Apis mellifera*. BMC Genomics.

[51] Gulia-Nuss M (2015). Genomic insights into the *Ixodes scapularis* tick vector of Lyme disease. Nat Commun.

[52] Grbić M (2011). The genome of *Tetranychus urticae* reveals herbivorous pest adaptations. Nature.

[53] Schoville SD, Chen YH, Andersson MN, Benoit JB, Bhandari A, Bowsher JH, Brevik K, Cappelle K, Chen MM, Childers AK (2018). A model species for agricultural pest genomics: the genome of the Colorado potato beetle, *Leptinotarsa decemlineata* (Coleoptera: Chrysomelidae). Sci Rep.

[54] Laetsch DR, Blaxter ML (2017). BlobTools: Interrogation of genome assemblies. F1000Research.

[55] Zhou Y, Cahan SH (2012). A novel family of terminal-repeat retrotransposon in miniature (TRIM) in the genome of the red harvester ant, *Pogonomyrmex barbatus*. PLoS One.

[56] Lin X, Faridi N, Casola C (2016). An ancient transkingdom horizontal transfer of penelope-like retroelements from arthropods to conifers. Genome Biol Evol.

[57] Piednoël M, Gonçalves IR, Higuet D, Bonnivard E (2011). Eukaryote DIRS1-like retrotransposons: an overview. BMC Genomics.

[58] Rider SD, Morgan MS, Arlian LG (2015). Draft genome of the scabies mite. Parasit Vectors.

[59] Bernini F, Carnevale G, Bagnoli G, Stouge S, Bernini F, Nannelli R, Nuzzaci G, de Lillo E (2002). An early Ordovician mite (Acari: Oribatida) from the island of Öland, Sweden. Acarid phylogeny and evolution: adaptation in mites and ticks.

[60] Jeyaprakash A, Hoy MA (2009). First divergence time estimate of spiders, scorpions, mites and ticks (subphylum: Chelicerata) inferred from mitochondrial phylogeny. Exp Appl Acarol.

[61] Peñalver E (2017). Ticks parasitised feathered dinosaurs as revealed by *Cretaceous amber* assemblages. Nat Commun.

[62] Dunlop JA, de Oliveira Bernardi LF (2014). An opilioacarid mite in Cretaceous Burmese amber. Naturwissenschaften.

[63] Chant DA (1958). Immature and adult stages of some British Phytoseiidae Berl., 1916 (Acarina). J Linn Soc Lond Zool.

[64] Hoy MA (1985). Recent advances in genetics and genetic improvement of the Phytoseiidae. Annu Rev Entomol.

[65] Carr AL, Roe M (2016). Acarine attractants: Chemoreception, bioassay, chemistry and control. Pestic Biochem Physiol.

[66] Kawakami Y, Goto SG, Ito K, Numata H (2009). Suppression of ovarian development and vitellogenin gene expression in the adult diapause of the two-spotted spider mite *Tetranychus urticae*. J Insect Physiol.

[67] Mitchell RD (2007). Molecular characterization, tissue-specific expression and RNAi knockdown of the first vitellogenin receptor from a tick. Insect Biochem Mol Biol.

[68] Zhao Y (2014). Food source affects the expression of vitellogenin and fecundity of a biological control agent, *Neoseiulus cucumeris*. Exp Appl Acarol.

[69] Gilbert LI, Granger NA, Roe RM (2000). The juvenile hormones: historical facts and speculations on future research directions. Insect Biochem Mol Biol.

[70] Goto SG (2016). Physiological and molecular mechanisms underlying photoperiodism in the spider mite: comparisons with insects. J Comp Physiol B.

[71] Cabrera AR, Donohue KV, Roe RM (2009). Regulation of female reproduction in mites: a unifying model for the Acari. J Insect Physiol.

[72] Ogihara MH (2015). Ovarian ecdysteroidogenesis in both immature and mature stages of an Acari, *Ornithodoros moubata*. PLoS One.

[73] Qu Z (2015). How Did Arthropod Sesquiterpenoids and Ecdysteroids Arise? Comparison of hormonal pathway genes in noninsect arthropod genomes. Genome Biol Evol.

[74] Lim J (2014). The octopamine receptor Octβ2R regulates ovulation in *Drosophila melanogaster*. PLoS One.

[75] Cole SH (2005). Two functional but noncomplementing *Drosophila* tyrosine decarboxylase genes: distinct roles for neural tyramine and octopamine in female fertility. J Biol Chem.

[76] Roeder T (2005). Tyramine and octopamine: ruling behavior and metabolism. Annu Rev Entomol.

[77] Wu K, Hoy MA (2014). Clathrin heavy chain is important for viability, oviposition, embryogenesis and, possibly, systemic RNAi response in the predatory mite *Metaseiulus occidentalis*. PLoS One.

[78] Zhou J (2006). Identification of a follistatin-related protein from the tick *Haemaphysalis longicornis* and its effect on tick oviposition. Gene.

[79] Newquist G (2013). Control of male and female fertility by the netrin axon guidance genes. PLoS One.

[80] Ross L, Shuker DM, Pen I (2011). The evolution and suppression of male suicide under paternal genome elimination. Evolution.

[81] Blackmon H, Hardy NB, Ross L (2015). The evolutionary dynamics of haplodiploidy: Genome architecture and haploid viability. Evolution.

[82] Nagelkerke CJ, Sabelis MW (1998). Precise control of sex allocation in pseudo-arrhenotokous phytoseiid mites. J Evol Biol.

[83] Bongiorni S (2009). Epigenetic marks for chromosome imprinting during spermatogenesis in coccids. Chromosoma.

[84] Schaefer M (2010). RNA methylation by Dnmt2 protects transfer RNAs against stress-induced cleavage. Genes Dev.

[85] Lyko F, Ramsahoye BH, Jaenisch R (2000). DNA methylation in *Drosophila melanogaster*. Nature.

[86] Takayama S (2014). Genome methylation in *D. melanogaster* is found at specific short motifs and is independent of DNMT2 activity. Genome Res.

[87] Breiling A, Lyko F (2015). Epigenetic regulatory functions of DNA modifications: 5-methylcytosine and beyond. Epigenetics Chromatin.

[88] Pomerantz AF, Hoy MA, Kawahara AY (2015). Molecular characterization and evolutionary insights into potential sex-determination genes in the western orchard predatory mite *Metaseiulus occidentalis* (Chelicerata: Arachnida: Acari: Phytoseiidae). J Biomol Struct Dyn.

[89] Geuverink E, Beukeboom LW (2014). Phylogenetic distribution and evolutionary dynamics of the sex determination genes doublesex and transformer in insects. Sex Dev.

[90] Xu J (2017). *Bombyx mori* P-element somatic inhibitor (BmPSI) is a key auxiliary factor for silkworm male sex determination. PLoS Genet.

[91] Verhulst EC, van de Zande L, Beukeboom LW (2010). Insect sex determination: it all evolves around transformer. Curr Opin Genet Dev.

[92] Torres M, Sánchez L (1992). The segmentation gene *runt* is needed to activate Sex-lethal, a gene that controls sex determination and dosage compensation in *Drosophila*. Genet Res.

[93] Hoshijima K (1995). Transcriptional regulation of the *Sex-lethal* gene by helix-loop-helix proteins. Nucleic Acids Res.

[94] Garrett-Engele CM (2002). Intersex, a gene required for female sexual development in *Drosophila*, is expressed in both sexes and functions together with doublesex to regulate terminal differentiation. Development.

[95] Schmieder S, Colinet D, Poirié M (2012). Tracing back the nascence of a new sex-determination pathway to the ancestor of bees and ants. Nat Commun.

[96] Privman E, Wurm Y, Keller L (2013). Duplication and concerted evolution in a master sex determiner under balancing selection. Proc Biol Sci.

[97] Barnett AA, Thomas RH (2012). The delineation of the fourth walking leg segment is temporally linked to posterior segmentation in the mite *Archegozetes longisetosus* (Acari: Oribatida, Trhypochthoniidae). Evol Dev.

[98] Barnett AA, Thomas RH (2013). Posterior Hox gene reduction in an arthropod: *Ultrabithorax* and *Abdominal-B* are expressed in a single segment in the mite *Archegozetes longisetosus*. Evodevo.

[99] Heffer A, Xiang J, Pick L (2013). Variation and constraint in *Hox* gene evolution. Proc Natl Acad Sci U S A.

[100] Pick L (2016). *Hox* genes, *evo-devo*, and the case of the *ftz* gene. Chromosoma.

[101] Vaquerizas JM (2009). A census of human transcription factors: function, expression and evolution. Nat Rev Genet.

[102] Nadimpalli S, Persikov AV, Singh M (2015). Pervasive variation of transcription factor orthologs contributes to regulatory network evolution. PLoS Genet.

[103] Lorick KL (1999). RING fingers mediate ubiquitin-conjugating enzyme (E2)-dependent ubiquitination. Proc Natl Acad Sci U S A.

[104] Aravind L (2000). The BED finger, a novel DNA-binding domain in chromatin-boundary- element- binding proteins and transposases. Trends Biochem Sci.

[105] Gocke CB, Yu H (2008). ZNF198 stabilizes the LSD1-CoREST-HDAC1 complex on chromatin through its MYM-type zinc fingers. PLoS One.

[106] Huang X (2009). Structure and function of the two tandem WW domains of the pre-mRNA splicing factor FBP21 (formin-binding protein 21). J Biol Chem.

[107] He G, Sun D, Ou Z, Ding A (2012). The protein Zfand5 binds and stabilizes mRNAs with AU-rich elements in their 3′-untranslated regions. J Biol Chem.

[108] Todorova T, Bock FJ, Chang P (2014). PARP13 regulates cellular mRNA post-transcriptionally and functions as a pro-apoptotic factor by destabilizing TRAILR4 transcript. Nat Commun.

[109] Siebel C, Lendahl U (2017). Notch signaling in development, tissue homeostasis, and disease. Physiol Rev.

[110] Han H (2016). Chi and dLMO function antagonistically on Notch signaling through directly regulation of *fng* transcription. Sci Rep.

[111] Guruharsha KG, Kankel MW, Artavanis-Tsakonas S (2012). The Notch signalling system: recent insights into the complexity of a conserved pathway. Nat Rev Genet.

[112] Robbins DJ, Fei DL, Riobo NA (2012). The Hedgehog signal transduction network. Sci Signal.

[113] Wilson CW, Chuang PT (2010). Mechanism and evolution of cytosolic Hedgehog signal transduction. Development.

[114] Meng Z, Moroishi T, Guan KL (2016). Mechanisms of Hippo pathway regulation. Genes Dev.

[115] Zou Z (2016). Effect of photoperiod on development and demographic parameters of *Neoseiulus barkeri* (Acari: Phytoseiidae) fed on *Tyrophagus putrescentiae* (Acari: Acaridae). Exp Appl Acarol.

[116] Tachi F, Osakabe M (2014). Spectrum-specific UV egg damage and dispersal responses in the phytoseiid predatory mite *Neoseiulus californicus* (Acari: Phytoseiidae). Environ Entomol.

[117] Okamoto N, Nishimori Y, Nishimura T (2012). Conserved role for the Dachshund protein with *Drosophila* Pax6 homolog Eyeless in insulin expression. Proc Natl Acad Sci U S A.

[118] Mishra AK (2016). Functional genomics identifies regulators of the phototransduction machinery in the *Drosophila* larval eye and adult ocelli. Dev Biol.

[119] Baker EK, Colley NJ, Zuker CS (1994). The cyclophilin homolog NinaA functions as a chaperone, forming a stable complex in vivo with its protein target rhodopsin. EMBO J.

[120] Sarfare S (2005). The *Drosophila ninaG* oxidoreductase acts in visual pigment chromophore production. J Biol Chem.

[121] Pelosi P (2014). Soluble proteins of chemical communication: an overview across arthropods. Front Physiol.

[122] Peñalva-Arana DC, Lynch M, Robertson HM (2009). The chemoreceptor genes of the waterflea *Daphnia pulex*: many Grs but no Ors. BMC Evol Biol.

[123] Ngoc PC (2016). Complex evolutionary dynamics of massively expanded chemosensory receptor families in an extreme generalist chelicerate herbivore. Genome Biol Evol.

[124] Pai TP (2013). *Drosophila* ORB protein in two mushroom body output neurons is necessary for long-term memory formation. Proc Natl Acad Sci U S A.

[125] Dermauw W (2013). A burst of ABC genes in the genome of the polyphagous spider mite *Tetranychus urticae*. BMC Genomics.

[126] Wu K, Hoy MA (2016). The glutathione-S-transferase, cytochrome P450 and carboxyl/cholinesterase gene superfamilies in predatory mite *Metaseiulus occidentalis*. PLoS One.

[127] Chen W (2015). Cloning and differential expression of five heat shock protein genes associated with thermal stress and development in the polyphagous predatory mite *Neoseiulus cucumeris* (Acari: Phytoseiidae). Exp Appl Acarol.

[128] Zhang GH (2014). Effects of thermal stress on lipid peroxidation and antioxidant enzyme activities of the predatory mite, *Neoseiulus cucumeris* (Acari: Phytoseiidae). Exp Appl Acarol.

[129] Shaposhnikov M (2015). Lifespan and stress resistance in *Drosophila* with overexpressed DNA repair genes. Sci Rep.

[130] Svetec N, Pavlidis P, Stephan W (2009). Recent strong positive selection on *Drosophila melanogaster* HDAC6, a gene encoding a stress surveillance factor, as revealed by population genomic analysis. Mol Biol Evol.

[131] Barnes VL (2014). SIN3 is critical for stress resistance and modulates adult lifespan. Aging (Albany NY).

[132] Nakajima E (2016). The Histone deacetylase gene *Rpd3* is required for starvation stress resistance. PLoS One.

[133] Palmer WJ, Jiggins FM (2015). Comparative genomics reveals the origins and diversity of Arthropod immune systems. Mol Biol Evol.

[134] Blandin S, Levashina EA (2004). Thioester-containing proteins and insect immunity. Mol Immunol.

[135] Sekiguchi R, Fujito NT, Nonaka M (2012). Evolution of the thioester-containing proteins (TEPs) of the arthropoda, revealed by molecular cloning of TEP genes from a spider, *Hasarius adansoni*. Dev Comp Immunol.

[136] Estévez-Lao TY, Hillyer JF (2014). Involvement of the *Anopheles gambiae* Nimrod gene family in mosquito immune responses. Insect Biochem Mol Biol.

[137] Lozano-Fernandez J, Tanner AR, Giacomelli M, Carton R, Vinther J, Edgecombe GD, Pisani D (2019). Increasing species sampling in chelicerate genomic-scale datasets provides support for monophyly of Acari and Arachnida. Nature Commun.

[138] Murray MG, Thompson WF (1980). Rapid isolation of high molecular weight plant DNA. Nucleic Acids Res.

[139] Li R (2010). The sequence and de novo assembly of the giant panda genome. Nature.

[140] Gnerre S (2011). High-quality draft assemblies of mammalian genomes from massively parallel sequence data. Proc Natl Acad Sci U S A.

[141] Boetzer M (2011). Scaffolding pre-assembled contigs using SSPACE. Bioinformatics.

[142] Luo R (2012). SOAPdenovo2: an empirically improved memory-efficient short-read de novo assembler. Gigascience.

[143] Jurka J (2005). Repbase Update, a database of eukaryotic repetitive elements. Cytogenet Genome Res.

[144] Han Y, Wessler SR (2010). MITE-Hunter: a program for discovering miniature inverted-repeat transposable elements from genomic sequences. Nucleic Acids Res.

[145] Xu Z, Wang H (2007). LTR_FINDER: an efficient tool for the prediction of full-length LTR retrotransposons. Nucleic Acids Res.

[146] Price AL, Jones NC, Pevzner PA (2005). De novo identification of repeat families in large genomes. Bioinformatics.

[147] Edgar RC, Myers EW (2005). PILER: identification and classification of genomic repeats. Bioinformatics.

[148] Tarailo-Graovac M, Chen N (2009). Using RepeatMasker to identify repetitive elements in genomic sequences. Curr Protoc Bioinformatics.

[149] Grabherr MG, Haas BJ, Yassour M, Levin JZ, Thompson DA, Amit I, Xian A, Fan L, Raychowdhury R, Zeng Q (2011). Trinity: reconstructing a full-length transcriptome without a genome from RNA-Seq data. Nat Biotechnol.

[150] Campbell MA, Haas BJ, Hamilton JP, Mount SM (2006). Buell CR Comprehensive analysis of alternative splicing in rice and comparative analyses with Arabidopsis. BMC Genomics.

[151] Stanke M, Waack S (2003). Gene prediction with a hidden Markov model and a new intron submodel. Bioinformatics.

[152] Majoros WH, Pertea M, Salzberg SL (2004). TigrScan and GlimmerHMM: two open source ab initio eukaryotic gene-finders. Bioinformatics.

[153] Korf I (2004). Gene finding in novel genomes. BMC Bioinformatics.

[154] Burge C, Karlin S (1997). Prediction of complete gene structures in human genomic DNA. J Mol Biol.

[155] Alioto T, Blanco E, Parra G, Guigó R (2018). Using geneid to identify genes. Curr Protoc Bioinformatics.

[156] Keilwagen J (2016). Using intron position conservation for homology-based gene prediction. Nucleic Acids Res.

[157] Marchler-Bauer A (2011). CDD: a Conserved Domain Database for the functional annotation of proteins. Nucleic Acids Res.

[158] Boeckmann B (2003). The SWISS-PROT protein knowledgebase and its supplement TrEMBL in 2003. Nucleic Acids Res.

[159] Zdobnov EM, Apweiler R (2001). InterProScan–an integration platform for the signature-recognition methods in InterPro. Bioinformatics.

[160] Kanehisa M, Goto S (2000). KEGG: Kyoto encyclopedia of genes and genomes. Nucleic Acids Res.

[161] Griffiths-Jones S (2005). Rfam: annotating non-coding RNAs in complete genomes. Nucleic Acids Res.

[162] Lowe TM, Eddy SR (1997). tRNAscan-SE: a program for improved detection of transfer RNA genes in genomic sequence. Nucleic Acids Res.

[163] Nawrocki EP, Eddy SR (2013). Infernal 1.1, 100-fold faster RNA homology searches. Bioinformatics.

[164] Trapnell C (2012). Differential gene and transcript expression analysis of RNA-seq experiments with TopHat and Cufflinks. Nat Protoc.

[165] Li L, Stoeckert CJ, Roos DS (2003). OrthoMCL: identification of ortholog groups for eukaryotic genomes. Genome Res.

[166] Edgar RC (2004). MUSCLE: multiple sequence alignment with high accuracy and high throughput. Nucleic Acids Res.

[167] Guindon S, Gascuel O (2003). A simple, fast, and accurate algorithm to estimate large phylogenies by maximum likelihood. Syst Biol.

[168] Guindon S (2010). New algorithms and methods to estimate maximum-likelihood phylogenies: assessing the performance of PhyML 3.0. Syst Biol.

[169] Yang Z (2007). PAML 4: phylogenetic analysis by maximum likelihood. Mol Biol Evol.

[170] Giribet G, Edgecombe GD (2012). Reevaluating the arthropod tree of life. Annu Rev Entomol.

[171] Sanders KL (2010). Phylogeny and divergence times of filesnakes (Acrochordus): inferences from morphology, fossils and three molecular loci. Mol Phylogenet Evol.

[172] Vidal N, Hedges SB (2009). The molecular evolutionary tree of lizards, snakes, and amphisbaenians. C R Biol.

[173] De Bie T (2006). CAFE: a computational tool for the study of gene family evolution. Bioinformatics.

[174] Talavera G, Castresana J (2007). Improvement of phylogenies after removing divergent and ambiguously aligned blocks from protein sequence alignments. Syst Biol.

[175] Stamatakis A (2014). RAxML version 8: a tool for phylogenetic analysis and post-analysis of large phylogenies. Bioinformatics.

[176] Keilwagen Jens, Wenk Michael, Erickson Jessica L., Schattat Martin H., Grau Jan, Hartung Frank (2016). Using intron position conservation for homology-based gene prediction. Nucleic Acids Research.

