# Supplementary material for: Genomic insights into mite phylogeny, fitness, development, and reproduction
Source: BMC Genomics. 2019 Dec 9;20:954. doi: 10.1186/s12864-019-6281-1 (PMC6902594; doi:10.1186/s12864-019-6281-1)
Supplement: Supplementary file 2 — Additional file 2: Figures S1-S7. [file 12864_2019_6281_MOESM2_ESM.pdf]

Zhang YX, Chen X, Wang JP, Zhang ZQ, Wei H, Yu HY, Zheng HK et al. (2019)

Genomic insights into mite phylogeny, fitness, development, and reproduction

BMC Genomics

DOI: 10.1186/s12864-019-6281-1

Additional file 2.

This file includes 7 supplementary figures:

Figure S1. Assessment of putative contaminants in the genome assembly of *N. cucumeris*. In bottom left panel, each circle represents the scaffold of *N. cucumeris* assembly and its radium is the result of logarithmical conversion of its length. The X-axis represents the GC content and the Y-axis represents the depth. In top left panel and bottom right panel, the bin is 0.01 and the length of scaffolds in each bin is summed. **Figure S2.** Phylogenetic trees. a. The maximum likelihood (ML)-based phylogenetic tree constructed with 1262 orthologs using PhyML4.0. b. The neighbor-joining (NJ)-based phylogenetic tree constructed using MEGA7.0. **Figure S3.** The ecdysteroid biosynthesis CYP450 genes identified in *N. cucumeris*. **Figure S4** The transformer and transformer-2 genes identified in *N. cucumeris*. **Figure S5.** The doublesex and DMRT genes identified in *N. cucumeris*. **Figure S6.** The four conserved limb gap genes identified in *N. cucumeris*. **Figure S7.** The chemoreception related receptor genes identified in *N. cucumeris*. (A): 24 gustatory receptor (GR) genes that can be clustered into three clans; and (B): 65 ionotropic receptor (IR) genes that can be clustered into four clans.

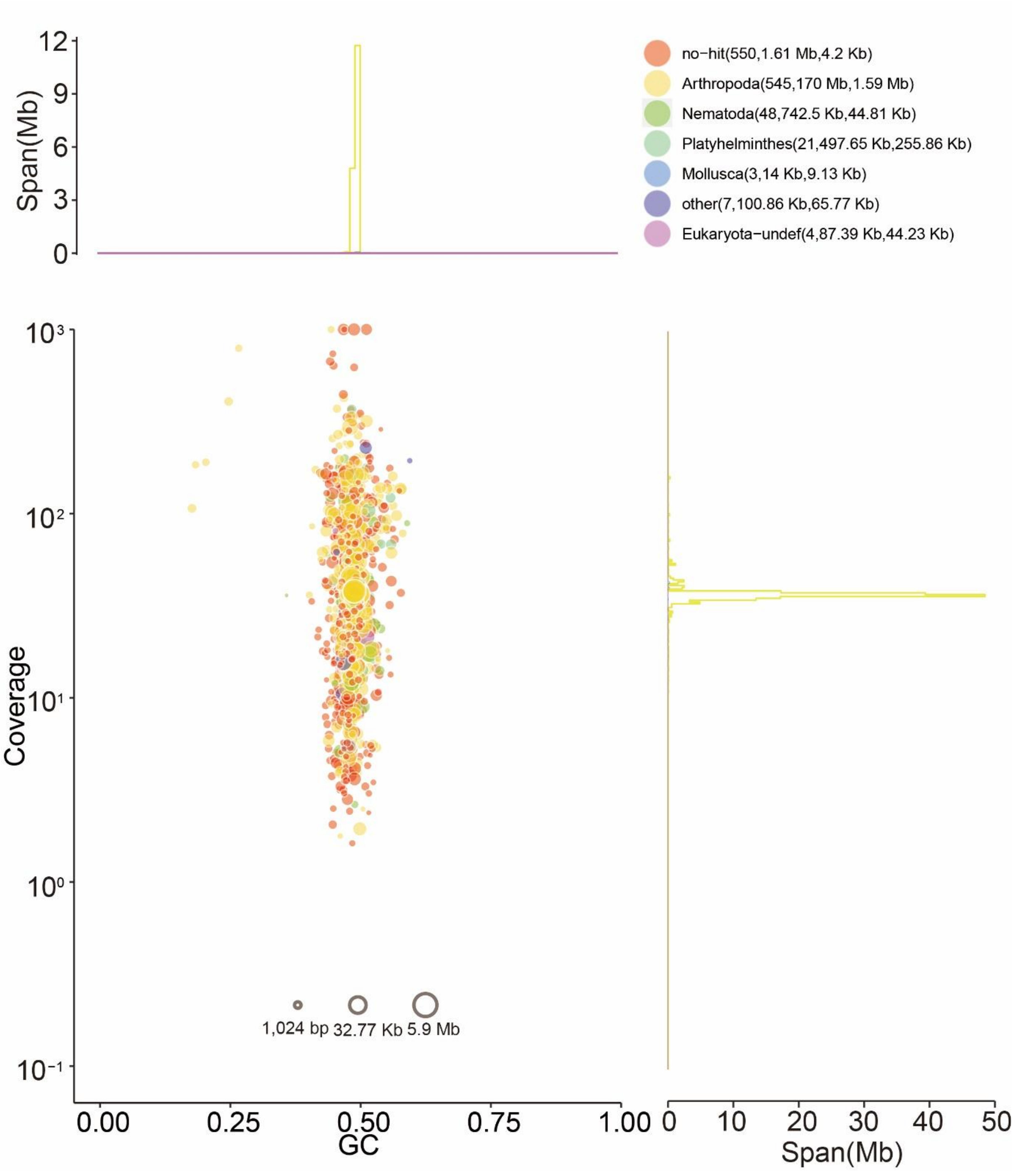

Fig. S1 Assessment of putative contaminants in the genome assembly of *N. cucumeris*. In bottom left panel, each circle represents the scaffold of *N. cucumeris* assembly and its radium is the result of logarithmical conversion of its length. The X-axis represents the GC content and the Y-axis represents the depth. In top left panel and bottom right panel, the bin is 0.01 and the length of scaffolds in each bin is summed.

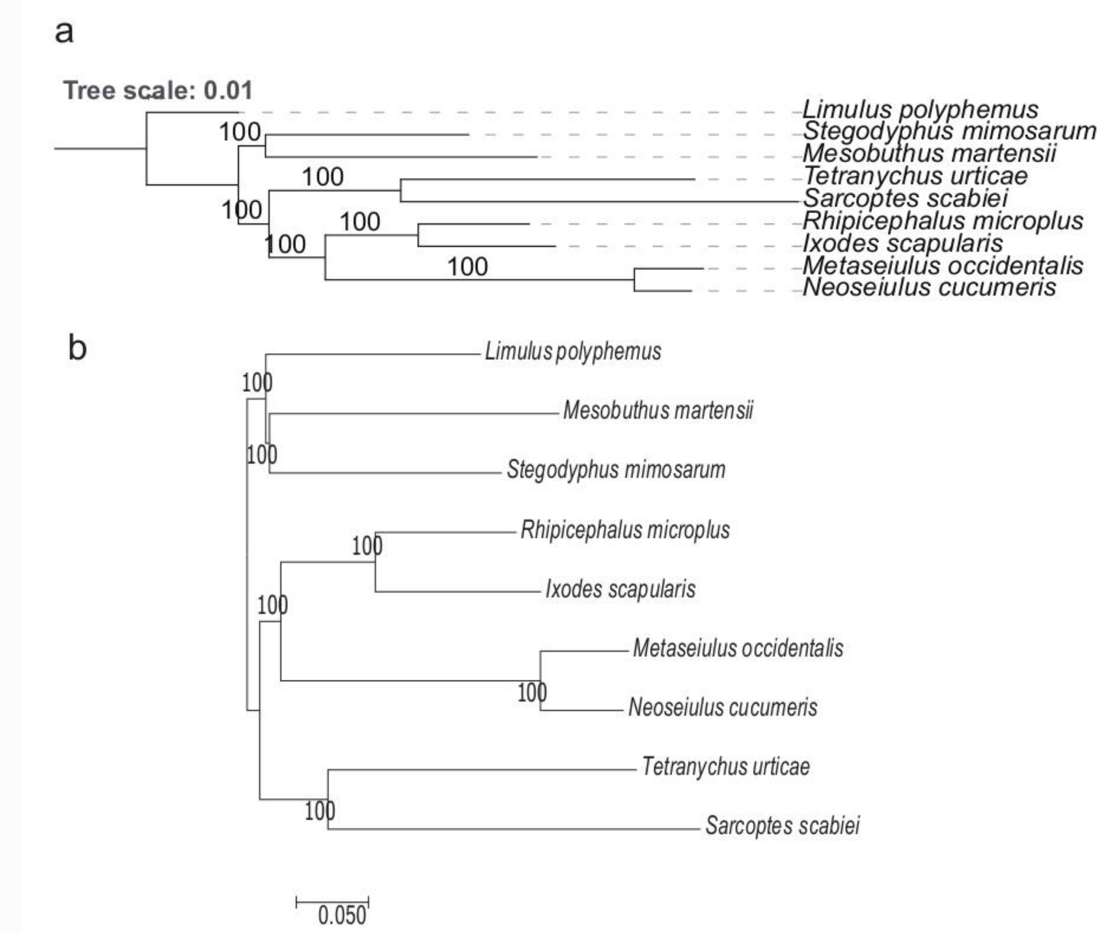

Fig S2 Phylogenetic trees. **a.** The maximum likelihood(ML)-based phylogenetic tree constructed with 1,262 orthologs using PhyML4.0. **b.** The neighbor-joining(NJ)-based phylogenetic tree constructed using MEGA7.0.

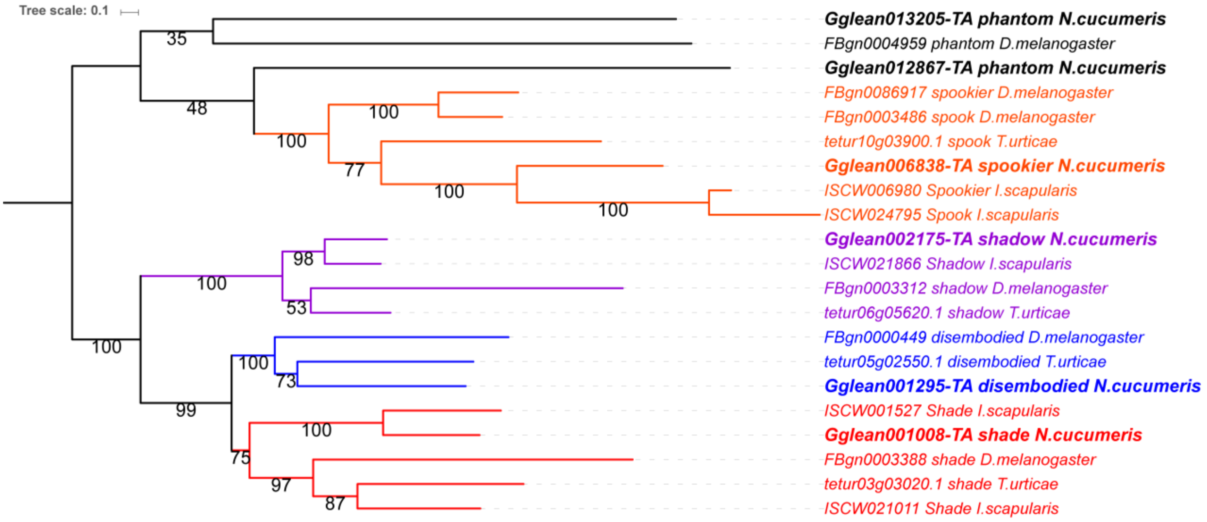

Fig. S3 The ecdysteroid biosynthesis *CYP450* genes identified in *N. cucumeris*

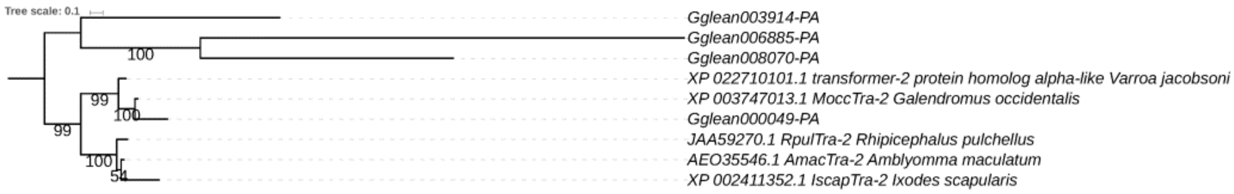

Fig. S4 The *transformer* and *transformer-2* genes identified in *N. cucumeris*

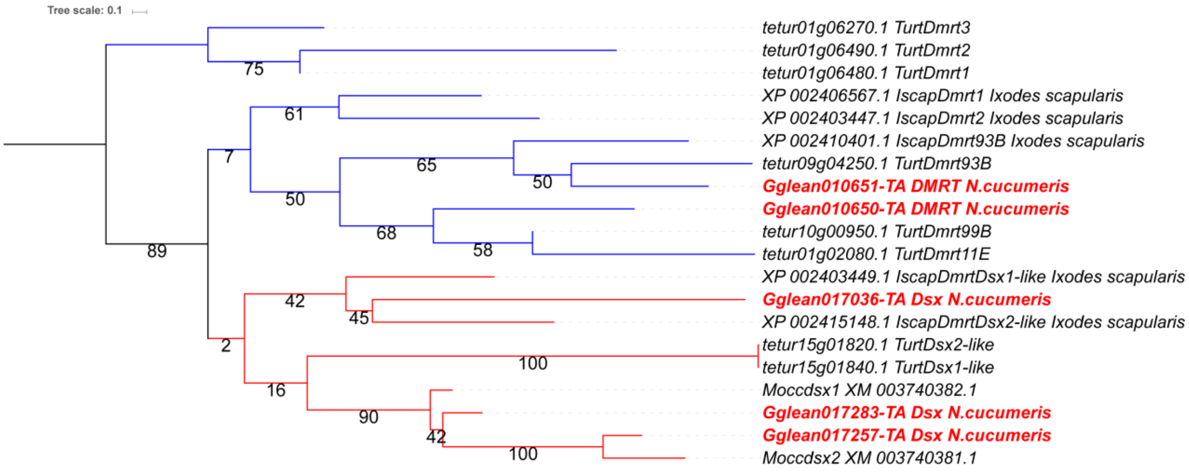

Fig. S5 The *doublesex* and *DMRT* genes identified in *N. cucumeris*

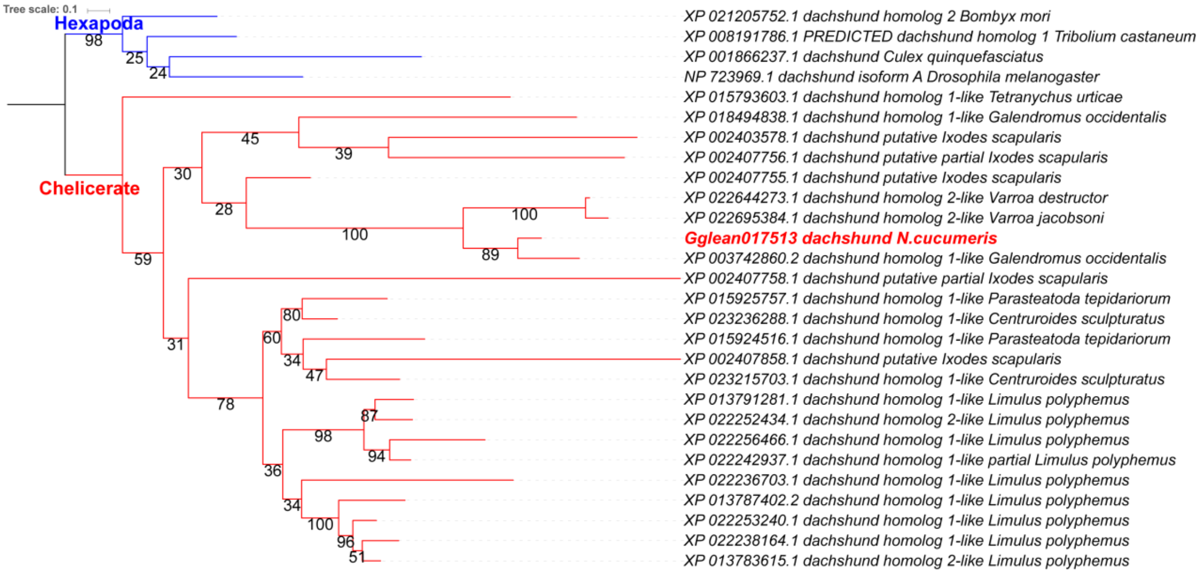

A. *dachshund* (*dac*)

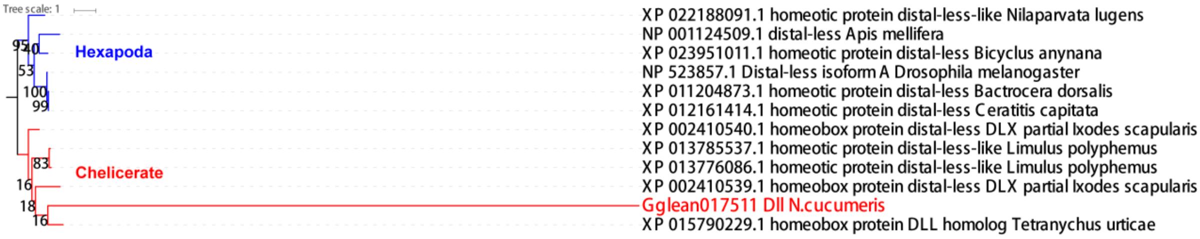

B. *Distal-less* (*Dll*)

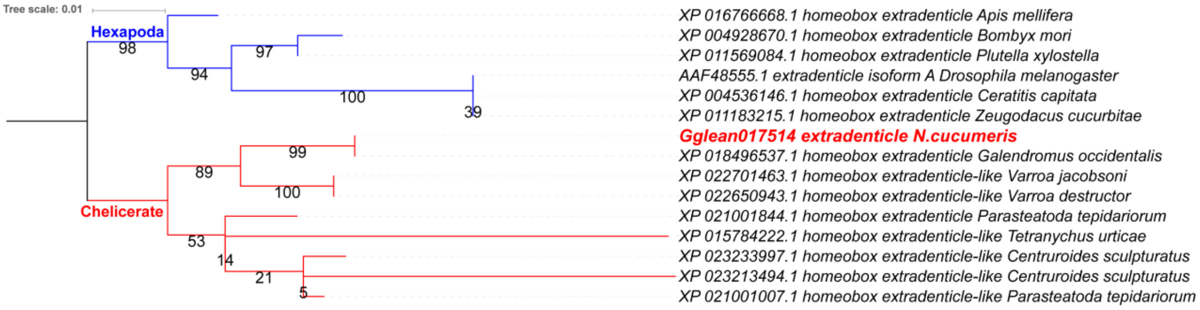

C. *extradenticle* (*exd*)

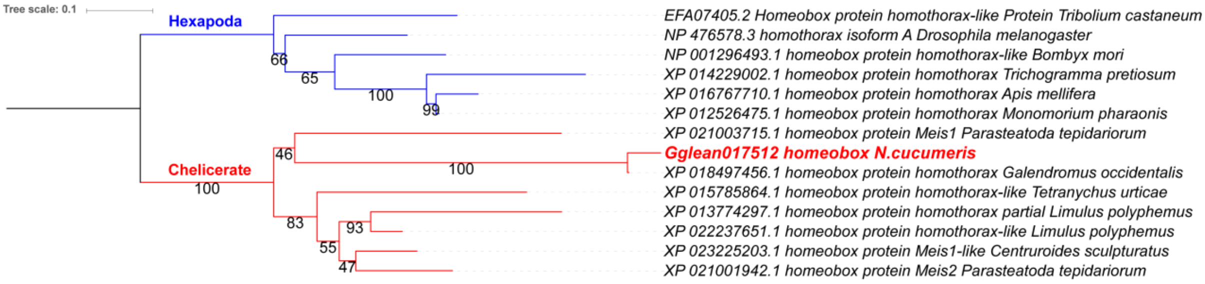

D. *homothorax* (*hth*)

Fig. S6 The four conserved limb gap genes identified in *N. cucumeris*

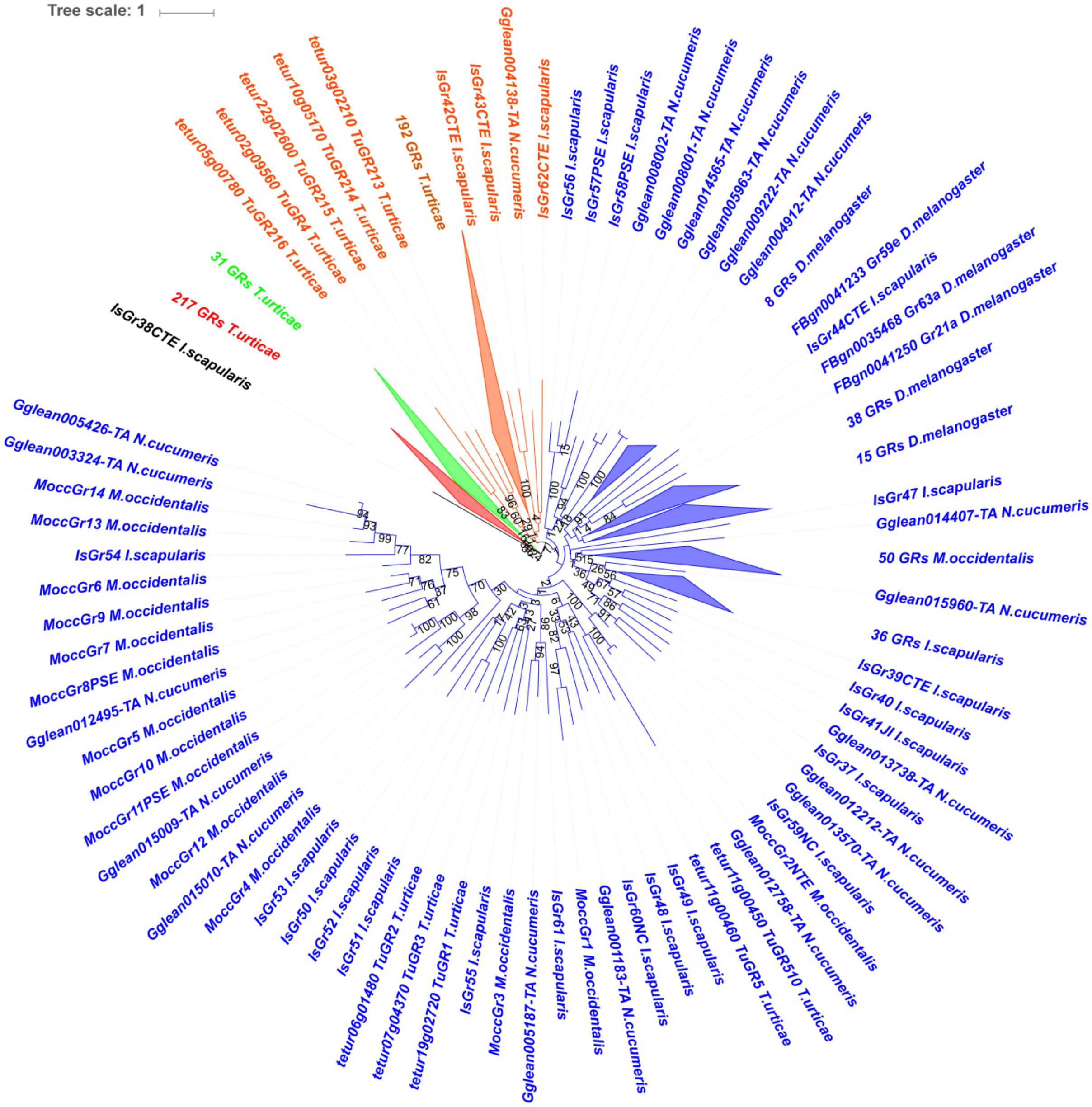

A. gustatory receptors (GR)

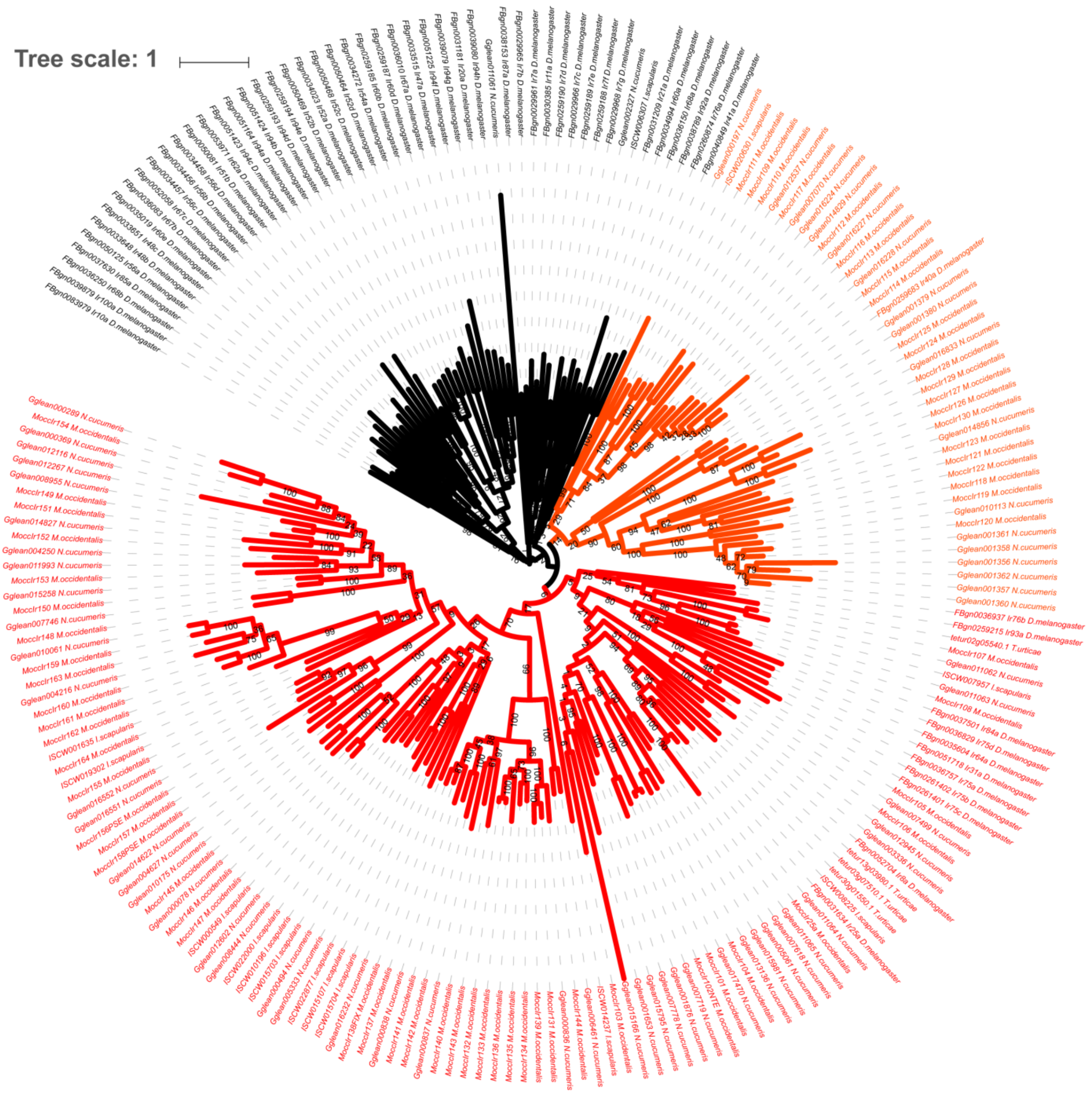

B. ionotropic receptors (IR)

Fig. S7 The chemoreception related receptor genes identified in *N. cucumeris*.

(A): 24 gustatory receptor (GR) genes that can be clustered into three clans; and (B): 65 ionotropic receptor (IR) genes that can be clustered into four clans
