# Supplementary material for: Genomic insights into mite phylogeny, fitness, development, and reproduction
Source: BMC Genomics. 2019 Dec 9;20:954. doi: 10.1186/s12864-019-6281-1 (PMC6902594; doi:10.1186/s12864-019-6281-1)
Supplement: Supplementary file 1 — Additional file 1: Tables S1-S34. [file 12864_2019_6281_MOESM1_ESM.pdf]

Zhang YX, Chen X, Wang JP, Zhang ZQ, Wei H, Yu HY, Zheng HK et al. (2019)

Genomic insights into mite phylogeny, fitness, development, and reproduction

BMC Genomics

DOI: 10.1186/s12864-019-6281-1

Additional file 1.

This file includes 34 supplementary tables:

Table S1. Summary of the sequencing data of the pair-end and mate-pair libraries. **Table S2.** Evaluation of genome assembly by BUSCO and RNA-seq data. **Table S3.** Comparison of the genome assembly among species in the class Arachnida. **Table S4.** Statistics of the predicted protein-coding gene models of *N. cucumeris*. **Table S5.** Functional annotation of the predicted gene models. **Table S6.** The 173 predicted pseudogenes in the *N. cucumeris* genome. **Table S7.** The predicted transposable elements in the *N. cucumeris* genome. **Table S8.** The predicted transposon-related genes in the *N. cucumeris* genome. **Table S9.** The predicted retrotransposon-related genes in the *N. cucumeris* genome. **Table S10.** Clustering analysis of the homologous gene families of nine species within the Subphylum Chelicerata. **Table S11.** Statistics of the shared gene families within the different taxonomic levels. **Table S12.** Clustering analysis of the homologous gene families of five species within the Subclass Acari. **Table S13.** Developmental transcriptomics in *N. cucumeris.* **Table S14.** The predicted genes involved in reproduction in the *N. cucumeris* genome. **Table S15.** The predicted epigenetic regulation related genes in the *N. cucumeris* genome. **Table S16.** The predicted sex-determining related genes in the *N. cucumeris* genome. **Table S17.** The predicted sex-determining related genes in the genomes of Acari and *Limulus polyphemus*. **Table S18.** Identification of the four conserved limb gap genes in the *N. cucumeris* genome. **Table S19.** Identification of the pair-rule and segment-polarity genes in the *N. cucumeris* genome. **Table S20.** Identification of the conserved Hox genes in the *N. cucumeris* genome. **Table S21.** The predicted zinc finger proteins in *N. cucumeris*. **Table S22.** Identification of the genes involved in the Notch signaling pathway in the *N. cucumeris* genome. **Table S23.** Identification of the genes involved in the Hedgehog (Hh) signaling. **Table S24.** Identification of the genes involved in the Hippo signaling pathway in the *N. cucumeris* genome. **Table S25.** The predicted eye specification and phototransduction related genes in the *N. cucumeris* genome. **Table S26.** Identification of the chemosensory receptor related genes in the *N. cucumeris* genome. **Table S27.** Identification of the cytochrome P450 (CYP) genes in the *N. cucumeris* genome. **Table S28.** Identification of the glutathione-S-transferase (GST) genes in the *N. cucumeris* genome. **Table S29.** Identification of the carboxyl/cholinesterase (CCE) genes in the *N. cucumeris* genome. **Table S30.** The predicted transporter-related genes in the *N. cucumeris* genome. **Table S31.** The predicted stress response-related genes in the *N. cucumeris* genome. **Table S32.** The predicted DNA repair-related genes in the *N. cucumeris* genome. **Table S33.** The predicted immunity-related genes in the *N. cucumeris* genome. **Table S34.** The predicted RNAi-related Dicer and Argonaute genes in the *N. cucumeris* genome.

Table S1 Summary of the sequencing data of the pair-end and mate-pair libraries

Library	Data (Gb)	Depth (X)	Q20 (%)	Q30 (%)
180 bp	5.52	30	94.36	89.75
220 bp	7.17	40	92.07	86.69
500 bp	7.8	43	92.05	85.21
2 Kb	5.93	33	88.14	80.16
3 Kb-1	2.57	14	81.57	80.66
3 Kb-2	2.77	15	89.1	79.92
4 Kb-1	4.15	23	88.68	79.52
4 Kb-2	3.25	18	89	81.09
5 Kb	4.31	24	88.76	79.51
8 Kb	3.2	18	89.09	79.99
10 Kb	2.3	13	89.43	80.58
15 Kb	2.8	16	88.81	79.49
Total	51.75	287	89.26	81.88

Table S2 Evaluation of genome assembly by BUSCO and RNA-seq data

Description		Number	Percentage(%)
Complete BUSCOs(C)		1015	95.22%
	Complete and single-copy BUSCOs(S)	959	89.96
	Complete and duplicated BUSCOs(D)	56	5.25
Fragmented BUSCOs(F)		12	1.13
Missing BUSCOs(M)		39	3.66
Total Lineage BUSCOs		1066	100
Assessing the gene region assembly by RNA sequencing data			
Total number of the transcripts with length of >500 nt			16,482
Aligned number of the transcripts with length of >500 nt			16,175
Percentage of the aligned transcripts with length of >500 nt			98.14%
Total number of the transcripts with length of >1000 nt			10,377
Aligned number of the transcripts with length of >1000 nt			10,306
Percentage of the aligned transcripts with length of >1000 nt			99.32%

Table S3 Comparison of the genome assembly among speceis in the class Arachnida

superorder	Parasitiformes					Acariformes						
order	Mesostigmata				Ixodida	Prostigmata	Astigmata		Oribatida			
family	Phytoseiidae		varroidae		Ixodidae	Tetranychidae	Pyroglyphidae	Sarcoptidae	Achipteriidae	Hypochthoniidae	Camisiidae	Steganacaridae
species	*Neoseiulus cucumeris*	*Metaseiulus occidentalis*	*Varroa destructor*	*Ixodes scapularis*	*Rhipicephalus microplus*	*Tetranychus urticae*	*Dermatophagoides farinae*	*Sarcoptes scabiei*	*Achipteria coleoptrata*	*Hypochthonius rufulus*	*Platynothrus peltifer*	*Steganacarus magnus*
Number of scaffolds	1,173	2,211	na	369,495	na	641	515	na	56,345	140,449	105,671	101,545
N50 scaffold length	1,572,811 bp	896,831 KB	na	76,228 bp	na	3.0 MB	186,342 bp	na	na	na	na	na
Number of contigs used in assembly	3,715	3,993	184,190	570,637	175,211	2,036	11,600	18,860	72,776	153,462	126,756	126,765
N50 contig length	222,916	200,706 bp	2,262 bp	2,942 bp	827 bp	212.8 KB	8,538 bp	11,197 bp	3,360 bp	3,126 bp	1,236 bp	1,617 bp
Total length of scaffolds (including gaps)	173051269 bp	151.724 MB	294.134 MB	1.8 Gb	144.692 MB	90.8 MB	53.5453 MB	56.2624 MB	88.4439 MB	172.365 MB	100.533 MB	113.565 MB
Estimated genome size	176 MB	na	na	2.1 Gb	na	90 MB	na	98 MB	na	na	na	na
Versions		GCA_000255335.1, Mocc_1.0	genomic survey	GCA_000208615.1	GCA_000181235.2	GCA_000239435.1	GCA_000767015.1	GCA_000828355.1	GCA_000988765.1	GCA_000988845.1	GCA_000988905.1	GCA_000988885.1

Table S4 Statistics of the predicted protein-coding gene models of *N. cucumeris*

Method	Software	Gene number	Gene length (bp)	Average gene length (bp)	Exon number	Exon length (bp)	Average exon length (bp)	Intron	Intron length (bp)	Average Intron length (bp)
Ab initio	Augustus	14,261	62,474,488	4,381	75,829	20,713,195	273	75,828	41,761,293	551
	GeneID	12,616	85,140,305	6,749	46,595	15,954,124	342	46,594	69,186,181	1,485
	Genscan	19,122	125,375,795	6,557	140,431	35,374,700	252	140,430	89,984,246	641
	GlimmerHMM	27,676	133,634,361	4,829	85,253	22,566,267	265	85,252	111,068,094	1,303
	SNAP	23,836	122,511,273	5,140	90,676	23,011,519	254	90,675	96,280,090	1,062
EST/Unigene	PASA	7,389	28,721,599	3,887	37,282	9,450,986	253	37,281	19,146,301	514
Integration	GLEAN	17,514	50,551,021	2,888	71,831	20,324,416	283	71,830	30,139,882	420

Table S5 Functional annotation of the predicted gene models

Database	Annotated_Number	300 bp<=length<1000 bp	length>=1000 bp	Percentage (%)
COG	4646	1676	2880	27
GO	4403	1795	2431	25
KEGG	4681	1726	2823	27
KOG	9534	3721	5567	54
Pfam	10246	4028	5983	59
Swissprot	9411	3559	5598	54
TrEMBL	12943	5593	6918	74
NT	14508	6629	7268	83
NR	9556	3787	5433	55
All_Annotated	14697	6745	7289	84

Table S6 The 176 predicted pseudogenes in the *N. cucumeris* genome

Scaffold	Start	End	The corresponding gene and the causing model
scaffold_67	10497	13603	Homolog=Gglean003033-PA;Model=premature stop codons;
scaffold_41	199082	199424	Homolog=Gglean009697-PA;Model=framesift;
scaffold_200	15981	16410	Homolog=Gglean002886-PA;Model=framesift and premature stop codons;
scaffold_29	580309	580607	Homolog=Gglean014389-PA;Model=framesift;
scaffold_63	556221	556626	Homolog=Gglean006004-PA;Model=framesift;
scaffold_47	369864	370134	Homolog=Gglean010408-PA;Model=framesift;
scaffold_111	156078	156517	Homolog=Gglean008284-PA;Model=framesift and premature stop codons;
scaffold_9	1706448	1707243	Homolog=Gglean016797-PA;Model=framesift;
scaffold_118	298541	298956	Homolog=Gglean003999-PA;Model=framesift;
scaffold_106	287609	288661	Homolog=Gglean013448-PA;Model=framesift;
scaffold_92	94954	95434	Homolog=Gglean005711-PA;Model=framesift;
scaffold_8	2741096	2743737	Homolog=Gglean015730-PA;Model=framesift;
scaffold_6	465585	465966	Homolog=Gglean006738-PA;Model=framesift;
scaffold_89	352976	353239	Homolog=Gglean000528-PA;Model=premature stop codons;
scaffold_48	1715	2085	Homolog=Gglean008232-PA;Model=framesift;
scaffold_49	1025572	1025786	Homolog=Gglean015814-PA;Model=framesift;
scaffold_17	464037	464981	Homolog=Gglean009972-PA;Model=framesift and premature stop codons;
scaffold_559	346	1026	Homolog=Gglean008258-PA;Model=premature stop codons;
scaffold_0	8561359	8561726	Homolog=Gglean017256-PA;Model=framesift;
scaffold_38	335333	336081	Homolog=Gglean007374-PA;Model=framesift;
scaffold_120	253923	254219	Homolog=Gglean003444-PA;Model=premature stop codons;
scaffold_143	191427	191944	Homolog=Gglean016972-PA;Model=framesift;
scaffold_198	16443	16992	Homolog=Gglean005968-PA;Model=premature stop codons;
scaffold_31	1058160	1058978	Homolog=Gglean009152-PA;Model=premature stop codons;
scaffold_3	5258839	5259115	Homolog=Gglean008190-PA;Model=framesift;
scaffold_29	68443	68869	Homolog=Gglean015730-PA;Model=framesift;
scaffold_194	32003	32548	Homolog=Gglean008021-PA;Model=premature stop codons;
scaffold_214	16571	16960	Homolog=Gglean005199-PA;Model=premature stop codons;
scaffold_36	971280	971562	Homolog=Gglean012534-PA;Model=framesift;
scaffold_76	582755	585038	Homolog=Gglean006731-PA;Model=framesift and premature stop codons;
scaffold_15	1213253	1213465	Homolog=Gglean004612-PA;Model=framesift;
scaffold_13	2261911	2262406	Homolog=Gglean011951-PA;Model=framesift;
scaffold_132	35021	35371	Homolog=Gglean010336-PA;Model=premature stop codons;
scaffold_414	1261	1528	Homolog=Gglean004978-PA;Model=framesift;
scaffold_17	721115	721960	Homolog=Gglean005329-PA;Model=premature stop codons;
scaffold_111	51527	51909	Homolog=Gglean015730-PA;Model=framesift and premature stop codons;
scaffold_74	553574	553763	Homolog=Gglean012258-PA;Model=framesift;
scaffold_90	153885	154187	Homolog=Gglean004148-PA;Model=premature stop codons;
scaffold_511	1211	3450	Homolog=Gglean011990-PA;Model=framesift;
scaffold_150	128179	128373	Homolog=Gglean010403-PA;Model=premature stop codons;
scaffold_48	714	1012	Homolog=Gglean003500-PA;Model=framesift;
scaffold_274	7917	8182	Homolog=Gglean005478-PA;Model=framesift;
scaffold_102	362149	362803	Homolog=Gglean006752-PA;Model=framesift;
scaffold_58	832280	833087	Homolog=Gglean016221-PA;Model=framesift;
scaffold_74	479632	480362	Homolog=Gglean000428-PA;Model=framesift and premature stop codons;
scaffold_81	33399	34893	Homolog=Gglean010386-PA;Model=framesift;

scaffold_151	153563	153792	Homolog=Gglean010327-PA;Model=framesift;
scaffold_213	3020	3736	Homolog=Gglean014488-PA;Model=premature stop codons;
scaffold_33	692891	693620	Homolog=Gglean014299-PA;Model=framesift;
scaffold_20	753540	753947	Homolog=Gglean006691-PA;Model=premature stop codons;
scaffold_85	279508	279968	Homolog=Gglean005468-PA;Model=framesift;
scaffold_787	1	569	Homolog=Gglean005226-PA;Model=framesift;
scaffold_33	438960	439326	Homolog=Gglean013120-PA;Model=framesift;
scaffold_41	1237432	1237888	Homolog=Gglean015389-PA;Model=framesift;
scaffold_90	169955	170334	Homolog=Gglean009336-PA;Model=framesift;
scaffold_111	83723	84071	Homolog=Gglean008996-PA;Model=framesift;
scaffold_1	3429695	3430484	Homolog=Gglean016705-PA;Model=framesift and premature stop codons;
scaffold_102	96585	96905	Homolog=Gglean011726-PA;Model=premature stop codons;
scaffold_76	202486	203060	Homolog=Gglean004999-PA;Model=framesift and premature stop codons;
scaffold_56	412277	414994	Homolog=Gglean003358-PA;Model=framesift;
scaffold_52	310072	310504	Homolog=Gglean008284-PA;Model=framesift and premature stop codons;
scaffold_45	38564	39177	Homolog=Gglean015483-PA;Model=framesift;
scaffold_17	601910	602225	Homolog=Gglean011179-PA;Model=framesift;
scaffold_105	380967	382302	Homolog=Gglean004029-PA;Model=premature stop codons;
scaffold_65	538501	539312	Homolog=Gglean014494-PA;Model=framesift;
scaffold_78	666427	666957	Homolog=Gglean004022-PA;Model=premature stop codons;
scaffold_263	3165	3485	Homolog=Gglean000519-PA;Model=premature stop codons;
scaffold_221	12593	25750	Homolog=Gglean013507-PA;Model=framesift and premature stop codons;
scaffold_105	77252	78582	Homolog=Gglean012277-PA;Model=framesift;
scaffold_1	4352407	4353621	Homolog=Gglean001582-PA;Model=framesift and premature stop codons;
scaffold_38	456123	456841	Homolog=Gglean010326-PA;Model=premature stop codons;
scaffold_132	119736	120107	Homolog=Gglean004058-PA;Model=premature stop codons;
scaffold_89	87668	93955	Homolog=Gglean002979-PA;Model=framesift and premature stop codons;
scaffold_125	202112	202653	Homolog=Gglean011182-PA;Model=framesift;
scaffold_117	301286	303207	Homolog=Gglean003131-PA;Model=framesift;
scaffold_14	568743	569284	Homolog=Gglean014662-PA;Model=framesift;
scaffold_116	61425	62975	Homolog=Gglean009921-PA;Model=premature stop codons;
scaffold_212	28329	28654	Homolog=Gglean006618-PA;Model=framesift and premature stop codons;
scaffold_33	1397204	1398568	Homolog=Gglean000441-PA;Model=premature stop codons;
scaffold_167	75791	76081	Homolog=Gglean005482-PA;Model=premature stop codons;
scaffold_38	1295314	1296518	Homolog=Gglean004065-PA;Model=framesift;
scaffold_100	43663	44220	Homolog=Gglean006340-PA;Model=premature stop codons;
scaffold_46	974882	975815	Homolog=Gglean015789-PA;Model=framesift and premature stop codons;
scaffold_98	752	7684	Homolog=Gglean003050-PA;Model=premature stop codons;
scaffold_325	22447	22894	Homolog=Gglean005917-PA;Model=framesift;
scaffold_45	999061	1001575	Homolog=Gglean013917-PA;Model=framesift and premature stop codons;
scaffold_6	1305926	1306245	Homolog=Gglean017044-PA;Model=framesift;
scaffold_53	218824	219253	Homolog=Gglean004962-PA;Model=framesift;
scaffold_5	1457578	1459422	Homolog=Gglean014579-PA;Model=framesift and premature stop codons;
scaffold_41	859192	860082	Homolog=Gglean007680-PA;Model=premature stop codons;
scaffold_1	2428518	2438016	Homolog=Gglean011291-PA;Model=framesift and premature stop codons;
scaffold_214	8596	9006	Homolog=Gglean005974-PA;Model=framesift;
scaffold_2	1555249	1555516	Homolog=Gglean011457-PA;Model=framesift;
scaffold_29	1000477	1001054	Homolog=Gglean006968-PA;Model=premature stop codons;

scaffold_1	4576039	4577043	Homolog=Gglean005387-PA;Model=framesift;
scaffold_304	3144	3490	Homolog=Gglean009115-PA;Model=framesift and premature stop codons;
scaffold_108	24324	25285	Homolog=Gglean003792-PA;Model=premature stop codons;
scaffold_320	8382	9098	Homolog=Gglean014488-PA;Model=premature stop codons;
scaffold_373	777	1127	Homolog=Gglean010336-PA;Model=premature stop codons;
scaffold_65	591661	592144	Homolog=Gglean009337-PA;Model=framesift;
scaffold_13	901266	902596	Homolog=Gglean000961-PA;Model=framesift;
scaffold_35	805619	806663	Homolog=Gglean009898-PA;Model=framesift;
scaffold_78	7497	7777	Homolog=Gglean012534-PA;Model=framesift;
scaffold_17	54546	55055	Homolog=Gglean005700-PA;Model=premature stop codons;
scaffold_132	7248	7583	Homolog=Gglean004051-PA;Model=premature stop codons;
scaffold_38	1070379	1071499	Homolog=Gglean004016-PA;Model=premature stop codons;
scaffold_29	72883	73310	Homolog=Gglean005932-PA;Model=framesift;
scaffold_42	959853	960083	Homolog=Gglean016765-PA;Model=premature stop codons;
scaffold_168	43090	43299	Homolog=Gglean003710-PA;Model=premature stop codons;
scaffold_49	779208	780387	Homolog=Gglean011736-PA;Model=premature stop codons;
scaffold_187	22601	28379	Homolog=Gglean001584-PA;Model=framesift;
scaffold_927	898	1300	Homolog=Gglean001332-PA;Model=framesift;
scaffold_24	1690805	1695095	Homolog=Gglean005471-PA;Model=framesift;
scaffold_2	4284646	4284854	Homolog=Gglean015221-PA;Model=framesift;
scaffold_85	607520	607749	Homolog=Gglean016587-PA;Model=framesift;
scaffold_99	280256	282154	Homolog=Gglean013824-PA;Model=framesift and premature stop codons;
scaffold_186	51393	52151	Homolog=Gglean004468-PA;Model=premature stop codons;
scaffold_11	21615	22193	Homolog=Gglean002688-PA;Model=premature stop codons;
scaffold_1	5193741	5194057	Homolog=Gglean011705-PA;Model=framesift;
scaffold_132	48697	49638	Homolog=Gglean010355-PA;Model=framesift and premature stop codons;
scaffold_89	469118	469396	Homolog=Gglean016743-PA;Model=premature stop codons;
scaffold_2	358304	358532	Homolog=Gglean017471-PA;Model=framesift;
scaffold_55	147984	148989	Homolog=Gglean004973-PA;Model=framesift;
scaffold_107	226551	227112	Homolog=Gglean016633-PA;Model=framesift and premature stop codons;
scaffold_65	451615	451951	Homolog=Gglean011496-PA;Model=framesift;
scaffold_114	150023	150402	Homolog=Gglean004560-PA;Model=framesift;
scaffold_46	963293	963643	Homolog=Gglean004605-PA;Model=premature stop codons;
scaffold_56	104799	105118	Homolog=Gglean002901-PA;Model=framesift;
scaffold_118	177594	178151	Homolog=Gglean006746-PA;Model=premature stop codons;
scaffold_197	22475	22972	Homolog=Gglean016972-PA;Model=premature stop codons;
scaffold_405	4652	5002	Homolog=Gglean005385-PA;Model=premature stop codons;
scaffold_25	1074789	1075308	Homolog=Gglean005711-PA;Model=framesift;
scaffold_864	519	935	Homolog=Gglean013781-PA;Model=premature stop codons;
scaffold_1	5105897	5106565	Homolog=Gglean003119-PA;Model=premature stop codons;
scaffold_317	5933	6874	Homolog=Gglean002346-PA;Model=premature stop codons;
scaffold_21	1317532	1318367	Homolog=Gglean009811-PA;Model=framesift and premature stop codons;
scaffold_87	143500	144501	Homolog=Gglean002906-PA;Model=framesift and premature stop codons;
scaffold_117	103170	103886	Homolog=Gglean003020-PA;Model=framesift;
scaffold_1	656262	657135	Homolog=Gglean001379-PA;Model=framesift;
scaffold_46	612190	612899	Homolog=Gglean011887-PA;Model=premature stop codons;
scaffold_24	1334019	1344696	Homolog=Gglean002178-PA;Model=framesift and premature stop codons;
scaffold_29	33131	33320	Homolog=Gglean012258-PA;Model=framesift;

scaffold_137	208865	209612	Homolog=Gglean004968-PA;Model=framesift;
scaffold_3	893682	895202	Homolog=Gglean008694-PA;Model=framesift;
scaffold_18	1094041	1097367	Homolog=Gglean006974-PA;Model=framesift and premature stop codons;
scaffold_24	1565190	1565854	Homolog=Gglean010764-PA;Model=framesift and premature stop codons;
scaffold_68	13400	13871	Homolog=Gglean011253-PA;Model=framesift;
scaffold_13	606683	606998	Homolog=Gglean016264-PA;Model=framesift;
scaffold_436	4411	4795	Homolog=Gglean007523-PA;Model=framesift;
scaffold_19	1635536	1636553	Homolog=Gglean016536-PA;Model=framesift and premature stop codons;
scaffold_147	165448	165738	Homolog=Gglean006978-PA;Model=premature stop codons;
scaffold_47	78403	80040	Homolog=Gglean011946-PA;Model=premature stop codons;
scaffold_51	655551	655991	Homolog=Gglean007621-PA;Model=premature stop codons;
scaffold_39	1189659	1194806	Homolog=Gglean010542-PA;Model=framesift and premature stop codons;
scaffold_90	8638	8911	Homolog=Gglean001132-PA;Model=framesift;
scaffold_71	215983	216277	Homolog=Gglean012534-PA;Model=framesift;
scaffold_56	107865	109199	Homolog=Gglean014485-PA;Model=framesift;
scaffold_97	296598	296927	Homolog=Gglean007904-PA;Model=premature stop codons;
scaffold_15	447241	447508	Homolog=Gglean017062-PA;Model=framesift;
scaffold_1	1115701	1116439	Homolog=Gglean003595-PA;Model=framesift;
scaffold_105	84107	86388	Homolog=Gglean010149-PA;Model=framesift and premature stop codons;
scaffold_189	16927	17108	Homolog=Gglean004485-PA;Model=framesift;
scaffold_53	710400	710733	Homolog=Gglean013037-PA;Model=framesift;
scaffold_139	151484	151713	Homolog=Gglean013904-PA;Model=framesift;
scaffold_54	18871	19284	Homolog=Gglean015499-PA;Model=premature stop codons;
scaffold_40	896030	896438	Homolog=Gglean011143-PA;Model=premature stop codons;
scaffold_111	135311	136913	Homolog=Gglean015848-PA;Model=framesift;
scaffold_123	134629	135043	Homolog=Gglean004015-PA;Model=framesift;
scaffold_29	185619	186609	Homolog=Gglean011947-PA;Model=framesift and premature stop codons;
scaffold_43	371380	371685	Homolog=Gglean011492-PA;Model=premature stop codons;
scaffold_42	806149	806904	Homolog=Gglean011430-PA;Model=premature stop codons;
scaffold_255	13838	18514	Homolog=Gglean016510-PA;Model=framesift and premature stop codons;
scaffold_42	459935	460966	Homolog=Gglean006143-PA;Model=framesift;
scaffold_83	55891	56353	Homolog=Gglean005199-PA;Model=framesift;
scaffold_115	312741	315842	Homolog=Gglean003033-PA;Model=framesift and premature stop codons;
scaffold_97	467619	468034	Homolog=Gglean003999-PA;Model=framesift;

Table S7 The predicted transposable elements in the *N. cucumeris* genome

Type	Number	Length (bp)	Percentage (%)
**ClassI**	**26,805**	**9,788,528**	**5.65643**
ClassI/DIRS	483	197,503	0.11
ClassI/LINE	7,909	2,581,729	1.49
ClassI/LTR	2,296	954,902	0.55
ClassI/LTR/Copia	3,490	1,957,757	1.13
ClassI/LTR/Gypsy	5,410	2,061,005	1.19
ClassI/PLE|LARD	6,700	2,036,156	1.18
ClassI/SINE	178	23,616	0.01
ClassI/TRIM	106	14,406	0.01
ClassI/Unknown	233	29,597	0.02
**ClassII**	**27,969**	**7,152,050**	**4.13291**
ClassII/Crypton	28	3,806	0
ClassII/Helitron	5,797	1,718,510	0.99
ClassII/MITE	7,617	1,764,994	1.02
ClassII/Maverick	685	115,646	0.07
ClassII/TIR	11,585	3,396,258	1.96
ClassII/Unknown	2,257	191,097	0.11
PotentialHostGene	604	234,557	0.14
SSR	5,009	358,038	0.21
**Unknown**	**21,636**	**4,871,972**	**2.82**
**Total**	**82,023**	**20,645,827**	**11.93**

Table S8 The predicted transposon-related genes in the *N. cucumeris* genome

Gene family	**Number**	Genes
**MULE transposase domain**		
GF_6864	**4**	Gglean004897-PA, Gglean009215-PA, Gglean016622-PA, Gglean004902-PA
GF_12390	**2**	Gglean003153-PA, Gglean007602-PA
GF_12451	**2**	Gglean012813-PA, Gglean016627-PA
GF_14214	**2**	Gglean001582-PA, Gglean001585-PA
**Transposase C of IS166 homeodomain**		
GF_11142	**3**	Gglean003498-PA, Gglean012410-PA, Gglean003277-PA
**Transposase**		
GF_11173	**3**	Gglean010344-PA, Gglean010356-PA, Gglean009546-PA
**Putative transposase DNA-binding domain**		
GF_12415	**2**	Gglean004636-PA, Gglean014620-PA
**Transposase, Mutator family**		
GF_12483	**2**	Gglean003719-PA, Gglean004016-PA
**Phage integrase family**		
GF_4406	**9**	Gglean012384-PA, Gglean015223-PA, Gglean016562-PA, Gglean015208-PA, Gglean013434-PA, Gglean007507-PA, Gglean011701-PA, Gglean015224-PA, Gglean015207-PA
GF_10157	**2**	Gglean015860-PA, Gglean015863-PA

Table S9 The predicted retrotransposon-related genes in the *N. cucumeris* genome

Gene family	**Number**	Genes
**reverse transcriptase**		
GF_266	**24**	Gglean002522-PA, Gglean016667-PA, Gglean006960-PA, Gglean004901-PA, Gglean003715-PA, Gglean005139-PA, Gglean012908-PA, Gglean016131-PA, Gglean014733-PA, Gglean006700-PA, Gglean016565-PA, Gglean002981-PA, Gglean017453-PA, Gglean003588-PA, Gglean014561-PA, Gglean003529-PA, Gglean012183-PA, Gglean013181-PA, Gglean006648-PA, Gglean000859-PA, Gglean012195-PA, Gglean002177-PA, Gglean003027-PA, Gglean005235-PA
GF_1332	**13**	Gglean003118-PA, Gglean017007-PA, Gglean014073-PA, Gglean017500-PA, Gglean003600-PA, Gglean013244-PA, Gglean017256-PA, Gglean013990-PA, Gglean003872-PA, Gglean004571-PA, Gglean000428-PA, Gglean016563-PA, Gglean013533-PA
GF_3705	**9**	Gglean017321-PA, Gglean017491-PA, Gglean006536-PA, Gglean016265-PA, Gglean014762-PA, Gglean007591-PA, Gglean010529-PA, Gglean007592-PA, Gglean010255-PA
GF_2885	**6**	Gglean005919-PA, Gglean012199-PA, Gglean009229-PA, Gglean005479-PA, Gglean005398-PA, Gglean005480-PA
GF_12375	**2**	Gglean002904-PA, Gglean004029-PA
GF_12380	**2**	Gglean002876-PA, Gglean005472-PA
GF_14048	**2**	Gglean002874-PA, Gglean014236-PA
Endonuclease-reverse transcriptase		
GF_3700	**9**	Gglean003133-PA, Gglean003166-PA, Gglean005705-PA, Gglean004916-PA, Gglean009208-PA, Gglean006162-PA, Gglean011947-PA, Gglean012909-PA, Gglean013114-PA
GF_3711	**7**	Gglean004536-PA, Gglean005254-PA, Gglean005255-PA, Gglean003021-PA, Gglean016699-PA, Gglean006637-PA, Gglean002387-PA
GF_2887	**5**	Gglean004484-PA, Gglean004945-PA, Gglean017096-PA, Gglean005476-PA, Gglean013745-PA
GF_12474	**2**	Gglean009862-PA, Gglean010363-PA
GF_12566	**2**	Gglean002194-PA, Gglean005238-PA
Retrotransposon gag protein		
GF_7798	**3**	Gglean004500-PA, Gglean009601-PA, Gglean016587-PA

Table S10 Clustering analysis of the homologous gene families of nine species within the Subphylum Chelicerata

Name	Totalgene	oneCopynum	MultiCopynum	Unigenenum	Othergenenum	Clusternum	UnClusternum(orphan)	Totalfamily	Unifamily
Rhipicephalus microplus	15429	635	2261	2025	7271	12192	3237	5669	683
Mesobuthus martensii	32016	949	1211	7845	12344	22349	9667	8283	1174
Metaseiulus occidentalis	11738	1100	779	283	8398	10560	1178	8538	103
Tetranychus urticae	18224	1043	1040	3837	6271	12191	6033	6602	709
Stegodyphus mimosarum	27135	936	1159	4528	10000	16623	10512	9295	591
Neoseiulus cucumeris	17501	1099	756	2102	8665	12622	4879	9352	619
Sarcoptes scabiei	10473	1172	518	360	4361	6411	4062	5577	109
Ixodes scapularis	20486	1118	765	1830	8557	12270	8216	9067	609
Limulus polyphemus	23300	571	3168	1606	14437	19782	3518	9272	588

Totalgene, total number of the genes used in this analysis; oneCopynum, number of the single-copy genes; MultiCopynum, number of the multi-copy genes; Unigenenum, number of the species-specific genes; Othergenenum, number of other genes; Clusternum, number of the genes being clustered; UnClusternum, number of the genes not being clustered; Totalfamily, total number of the gene families being clustered; Unifamily, number of the species-specific gene families.

Table S11 Statistics of the shared gene families within the different taxonomic levels

	Number of gene families	Percentage
Within the order Mesostigmata	8060/9352	~86.18%
Within the Superorder Parasitiformes	2756/9352	~29.47%
Within the Subclass Acari	1904/9352	~20.36%
Within the Class Arachnida	1392/9352	~14.88%
Within the Subphylum Chelicerata	1380/9352	~14.76%

Order Mesostigmata: *N. cucumeris* and *M. occidentalis*; Superorder Parasitiformes: *N. cucumeris*, *M. occidentalis*, *I. scapularis* and *R. microplus*; Subclass Acari: *N. cucumeris*, *M. occidentalis*, *I. scapularis*, *R. microplus*, *T. urticae* and *S. scabiei*; Class Arachnida: *N. cucumeris*, *M. occidentalis*, *I. scapularis*, *R. microplus*, *T. urticae*, *S. mimosarum*, *S. mimosarum* and *M. martensii*; Subphylum Chelicerata: *N. cucumeris*, *M. occidentalis*, *I. scapularis*, *R. microplus*, *T. urticae*, *S. mimosarum*, *S. mimosarum*, *M. martensii* and *L. polyphemus*

Table S12 Clustering analysis of the homologous gene families of five species within the Subclass Acari

Species	Totalgene	oneCopynum	MultiCopynum	Unigenenum	Othergenenum	Clusternum	UnClusternum	Totalfamily	Unifamily
Rhipicephalus microplus	15429	1079	3120	2296	5365	11860	3569	5407	771
Metaseiulus occidentalis	11738	1793	948	331	7423	10495	1243	8539	109
Neoseiulus cucumeris	17501	1798	927	2152	7492	12369	5132	9214	628
Ixodes scapularis	20486	1851	769	2168	6238	11026	9460	7938	702
Tetranychus urticae	18224	1708	1410	4333	4277	11728	6496	6070	824

Table S13 Developmental transcriptpmics in *N. cucumeris* (see theend of this file, which is too long to be added here).

Table S14 The predicted genes involved in reproduction in the *N. cucumeris* genome.

Gene ID	Functional annotation
**Vitellogenins and vitellogenesis**	
Gglean009529-TA	vitellogenin 2
Gglean009616-TA	vitellogenin 2
Gglean014915-TA	Vitellogenin 1
Gglean015031-TA	Vitellogenin 1
Gglean011739-TA	vitellogenin receptor isoform X2
Gglean014400-TA	vitellogenin receptor
Gglean007353-TA	follistatin-related protein 5-like
Gglean007354-TA	follistatin-related protein 5-like
Gglean007355-TA	follistatin-related protein 5-like
**ecdysone synthesis and regulation**	
Gglean013205-TA	phantom
Gglean012867-TA	phantom
Gglean006838-TA	spookier
Gglean002175-TA	shadow
Gglean001295-TA	disembodied
Gglean001008-TA	shade
Gglean003305-TA	ecdysone receptor-like, EcR
Gglean003306-TA	ecdysone receptor-like, EcR
Gglean004659-TA	ecdysone receptor-like, EcR
Gglean013376-TA	Ultraspiracle, Usp
Gglean008810-TA	fibroblast growth factor receptor 3-like
Gglean005674-TA	ecdysone 20-monooxygenase-like
Gglean008707-TA	ecdysone-induced protein 78C-like, E78
Gglean002523-TA	nuclear hormone receptor FTZ-F1-like, FTZ-F1
Gglean003659-TA	follicle-stimulating hormone receptor-like
Gglean001019-TA	follicle-stimulating hormone receptor-like
Gglean004832-TA	probable nuclear hormone receptor HR3-like
Gglean004833-TA	probable nuclear hormone receptor HR3-like
Gglean000303-TA	thyrotropin-releasing hormone receptor-like
Gglean000304-TA	thyrotropin-releasing hormone receptor-like
Gglean011294-TA	thyrotropin-releasing hormone receptor-like
Gglean000361-TA	probable nuclear hormone receptor HR38-like
Gglean006376-TA	probable nuclear hormone receptor HR3-like
Gglean009896-TA	adrenocorticotropic hormone receptor-like
Gglean009904-TA	adrenocorticotropic hormone receptor-like
Gglean013783-TA	gonadotropin-releasing hormone receptor-like
Gglean014841-TA	nuclear hormone receptor E75-like
Gglean001765-TA	diuretic hormone receptor-like
Gglean011765-TA	hepatocyte growth factor receptor-like
Gglean001682-TA	protein tailless-like
Gglean011119-TA	estrogen-related receptor gamma-like
Gglean011120-TA	estrogen-related receptor gamma-like
Gglean006601-TA	fibroblast growth factor receptor 3-like
Gglean008811-TA	fibroblast growth factor receptor 1-like
Gglean008812-TA	fibroblast growth factor receptor 1-like
Gglean008813-TA	fibroblast growth factor receptor 2-like
**Oviposition**	
Gglean013954-TA	aromatic-L-amino-acid decarboxylase-like (tyrosine decarboxylase, TDC)
Gglean009850-PA	dopamine beta-hydroxylase-like (tyramine beta-hydroxylase, TβH)
Gglean000132-TA	octopamine receptor 1-like

Gglean009127-TA	octopamine receptor beta-3R-like, OctβR
Gglean000731-TA	tyramine/octopamine receptor-like
Gglean007939-TA	synaptic vesicular amine transporter-like, (vesicular monoamine transporter, VMAT)
Gglean007465-TA	calcium/calmodulin-dependent protein kinase type II alpha chain-like, CaMKII
Gglean013006-TA	nitric oxide synthase protein-like, NOS
Gglean003252-TA	clathrin heavy chain 1
Gglean007355-TA	follistatin-related protein 5-like, FRP
/	Newgene, Netrin-A
Gglean004139-TA	uncharacterized protein LOC100906466, Netrin-B
Gglean012970-TA	netrin receptor UNC5B-like
Gglean017231-TA	netrin receptor UNC5C-like
Gglean007940-TA	synaptic vesicular amine transporter-like
Gglean009086-TA	synaptic vesicular amine transporter-like
Gglean009087-TA	synaptic vesicular amine transporter-like
Gglean000093-TA	glycine/tyrosine-rich eggshell protein
Gglean000570-TA	glycine/tyrosine-rich eggshell protein
Gglean005811-TA	secretory eggshell protein precursor
Gglean009016-TA	secretory eggshell protein precursor
Gglean015104-TA	secretory eggshell protein precursor
Gglean000405-TA	major egg antigen-like
Gglean010031-TA	major egg antigen-like
Gglean002781-TA	FMRFamide-related neuropeptides-like
Gglean003230-TA	neuropeptide F receptor-like
Gglean007140-TA	neuropeptide F receptor-like
Gglean010458-TA	neuropeptide F receptor-like
Gglean005713-TA	neuropeptide FF receptor-like
Gglean006645-TA	neuropeptide FF receptor-like
Gglean001707-TA	neuropeptide FF receptor 2-like
Gglean006468-TA	neuropeptides capa receptor-like
Gglean006469-TA	neuropeptides capa receptor-like
Gglean000887-TA	neuropeptides capa receptor-like
Gglean012105-TA	neuropeptides capa receptor-like
Gglean007566-TA	neuropeptide Y receptor-like
Gglean014817-TA	neuropeptide Y receptor type 2-like
Gglean014818-TA	neuropeptide Y receptor type 5-like

Table S15 The predicted epigenetic regulation related genes in the *N. cucumeris* genome.

Gene ID	Functional annotation
**Histone methylation/demethylation**	
Gglean003268-TA	histone-lysine N-methyltransferase 2C-like
Gglean003269-TA	hypothetical protein IscW_ISCW023204, HMT|nonSET
Gglean003270-TA	histone-lysine N-methyltransferase 2D-like, HMT|SET1
Gglean003357-TA	histidine protein methyltransferase 1 homolog
Gglean003543-TA	N-lysine methyltransferase SMYD2-A-like, HMT|SMYD
Gglean003548-TA	N-lysine methyltransferase SMYD2-A-like, HMT|SMYD
Gglean003549-TA	N-lysine methyltransferase SMYD2-A-like, HMT|SMYD
Gglean003572-TA	N-lysine methyltransferase SMYD2-A-like, HMT|SMYD
Gglean007308-TA	N-lysine methyltransferase SMYD2-B-like, HMT|SMYD
Gglean011908-TA	N-lysine methyltransferase SMYD2-A-like, HMT|SMYD
Gglean003668-TA	histone-lysine N-methyltransferase ASHR1-like
Gglean011568-TA	histone-lysine N-methyltransferase ASHR1-like
Gglean005825-TA	histone-lysine N-methyltransferase setd3-like, HMT|SET3
Gglean005827-TA	histone-lysine N-methyltransferase setd3-like, HMT|SET3
Gglean006888-TA	histone-lysine N-methyltransferase SUV39H2-like, HMT|SUV39
Gglean006889-TA	histone-lysine N-methyltransferase SUV39H2-like, HMT|SUV39
Gglean009212-TA	histone-lysine N-methyltransferase SUV39H2-like, HMT|SUV39
Gglean009573-TA	histone-lysine N-methyltransferase Su(var)3-9-like, HMT|SUV3-9
Gglean009575-TA	histone-lysine N-methyltransferase Su(var)3-9-like, HMT|SUV3-9
Gglean011236-TA	histone-lysine N-methyltransferase SUV420H1-like, HMT|SUV4-20
Gglean007411-TA	histone-lysine N-methyltransferase EHMT1-like, HMT|SUV39
Gglean008942-TA	histone-lysine N-methyltransferase EZH2-like, HMT|EZ
Gglean009250-TA	probable histone-lysine N-methyltransferase Mes-4-like
Gglean009459-TA	Histone-lysine N-methyltransferase PRDM9
Gglean012286-TA	histone-lysine N-methyltransferase, H3 lysine-79 specific-like
Gglean015242-TA	histone-lysine N-methyltransferase ASH1L-like, HMT|SET2
Gglean016034-TA	histone-lysine N-methyltransferase SETD1B-A-like, HMT|SET1
Gglean016326-TA	N-lysine methyltransferase SETD8-like, HMT|HMT_other
Gglean016180-TA	histone-lysine N-methyltransferase eggless-like, egg, HMT|SUV39
Gglean016181-TA	histone-lysine N-methyltransferase eggless-like, HMT|SUV39
Gglean008421-TA	histone-arginine methyltransferase CARMER-like
Gglean013613-TA	histone-arginine methyltransferase CARMER-like
Gglean016088-TA	histone-arginine methyltransferase CARMER-like
Gglean008711-TA	probable histone-arginine methyltransferase CARM1-like
Gglean005748-TA	protein arginine N-methyltransferase 5-like
Gglean007919-TA	protein arginine N-methyltransferase 3-like
Gglean013678-TA	protein arginine N-methyltransferase 7-like
Gglean014593-TA	protein arginine N-methyltransferase 1-like
Gglean008343-TA	set1/Ash2 histone methyltransferase complex subunit ASH2-like
Gglean000048-TA	lysine-specific demethylase 3B-like
Gglean006207-TA	lysine-specific histone demethylase 1B-like
Gglean009719-TA	lysine-specific demethylase 5A-like
Gglean000981-TA	lysine-specific demethylase NO66-like
Gglean012061-TA	lysine-specific demethylase 2B-like
Gglean012214-TA	lysine-specific histone demethylase 1A
Gglean012215-TA	lysine-specific histone demethylase 1A
Gglean013994-TA	lysine-specific demethylase 8-like
Gglean014135-TA	lysine-specific demethylase 7B-like
Gglean014137-TA	lysine-specific demethylase PHF2-like
Gglean015525-TA	lysine-specific histone demethylase 1A-like

Gglean016097-TA	lysine-specific demethylase 4C-like
Gglean016242-TA	lysine-specific demethylase 4C-like
Gglean002266-TA	bifunctional arginine demethylase and lysyl-hydroxylase JMJD6-like
Gglean011026-TA	histone demethylase UTY-like
**histone acetylation/deacetylation**	
Gglean002505-TA	N-terminal acetyltransferase complex ard1 subunit
Gglean003218-TA	N-acetyltransferase 10
Gglean005421-TA	histone acetyltransferase p300-like, HAT|p300_CBP
Gglean016174-TA	Histone acetyltransferase p300, HAT|p300_CBP
Gglean005941-TA	histone acetyltransferase KAT2B, HAT|GCN5
Gglean016335-TA	histone acetyltransferase KAT2B, HAT|GCN5
Gglean000965-TA	histone acetyltransferase KAT8-like, HAT|MYST
Gglean013506-TA	histone acetyltransferase KAT5-like, HAT|MYST
Gglean016832-TA	histone acetyltransferase KAT7-like, HAT|MYST
Gglean015344-TA	histone acetyltransferase type B catalytic subunit-like, HAT|HAT1
Gglean002656-TA	elongator complex protein 3-like, HAT|ELP3
Gglean008331-TA	cysteine-rich protein 2-binding protein-like, HAT|GNAT_other
Gglean007276-TA	bromodomain testis-specific protein-like, HAT|Bromodomain
Gglean001408-TA	bromodomain and WD repeat-containing protein 3-like, HAT|Bromodomain
Gglean003519-TA	histone deacetylase 6-like, HDAC6
Gglean003520-TA	histone deacetylase 6-like, HDAC10
Gglean003521-TA	histone deacetylase 6-like, HDAC10
Gglean004265-TA	histone deacetylase 1-like, HDAC1
Gglean004272-TA	histone deacetylase 3-like, HDAC3
Gglean001306-TA	histone deacetylase 4-like, HDAC4
Gglean008244-TA	NAD-dependent protein deacetylase sirtuin-1-like, Sirt1
Gglean000875-TA	NAD-dependent protein deacetylase sirtuin-6-like, Sirt6
Gglean017298-TA	NAD-dependent protein deacetylase sirtuin-2-like, Sirt2
Gglean012987-TA	histone deacetylase complex subunit SAP18-like
Gglean017353-TA	sin3 histone deacetylase corepressor complex component SDS3-like
Gglean017354-TA	sin3 histone deacetylase corepressor complex component SDS3-like
**Other protein acetylation/deacetylation**	
Gglean004418-TA	N-alpha-acetyltransferase 15, NatA auxiliary subunit-like
Gglean010840-TA	N-alpha-acetyltransferase 25, NatB auxiliary subunit-like
Gglean013703-TA	N-alpha-acetyltransferase 35, NatC auxiliary subunit-like
Gglean006912-TA	N-alpha-acetyltransferase 20-like
Gglean006913-TA	N-alpha-acetyltransferase 20-like
Gglean007782-TA	N-alpha-acetyltransferase 10-like
Gglean010807-TA	N-alpha-acetyltransferase 60-like
Gglean016013-TA	N-alpha-acetyltransferase 60-like
Gglean017326-TA	N-alpha-acetyltransferase 40-like
Gglean013564-TA	N-acetyltransferase 9-like protein-like
Gglean009098-TA	N-acetyltransferase YitI-like
Gglean009099-TA	N-acetyltransferase YitI-like
Gglean009100-TA	N-acetyltransferase YitI-like
Gglean016664-TA	N-acetyltransferase YitI-like
Gglean014542-TA	probable N-acetyltransferase san-like
Gglean016662-TA	N-acetyltransferase 6-like
**DNA methylation**	
Gglean010642-TA	N(6)-adenine-specific DNA methyltransferase 2-like
Gglean016482-TA	N6-adenosine-methyltransferase 70 kDa subunit
Gglean007810-TA	DNMT2, Methyltransferase 2, tRNA (cytosine-5-)-methyltransferase-like,
Gglean010555-TA	DNA methyltransferase 1-associated protein 1-like

**RNA methylation**	
Gglean002202-TA	tRNA (uracil-5-)-methyltransferase homolog A-like
Gglean017031-TA	tRNA (uracil-5-)-methyltransferase homolog A-like
Gglean002434-TA	tRNA (adenine(58)-N(1))-methyltransferase catalytic subunit TRMT61A-like
Gglean002435-TA	tRNA (adenine(58)-N(1))-methyltransferase catalytic subunit TRMT61A-like
Gglean002640-TA	tRNA (guanine(26)-N(2))-dimethyltransferase-like
Gglean002883-TA	tRNA (guanine-N(7)-)-methyltransferase subunit WDR4-like
Gglean009356-TA	tRNA (cytosine(34)-C(5))-methyltransferase-like
Gglean010829-TA	tRNA guanosine-2'-O-methyltransferase
Gglean011590-TA	tRNA (guanine-N(7)-)-methyltransferase-like
Gglean001387-TA	tRNA (guanine-N(7)-)-methyltransferase subunit WDR4-like
Gglean017053-TA	tRNA (guanine-N(7)-)-methyltransferase subunit WDR4-like
Gglean011787-TA	tRNA (guanine(10)-N2)-methyltransferase homolog
Gglean013656-TA	probable tRNA (uracil-O(2)-)-methyltransferase-like
Gglean014396-TA	tRNA (guanine(37)-N1)-methyltransferase-like
Gglean005186-TA	putative rRNA methyltransferase 3-like
Gglean008370-TA	RNA (guanine-9-)-methyltransferase domain-containing protein 2-like
Gglean008710-TA	rRNA/tRNA 2'-O-methyltransferase fibrillarin-like
Gglean009105-TA	ribosomal RNA small subunit methyltransferase NEP1-like
Gglean011432-TA	putative ribosomal RNA methyltransferase 1-like
Gglean012265-TA	rRNA methyltransferase 1, mitochondrial-like
Gglean013865-TA	putative ribosomal RNA methyltransferase NOP2-like
Gglean016637-TA	putative ribosomal RNA methyltransferase CG11447-like
Gglean012037-TA	mRNA cap guanine-N7 methyltransferase-like
**Other methyltransferases**	
Gglean002394-TA	N-methyltransferase
Gglean005036-TA	N-methyltransferase
Gglean002431-TA	glycine N-methyltransferase-like
Gglean002518-TA	methylated-DNA--protein-cysteine methyltransferase-like
Gglean002940-TA	uncharacterized methyltransferase WBSCR22-like
Gglean002941-TA	uncharacterized methyltransferase WBSCR22-like
Gglean002958-TA	methyltransferase-like protein 14-like
Gglean000178-TA	putative methyltransferase 10 domain containing protein
Gglean004158-TA	*hemK* methyltransferase family member 2-like
Gglean014477-TA	*hemK* methyltransferase family member 2-like
Gglean004806-TA	protein-S-isoprenylcysteine O-methyltransferase-like
Gglean000232-TA	methyltransferase-like protein 7A-like
Gglean013764-TA	methyltransferase-like protein 7A-like
Gglean017422-TA	methyltransferase-like protein 7A-like
Gglean017423-TA	methyltransferase-like protein 7A-like
Gglean017424-TA	methyltransferase-like protein 7A-like
Gglean017425-TA	methyltransferase-like protein 7A-like
Gglean008770-TA	methyltransferase-like protein 7B-like
Gglean005991-TA	trans-aconitate 3-methyltransferase-like
Gglean006239-TA	methyltransferase-like protein 4-like
Gglean006512-TA	alpha N-terminal protein methyltransferase 1A-like
Gglean007132-TA	probable methyltransferase BCDIN3D-like
Gglean007153-TA	O-methyltransferase family 3 protein
Gglean007741-TA	hexaprenyldihydroxybenzoate methyltransferase, mitochondrial-like
Gglean000653-TA	probable methyltransferase C20orf7 homolog, mitochondrial-like
Gglean012245-TA	methyltransferase-like protein 17, mitochondrial-like
Gglean016644-TA	aminomethyltransferase, mitochondrial-like
Gglean007993-TA	N-acetylserotonin O-methyltransferase-like protein-like

Gglean008987-TA	methyltransferase-like protein 5-like
Gglean009011-TA	ftsJ methyltransferase domain-containing protein 1-like
Gglean009310-TA	putative methyltransferase NSUN6-like
Gglean001473-TA	putative methyltransferase NSUN4-like
Gglean009487-TA	methyltransferase-like protein 21D-like
Gglean012333-TA	methyltransferase-like protein 6-like
Gglean013284-TA	methyltransferase-like protein 22-like
Gglean013436-TA	putative methyltransferase DDB_G0268948-like
Gglean013717-TA	serine hydroxymethyltransferase, cytosolic-like
Gglean001193-TA	calmodulin-lysine N-methyltransferase-like
Gglean014590-TA	leucine carboxyl methyltransferase 1-like
Gglean014680-TA	methyltransferase-like protein 13-like
Gglean015780-TA	probable methyltransferase-like protein 15-like
Gglean016883-TA	protein-L-isoaspartate(D-aspartate) O-methyltransferase-like
Gglean016943-TA	methyltransferase-like protein 2-like
Gglean001688-TA	methyltransferase-like protein 9-like

Table S16 The predicted sex-determining related genes in the *N. cucumeris* genome

Gene ID	Gene name	Functional annotation
**signal cascade genes**		
Gglean014548-TA	*Sex lethal*, *Sxl*	ELAV-like protein 2-like
Gglean003914-TA	*transformer*, *tra*	RNA-binding protein 39-like
Gglean006885-TA	*transformer*, *tra*	huntingtin interacting protein
Gglean008070-TA	*transformer*, *tra*	uncharacterized protein LOC100906475
Gglean000049-TA	*transformer2*, *tra2*	transformer-2 protein homolog beta-like
Gglean017257-TA	*doublesex*, *dsx*	uncharacterized protein LOC100908210
Gglean010651-TA	*doublesex*, *dsx*	Doublesex- and mab-3-related transcription factor A2
Gglean017283-TA	*doublesex*, *dsx*	uncharacterized protein LOC100908352
Gglean007133-TA	*fruitless*, *fru*	uncharacterized protein LOC100906870
**X:A signal transducer genes**		
Gglean007363-TA	*sisterless B scute*, *sisB sc*	lethal of scute
Gglean005407-TA	*deadpan*, *dpn*	protein deadpan-like
Gglean001091-TA	*runt*, *run*	uncharacterized protein LOC100906311
Gglean013198-TA	*intersex*, *ix*	mediator of RNA polymerase II transcription subunit 29-like
Gglean012257-TA	*intersex*, *ix*	mediator of RNA polymerase II transcription subunit 29-like
Gglean000286-TA	*hnRNPA/B-like 28*, *Hrp28*	heterogeneous nuclear ribonucleoprotein 27C-like
Gglean004674-TA	*daughterless*	protein daughterless-like
Gglean013046-TA	*daughterless*	protein daughterless-like
**dosage compensation genes**		
Gglean001050-TA	*male-specific lethal 3*	male-specific lethal 3 homolog
Gglean001139-TA	*male-specific lethal 1*	male-specific lethal 1 homolog
**sex peptide receptor**		
Gglean011294-TA	*sex peptide receptor*, *spr*	thyrotropin-releasing hormone receptor-like
**Heterochromatin Protein**		
Gglean007899-TA	*Heterochromatin Protein 1L chromoshadow domain, HP1Lcsd*	sarcalumenin-like
Gglean008327-TA	*HP1*	heterochromatin protein 1-like
Gglean010210-TA	*HP1Lcsd*	uncharacterized protein LOC100906063
Gglean017167-TA	*heterochromatin protein 1B*, *Hp1b*	chromobox protein homolog 3-like
Gglean003559-TA	*heterochromatin protein 1B*, *Hp1b*	chromobox protein homolog 3-like
Gglean010127-TA	*Heterochromatin protein 5*, *HP5*	GA28568

Table S17 The predicted sex-determining related genes in the genomes of Acari and *Limulus polyphemus*

Gene	***M. occidentalis***	***N. cucumeris***	***I. scapularis***	***T. urticae***	***S. scabiei***	***L. polyphemus***
Sxl	1	1	1	0	0	1
tra	1	3	1	2	1	1
Tra2	1	1	1	0	1	4
dsx	1	3	0	1	1	3
fru	2	2	0	2	2	8
sisB sc	1	1	1	0	0	1
dpn	1	1	1	2	1	2

Table S18 Identification of the four conserved limb gap genes in the *N. cucumeris* genome.

Gene	Reference	Gene ID
Distal-less, *Dll*	FBgn0000157	Gglean017511-TA
Homothorax, *hth*	FBgn0005677	Gglean017512-TA
dachshund, *dac*	FBgn0000611	Gglean017513-TA
Extradenticle, *exd*	FBgn0001235	Gglean017514-TA<br>(without terminator codon)

Table S19 Identification of the pair-rule and segment-polarity genes in the *N. cucumeris* genome.

Gene	Reference	Gene ID	Functional annotation
**pair-rule genes**			
hairy, *h*	FBgn0001168	Gglean005407-TA	protein deadpan-like
even-skipped, *eve*	FBgn0000606	Gglean014402-TA	homeobox even-skipped homolog protein 1-like
runt, *run*	FBgn0003300	Gglean001091-TA	uncharacterized protein LOC100906311
odd-skipped, *odd*	FBgn0002985	Gglean004593-TA	putative zinc finger protein 724-like
		Gglean016652-TA	zinc finger protein, putative
fushi tarazu, *ftz*	FBgn0001077	Gglean017505-TA	
fushi tarazu transcription factor 1, *ftz-f1*	FBgn0001078	Gglean017506-TA	
paired, *prd*	FBgn0003145	Gglean002931-TA	protein gooseberry-neuro-like
sloppy-paired, *slp*	FBgn0003430	Gglean013091-TA	uncharacterized protein LOC100909139
**segment-polarity genes**			
engrailed, *en*	FBgn0000577	Gglean009236-TA	uncharacterized protein LOC100908620
hedgehog, *hh*	FBgn0004644	Gglean004680-TA	
		Gglean004851-TA	sonic hedgehog protein-like
wingless, *wg*	FBgn0284084	Gglean002092-TA	protein Wnt-5b-like

Table S20 Identification of the conserved *Hox* genes in the *N. cucumeris* genome.

Gene	Reference	Gene ID
labial, *lab*	FBgn0002522	Gglean017502-TA
proboscipedia, *pb*	FBgn0051481	Gglean017503-TA
zerknullt, *zen*	FBgn0004053	no
bicoid, *bcd*	FBgn0000166	no
Deformed, *Dfd*	FBgn0000439	Gglean017504-TA
Sex combs reduced, *Scr*	FBgn0003339	no
fushi tarazu, *ftz*	FBgn0001077	Gglean017505-TA
fushi tarazu transcription factor 1, *ftz-f1*	FBgn0001078	Gglean017506-TA
Antennapedia, *Antp*	FBgn0260642	Gglean017507-TA
Ultrabithorax, *Ubx*	FBgn0003944	Gglean017508-TA
Abdominal-A, *Abd-A*	FBgn0000014	Gglean017509-TA
Abdominal-B, *Abd-B*	FBgn0000015	Gglean017510-TA

Table S21 The predicted zinc finger proteins in *N. cucumeris*.

Gene ID	Functional annotation	Subgroup
Gglean007097-TA	AN1-type zinc finger protein 2B-like	AN1-type
Gglean008592-TA	AN1-type zinc finger protein 1-like	AN1-type
Gglean011466-TA	AN1-type zinc finger protein 4-like	AN1-type
Gglean008307-TA	B-box type zinc finger protein ncl-1-like	B box-type
Gglean008308-TA	B-box type zinc finger protein ncl-1-like	B box-type
Gglean017462-TA	B-box type zinc finger protein ncl-1-like	B box-type
Gglean003142-TA	zinc finger BED domain-containing protein 1-like	BED-type
Gglean003156-TA	zinc finger BED domain-containing protein 1-like	BED-type
Gglean004586-TA	zinc finger BED domain-containing protein 5-like	BED-type
Gglean004937-TA	zinc finger BED domain-containing protein 5-like	BED-type
Gglean004971-TA	zinc finger BED domain-containing protein 5-like	BED-type
Gglean006118-TA	zinc finger BED domain-containing protein 5-like	BED-type
Gglean006119-TA	zinc finger BED domain-containing protein 5-like	BED-type
Gglean007530-TA	zinc finger BED domain-containing protein 1-like	BED-type
Gglean008753-TA	zinc finger BED domain-containing protein 1-like	BED-type
Gglean010257-TA	zinc finger BED domain-containing protein 5-like	BED-type
Gglean010341-TA	zinc finger BED domain-containing protein 1-like	BED-type
Gglean010376-TA	zinc finger BED domain-containing protein 1-like	BED-type
Gglean010408-TA	zinc finger BED domain-containing protein 5-like	BED-type
Gglean010900-TA	zinc finger BED domain-containing protein 5-like	BED-type
Gglean013325-TA	zinc finger BED domain-containing protein 5-like	BED-type
Gglean015818-TA	zinc finger BED domain-containing protein 1-like	BED-type
Gglean016773-TA	zinc finger BED domain-containing protein 5-like	BED-type
Gglean016774-TA	zinc finger BED domain-containing protein 5-like	BED-type
Gglean003713-TA	SCAN domain-containing protein 3-like	BED-type
Gglean011851-TA	SCAN domain-containing protein 3-like	BED-type
Gglean015604-TA	SCAN domain-containing protein 3-like	BED-type
Gglean016687-TA	SCAN domain-containing protein 3-like	BED-type
Gglean016688-TA	SCAN domain-containing protein 3-like	BED-type
Gglean001821-TA	zinc finger protein 704-like	C2H2-type
Gglean001822-TA	zinc finger protein 395-like	C2H2-type
Gglean001844-TA	Zinc finger protein 512B	C2H2-type
Gglean002439-TA	zinc finger protein 512B-like	C2H2-type
Gglean002842-TA	zinc finger protein 512B-like	C2H2-type
Gglean002843-TA	zinc finger protein 512B-like	C2H2-type
Gglean002844-TA	zinc finger protein 512B-like	C2H2-type
Gglean003088-TA	zinc finger protein 512B-like	C2H2-type
Gglean003089-TA	zinc finger protein 512B-like	C2H2-type
Gglean003090-TA	zinc finger protein 512B-like	C2H2-type
Gglean004340-TA	zinc finger protein 512B-like	C2H2-type
Gglean006205-TA	zinc finger protein 512B-like	C2H2-type
Gglean009120-TA	zinc finger protein 512B-like	C2H2-type
Gglean009680-TA	zinc finger protein 512B-like	C2H2-type
Gglean011777-TA	zinc finger protein 512B-like	C2H2-type
Gglean012931-TA	zinc finger protein 512B-like	C2H2-type
Gglean013011-TA	zinc finger protein 512B-like	C2H2-type
Gglean013012-TA	zinc finger protein 512B-like	C2H2-type
Gglean016805-TA	zinc finger protein 512B-like	C2H2-type
Gglean001938-TA	zinc finger protein Xfin-like, KRAB domain-containing	C2H2-type
Gglean011037-TA	zinc finger protein Xfin-like, KRAB domain-containing	C2H2-type
Gglean005557-TA	PR domain zinc finger protein 14-like	C2H2-type

Gglean007058-TA	PR domain zinc finger protein 5-like	C2H2-type
Gglean016603-TA	PR domain zinc finger protein 1-like	C2H2-type
Gglean001742-TA	PR domain zinc finger protein 13-like	C2H2-type
Gglean002351-TA	zinc finger protein 460-like	C2H2-type
Gglean002353-TA	zinc finger protein Gfi-1-like	C2H2-type, TF(r)
Gglean006041-TA	zinc finger protein Gfi-1-like	C2H2-type, TF(r)
Gglean002365-TA	zinc finger protein 271-like	C2H2-type
Gglean004479-TA	zinc finger protein 271-like	C2H2-type
Gglean006624-TA	zinc finger protein 271-like	C2H2-type
Gglean008386-TA	zinc finger protein 271-like	C2H2-type
Gglean009111-TA	zinc finger protein 271-like	C2H2-type
Gglean015157-TA	zinc finger protein 271-like	C2H2-type
Gglean002467-TA	zinc finger protein Pegasus-like	C2H2-type, TF(r)
Gglean002576-TA	zinc finger protein 551-like	C2H2-type
Gglean002577-TA	zinc finger protein 551-like	C2H2-type
Gglean002578-TA	zinc finger protein 775-like	C2H2-type
Gglean002579-TA	zinc finger protein 775-like	C2H2-type
Gglean000554-TA	zinc finger protein 775-like	C2H2-type
Gglean000555-TA	zinc finger protein 775-like	C2H2-type
Gglean002583-TA	zinc finger protein 90-like	C2H2-type
Gglean002584-TA	zinc finger Y-chromosomal protein 1-like	C2H2-type, TF(a)
Gglean002587-TA	KRAB box and zinc finger, C2H2 type domain	C2H2-type
Gglean002890-TA	zinc finger protein 112 homolog	C2H2-type
Gglean003220-TA	zinc finger protein 658-like	C2H2-type
Gglean003221-TA	zinc finger protein 658-like	C2H2-type
Gglean003477-TA	zinc finger protein 510-like	C2H2-type
Gglean003732-TA	zinc finger protein 250-like	C2H2-type
Gglean000339-TA	zinc finger protein 250-like	C2H2-type
Gglean003874-TA	zinc finger protein 830-like	C2H2-type
Gglean003925-TA	zinc finger protein 358-like	C2H2-type
Gglean004149-TA	zinc finger protein 569-like	C2H2-type
Gglean004372-TA	zinc finger protein 277-like	C2H2-type
Gglean001150-TA	zinc finger protein 277-like	C2H2-type
Gglean001151-TA	zinc finger protein 277-like	C2H2-type
Gglean001564-TA	zinc finger protein 277-like	C2H2-type
Gglean004593-TA	putative zinc finger protein 724-like	C2H2-type
Gglean004742-TA	zinc finger protein SNAI2-like	C2H2-type, TF(r)
Gglean000550-TA	zinc finger protein SNAI2-like	C2H2-type, TF(r)
Gglean004982-TA	zinc finger protein with KRAB and SCAN domains 1-like	C2H2-type
Gglean015243-TA	zinc finger protein with KRAB and SCAN domains 1-like	C2H2-type
Gglean005046-TA	zinc finger protein 394-like	C2H2-type, TF(r)
Gglean010524-TA	zinc finger protein 394-like	C2H2-type, TF(r)
Gglean005299-TA	zinc finger protein 563-like, KRAB domain-containing	C2H2-type
Gglean005316-TA	zinc finger protein 567-like, KRAB domain-containing	C2H2-type
Gglean000222-TA	zinc finger protein 287-like, KRAB domain-containing	C2H2-type
Gglean015410-TA	zinc finger protein 287-like, KRAB domain-containing	C2H2-type
Gglean000241-TA	zinc finger protein 45-like	C2H2-type
Gglean000332-TA	putative zinc finger transcription factor protein 17	C2H2-type
Gglean005848-TA	zinc finger protein 324B-like, KRAB domain-containing	C2H2-type
Gglean011183-TA	zinc finger protein 324B-like, KRAB domain-containing	C2H2-type
Gglean005993-TA	zinc finger homeobox protein 4	C2H2-type
Gglean006039-TA	zinc finger protein 62 homolog	C2H2-type
Gglean007597-TA	zinc finger protein 62 homolog	C2H2-type

Gglean009612-TA	zinc finger protein 62 homolog	C2H2-type
Gglean012844-TA	zinc finger protein 62 homolog	C2H2-type
Gglean014682-TA	zinc finger protein 62 homolog	C2H2-type
Gglean006070-TA	gastrula zinc finger protein XlCGF57.1-like	C2H2-type
Gglean000543-TA	gastrula zinc finger protein XlCGF26.1-like	C2H2-type
Gglean000544-TA	gastrula zinc finger protein XlCGF26.1-like	C2H2-type
Gglean006260-TA	Zinc finger protein 418, KRAB domain-containing	C2H2-type
Gglean006349-TA	ankyrin repeat and zinc finger domain-containing protein 1-like	C2H2-type
Gglean006370-TA	zinc finger protein 808-like	C2H2-type
Gglean000548-TA	zinc finger protein 808-like	C2H2-type
Gglean013040-TA	zinc finger protein 808-like	C2H2-type
Gglean015840-TA	zinc finger protein 808-like	C2H2-type
Gglean006418-TA	zinc finger protein 532-like	C2H2-type
Gglean006419-TA	zinc finger protein 532-like	C2H2-type
Gglean006531-TA	zinc finger protein 341-like	C2H2-type
Gglean006724-TA	zinc finger protein ZIC 4-like	C2H2-type
Gglean006803-TA	zinc finger protein 235-like	C2H2-type
Gglean009041-TA	zinc finger protein 235-like	C2H2-type
Gglean007626-TA	zinc finger protein 142-like, KRAB domain-containing	C2H2-type
Gglean007705-TA	zinc finger protein 425-like	C2H2-type, TF(r)
Gglean001958-TA	zinc finger protein 91-like	C2H2-type
Gglean005282-TA	zinc finger protein 91-like	C2H2-type
Gglean007707-TA	zinc finger protein 91-like	C2H2-type
Gglean000402-TA	zinc finger protein 593-like	C2H2-type
Gglean000539-TA	Zinc finger protein 596	C2H2-type
Gglean000559-TA	zinc finger protein 596-like	C2H2-type
Gglean016889-TA	zinc finger protein 596-like	C2H2-type
Gglean000540-TA	zinc finger protein 568-like	C2H2-type, TF(r)
Gglean000541-TA	zinc finger protein 568-like	C2H2-type, TF(r)
Gglean000542-TA	zinc finger protein 568-like	C2H2-type, TF(r)
Gglean008603-TA	zinc finger protein 568-like	C2H2-type, TF(r)
Gglean009962-TA	zinc finger protein 568-like	C2H2-type, TF(r)
Gglean000545-TA	zinc finger protein 711-like	C2H2-type, TF
Gglean000546-TA	zinc finger protein 711-like, neuron development	C2H2-type, TF
Gglean000547-TA	zinc finger protein 813-like	C2H2-type
Gglean000549-TA	Zinc finger protein 26	C2H2-type
Gglean000556-TA	zinc finger protein 184-like, KRAB domain-containing	C2H2-type
Gglean000558-TA	zinc finger protein 184-like, KRAB domain-containing	C2H2-type
Gglean000560-TA	zinc finger protein 182-like	C2H2-type
Gglean000595-TA	zinc finger protein 814-like	C2H2-type
Gglean008016-TA	zinc finger, C2H2 type	C2H2-type
Gglean008606-TA	zinc finger protein 236-like	C2H2-type
Gglean008615-TA	zinc finger protein 408-like	C2H2-type
Gglean008745-TA	zinc finger protein 69-like	C2H2-type
Gglean008779-TA	zinc finger protein 229-like	C2H2-type
Gglean008851-TA	zinc finger protein 567-like	C2H2-type
Gglean008906-TA	zinc finger autosomal protein-like	C2H2-type, TF(a)
Gglean008950-TA	zinc finger protein 64 homolog	C2H2-type
Gglean008951-TA	zinc finger protein 64 homolog	C2H2-type
Gglean015906-TA	zinc finger protein 64 homolog	C2H2-type
Gglean009580-TA	zinc finger and BTB domain-containing protein 24-like	C2H2-type
Gglean009653-TA	zinc finger protein 706-like	C2H2-type, TF(r)
Gglean017360-TA	zinc finger protein 706-like	C2H2-type, TF(r)

Gglean011053-TA	zinc finger protein 449-like	C2H2-type
Gglean011258-TA	zinc finger, C2H2 type	C2H2-type
Gglean011546-TA	zinc finger protein 28-like	C2H2-type
Gglean000830-TA	zinc finger protein 407-like	C2H2-type
Gglean012205-TA	zinc finger protein 2-like	C2H2-type
Gglean012707-TA	zinc finger protein 99-like	C2H2-type
Gglean012708-TA	zinc finger protein 99-like	C2H2-type
Gglean012864-TA	zinc finger protein 92-like	C2H2-type
Gglean013313-TA	Zinc finger protein 850	C2H2-type
Gglean013504-TA	zinc finger protein 143-like	C2H2-type, TF(a)
Gglean013520-TA	zinc finger protein 33B-like	C2H2-type
Gglean013606-TA	zinc finger protein 674-like	C2H2-type
Gglean013716-TA	zinc finger protein 12, KRAB domain-containing	C2H2-type
Gglean013794-TA	zinc finger protein 263-like, KRAB domain-containing	C2H2-type
Gglean001038-TA	zinc finger protein 595-like	C2H2-type
Gglean001192-TA	zinc finger protein 93-like	C2H2-type, TF(r)
Gglean014798-TA	zinc finger protein 189-like	C2H2-type
Gglean015040-TA	zinc finger protein 358-like	C2H2-type
Gglean015283-TA	zinc finger protein ZFAT-like	C2H2-type, TF(r)
Gglean015360-TA	zinc finger protein 70-like	C2H2-type
Gglean015534-TA	zinc finger protein 460-like	C2H2-type
Gglean015591-TA	zinc finger protein 460-like	C2H2-type
Gglean015668-TA	Zinc finger protein 335	C2H2-type, TF(a)
Gglean016399-TA	zinc finger protein 366-like	C2H2-type
Gglean016480-TA	zinc finger protein 564-like	C2H2-type
Gglean017335-TA	zinc finger protein 564-like	C2H2-type
Gglean016593-TA	zinc finger protein 112-like	C2H2-type
Gglean016601-TA	zinc finger protein 161 homolog	C2H2-type
Gglean001721-TA	fez family zinc finger protein 2-like	C2H2-type, TF(r)
Gglean002680-TA	serrate RNA effector molecule homolog B-like	C2H2-type, RNAi
Gglean001085-TA	serrate RNA effector molecule homolog B-like	C2H2-type, RNAi
Gglean013848-TA	serrate RNA effector molecule homolog B-like	C2H2-type, RNAi
Gglean002831-TA	zinc finger CCCH domain-containing protein 13-like	C3H1-type
Gglean000167-TA	zinc finger CCCH domain-containing protein 15 homolog	C3H1-type
Gglean004260-TA	zinc finger RNA-binding protein-like	C3H1-type
Gglean014731-TA	zinc finger CCCH domain-containing protein 10-like	C3H1-type
Gglean001227-TA	putative zinc finger CCCH domain-containing protein 57-like	C3H1-type
Gglean009913-TA	zinc finger C4H2 domain-containing protein-like	C4H2-type
Gglean012379-TA	zinc finger protein ZPR1-like	C4-type
Gglean014458-TA	zinc finger protein 330 homolog	C4-type
Gglean003666-TA	CCHC-type zinc finger protein CG3800-like	CCHC-type
Gglean000512-TA	zinc finger CCHC domain-containing protein 9-like	CCHC-type
Gglean016906-TA	zinc finger CCHC domain-containing protein 4-like	CCHC-type
Gglean016590-TA	FLYWCH-type zinc finger-containing protein 1-like	FLYWCH-type
Gglean003686-TA	zinc finger FYVE domain-containing protein 9-like	FYVE-type
Gglean004646-TA	GATA zinc finger domain-containing protein 1-like	GATA-type
Gglean010240-TA	zinc finger HIT domain-containing protein 3-like	HIT-type
Gglean016908-TA	zinc finger HIT domain-containing protein 1-like	HIT-type
Gglean001140-TA	zinc finger matrin-type protein 2-like	Matrin-type
Gglean002305-TA	zinc finger MYM-type protein 1-like	MYM-type
Gglean002346-TA	Zinc finger MYM-type protein	MYM-type
Gglean002974-TA	zinc finger MYM-type protein 2-like	MYM-type
Gglean003164-TA	Zinc finger MYM-type protein 1	MYM-type

Gglean003599-TA	zinc finger MYM-type protein 5-like	MYM-type
Gglean004207-TA	zinc finger MYM-type protein 1-like	MYM-type
Gglean004956-TA	zinc finger MYM-type protein 1-like	MYM-type
Gglean005379-TA	zinc finger MYM-type protein 1-like	MYM-type
Gglean005473-TA	zinc finger MYM-type protein 1-like	MYM-type
Gglean005694-TA	zinc finger MYM-type protein 1-like	MYM-type
Gglean005721-TA	zinc finger MYM-type protein 1-like	MYM-type
Gglean006341-TA	zinc finger MYM-type protein 1-like	MYM-type
Gglean006342-TA	zinc finger MYM-type protein 1-like	MYM-type
Gglean007594-TA	zinc finger MYM-type protein 1-like	MYM-type
Gglean008368-TA	zinc finger MYM-type protein 1-like	MYM-type
Gglean011460-TA	zinc finger MYM-type protein 1-like	MYM-type
Gglean011826-TA	zinc finger MYM-type protein 1-like	MYM-type
Gglean012816-TA	zinc finger MYM-type protein 1-like	MYM-type
Gglean013725-TA	zinc finger MYM-type protein 1-like	MYM-type
Gglean013798-TA	zinc finger MYM-type protein 1-like	MYM-type
Gglean016573-TA	zinc finger MYM-type protein 1-like	MYM-type
Gglean016574-TA	zinc finger MYM-type protein 1-like	MYM-type
Gglean017107-TA	zinc finger MYM-type protein 1-like	MYM-type
Gglean017324-TA	zinc finger MYM-type protein 1-like	MYM-type
Gglean002538-TA	NF-X1-type zinc finger protein NFXL1-like	NF-X1-type, TF
Gglean004226-TA	NFX1-type zinc finger-containing protein 1-like	NF-X1-type, TF
Gglean004227-TA	NFX1-type zinc finger-containing protein 1-like	NF-X1-type, TF
Gglean004228-TA	NFX1-type zinc finger-containing protein 1-like	NF-X1-type, TF
Gglean007404-TA	NFX1-type zinc finger-containing protein 1-like	NF-X1-type, TF
Gglean007473-TA	NF-X1-type zinc finger protein NFXL1-like	NF-X1-type, TF
Gglean011674-TA	NFX1-type zinc finger-containing protein 1-like	NF-X1-type, TF
Gglean011675-TA	NFX1-type zinc finger-containing protein 1-like	NF-X1-type, TF
Gglean011676-TA	NFX1-type zinc finger-containing protein 1-like	NF-X1-type, TF
Gglean011678-TA	NFX1-type zinc finger-containing protein 1-like	NF-X1-type, TF
Gglean016920-TA	NFX1-type zinc finger-containing protein 1-like	NF-X1-type, TF
Gglean009764-TA	zinc finger protein DPF3-like, mediate the binding to acetylated histones	PHD-type
Gglean011512-TA	zinc finger protein DPF3-like, mediate the binding to acetylated histones	PHD-type
Gglean010643-TA	bromodomain adjacent to zinc finger domain protein 1A-like	PHD-type
Gglean016505-TA	bromodomain adjacent to zinc finger domain protein 1A-like	PHD-type
Gglean016594-TA	PHD finger-like domain-containing protein 5A-like	PHD-type
Gglean003775-TA	zinc finger protein-like 1-like	RING-type
Gglean007996-TA	zinc finger MIZ domain-containing protein 1	SP-RING-type
Gglean006899-TA	zinc finger SWIM domain-containing protein 5-like	SWIM-type
Gglean006900-TA	zinc finger SWIM domain-containing protein 5-like	SWIM-type
Gglean010104-TA	zinc finger SWIM domain-containing protein KIAA0913-like	SWIM-type
Gglean013972-TA	zinc finger protein 622-like, embryonic development	U1-type
Gglean003553-TA	ZZ-type zinc finger-containing protein 3-like	ZZ-type
Gglean002134-TA	zinc finger protein	/
Gglean004637-TA	zinc finger protein	/
Gglean000557-TA	zinc finger protein	/
Gglean000561-TA	zinc finger protein	/
Gglean008593-TA	zinc finger protein	/
Gglean009173-TA	zinc finger protein	/
Gglean011850-TA	zinc finger protein	/
Gglean014540-TA	zinc finger protein	/

Gglean016652-TA	zinc finger protein	/
Gglean017125-TA	zinc finger protein	/

Table S22 Identification of the genes involving in the Notch signaling pathway in the *N. cucumeris* genome.

Gene ID	Gene name	Functional annotation
Gglean012785-TA	*Notch*, *N*	neurogenic locus Notch protein-like
Gglean012786-TA	*Notch*, *N*	neurogenic locus Notch protein-like
Gglean012787-TA	*Notch*, *N*	neurogenic locus Notch protein-like
Gglean016194-TA	*Delta*, *Dl*	neurogenic locus protein delta-like
	Serrate, *Ser*	
Gglean004368-TA	*Suppressor of Hairless*, *Su(H)*	suppressor of hairless protein homolog
Gglean005249-TA	*Bx42*	SNW domain-containing protein 1-like
Gglean014981-TA	*Beadex*, *Bx*	insulin gene enhancer protein isl-1-like
Gglean015396-TA	*Chip*, *Chi*	LIM domain-binding protein 2-like
Gglean016352-TA	*mind bomb 1*, *mib1*	E3 ubiquitin-protein ligase MIB1-like
Gglean004027-TA	*neutralized*, *neur*	Protein neuralized
Gglean016335-TA	*Gcn5 acetyltransferase*, *Gcn5*	histone acetyltransferase KAT2B
Gglean004265-TA	*Histone deacetylase 1*, *HDAC1*	histone deacetylase 1-like
Gglean004272-TA	*Histone deacetylase 3*, *HDAC3*	histone deacetylase 3-like
Gglean001306-TA	*Histone deacetylase 4*, *HDAC4*	histone deacetylase 4-like
Gglean003519-TA	*Histone deacetylase 6*, *HDAC4*	histone deacetylase 6-like
Gglean009200-TA	*C-terminal Binding Protein, CtBP*	C-terminal-binding protein-like
Gglean012866-TA	*nejire*, *nej*	uncharacterized protein LOC100909321
Gglean003189-TA	*groucho*, *gro*	transducin-like enhancer protein 4
Gglean012143-TA	*fringe*, *fng*	fringe glycosyltransferase-like

Table S23 Identification of the genes involving in the Hedgehog (Hh) signaling pathway in the *N. cucumeris* genome.

Gene ID	Gene name	Functional annotation
Gglean004680-TA	*hedgehog*, *hh*	
Gglean004851-TA	*hedgehog*, *hh*	sonic hedgehog protein-like, sHh
Gglean010611-TA	*patched*, *ptc*	protein patched-like
Gglean016665-TA	*Patched-related*, *ptr*	patched domain-containing protein 3-like
Gglean000973-TA	*smoothened*, *smo*	smoothened homolog
Gglean003043-TA	*Suppressor of fused*, *Su(fu)*	suppressor of fused homolog
Gglean000425-TA	*cubitus interruptus*, *ci*	uncharacterized protein LOC100907670
Gglean008847-TA	*G protein-coupled receptor kinase 2*, *Gprk2*	G protein-coupled receptor kinase 5-like
Gglean010015-TA	*Protein kinase, cAMP-dependent, catalytic subunit 1*, *Pka-C1*	cAMP-dependent protein kinase catalytic subunit alpha-like
Gglean000631-TA	*Protein kinase, cAMP-dependent, catalytic subunit 2*, *Pka-C2*	cAMP dependent protein kinase catalytic subunit
Gglean017515	*Protein kinase, cAMP-dependent, catalytic subunit 3*, *Pka-C3*	cAMP dependent protein kinase catalytic subunit
Gglean002971-TA	*shaggy*, *sgg*	glycogen synthase kinase-3 beta-like, GSK3β
Gglean015280-TA	*SKP1-related A*, *SkpA*	S-phase kinase-associated protein 1-like
Gglean011863-TA	*discs overgrown*, *dco*	casein kinase I isoform epsilon-like
Gglean009280-TA	*gilgamesh, gish*	casein kinase I isoform gamma-3-like
Gglean000844-TA	*SMAD specific E3 ubiquitin protein ligase*, *Smurf*	E3 ubiquitin-protein ligase SMURF2-like
Gglean012084-TA	*Ubiquitin specific protease 8*, *Usp8*	ubiquitin carboxyl-terminal hydrolase 4-like
Gglean013831-TA	*Cullin 1*, *Cul1*	cullin-1-like
Gglean013843-TA	*Cullin 2*, *Cul2*	cullin-2-like
Gglean008080-TA	*Cullin 3*, *Cul3*	cullin-3
Gglean002599-TA	*Cullin 4*, *Cul4*	cullin-4A
Gglean015116-TA	*Cullin 5*, *Cul5*	cullin-5-like
Gglean015115-TA	*Cullin 5*, *Cul5*	cullin-5-like
Gglean007009-TA	*decapentaplegic*, *dpp*	bone morphogenetic protein 2-like
Gglean000707-TA	*roadkill*, *rdx*	protein roadkill-like

Table S24 Identification of the genes involving in the Hippo signaling pathway in the *N. cucumeris* genome.

Gene ID	Gene name	Functional annotation
Gglean005781-TA	*hippo*, *hpo*	serine/threonine-protein kinase 3-like
Gglean005678-TA	*warts, wts*	uncharacterized protein LOC100903008
Gglean009794-TA	*mob as tumor suppressor*, *mats*	MOB kinase activator-like 1-like
Gglean006474-TA	*yorkie*, *yki*	yorkie homolog
Gglean003222-TA	*Merlin*, *Mer*	merlin-like
Gglean016828-TA	*Merlin*, *Mer*	merlin-like
Gglean016631-TA	*Merlin*, *Mer*	moesin/ezrin/radixin homolog 1-like
Gglean005992-TA	*misshapen*, *msn*	misshapen-like kinase 1-like
Gglean011722-TA	*happyhour*, *hppy*	uncharacterized protein LOC100900712
Gglean000920-TA	*14-3-3zeta*	14-3-3 protein zeta-like
Gglean012442-TA	*14-3-3zeta*	14-3-3 protein zeta-like
Gglean005844-TA	*Ajuba LIM protein*, *jub*	LIM domain-containing protein jub-like
Gglean017516-TA	*teashirt*, *tsh*	
Gglean013873-TA	*scalloped*, *sd*	transcriptional enhancer factor TEF-3-like
Gglean012858-TA	*Mothers against dpp*, *Mad*	protein mothers against dpp-like
Gglean008648-TA	*Cyclin E*, *CycE*	G1/S-specific cyclin-E1-like
Gglean014601-TA	*four-jointed*, *fj*	protein four-jointed-like
Gglean000881-TA	*Death-associated inhibitor of apoptosis 1*, *Diap1*	baculoviral IAP repeat-containing protein 2-like
Gglean010122-TA	*kibra*	protein kibra-like

Table S25 The predicted eye specification and phototransduction related genes in the *N. cucumeris* genome.

Gene ID	Gene name	Functional annotation
**eye specification and retinal differentiation**		
Gglean003351-TA	*eyeless* (*ey*)	paired box protein Pax-6-like
Gglean001941-TA	*sine oculis* (*so*)	homeobox protein SIX1-like
Gglean016071-TA	*eyes absent* (*eya*)	developmental protein eyes absent-like
Gglean003458-TA	*atonal* (*ato*)	bHLH transcription factor
Gglean002438-TA	*homothorax* (*hth*)	homeobox protein Meis1-like
Gglean017516-TA	*teashirt*, *tsh*	
Gglean000363-TA	*Rx1*	retinal homeobox protein Rx1-like
Gglean010890-TA	*Rx2*	retina and anterior neural fold homeobox protein 2-like
Gglean010891-TA	*Rx2*	retina and anterior neural fold homeobox protein 2-like
Gglean001454-TA	*Rx3*	retinal homeobox protein Rx3-like
**phototransduction**		
Gglean005813-TA	*neither inactivation nor afterpotential G*, *ninaG*	glucose dehydrogenase [acceptor]-like
Gglean011164-TA	*ninaA*	peptidyl-prolyl cis-trans isomerase B-like
Gglean000228-TA	*ninaC*	myosin-IIIb
Gglean004279-TA	*inactivation no afterpotential E*, *inaE*	sn1-specific diacylglycerol lipase alpha-like
Gglean015961-TA	*inaE*	multiple PDZ domain protein-like
Gglean014284-TA	*inaC*	protein kinase C, brain isozyme-like
Gglean007816-TA	*arrestin 1*, *Arr1*	arrestin domain-containing protein 1-like
Gglean012528-TA	*Arr2*	probable beta-arrestin-like
Gglean013749-TA	*rdgBbeta*	protein retinal degeneration B-like
Gglean013750-TA	*rdgB*	protein retinal degeneration B-like
Gglean012794-TA	*CDP diglyceride synthetase*, *CdsA*	phosphatidate cytidylyltransferase, photoreceptor-specific-like
Gglean000437-TA	*Calx*	sodium/calcium exchanger 1-like
Gglean004326-TA	*Calx*	sodium/calcium exchanger 1-like
Gglean015878-TA	phosphatidylinositol synthase, *Pis*	putative phosphatidate phosphatase-like
Gglean002986-TA	*Cam*	calmodulin, isoform A
Gglean004155-TA	*Cam*	calmodulin-like
Gglean011284-TA	*Cam*	calmodulin-like
Gglean007465-TA	*CaMKII*	calcium/calmodulin-dependent protein kinase type II alpha chain-like
Gglean016857-TA	*Cnx99A*	calnexin-like
Gglean015269-TA	*shaker cognate b*, *Shab*	potassium voltage-gated channel protein Shab-like
Gglean015620-TA	*Syt1*	synaptotagmin 1-like
Gglean013776-TA	*Syt1*	synaptotagmin 1-like
Gglean002452-TA	*eSyt1*	extended synaptotagmin-1-like
Gglean000790-TA	*Syt4*	synaptotagmin-4-like
Gglean002662-TA	*Syt10*	synaptotagmin-10-like
Gglean002663-TA	*Syt2*	synaptotagmin-2-like
Gglean006002-TA	*Syt2*	synaptotagmin-2-like
Gglean005153-TA	*Syt15*	synaptotagmin-15-like
Gglean005154-TA	*Syt15*	synaptotagmin-15-like
Gglean010125-TA	*Syt9*	synaptotagmin-9-like
Gglean013957-TA	*Syt16*	synaptotagmin-16-like

Gglean000573-TA	*Trpm*	transient receptor potential cation channel subfamily M member 1-like
Gglean000574-TA	*Trpm*	transient receptor potential cation channel subfamily M member 1-like
Gglean008253-TA	*Trpgamma*	transient receptor potential-gamma protein-like
Gglean010595-TA	*TrpA1*	transient receptor potential cation channel subfamily A member 1-like
Gglean014192-TA	*Trp7*	short transient receptor potential channel 7-like
Gglean007781-TA	*AMPKalpha*	AMP-activated protein kinase catalytic subunit alpha-2-like
Gglean015830-TA	*AMPKalpha*	AMP-activated protein kinase catalytic subunit alpha-2-like
Gglean002077-TA	*Galphai*	guanine nucleotide-binding protein G(i) subunit alpha-like
Gglean006153-TA	*Galphao*	guanine nucleotide-binding protein G(o) subunit alpha-like isoform 1
Gglean012428-TA	*Galphao*	guanine nucleotide-binding protein G(o) subunit alpha-like
Gglean012429-TA	*Galphao*	guanine nucleotide-binding protein G(o) subunit alpha-like
Gglean011238-TA	*Galphaq*	guanine nucleotide-binding protein G(q) subunit alpha-like
Gglean015488-TA	*Galphas*	guanine nucleotide-binding protein G(s) subunit alpha-like
Gglean004740-TA	*Galpha11*	guanine nucleotide-binding protein subunit alpha-11-like
Gglean004741-TA	*Galpha11*	guanine nucleotide-binding protein subunit alpha-11-like
Gglean010864-TA	*Galpha14*	guanine nucleotide-binding protein subunit alpha-14-like
Gglean007567-TA	*Gbeta12*	guanine nucleotide-binding protein subunit beta-2-like
Gglean000501-TA	*Gbeta12*	guanine nucleotide-binding protein subunit beta-2-like
Gglean013965-TA	*Gbeta12*	guanine nucleotide-binding protein subunit beta-2-like
Gglean013966-TA	*Gbeta12*	guanine nucleotide-binding protein subunit beta-2-like
Gglean014787-TA	*Gbetal1*	guanine nucleotide-binding protein G(I)/G(S)/G(T) subunit beta-1-like
Gglean004843-TA	*Ggamma1*	guanine nucleotide-binding protein subunit gamma-1-like
Gglean006666-TA	*Ggammae*	guanine nucleotide-binding protein subunit gamma-e-like
Gglean006667-TA	*Ggammae*	guanine nucleotide-binding protein subunit gamma-e-like
Gglean016121-TA		guanine nucleotide-binding protein-like 3 homolog
Gglean017296-TA		guanine nucleotide-binding protein-like 1-like
Gglean001019-TA	*Lgr1*	follicle-stimulating hormone receptor-like
Gglean009453-TA	*Lgr3*	relaxin receptor 1-like
Gglean010207-TA	*Lgr4*	relaxin receptor 2-like
Gglean009845-TA	*Lgr5*	leucine-rich repeat-containing G-protein coupled receptor 5-like
Gglean017459-TA	*rk*	thyrotropin receptor-like
Gglean005134-TA	*Gpcr*	probable G-protein coupled receptor B0563.6-like
Gglean016975-TA	*Gpcr*	probable G-protein coupled receptor B0563.6-like
Gglean009006-TA	*Gpcr*	probable G-protein coupled receptor AH9.1-like
Gglean015986-TA	*Gpcr*	probable G-protein coupled receptor AH9.1-like
Gglean011167-TA	*Gpcr*	G-protein coupled receptor Mth-like
Gglean011168-TA	*Gpcr*	G-protein coupled receptor Mth-like
Gglean011536-TA	*Gpcr*	G-protein coupled receptor moody-like
Gglean016089-TA	*Gpcr*	G-protein coupled receptor 161-like
Gglean016090-TA	*Gpcr*	G-protein coupled receptor 161-like
Gglean013901-TA	*Gprk1*	G protein-coupled receptor kinase 1-like
Gglean008846-TA	*Gprk2*	G protein-coupled receptor kinase 5-like
Gglean008847-TA	*Gprk2*	G protein-coupled receptor kinase 5-like
Gglean013829-TA	*Baboon/Gprk3*	protein kinase 4-like
Gglean001838-TA	*PLA2*	phospholipase A2-like

Gglean001924-TA	*PLA2*	phospholipase A2-like
Gglean001799-TA	*PLA2*	phospholipase A2-like
Gglean006372-TA	*iPLA2*	calcium-independent phospholipase A2-gamma-like
Gglean007836-TA	*iPLA2*	85 kDa calcium-independent phospholipase A2-like
Gglean008204-TA	*iPLA2*	85 kDa calcium-independent phospholipase A2-like
Gglean007105-TA	*sPLA2*	salivary phospholipase A2, putative
Gglean006094-TA	*Pld*	phospholipase D1-like
Gglean001186-TA	*sl*	phosphodiesterase C gamma-1-like
Gglean007784-TA	*No receptor potential A*, *norpA*	1-phosphatidylinositol 4,5-bisphosphate phosphodiesterase
Gglean003276-TA	*Phospholipase C at 21C*, *Plc21C*	1-phosphatidylinositol 4,5-bisphosphate phosphodiesterase
Gglean011713-TA	*Phospholipase A2 activator protein*, *Plap*	inactive phospholipase C-like protein 1-like
Gglean011052-TA	*Phosphatidic Acid Phospholipase A1*, *PAPLA1*	phospholipase DDHD2

Table S26 Identification of the chemosensory receptor related genes in the *N. cucumeris* genome.

Gene ID	Functional annotation
**gustatory receptors 24**	
Gglean004138	
Gglean001183-TA	hypothetical protein IscW_ISCW002110
Gglean005187-TA	hypothetical protein IscW_ISCW018260
Gglean012758-TA	uncharacterized protein LOC100898984
Gglean012495-TA	hypothetical protein IscW_ISCW023798
Gglean015010-TA	uncharacterized protein LOC100907147
Gglean015009-TA	uncharacterized protein LOC100907580
Gglean005426-TA	uncharacterized protein LOC100907580
Gglean003324-TA	uncharacterized protein LOC100907580
Gglean014407	
Gglean002351-TA	zinc finger protein 460-like
Gglean002350-TA	uncharacterized protein LOC100902401
Gglean013527-TA	uncharacterized protein LOC100899404
Gglean013528-TA	uncharacterized protein LOC100902401
Gglean015960-TA	hypothetical protein IscW_ISCW011824
Gglean013738-TA	hypothetical protein IscW_ISCW018421
Gglean012212-TA	hypothetical protein IscW_ISCW018421
Gglean013570-TA	hypothetical protein IscW_ISCW018421
Gglean008001-TA	hypothetical protein LOTGIDRAFT_152656
Gglean008002-TA	hypothetical protein LOTGIDRAFT_152656
Gglean014565-TA	uncharacterized protein LOC100905738
Gglean005963-TA	uncharacterized protein LOC100905738
Gglean004912-TA	uncharacterized protein LOC100900146
Gglean009222-TA	uncharacterized protein LOC100900146
**ionotropic receptors 65**	
Gglean000197-TA	elongation of very long chain fatty acids protein 4-like
Gglean015166-TA	glutamate receptor, ionotropic kainate 2-like
Gglean011061-TA	uncharacterized protein LOC100904564
Gglean012537-TA	uncharacterized protein LOC100898662
Gglean007070-TA	uncharacterized protein LOC100898662
Gglean016224-TA	uncharacterized protein LOC100898662
Gglean016227-TA	uncharacterized protein LOC100898662
Gglean016228-TA	conserved hypothetical protein
Gglean014829-TA	uncharacterized protein LOC100898662
Gglean002327-TA	conserved hypothetical protein
Gglean014856-TA	hypothetical protein L798_03893
Gglean016833-TA	hypothetical protein L798_03054
Gglean010113	
Gglean001356-TA	glutamate receptor, ionotropic kainate 4-like
Gglean001357-TA	hypothetical protein M513_07976
Gglean001358-TA	hypothetical protein TRIADDRAFT_14565
Gglean001360-TA	hypothetical protein BRAFLDRAFT_85343
Gglean001361-TA	glutamate receptor ionotropic, NMDA 2B-like
Gglean001362-TA	hypothetical protein TRIADDRAFT_14565
Gglean000836-TA	uncharacterized protein LOC100906306
Gglean000837-TA	uncharacterized protein LOC100906306
Gglean000838-TA	uncharacterized protein LOC100900966
Gglean006461-TA	uncharacterized protein LOC100906306
Gglean016232-TA	conserved hypothetical protein
Gglean014622-TA	uncharacterized protein LOC100902495

Gglean004627-TA	hypothetical protein IscW_ISCW019301
Gglean000494-TA	glutamate receptor U1, putative
Gglean005333-TA	glutamate receptor U1, putative
Gglean012602-TA	conserved hypothetical protein
Gglean008444-TA	glutamate receptor, putative
Gglean000078-TA	glutamate receptor-like
Gglean010175-TA	glutamate receptor-like
Gglean007746-TA	glutamate receptor U1, putative
Gglean004216-TA	uncharacterized protein LOC100906917
Gglean010061-TA	uncharacterized protein LOC100906917
Gglean016551-TA	uncharacterized protein LOC100905899
Gglean016552-TA	uncharacterized protein LOC100905899
Gglean012267-TA	uncharacterized protein LOC100897865
Gglean011993-TA	uncharacterized protein LOC100909323
Gglean004250-TA	uncharacterized protein LOC100909323
Gglean015258-TA	uncharacterized protein LOC100905362
Gglean014827-TA	uncharacterized protein LOC100897721
Gglean008955-TA	uncharacterized protein LOC100897865
Gglean012116-TA	glutamate receptor delta-1 subunit-like
Gglean000289-TA	glutamate receptor delta-1 subunit-like
Gglean000369-TA	uncharacterized protein LOC100897865
Gglean011062-TA	uncharacterized protein LOC100904564
Gglean011063-TA	uncharacterized protein LOC100904564
Gglean017470-TA	glutamate receptor, ionotropic kainate 2-like
Gglean007719-TA	glutamate receptor, ionotropic kainate 3-like
Gglean007499-TA	glutamate receptor 3-like
Gglean013136-TA	glutamate receptor 1-like
Gglean015981-TA	glutamate receptor 1-like
Gglean001379-TA	glutamate receptor 2-like
Gglean001380-TA	glutamate receptor, ionotropic kainate 4
Gglean003336-TA	glutamate [NMDA] receptor subunit 1-like
Gglean012945-TA	glutamate [NMDA] receptor subunit epsilon-2-like
Gglean011065-TA	glutamate receptor, ionotropic kainate 3-like
Gglean011064-TA	glutamate receptor, ionotropic kainate 3-like
Gglean001653-TA	glutamate receptor, ionotropic kainate 2-like
Gglean007778-TA	glutamate receptor, ionotropic kainate 2-like
Gglean015795-TA	glutamate receptor, ionotropic kainate 2-like
Gglean001876-TA	glutamate receptor, ionotropic kainate 2-like
Gglean007618-TA	glutamate receptor 1-like
Gglean005061-TA	glutamate receptor 1-like
**Transient Receptor Potential (TRP) cation channels 5**	
Gglean000573-TA	transient receptor potential cation channel subfamily M member 1-like, Trpm
Gglean000574-TA	transient receptor potential cation channel subfamily M member 1-like, Trpm
Gglean008253-TA	transient receptor potential-gamma protein-like, Trpgamma
Gglean010595-TA	transient receptor potential cation channel subfamily A member 1-like, TrpA1
Gglean002276-TA	mucolipin-3-like, Trpml
**Niemann-Pick type C2 (NPC2) 2**	
Gglean000254-TA	Niemann-Pick type C-2a
Gglean009788-TA	protein NPC2 homolog
Gglean007619-TA	Niemann-Pick C1 protein-like
Gglean008738-TA	Niemann-Pick C1 protein-like
Gglean015570-TA	Niemann-Pick C1 protein-like
Gglean016650-TA	Niemann-Pick C1 protein-like

**oo18 RNA-binding protein 2 (ORB2)**	
Gglean015409-TA	oo18 RNA-binding protein 2-like
Gglean010824-TA	oo18 RNA-binding protein 2-like

Table S27 Identification of the cytochrome P450 (CYP) genes in the *N. cucumeris* genome.

Gene family	Gene members
CYP2	Gglean006838, Gglean006839, Gglean000021, Gglean012867, Gglean006560, Gglean006559, Gglean008184, Gglean014734, Gglean001311, Gglean005679, Gglean000153, Gglean017019, Gglean014676, Gglean013205, Gglean017147, Gglean000385, Gglean000394
CYP3	Gglean006156, Gglean014888, Gglean014885, Gglean011856, Gglean013412, Gglean013410, Gglean017197, Gglean007761, Gglean009361, Gglean016064, Gglean016065, Gglean012463, Gglean012464, Gglean001702, Gglean011084, Gglean005873, Gglean005871, Gglean004440, Gglean005872, Gglean004996, Gglean012093, Gglean010464, Gglean010465, Gglean012615, Gglean012616, Gglean012617, Gglean006077, Gglean007120, Gglean004599, Gglean012390, Gglean008366, Gglean11456, Gglean006163
CYP4	Gglean006048, Gglean009559, Gglean006049, Gglean005535, Gglean015743, Gglean007233, Gglean008774, Gglean015379, Gglean006430, Gglean004134, Gglean007497, Gglean000393;
Mitochondrial CYPM	Gglean002176, Gglean005674, Gglean001295, Gglean001296, Gglean001008, Gglean005870

Table S28 Identification of the glutathione-S-transferase (GST) genes in the *N. cucumeris* genome.

Gene family	Gene members
GST Delta	Gglean009873, Gglean009874, Gglean009875, Gglean014808, Gglean003304, Gglean000910
GST Omega	Gglean016716, Gglean016719, Gglean016646
GST Zeta	Gglean013971
GST Mu	Gglean007225, Gglean013462, Gglean011436, Gglean011437, Gglean011266

Table S29 Identification of the carboxyl/cholinesterase (CCE) genes in the *N. cucumeris* genome.

Gene family	Gene members
CCE J	Gglean017272, Gglean017401
CCE K	Gglean000027
CCE L	Gglean000279, Gglean000778, Gglean007067, Gglean000225
CCE J'	Gglean017272, Gglean007967, Gglean009583, Gglean005099, Gglean010771, Gglean007966, Gglean002608, Gglean007965, Gglean010452, Gglean007035, Gglean005418, Gglean001578, Gglean012842, Gglean009714, Gglean000395, Gglean014687, Gglean016156, Gglean004299
CCE J"	Gglean000798, Gglean009621, Gglean009032, Gglean006036, Gglean017402, Gglean017403, Gglean000565, Gglean000566, Gglean002909, Gglean017405, Gglean002910, Gglean017406, Gglean017404, Gglean007558, Gglean002908, Gglean002912, Gglean017408, Gglean017411, Gglean017413, Gglean017410, Gglean017409, Gglean002913, Gglean002911, Gglean017407, Gglean002915, Gglean017414, Gglean000031, Gglean000026, Gglean000580

Table S30 The predicted transportor-related genes in the *N. cucumeris* genome.

Gene family	Number	Genes
**ABC transporter (35)**		
GF_19	**28**	Gglean005786-PA, Gglean010803-PA, Gglean015865-PA, Gglean010801-PA, Gglean011654-PA, Gglean006640-PA, Gglean008676-PA, Gglean007731-PA, Gglean017172-PA, Gglean009595-PA, Gglean015450-PA, Gglean014433-PA, Gglean008025-PA, Gglean016082-PA, Gglean011652-PA, Gglean015688-PA, Gglean010620-PA, Gglean004877-PA, Gglean005138-PA, Gglean011390-PA, Gglean008677-PA, Gglean001520-PA, Gglean001521-PA, Gglean001522-PA, Gglean001700-PA, Gglean010619-PA, Gglean010497-PA, Gglean003978-PA
GF_11154	**3**	Gglean005654-PA, Gglean005655-PA, Gglean005657-PA
GF_12403	**2**	Gglean004132-PA, Gglean004961-PA
GF_14162	**2**	Gglean009656-PA, Gglean009779-PA
**Bacterial extracellular solute-binding proteins, family 3 (32)**		
GF_289	**22**	Gglean007893-PA, Gglean016714-PA, Gglean008063-PA, Gglean014856-PA, Gglean010113-PA, Gglean015748-PA, Gglean011018-PA, Gglean001355-PA, Gglean001360-PA, Gglean001358-PA, Gglean001362-PA, Gglean001361-PA, Gglean001356-PA, Gglean001357-PA, Gglean001359-PA, Gglean008540-PA, Gglean008541-PA, Gglean003181-PA, Gglean009026-PA, Gglean001129-PA, Gglean008542-PA, Gglean001354-PA
GF_7326	**6**	Gglean009560-PA, Gglean009561-PA, Gglean014123-PA, Gglean011394-PA, Gglean011380-PA, Gglean011381-PA
GF_6872	**2**	Gglean000494-PA, Gglean005333-PA
GF_10098	**2**	Gglean008286-PA, Gglean012262-PA
**Major Facilitator Superfamily (27)**		
GF_63	**11**	Gglean015120-PA, Gglean015121-PA, Gglean002624-PA, Gglean007519-PA, Gglean002625-PA, Gglean007521-PA, Gglean003393-PA, Gglean010683-PA, Gglean005497-PA, Gglean002623-PA, Gglean008045-PA
GF_39	**5**	Gglean010599-PA, Gglean011839-PA, Gglean011840-PA, Gglean011956-PA, Gglean011950-PA
GF_8837	**3**	Gglean008154-PA, Gglean008155-PA, Gglean013127-PA
GF_11729	**2**	Gglean011352-PA, Gglean011351-PA
GF_12463	**2**	Gglean009003-PA, Gglean009005-PA
GF_12501	**2**	Gglean012154-PA, Gglean012157-PA
GF_12502	**2**	Gglean012155-PA, Gglean012158-PA
**Amiloride-sensitive sodium channel (20)**		
GF_2883	**10**	Gglean008220-PA, Gglean013537-PA, Gglean013535-PA, Gglean016222-PA, Gglean013534-PA, Gglean016221-PA, Gglean013538-PA, Gglean013531-PA, Gglean016220-PA, Gglean013532-PA
GF_9787	**2**	Gglean015407-PA, Gglean015408-PA
GF_9817	**2**	Gglean001127-PA, Gglean007900-PA
GF_10164	**2**	Gglean016753-PA, Gglean016873-PA
GF_12457	**2**	Gglean007408-PA, Gglean007409-PA
GF_12521	**2**	Gglean013581-PA, Gglean013583-PA
**Binding-protein-dependent transport system inner membrane component (9)**		
GF_5859	**7**	Gglean002497-PA, Gglean010201-PA, Gglean010205-PA, Gglean013773-PA, Gglean010203-PA, Gglean010204-PA, Gglean010202-PA
GF_14165	**2**	Gglean010971-PA, Gglean010972-PA
**Ion transport protein (6)**		
GF_79	**6**	Gglean006179-PA, Gglean006274-PA, Gglean006266-PA, Gglean006269-PA, Gglean006270-PA, Gglean004320-PA
**Sodium:neurotransmitter symporter family (4)**		
GF_610	**4**	Gglean009733-PA, Gglean015083-PA, Gglean006949-PA, Gglean011646-PA
**HCO_3 - transporter family (3)**		
GF_452	**3**	Gglean000750-PA, Gglean000751-PA, Gglean003832-PA
**Ligand-gated ion channel (7)**		
GF_5875	**3**	Gglean000288-PA, Gglean000289-PA, Gglean000369-PA
GF_8846	**2**	Gglean008444-PA, Gglean012602-PA
GF_12385	**2**	Gglean002325-PA, Gglean002327-PA
**Sodium/calcium exchanger protein (4)**		
GF_8798	**2**	Gglean012451-PA, Gglean012454-PA
GF_10170	**2**	Gglean008506-PA, Gglean008507-PA
**Sugar (and other) transporter (4)**		

GF_8852	**2**	Gglean010439-PA, Gglean010521-PA
GF_14133	**2**	Gglean004677-PA, Gglean004678-PA
**CorA-like Mg^{2+} transporter protein (2)**		
GF_12558	**2**	Gglean013128-PA, Gglean013129-PA
**Sodium:alanine symporter family (2)**		
GF_14096	**2**	Gglean007071-PA, Gglean014810-PA
**Citrate transporter (2)**		
GF_14181	**2**	Gglean011824-PA, Gglean014557-PA
**Cation transport protein (2)**		
GF_14208	**2**	Gglean001130-PA, Gglean001131-PA
**Divalent cation transporter (2)**		
GF_14223	**2**	Gglean015706-PA, Gglean015738-PA
**Plasma membrane calcium transporter ATPase C terminal (2)**		
GF_14232	**2**	Gglean002054-PA, Gglean003000-PA

Table S31 The predicted stress response-related genes in the *N. cucumeris* genome.

Gene ID	Functional annotation
**Heat shock protein 26**	
Gglean000401-TA	97 kDa heat shock protein
Gglean001667-TA	Heat shock 70 kDa protein cognate 5
Gglean003130-TA	Heat shock protein 70
Gglean003131-TA	Heat shock protein 70
Gglean003203-TA	Heat shock protein HSP70-12A, putative
Gglean004137-TA	Heat shock protein Hsp20 domain containing protein
Gglean004377-TA	Heat shock protein 90
Gglean005732-TA	Heat shock 70 kDa protein 4-like
Gglean005792-TA	Putative heat shock protein
Gglean006224-TA	Heat shock protein 40
Gglean007169-TA	Heat shock protein 75
Gglean007474-TA	Heat shock protein 90
Gglean009449-TA	Heat shock protein 67B2
Gglean010031-TA	Heat shock protein Hsp20 domain containing protein
Gglean010574-TA	Heat shock protein 90
Gglean010904-TA	Heat shock protein 68-like
Gglean012511-TA	Heat shock HSP20 protein, putative
Gglean013401-TA	Heat shock protein 67B2
Gglean014423-TA	Putative heat shock protein
Gglean016288-TA	Heat shock protein beta-1-like
Gglean016290-TA	Heat shock protein beta-1-like
Gglean017183-TA	Heat shock protein beta-1
Gglean017184-TA	Heat shock protein beta-1
Gglean017186-TA	Heat shock protein beta-1
Gglean017288-TA	Heat shock 70 kDa protein cognate 4-like
Gglean017400-TA	Heat shock protein 60
**Antioxidant enzymes-Catalase (1)**	
Gglean016973-TA	Catalase-like
**Antioxidant enzymes-Peroxidase (26)**	
Gglean000669-TA	Glutathione peroxidase-like
Gglean000856-TA	Chorion peroxidase-like
Gglean003414-TA	Peroxidasin (heme-containing peroxidase)
Gglean003655-TA	Peroxidasin
Gglean003656-TA	Peroxidasin
Gglean003792-TA	Glutathione peroxidase-like
Gglean006065-TA	Peroxidasin-like
Gglean007041-TA	Peroxidasin-like
Gglean007111-TA	Peroxidasin
Gglean008137-TA	Peroxidasin
Gglean008526-TA	Chorion peroxidase-like
Gglean008527-TA	Chorion peroxidase-like
Gglean009713-TA	Probable phospholipid hydroperoxide glutathione peroxidase 6, mitochondrial-like
Gglean009723-TA	Peroxidasin-like
Gglean010675-TA	Peroxidasin-like
Gglean010676-TA	Peroxidasin-like
Gglean012169-TA	Thioredoxin-dependent peroxide reductase, mitochondrial-like isoform 1 (thioredoxin peroxidase)
Gglean012246-TA	Glutathione peroxidase-like
Gglean012518-TA	Chorion peroxidase-like

Gglean012519-TA	Peroxidasin
Gglean013170-TA	Peroxiredoxin-6-like
Gglean014254-TA	Peroxidase
Gglean014999-TA	Peroxiredoxin-6-like
Gglean015231-TA	Glutathione peroxidase-like
Gglean016749-TA	Peroxidase-like
Gglean016983-TA	Peroxidase-like
**Antioxidant enzymes-Superoxide dismutase (4)**	
Gglean000767-TA	Extracellular superoxide dismutase [Cu-Zn]-like
Gglean001565-TA	Superoxide dismutase [Mn] 1, mitochondrial-like isoform 1
Gglean011117-TA	Extracellular superoxide dismutase [Cu-Zn]-like
Gglean014403-TA	Superoxide dismutase [Cu-Zn]-like

Table S32 The predicted DNA repair-related genes in the *N. cucumeris* genome.

Gene ID	Functional annotation
Gglean001612-TA	endonuclease III-like protein 1-like
Gglean002429-TA	G/T mismatch-specific thymine DNA glycosylase-like
Gglean012646-TA	single-strand selective monofunctional uracil DNA glycosylase-like
Gglean002485-TA	mismatch repair endonuclease PMS2
Gglean002486-TA	mismatch repair endonuclease PMS2
Gglean003037-TA	DNA mismatch repair protein Mlh1-like
Gglean003038-TA	DNA mismatch repair protein Mlh1-like
Gglean003191-TA	DNA mismatch repair protein Msh6-like
Gglean003192-TA	DNA mismatch repair protein Msh6-like
Gglean004214-TA	DNA mismatch repair protein Msh6-like
Gglean016202-TA	DNA mismatch repair protein Msh2-like
Gglean009727-TA	DNA repair protein XRCC1-like
Gglean013876-TA	DNA repair and recombination protein RAD54-like
Gglean016457-TA	DNA repair and recombination protein RadA-like
Gglean000983-TA	DNA ligase 3
Gglean007728-TA	DNA ligase 1-like
Gglean012704-TA	DNA ligase 1-like
Gglean015123-TA	DNA ligase 4-like
Gglean006052-TA	proliferating cell nuclear antigen-like, PCNA
Gglean002256-TA	DNA polymerase eta-like
Gglean003558-TA	DNA polymerase delta catalytic subunit-like
Gglean003619-TA	DNA polymerase theta-like
Gglean003620-TA	DNA polymerase theta-like
Gglean003621-TA	DNA polymerase theta-like
Gglean006323-TA	DNA polymerase zeta catalytic subunit-like
Gglean006374-TA	DNA polymerase alpha subunit B-like
Gglean012894-TA	DNA polymerase epsilon catalytic subunit A-like
Gglean012895-TA	DNA polymerase epsilon catalytic subunit A-like
Gglean013097-TA	DNA polymerase epsilon subunit 2-like
Gglean016790-TA	DNA polymerase epsilon subunit 4-like
Gglean013719-TA	DNA polymerase kappa-like
Gglean012234-TA	flap endonuclease 1-like
Gglean002599-TA	cullin-4A
Gglean012958-TA	cullin-associated NEDD8-dissociated protein 1-like (regulator of cullins 1)
Gglean008030-TA	protein piccolo-like
Gglean012028-TA	piccolo, putative
Gglean015254-TA	piccolo, putative
Gglean010762-TA	UV excision repair protein RAD23 homolog B-like
Gglean004341-TA	cyclin-dependent kinase 7-like, CDK7
Gglean011954-TA	CDK-activating kinase assembly factor MAT1-like
Gglean007771-TA	cyclin-H-like
Gglean008743-TA	DNA excision repair protein haywire-like
Gglean014415-TA	TFIIH basal transcription factor complex helicase XPD subunit-like
Gglean001970-TA	general transcription factor IIH subunit 5-like, TFIIH5
Gglean001994-TA	general transcription factor IIH subunit 1-like, TFIIH2
Gglean000137-TA	general transcription factor IIH subunit 4-like, TFIIH4
Gglean008109-TA	general transcription factor IIH subunit 2-like, TFIIH2
Gglean010590-TA	general transcription factor IIH subunit 3-like, TFIIH3
Gglean017418-TA	general transcription factor IIH subunit 3-like, TFIIH3
Gglean004109-TA	DNA excision repair protein ERCC-6-like
Gglean010667-TA	DNA excision repair protein ERCC-1-like

Gglean012222-TA	DNA excision repair protein ERCC-8-like
Gglean017482-TA	DNA excision repair protein ERCC-6-like
Gglean017483-TA	DNA excision repair protein ERCC-6-like
Gglean002480-TA	replication protein A 70 kDa DNA-binding subunit, RpA-70
Gglean003022-TA	Replication protein A DNA-binding subunit
Gglean003159-TA	replication protein A 70 kDa DNA-binding subunit-like
Gglean003368-TA	replication protein A 70 kDa DNA-binding subunit
Gglean003538-TA	replication protein A 70 kDa DNA-binding subunit-like
Gglean004047-TA	replication protein A 70 kDa DNA-binding subunit
Gglean004110-TA	replication protein A 32 kDa subunit-like
Gglean004472-TA	replication protein A 70 kDa DNA-binding subunit
Gglean004565-TA	replication protein A 70 kDa DNA-binding subunit-like
Gglean004969-TA	replication protein A 70 kDa DNA-binding subunit-like
Gglean005211-TA	replication protein A 70 kDa DNA-binding subunit-like
Gglean005234-TA	replication protein A 70 kDa DNA-binding subunit-like
Gglean005259-TA	replication protein A 70 kDa DNA-binding subunit-like
Gglean005378-TA	replication protein A 70 kDa DNA-binding subunit-like
Gglean005413-TA	replication protein A 70 kDa DNA-binding subunit-like
Gglean005456-TA	replication protein A 70 kDa DNA-binding subunit-like
Gglean005696-TA	replication protein A 70 kDa DNA-binding subunit-like
Gglean007858-TA	replication protein A 70 kDa DNA-binding subunit-like
Gglean008467-TA	replication protein A 70 kDa DNA-binding subunit-like
Gglean009322-TA	replication protein A 70 kDa DNA-binding subunit-like
Gglean010247-TA	replication protein A 70 kDa DNA-binding subunit-like
Gglean011892-TA	replication protein A 70 kDa DNA-binding subunit-like
Gglean011926-TA	replication protein A 70 kDa DNA-binding subunit-like
Gglean012291-TA	replication protein A 70 kDa DNA-binding subunit-like
Gglean014050-TA	replication protein A 70 kDa DNA-binding subunit-like
Gglean014085-TA	replication protein A 70 kDa DNA-binding subunit-like
Gglean015523-TA	replication protein A 70 kDa DNA-binding subunit-like
Gglean015524-TA	replication protein A 70 kDa DNA-binding subunit-like
Gglean015588-TA	replication protein A 70 kDa DNA-binding subunit-like
Gglean016572-TA	replication protein A 70 kDa DNA-binding subunit-like
Gglean016620-TA	replication protein A 70 kDa DNA-binding subunit-like
Gglean016628-TA	replication protein A 70 kDa DNA-binding subunit-like
Gglean016629-TA	replication protein A 70 kDa DNA-binding subunit-like
Gglean016770-TA	replication protein A 70 kDa DNA-binding subunit-like
Gglean001610-TA	replication protein A 70 kDa DNA-binding subunit-like
Gglean005931-TA	replication factor C subunit 1, RFC
Gglean006337-TA	replication factor C subunit 2-like
Gglean007199-TA	replication factor C subunit 4-like
Gglean007434-TA	replication factor C subunit 3-like
Gglean011245-TA	replication factor C subunit 5-like
Gglean015871-TA	single-stranded DNA-binding protein 3-like
Gglean004456-TA	exonuclease 1-like, ExoI
Gglean000016-TA	DNA repair protein RAD51 homolog B-like
Gglean005111-TA	DNA repair protein RAD50-like
Gglean005121-TA	RAD50-interacting protein 1-like
Gglean006319-TA	double-strand-break repair protein RAD21 homolog
Gglean008868-TA	meiotic recombination protein DMC1/LIM15 homolog
Gglean011304-TA	meiotic recombination protein SPO11-like
Gglean008252-TA	DNA topoisomerase 3-alpha
Gglean008842-TA	DNA topoisomerase 3-beta-1-like

Gglean001030-TA	DNA topoisomerase 1
Gglean014833-TA	DNA topoisomerase 1-like
Gglean014634-TA	DNA topoisomerase 2-alpha-like
Gglean001351-TA	DNA topoisomerase 2-beta

Table S33 The predicted immunity-related genes in the *N. cucumeris* genome.

Gene ID	Gene name	Functional annotation
**Immune receptor and adhesin**		
Gglean013252-TA	PGRP-LA	Peptidoglycan recognition protein LA
Gglean000452-TA	Dscam	Down syndrome cell adhesion molecule-like protein Dscam2
Gglean001796-TA	Dscam	Down syndrome cell adhesion molecule-like protein Dscam2
Gglean003187-TA	Dscam	Down syndrome cell adhesion molecule-like protein Dscam2
Gglean003524-TA	Dscam	Down syndrome cell adhesion molecule-like protein Dscam2
Gglean004926-TA	Dscam	Down syndrome cell adhesion molecule-like protein Dscam2
Gglean006329-TA	Dscam	Down syndrome cell adhesion molecule-like protein Dscam2
Gglean006580-TA	Dscam	Down syndrome cell adhesion molecule-like protein 1
Gglean006597-TA	Dscam	Down syndrome cell adhesion molecule-like protein 1-like
Gglean006757-TA	Dscam	Down syndrome cell adhesion molecule-like protein Dscam2
Gglean008099-TA	Dscam	Down syndrome cell adhesion molecule-like protein Dscam2
Gglean010096-TA	Dscam	Down syndrome cell adhesion molecule-like protein Dscam2
Gglean012421-TA	Dscam	Down syndrome cell adhesion molecule-like protein Dscam2
Gglean014313-TA	Dscam	Down syndrome cell adhesion molecule-like protein Dscam2
Gglean014434-TA	Dscam	Down syndrome cell adhesion molecule-like protein Dscam2
Gglean014436-TA	Dscam	Down syndrome cell adhesion molecule-like protein Dscam2
Gglean014439-TA	Dscam	Down syndrome cell adhesion molecule-like protein Dscam2
Gglean014440-TA	Dscam	Down syndrome cell adhesion molecule-like protein Dscam2
Gglean016367-TA	Dscam	Down syndrome cell adhesion molecule-like protein Dscam2
Gglean016471-TA	Dscam	Down syndrome cell adhesion molecule-like protein Dscam2
Gglean006741-TA	FRP	bacterial fibrinogen-binding adhesin
Gglean013274-TA	Nimrod-like	Putative draper
Gglean015872-TA	TEP	thioester-containing protein
**Toll singaling pathway**		
Gglean000513-TA	TLR	Putative toll
Gglean003045-TA	TLR	Protein toll (Precursor)
Gglean008685-TA	TLR	protein toll-like
Gglean009074-TA	TLR	Protein toll (Precursor)
Gglean009663-TA	TLR	Protein toll (Precursor)
Gglean010117-TA	TLR	Protein toll (Precursor)
Gglean010215-TA	TLR	protein toll-like
Gglean017120-TA	TLR	Protein toll (Precursor)
Gglean007460-TA	Spätzle	Putative spätzle 3
Gglean008338-TA	Spätzle	Protein spaetzle C-106 (Precursor)
Gglean004879-TA	pellino	protein pellino-like, Toll signaling pathway, kinase regulator activity
Gglean006179-TA		voltage-dependent calcium channel type A subunit alpha-1, innate immune response
Gglean006274-TA		voltage-dependent calcium channel type A subunit alpha-1, innate immune response
Gglean008846-TA	GPRK	G protein-coupled receptor kinase 5-lik, regulation of Toll signaling pathway
Gglean014720-TA		evolutionarily conserved signaling intermediate in Toll pathway, mitochondrial-like
Gglean014792-TA		SUMO-conjugating enzyme UBC9-like, negative regulation of Toll signaling pathway
Gglean008794-TA	MyD88	myeloid differentiation primary response protein MyD88-like
Gglean002970-TA	pelle	Serine/threonine-protein kinase pelle
Gglean005940-TA	pelle	Serine/threonine-protein kinase pelle
Gglean003339-TA	cactus	NF-kappa-B inhibitor cactus
Gglean012498-TA	dorsal	embryonic polarity protein dorsal-like

Gglean003657-TA	persephone	serine proteinase stubble-like
Gglean004584-TA	persephone	Serine proteinase stubble catalytic chain
Gglean016504-TA	persephone	Serine proteinase stubble catalytic chain
Gglean007989-TA	persephone	Serine proteinase stubble catalytic chain
Gglean000012-TA	persephone	serine proteinase stubble-like
Gglean000237-TA	spheroide	Tick serine proteinase
Gglean009378-TA	spirit	coagulation factor precursor, Serine Protease Immune Response Integrator
**IMD singaling pathway**		
Gglean013561-TA	Ankyrin	Ankyrin repeat
Gglean000881-TA	Diap2	Death-associated inhibitor of apoptosis 2
Gglean003229-TA	effete	ubiquitin-conjugating enzyme E2 variant 2-like
Gglean010670-TA	effete	ubiquitin-conjugating enzyme E2 variant 2-like
Gglean017289-TA	effete	ubiquitin-conjugating enzyme E2-17 kDa-like
Gglean008479-TA	IKKβ	Inhibitor of nuclear factor kappa-B kinase subunit beta
Gglean012054-TA	kayak	Putative transcription factor kayak
Gglean002899-TA	Mkk7	mitogen-activated protein kinase kinase kinase 7-like
Gglean017190-TA	Tak1	TGF-beta activated kinase 1
Gglean012787-TA	Tab2	neurogenic locus Notch protein-like, TAK1-associated binding protein 2
Gglean007142-TA	Uev1A	ubiquitin-conjugating enzyme E2 variant 2-like
**JNK singaling pathway**		
Gglean010895-TA	bendless	ubiquitin-conjugating enzyme E2 N-like
Gglean003173-TA	Basket	stress-activated protein kinase JNK-like
Gglean003551-TA	Basket	stress-activated protein kinase JNK-like
Gglean013014-TA	hemipterous	dual specificity mitogen-activated protein kinase kinase hemipterous-like
Gglean004867-TA		JNK-interacting protein 1-like
Gglean012604-TA		JNK-interacting protein 3-like
Gglean000707-TA		protein roadkill-like, positive regulation of JNK cascade
Gglean001432-TA		striatin-3-like, positive regulation of JNK cascade
Gglean004952-TA		Protein roadkill, positive regulation of JNK cascade
Gglean005992-TA	MINK1	misshapen-like kinase 1-like, JNK cascade
Gglean007051-TA		JNK1/MAPK8-associated membrane protein
Gglean011219-TA		JNK_SAPK-associated protein-1, RILP-like protein homolog
Gglean013805-TA		RNA-binding protein Nova-1-like, negative regulation of JNK cascade
Gglean013898-TA		cdc42 homolog, JNK cascade
Gglean017138-TA		Protein roadkill, positive regulation of JNK cascade
**JAK/STAT singaling pathway**		
Gglean003643-TA	Domeless	cytokine receptor-like
Gglean001915-TA	JAK	tyrosine-protein kinase JAK2-like
Gglean001690-TA	Stat	Signal transducer and transcription activator
Gglean003122-TA	Stat92E	Signal transducer and transcription activator
Gglean003525-TA	Stat92E	Signal transducer and transcription activator
Gglean013404-TA	Stat92E	Signal transducer and transcription activator
Gglean015272-TA	Stat92E	Signal transducer and transcription activator 5A
Gglean015301-TA	Stat92E	Signal transducer and transcription activator 5B
Gglean017318-TA	Stat92E	Signal transducer and transcription activator
Gglean017323-TA	Stat92E	Signal transducer and transcription activator
**Lysozymes**		
Gglean001248-TA	Lys	Putative alpha-lactalbumin / lysozyme c
Gglean001734-TA	Lys	lysozyme C-like

Gglean001990-TA	Lys	lysozyme C3-like
Gglean004205-TA	Lys	lysozyme C-like
Gglean004381-TA	Lys	Lysozyme c-1
Gglean005404-TA	Lys	Probable GH family 25 lysozyme 3
Gglean006356-TA	Lys	Lysozyme c-1
Gglean007847-TA	Lys	lysozyme-like
Gglean009192-TA	Lys	Lysozyme D, LysD
Gglean014066-TA	Lys	lysozyme 2-like
Gglean016783-TA	Lys	Putative lysozyme-like protein

Table S34 The predicted RNAi-related *Dicer* and *Argonaute* genes in the *N. cucumeris* genome.

Gene ID	Gene name	Functional annotation
**Dicer**		
Gglean005664-TA	Dicer-1	endoribonuclease Dicer-like
Gglean007004-TA	Dicer-1	endoribonuclease Dicer-like
Gglean013004-TA	Dicer-1	endoribonuclease Dicer-like
Gglean014732-TA	Dicer-1	endoribonuclease Dicer-like
Gglean005508-TA	Dicer-2	endoribonuclease Dicer-like
Gglean005665-TA	Dicer-2	endoribonuclease Dicer-like
**Argonaute**		
Gglean004717-TA	Argonaute-1	protein argonaute PNH1-like
Gglean008295-TA	Argonaute-1	protein argonaute PNH1-like
Gglean010150-TA	Argonaute-1	protein argonaute PNH1-like
Gglean011961-TA	Argonaute-1	protein argonaute PNH1-like
Gglean004718-TA	Argonaute-2	protein argonaute-2-like
Gglean008296-TA	Argonaute-2	protein argonaute-2-like
Gglean008997-TA	Argonaute-2	protein argonaute-2-like
Gglean010151-TA	Argonaute-2	protein argonaute-2-like
Gglean011962-TA	Argonaute-2	protein argonaute-2-like
Gglean016138-TA	Argonaute-2	protein argonaute-2-like
Gglean009648-TA	Argonaute-3	protein argonaute-3-like
Gglean014107-TA	Argonaute-3	protein argonaute-3-like
Gglean016132-TA	Argonaute-3	protein argonaute-3-like
Gglean016134-TA	Argonaute-3	protein argonaute-3-like
Gglean016135-TA	Argonaute-3	protein argonaute-3-like

Table S13 (852 pages)

GeneID	**12h**	**12h**	**36h**	**36h**
Gglean000001	80.24082	101.93806	61.60668	58.79188
Gglean000002	29.530501	39.4609	30.460748	29.72722
Gglean000003	29.530501	39.4609	30.460748	29.72722
Gglean000004	119.286912	119.387056	127.820792	128.297751
Gglean000005	119.286912	119.387056	127.820792	128.297751
Gglean000006	3.518322676	3.708960444	5.274594	6.30600579
Gglean000007	3.518322676	3.708960444	5.274594	6.30600579
Gglean000008	10.39222955	10.98671381	21.31337901	22.05785097
Gglean000009	10.39222955	10.98671381	21.31337901	22.05785097
Gglean000010	11.03876	8.5279	20.26442	21.2278
Gglean000011	41.26189	39.36779062	32.18513	30.32884
Gglean000012	19.39438	17.50124	22.953707	28.8304
Gglean000013	0	0	0	0
Gglean000014	0	1.31665	0	0
Gglean000015	32.02341653	20.516994	36.874741	38.204513
Gglean000016	11.90207694	13.39050034	4.762911	4.473924802
Gglean000017	5.534875363	5.7064539	6.918852408	6.29656536
Gglean000018	18.832704	18.69508	10.808934	9.660381
Gglean000019	30.9974	36.4938	27.181	27.2934
Gglean000020	30.821597	27.71495	20.645124	19.287154
Gglean000021	25.12479	26.96974	19.63478	18.46469
Gglean000022	18.1505087	16.216102	21.44395	28.6893065
Gglean000023	18.1505087	16.216102	21.44395	28.6893065
Gglean000024	0.0911824	0.0951762	0.225632	0
Gglean000025	2.83592	3.24055	3.538363	8.325455
Gglean000026	24.53490219	29.30603269	47.80711787	44.14031341
Gglean000027	24.53490219	29.30603269	47.80711787	44.14031341
Gglean000028	4.65128124	2.696898421	3.519365001	4.68279014
Gglean000029	2.158667	2.316836	1.998242	2.01062
Gglean000030	8.06766	8.669553	5.701262	6.85584
Gglean000031	8.06766	8.669553	5.701262	6.85584
Gglean000032	0	0	0	0
Gglean000033	1.94864	1.89315	4.68483	4.77455
Gglean000034	63.37827	69.45225	57.274096	69.370212
Gglean000035	0.4890177	0.385848	0.370155	0.489568
Gglean000036	0	0	0	0
Gglean000037	0.632792266	1.292504	1.275518	0.30509213
Gglean000038	0.230471	0	0.383645	0.911007
Gglean000039	3.27772	4.83866	1.41543	2.10036
Gglean000040	2.86211	2.71264	1.69531	1.20635
Gglean000041	1.46601	1.37708	1.19261	0.941285
Gglean000042	29.941482	33.619899	27.719968	28.343362
Gglean000043	29.941482	33.619899	27.719968	28.343362

Gglean000044	29.941482	33.619899	27.719968	28.343362
Gglean000045	0	0	0	0
Gglean000046	0	0	0	0.980106
Gglean000047	0.17048	0	0	0
Gglean000048	38.31574309	42.56131147	37.7860285	37.79766729
Gglean000049	103.1304499	110.908068	78.30971	74.5793
Gglean000050	103.1304499	110.908068	78.30971	74.5793
Gglean000051	57.70898296	67.86949979	65.19721514	57.13442604
Gglean000052	47.6046627	47.17253864	35.565341	37.057728
Gglean000053	53.07904	55.5408	59.731798	67.556282
Gglean000054	29.640113	31.8449646	30.222517	33.619913
Gglean000055	0	0	0	0
Gglean000056	0	3.47E-73	3.37352	1.68E-240
Gglean000057	0	0.012058531	0.016445718	0.100400039
Gglean000058	0	0.012058531	0.016445718	0.100400039
Gglean000059	13.61601	14.92324254	20.24887005	22.79286
Gglean000060	0.845727	1.030277	1.13933596	1.105744654
Gglean000061	53.271509	49.849188	46.35785	46.38745
Gglean000062	14.8697	7.67633	10.9552	19.1542
Gglean000063	0	0	0.0113669	0.017205836
Gglean000064	22.46072	20.50804	24.96256	24.77002
Gglean000065	1554.962	1581.734	1065.431	981.5
Gglean000066	0	0	0	0
Gglean000067	29.9484	38.2238	15.7526	13.2295
Gglean000068	0	0	0	0
Gglean000069	37.95842298	41.07098	29.53635825	26.804841
Gglean000070	4.288995	3.933715025	4.819022251	5.156565595
Gglean000071	34.60919	34.40623	22.73158317	25.433997
Gglean000072	887.734474	833.952574	674.924345	753.082612
Gglean000073	12.42768601	12.99197002	8.303978	7.100106
Gglean000074	0.679984	1.40407	1.33785	0.818042
Gglean000075	3.799327	3.453119	5.006490685	5.891188661
Gglean000076	3.799327	3.453119	5.006490685	5.891188661
Gglean000077	9.536163092	11.32408292	11.14905402	16.413823
Gglean000078	9.536163092	11.32408292	11.14905402	16.413823
Gglean000079	9.536163092	11.32408292	11.14905402	16.413823
Gglean000080	0.4045144	1.470241562	1.236660254	0.1902482
Gglean000081	0.4045144	1.470241562	1.236660254	0.1902482
Gglean000082	0	0	0	0
Gglean000083	10.9263	8.70514	10.9562	12.0491
Gglean000084	98.3384047	107.4540045	55.91840288	36.50810218
Gglean000085	33.03547	34.52468	28.14061	25.40565
Gglean000086	33.03547	34.52468	28.14061	25.40565
Gglean000087	0	0	0	0

Gglean000088	0.210195	0	0.258383	0
Gglean000089	41.2002	42.9731	20.6693	17.4032
Gglean000090	11.40105	11.90061	16.48286	17.48963
Gglean000091	226.9411437	249.165126	272.6427915	276.5339984
Gglean000092	9.36742	9.62345	5.19383	6.00187
Gglean000093	36.9033	27.9321	27.7631	24.7361
Gglean000094	45.699	47.9282	46.9939	56.1842
Gglean000095	149.918	102.79	136.881	159.862
Gglean000096	509.216	405.841	509.09	783.125
Gglean000097	0.214984	0.0557513	0	0
Gglean000098	52.978153	54.0714514	85.084894	99.2785892
Gglean000099	52.978153	54.0714514	85.084894	99.2785892
Gglean000100	0.718484	1.90887	1.87888	2.73865
Gglean000101	52.978153	54.0714514	85.084894	99.2785892
Gglean000102	52.978153	54.0714514	85.084894	99.2785892
Gglean000103	0.0668105	0.0745442	0.00594905	0.0210376
Gglean000104	0.154787077	0.218653908	0.038026644	0.01120692
Gglean000105	0.154787077	0.218653908	0.038026644	0.01120692
Gglean000106	64.8596333	70.37273001	69.4655706	74.578833
Gglean000107	64.8596333	70.37273001	69.4655706	74.578833
Gglean000108	18.58311173	21.91835888	28.86559239	27.97243484
Gglean000109	18.58311173	21.91835888	28.86559239	27.97243484
Gglean000110	18.58311173	21.91835888	28.86559239	27.97243484
Gglean000111	0	0	0.0718843	0
Gglean000112	0	0.0424835	0.100319	0.0395989
Gglean000113	0.0357054	0.0743054	0	0
Gglean000114	23.5869	27.2353	11.7087	10.612
Gglean000115	6.571653428	7.445632	6.2593233	5.343469
Gglean000116	28.1682353	31.5443143	57.3098154	62.8922157
Gglean000117	28.1682353	31.5443143	57.3098154	62.8922157
Gglean000118	11.8105497	12.8008233	20.52639178	22.92970714
Gglean000119	0.670398583	0.880327	0.975862	1.361609
Gglean000120	21.4089	21.830586	20.170157	17.6667
Gglean000121	53.5153773	52.7086	36.86722446	34.144359
Gglean000122	53.5153773	52.7086	36.86722446	34.144359
Gglean000123	152.5020663	147.6065559	233.8967131	282.83103
Gglean000124	152.5020663	147.6065559	233.8967131	282.83103
Gglean000125	0	0	0.182322	0
Gglean000126	39.54706	44.09476	27.60377	23.21791
Gglean000127	7.438668992	9.04396385	7.384799616	8.21824941
Gglean000128	7.438668992	9.04396385	7.384799616	8.21824941
Gglean000129	7.438668992	9.04396385	7.384799616	8.21824941
Gglean000130	1.42659	2.69755	2.119865262	2.355418894
Gglean000131	1.26605	0.23211509	0.759918	0.962335

Gglean000132	6.51066145	7.769305853	9.795887963	10.71843908
Gglean000133	127.379	128.112	93.9611	88.074
Gglean000134	165.8693234	174.353456	91.715599	83.054044
Gglean000135	108.088	122.492	21.9463	17.4756
Gglean000136	22.055	23.8079	21.05376	18.72651
Gglean000137	19.22838	20.04384	9.29802	8.35539
Gglean000138	8.64796	12.73125	11.48458	13.45337
Gglean000139	8.64796	12.73125	11.48458	13.45337
Gglean000140	33.14108917	32.3992	32.35204	38.13049
Gglean000141	49.4342302	55.67198478	82.09258444	81.91948196
Gglean000142	2.325822	1.931833	0.651333	0.678294884
Gglean000143	20.712883	18.12735007	10.35356278	12.89233901
Gglean000144	0	0	0	0
Gglean000145	0	0	0	0
Gglean000146	7.81882075	9.8467445	8.216982125	9.8213917
Gglean000147	7.81882075	9.8467445	8.216982125	9.8213917
Gglean000148	2.104155404	2.39695785	2.507440445	2.939031671
Gglean000149	2.104155404	2.39695785	2.507440445	2.939031671
Gglean000150	46.94056582	50.59184742	24.41400459	22.8101143
Gglean000151	0.268162667	0.111508963	0.168136824	0.213924101
Gglean000152	0	0	0	0
Gglean000153	5.526932	6.281740907	5.146540147	6.225167323
Gglean000154	1.496270546	2.613952	3.169412066	3.142842973
Gglean000155	1.1255752	1.443286787	1.27860966	2.087164438
Gglean000156	26.06622	21.72023	20.34014	18.71323
Gglean000157	0	0	0	0
Gglean000158	0.336347	0.0877756	0	0
Gglean000159	0	0	0	0
Gglean000160	0.466753335	0	0.380118869	0.664785
Gglean000161	0.466753335	0	0.380118869	0.664785
Gglean000162	11.19923097	12.47622001	7.14694023	5.8241225
Gglean000163	12.6303	8.54452	4.20281	6.09764
Gglean000164	3.026299732	3.315852128	6.45089124	5.685139659
Gglean000165	3.026299732	3.315852128	6.45089124	5.685139659
Gglean000166	0.4387589	0	0.089501352	0.0531766
Gglean000167	42.9071	43.7041	33.1545	32.7374
Gglean000168	0	0	0	0
Gglean000169	2.08925	1.90143	2.93562	2.93444
Gglean000170	20.54324413	19.68865359	15.46638217	13.93647991
Gglean000171	4.1813223	3.902731146	6.524365859	6.057214426
Gglean000172	4.1813223	3.902731146	6.524365859	6.057214426
Gglean000173	3.76299	2.90727	2.17229	2.52735
Gglean000174	50.83213	53.01377	36.12117	30.575703
Gglean000175	0	0	0	0

Gglean000176	3.23111	4.01247	2.58812	1.64182
Gglean000177	0	0	0.53672	0
Gglean000178	0.0687943	0.133459	2.805	0.0596157
Gglean000179	15.6303	28.3693	22.03959	22.34257
Gglean000180	6.757471572	6.612265152	4.006604399	3.916178281
Gglean000181	0.495857759	0.642098941	0.625301134	1.171773107
Gglean000182	1416.325	1457.389	977.467	925.807
Gglean000183	3.80099	4.83717	2.31851	1.89124
Gglean000184	7.832094	9.799046	9.32291	11.41986
Gglean000185	29.96710134	30.37160002	33.27910023	33.11267843
Gglean000186	36.14674355	36.20107	44.20636981	47.94987301
Gglean000187	6.49118	6.23562	5.648092973	8.52043
Gglean000188	0	0	2.21424	0.884777
Gglean000189	3.19182	3.45825	2.0726	4.58644
Gglean000190	16.73378	17.19167	21.91874	30.88253
Gglean000191	62.815689	67.42768968	59.25102149	60.551275
Gglean000192	62.815689	67.42768968	59.25102149	60.551275
Gglean000193	6.0866	6.43327	5.284414	4.18619088
Gglean000194	6.0866	6.43327	5.284414	4.18619088
Gglean000195	31.41900155	29.05940648	34.957186	39.97546938
Gglean000196	19.20008015	17.44402643	29.44881485	31.93082569
Gglean000197	10.866357	9.5128225	16.333223	17.493174
Gglean000198	67.947653	74.20923	85.53949	71.44075
Gglean000199	24.4508	24.5545	40.0725	55.3898
Gglean000200	16.1330165	17.02504813	14.16696561	14.56897776
Gglean000201	33.38147	38.12449	29.37488	29.88503
Gglean000202	29.68023	30.98917	19.966	18.88156
Gglean000203	24.95667576	29.50044541	28.45458898	27.877133
Gglean000204	27.69324804	25.56872954	29.49732	31.99244634
Gglean000205	8.156076	8.048641	7.224484176	4.713366239
Gglean000206	8.156076	8.048641	7.224484176	4.713366239
Gglean000207	8.156076	8.048641	7.224484176	4.713366239
Gglean000208	44.3814	44.12946	43.68190816	41.966321
Gglean000209	87.2932	74.1996	69.0632	67.6208
Gglean000210	7.117359138	6.28203776	8.162981631	8.391456245
Gglean000211	7.117359138	6.28203776	8.162981631	8.391456245
Gglean000212	45.71989833	45.32700558	38.43246468	33.70114576
Gglean000213	0	0	0	0
Gglean000214	0	0	0	0
Gglean000215	0	0.150931	0	0
Gglean000216	0	0	0	0
Gglean000217	1.334677515	1.2245365	2.107954398	2.272892
Gglean000218	9.61008	9.90566	12.46613	18.90016
Gglean000219	9.61008	9.90566	12.46613	18.90016

Gglean000220	35.8133	37.0537	37.8809	37.9142
Gglean000221	32.09788597	32.648884	26.47406615	24.63891243
Gglean000222	17.02381	17.108732	8.801687346	9.758517
Gglean000223	32.8524	39.7844	10.19742	8.62404
Gglean000224	12.3727	10.9509	19.9481	15.2709
Gglean000225	29.61344738	28.48498112	47.12016167	53.3595968
Gglean000226	0	0	0	0
Gglean000227	0	0	0	0
Gglean000228	5.41973794	5.09652632	6.61186671	9.0026669
Gglean000229	2.75E-15	0.0677004	5.10E-06	3.06E-05
Gglean000230	35.964556	37.035096	65.63827107	70.65116418
Gglean000231	35.964556	37.035096	65.63827107	70.65116418
Gglean000232	32.59143342	29.369002	38.38822886	66.255723
Gglean000233	35.17975	35.36152	15.34069	13.46054
Gglean000234	74.69137936	79.95294854	87.82353554	103.251614
Gglean000235	335.6937506	347.4289	220.5703	248.139168
Gglean000236	0	0.971009	0	0
Gglean000237	2.97957	5.19297	6.43683	7.35317
Gglean000238	1.53E-28	0.00936998	1.32E-29	1.52E-19
Gglean000239	22.6891	37.4108	80.1703	73.4601
Gglean000240	9.2824	8.45042	7.392819	5.85594161
Gglean000241	13.93358827	12.8804	25.11300075	26.75301445
Gglean000242	29.815	33.8218	16.1066	27.1597
Gglean000243	37.42681	68.1782	62.68171	87.29066
Gglean000244	37.42681	68.1782	62.68171	87.29066
Gglean000245	23.4407891	23.9581	36.2805949	38.5437
Gglean000246	20.24451918	21.3716766	16.49900061	15.822018
Gglean000247	0.111367	0.115645	0.0905067	0
Gglean000248	20.24451918	21.3716766	16.49900061	15.822018
Gglean000249	0.086212735	0.589970115	0.264044436	0.482169965
Gglean000250	0	0	0	0
Gglean000251	45.9943	65.20548	43.8779	53.0114
Gglean000252	45.9943	65.20548	43.8779	53.0114
Gglean000253	4.08E-06	4.41E-17	4.59186	3.61E-33
Gglean000254	0	0	0	0
Gglean000255	159.9118	156.67087	363.89854	314.21304
Gglean000256	10.99388	11.8486	7.9679	7.76505
Gglean000257	14.47868027	12.656106	9.580546	8.492271106
Gglean000258	14.47868027	12.656106	9.580546	8.492271106
Gglean000259	31.15238995	34.48589	39.26001	40.1239
Gglean000260	31.15238995	34.48589	39.26001	40.1239
Gglean000261	1.425036914	1.0383344	2.026291209	3.19181
Gglean000262	1.115647	1.40199558	1.069592261	2.228443
Gglean000263	4.765905015	4.314005274	4.671615003	4.230964784

Gglean000264	4.765905015	4.314005274	4.671615003	4.230964784
Gglean000265	4.765905015	4.314005274	4.671615003	4.230964784
Gglean000266	6.087442002	5.39059416	8.511575	5.8953053
Gglean000267	0.0893394	0.2347573	0.241467739	0.2128502
Gglean000268	0.0893394	0.2347573	0.241467739	0.2128502
Gglean000269	0.0893394	0.2347573	0.241467739	0.2128502
Gglean000270	6.028600947	10.20819312	8.914344379	11.71679941
Gglean000271	810.38403	804.57504	903.469634	955.19633
Gglean000272	17.3956509	16.72439	19.000287	19.11
Gglean000273	59.5748	55.159	51.6218	55.934
Gglean000274	42.926967	44.264523	32.932291	30.847929
Gglean000275	42.926967	44.264523	32.932291	30.847929
Gglean000276	21.81947	22.782536	18.34086093	23.2794966
Gglean000277	21.81947	22.782536	18.34086093	23.2794966
Gglean000278	27.59956	33.3419843	21.844705	20.658489
Gglean000279	810.38403	804.57504	903.469634	955.19633
Gglean000280	810.38403	804.57504	903.469634	955.19633
Gglean000281	1527.1247	1551.71919	1242.3212	1127.6432
Gglean000282	1527.1247	1551.71919	1242.3212	1127.6432
Gglean000283	1527.1247	1551.71919	1242.3212	1127.6432
Gglean000284	2.35667	3.18627	4.42897	5.17171
Gglean000285	55.01377	57.45923	61.50172	63.1778
Gglean000286	219.8646774	243.052321	145.1604906	133.7916803
Gglean000287	0	0	0	0
Gglean000288	0	0	0	0.29133
Gglean000289	0.510827206	0.65720519	0.243851307	0.17256788
Gglean000290	1.11497	0.621025	3.21219	4.40845
Gglean000291	0	0	0	0
Gglean000292	164.177921	174.469154	118.633312	104.408684
Gglean000293	54.712903	56.388081	54.04341151	59.04426774
Gglean000294	32.528935	32.463951	51.29053	55.13615
Gglean000295	0	0	0	0.730983
Gglean000296	0	0	0	0
Gglean000297	23.70650299	25.08203663	24.164111	25.656651
Gglean000298	0.114068	0.324311	0.0463874	0.109622
Gglean000299	16.82012	14.772638	17.081297	24.851815
Gglean000300	0	0	0	0
Gglean000301	34.39527	38.28001002	32.5323947	34.00385
Gglean000302	0.0817594	0	0	0
Gglean000303	7.88046113	6.938272	10.657372	13.43707837
Gglean000304	8.055982962	6.979650468	9.664896564	13.07909672
Gglean000305	156.977	157.902	5.58992	1.21296
Gglean000306	28.97690925	20.73515	32.27433	39.490621
Gglean000307	11.94248251	9.429484023	18.31830483	20.54230562

Gglean000308	0.05631923	0.11556225	0.045496603	0.068298266
Gglean000309	9.3432068	8.26262	8.7307946	8.905602
Gglean000310	18.375395	19.3040691	13.5277153	11.044716
Gglean000311	32.6033	29.72943	27.30511	27.59435
Gglean000312	3.8663695	3.940545891	6.6156117	7.101638363
Gglean000313	11.3469	7.0247	7.41598	7.12949
Gglean000314	0.410558568	1.037512	0.32293	0.4370571
Gglean000315	0.410558568	1.037512	0.32293	0.4370571
Gglean000316	61.57879	55.31022	50.291648	48.736
Gglean000317	61.51641	72.74929	45.82271	46.99799
Gglean000318	42.8002769	44.77194388	31.01012074	30.9536648
Gglean000319	42.8002769	44.77194388	31.01012074	30.9536648
Gglean000320	0.144392	0	0.118498	0
Gglean000321	10.17605515	8.65436	11.96037	12.76963
Gglean000322	10.17605515	8.65436	11.96037	12.76963
Gglean000323	81.03677246	82.80229168	72.32695446	76.68943039
Gglean000324	81.03677246	82.80229168	72.32695446	76.68943039
Gglean000325	23.75097895	22.77449445	26.64541973	28.118522
Gglean000326	15.64695	16.70545	18.56104	26.95087
Gglean000327	0	0.855828006	0	0
Gglean000328	0	0	0	0.0963511
Gglean000329	0	0.316767	0	0
Gglean000330	0	0	0	0.0745885
Gglean000331	2.46576	3.13E-09	0.561131	6.46E-10
Gglean000332	4.14E-06	0.000255163	0.000186209	4.40E-05
Gglean000333	62.79733	56.1846	59.3462	64.3661
Gglean000334	52.28925	50.00855	45.57137	40.24697
Gglean000335	31.63607	35.29591	27.9086	29.62911
Gglean000336	2.097051	3.316655025	1.614705102	8.04735394
Gglean000337	80.78406049	88.2491228	66.52919264	63.99937375
Gglean000338	51.19704518	94.7727	107.7551	150.4733
Gglean000339	42.061514	36.79588279	37.805165	36.100864
Gglean000340	0	0.327174	0.932357	0.703002
Gglean000341	80.635899	85.330096	46.53675037	41.15919889
Gglean000342	80.635899	85.330096	46.53675037	41.15919889
Gglean000343	0	0	0	0
Gglean000344	82.50831089	87.77403609	78.49473441	73.14644067
Gglean000345	0	0.235153	0.54491	0.432295
Gglean000346	0	0	0	0
Gglean000347	215.878	240.0389	96.433	64.80467
Gglean000348	215.878	240.0389	96.433	64.80467
Gglean000349	42.7057	40.7771	35.0215	33.9477
Gglean000350	47.0996	46.1419	53.7732	109.821
Gglean000351	0.0690899	0	0.0563056	0.0669542

Gglean000352	5.22512	6.91094	6.1066	7.35982
Gglean000353	29.57442601	33.79231	35.82591	35.61644
Gglean000354	88.6593	87.5053	90.8863	96.5085
Gglean000355	16.377	13.4168	12.6071	14.3555
Gglean000356	55.39564139	52.95053106	85.40029852	95.85705268
Gglean000357	55.39564139	52.95053106	85.40029852	95.85705268
Gglean000358	87.2581	92.0656	99.9975	111.3463
Gglean000359	24.9029366	26.30402144	28.57834031	29.75608488
Gglean000360	12.18774816	12.09232125	18.01381744	21.19771581
Gglean000361	5.434192887	7.027445	8.43485	10.169366
Gglean000362	1.05E-36	0.337251	3.68E-11	7.61E-68
Gglean000363	7.034551	6.182507	6.407819	5.924334
Gglean000364	21.57947	61.99246	41.18983	67.00791
Gglean000365	19.9943	20.9787	14.53537117	15.0756576
Gglean000366	19.9943	20.9787	14.53537117	15.0756576
Gglean000367	25.8336	24.2627	21.8762	23.4151
Gglean000368	16.295	15.67543	14.10376	13.58824
Gglean000369	0.654323	1.20479003	0.82444	1.36155
Gglean000370	49.60714	48.66996	74.2923	78.9602
Gglean000371	7.79696	9.36035	9.52229	14.79094
Gglean000372	0	0	0	0
Gglean000373	72.42538281	70.87025477	55.13569078	56.81931695
Gglean000374	15.24532	17.15293	12.28546	11.69437
Gglean000375	0	0	0	0
Gglean000376	98.42464172	91.393723	79.2081371	88.4216533
Gglean000377	83.157	89.1399	65.0854	62.9968
Gglean000378	0	0.176232	0.137238	0
Gglean000379	0.16834163	0	0.252711214	0.199110201
Gglean000380	10.66695422	9.243945102	15.28209742	18.9744084
Gglean000381	0	0	0	0
Gglean000382	0.166239993	0.086406063	0.104304028	0.047867484
Gglean000383	0.5852451	0.0560609	0	0.5698933
Gglean000384	1.668172293	1.715578049	0.967313535	0.84408225
Gglean000385	0	0.0440813	0	0
Gglean000386	59.11820662	63.108254	86.47159806	77.9609356
Gglean000387	30.2746	20.0428	34.8598	34.1234
Gglean000388	72.49236	78.8743	57.49464	54.80784
Gglean000389	72.49236	78.8743	57.49464	54.80784
Gglean000390	41.27216	35.753872	18.819394	14.81936004
Gglean000391	0.0234285	0.0724851	0.0759635	0.202068
Gglean000392	0.226009313	0.263971046	0.656156404	0.734910156
Gglean000393	6.05925	5.8465	6.8149	8.51713
Gglean000394	3.36826	3.98094	5.8842	6.61697
Gglean000395	13.95163666	12.07538462	23.5433631	27.27433647

Gglean000396	49.71258	50.47617	51.69412	47.02731
Gglean000397	57.30217	68.676881	81.2847935	98.82343
Gglean000398	57.30217	68.676881	81.2847935	98.82343
Gglean000399	56.52082	54.445083	60.44573	65.02538021
Gglean000400	56.52082	54.445083	60.44573	65.02538021
Gglean000401	35.4696	38.3728	46.752	46.9586
Gglean000402	30.40849	33.47598	23.58172	25.86374
Gglean000403	0	0	0	0
Gglean000404	96.5745	90.25356	43.07532	33.75153
Gglean000405	33.93754	35.27978	39.89598	56.95703
Gglean000406	1.8563242	1.766691833	3.665362	2.755845521
Gglean000407	138.9616715	143.13496	155.4585349	261.184728
Gglean000408	9.843610567	9.231162002	7.642870004	5.69372
Gglean000409	9.843610567	9.231162002	7.642870004	5.69372
Gglean000410	9.843610567	9.231162002	7.642870004	5.69372
Gglean000411	9.843610567	9.231162002	7.642870004	5.69372
Gglean000412	0	0.299866	0	0
Gglean000413	0.197991	0.47545	1.54E-05	0.471516
Gglean000414	0	0	0	0
Gglean000415	0	0	0	0
Gglean000416	0	0	0	0
Gglean000417	0	0	0	0
Gglean000418	16.44758	15.21317161	16.764219	15.326237
Gglean000419	27.462434	13.13637	64.229069	7.994368
Gglean000420	34.606028	38.028	23.621014	20.70624396
Gglean000421	34.606028	38.028	23.621014	20.70624396
Gglean000422	43.312654	47.35022292	57.303213	53.03164115
Gglean000423	19.94381509	21.24990421	13.70421384	11.31952167
Gglean000424	0	0	0	0
Gglean000425	62.7433	68.8035	35.4691	26.9344
Gglean000426	5.677000364	6.226238626	4.0093345	3.425575313
Gglean000427	5.677000364	6.226238626	4.0093345	3.425575313
Gglean000428	0.107908	0.337039	0.350165	0.104039
Gglean000429	43.86134	52.09633	64.9359	60.48571
Gglean000430	13.4504	13.0355	9.82749	8.10886
Gglean000431	121.7996644	106.825418	131.1737436	111.909642
Gglean000432	0	0	0	0
Gglean000433	4.95201	4.92699	3.58399	3.23137
Gglean000434	39.55972495	33.77407632	57.52395258	57.62185033
Gglean000435	39.55972495	33.77407632	57.52395258	57.62185033
Gglean000436	48.22041069	56.24697818	100.8235663	92.15793855
Gglean000437	48.22041069	56.24697818	100.8235663	92.15793855
Gglean000438	24.29116	22.94048	20.683	20.57226
Gglean000439	14.50414	10.65343	6.697603	7.673236

Gglean000440	30.6941	32.5357	26.8023	22.2851
Gglean000441	34.74407	39.78944	29.09287751	28.38887
Gglean000442	352.4187642	357.03563	380.832738	392.424334
Gglean000443	22.4442	20.3828	16.9224	13.9569
Gglean000444	34.92931	33.416017	25.72575761	23.19974
Gglean000445	19.44974791	20.14843554	24.7691	24.83371136
Gglean000446	34.92931	33.416017	25.72575761	23.19974
Gglean000447	26.17336	28.60497767	16.69571741	14.66390184
Gglean000448	69.5234	69.6085	57.7268	58.878
Gglean000449	1.435231	0.90188	0.884149	1.757834
Gglean000450	2.57681101	0.911910313	0.050239221	0.059597972
Gglean000451	28.4710678	27.757296	48.7288603	43.0300671
Gglean000452	28.4710678	27.757296	48.7288603	43.0300671
Gglean000453	23.634027	22.68369	24.1886	23.83849
Gglean000454	1188.22	1244.70855	771.5973056	751.9455907
Gglean000455	47.2389	47.7419	45.8462	39.8389
Gglean000456	0.00362088	7.22508	0.016775	0.00578631
Gglean000457	0	0	0	0
Gglean000458	584.973	613.4209	778.861	1070.954
Gglean000459	287.988	280.668	254.045	431.61
Gglean000460	0	0.432417	0.56801	1.21958
Gglean000461	2.33867008	1.695178	2.21909	4.699921
Gglean000462	7.1804842	5.848554	8.509077042	11.575471
Gglean000463	15.323291	15.88030125	13.501979	25.57390138
Gglean000464	23.8087	28.35461	23.44889	48.8493
Gglean000465	3.495756259	6.509405841	3.63802324	14.62687751
Gglean000466	4.578491	4.7819547	4.2179288	4.100414
Gglean000467	205.6085	186.923	222.0696	193.1373
Gglean000468	4.24838	7.62208	4.46214	5.42212
Gglean000469	8.9029844	11.2814447	6.1463058	7.0330463
Gglean000470	0.6887044	1.308737	1.16759078	1.711575653
Gglean000471	0.6887044	1.308737	1.16759078	1.711575653
Gglean000472	0	0	0	0
Gglean000473	94.88762	74.161944	48.395029	46.09493
Gglean000474	94.88762	74.161944	48.395029	46.09493
Gglean000475	94.88762	74.161944	48.395029	46.09493
Gglean000476	7.88861	9.84153	3.54696	2.88331
Gglean000477	80.774789	94.132145	128.463783	150.611501
Gglean000478	0.234052	0.498097	0.192419	0.229964
Gglean000479	0.913352	1.31215	1.41029	1.55431
Gglean000480	12.45328061	11.84736572	18.45947411	22.377981
Gglean000481	12.45328061	11.84736572	18.45947411	22.377981
Gglean000482	117.22592	119.676156	42.58072362	33.34781051
Gglean000483	55.46749	63.22957493	65.58552438	53.02373067

Gglean000484	28.935301	34.571421	34.229651	38.86664
Gglean000485	47.0847	40.1112	21.3927	16.6442
Gglean000486	0	0	0	0
Gglean000487	0	0	0	0
Gglean000488	1.13191	0.300786	0.229494	0.274107
Gglean000489	12.999013	12.9428	7.95765	6.9396
Gglean000490	0	0	0	0
Gglean000491	26.148865	28.064386	26.173365	23.81927
Gglean000492	21.59567	22.3410993	25.869088	25.67090486
Gglean000493	15.7301	15.3763	13.0835	11.8913
Gglean000494	1.84133	1.5874	1.03595	0.807441
Gglean000495	0	0	0.040467646	0.034966275
Gglean000496	0	0	0.040467646	0.034966275
Gglean000497	4.829488	5.118345	7.937769	6.58709224
Gglean000498	9.780323	8.822573	14.161691	14.328071
Gglean000499	37.45333	39.07093349	57.8155	67.16547
Gglean000500	2.18E-09	0.00322836	4.39E-05	0.000108026
Gglean000501	22.5313119	22.14065355	30.5633249	34.75162414
Gglean000502	0	0	0	0
Gglean000503	23.68141145	28.159774	9.093372416	8.921020186
Gglean000504	30.72909174	33.28863008	25.04247774	24.0545501
Gglean000505	25.0777	21.3156	23.8225	23.6517
Gglean000506	55.4413	55.6181	44.8086	35.5016
Gglean000507	16.95097	13.29876	8.490124	12.15944
Gglean000508	0	0	0	0
Gglean000509	29.571267	29.403382	28.20421633	28.89706
Gglean000510	25.81377	21.93319	27.33025	30.21463
Gglean000511	47.27643835	48.30667983	37.834923	37.19905313
Gglean000512	47.27643835	48.30667983	37.834923	37.19905313
Gglean000513	31.65475	35.17999	36.8405	37.79436
Gglean000514	51.50078	55.49834	65.0202007	61.558586
Gglean000515	23.0188	22.8374	12.9537	11.5005
Gglean000516	35.81246	43.0004	20.00811	15.59586
Gglean000517	3.95721	12.5459	5.49788	3.28963
Gglean000518	15.12135	13.87189	5.66507	6.36462611
Gglean000519	1.12E-10	3.91E-08	3.48E-07	1.03E-09
Gglean000520	1.12E-10	3.91E-08	3.48E-07	1.03E-09
Gglean000521	9.6102	8.42168	10.32631	8.4853
Gglean000522	21.771029	20.65089646	9.4525621	10.32605843
Gglean000523	38.59114	41.64249	27.52691	24.73153
Gglean000524	2.096435	3.95065001	3.196763	4.50847
Gglean000525	2.096435	3.95065001	3.196763	4.50847
Gglean000526	0.234772007	0.081147031	0.575019337	0.377792692
Gglean000527	38.441585	36.642298	42.1121595	41.45983501

Gglean000528	38.441585	36.642298	42.1121595	41.45983501
Gglean000529	0.386698	0	0.321674	0
Gglean000530	0	0	0	0
Gglean000531	36.5195424	38.1508809	50.03224058	53.92539684
Gglean000532	19.53355303	20.94237101	18.34896698	18.41788458
Gglean000533	19.53355303	20.94237101	18.34896698	18.41788458
Gglean000534	62.05040529	67.17446	38.09585417	35.64508952
Gglean000535	62.05040529	67.17446	38.09585417	35.64508952
Gglean000536	0	0	0	0
Gglean000537	47.27696	53.2448	51.7701	51.68452
Gglean000538	264.8192529	234.0053694	198.3214557	205.9873978
Gglean000539	264.8192529	234.0053694	198.3214557	205.9873978
Gglean000540	264.8192529	234.0053694	198.3214557	205.9873978
Gglean000541	264.8192529	234.0053694	198.3214557	205.9873978
Gglean000542	264.8192529	234.0053694	198.3214557	205.9873978
Gglean000543	264.8192529	234.0053694	198.3214557	205.9873978
Gglean000544	264.8192529	234.0053694	198.3214557	205.9873978
Gglean000545	264.8192529	234.0053694	198.3214557	205.9873978
Gglean000546	264.8192529	234.0053694	198.3214557	205.9873978
Gglean000547	264.8192529	234.0053694	198.3214557	205.9873978
Gglean000548	264.8192529	234.0053694	198.3214557	205.9873978
Gglean000549	264.8192529	234.0053694	198.3214557	205.9873978
Gglean000550	264.8192529	234.0053694	198.3214557	205.9873978
Gglean000551	264.8192529	234.0053694	198.3214557	205.9873978
Gglean000552	264.8192529	234.0053694	198.3214557	205.9873978
Gglean000553	264.8192529	234.0053694	198.3214557	205.9873978
Gglean000554	264.8192529	234.0053694	198.3214557	205.9873978
Gglean000555	264.8192529	234.0053694	198.3214557	205.9873978
Gglean000556	264.8192529	234.0053694	198.3214557	205.9873978
Gglean000557	264.8192529	234.0053694	198.3214557	205.9873978
Gglean000558	264.8192529	234.0053694	198.3214557	205.9873978
Gglean000559	264.8192529	234.0053694	198.3214557	205.9873978
Gglean000560	264.8192529	234.0053694	198.3214557	205.9873978
Gglean000561	264.8192529	234.0053694	198.3214557	205.9873978
Gglean000562	10.807429	12.90708104	11.014199	13.53353
Gglean000563	7.828324	11.563079	7.076781326	9.586515
Gglean000564	16.25903287	13.2730233	13.16491752	11.63901255
Gglean000565	17.247614	21.49578067	17.171657	17.477207
Gglean000566	17.247614	21.49578067	17.171657	17.477207
Gglean000567	25.1156655	23.888997	31.224729	33.805245
Gglean000568	25.1156655	23.888997	31.224729	33.805245
Gglean000569	25.1156655	23.888997	31.224729	33.805245
Gglean000570	0.42311	0.924425	1.42488	2.13286
Gglean000571	0	0	0	0.485778

Gglean000572	56.1166	46.7724	64.1173	72.1599
Gglean000573	21.186821	20.2098372	20.88713977	19.024481
Gglean000574	21.186821	20.2098372	20.88713977	19.024481
Gglean000575	0	0	0	0
Gglean000576	30.8972	32.7982	28.4235	26.9729
Gglean000577	6.30425	14.20682	6.92517	10.96317
Gglean000578	6.30425	14.20682	6.92517	10.96317
Gglean000579	38.69241	36.48211	47.40368	44.74744
Gglean000580	38.69241	36.48211	47.40368	44.74744
Gglean000581	7.039717702	7.148901195	15.48154752	14.86404489
Gglean000582	4.698797577	4.14145048	3.050286931	2.581097754
Gglean000583	0	0	0	0
Gglean000584	0	0	0	0
Gglean000585	17.09149	17.63262269	19.541182	22.137417
Gglean000586	18.1094	21.024	10.1158	12.839
Gglean000587	236.2981	257.3846	237.7063	300.9735
Gglean000588	25.39483984	23.130419	15.46761261	13.1648269
Gglean000589	1.15475	1.91E-08	6.13E-07	2.60013
Gglean000590	32.3408	38.59291	18.65976	19.04745
Gglean000591	0	0	0	0
Gglean000592	47.17817775	46.69303404	35.5979076	32.47843049
Gglean000593	0.138366255	0.091607171	0.167038092	0.23063889
Gglean000594	0	0	0	0
Gglean000595	2.429000281	1.849820347	1.6899437	1.827800097
Gglean000596	20.94325	16.20919	15.89137	14.70274
Gglean000597	20.94325	16.20919	15.89137	14.70274
Gglean000598	20.94325	16.20919	15.89137	14.70274
Gglean000599	37.395101	37.699008	23.50249526	27.37611
Gglean000600	37.395101	37.699008	23.50249526	27.37611
Gglean000601	1.41661	2.41936	2.69629	3.92493
Gglean000602	7.705981846	8.19941767	11.646087	12.82803109
Gglean000603	110.6276	130.1642	118.9663	128.7104
Gglean000604	13.73661142	16.11336593	13.85971461	12.80410916
Gglean000605	30.370151	31.23478933	29.32257408	30.619352
Gglean000606	3.825408	3.511244276	4.911771	5.170873655
Gglean000607	3.825408	3.511244276	4.911771	5.170873655
Gglean000608	2.104608746	1.187040708	3.199836	2.255608
Gglean000609	0	0	0	0
Gglean000610	37.52905801	38.48522471	52.673173	63.692969
Gglean000611	2.736458	2.357582	4.34608	4.26949
Gglean000612	37.52905801	38.48522471	52.673173	63.692969
Gglean000613	7.36475	5.04944	7.02983	6.60831
Gglean000614	1.14642366	1.071979607	1.327193441	1.228941195
Gglean000615	0.673341099	2.360104331	1.288651837	1.158723482

Gglean000616	0.597613	0.802877	1.579085	1.6199
Gglean000617	22.10891773	19.97872902	21.00007303	19.35225749
Gglean000618	22.10891773	19.97872902	21.00007303	19.35225749
Gglean000619	65.4668	73.1884	32.13036	33.70415
Gglean000620	35.34485	31.07923	15.20033	14.04966
Gglean000621	1.996584406	5.17777	6.82195842	9.868808
Gglean000622	5.970265086	9.802387494	2.782707844	3.077413356
Gglean000623	5.300381	5.913056	6.067583	9.34237
Gglean000624	23.453681	20.794123	20.985297	23.023264
Gglean000625	0.2754	0.291464	0.451209	0.271772
Gglean000626	7.01843	7.02193	9.64704	12.03688
Gglean000627	28.80867	32.34144	19.29229	21.91923
Gglean000628	66.3641	68.4076	60.7842	57.2777
Gglean000629	19.754713	18.179403	15.447944	13.601198
Gglean000630	19.3777	22.0553	10.5856	7.02078
Gglean000631	0.244563	0.254815	0.349479	0.353461
Gglean000632	86.78712	94.34973	56.53523	48.93145
Gglean000633	36.8021	41.9581	25.7839	24.9569
Gglean000634	18.3399909	19.05084	26.221741	24.14734674
Gglean000635	0.707909198	0.689846299	0.576297903	0.4946595
Gglean000636	1.74104	1.66651	1.90286	2.33913
Gglean000637	47.79835	55.31405	41.33703	37.02334
Gglean000638	0	0	0	0
Gglean000639	0	0	0	0
Gglean000640	93.9013	102.5724	75.7515	74.5665
Gglean000641	96.9684	90.10584	114.484	155.1709
Gglean000642	16.320477	13.17502904	14.97443	21.47715002
Gglean000643	16.320477	13.17502904	14.97443	21.47715002
Gglean000644	25.45136	26.45206	18.59016	14.974136
Gglean000645	2.348900699	2.318230268	1.210171056	2.238691249
Gglean000646	21.92488624	22.88060413	23.88240256	24.69975685
Gglean000647	21.92488624	22.88060413	23.88240256	24.69975685
Gglean000648	0	0	0	0
Gglean000649	0.726826	1.50852	1.18166	1.89559
Gglean000650	2.097051	3.316655025	1.614705102	8.04735394
Gglean000651	0	0	0	0
Gglean000652	55.80673	40.2177	53.20129	59.5226
Gglean000653	13.5195	11.5676	12.8722	14.6406
Gglean000654	55.80673	40.2177	53.20129	59.5226
Gglean000655	21.079986	21.5383689	21.54530348	19.194389
Gglean000656	2.210914	3.5403	4.35789	4.435244
Gglean000657	34.7843	35.3012	19.9436	18.2909
Gglean000658	52.4619	44.20904	31.95231	28.373
Gglean000659	56.16007545	54.29204303	41.454814	35.02763833

Gglean000660	0	0	0	0
Gglean000661	32.9733	36.0476	34.0956	27.1647
Gglean000662	0	0	0	0
Gglean000663	0.163937	0	0.133596	0.158252
Gglean000664	8.018479	6.73043199	13.21926456	14.22582066
Gglean000665	0	0	0	0
Gglean000666	6.38993262	5.928655462	5.1363439	6.737466466
Gglean000667	5.69019	6.13683	4.37239	5.623
Gglean000668	45.2910697	49.19009333	30.4455647	28.28689543
Gglean000669	121.9210238	121.9150108	143.9850091	153.4120181
Gglean000670	36.8783316	35.76674588	52.0081721	91.90312835
Gglean000671	6.1127	5.231849	7.483012	15.92152
Gglean000672	38.040323	38.043688	45.10875221	47.551972
Gglean000673	38.040323	38.043688	45.10875221	47.551972
Gglean000674	7.444936477	15.01140284	10.60880626	11.7092138
Gglean000675	7.444936477	15.01140284	10.60880626	11.7092138
Gglean000676	7.444936477	15.01140284	10.60880626	11.7092138
Gglean000677	0.632315	0.407223	0.190396	0.301159
Gglean000678	0.48218848	0.658863454	0.654492692	1.029631149
Gglean000679	58.1298	55.5979	38.67475	35.23227
Gglean000680	39.7866	7.6203	21.3193	23.6202
Gglean000681	0	0	0	0
Gglean000682	25.58293	32.59244419	19.16203	15.66597
Gglean000683	41.783432	41.458677	42.903765	41.958357
Gglean000684	3.40735	4.37345	3.34933	3.82089
Gglean000685	88.73413381	84.35736468	78.48846287	79.8920711
Gglean000686	116.1963	112.5474	3.04293	0.655139
Gglean000687	13.561956	14.14607784	11.3740491	9.910471481
Gglean000688	13.561956	14.14607784	11.3740491	9.910471481
Gglean000689	0	0	0	0
Gglean000690	0	0	0	0
Gglean000691	0	0	0	0
Gglean000692	0	0	0	0
Gglean000693	25.36102	29.01318	17.81666	21.6025
Gglean000694	12.80168061	15.77826249	10.86516248	11.19323811
Gglean000695	72.17207	77.99814	64.281	60.22838
Gglean000696	0.202209348	0.889799987	0.3939603	0.491334
Gglean000697	0	0.914422	0.675239	0
Gglean000698	0	0	0	0
Gglean000699	8.86382	9.02296	3.26025	2.47633
Gglean000700	0	0	0	0
Gglean000701	0	0.133156506	0.0297616	0.0351868
Gglean000702	3.22644	2.156326	2.160848	1.96796
Gglean000703	209.1753	220.5962	116.2529	99.55247

Gglean000704	5.494833561	5.249663	7.543305621	8.16507
Gglean000705	95.27866	93.0970816	151.56166	184.9909
Gglean000706	40.739895	45.38069	25.9025044	23.50126291
Gglean000707	3.52E-05	5.56E-05	2.79E-05	5.04E-05
Gglean000708	13.55326	14.8887	8.38621	6.80275
Gglean000709	13.55326	14.8887	8.38621	6.80275
Gglean000710	16.7922	17.11742	18.50273	18.88398
Gglean000711	39.64171	39.64429	11.62146	10.70779
Gglean000712	107.072	111.167	77.7925	82.3395
Gglean000713	1.510743566	1.465155173	1.894839751	2.064258163
Gglean000714	1.510743566	1.465155173	1.894839751	2.064258163
Gglean000715	0.730636	0.285541	0.148996	0.0882476
Gglean000716	31.3159	31.45849	39.44027	54.47863
Gglean000717	31.3159	31.45849	39.44027	54.47863
Gglean000718	31.3159	31.45849	39.44027	54.47863
Gglean000719	110.918	107.514	59.7056	52.8104
Gglean000720	1.03325	5.88532	3.46997	2.07346
Gglean000721	7.18633	8.254132	10.1972381	10.6892605
Gglean000722	18.31992	19.48679	17.45841	28.6512
Gglean000723	24.834131	25.017898	23.289206	23.42757
Gglean000724	18.93265	17.09818	18.03312	19.49993
Gglean000725	34.48428412	28.8860816	30.66455763	29.23230748
Gglean000726	18.4023	21.16855	10.15256	7.48339
Gglean000727	18.4023	21.16855	10.15256	7.48339
Gglean000728	18.4023	21.16855	10.15256	7.48339
Gglean000729	5.34475	5.41294	4.24934	4.43574
Gglean000730	9.79302	9.2258	5.93566	6.14336
Gglean000731	12.954842	14.82042	20.51086	25.13505
Gglean000732	4.633276628	3.919981576	6.026748522	7.354724983
Gglean000733	0	0	0.372775	0.1925773
Gglean000734	0.701378	2.05159	0.908194	1.38455
Gglean000735	5.2378	5.138820159	2.929120057	2.83595
Gglean000736	2.793709	2.843396478	4.315824	3.8704351
Gglean000737	38.4732	46.1075	51.6102	56.1892
Gglean000738	5.48859	4.97292	8.08467	10.4561
Gglean000739	0.035281289	0	0	0
Gglean000740	4.4161292	4.905097474	2.252314884	2.179647
Gglean000741	4.4161292	4.905097474	2.252314884	2.179647
Gglean000742	5.07633	4.82135	4.48201	7.78105
Gglean000743	35.0281307	35.02920871	44.39369619	56.0220157
Gglean000744	35.0281307	35.02920871	44.39369619	56.0220157
Gglean000745	49.94064	48.9783	22.46584	13.973313
Gglean000746	33.29394151	31.283604	28.15149792	31.060757
Gglean000747	1.658558803	1.203882203	2.067615869	2.118034789

Gglean000748	1.658558803	1.203882203	2.067615869	2.118034789
Gglean000749	46.88938932	58.9773	69.37201	68.14668356
Gglean000750	46.88938932	58.9773	69.37201	68.14668356
Gglean000751	46.88938932	58.9773	69.37201	68.14668356
Gglean000752	28.6031	27.2774	26.2474	25.4129
Gglean000753	0	0	0.097960486	0.232256589
Gglean000754	0.145779	0	0.236074	0.27951
Gglean000755	0	0	0	0.354277
Gglean000756	0	0	0	0
Gglean000757	26.0276	27.147	49.3349	51.0504
Gglean000758	39.47153	37.3627	30.22547	29.5065
Gglean000759	70.35715069	63.76573399	65.66682006	66.017067
Gglean000760	9.53659	9.668437	17.87290595	22.63964
Gglean000761	9.53659	9.668437	17.87290595	22.63964
Gglean000762	9.53659	9.668437	17.87290595	22.63964
Gglean000763	5.073740041	4.05244	3.63203	4.17606
Gglean000764	12.92710653	9.68583	7.59269475	10.84497
Gglean000765	0	0	0	0
Gglean000766	0	0	0	0
Gglean000767	57.3526	53.3779	77.2681	88.3262
Gglean000768	0	0	0	0
Gglean000769	0	0	0	0
Gglean000770	0	0	0	0
Gglean000771	12.52232203	11.55112189	21.44112845	24.20108113
Gglean000772	1084.78	933.508	863.317	1260.8
Gglean000773	1134.187	1215.599	1342.394	1863.99
Gglean000774	845.946	721.573	713.502	1069.72
Gglean000775	0	0	0	0
Gglean000776	0	0	0	0
Gglean000777	0.378281	1.06E-05	4.52E-06	0.485225
Gglean000778	15.537474	13.959053	24.22111	27.03109
Gglean000779	0	0	0	0
Gglean000780	46.42242349	43.70483171	73.71790087	71.0787034
Gglean000781	10.6212	11.3924	5.49951	4.35677
Gglean000782	20.751983	22.53257802	25.53117152	26.52955824
Gglean000783	0.012589	1.18E-14	2.78E-11	1.85E-07
Gglean000784	5.936167457	6.231714705	5.814707584	6.054436472
Gglean000785	51.77245	53.07662	55.04822	71.23327
Gglean000786	115.6647	106.48539	103.3712	98.85932
Gglean000787	19.23731	23.44101	13.21468	9.23211
Gglean000788	46.06187482	59.26161525	38.05017083	35.43444118
Gglean000789	0.0382645	0	0.0929739	0.0733839
Gglean000790	79.309485	79.97678	129.01269	142.45091
Gglean000791	9.64519	9.16136	6.29676	5.7905

Gglean000792	0	0.0367144	0	0
Gglean000793	54.94926	52.7490873	67.536564	48.41027
Gglean000794	54.94926	52.7490873	67.536564	48.41027
Gglean000795	21.2757	21.0381	0.808782	0
Gglean000796	18.95275	21.1635	29.62541	33.67616
Gglean000797	16.392872	15.755826	19.239669	29.76062
Gglean000798	3.7324499	3.8588398	2.69530524	3.680794235
Gglean000799	0	0.126240601	0	0
Gglean000800	0	0.126240601	0	0
Gglean000801	0	0	0	0
Gglean000802	1.33839	1.09134	0.278141	0.666603
Gglean000803	10.170478	9.52395	10.747716	11.49856
Gglean000804	1.75601	1.3342	2.66253	2.70559
Gglean000805	48.816193	48.31193872	59.9699669	72.69444717
Gglean000806	48.816193	48.31193872	59.9699669	72.69444717
Gglean000807	5.00638	0	3.09743	6.21383
Gglean000808	114.17998	93.523763	93.09001	68.07011
Gglean000809	114.17998	93.523763	93.09001	68.07011
Gglean000810	1137.741159	1092.080517	1627.051953	2011.24026
Gglean000811	75.13452896	67.70005302	88.44341654	108.3567516
Gglean000812	75.13452896	67.70005302	88.44341654	108.3567516
Gglean000813	24.5556	26.179	28.9635	29.8347
Gglean000814	75.13452896	67.70005302	88.44341654	108.3567516
Gglean000815	75.13452896	67.70005302	88.44341654	108.3567516
Gglean000816	2.30645	3.40093	4.35294	6.22566
Gglean000817	0.00193944	0.000149048	0.448177	3.10234
Gglean000818	18.475534	18.847809	15.32059547	16.860157
Gglean000819	18.475534	18.847809	15.32059547	16.860157
Gglean000820	18.475534	18.847809	15.32059547	16.860157
Gglean000821	17.51342209	22.69779219	8.009943659	8.582745
Gglean000822	17.51342209	22.69779219	8.009943659	8.582745
Gglean000823	778.1156448	872.1360078	529.4440291	466.0538609
Gglean000824	13.84398	13.30982	10.53379	9.18859
Gglean000825	114.71251	113.16313	88.55598	75.11777
Gglean000826	114.71251	113.16313	88.55598	75.11777
Gglean000827	28.5506	25.8983	20.35084	19.79078
Gglean000828	874.591	1056.057	477.4885	412.6866
Gglean000829	315.6329	291.2463	318.1989	312.2251
Gglean000830	315.6329	291.2463	318.1989	312.2251
Gglean000831	40.79506	52.87695	55.83622	49.82134747
Gglean000832	40.79506	52.87695	55.83622	49.82134747
Gglean000833	52.686714	55.251048	64.861463	63.467099
Gglean000834	0	0	0	0
Gglean000835	4.674280784	5.727859036	2.28122059	2.2621388

Gglean000836	0.554462	0.136604838	0.268702279	0.573814097
Gglean000837	0.162465605	0.07121292	0.115716274	0.241506847
Gglean000838	0.274241	0.445341	0.493524	0.709168
Gglean000839	0.00106797	0.00137786	0.0029138	0.251254
Gglean000840	49.29725677	46.18182576	47.09303061	56.0744442
Gglean000841	49.29725677	46.18182576	47.09303061	56.0744442
Gglean000842	49.29725677	46.18182576	47.09303061	56.0744442
Gglean000843	45.4625675	51.462307	39.88942	38.24139
Gglean000844	49.329614	56.2179154	45.27145699	42.75584617
Gglean000845	8.832745533	7.678186683	10.13472	10.73360918
Gglean000846	19.82751	21.29977	20.06169	31.71472
Gglean000847	38.65437008	41.50948147	44.32033676	42.144694
Gglean000848	1.90453	3.71506	1.8857	0.377745
Gglean000849	0	0	0	0
Gglean000850	19.45199	19.447974	29.97884973	35.90008619
Gglean000851	19.45199	19.447974	29.97884973	35.90008619
Gglean000852	29.38044	30.896237	40.10447683	45.834509
Gglean000853	0	0.144022	0.0566473	0.0334901
Gglean000854	8.587087175	7.892240797	10.29231219	12.38978877
Gglean000855	1.4318736	2.12430128	1.395870128	1.758621663
Gglean000856	0.331086	0.17121	0.223189	0.370835
Gglean000857	47.29972	43.98622	37.33367	36.81329
Gglean000858	0	0	0.17671	0.105284
Gglean000859	0	0	0	0
Gglean000860	0.021075813	0.021306293	0.035784526	0
Gglean000861	0.021075813	0.021306293	0.035784526	0
Gglean000862	0.05961304	0.147352493	0.077529986	0
Gglean000863	6.678553174	7.272796	8.909128131	12.35563373
Gglean000864	0	0	0	0
Gglean000865	0.360875764	0.3064257	0.432610136	0.609525218
Gglean000866	5.00149	0.782986	2.22E-05	0.000899963
Gglean000867	1085.107	1255.656	779.471	717.715
Gglean000868	7.579744969	7.021131	12.661884	11.321864
Gglean000869	12.59098033	11.88366335	22.32994355	22.45785407
Gglean000870	0	0	0	0
Gglean000871	103.26813	116.94267	159.33078	178.62726
Gglean000872	103.26813	116.94267	159.33078	178.62726
Gglean000873	1.043568	0.2188383	0.825503	1.110986428
Gglean000874	1.043568	0.2188383	0.825503	1.110986428
Gglean000875	34.99948	36.968364	19.787553	17.226313
Gglean000876	23.890498	25.011551	16.118245	14.078723
Gglean000877	0.114712	0.363008	0.378584	0.223897674
Gglean000878	32.9535	37.8123	31.0485	29.6673
Gglean000879	22.49452089	23.00345211	29.3045051	33.4653507

Gglean000880	22.49452089	23.00345211	29.3045051	33.4653507
Gglean000881	106.6856	110.8591	106.272	111.0173
Gglean000882	2.288909396	2.63146148	3.737634306	3.476385311
Gglean000883	39.801	33.70494	47.04954	53.14145
Gglean000884	9.704301584	11.25256105	22.236133	20.7156058
Gglean000885	9.704301584	11.25256105	22.236133	20.7156058
Gglean000886	39.6748	37.0828	14.3591	9.8822
Gglean000887	10.92781215	11.69573644	14.09105141	17.78583253
Gglean000888	39.2291	45.4408	40.7352	40.1159
Gglean000889	25.45688	27.67326	32.74112	35.5014
Gglean000890	17.68803	23.52558	8.02636	7.81347
Gglean000891	1.036396	1.245422	1.024460694	1.481864
Gglean000892	5.574461	7.263227	7.36635	11.36919
Gglean000893	5.574461	7.263227	7.36635	11.36919
Gglean000894	5.574461	7.263227	7.36635	11.36919
Gglean000895	5.805455	6.643531	5.572563	5.521429
Gglean000896	5.805455	6.643531	5.572563	5.521429
Gglean000897	0	0	0	0
Gglean000898	0.0766615	0.0394229	0.15536	0.368881
Gglean000899	12.80867	12.0509	5.954239	5.2347
Gglean000900	12.80867	12.0509	5.954239	5.2347
Gglean000901	0	0.741804	0	0.65188
Gglean000902	30.54330631	31.00214431	24.2924283	23.617617
Gglean000903	579.7569	462.6609	544.8591	555.7233
Gglean000904	1.08062	0.595328	0.444172	1.06798
Gglean000905	15.59506	16.228	18.70159	18.96187
Gglean000906	3.571354926	5.550221	5.197196289	5.467313169
Gglean000907	3.571354926	5.550221	5.197196289	5.467313169
Gglean000908	0.337647608	0.904695158	0.732739714	0.838226869
Gglean000909	0.337647608	0.904695158	0.732739714	0.838226869
Gglean000910	3.825972121	4.02314502	4.10092	5.02413
Gglean000911	0.089078518	0	0.091922891	0.16900985
Gglean000912	0.089078518	0	0.091922891	0.16900985
Gglean000913	3.60455	4.44581	6.9285	5.98869
Gglean000914	0	0	0	0
Gglean000915	10.87718106	12.25363779	16.108039	20.81534207
Gglean000916	1.917188	1.5560875	1.400958	2.457428
Gglean000917	191.995	178.232	205.931	203.339
Gglean000918	297.9240014	195.87558	229.880124	144.0757628
Gglean000919	297.9240014	195.87558	229.880124	144.0757628
Gglean000920	631.002	680.686	486.684	434.742
Gglean000921	43.52191	43.46607	62.9898	74.62274
Gglean000922	3.694699	6.853817	4.9276	5.607283
Gglean000923	21.39822	22.04703	22.18662	21.58912

Gglean000924	12.90596539	14.2124677	18.97504767	21.931334
Gglean000925	3.94204	5.93252	2.07728	3.68163
Gglean000926	20.66933	22.09016	13.708171	15.30587
Gglean000927	2.010688506	2.624975552	4.218249655	5.794627
Gglean000928	20.147409	5.879724	4.924265	6.23007
Gglean000929	3.46141	0.330613199	2.485615694	1.870324577
Gglean000930	6.26832	2.958496	1.89728129	4.8688
Gglean000931	0	0	0.760921	0
Gglean000932	0	0	0	0
Gglean000933	0.129942	1.037403	0.77157	0.207391
Gglean000934	3.00008374	3.160082	4.736520649	5.573894776
Gglean000935	3.187019	0.782653	1.177316	2.231776
Gglean000936	58.346	57.47553628	51.23947	41.33729028
Gglean000937	58.346	57.47553628	51.23947	41.33729028
Gglean000938	58.346	57.47553628	51.23947	41.33729028
Gglean000939	34.60919	34.40623	22.73158317	25.433997
Gglean000940	19.3586	19.1367	18.703	16.2853
Gglean000941	18.79377	19.08695	29.08143	31.51454
Gglean000942	44.4713089	48.127017	49.75500884	49.70476401
Gglean000943	33.51979718	38.59217932	53.53942576	65.74289715
Gglean000944	33.51979718	38.59217932	53.53942576	65.74289715
Gglean000945	33.51979718	38.59217932	53.53942576	65.74289715
Gglean000946	33.51979718	38.59217932	53.53942576	65.74289715
Gglean000947	12.07264	12.61393	18.73872	21.03187
Gglean000948	15.7977	16.8406	18.837	21.6353
Gglean000949	6.84025	7.456476247	11.0917	11.86014653
Gglean000950	6.84025	7.456476247	11.0917	11.86014653
Gglean000951	0.659427	0.513317	0.759598	0.87309
Gglean000952	0.0790987	0.163513	0.89871	0.379913
Gglean000953	51.9715	46.9415	57.213	92.6073
Gglean000954	48.24170737	54.4083487	44.77832674	40.98782174
Gglean000955	48.24170737	54.4083487	44.77832674	40.98782174
Gglean000956	1.04356	0.575656	2.17102	1.03884
Gglean000957	5.810236264	3.056265	3.484941696	3.884252
Gglean000958	124.187	128.226	128.723	139.651
Gglean000959	61.052018	60.407914	69.315574	69.165356
Gglean000960	61.052018	60.407914	69.315574	69.165356
Gglean000961	34.965495	31.84053999	23.61618198	20.84722009
Gglean000962	0.0200054	0.122255	0.0809877	0.0961586
Gglean000963	12.69074279	12.81812139	11.808888	11.38001525
Gglean000964	13.30380335	12.14865666	17.89007007	27.75170647
Gglean000965	32.26324	33.79597	20.74031	19.4741
Gglean000966	0	0	0	0
Gglean000967	0	0	0	0

Gglean000968	0.061060825	0	0.111283302	0.032968833
Gglean000969	5.03909	7.7749	9.22314	9.92537
Gglean000970	47.54864	39.491695	76.25540443	83.92117
Gglean000971	83.5874432	79.9718704	71.4381323	58.5865619
Gglean000972	28.33275	27.21697331	19.24133	20.38358978
Gglean000973	19.0692	25.288	6.2828	6.49911
Gglean000974	13.2825	17.1945	10.7425	12.5545
Gglean000975	10.68212364	8.9668436	10.08439302	10.43669066
Gglean000976	0	0	0	0
Gglean000977	3.357189428	3.486462058	3.701098	4.198791283
Gglean000978	74.57313	78.24672	105.07497	100.83962
Gglean000979	4.55102235	4.76550679	6.6299946	10.088966
Gglean000980	2.458086	1.891511806	3.542119	3.513016397
Gglean000981	3.41564	2.47587	2.1714	0.00865556
Gglean000982	14.8471	13.8696	12.6358	11.1786
Gglean000983	5.01479	4.97166	3.88898	3.12591
Gglean000984	0	0	0.0815588	0
Gglean000985	25.61740494	27.309543	34.307976	45.24389
Gglean000986	2.80021	2.62297	2.3195	1.34315
Gglean000987	130.1271	149.7797	106.4286	109.1541
Gglean000988	68.82682	66.35437025	60.50493	58.20522
Gglean000989	28.9064	32.5235	29.90108	31.62411
Gglean000990	28.9064	32.5235	29.90108	31.62411
Gglean000991	0	0	0	0
Gglean000992	0	0	0	0
Gglean000993	0	0	0	0
Gglean000994	0	0	0	0
Gglean000995	12.2437	13.1783	13.6841	28.1522
Gglean000996	16.4308182	16.11155107	19.41192075	22.85939961
Gglean000997	0.178982	1.20E-38	0.156129	1.68E-41
Gglean000998	0	0	0	0
Gglean000999	0	0	0	0
Gglean001000	0.394918	0.164366	0.128321	0.0759504
Gglean001001	60.327572	72.55005386	58.77138	60.37771
Gglean001002	9.504827648	9.344266094	6.366841076	6.705198658
Gglean001003	0	0	0	0
Gglean001004	7.93841	12.77665	3.976419322	3.713956938
Gglean001005	1642.025	1647.216	1065.447	1019.919
Gglean001006	20.532859	26.14757215	38.54452099	48.31697128
Gglean001007	0.327651	1.03227	0.54008	0.48283
Gglean001008	10.72268	13.84614	10.86498	15.87454
Gglean001009	50.601214	46.40004412	40.99125107	35.63140201
Gglean001010	50.601214	46.40004412	40.99125107	35.63140201
Gglean001011	50.601214	46.40004412	40.99125107	35.63140201

Gglean001012	91.889	92.2401	65.3151	55.2186
Gglean001013	261.4598803	272.0344351	97.0162984	75.2160732
Gglean001014	9.12126	12.2497	4.705166	7.21557
Gglean001015	4.680634511	5.593106778	5.533645801	7.589590218
Gglean001016	0	5.09E-07	8.75E-08	6.42E-06
Gglean001017	5.40633243	4.686701612	6.6226041	8.178586502
Gglean001018	19.9066	17.4686	8.4876	8.96544
Gglean001019	5.40633243	4.686701612	6.6226041	8.178586502
Gglean001020	0	0	0	0.287492
Gglean001021	0.119857	0.442088	0.0976442	0.231157
Gglean001022	18.87944	18.266841	15.4745	13.11262289
Gglean001023	32.62146	35.207403	22.95099	18.256711
Gglean001024	32.62146	35.207403	22.95099	18.256711
Gglean001025	51.95410017	55.12823842	57.76270638	57.42133113
Gglean001026	1.33548578	2.159774342	3.024658452	3.767003
Gglean001027	44.134137	46.05309	56.9316	51.23444
Gglean001028	44.134137	46.05309	56.9316	51.23444
Gglean001029	14.85911673	16.28602445	22.36802748	25.05581443
Gglean001030	5.20E-172	0.0752593	0.0213027	0.0152417
Gglean001031	5.20E-172	0.0752593	0.0213027	0.0152417
Gglean001032	1.839284	1.469728476	3.516793	3.529377866
Gglean001033	605.143	692.24	678.788	1238.696
Gglean001034	36.61172	33.80692	51.2355	57.75286
Gglean001035	5.457900012	3.331397	5.284792	7.373800006
Gglean001036	17.39875	14.55363	10.28942	10.62736
Gglean001037	8.862044	14.30805814	1.716924967	1.224559272
Gglean001038	40.58660101	31.14441174	31.7584003	32.60420883
Gglean001039	24.65841	24.8206078	18.669965	17.454987
Gglean001040	45.50701825	51.77878	39.76657	40.2688152
Gglean001041	3.34184	4.21097	5.04213	6.9797
Gglean001042	295.968	333.0145	412.15404	533.1004
Gglean001043	37.07766	40.46514	32.40977	29.74164
Gglean001044	15.0326	17.7845	8.82466	8.2457
Gglean001045	5.22E-09	7.03E-08	2.93E-08	6.05E-09
Gglean001046	42.20186929	36.22586741	24.65437321	27.627672
Gglean001047	18.04012318	14.11946661	10.53333218	11.86550662
Gglean001048	15.74516	15.97873	26.4867	27.6643
Gglean001049	11.66892	13.17713	8.29852	10.34499
Gglean001050	7.04897	5.5929	3.24999	3.12783
Gglean001051	16.56646406	18.31870509	21.89174857	22.89530132
Gglean001052	0	0	0	0
Gglean001053	16.56646406	18.31870509	21.89174857	22.89530132
Gglean001054	13.92026126	13.68162813	19.1859156	19.8182659
Gglean001055	13.92026126	13.68162813	19.1859156	19.8182659

Gglean001056	313.0549	326.86853	313.71422	371.31703
Gglean001057	12.81865	14.0268	13.6606	13.81125
Gglean001058	0	0	0	0
Gglean001059	74.11071	75.56023392	63.3513123	62.43340501
Gglean001060	74.11071	75.56023392	63.3513123	62.43340501
Gglean001061	74.11071	75.56023392	63.3513123	62.43340501
Gglean001062	22.20588	21.75719	39.1854	35.6981
Gglean001063	0	0	0.215454	0
Gglean001064	0	0	0	0
Gglean001065	0.399233145	0.776555266	0.537731172	0.679645008
Gglean001066	20.07167	23.24536	17.72755	18.60715
Gglean001067	0.210474	0.106523	0.0846726	0.0334452
Gglean001068	6.303422238	13.96949358	12.458383	17.59642223
Gglean001069	13.04279	15.40408	7.209252	5.75949
Gglean001070	12.16172	10.8529791	14.35972	17.60669
Gglean001071	7.38436	10.543	6.34693	4.28016
Gglean001072	14.6404	15.504	6.63748	5.92654
Gglean001073	0.553359	0	0	0
Gglean001074	0	0	0	0
Gglean001075	23.52291	22.97746	22.14149	21.48225
Gglean001076	59.7549	60.0497	53.3693	53.1761
Gglean001077	6.14214	7.6851	5.17242	6.25539
Gglean001078	18.94963989	16.03744874	16.59235855	18.52633905
Gglean001079	9.771335369	8.369634529	12.38743932	11.51552951
Gglean001080	9.771335369	8.369634529	12.38743932	11.51552951
Gglean001081	40.72699	41.42652	58.67303	75.3267
Gglean001082	115.26104	113.8169	132.7958	166.1162
Gglean001083	27.75769337	24.50369937	35.55281187	37.08622823
Gglean001084	27.75769337	24.50369937	35.55281187	37.08622823
Gglean001085	116.3355287	103.4210781	99.2667806	97.15748
Gglean001086	19.15056	20.404723	15.68232	16.9544
Gglean001087	57.81260014	50.32934	70.59948	78.10677
Gglean001088	29.2399	24.4954	30.7731	36.4827
Gglean001089	47.96331	48.97558001	55.62117	53.98494
Gglean001090	0	0	0	0
Gglean001091	14.80924	20.44539619	6.265156	6.03829241
Gglean001092	6.36093	8.59994	21.98262	18.78525
Gglean001093	10.40989	10.48711	5.47279	5.3047
Gglean001094	10.40989	10.48711	5.47279	5.3047
Gglean001095	23.00193	20.50577	13.72534	10.69253
Gglean001096	61.55800471	69.93007162	57.27507314	49.75489798
Gglean001097	61.55800471	69.93007162	57.27507314	49.75489798
Gglean001098	61.55800471	69.93007162	57.27507314	49.75489798
Gglean001099	30.2575	30.9863	63.7272	65.8506

Gglean001100	0	0	0	0
Gglean001101	41.3985	40.5278	62.701	72.2069
Gglean001102	0.018828732	0	0.035323289	0.012959401
Gglean001103	10.25903	9.217276541	4.37554	3.81753
Gglean001104	347.5466	323.5488	456.5061	706.2184
Gglean001105	52.403	61.14205412	82.27840005	93.5076
Gglean001106	25.474155	25.39087026	33.9626759	44.621378
Gglean001107	12.5658	13.5324	16.7839	21.1831
Gglean001108	0.892430943	0.864998616	1.020053693	1.331277223
Gglean001109	1.955670786	1.217259316	1.4126272	1.24309952
Gglean001110	0	0	0	0.606051
Gglean001111	33.44513296	34.23860272	52.50011162	57.11983286
Gglean001112	30.98676	34.20238	41.30667	41.46378
Gglean001113	30.98676	34.20238	41.30667	41.46378
Gglean001114	13.6832	13.6387	6.97956	5.72973
Gglean001115	78.9841061	73.7617234	65.5776752	63.2113288
Gglean001116	78.9841061	73.7617234	65.5776752	63.2113288
Gglean001117	78.9841061	73.7617234	65.5776752	63.2113288
Gglean001118	78.9841061	73.7617234	65.5776752	63.2113288
Gglean001119	30.39218	33.57113	32.29163	32.91951
Gglean001120	30.39218	33.57113	32.29163	32.91951
Gglean001121	15.51062265	16.63264627	19.33592414	25.00739855
Gglean001122	0	0	0	0
Gglean001123	0	0	0	0
Gglean001124	0.149464	0	0	0
Gglean001125	0	0	0	0
Gglean001126	0.0672597	0	0	0.0647041
Gglean001127	0.39677789	0.091730069	0	0.164449
Gglean001128	8.445539	4.9004839	12.340277	16.593506
Gglean001129	0.284613	0.131226	0.385997	0.33584
Gglean001130	0	0	0	0
Gglean001131	0	0	0	0
Gglean001132	0	0	0	0
Gglean001133	0.4330139	0.7029	0.30964	0.699527
Gglean001134	10.71277659	8.81674	11.1856196	17.72778608
Gglean001135	10.71277659	8.81674	11.1856196	17.72778608
Gglean001136	14.78755789	15.33378328	15.04438563	13.83037342
Gglean001137	3.315607897	3.956291733	5.042250146	4.857768225
Gglean001138	0.160939	0.120637	0.460096	0.250316
Gglean001139	30.499819	27.753559	18.687832	16.467036
Gglean001140	26.97644568	27.3057	18.851953	12.657868
Gglean001141	26.97644568	27.3057	18.851953	12.657868
Gglean001142	4.52621	6.72645	1.46382	0.582253
Gglean001143	0	0	0	0

Gglean001144	0	0	0	0
Gglean001145	0.64147	0	0.531005	0
Gglean001146	15.717983	14.368793	12.814526	12.90894
Gglean001147	40.72499	38.03942	45.11538	49.29836
Gglean001148	40.72499	38.03942	45.11538	49.29836
Gglean001149	28.72523	31.180558	17.05918006	14.1439662
Gglean001150	0	0	0	0
Gglean001151	0	0	0	0
Gglean001152	0.0429955	3.27E-08	0.293558036	6.11E-08
Gglean001153	7.440261126	8.4029475	12.06908728	13.70776
Gglean001154	20.6770239	21.50394289	21.66522538	29.02926
Gglean001155	20.80591	17.48743	21.16429	23.40996
Gglean001156	20.80591	17.48743	21.16429	23.40996
Gglean001157	19.5889	20.6992	18.8735	19.4267
Gglean001158	19.21287892	23.71526	15.840939	14.715152
Gglean001159	177.4152	191.4687971	154.069478	203.7328591
Gglean001160	5.713282071	6.750846	4.126554	3.9794833
Gglean001161	133.307	129.6	91.8845	101.771
Gglean001162	58.90161329	53.61436991	46.56429638	72.08444218
Gglean001163	56.816298	56.37706002	93.642622	106.271951
Gglean001164	0	0.153832	0.162302	0
Gglean001165	0	0	0	0
Gglean001166	0	0	0	0
Gglean001167	157.7437694	204.129842	180.131411	164.9473701
Gglean001168	51.59707	83.59659	79.66997	120.66899
Gglean001169	33.0496	36.2622	28.4944	26.2956
Gglean001170	26.883367	32.18109	23.03915	20.98678231
Gglean001171	13.10896	13.20609	12.81638	14.46143
Gglean001172	33.01474722	34.58635657	21.39691488	20.24674927
Gglean001173	31.203799	31.48842	30.17682	31.81308
Gglean001174	0	0.155246	0	0
Gglean001175	42.45296	43.68448	33.87515	31.67287
Gglean001176	40.61060107	42.87611531	58.32520359	54.77600573
Gglean001177	0	0.0353235	0.056044	0
Gglean001178	0	0	0.059884	0.142075
Gglean001179	0	0	0	0
Gglean001180	0	0	0	0
Gglean001181	14.70678067	14.91124	11.55164483	9.8603841
Gglean001182	13.423269	17.33462	14.48936	14.31958
Gglean001183	0.381173	0	0.0772521	0.365782
Gglean001184	17.258951	19.078344	16.2375	19.962532
Gglean001185	72.1233	64.45993	73.0146	71.4991
Gglean001186	21.7264778	22.09842098	15.25372925	13.56633384
Gglean001187	0.339531907	0.21539805	0.520204726	0.424052145

Gglean001188	1.331684057	0.9574	2.078205145	2.316374
Gglean001189	0.457474328	0.349450634	0.640469832	0.728852164
Gglean001190	0	0	0	0
Gglean001191	0	0	0	0
Gglean001192	57.6004	53.8231	69.326	66.8575
Gglean001193	21.64988	23.6311	26.5748	32.8062
Gglean001194	43.6968	50.4085	42.8907	41.5487
Gglean001195	0.000307272	0.098548	1.06119	0.000453914
Gglean001196	2.284252671	4.42577745	4.13127	6.287054164
Gglean001197	32.3424	26.3367	24.995	26.3043
Gglean001198	2.018476445	2.933278776	2.679675	2.924836131
Gglean001199	29.32541465	31.43240007	39.14164712	56.59188068
Gglean001200	29.32541465	31.43240007	39.14164712	56.59188068
Gglean001201	0.371623	0.331637	0.17215	0.305482
Gglean001202	28.46161613	26.11377501	38.58986023	30.70904593
Gglean001203	28.46161613	26.11377501	38.58986023	30.70904593
Gglean001204	60.0885	56.1732	65.1807	61.1819
Gglean001205	9.79732	9.56966	16.81018	17.07645
Gglean001206	0	0.05135	0.040232	0.239382947
Gglean001207	2.726634556	3.119210257	3.254720842	3.11940552
Gglean001208	0.695074	0.4337	0.562748	0.811908
Gglean001209	0	0	0	0
Gglean001210	0.086870979	0.0450253	0.105792068	0.083415713
Gglean001211	0.06754808	0.0345975	0	0.129656407
Gglean001212	0.258804309	0.358050146	0.175463464	0.408796569
Gglean001213	82.0718733	62.85237539	80.18098451	76.34812975
Gglean001214	82.0718733	62.85237539	80.18098451	76.34812975
Gglean001215	82.0718733	62.85237539	80.18098451	76.34812975
Gglean001216	19.2346	19.1191	14.8484	13.9512
Gglean001217	1.34057	2.98869	0.316087	0.620027
Gglean001218	28.948079	27.323878	23.64520818	28.203324
Gglean001219	28.948079	27.323878	23.64520818	28.203324
Gglean001220	28.948079	27.323878	23.64520818	28.203324
Gglean001221	78.10215481	81.50974317	110.78531	124.672244
Gglean001222	0.322924	0.111897	0.350278	0.60093606
Gglean001223	8.357226	8.412052	7.85508	8.46256
Gglean001224	5.72091	5.228040001	9.03189	8.344233
Gglean001225	5.72091	5.228040001	9.03189	8.344233
Gglean001226	1.042217	3.209472	4.366681	4.90084
Gglean001227	5.19E-05	2.84E-09	1.50E-05	0.993238
Gglean001228	8.4722835	7.329567	5.042890605	4.954787358
Gglean001229	5.59260859	5.4916962	6.704684	8.42961881
Gglean001230	19.923209	14.91223	11.915875	10.675036
Gglean001231	0.394101	0.428572	0.656046	0.392622

Gglean001232	0.57124575	0.406969933	0.698572016	0.378994323
Gglean001233	11.49001	14.18238	10.73068	10.792352
Gglean001234	0	0	0	0
Gglean001235	23.07746278	21.0939092	28.06741391	25.23798992
Gglean001236	3.24142	3.55956	4.09921	3.13572
Gglean001237	0	0	0	0
Gglean001238	8.36177	4.16696	7.35095	9.32624
Gglean001239	0.22711	2.653313	0	2.227546
Gglean001240	63.0477	77.9762	43.41644	35.15308
Gglean001241	19.88694714	17.59905756	21.9222669	32.79565141
Gglean001242	19.88694714	17.59905756	21.9222669	32.79565141
Gglean001243	0.0366395	0	0.0297299	0
Gglean001244	0	0	0	0
Gglean001245	342.1417841	366.6591081	227.0791894	195.2336307
Gglean001246	0	0	0	0
Gglean001247	4.542874	4.758261584	6.691057	7.056503
Gglean001248	8.052381	7.065386089	11.301435	14.15794
Gglean001249	141.0499697	126.902019	222.7549927	223.040443
Gglean001250	141.0499697	126.902019	222.7549927	223.040443
Gglean001251	27.83349051	29.97128603	39.22142766	43.22439024
Gglean001252	0	0	0	0
Gglean001253	0	0	0.0201112	0
Gglean001254	59.876101	58.42157771	36.668325	37.47446
Gglean001255	59.876101	58.42157771	36.668325	37.47446
Gglean001256	59.876101	58.42157771	36.668325	37.47446
Gglean001257	59.876101	58.42157771	36.668325	37.47446
Gglean001258	25.689549	30.20385	18.97489	17.484326
Gglean001259	0.279920038	0.192779384	0.5274991	0.553129608
Gglean001260	0.279920038	0.192779384	0.5274991	0.553129608
Gglean001261	12.546138	17.729031	22.75006	24.85048019
Gglean001262	6.457131192	8.17654273	6.911882304	7.0076805
Gglean001263	3377.6959	3555.6759	2120.5213	1951.0166
Gglean001264	10.50889659	13.896275	8.408782223	9.913074079
Gglean001265	7.634706211	8.797090089	6.486909	6.5413211
Gglean001266	52.6612	43.0581	43.7486	42.9063
Gglean001267	89.4233	94.0525	95.22737	87.03706
Gglean001268	0	0	0.268203	0
Gglean001269	5.627451	5.479411054	5.967517	7.007380089
Gglean001270	5.627451	5.479411054	5.967517	7.007380089
Gglean001271	61.78558938	56.121341	42.10681	40.43745948
Gglean001272	28.9695	31.0292	4.74958	7.70044
Gglean001273	61.78558938	56.121341	42.10681	40.43745948
Gglean001274	1450.5042	1545.118652	918.3926	865.7052
Gglean001275	23.36047	22.76877	13.52606	12.14718

Gglean001276	5.48E-302	0.274119	0.641652	0
Gglean001277	1.91631	3.11E-05	0.656387	1.848
Gglean001278	1.002768	1.365586	1.276997	0.922332
Gglean001279	23.43634	25.07723808	12.96114414	12.20039918
Gglean001280	23.43634	25.07723808	12.96114414	12.20039918
Gglean001281	0	0	0	0
Gglean001282	0.00045705	2.64414	1.62313	4.98585
Gglean001283	76.21297	70.98324002	95.49428	81.19694
Gglean001284	76.21297	70.98324002	95.49428	81.19694
Gglean001285	76.21297	70.98324002	95.49428	81.19694
Gglean001286	0	0.149296	0.572849	0
Gglean001287	0.5566319	0.524160895	0.170727124	0.322841523
Gglean001288	0.235583128	0.1203134	0.2652948	0.262075578
Gglean001289	0.139483	0.289658	0.379146	0.447954
Gglean001290	15.2587637	14.868258	14.635315	18.568728
Gglean001291	38.26728	34.18681	34.12353	34.3553718
Gglean001292	25.9911	22.14988	12.68998	8.09598
Gglean001293	23.100209	26.56643419	25.37049	26.02494991
Gglean001294	28.31282598	29.34034174	15.81796239	14.11125045
Gglean001295	28.31282598	29.34034174	15.81796239	14.11125045
Gglean001296	28.31282598	29.34034174	15.81796239	14.11125045
Gglean001297	12.5021	12.585	11.4216	11.0675
Gglean001298	10.4678	10.6582	8.79399	8.12147
Gglean001299	86.31486	82.27991	64.55623	64.74922
Gglean001300	86.31486	82.27991	64.55623	64.74922
Gglean001301	68.25187785	70.91267371	93.57004505	98.19801913
Gglean001302	68.25187785	70.91267371	93.57004505	98.19801913
Gglean001303	31.6911	35.2025	27.3178	29.171
Gglean001304	15.95086021	11.83900772	16.55598463	15.8246953
Gglean001305	14.878226	15.201518	22.170737	23.661508
Gglean001306	10.92631	13.6984537	18.986478	21.85774
Gglean001307	1.224113739	0.3527313	0.072805647	0.221939507
Gglean001308	83.22850558	83.06380937	93.9051	82.2638
Gglean001309	83.22850558	83.06380937	93.9051	82.2638
Gglean001310	10.49432	13.12655	13.45066	17.63963
Gglean001311	10.49432	13.12655	13.45066	17.63963
Gglean001312	44.0013	35.8227	41.5161	43.2369
Gglean001313	0	0	0	0
Gglean001314	23.12232876	21.55325966	20.89430418	18.93246503
Gglean001315	23.12232876	21.55325966	20.89430418	18.93246503
Gglean001316	8.406632404	6.286469	6.979966414	10.18585317
Gglean001317	0	0	0	0.0844477
Gglean001318	4.832996004	6.2152985	8.646156235	6.717663825
Gglean001319	4.832996004	6.2152985	8.646156235	6.717663825

Gglean001320	4.832996004	6.2152985	8.646156235	6.717663825
Gglean001321	0.537692	0.464616	0.990438	0.956705
Gglean001322	11.64854993	11.89737615	16.90441237	19.03869048
Gglean001323	15.0229	14.5164	12.6733	13.3047
Gglean001324	11.804555	11.698958	14.958469	17.065387
Gglean001325	36.69484	32.88213	22.42358	23.59849
Gglean001326	36.69484	32.88213	22.42358	23.59849
Gglean001327	0	0	0	0
Gglean001328	0	0	0	0
Gglean001329	7.36441676	7.979334401	10.122015	9.887538
Gglean001330	39.51714	39.0438	15.94908	10.52464
Gglean001331	25.90594498	22.08937986	12.0050496	10.86021648
Gglean001332	25.90594498	22.08937986	12.0050496	10.86021648
Gglean001333	12.39052806	14.1437266	19.950233	19.09230914
Gglean001334	12.39052806	14.1437266	19.950233	19.09230914
Gglean001335	0	0	0	0
Gglean001336	45.443406	49.54276048	41.20768	42.02474638
Gglean001337	31.485487	24.880584	48.43926	52.86621
Gglean001338	31.485487	24.880584	48.43926	52.86621
Gglean001339	31.485487	24.880584	48.43926	52.86621
Gglean001340	0	0	1.99948	0
Gglean001341	77.91033733	81.22228287	104.7280538	102.246426
Gglean001342	0	0	0	0
Gglean001343	4.102688441	3.228512	6.868608787	4.38395485
Gglean001344	4.102688441	3.228512	6.868608787	4.38395485
Gglean001345	8.09665	13.81726	13.30586	12.89776
Gglean001346	0.261830451	0.4385142	0.521682014	0.54539035
Gglean001347	5.12E-06	1.63E-05	2.74E-05	2.06E-05
Gglean001348	0.55967	0.286822	0.884104	0.502256121
Gglean001349	18.057	19.85417	16.27973	15.43114
Gglean001350	0	0	0	0
Gglean001351	95.3943	109.1797	36.7999	24.08811
Gglean001352	0.637973	0.656872	0.0643289	0.0763116
Gglean001353	5.528566	6.204071	1.529322	2.856139
Gglean001354	0	0	0	0
Gglean001355	0.135461	0.0711557	0.0554711	0
Gglean001356	0	0	0.0724558	0
Gglean001357	0.051385	0.0531214	0.125274	0.123489
Gglean001358	0.0373714	0.0781611	0.030357	0
Gglean001359	0.0605345	0	0	0
Gglean001360	0	0	0.0868427	0.0684027
Gglean001361	0.037382	0	0	0
Gglean001362	0.0344163	0.0355733	0	0
Gglean001363	13.12322	12.05273	11.6682	10.36901

Gglean001364	10.1279	6.14965	10.9659	15.9289
Gglean001365	24.903116	23.637636	19.563225	15.2516
Gglean001366	18.4715	18.321	13.494	16.0587
Gglean001367	19.91234	21.70312	21.58264	20.77543
Gglean001368	16.047075	17.097892	25.609102	29.747359
Gglean001369	11.64854993	11.89737615	16.90441237	19.03869048
Gglean001370	41.18919114	43.93103926	72.050527	74.208424
Gglean001371	15.98240107	16.10400388	25.848439	30.69701
Gglean001372	0	0	0	0
Gglean001373	0.886535004	0.71416423	0.836850064	1.413974414
Gglean001374	4.453763608	5.125289	5.575877269	5.052704197
Gglean001375	1.403322	0.7702865	1.04492	0.94171
Gglean001376	10.82886	15.59744	17.65671	22.90445
Gglean001377	5.554451242	5.52823374	8.069464783	9.24754166
Gglean001378	101.4614	95.1097	68.9769	72.8544
Gglean001379	0	0	0	0
Gglean001380	0	0	0	0
Gglean001381	21.76395	21.11057	19.989169	19.09267035
Gglean001382	4.00272	3.32873	4.193142178	7.00677
Gglean001383	15.07894	16.53199	11.26307	10.35197
Gglean001384	55.38567	25.17836995	69.57236421	104.2551307
Gglean001385	6.22781	6.33966	4.92511	5.30303
Gglean001386	35.64676	36.5182	24.27122	22.10861
Gglean001387	3.39782	4.69191	4.130433	5.3975
Gglean001388	14.351944	16.6963	10.79114695	9.699901057
Gglean001389	186.5181772	199.9768209	220.944689	192.672004
Gglean001390	186.5181772	199.9768209	220.944689	192.672004
Gglean001391	186.5181772	199.9768209	220.944689	192.672004
Gglean001392	186.5181772	199.9768209	220.944689	192.672004
Gglean001393	53.541838	55.419742	49.736008	49.616648
Gglean001394	53.541838	55.419742	49.736008	49.616648
Gglean001395	1.11739	1.32129	1.638	2.58761
Gglean001396	0	0	0	0
Gglean001397	12.39027	11.46436	17.89157	22.95624
Gglean001398	63.10449	69.18546	42.11379	45.72956228
Gglean001399	63.10449	69.18546	42.11379	45.72956228
Gglean001400	189.988	180.001	196.817	223.333
Gglean001401	120.528789	125.1096089	106.9434861	94.52436326
Gglean001402	29.896474	34.56005	23.730486	22.34871713
Gglean001403	57.77065403	58.27148797	57.08488663	61.38892002
Gglean001404	0.131676	0.234549	0.398286	0.378208
Gglean001405	25.92788	27.64103	36.919795	39.8564405
Gglean001406	11.64394331	12.06857611	9.975516031	8.681233753
Gglean001407	11.64394331	12.06857611	9.975516031	8.681233753

Gglean001408	31.7793	33.4644	23.9013	21.0337
Gglean001409	22.4753939	25.21571061	27.2540398	29.30803106
Gglean001410	5.188504	5.194862	5.917788	6.546493
Gglean001411	5.188504	5.194862	5.917788	6.546493
Gglean001412	65.8879	65.5125	40.29132601	32.735725
Gglean001413	65.8879	65.5125	40.29132601	32.735725
Gglean001414	0.3169543	0.5825	0.425087785	0.650162
Gglean001415	6.012346036	6.21962	4.971468264	5.129259761
Gglean001416	15.67512	15.9344	21.386	26.97001
Gglean001417	0	0.2295	0.0441042	0.361003
Gglean001418	0.732462	1.02586	0.520476	0.641586
Gglean001419	0	0	0	0
Gglean001420	0	0.0871228	0.136577	0.202101
Gglean001421	3.66E-05	1.30E-06	4.01E-06	0.218999
Gglean001422	31.15328	32.37641	50.44669	53.99277
Gglean001423	22.09778948	23.79701	18.47978	20.04478484
Gglean001424	14.90466396	35.07754518	18.38706	25.727088
Gglean001425	245.7063	219.6645	169.9805	180.2365
Gglean001426	17.4353	22.5778	3.8265	7.16711
Gglean001427	6.842873	7.66507	7.64173	6.64951
Gglean001428	16.6745	18.4249	26.546	31.9233
Gglean001429	0	0.0597497	0	0
Gglean001430	15.76224	16.49527	11.31917	9.903647
Gglean001431	28.87190375	32.8433083	44.75750604	43.71000453
Gglean001432	52.6366195	58.02193465	48.49708725	43.85302552
Gglean001433	3.89E-05	2.07E-05	5.57E-05	7.69E-05
Gglean001434	0	0	0	0
Gglean001435	12.29859	12.7461	24.30062	25.31977
Gglean001436	0	0	0	0
Gglean001437	0	0	0	0
Gglean001438	20.6239331	21.321055	15.249628	14.833757
Gglean001439	20.6239331	21.321055	15.249628	14.833757
Gglean001440	61.2149857	59.02004505	49.50531819	54.33658998
Gglean001441	61.2149857	59.02004505	49.50531819	54.33658998
Gglean001442	0.058310113	0	0	0
Gglean001443	0	0	0	0
Gglean001444	1.07413	0.675596	0.334088	0.282206
Gglean001445	0	0	0	0
Gglean001446	0	0	0.146194	0
Gglean001447	0	0	0	0
Gglean001448	33.4244	30.426859	25.75922	25.20776691
Gglean001449	6.11919	7.62925	7.80685	8.403084
Gglean001450	9.58987	6.61487	5.36273	4.52348
Gglean001451	143.2098	180.2029	166.2954	187.2068

Gglean001452	7.990018831	5.729441929	10.89485891	14.413
Gglean001453	7.990018831	5.729441929	10.89485891	14.413
Gglean001454	8.492444712	7.378364964	5.903433	6.86379696
Gglean001455	0.3578	0.366162	0.482388	0.931958
Gglean001456	0.994614	0.561393	0.268893	1.06619
Gglean001457	0	0.110386	0.088325	0.105312
Gglean001458	0	0	0	0
Gglean001459	0	0	0	0
Gglean001460	1.16808	1.80413	1.07343	1.1358
Gglean001461	1.04882	0.805594	1.38993	1.27203
Gglean001462	1.5046	1.10015	0.789135	0.93846
Gglean001463	13.61862581	13.48835058	16.4336204	18.267892
Gglean001464	0.0891661	0	0	0
Gglean001465	10.455737	13.01071345	7.19881654	6.039989002
Gglean001466	10.455737	13.01071345	7.19881654	6.039989002
Gglean001467	10.455737	13.01071345	7.19881654	6.039989002
Gglean001468	0	0	0	0
Gglean001469	31.971007	29.7425385	29.11368744	25.87667605
Gglean001470	31.971007	29.7425385	29.11368744	25.87667605
Gglean001471	36.50294678	34.71376449	61.21387369	67.33168157
Gglean001472	30.3876	31.3504	25.97113	24.3074
Gglean001473	15.16378	15.1302	14.42872	12.86279
Gglean001474	48.28052017	51.3421	29.52553	27.539264
Gglean001475	0	0.043794834	0.149019418	0.125392188
Gglean001476	144.995368	162.94958	135.137601	132.9501015
Gglean001477	12.44323957	14.07095133	6.72986597	6.207693705
Gglean001478	51.3165	57.1287	52.5171	54.4157
Gglean001479	14.5232	18.0191	18.3551	12.1262
Gglean001480	25.1896	19.7401	24.9845	27.6613
Gglean001481	86.6394	102.716	41.2714	42.4119
Gglean001482	3.594061681	4.149730393	4.34659	4.44065831
Gglean001483	4.66827628	7.100392227	3.841261	4.878111435
Gglean001484	0	0	0	0
Gglean001485	0	0	0	0
Gglean001486	0	0	0	0
Gglean001487	12.76012	13.61498	17.36336	25.78746
Gglean001488	12.76012	13.61498	17.36336	25.78746
Gglean001489	0	0	0.0733531	0.087311
Gglean001490	6.59372086	6.271900528	8.69765234	8.79667132
Gglean001491	6.59372086	6.271900528	8.69765234	8.79667132
Gglean001492	40.86327	57.2933	50.54232	82.2814
Gglean001493	8.1375796	8.04087	9.286220911	10.557819
Gglean001494	9.758757256	8.59412098	9.338831092	10.41949677
Gglean001495	10.187846	10.378087	2.402811	2.228468194

Gglean001496	10.187846	10.378087	2.402811	2.228468194
Gglean001497	10.187846	10.378087	2.402811	2.228468194
Gglean001498	3.60623	3.38967	8.6307	7.27784
Gglean001499	53.97707283	61.20116377	56.79213055	63.19115117
Gglean001500	53.97707283	61.20116377	56.79213055	63.19115117
Gglean001501	45.2621	40.6999	48.3527	47.069
Gglean001502	55.03149	62.53163	73.77688	77.47133
Gglean001503	55.03149	62.53163	73.77688	77.47133
Gglean001504	0.0897833	0.000272591	0.149204	0
Gglean001505	32.95438	31.73103	26.39526	23.30448737
Gglean001506	59.686584	61.4092	61.30683	58.21235
Gglean001507	35.21370245	34.466154	55.5537367	46.1813262
Gglean001508	43.52328	41.36606	14.112634	12.32973
Gglean001509	27.43991	27.1633	24.66789	24.62165
Gglean001510	19.37147	19.71324	25.03672	29.58872
Gglean001511	22.10628	24.9103	15.56133	14.5543
Gglean001512	17.42833218	16.56072516	22.01660176	31.21451427
Gglean001513	17.42833218	16.56072516	22.01660176	31.21451427
Gglean001514	14.50145003	14.88566814	20.30776239	25.522272
Gglean001515	0	0	0	0
Gglean001516	0	0	0	0
Gglean001517	0	0	0	0
Gglean001518	0	0	0	0
Gglean001519	382.7478974	364.738655	374.2575469	447.2860394
Gglean001520	1.605949124	1.854289847	2.605487218	2.389212188
Gglean001521	0	0.038415408	0	0.016451728
Gglean001522	0.03030833	0	0	0.018344841
Gglean001523	45.77737	49.38615	51.62855	48.77025
Gglean001524	45.77737	49.38615	51.62855	48.77025
Gglean001525	0	0	0	0
Gglean001526	0	0	0	0
Gglean001527	31.02304	28.997263	19.61593	19.37565
Gglean001528	0	0	0	0
Gglean001529	99.58609725	105.3900801	53.92648083	49.02552
Gglean001530	99.58609725	105.3900801	53.92648083	49.02552
Gglean001531	4.876703	3.365219	3.021238	1.712005
Gglean001532	1.078404467	1.120560068	1.50768091	1.268719997
Gglean001533	21.98073	22.02411	10.948723	15.106968
Gglean001534	46.98836994	45.33697	49.50801	72.45574
Gglean001535	0.0863272	0	0	0
Gglean001536	0	0	0.102832	0.121824
Gglean001537	0	0	0	0.2129491
Gglean001538	15.7605	14.7138	14.2387	16.7369
Gglean001539	0	0	0	0

Gglean001540	17.23699	20.20258	23.467	28.5027
Gglean001541	66.7793452	64.68104941	64.68243648	69.49895
Gglean001542	29.992	29.8391	21.85377	19.30952
Gglean001543	7.641257563	3.830484654	1.875166	1.58487
Gglean001544	0.959853	1.234724	0.5957517	0.3187614
Gglean001545	177.215	196.825	185.2	198.799
Gglean001546	17.84211087	23.84043	21.01455012	21.787825
Gglean001547	17.84211087	23.84043	21.01455012	21.787825
Gglean001548	3.38577	3.802081	4.015496	3.727979
Gglean001549	0.527505	0.4598915	1.143455	0.49064971
Gglean001550	18.053315	19.03866178	17.15413333	15.410367
Gglean001551	4.013863361	4.477468	1.7341284	1.071477039
Gglean001552	0.155014989	0.536965697	0.652899149	0.336219037
Gglean001553	3.20315	0.00913836	7.73001	0.0837249
Gglean001554	0.190491	0.342821	0.620887	0.184225
Gglean001555	0	0	0	0
Gglean001556	18.18756054	17.47106296	21.14997936	30.51343104
Gglean001557	18.18756054	17.47106296	21.14997936	30.51343104
Gglean001558	23.021365	25.85056135	13.84780548	10.667272
Gglean001559	0.131432	0	0	0
Gglean001560	0	0.0594056	0	0
Gglean001561	11.369	12.4576	4.04776	4.15557
Gglean001562	1.23679	1.00554	1.5086	1.3185
Gglean001563	24.70819989	27.441543	43.03920692	47.18011008
Gglean001564	0	0	0	0
Gglean001565	64.6353	62.4276	54.2333	59.4176
Gglean001566	18.6477	16.648	20.7904	22.7771
Gglean001567	0.4126688	0.3285549	0.411861	0.488806
Gglean001568	0.234508189	0.079859376	0.315330807	0.223753877
Gglean001569	16.63949	16.248897	17.921987	17.192411
Gglean001570	107.6642491	111.3598423	97.68882214	102.0458307
Gglean001571	43.23142235	51.0874088	23.20833415	25.8230443
Gglean001572	43.23142235	51.0874088	23.20833415	25.8230443
Gglean001573	55.845448	59.489754	73.58407598	64.8680674
Gglean001574	55.845448	59.489754	73.58407598	64.8680674
Gglean001575	4.0998	3.81074	6.51068	9.00037
Gglean001576	0.132784	0	0.107801	0.0637343
Gglean001577	73.21688	64.61727	94.32673	104.62187
Gglean001578	20.51035	17.43004	19.64164	24.06421
Gglean001579	20.39698	23.73073	12.69934	11.928744
Gglean001580	20.39698	23.73073	12.69934	11.928744
Gglean001581	8.513052386	9.236087651	11.912983	12.21308565
Gglean001582	0	0	0	0
Gglean001583	1.90333	2.15571	3.16171	1.89513

Gglean001584	3.73541	3.38257	1.737051	1.374212
Gglean001585	0	0	0	0
Gglean001586	0.111454638	0.019954677	0.051771858	0
Gglean001587	42.65000949	43.1886178	70.32940787	79.75101297
Gglean001588	42.65000949	43.1886178	70.32940787	79.75101297
Gglean001589	0	0	0	0
Gglean001590	0	0	0	0
Gglean001591	0	0	0.285313	0
Gglean001592	0	0.199027	0	0
Gglean001593	0.0739006	0	0	0
Gglean001594	0.12604	0.260851	0	0
Gglean001595	7.24445	7.35217	6.36918	7.25004
Gglean001596	18.3077148	18.66795589	23.7253668	26.643605
Gglean001597	18.3077148	18.66795589	23.7253668	26.643605
Gglean001598	23.46033211	24.86999	4.92417	3.83198
Gglean001599	17.8073	18.9289	19.3507	18.5806
Gglean001600	11.36800997	12.63990533	7.108746	6.844538605
Gglean001601	4.438338	4.851161	2.999961	2.110899
Gglean001602	26.0314	26.105	25.1666	26.0576
Gglean001603	37.1936676	40.1795638	21.55222	19.91964228
Gglean001604	30.10311177	28.59824604	30.48616612	29.94191734
Gglean001605	30.10311177	28.59824604	30.48616612	29.94191734
Gglean001606	17.89729	16.608991	44.63631402	48.12697807
Gglean001607	90.47020136	80.844276	22.334745	7.8417565
Gglean001608	1.768368564	1.462941989	1.966411399	2.600975134
Gglean001609	0	0	0	0
Gglean001610	0	0	0	0
Gglean001611	13.8109	10.4525	20.8025	26.073
Gglean001612	19.25991	18.361114	9.220294	9.962124
Gglean001613	271.9134	246.2507	262.557	247.6691
Gglean001614	0	0	0	0.502477
Gglean001615	95.20442619	93.68150004	54.72911523	61.90251383
Gglean001616	0.262611	0.196905	8.86E-06	0.116938
Gglean001617	86.9186	93.9505	95.4968	90.1932
Gglean001618	4.913242	6.559398	6.653657	7.73355
Gglean001619	59.7666166	70.72852	54.892858	50.422817
Gglean001620	0.105127	0.321028116	0.253491054	0.302145
Gglean001621	101.7367195	112.387591	188.952	220.77619
Gglean001622	70.68088419	68.44763	113.2885822	122.3318422
Gglean001623	37.6188	38.852	31.4073	32.7531
Gglean001624	11.9809	12.5014	8.04768	10.788
Gglean001625	8.450724538	12.65618	4.147773691	4.21115
Gglean001626	0.840292889	0.874147478	0.568203	0.570413
Gglean001627	0	0	0	0

Gglean001628	482.160119	448.900362	540.455866	818.1739442
Gglean001629	0.268823	0.359951	0.6058015	0.3208453
Gglean001630	21.48222	17.04532397	24.29214	19.54158
Gglean001631	14.712621	16.742281	9.622701	9.627570452
Gglean001632	19.02572	20.28524	13.09154	13.12466
Gglean001633	12.87660201	11.67360503	6.472431655	4.56818502
Gglean001634	12.87660201	11.67360503	6.472431655	4.56818502
Gglean001635	76.5771	81.1256	38.65017	27.09521
Gglean001636	20.95814998	23.72352076	16.481092	17.83222435
Gglean001637	37.334501	41.909352	42.004643	45.052029
Gglean001638	17.947568	16.6248441	14.17512	14.673021
Gglean001639	25.67167454	28.67072945	18.34948937	16.13370751
Gglean001640	1.764880484	2.06821	1.622690052	1.073022161
Gglean001641	39.67101456	44.36315003	27.69283799	24.49551731
Gglean001642	39.67101456	44.36315003	27.69283799	24.49551731
Gglean001643	5.194019	7.21787	9.63657	11.61081
Gglean001644	5.194019	7.21787	9.63657	11.61081
Gglean001645	15.64433272	14.45040509	10.666037	9.757438501
Gglean001646	0	0.0319841	0	0.0297744
Gglean001647	0	0	0	0
Gglean001648	5.148053	6.260888	6.2964749	7.653768
Gglean001649	13.540605	14.9993885	23.02026313	25.57933261
Gglean001650	22.72642001	23.61598963	10.79331788	9.505212
Gglean001651	0.248914	0.128558	0.100776	0
Gglean001652	12.908625	13.31118	24.29752	23.9332
Gglean001653	3.5189131	4.312984	6.936285	7.458494
Gglean001654	4.15874	0.000165192	5.21897	0.047275
Gglean001655	0	0	0	0
Gglean001656	28.359686	27.388513	39.349997	44.165634
Gglean001657	80.52692	93.32434	57.682625	54.46495
Gglean001658	80.52692	93.32434	57.682625	54.46495
Gglean001659	0	0.0743799	0	0.0495631
Gglean001660	10.58509345	9.331709	6.5211423	6.264245
Gglean001661	0.047671497	0	0.077389041	0
Gglean001662	0.047671497	0	0.077389041	0
Gglean001663	0	0	0	0
Gglean001664	13.81316	12.0081	12.33604392	18.13820656
Gglean001665	67.3013	73.4917	48.1426	37.63793
Gglean001666	67.3013	73.4917	48.1426	37.63793
Gglean001667	89.49315	85.85528811	89.51431387	79.95060064
Gglean001668	89.49315	85.85528811	89.51431387	79.95060064
Gglean001669	36.162186	36.314479	23.87968719	19.299998
Gglean001670	67.632841	75.9564866	89.1341498	116.6031021
Gglean001671	0.589590916	0.0666209	0.696163221	1.392777

Gglean001672	0.415077	0.536169	0	0
Gglean001673	5.50949873	5.676388327	7.545465409	8.07152975
Gglean001674	76.78796472	59.05081546	98.68701189	118.2607386
Gglean001675	76.78796472	59.05081546	98.68701189	118.2607386
Gglean001676	76.78796472	59.05081546	98.68701189	118.2607386
Gglean001677	0	0	0	0
Gglean001678	11.89942023	12.81963	18.48403	22.80692
Gglean001679	0	0	0	0
Gglean001680	7.309113692	8.9789202	4.675908	2.7689083
Gglean001681	7.309113692	8.9789202	4.675908	2.7689083
Gglean001682	12.82093193	17.35925676	3.25601379	1.420166701
Gglean001683	49.7766	49.9859	33.4075	29.9977
Gglean001684	3.26E-05	2.41E-06	1.72951	0.000108842
Gglean001685	9.500436863	10.38878924	7.900004186	7.768473333
Gglean001686	9.500436863	10.38878924	7.900004186	7.768473333
Gglean001687	44.62680001	48.36890005	19.021179	13.393493
Gglean001688	22.7938	23.4284	23.3898	18.7666
Gglean001689	44.13710478	42.27834	44.827455	42.473741
Gglean001690	3.22996	2.84115	2.634432	2.294874
Gglean001691	41.83954	42.35045	47.881682	46.93521
Gglean001692	23.90878	25.37491	9.13062	8.60025
Gglean001693	152.65233	152.08192	167.6967	170.7453
Gglean001694	152.65233	152.08192	167.6967	170.7453
Gglean001695	0.852614	0	0	0.838844
Gglean001696	53.82	58.2931	39.9982	38.2945
Gglean001697	50.52777	54.36731	59.50714	51.54411
Gglean001698	50.52777	54.36731	59.50714	51.54411
Gglean001699	44.89132	39.12968	20.24537	22.23187
Gglean001700	2.740736546	2.48873942	2.305350594	2.403411
Gglean001701	38.05574118	37.39390944	35.01574437	36.34727179
Gglean001702	14.4546	22.1185	18.473	20.5799
Gglean001703	0	0.0670064	0	0
Gglean001704	0	0.833572	0	0.745934
Gglean001705	0	1.09065	0	0
Gglean001706	0	0	0	0
Gglean001707	1.027887227	1.40386544	1.5956563	1.720831024
Gglean001708	40.3563	42.64232	60.2044	54.0628
Gglean001709	40.3563	42.64232	60.2044	54.0628
Gglean001710	0.000908651	0.584761	1.75914	0.623913
Gglean001711	7.784117542	6.025221081	8.449772673	12.47876773
Gglean001712	15.00611	19.08857	18.5436	23.20005
Gglean001713	43.03243	42.84731	70.6703	74.9505
Gglean001714	0.136543	0	0.0555674	0.0656399
Gglean001715	8.53E-06	8.55E-08	1.88E-06	7.16E-06

Gglean001716	22.85305	25.11242	14.28093	11.76028
Gglean001717	11.87805	15.16969	22.020705	28.010562
Gglean001718	11.87805	15.16969	22.020705	28.010562
Gglean001719	18.22632	15.36383	11.30696	9.632331
Gglean001720	121.14834	108.49342	121.844821	184.2555
Gglean001721	8.67783	11.17788	2.83789	3.598067
Gglean001722	88.59391	89.30867	82.09842	95.21313
Gglean001723	88.59391	89.30867	82.09842	95.21313
Gglean001724	14.451581	14.684593	10.8362	9.92538
Gglean001725	11.52062	11.85412944	16.64339	18.83938957
Gglean001726	226.9478101	228.5983301	246.2859101	442.8636101
Gglean001727	226.9478101	228.5983301	246.2859101	442.8636101
Gglean001728	46.2333	60.9611	61.4668	87.0637
Gglean001729	1.66139	1.51048	3.10112	3.43827
Gglean001730	0.280274	0.350692	0.577882	0.714293
Gglean001731	0.75047	0.58071574	0.5404506	0.725109
Gglean001732	28.4083	27.87410892	25.32602	27.7743
Gglean001733	31.97786	29.8399	34.77859	29.37001
Gglean001734	3.615887	2.212475	2.946491332	4.852802
Gglean001735	3.615887	2.212475	2.946491332	4.852802
Gglean001736	20.782361	17.568353	11.569324	12.581031
Gglean001737	86.4661	73.58614	47.14338	43.54892
Gglean001738	50.7936	53.21163	53.75577	52.81600002
Gglean001739	15.132347	17.27020004	13.956121	14.695461
Gglean001740	25.19644	20.54677	13.96153	13.40069
Gglean001741	1.297853583	1.2600067	0.629630871	0.621618659
Gglean001742	1.297853583	1.2600067	0.629630871	0.621618659
Gglean001743	79.50043524	81.39123663	112.042243	101.46572
Gglean001744	45.41874079	45.8978609	55.523979	56.556834
Gglean001745	3.801810081	4.432097109	6.806395397	6.836720787
Gglean001746	1.19752	0.193886	0.320913	0.00889713
Gglean001747	150.1905	155.9111783	100.6486479	103.2566299
Gglean001748	150.1905	155.9111783	100.6486479	103.2566299
Gglean001749	93.415412	88.546991	106.07869	122.55267
Gglean001750	7.837392	6.12494	7.26161	9.41328
Gglean001751	11.51823379	11.86989323	16.08780157	19.81275
Gglean001752	8.30444	9.74204	10.32601	10.63235
Gglean001753	26.3663	28.3229	25.9973	26.1116
Gglean001754	28.17159	34.7109	38.1424	43.77897
Gglean001755	218.2942185	222.2293448	198.7948322	177.0705704
Gglean001756	0	0	0	0
Gglean001757	52.1695	54.1862	42.4137	42.6571
Gglean001758	27.337868	25.58174	24.351002	22.886658
Gglean001759	2.570127011	2.299703135	6.293892036	5.834718662

Gglean001760	0.427503	0	3.77E-05	0.0940395
Gglean001761	95.017008	98.44592	108.17308	141.76425
Gglean001762	0.26408	0.283458	0.217622	0.778096
Gglean001763	16.82991362	18.3904596	25.61626841	23.33614704
Gglean001764	1.548199	1.849907383	2.411797	3.257548
Gglean001765	15.2205059	14.42556144	22.97554917	24.70683303
Gglean001766	0	0	0	0
Gglean001767	15.81106	15.00325027	15.599905	20.32495808
Gglean001768	1.419304574	1.112433602	1.269713	1.597216183
Gglean001769	70.58293	70.59885	78.75763	89.14477
Gglean001770	35.6106	38.5176	29.5168	32.190753
Gglean001771	2.86103	1.74193	4.34073	1.14738
Gglean001772	47.02888	45.85317	38.64481	35.52242
Gglean001773	55.33985	58.8945512	108.1719248	102.62854
Gglean001774	55.33985	58.8945512	108.1719248	102.62854
Gglean001775	32.8994517	28.59015099	40.74167292	48.43963841
Gglean001776	2.253932692	3.173813	3.450001	3.535479
Gglean001777	0.31863138	0.80498268	0.59161879	0.905053826
Gglean001778	3.669886341	4.106017051	6.479212193	5.407371387
Gglean001779	3.669886341	4.106017051	6.479212193	5.407371387
Gglean001780	0.451143	0.1356589	0.3883113	0.0575484
Gglean001781	0.986578	1.78209488	0.77125849	1.200547
Gglean001782	34.40968583	35.20552811	27.44567849	25.7301364
Gglean001783	34.40968583	35.20552811	27.44567849	25.7301364
Gglean001784	0	0.165537	0	0
Gglean001785	0	0	0	0
Gglean001786	19.57353779	18.75669972	14.260698	13.56255888
Gglean001787	19.29175283	19.612374	26.579272	25.11363513
Gglean001788	0	0	0	0
Gglean001789	0	0	0	0
Gglean001790	58.1709	59.1618	48.422	49.1651
Gglean001791	84.80495	92.55572	29.93722	27.34768
Gglean001792	1.68086	0	0	0.416097
Gglean001793	0.355265	0.364127	0.623315	0.428519
Gglean001794	83.265788	80.370207	139.33236	128.08468
Gglean001795	0.859888	0.76446	2.99796	2.07985
Gglean001796	83.265788	80.370207	139.33236	128.08468
Gglean001797	83.265788	80.370207	139.33236	128.08468
Gglean001798	18.5374	19.3652	19.5631	22.0086
Gglean001799	4.53021	3.825185	4.34101	6.04979
Gglean001800	3.063789	4.51991789	6.26526783	7.420807
Gglean001801	27.7020542	32.23291	23.535	22.78462794
Gglean001802	27.7020542	32.23291	23.535	22.78462794
Gglean001803	0.0341016	0	0	0

Gglean001804	0.0341016	0	0	0
Gglean001805	0	0	0	0
Gglean001806	11.92612205	11.12958219	16.38504457	12.90960089
Gglean001807	11.92612205	11.12958219	16.38504457	12.90960089
Gglean001808	11.92612205	11.12958219	16.38504457	12.90960089
Gglean001809	0.343154643	0.035769654	1.070999091	0.4991981
Gglean001810	34.117	39.8356	48.89843	53.1594
Gglean001811	34.117	39.8356	48.89843	53.1594
Gglean001812	38.56614	40.8741	23.78195	24.0396
Gglean001813	10.5616	11.51897407	17.71560421	18.2987
Gglean001814	10.5616	11.51897407	17.71560421	18.2987
Gglean001815	52.6348496	47.77437395	84.3278145	80.03664606
Gglean001816	29.31607952	27.20294288	27.43113454	27.88005807
Gglean001817	0.137925845	0.01213771	0.131023956	0.266657506
Gglean001818	0.10442	0	0	0
Gglean001819	1.347628001	2.530098585	2.864627945	3.098225
Gglean001820	1.347628001	2.530098585	2.864627945	3.098225
Gglean001821	65.3997514	72.03184916	85.7069862	95.60920544
Gglean001822	65.3997514	72.03184916	85.7069862	95.60920544
Gglean001823	6.102207192	6.806229	8.641555	11.549758
Gglean001824	6.102207192	6.806229	8.641555	11.549758
Gglean001825	41.50429645	47.61811073	37.94639006	34.61214803
Gglean001826	41.50429645	47.61811073	37.94639006	34.61214803
Gglean001827	31.83564023	30.01022	9.760659809	10.11444137
Gglean001828	31.83564023	30.01022	9.760659809	10.11444137
Gglean001829	24.4933123	24.08911755	29.79240881	31.65659363
Gglean001830	15.81133	23.484753	13.18813101	12.341454
Gglean001831	6.346700618	5.486462391	4.273648952	6.263149755
Gglean001832	6.346700618	5.486462391	4.273648952	6.263149755
Gglean001833	6.346700618	5.486462391	4.273648952	6.263149755
Gglean001834	0	0	0	0
Gglean001835	0	0	0	0
Gglean001836	0.272152	0.281177	0.827805	0
Gglean001837	0	0	0.0584478	0
Gglean001838	21.380223	18.44385	22.94053	30.18715624
Gglean001839	0	0	0	0
Gglean001840	0	0	0	0
Gglean001841	4.64778596	5.609075	7.595241	7.216536
Gglean001842	8.075444	8.39235	6.71505	6.084092
Gglean001843	17.8242	19.0603	15.8636	16.2679
Gglean001844	0.175917	0.305097	0.190394	0.397434
Gglean001845	1.055687539	0.670714657	1.204599299	1.90797105
Gglean001846	15.5971	10.7716	8.83259	8.93989
Gglean001847	54.35940096	57.61912463	34.702998	32.617084

Gglean001848	0	0	5.49E-247	0
Gglean001849	21.56538404	21.15881815	15.88198584	14.26933819
Gglean001850	2.88E-22	1.40E-123	9.49E-194	2.16E-18
Gglean001851	9.56E-06	0.000284154	0.000103893	5.34E-05
Gglean001852	0	0.0470309	0	0
Gglean001853	19.2863828	25.82875	9.799336199	11.36784945
Gglean001854	141.4454332	144.7420339	125.6229266	132.935913
Gglean001855	355.25802	405.69382	370.97538	685.86804
Gglean001856	616.7084	684.0511	647.2887	1235.1571
Gglean001857	54.7436	60.752	57.502	102.6701
Gglean001858	36.589604	40.971359	37.513135	70.58744
Gglean001859	55.42091217	76.72418	63.68571741	115.6503394
Gglean001860	20.55387604	20.43831658	26.29778369	27.025538
Gglean001861	49.296955	49.978074	63.723076	64.40136
Gglean001862	49.296955	49.978074	63.723076	64.40136
Gglean001863	49.296955	49.978074	63.723076	64.40136
Gglean001864	30.00474444	39.66130653	49.82554219	61.8497107
Gglean001865	9.301813	9.421487	18.499612	24.840245
Gglean001866	0	0	0	0
Gglean001867	0.769094225	0.598615	0.982819739	1.228801618
Gglean001868	41.44374488	48.3232417	36.99934804	28.34203649
Gglean001869	41.44374488	48.3232417	36.99934804	28.34203649
Gglean001870	0.696267	0.486618	0.89565	1.0003
Gglean001871	7.962196773	8.1685364	3.793561	2.627544328
Gglean001872	7.146447	6.783613	4.309399	4.959684
Gglean001873	67.53113	68.13401	13.73077603	10.3532986
Gglean001874	5.378715	15.894285	18.129926	19.8717513
Gglean001875	5.378715	15.894285	18.129926	19.8717513
Gglean001876	5.378715	15.894285	18.129926	19.8717513
Gglean001877	5.378715	15.894285	18.129926	19.8717513
Gglean001878	5.378715	15.894285	18.129926	19.8717513
Gglean001879	108.167	119.772	55.2298	49.8919
Gglean001880	2.81855	4.46107	0.385092	2.04404
Gglean001881	0	0	0	0
Gglean001882	23.39643	25.95301	7.51503	4.88572
Gglean001883	6.272759171	8.86027686	10.078813	12.817143
Gglean001884	0.196011333	0.2020474	0.2330745	0.0354547
Gglean001885	10.06714	9.3205	10.38689	9.297136
Gglean001886	8.885655094	9.37504971	2.633064481	2.553044261
Gglean001887	8.885655094	9.37504971	2.633064481	2.553044261
Gglean001888	28.62304894	29.18593843	23.1907264	20.48237293
Gglean001889	87.3767	104.237	58.8136	54.7372
Gglean001890	48.11624714	41.99234609	55.389486	65.53821364
Gglean001891	52.841	49.5368	52.8692	51.6665

Gglean001892	48.11624714	41.99234609	55.389486	65.53821364
Gglean001893	48.11624714	41.99234609	55.389486	65.53821364
Gglean001894	48.11624714	41.99234609	55.389486	65.53821364
Gglean001895	3.657959002	2.62069041	1.926470002	2.714328
Gglean001896	67.29124	61.482134	63.176799	64.048292
Gglean001897	17.755425	19.524748	15.97349419	14.59975981
Gglean001898	0	0	0	0
Gglean001899	30.56862	34.60349052	45.33561	39.34868
Gglean001900	7.10814	4.69637	2.51532	3.71402
Gglean001901	2.2383216	1.55638521	1.113021969	1.108699638
Gglean001902	25.02022983	23.06115926	13.72351	13.59646
Gglean001903	3.071909	2.715877	4.23175	3.59182
Gglean001904	0	0	0	0
Gglean001905	13.783363	13.99887551	18.87740863	21.29822746
Gglean001906	13.783363	13.99887551	18.87740863	21.29822746
Gglean001907	0	0	0	0
Gglean001908	5.06971	3.58469	6.39092	5.94332
Gglean001909	5.06971	3.58469	6.39092	5.94332
Gglean001910	4.488221347	3.555918789	4.047409492	4.572373775
Gglean001911	0.165301646	0.184329555	0.134227499	0.256494082
Gglean001912	1.438170299	1.6627456	2.229468116	2.1438279
Gglean001913	4.990289268	5.608059592	4.42313697	3.318312878
Gglean001914	0.059143941	0	0.048123199	0.0422428
Gglean001915	5.040532	5.8867	3.651610747	2.948457928
Gglean001916	18.90592	24.37839	5.93711	6.0747
Gglean001917	23.7923	23.9862	13.85164	16.79704
Gglean001918	52.5189	53.6358	36.7659	30.0409
Gglean001919	17.8556	19.81954	10.758178	9.632609
Gglean001920	0.144077988	0.301315984	0.467241267	0.472387589
Gglean001921	0	0	0	0
Gglean001922	0	0	0	0
Gglean001923	78.84185	91.41617	56.33537	56.40097
Gglean001924	7.99515	10.05529	15.48782	17.81851
Gglean001925	0.142293	0.358843	0.0574784	0.206048
Gglean001926	9.18148	15.97363	14.04634	19.21803
Gglean001927	65.36723	73.1519	22.42267	18.82874168
Gglean001928	0	0	0	0.559341
Gglean001929	30.07413208	32.172459	28.093241	26.99480422
Gglean001930	30.07413208	32.172459	28.093241	26.99480422
Gglean001931	0	0	0	0
Gglean001932	0	0	0	0
Gglean001933	4.248973821	4.146627098	4.796244694	5.4435683
Gglean001934	0	0	0	0
Gglean001935	0	0	0	0

Gglean001936	18.15310888	17.10788582	9.85213643	8.857758011
Gglean001937	0.205741	0.108313	0.0837856	0
Gglean001938	1.134681309	0.959371418	1.331539447	1.487449582
Gglean001939	94.19911	74.44202	74.5167	77.66913
Gglean001940	94.19911	74.44202	74.5167	77.66913
Gglean001941	26.96684	25.32755	23.41737	21.6366
Gglean001942	73.20302	73.18352513	124.920089	145.5339318
Gglean001943	73.20302	73.18352513	124.920089	145.5339318
Gglean001944	73.20302	73.18352513	124.920089	145.5339318
Gglean001945	51.7382	78.2509	70.3538	123.876
Gglean001946	109.6704	99.3522	98.8903	100.0142
Gglean001947	30.36474714	28.5955865	54.14815496	53.58057476
Gglean001948	0	0	0	0.431643
Gglean001949	0	0	0	0
Gglean001950	0	0	0	0
Gglean001951	26.69282	27.08788	29.09301	27.75208
Gglean001952	26.69282	27.08788	29.09301	27.75208
Gglean001953	76.32106	59.24256	66.80627	74.35805
Gglean001954	76.32106	59.24256	66.80627	74.35805
Gglean001955	0.000175642	0.901653	0.00136286	0.000110683
Gglean001956	7.291745	6.94864146	6.310485	6.130542
Gglean001957	1.489455	1.724635	1.412567	0.893929
Gglean001958	36.828255	36.15354802	18.440371	18.55705732
Gglean001959	36.828255	36.15354802	18.440371	18.55705732
Gglean001960	26.88194378	30.9335648	40.2333573	46.07341382
Gglean001961	14.76075523	13.84513899	20.86408716	23.22427489
Gglean001962	119.7729	119.1352	161.1883	155.3526
Gglean001963	20.8382416	19.98861	19.538128	21.87517
Gglean001964	71.5601953	68.827072	82.26097662	86.4768655
Gglean001965	32.98662058	37.21128307	52.16954571	58.36988323
Gglean001966	45.66432112	60.62881722	27.37077277	29.70588
Gglean001967	8.3572	6.77184	9.26662	11.6257
Gglean001968	10.96401002	9.509564728	7.557673483	6.84925014
Gglean001969	0.0475062	1.63E-56	2.60E-53	5.15E-66
Gglean001970	78.3029	59.13161953	52.8704	36.20674
Gglean001971	78.3029	59.13161953	52.8704	36.20674
Gglean001972	28.042704	33.920699	25.57395229	25.88711379
Gglean001973	2.35E-05	0.286528	0.000122832	0.00011472
Gglean001974	0.270082	0.275768	0.289197	0.086396
Gglean001975	6.942197269	6.558165088	5.209458	6.287981556
Gglean001976	6.942197269	6.558165088	5.209458	6.287981556
Gglean001977	27.6218	28.5339	11.39299	11.74483
Gglean001978	4.900260345	4.320160178	3.468066094	3.084721982
Gglean001979	0.5212965	0.132983857	0.466317606	0.658512575

Gglean001980	30.77032	31.4322	16.21463	14.688
Gglean001981	20.6094	19.382612	17.67729	22.9327
Gglean001982	10.88166	12.21804	5.841586	4.427437
Gglean001983	9.24603	9.05101	10.20246	9.75728
Gglean001984	52.5831	67.6923	36.547	37.6835
Gglean001985	55.00962	52.31839	38.31145	40.46615
Gglean001986	5.71899	5.914797	3.6990559	3.58411082
Gglean001987	2.618001113	2.177884429	4.046387	4.665128
Gglean001988	0.153463	0.651102	0.634341	0.301471
Gglean001989	2.618001113	2.177884429	4.046387	4.665128
Gglean001990	0.593243	1.030999401	0.889732	0.575034795
Gglean001991	23.43569495	25.39361368	37.11034102	36.545887
Gglean001992	23.43569495	25.39361368	37.11034102	36.545887
Gglean001993	2.193183373	2.496151172	3.082534636	3.639788532
Gglean001994	15.07617	16.29544	9.77023	9.21584
Gglean001995	51.1183	41.4196	28.3783	33.9098
Gglean001996	21.98423607	25.16168864	24.81260729	22.51936323
Gglean001997	21.98423607	25.16168864	24.81260729	22.51936323
Gglean001998	1.50379	1.77774	2.94393	2.80142
Gglean001999	3.327901793	2.874890832	3.544201366	2.875391271
Gglean002000	0	0.0476801	0.0373836	0.044261
Gglean002001	1.825354052	2.45644576	2.1121378	2.80360065
Gglean002002	0.659817	0.750538	0.757653	1.09036
Gglean002003	0.657853191	0.72430521	0.571346014	0.85913476
Gglean002004	24.79581554	22.9836209	43.76583436	42.75986545
Gglean002005	0	0	0	0
Gglean002006	0.199281	0.413902	0	0
Gglean002007	0	0	0	0
Gglean002008	0.0766059	0.0384146	0.0613861	0
Gglean002009	0.0459132	0	0	0
Gglean002010	0	0	0.139034	0
Gglean002011	0	0	0	0
Gglean002012	0	0	0	0
Gglean002013	12.580506	19.34089	3.988712316	1.78135
Gglean002014	32.91171	40.23975	12.43048	8.10365
Gglean002015	29.19909555	28.61740312	27.83611824	30.73372437
Gglean002016	22.93021021	22.55493677	36.57286743	39.8460588
Gglean002017	0.0843663	0.175311	0.208994	0.0823333
Gglean002018	0	0	0	0
Gglean002019	31.36768	28.51974	42.82734	54.49823
Gglean002020	31.36768	28.51974	42.82734	54.49823
Gglean002021	0	0	0	0
Gglean002022	0	0	0	0
Gglean002023	0	0	0	0

Gglean002024	0	0	0	0
Gglean002025	0.0495708	0	0.0400547	0
Gglean002026	0.26675	0.342654	0.0539463	0
Gglean002027	0	0	0	0
Gglean002028	0	0	0	0
Gglean002029	0	0.0694847	0.0549203	0.130279
Gglean002030	0	0	0	0
Gglean002031	0	1.16995	0	0
Gglean002032	0	0	0	0
Gglean002033	0	0	0	0
Gglean002034	0	0	0	0
Gglean002035	20.27817	21.91831	32.4488	41.477
Gglean002036	6.1818167	6.58748	4.733118368	4.76454765
Gglean002037	0	0	0	0.149683
Gglean002038	0.276517	0	0	0
Gglean002039	0	0	0	0
Gglean002040	0	0	0	0.184302
Gglean002041	0	0	0	0
Gglean002042	0.0868263	0	0	0
Gglean002043	0	0	0	0
Gglean002044	0	0	0	0
Gglean002045	0	0	0	0
Gglean002046	0	0	0	0.0900072
Gglean002047	0	0	0	0
Gglean002048	2.64445	0.000279745	1.21437	0.521804
Gglean002049	74.043133	73.33581	66.395733	60.154357
Gglean002050	0.264371	0.233831	0	0.036208
Gglean002051	0	0	0	0
Gglean002052	0.00148117	6.72E-13	2.71E-06	2.62E-33
Gglean002053	0	0	0	0
Gglean002054	5.951872894	6.347594	9.414352188	20.74729
Gglean002055	5.951872894	6.347594	9.414352188	20.74729
Gglean002056	10.60716062	10.7723	6.978610048	6.62678166
Gglean002057	10.60716062	10.7723	6.978610048	6.62678166
Gglean002058	1.93495	2.41684	1.80949	1.85667
Gglean002059	210.97177	221.3405839	80.94716	82.678192
Gglean002060	210.97177	221.3405839	80.94716	82.678192
Gglean002061	16.13759	16.838343	12.60251	10.835959
Gglean002062	20.07033	22.5823	18.96966	18.91942
Gglean002063	26.56501237	26.78568698	31.13878463	33.85445322
Gglean002064	1.000202484	0.961119569	1.713834685	1.214069543
Gglean002065	1.000202484	0.961119569	1.713834685	1.214069543
Gglean002066	0.0863269	0	0.0353732	0
Gglean002067	35.7527	34.11	43.7199	43.9059

Gglean002068	10.64362	9.86082	10.41541	10.31941
Gglean002069	24.274515	25.48740501	37.3964084	44.83759035
Gglean002070	24.274515	25.48740501	37.3964084	44.83759035
Gglean002071	24.274515	25.48740501	37.3964084	44.83759035
Gglean002072	121.038	119.717	67.2175	71.1767
Gglean002073	55.07034428	60.2367277	54.09447	51.65688629
Gglean002074	2.137718821	2.915426873	3.297518571	5.112819714
Gglean002075	2.137718821	2.915426873	3.297518571	5.112819714
Gglean002076	2.137718821	2.915426873	3.297518571	5.112819714
Gglean002077	80.40460987	96.63475254	41.42672196	41.18631017
Gglean002078	35.82628	34.1216	35.60631	37.38617
Gglean002079	30.46404	32.39455	16.23888	13.58617
Gglean002080	0.95874242	1.511347148	1.093050569	2.381389
Gglean002081	7.40568	4.1746257	7.51947	8.61657
Gglean002082	22.38011	23.44724	20.52038	19.68893
Gglean002083	14.88879035	13.57006907	5.984001782	5.624296618
Gglean002084	0.239069	0.563817	0.482301	0.389726
Gglean002085	0.568685	3.92E-05	2.92E-05	4.66E-06
Gglean002086	12.636262	14.09032498	15.29257252	16.43183188
Gglean002087	16.052124	18.432264	21.34446	23.599454
Gglean002088	58.79558	63.41485	46.55961	43.28148
Gglean002089	1.10329	0.625089	0.342606326	0.269484
Gglean002090	0.932306	0	0	0
Gglean002091	50.4151091	48.96264321	72.70485282	88.89807441
Gglean002092	86.935849	82.55395	87.10901	82.99857
Gglean002093	1.57617	2.89143	8.36694	11.8659
Gglean002094	15.3613908	16.3747906	18.680737	20.68836087
Gglean002095	62.357003	61.0398619	77.734089	88.29106
Gglean002096	31.5051	30.9682	34.0661	31.2517
Gglean002097	13.88894	11.73406	9.04547	7.919263
Gglean002098	3.88005	4.73	5.7223	7.75035
Gglean002099	6.20159	4.78322	3.58548	5.42102
Gglean002100	8.35988	8.61859	5.73062	5.47728
Gglean002101	18.15118	18.18055	8.988536	6.602812891
Gglean002102	17.211618	19.249336	27.595633	24.71838961
Gglean002103	17.211618	19.249336	27.595633	24.71838961
Gglean002104	0.935043	8.26134	0.375569	0.247415
Gglean002105	195.8365766	205.2071761	105.1876584	94.80335
Gglean002106	0	0	0	0
Gglean002107	44.1705	46.7658	31.5188	29.3843
Gglean002108	29.32691	28.06222	26.665002	24.784741
Gglean002109	143.225	151.345	106.795	95.8516
Gglean002110	17.1865	18.3782	13.4793	12.8684
Gglean002111	34.01433	35.92812	24.52156	20.15415147

Gglean002112	34.01433	35.92812	24.52156	20.15415147
Gglean002113	0	1.35919	0	0.614761
Gglean002114	180.9385906	194.5473606	118.8271541	109.3275255
Gglean002115	35.1352	24.8798	23.9303	18.1177
Gglean002116	24.97646	27.95295	22.82708	26.67438
Gglean002117	8.980173	9.912132	7.455861758	7.467989755
Gglean002118	8.980173	9.912132	7.455861758	7.467989755
Gglean002119	0.346890047	0.857592772	1.061818	0.182934784
Gglean002120	0.346890047	0.857592772	1.061818	0.182934784
Gglean002121	0.00276121	4.48E-05	5.19E-05	1.21E-05
Gglean002122	0.00276121	4.48E-05	5.19E-05	1.21E-05
Gglean002123	0	0	0	0
Gglean002124	12.22874045	12.4653876	17.33258499	19.5873565
Gglean002125	2.6782843	2.448051246	4.129545212	4.372011246
Gglean002126	60.8312	53.3412	72.1209	81.0207
Gglean002127	0	0	0.0532898	0
Gglean002128	0.565334	0.878683	0.638506	0.64934
Gglean002129	21.5528905	21.38289484	17.508354	16.85785107
Gglean002130	0	0	0	0
Gglean002131	0	0	0	0
Gglean002132	0	0	0	0
Gglean002133	29.0372	29.753568	20.469923	15.99885
Gglean002134	29.0372	29.753568	20.469923	15.99885
Gglean002135	0	0	0	0
Gglean002136	1.49947	0	0	0
Gglean002137	2.713857234	1.443744002	1.73402207	2.2136988
Gglean002138	2.713857234	1.443744002	1.73402207	2.2136988
Gglean002139	0.342960738	0.660512245	0.590841078	0.796734799
Gglean002140	0.0743981	0	0	0
Gglean002141	54.5991	83.4538	33.0798	29.8621
Gglean002142	5.25648	5.54326	0.61196	0.414664
Gglean002143	0	0	0	0
Gglean002144	0	0	0	0
Gglean002145	0	0	0	0
Gglean002146	0.185643	0	0	0
Gglean002147	26.2431241	25.54629461	25.64303084	26.15978849
Gglean002148	26.2431241	25.54629461	25.64303084	26.15978849
Gglean002149	52.6569	44.04373668	56.03083862	78.19160062
Gglean002150	52.6569	44.04373668	56.03083862	78.19160062
Gglean002151	0	0	0	0
Gglean002152	0	0.0246188	0	0
Gglean002153	19.80529	21.32473	17.7732	17.9125
Gglean002154	45.6391215	42.23595311	26.38004415	25.51335192
Gglean002155	20.88404542	16.68800947	12.15213163	11.05884588

Gglean002156	17.40063	16.23031	15.11392	14.01176
Gglean002157	0	0	0	0
Gglean002158	24.15885182	25.37369956	25.4135438	25.7239256
Gglean002159	36.24691	38.86357	36.24612	37.6581
Gglean002160	0	0	0	0
Gglean002161	1.02829	0.479769	0.800781	0.589205
Gglean002162	4.569256494	6.813343962	4.388667188	6.202788286
Gglean002163	0	0.116947937	0	0
Gglean002164	86.80839398	84.72808872	45.84848888	40.33410528
Gglean002165	86.80839398	84.72808872	45.84848888	40.33410528
Gglean002166	86.80839398	84.72808872	45.84848888	40.33410528
Gglean002167	52.47287507	57.14844399	62.483356	65.09706452
Gglean002168	0	0	0	0
Gglean002169	0	0	0	0
Gglean002170	37.5803	33.96071	26.13397	26.8728
Gglean002171	9.11191	8.332708	9.463059	8.975240613
Gglean002172	105.8897717	122.1686246	163.2886741	151.7668312
Gglean002173	105.8897717	122.1686246	163.2886741	151.7668312
Gglean002174	105.8897717	122.1686246	163.2886741	151.7668312
Gglean002175	11.84184	12.35365	12.27001	10.904379
Gglean002176	11.84184	12.35365	12.27001	10.904379
Gglean002177	0.371288	0.229021	0	0.217029
Gglean002178	4.88995	5.076819	6.049076	6.89256118
Gglean002179	111.00419	118.2683651	124.42848	141.9329195
Gglean002180	86.97023	107.1003932	96.55789955	102.14037
Gglean002181	145.57793	153.60795	180.61432	244.21902
Gglean002182	0	0	0	0
Gglean002183	0	0	1.75786	0.526821
Gglean002184	0	0	0	0
Gglean002185	22.26705033	6.10791016	6.525770375	11.67344
Gglean002186	22.26705033	6.10791016	6.525770375	11.67344
Gglean002187	92.2654	87.6123	84.7342	96.3479
Gglean002188	3.31777	6.0681	2.89313	6.346483
Gglean002189	19.52864198	14.87117602	28.12425347	29.94697848
Gglean002190	19.52864198	14.87117602	28.12425347	29.94697848
Gglean002191	29.94115844	29.76924	17.16788829	14.17330383
Gglean002192	0.432575	1.4066	0.712028	0.854294
Gglean002193	0	0	0	0
Gglean002194	0	0	0	0
Gglean002195	52.0631978	48.32340156	38.3225805	34.55369182
Gglean002196	38.4917	43.0744	39.6007	33.90691
Gglean002197	60.16245	56.91073	35.304791	30.40447
Gglean002198	95.40130888	108.3272074	133.3843862	142.7654129
Gglean002199	95.40130888	108.3272074	133.3843862	142.7654129

Gglean002200	17.07210228	16.63266909	34.92348784	38.93007563
Gglean002201	1.006666	0.896098	0.98129	1.373084
Gglean002202	1.57622	2.37394	0.569053	0.673599
Gglean002203	0	0	0	0
Gglean002204	11.359	9.41623	8.1736	9.72753
Gglean002205	25.59057727	24.37175753	16.71902209	17.81505018
Gglean002206	25.59057727	24.37175753	16.71902209	17.81505018
Gglean002207	25.59057727	24.37175753	16.71902209	17.81505018
Gglean002208	62.68451648	65.42457567	46.23163576	41.99816511
Gglean002209	62.68451648	65.42457567	46.23163576	41.99816511
Gglean002210	0.119816	0	4.05E-06	0
Gglean002211	51.55715	48.63367	40.40065	34.75639
Gglean002212	40.55068618	44.43483554	37.2426793	34.991965
Gglean002213	12.66	13.5675	11.1309	11.34212915
Gglean002214	7.031354087	8.650611628	2.184453708	3.03460269
Gglean002215	0	0	0.0721445	0
Gglean002216	39.8794	43.8715	34.387	32.623
Gglean002217	5.964404	8.4449	2.352737	2.361553647
Gglean002218	12.3300316	14.09448181	16.844868	18.647923
Gglean002219	92.7894	84.7599	103.3867	88.4761
Gglean002220	2857.77757	2971.701504	4248.804412	5362.606831
Gglean002221	2857.77757	2971.701504	4248.804412	5362.606831
Gglean002222	13.8338	13.8425	21.2661	22.9022
Gglean002223	21.340353	21.664198	32.395964	34.145941
Gglean002224	62.47905	70.503312	53.78470001	53.79653
Gglean002225	62.47905	70.503312	53.78470001	53.79653
Gglean002226	21.340353	21.664198	32.395964	34.145941
Gglean002227	18.77823	17.605537	21.88505	22.590112
Gglean002228	0	0.299037	0	0
Gglean002229	3.23811	1.871953	4.56761	6.11642
Gglean002230	21.08106	23.20896	35.43383	39.89892
Gglean002231	21.08106	23.20896	35.43383	39.89892
Gglean002232	1.70911	2.0386	0.468355	0.112023
Gglean002233	238.812	261.805	3.84435	0.169738
Gglean002234	24.882751	24.43774489	20.298978	20.044492
Gglean002235	58.9568	43.501	58.0769	35.568
Gglean002236	2857.77757	2971.701504	4248.804412	5362.606831
Gglean002237	8.554372697	8.214148186	9.205094	12.51558077
Gglean002238	50.7473	48.18361	28.27572	25.03188
Gglean002239	48.9086	52.7848	19.4664	17.1332
Gglean002240	12.67776	10.54817	8.22273	9.3428
Gglean002241	0.2291636	0.258150537	0.321150354	0.597067
Gglean002242	0	0	0.115836	0
Gglean002243	0	0	0	0

Gglean002244	9.983707905	9.51781	9.427947	7.735620189
Gglean002245	11.28165	12.66067	6.10123	5.799203
Gglean002246	14.00627	11.48791103	17.277901	22.09847173
Gglean002247	14.00627	11.48791103	17.277901	22.09847173
Gglean002248	0.2149948	0.0406279	0.316871	0.143411
Gglean002249	26.01126	27.1153945	12.53544	11.41912
Gglean002250	34.6350203	37.11015221	28.4481122	26.20150443
Gglean002251	220.0701	223.0957	144.2437	139.7634
Gglean002252	16.1237	21.3501	9.79208	7.13694
Gglean002253	30.2097	26.2455	15.0658	10.6885
Gglean002254	39.0623	46.0409	39.9985	31.6782
Gglean002255	1501.57	1541.33	1015.12	942.214
Gglean002256	3.958301	4.299341	1.60261	1.838421
Gglean002257	0	0	0.274043	0
Gglean002258	44.229	43.7419	29.13073	29.7579
Gglean002259	20.3784	38.9504	18.6024	12.1132
Gglean002260	18.84680708	21.81775459	12.11136014	12.550682
Gglean002261	18.84680708	21.81775459	12.11136014	12.550682
Gglean002262	22.3784	29.7001	26.3587	23.9069
Gglean002263	0	0	0	0
Gglean002264	14.53674	14.26269	11.81611	8.25656
Gglean002265	98.69416	114.6307281	76.59497649	82.67168611
Gglean002266	33.3156	38.0795	26.1042	21.4795
Gglean002267	70.85743683	74.142469	74.77579391	73.001664
Gglean002268	70.85743683	74.142469	74.77579391	73.001664
Gglean002269	70.85743683	74.142469	74.77579391	73.001664
Gglean002270	26.17789	26.79419	14.208862	16.591527
Gglean002271	7.105962448	7.662688935	7.429737383	7.777628003
Gglean002272	0	0.17055	0	0
Gglean002273	123.4636322	98.5175	124.0127005	173.033
Gglean002274	2.275982457	2.487484124	3.394021493	3.720089569
Gglean002275	1.556983495	1.531507178	3.3403147	3.587747682
Gglean002276	15.0689	17.7583	9.49166	11.3613
Gglean002277	42.84155083	40.65960593	50.286159	47.97110986
Gglean002278	42.84155083	40.65960593	50.286159	47.97110986
Gglean002279	41.83657717	54.99927213	58.20463398	72.15784981
Gglean002280	30.64049	42.25582	49.94723	42.50843
Gglean002281	0.424882	0	0	0
Gglean002282	16.79139	16.81986	15.02745	16.24262
Gglean002283	8.515798518	8.39412	4.016431476	2.740115762
Gglean002284	0.0830127	0.0858716	0	0
Gglean002285	36.2923754	35.12479	35.240595	35.18041389
Gglean002286	36.2923754	35.12479	35.240595	35.18041389
Gglean002287	6.3424	5.42562	4.74147	3.688153

Gglean002288	17.05785	19.94463	12.27591	12.50263
Gglean002289	27.2755	27.1034	35.6224	41.6819
Gglean002290	10.0388	9.54106	11.9118	9.36025
Gglean002291	43.08835	43.49280804	43.80945	35.680103
Gglean002292	71.11915	76.579963	63.4137177	59.77381
Gglean002293	4.97862	4.39412	4.07411	2.976044
Gglean002294	4.97862	4.39412	4.07411	2.976044
Gglean002295	0.313374	0.213904	0	0.300577
Gglean002296	1.45712	1.37879	1.39894	1.42424
Gglean002297	0.439712	0.478145	0.732521	0.500941
Gglean002298	0	0	0	0
Gglean002299	0	0	0	0
Gglean002300	0	0	0	0
Gglean002301	0	0	0	0
Gglean002302	0	0	0.224998	0
Gglean002303	0	0	0	0
Gglean002304	0	0	0	0
Gglean002305	0	0.0228657	0.0178384	0
Gglean002306	0	0	0	0
Gglean002307	11.6130948	8.036881264	4.428857813	3.366462429
Gglean002308	11.6130948	8.036881264	4.428857813	3.366462429
Gglean002309	0	0	0	0
Gglean002310	0	0.147867	0.0641086	0
Gglean002311	0.0996093	0	0	0
Gglean002312	0.278614	0.1934988	0.075296952	0.447737
Gglean002313	0	0	0	0
Gglean002314	29.06096309	32.04071215	25.83395513	28.01248425
Gglean002315	118.731	106.666	67.371	60.453
Gglean002316	96.37338	103.03371	62.59976	51.967756
Gglean002317	96.37338	103.03371	62.59976	51.967756
Gglean002318	1.95183	2.22477	3.2392	2.87943
Gglean002319	28.86271	29.83936	29.1234	24.71644
Gglean002320	65.68952527	56.49022	58.02642	57.26989459
Gglean002321	93.3926	101.5565	72.5839	66.0223
Gglean002322	0	0	0	0
Gglean002323	25.66753767	27.67252485	21.76251793	20.9444036
Gglean002324	4.570122	4.380219869	4.474195025	7.288969211
Gglean002325	0.313996073	0.591846	0.184235672	0.354128956
Gglean002326	0	0	0	0
Gglean002327	0.0716833	0.0738004	0.0580563	0.0687132
Gglean002328	2.535294067	3.340524026	5.898109796	5.70005178
Gglean002329	0.728495	1.404338	1.056793173	3.21718
Gglean002330	13.54391	11.43265	10.45576	6.12539
Gglean002331	0	0	0	0

Gglean002332	0	0	0	0
Gglean002333	26.9915	32.62005	31.03684	28.42237
Gglean002334	0	0	0	0
Gglean002335	0	0	0	0
Gglean002336	0.140523	0.2850936	0.320990914	0.201649
Gglean002337	15.09199	18.380288	1.210608865	0.119133444
Gglean002338	0	0	0	0
Gglean002339	0.167154	0.9435	0.4672721	1.205096097
Gglean002340	2.236024583	1.981672	2.524402624	3.1483475
Gglean002341	0.184091	0	0.448137	0.884847
Gglean002342	0.0412805	0.0852548	0.134289	0.159151
Gglean002343	3.332082	7.17892	4.131672	4.0811
Gglean002344	0	0	0	0
Gglean002345	0.330569	0	0.133439	0.158253
Gglean002346	0	0	0	0
Gglean002347	0	0	0.0580121	0.0683141
Gglean002348	30.7779	27.5199	27.2789	55.4244
Gglean002349	23.5566	25.2551	34.3613	31.6869
Gglean002350	0	0.1909	0.0300258	0.0710404
Gglean002351	7.682710268	7.393667693	12.37874323	14.505378
Gglean002352	0.0237125	0	0	0
Gglean002353	37.554919	38.024072	40.257856	40.165002
Gglean002354	0	0	0	0
Gglean002355	0	0	0	0
Gglean002356	0	0	0	0
Gglean002357	0.113818	0.116885	0.0308604	0.072929
Gglean002358	0.630216	0.495817	0.514433	0.915799
Gglean002359	14.98984	14.5962	14.95585	14.47269
Gglean002360	0	0	0.428933	0
Gglean002361	0.0596517	0.0624599	0	0
Gglean002362	0.08382791	0	0.136600297	0.0811517
Gglean002363	0.0605475	0	0.147828	0.234778
Gglean002364	0.630145516	0.146958246	0.361902	1.582419
Gglean002365	0.630145516	0.146958246	0.361902	1.582419
Gglean002366	0	0.0937801	0	0.346634
Gglean002367	4.71E-07	0	5.86E-07	8.34E-06
Gglean002368	0	0	0	0
Gglean002369	0	0.113951	0	0.106516
Gglean002370	0	0	0	0
Gglean002371	0	0	0	0
Gglean002372	0.0613197	0	0.0992569	0
Gglean002373	0	0	0	0.222403
Gglean002374	3.379251912	4.1087	0.176952524	0.2044922
Gglean002375	0.694036725	0.513076	0.681253014	0.566532147

Gglean002376	0	0	0	0
Gglean002377	0	0	0	0
Gglean002378	0	0	0	0.11602
Gglean002379	0	0	0.0614721	0.144915
Gglean002380	0	0	0	0
Gglean002381	0	0	0	0
Gglean002382	0	0	0.0702335	0.165732
Gglean002383	0	0	0	0
Gglean002384	0	0	0	0
Gglean002385	0	0	0	0
Gglean002386	0	0	0	0
Gglean002387	0.0509804	0.0519975	0	0.0487969
Gglean002388	1.0884024	1.589588676	1.045529705	1.401528147
Gglean002389	3.42847	2.56097	1.9457	1.71788
Gglean002390	0	0	0	0
Gglean002391	17.5188	16.1902	15.77265	13.85789
Gglean002392	106.713	109.467	139.995	141.843
Gglean002393	8.98702	15.0281	2.60378	3.37736
Gglean002394	24.63112501	28.302834	33.54743901	43.22905279
Gglean002395	9.69731115	8.835001204	2.266769262	1.844050889
Gglean002396	1.395647	2.263531	3.108217	3.129578
Gglean002397	4.95076981	4.39178659	5.52991828	6.590687897
Gglean002398	32.1554	32.8043	27.2094	25.7335
Gglean002399	4.12763	3.14629	3.48917	3.48288
Gglean002400	0	0	0	0
Gglean002401	13.92713	14.19539	10.38503	9.90598
Gglean002402	0.00722092	9.30E-05	0.000122366	0.00352781
Gglean002403	60.3357	57.414594	85.95853	100.42896
Gglean002404	0.0715432	0.0740282	0.058225	0.0689943
Gglean002405	14.10265	11.58511	12.98724	11.70748
Gglean002406	16.7625	14.0113	8.38854	8.5952
Gglean002407	4.18568	4.12792	2.63633	2.55495
Gglean002408	18.3439	15.9986	11.0559	7.82139
Gglean002409	27.3125	18.0638	12.5718	12.3286
Gglean002410	26.9954	34.304	38.5441	53.1934
Gglean002411	3.30719	2.33114	5.62228	4.91149
Gglean002412	3.117015034	2.107909113	3.42509658	4.82862281
Gglean002413	0.406759	0.420238	0	0.0979716
Gglean002414	1.395334714	2.415878004	2.511993243	2.6286911
Gglean002415	1.395334714	2.415878004	2.511993243	2.6286911
Gglean002416	12.9424	34.1499	32.0351	42.3737
Gglean002417	18.73322	19.33764	17.49892	34.1954
Gglean002418	572.296	554.56	439.062	961.788
Gglean002419	18.73322	19.33764	17.49892	34.1954

Gglean002420	1.395334714	2.415878004	2.511993243	2.6286911
Gglean002421	15.83040082	14.52410028	13.88150029	13.33770049
Gglean002422	15.83040082	14.52410028	13.88150029	13.33770049
Gglean002423	0	0	0	0
Gglean002424	0.0535084	0.055592	0.130426	0.154224
Gglean002425	13.360582	15.1185635	28.58335672	28.95491
Gglean002426	0	0.0321005	0.0250406	0
Gglean002427	9.73294	8.86195	11.6846	12.25571
Gglean002428	0	0	0	0
Gglean002429	4.301935	4.299601	2.549505	0.911363
Gglean002430	31.61694087	34.83117717	17.37663414	13.94611301
Gglean002431	7.07400456	7.59537	10.88433853	11.26687733
Gglean002432	15.334	16.5304	12.8604	13.3078
Gglean002433	16.70751	15.49473	15.89282	11.79053
Gglean002434	23.47816	24.61731	6.00094	4.039046
Gglean002435	23.47816	24.61731	6.00094	4.039046
Gglean002436	1.86856	1.37159	3.52315	2.92133
Gglean002437	28.89467462	27.17595552	21.249436	25.19063437
Gglean002438	267.461819	274.300328	289.079427	267.645534
Gglean002439	39.7736	50.6976	48.997	66.5055
Gglean002440	2.62888	2.527976	1.361187	0.6956428
Gglean002441	2.16166	3.56333	4.00441	3.69941
Gglean002442	0.701199	0.688574	0.272341	0.624886
Gglean002443	3.76983	3.04197	4.85583	4.87652
Gglean002444	38.840776	43.74805257	37.533163	40.8006081
Gglean002445	38.840776	43.74805257	37.533163	40.8006081
Gglean002446	54.65228616	58.32685689	36.23618536	31.00974594
Gglean002447	31.6167	33.3289	24.0149	22.2927
Gglean002448	41.7487	53.82220056	55.4636	59.09060026
Gglean002449	41.7487	53.82220056	55.4636	59.09060026
Gglean002450	41.7487	53.82220056	55.4636	59.09060026
Gglean002451	0	0	0	0
Gglean002452	9.050079749	9.73895277	15.27752409	16.92126205
Gglean002453	9.67747	10.17882	9.22811	11.41449
Gglean002454	0.727034459	1.825661598	1.6520154	1.234087798
Gglean002455	0.727034459	1.825661598	1.6520154	1.234087798
Gglean002456	0.727034459	1.825661598	1.6520154	1.234087798
Gglean002457	0.727034459	1.825661598	1.6520154	1.234087798
Gglean002458	1.084386006	1.407486268	1.844660204	3.443260808
Gglean002459	161	128.814	206.111	239.502
Gglean002460	0.617215	0.122790734	0.064146097	0.169967161
Gglean002461	38.3499	42.9675	22.11241	17.49317
Gglean002462	0	0	0	0
Gglean002463	0	1.06793	0.497745	0.79402

Gglean002464	0	0.310808	0	0
Gglean002465	0.357764	0.191144	0	0
Gglean002466	39.99206464	39.196749	53.066352	45.88434318
Gglean002467	79.0876	83.2601	25.64739	24.74777
Gglean002468	110.1378312	121.0518863	43.08946926	34.96976457
Gglean002469	3.698401634	5.031761339	4.285038728	6.785210883
Gglean002470	3.698401634	5.031761339	4.285038728	6.785210883
Gglean002471	3.698401634	5.031761339	4.285038728	6.785210883
Gglean002472	1.669142008	2.734677	1.893757431	2.193416335
Gglean002473	1.669142008	2.734677	1.893757431	2.193416335
Gglean002474	1.669142008	2.734677	1.893757431	2.193416335
Gglean002475	15.9418231	13.37222123	18.08945862	17.23325617
Gglean002476	0.140266168	0	0.228556161	0
Gglean002477	0	0	0	0
Gglean002478	17.72117	19.32451	5.903965	5.09345
Gglean002479	80.2917	85.0833	61.4433	56.3012
Gglean002480	0	0	0	0
Gglean002481	18.6790948	14.46780804	11.29095022	13.8748259
Gglean002482	18.6790948	14.46780804	11.29095022	13.8748259
Gglean002483	18.6790948	14.46780804	11.29095022	13.8748259
Gglean002484	18.6790948	14.46780804	11.29095022	13.8748259
Gglean002485	18.6790948	14.46780804	11.29095022	13.8748259
Gglean002486	18.6790948	14.46780804	11.29095022	13.8748259
Gglean002487	38.896	44.6127	19.83005	26.3359
Gglean002488	7.89E-17	2.45E-22	5.76E-27	3.13E-16
Gglean002489	7.89E-17	2.45E-22	5.76E-27	3.13E-16
Gglean002490	0.087691586	0.15021605	0.174623508	0.2823017
Gglean002491	0	0	0	0
Gglean002492	0	0	0	0
Gglean002493	0	0	0	0
Gglean002494	7.658522108	6.460902883	4.480142104	3.771332856
Gglean002495	7.658522108	6.460902883	4.480142104	3.771332856
Gglean002496	0	0	0	0
Gglean002497	0.0301753	0	0	0
Gglean002498	0	0	0	0.0986088
Gglean002499	6.40E-41	2.04E-44	0.0187637	0
Gglean002500	0.041781121	0.127583619	0.336377847	0.5667682
Gglean002501	126.48726	134.75285	171.48628	230.2844
Gglean002502	0	0	0	0
Gglean002503	18.676945	16.1133878	20.86627371	28.63632
Gglean002504	324.124879	410.738175	590.843261	743.94213
Gglean002505	0	0	0	0
Gglean002506	14.655007	17.455035	13.6144607	13.487769
Gglean002507	15.823076	15.601542	13.503324	12.67668

Gglean002508	15.823076	15.601542	13.503324	12.67668
Gglean002509	12.514582	12.74615	12.571678	10.343954
Gglean002510	21.0179	19.51337	19.47951	25.0998
Gglean002511	28.91	31.1752	26.8076	25.9782
Gglean002512	18.39206	20.30239	18.93525	16.96137
Gglean002513	0	0	0	0
Gglean002514	0	0	0	0
Gglean002515	17.30313801	16.55687	31.57428	36.01159
Gglean002516	122.2782001	121.0974784	210.0537438	236.9684182
Gglean002517	36.2044	38.6664	9.02732	7.56218
Gglean002518	10.84798179	10.72594202	5.522327517	6.03745
Gglean002519	76.7956813	76.04769785	74.27581913	84.45776075
Gglean002520	0	0	0	0
Gglean002521	9.76151754	8.119638473	9.689876287	12.14699
Gglean002522	0	0	0.0823401	0
Gglean002523	83.01618646	93.00498636	99.88920204	127.8022746
Gglean002524	83.01618646	93.00498636	99.88920204	127.8022746
Gglean002525	0	0	0.0593582	0
Gglean002526	40.4809461	48.7753977	47.791349	54.6141392
Gglean002527	40.4809461	48.7753977	47.791349	54.6141392
Gglean002528	41.64859758	47.2307758	53.30964	47.0542107
Gglean002529	12.3626304	11.73371539	17.83219309	22.21295558
Gglean002530	29.59506	33.66671	18.88337	17.32254
Gglean002531	6.07012	3.699682	13.38391	17.42949
Gglean002532	56.38279	56.98917	59.58583	56.79732
Gglean002533	0	0	6.51034	0
Gglean002534	161.3024	162.5007	70.4357	56.294
Gglean002535	0.101461	0.210369	0.493473357	0
Gglean002536	13.89800927	10.594978	16.59421175	13.97320253
Gglean002537	18.2105688	18.6902351	25.90981028	30.30430451
Gglean002538	8.147824941	10.299651	8.11241	8.296140267
Gglean002539	8.147824941	10.299651	8.11241	8.296140267
Gglean002540	0	0	0	0
Gglean002541	3.205610016	2.89436	1.289222	0.919191
Gglean002542	3.205610016	2.89436	1.289222	0.919191
Gglean002543	56.607738	56.415297	59.26878	61.15997607
Gglean002544	56.607738	56.415297	59.26878	61.15997607
Gglean002545	46.76922	45.39427	45.92497	51.74721
Gglean002546	55.73581269	65.13828	17.32469	15.90465
Gglean002547	55.73581269	65.13828	17.32469	15.90465
Gglean002548	33.41043	31.46874	31.85979	37.9281
Gglean002549	112.6739	120.3918	76.4438	67.184
Gglean002550	0	0	0	0
Gglean002551	24.16494	25.70886	16.14473	15.43772

Gglean002552	6.42419	7.82773	7.84187	7.46127
Gglean002553	5.34347	8.22241	6.23368	8.87658
Gglean002554	20.2695	19.4877	14.6192	13.0252
Gglean002555	24.72408	25.22714	23.68497	24.25626
Gglean002556	11.11629	8.79635	11.75677	8.01516
Gglean002557	1053.546966	918.4712636	1698.535165	1984.484539
Gglean002558	12.54966	10.95655	12.30772301	12.46012538
Gglean002559	12.54966	10.95655	12.30772301	12.46012538
Gglean002560	38.60492122	47.76885679	6.800362	6.748949986
Gglean002561	28.090947	26.86035928	35.302088	37.93178119
Gglean002562	24.85389	24.141211	12.98934	12.59428
Gglean002563	24.85389	24.141211	12.98934	12.59428
Gglean002564	0.31071	0.106946	0.168537	0.600115
Gglean002565	7.40889	12.37295	12.05136	8.4402119
Gglean002566	65.1463	69.9082	65.787	66.8251
Gglean002567	7.40889	12.37295	12.05136	8.4402119
Gglean002568	123.4617087	154.11864	157.0394	125.56891
Gglean002569	16.0189	15.0487	3.37811	2.64543
Gglean002570	123.4617087	154.11864	157.0394	125.56891
Gglean002571	16.3029	17.086	8.31712	6.58368
Gglean002572	34.55325	28.44946	19.99018	18.811945
Gglean002573	80.6723	70.82444	28.04706	24.24159098
Gglean002574	0	0	0	0
Gglean002575	50.0469513	50.74644708	53.34586056	56.23638586
Gglean002576	74.2102769	84.4387962	95.3930477	108.3125091
Gglean002577	74.2102769	84.4387962	95.3930477	108.3125091
Gglean002578	74.2102769	84.4387962	95.3930477	108.3125091
Gglean002579	74.2102769	84.4387962	95.3930477	108.3125091
Gglean002580	74.2102769	84.4387962	95.3930477	108.3125091
Gglean002581	74.2102769	84.4387962	95.3930477	108.3125091
Gglean002582	74.2102769	84.4387962	95.3930477	108.3125091
Gglean002583	74.2102769	84.4387962	95.3930477	108.3125091
Gglean002584	74.2102769	84.4387962	95.3930477	108.3125091
Gglean002585	74.2102769	84.4387962	95.3930477	108.3125091
Gglean002586	74.2102769	84.4387962	95.3930477	108.3125091
Gglean002587	74.2102769	84.4387962	95.3930477	108.3125091
Gglean002588	5.33038518	5.620871	4.94477068	6.952511742
Gglean002589	4.72243894	6.443463	4.383093839	4.13910081
Gglean002590	4.72243894	6.443463	4.383093839	4.13910081
Gglean002591	0	0	0	0
Gglean002592	60.27010225	48.63728131	64.42130251	98.07860006
Gglean002593	2.566585	2.073198001	1.135917496	0.763364733
Gglean002594	2.566585	2.073198001	1.135917496	0.763364733
Gglean002595	0	0	0	0

Gglean002596	0	0	0	0
Gglean002597	40.10463	40.8648	57.84657	73.01451
Gglean002598	40.10463	40.8648	57.84657	73.01451
Gglean002599	47.025685	50.794587	37.585701	33.971096
Gglean002600	0.0694112	0.1100023	0.0758064	0.276949
Gglean002601	11.39341789	10.88262001	17.75850197	15.75505247
Gglean002602	4.671	4.8318	2.19953	2.20476
Gglean002603	3.704299	5.835545031	8.628569917	10.21002841
Gglean002604	53.054647	57.112591	45.932583	42.393378
Gglean002605	35.9547298	36.04704819	55.5632411	70.43083
Gglean002606	12.99456456	12.61866672	12.87352302	18.066227
Gglean002607	12.99456456	12.61866672	12.87352302	18.066227
Gglean002608	12.99456456	12.61866672	12.87352302	18.066227
Gglean002609	48.565445	54.33546	26.3638516	25.16295
Gglean002610	60.9125	64.3229	42.6774	38.18
Gglean002611	7.73063948	8.67764365	12.14908966	14.252837
Gglean002612	17.068643	16.133656	11.18999048	9.1032841
Gglean002613	24.099	31.1559	24.8024	24.4672
Gglean002614	2.82696	2.90339	3.2067	3.251632
Gglean002615	9.777354	8.400877867	6.584575	7.001157467
Gglean002616	4.8719	7.94631	1.40055	0.274183
Gglean002617	16.68853551	16.52208941	19.6183976	21.287506
Gglean002618	0	0	0.062509	0
Gglean002619	0.8458358	0.767688936	1.176210798	0.751034865
Gglean002620	0.8458358	0.767688936	1.176210798	0.751034865
Gglean002621	2.070978323	1.034482155	2.747823	2.776783508
Gglean002622	1.21484041	0.547571237	1.513323249	1.108116706
Gglean002623	1.21484041	0.547571237	1.513323249	1.108116706
Gglean002624	0.186282258	0.173915994	0.235615162	0.531867631
Gglean002625	1.8011704	2.012006816	3.12236805	3.03371754
Gglean002626	0.278754	0.175278	0.306195	0.71414
Gglean002627	0.278754	0.175278	0.306195	0.71414
Gglean002628	100.7656597	119.315606	143.9491585	173.1801925
Gglean002629	100.7656597	119.315606	143.9491585	173.1801925
Gglean002630	43.72480896	43.6127167	50.90687476	51.31168014
Gglean002631	17.7605	18.78071	13.77266	11.35366
Gglean002632	21.99172865	21.626647	33.79996942	37.8133193
Gglean002633	21.99172865	21.626647	33.79996942	37.8133193
Gglean002634	3.97803	3.56731	1.79186	1.62634
Gglean002635	9.368550565	9.275110002	16.87553052	16.97560785
Gglean002636	9.368550565	9.275110002	16.87553052	16.97560785
Gglean002637	32.3863	30.6342	20.35536	17.47561
Gglean002638	16.75768563	17.369083	9.87441024	7.316104726
Gglean002639	16.75768563	17.369083	9.87441024	7.316104726

Gglean002640	14.41925	16.41581	10.5446	9.00992
Gglean002641	39.4643536	46.66579	75.028335	85.50876007
Gglean002642	22.7935	22.5638	13.7289	10.3885
Gglean002643	19.37315	18.80184	15.1908	14.51307
Gglean002644	48.10972	50.26988	55.7955	67.18952
Gglean002645	1.03588	1.70271	2.05387	3.29246
Gglean002646	0.277290605	0.461615004	0.741674	0.773094509
Gglean002647	19.7548	23.5029	14.7592	15.3007
Gglean002648	0.255312	1.64142	4.07642	1.77392
Gglean002649	8.639007932	8.109403	16.42362593	17.05981929
Gglean002650	11.313583	11.809824	15.97758941	19.70587417
Gglean002651	14.6625008	15.7309649	10.70909048	10.12868483
Gglean002652	13.15074115	14.58389984	6.468598718	5.413096754
Gglean002653	0.794434831	0.485082445	0.64303686	0.724515397
Gglean002654	37.4689	34.2984	20.6538	18.7074
Gglean002655	13.27854	15.59182	7.37415	6.560411
Gglean002656	13.27854	15.59182	7.37415	6.560411
Gglean002657	14.83744	14.48805	2.045616	0.404714
Gglean002658	19.56033	18.57803	32.0203	40.9288
Gglean002659	24.4086	26.94174	22.17841	20.7021
Gglean002660	0.479713	0.198835	0.31509	0.093622
Gglean002661	13.8258	15.7166	5.53235	5.72473
Gglean002662	8.243552645	7.065081	13.6807	12.35294816
Gglean002663	8.243552645	7.065081	13.6807	12.35294816
Gglean002664	7.11192737	6.940590238	9.891816214	11.54900173
Gglean002665	41.284	29.379	1.96075	0
Gglean002666	1.17333	1.05131	0.963182	1.396765
Gglean002667	66.44500599	69.63663	55.39922293	53.868482
Gglean002668	16.6197	16.6681	22.3712	36.9646
Gglean002669	96.86462557	96.13046847	58.55430277	49.31546554
Gglean002670	63.106358	51.01322	94.96856	104.54047
Gglean002671	21.8553	24.30647	7.76914	6.242031
Gglean002672	27.642793	26.506072	8.361118306	4.956411376
Gglean002673	27.642793	26.506072	8.361118306	4.956411376
Gglean002674	0	0	0	0
Gglean002675	0.125577037	0.12871627	0.101458389	0.144090917
Gglean002676	1.17094971	1.206576474	1.318972549	2.238756006
Gglean002677	1.17094971	1.206576474	1.318972549	2.238756006
Gglean002678	38.5476	39.5562	32.397	28.5933
Gglean002679	22.3946793	28.665825	39.56404232	48.39088202
Gglean002680	59.18952001	57.38147433	37.16704414	32.25068825
Gglean002681	2.90882	4.33206	1.60497	3.76542
Gglean002682	28.7538	33.7697	20.95867	19.47026
Gglean002683	45.18066	50.98100629	32.86921	27.42327

Gglean002684	0	0	0	0
Gglean002685	51.41063634	53.28731848	78.359272	72.35448281
Gglean002686	0	0.342835	0.134214	0.159056
Gglean002687	0	0	0	0
Gglean002688	0	0.238776	0	0
Gglean002689	0.490619	1.37499	2.27462	1.90885
Gglean002690	55.68846	60.630804	73.17375	73.30078
Gglean002691	55.68846	60.630804	73.17375	73.30078
Gglean002692	4.06313	8.04666	6.58835	4.51434
Gglean002693	467.185	500.73	725.699	861.487
Gglean002694	76.199719	65.388878	97.877475	129.62783
Gglean002695	14.36218	16.96382	4.38571	2.832529
Gglean002696	20.61424	20.79969	18.71398	16.55418
Gglean002697	0	0	0	0.186725
Gglean002698	0	0	0	0
Gglean002699	18.13693	18.34604	5.443782	4.726575
Gglean002700	18.13693	18.34604	5.443782	4.726575
Gglean002701	59.23739	61.47928	63.91353	67.14234
Gglean002702	8.29502052	8.73561	3.742527993	3.383968
Gglean002703	49.7846	54.7274	31.4483	36.5334
Gglean002704	21.6043875	23.9774743	14.56052674	12.79553292
Gglean002705	171.361	151.697	270.45	251.283
Gglean002706	17.12816	18.81346	22.20285	24.92252
Gglean002707	257.963	264.5483	247.2863	275.8453
Gglean002708	37.86336	35.7489	19.19165	15.30277
Gglean002709	318.4018694	306.4376018	276.0768608	296.1195698
Gglean002710	318.4018694	306.4376018	276.0768608	296.1195698
Gglean002711	11.856911	10.2241196	12.617154	13.79794606
Gglean002712	0	0.128754	0.100915	0
Gglean002713	0.386509	0	0.105935	0
Gglean002714	72.52410134	74.7843831	69.556844	63.395714
Gglean002715	72.52410134	74.7843831	69.556844	63.395714
Gglean002716	15.024229	15.16171901	18.24179	16.17527
Gglean002717	43.99530078	50.93117714	60.89261763	59.38232681
Gglean002718	0.000191912	0.000819735	3.03305	7.69E-05
Gglean002719	192.93447	195.2515	163.26014	168.48881
Gglean002720	4.029962372	4.296815562	6.14007586	7.297859007
Gglean002721	36.76853438	41.393467	35.967469	34.484409
Gglean002722	36.76853438	41.393467	35.967469	34.484409
Gglean002723	0	0	0	0
Gglean002724	0	0	0	0
Gglean002725	0	0	0	0
Gglean002726	18.04107849	17.34991	25.48011716	33.90417101
Gglean002727	51.47101438	61.04355	21.35090006	23.91526606

Gglean002728	10.8004	9.54037	10.94478	12.99406
Gglean002729	13.8853	14.988	11.0146	10.5501
Gglean002730	17.97691	18.04797	25.49707	27.03968
Gglean002731	20.259306	17.700324	13.56977	9.52816
Gglean002732	0	0	0	0
Gglean002733	18.6511	17.5051	11.2057	10.5893
Gglean002734	24.92091	23.73689	13.48659	14.17187
Gglean002735	9.07715	9.59664	8.16108	8.50273
Gglean002736	72.9107	73.35241	39.68599	39.05882
Gglean002737	72.9107	73.35241	39.68599	39.05882
Gglean002738	72.9107	73.35241	39.68599	39.05882
Gglean002739	15.7343154	16.93550041	27.698597	26.5272233
Gglean002740	1.680884	1.3351665	2.478802468	1.50367271
Gglean002741	1.680884	1.3351665	2.478802468	1.50367271
Gglean002742	6.70243	7.76624	4.77296	3.60941
Gglean002743	122.66587	117.88788	76.06547	78.26776
Gglean002744	122.66587	117.88788	76.06547	78.26776
Gglean002745	122.66587	117.88788	76.06547	78.26776
Gglean002746	12.2311	10.824139	17.946643	18.808995
Gglean002747	12.2311	10.824139	17.946643	18.808995
Gglean002748	19.43701431	16.7298897	26.16170904	27.784662
Gglean002749	14.5965	12.195	15.7304	15.0667
Gglean002750	84.9392	93.9856	59.3271	48.9791
Gglean002751	86.0817	76.0534	64.3621	63.8715
Gglean002752	29.40421	32.00735	49.98932	58.68123
Gglean002753	2.40E-231	2.77E-129	0	1.97E-64
Gglean002754	4.62632	5.05056	3.15681	3.39215
Gglean002755	32.2015	36.6755	18.1023	14.7853
Gglean002756	45.7699	35.3556	5.74328	2.969714
Gglean002757	6.89584	5.61504285	9.03101	9.791165596
Gglean002758	24.099	31.1559	24.8024	24.4672
Gglean002759	0.0425892	0.0874204	0.1383	0.0820398
Gglean002760	22.164834	24.273288	30.36878115	35.56338356
Gglean002761	0	0	0	0
Gglean002762	7.35289	6.21013	6.02824	5.7293
Gglean002763	0.76945408	0.902101805	2.332335	1.493266034
Gglean002764	0	0	0	0
Gglean002765	0	0	0	0
Gglean002766	20.6422	23.3757	14.9427	28.1162
Gglean002767	0	0	0	0.210549
Gglean002768	21.47366363	21.68612438	30.34153202	34.7838389
Gglean002769	21.47366363	21.68612438	30.34153202	34.7838389
Gglean002770	33.19388	42.72893	44.90973	52.66744
Gglean002771	33.19388	42.72893	44.90973	52.66744

Gglean002772	28.71228	26.37897	16.35441328	17.69022124
Gglean002773	39.9044	31.3494	35.8978	31.8734
Gglean002774	0	0	0	0
Gglean002775	0.039975	0.317952	0.128696669	0.0380642
Gglean002776	1.124693	1.055066701	1.0927012	1.064071001
Gglean002777	1.124693	1.055066701	1.0927012	1.064071001
Gglean002778	0.162833311	0.169852369	0.298341	0.389965179
Gglean002779	1.862868934	1.340888912	1.554645	2.880284
Gglean002780	1.862868934	1.340888912	1.554645	2.880284
Gglean002781	0.949788	0.417613	0.423562	0.502721
Gglean002782	62.62316	65.7460599	47.38126	44.36638
Gglean002783	1.801183	1.461603	1.779742	1.151663
Gglean002784	17.9097	15.4299	11.2688	11.3944
Gglean002785	21.59816	19.38899	22.66868	21.34818
Gglean002786	41.8222	27.9918	35.7775	40.4637
Gglean002787	0	0	0	0
Gglean002788	29.50504169	29.043339	21.226099	20.65830155
Gglean002789	169.1424	161.9227	139.818	140.8491
Gglean002790	47.25597873	47.431807	40.16984	40.16638005
Gglean002791	0.66961505	0.775086522	0.799765523	0.669177552
Gglean002792	46.36273	41.17963438	23.99986	19.630613
Gglean002793	2.78836	3.25981	4.08841	4.09035
Gglean002794	64.53837215	65.58209622	53.9870717	53.07920869
Gglean002795	23.41232792	24.081648	20.010754	19.28524054
Gglean002796	23.41232792	24.081648	20.010754	19.28524054
Gglean002797	23.41232792	24.081648	20.010754	19.28524054
Gglean002798	22.43931	21.86153	17.72511	18.86444
Gglean002799	0	0	0	0
Gglean002800	0.0676818	0.20897972	0.109930497	0.16494021
Gglean002801	0.0676818	0.20897972	0.109930497	0.16494021
Gglean002802	3.03646	0.884811	3.1785	3.25632
Gglean002803	23.59008517	23.98988766	21.34664	21.99711215
Gglean002804	23.59008517	23.98988766	21.34664	21.99711215
Gglean002805	25.4244	26.27255	22.86076201	22.079623
Gglean002806	102.5427	109.7664	66.5356	59.8774
Gglean002807	8.576463992	6.333946823	10.33664587	10.102009
Gglean002808	8.576463992	6.333946823	10.33664587	10.102009
Gglean002809	13.84771908	15.130078	7.679832	7.06597399
Gglean002810	72.51288656	75.01867767	20.09117492	19.7126746
Gglean002811	20.31253	22.4235	12.60528	12.3401
Gglean002812	22.78086	21.52346	11.79519	12.66673
Gglean002813	43.03911	42.162448	25.6109078	24.17794334
Gglean002814	32.58369571	32.65768948	46.80602059	52.99386466
Gglean002815	32.58369571	32.65768948	46.80602059	52.99386466

Gglean002816	5.678952	5.301137	9.038280108	11.63090006
Gglean002817	9.947893	8.929397	5.788159	4.84291
Gglean002818	5.397521782	6.665654289	3.467446263	2.822861524
Gglean002819	9.15902	6.12109	4.67172	5.27327
Gglean002820	37.8509	34.5551	21.72575	22.21478
Gglean002821	0.297531	0	0.244499	1.16703
Gglean002822	8.097868701	7.026136045	7.519847168	12.7037442
Gglean002823	8.097868701	7.026136045	7.519847168	12.7037442
Gglean002824	1.82671	1.0638	1.66223	1.18767
Gglean002825	5.7908098	5.408330433	10.26783208	9.558088176
Gglean002826	1.21036	1.95239	1.25341	1.79911
Gglean002827	5.7908098	5.408330433	10.26783208	9.558088176
Gglean002828	5.7908098	5.408330433	10.26783208	9.558088176
Gglean002829	5.7908098	5.408330433	10.26783208	9.558088176
Gglean002830	31.14485	31.64396	17.142866	14.415879
Gglean002831	2.24223	1.2833	1.85696	2.22454
Gglean002832	0	0	0.221847	0.0656023
Gglean002833	3.437952028	2.923605576	2.167682119	6.181636747
Gglean002834	5.9382693	6.145626294	12.172761	12.405014
Gglean002835	5.9382693	6.145626294	12.172761	12.405014
Gglean002836	0	0.664392	0	0
Gglean002837	0.0250799	0	0.16246	0.0479583
Gglean002838	0	0	0	0.0358542
Gglean002839	0	0	0	0
Gglean002840	0.046231172	0.265174314	0.150863075	0.210816774
Gglean002841	0.046231172	0.265174314	0.150863075	0.210816774
Gglean002842	0	0.1845862	0.113986137	0
Gglean002843	0.38348368	0.349994631	1.436629	1.180984
Gglean002844	1232.3391	1333.5153	1311.9418	2204.634
Gglean002845	1232.3391	1333.5153	1311.9418	2204.634
Gglean002846	1.120518204	1.336735	0.185839718	0.626712893
Gglean002847	10.76	5.11681	5.12043	6.67261
Gglean002848	22.42392	22.41475	14.56826	14.96972
Gglean002849	0.198055	0.209588	0	0.192448
Gglean002850	108.35779	116.28507	85.32508	89.26221
Gglean002851	0	0	0	0
Gglean002852	67.0636	81.2687	137.3854	139.2392
Gglean002853	29.74473	33.07366	14.76178	12.531
Gglean002854	0.1251	0	0.0507426	0.241247
Gglean002855	84.3386	80.1884	67.446	80.6659
Gglean002856	0	0	0	0
Gglean002857	0	0	0	0
Gglean002858	0	0	0	0
Gglean002859	0.728593204	0.916148546	0.132261962	0.351471205

Gglean002860	0.0915957	0.0470723	0.258863	0.131467
Gglean002861	0.173210588	0.0434343	0.465365381	0.241397176
Gglean002862	0.059006165	0.184312492	0.215537826	0.33836485
Gglean002863	0	0	0	0
Gglean002864	6.944245	7.133165	8.7355	9.222627
Gglean002865	25.269601	23.87592066	39.9687	33.2157
Gglean002866	86.43803688	100.27702	112.25993	115.817477
Gglean002867	22.24208	24.031181	35.20381124	43.33462723
Gglean002868	16.398532	15.277084	17.33848	19.82534
Gglean002869	22.24208	24.031181	35.20381124	43.33462723
Gglean002870	1512.685011	1159.5332	1340.307568	1932.271005
Gglean002871	153.593882	168.31355	70.601641	55.90641959
Gglean002872	35.83699	31.4021	29.73114	32.07247
Gglean002873	29.125194	28.68881	15.5549913	14.93749105
Gglean002874	0	0	0	0.0957085
Gglean002875	0	0	0	0
Gglean002876	0	0	0	0
Gglean002877	0.092806306	0	0	0
Gglean002878	0.214714	0.0738203	0.175406	0.0697938
Gglean002879	0	0	0	0
Gglean002880	0.123364	0.0550884	0.204062	0.0711051
Gglean002881	0	0	0	0
Gglean002882	0	0	0	0
Gglean002883	0	0.0818142	0	0
Gglean002884	0	1.07185	0	0
Gglean002885	0	0.118049	0	0.326731
Gglean002886	0	0	0	0
Gglean002887	16.74062733	18.14412167	14.20770858	11.42062687
Gglean002888	0	0	0	0
Gglean002889	0	0	0	0
Gglean002890	37.63506104	37.8647351	43.466672	45.001115
Gglean002891	16.95061	18.76405	7.54237	8.24207
Gglean002892	16.95061	18.76405	7.54237	8.24207
Gglean002893	78.8385	73.7953	75.6053	80.8137
Gglean002894	172.5035	159.2138	56.6229	41.036326
Gglean002895	0.188128	0.0779308	0.3655729	0.181578
Gglean002896	0	0	0	0
Gglean002897	28.6704	26.6521	18.511	18.4863
Gglean002898	36.4119	49.0495	76.0823	56.5594
Gglean002899	17.919786	19.251532	14.66591	15.518854
Gglean002900	0	0	0	0
Gglean002901	0	0	0	0
Gglean002902	0	0	0	0
Gglean002903	0	0	0	0

Gglean002904	0.0763894	0.0394008	0	0
Gglean002905	0	0	0	0
Gglean002906	0.0579555	0	0	0
Gglean002907	0	0	0	0
Gglean002908	0.452604	6.44E-07	5.90E-05	0.199243
Gglean002909	0.495054	1.18069	1.23287	2.15131
Gglean002910	3.21979	4.36712	5.32494	6.20928
Gglean002911	0.000114994	0.230474061	0.005457303	1.54E-06
Gglean002912	0.000114994	0.230474061	0.005457303	1.54E-06
Gglean002913	0.15087	0.486027	0.921347985	0.990216225
Gglean002914	0	0.480158	0.369633	0
Gglean002915	5.76E-07	2.29E-05	3.97E-07	2.14E-06
Gglean002916	4.40994	3.98405	5.96983	5.15624
Gglean002917	6.74406279	7.019247505	6.993794053	7.805740181
Gglean002918	0.595323	1.00234	0.499833	0.511886
Gglean002919	0.6263188	0.431368246	0.485626823	0.594512643
Gglean002920	0.6263188	0.431368246	0.485626823	0.594512643
Gglean002921	18.70733	17.24885	11.8217	11.038615
Gglean002922	0.270091	0	0.45001	0.399584
Gglean002923	0.404257	0.103892	0.481925	0.608138
Gglean002924	8.54E-21	1.52E-30	8.72E-45	1.45E-29
Gglean002925	37.9463321	40.2848468	20.27601	17.3363416
Gglean002926	44.11755	50.74678	47.66508	47.85594
Gglean002927	88.465808	94.75059	61.00842208	55.61581203
Gglean002928	88.465808	94.75059	61.00842208	55.61581203
Gglean002929	16.92172	20.25909	16.86455	14.46492
Gglean002930	35.391453	37.70753	46.714595	54.86022
Gglean002931	16.66883238	19.48362699	2.175472438	1.1408748
Gglean002932	8.068035	10.035999	15.95106	23.125447
Gglean002933	7.03813	6.72361	1.92254	1.42761
Gglean002934	0	0.268207	0	0
Gglean002935	0.0861296	0	0.208429	0.50247
Gglean002936	0	0	0	0
Gglean002937	0.0605553	0.187324	0	0
Gglean002938	0	0	0	0
Gglean002939	0	0.0549913	0	0
Gglean002940	383.4942	381.555912	373.8901605	463.504188
Gglean002941	383.4942	381.555912	373.8901605	463.504188
Gglean002942	383.4942	381.555912	373.8901605	463.504188
Gglean002943	0	0	0	0
Gglean002944	2.87115044	3.394122	6.252714	6.9881787
Gglean002945	47.2671	50.373	24.6425	19.5807
Gglean002946	55.4137	68.3818	45.2805	44.5222
Gglean002947	3.34930403	3.786041421	4.063021549	3.039564857

Gglean002948	3.34930403	3.786041421	4.063021549	3.039564857
Gglean002949	1.01136	0.561955	0.58481	0.332392
Gglean002950	0	0	0.134912	0.159931
Gglean002951	0	0	0	0
Gglean002952	0	0.0452294	0	0.0417203
Gglean002953	0.0457216	0.0476179	0	0
Gglean002954	0	0	0	0
Gglean002955	1.0905	1.0414	0.798255	0.575788
Gglean002956	0	0	0	0
Gglean002957	32.86729	28.67741	42.01523	44.94013
Gglean002958	16.49109	16.88523	10.36333	11.26889
Gglean002959	29.3632069	19.01401499	40.527572	61.381771
Gglean002960	29.3632069	19.01401499	40.527572	61.381771
Gglean002961	8.72933	5.55487	9.02893	14.00306
Gglean002962	35.13522	31.37826	37.64427	44.96169
Gglean002963	0	0	0	0
Gglean002964	54.1707	58.7581	40.0544	35.8316
Gglean002965	6.59605	5.84012	4.5555	3.45351
Gglean002966	71.1527	84.5558	63.9489	109.385
Gglean002967	24.2358	26.1206	19.958	37.6899
Gglean002968	427.215	482.31	465.306	972.918
Gglean002969	0	0	0	0
Gglean002970	26.61947502	30.40611673	24.833579	22.731485
Gglean002971	93.91007114	104.99989	97.32100967	102.2256724
Gglean002972	11.68358178	10.46028282	17.61910344	20.05348887
Gglean002973	30.9015	31.0721	23.302	23.1476
Gglean002974	49.60618	52.95684536	26.97667	23.0138799
Gglean002975	48.32117	44.08769	40.80378	40.60164
Gglean002976	48.32117	44.08769	40.80378	40.60164
Gglean002977	397.628	381.8802	363.2677	379.662
Gglean002978	14.8585	13.29872	25.37344	28.3853
Gglean002979	0	0	0	0
Gglean002980	0.2130857	0.1485148	0.041889403	0.074535925
Gglean002981	0.2130857	0.1485148	0.041889403	0.074535925
Gglean002982	19.67314289	17.63499257	21.07739066	23.655803
Gglean002983	19.67314289	17.63499257	21.07739066	23.655803
Gglean002984	19.67314289	17.63499257	21.07739066	23.655803
Gglean002985	448.501	450.237	426.81	509.137
Gglean002986	363.4158136	369.8620367	534.4192817	581.7312424
Gglean002987	15.28585006	17.483013	17.80180001	19.4465987
Gglean002988	53.12563	51.73309	39.395244	39.75516
Gglean002989	5.37397	6.59948	6.13343	13.7431
Gglean002990	30.3649	40.4012	26.1838	34.0568
Gglean002991	61.3501	51.9645	38.41981	43.8994

Gglean002992	25.38026	27.98371	7.37197	4.98125
Gglean002993	2.444824	2.557825793	0.996717301	0.831649001
Gglean002994	2.444824	2.557825793	0.996717301	0.831649001
Gglean002995	3.52545	4.23897	1.82496	2.13595
Gglean002996	10.8607	0.691757	2.48814	6.04448
Gglean002997	1.515131492	1.3398981	0.704154	0.414456
Gglean002998	15.85538	12.59006	9.00944	11.27078
Gglean002999	76.35228	102.45459	128.69169	135.23706
Gglean003000	0	0	0.115314	0
Gglean003001	76.35228	102.45459	128.69169	135.23706
Gglean003002	7.92818	8.55224	5.61695	3.67684
Gglean003003	0	0	0	0
Gglean003004	0	0	0	0
Gglean003005	0	0	0	0
Gglean003006	0	0	0	0
Gglean003007	0.0573804	0	0	0
Gglean003008	0	0	0.531587	0
Gglean003009	0.0996815	0	0.0403715	0.0958851
Gglean003010	0	0	0	0
Gglean003011	0	0	0	0
Gglean003012	0	0	0	0
Gglean003013	0	0	0	0
Gglean003014	0	0	0.0373172	0
Gglean003015	0.864395	0.538718032	0.438602158	1.064044
Gglean003016	0.000194829	2.72E-06	0.000103015	0.0611729
Gglean003017	0	0	0	0
Gglean003018	0	0	0	0
Gglean003019	0.574498	0.75651	0.351445	0.139604
Gglean003020	1.23628	0.584596	1.47622	1.53309
Gglean003021	0	0	0	0
Gglean003022	0	0.0971123	0	0
Gglean003023	0	0	0	0
Gglean003024	0.0892476	0	0.0732452	0
Gglean003025	0	0.0382228	0.0304073	0.0720894
Gglean003026	0	0.0208128	0	0
Gglean003027	0	0	0	0
Gglean003028	0	0	0	0
Gglean003029	0	0	0.12868	0
Gglean003030	0	0	0	0
Gglean003031	0	0	0	0
Gglean003032	0	0	0	0
Gglean003033	0	0	0	0
Gglean003034	15.1479	17.1437	8.33498	9.12685
Gglean003035	25.2612	34.3716	11.071	13.691

Gglean003036	18.26671	17.82783	10.18452	11.84192
Gglean003037	5.948867	8.18415769	2.5035664	2.873225922
Gglean003038	5.948867	8.18415769	2.5035664	2.873225922
Gglean003039	31.6726747	35.0297481	26.5798368	21.60719143
Gglean003040	105.5788198	104.360065	152.06701	141.2644901
Gglean003041	35.54109	34.32015	41.3664	50.17649
Gglean003042	16.29818	16.64858	20.82719	19.44213
Gglean003043	53.55684395	53.12372392	29.41797534	26.67418909
Gglean003044	0.905011	0.498974	0	1.79078
Gglean003045	11.30847162	10.72274472	14.58834568	18.61769428
Gglean003046	10.34144826	13.05009597	0.300701945	0.3466139
Gglean003047	10.34144826	13.05009597	0.300701945	0.3466139
Gglean003048	52.6823	65.0603	76.7254	121.404
Gglean003049	24.458931	26.2885424	14.51475873	12.31936508
Gglean003050	54.258	47.8305	33.2761	28.716
Gglean003051	36.3568	37.85802	24.9597	24.66045
Gglean003052	36.3568	37.85802	24.9597	24.66045
Gglean003053	0.060883176	0.0628237	0.0493476	0.52252
Gglean003054	35.3328	38.1468	34.5825	28.0966
Gglean003055	146.1352	156.16273	50.8469727	33.89979916
Gglean003056	17.29123	17.78512	12.90577	10.73455
Gglean003057	44.88806802	46.10976799	47.64758294	43.82117902
Gglean003058	5.21656	5.30822	2.45332	2.41805
Gglean003059	15.910107	13.17522677	13.25521435	10.775188
Gglean003060	15.910107	13.17522677	13.25521435	10.775188
Gglean003061	15.910107	13.17522677	13.25521435	10.775188
Gglean003062	48.42219	45.76987	32.54894	34.15651
Gglean003063	0	0	0	0
Gglean003064	0	0	0	0
Gglean003065	0	0	0	0
Gglean003066	0.266044	0.091891	0.431635	0.170238
Gglean003067	0.129593608	0.0369938	0.035020858	0.0345047
Gglean003068	0	0	0	0
Gglean003069	104.0660493	108.1610438	118.9186	86.278
Gglean003070	12.064042	12.66308	4.203867	3.569073381
Gglean003071	16.0359	18.3952	14.3817	12.287
Gglean003072	1309.79843	1354.62085	864.15567	816.1611258
Gglean003073	5.98715582	3.973756	0.722214602	0.630433
Gglean003074	5.98715582	3.973756	0.722214602	0.630433
Gglean003075	5.98715582	3.973756	0.722214602	0.630433
Gglean003076	7.51E-14	1.55E-283	0.721333	2.14E-05
Gglean003077	61.4728	64.0935	56.5298	51.5247
Gglean003078	1.24064	2.35516	2.3953	3.18616
Gglean003079	1.6039	1.69773	0.517422	0.511563

Gglean003080	0	1.34166	1.51231	0.602922
Gglean003081	1.77765272	2.951932693	1.17270082	0.951160233
Gglean003082	2.328961487	3.684297002	4.791942315	4.678197002
Gglean003083	2.328961487	3.684297002	4.791942315	4.678197002
Gglean003084	3.86371	1.38066	0.723762	0.558808
Gglean003085	76.54584609	80.669664	79.00617	68.97716
Gglean003086	2.62383	2.110176	3.167261	3.75187
Gglean003087	6.40262	5.64881	6.42396	11.2181
Gglean003088	0	0.333292033	1.022720338	0.611454757
Gglean003089	0.388665	0.297715	0.555769	0.569737
Gglean003090	212.518	182.928	291.982	482.745
Gglean003091	68.2643989	81.3131205	72.65851505	115.0250924
Gglean003092	68.2643989	81.3131205	72.65851505	115.0250924
Gglean003093	68.2643989	81.3131205	72.65851505	115.0250924
Gglean003094	68.2643989	81.3131205	72.65851505	115.0250924
Gglean003095	68.2643989	81.3131205	72.65851505	115.0250924
Gglean003096	1.321966524	1.029518	1.196042	1.115958069
Gglean003097	11.67549609	10.79002	6.240154042	5.948949092
Gglean003098	69.8198	66.817	61.9807	65.2601
Gglean003099	0	0	0	0
Gglean003100	5.83246	5.76352	3.49007	3.21714
Gglean003101	0.077669272	0.082324462	0.301707211	0.014052
Gglean003102	0.077669272	0.082324462	0.301707211	0.014052
Gglean003103	0.122202327	0	0	0.030166432
Gglean003104	0.195708531	0.609320693	0.184095909	0.092408267
Gglean003105	0.195708531	0.609320693	0.184095909	0.092408267
Gglean003106	83.57853	85.84357	106.65711	123.88577
Gglean003107	8.132283006	6.629413784	12.43323958	11.9453422
Gglean003108	0	0	0	0
Gglean003109	0	0	0	0
Gglean003110	7.774474934	8.404163287	5.60186777	6.186842408
Gglean003111	2.292428	2.302514	1.825117116	2.210458712
Gglean003112	0	0.0902673	0	0
Gglean003113	0	0	0	0
Gglean003114	0	0.394539	0.598662	0.358487
Gglean003115	0	0	0	0
Gglean003116	0	0	0	0
Gglean003117	0	0	0.166605	0.0982899
Gglean003118	0	0	0.128133	0
Gglean003119	0	0	0	0
Gglean003120	0	0	0	0
Gglean003121	0	0	0	0
Gglean003122	0	0	0	0
Gglean003123	0	0	0	0

Gglean003124	0	0	0	0
Gglean003125	0	0	0	0
Gglean003126	0	0.19626	1.356510825	0
Gglean003127	31.31422	41.24163	60.78378	73.73629
Gglean003128	28.7787	24.9865	24.2889	26.417
Gglean003129	31.31422	41.24163	60.78378	73.73629
Gglean003130	15.786925	16.644231	14.7411	13.28487
Gglean003131	15.786925	16.644231	14.7411	13.28487
Gglean003132	0.0709574	0	0	0
Gglean003133	0	0	0	0
Gglean003134	0	0	0	0.613873
Gglean003135	0.33003	0.33521709	0.1445189	0.471179
Gglean003136	0	0	0	0
Gglean003137	0	0	0	0
Gglean003138	0	0	0	0
Gglean003139	0.245824	0.10012	0.159644	0.094821
Gglean003140	0	0.103436	0	0.190313
Gglean003141	0	0	0	0
Gglean003142	0	0	0	0
Gglean003143	0	0	0	0
Gglean003144	0	0	0	0
Gglean003145	0	0	0	0
Gglean003146	0	0	0	0
Gglean003147	0	0	0	0
Gglean003148	0	0	0	0
Gglean003149	0	0	0	0
Gglean003150	0	0	0	0
Gglean003151	0	0.0326219	0	0
Gglean003152	0	0	0	0
Gglean003153	0	0	0	0
Gglean003154	0	0	0	0
Gglean003155	0.0703108	0.110285	0.171912	0
Gglean003156	0	0	0	0
Gglean003157	0	0	0	0
Gglean003158	0	0	0.144158	0
Gglean003159	0	0	0	0
Gglean003160	0	0	0	0
Gglean003161	0.184931	0	0.301617	0
Gglean003162	0	0	0	0
Gglean003163	0	0	0	0
Gglean003164	0	0	0	0
Gglean003165	0	0	0	0
Gglean003166	0	0	0	0
Gglean003167	0	0	0	0

Gglean003168	5.23624	0	2.32361	1.47E-13
Gglean003169	10.67298	8.98387	5.95587	4.95284
Gglean003170	115.711	121.738	63.1189	77.8893
Gglean003171	0.0452123	0.0935526	0	0.0442698
Gglean003172	3.98192	3.44091	4.53831	5.42078
Gglean003173	59.67401505	64.015533	78.37801215	73.28272158
Gglean003174	0	0	0	0
Gglean003175	55.77173537	47.43653488	59.07533	78.1966816
Gglean003176	72.85321002	72.76334238	75.13732052	66.02987362
Gglean003177	50.644015	41.09828	37.923204	41.00319
Gglean003178	50.644015	41.09828	37.923204	41.00319
Gglean003179	1.71773	2.03063	2.93763	2.69851
Gglean003180	1.58328	1.02252	1.25141	2.45881
Gglean003181	0	0	0.0751889	0
Gglean003182	57.37828415	53.16383463	65.72523865	63.51889658
Gglean003183	61.29135003	61.627911	61.3957047	65.34208001
Gglean003184	61.29135003	61.627911	61.3957047	65.34208001
Gglean003185	75.42641203	82.71240445	61.22589167	61.42720851
Gglean003186	0.362983558	0.663850678	0.519462172	0.926981905
Gglean003187	28.3886	30.0992	18.56919	16.23805
Gglean003188	9.8363	8.62982	5.909562	4.038026757
Gglean003189	218.2083	225.6291713	167.6561289	166.4483397
Gglean003190	41.17443	47.445424	34.228136	35.047252
Gglean003191	16.838301	19.05393587	6.0767109	5.786967682
Gglean003192	16.838301	19.05393587	6.0767109	5.786967682
Gglean003193	2.205091739	5.708345206	4.31059428	4.15851551
Gglean003194	2.205091739	5.708345206	4.31059428	4.15851551
Gglean003195	0	0	0	0
Gglean003196	93.09642669	108.0247155	75.00700123	65.82325784
Gglean003197	93.09642669	108.0247155	75.00700123	65.82325784
Gglean003198	4.693245814	5.033753177	9.03626118	10.304906
Gglean003199	1.403128945	1.447097858	2.517105953	2.997163456
Gglean003200	14.34557	16.603262	4.086392	4.247568603
Gglean003201	108.9428867	114.8609104	129.6042754	217.4165059
Gglean003202	108.9428867	114.8609104	129.6042754	217.4165059
Gglean003203	108.9428867	114.8609104	129.6042754	217.4165059
Gglean003204	0	0	0	0
Gglean003205	26.60109948	27.45741201	19.3171565	21.68525228
Gglean003206	0	0	0	0
Gglean003207	2.654436	1.75261	1.87277	1.46174
Gglean003208	28.89128	36.4446542	31.62694061	34.8587913
Gglean003209	1.402422	1.527554	2.850399	2.71234
Gglean003210	5.99E-06	7.39E-09	3.83E-07	0.140751
Gglean003211	203.78987	194.69029	137.727753	131.10029

Gglean003212	36.01342	39.29993	39.30078	35.39395
Gglean003213	61.2588	60.2864	45.6382	39.8507
Gglean003214	6.109936	6.765141	9.80326	11.25372
Gglean003215	12.67565446	10.93332565	13.38236027	19.10004561
Gglean003216	12.67565446	10.93332565	13.38236027	19.10004561
Gglean003217	25.1138	24.3671	39.1804	48.0678
Gglean003218	14.06299	16.94522	9.09769	8.61501
Gglean003219	33.6077	40.678	26.2406	29.5232
Gglean003220	45.87458211	44.3564253	23.90842144	24.64073042
Gglean003221	45.87458211	44.3564253	23.90842144	24.64073042
Gglean003222	45.87458211	44.3564253	23.90842144	24.64073042
Gglean003223	13.60632343	14.21466015	16.47417524	19.4430635
Gglean003224	11.12845543	11.41448295	14.845876	17.9846791
Gglean003225	11.12845543	11.41448295	14.845876	17.9846791
Gglean003226	11.12845543	11.41448295	14.845876	17.9846791
Gglean003227	18.55319	19.76171277	25.089685	22.092306
Gglean003228	2.046738	4.18392	4.42816	6.3521
Gglean003229	42.44381	39.22319893	39.4953	34.58879
Gglean003230	1.976998154	2.182436465	2.40291182	3.010744326
Gglean003231	11.09284507	10.64721	14.24376	15.69246
Gglean003232	28.96882	25.05271	21.384477	40.94189
Gglean003233	6.25928	6.67581	7.65302	8.18183
Gglean003234	1.0692883	1.15194556	0.904885673	1.038591685
Gglean003235	1.044261976	1.00002306	1.603072305	1.984225066
Gglean003236	3.080601521	5.835177633	5.264106795	6.541669729
Gglean003237	36.73321	33.72610999	44.529308	44.199083
Gglean003238	12.79180629	14.28853695	23.4827744	22.860588
Gglean003239	12.79180629	14.28853695	23.4827744	22.860588
Gglean003240	12.79180629	14.28853695	23.4827744	22.860588
Gglean003241	12.79180629	14.28853695	23.4827744	22.860588
Gglean003242	15.60972	15.89014	8.12763	5.97684
Gglean003243	14.14464174	17.632914	15.73983906	12.91753447
Gglean003244	0	0	0	0
Gglean003245	0	0.211279	0.378215	0.398353
Gglean003246	58.22699	55.08936	49.35674001	51.16966
Gglean003247	17.570781	20.53411	23.663201	26.313614
Gglean003248	27.32759	25.7721	30.26055	29.45294
Gglean003249	26.7353	28.7711	16.50767	15.70151
Gglean003250	3.097309183	3.154377	2.717519	3.73061415
Gglean003251	92.0855	98.2329	113.0256	101.6133
Gglean003252	101.6660623	120.6321	113.69105	106.8860751
Gglean003253	9.948707	13.38008	12.0117	18.48115
Gglean003254	47.26115	45.37101	35.1907	31.04571
Gglean003255	47.26115	45.37101	35.1907	31.04571

Gglean003256	47.26115	45.37101	35.1907	31.04571
Gglean003257	47.26115	45.37101	35.1907	31.04571
Gglean003258	0	0	0	0
Gglean003259	0	0	0	0
Gglean003260	22.385852	18.887641	14.806452	12.15573082
Gglean003261	38.55	37.4543	39.306	37.5218
Gglean003262	2.765308	3.3659	4.71031	5.87628
Gglean003263	24.366638	33.21261002	21.34500481	19.35648279
Gglean003264	7.16568	7.928651	2.1038041	1.763931
Gglean003265	99.9941	94.7043	55.6567	48.9818
Gglean003266	29.07936603	31.44683431	29.49840281	29.11496164
Gglean003267	26.65891248	31.98084901	29.49892743	29.09537054
Gglean003268	55.70177	55.521358	48.485667	45.66769
Gglean003269	55.70177	55.521358	48.485667	45.66769
Gglean003270	55.70177	55.521358	48.485667	45.66769
Gglean003271	19.3456	25.0586	25.9079	27.2793
Gglean003272	15.28982013	15.59810902	12.422066	12.00909675
Gglean003273	14.53328	42.81423	9.97988	8.63482
Gglean003274	16.46826	20.18218	14.9176	9.185
Gglean003275	10.4991	10.87593	9.3013	7.53843
Gglean003276	11.15055	12.94105	16.71313	18.64329
Gglean003277	1.48792	1.4595	0.0614364	1.43404
Gglean003278	0.786787405	0.96991381	1.140430003	1.678430076
Gglean003279	10.23436977	10.99108406	7.192739799	5.350357718
Gglean003280	10.23436977	10.99108406	7.192739799	5.350357718
Gglean003281	3.505054525	3.973998495	3.66776516	5.759033346
Gglean003282	10.6931	12.0998	3.63631	1.62757
Gglean003283	0	0	0	0
Gglean003284	13.07238	10.57592125	18.94931061	21.19026
Gglean003285	89.3774	90.7159	86.29558	83.55105
Gglean003286	0	0	0	0
Gglean003287	0	0	0	0
Gglean003288	17.06117	18.09859	17.12625	17.28425
Gglean003289	277.68096	213.8404	593.38545	653.659
Gglean003290	28.7545	26.7211	8.1061	6.39956
Gglean003291	22.62001754	21.119789	20.49861	20.82534
Gglean003292	6.011316	8.3124	9.87104	7.61358
Gglean003293	62.2547	66.5853	67.6364	73.1803
Gglean003294	0.551265	0	0.152503	0.181212
Gglean003295	101.594939	99.6014107	87.97750125	87.534073
Gglean003296	101.594939	99.6014107	87.97750125	87.534073
Gglean003297	0	0	0	0
Gglean003298	11.62982424	12.685845	6.816135	5.832426
Gglean003299	0	0	0	0

Gglean003300	0	0.139225	0	0.1294
Gglean003301	0	0.161007	0.12602	0.149143
Gglean003302	9.20944	12.8759	12.8226	7.96529
Gglean003303	12.7431457	14.807282	19.83384	22.85157
Gglean003304	44.83286	39.94558	58.63252	58.87599
Gglean003305	28.91988055	32.96007131	22.42447674	20.873469
Gglean003306	28.91988055	32.96007131	22.42447674	20.873469
Gglean003307	97.3255	99.7958	105.8021	103.4318
Gglean003308	0	0.189437	0	0
Gglean003309	0.339378286	0.142158	0.599480001	0.6122472
Gglean003310	0.339378286	0.142158	0.599480001	0.6122472
Gglean003311	99.89162733	115.1950814	57.44436541	54.779728
Gglean003312	99.89162733	115.1950814	57.44436541	54.779728
Gglean003313	21.9922825	22.49733	14.818929	13.52416
Gglean003314	21.9922825	22.49733	14.818929	13.52416
Gglean003315	17.0713	19.3786	19.1328	18.0184
Gglean003316	14.048619	15.085117	21.858344	21.151226
Gglean003317	46.46856562	48.48085552	38.4870977	36.81703
Gglean003318	46.46856562	48.48085552	38.4870977	36.81703
Gglean003319	8.061534833	8.270434	11.32339172	12.84315877
Gglean003320	84.8602	92.3759	108.3563	131.0246
Gglean003321	98.42468317	97.39396891	130.5105819	155.4618762
Gglean003322	98.42468317	97.39396891	130.5105819	155.4618762
Gglean003323	98.42468317	97.39396891	130.5105819	155.4618762
Gglean003324	0.177319	0.123178	0.192474	0.398335
Gglean003325	62.13036	65.02298	42.0386	38.09442
Gglean003326	0	0	0	0
Gglean003327	0	0	0	0
Gglean003328	23.48154869	24.23334434	25.18268732	23.40353428
Gglean003329	27.31349443	29.89861794	22.50785401	22.34040511
Gglean003330	6.73943	5.02151	4.21484	4.24868
Gglean003331	0	0	0	0
Gglean003332	0	0	0	0
Gglean003333	56.26784	56.15810651	53.0405493	84.462209
Gglean003334	5.71419	6.32364	2.33215	3.45969
Gglean003335	0	0	0	0
Gglean003336	20.333274	22.651196	30.298663	36.930794
Gglean003337	57.12115	60.43052	96.53637	100.68525
Gglean003338	39.93142	41.42823	40.3268	45.24912
Gglean003339	39.93142	41.42823	40.3268	45.24912
Gglean003340	21.09199404	20.74772698	20.27023836	21.66812785
Gglean003341	0.271057	0	0.222233	0
Gglean003342	43.1045	46.14427	69.17734	76.51923
Gglean003343	6.345574	8.9254	5.5303	5.624534

Gglean003344	0	0	0	0
Gglean003345	22.57784595	25.79374978	20.39130876	20.55876743
Gglean003346	22.57784595	25.79374978	20.39130876	20.55876743
Gglean003347	16.75101756	18.61881976	4.39493276	3.756403
Gglean003348	76.98153	77.41324	73.27239	76.40469
Gglean003349	15.619193	14.800594	12.64647884	13.153511
Gglean003350	36.617343	35.72080911	26.531219	25.9486
Gglean003351	29.34196905	33.82800991	36.69368333	34.62613042
Gglean003352	9.66658	5.87448	5.88671	6.64214
Gglean003353	44.2416559	40.20810217	24.36416436	20.089688
Gglean003354	44.2416559	40.20810217	24.36416436	20.089688
Gglean003355	44.2416559	40.20810217	24.36416436	20.089688
Gglean003356	22.386659	27.39371831	27.1218	27.75123185
Gglean003357	22.386659	27.39371831	27.1218	27.75123185
Gglean003358	4.83523	2.12543	5.56738	7.82875
Gglean003359	34.62377216	35.179697	21.18428894	21.21785183
Gglean003360	8.422623109	8.629514399	10.59890513	13.42410398
Gglean003361	0.201970608	0.447866848	0.309837307	0.450493807
Gglean003362	0.448478955	0.81567792	0.502451697	0.886702682
Gglean003363	39.30284453	38.84779743	41.66063646	40.90242131
Gglean003364	39.30284453	38.84779743	41.66063646	40.90242131
Gglean003365	54.8302858	63.86912725	44.08616923	39.094657
Gglean003366	28.64407517	30.25135797	38.33198528	36.11967027
Gglean003367	28.64407517	30.25135797	38.33198528	36.11967027
Gglean003368	0	0	0	0
Gglean003369	0.425119164	0.270891964	0.041054283	0.237552913
Gglean003370	120.5674	126.1236	163.4802	174.3974
Gglean003371	0.162344	0.172034	0	0
Gglean003372	65.1804105	55.452	34.9892223	29.4455194
Gglean003373	49.47564	46.49168	29.55657	24.7037
Gglean003374	0.281266	0.345191024	0.370925012	0.270186
Gglean003375	0	0	0.0379689	0
Gglean003376	0.227803529	0.273561	0.129672067	0.131689377
Gglean003377	0	0	0	0
Gglean003378	0	0.115874	0.0912	0
Gglean003379	37.87748402	38.37107616	33.16356672	29.81371272
Gglean003380	37.87748402	38.37107616	33.16356672	29.81371272
Gglean003381	33.76413	33.32271	32.38874	33.51540088
Gglean003382	43.46418	40.09134	24.30657997	24.22574
Gglean003383	0	0	0	0
Gglean003384	22.87867243	23.6202175	33.02101766	43.83403781
Gglean003385	22.87867243	23.6202175	33.02101766	43.83403781
Gglean003386	22.87867243	23.6202175	33.02101766	43.83403781
Gglean003387	0.203401	0.104254	0.586057	0.595174

Gglean003388	0.2748975	0.0786094	0.276379	0.0723188
Gglean003389	0.252617	0.264792	0	0
Gglean003390	0	0.0298607	0	0
Gglean003391	78.746103	80.91589169	80.34426252	60.73584908
Gglean003392	78.746103	80.91589169	80.34426252	60.73584908
Gglean003393	54.3763	62.0166	0.85504	0.197740054
Gglean003394	1.91843	0.000652499	1.68404	0.0264285
Gglean003395	61.26989865	61.594718	52.41064825	42.56568872
Gglean003396	0	0	0	0
Gglean003397	34.464987	38.136627	34.45819	34.22541
Gglean003398	14.93853	15.141	16.14357	20.27589
Gglean003399	39.48621	42.31103	23.47164	21.53839901
Gglean003400	7.37301101	8.01115	8.529174	8.81775
Gglean003401	7.37301101	8.01115	8.529174	8.81775
Gglean003402	45.04118	38.22598	58.4294	52.7043
Gglean003403	22.15324	25.21281	29.91523	32.33509
Gglean003404	0.0761747	0.116181	0.152406	0.0720781
Gglean003405	0.17675	0.287182	0.255033	0.234453
Gglean003406	31.75226378	29.1684809	26.5264656	24.93677699
Gglean003407	31.75226378	29.1684809	26.5264656	24.93677699
Gglean003408	318.94072	438.8916058	498.4353112	616.9146194
Gglean003409	318.94072	438.8916058	498.4353112	616.9146194
Gglean003410	7.92559	10.38157	11.34714	15.189
Gglean003411	37.21960372	36.735287	35.04445133	35.97450996
Gglean003412	0	1.41176	0	1.235
Gglean003413	98.93209637	103.3717867	79.39058221	76.50124176
Gglean003414	98.93209637	103.3717867	79.39058221	76.50124176
Gglean003415	0	0	0	0.052454899
Gglean003416	73.03631	56.41267	24.9434	23.93934
Gglean003417	72.81894671	72.90704	50.995011	39.95518501
Gglean003418	0	0	0	0
Gglean003419	0	0	0	0
Gglean003420	0	0	0	0
Gglean003421	28.5895891	51.49673	30.768918	35.395691
Gglean003422	28.5895891	51.49673	30.768918	35.395691
Gglean003423	0.027316028	0.0978358	0.088553106	0.137914299
Gglean003424	0	0	0	0
Gglean003425	22.29400001	24.558426	31.287857	34.595393
Gglean003426	0	0	0	0
Gglean003427	17.2072	18.555	13.5054	9.02801
Gglean003428	31.603247	31.073746	34.270767	34.262679
Gglean003429	0	0	0	0
Gglean003430	13.649653	15.07706887	21.97528003	24.00800017
Gglean003431	0	0	0.043814977	0.025884995

Gglean003432	0	0	0.043814977	0.025884995
Gglean003433	0	0	0.043814977	0.025884995
Gglean003434	0	0.207611	0	0
Gglean003435	0.60993	2.70021	2.51931	2.42055
Gglean003436	0.310828	0.681685	0.262906	0
Gglean003437	1.25245	4.16989	0	3.62428
Gglean003438	0	0	0	0
Gglean003439	0	0.133778	0	0
Gglean003440	0	0	0	0
Gglean003441	0	0	0	0
Gglean003442	0.955567	1.280657	1.374809	0.9907551
Gglean003443	0	0	0	0
Gglean003444	0.97121	0	0	0
Gglean003445	0	0	0	0.421205
Gglean003446	0	0	0	0
Gglean003447	0.37418916	0.415384901	0.27952958	0.516841864
Gglean003448	0	0	0	0
Gglean003449	0.171025	0	0	0
Gglean003450	0	0.176842	0	0.163768
Gglean003451	46.59971002	44.6631176	34.29180901	31.94100696
Gglean003452	0	0	0	0
Gglean003453	0	0.163013	0.25178	0
Gglean003454	0	0	0	0
Gglean003455	0	0	0	0
Gglean003456	0	0	0.140485	0.502215
Gglean003457	0	0	0	0
Gglean003458	62.0974	50.2647	8.57408	12.3045
Gglean003459	0	0	0	0
Gglean003460	18.651028	20.159702	8.741183542	7.708252784
Gglean003461	18.651028	20.159702	8.741183542	7.708252784
Gglean003462	17.8073	18.513	9.99111	10.5556
Gglean003463	0	0	0	0
Gglean003464	0	0	0	0
Gglean003465	0	0	0	0
Gglean003466	0	0	0	0
Gglean003467	0	0	0	0
Gglean003468	101.40242	83.63751	150.533967	147.804546
Gglean003469	0	0	0	0
Gglean003470	32.60551423	29.71029608	16.47654292	16.73157714
Gglean003471	166.9958621	167.426034	169.8618696	171.5246057
Gglean003472	130.471	131.9692	83.1833	74.6413
Gglean003473	58.67227131	69.07521734	43.76996637	39.82118788
Gglean003474	32.10972	31.34847	47.2025	48.1268
Gglean003475	30.16134	14.40031	19.89724	18.31193

Gglean003476	0.4642	0.0954476	0.372569	1.258233
Gglean003477	103.858	84.5741	34.5524	21.8386
Gglean003478	26.47475065	26.66548445	11.115159	9.4564759
Gglean003479	24.06405911	24.717738	21.4737585	20.5909831
Gglean003480	8.43505	7.33654	11.80803	16.29742
Gglean003481	0	0	0	0
Gglean003482	28.1707	29.3787	39.367733	46.847394
Gglean003483	16.7196	17.6655	15.3832	16.6183
Gglean003484	10.2352	11.512	7.96311	5.58021
Gglean003485	3.31362	2.158131	1.984125	1.604328
Gglean003486	69.42507	71.98229	31.95382	25.90691
Gglean003487	58.2412	68.3488	57.8484	55.5035
Gglean003488	3.02793	2.04116	4.80137	5.52921
Gglean003489	66.4787	62.84501	56.14723	60.08116
Gglean003490	17.50561	16.82623	6.987869	4.591756584
Gglean003491	17.50561	16.82623	6.987869	4.591756584
Gglean003492	138.7601	135.48888	80.07544	76.24523
Gglean003493	1.0171	0.42252	2.65297	2.78771
Gglean003494	15.08276	14.21367	6.62523	4.5473
Gglean003495	6.28106934	5.852014052	9.975604399	8.937527
Gglean003496	26.598337	30.48299	28.833836	35.81532237
Gglean003497	26.598337	30.48299	28.833836	35.81532237
Gglean003498	0.0516581	0.673214	1.83789	0.352373
Gglean003499	11.23731372	10.58808902	9.620077047	9.842084897
Gglean003500	0	0	0	0
Gglean003501	3.707201	4.33939	1.641848	2.05357
Gglean003502	0.0564134	0	0	0.0544145
Gglean003503	13.46631296	13.0655673	7.488462322	6.925118872
Gglean003504	13.46631296	13.0655673	7.488462322	6.925118872
Gglean003505	31.06550583	30.365864	34.07738062	33.2563721
Gglean003506	1157.961292	1227.864466	755.7766415	715.172961
Gglean003507	119.9271104	117.1972848	122.3560611	120.6125038
Gglean003508	119.9271104	117.1972848	122.3560611	120.6125038
Gglean003509	0	0	0	0
Gglean003510	22.61161	20.64072	21.218849	21.73477
Gglean003511	55.51928684	58.96074404	67.37452962	75.36403599
Gglean003512	26.8452918	30.30366399	39.43182	38.067951
Gglean003513	26.8452918	30.30366399	39.43182	38.067951
Gglean003514	0	0	0	0
Gglean003515	14.556492	14.545622	16.13468	16.983379
Gglean003516	20.34096	14.43055119	12.24144	13.05172
Gglean003517	20.34096	14.43055119	12.24144	13.05172
Gglean003518	20.77156	21.94283	16.78405	18.5565
Gglean003519	20.77156	21.94283	16.78405	18.5565

Gglean003520	20.77156	21.94283	16.78405	18.5565
Gglean003521	8.775154332	10.5570384	14.06816164	14.09574364
Gglean003522	1.305963038	1.20274571	1.743051743	1.549102311
Gglean003523	0.385294	0.489649	1.68964	1.64666
Gglean003524	7.187972	8.399920073	12.48020027	12.92450016
Gglean003525	0	0	0	0
Gglean003526	0.141746	0.147767	0.233391	0.279235
Gglean003527	0	0	0	0.077042
Gglean003528	0	0	0	0
Gglean003529	0.0689731	0	0	0
Gglean003530	0	0	0	0
Gglean003531	0	0	0	0
Gglean003532	0	0	0	0
Gglean003533	0	0.594962	0	0
Gglean003534	0	0	0	0
Gglean003535	0	0	0	0
Gglean003536	0	0	0	0
Gglean003537	0	0	0	0
Gglean003538	0	0	0	0
Gglean003539	0	0	0	0
Gglean003540	0	0	0	0
Gglean003541	0	0.211906	0	0
Gglean003542	0.4202	0.312346	0.31094	0.300621
Gglean003543	0	0	0	0
Gglean003544	0	0	0	0
Gglean003545	0	0	0	0
Gglean003546	0	0	0	0
Gglean003547	0	0	0	0
Gglean003548	0	0	0	0
Gglean003549	0	0	0	0
Gglean003550	0	0	0	0
Gglean003551	18.50493684	20.26420376	24.841299	26.24261085
Gglean003552	1.618699499	1.926557267	1.928850026	1.96656619
Gglean003553	11.89225852	11.58755264	7.966183134	7.354968451
Gglean003554	24.50153	23.67601	22.2274	22.05279
Gglean003555	0.285472	0	0.469383	0
Gglean003556	2.70243	1.05E-06	0.00267021	0.0121312
Gglean003557	2.18635	0.0227028	8.63E-14	2.62541
Gglean003558	18.5368	21.4804	6.13968	6.19061
Gglean003559	414.543928	437.2698821	201.7552572	163.925333
Gglean003560	8.896219576	7.760199	5.29641	3.523560398
Gglean003561	0	0	0	0
Gglean003562	10.54861	7.706034173	15.57651654	20.02820021
Gglean003563	10.54861	7.706034173	15.57651654	20.02820021

Gglean003564	10.54861	7.706034173	15.57651654	20.02820021
Gglean003565	18.19411	15.709045	10.295198	7.173037
Gglean003566	18.19411	15.709045	10.295198	7.173037
Gglean003567	14.884525	14.60047	18.54885	20.40577
Gglean003568	25.15514	22.54368	21.57076	20.30602
Gglean003569	95.46567179	98.66851588	83.8697576	80.28076829
Gglean003570	16.13324	14.47847	22.27136	25.87413
Gglean003571	16.13324	14.47847	22.27136	25.87413
Gglean003572	0.0689999	0	0	0.132516
Gglean003573	0	0	0	0
Gglean003574	1.5094618	1.19052137	1.931684422	1.74828137
Gglean003575	1.5094618	1.19052137	1.931684422	1.74828137
Gglean003576	0	0	0	0
Gglean003577	0	0	0.0226838	0
Gglean003578	0.180506	0	0	0
Gglean003579	0	0	0	0
Gglean003580	0.0686578	0.31739	0.0835188	0.0329762
Gglean003581	0	0	0	0
Gglean003582	0.206829053	0.192840258	0.076055449	0.036162708
Gglean003583	0	0	0.339445	0
Gglean003584	0	0	0	0
Gglean003585	0	0	0.323166	0
Gglean003586	0	0	0	0
Gglean003587	0.2425841	0.319917249	0.278806767	0.361484339
Gglean003588	0.40114183	0.367144502	0.163273593	0.344892192
Gglean003589	0	0	0	0.114396
Gglean003590	0	0	0	0
Gglean003591	0	0	0.10607	0
Gglean003592	2.68996	4.43485	1.39692	0.782463
Gglean003593	0.0531049	0	0.0431263	0
Gglean003594	0	0	0	0
Gglean003595	0	0	0	0
Gglean003596	0	0	0	0
Gglean003597	0	0	0	0
Gglean003598	0	0	0	0
Gglean003599	0	0	0.105458	0
Gglean003600	0.183045	0	0	0
Gglean003601	0	0	0	0
Gglean003602	0	0	0.245777	0.293466
Gglean003603	0	0.457435	0	0
Gglean003604	0	0	0	0
Gglean003605	0	0	0	0
Gglean003606	0.247055	0.320034232	0.349124	0.219116529
Gglean003607	0	0	0.0422994	0

Gglean003608	2.283217345	1.786197427	2.740117519	2.8774116
Gglean003609	0	0	0	0
Gglean003610	0	0	0.0177583	0
Gglean003611	0	0	0	0
Gglean003612	0	0	0	0
Gglean003613	0.983157	0.629202233	1.092837	1.029807
Gglean003614	0	0.044919	0.0119312	0
Gglean003615	2.251555	1.7858024	2.3942326	2.3196146
Gglean003616	56.9755	51.8615	44.85	40.5704
Gglean003617	59.788939	58.53779	43.90365956	49.453887
Gglean003618	59.788939	58.53779	43.90365956	49.453887
Gglean003619	11.31129506	8.960708957	4.128335002	3.5549829
Gglean003620	11.31129506	8.960708957	4.128335002	3.5549829
Gglean003621	11.31129506	8.960708957	4.128335002	3.5549829
Gglean003622	9.14997	11.0129	10.7904	11.9348
Gglean003623	29.473253	32.6602935	22.2649882	21.41298698
Gglean003624	10.279854	15.97140583	8.170454	10.560221
Gglean003625	0.36009	0.203705	0.0965871	0
Gglean003626	38.1288	38.5784	41.5119	41.6646
Gglean003627	68.17914734	74.126624	77.854378	71.209506
Gglean003628	12.16326	11.080972	10.632817	12.13035
Gglean003629	0	0	0	0
Gglean003630	25.932903	29.748466	45.725645	48.4458
Gglean003631	1.691132	1.291015	5.802645	2.233307
Gglean003632	46.00595	49.13868239	35.90904884	36.15077201
Gglean003633	46.00595	49.13868239	35.90904884	36.15077201
Gglean003634	0.165304	0.215169	0.380994	0.285102
Gglean003635	513.551275	518.54627	361.9511	317.700907
Gglean003636	513.551275	518.54627	361.9511	317.700907
Gglean003637	0	0	0.0944746	0
Gglean003638	30.88269964	37.4572489	30.013186	30.01042643
Gglean003639	35.18975794	34.97987007	29.381126	26.671556
Gglean003640	18.90221	17.78745	15.04322	13.06441
Gglean003641	1.48127	1.54356	2.27022	2.17137
Gglean003642	0	0	0	0
Gglean003643	62.97444949	78.18531	39.81586579	30.984469
Gglean003644	62.97444949	78.18531	39.81586579	30.984469
Gglean003645	70.2133	53.4129	83.0244	109.589
Gglean003646	113.25229	107.091602	122.0452651	125.685046
Gglean003647	14.75005	13.66956	19.11929	27.11521
Gglean003648	38.15273	41.27859	29.79408	27.93434
Gglean003649	3.013794	4.25751	6.3015	4.765194
Gglean003650	59.55649	80.06995	86.05732	108.4509
Gglean003651	4.1697001	4.035854505	7.1778	6.03935125

Gglean003652	0	0	0	0
Gglean003653	22.47716998	22.7481772	36.2194015	33.649905
Gglean003654	22.47716998	22.7481772	36.2194015	33.649905
Gglean003655	2.86000169	2.668156	6.79041	6.713290755
Gglean003656	2.86000169	2.668156	6.79041	6.713290755
Gglean003657	1.38757	0.00101905	0.230639	0.00183791
Gglean003658	0.100331	0.0767607	0.0812337	0
Gglean003659	0	0	0	1.72781
Gglean003660	0.298276458	0.262255061	0.492873595	0.306531173
Gglean003661	0.298276458	0.262255061	0.492873595	0.306531173
Gglean003662	24.9892	27.7545	35.8803	32.6683
Gglean003663	26.26056196	50.87270047	65.813763	65.25862775
Gglean003664	3.634240547	3.24229438	2.344489397	3.431207036
Gglean003665	31.80087	29.85632	49.85011	55.75123
Gglean003666	106.637223	118.8431266	169.9763061	175.2398122
Gglean003667	19.97903	14.31644	11.1921	15.85274
Gglean003668	1.0151468	1.166219197	2.380685329	2.051603
Gglean003669	449.8627734	463.19987	243.6266362	207.3523294
Gglean003670	449.8627734	463.19987	243.6266362	207.3523294
Gglean003671	13.36618221	13.39234511	11.48835924	12.68700989
Gglean003672	374.154	347.046	381.236	397.837
Gglean003673	3.86E-37	0.160356	1.51201	7.40E-07
Gglean003674	0	0	0	0.355947
Gglean003675	0	0	0	0
Gglean003676	39.71872	42.031	34.96555	33.44296
Gglean003677	40.40296498	43.06095509	27.50289352	23.31672245
Gglean003678	40.55535486	52.7025	18.2841975	16.69251973
Gglean003679	8.713744936	8.128889226	9.80459239	13.6357927
Gglean003680	8.713744936	8.128889226	9.80459239	13.6357927
Gglean003681	5.19604	5.79577	8.37594	10.6565
Gglean003682	18.40918	18.71659	25.81674	25.002309
Gglean003683	0	0	0	0
Gglean003684	36.3876161	31.98823007	40.65175745	53.43656401
Gglean003685	38.473475	36.87967341	35.50916378	32.805091
Gglean003686	38.473475	36.87967341	35.50916378	32.805091
Gglean003687	6.11872	4.85371	3.89407	3.93787
Gglean003688	39.69802669	35.84623386	21.12510879	20.64280291
Gglean003689	32.701999	37.34244	44.528032	44.45025062
Gglean003690	32.701999	37.34244	44.528032	44.45025062
Gglean003691	38.73645	35.64606	23.10282	20.608974
Gglean003692	11.3775	14.7064	8.67402	9.15949
Gglean003693	1.77603	0	1.4484	0.690778
Gglean003694	103.7993	106.1514	66.4869	67.5154
Gglean003695	243.0748158	277.90833	124.1930045	114.20913

Gglean003696	27.30847	27.98654	35.10453	40.00785
Gglean003697	0	0	0	0
Gglean003698	0	0	0	0
Gglean003699	0	0	0	2.30915
Gglean003700	34.516016	33.60075	30.68191	33.21849
Gglean003701	0.318112	0.479956	0.415344	0.623285
Gglean003702	21.0384	27.3842	9.10816	6.80555
Gglean003703	0.162377	0	0	0
Gglean003704	3.89374	3.48012	3.057512	2.74599
Gglean003705	3.89374	3.48012	3.057512	2.74599
Gglean003706	1.253990627	1.304756372	1.570199045	1.822202489
Gglean003707	1.066196	1.203571	1.201296	1.438981
Gglean003708	0	0	0	0
Gglean003709	34.0635	34.9224	12.5496	11.2805
Gglean003710	0	0	0	0
Gglean003711	0	0	0	0
Gglean003712	0	0	0	0
Gglean003713	0	0	0	0
Gglean003714	0	0	0	0
Gglean003715	0.141685	0.295085	0.173745	0
Gglean003716	0	0	0	0
Gglean003717	0	0	0	0
Gglean003718	0	0	0	0
Gglean003719	0	0	0	0
Gglean003720	0	0.133455	0	0
Gglean003721	0	0	0	0
Gglean003722	0	0	0	0
Gglean003723	0	0	0.146774	0.175857
Gglean003724	9.325451561	10.23948881	7.452104111	11.9335876
Gglean003725	9.325451561	10.23948881	7.452104111	11.9335876
Gglean003726	9.325451561	10.23948881	7.452104111	11.9335876
Gglean003727	0.119935	0.332480658	0.0973389	0.154412524
Gglean003728	0.19858	0	0	0
Gglean003729	21.47544	20.60795	14.02763	11.8432
Gglean003730	21.47544	20.60795	14.02763	11.8432
Gglean003731	38.10789	40.79420915	22.8495014	19.8402736
Gglean003732	53.86787	52.13407	27.37442	26.2367
Gglean003733	19.91045211	21.59613045	31.40554187	31.37503522
Gglean003734	201.923	314.252	140.238	179.154
Gglean003735	57.52194	56.1802	60.44817	61.73282
Gglean003736	62.23222	61.47814	72.13838	65.65569
Gglean003737	1392.82923	1348.90856	861.07076	816.91477
Gglean003738	7.68735	8.80861	5.471737	5.103046
Gglean003739	7.68735	8.80861	5.471737	5.103046

Gglean003740	16.4242	19.3711	13.4853	14.2808
Gglean003741	71.7685249	72.685232	45.18158013	44.443608
Gglean003742	71.7685249	72.685232	45.18158013	44.443608
Gglean003743	107.879	121.829	84.2928	73.8335
Gglean003744	23.9081	20.7474	18.07	18.6069
Gglean003745	37.9493284	43.30424894	55.36324858	64.7102809
Gglean003746	36.5643	37.8203	38.66	44.3358
Gglean003747	15.57114935	13.4941162	9.26298204	7.886958
Gglean003748	15.57114935	13.4941162	9.26298204	7.886958
Gglean003749	1.604544798	1.02784	2.314525	1.456023776
Gglean003750	44.2765012	55.2844654	55.87260153	56.41686
Gglean003751	44.2765012	55.2844654	55.87260153	56.41686
Gglean003752	48.6745	54.5396	54.1705	52.34569585
Gglean003753	1.222844	0.514927346	1.196255	0.92293508
Gglean003754	40.4126882	41.80390324	41.51888633	42.73244947
Gglean003755	53.154	93.3951	15.3816	29.2536
Gglean003756	41.3297	39.2659	18.75435	20.8297
Gglean003757	19.93426986	10.78815294	18.14771957	21.71486877
Gglean003758	19.93426986	10.78815294	18.14771957	21.71486877
Gglean003759	19.93426986	10.78815294	18.14771957	21.71486877
Gglean003760	6.801506127	6.482631654	11.26666	10.0920914
Gglean003761	214.3412	206.7531	193.2654	240.3841
Gglean003762	126.2541691	122.2719871	125.3455298	128.8242568
Gglean003763	16.645898	13.84124257	14.30577185	16.30700519
Gglean003764	16.645898	13.84124257	14.30577185	16.30700519
Gglean003765	35.74152	37.28548	28.527823	21.067224
Gglean003766	22.49478	23.72496	16.95379	14.29195
Gglean003767	38.318	45.8039	44.088	43.6637
Gglean003768	0	0	0	0
Gglean003769	57.59416835	55.98092245	76.83979357	76.08891067
Gglean003770	18.6198	18.2472	14.7291	12.6172
Gglean003771	12.9325	9.23798	10.8068	13.1022
Gglean003772	29.80801	30.79694	23.50704	19.69778
Gglean003773	17.48610013	20.67261124	25.11114432	37.98059992
Gglean003774	13.39116906	13.76199549	18.75594423	17.79398276
Gglean003775	12.78017125	10.399756	12.38682478	11.60454465
Gglean003776	25.126847	27.91847	8.329004	7.254189
Gglean003777	201.4941	198.6123	105.4831	96.80473
Gglean003778	28.73844	28.91648854	36.70887712	37.92837716
Gglean003779	27.27497	34.3554	45.538	58.4303
Gglean003780	2.232591	1.553135285	3.00772	3.003982
Gglean003781	35.614213	40.168673	36.424595	34.9618885
Gglean003782	35.614213	40.168673	36.424595	34.9618885
Gglean003783	14.09505	11.08701	7.961039	6.686225

Gglean003784	14.09505	11.08701	7.961039	6.686225
Gglean003785	34.9245789	34.49100155	40.1793112	39.22468385
Gglean003786	1.95E-05	0.345824	5.00E-05	0.142599
Gglean003787	32.10982391	29.76918618	25.3587301	23.0670217
Gglean003788	32.10982391	29.76918618	25.3587301	23.0670217
Gglean003789	32.10982391	29.76918618	25.3587301	23.0670217
Gglean003790	39.801186	46.4365445	9.460517676	8.318544617
Gglean003791	57.12551078	61.16959	51.85275	56.542064
Gglean003792	57.12551078	61.16959	51.85275	56.542064
Gglean003793	33.8585	33.95631	20.79844	21.30344
Gglean003794	101.9153	113.705402	95.06443545	91.0242
Gglean003795	54.38982	58.0331	42.16635	37.58482
Gglean003796	36.886791	36.41878	40.59131	58.222924
Gglean003797	36.886791	36.41878	40.59131	58.222924
Gglean003798	14.77502	16.486137	20.385398	21.225273
Gglean003799	63.79682267	73.50976178	100.3128889	120.6641998
Gglean003800	63.79682267	73.50976178	100.3128889	120.6641998
Gglean003801	63.79682267	73.50976178	100.3128889	120.6641998
Gglean003802	63.79682267	73.50976178	100.3128889	120.6641998
Gglean003803	0.0183531	0.113189	0.0788587	0.0870051
Gglean003804	0.0442205	0.113222	0.0531683	0.0208543
Gglean003805	0	0	0	0
Gglean003806	0.0129458	0.133303	0.0416814	0.0613453
Gglean003807	0	0.147868	0.116463	0.137521
Gglean003808	0	0.128054	0.0333209	0
Gglean003809	0.0185292	0.0851979	0.118605	0.0611266
Gglean003810	0.0381936	0.263083	0.199009	0.216768
Gglean003811	63.79682267	73.50976178	100.3128889	120.6641998
Gglean003812	63.79682267	73.50976178	100.3128889	120.6641998
Gglean003813	63.79682267	73.50976178	100.3128889	120.6641998
Gglean003814	63.79682267	73.50976178	100.3128889	120.6641998
Gglean003815	63.79682267	73.50976178	100.3128889	120.6641998
Gglean003816	63.79682267	73.50976178	100.3128889	120.6641998
Gglean003817	63.79682267	73.50976178	100.3128889	120.6641998
Gglean003818	78.39774347	78.4590283	57.731897	55.25968627
Gglean003819	16.61756569	15.41787283	14.10026596	15.685058
Gglean003820	51.8759	56.3487	44.2259	37.99647
Gglean003821	51.8759	56.3487	44.2259	37.99647
Gglean003822	31.75592966	29.55896315	49.70165807	47.02998491
Gglean003823	31.75592966	29.55896315	49.70165807	47.02998491
Gglean003824	31.75592966	29.55896315	49.70165807	47.02998491
Gglean003825	31.75592966	29.55896315	49.70165807	47.02998491
Gglean003826	25.917239	28.192212	26.66787499	28.431878
Gglean003827	7.662022	8.582008	15.659928	19.730094

Gglean003828	30.28007	33.41568	28.031698	20.586105
Gglean003829	30.28007	33.41568	28.031698	20.586105
Gglean003830	0	0.109243	0	0
Gglean003831	22.07786084	20.2843354	8.379883808	5.674038107
Gglean003832	22.07786084	20.2843354	8.379883808	5.674038107
Gglean003833	9.19411	7.47811	5.61389	7.88303
Gglean003834	142.362097	135.4187682	115.9433497	97.52365711
Gglean003835	51.5639843	50.61891604	76.46195801	64.30303285
Gglean003836	51.5639843	50.61891604	76.46195801	64.30303285
Gglean003837	51.5639843	50.61891604	76.46195801	64.30303285
Gglean003838	13.82424	12.855445	22.98073	25.11596
Gglean003839	91.69798	97.17125646	88.469314	80.3461504
Gglean003840	1.885140352	0.922274144	0.853094917	1.535015407
Gglean003841	1.885140352	0.922274144	0.853094917	1.535015407
Gglean003842	32.82337	29.70104295	23.486069	22.929264
Gglean003843	0.037462129	0	0.011015249	0
Gglean003844	0.037462129	0	0.011015249	0
Gglean003845	0.037462129	0	0.011015249	0
Gglean003846	37.9036	60.2992	50.723	42.4898
Gglean003847	37.9036	60.2992	50.723	42.4898
Gglean003848	0.7986119	1.018891734	1.029776645	1.025600979
Gglean003849	6.309392538	7.453364	10.71778	11.64904704
Gglean003850	4366.599069	4205.816399	1858.635631	1576.749578
Gglean003851	60.3421	57.7311	34.8367	29.4566
Gglean003852	14.8115	8.02865	14.6402	14.9254
Gglean003853	4366.599069	4205.816399	1858.635631	1576.749578
Gglean003854	551.212	569.702	635.124	948.049
Gglean003855	17.936907	21.33154	8.513137	8.639574966
Gglean003856	23.0952	22.4175	25.7555	25.8171
Gglean003857	36.10073	42.504637	32.96117	30.40089
Gglean003858	36.10073	42.504637	32.96117	30.40089
Gglean003859	36.10073	42.504637	32.96117	30.40089
Gglean003860	33.20834461	39.92225718	20.36237176	18.85842883
Gglean003861	7.966368127	8.71179	7.005283737	6.761866354
Gglean003862	4.4101594	4.709840468	5.015776354	7.723190453
Gglean003863	20.35343778	20.4866418	25.46933851	31.818835
Gglean003864	3.170380114	2.076209736	1.531606591	2.521898876
Gglean003865	13.55780358	13.79475458	13.60571321	11.895193
Gglean003866	13.3953703	12.22239266	6.76647	8.154874
Gglean003867	24.043422	26.320571	11.804801	11.196553
Gglean003868	12.57362	12.993466	12.427297	11.07232647
Gglean003869	3.37874	3.74639	4.16061	3.98803
Gglean003870	12.57362	12.993466	12.427297	11.07232647
Gglean003871	0.0807543	0	0	0

Gglean003872	0.920525	1.01735	0.761582	0
Gglean003873	26.40452	27.4441929	19.415261	17.651852
Gglean003874	25.4112	21.7889	18.2545	19.1669
Gglean003875	15.70068777	14.78346162	16.76873591	16.92378256
Gglean003876	15.70068777	14.78346162	16.76873591	16.92378256
Gglean003877	9.348532817	9.90196967	11.4410229	13.38234201
Gglean003878	9.348532817	9.90196967	11.4410229	13.38234201
Gglean003879	6.077665	5.694366	4.462955	4.489973436
Gglean003880	7.338890043	5.646490079	8.758790016	10.61060001
Gglean003881	18.29286	21.82084	25.97845155	27.70652999
Gglean003882	0	0	0	0
Gglean003883	0	0	0	0
Gglean003884	37.32513	36.3252	35.91167375	37.60665
Gglean003885	13.41479	13.865794	22.554814	26.892896
Gglean003886	13.41479	13.865794	22.554814	26.892896
Gglean003887	42.13519331	38.1937071	24.39287406	19.49556708
Gglean003888	0	0	0	0
Gglean003889	53.97010119	48.49944603	36.17634736	35.08216106
Gglean003890	67.588613	65.48097	123.017073	129.80354
Gglean003891	28.9525	28.4214	30.6541	28.9533
Gglean003892	46.8612006	36.42416085	60.86162134	71.17372874
Gglean003893	46.8612006	36.42416085	60.86162134	71.17372874
Gglean003894	131.0314	132.7661	129.817	154.7855
Gglean003895	259.9441869	251.2327575	200.9550822	201.2702674
Gglean003896	54.66331	51.78384	48.90718	48.64154
Gglean003897	54.66331	51.78384	48.90718	48.64154
Gglean003898	63.95	77.5606	100.3251	123.1828
Gglean003899	0	0	0	0
Gglean003900	0.0252018	0	0	0
Gglean003901	0	0	0	0
Gglean003902	0.346707401	0.24185432	0.333613	0.111911094
Gglean003903	0	0	0.04358509	0.051478582
Gglean003904	48.627795	44.889742	42.871406	46.869706
Gglean003905	157.1904	162.3511	177.0915	216.4574
Gglean003906	0.174036	0.361361	0.636962	0.918825
Gglean003907	32.14827003	39.372007	31.82672	34.53774774
Gglean003908	43.52004568	44.40511929	99.64074836	97.41526812
Gglean003909	7.388095	6.68153	3.3491	3.556682
Gglean003910	12.792922	15.42132458	18.27176197	18.97010587
Gglean003911	13.25697138	13.26953016	13.75821313	14.37908725
Gglean003912	44.8662799	49.08434778	41.8018518	40.0485417
Gglean003913	44.8662799	49.08434778	41.8018518	40.0485417
Gglean003914	121.49035	120.88829	128.34428	120.91908
Gglean003915	4.48683	6.33856	9.32801	14.3827

Gglean003916	62.1212	70.223545	94.1048	109.8072
Gglean003917	62.1212	70.223545	94.1048	109.8072
Gglean003918	0.36944	0.322338	0.347374247	0.618395
Gglean003919	41.346262	39.092261	43.078464	39.33734783
Gglean003920	0.493346	0.386328	0.605603	0.240646
Gglean003921	15.46157	17.50624028	11.2053501	13.753911
Gglean003922	58.37436859	69.14401135	70.06125165	65.69713382
Gglean003923	46.8173	44.9738	71.1542	57.4678
Gglean003924	23.3622	26.1728	21.3817	26.1814
Gglean003925	39.23533	37.08779	36.9665	39.991749
Gglean003926	29.3752	31.0817	20.17526	17.4019
Gglean003927	41.713	43.6824	28.65452	32.5666
Gglean003928	27.020417	25.96467	28.38504059	27.63695956
Gglean003929	59.2857	61.2881	48.1481	61.9171
Gglean003930	36.7805	41.4068	28.15526	25.56084
Gglean003931	44.32908	40.54479	31.22188	29.13728
Gglean003932	29.2149	28.3786	22.8427	23.9252
Gglean003933	66.3917	62.7979	83.6145	88.4998
Gglean003934	125.4412	152.1775	49.2736	37.6782
Gglean003935	0	0	0	0
Gglean003936	105.5167801	112.4004936	132.1544325	155.1082379
Gglean003937	20.7677	19.454	8.27574	13.5059
Gglean003938	23.06856449	21.80715601	24.11911193	21.69852685
Gglean003939	38.16697587	36.41944	45.2719147	56.8394782
Gglean003940	38.16697587	36.41944	45.2719147	56.8394782
Gglean003941	363.461	374.974	294.791	310.226
Gglean003942	51.747353	49.750972	51.8843	49.984691
Gglean003943	20.6654	17.4086	16.8149	17.5469
Gglean003944	35.89199663	37.22443288	50.20261443	45.0132034
Gglean003945	35.89199663	37.22443288	50.20261443	45.0132034
Gglean003946	16.21895372	17.71307618	14.86854982	18.52154027
Gglean003947	42.5672579	38.64281097	27.600804	21.80908732
Gglean003948	0.7970582	0.70929	1.224690549	1.300799404
Gglean003949	0	0	0	0
Gglean003950	0	0	0	0
Gglean003951	221.4625	216.3552	263.22016	382.5949
Gglean003952	30.09129039	34.5932314	41.8885979	43.43020523
Gglean003953	16.72421	16.63812	17.21873	16.61259
Gglean003954	57.9288	71.6994	49.6976	50.3148
Gglean003955	4.832777	5.535621	5.061813	6.220984
Gglean003956	4.832777	5.535621	5.061813	6.220984
Gglean003957	15.2124	17.021	13.914	13.1264
Gglean003958	46.93614	45.73544	48.34955	46.1543063
Gglean003959	46.93614	45.73544	48.34955	46.1543063

Gglean003960	46.93614	45.73544	48.34955	46.1543063
Gglean003961	46.93614	45.73544	48.34955	46.1543063
Gglean003962	40.0930154	41.03934	19.8771	16.86244
Gglean003963	20.5699084	19.11441181	19.811242	18.1720704
Gglean003964	131.84	131.3395	109.4048	104.4816
Gglean003965	88.98021002	93.165327	86.315535	81.11139
Gglean003966	35.34879718	34.341228	35.568823	33.17427988
Gglean003967	35.34879718	34.341228	35.568823	33.17427988
Gglean003968	51.8487453	54.83503462	44.84802662	42.6043379
Gglean003969	106.817216	103.403807	112.887714	112.893601
Gglean003970	30.90077	32.269932	31.65945	30.186881
Gglean003971	36.04096002	41.185695	36.50113078	36.34141
Gglean003972	36.04096002	41.185695	36.50113078	36.34141
Gglean003973	59.44348357	56.31877	81.8755	85.03158
Gglean003974	49.97796471	41.19392633	22.9715701	26.6625178
Gglean003975	49.97796471	41.19392633	22.9715701	26.6625178
Gglean003976	49.97796471	41.19392633	22.9715701	26.6625178
Gglean003977	11.86924472	14.17129584	7.4358631	6.597392248
Gglean003978	11.86924472	14.17129584	7.4358631	6.597392248
Gglean003979	0.582714	0.780722	0.339265	1.12753
Gglean003980	14.801149	13.82573083	13.498482	19.879639
Gglean003981	18.4667	23.9781	19.9815	18.5356
Gglean003982	126.243727	109.96936	169.55059	242.69957
Gglean003983	138.93814	163.10247	178.41426	168.07937
Gglean003984	32.70239114	29.84442542	32.01441398	31.72232457
Gglean003985	32.70239114	29.84442542	32.01441398	31.72232457
Gglean003986	27.482	34.0315	38.0171	43.5035
Gglean003987	118.667	108.237	105.726	99.6527
Gglean003988	122.8753245	98.799707	110.6607486	126.0956386
Gglean003989	19.3123	21.1419	18.8698	20.6604
Gglean003990	36.73734038	39.09221038	36.999486	36.31066558
Gglean003991	36.73734038	39.09221038	36.999486	36.31066558
Gglean003992	0	0	0	0
Gglean003993	0.239845	0	0.398138	0
Gglean003994	34.03619	31.70249	20.992934	25.156547
Gglean003995	16.4289	20.48792	11.998424	8.651159
Gglean003996	17.89023561	18.36416	14.32583759	12.6578763
Gglean003997	5.23625	1.37695	7.35522	0.668989
Gglean003998	15.26013	14.91229	11.48633	11.67549
Gglean003999	0	0	0	0
Gglean004000	0	0	0	0
Gglean004001	0.355929	0.284333	0.355919	0.236213082
Gglean004002	0	0	0	0.231103
Gglean004003	0	0	0	0

Gglean004004	0.140357	0.2588	0.0909399	0.026903
Gglean004005	0	0	0	0
Gglean004006	0	0	0	0.898405
Gglean004007	0	0	0	0
Gglean004008	0	0	0	0
Gglean004009	0	0	0	0
Gglean004010	0	0	0	0
Gglean004011	0	0	0	0
Gglean004012	0	0	0	0
Gglean004013	0	0	0	0
Gglean004014	0	0	0	0
Gglean004015	0.313939	0	0.257548	0
Gglean004016	1.84209	3.43185	2.25748	0.224672
Gglean004017	0	0	0	0
Gglean004018	0.0344201	0	0.0550052	0
Gglean004019	0	0	0	0
Gglean004020	0	0	0	0
Gglean004021	12.49488	12.91648	21.7187	20.77105
Gglean004022	0	0.200534	0	0
Gglean004023	0	0	0	0
Gglean004024	0	0	0	0
Gglean004025	0	0	0	0
Gglean004026	0.211213	0.650796	1.19138	0.303722
Gglean004027	18.2418	28.1872	1.52684	0.911799
Gglean004028	0.0896983	0	0	0
Gglean004029	0	0	0	0.0978782
Gglean004030	0	0	0	0.176413
Gglean004031	15.28053	16.11736	14.765942	12.342471
Gglean004032	43.30073	40.03466	23.43866	20.11711
Gglean004033	0	0	0	0
Gglean004034	27.81097654	32.206541	38.155828	40.7147566
Gglean004035	27.81097654	32.206541	38.155828	40.7147566
Gglean004036	0.677678866	2.687631	0.54727	1.136198094
Gglean004037	0	0.205027	0.0810841	0.0960366
Gglean004038	0	0	0	0
Gglean004039	17.81355	16.70272	19.99355	23.17869
Gglean004040	25.2054	23.2108	54.0267	66.026
Gglean004041	33.1134	28.8698	69.7013	74.8359
Gglean004042	0	0	0	0
Gglean004043	0.0588475	0.0600035	0	0
Gglean004044	0	0	0	0
Gglean004045	0	0	0	0
Gglean004046	0	0.0566048	0	0
Gglean004047	0	0	0	0

Gglean004048	2.48274	3.05237	1.157192	1.1387593
Gglean004049	2.48274	3.05237	1.157192	1.1387593
Gglean004050	3.579264171	5.012585938	1.672602641	3.67741428
Gglean004051	3.579264171	5.012585938	1.672602641	3.67741428
Gglean004052	0	0	0	0.149042
Gglean004053	5.07236	4.65152	6.99435	7.72895
Gglean004054	0	0	0.783598	0
Gglean004055	0	0	0	0
Gglean004056	0	0	0	0
Gglean004057	0.0891196	0.0914639	0	0
Gglean004058	0	0	0	0
Gglean004059	0	0	0	0
Gglean004060	0	0	0	0
Gglean004061	0	0	0	0
Gglean004062	0	0	0	0
Gglean004063	0	0	0	0
Gglean004064	0	0	0	0
Gglean004065	0.114961	0.0596956	0	0.273719
Gglean004066	0	0	0	0
Gglean004067	1.80608	1.38296	0.54625	1.01858
Gglean004068	0.794481826	0.664927394	0.995510721	0.84600758
Gglean004069	41.24885182	42.60990426	35.84411106	45.3332761
Gglean004070	41.24885182	42.60990426	35.84411106	45.3332761
Gglean004071	20.03195	16.57954	12.13628	10.10710097
Gglean004072	3.33433	0.946347	1.56441	1.39406
Gglean004073	5.79364	6.43878	3.1389	2.96635
Gglean004074	144.0697	139.0517022	164.580825	218.9551
Gglean004075	44.35119	48.33375	38.84789	37.49808
Gglean004076	43.1692	52.80078	50.27856	63.20089
Gglean004077	19.56429	23.62881	9.16423	6.72957
Gglean004078	34.172649	33.16629	51.784838	62.1689551
Gglean004079	8.978401585	7.970779853	11.66393948	14.39789979
Gglean004080	35.811648	37.505747	68.726155	67.48279571
Gglean004081	30.5987	25.8705	23.2217	15.27493
Gglean004082	0.4786498	0.665013612	0.130068078	0.569914
Gglean004083	68.2394	73.5881	51.0994	45.0961
Gglean004084	84.14483345	92.78077128	104.8230737	112.4070779
Gglean004085	30.80697	28.9052	27.630045	35.36111
Gglean004086	15.39948776	15.5488826	13.3471636	13.8060804
Gglean004087	2.345846	2.64623	2.83282	3.4336
Gglean004088	32.60463661	37.39737182	31.28076086	30.16382804
Gglean004089	166.3004	173.7891	127.6499	159.6145
Gglean004090	26.453688	25.480187	37.589706	42.54372
Gglean004091	16.809275	17.113298	19.22079205	19.63143598

Gglean004092	1534.6942	1625.8549	971.4936	933.0458
Gglean004093	2.057679771	1.942931181	3.456920764	4.16945988
Gglean004094	2.057679771	1.942931181	3.456920764	4.16945988
Gglean004095	20.46074272	24.58713738	5.601887	6.017291656
Gglean004096	20.46074272	24.58713738	5.601887	6.017291656
Gglean004097	99.89817414	113.920068	23.17388649	21.27686074
Gglean004098	99.89817414	113.920068	23.17388649	21.27686074
Gglean004099	99.89817414	113.920068	23.17388649	21.27686074
Gglean004100	99.89817414	113.920068	23.17388649	21.27686074
Gglean004101	0.90079	0.637304153	0.814549	0.65925
Gglean004102	16.318915	23.99981581	18.5231	20.40625057
Gglean004103	0.109727	0.11405	0	0
Gglean004104	2.98131	3.16662	2.16452	1.02952
Gglean004105	0.0822011	0.33897	0.0661867	0.156837
Gglean004106	16.64887	17.58202	19.15689	24.38955
Gglean004107	22.83353	24.24269	14.71839	12.13145
Gglean004108	14.20876	14.45181	9.22349	8.23321
Gglean004109	24.32096	23.9412	9.06358	8.37756
Gglean004110	124.0153028	138.4647431	42.6377024	41.35894956
Gglean004111	39.40694136	36.7890657	37.71206	34.2105193
Gglean004112	0.218183557	0.19633004	0.276602699	0.627122
Gglean004113	0.218183557	0.19633004	0.276602699	0.627122
Gglean004114	0	0	0	0
Gglean004115	0	0	0	0
Gglean004116	13.9471	12.84637	16.24086	24.2341
Gglean004117	6.60939	9.36542	7.14078	1.20866
Gglean004118	27.477	20.9777	23.3961	36.1877
Gglean004119	10.56658	10.22758	9.765486	10.86183
Gglean004120	44.77257	37.98788	90.0615	87.0682
Gglean004121	0.045185273	0.092812216	0.036421732	0.086534314
Gglean004122	0	0	0	0
Gglean004123	0	0	0	0
Gglean004124	0.0690299	0	0.0562603	0.0664822
Gglean004125	3.5612	0	5.683169	0.838603
Gglean004126	6.69E-06	0.000764238	5.16E-06	0.192325
Gglean004127	0.0973015	0.5626731	1.4643023	0.878309
Gglean004128	0.0973015	0.5626731	1.4643023	0.878309
Gglean004129	0.565170281	0	1.849198	2.769474
Gglean004130	1.660378	2.499125	0.55918	0.767578
Gglean004131	0	0	0	0
Gglean004132	0	0	0	0
Gglean004133	6.225743336	8.1156392	9.592775	8.557594201
Gglean004134	6.225743336	8.1156392	9.592775	8.557594201
Gglean004135	0	0	0	0

Gglean004136	6.997714	7.837974626	14.997867	16.72753614
Gglean004137	6.997714	7.837974626	14.997867	16.72753614
Gglean004138	2.132495	1.476374	2.320381	2.05893
Gglean004139	12.7502205	11.84367865	20.89968398	21.170026
Gglean004140	13.93523	12.01187	6.96658319	7.179645
Gglean004141	3.49991	3.61316	5.84529	6.43699
Gglean004142	13.93523	12.01187	6.96658319	7.179645
Gglean004143	9.170547688	11.00975221	9.943845	17.35277
Gglean004144	9.170547688	11.00975221	9.943845	17.35277
Gglean004145	0	0	0	0
Gglean004146	0	0	0.0202262	0
Gglean004147	0	0	0	0
Gglean004148	0	0	0	0
Gglean004149	5.15182	1.57877	0.856583	8.12E-07
Gglean004150	0	0	0	0
Gglean004151	0	0	0	0
Gglean004152	156.74925	137.7114413	158.3780353	225.44727
Gglean004153	0.716152	0.426281	0.432297	1.14227
Gglean004154	3.02081	2.31396	5.70178	5.27854
Gglean004155	11.399088	12.343002	13.868485	21.96458
Gglean004156	11.73843405	17.71555187	15.973125	18.83717658
Gglean004157	35.54979465	37.186501	52.16225353	51.18458348
Gglean004158	35.54979465	37.186501	52.16225353	51.18458348
Gglean004159	35.54979465	37.186501	52.16225353	51.18458348
Gglean004160	35.54979465	37.186501	52.16225353	51.18458348
Gglean004161	20.59868905	19.50858	12.85381	12.42321
Gglean004162	0	0	0	0
Gglean004163	53.8843	61.1196	55.4852	62.9838
Gglean004164	0	0	0	0
Gglean004165	2.65226	2.22605	2.19153	2.38487
Gglean004166	0.349535	0.353125	8.98E-05	7.44E-05
Gglean004167	2.32236	2.22521	2.71633	1.82407
Gglean004168	4.54572	4.06834	1.74578	1.20169
Gglean004169	0	0	0	0
Gglean004170	19.3167	18.1521	20.3887	22.267
Gglean004171	32.3115731	33.162242	46.80899897	52.87494204
Gglean004172	32.3115731	33.162242	46.80899897	52.87494204
Gglean004173	34.89195	32.90394	43.98142	56.25628
Gglean004174	2.41392	0.00556073	2.07E-17	0.000702086
Gglean004175	34.4577325	34.4684947	41.85615277	42.0586399
Gglean004176	30.9510095	30.735719	20.57294414	19.417772
Gglean004177	26.23140934	29.527316	29.325646	28.00755188
Gglean004178	10.7248	10.4351	8.32081	6.82546
Gglean004179	0	0	0	0

Gglean004180	0	0	0	0.191633
Gglean004181	0	0	0	0
Gglean004182	0	0	0.373789	0
Gglean004183	67.02465123	71.304683	64.019591	55.04384474
Gglean004184	0	0	0	0
Gglean004185	0	0	0	0
Gglean004186	0	0	0	0
Gglean004187	0	0	0	0
Gglean004188	0	0	0	0
Gglean004189	0.315914	0.327846	0.127937	0.151943
Gglean004190	0	0	0	0
Gglean004191	15.908285	14.895075	22.512672	26.591981
Gglean004192	0	0	0.0965116	0
Gglean004193	0.000409697	0.00470805	0.000185722	1.21341
Gglean004194	0	0	0	0
Gglean004195	14.14071411	16.36648871	18.68712704	18.38358186
Gglean004196	4.793344625	5.220844672	5.029652255	6.53562602
Gglean004197	4.793344625	5.220844672	5.029652255	6.53562602
Gglean004198	58.36650241	78.1227138	68.50344338	51.25880333
Gglean004199	0	0.110211	0.130852	0.0516782
Gglean004200	54.1526	49.028	63.6703	62.5001
Gglean004201	0	0	0	0
Gglean004202	0	0	0	0
Gglean004203	1.17565703	1.017291063	0.265922	0.157511412
Gglean004204	0.976280892	0.936001493	0.595187424	0.59622568
Gglean004205	2.292924	1.749467796	0.386658	0
Gglean004206	109.746218	100.97922	74.317695	67.5066
Gglean004207	27.27932234	27.247783	26.602712	23.42287843
Gglean004208	27.27932234	27.247783	26.602712	23.42287843
Gglean004209	27.27932234	27.247783	26.602712	23.42287843
Gglean004210	27.27932234	27.247783	26.602712	23.42287843
Gglean004211	27.27932234	27.247783	26.602712	23.42287843
Gglean004212	0.44928	0.26457	0.462091433	0.275157
Gglean004213	0	0	0.153125	0
Gglean004214	0	0	0	0
Gglean004215	0.400515	0	0	0
Gglean004216	3.067939266	2.85238915	2.270182965	2.7524735
Gglean004217	7.38448481	6.402493286	4.80862547	4.860095
Gglean004218	0	0	0	0
Gglean004219	0.375364	0	0	0
Gglean004220	0	0	0	0
Gglean004221	0	0	0	0
Gglean004222	0.110284	0	0.133328	0.0789392
Gglean004223	0.352981	0.56601	0.265417	0.145014

Gglean004224	2.533094	2.156068	2.491444	1.927571
Gglean004225	11.2334	10.6792	10.3908	15.0112
Gglean004226	7.09304158	6.810008283	4.436216002	3.803546752
Gglean004227	0.892743	0.925069	0.181926	0.324139
Gglean004228	0.885973	0.658696	0.290962	0.424325
Gglean004229	33.7795	31.7808	19.46452	16.62594
Gglean004230	20.85327	21.24978	15.25755	23.31
Gglean004231	60.1343	61.0336	15.21081	15.24676
Gglean004232	0	0	0	0.804289
Gglean004233	0	0	0	0
Gglean004234	0	0	0	0
Gglean004235	0	0	2.4898	0
Gglean004236	9.019867012	10.54200627	14.97662	14.286727
Gglean004237	9.019867012	10.54200627	14.97662	14.286727
Gglean004238	16.98072	16.01647	20.12527	19.29738
Gglean004239	58.60633793	63.74493198	47.38831187	42.95373227
Gglean004240	72.82547	71.134904	86.696599	83.3704647
Gglean004241	0	0	0	0
Gglean004242	23.5006	26.003	36.5778	40.3162
Gglean004243	15.95786995	14.897813	20.6106	19.488
Gglean004244	18.40607	20.37536	23.02808	24.3137
Gglean004245	50.61824	49.68188328	70.17500304	80.73560425
Gglean004246	50.61824	49.68188328	70.17500304	80.73560425
Gglean004247	50.256013	45.901705	61.29065	73.10834007
Gglean004248	50.256013	45.901705	61.29065	73.10834007
Gglean004249	62.8934	66.90144	62.71045	62.00844
Gglean004250	0	0	0	0
Gglean004251	3.9088273	3.30106	4.094623805	5.419617322
Gglean004252	3.9088273	3.30106	4.094623805	5.419617322
Gglean004253	3.9088273	3.30106	4.094623805	5.419617322
Gglean004254	28.235908	26.849987	24.028862	22.6185
Gglean004255	9.44346	11.8188	15.2962	18.0289
Gglean004256	165.2441005	158.4911808	129.9746113	128.3217961
Gglean004257	165.2441005	158.4911808	129.9746113	128.3217961
Gglean004258	32.61331409	35.29115665	26.85168352	24.07215844
Gglean004259	6.37582	5.60716	7.1625	5.37881
Gglean004260	113.1956793	110.3662904	94.503723	89.96119557
Gglean004261	113.1956793	110.3662904	94.503723	89.96119557
Gglean004262	113.1956793	110.3662904	94.503723	89.96119557
Gglean004263	0.0903505	0.0949145	0.398462	0.604561
Gglean004264	0.16252	0.167114	0.0661677	0.156366
Gglean004265	191.493	219.291	87.629	67.5536
Gglean004266	47.27560728	52.74942673	43.08551415	41.6781197
Gglean004267	13.10601761	14.64198747	21.695241	22.370006

Gglean004268	9.85332	11.3251	5.70256	7.10982
Gglean004269	39.8899235	37.833809	40.420812	39.8163175
Gglean004270	135.7991	123.2542	158.8724	179.8255
Gglean004271	93.2997	99.2046	88.9505	104.898
Gglean004272	30.1526	32.8064	17.03051	16.28266
Gglean004273	32.247	28.1351	31.7451	31.232
Gglean004274	57.33139207	64.59792479	69.116839	69.86196127
Gglean004275	57.33139207	64.59792479	69.116839	69.86196127
Gglean004276	57.33139207	64.59792479	69.116839	69.86196127
Gglean004277	15.187538	19.7952	25.47042	26.412639
Gglean004278	6.41358	4.81127	9.19933	10.05851
Gglean004279	30.57356888	31.10510428	39.115879	35.292245
Gglean004280	27.61211	23.661246	29.47803	34.58154015
Gglean004281	27.61211	23.661246	29.47803	34.58154015
Gglean004282	27.61211	23.661246	29.47803	34.58154015
Gglean004283	221.78468	254.67418	247.7368401	244.899179
Gglean004284	15.6088	16.5103	8.55018	6.68724
Gglean004285	0	0	0.112315	0
Gglean004286	28.38170095	31.55075651	31.18854	28.14179333
Gglean004287	28.38170095	31.55075651	31.18854	28.14179333
Gglean004288	45.83525078	48.76966546	61.8402664	57.94237969
Gglean004289	73.15243	70.27653	59.66851	60.69489
Gglean004290	19.2053	20.8001	11.3371	11.6755
Gglean004291	51.25884122	57.5441513	33.18429026	28.54982983
Gglean004292	709.825	699.816	719.432	603.608
Gglean004293	36.01998019	33.01476019	30.966793	26.07307
Gglean004294	51.45602	50.2796	29.28756	23.32899
Gglean004295	51.45602	50.2796	29.28756	23.32899
Gglean004296	19.3233	19.03039	13.13373	12.838087
Gglean004297	19.3233	19.03039	13.13373	12.838087
Gglean004298	0	0	0	0
Gglean004299	19.85159012	23.18462797	31.9099607	40.01217588
Gglean004300	0.614922	0.0798403	0.815963	0.893841
Gglean004301	4.45E-06	8.57E-06	7.01E-05	1.25E-05
Gglean004302	2.15731	3.52178	5.66687	4.15377
Gglean004303	13.9066	15.35751	22.182244	24.418774
Gglean004304	60.97896512	57.05205005	48.39723	53.55822162
Gglean004305	20.60603	22.9436	13.10854	11.19452
Gglean004306	0.0357054	0.0730969	0.0292413	0
Gglean004307	56.6389	58.48615	57.2612	58.74329
Gglean004308	24.45463	27.72702	17.17895	14.94634
Gglean004309	19.78291	21.49886	16.78691	19.3011
Gglean004310	13.90400957	12.98120882	23.208967	28.009573
Gglean004311	0.597775	0.83429	0.206433	0.325732

Gglean004312	7.98126	9.68257	9.76336	10.0777
Gglean004313	10.179768	9.7076	3.780373	3.25186
Gglean004314	16.3970423	16.10097948	16.33757212	16.05441138
Gglean004315	8.848826323	10.59103399	7.76658396	7.867809906
Gglean004316	1694.826	1611.955	1034.102	992.618
Gglean004317	61.6936	71.7767	26.81664	24.59114
Gglean004318	29.9988	38.5172	15.1981	13.4785
Gglean004319	23.18662	26.18622	35.2678	44.5324
Gglean004320	40.8336426	42.46068118	48.3797484	44.2647796
Gglean004321	40.8336426	42.46068118	48.3797484	44.2647796
Gglean004322	7.60082	8.88256	12.56953	16.75508
Gglean004323	23.38364654	24.56570252	34.83982352	41.86283089
Gglean004324	21.92003	29.83402723	38.99311	44.55401
Gglean004325	0	0	0	0
Gglean004326	61.1305041	75.730884	92.62844665	115.8680416
Gglean004327	21.98888089	23.13677379	20.38625281	20.00557505
Gglean004328	29.3936653	31.90154124	34.3534822	43.97317317
Gglean004329	0.69444701	0.521313019	1.760421	0.882799
Gglean004330	226.0371006	200.927612	523.7532003	581.902826
Gglean004331	77.68409965	73.18109761	180.8296872	194.9454004
Gglean004332	9.276848987	8.356661253	10.219271	13.192001
Gglean004333	13.49747558	15.93722092	9.147391725	6.502768291
Gglean004334	7.94876	6.12182	4.8976	4.78375
Gglean004335	1.54575	0.986681	0.90642	1.73776
Gglean004336	27.36591054	26.75919024	13.82534323	13.49490942
Gglean004337	15.75654267	19.64112	27.323247	28.965353
Gglean004338	5.15154	4.52472	6.33067	7.25773
Gglean004339	38.65622	41.62906	42.8814	42.90391
Gglean004340	12.549968	14.22218	20.23254	23.21409
Gglean004341	21.52271	22.11399856	11.9555904	12.75812
Gglean004342	19.31696	11.7047	10.54946	34.72768
Gglean004343	319.844042	245.220748	324.373404	378.3696858
Gglean004344	0.376925	0.0976488	0.127491	0.0902708
Gglean004345	41.4142	40.1572	64.6976	76.5831
Gglean004346	3.09562	4.29915	3.65674	4.87925
Gglean004347	9.957069938	11.10343936	5.767273875	5.177098236
Gglean004348	9.957069938	11.10343936	5.767273875	5.177098236
Gglean004349	29.606	31.6254	34.1761	35.1918
Gglean004350	35.05778597	32.7145118	48.29611396	55.5795851
Gglean004351	35.05778597	32.7145118	48.29611396	55.5795851
Gglean004352	35.05778597	32.7145118	48.29611396	55.5795851
Gglean004353	35.05778597	32.7145118	48.29611396	55.5795851
Gglean004354	0	0	0	0
Gglean004355	4.19336	4.796327	3.52301	7.57868

Gglean004356	30.7941134	31.998413	23.1159	18.97878638
Gglean004357	15.4311	17.8119	23.3481	30.5876
Gglean004358	25.5888	31.7145	23.0785	23.5952
Gglean004359	2.56915	1.83605	3.2878	3.46567
Gglean004360	29.69155	29.67496	37.02675	42.84023
Gglean004361	16.79254	19.12148	13.03342	12.16785
Gglean004362	20.2049066	30.2466383	43.797866	47.07408
Gglean004363	28.75785	26.88346504	36.33132	47.221207
Gglean004364	28.75785	26.88346504	36.33132	47.221207
Gglean004365	0	0	0	0
Gglean004366	2.5299	2.42975	3.12764	3.78355
Gglean004367	0	0.311419	0	0.288413
Gglean004368	33.89699	39.8212	38.3627	33.49303
Gglean004369	25.28714	28.9724122	27.73409019	26.69049301
Gglean004370	48.5491	42.7115	45.2792	43.1323
Gglean004371	224.1625	226.2825	129.9324	117.7933
Gglean004372	14.9907	14.2071	10.9881	10.4879
Gglean004373	18.2207	14.7155	13.3186	10.9396
Gglean004374	5.795725673	6.546967178	9.638580884	9.547550966
Gglean004375	0.401023	0.0767415	0.210734	0.466091
Gglean004376	28.45587558	26.43368301	33.74151655	38.13833476
Gglean004377	122.0639	113.7705	144.4433	127.4061
Gglean004378	182.8908	208.2363	201.7854	242.7197
Gglean004379	72.2383	75.3480475	76.25606	67.08701
Gglean004380	72.2383	75.3480475	76.25606	67.08701
Gglean004381	0	0.352216	0.269288	0.643094
Gglean004382	2.30E-07	1.01E-07	2.23E-07	1.27E-10
Gglean004383	33.13285	34.29571	31.36191	32.01014
Gglean004384	41.9253	39.0641	26.5959	28.4391
Gglean004385	1803.33	2111.7	1251.93	1193.79
Gglean004386	11.0488	18.0781	13.113	19.1177
Gglean004387	5.312831919	6.29852178	5.569125	7.530245
Gglean004388	0.00024194	1.69E-09	1.1046	0.475905
Gglean004389	23.6349	24.7987	20.0823	22.7269
Gglean004390	25.5244	22.459477	6.97763	4.13088
Gglean004391	17.1227	17.87129	13.34793	11.96251
Gglean004392	19.16223	21.17008	21.56074	20.22645
Gglean004393	110.52866	109.46685	92.66101498	90.81211006
Gglean004394	110.52866	109.46685	92.66101498	90.81211006
Gglean004395	0	0	0	0
Gglean004396	20.1703	21.0136	5.73249	4.82233
Gglean004397	476.461	557.921	648.145	736.147
Gglean004398	37.70243	37.20309813	38.22967383	34.414264
Gglean004399	43.28874206	42.58803133	61.50147832	67.52775553

Gglean004400	1.482316	1.249298	1.439561	1.845491
Gglean004401	40.73924341	42.0741696	31.51608265	30.0809091
Gglean004402	0.72321	0.656561	0.947373	0.6052
Gglean004403	0	0.186378	0.19751	0
Gglean004404	0	0	0	0
Gglean004405	248.2315	182.4574	280.906	256.9945
Gglean004406	1.640593	0.3053	1.704574002	2.473444
Gglean004407	10.4003	9.91656	9.65502	7.47729
Gglean004408	22.38175	20.61711	16.28856	16.07977
Gglean004409	20.84589258	24.26204545	14.06085754	15.76229081
Gglean004410	54.59027	53.4186	54.54647	47.59208323
Gglean004411	54.59027	53.4186	54.54647	47.59208323
Gglean004412	11.1269	13.4026	4.06716	3.40526
Gglean004413	19.48075148	21.11985019	20.78161	18.463176
Gglean004414	11.285675	13.35591	18.55002	23.30661
Gglean004415	65.33461828	62.634632	84.07787699	88.078331
Gglean004416	5.80037	7.23261	2.250415	1.3246977
Gglean004417	11.6384	13.8769	20.2128	19.6209
Gglean004418	22.0961	20.7532	16.9938	16.361
Gglean004419	4.801498	4.8985448	4.791627	5.977847
Gglean004420	7.101870292	5.62246494	5.209352301	3.609740006
Gglean004421	7.101870292	5.62246494	5.209352301	3.609740006
Gglean004422	0	0	0	0
Gglean004423	11.00712436	11.91725009	13.82630383	16.059144
Gglean004424	7.45961	7.33736	12.09912	15.0542
Gglean004425	45.842485	55.555205	38.777399	43.768064
Gglean004426	45.842485	55.555205	38.777399	43.768064
Gglean004427	39.9642	42.5133	39.8685	43.2268
Gglean004428	0.24417	0	0	0
Gglean004429	38.26229	41.71074	15.062102	12.377272
Gglean004430	12.34101	11.46572	8.66138	8.32493
Gglean004431	52.0725	61.8907	59.037	90.2916
Gglean004432	0.741536	1.19692	0.30701	0
Gglean004433	1.85289	3.063238	2.301147	2.46771
Gglean004434	0.169763	0	0.138129	0.326534
Gglean004435	1.232191573	0.850162158	1.1690245	1.884423042
Gglean004436	0.259733	0.148205	0.302700014	9.82E-08
Gglean004437	16.03308965	19.37733	4.55172698	4.230897
Gglean004438	21.8699	20.51904	32.98542	43.32049
Gglean004439	0.863063744	1.192675931	2.495590157	2.271031072
Gglean004440	2.91E-32	2.10E-16	1.08E-05	9.69E-13
Gglean004441	1.0170474	0.62446	0.720127	0.864493
Gglean004442	20.43147332	20.27506657	16.58833119	16.78841777
Gglean004443	30.0632299	29.423373	9.1453483	7.667285601

Gglean004444	11.690297	13.87111	7.00205	7.25532
Gglean004445	25.94736	23.05271	30.36494	30.08949
Gglean004446	18.12824	20.22452	20.1984	18.93213
Gglean004447	0	0	0	0
Gglean004448	13.55164	17.77388	16.92173	23.44043
Gglean004449	13.55164	17.77388	16.92173	23.44043
Gglean004450	21.09990037	29.15510045	28.60530024	40.60920037
Gglean004451	23.81962	25.36943	17.840519	19.802924
Gglean004452	23.81962	25.36943	17.840519	19.802924
Gglean004453	42.52166026	41.99016788	40.79953124	40.10476047
Gglean004454	13.74	14.0103	9.78491	9.20032
Gglean004455	13.11551	14.203128	13.80427	15.04743
Gglean004456	7.33968	9.19385	2.52158	1.87972
Gglean004457	0.000494281	9.20E-23	0.0141456	0.000548763
Gglean004458	5.996805481	5.438521601	5.125361	4.187824
Gglean004459	16.081387	17.779225	7.93811	6.843268
Gglean004460	88.761022	98.408438	76.739325	69.27100566
Gglean004461	88.761022	98.408438	76.739325	69.27100566
Gglean004462	88.761022	98.408438	76.739325	69.27100566
Gglean004463	25.5966	25.2392	8.19181	6.17336
Gglean004464	0.670922436	3.2940958	1.392079625	1.71955237
Gglean004465	0.670922436	3.2940958	1.392079625	1.71955237
Gglean004466	0	0	0	0
Gglean004467	0	0	0	0
Gglean004468	0	0	0	0
Gglean004469	2.1846	2.07202	2.95191	3.07734
Gglean004470	0.0599243	0.0618261	0.0487247	0
Gglean004471	0	0	0	0
Gglean004472	0	0	0.0360254	0.0851144
Gglean004473	0	0	0	0
Gglean004474	0	0	0	0
Gglean004475	3.80E-06	1.79576	0	0
Gglean004476	3.80E-06	1.79576	0	0
Gglean004477	14.9514	16.251	6.41097	7.03018
Gglean004478	2.803384167	4.128313127	4.293744635	5.129498693
Gglean004479	2.23489	1.87719	1.9517	1.50503
Gglean004480	1.68355	1.48767	1.90472	2.20117
Gglean004481	0	0	0.0775243	0
Gglean004482	0	0	0	0
Gglean004483	0	0	0	0
Gglean004484	0.78410744	1.738313	1.452149	1.279999
Gglean004485	0	0	0	0
Gglean004486	0	0	0	0
Gglean004487	0	0	0	0

Gglean004488	0	0	0	0
Gglean004489	0	0	0	0
Gglean004490	0.50758203	0.219678068	0.443357513	0.40569978
Gglean004491	31.34313462	30.96002292	27.83267486	26.45420046
Gglean004492	1.08838	1.17806	0.606696	0.722479
Gglean004493	5.20943	5.08799	1.58388	1.44584
Gglean004494	3.43417	4.45269	1.9348	1.84621
Gglean004495	7.61174	1.690806	11.31112	13.49531
Gglean004496	0.181868	0	0	0
Gglean004497	1.873082	3.970677	0.67212	0.842815
Gglean004498	45.56314658	54.13394431	51.13067408	46.43846687
Gglean004499	0	0	0	0
Gglean004500	0	0	0	0
Gglean004501	0	0	0	0
Gglean004502	0	0	0	0
Gglean004503	0	0	0	0
Gglean004504	3.236733	2.644042098	5.266479	5.467813099
Gglean004505	35.361392	41.3233531	32.542017	34.6379045
Gglean004506	33.14898	37.78876	56.34543	71.55489
Gglean004507	33.14898	37.78876	56.34543	71.55489
Gglean004508	32.292616	33.426464	39.43285901	43.683627
Gglean004509	1014.877	1008.913	626.2538	600.5218
Gglean004510	5.114011951	6.420057376	12.4282045	8.539377
Gglean004511	0	0	0	0
Gglean004512	25.4034	25.9761	22.8243	23.0227
Gglean004513	0.09395702	0.080850543	0.177254213	0.314630833
Gglean004514	0	0.110743	0	0
Gglean004515	5.007406315	5.287730038	2.024736427	1.82130382
Gglean004516	1203.53	1188.6	796.641	751.749
Gglean004517	30.7699	30.1435	21.5822	25.0151
Gglean004518	21.61249	24.5577	33.2199	33.7594
Gglean004519	310.3488	335.0776	288.1802	304.4415
Gglean004520	0	0	0.057495275	0
Gglean004521	7.809518	7.421507	11.91113017	12.69955008
Gglean004522	0	0.121674	0.333283	0.112928
Gglean004523	0.483848	1.03022	0	0
Gglean004524	0.779411	0.41005	0.316477	0
Gglean004525	0	0	0	0
Gglean004526	0	0	0	0
Gglean004527	0	0	0	0
Gglean004528	0	0	0	0
Gglean004529	0	0	0	0
Gglean004530	0	0	0	0
Gglean004531	0	0	0	0

Gglean004532	0.375420109	0.533905702	0.467078111	0.188116468
Gglean004533	0	0.073341	0	0
Gglean004534	0	0	0	0
Gglean004535	0	0	0	0
Gglean004536	0	0	0	0
Gglean004537	0.0596072	0.0306578	0.0240198	0
Gglean004538	48.775912	51.326	35.20082326	30.95862368
Gglean004539	48.775912	51.326	35.20082326	30.95862368
Gglean004540	18.39595	19.99481	11.30647	11.28459
Gglean004541	0	0	0	0.100465307
Gglean004542	371.6886346	309.447378	221.8734137	230.7669951
Gglean004543	16.19454	15.3848011	12.798979	11.8681052
Gglean004544	16.19454	15.3848011	12.798979	11.8681052
Gglean004545	50.4818	59.3567	58.917	65.6053
Gglean004546	2.593331531	2.283306	3.171374	3.682238859
Gglean004547	3.689185516	4.13573395	5.090347675	4.383190349
Gglean004548	53.66361	46.22088	49.46366	52.2316
Gglean004549	1.71519	1.49667	1.33207	1.39813
Gglean004550	1.24606	1.40743	1.56444	1.95129
Gglean004551	28.786	28.044271	46.42028	49.78124
Gglean004552	25.21022415	23.33392279	38.63618379	45.19144685
Gglean004553	19.2429	18.0977	25.6208	28.6807
Gglean004554	0.0363315	0	0	0
Gglean004555	0	4.36656	2.1084	2.50269
Gglean004556	0.0498151	0.208422	0	0
Gglean004557	0.14951817	0.082481852	0.043188207	0
Gglean004558	0	0	0	0
Gglean004559	1.11618	0.793189	0.6506425	0.0197645
Gglean004560	2.22004	0.455204	0	0
Gglean004561	0	0	0	0
Gglean004562	0.150829	0.0516675	0.203173	0.192841
Gglean004563	0.382078	0.305071	0.171359	0
Gglean004564	0	0	0	0
Gglean004565	0.642743323	1.036130695	0.854303441	0.467572194
Gglean004566	0.362765	0.190605	0.438766	0.174517
Gglean004567	0	0.396597	0.152872	0
Gglean004568	0.457715272	1.319353	0.443624605	0.262168756
Gglean004569	0	0	0	0
Gglean004570	0.852243	0	0	0
Gglean004571	0.670702	0	0	0.391193
Gglean004572	2.127071	1.960270888	1.358677	1.319161838
Gglean004573	0	0	0	0
Gglean004574	1.28643827	1.741570795	2.075456011	1.940070675
Gglean004575	16.74692138	15.133855	14.47703056	13.85706304

Gglean004576	16.74692138	15.133855	14.47703056	13.85706304
Gglean004577	31.04951814	31.02014529	58.00100162	56.0152321
Gglean004578	31.04951814	31.02014529	58.00100162	56.0152321
Gglean004579	12.645707	13.52303812	18.587749	20.591062
Gglean004580	12.645707	13.52303812	18.587749	20.591062
Gglean004581	0	0	0	0
Gglean004582	0	0	0	0
Gglean004583	0	0	0.176323	0
Gglean004584	0	0.0952475	0	0
Gglean004585	0	0	0	0
Gglean004586	0	0.468566	0.352724	0
Gglean004587	0	0	0	0
Gglean004588	0	0	0	0
Gglean004589	0	0	0	0
Gglean004590	0	0.0834178	0	0.157671
Gglean004591	15.3617335	16.64780183	24.6561391	29.677637
Gglean004592	15.3617335	16.64780183	24.6561391	29.677637
Gglean004593	41.4381814	39.52919673	25.65202994	21.72019698
Gglean004594	0	0	0	0
Gglean004595	0	0	0	0
Gglean004596	0	0	0	0
Gglean004597	0	0	0	0
Gglean004598	0	0	0	0
Gglean004599	3.192253503	2.70217097	3.80392353	6.025603669
Gglean004600	3.192253503	2.70217097	3.80392353	6.025603669
Gglean004601	0.619660224	0.887611214	0.867813428	0.802600297
Gglean004602	0	0	0	0
Gglean004603	0	0	0	0
Gglean004604	0	0	0	0
Gglean004605	0	0	0	0
Gglean004606	0	0	0	0
Gglean004607	0	0	0	0
Gglean004608	173.84418	148.51094	192.02233	243.5780463
Gglean004609	72.276	75.4282	30.98	29.2386
Gglean004610	37.4238	35.7551	23.95602	20.91434
Gglean004611	27.99055551	25.5337572	27.9027925	29.35785374
Gglean004612	0	0	0	0
Gglean004613	0.0384363	0.158397	0.0929587	0.0366432
Gglean004614	1.084395	1.985277	0.677093	1.06027
Gglean004615	22.49203	22.25162	17.10202963	16.52262158
Gglean004616	22.49203	22.25162	17.10202963	16.52262158
Gglean004617	1548.51	1493.24	1033.97	982.26
Gglean004618	63.12089783	62.1208622	52.46748197	48.17045
Gglean004619	25.28938806	22.79920976	14.0697934	11.22289715

Gglean004620	1529.53291	1468.76992	1027.12993	940.82049
Gglean004621	6.792441	6.295331347	2.4631215	2.258789721
Gglean004622	29.58204	35.85292	42.71621	47.90404
Gglean004623	422.923	503.027	156.6357	129.592
Gglean004624	10.66077304	10.77041868	6.211819482	7.011309193
Gglean004625	0	0	0.0275194	0
Gglean004626	146.00307	154.5034	110.1095	92.858305
Gglean004627	5.585759	5.4857	0.1970965	0.1550192
Gglean004628	5.585759	5.4857	0.1970965	0.1550192
Gglean004629	2.283446831	3.454048369	3.976802585	3.995171389
Gglean004630	2.283446831	3.454048369	3.976802585	3.995171389
Gglean004631	2.283446831	3.454048369	3.976802585	3.995171389
Gglean004632	3.7582961	3.360145668	2.247332521	2.9854084
Gglean004633	2.655663492	2.625987	5.039933159	5.2210854
Gglean004634	0	0	0	0
Gglean004635	0	0	0	0
Gglean004636	0.902221	2.05216	0	0.892898
Gglean004637	0	0	0	0
Gglean004638	0	0.177281	0	0.329603
Gglean004639	14.2778137	15.26350887	17.67180853	17.3175243
Gglean004640	1.554704	1.914374	1.935049	2.37993
Gglean004641	0.56998	0.403265055	0.498529276	0.994061347
Gglean004642	13.57560385	13.93726091	10.30917561	12.752522
Gglean004643	13.57560385	13.93726091	10.30917561	12.752522
Gglean004644	31.455	36.38808	24.75841	23.03788
Gglean004645	26.69586273	29.28586111	8.366405804	8.361608107
Gglean004646	26.69586273	29.28586111	8.366405804	8.361608107
Gglean004647	32.37577903	32.362473	32.127486	27.790504
Gglean004648	33.05499642	27.7165	23.06483631	23.14800806
Gglean004649	0.275528	0.223629	0.0447727	0.0531856
Gglean004650	9.969121	11.62955	3.042206268	3.383198147
Gglean004651	9.969121	11.62955	3.042206268	3.383198147
Gglean004652	9.969121	11.62955	3.042206268	3.383198147
Gglean004653	0	0	0	0
Gglean004654	0	0	0	0.805111
Gglean004655	9.071184	13.127003	5.665398	7.449564
Gglean004656	0.369956	0.388352	0.101522	0.24051
Gglean004657	0	0	0	0
Gglean004658	0.087961913	0.37443	0.196283094	0.603038528
Gglean004659	28.16986019	34.86651716	36.29861849	39.3137416
Gglean004660	28.16986019	34.86651716	36.29861849	39.3137416
Gglean004661	53.77894	59.09857	43.35058	38.97249
Gglean004662	221.2453484	200.2619961	299.2912386	378.6309663
Gglean004663	26.46490083	28.18807	25.76623141	25.40880333

Gglean004664	52.239105	53.40212	41.55696322	36.626384
Gglean004665	15.3628	12.419	17.1147	14.7081
Gglean004666	45.43626	39.88296	25.50391	25.12843
Gglean004667	0	0	0	0.248828
Gglean004668	26.90685	26.6047	26.34806	24.58108
Gglean004669	14.882099	14.9781	17.43759851	21.216726
Gglean004670	53.866	55.8695	53.7904	50.6682
Gglean004671	1.47544	1.202483345	1.651487	2.565761
Gglean004672	68.63269661	71.20442553	42.04725717	40.96934405
Gglean004673	42.28036	46.88766	51.12432	41.3612106
Gglean004674	112.3048	134.3036	43.8671	32.31507
Gglean004675	2.839372607	4.030402	5.03246907	14.4041498
Gglean004676	2.839372607	4.030402	5.03246907	14.4041498
Gglean004677	2.839372607	4.030402	5.03246907	14.4041498
Gglean004678	2.839372607	4.030402	5.03246907	14.4041498
Gglean004679	12.175648	12.92471778	8.833489713	9.106947641
Gglean004680	0	0	0	0
Gglean004681	0.247652192	0.3312857	0.8572475	0.6601469
Gglean004682	0	0	0	0
Gglean004683	42.26112206	33.24127481	42.80924071	53.71844902
Gglean004684	42.26112206	33.24127481	42.80924071	53.71844902
Gglean004685	31.270327	33.86013	35.61850374	35.165455
Gglean004686	112.851442	129.84731	93.54012878	83.26510371
Gglean004687	686.80971	714.351169	762.9360002	846.034295
Gglean004688	21.2305379	23.1997994	28.17417459	32.8677877
Gglean004689	0.154071	0	0	0
Gglean004690	19.28465	20.567173	19.07845	17.34595614
Gglean004691	67.862762	72.15124886	103.880062	99.501451
Gglean004692	67.862762	72.15124886	103.880062	99.501451
Gglean004693	67.862762	72.15124886	103.880062	99.501451
Gglean004694	34.65249017	35.6595	24.07080273	22.11706013
Gglean004695	41.276809	44.9539695	41.0256667	35.5673066
Gglean004696	0	0	0	0
Gglean004697	4.16124	4.01356	8.0323	8.57108
Gglean004698	128.44992	135.74021	188.08552	218.41084
Gglean004699	23.136185	22.166532	34.914303	39.994714
Gglean004700	113.370803	126.142036	43.85780538	36.99356141
Gglean004701	0.0935819	0.0433636	0.0553272	0.0884935
Gglean004702	1.764696	1.890035	1.96562	1.231852
Gglean004703	17.73397466	21.87172547	23.71217908	28.13809411
Gglean004704	59.41690427	55.614266	65.14338617	61.78514237
Gglean004705	0	0	0	0
Gglean004706	45.4625	47.381	43.4057	49.551
Gglean004707	58.56408818	62.95327396	89.15801665	88.60484486

Gglean004708	5.730118403	5.770490828	4.98598789	6.784288863
Gglean004709	0.394528389	1.10536226	0.3314282	0.107189749
Gglean004710	18.46602807	19.47907065	12.78476491	10.43161264
Gglean004711	18.46602807	19.47907065	12.78476491	10.43161264
Gglean004712	56.320067	57.76190734	62.43749446	58.65133457
Gglean004713	56.320067	57.76190734	62.43749446	58.65133457
Gglean004714	68.63532598	65.06232318	59.37639969	63.30639624
Gglean004715	0	0	0	0
Gglean004716	17.5026	15.9806	15.2737	14.1278747
Gglean004717	38.644398	53.65432	40.13548	36.58977
Gglean004718	38.644398	53.65432	40.13548	36.58977
Gglean004719	1.699580035	2.10257	0.804148108	0.680273
Gglean004720	9.05377	6.7502	7.735441	6.17999
Gglean004721	9.05377	6.7502	7.735441	6.17999
Gglean004722	0	0	0	0
Gglean004723	0	0	0	0
Gglean004724	0	0	0.0580913	0
Gglean004725	3.768037332	4.205916013	4.872313698	5.338056214
Gglean004726	3.768037332	4.205916013	4.872313698	5.338056214
Gglean004727	3.768037332	4.205916013	4.872313698	5.338056214
Gglean004728	9.09956	4.68489	6.35E-15	1.55E-211
Gglean004729	189.81038	152.296341	162.623064	210.37113
Gglean004730	20.07389	24.73561	14.8859	11.30305
Gglean004731	20.07389	24.73561	14.8859	11.30305
Gglean004732	81.42297526	93.93206669	15.61107952	13.45102376
Gglean004733	32.56186	38.85205407	29.14683005	28.22063573
Gglean004734	32.56186	38.85205407	29.14683005	28.22063573
Gglean004735	17.3994307	18.0211268	28.0951543	29.5724731
Gglean004736	64.66080473	64.91997609	59.92679514	57.54367901
Gglean004737	14.07114	6.39371	7.2931	4.99273
Gglean004738	17.71267	19.57211	9.11055	7.783559
Gglean004739	35.80802	33.69177	39.80955	35.19595
Gglean004740	4.885669569	11.98852901	3.938998034	3.073638084
Gglean004741	4.885669569	11.98852901	3.938998034	3.073638084
Gglean004742	19.599164	18.3870229	30.258957	22.87740343
Gglean004743	0	0	0	0
Gglean004744	0	0	0	0
Gglean004745	0	0	5.03851	0
Gglean004746	0	0	0	0.393654
Gglean004747	0.123474	0	0.0995457	0
Gglean004748	0.193141	0.20115	0.313178	0.309041
Gglean004749	0	0	0.0534695	0
Gglean004750	0	0	0	0
Gglean004751	25.37223342	28.5039231	14.885299	12.68595551

Gglean004752	25.37223342	28.5039231	14.885299	12.68595551
Gglean004753	32.10097	31.39853293	41.910796	50.449687
Gglean004754	11.69892	12.73013	10.60115	11.46834
Gglean004755	0	0	0	0.599702
Gglean004756	1.662744	0.547243994	0.617286	0.8952538
Gglean004757	16.63823411	15.25274561	27.25982685	33.892237
Gglean004758	8.38E-06	0.000537563	0.438846	1.06337
Gglean004759	0.995441229	0.5199154	0.825516459	0.226860473
Gglean004760	38.73599	47.92089	30.20036	28.83099
Gglean004761	0	0	0	0
Gglean004762	21.10175945	20.26040719	26.73720408	23.37300638
Gglean004763	28.744464	28.13054	44.892989	51.74583
Gglean004764	28.744464	28.13054	44.892989	51.74583
Gglean004765	0	0	0	0
Gglean004766	0	0	0	0
Gglean004767	0	0	0.154557	0
Gglean004768	0.0229833	0	7.00E-18	3.34E-25
Gglean004769	0	0	0	0
Gglean004770	0	0	0	0
Gglean004771	7.39432	8.98842	4.37625	5.46147
Gglean004772	58.459512	58.763218	58.98291936	62.08968152
Gglean004773	58.459512	58.763218	58.98291936	62.08968152
Gglean004774	58.459512	58.763218	58.98291936	62.08968152
Gglean004775	7.98061757	11.19259617	6.539096	5.187713447
Gglean004776	7.98061757	11.19259617	6.539096	5.187713447
Gglean004777	0.0955262	0.148887	0.135455	0.0912357
Gglean004778	0	0	0	0
Gglean004779	0	0	0	0
Gglean004780	0	0	0	0
Gglean004781	2.934678497	2.741683	1.488618763	1.473821291
Gglean004782	0	0	0	0
Gglean004783	0	0	0	0
Gglean004784	36.4405259	32.11972489	57.40671738	61.4667595
Gglean004785	36.4405259	32.11972489	57.40671738	61.4667595
Gglean004786	36.4405259	32.11972489	57.40671738	61.4667595
Gglean004787	36.4405259	32.11972489	57.40671738	61.4667595
Gglean004788	0.0865613	0.357012	0.139634	0.248732
Gglean004789	46.0439	46.3962	32.11237	26.93578
Gglean004790	0.366129	3.1745	3.32E-06	0.195725
Gglean004791	4.038330822	3.367920123	5.714273105	3.365385
Gglean004792	0	0	0	0
Gglean004793	0	0	0	0
Gglean004794	0	0	0	0
Gglean004795	0	0	0	0

Gglean004796	0	0	0	0
Gglean004797	7.682107337	8.160017788	10.24265229	11.13787275
Gglean004798	7.682107337	8.160017788	10.24265229	11.13787275
Gglean004799	71.58170513	70.61079661	89.90371	94.06697175
Gglean004800	25.35726	23.97327	35.23613	37.90564
Gglean004801	5.96353	6.08757	3.91405	3.63246
Gglean004802	41.580849	43.49298	40.11466	37.794046
Gglean004803	41.580849	43.49298	40.11466	37.794046
Gglean004804	32.257895	38.61631	63.7242	65.46315
Gglean004805	40.6461966	48.78756	45.91742989	37.4207571
Gglean004806	40.6461966	48.78756	45.91742989	37.4207571
Gglean004807	40.6461966	48.78756	45.91742989	37.4207571
Gglean004808	57.18238	55.50845813	55.992933	59.3484871
Gglean004809	0.000376454	0.0328516	0.000340927	0.000565146
Gglean004810	1301.558838	1450.42611	951.7332461	911.841715
Gglean004811	143.2806	86.4968	132.1204	168.8679
Gglean004812	64.6247	83.6034	112.941	94.545
Gglean004813	0	0	0	0
Gglean004814	21.19218198	21.2862508	31.2956724	30.7643731
Gglean004815	0	0	0	0
Gglean004816	21.19218198	21.2862508	31.2956724	30.7643731
Gglean004817	14.94115434	17.42199181	2.514391265	2.948472179
Gglean004818	14.94115434	17.42199181	2.514391265	2.948472179
Gglean004819	0	0	0	0
Gglean004820	0	0	0	0
Gglean004821	0	0	0	0
Gglean004822	0	0	0	0
Gglean004823	0	0.326686	0	0
Gglean004824	68.33644846	76.28770074	52.84581004	44.01391461
Gglean004825	23.70061	19.52593	34.37945	33.4434
Gglean004826	26.1356326	30.78237488	16.9614088	15.1282
Gglean004827	31.07206408	37.65700153	32.8424	29.76644
Gglean004828	5.75553	6.60313	4.7413	0
Gglean004829	35.80689	35.35659	28.083403	28.573025
Gglean004830	35.80689	35.35659	28.083403	28.573025
Gglean004831	35.80689	35.35659	28.083403	28.573025
Gglean004832	15.740887	17.97937	18.28965	22.0044
Gglean004833	15.72398	22.344419	30.1648337	30.34121245
Gglean004834	15.72398	22.344419	30.1648337	30.34121245
Gglean004835	15.72398	22.344419	30.1648337	30.34121245
Gglean004836	172.91	131.563	86.3028	90.7648
Gglean004837	18.27890041	19.6860303	5.792707216	4.6277465
Gglean004838	16.44069969	15.98547174	19.49965367	22.054414
Gglean004839	0.0866183	0.0453256	0	0

Gglean004840	66.86971	66.70382	67.87290373	66.23457
Gglean004841	9.01696	8.11685	15.7848	16.1876
Gglean004842	21.68151	20.96245	14.28863	13.76275
Gglean004843	4.78E-83	1.10E-177	6.32E-104	1.88E-19
Gglean004844	22.961327	26.94876419	23.77987	22.80473
Gglean004845	1.032754	0.9339802	1.99225	2.639395
Gglean004846	134.8231	153.7486	66.5157	64.95121
Gglean004847	82.69940004	87.31869	44.9349	39.91109
Gglean004848	82.69940004	87.31869	44.9349	39.91109
Gglean004849	0.249564021	0	0	0
Gglean004850	51.39465558	50.5546328	49.75492347	46.56288153
Gglean004851	9.07469	12.03299	3.97692	2.5528
Gglean004852	2.216211	2.232181908	3.640756	3.425116
Gglean004853	10.391085	8.88507	12.46695	15.02992
Gglean004854	215.800826	220.93151	217.58124	255.57683
Gglean004855	215.800826	220.93151	217.58124	255.57683
Gglean004856	51.693872	52.10004	48.458706	52.827202
Gglean004857	38.5998	41.8996	39.8209	34.7982
Gglean004858	1140.418	1143.305	737.323	684.712
Gglean004859	71.6407475	78.8613943	37.69307501	29.63199732
Gglean004860	12.16071	11.497055	8.628697	9.6699
Gglean004861	20.609036	27.2999	42.6584	42.1299
Gglean004862	26.90685	27.2999	42.6584	42.1299
Gglean004863	72.6176	77.0618	81.0448	93.5748
Gglean004864	0.31164725	0.284598	0.453729053	0.0435712
Gglean004865	0.31164725	0.284598	0.453729053	0.0435712
Gglean004866	21.41905	24.95052	38.6824	33.5009
Gglean004867	22.10247719	22.055462	22.49847564	21.7239058
Gglean004868	9.540808	8.937764825	12.7143198	10.86276834
Gglean004869	111.93948	108.54572	57.79233793	58.3435555
Gglean004870	111.93948	108.54572	57.79233793	58.3435555
Gglean004871	111.93948	108.54572	57.79233793	58.3435555
Gglean004872	4.82884	4.86009	2.8109838	2.92108
Gglean004873	95.0176	97.2339	69.7003	60.28078
Gglean004874	95.0176	97.2339	69.7003	60.28078
Gglean004875	30.31916	25.67184	36.9328	40.7561
Gglean004876	19.900006	33.376353	21.634013	13.747724
Gglean004877	19.900006	33.376353	21.634013	13.747724
Gglean004878	0	0	0	0
Gglean004879	13.90598	17.14682	14.60213	10.83941
Gglean004880	13.90598	17.14682	14.60213	10.83941
Gglean004881	20.12061302	21.06611913	18.340718	15.60178
Gglean004882	8.59994	11.6331	17.2465	17.668
Gglean004883	88.08758	90.96348658	24.304386	21.569214

Gglean004884	5.81456	5.128058	8.40326	9.267134
Gglean004885	0.0387671	0.039328	0.0549024	0.0557175
Gglean004886	0	0	0	0
Gglean004887	0.0521768	0.105216	0	0.0993765
Gglean004888	0.157541	0	0.254965	0
Gglean004889	0	0	0	0
Gglean004890	0	0	0.123572	0.0181662
Gglean004891	0.0400135	0	0	0
Gglean004892	0	0	0	0
Gglean004893	0.0287411	0	0.0686173	0.134824
Gglean004894	1.22135	0.564021	0.863231	0.68612
Gglean004895	0	0	0	0
Gglean004896	0	0.0731304	0.0289172	0.0344222
Gglean004897	0	0	0	0
Gglean004898	0	0	0.155974	0
Gglean004899	0	0	0	0
Gglean004900	0	0	0	0
Gglean004901	0	0	0.0515425	0
Gglean004902	0.0234208	0	0	0
Gglean004903	0	0	0	0
Gglean004904	0	0	0	0
Gglean004905	0	0	0	0
Gglean004906	13.0767	11.5916	10.9641	10.5331
Gglean004907	0	0	0	0
Gglean004908	0	0	0.12412	0
Gglean004909	0	0	0	0
Gglean004910	137.0913577	134.5522068	139.712783	214.108983
Gglean004911	0	0	0	0
Gglean004912	0	0	0	0
Gglean004913	0	0	0.0893202	0
Gglean004914	1.3620336	2.022312883	1.807482685	2.064077627
Gglean004915	0	0	0	0
Gglean004916	0	0	0	0
Gglean004917	0.312942	0.16286	0.643166	0.457936
Gglean004918	0	0	0	0
Gglean004919	0	0	0	0
Gglean004920	36.79197186	43.36901401	40.47446596	61.58349916
Gglean004921	24.76505001	23.318543	18.67331393	15.10928
Gglean004922	36.85307	36.13686	20.83267	17.25403
Gglean004923	2.0432333	1.927311	2.742442	2.686006272
Gglean004924	19.4601	23.5661	21.2044	19.7109
Gglean004925	52.94959	60.60004	41.94706	40.72457
Gglean004926	28.0256	25.5487	33.1054	40.72523
Gglean004927	0	0	0	0

Gglean004928	18.30461667	21.81297214	18.23135444	20.815443
Gglean004929	70.4294	67.1467	65.1383	63.3462
Gglean004930	3105.25435	3421.45167	2102.85467	1851.933885
Gglean004931	3105.25435	3421.45167	2102.85467	1851.933885
Gglean004932	0	0	0	0
Gglean004933	0.542765008	1.06948407	1.076260258	0.755880406
Gglean004934	0	0	0	0
Gglean004935	0	0	0	0
Gglean004936	0	0	0	0
Gglean004937	0	0	0	0
Gglean004938	0	0	0	0
Gglean004939	0	0	0	0
Gglean004940	0	0	0	0
Gglean004941	0	0	0	0
Gglean004942	0	0	0	0
Gglean004943	0	0	0	0
Gglean004944	0.702211	0.680716	0.358188	0.211646
Gglean004945	0.0542883	0.111906	0.0437539	0.0516345
Gglean004946	0	0	0	0
Gglean004947	87.83116032	91.39695673	98.37720295	93.1817431
Gglean004948	87.83116032	91.39695673	98.37720295	93.1817431
Gglean004949	87.83116032	91.39695673	98.37720295	93.1817431
Gglean004950	3.08687	2.22511	1.44349	2.02822
Gglean004951	11.4372	9.27182	6.46494	5.48744
Gglean004952	9.16856	6.7106	5.26536	5.12136
Gglean004953	13.381	14.3622	9.24852	8.37389
Gglean004954	0.23623	0.305566	0	0
Gglean004955	0	0	0	0
Gglean004956	0.0561436	0	0	0
Gglean004957	0	0	0	0
Gglean004958	0	0	0	0
Gglean004959	0	0	0	0
Gglean004960	0	0	0	0
Gglean004961	0	0	0	0
Gglean004962	0	0	0	0
Gglean004963	0	0	0	0
Gglean004964	0	0	0	0
Gglean004965	0.476673	0	0	0
Gglean004966	0	0	0	0
Gglean004967	0	0	0	0
Gglean004968	0	0	0	0
Gglean004969	0	0	0	0
Gglean004970	0	0	0	0
Gglean004971	0	0	0	0

Gglean004972	0	0	0	0
Gglean004973	0	0.0635657	0	0
Gglean004974	0	0	0	0
Gglean004975	21.4649	24.8464	22.7818	23.7671
Gglean004976	0	0	0.0553441	0.131119
Gglean004977	0	0	0	0
Gglean004978	0	0	1.05661	0
Gglean004979	0	0	0.0303925	0
Gglean004980	0	0	0	0
Gglean004981	0	0	0	0
Gglean004982	10.9563	9.09282	8.29189	5.90829
Gglean004983	23.2966	19.919	11.3505	13.256
Gglean004984	19.97142735	23.4043433	18.6251069	23.24031082
Gglean004985	19.97142735	23.4043433	18.6251069	23.24031082
Gglean004986	0.207168	0	0	0
Gglean004987	0	0	0	0
Gglean004988	0	0	0	0
Gglean004989	0	0	0	0
Gglean004990	0.269898	8.77E-06	4.37E-06	0
Gglean004991	0.0724019	0.0761457	0	0.0693746
Gglean004992	0.217158	0	0.0586728	0.0694738
Gglean004993	0	0	0	0
Gglean004994	0	0.343077202	0.267889102	0.212036319
Gglean004995	0.143488	0.047609	0	0.000129487
Gglean004996	5.348766718	4.871698212	6.716218791	7.565468622
Gglean004997	44.34597804	51.29311187	23.87098343	21.20881527
Gglean004998	0	0	0.0824643	0.194883
Gglean004999	18.5574	19.1372	4.94637	6.88728
Gglean005000	1093.385	1086.804	728.307	677.707
Gglean005001	0	0	0	0
Gglean005002	0	0	0	0
Gglean005003	0	0	0	0
Gglean005004	0.190251	0.501400401	0.469544	0.748333
Gglean005005	65.89066	69.22825	53.19038	47.56427
Gglean005006	65.89066	69.22825	53.19038	47.56427
Gglean005007	20.51150907	19.506866	14.26535302	12.37328111
Gglean005008	102.8907546	110.7318966	117.1922018	117.5491508
Gglean005009	102.8907546	110.7318966	117.1922018	117.5491508
Gglean005010	102.8907546	110.7318966	117.1922018	117.5491508
Gglean005011	102.8907546	110.7318966	117.1922018	117.5491508
Gglean005012	0	0	0	0
Gglean005013	0	0	0	0
Gglean005014	0	0	0	0
Gglean005015	0	0.0196301	0	0

Gglean005016	0	0.071680111	0	0
Gglean005017	27.8414	33.95316136	55.0106992	64.19574
Gglean005018	49.96978	51.37426	41.67814	40.63952
Gglean005019	30.17231	27.14147	29.99886	29.25198
Gglean005020	3.56668	3.70794	2.44861	1.60252
Gglean005021	13.2403	16.9333	9.39699	8.89569
Gglean005022	23.1757	22.9187	14.5019	13.2526
Gglean005023	1.29234	1.21699	0.459981	0.544077
Gglean005024	9.8936	7.1942	5.061018	4.0030875
Gglean005025	0	0	0	0
Gglean005026	0	0.183957	0	0.167166
Gglean005027	0.0945999	0	0	0
Gglean005028	0	0	0	0
Gglean005029	2.57085	2.15484	3.25E-05	1.8897
Gglean005030	0.572079	0.394146	0.202193	1.02E-05
Gglean005031	0	0	0	0
Gglean005032	0	0	0	0
Gglean005033	3.11E-159	5.46E-27	3.14E-10	1.23E-35
Gglean005034	11.4885	15.1809	13.8247	16.9696
Gglean005035	88.26508	103.3300429	63.81197	64.36065
Gglean005036	1.607489	1.990628	2.713992	5.26213
Gglean005037	0.764261	0.142923	0.225498	0.134435
Gglean005038	0	0	0	0
Gglean005039	0	0	0	0
Gglean005040	1.27801	1.926904363	3.3045	2.684952
Gglean005041	1.27801	1.926904363	3.3045	2.684952
Gglean005042	3.616967	3.35623	4.748736	6.66605
Gglean005043	50.14101764	53.7363814	34.66202831	30.59706168
Gglean005044	0.146836	0	0.0397639	0.141018
Gglean005045	0.843002	1.293109	1.995625	1.812339
Gglean005046	22.642306	21.505624	14.97303485	14.51172577
Gglean005047	50.8949	47.57	45.29701	42.494919
Gglean005048	19.62398016	23.00356044	26.09418045	35.1213304
Gglean005049	19.62398016	23.00356044	26.09418045	35.1213304
Gglean005050	0.340581482	0.172923563	0.285234038	0.239991295
Gglean005051	0	0	0	0
Gglean005052	24.61767	28.17617	5.22297	4.68779
Gglean005053	0	0	0	0.112506
Gglean005054	0	0	0	0
Gglean005055	0	0	0	0
Gglean005056	5.92222	5.09073	8.64815	8.86074
Gglean005057	0	0	0	0
Gglean005058	59.68386679	63.180954	44.2593817	46.38806
Gglean005059	0	0	0	0

Gglean005060	100.893	91.5245	77.6538	72.2456
Gglean005061	31.35720376	28.1950025	42.90758	48.8710557
Gglean005062	0	0	0.131952	0.0519587
Gglean005063	0	0	0	0
Gglean005064	5.868444	6.761207	10.955121	10.33993
Gglean005065	725.783749	789.9190365	638.3594833	1329.471592
Gglean005066	16.3008	13.141144	11.576751	10.2209
Gglean005067	23.232342	23.202298	12.59152465	10.55507901
Gglean005068	32.47348	37.28444	32.97746	28.89889
Gglean005069	61.9535	63.33546	43.96863	38.643865
Gglean005070	61.9535	63.33546	43.96863	38.643865
Gglean005071	56.6068	51.282	22.63434	18.88233
Gglean005072	0	0	24.7502	0
Gglean005073	46.68889716	49.253069	67.831438	67.906576
Gglean005074	46.68889716	49.253069	67.831438	67.906576
Gglean005075	0	0.670592	0.0219221	0
Gglean005076	31.642756	38.24432265	30.6625473	27.4514205
Gglean005077	54.056541	46.215811	47.9800112	44.411793
Gglean005078	0	0	0	2.76981
Gglean005079	37.4642	11.951	21.3499	20.1278
Gglean005080	3.818024041	4.764836462	2.520822595	1.728193811
Gglean005081	0	0	0	0
Gglean005082	1.27E-05	7.81E-05	9.89E-06	1.37E-05
Gglean005083	1389.909801	1341.669369	922.6490568	911.3944088
Gglean005084	102.00824	97.80269	83.44792929	93.60136
Gglean005085	102.00824	97.80269	83.44792929	93.60136
Gglean005086	260.89862	256.35719	181.30466	165.64709
Gglean005087	5.854311338	5.998991259	6.861121569	8.53629
Gglean005088	0.799090802	0.7501451	1.454778822	2.151514
Gglean005089	0	0	0	0.258537
Gglean005090	0.915852035	0.514449802	0.842077792	0.785855113
Gglean005091	0	0	0	0
Gglean005092	53.73214605	51.89806112	46.84058327	49.84994885
Gglean005093	30.730656	40.43692	55.9179	65.15409
Gglean005094	30.730656	40.43692	55.9179	65.15409
Gglean005095	45.0968418	47.4505188	65.46188958	78.35249
Gglean005096	0.0333416	0.103896	0	0
Gglean005097	2.81296	1.84463	1.837929	1.830659
Gglean005098	4.645213001	3.928454	5.41408	5.553097691
Gglean005099	0.678337	0.000228835	0.00312781	0.000188151
Gglean005100	39.83488753	42.01828551	27.12031041	24.93437073
Gglean005101	0.145143	0	0.0589476	0
Gglean005102	0	0	0	0
Gglean005103	0	0	0	0

Gglean005104	4.893994162	4.76262704	6.608661165	7.188149427
Gglean005105	6.565270165	7.118990018	9.02016005	9.491830063
Gglean005106	6.565270165	7.118990018	9.02016005	9.491830063
Gglean005107	33.57288	38.7533	30.34991	32.70146
Gglean005108	0	0.863027	0.986934	1.1797
Gglean005109	29.7117	33.36583	16.89900154	19.2314
Gglean005110	5.817380356	6.262248	7.409903	7.9842142
Gglean005111	9.724878242	10.366554	5.16536692	4.310956975
Gglean005112	0	0	0	0
Gglean005113	0.640036	0	0.788446	0
Gglean005114	7.311610001	6.80303922	4.810435467	3.981535188
Gglean005115	11.66968649	13.13403305	13.57222001	14.99556894
Gglean005116	0	0.58206	0.0768034	0.0909967
Gglean005117	0	0	0	0.0543112
Gglean005118	0	0	0	0
Gglean005119	0	0	0	0
Gglean005120	13.29294	11.18891001	13.83370123	22.52812032
Gglean005121	31.10871734	30.0945868	30.78581585	32.90814657
Gglean005122	18.21297236	18.99849907	10.10303754	9.826594
Gglean005123	18.21297236	18.99849907	10.10303754	9.826594
Gglean005124	2.61781891	2.975698869	3.558585	4.371268806
Gglean005125	0	0	0	0
Gglean005126	0.29282	0	0	0
Gglean005127	0.212687	0.240242699	0.110315317	0.414321
Gglean005128	0.19226747	0.627093	0.541372	1.028161
Gglean005129	0	0	0	0.117666
Gglean005130	7.112905791	6.978934054	6.191586218	8.993731784
Gglean005131	27.182014	27.5797508	23.93331241	24.00461669
Gglean005132	27.182014	27.5797508	23.93331241	24.00461669
Gglean005133	76.96394	77.29165	76.374026	85.053727
Gglean005134	0.447858076	0.511490784	0.725579823	1.328545
Gglean005135	0.0456872	0	0.0735297	0
Gglean005136	5.82261	8.78623	2.827737	2.080691
Gglean005137	0	0	0	0
Gglean005138	0	0	0	0.0335333
Gglean005139	1.0146	0.8845	0.583942	1.75995
Gglean005140	0	0.0234771	0	0.045136
Gglean005141	0	0	0	0
Gglean005142	50.5993	53.3781	49.6551	53.3574
Gglean005143	23.9246	21.795	18.2751	15.1544
Gglean005144	23.7729	25.0412	14.8058	13.771
Gglean005145	68.9545	71.11543998	79.70842142	79.390098
Gglean005146	68.9545	71.11543998	79.70842142	79.390098
Gglean005147	994.394	1112.679	665.472	666.924

Gglean005148	18.38006	16.99637	15.07893	14.31783
Gglean005149	74.82565099	72.38953405	46.11264052	43.92061
Gglean005150	74.82565099	72.38953405	46.11264052	43.92061
Gglean005151	1.434689195	1.830503958	2.014364451	3.295163451
Gglean005152	41.3601697	50.026073	39.694151	34.0248162
Gglean005153	4.19142319	4.577429002	5.232054373	9.605271702
Gglean005154	4.19142319	4.577429002	5.232054373	9.605271702
Gglean005155	5.164037093	4.926959245	5.034730746	7.822962685
Gglean005156	5.164037093	4.926959245	5.034730746	7.822962685
Gglean005157	0	0	0	0
Gglean005158	0	0.03352281	0	0
Gglean005159	0	0	0	0
Gglean005160	0	0	0	0.0697215
Gglean005161	0	0	0.22143	0
Gglean005162	0	0	0	0
Gglean005163	0	0	0	0
Gglean005164	0.0415375	0	0.0336476	0
Gglean005165	39.28850237	45.287072	38.314372	39.024551
Gglean005166	0	0	0	0
Gglean005167	0	0	0	0
Gglean005168	0.156194	0.164367	0.128608	0
Gglean005169	0	0	0	0
Gglean005170	0.354433	0	0	0.173943
Gglean005171	0	0	0.20204	0.119805
Gglean005172	39.15617	39.4917	22.59266	21.25722
Gglean005173	0	0	0	0
Gglean005174	13.48355	11.12813	18.33556	19.83235
Gglean005175	2.35514	2.348856	1.628627	1.956189
Gglean005176	0.757003651	0.728998743	0.485844297	0.321750086
Gglean005177	8.34814	8.28771	12.03924	17.94397
Gglean005178	28.61799	27.56424	20.50317	21.14078
Gglean005179	0.0187454	0	0	0
Gglean005180	0	0	0	0
Gglean005181	0	0	0	0
Gglean005182	22.0585	25.15327	47.6178	57.1807
Gglean005183	0	0	0	0
Gglean005184	52.5241403	52.698606	48.247873	53.62161519
Gglean005185	5.0162	3.97336	5.87226	8.41151
Gglean005186	23.4282	25.08303	14.0779	11.78712
Gglean005187	0.111985578	0.1096687	0.0757475	0.1873373
Gglean005188	0.454206027	0.7343835	0.905940487	1.04990283
Gglean005189	8.211734082	15.18014522	10.17483	18.706822
Gglean005190	0	0	0	0.235548
Gglean005191	0	0	0	0

Gglean005192	0	0	0	0
Gglean005193	52.1242	54.1543	62.9195	58.2985
Gglean005194	0.7262256	1.073599428	1.366391	1.011954247
Gglean005195	0.7262256	1.073599428	1.366391	1.011954247
Gglean005196	25.48533339	29.92911863	27.75803972	27.93859337
Gglean005197	25.48533339	29.92911863	27.75803972	27.93859337
Gglean005198	0	0	0	0
Gglean005199	0	0	0	0
Gglean005200	0	0	0	0
Gglean005201	0.0441632	0	0.0354485	0
Gglean005202	1.66448	1.51871	2.09638	1.95671
Gglean005203	0	0	0	0
Gglean005204	0	0	0.0559174	0
Gglean005205	0	0	0.110887	0
Gglean005206	0	0	0	0
Gglean005207	0	0	0	0
Gglean005208	7.71E-08	2.75E-07	2.83E-07	2.69E-08
Gglean005209	0	0	0	0
Gglean005210	0	0	0	0
Gglean005211	0	0	0	0
Gglean005212	0	0.0423615	0	0
Gglean005213	24.18716058	29.27555378	26.52663539	22.43224689
Gglean005214	0	0	0	0
Gglean005215	278.947	204.3466	262.8252	296.3204
Gglean005216	23.14362	23.4831	19.49135	17.47899
Gglean005217	0	0	0.280116	0.666238
Gglean005218	16.0732	17.2977	12.5406	10.9277
Gglean005219	6.9056	7.73714	8.15165	8.95695
Gglean005220	87.98501426	94.003518	101.827185	120.1854562
Gglean005221	0	0	0	0
Gglean005222	32.94188	33.42135	9.50557	8.55453
Gglean005223	13.04131	15.40495	18.63565	17.94972
Gglean005224	31.3304	31.2324	34.8846	31.5395
Gglean005225	0	0	0	0
Gglean005226	0.078820199	0.896764749	0	0.071468595
Gglean005227	0.196013393	0.21982328	0.115321438	0.175826041
Gglean005228	0	0	0.0622697	0
Gglean005229	1.18031	0.613277	0.927884	0.567808
Gglean005230	0	0	0	0
Gglean005231	0	0	0	0
Gglean005232	0	0	0	0
Gglean005233	0	0	0	0
Gglean005234	0	0	0	0
Gglean005235	0	0	0	0.0704549

Gglean005236	0	0	0	0
Gglean005237	0	0.240127	0	0.438129
Gglean005238	0	1.17494	0	0
Gglean005239	6.724024644	5.709122034	7.685707164	10.55652613
Gglean005240	35.52001	31.26694813	41.60686	39.65457
Gglean005241	1561.098011	1616.421212	1023.12929	1021.467393
Gglean005242	0	0	0	0
Gglean005243	0.141836	0.145814	0	0.0681043
Gglean005244	0.164414	0	0.266618	0.158674
Gglean005245	21.4532	22.7109	27.7693	24.38
Gglean005246	13.6564267	11.822531	7.610122174	7.412553075
Gglean005247	13.6564267	11.822531	7.610122174	7.412553075
Gglean005248	12.47855	11.596909	14.27955	14.7359
Gglean005249	61.99844	55.54564431	40.5173575	35.23529244
Gglean005250	0	0	0	0
Gglean005251	0.50656584	0.412046163	0.328169913	0.351923878
Gglean005252	0.50656584	0.412046163	0.328169913	0.351923878
Gglean005253	11.22463	9.80477	7.80111	5.887002316
Gglean005254	0	0	0	0
Gglean005255	0	0	0	0
Gglean005256	7.92627	9.591051	8.031108	6.511051
Gglean005257	1.10653	1.03013	1.30181	0.795237
Gglean005258	0	0	0	0
Gglean005259	0.0551778	0	0	0
Gglean005260	45.5826	43.32418	41.62052	43.41284
Gglean005261	2.234629531	1.725332058	2.980377757	4.479688748
Gglean005262	1.405086084	1.07226737	1.85009089	2.245519587
Gglean005263	6.11809395	6.459782589	4.527442827	4.569469993
Gglean005264	15.5956	14.54008	8.18162	6.43162
Gglean005265	0	0	0	0
Gglean005266	0.114169236	0.250111582	0.16236649	0.16428795
Gglean005267	108.5974	106.1071	98.4329	101.6962
Gglean005268	19.20034	23.23218	18.72152	16.64967
Gglean005269	0	0	0	0
Gglean005270	53.293	52.6613	49.1359	50.9726
Gglean005271	40.20480849	48.5220815	29.845977	32.117886
Gglean005272	40.20480849	48.5220815	29.845977	32.117886
Gglean005273	6.3775	5.77677	5.90018	10.9152
Gglean005274	0	0	0	0
Gglean005275	2.255456	6.038419	3.486716	5.546798176
Gglean005276	2.81189	1.79733	2.34181	4.2721
Gglean005277	0.0975684	0.204197592	0.3725069	0.222982
Gglean005278	2.09738	1.96345	1.34901	2.47391
Gglean005279	3.403660071	4.9535	6.596516868	5.467156001

Gglean005280	3.403660071	4.9535	6.596516868	5.467156001
Gglean005281	111.14822	109.6910018	164.48673	182.148685
Gglean005282	27.303636	27.090571	21.971604	21.582961
Gglean005283	113.553	116.5539	64.3339	50.3049
Gglean005284	67.64326155	68.25080228	42.1120135	32.44304603
Gglean005285	67.64326155	68.25080228	42.1120135	32.44304603
Gglean005286	67.64326155	68.25080228	42.1120135	32.44304603
Gglean005287	0.383770527	0.6253692	0.427125	0.767097
Gglean005288	5.27401	6.62641	13.1639	21.8312
Gglean005289	18.62816	15.968253	16.8525913	17.4673564
Gglean005290	17.01874188	16.85686718	16.67449	18.006073
Gglean005291	0.0418289	0	0.034018	0
Gglean005292	6.72507	5.60892	9.60326	10.3916
Gglean005293	4.41917	5.49043	6.8394	7.814795
Gglean005294	9.132754	8.109269	11.3178	12.56632747
Gglean005295	1.718921794	4.01141275	0.02858436	0.06781819
Gglean005296	69.65817	69.01034	32.08919	30.95077
Gglean005297	15.53563	17.2683946	14.69077737	15.21713329
Gglean005298	15.53563	17.2683946	14.69077737	15.21713329
Gglean005299	13.1303	14.1949	12.652	10.6211
Gglean005300	0	0	0	0
Gglean005301	248.073	225.773	192.3385	165.8109
Gglean005302	0.183367	0.319876	2.00E-05	0.00191423
Gglean005303	136.2485	141.9858	157.6239	228.0998
Gglean005304	0.0456439	0.0155467	0.045552792	0
Gglean005305	0	0	0	0
Gglean005306	0.2469183	0.961958	0.2064715	0.0612847
Gglean005307	1.040717	1.044144	1.093072	0.816509
Gglean005308	5.00621	5.40111	3.07134	2.66792
Gglean005309	35.71793	36.03749	31.78532	31.82096
Gglean005310	0	0	0	0
Gglean005311	0.176601	0.821682	0.538172	2.40221
Gglean005312	21.438	23.562918	19.244255	17.338804
Gglean005313	223.254271	199.1494978	39.12275883	20.18453627
Gglean005314	7.749207061	6.97447	8.40925977	11.21847246
Gglean005315	0.275979	0.0941277	0.0739583	0.0876711
Gglean005316	0.00231974	0.000211283	0.451567	6.99E-05
Gglean005317	19.661355	20.364119	32.039556	26.884628
Gglean005318	12.2959	11.36166	7.6872	7.56076
Gglean005319	12.1671858	17.47513861	0.44105	0.529407
Gglean005320	25.338406	28.364779	20.8837	20.35828
Gglean005321	32.45431	35.33420131	18.28574744	40.05744
Gglean005322	0	0	0	0
Gglean005323	14.76233	16.66212	11.67172675	9.9262

Gglean005324	0	0	0	0
Gglean005325	0	0	0	0
Gglean005326	0	0	0.0222112	0
Gglean005327	0	0	0	0
Gglean005328	0	0	0	0
Gglean005329	0	0	0	0
Gglean005330	0.230554391	0.0966029	0.062172521	0.221993417
Gglean005331	0	0	0.0572589	0
Gglean005332	17.569488	16.92410954	15.99281367	15.38043176
Gglean005333	0.619209	0.711194	0.984804	0.93591
Gglean005334	1.39248	0.77446	0	0
Gglean005335	58.9826	60.53410001	59.12618	56.99916
Gglean005336	0	0.490665	0	0
Gglean005337	0	0	0	0
Gglean005338	0	0	0	0
Gglean005339	0	0	0	0
Gglean005340	0	0	0	0
Gglean005341	0	0	0	0
Gglean005342	0	0	0	0
Gglean005343	0	0	0	0
Gglean005344	0.109785	0.116326	0	0
Gglean005345	5.30526	12.2282	4.35343	0
Gglean005346	5.13778	6.16053	3.51617	2.17008
Gglean005347	0	0	0	0
Gglean005348	0	0.168166	0	0
Gglean005349	0	0	0	0
Gglean005350	0	0	0	0
Gglean005351	0	0	0	0
Gglean005352	0	0	0	0
Gglean005353	0	0	0	0
Gglean005354	0	0	0	0
Gglean005355	0	0	0	0
Gglean005356	0	0	0	0
Gglean005357	0	0	0	0
Gglean005358	0	0	0	0
Gglean005359	0	0	0	0
Gglean005360	0	0	0	0
Gglean005361	0	0	0	0
Gglean005362	0	0	0	0
Gglean005363	0	0	0	0
Gglean005364	0	0.511215	0	0.466763
Gglean005365	0	0	0	0
Gglean005366	0.113137	0	0	0
Gglean005367	0	0	0	0

Gglean005368	0	0	0	0
Gglean005369	3.013613	4.71987	2.872874	3.734956
Gglean005370	4.529070767	4.397290688	5.685843447	7.056780723
Gglean005371	0	0.023220105	0	0
Gglean005372	0	0	0	0
Gglean005373	0	0	0	0
Gglean005374	0	0	0	0
Gglean005375	0	0	0	0
Gglean005376	0	0	0	0.157306
Gglean005377	0	0.319535	0	0.149888
Gglean005378	0	0	0	0
Gglean005379	0	0	0	0
Gglean005380	0	0	0	0
Gglean005381	0.217192	0	0	0.103816
Gglean005382	0	0	0	0
Gglean005383	0.19797	0.102906	0.159509	0
Gglean005384	22.369016	22.47649903	18.805294	19.35638449
Gglean005385	0	0	0	0
Gglean005386	0	0	0	0
Gglean005387	13.20126515	11.46846136	7.741111976	7.912307663
Gglean005388	13.20126515	11.46846136	7.741111976	7.912307663
Gglean005389	148.5908	153.66285	207.82722	256.364611
Gglean005390	148.5908	153.66285	207.82722	256.364611
Gglean005391	5.22584	5.12722	4.6886	4.44409
Gglean005392	0.143566	1.49687	2.00314	2.24506
Gglean005393	0	0	3.49313	6.18017
Gglean005394	9.595632	8.70271	6.33833532	5.31529
Gglean005395	0	0	0	0
Gglean005396	28.30498106	22.00045383	40.51199412	37.24136897
Gglean005397	0	0	0	0
Gglean005398	0	0	0	0
Gglean005399	0	0	0	0
Gglean005400	0	0	0	0
Gglean005401	32.1972	36.4392	24.8156	24.6756
Gglean005402	0.096916164	0.056295191	0.132820291	0.134714949
Gglean005403	59.41494367	63.63622	49.18182	48.52143
Gglean005404	32.4695	25.6749	13.444	23.379
Gglean005405	84.96471	84.7673	73.45869	71.42684
Gglean005406	117.5328774	134.339027	153.1541479	141.5534733
Gglean005407	292.879	249.009	85.1752	69.2075
Gglean005408	0.102254	0.212085	0	0
Gglean005409	15.0061	12.5774	8.68062	6.49649
Gglean005410	0.390979455	0.75764	0.6055787	0.635020648
Gglean005411	0	0	0	0

Gglean005412	0	0	0	0
Gglean005413	0.608321	0.000380207	0.0212779	3.71E-13
Gglean005414	50.32760353	53.07230491	47.90180391	44.62310395
Gglean005415	38.20965222	36.02444755	51.13330213	64.26163797
Gglean005416	0.537851462	0.771629	0.996115282	1.840135
Gglean005417	4.736387765	4.703944005	5.803719106	5.053747242
Gglean005418	9.101749528	8.85407816	10.2109589	13.32756174
Gglean005419	0	0	0	0
Gglean005420	0	0	0	0
Gglean005421	0	0	0	0
Gglean005422	7.61493	3.72605	6.86137	5.06382
Gglean005423	26.6166	25.4134	13.8042	10.0056
Gglean005424	0	0	5.88E-06	0
Gglean005425	0	0	0	0
Gglean005426	0.180747	0.0623757	0.0977743	0.115707
Gglean005427	19.673209	20.50596822	11.960257	11.060038
Gglean005428	20.1643707	19.588399	24.67626116	32.387866
Gglean005429	0	0	0.173419	0
Gglean005430	1.57753	2.00628	1.46231	0.760799
Gglean005431	0	0	0	0
Gglean005432	0	0	0	0
Gglean005433	9.208618672	9.13993	10.65594006	20.05291
Gglean005434	0	0	0	0
Gglean005435	1.424268	1.02894045	1.693587	1.625637463
Gglean005436	37.22141	35.15519824	36.839005	33.833537
Gglean005437	17.41791	15.65326	15.35843779	18.69158762
Gglean005438	231.25033	273.8238	184.7002	161.00079
Gglean005439	231.25033	273.8238	184.7002	161.00079
Gglean005440	0	0	0	0
Gglean005441	1.258796136	1.609992	2.410491	1.683018
Gglean005442	18.59787	19.4340295	14.62592	12.65938
Gglean005443	23.7362027	26.13093238	20.26920299	17.61764347
Gglean005444	23.7362027	26.13093238	20.26920299	17.61764347
Gglean005445	23.7362027	26.13093238	20.26920299	17.61764347
Gglean005446	46.4904776	47.8039756	43.777275	40.2478481
Gglean005447	32.9983	36.20499	30.07176	25.28332
Gglean005448	10.1377	9.69939	0.000277136	2.38565
Gglean005449	22.27189043	21.71344144	13.25638825	11.9503202
Gglean005450	5.2737958	5.957194914	7.837390032	8.669500237
Gglean005451	0.757029397	0.993589	0.591214	0.376338059
Gglean005452	106.8549	113.8923	135.2489	113.1399
Gglean005453	101.439	123.91	67.2473	63.4568
Gglean005454	7.819156	6.604235274	3.843590631	3.572080005
Gglean005455	0	0	0	0

Gglean005456	0	0	0	0
Gglean005457	0	0.0424674	0	0
Gglean005458	0	0	0	0
Gglean005459	0	0	0	0.0645587
Gglean005460	0	0	0	0
Gglean005461	0.620816	1.08549	0.807138	0.990007
Gglean005462	0.354069	0	0	0.351119
Gglean005463	0	0	0	0
Gglean005464	0.109394	0	0	0
Gglean005465	0.103773	0.433849	0.169529	0.100653
Gglean005466	0.193958	0.0661912	0.103731	0.061533
Gglean005467	0	0	0	0
Gglean005468	18.1108	21.7256	6.37735	6.24721
Gglean005469	0	0	0	0
Gglean005470	47.03444	52.85453	32.10924	24.16305
Gglean005471	47.03444	52.85453	32.10924	24.16305
Gglean005472	0	0	0	0
Gglean005473	0	0	0	0
Gglean005474	0	0	0	0
Gglean005475	8.371288166	6.601178	5.880131038	4.9375958
Gglean005476	8.371288166	6.601178	5.880131038	4.9375958
Gglean005477	0	0	0	0
Gglean005478	0	0	0	0
Gglean005479	0.436685	0	0	0
Gglean005480	0	0	0	0
Gglean005481	0	0	0.0537388	0.128216
Gglean005482	0.525339	0	0	0
Gglean005483	52.1124	58.9216	65.2259	61.0115
Gglean005484	1.38687	1.57748	0	1.35037
Gglean005485	0	0	0	0
Gglean005486	0	0	0.629054	0
Gglean005487	0.138824	0.0479584	0.113351	0.223534
Gglean005488	15.240868	17.12599	32.8111987	36.45292014
Gglean005489	0.000360835	0.951607	1.32E-07	4.87E-05
Gglean005490	15.240868	17.12599	32.8111987	36.45292014
Gglean005491	22.0067	23.512521	11.66257	9.489313544
Gglean005492	5.72999	7.32577	2.63633	3.70699
Gglean005493	116.7302	118.7269	89.8954	75.2655
Gglean005494	48.8057	60.5862	61.7844	71.3199
Gglean005495	4.86815	3.95124	5.24669	5.8285
Gglean005496	14.8322	14.1553	8.94712	8.88987
Gglean005497	0.592776	0.600070789	0.195550534	0.549555051
Gglean005498	13.77971	11.71172	7.53687	7.12002
Gglean005499	408.599035	537.639582	269.849016	214.86348

Gglean005500	408.599035	537.639582	269.849016	214.86348
Gglean005501	0.684440554	0.43716645	0.349629056	0.40291075
Gglean005502	14.78129006	19.1659	6.1219836	7.352110691
Gglean005503	23.5182	23.4867	13.3894	13.8869
Gglean005504	37.869934	33.665831	42.39641	43.85745
Gglean005505	10.1214	7.47363	6.03646	4.9556
Gglean005506	7.26079	6.63171	5.14556	4.2597
Gglean005507	48.28955567	47.793546	42.77969153	41.714266
Gglean005508	48.28955567	47.793546	42.77969153	41.714266
Gglean005509	86.9968	63.88351	27.39489	14.89701
Gglean005510	0	0	0	0
Gglean005511	54.99928463	58.25923642	18.90856878	16.470834
Gglean005512	54.99928463	58.25923642	18.90856878	16.470834
Gglean005513	2.25E-06	0.614928	0.23506	2.88E-07
Gglean005514	58.24977106	60.11682787	48.95182283	43.06244667
Gglean005515	58.24977106	60.11682787	48.95182283	43.06244667
Gglean005516	34.7073	33.0237	19.04108	17.24563
Gglean005517	873.927	912.153	569.321	503.189
Gglean005518	176.2019328	224.7605661	173.71441	211.55593
Gglean005519	176.2019328	224.7605661	173.71441	211.55593
Gglean005520	0	0	0	0
Gglean005521	20.546422	25.061065	22.42279	20.12584068
Gglean005522	20.546422	25.061065	22.42279	20.12584068
Gglean005523	0	0	0	0
Gglean005524	27.36164256	29.477138	40.89833988	45.65405818
Gglean005525	0	0	0	0
Gglean005526	0	0	0	0
Gglean005527	0	0	0	0.059155359
Gglean005528	0	0.0491967	0	0
Gglean005529	12.98895843	14.33700254	16.17380245	13.81863983
Gglean005530	13.8215674	13.22332	19.132	23.01279
Gglean005531	0	0	0	0
Gglean005532	20.16414	19.79835	20.49114	19.31194
Gglean005533	54.48331	50.58277	47.571913	45.625625
Gglean005534	12.084115	13.06981725	13.091085	13.468981
Gglean005535	34.6997	35.657363	38.567511	45.86603
Gglean005536	31.18299	38.75637	53.9632512	60.78235
Gglean005537	31.18299	38.75637	53.9632512	60.78235
Gglean005538	60.90819002	59.55828182	53.864767	73.810569
Gglean005539	207.903	107.049	132.505	217.724
Gglean005540	43.8883	46.73378	38.03993	42.6923
Gglean005541	19.6397	20.3742	19.0606	17.4288
Gglean005542	36.7574	25.7726	19.3042	21.7923
Gglean005543	0	0	0	0

Gglean005544	1.278812	5.751311	0.520056	0.280369513
Gglean005545	21.11302731	20.26731596	14.71334511	13.15291688
Gglean005546	24.69417	24.59655	22.23543	22.63621
Gglean005547	22.09079	24.53617	26.68927	28.10353
Gglean005548	1181.1394	1174.4481	779.6232	739.7157
Gglean005549	8.18408	9.84571	17.10262	14.05763
Gglean005550	21.93803	23.88003	25.2998	24.5166
Gglean005551	29.41093	64.2123	0	0.140865764
Gglean005552	0	0.21634	0.135283	0.16016
Gglean005553	0.085045	0.0291824	0	0
Gglean005554	0.532573	0.628013	0.737091	1.11299
Gglean005555	0	0.0469976	0	0
Gglean005556	8.308686	6.560634	4.769450918	4.459954668
Gglean005557	1.91667	1.20761	0.13212	3.46E-06
Gglean005558	0	0	0	0
Gglean005559	38.60621	14.53388	24.747578	27.88594
Gglean005560	38.60621	14.53388	24.747578	27.88594
Gglean005561	66.75072951	69.68867278	64.82843842	62.56941539
Gglean005562	4.5409	2.38556	1.50829	3.83428
Gglean005563	44.145994	42.1217	32.186351	31.88685
Gglean005564	44.145994	42.1217	32.186351	31.88685
Gglean005565	44.145994	42.1217	32.186351	31.88685
Gglean005566	44.145994	42.1217	32.186351	31.88685
Gglean005567	11.998	13.2608	8.91814	7.37631
Gglean005568	0.596376	1.059944	0.367563	0.876398
Gglean005569	6.913033228	7.639497072	3.152901501	5.209201581
Gglean005570	6.913033228	7.639497072	3.152901501	5.209201581
Gglean005571	17.2561238	21.21646475	30.24149461	37.55436669
Gglean005572	11.22590296	11.071923	19.41859018	21.911105
Gglean005573	43.56377105	55.69505447	38.048748	36.477106
Gglean005574	2.709693	3.81651	2.032731	3.43125
Gglean005575	65.345534	64.588171	73.95872199	73.24237971
Gglean005576	18.3333	15.6614	13.1031	11.4043
Gglean005577	65.345534	64.588171	73.95872199	73.24237971
Gglean005578	65.345534	64.588171	73.95872199	73.24237971
Gglean005579	0.00323525	4.61E-05	6.63E-05	0.7954618
Gglean005580	0.00323525	4.61E-05	6.63E-05	0.7954618
Gglean005581	0	0	0	0.384902
Gglean005582	1.522648001	1.05930572	0.8513348	0.592402666
Gglean005583	1.522648001	1.05930572	0.8513348	0.592402666
Gglean005584	1.522648001	1.05930572	0.8513348	0.592402666
Gglean005585	5.556992118	5.502537871	2.954048	2.957781374
Gglean005586	27.80736205	24.06585564	40.46966209	36.79395801
Gglean005587	6.718821381	6.016872599	7.738233799	8.190751

Gglean005588	36.8951	34.3006	14.46613	12.99735
Gglean005589	3.499876	2.596503422	2.494242607	2.369379355
Gglean005590	3.499876	2.596503422	2.494242607	2.369379355
Gglean005591	0.709578447	0.87099096	0.5352792	0.499291348
Gglean005592	0.709578447	0.87099096	0.5352792	0.499291348
Gglean005593	53.00965	53.74729	55.335296	57.94178
Gglean005594	15.47231	14.44119639	19.774166	21.30598
Gglean005595	24.00209	24.57014	10.939872	10.55457
Gglean005596	57.22130668	58.77453911	94.65621348	112.095035
Gglean005597	32.25261664	34.15621684	38.613885	44.69757728
Gglean005598	0.0459501	0	0	0
Gglean005599	10.60430434	8.206448	10.30480565	11.4264023
Gglean005600	0	0	0	0
Gglean005601	0	0	0	0
Gglean005602	1.716733	1.079111	1.002242	1.545099
Gglean005603	0.0683327	0	0.166414	0.0662956
Gglean005604	0.84605	0.439533	0.206357	0.325334
Gglean005605	0	0	0	0.111618
Gglean005606	0	0	0	0
Gglean005607	0	0	0	0
Gglean005608	10.50409	11.96067	13.19729	12.14347
Gglean005609	20.66493234	20.50903102	17.10869823	16.0688554
Gglean005610	0	0	0	0
Gglean005611	33.1931311	30.88052	32.07111667	24.41266268
Gglean005612	33.1931311	30.88052	32.07111667	24.41266268
Gglean005613	63.35864123	63.591575	62.80916288	62.86169014
Gglean005614	63.35864123	63.591575	62.80916288	62.86169014
Gglean005615	14.07980792	14.59491886	16.889677	17.723179
Gglean005616	5.403374986	6.632656711	6.2337357	7.362507364
Gglean005617	2.321379	2.482601451	2.841814081	3.772378
Gglean005618	0.23502	0	1.54697	0.231318
Gglean005619	8.605766	4.372139	5.070980043	8.379295
Gglean005620	0	0	0	0
Gglean005621	35.407972	35.91293605	38.49662	39.045824
Gglean005622	24.39940705	24.60142665	22.67074613	22.04477348
Gglean005623	9.89025	10.6089	8.22225	8.17493
Gglean005624	14.568846	10.634547	22.229875	35.71731482
Gglean005625	0.422412	0	0.70629	0.845587
Gglean005626	1.56875	0	0.435938	0
Gglean005627	33.0523	38.2573	9.9553	10.1502
Gglean005628	11.98645	11.41166	9.68127	7.983866
Gglean005629	0.0951345	0	0.154706	0
Gglean005630	0	0	0	0
Gglean005631	0	0	0	0.099249787

Gglean005632	46.58006132	47.937197	57.950243	72.08065329
Gglean005633	30.72237855	32.12013	13.98716	12.497876
Gglean005634	96.01094803	96.57159091	64.09732696	69.09821276
Gglean005635	4.868311	5.90626	0.365255	0.1297657
Gglean005636	0	0	0	0
Gglean005637	0	0.101035709	0	0
Gglean005638	0	0	0	0
Gglean005639	0.0422232	0	0	0
Gglean005640	0.035599334	0.136069273	0.114122	0.15029465
Gglean005641	0	0	0	0.125058
Gglean005642	0.106996	0.335098	1.98253	0.103141
Gglean005643	134.513	135.247	77.4739	76.2909
Gglean005644	0.411285542	0	0.0939382	0.882175094
Gglean005645	30.11987	28.42915	27.428739	30.59879
Gglean005646	3.125995649	3.08256317	4.95530738	5.024236654
Gglean005647	98.55433	34.5249	147.65728	113.07116
Gglean005648	98.55433	34.5249	147.65728	113.07116
Gglean005649	25.942445	26.277681	26.75592	26.98325
Gglean005650	1.472007	0.964898	1.643411296	1.834735
Gglean005651	1274.74798	1329.885814	850.8731241	815.7361613
Gglean005652	1.11002	0.402629	0	0
Gglean005653	13.13733	12.086672	13.93281	24.11047
Gglean005654	0	0	0	0
Gglean005655	0	0	0	0
Gglean005656	4.209285	6.414884635	4.130263731	5.1038505
Gglean005657	0	0.150515	0	0.141154
Gglean005658	44.4345	46.9768	89.4078	105.1094
Gglean005659	0.000249186	0.000738913	0.000732813	5.62E-05
Gglean005660	4.84524	4.31261	2.96692	3.3008
Gglean005661	3.96481	0	0	7.55474
Gglean005662	86.06360003	92.46950007	121.7692	122.4275382
Gglean005663	1.361817	0.7048901	0.724568	0.6107
Gglean005664	22.11832984	21.8482632	10.39070602	7.72999695
Gglean005665	3.875289357	3.777719668	2.18564919	2.508910403
Gglean005666	11.912387	10.134687	7.691754	7.267914
Gglean005667	559.9293	657.55143	392.09562	303.21169
Gglean005668	23.67683	25.178761	19.185473	18.882875
Gglean005669	1.92043	0	6.37274	9.39878
Gglean005670	17.13449273	17.94612483	27.44065433	27.08849344
Gglean005671	0	0	0	0
Gglean005672	17.13449273	17.94612483	27.44065433	27.08849344
Gglean005673	9.604940381	15.38951227	14.25431069	20.903923
Gglean005674	60.576579	65.4343471	34.00591328	31.8008797
Gglean005675	60.576579	65.4343471	34.00591328	31.8008797

Gglean005676	60.24462	51.730548	21.46667384	22.95136
Gglean005677	60.24462	51.730548	21.46667384	22.95136
Gglean005678	35.68545	38.41811853	26.03748662	24.05363217
Gglean005679	150.52469	116.3825921	126.0268903	116.6481805
Gglean005680	150.52469	116.3825921	126.0268903	116.6481805
Gglean005681	40.355419	41.55268	43.385682	42.2458607
Gglean005682	40.355419	41.55268	43.385682	42.2458607
Gglean005683	17.8886	24.86719	29.40415	25.75295
Gglean005684	17.8886	24.86719	29.40415	25.75295
Gglean005685	148.1877841	166.5337058	103.2575127	98.43314368
Gglean005686	148.1877841	166.5337058	103.2575127	98.43314368
Gglean005687	17.71967846	17.46703821	30.8849585	30.40035055
Gglean005688	0	0	0	0
Gglean005689	0	0	0	0
Gglean005690	0	0	0	0
Gglean005691	0	0.0400509	0	0
Gglean005692	0	0	0	0
Gglean005693	0	0	0	0
Gglean005694	0	0.188828	0.0245095	0
Gglean005695	0	0	0	0
Gglean005696	0	0	0	0
Gglean005697	0	0	0	0
Gglean005698	0	0	0	0
Gglean005699	0	0	0	0
Gglean005700	0	0	0	0.205008
Gglean005701	0.0841205	0	0.0683106	0
Gglean005702	0	0	0	0
Gglean005703	0	0	0	0
Gglean005704	0.6237652	0.2985187	0.4402503	0.4645149
Gglean005705	0.6237652	0.2985187	0.4402503	0.4645149
Gglean005706	0.531494	1.03803	0.236319	0.329154
Gglean005707	0	0	0	0
Gglean005708	0	0	0	0
Gglean005709	0	0	0	0
Gglean005710	0	0	0	0
Gglean005711	0	0	0	0
Gglean005712	0.415531793	0.188160685	0.541305358	1.1329118
Gglean005713	3.28216	4.10942	6.39001	5.91446
Gglean005714	0	0	0	0
Gglean005715	0	0	0	0
Gglean005716	0	0	0	0
Gglean005717	0.416327	0.106482	0.223818	0.232321
Gglean005718	0	0	0	0
Gglean005719	0	0	0	0

Gglean005720	0	0	0	0
Gglean005721	0	0	0	0
Gglean005722	0.101279	0	0	0
Gglean005723	0.109765	0.0563407	0.0668471	0.0528787
Gglean005724	9.693352	10.874983	9.604057	8.426196
Gglean005725	41.41463	40.8237553	15.672219	11.92413189
Gglean005726	0.231358036	0.284998509	0	0.0442537
Gglean005727	0	0	0	0
Gglean005728	0	0	0	0
Gglean005729	46.781909	48.64553	59.04709	73.8888
Gglean005730	47.61606351	62.30802958	46.00882273	48.04872547
Gglean005731	17.424593	20.385588	9.37942	7.95742
Gglean005732	50.24200707	48.43420985	44.31021271	42.19667448
Gglean005733	1.13912	0.909959	1.11698	2.00537
Gglean005734	10.18948	10.91556	7.24946	7.73625
Gglean005735	0	0	0.276998	0.229996
Gglean005736	0	0	0	0
Gglean005737	0.294334	0	0.547676983	0
Gglean005738	9.031732407	10.24050337	12.5308484	13.8755917
Gglean005739	23.49455543	23.91867183	21.88067653	23.26511739
Gglean005740	1.21962	0.727038	1.92733	1.7766
Gglean005741	16.944858	16.36655365	18.27441526	14.61980817
Gglean005742	0	0	0.163335	0.194437
Gglean005743	0	0	0	0
Gglean005744	57.5046001	64.10304746	78.48865106	71.32186408
Gglean005745	0	0	0	0
Gglean005746	1.58575151	1.851780288	4.076035494	3.642995852
Gglean005747	1.58575151	1.851780288	4.076035494	3.642995852
Gglean005748	20.62572614	20.3985	13.963671	11.272065
Gglean005749	0	0	0	0
Gglean005750	4.55166	5.147523	5.985803	2.444286
Gglean005751	6.036382418	5.282240003	8.431112684	9.418819089
Gglean005752	89.751964	104.2880069	75.293533	84.7289066
Gglean005753	110.815122	105.135322	104.413644	112.564116
Gglean005754	110.815122	105.135322	104.413644	112.564116
Gglean005755	20.61457089	21.41182	25.78329	27.99381
Gglean005756	20.61457089	21.41182	25.78329	27.99381
Gglean005757	54.35355	46.18811	38.620942	42.1274
Gglean005758	59.21415238	64.33380427	68.07886157	60.1243016
Gglean005759	266.3564056	300.85186	128.573706	116.9003
Gglean005760	48.36977	55.63039	46.87226	44.1841
Gglean005761	153.5355	179.658	61.3897	51.20510109
Gglean005762	26.2232	32.196934	44.27971957	55.6247
Gglean005763	18.3062	16.4881	10.1338	7.87431

Gglean005764	15.4462	17.77314976	9.83688501	11.2367758
Gglean005765	58.26025	59.8609	44.75864	39.97234
Gglean005766	58.26025	59.8609	44.75864	39.97234
Gglean005767	0.148689	0.15613	0.121389	0
Gglean005768	44.731001	47.09394	38.77578	35.13004
Gglean005769	2.26232	3.459264	4.199515	5.54556
Gglean005770	8.75926	10.0953	10.6582	13.8263
Gglean005771	19.548915	19.87970004	19.2125	16.683115
Gglean005772	19.548915	19.87970004	19.2125	16.683115
Gglean005773	32.751946	33.282743	45.66005	52.052911
Gglean005774	0	0	0	0
Gglean005775	2.26232	3.459264	4.199515	5.54556
Gglean005776	47.605156	47.6057	42.77315	41.79296
Gglean005777	0.877872916	0.909454	2.528637	1.509155925
Gglean005778	21.21571157	20.927052	24.41209	23.6505583
Gglean005779	0	0	0.47037	0
Gglean005780	0.625947	0.657222	0	0.322279
Gglean005781	56.94989668	55.22549256	47.0810573	47.19427821
Gglean005782	56.94989668	55.22549256	47.0810573	47.19427821
Gglean005783	14.1139	19.65858	23.3952	18.99641
Gglean005784	36.1481	35.6903	35.4633	36.3625
Gglean005785	52.1725	57.8257	28.7274	27.024
Gglean005786	1.724604	1.679233626	2.5130949	2.370916719
Gglean005787	257.6891002	251.16514	267.234223	335.302665
Gglean005788	257.6891002	251.16514	267.234223	335.302665
Gglean005789	257.6891002	251.16514	267.234223	335.302665
Gglean005790	3.28966	2.42807	1.87707	2.40506
Gglean005791	3.93553472	2.426474102	2.405429	7.95093
Gglean005792	51.0714	56.5856	67.8452	87.2381
Gglean005793	53.1912	67.8458	47.8262	38.8317
Gglean005794	10.830912	11.815693	8.0776678	6.97001055
Gglean005795	32.2511	33.27276	32.46698	33.06683
Gglean005796	76.6775	79.8341	54.9966	51.7524
Gglean005797	12.461009	12.239397	10.66205903	8.52445
Gglean005798	1.701269	1.601419	1.228185	1.0401744
Gglean005799	38.5646	33.1631	29.1523	25.4459
Gglean005800	28.778511	30.951658	18.882117	18.506966
Gglean005801	42.05441	42.64796	45.9519	48.43712
Gglean005802	7.45803	5.3761	7.14141	8.28622
Gglean005803	23.3237	24.4252	12.4742	9.25137
Gglean005804	134.0363	133.287	76.6535	71.2232
Gglean005805	33.98321	41.36163	45.39737	36.41911205
Gglean005806	33.98321	41.36163	45.39737	36.41911205
Gglean005807	33.98321	41.36163	45.39737	36.41911205

Gglean005808	12.620367	12.448602	12.439886	10.339807
Gglean005809	147.2599708	164.0615005	172.997297	148.358276
Gglean005810	28.778511	30.951658	18.882117	18.506966
Gglean005811	0	0	0.0600642	0
Gglean005812	56.70338	41.62296	90.33153016	91.70134
Gglean005813	7.82986	6.42164	4.45466	3.86103
Gglean005814	11.650973	11.50431	17.94444505	22.311497
Gglean005815	3.603765	3.49194	6.67921	6.258059
Gglean005816	36.822273	29.465844	23.19598	22.79241
Gglean005817	30.63695	31.02936	18.12797	15.92345
Gglean005818	30.72731	30.168558	49.6208	52.19109
Gglean005819	21.318701	20.8722	21.130849	22.5207
Gglean005820	0.331846	0	1.599453	0.970631
Gglean005821	15.017887	12.41957154	18.02602	19.189271
Gglean005822	15.017887	12.41957154	18.02602	19.189271
Gglean005823	0	0	0	0
Gglean005824	18.76505431	20.8570637	12.90827042	11.5254412
Gglean005825	34.252573	28.816047	36.68221	28.73521332
Gglean005826	0.11366	0.466457	0.571855	0.2198
Gglean005827	34.252573	28.816047	36.68221	28.73521332
Gglean005828	35.678212	39.93851747	24.33178019	18.92027534
Gglean005829	0	0.24305	0	0
Gglean005830	0	0.24305	0	0
Gglean005831	0.240975	0.0495479	0.0389049	0
Gglean005832	3.39879278	3.295489148	1.496230279	1.975626931
Gglean005833	3.39879278	3.295489148	1.496230279	1.975626931
Gglean005834	0.922451079	0.602974	0	0.39676923
Gglean005835	24.273709	20.008403	32.97159241	38.45268012
Gglean005836	0.6286	0.793287	0.709018359	1.040652782
Gglean005837	55.08084	54.50069	23.6179	21.45328
Gglean005838	44.67987	44.93701	50.2188	59.09129
Gglean005839	0	0	0	0
Gglean005840	0.74408	0.588626	1.25313	0.772405
Gglean005841	21.4967	20.0798	14.4972	12.4166
Gglean005842	30.9045	26.4386	25.6609	28.122
Gglean005843	26.1185	29.3277	30.7285	32.9774
Gglean005844	28.22512453	27.10366814	22.381868	21.538855
Gglean005845	34.38484197	36.44227858	44.27590001	44.25344536
Gglean005846	34.38484197	36.44227858	44.27590001	44.25344536
Gglean005847	0	0	0	0.336321
Gglean005848	0	0	0	0
Gglean005849	169.049204	153.044298	127.190576	126.030604
Gglean005850	24.72184109	26.24652248	16.06726409	13.71931579
Gglean005851	68.08221169	70.54572395	93.66420689	115.218469

Gglean005852	68.08221169	70.54572395	93.66420689	115.218469
Gglean005853	68.08221169	70.54572395	93.66420689	115.218469
Gglean005854	0.280151	0.594685	0	1.38429
Gglean005855	5.64853	6.32013	5.85215	7.1673
Gglean005856	34.99392719	37.33517024	42.57832443	41.13035568
Gglean005857	0	0	0	0
Gglean005858	0	0	0	0
Gglean005859	5.784879995	5.369535691	8.163142295	9.624801926
Gglean005860	24.273709	20.008403	32.97159241	38.45268012
Gglean005861	0	0	0	0
Gglean005862	0	0	0	0
Gglean005863	3.00E-10	3.15E-06	0.000187881	4.34E-06
Gglean005864	161.638	156.595	177.097	200.898
Gglean005865	31.43037	24.64658	24.57717	22.34236
Gglean005866	31.43037	24.64658	24.57717	22.34236
Gglean005867	38.3515	39.6111	32.6541	35.7846
Gglean005868	7.5965	7.69011	3.25051	3.53169
Gglean005869	0	0	0	0
Gglean005870	0	0	0	0
Gglean005871	0	0	0	0.138185
Gglean005872	0.592334	1.36183	0.769983	1.14919
Gglean005873	0.723903	1.40973	1.4131	1.53321
Gglean005874	34.252573	32.7863	36.68221	28.73521332
Gglean005875	53.76167	58.13883	73.01111	81.6394
Gglean005876	14.335532	16.376183	12.063019	12.058
Gglean005877	30.01667	29.83923	32.80611	31.10357213
Gglean005878	12.5954	14.1975	11.0138	10.6475
Gglean005879	9.54E-156	0.509552	0.538723	0.206509
Gglean005880	18.07041	19.23741	4.446527	4.852457
Gglean005881	25.00663	34.390032	22.518854	17.487522
Gglean005882	25.00663	34.390032	22.518854	17.487522
Gglean005883	0	0	0	0.10886
Gglean005884	0	0.0413249	0	0.0381047
Gglean005885	34.252573	28.816047	36.68221	38.3416
Gglean005886	35.70098	33.95283	38.35216	34.44945
Gglean005887	22.7278	32.35381	15.2674	16.3978
Gglean005888	56.98787	61.90187	46.74958	48.87665
Gglean005889	56.98787	61.90187	46.74958	48.87665
Gglean005890	56.98787	61.90187	46.74958	48.87665
Gglean005891	2.71232	2.55274	4.40749	4.19777
Gglean005892	183.983	192.31	63.2001	59.1794
Gglean005893	819.5947	889.7756	355.88398	270.97732
Gglean005894	39.8894	41.7952	53.4705	49.0691
Gglean005895	28.1649	28.9617	37.2812	44.0051

Gglean005896	14.84781	15.19718	10.0627	8.41275
Gglean005897	74.8434	79.7958	73.9724	71.0626
Gglean005898	33.2596	46.7203	72.4229	78.931
Gglean005899	3.720385	4.466637	3.670532502	5.027963165
Gglean005900	3.720385	4.466637	3.670532502	5.027963165
Gglean005901	3.871983	4.1934751	7.3194471	7.329663
Gglean005902	3.871983	4.1934751	7.3194471	7.329663
Gglean005903	8.04177	7.321110762	11.04526	15.239929
Gglean005904	8.04177	7.321110762	11.04526	15.239929
Gglean005905	57.78481488	59.79709254	63.2512132	61.85298276
Gglean005906	32.736315	33.50052594	35.04642543	32.078766
Gglean005907	0.132492	0.137208	0	0.128204
Gglean005908	1.96E-09	0.000263	7.73E-06	2.54E-07
Gglean005909	72.87177146	72.17734116	71.05084065	78.46247065
Gglean005910	72.87177146	72.17734116	71.05084065	78.46247065
Gglean005911	17.9193309	19.289814	29.142421	31.511318
Gglean005912	3.23523	3.37898	4.72105	5.58049
Gglean005913	3.23523	3.37898	4.72105	5.58049
Gglean005914	6.63712902	7.32042247	10.77942897	12.01288946
Gglean005915	23.853758	26.5407	9.690409	11.484708
Gglean005916	8.03296	9.818376	3.93149	3.0158
Gglean005917	8.03296	9.818376	3.93149	3.0158
Gglean005918	5.73357	5.326729124	5.032900994	5.123151015
Gglean005919	0	0	0	0
Gglean005920	105.969	103.493	77.795	70.3066
Gglean005921	18.18119005	19.35176718	20.391637	21.33492269
Gglean005922	22.0337	21.3593	19.0793	18.9412
Gglean005923	149.05882	150.75363	127.22435	138.40226
Gglean005924	48.4290152	52.2948865	29.1342713	24.71750755
Gglean005925	48.4290152	52.2948865	29.1342713	24.71750755
Gglean005926	48.4290152	52.2948865	29.1342713	24.71750755
Gglean005927	30.25213572	36.23155837	49.174595	58.963117
Gglean005928	27.31371	30.64418	28.68805	28.7036185
Gglean005929	1.95233	1.97397	3.015519	2.41188
Gglean005930	10.36287971	12.09534365	13.59578162	13.882319
Gglean005931	22.902612	25.35368	10.020057	7.814426
Gglean005932	0.928896	0.521122	0.250679	0.904374
Gglean005933	62.12018606	65.40206434	55.092986	53.107037
Gglean005934	28.331124	28.155124	24.170568	21.213683
Gglean005935	3.30238	3.80503	2.93759	3.12609
Gglean005936	9.335482	9.33764683	11.428583	10.96855691
Gglean005937	19.67742945	13.51364475	17.521605	17.14501306
Gglean005938	46.7062196	62.8696	36.21901003	33.524819
Gglean005939	46.7062196	62.8696	36.21901003	33.524819

Gglean005940	46.7062196	62.8696	36.21901003	33.524819
Gglean005941	7.1474	7.5419	6.51607	4.757
Gglean005942	6.493575	6.232107	7.13092	9.32435
Gglean005943	21.02751718	20.69637529	31.5591283	34.58158272
Gglean005944	39.2382	38.39810997	21.26384	20.7713141
Gglean005945	39.2382	38.39810997	21.26384	20.7713141
Gglean005946	39.2382	38.39810997	21.26384	20.7713141
Gglean005947	7.63557	9.66863	8.473723	7.385363
Gglean005948	7.63557	9.66863	8.473723	7.385363
Gglean005949	0.938061	0.122844852	0.0479414	0.05648
Gglean005950	0	0	0	0
Gglean005951	31.20404461	34.2263746	29.554414	32.1530461
Gglean005952	31.434412	34.634578	33.0219532	30.784441
Gglean005953	31.434412	34.634578	33.0219532	30.784441
Gglean005954	22.54901518	26.83564	37.47015	34.65611
Gglean005955	43.9779	34.6163	43.2142	60.8345
Gglean005956	38.099	44.3287	36.8839	32.0856
Gglean005957	0	0	0	0
Gglean005958	0	0	0	0
Gglean005959	0	0	0	0
Gglean005960	0	0	0	0
Gglean005961	0	0	0	0
Gglean005962	0	0	0	0
Gglean005963	14.42615	15.60182	14.16892	15.16887
Gglean005964	14.42615	15.60182	14.16892	15.16887
Gglean005965	0	0	0.0602186	0
Gglean005966	0.0831662	0	0.134486	0
Gglean005967	211.174	250.306	147.789	382.66
Gglean005968	0	0	0	0
Gglean005969	0	0	0	0
Gglean005970	0	0	0	0
Gglean005971	0	0	0	0
Gglean005972	0	0	0	0
Gglean005973	1.49685	1.54178	1.88861	3.19606
Gglean005974	0	0	0	0
Gglean005975	0	0	0	0
Gglean005976	0	0	0.0254609	0
Gglean005977	0.0580764	0.118839	0.0234744	0.0138727
Gglean005978	0	0	0	0
Gglean005979	1.25077	1.44696	0.355706	0.281051
Gglean005980	0	0	0	0
Gglean005981	0	0	0	0
Gglean005982	1.02697	0.739938	0	0
Gglean005983	4.891757	3.275359	1.984183	8.834702

Gglean005984	25.412492	28.116748	18.24297101	17.99688307
Gglean005985	25.412492	28.116748	18.24297101	17.99688307
Gglean005986	0	0.107215	0.0425129	0.10042
Gglean005987	58.46293	69.63459945	85.43485	87.47663
Gglean005988	58.46293	69.63459945	85.43485	87.47663
Gglean005989	95.9554	91.8388	85.8874	90.6504
Gglean005990	21.0425	23.373	25.5378	23.0802
Gglean005991	6.03322	7.60523	8.30298	9.82555
Gglean005992	77.16040718	84.7655698	83.08146013	70.5368957
Gglean005993	61.3714366	69.97371557	73.6732261	65.622637
Gglean005994	12.8314	16.4025	13.7971	13.668
Gglean005995	8.60069	9.59632	12.2677	15.6446
Gglean005996	9.71586	0	2.01611	9.50991
Gglean005997	24.3646	17.8185	4.31515	1.71298
Gglean005998	0.0793605	0	0	0
Gglean005999	97.21980003	107.8981653	92.55328391	88.92653922
Gglean006000	27.904566	23.55755	23.69115	25.717318
Gglean006001	27.904566	23.55755	23.69115	25.717318
Gglean006002	1.617498896	1.6892337	2.178918762	2.164463653
Gglean006003	15.6078	15.6343	18.6942	30.5337
Gglean006004	30.08336	27.144159	24.06357525	24.671253
Gglean006005	4.92188406	5.12148956	8.10155	10.16682
Gglean006006	47.59159474	55.4908	58.04926	55.31831
Gglean006007	47.59159474	55.4908	58.04926	55.31831
Gglean006008	2.17821	2.60382	2.16248	2.841
Gglean006009	4.579599	5.182610323	7.525556	8.993538
Gglean006010	20.248147	21.756184	8.208151	9.064620001
Gglean006011	5.132161634	3.386248655	4.074654	3.69092386
Gglean006012	0	0	0	0
Gglean006013	64.8257	67.41140001	85.6171	90.71550003
Gglean006014	64.8257	67.41140001	85.6171	90.71550003
Gglean006015	0.099865539	0.1187349	0.143834193	0.0340311
Gglean006016	10.7295	9.67173	9.69214	13.5337
Gglean006017	0.0807593	0	0	0.155101
Gglean006018	0.514646	0.747989	0.582237	2.46532
Gglean006019	162.87229	177.68465	173.5797	185.0233778
Gglean006020	162.87229	177.68465	173.5797	185.0233778
Gglean006021	186.2899268	175.356734	200.58278	274.1955241
Gglean006022	38.91123	29.16159	20.08429	19.95505
Gglean006023	16.49669396	28.70141	29.92661	34.75895
Gglean006024	16.49669396	28.70141	29.92661	34.75895
Gglean006025	16.49669396	28.70141	29.92661	34.75895
Gglean006026	48.5756	42.2667	25.9335	23.5841
Gglean006027	47.7154056	42.401861	29.16368606	27.92192656

Gglean006028	13.8494	35.6299	42.8832	42.793
Gglean006029	12.13658	12.66373963	19.00816009	22.50662065
Gglean006030	12.13658	12.66373963	19.00816009	22.50662065
Gglean006031	91.02263285	93.3498408	147.2080465	177.6966016
Gglean006032	93.2159	90.6985	97.8209	112.375
Gglean006033	17.8007	16.0967	17.6521	17.3071
Gglean006034	88.00518694	90.79954813	28.11426631	24.04980559
Gglean006035	8.51525	7.57048	7.54084	6.33483
Gglean006036	0.000150913	0.417995	3.93E-20	2.60E-05
Gglean006037	70.75834	75.30774	86.46484	88.09929
Gglean006038	36.0825	39.162	50.6196	53.4487
Gglean006039	4.080509	5.024317	7.096706	8.212048727
Gglean006040	0	0	0	0
Gglean006041	17.39346813	15.969188	20.15523791	19.32760725
Gglean006042	110.244718	107.5935	119.9257983	175.1831498
Gglean006043	7.969659	7.019302351	6.384629008	7.3114063
Gglean006044	0.0152511	0.000922807	5.39E-06	0.0129642
Gglean006045	0	0	0	0
Gglean006046	2.498900492	3.18699	4.14754	4.143845
Gglean006047	0	0	0	0
Gglean006048	0.601951748	0.517025305	1.098334919	1.052942076
Gglean006049	9.39292692	14.64982004	16.3105599	14.47497773
Gglean006050	22.07522	21.382855	19.39113797	19.77426744
Gglean006051	10.554886	13.71768	13.59388	16.28387
Gglean006052	340.34005	373.20715	66.324943	61.79724
Gglean006053	6.73497792	7.696451606	9.276689497	9.999927009
Gglean006054	6.73497792	7.696451606	9.276689497	9.999927009
Gglean006055	0	0	0	0
Gglean006056	0.158416	0.0819307	0.0641328	0
Gglean006057	0	0	0	0
Gglean006058	94.20081	79.9969	184.443	195.7266
Gglean006059	16.54096	20.29559	10.75054	8.75607
Gglean006060	56.1947846	71.481263	67.48616	71.0784655
Gglean006061	65.32193017	72.99816279	63.35344206	54.21220328
Gglean006062	0	0	0	0
Gglean006063	1115.1683	1210.5823	773.4288	732.273
Gglean006064	57.7927	58.6113036	41.4218116	40.601926
Gglean006065	1.278638006	1.229994611	2.303172211	2.591939504
Gglean006066	8.88827635	10.408707	6.5246565	6.250232001
Gglean006067	55.9142	62.6011	77.9976	85.8716
Gglean006068	24.5734	25.8802	22.7289	24.2507
Gglean006069	13.668171	14.61028665	21.058005	29.0502667
Gglean006070	13.668171	14.61028665	21.058005	29.0502667
Gglean006071	0.398483697	0.550497	0.4215666	0.089808757

Gglean006072	82.77839504	93.7573947	75.14447756	66.64405638
Gglean006073	82.77839504	93.7573947	75.14447756	66.64405638
Gglean006074	6.399984482	2.465876824	7.478119473	5.302979493
Gglean006075	60.22979586	47.90708676	84.2953875	81.04935441
Gglean006076	0.120441415	0.0817571	0.1579167	0.0191835
Gglean006077	3.91278	5.30958	4.8052	5.17929
Gglean006078	23.75990144	18.8895136	12.11934709	8.453837548
Gglean006079	21.751822	19.11578492	19.10634256	18.1902999
Gglean006080	21.751822	19.11578492	19.10634256	18.1902999
Gglean006081	43.61718	50.683716	35.759214	32.308214
Gglean006082	43.61718	50.683716	35.759214	32.308214
Gglean006083	9.01825	8.158385	7.45316	4.983784
Gglean006084	1.1832509	1.353634486	0.424473	0.2819137
Gglean006085	9.01825	8.158385	7.45316	4.983784
Gglean006086	36.34176	42.91497	39.53172	39.99108
Gglean006087	0	0	0	0
Gglean006088	0.1812477	0.3393969	0.3229055	0.295595674
Gglean006089	3.99252	4.60295	5.33458	6.97163
Gglean006090	27.880222	30.42048639	6.314077	6.270641167
Gglean006091	157.926	159.605	202.024	243.597
Gglean006092	34.49952	34.04845	28.88103001	25.12808
Gglean006093	34.49952	34.04845	28.88103001	25.12808
Gglean006094	8.160356705	8.498900336	9.70348646	10.90422252
Gglean006095	19.16038	16.36286	13.8688598	14.89088
Gglean006096	19.16038	16.36286	13.8688598	14.89088
Gglean006097	55.2892	57.4608	38.2389	30.6244
Gglean006098	9.78807	8.57863	6.72475	9.67374
Gglean006099	8.98318	6.85087	12.35425	12.35546
Gglean006100	181.2503	158.0336	117.76862	118.35218
Gglean006101	0	0	0	0
Gglean006102	18.5786	22.2085	16.3412	14.8894
Gglean006103	23.46501	27.068773	30.20214	39.62416
Gglean006104	27.8082	31.8467	20.7368	16.6715
Gglean006105	7.45604	14.0945	22.4065	24.3602
Gglean006106	16.48337	11.94478163	11.349766	10.1102
Gglean006107	16.48337	11.94478163	11.349766	10.1102
Gglean006108	2.157075427	3.16708787	1.863801219	1.576406108
Gglean006109	17.27984572	16.180459	29.57109	31.04827753
Gglean006110	34.7328	40.4547	39.9749	38.7107
Gglean006111	295.567	279.187	289.301	354.243
Gglean006112	94.8029	104.3777	96.5132	91.95324
Gglean006113	21.396879	20.510245	28.647329	35.0702
Gglean006114	64.650758	64.330506	23.4139405	18.002964
Gglean006115	64.650758	64.330506	23.4139405	18.002964

Gglean006116	18.71279897	19.271688	13.223466	13.00124044
Gglean006117	9.22222	7.20353	9.255540013	14.2087
Gglean006118	9.22222	7.20353	9.255540013	14.2087
Gglean006119	9.22222	7.20353	9.255540013	14.2087
Gglean006120	17.3801	18.6881	12.9849	13.165
Gglean006121	56.9628775	70.6379549	58.4988262	54.531877
Gglean006122	56.9628775	70.6379549	58.4988262	54.531877
Gglean006123	21.0546	24.39561	20.62983	19.82631
Gglean006124	0	0	0.56592	0
Gglean006125	0	0	0	0
Gglean006126	0	0	0	0
Gglean006127	0.173295591	0.359622509	0.329984169	0.502669484
Gglean006128	0.173295591	0.359622509	0.329984169	0.502669484
Gglean006129	0	0.320858	0.733627	0
Gglean006130	0	0	0	0
Gglean006131	0	0	0	0
Gglean006132	0	0	0.054415	0
Gglean006133	181.8825	192.6644	110.1417	100.3749
Gglean006134	50.40001744	51.71170944	70.56352167	63.2004137
Gglean006135	1.173390946	0.731739298	1.82057957	2.447965767
Gglean006136	51.23504942	52.56562718	59.04798622	55.6748523
Gglean006137	6.49355	15.83304	0.197494	0.204637
Gglean006138	0	0	0	0
Gglean006139	21.35663816	25.95549737	20.46835109	21.95932105
Gglean006140	18.6709386	17.56077056	20.26375743	18.92526003
Gglean006141	2.64737	1.986675	1.780018	2.736517
Gglean006142	0	0	0	0
Gglean006143	0.047336848	0.049005057	0.038480079	0
Gglean006144	11.7293665	10.22650276	14.326303	13.028965
Gglean006145	11.7293665	10.22650276	14.326303	13.028965
Gglean006146	52.611476	53.4004414	56.43062711	49.790782
Gglean006147	52.611476	53.4004414	56.43062711	49.790782
Gglean006148	20.8436359	19.53620408	29.03644353	32.10750164
Gglean006149	305.096	302.618	316.539	308.161
Gglean006150	69.240609	65.42587	38.370968	37.028346
Gglean006151	10.68958046	10.95947	15.48042775	17.94499608
Gglean006152	6.727709756	5.646490014	5.93960249	4.581999257
Gglean006153	85.7644	105.0023	120.5735	113.0589
Gglean006154	26.06201884	26.22508403	39.87232616	42.49820568
Gglean006155	11.71927521	12.39206957	3.232898827	2.674984262
Gglean006156	0.779045	0.999463	1.20413	0.732929
Gglean006157	55.31987	57.10846	50.74633	56.81364
Gglean006158	327.8100316	342.1401101	196.8620497	156.7400918
Gglean006159	169.8235866	179.3980916	78.86532992	68.61161322

Gglean006160	1.08919	2.61E-05	0.724949	1.36533
Gglean006161	34.14536	37.90482	34.23775	31.94225
Gglean006162	0.359902397	0.669263206	0.938193066	0.868589526
Gglean006163	2.602922	4.54605	6.07149	6.79605
Gglean006164	0	0	0	0
Gglean006165	48.67602	64.18523	42.07444	37.71753
Gglean006166	35.4369	35.6265	35.429	35.5116
Gglean006167	66.6410915	78.1499046	78.32475881	89.4101769
Gglean006168	66.6410915	78.1499046	78.32475881	89.4101769
Gglean006169	35.55108192	36.70594542	57.640626	57.71097
Gglean006170	19.3763006	18.94180174	28.71050048	32.51660035
Gglean006171	5.36394	3.00038	3.22785	2.62372
Gglean006172	61.22402199	61.21153243	66.85062671	74.66109591
Gglean006173	1167.389	1216.736	785.031	726.288
Gglean006174	0	0	0	0
Gglean006175	0	0.412737	0	0
Gglean006176	0	0.285843	0	0
Gglean006177	17.55487	16.69037	36.1446	32.3614
Gglean006178	50.35516757	51.17424238	70.06801652	77.99540716
Gglean006179	50.35516757	51.17424238	70.06801652	77.99540716
Gglean006180	4.77624	3.59446	6.56092	6.31425
Gglean006181	31.4335409	40.65821	71.73171	77.54076
Gglean006182	48.4562	53.4295	28.6971	24.9845
Gglean006183	12.8764	13.99782	19.76488	16.52462
Gglean006184	56.0314894	58.57875855	80.2815769	103.2000641
Gglean006185	33.33015	36.59826	34.38046	40.02588
Gglean006186	33.33015	36.59826	34.38046	40.02588
Gglean006187	34.0675	31.595	35.2016	35.7131
Gglean006188	12.63672076	12.63169	12.09332177	13.87045883
Gglean006189	56.37783	51.34451853	14.28757	13.614686
Gglean006190	168.1133701	189.88468	146.4255567	127.99614
Gglean006191	23.2635684	24.10702939	38.652639	41.5823216
Gglean006192	46.99713775	48.20352112	68.23356584	72.4635279
Gglean006193	24.75506974	26.333082	38.787204	42.791367
Gglean006194	24.75506974	26.333082	38.787204	42.791367
Gglean006195	24.75506974	26.333082	38.787204	42.791367
Gglean006196	0.00806642	1.21839	3.24062	2.48636
Gglean006197	10.38542816	7.262407968	5.263294173	5.457656907
Gglean006198	3.34867	3.54047	6.13264	7.08891
Gglean006199	0	0	0.0801158	0
Gglean006200	18.8629	19.9925	19.8522	17.7103
Gglean006201	128.1877005	149.4253575	98.16580021	78.674675
Gglean006202	128.1877005	149.4253575	98.16580021	78.674675
Gglean006203	54.50564472	52.11141129	52.319658	57.38092943

Gglean006204	22.961308	19.502473	36.79163	32.92862
Gglean006205	441.625	404.236	608.403	1013.05
Gglean006206	159.85067	177.6802	121.39116	113.44039
Gglean006207	21.9353945	23.48268723	14.61510139	13.5316271
Gglean006208	80.989688	82.77785	90.74376	109.44028
Gglean006209	80.989688	82.77785	90.74376	109.44028
Gglean006210	80.989688	82.77785	90.74376	109.44028
Gglean006211	115.7803	128.9987	226.8685	220.6393
Gglean006212	46.286	53.2096	70.3061	88.9597
Gglean006213	184.0743	202.8891	347.124	350.485
Gglean006214	80.989688	82.77785	90.74376	109.44028
Gglean006215	80.989688	82.77785	90.74376	109.44028
Gglean006216	49.013	57.2368	6.95166	4.81677
Gglean006217	0.436113	0.376563	0	0.138872
Gglean006218	426.0480035	449.6040037	163.4510035	112.9590066
Gglean006219	0	0	0	0
Gglean006220	2.82463	0.000487317	3.88974	1.92193
Gglean006221	12.47061	9.95638	7.58488	8.605
Gglean006222	0	0	0	0
Gglean006223	1.72946	1.84828	2.53175	4.16481
Gglean006224	72.5826	73.8703	90.2111	103.361
Gglean006225	10.7737	6.120109	7.58427	5.82584
Gglean006226	10.7737	6.120109	7.58427	5.82584
Gglean006227	43.89516	47.08867	33.13418	37.6854156
Gglean006228	103.365669	132.008156	101.807854	143.8467891
Gglean006229	5.84484	5.66082	5.197978	5.7174
Gglean006230	0.79608	0.492838	0.4472215	0.688716657
Gglean006231	0.79608	0.492838	0.4472215	0.688716657
Gglean006232	7.25157	7.34728	6.68767	7.48486
Gglean006233	53.955491	55.60073	27.33093	22.02343
Gglean006234	118.040833	121.45848	52.699128	39.943232
Gglean006235	118.040833	121.45848	52.699128	39.943232
Gglean006236	139.155193	149.2839404	200.651918	265.182931
Gglean006237	6.318005886	7.877413	11.21053	13.1081224
Gglean006238	46.66407416	44.18718	65.53146275	81.50603193
Gglean006239	1.31116	1.63051	1.45156	1.79426
Gglean006240	31.3217988	33.89332	21.491677	21.08984774
Gglean006241	0	0	0	0
Gglean006242	41.00375	42.32318706	52.9423273	57.21119091
Gglean006243	15.98757	16.70084265	19.799697	20.45925
Gglean006244	0	0	0	0
Gglean006245	77.003264	74.483379	48.651722	41.868307
Gglean006246	0	0	0	0
Gglean006247	0	0	0	0

Gglean006248	0.6690971	0.235936	0.431973095	0.693444637
Gglean006249	4.78391	16.3062	15.6858	12.4913
Gglean006250	35.896	28.6656	30.5834	28.2687
Gglean006251	12.441054	13.68257672	15.75486522	19.10852
Gglean006252	0	0	0	0
Gglean006253	0.504546	0.919407	0.614744	2.07206
Gglean006254	63.4159	63.5769	71.6759	83.5236
Gglean006255	2.53985	1.919909	1.896287	4.186794
Gglean006256	84.942103	82.38191892	59.50473344	67.03296
Gglean006257	1470.031	1558.268	936.171	848.3916
Gglean006258	13.93236	19.40323	15.58368	16.36799
Gglean006259	13.93236	19.40323	15.58368	16.36799
Gglean006260	8.724044497	7.539419403	16.14554894	18.21090751
Gglean006261	11.39551	11.733768	4.735235	4.31306
Gglean006262	36.55636313	37.94404144	31.16692641	30.5879825
Gglean006263	20.24837526	26.34689426	45.78946494	46.52657725
Gglean006264	60.2431	50.4375	24.3519	19.2449
Gglean006265	0	0	0	0
Gglean006266	0.0323733	0.100382	0.0262367	0.0622301
Gglean006267	0	0	0	0
Gglean006268	0	0	0	0
Gglean006269	0	0	0.0263486	0
Gglean006270	0	0	0	0.0322525
Gglean006271	25.84919226	19.71172816	35.32912079	34.93822767
Gglean006272	25.84919226	19.71172816	35.32912079	34.93822767
Gglean006273	0	0	0	0
Gglean006274	25.84919226	19.71172816	35.32912079	34.93822767
Gglean006275	25.84919226	19.71172816	35.32912079	34.93822767
Gglean006276	25.84919226	19.71172816	35.32912079	34.93822767
Gglean006277	25.84919226	19.71172816	35.32912079	34.93822767
Gglean006278	7.23850306	8.019411981	11.14812482	11.493085
Gglean006279	7.1448	6.02629	7.51951	7.09788
Gglean006280	3.301858121	4.17169	5.803523842	6.931149
Gglean006281	0	0.241825	0	0
Gglean006282	0	0	0	0
Gglean006283	0	0	0.441064	0
Gglean006284	0	0	0	0
Gglean006285	0.462465739	0	0.039916988	0
Gglean006286	0.029552085	0	0.071840788	0.085622418
Gglean006287	15.51504	14.14047	11.636058	13.25722
Gglean006288	28.14384694	30.5188388	35.38918001	36.74217944
Gglean006289	28.14384694	30.5188388	35.38918001	36.74217944
Gglean006290	37.2001	34.364	36.7196	48.2293
Gglean006291	0	0	0	0

Gglean006292	5.90526	9.83765	10.3634	14.7743
Gglean006293	0	0	0	0
Gglean006294	18.87803799	18.77944612	26.420986	30.62424693
Gglean006295	18.87803799	18.77944612	26.420986	30.62424693
Gglean006296	0	0	0	0
Gglean006297	115.8632	127.375	30.13822	26.61327
Gglean006298	34.82941	35.06563	33.3263	35.68635
Gglean006299	51.12665	50.46724	48.1743	50.30833
Gglean006300	171.9292135	189.4285346	172.191458	173.2252261
Gglean006301	184.6830007	183.319924	124.5082	120.0859305
Gglean006302	184.6830007	183.319924	124.5082	120.0859305
Gglean006303	30.12569346	29.67490376	23.663054	24.29013119
Gglean006304	9.68004	8.85402	6.04086	5.5928
Gglean006305	30.40751	35.48857	34.64076	39.62667
Gglean006306	4.72415	6.18199	9.02835	8.82773
Gglean006307	0	0	0.0287312	0
Gglean006308	59.1963948	54.09931605	81.702383	97.0963933
Gglean006309	37.1798739	41.3023701	41.95532439	40.1482222
Gglean006310	370.2222667	450.686141	212.250859	193.9400324
Gglean006311	20.737605	20.82663	16.68511	17.48665
Gglean006312	81.93705	76.11487	177.6557	165.0032
Gglean006313	8.55425	8.10561	4.72262	4.56477
Gglean006314	0	0	0	0.51199
Gglean006315	0	0	0	0
Gglean006316	79.63799462	79.46414519	69.1957088	66.7861891
Gglean006317	79.63799462	79.46414519	69.1957088	66.7861891
Gglean006318	0.714468258	0.955534876	1.326123832	1.219391227
Gglean006319	49.2466	50.2387	32.4647	33.6852
Gglean006320	0	0	0	0
Gglean006321	11.169405	21.91270934	9.531278766	10.2635909
Gglean006322	11.169405	21.91270934	9.531278766	10.2635909
Gglean006323	11.169405	21.91270934	9.531278766	10.2635909
Gglean006324	0	0	0	0
Gglean006325	37.03676097	36.2048359	53.87340745	73.88506649
Gglean006326	37.03676097	36.2048359	53.87340745	73.88506649
Gglean006327	5.33033	5.46361	7.02516	7.43451
Gglean006328	22.06600627	22.54179362	35.31021987	40.69179554
Gglean006329	22.06600627	22.54179362	35.31021987	40.69179554
Gglean006330	0	0	0	0
Gglean006331	91.27751013	109.1136675	171.2140962	173.1546612
Gglean006332	0.215355291	0.448704591	0.555882	1.285299
Gglean006333	19.70139	24.19604	24.53803	34.51371
Gglean006334	5.97554	9.3954	2.415566	2.328324
Gglean006335	9.90009	10.62962	9.98466	9.40864

Gglean006336	0	0	0	0
Gglean006337	30.0399	27.3325	4.87352	4.96717
Gglean006338	142.759	146.471	114.122	101.442
Gglean006339	25.41034	23.63526	26.71437	24.68046
Gglean006340	0.146273	0.152128	0.120595	0
Gglean006341	0	0	0	0
Gglean006342	0	0	0	0
Gglean006343	0.0755275	0	0.367264	0.289795
Gglean006344	22.6632	26.8329	13.76239	11.00366
Gglean006345	2.561742	2.73356	2.49518	1.97068
Gglean006346	69.58791425	67.400446	65.37664971	62.67489846
Gglean006347	69.58791425	67.400446	65.37664971	62.67489846
Gglean006348	69.58791425	67.400446	65.37664971	62.67489846
Gglean006349	35.02775	37.1496	41.66841	45.52212
Gglean006350	35.02775	37.1496	41.66841	45.52212
Gglean006351	892.397	952.947	635.2170005	600.063
Gglean006352	0.110562	0.201468256	0.13396988	0.2181299
Gglean006353	70.216517	72.70719274	59.295379	57.24624677
Gglean006354	73.2867	63.3765	61.3156	65.2158
Gglean006355	29.572066	34.42279	45.2322684	45.47891
Gglean006356	17.96168	17.24396974	24.47253	30.97319647
Gglean006357	0	0	0	0
Gglean006358	0	0	0	0
Gglean006359	15.2398	7.61518	3.01809	0.525308
Gglean006360	0	0	0	0
Gglean006361	0.377074	0.6101393	1.90283502	1.499574
Gglean006362	0.377074	0.6101393	1.90283502	1.499574
Gglean006363	0	0	5.5311	0
Gglean006364	24.686615	26.49620304	13.89081	10.71724
Gglean006365	24.686615	26.49620304	13.89081	10.71724
Gglean006366	36.008457	36.80532295	37.305423	46.8989441
Gglean006367	36.008457	36.80532295	37.305423	46.8989441
Gglean006368	21.65624452	23.51052066	33.27536885	38.5883888
Gglean006369	21.65624452	23.51052066	33.27536885	38.5883888
Gglean006370	29.873874	31.965707	45.030946	49.685084
Gglean006371	59.0066	54.1327	48.77134	45.0092
Gglean006372	11.37736525	11.86173214	11.56563472	11.29345278
Gglean006373	35.08981307	37.8323	54.07211	62.11577
Gglean006374	28.31363	34.50745	8.795968	8.158597
Gglean006375	2.17789	0.163496	2.53323	2.27264
Gglean006376	29.16444	25.83282	57.64735	68.30724
Gglean006377	29.16444	25.83282	57.64735	68.30724
Gglean006378	0.3611136	0.326593	0.0881173	0.677186
Gglean006379	7.47760061	9.649972	13.63486045	18.326717

Gglean006380	6.68316	7.89178	9.65606	13.50123
Gglean006381	26.4461	28.8036	21.5342	29.7759
Gglean006382	109.2952262	127.8960841	150.523056	174.6836386
Gglean006383	37.58771725	39.37355615	44.0435618	42.00104
Gglean006384	6.61253	6.36679	3.622179	4.769194
Gglean006385	33.28058	37.65349	13.98595	12.61811
Gglean006386	24.5269	22.9082	34.5875	33.288
Gglean006387	50.01100531	45.39812279	58.05348607	67.825282
Gglean006388	50.01100531	45.39812279	58.05348607	67.825282
Gglean006389	50.01100531	45.39812279	58.05348607	67.825282
Gglean006390	9.036600937	9.042203736	5.24592302	5.854605101
Gglean006391	194.116	164.644	214.324	274.587
Gglean006392	0.127234	0.776397	0.832874716	1.361020733
Gglean006393	0	0	0	0
Gglean006394	0	0	0	0
Gglean006395	0	0	0	0
Gglean006396	0	0	0.0306943	0
Gglean006397	21.2943	27.11951	37.94116	37.54125
Gglean006398	25.85657013	28.3797	20.5576	19.497508
Gglean006399	25.85657013	28.3797	20.5576	19.497508
Gglean006400	50.8246416	58.21307311	30.41738113	27.54048859
Gglean006401	57.52452431	66.82171621	36.74902791	40.18479432
Gglean006402	3.83767	2.195	0.818949	0.235111
Gglean006403	27.58935	29.92538549	39.45032456	48.77424
Gglean006404	35.27592222	38.30590771	34.42485	32.08868311
Gglean006405	125.9381554	137.2562704	239.0823905	248.32779
Gglean006406	0.0740422	0	0.0600906	0.0710219
Gglean006407	23.764	47.835	29.3245	34.6638
Gglean006408	49.32689516	48.45052	48.18177	46.9785048
Gglean006409	43.70666	38.8466	37.97205	34.63415
Gglean006410	1105.936	1131.623	691.063	680.795
Gglean006411	34.940422	32.428584	51.6620485	59.0410117
Gglean006412	34.940422	32.428584	51.6620485	59.0410117
Gglean006413	34.940422	32.428584	51.6620485	59.0410117
Gglean006414	96.87490515	104.3379178	120.9906135	109.4390297
Gglean006415	31.2964952	36.38219579	37.20145433	34.09383918
Gglean006416	4.4322	6.299994014	6.202429	5.98692
Gglean006417	6.36401	6.75334	15.52289	16.51006
Gglean006418	50.88647	54.46006	56.6096	58.58323
Gglean006419	50.88647	54.46006	56.6096	58.58323
Gglean006420	50.98726	52.05618	35.89488	36.64249
Gglean006421	69.18900673	68.3588059	54.1613362	51.4466066
Gglean006422	0	0	0.214257	0
Gglean006423	0.610721	0.57336372	0.456598	0.718902

Gglean006424	75.77094241	75.26960934	68.55307234	59.3541896
Gglean006425	12.4599	11.8507	6.56278	6.6908
Gglean006426	76.1307461	82.75657143	78.22295084	78.21198617
Gglean006427	76.1307461	82.75657143	78.22295084	78.21198617
Gglean006428	3.098392832	2.550742644	1.563955	1.511306
Gglean006429	4.593668428	5.218401038	5.245381457	8.1048
Gglean006430	12.196792	14.80349	17.46792	20.3379
Gglean006431	0.0694896	0.142631	0	0.0669274
Gglean006432	0.311274	0.642672	0.252382	0.75185
Gglean006433	10.01999	10.13853	11.52321	9.82374
Gglean006434	9.91335	11.8614	10.8444	13.8805
Gglean006435	31.367084	30.611653	46.572423	56.049289
Gglean006436	25.3228516	32.23044305	37.84787773	40.6920223
Gglean006437	25.3228516	32.23044305	37.84787773	40.6920223
Gglean006438	25.3228516	32.23044305	37.84787773	40.6920223
Gglean006439	33.380008	32.55420707	26.814734	23.45992026
Gglean006440	67.6414	71.1073	53.1931	47.5377
Gglean006441	36.69541001	35.44861714	46.46393116	47.18009181
Gglean006442	12.13036	11.97606822	20.05909	21.58704
Gglean006443	33.0433551	25.70780022	56.222457	55.99643
Gglean006444	33.0433551	25.70780022	56.222457	55.99643
Gglean006445	5.12924	0.00249977	0.00351543	6.76566
Gglean006446	39.6662	40.4251	43.8534	43.4894
Gglean006447	16.80168	15.83487	11.31034	10.39371
Gglean006448	5.410806363	7.234651657	10.416922	8.160911517
Gglean006449	5.410806363	7.234651657	10.416922	8.160911517
Gglean006450	22.01130426	22.65788	21.812988	21.07190324
Gglean006451	25.1564	28.9513	14.9635	11.3964
Gglean006452	6.83707	8.49749	5.91139	5.34937
Gglean006453	3.776048	5.26157	3.7168	5.023709
Gglean006454	14.46212924	15.53488185	22.69530691	23.07842382
Gglean006455	14.46212924	15.53488185	22.69530691	23.07842382
Gglean006456	1.010685746	1.239413374	1.3832172	1.313144054
Gglean006457	3.30E-62	4.74E-68	1.68E-116	2.18E-57
Gglean006458	16.59279138	17.94276296	20.46801614	29.14985147
Gglean006459	16.59279138	17.94276296	20.46801614	29.14985147
Gglean006460	5.43604	9.0974	8.52345	10.7257
Gglean006461	0.0545053	0.111843	0.263497	0.208283
Gglean006462	1.803595273	1.297530441	1.79301738	2.436325
Gglean006463	9.387969	8.3632528	11.07577727	13.67607311
Gglean006464	0	0	0	0
Gglean006465	33.87862296	34.1407944	39.83043756	52.27788416
Gglean006466	0	0	0	0
Gglean006467	0	0	0	0

Gglean006468	4.574462	5.112924	5.66745931	6.650208506
Gglean006469	4.574462	5.112924	5.66745931	6.650208506
Gglean006470	4.574462	5.112924	5.66745931	6.650208506
Gglean006471	19.2035	18.1062	12.185	10.409
Gglean006472	14.6354915	16.31697376	18.31478517	32.18011665
Gglean006473	52.32976	52.3440463	53.41450608	50.27689574
Gglean006474	130.9117378	145.3475243	49.66644852	39.99641407
Gglean006475	0	0	0.0659632	0
Gglean006476	0.059873	0.0620094	0	0.0579611
Gglean006477	0.0744466	0	0.181106	0.0715175
Gglean006478	1.32101147	1.333812612	1.813272126	1.990458597
Gglean006479	0	0	0	0
Gglean006480	0	0	0	0
Gglean006481	90.194166	88.516252	67.20185	61.65468
Gglean006482	90.194166	88.516252	67.20185	61.65468
Gglean006483	1228.498	1468.135	858.015	902.847
Gglean006484	0.6839246	0.864874278	0.647803	0.623961
Gglean006485	80.8528	88.4704	102.0949	110.207
Gglean006486	35.17638	36.5978339	43.78988	43.66104
Gglean006487	27.37667506	27.30848417	17.67949505	17.40267609
Gglean006488	27.37667506	27.30848417	17.67949505	17.40267609
Gglean006489	27.05349	24.85463	33.9733	33.49717
Gglean006490	27.4086	28.209	25.0812	22.9579
Gglean006491	31.1269	30.3383	14.90259	15.01113
Gglean006492	34.0244	38.6835	15.1506	15.2711
Gglean006493	31.6787	36.0562	27.0186	24.4607
Gglean006494	4.599855	7.044300001	2.696006	1.878419
Gglean006495	4.599855	7.044300001	2.696006	1.878419
Gglean006496	64.5084	60.9724	54.14609	56.5336
Gglean006497	8.363998022	8.4540291	11.91463861	14.73126983
Gglean006498	8.363998022	8.4540291	11.91463861	14.73126983
Gglean006499	89.82363337	88.39249025	138.5712882	158.3468703
Gglean006500	0	0	0	0
Gglean006501	0	0	0	0
Gglean006502	20.909962	21.110918	23.69517003	20.38155
Gglean006503	25.7344	29.2378	10.789	8.13144
Gglean006504	0.269761	0.069547	0.0542315	0.321684
Gglean006505	7.23855	2.73697	0	0
Gglean006506	1.00609	0.9215	0.781673	0.720409
Gglean006507	0	0	0	0
Gglean006508	0	0	0	0
Gglean006509	0.549922	5.773461	0.804345	1.70871
Gglean006510	16.45501206	12.40147721	5.44345459	4.345029024
Gglean006511	0.041830035	0.078999	0.091501198	0

Gglean006512	20.38344	21.39193	16.48418	14.3459867
Gglean006513	0.659391	0	0	0
Gglean006514	0.0532068	0.251626196	0.132084997	0.050638723
Gglean006515	0	0	0	0
Gglean006516	46.5569	50.7072	30.90216	27.30154
Gglean006517	32.130676	29.54755008	15.49174731	14.64634003
Gglean006518	32.130676	29.54755008	15.49174731	14.64634003
Gglean006519	0.124531314	0.224502431	0.297648	0.1123882
Gglean006520	0	0	0	0
Gglean006521	0	0	0	0
Gglean006522	0.027797676	0	0	0
Gglean006523	0	0	0	0
Gglean006524	0	0	0	0
Gglean006525	42.02641089	43.38852171	35.80590167	28.12262135
Gglean006526	0.258281	0.32742	0.0836747	0.148596
Gglean006527	11.45850169	12.42737133	17.33218008	17.57467484
Gglean006528	3.3135267	3.385921	2.320916727	2.101200886
Gglean006529	3.3135267	3.385921	2.320916727	2.101200886
Gglean006530	174.043	147.9	111.273	130.628
Gglean006531	11.334324	11.895918	10.182283	10.17046321
Gglean006532	11.334324	11.895918	10.182283	10.17046321
Gglean006533	17.58647476	18.30102704	23.551287	29.2146742
Gglean006534	17.58647476	18.30102704	23.551287	29.2146742
Gglean006535	0.883528	0.387814	0.256289	0.365787
Gglean006536	0	0.141257	0.552462	0.262666
Gglean006537	3.49223	2.70539	3.33679	1.74729
Gglean006538	7.26593	7.8994	0.877082	0.573031
Gglean006539	0.380047	0.499062	0	0
Gglean006540	15.81830432	18.44404918	26.14731979	30.227452
Gglean006541	0	0	0	0.0521982
Gglean006542	0	0	0	0
Gglean006543	0	0.234871	0	0
Gglean006544	0	0	0.372628	0.449222
Gglean006545	24.97331172	24.72018906	35.32660779	32.24411865
Gglean006546	0	0	0	0
Gglean006547	0	0	0	0
Gglean006548	0	0	0	0
Gglean006549	4.83525	4.49176	6.20976	9.65119
Gglean006550	33.5098	33.0032	17.0506	15.0986
Gglean006551	173.04195	188.4543	122.423271	117.397352
Gglean006552	0.153265482	0.404722197	0.11592808	0.16794455
Gglean006553	0.153265482	0.404722197	0.11592808	0.16794455
Gglean006554	30.9172	52.1758	15.917	13.3434
Gglean006555	30.9172	52.1758	15.917	13.3434

Gglean006556	3.14445	3.3527	1.55279	4.02977
Gglean006557	0	0	0	0.112841
Gglean006558	0.0808125	0	0.132476	0.31445
Gglean006559	0.88474477	1.593447081	1.98219249	2.04562645
Gglean006560	0.88474477	1.593447081	1.98219249	2.04562645
Gglean006561	0	0	0	0
Gglean006562	15.48658	15.96318	23.48219	29.4769
Gglean006563	0	0	0.0369639	0.0436038
Gglean006564	0	0	0.0563192	0
Gglean006565	15.60608	18.88247	14.134419	10.344
Gglean006566	69.3920037	81.90100292	60.58890819	56.22520589
Gglean006567	0.780985895	0.676824027	0.932127021	1.29916865
Gglean006568	0.780985895	0.676824027	0.932127021	1.29916865
Gglean006569	0	0	0	0
Gglean006570	0	0	0	0
Gglean006571	0.739632762	0.849844678	1.307560887	1.093420625
Gglean006572	76.81455351	82.51179388	123.134156	112.4316961
Gglean006573	80.81800504	84.19091271	135.1880108	127.547021
Gglean006574	1.48587	1.60253	1.5637	2.56842
Gglean006575	0	0	0	0
Gglean006576	0	0	0	0.0624792
Gglean006577	0	0	0	0
Gglean006578	0	0	0	0
Gglean006579	0	0	0	0
Gglean006580	2.68095	2.15591	4.4699	3.9942
Gglean006581	1.156755	1.582587	2.809293	2.016871
Gglean006582	102.0700681	88.342151	160.1728606	148.605543
Gglean006583	102.0700681	88.342151	160.1728606	148.605543
Gglean006584	1.66048	2.20731	3.43535	3.88056
Gglean006585	4.57E-07	2.20638	1.1521	1.71739
Gglean006586	102.0700681	88.342151	160.1728606	148.605543
Gglean006587	2.60073	3.29037	3.98911	4.2037
Gglean006588	2.09794	3.89036	3.89604	2.81661
Gglean006589	0.508977	0.522228	1.19048	0.596505
Gglean006590	1.45686	1.2284	3.33226	2.04499
Gglean006591	4.66249	4.09319	7.47979	8.07157
Gglean006592	4.18484	4.43418	8.02871	3.71718
Gglean006593	1.20042	1.2685	4.10885	2.54029
Gglean006594	3.08452	4.15079	8.37809	6.09877
Gglean006595	4.67546	3.49339	6.90218	5.1953
Gglean006596	3.757164	3.4878994	8.193682782	5.21876
Gglean006597	102.0700681	88.342151	160.1728606	148.605543
Gglean006598	102.0700681	88.342151	160.1728606	148.605543
Gglean006599	102.0700681	88.342151	160.1728606	148.605543

Gglean006600	0	0	0	0
Gglean006601	6.706692553	7.081530078	6.2179146	7.46484361
Gglean006602	6.706692553	7.081530078	6.2179146	7.46484361
Gglean006603	1.98747	2.74695	2.58297	2.80787
Gglean006604	0	0	0	0
Gglean006605	0	0	0	0
Gglean006606	59.07473018	62.377969	92.51165889	106.5838726
Gglean006607	26.2666073	24.37160673	22.9942555	21.99411003
Gglean006608	12.90917268	12.84994339	17.933276	20.41316332
Gglean006609	12.90917268	12.84994339	17.933276	20.41316332
Gglean006610	3.9870246	4.152455	4.146106	5.576648
Gglean006611	3.9870246	4.152455	4.146106	5.576648
Gglean006612	3.951870586	3.692532409	3.833246517	6.495399
Gglean006613	3.951870586	3.692532409	3.833246517	6.495399
Gglean006614	0.259121985	0	0.534465711	0.585491
Gglean006615	0	0	0	0
Gglean006616	0	0	0	0
Gglean006617	0	0	0	0
Gglean006618	0	0.378153	0.290814	0
Gglean006619	0	0	4.36037	0
Gglean006620	0.24972	0	0.100736	0
Gglean006621	0.606445	0.739829	0.224407	0.90152
Gglean006622	0	0	0	0
Gglean006623	0	0	0	0
Gglean006624	0.990538	0.555948	0.41152	0.952148
Gglean006625	0	0	0	0
Gglean006626	0	0	0	0
Gglean006627	0.1585101	0.0473448	0.0248227	0
Gglean006628	0.1585101	0.0473448	0.0248227	0
Gglean006629	85.4567	79.9268	64.5551	64.1085
Gglean006630	85.4567	79.9268	64.5551	64.1085
Gglean006631	26.53904	27.566367	10.45612	8.00366
Gglean006632	58.708	63.7071	31.8179	28.6914
Gglean006633	65.1913	68.17354656	81.43326	91.6202
Gglean006634	24.288898	32.882607	39.2135	51.158679
Gglean006635	24.288898	32.882607	39.2135	51.158679
Gglean006636	114.36138	116.3495	92.4364	78.864
Gglean006637	0	0	0	0.35613
Gglean006638	0	0	0	0
Gglean006639	0.225296	0.280424	0.441893	0.827897
Gglean006640	1.309832995	1.733376	1.81475	2.566778
Gglean006641	0.248411	0.823348	0.4638942	0.3008283
Gglean006642	3.8762	12.9544	9.96672	5.68549
Gglean006643	3.8762	12.9544	9.96672	5.68549

Gglean006644	3.66283	3.59933	2.58699	3.75304
Gglean006645	3.56187	4.92851	5.57801	6.89631
Gglean006646	6.790156	6.019919	8.817756526	10.653723
Gglean006647	0.440634	0.131195	0.306295	0.241854
Gglean006648	1.64E-246	5.81E-18	0.239007	3.88E-37
Gglean006649	0.0540088	0.0561825	0.15457	0
Gglean006650	37.40408174	39.3011876	24.64779937	18.6022357
Gglean006651	18.49275	18.36382	27.49899	35.58816
Gglean006652	68.6671	69.3017	35.1815	27.5179
Gglean006653	14.67322	14.161065	10.42227007	8.37111
Gglean006654	9.384146	9.121034	7.281894	8.2927012
Gglean006655	0	0.103152	0	0
Gglean006656	0	0	0	0
Gglean006657	0.661612	1.07487	1.3634	0.98929
Gglean006658	0	0	0	0
Gglean006659	0	0	1.11424	0
Gglean006660	0.20736	0.169265	0.133501	0.119332
Gglean006661	0	0	0	0
Gglean006662	0	0	0	0
Gglean006663	0	0	0	0
Gglean006664	0	0	0	0
Gglean006665	0	0	0	0
Gglean006666	100.0661	74.4243	91.0781	103.735
Gglean006667	100.0661	74.4243	91.0781	103.735
Gglean006668	29.76845	33.54739	31.00024	29.833686
Gglean006669	94.36288407	98.77236937	53.37250152	47.98213427
Gglean006670	0	0	0	0
Gglean006671	0	0	0	0
Gglean006672	0.274596	0	0.221522	0
Gglean006673	2.641416718	3.007652061	3.687579556	4.196868532
Gglean006674	0	0	0	0
Gglean006675	0.0990943	0.608533	0	0
Gglean006676	0.725023	0.669227	0.222722	0.199234
Gglean006677	2.22298	2.12873	2.74569	2.57268
Gglean006678	1.969702503	1.98973744	1.181826	0.831307372
Gglean006679	0	0	0	0
Gglean006680	0	0	0	0
Gglean006681	75.7181	83.325	217.523	216.173
Gglean006682	30.59595	27.1637	31.1049	33.0661
Gglean006683	0.4865085	0.331625104	0.727500977	0.572642514
Gglean006684	0.4865085	0.331625104	0.727500977	0.572642514
Gglean006685	198.871888	195.0434017	261.591598	342.938265
Gglean006686	3.76634	6.65083	3.09898	3.00035
Gglean006687	0	0	0	0

Gglean006688	0.100891	0.102981	0.13493	0.0319276
Gglean006689	0	0.0997788	0.0522201	0
Gglean006690	0.22728	0.481523	0.184549	0.220927
Gglean006691	612.737	641.957	315.162	319.404
Gglean006692	125.456	129.936	29.5326	29.7169
Gglean006693	48.504857	37.862162	54.854333	61.109921
Gglean006694	0.043111048	0.228686404	0.208560062	0.043607831
Gglean006695	105.7932	104.72	49.5975	44.1615
Gglean006696	0.383862	0.51242	0.403437	0.257881
Gglean006697	21.01196857	18.50611469	12.40800202	10.99023938
Gglean006698	0	0	0	0
Gglean006699	0.644789	0.402804	0.122330093	0.33955
Gglean006700	0.644789	0.402804	0.122330093	0.33955
Gglean006701	0	0	0	0
Gglean006702	0	0	0	0
Gglean006703	1.99622	2.53974	0.698956	0.864803
Gglean006704	0	0	0	0
Gglean006705	0.6095111	0.5062507	1.143495	0.356274096
Gglean006706	30.78608371	37.071657	29.195301	30.115934
Gglean006707	30.78608371	37.071657	29.195301	30.115934
Gglean006708	30.78608371	37.071657	29.195301	30.115934
Gglean006709	21.21945	21.8212	19.44334774	18.27967
Gglean006710	0.910621	1.27374	1.44982	3.30353
Gglean006711	0	0	0.329358	0.197377
Gglean006712	0	0.124316	0	0
Gglean006713	68.51054	75.96217	99.983923	102.39076
Gglean006714	292.02666	289.5829	303.458101	321.2162131
Gglean006715	39.8690332	47.36081201	45.77643934	48.03364
Gglean006716	66.52328201	96.9946	5.66713	3.19318706
Gglean006717	21.99520522	22.45777113	18.79200475	17.8408307
Gglean006718	1.167283055	1.570314081	1.925394313	2.438561076
Gglean006719	1.167283055	1.570314081	1.925394313	2.438561076
Gglean006720	0	0	0	0
Gglean006721	9.63321	11.0344	15.9347	20.3214
Gglean006722	1.016260623	1.228406186	0.384148042	0.4372308
Gglean006723	38.66134853	39.0979887	47.26804645	51.19900821
Gglean006724	35.67182	40.50733	16.580418	14.217369
Gglean006725	62.08444431	61.08643112	65.06055686	69.70551103
Gglean006726	1.58585	1.5139937	1.79674	1.14867
Gglean006727	86.82481152	83.2966196	55.1257115	49.81951985
Gglean006728	0	0	0.501578	0.298916
Gglean006729	54.79287249	56.32196032	77.31930768	109.3200107
Gglean006730	0	0	0	0
Gglean006731	0.063121	0.0971931	0	0

Gglean006732	0	0	0	0
Gglean006733	0.400294	0.365554	0.449979	0.145028
Gglean006734	0	0	0	0
Gglean006735	0	0	0	0
Gglean006736	0	0	0	0
Gglean006737	0	0	0	0
Gglean006738	0	0	0.992947	0
Gglean006739	0.793248	0.993711	0.904085	0.615135
Gglean006740	0	0	0	0
Gglean006741	0.0596952	0.0304592	0.0483481	0
Gglean006742	0.336237029	0.992766553	0.230737695	0.070746809
Gglean006743	0.336237029	0.992766553	0.230737695	0.070746809
Gglean006744	0	0	0	0
Gglean006745	0	0	0	0
Gglean006746	0.0866833	0	0	0
Gglean006747	0.0258714	0.0266249	0.0208866	0
Gglean006748	2.11769	0.497893	0.580456	1.38147
Gglean006749	7.570315845	6.649253123	6.382685391	6.511989425
Gglean006750	0	0	0	0
Gglean006751	0	0	0	0
Gglean006752	0	0	0	0
Gglean006753	0	0	0	0
Gglean006754	0	0	0	0
Gglean006755	0	0.0146599	0.0241337	0.0143612
Gglean006756	0.515106	0.472296	1.79921	1.45576
Gglean006757	21.76631787	19.96505718	30.302546	35.36436921
Gglean006758	7.54073	11.0503	0.96828	0.946888
Gglean006759	0.916899413	0.925642	1.316099813	0.841693942
Gglean006760	40.41644617	40.02665214	15.74509693	13.478716
Gglean006761	17.06792	14.2528	16.88869	16.67693
Gglean006762	0.147825795	0.450692205	0.088858992	0.371115148
Gglean006763	14.1122	16.50831	3.425169	2.969454
Gglean006764	10.29938	8.57811	11.47693	14.4873
Gglean006765	19.73012221	18.88503195	10.06052847	8.840064285
Gglean006766	19.73012221	18.88503195	10.06052847	8.840064285
Gglean006767	236.1507	233.7279	70.1117	64.4314
Gglean006768	89.6739	129.339	130.619	140.338
Gglean006769	22.133	19.1569	19.6693	22.5013
Gglean006770	3.78562	6.3926441	4.562824414	6.947396
Gglean006771	3.78562	6.3926441	4.562824414	6.947396
Gglean006772	15.57956452	14.01990839	20.280508	21.912198
Gglean006773	0	0	0	0
Gglean006774	0	0.144107	0	0
Gglean006775	0	0.17869	0	0

Gglean006776	0	0	0	0.19016
Gglean006777	0	0	0.212858	0
Gglean006778	0.19955	0.207271	0	0
Gglean006779	20.084429	20.41091237	24.94325	32.65730777
Gglean006780	0	0	0	0
Gglean006781	61.3022349	64.14783446	39.99234677	41.32277198
Gglean006782	9.531414294	8.680003	6.078121	6.721466262
Gglean006783	15.7022	14.5216	16.6949	20.6623
Gglean006784	15.62196	15.12072	18.49652	15.41016
Gglean006785	28.16544	19.6435	16.051698	17.700232
Gglean006786	28.16544	19.6435	16.051698	17.700232
Gglean006787	33.41418247	35.56972135	41.14637323	42.7363328
Gglean006788	0.538297	0	0	0.532252
Gglean006789	16.101	16.2851	8.12233	8.31379
Gglean006790	11.0205136	11.70937113	18.2427135	23.39348952
Gglean006791	11.0205136	11.70937113	18.2427135	23.39348952
Gglean006792	45.23481239	57.22980987	45.31771612	36.15906764
Gglean006793	16.10781	19.97102	11.20036	11.04357
Gglean006794	115.1583505	127.9758535	147.4717035	148.7878
Gglean006795	39.25728	37.9357	53.30965	68.4119
Gglean006796	0	0	0	0
Gglean006797	3.77403	3.4535	4.60761	3.63202
Gglean006798	15.45855406	18.15903746	9.038104797	7.128427984
Gglean006799	0	0	0.685523	0.821136
Gglean006800	264.6787	306.1141	247.9024	248.4395
Gglean006801	7.08029	8.49401	2.41163	1.83932
Gglean006802	51.292908	51.21838853	62.26626	73.7022
Gglean006803	21.63666338	15.29860918	15.1742817	18.18852559
Gglean006804	0.989474	0.837839	1.47581	0.977375
Gglean006805	30.67861333	31.63521064	31.49251979	30.30010898
Gglean006806	3.61349	3.21619	2.1873	3.82382
Gglean006807	26.59736	31.6918	9.30362	8.89958
Gglean006808	5.5851275	7.69889	8.827429746	12.20963404
Gglean006809	14.62627	17.004231	12.93803	8.176292
Gglean006810	12.51463	10.45664	10.34588	14.38883
Gglean006811	24.34457786	33.37272061	44.65417322	50.24908748
Gglean006812	11.62304	10.49831	16.01084	20.49225
Gglean006813	24.71881519	20.01484542	25.10001199	35.05061589
Gglean006814	0	0	0	0
Gglean006815	5.393676	4.006537	7.28947	9.003927
Gglean006816	94.18434064	105.985592	83.569967	93.61781002
Gglean006817	64.3527	71.5314	47.98394	54.8488
Gglean006818	36.5968	36.659	11.38235	8.6796
Gglean006819	64.60428	66.38458	15.21588	12.56893

Gglean006820	19.99039627	17.95062336	31.50330886	30.17543747
Gglean006821	19.99039627	17.95062336	31.50330886	30.17543747
Gglean006822	19.99039627	17.95062336	31.50330886	30.17543747
Gglean006823	39.9661	43.1825	33.4981	25.4238
Gglean006824	0	0	0	0
Gglean006825	34.27132735	36.20086631	14.1721922	16.8851544
Gglean006826	34.27132735	36.20086631	14.1721922	16.8851544
Gglean006827	7.91933	4.818260884	10.87840096	8.965687831
Gglean006828	72.3125067	74.322814	123.592337	131.006631
Gglean006829	72.3125067	74.322814	123.592337	131.006631
Gglean006830	20.69250974	22.89937	11.45962	8.945319
Gglean006831	0	0	0	0
Gglean006832	51.338437	52.25963134	12.46482	9.52621
Gglean006833	72.93317294	76.81448	41.24335496	35.19914
Gglean006834	32.8333	32.5925	23.88612	24.5471
Gglean006835	8.57179	7.979719628	5.488647	5.061037
Gglean006836	8.57179	7.979719628	5.488647	5.061037
Gglean006837	20.23764	21.54094	15.28752	14.56824
Gglean006838	20.71066	37.313	10.04019	7.85405
Gglean006839	17.00653	15.16427	13.90327	10.77932
Gglean006840	19.5582	24.5316	37.8125	45.9579
Gglean006841	31.0043	31.32262	37.8125	45.9579
Gglean006842	31.8285	27.3072	42.539114	28.419792
Gglean006843	31.8285	27.3072	42.539114	28.419792
Gglean006844	155.5603	154.8724	112.4006	100.6555
Gglean006845	32.418171	31.379534	70.8787239	66.380339
Gglean006846	0	0	0	0
Gglean006847	0	0	0	0
Gglean006848	153.327031	176.2720083	28.27320819	27.39850633
Gglean006849	0	0	0	0
Gglean006850	0	0	0.173602	0.205301
Gglean006851	47.917599	46.68689	33.5826819	32.5132672
Gglean006852	33.552219	33.446215	31.360375	35.323809
Gglean006853	33.552219	33.446215	31.360375	35.323809
Gglean006854	3.999072	3.048419	4.733458	7.5801473
Gglean006855	0	0	0	0
Gglean006856	0.864036	0.493777	1.02001	0.653802
Gglean006857	0.825822428	0.718784005	1.346108088	1.282097383
Gglean006858	24.636214	23.64568722	10.82312511	10.10544695
Gglean006859	20.6885	18.3093	12.215	12.2019
Gglean006860	10.5745	10.9221	5.75007	4.78153
Gglean006861	41.25596	47.19040004	39.35288	37.005
Gglean006862	0.0753534	3.27E-06	0.000189446	0.038525
Gglean006863	0	0	0	0

Gglean006864	0.0808113	0.16562	0	0.23716
Gglean006865	68.40563715	71.05363347	45.08611	45.79825
Gglean006866	68.40563715	71.05363347	45.08611	45.79825
Gglean006867	31.88052567	31.19162	34.57781235	34.35399
Gglean006868	17.791771	17.832898	5.857913	5.877209
Gglean006869	17.791771	17.832898	5.857913	5.877209
Gglean006870	25.02004	24.63015	19.315	20.91638
Gglean006871	125.9552	117.8267	161.63978	157.7432
Gglean006872	0.04381	0.0887495	0.072912	0.0437204
Gglean006873	40.1459	45.55087107	37.3088	36.456
Gglean006874	118.776118	124.9157736	121.6010386	118.7388916
Gglean006875	22.14776	24.807839	30.39884	28.65475
Gglean006876	44.24874	49.3893	73.221	72.2958
Gglean006877	20.433	20.1424	4.04699	6.65759
Gglean006878	6.47693	6.561798	10.4658	10.73957
Gglean006879	29.1473	28.1358	19.9969	16.7412
Gglean006880	9.26374	12.0361	2.2406	3.75482
Gglean006881	38.51057459	44.44176473	34.279995	36.04407589
Gglean006882	46.8996	54.3324	37.48	31.4885
Gglean006883	88.08657	96.9998	80.40204	82.0452
Gglean006884	8.137837	9.38964	6.79458	8.45813
Gglean006885	18.0985	19.565	13.3255	12.6195
Gglean006886	10.92467	12.60996	9.18328	7.61979764
Gglean006887	10.92467	12.60996	9.18328	7.61979764
Gglean006888	23.88916213	26.18019	6.926448147	5.732028
Gglean006889	5.782528353	5.464467672	2.97496904	3.188769211
Gglean006890	224.9385	213.3673	143.999	145.7888
Gglean006891	20.26524467	18.46307501	35.13129203	41.95955574
Gglean006892	79.31887762	98.788262	117.0793949	117.3090002
Gglean006893	79.31887762	98.788262	117.0793949	117.3090002
Gglean006894	27.65235	30.24056	28.34215	29.37201
Gglean006895	7.08125	5.56417	4.55855	4.64974
Gglean006896	23.18065	19.63946	20.04699	22.48137
Gglean006897	30.05412	27.8965	32.36627	34.26619
Gglean006898	36.42	38.0474	27.6958	31.3111
Gglean006899	64.0941527	73.5816361	63.04540805	54.53087428
Gglean006900	64.0941527	73.5816361	63.04540805	54.53087428
Gglean006901	19.47153231	18.64319628	15.93907834	15.6556967
Gglean006902	0	0	0	0
Gglean006903	0.0274881	0.0283239	0.0223162	0
Gglean006904	8.43633	8.767192849	11.725065	13.35955807
Gglean006905	8.43633	8.767192849	11.725065	13.35955807
Gglean006906	22.85854	25.2201	21.25456	24.18731
Gglean006907	23.3055	27.1454	13.297	12.8548

Gglean006908	76.6873	80.6226	88.6917	97.6779
Gglean006909	156.918	158.78	109.948	96.0604
Gglean006910	8.375752	7.197750638	11.78406	13.46916768
Gglean006911	8.375752	7.197750638	11.78406	13.46916768
Gglean006912	8.522794	8.63161	5.62829	4.59777
Gglean006913	7.64302	10.2184	5.81623	5.02783
Gglean006914	149.02229	151.35326	160.33226	171.84724
Gglean006915	38.72372	31.434305	35.3059	32.95875
Gglean006916	43.57965	46.72745	41.46119	41.06038
Gglean006917	14.29793	16.28038	22.1781	27.4337
Gglean006918	0	0	0	0
Gglean006919	13.855283	12.41735	17.09788	20.02185
Gglean006920	1393.8162	1552.18317	945.6278948	925.268706
Gglean006921	0	0	0	0
Gglean006922	0	0	0.127099	0.0753185
Gglean006923	0	0	0	0.205193
Gglean006924	0	0	0	0
Gglean006925	0	0.334075	0	0
Gglean006926	14.77207	13.5512	19.23283	20.31238
Gglean006927	185.3027	195.7134	54.0138	33.7646
Gglean006928	0	0	0	0
Gglean006929	0.452658898	0.536854084	0.0731161	0.43676229
Gglean006930	0	0.055314	0.0437365	0.471216
Gglean006931	0	0	0	0
Gglean006932	0	0	0	0
Gglean006933	1.29E-05	5.54E-05	0.000402996	0.000756284
Gglean006934	10.48535	11.5624	3.181832	2.553986
Gglean006935	0	0	0.1926359	0
Gglean006936	2.829048671	2.831361189	1.9209764	1.928422618
Gglean006937	1.896907949	1.220292	0.631183841	0.67043
Gglean006938	0	0	0	0
Gglean006939	0.460791	0.680136	0.627818	0.481201
Gglean006940	15.5058	17.7487	7.59422	6.32292
Gglean006941	39.8000159	40.08638438	11.27883013	10.95236684
Gglean006942	39.8000159	40.08638438	11.27883013	10.95236684
Gglean006943	18.0656539	24.524454	17.4961987	20.710397
Gglean006944	18.0656539	24.524454	17.4961987	20.710397
Gglean006945	18.0656539	24.524454	17.4961987	20.710397
Gglean006946	18.0656539	24.524454	17.4961987	20.710397
Gglean006947	51.7961	49.407	52.0538	60.519
Gglean006948	19.30201	20.4184301	26.07273421	30.947718
Gglean006949	19.30201	20.4184301	26.07273421	30.947718
Gglean006950	3.8575	3.886982	4.709618	7.01813
Gglean006951	2.253093	2.144191	3.198824	3.149491822

Gglean006952	10.086967	8.362720273	13.423757	15.02262
Gglean006953	0.111281	0	0	1.218912215
Gglean006954	0	0	0	0.145471
Gglean006955	0	0.396124	0	0
Gglean006956	0	0	0	0
Gglean006957	0	0	0	0
Gglean006958	0	0	0	0
Gglean006959	0.0466824	0	0.0380619	0.0454796
Gglean006960	0	0.0437212	0.0343236	0.0407614
Gglean006961	0	0	0	0
Gglean006962	0.0843771	0	0	0
Gglean006963	0	0	0	0
Gglean006964	0	0	0	0
Gglean006965	0	0	0	0
Gglean006966	0	0	0	0
Gglean006967	0.237525139	0.122586571	0.319077443	0.189088189
Gglean006968	31.2543	28.78391	33.43973	29.00504
Gglean006969	0	0	0	0
Gglean006970	0	0	0	0
Gglean006971	4.02554	4.69086	4.50284	4.05822
Gglean006972	0.118707	0	0	0.11658
Gglean006973	0	0	0	0
Gglean006974	0.019867584	0.047951321	0.024982375	0.018819507
Gglean006975	0	0	0	0
Gglean006976	0	0	0	0
Gglean006977	0	0	0	0
Gglean006978	0	0	0	0
Gglean006979	0	0	0	0
Gglean006980	20.53289	18.2238	30.47608246	31.4086
Gglean006981	0.0969788	3.43E-05	5.89915	2.67738
Gglean006982	2.97123	3.48446	11.2571	8.87204
Gglean006983	43.58623	41.55456	32.06102	29.92133
Gglean006984	10.36164811	10.46987897	8.7515215	9.97608087
Gglean006985	10.36164811	10.46987897	8.7515215	9.97608087
Gglean006986	10.36164811	10.46987897	8.7515215	9.97608087
Gglean006987	0.0257252	0	0.0309998	0.0122676
Gglean006988	14.43787	14.7687	26.9458	28.12218
Gglean006989	27.0414	27.3246	21.5996	40.6445
Gglean006990	24.1146	24.2567	27.4616	24.3633
Gglean006991	2.35699	2.27E-280	0	2.31719
Gglean006992	19.26087	17.37599	15.82449	30.8742
Gglean006993	0.600708904	0.578846377	0.773718358	0.96367865
Gglean006994	0.40927749	1.108095972	0.385630395	12.87770914
Gglean006995	0.0620338	0.0641374	0.452943	0.23993

Gglean006996	31.1664	48.6435	52.2562	58.067
Gglean006997	1.44299	2.2468	0.118072	0.42131
Gglean006998	0.191316	0.0323986	0.0257786	0.0307542
Gglean006999	4.74834	4.28552	6.96112	2.65617914
Gglean007000	33.35312674	34.97962626	61.97985044	52.7880977
Gglean007001	29.2695	28.9806	18.8738	17.86012
Gglean007002	106.8384	118.2683	102.5893	90.4133
Gglean007003	3.860295971	5.82432	3.641455782	3.719495
Gglean007004	3.860295971	5.82432	3.641455782	3.719495
Gglean007005	5.88847	5.48446	4.59418	3.3887
Gglean007006	0	0	0	0
Gglean007007	23.047414	21.040524	7.877548	6.262487
Gglean007008	68.79775053	66.38393	105.21403	112.16784
Gglean007009	44.75798	43.12517	20.62155	16.89319
Gglean007010	4.45948	0	0	0
Gglean007011	0.687830155	0.216621749	0.359445428	0.7543747
Gglean007012	50.923393	86.680732	108.00967	105.104732
Gglean007013	2.12227	4.93699	0.364434	0.216903
Gglean007014	1.12486	7.08083	0	0
Gglean007015	0	0	0	0
Gglean007016	0	0	0	0
Gglean007017	40.13197271	36.98647269	58.37361229	74.46323237
Gglean007018	155.2406	167.3981	134.4397	168.7846
Gglean007019	27.92726514	30.563316	30.7208095	29.94289754
Gglean007020	13.190241	11.419815	14.72377	12.12564
Gglean007021	1.83572	2.64681	1.97263	1.85434
Gglean007022	20.0643	21.4387	11.6671	10.4153
Gglean007023	17.34543694	17.90324827	29.73130621	32.01720864
Gglean007024	201.3914	195.7036	175.7734	180.422
Gglean007025	1.41368	1.0663	2.53558	2.25633
Gglean007026	0.045987	0	0	0.0443245
Gglean007027	37.1149	36.3851	42.1482	39.4404
Gglean007028	87.61234	73.296695	88.515078	130.792563
Gglean007029	8.40353418	7.089146921	13.3988085	12.8810043
Gglean007030	2.97187	3.124763	0.526861	1.766242
Gglean007031	463.5492239	499.6745198	217.262522	168.8317164
Gglean007032	3.64119	3.25223	1.84665	1.41013
Gglean007033	20.62346	23.71585	20.26767738	21.38032
Gglean007034	80.46082	77.16405725	64.53628972	67.09743588
Gglean007035	49.5523	57.5441	56.2083	79.6386
Gglean007036	325.211	309.878	377.964	480.361
Gglean007037	0.32044	0.708816	0.0427307	0.558842
Gglean007038	3.69552	4.21258	3.8483	6.42459
Gglean007039	118.8949183	120.2200101	135.118159	196.0536142

Gglean007040	602.5728	587.3797	657.1672	970.864
Gglean007041	0.168958793	0.31337375	0.565774938	0.606080832
Gglean007042	21.10827452	23.82119352	12.83315287	11.00268247
Gglean007043	71.43049533	70.78697761	60.63381032	49.93112499
Gglean007044	0.378668773	0.706078378	0.646451735	0.512405
Gglean007045	0.378668773	0.706078378	0.646451735	0.512405
Gglean007046	0.02056719	0	0	0.03941725
Gglean007047	0.563503599	1.698941	1.008692672	0.649966213
Gglean007048	0.563503599	1.698941	1.008692672	0.649966213
Gglean007049	4.499215427	4.807193149	5.0533599	7.88389286
Gglean007050	7.734893982	9.165766519	13.30707551	12.319928
Gglean007051	10.41028	10.52845	7.040829	7.00516
Gglean007052	10.41028	10.52845	7.040829	7.00516
Gglean007053	30.31826	30.57062	25.22534	26.1515
Gglean007054	13.84983	16.66142	24.90551	20.17691
Gglean007055	79.73793	80.369672	56.041323	51.63576
Gglean007056	79.73793	80.369672	56.041323	51.63576
Gglean007057	0	0	0	0.0641468
Gglean007058	33.7592466	29.06194487	26.03183886	26.74356744
Gglean007059	1065.086933	1151.748624	1540.068979	1867.81512
Gglean007060	1065.086933	1151.748624	1540.068979	1867.81512
Gglean007061	1065.086933	1151.748624	1540.068979	1867.81512
Gglean007062	14.46151231	17.234347	7.625289635	8.382654
Gglean007063	10.7845	10.0826	12.1497	8.85427
Gglean007064	0.469629	0	0.253872	0.525636
Gglean007065	29.26350712	28.431463	42.98694	48.95014
Gglean007066	0.31299428	0.464036141	0.067947151	0.264778835
Gglean007067	13.18840291	13.94013	21.402915	24.155346
Gglean007068	9.6156836	12.98914038	12.54905622	19.76477236
Gglean007069	48.703681	45.38342413	28.89169825	28.4801888
Gglean007070	0	0	0	0.0308046
Gglean007071	0	0	0	0
Gglean007072	0.231419639	0.37579109	0.395406	0.429485761
Gglean007073	0	0.410304	0.159869	0
Gglean007074	1.37497	1.57696	1.66995	2.37655
Gglean007075	0	0	0	0
Gglean007076	9.684461	9.964054274	13.19218459	15.70077757
Gglean007077	0	0	0.250409	0
Gglean007078	19.09517474	19.81016635	28.9233737	24.25122195
Gglean007079	19.09517474	19.81016635	28.9233737	24.25122195
Gglean007080	19.09517474	19.81016635	28.9233737	24.25122195
Gglean007081	59.90196645	44.44096	140.0841	147.67024
Gglean007082	12.65151126	12.86004638	15.01258461	19.07456907
Gglean007083	8.395581982	7.481189	15.007155	15.2343728

Gglean007084	8.395581982	7.481189	15.007155	15.2343728
Gglean007085	8.395581982	7.481189	15.007155	15.2343728
Gglean007086	19.11038	21.62691	33.02344	31.11084
Gglean007087	19.11038	21.62691	33.02344	31.11084
Gglean007088	2.54761	2.48659	2.47331	2.26906
Gglean007089	0.0366597	0	0.0299351	0.0719703
Gglean007090	8.523519751	7.926307418	13.20485941	13.52299262
Gglean007091	22.734477	27.6312	20.418052	26.000884
Gglean007092	0	0.143288062	0.336139258	0.862978081
Gglean007093	0	0.143288062	0.336139258	0.862978081
Gglean007094	0	0.143288062	0.336139258	0.862978081
Gglean007095	0	0.143288062	0.336139258	0.862978081
Gglean007096	0	0	0	0
Gglean007097	36.0143	30.6021	25.27618	27.9847
Gglean007098	93.5684	99.9802	52.0413	50.8479
Gglean007099	12.806441	12.23534151	8.809542	8.230452132
Gglean007100	32.30842717	36.65590529	47.64971	52.80573127
Gglean007101	7.1509895	10.510552	13.55732	16.14939
Gglean007102	2.4442773	5.23979	4.132754	5.695237056
Gglean007103	9.47543	8.7652798	4.697740902	8.680814
Gglean007104	9.47543	8.7652798	4.697740902	8.680814
Gglean007105	0.426462	0.683654	0	0
Gglean007106	14.9104	12.9297568	17.894524	24.67869
Gglean007107	14.9104	12.9297568	17.894524	24.67869
Gglean007108	7.49295	4.5708	9.46269	10.4628
Gglean007109	12.7343	7.36254	14.1637	13.1991
Gglean007110	7.58047	5.77977	8.4988	10.4877
Gglean007111	0	0.346207	1.32431	0.630546
Gglean007112	0.690553536	0.849212012	1.6889622	1.543412412
Gglean007113	19.7658	19.34783	29.50680064	30.58704002
Gglean007114	0.923539	2.05545	0.000193021	6.97654
Gglean007115	3.733717502	2.774257	6.095966	7.523704
Gglean007116	20.79719483	20.21222896	43.65001632	43.92411407
Gglean007117	0	0	0.186925	0
Gglean007118	0	0	0	0
Gglean007119	0	0	0	0
Gglean007120	3.019835981	3.910764209	3.261379	4.854418
Gglean007121	22.86865	20.2920622	20.62319	23.064066
Gglean007122	22.86865	20.2920622	20.62319	23.064066
Gglean007123	45.1449	37.8634	19.7307	29.3331
Gglean007124	29.2063	35.7794	25.4328	23.5139
Gglean007125	105.4747535	110.389426	118.4569923	112.9599816
Gglean007126	54.2437	54.6571	56.0051	60.8566
Gglean007127	27.85757552	29.27981605	18.98949333	21.52159

Gglean007128	2.87E-26	1.44E-20	2.53E-16	3.83E-19
Gglean007129	2.91852	2.15888	3.230460035	3.463049
Gglean007130	17.413	16.6385	14.7505	12.6672
Gglean007131	0	0	0	0
Gglean007132	33.5813	33.27934	14.02942	19.54712
Gglean007133	4191.723	3719.997	4355.588	4132.765
Gglean007134	32.9897	33.8463	32.6045	32.2513
Gglean007135	127.972561	153.621668	97.41588	103.67927
Gglean007136	27.7035	20.9246	28.717	29.39708
Gglean007137	10.6186	7.49697	6.66396	5.80881
Gglean007138	32.603976	30.66757	31.09827	30.286519
Gglean007139	22.01682006	18.15860629	25.74545	27.98990007
Gglean007140	3.349824878	3.462852272	3.520862247	4.57943
Gglean007141	29.93488088	37.322424	4.994716512	2.951915
Gglean007142	10.89799223	10.57833561	8.11360422	8.746575537
Gglean007143	10.89799223	10.57833561	8.11360422	8.746575537
Gglean007144	29.57633	33.84842	27.37049	23.12446
Gglean007145	62.65544707	68.76659462	58.5844461	61.85805002
Gglean007146	0	0.269861	0.631814	0.627747687
Gglean007147	5.46E-05	0.202734	0.561051	3.56E-05
Gglean007148	55.64906	56.0015	35.634065	32.741653
Gglean007149	1.009792	1.209455087	0.762166279	0.951134792
Gglean007150	2.54738	1.38207	1.87539	2.87776
Gglean007151	27.69481	31.30518	17.29761	15.97358
Gglean007152	10.410362	11.81165	6.374056523	5.77151
Gglean007153	5.74184	5.46844	2.577762	1.869983
Gglean007154	12.67368688	11.05919724	15.6025715	18.12902215
Gglean007155	0.39893	0.320351	0.174204	0.470264
Gglean007156	1.545412	0.990211	1.053567	1.181278
Gglean007157	1.23451	2.12892	2.81933	2.06451
Gglean007158	12.82804	14.14321	14.53399	16.74313
Gglean007159	3.033331	4.50094	5.33942	10.7706
Gglean007160	2.502228	4.572667	4.041709	6.70495
Gglean007161	0	0	0	0
Gglean007162	32.8997855	35.34202011	47.94315224	50.7723416
Gglean007163	32.8997855	35.34202011	47.94315224	50.7723416
Gglean007164	131.36539	130.78974	196.3859855	224.90379
Gglean007165	131.36539	130.78974	196.3859855	224.90379
Gglean007166	55.13732235	53.86141204	33.7806389	31.51315
Gglean007167	0.504632204	0.984621	1.240109	1.905285
Gglean007168	71.14808	112.57585	135.5426	193.8654
Gglean007169	48.698582	47.31576195	46.39858595	47.72377973
Gglean007170	48.698582	47.31576195	46.39858595	47.72377973
Gglean007171	48.698582	47.31576195	46.39858595	47.72377973

Gglean007172	5.75374	6.11203	6.09744	5.705774
Gglean007173	0	0	0.12708	0
Gglean007174	29.26626691	52.29538806	77.91323212	64.22106765
Gglean007175	6.68951	6.36934	3.11405	3.79139
Gglean007176	1.20826	1.31603	1.03205	1.24771
Gglean007177	0	0	0	0
Gglean007178	0	0	0	0
Gglean007179	0	0	0	0
Gglean007180	3.30215	2.82267	1.98445	2.95944
Gglean007181	0.0854727	0.0870808	0.0683696	0
Gglean007182	0.195159	0.101661	0	0
Gglean007183	0	0	0	0
Gglean007184	0.142952	0.147949	0	0.0685096
Gglean007185	0	0	0	0
Gglean007186	0.376367	0.482672	0.456498	0.588085
Gglean007187	0.0933177	0.143468	0.264115	0.17946
Gglean007188	60.816241	66.893359	52.752147	47.739801
Gglean007189	60.816241	66.893359	52.752147	47.739801
Gglean007190	61.3707007	57.072499	102.571396	125.307341
Gglean007191	17.9366873	19.55403	31.87558	34.46467
Gglean007192	17.9366873	19.55403	31.87558	34.46467
Gglean007193	24.595148	21.63757349	21.40049	18.0422863
Gglean007194	36.379702	32.68663405	43.396439	79.466657
Gglean007195	24.2495	19.0498	16.8234	32.1667
Gglean007196	72.25493	78.96591	81.29334	73.60404
Gglean007197	29.82891174	42.3709738	23.84957149	19.77194403
Gglean007198	17.9245	17.9935	10.4877	8.49175
Gglean007199	39.51691337	41.96099905	18.82386	16.95451
Gglean007200	642.284	813.592	640.101	1245.31
Gglean007201	44.2652	33.7817	42.8072	66.8985
Gglean007202	36.5711	31.5537	18.3305	19.0518
Gglean007203	33.183258	38.109924	8.824465	7.736750617
Gglean007204	146.70785	142.16461	110.20114	102.34493
Gglean007205	32.71867857	31.25047414	34.84682827	33.76906029
Gglean007206	32.71867857	31.25047414	34.84682827	33.76906029
Gglean007207	0	0.084779	0.0167263	0
Gglean007208	281.69205	291.0272803	272.9281901	313.30327
Gglean007209	10.65544586	12.24858119	13.26649	16.47667229
Gglean007210	0	5.73371	0	0
Gglean007211	36.44003908	38.50008881	30.4842	28.21148178
Gglean007212	36.44003908	38.50008881	30.4842	28.21148178
Gglean007213	9.20529	9.2984	6.87433	20.6027
Gglean007214	21.552	19.86385	8.5935	6.99768
Gglean007215	14.74998	14.31594	5.983444905	5.063267707

Gglean007216	118.0886146	113.2117675	118.8750259	136.0887488
Gglean007217	27.27180947	26.02406912	44.62041144	42.1701939
Gglean007218	27.27180947	26.02406912	44.62041144	42.1701939
Gglean007219	27.27180947	26.02406912	44.62041144	42.1701939
Gglean007220	321.52641	382.7290307	506.5018488	566.0717493
Gglean007221	6.90438	8.21435	8.9458	8.81977
Gglean007222	19.363728	14.44025687	6.708679	6.884147
Gglean007223	19.363728	14.44025687	6.708679	6.884147
Gglean007224	19.363728	14.44025687	6.708679	6.884147
Gglean007225	301.648	326.178	319.811	278.175
Gglean007226	23.7441575	24.1413667	22.0260064	20.73245344
Gglean007227	11.01175423	11.59375741	10.3174616	9.970050177
Gglean007228	18.997434	18.11153	15.27749822	14.36938541
Gglean007229	52.35694	51.88550364	42.20816239	42.72546
Gglean007230	0	0	0	0
Gglean007231	6.795372	6.580025	11.16824	12.87839
Gglean007232	24.5014	30.466474	17.657053	17.26751
Gglean007233	1.79837	2.35326	2.509041	2.092295
Gglean007234	2.817956	2.942121	3.89136	4.5352772
Gglean007235	22.39429	22.34685	23.00986	25.144133
Gglean007236	0	0	0	0
Gglean007237	6.9851	7.47778	11.54544	15.20677
Gglean007238	202.768964	233.7410189	176.400858	160.201434
Gglean007239	89.60358	85.652973	59.08137	54.73442
Gglean007240	89.60358	85.652973	59.08137	54.73442
Gglean007241	0	0.115678	0.045419	0
Gglean007242	0	0	0.054696142	0
Gglean007243	0	0	0	0
Gglean007244	18.951786	18.83973043	26.237157	31.278737
Gglean007245	61.79934	58.97461	49.24291	45.60907
Gglean007246	61.79934	58.97461	49.24291	45.60907
Gglean007247	37.78233	42.84612	37.26904	38.77474
Gglean007248	20.27972	20.52755	16.81569	16.99364
Gglean007249	116.87694	143.86456	65.80913	63.71624
Gglean007250	1.16578	0.757633	1.10346	0.55852
Gglean007251	5.06964	1.07492	5.31617	6.12079
Gglean007252	17.48373	22.432	5.308605	4.519214203
Gglean007253	0	0	0	0
Gglean007254	2.58509	2.532513	2.50752	4.69808
Gglean007255	41.68896	48.83359559	42.83529997	78.23088716
Gglean007256	5.14562	11.7055	12.4775	34.2762
Gglean007257	4.511490099	4.698283909	5.80069898	6.917612105
Gglean007258	1.1468	1.595250002	2.01042	2.31178
Gglean007259	1.1468	1.595250002	2.01042	2.31178

Gglean007260	50.19360001	49.56931698	104.2148195	102.7109505
Gglean007261	5.7315288	5.976024667	8.59284	9.192134321
Gglean007262	14.10996484	15.3822283	20.93779813	18.78819193
Gglean007263	59.39171	56.42765	56.32784	58.95552
Gglean007264	15.1785	16.6854	7.72815	6.27587
Gglean007265	191.270615	199.9493038	92.22186291	82.153302
Gglean007266	21.55665628	26.9641176	24.17299785	26.51481381
Gglean007267	32.0151	34.1694	20.41274	18.16144
Gglean007268	0	0	0	0
Gglean007269	0	0	0	0
Gglean007270	0	0	0	0
Gglean007271	0	0	0.0972293	0
Gglean007272	18.121676	20.09049	13.09650141	12.75000081
Gglean007273	20.2404	23.6014	28.3022	34.8123
Gglean007274	12.181298	12.143598	6.883037393	8.036325259
Gglean007275	7.06859	4.980269331	14.76253	16.26156
Gglean007276	136.0173602	152.038042	98.82895414	83.76037008
Gglean007277	41.22124801	46.6898414	41.69141286	45.61076634
Gglean007278	0.0838693	0.0576968	0	0.0267311
Gglean007279	6.01791	4.8001637	7.64542	8.78911
Gglean007280	29.4128	28.1514	26.6104	25.1318
Gglean007281	10.61867763	10.861295	8.2270673	6.919288
Gglean007282	23.590433	27.63419885	41.97147601	35.175205
Gglean007283	2.35087	2.451999707	4.4318807	4.03886
Gglean007284	10.18854543	10.91706	12.35873605	14.474594
Gglean007285	48.4589	47.2765	32.77777	30.33426
Gglean007286	11.131622	10.826916	4.379403	3.639530679
Gglean007287	35.939201	37.976666	41.19500729	65.41617479
Gglean007288	27.8661718	28.17328957	25.48220152	25.81118894
Gglean007289	20.692736	22.61150279	16.514404	14.385714
Gglean007290	19.01711	19.15038	19.21113	18.04475
Gglean007291	89.64277	103.61271	37.48184	25.8639
Gglean007292	0.255757	0.326772	0	0
Gglean007293	0	0	0	0.276565
Gglean007294	0	0	0	0
Gglean007295	0	0	0	0.312543
Gglean007296	0.459918392	0.375962051	0.576132268	0.529566864
Gglean007297	0	0	0	0
Gglean007298	0	0	0	0
Gglean007299	0	0	0	0
Gglean007300	0	0	0	0
Gglean007301	0.156404	0	0	0
Gglean007302	4.91798	4.13278	2.99071	2.323371
Gglean007303	32.1819	38.131	11.80363	9.96655

Gglean007304	16.27212	17.3119	19.73232	23.42568
Gglean007305	14.95424	15.26481	16.92839	17.67251
Gglean007306	136.253461	198.4813854	143.3121048	128.302603
Gglean007307	136.253461	198.4813854	143.3121048	128.302603
Gglean007308	0	0.3508453	0	0.034554181
Gglean007309	1.160327461	1.333520303	1.541587009	1.991714767
Gglean007310	3.025000748	3.779456685	6.415169	6.389168623
Gglean007311	0	0	0	0
Gglean007312	391.695	249.727	462.695	552.617
Gglean007313	6.735385	6.7616875	8.141899306	12.885194
Gglean007314	0	0	0	0
Gglean007315	0	0	0	0
Gglean007316	1.26993	1.0227	1.75524	2.55379
Gglean007317	11.336262	14.802894	12.80798	10.98437
Gglean007318	0.77991	0.618311	0	0.377686
Gglean007319	24.64979372	23.34964179	16.91876514	16.7565436
Gglean007320	12.260724	10.005367	11.00316	12.84924
Gglean007321	1.144779	1.077893	1.717985	1.763262
Gglean007322	1.144779	1.077893	1.717985	1.763262
Gglean007323	6.24362	5.25417	4.68226	3.44755
Gglean007324	62.7008	83.885	98.7002	83.3003
Gglean007325	114.0543238	115.39035	44.188347	34.15787423
Gglean007326	15.95432	15.16651	11.81852	15.99418
Gglean007327	61.1108359	70.514951	76.37087061	87.62718
Gglean007328	16.46962	17.58379	31.98862	33.01383
Gglean007329	0	0	0	0
Gglean007330	16.46962	17.58379	31.98862	33.01383
Gglean007331	31.9603	27.53374	30.4595	34.4556
Gglean007332	32.930631	37.18869768	39.70500483	39.13787201
Gglean007333	32.930631	37.18869768	39.70500483	39.13787201
Gglean007334	7.98344	6.17699	8.90503	10.3306
Gglean007335	10.63486	12.24395	3.47706	3.36856
Gglean007336	26.59271873	26.39267929	42.79863926	46.6651897
Gglean007337	10.33552405	9.207369	5.51063891	7.081611
Gglean007338	10.33552405	9.207369	5.51063891	7.081611
Gglean007339	0	0	0	0
Gglean007340	21.071642	20.824434	28.038757	34.292351
Gglean007341	0	0	0	0
Gglean007342	3.955086	4.395075676	2.832534213	4.86833
Gglean007343	3.955086	4.395075676	2.832534213	4.86833
Gglean007344	58.86303	59.75337	42.80998658	39.68401
Gglean007345	3.2174	1.74026	2.72955	3.72373
Gglean007346	29.047693	27.937147	32.926666	26.983439
Gglean007347	29.047693	27.937147	32.926666	26.983439

Gglean007348	29.047693	27.937147	32.926666	26.983439
Gglean007349	10.427829	9.912805	4.609620582	3.911286
Gglean007350	3.498521	3.549570756	3.830908695	5.871069
Gglean007351	27.2705	30.0286	19.74677	20.61812
Gglean007352	12.22083893	12.13866865	18.94689776	21.47062959
Gglean007353	0.209424	2.021524633	1.468717166	0.815141
Gglean007354	0	0	0.662083	0
Gglean007355	55.52865475	50.07519784	86.95597014	97.13632776
Gglean007356	1.32750321	2.785389	3.131754	3.55820872
Gglean007357	24.04606507	33.03484044	36.99292723	44.0479368
Gglean007358	68.45050539	76.1978041	97.47570867	120.9310105
Gglean007359	76.70204	71.45124	59.75591	59.50303
Gglean007360	45.05902	43.46456	21.16261	22.12386
Gglean007361	16.05551	14.64994	10.485196	10.386539
Gglean007362	0	1.38114	2.57577	1.2367
Gglean007363	54.5637	43.1851	2.51798	0.70599
Gglean007364	0.768894251	0.813024399	0.5658153	0.427319689
Gglean007365	23.20395704	20.08820019	31.7042168	32.59928687
Gglean007366	0	0	1.252662	0.112817
Gglean007367	5.014446217	4.646128	8.34293186	9.80634306
Gglean007368	2.720422255	3.373791062	3.165488	2.761495456
Gglean007369	2.720422255	3.373791062	3.165488	2.761495456
Gglean007370	6.20568	5.26855	0.967742	4.90785
Gglean007371	0.220063	0	0.7175	0.638802
Gglean007372	114.0771709	122.9125611	111.648623	106.745483
Gglean007373	0	0	0	0
Gglean007374	1.17433	0.9169	0.830521	1.27029
Gglean007375	0	0	0	0
Gglean007376	0.0528388	0.0538534	0	0
Gglean007377	0.391707	0	0	0
Gglean007378	1.86873	2.03769	3.21108	2.51958
Gglean007379	0	0	0	0
Gglean007380	0	0.0101321	0	0.00952468
Gglean007381	4.163052	6.100435	3.368557	2.270388
Gglean007382	0	0	0	0
Gglean007383	0.0368597	0	0	0
Gglean007384	0.111565	0.0829179	0.368287	0.124795
Gglean007385	0	0	0	0
Gglean007386	0	0	0	0
Gglean007387	0	0	0	0
Gglean007388	0	0.49826	0	0
Gglean007389	0	0	0	0.071011
Gglean007390	0.117755	0.368693	0	0
Gglean007391	7.06E-08	0.109743	7.88E-08	5.96E-08

Gglean007392	8.42704	8.9541	12.80469	13.57819
Gglean007393	43.7332	40.5973	43	40.6573
Gglean007394	27.16756	26.43451375	35.2594242	40.9559972
Gglean007395	0	0	0	0
Gglean007396	0	0	0.372035	0
Gglean007397	4.736386795	4.55305676	5.945906643	6.409110706
Gglean007398	4.736386795	4.55305676	5.945906643	6.409110706
Gglean007399	0	0	0	0.156018
Gglean007400	186.0766072	166.9094369	129.9206087	116.35447
Gglean007401	186.0766072	166.9094369	129.9206087	116.35447
Gglean007402	0	0	0	0
Gglean007403	14.005535	12.236985	13.30609	16.25037
Gglean007404	19.77679609	17.9086499	16.064607	15.07487773
Gglean007405	14.24874	13.23413	10.06579	8.5881
Gglean007406	4.937237	4.743217	3.463309	4.04238
Gglean007407	0	0	0	0
Gglean007408	0	0	0	0
Gglean007409	1.32363	1.59123	0.7486804	0.547477067
Gglean007410	1.32363	1.59123	0.7486804	0.547477067
Gglean007411	46.1758524	51.90649062	22.13163291	18.98221794
Gglean007412	33.63502775	34.93699621	8.411177019	6.564864434
Gglean007413	33.63502775	34.93699621	8.411177019	6.564864434
Gglean007414	33.63502775	34.93699621	8.411177019	6.564864434
Gglean007415	25.409759	27.247828	18.969913	18.17820502
Gglean007416	8.576362563	6.852661785	12.511374	10.8902377
Gglean007417	0	0	0	0
Gglean007418	10.03409	9.094827	10.544169	13.083626
Gglean007419	29.4468	33.21849719	27.73827137	28.02628723
Gglean007420	376.054	276.132	706.95301	781.945
Gglean007421	0.400023839	0.416498414	0.367318647	0.240809761
Gglean007422	33.24490049	35.87529736	20.1478488	18.69581128
Gglean007423	0.446226	0.526407836	0.393454935	0.467580202
Gglean007424	0	0	0	0
Gglean007425	96.9585	107.505	258.9714	253.5038
Gglean007426	0.793614	1.124078	0.888182267	0.994442
Gglean007427	21.438156	22.25537	26.16081	28.25406
Gglean007428	96.54799853	85.80564	48.88083269	62.74117
Gglean007429	37.60430442	39.03152561	52.48698436	53.63667534
Gglean007430	37.60430442	39.03152561	52.48698436	53.63667534
Gglean007431	279.6285816	234.8224142	174.0749774	161.4318099
Gglean007432	279.6285816	234.8224142	174.0749774	161.4318099
Gglean007433	6.36084	5.67919	12.5553	9.67977
Gglean007434	33.92282	36.00629	13.45855	11.46213
Gglean007435	97.54335	103.50889	49.73281	43.1923

Gglean007436	7.22373379	8.195998	9.615058881	11.92796079
Gglean007437	74.54072	77.7879	71.971	68.18579
Gglean007438	17.81088	21.20875	18.85637	18.72463
Gglean007439	0.956252	1.44976	0.212421	30.496655
Gglean007440	19.198324	21.287701	33.191843	30.496655
Gglean007441	14.2343163	11.02179933	17.30645734	21.1114559
Gglean007442	14.2343163	11.02179933	17.30645734	21.1114559
Gglean007443	1107.55	965.959	1053.8	1628.78
Gglean007444	2.68936	1.078412297	2.797601	4.90374
Gglean007445	0	0	0	0
Gglean007446	11.274198	14.62585106	22.395165	22.78099707
Gglean007447	1.54179	1.93992	2.45874	3.51392
Gglean007448	69.23210002	57.59805021	86.74412	101.28588
Gglean007449	0	0	0	0
Gglean007450	0	0	0	0
Gglean007451	69.23210002	57.59805021	86.74412	101.28588
Gglean007452	6.552491	8.09935	6.189501	6.000981
Gglean007453	2232.5272	1924.4043	2263.9781	3136.1485
Gglean007454	207.63	181.299	171.766	257.599
Gglean007455	3.132015	2.923340019	3.7888527	4.091280039
Gglean007456	585.3307169	481.069016	522.228411	763.9368125
Gglean007457	3.166143909	3.036889	7.051459	7.593861
Gglean007458	19.198324	21.287701	33.191843	30.496655
Gglean007459	27.6812	25.9889	23.2211	20.6609
Gglean007460	72.8206067	77.9081027	41.0368798	31.42980955
Gglean007461	1.4615	0.000521246	0.862499	0.691713
Gglean007462	0.31940841	0.309039453	0.081271987	0.504212154
Gglean007463	0.270063495	0.425305188	1.124653	1.571481
Gglean007464	53.2601	58.0085	33.4334	27.1571
Gglean007465	107.0598515	109.3060052	157.4357316	181.9919938
Gglean007466	107.0598515	109.3060052	157.4357316	181.9919938
Gglean007467	10.34065161	10.782734	11.00951078	12.8424
Gglean007468	10.34065161	10.782734	11.00951078	12.8424
Gglean007469	10.42453	10.62162	14.644447	19.242429
Gglean007470	10.42453	10.62162	14.644447	19.242429
Gglean007471	774.287705	825.227792	522.22093	493.97348
Gglean007472	774.287705	825.227792	522.22093	493.97348
Gglean007473	13.32689	16.33364	10.5848	10.10264
Gglean007474	0.495244	0.806966	0.0590291	8.88E-11
Gglean007475	0.396164	0.152589	0.489038	0.290048
Gglean007476	0	0	0	0
Gglean007477	14.35325375	12.66859921	18.47133542	22.51604923
Gglean007478	120.5076	120.132	81.387	71.6917
Gglean007479	8.214623	8.81754	12.26547	14.63089

Gglean007480	129.7769779	156.324034	117.4331341	101.781399
Gglean007481	319.09521	341.29694	298.08942	269.62358
Gglean007482	16.83692	17.85168811	26.25243	29.89068
Gglean007483	99.90966145	98.89932603	73.49170193	77.24375724
Gglean007484	99.90966145	98.89932603	73.49170193	77.24375724
Gglean007485	20.699332	24.512452	22.585059	23.649501
Gglean007486	3.918134	3.040490299	6.15498	7.105971568
Gglean007487	64.64707	66.20488	63.81665	64.02939
Gglean007488	64.64707	66.20488	63.81665	64.02939
Gglean007489	64.64707	66.20488	63.81665	64.02939
Gglean007490	0	0	0	0
Gglean007491	2.51594	2.86438	2.84038	4.12397
Gglean007492	19.47693	20.01383	7.93832	6.56145
Gglean007493	6.89251	6.49604	4.32456	4.98058
Gglean007494	222.14	254.777	117.374	60.1486
Gglean007495	1633.9199	1739.9515	1132.0651	1112.3914
Gglean007496	14.1770628	22.17163616	6.496442419	5.41744653
Gglean007497	14.1770628	22.17163616	6.496442419	5.41744653
Gglean007498	47.0191	43.0169	36.7554	31.9514
Gglean007499	84.617946	78.65392443	113.6479623	112.262252
Gglean007500	0	0	0	0
Gglean007501	0	0	0	0
Gglean007502	56.6049	58.6472	45.6369	46.09602
Gglean007503	0.0825092	0.173085	0.201664	0.874559
Gglean007504	0	0	0	0
Gglean007505	0	0	0	0
Gglean007506	13.79776	15.48221	9.51647	7.07845
Gglean007507	0	0	0	0.461485456
Gglean007508	0	0	0	0
Gglean007509	24.37013	23.76909252	33.10112771	38.88695
Gglean007510	0	0	0	0
Gglean007511	0.0390392	0	0	0
Gglean007512	0	0	0	0
Gglean007513	0	0	0	0
Gglean007514	0	0	0	0
Gglean007515	11.8156	9.13479	3.1014	1.92581
Gglean007516	0.234579	0.120462	0.571258	0.45356
Gglean007517	0	0.243674	0	0
Gglean007518	0	0	0.24414	0
Gglean007519	0	0	0	0
Gglean007520	0	0	0	0
Gglean007521	0	0	0	0
Gglean007522	0	0	0	0
Gglean007523	0	0	0	0

Gglean007524	0	0	0	0
Gglean007525	0	0	0	0
Gglean007526	0	0	0	0
Gglean007527	0	0	0	0
Gglean007528	0	0	0	0.167293
Gglean007529	0	0	1.39786	0
Gglean007530	0	0	0	0
Gglean007531	0	0	0	0
Gglean007532	22.83022	29.39989	41.08486	49.32854
Gglean007533	36.14494493	41.904087	28.149469	30.40906
Gglean007534	9.38285	8.52865	7.76133	6.68477
Gglean007535	27.55384	31.81961	16.36073	13.30975
Gglean007536	120.7128937	118.49236	182.8099965	197.184813
Gglean007537	0	0	0	0
Gglean007538	12.2286098	12.2526903	20.6107616	26.3347239
Gglean007539	12.2286098	12.2526903	20.6107616	26.3347239
Gglean007540	0	0	0	0
Gglean007541	27.286984	29.45925	28.80393582	28.545403
Gglean007542	59.13281227	65.59961184	48.47513024	42.96660425
Gglean007543	82.6967	37.3615	85.1665	137.886
Gglean007544	4.609994	5.12859	4.92964	6.65533
Gglean007545	0	0	0	0
Gglean007546	3.684168	3.781024	3.78699	5.473760933
Gglean007547	3.684168	3.781024	3.78699	5.473760933
Gglean007548	0	0	0	0
Gglean007549	0.347201	0	0	0
Gglean007550	5.530558	4.895513	8.77389	8.88486
Gglean007551	5.530558	4.895513	8.77389	8.88486
Gglean007552	0	0	0	0
Gglean007553	0	0	0	0
Gglean007554	0.890295	0	0	0.289737
Gglean007555	24.4039239	24.04413491	32.36059611	41.86400708
Gglean007556	30.1074	21.8105	40.0057	50.4597
Gglean007557	20.71033	22.9634	28.6442	39.9876
Gglean007558	2.43405	5.24885	3.88999	1.70444
Gglean007559	0	0	0.608575	0
Gglean007560	0	0	0	0
Gglean007561	3.13808	6.82548	1.87706	1.25732
Gglean007562	102.716202	96.371662	132.84794	131.130244
Gglean007563	0.122124	0.0919281	7.74E-06	0.150377
Gglean007564	0.257937	0.413867209	0.666897	0.852084
Gglean007565	0.619965	1.33423	1.0266	1.83427
Gglean007566	3.82729612	3.6607151	5.0448508	6.34053505
Gglean007567	1284.621449	1314.625638	861.4997359	794.3046373

Gglean007568	17.11089635	17.59620209	16.6805427	16.10634389
Gglean007569	39.8938	39.8752	37.9297	42.6139
Gglean007570	39.01996178	39.9115367	50.1606295	48.15555795
Gglean007571	14.683395	14.419082	11.932789	12.508873
Gglean007572	34.59393	38.96628	43.90425	38.55527821
Gglean007573	1.27744361	1.336051301	1.356166779	1.853400453
Gglean007574	0	0.305211	0.235346	0
Gglean007575	63.6747	66.4879	44.3506	40.4666
Gglean007576	22.7363	39.6526	43.89614	59.9087
Gglean007577	59.2654546	49.17858313	47.18171711	67.62001221
Gglean007578	59.2654546	49.17858313	47.18171711	67.62001221
Gglean007579	0	0	0	0
Gglean007580	21.46328955	21.41930424	23.6497353	26.6896169
Gglean007581	3.835216145	3.097663503	6.054873128	6.862089791
Gglean007582	7.296355	2.101937	2.897661513	1.0554609
Gglean007583	7.296355	2.101937	2.897661513	1.0554609
Gglean007584	2.247278001	4.709620867	0.589860982	0.401186671
Gglean007585	46.85761247	49.241295	20.85717495	16.58293571
Gglean007586	0	0	0.066790688	0
Gglean007587	0	0	0	0
Gglean007588	0	0	0	0
Gglean007589	0.356021	0	0	0.178689392
Gglean007590	0	0	0	0
Gglean007591	0	0	0	0
Gglean007592	0	0	0	0
Gglean007593	47.73524	54.76697	39.54037	32.38002
Gglean007594	0	0	0	0
Gglean007595	3.33718	1.64628	4.41561	2.52552
Gglean007596	0	0	0	0
Gglean007597	9.14626	12.2591	3.20436	2.27116
Gglean007598	0	0.159528	0.124057	0
Gglean007599	4.094871639	3.632672002	2.984356019	3.588059
Gglean007600	0	0	1.4038	0
Gglean007601	0.0910576	0.282564	0.225379	0.268207
Gglean007602	0	0	0	0
Gglean007603	0	0	0	0
Gglean007604	0.102374	0.0534138	0	0.097518
Gglean007605	0	0	0	0
Gglean007606	69.05904002	60.21321	51.255121	54.04087281
Gglean007607	69.05904002	60.21321	51.255121	54.04087281
Gglean007608	69.05904002	60.21321	51.255121	54.04087281
Gglean007609	7.083742887	7.130458441	11.2662	11.058255
Gglean007610	24.41110143	34.157119	38.758514	28.6341048
Gglean007611	0.802933	3.29509	1.09973	0.171282

Gglean007612	0	0	0	0
Gglean007613	0	0	0	0
Gglean007614	0	0	0	0
Gglean007615	0.157561	0.0921874	0.108963	0.107491
Gglean007616	0	0.23509	0	0
Gglean007617	16.29075309	19.7926205	27.1650993	29.07805078
Gglean007618	16.29075309	19.7926205	27.1650993	29.07805078
Gglean007619	37.30276	37.395236	44.56994	48.87284
Gglean007620	37.30276	37.395236	44.56994	48.87284
Gglean007621	7.37976	3.54675	9.07114	11.40713
Gglean007622	16.67458	11.661	13.92247	14.20901
Gglean007623	3.835216145	3.097663503	6.054873128	6.862089791
Gglean007624	3.835216145	3.097663503	6.054873128	6.862089791
Gglean007625	25.99170574	28.14138003	34.516759	39.44011992
Gglean007626	9.079097088	12.87044756	14.16072087	16.51829316
Gglean007627	9.079097088	12.87044756	14.16072087	16.51829316
Gglean007628	20.773541	21.985914	26.53452	25.11522
Gglean007629	14.48766857	14.03986054	28.42381766	22.90686689
Gglean007630	66.62630626	67.8628099	117.169745	126.7185004
Gglean007631	0.680787	0	0	0
Gglean007632	0.182072165	0.12469941	0.18700923	0.116381627
Gglean007633	7.499791252	7.935813479	8.447718295	8.65991404
Gglean007634	41.10274	41.03731	38.65184	41.27893
Gglean007635	8.27146	6.86113	10.7612	11.4546
Gglean007636	73.7788	80.47404	39.81879	39.5214
Gglean007637	186.3707226	210.145597	143.88236	135.35418
Gglean007638	0.510542167	0.691155249	0.710275812	0.625096216
Gglean007639	0	0	0	0
Gglean007640	5.5785	6.17293	6.56784	7.29196
Gglean007641	0.298009096	0.508880005	0.364905	0.549124277
Gglean007642	30.303383	35.86017911	13.84160844	13.08237675
Gglean007643	30.303383	35.86017911	13.84160844	13.08237675
Gglean007644	30.303383	35.86017911	13.84160844	13.08237675
Gglean007645	0.467783	0.29522	0.924625	0.547294
Gglean007646	1.12E-11	3.32554	9.66E-06	3.32794
Gglean007647	36.816	43.4699	29.9648	33.7323
Gglean007648	0	0	0	2.03E-25
Gglean007649	9.516601469	10.43483	15.021706	14.193515
Gglean007650	9.516601469	10.43483	15.021706	14.193515
Gglean007651	5.02247	5.12786	8.59039	9.97064
Gglean007652	11.680102	11.248652	8.539513	8.198239
Gglean007653	0	0	0.103627	0
Gglean007654	10.67955	10.60962	19.52715	20.64439
Gglean007655	0	0	0.111618	0.403772

Gglean007656	0	0	0	0
Gglean007657	1.29794	1.26897	0.704001	0.729241
Gglean007658	9.16421	8.84974	7.54584	6.15991
Gglean007659	22.1109113	26.558646	10.7702087	9.716427
Gglean007660	22.1109113	26.558646	10.7702087	9.716427
Gglean007661	11.0885	9.84551	5.4113	6.50255
Gglean007662	7.48722	7.48765	13.6446	11.766
Gglean007663	22.1109113	26.558646	10.7702087	9.716427
Gglean007664	5.76331	7.564693	8.937605	8.18244642
Gglean007665	16.31742	20.99957	24.91463	34.71095
Gglean007666	19.08203656	17.42911371	26.1807883	27.35220355
Gglean007667	19.08203656	17.42911371	26.1807883	27.35220355
Gglean007668	8.1844225	5.85673621	10.13920019	8.42193833
Gglean007669	8.1844225	5.85673621	10.13920019	8.42193833
Gglean007670	9.051106727	9.21955294	12.47687309	15.06711818
Gglean007671	0	0	0	0
Gglean007672	65.739279	58.8865	91.99392648	120.15741
Gglean007673	95.6431	91.86446689	94.60033	136.6574123
Gglean007674	95.6431	91.86446689	94.60033	136.6574123
Gglean007675	1.733246	2.031164	3.11649	3.52685
Gglean007676	8.096054	8.833002	4.0288289	4.34495
Gglean007677	0	0	0	0
Gglean007678	2.799242	2.872906	3.42827	3.89367
Gglean007679	0	0	0	0
Gglean007680	1.415465261	1.360065888	0.940690124	0.947482012
Gglean007681	0.142421	0.250693	0.313174	0.312868
Gglean007682	0.487899	0.603045	0.649535	0.832336
Gglean007683	0	0.110639	0.256976	0.102114
Gglean007684	0	0	0	0
Gglean007685	1.41228914	2.001474858	1.018760235	0.942770307
Gglean007686	2.54735438	2.05859583	2.370122	1.31961977
Gglean007687	2.54735438	2.05859583	2.370122	1.31961977
Gglean007688	2.54735438	2.05859583	2.370122	1.31961977
Gglean007689	2.54735438	2.05859583	2.370122	1.31961977
Gglean007690	0.316775	0.484145	0.470039	0.607977
Gglean007691	2.0242635	2.143894023	1.65639927	0.83877
Gglean007692	2.0242635	2.143894023	1.65639927	0.83877
Gglean007693	0	0	0	0
Gglean007694	98.132374	108.643555	45.568265	34.515672
Gglean007695	98.132374	108.643555	45.568265	34.515672
Gglean007696	20.21907	20.9768019	25.43501	23.48182
Gglean007697	2.7288842	2.562839	3.253860324	3.871879
Gglean007698	0	0	0	0
Gglean007699	14.94311906	14.91026332	11.09350023	9.701770179

Gglean007700	21.16602624	22.15841462	18.852687	15.79853433
Gglean007701	0.2633512	0.1124675	0.3413278	0.549018011
Gglean007702	0.2633512	0.1124675	0.3413278	0.549018011
Gglean007703	208.55987	187.02191	258.040092	330.60912
Gglean007704	0	0	0	0
Gglean007705	19.593218	17.39973901	19.87799689	18.95877
Gglean007706	1.936625278	1.249736615	0.702925881	0.75737117
Gglean007707	1.936625278	1.249736615	0.702925881	0.75737117
Gglean007708	36.63508407	37.71219754	20.6337275	17.1046979
Gglean007709	22.5121	24.56735	16.89509	14.49561
Gglean007710	137.675	137.612	38.4667	34.8308
Gglean007711	304.3743858	296.24286	264.0152777	219.4355894
Gglean007712	304.3743858	296.24286	264.0152777	219.4355894
Gglean007713	38.59564957	42.090918	33.77522	39.59876253
Gglean007714	123.63868	143.23203	99.8062	95.7404
Gglean007715	123.63868	143.23203	99.8062	95.7404
Gglean007716	0.91736	6.877986	5.026731	2.47849
Gglean007717	0.93793	1.5651	1.69076	3.29067
Gglean007718	17.80608632	16.80368849	30.801274	36.838641
Gglean007719	17.80608632	16.80368849	30.801274	36.838641
Gglean007720	23.19666246	23.36968525	27.113409	24.9791619
Gglean007721	0	0	0	0
Gglean007722	2.828374144	2.994749359	5.117678558	5.278701425
Gglean007723	1.346396	9.138783713	3.626322834	5.130623
Gglean007724	0	0	0	0
Gglean007725	1.44651	1.73467	1.01697	1.41366
Gglean007726	0	0.0442979	0.0345171	0.0407826
Gglean007727	0	0.700392	0	0
Gglean007728	22.8981	25.96739	5.88253	5.7025
Gglean007729	380.876668	404.5626451	539.5091973	705.2580712
Gglean007730	0.927034	1.015280435	1.26389023	1.675020039
Gglean007731	1.697247058	2.285404431	3.483124632	4.174138799
Gglean007732	28.78618981	26.24222225	37.31550706	42.3017527
Gglean007733	12.45556282	13.44040182	9.197147621	7.243697874
Gglean007734	28.19797	27.70038	15.11874	12.35052
Gglean007735	28.19797	27.70038	15.11874	12.35052
Gglean007736	30.274333	43.873378	39.56338	41.32385
Gglean007737	30.274333	43.873378	39.56338	41.32385
Gglean007738	30.274333	43.873378	39.56338	41.32385
Gglean007739	1079.4834	1131.2627	781.4733	780.2348
Gglean007740	0	0.363242	0	0
Gglean007741	25.46773	25.26024948	26.56743479	26.50697468
Gglean007742	0	0	0	0
Gglean007743	11.61263	11.0507	5.70616	5.62038

Gglean007744	1409.946	1403.095681	904.08335	835.6532836
Gglean007745	123.3540839	122.6134	61.7209158	53.931594
Gglean007746	123.3540839	122.6134	61.7209158	53.931594
Gglean007747	46.51770137	41.19820191	28.81530077	22.43870021
Gglean007748	0	0	0	0
Gglean007749	0	0	0	0
Gglean007750	0.080726413	0.0655802	0	0
Gglean007751	0.052803	0.0548062	0.401699	0.151695
Gglean007752	3.9650469	2.208395345	14.15778	8.590891931
Gglean007753	0	0	0	0
Gglean007754	8.62497	8.273162	12.88031	13.18411
Gglean007755	69.3177064	70.35143286	86.44156544	93.5040711
Gglean007756	69.3177064	70.35143286	86.44156544	93.5040711
Gglean007757	26.31561639	27.65486903	24.74352222	23.09390197
Gglean007758	37.95703	37.56311	42.07511	38.20204
Gglean007759	0	0.458268	0.0722757	0.257601
Gglean007760	29.57790706	27.47236	15.871963	15.631568
Gglean007761	0.493558904	0.538476	1.480878	1.495036
Gglean007762	190.4360311	192.9836	131.4177309	114.6896894
Gglean007763	190.4360311	192.9836	131.4177309	114.6896894
Gglean007764	0.884389	0.899024	1.2473423	1.362396
Gglean007765	0.884389	0.899024	1.2473423	1.362396
Gglean007766	0.884389	0.899024	1.2473423	1.362396
Gglean007767	0.138419085	0.22369341	0.535519	0.2057685
Gglean007768	11.12705	8.90966	5.159970357	5.591070739
Gglean007769	16.3702795	14.973245	18.06403	15.80221836
Gglean007770	43.59566	44.59528	39.338104	36.517111
Gglean007771	37.08011	35.37231	26.49618	22.80852
Gglean007772	1.10674	1.17105	1.53376	1.78498
Gglean007773	19.657487	19.03582	25.48004	26.441049
Gglean007774	19.657487	19.03582	25.48004	26.441049
Gglean007775	111.637	106.749	62.0683	66.4976
Gglean007776	11.36020335	10.1656593	11.591546	12.50751578
Gglean007777	49.544	39.536	50.0314	70.269
Gglean007778	8.003337011	8.802665215	11.60307	15.20104832
Gglean007779	32.28322199	28.82951157	29.60591399	31.59402
Gglean007780	32.28322199	28.82951157	29.60591399	31.59402
Gglean007781	29.014051	27.773101	42.09844	51.396143
Gglean007782	54.2412	47.8891	38.1431	38.5506
Gglean007783	29.69801341	32.77365254	46.713076	53.74002196
Gglean007784	29.69801341	32.77365254	46.713076	53.74002196
Gglean007785	29.69801341	32.77365254	46.713076	53.74002196
Gglean007786	29.69801341	32.77365254	46.713076	53.74002196
Gglean007787	5.638936	6.06226	5.037463	4.392354023

Gglean007788	162.34579	140.78889	172.85384	167.52278
Gglean007789	70.91593	69.28808	89.65568	84.87838
Gglean007790	70.91593	69.28808	89.65568	84.87838
Gglean007791	1.581411376	1.423706	2.301453465	2.639775
Gglean007792	0	0	0.0398907	0.0945922
Gglean007793	2.844078	1.810467622	2.815064	3.602395
Gglean007794	0.274322826	0.639639974	0.60616004	0.599108015
Gglean007795	0.279177	0.371032	1.00304	0.885172
Gglean007796	0	0	0	0
Gglean007797	0.507173	0.715418	1.01296	1.20227
Gglean007798	2.910026885	3.507263	3.109832	3.994012
Gglean007799	16.6965	18.4039	14.4696	13.9159
Gglean007800	30.05446004	32.27495044	34.52683654	35.874441
Gglean007801	3.670176	5.20799	5.735070005	6.594220308
Gglean007802	3.670176	5.20799	5.735070005	6.594220308
Gglean007803	0.540842	0.079148	5.67E-25	0.000446619
Gglean007804	29.7626	32.6824	39.8282	36.0841
Gglean007805	1237.103	1295.271	813.148	781.395
Gglean007806	9.78251	8.769685	10.298889	11.433682
Gglean007807	39.60574352	41.9746159	29.83600147	29.77005011
Gglean007808	0.911591986	1.2638427	0.90229015	0.877264929
Gglean007809	1.68E-50	1.94E-49	1.09E-42	1.13E-23
Gglean007810	7.09357567	6.222248	5.571246	4.077036464
Gglean007811	18.09259	14.17541	11.81245	10.62222
Gglean007812	4.377104	5.7653	8.84016	10.13356
Gglean007813	22.40163	22.98201	16.91582	18.63563
Gglean007814	6.31393	7.35919	3.43383	3.17075
Gglean007815	250.0758	205.836	280.0639	257.5905
Gglean007816	15.5043	14.0681	13.8	20.1072
Gglean007817	53.75455	62.58793	44.1317	48.77406
Gglean007818	0	0	0	0
Gglean007819	0	0	0	0
Gglean007820	61.8144	57.005	29.0414	22.94182
Gglean007821	0.14887	0	0	0
Gglean007822	2.761663	1.992611813	2.862152202	3.599009
Gglean007823	2.861191958	2.419907303	2.828656833	2.903912514
Gglean007824	2.861191958	2.419907303	2.828656833	2.903912514
Gglean007825	0.110571	0.0572279	0.0450669	0
Gglean007826	0	0	0	0
Gglean007827	30.3999096	26.3807469	29.73308	29.13652354
Gglean007828	30.3999096	26.3807469	29.73308	29.13652354
Gglean007829	1.67576	0.512673	1.76191	1.63137
Gglean007830	0	0.412277	0	0.74935
Gglean007831	0	0	0	0

Gglean007832	16.50052	26.53003	37.5791589	38.65777574
Gglean007833	19.34459	24.87377	13.145952	16.5351
Gglean007834	61.89047	67.32204	87.27628	109.08681
Gglean007835	31.694	33.3566	40.9755	47.8713
Gglean007836	15.8737345	15.68717177	17.10256782	16.68496828
Gglean007837	12.66058	9.784749	13.271832	16.0415
Gglean007838	28.02547	24.817211	27.07086	24.88191
Gglean007839	66.02760964	64.42529665	24.90009069	19.08217446
Gglean007840	0.0644054	0.0647391	0.103272093	0.061561977
Gglean007841	0	0	0.106816448	0.600980994
Gglean007842	0	0	0.106816448	0.600980994
Gglean007843	0	0	0	0
Gglean007844	0	0	0	0
Gglean007845	21.465153	21.60197619	16.83364275	15.75811689
Gglean007846	34.124	35.8804	31.5953	31.1122
Gglean007847	5.834934	4.425980229	6.997778032	7.01147
Gglean007848	0.373385	0.198549	0.622007	0.741675
Gglean007849	15.13327	15.78864	13.412318	14.605941
Gglean007850	0	0	0.0579951	0
Gglean007851	0	0	0	0
Gglean007852	8.095851	9.617996	4.087563	3.058549
Gglean007853	0.2358314	0.1252059	0.265213	0.17125
Gglean007854	0	0	0	0.138488
Gglean007855	0	0	0	0
Gglean007856	0.147586	0.0761436	0	0
Gglean007857	0.262324	0.184554	0	0.0851981
Gglean007858	0	0	0	0
Gglean007859	25.9625	23.8335	16.6711	21.2735
Gglean007860	12.52219894	11.88881749	5.625021052	4.735108905
Gglean007861	2.56504	1.97385	2.09933	1.01858
Gglean007862	4.94281	4.69505	3.57676	3.23295
Gglean007863	0	0	0	0.079049
Gglean007864	0	0	0	0
Gglean007865	0	0	0	0
Gglean007866	0	0	0	0
Gglean007867	0.227572	0	0	0.072645
Gglean007868	0.0813667	0	0	0
Gglean007869	0.0813667	0	0	0
Gglean007870	0.0813667	0	0	0
Gglean007871	0.0813667	0	0	0
Gglean007872	0	0	0	0
Gglean007873	0	0	0	0
Gglean007874	0.0875542	0	0	0
Gglean007875	0.0742292	0.15449	0.060079	0.214362

Gglean007876	0	0	0	0
Gglean007877	0.299836588	0.405713	0.410587	0.433820826
Gglean007878	0.299836588	0.405713	0.410587	0.433820826
Gglean007879	0.949329	0.458995	1.01588	1.7393
Gglean007880	36.62874	37.68754	41.79316	41.05013
Gglean007881	66.5817	69.7034157	92.3147	91.7512
Gglean007882	1.954246446	1.0536	3.46572	3.460524
Gglean007883	0.4970301	0.850295399	1.52867	1.529425
Gglean007884	0	0	0	0
Gglean007885	32.84891511	30.1105531	21.0565335	19.96805853
Gglean007886	34.64304722	35.41736425	47.99146867	56.300071
Gglean007887	7.69003	11.44687	9.414188806	11.1909
Gglean007888	0	0	0	0
Gglean007889	35.02563001	31.48724276	65.9445345	74.96656382
Gglean007890	77.8013	45.9556	50.594	51.5453
Gglean007891	12.53942	13.24586	6.75115	5.86918
Gglean007892	15.27010136	16.63669791	15.60803107	18.70741777
Gglean007893	0.0898115	0.0936882	0.0728425	0
Gglean007894	15.27010136	16.63669791	15.60803107	18.70741777
Gglean007895	15.27010136	16.63669791	15.60803107	18.70741777
Gglean007896	3.949316055	2.99070394	3.91006543	4.619421041
Gglean007897	2.071388	1.878231968	2.526189023	2.770160976
Gglean007898	245.4856244	231.9976058	281.8935863	385.9641158
Gglean007899	245.4856244	231.9976058	281.8935863	385.9641158
Gglean007900	0.431096077	0.231610234	0.0301827	0.079277482
Gglean007901	0	0	0	0
Gglean007902	5.20528	14.1118	23.5468	35.8062
Gglean007903	24.223299	30.140669	16.7871799	24.151266
Gglean007904	24.223299	30.140669	16.7871799	24.151266
Gglean007905	24.223299	30.140669	16.7871799	24.151266
Gglean007906	49.7641	51.868	39.8072	38.6848
Gglean007907	22.005172	23.165221	29.817795	28.23667326
Gglean007908	13.9221	16.7128	18.8563	16.3903
Gglean007909	29.545664	32.62298033	19.02626078	20.27363
Gglean007910	27.69894	25.50707891	26.68021	32.3104
Gglean007911	67.79888939	75.08565754	17.40539286	13.00381272
Gglean007912	46.59056	44.4427	35.79011	32.54372162
Gglean007913	18.65520412	18.62348152	28.01789832	29.69883508
Gglean007914	18.65520412	18.62348152	28.01789832	29.69883508
Gglean007915	18.65520412	18.62348152	28.01789832	29.69883508
Gglean007916	11.6318321	12.46246701	20.19108	21.97063
Gglean007917	11.6318321	12.46246701	20.19108	21.97063
Gglean007918	21.91046	24.6431	27.6259	24.6713
Gglean007919	14.81383	14.54483	9.36199	7.89799

Gglean007920	16.15949	19.12835	28.13244	29.857
Gglean007921	4.387990683	5.6889	5.157987912	6.941608863
Gglean007922	0.107185	0	0	0.0517323
Gglean007923	0	0	0.151014	0
Gglean007924	81.4948	82.9839	41.5111	36.8117
Gglean007925	58.94967	67.02389	61.336287	60.84983
Gglean007926	58.94967	67.02389	61.336287	60.84983
Gglean007927	41.1106	38.0635	26.8356	25.5095
Gglean007928	12.17764096	17.92429	18.72537	23.90233
Gglean007929	21.641284	20.71153535	37.64232	38.38362
Gglean007930	0	0	0	0
Gglean007931	35.4112806	37.50636184	28.08005016	26.02825164
Gglean007932	0	0	0	0
Gglean007933	52.70228	56.16736	37.23498	36.29215
Gglean007934	43.9976774	44.61417698	65.02653055	66.76408949
Gglean007935	30.8658	30.9449	19.9361	19.7684
Gglean007936	28.35324	23.24721	30.12199	31.80915
Gglean007937	985.165	983.272	621.337	609.859
Gglean007938	1396.24	1342.56	1349.37	1628.54
Gglean007939	14.7854	10.93584	19.96177	26.66542
Gglean007940	14.7854	10.93584	19.96177	26.66542
Gglean007941	4.899540634	5.785795643	5.341300879	7.201825017
Gglean007942	4.899540634	5.785795643	5.341300879	7.201825017
Gglean007943	22.291775	20.81820173	28.722116	36.94516
Gglean007944	0	0	0	0
Gglean007945	912.27829	958.9415749	546.4701087	518.0776497
Gglean007946	912.27829	958.9415749	546.4701087	518.0776497
Gglean007947	9.921180055	10.79111233	8.686553784	7.838633339
Gglean007948	0.084221572	0.086262981	0.045368921	0.042642244
Gglean007949	0.084221572	0.086262981	0.045368921	0.042642244
Gglean007950	0.084221572	0.086262981	0.045368921	0.042642244
Gglean007951	9.209612488	9.116229	10.178718	11.511747
Gglean007952	29.10154	28.15997	18.16192	16.701733
Gglean007953	12.48332	8.7543	11.81332	13.46288
Gglean007954	99.7609	76.527	72.6121	77.9048
Gglean007955	0	0.723907	0.2756	0.329531
Gglean007956	44.70321102	50.74687157	41.8794882	43.3546007
Gglean007957	44.70321102	50.74687157	41.8794882	43.3546007
Gglean007958	120.65	115.436	71.0565	62.8059
Gglean007959	61.184324	63.59509701	49.33183	45.40252
Gglean007960	4.13383	6.99652	7.8878	10.45338
Gglean007961	0.273942573	0.3109016	0.330480455	1.683744153
Gglean007962	0.273942573	0.3109016	0.330480455	1.683744153
Gglean007963	0.35936057	0.5906539	0.697487943	0.779325921

Gglean007964	18.0401	21.13288	6.02976	5.75096
Gglean007965	10.569896	12.78282382	15.35272759	19.359632
Gglean007966	10.569896	12.78282382	15.35272759	19.359632
Gglean007967	10.569896	12.78282382	15.35272759	19.359632
Gglean007968	50.05655629	54.955675	47.04218269	41.67884352
Gglean007969	139.3408	150.6547	138.74138	142.72794
Gglean007970	39.0104	38.2306	35.8547	31.6601
Gglean007971	4.734270674	7.126107	4.611074	4.219657089
Gglean007972	3.578549	4.56677	6.083219	6.838302
Gglean007973	36.1729	40.9155	20.57612	17.77573
Gglean007974	71.8204	68.23436	55.49533	54.95905
Gglean007975	71.8204	68.23436	55.49533	54.95905
Gglean007976	2.478724	2.936473	1.808784	1.722737
Gglean007977	191.4312	161.5371	187.959	238.103331
Gglean007978	1.3323	1.58183	2.49452	2.17954
Gglean007979	0.293626	0.116148	0.293445	1.388053
Gglean007980	1.13806	0	1.274193	0.559631
Gglean007981	3.130473769	3.534878837	3.5855709	4.102529399
Gglean007982	3.304331312	4.036950463	4.310000769	7.758390444
Gglean007983	24.95825	24.64371	18.6272	18.9242
Gglean007984	40.28118002	44.52226253	45.879385	42.56163134
Gglean007985	13.38248	15.76865	8.8981	8.22262
Gglean007986	25.4871	24.8043	20.5024	17.762
Gglean007987	39.40473	42.09416	30.671918	25.891346
Gglean007988	40.69150106	41.3456435	48.47686814	51.14343402
Gglean007989	0.0492504	0	0	0
Gglean007990	12.7161	13.91922	16.64175	18.65083
Gglean007991	0	0	0	0
Gglean007992	0.0307962	0.086445594	0.0750809	0.0593561
Gglean007993	5.252179087	5.269977237	4.4481642	4.295156143
Gglean007994	15.859537	15.02411	22.722574	25.401994
Gglean007995	0	0	0	0
Gglean007996	0	0	0	0
Gglean007997	549.6061778	527.1080609	940.3577355	1030.305865
Gglean007998	11.756201	12.53387	9.167901	9.514857746
Gglean007999	0.0713989	0.0744779	0.232512	0.172083
Gglean008000	8.205953	9.512424	3.511182	3.523547
Gglean008001	8.205953	9.512424	3.511182	3.523547
Gglean008002	3.32637279	2.617356038	3.65923998	4.082788522
Gglean008003	928.0969254	928.7190872	1313.777874	1587.663753
Gglean008004	928.0969254	928.7190872	1313.777874	1587.663753
Gglean008005	28.775071	30.744203	26.880346	25.56984
Gglean008006	34.35236	30.43584	31.86969	32.44024
Gglean008007	19.191408	18.3445618	25.92174457	32.2634

Gglean008008	0.0413775	0	0.045569068	0.12016696
Gglean008009	0	0	0	0
Gglean008010	4.27033	3.889522	4.30795655	5.773294171
Gglean008011	4.27033	3.889522	4.30795655	5.773294171
Gglean008012	16.18594	15.90236	23.02186	22.61614
Gglean008013	66.5817	69.7034157	92.3147	91.7512
Gglean008014	289.3169304	301.7762953	214.83754	221.3942509
Gglean008015	0	0	0	0
Gglean008016	15.79896109	16.92012261	25.07872205	30.10295124
Gglean008017	15.79896109	16.92012261	25.07872205	30.10295124
Gglean008018	0.392363	0.102681	0	0.0956008
Gglean008019	0	0.150174	0	0
Gglean008020	0	0.123208	0	0
Gglean008021	0	0	0	0.193816
Gglean008022	0	0	0	0
Gglean008023	0	0	0	0
Gglean008024	0	0	0.186714	0
Gglean008025	0.0232935	0.0479126	0.0376857	0.111286
Gglean008026	0	0	0.258122	0
Gglean008027	0	0	0	0
Gglean008028	0	0	0	0
Gglean008029	0.391652	0.138137	0.106927	0
Gglean008030	13.19539433	14.070568	18.03220791	19.29894594
Gglean008031	9.92598	11.9094	8.04738	7.37042
Gglean008032	22.237374	20.06312	24.16434	26.55283
Gglean008033	7.781639345	7.62879	10.572045	13.550206
Gglean008034	676.083	489.457	764.624	841.576
Gglean008035	7.14914	5.07491	4.16260523	4.030075
Gglean008036	4.47539	4.410956	2.009401	1.610045
Gglean008037	4.47539	4.410956	2.009401	1.610045
Gglean008038	33.4989	33.1332	15.0558	14.5311
Gglean008039	1.589284	1.31082	4.94497	3.281721
Gglean008040	2.07253	1.88421	3.17058	2.9874
Gglean008041	15.3629	9.19618992	29.51839	16.28565127
Gglean008042	15.3629	9.19618992	29.51839	16.28565127
Gglean008043	100.3858592	99.32844298	80.01222917	75.10516011
Gglean008044	32.08095002	34.28300598	28.26852106	27.21907696
Gglean008045	21.18440827	26.326857	30.9908883	33.97467608
Gglean008046	15.62751	18.81755	17.87251	15.72301
Gglean008047	61.088183	71.93281806	34.52013073	31.44609955
Gglean008048	57.8653774	66.92870369	49.050804	41.45828243
Gglean008049	57.8653774	66.92870369	49.050804	41.45828243
Gglean008050	10.35873	9.5235	14.91348	18.89804
Gglean008051	38.86191	42.37197198	49.14652686	49.53953634

Gglean008052	0.431637612	0.117518362	0.206509732	0.460655217
Gglean008053	4.79009	4.44507	3.31531	2.72844
Gglean008054	58.86958797	73.07617645	80.80911996	81.14540205
Gglean008055	18.87158694	19.77521001	7.249274009	6.391769091
Gglean008056	18.87158694	19.77521001	7.249274009	6.391769091
Gglean008057	4.68528232	6.381540595	8.076752145	9.668973143
Gglean008058	2.986529	2.786239	4.61963	5.45271
Gglean008059	6.45498	1.973153	3.748758	4.42191
Gglean008060	0.207368	0	0	0
Gglean008061	10.04041482	10.4006624	15.38038354	18.42014961
Gglean008062	8.69647	10.12584	14.19093	17.4986
Gglean008063	0.132336	0.172127	0.375195	0.349457
Gglean008064	13.311	16.5991	9.58832	8.09059
Gglean008065	79.97565	77.17178047	88.86759618	82.78809387
Gglean008066	20.07956	21.30512	22.07891	25.06856
Gglean008067	27.92922	25.6587	48.6356	60.6828
Gglean008068	21.63632	24.21425	9.670124	8.33385
Gglean008069	2.53195	3.84168	3.86826	4.26133
Gglean008070	77.65243	83.81721	46.620429	45.094048
Gglean008071	77.65243	83.81721	46.620429	45.094048
Gglean008072	2.675092616	3.642690784	2.9308403	3.338518
Gglean008073	30.2784	32.8189	17.77742	16.67869
Gglean008074	3.710776	3.795041	5.598822	5.120810388
Gglean008075	0	0	0.0691556	0
Gglean008076	0	0	0.042195788	0.01996145
Gglean008077	49.69871	45.68355	43.58042	49.88784
Gglean008078	93.351827	76.70298677	91.252012	102.2347434
Gglean008079	11.8567	11.8849	7.5823	9.15456
Gglean008080	85.18049	96.23411877	66.52053164	63.32641249
Gglean008081	15.04562	17.57444	4.612012	4.48819
Gglean008082	14.7326	13.691	1.74997	0.861655
Gglean008083	0.193965509	0.066384188	0.261292252	0.579580099
Gglean008084	1.3462	1.19905	1.16247	0.946665
Gglean008085	118.1339	115.0998	97.7717	90.3786
Gglean008086	13.55660753	15.14364324	17.02005643	19.029216
Gglean008087	21.03813	22.6464	16.64005	14.10583
Gglean008088	1686.786379	1668.216994	1048.67518	1032.351745
Gglean008089	1686.786379	1668.216994	1048.67518	1032.351745
Gglean008090	42.0544	35.2599	44.8773	46.4783
Gglean008091	99.616878	98.466	136.578707	131.336481
Gglean008092	0	0	0	0
Gglean008093	0.511787	0.134977	0	0
Gglean008094	16.49033	18.7696	18.761	22.25915
Gglean008095	0	0	0	0

Gglean008096	0.0550738	0	0.0445963	0.105237
Gglean008097	0	0	0	0
Gglean008098	0	0	0	0
Gglean008099	21.33632253	21.76885309	37.223982	38.93816338
Gglean008100	0	0.347648	0	0.161096
Gglean008101	15.03067	14.95758	10.61228	11.59228
Gglean008102	35.05113113	31.489483	9.630801	8.633793472
Gglean008103	5.2148	3.94308	7.37665	6.47949
Gglean008104	38.549	39.9396	17.11299	15.3503
Gglean008105	12.45605	12.46038	9.30324	10.17216
Gglean008106	5.36925	4.94835	6.44327	11.46951
Gglean008107	29.976341	27.019709	25.75597	27.659171
Gglean008108	2.99401	3.99776	0.902612	0.548732
Gglean008109	21.30514	23.62316	11.31712	10.011867
Gglean008110	207.7747	125.813704	161.7825	266.36233
Gglean008111	14.7326	13.691	7.10831	6.85908
Gglean008112	7.74817	8.65878	15.79821	16.80037
Gglean008113	351.678	425.988	331.973	442.084
Gglean008114	24.15357206	24.44760365	19.480885	18.1196606
Gglean008115	0	0	0	0
Gglean008116	0.097458743	0.0959012	0.0839957	0.048402213
Gglean008117	39.01010566	36.46775846	67.72207758	75.36817126
Gglean008118	1.21313	1.26588	4.29052	2.16254
Gglean008119	88.78258	95.83702	66.381317	66.903643
Gglean008120	88.78258	95.83702	66.381317	66.903643
Gglean008121	0.0819955	0.55547	0.747601	1.97415
Gglean008122	2.08903	1.55808	1.90195	1.46001
Gglean008123	24.28371132	24.36016802	35.812334	36.89084186
Gglean008124	11.57101	11.97008	8.85325	10.06135
Gglean008125	730.038	670.71	467.243	505.248
Gglean008126	48.7666	57.1704	23.23499	17.89143
Gglean008127	0.19023863	0	0.146921154	0.309249024
Gglean008128	0.177937972	0.082926725	0.259273624	0.238951155
Gglean008129	13.204175	12.6948104	19.49423924	19.93776351
Gglean008130	0	0	0	0
Gglean008131	0	0.110066	0.261007	0.206258
Gglean008132	3.244451353	4.454014977	5.14959782	6.739698884
Gglean008133	45.637972	46.61844306	52.68716002	90.80918894
Gglean008134	144.9614813	145.41378	150.0907	148.7143
Gglean008135	41.91618971	52.0697421	41.40083705	38.1719709
Gglean008136	0	0	0	0
Gglean008137	81.452107	74.6974447	100.728407	143.24843
Gglean008138	1.286466	1.281785	0.134949	0.111029568
Gglean008139	1.286466	1.281785	0.134949	0.111029568

Gglean008140	3.058133079	1.822189	2.2757966	5.172267898
Gglean008141	19.2803189	20.56608715	25.14166097	32.13742395
Gglean008142	40.42429382	44.38354171	15.54133239	14.5254184
Gglean008143	40.42429382	44.38354171	15.54133239	14.5254184
Gglean008144	40.42429382	44.38354171	15.54133239	14.5254184
Gglean008145	40.42429382	44.38354171	15.54133239	14.5254184
Gglean008146	40.42429382	44.38354171	15.54133239	14.5254184
Gglean008147	49.742529	45.1851847	62.58607618	73.11030516
Gglean008148	49.742529	45.1851847	62.58607618	73.11030516
Gglean008149	19.1882254	23.53766	12.69454	23.36481
Gglean008150	19.1882254	23.53766	12.69454	23.36481
Gglean008151	19.1882254	23.53766	12.69454	23.36481
Gglean008152	94.79141	100.37342	64.37644	55.40457
Gglean008153	39.33924	31.56536	49.18939	45.85606
Gglean008154	39.33924	31.56536	49.18939	45.85606
Gglean008155	0.472112751	0.341582341	0.44166719	0.5468004
Gglean008156	7.66879	7.19886	10.0836	12.8425
Gglean008157	34.220223	33.99750367	22.92080902	18.77926327
Gglean008158	27.80662	27.73301	27.6979	25.33515
Gglean008159	6.542967	7.3562912	7.740469139	8.0962457
Gglean008160	27.654438	27.22284	25.70271	23.63024
Gglean008161	59.1217	65.004	72.7573	68.6309
Gglean008162	23.93994403	23.82517852	41.79140806	39.85264022
Gglean008163	23.93994403	23.82517852	41.79140806	39.85264022
Gglean008164	28.4325	27.5963	24.33193	20.673
Gglean008165	11.89765352	12.079433	4.29927	4.27062696
Gglean008166	11.89765352	12.079433	4.29927	4.27062696
Gglean008167	82.452458	93.599598	18.418494	15.10921604
Gglean008168	6.27661	7.70169	11.09756	13.83193
Gglean008169	6.27661	7.70169	11.09756	13.83193
Gglean008170	25.34873	29.82907	17.2967243	17.13592
Gglean008171	65.84657607	60.04273636	52.35762362	47.83302614
Gglean008172	6.74812	8.4288	7.71147	7.19869
Gglean008173	34.39201807	36.80081934	50.69612992	49.12674713
Gglean008174	16.61398	13.27919	14.79827	22.1577
Gglean008175	45.30652855	43.54655743	39.79718	33.2190556
Gglean008176	0	0	0	0
Gglean008177	0	0	0	0
Gglean008178	0	0	0	0
Gglean008179	0.137618	0.0230536	0.0373245	0.0444689
Gglean008180	0	0	0	0
Gglean008181	0	0	0	0
Gglean008182	3.380801	6.608485	2.47204	1.370472
Gglean008183	0	0	0	0

Gglean008184	1.191387602	1.221201134	0.956929341	1.165834681
Gglean008185	0	0	0	0
Gglean008186	0	0	0	0
Gglean008187	0	0	0	0.06154703
Gglean008188	0	0	0	0.06154703
Gglean008189	2.806236322	2.601595	2.010351622	2.944175352
Gglean008190	0	0	0	0
Gglean008191	0	0	0	0
Gglean008192	61.11448127	66.61320732	15.09797264	12.77047158
Gglean008193	61.11448127	66.61320732	15.09797264	12.77047158
Gglean008194	55.3343	56.6244	51.8128	51.6979
Gglean008195	44.364141	45.95892	33.865822	31.96388
Gglean008196	59.62446	82.284263	98.506599	112.646858
Gglean008197	59.62446	82.284263	98.506599	112.646858
Gglean008198	973.3913	1039.3615	655.7238	624.7869
Gglean008199	0.000784052	1.48676	0.000877111	1.89E-06
Gglean008200	0	0	0	0
Gglean008201	3.549571405	4.235640001	5.000650008	5.294360001
Gglean008202	17.0814107	16.23198277	12.70287	9.61663316
Gglean008203	3.36021	5.57047	4.4906	6.34257
Gglean008204	24.27567	24.990045	15.61927584	13.102571
Gglean008205	0	0	0	0
Gglean008206	25.66794	24.26411	16.3833135	13.01541
Gglean008207	0	0	0.084515	0
Gglean008208	0	0	0	0
Gglean008209	33.11230721	34.35901318	37.57789359	38.66508444
Gglean008210	8.715209	8.419881	5.820748	4.304585497
Gglean008211	130.75756	131.40925	151.28	193.90791
Gglean008212	45.56341	41.55831	42.14224	39.44721
Gglean008213	14.51445	14.38094	9.99724	9.585213
Gglean008214	56.0738	64.6072	57.7248	54.0487
Gglean008215	583.413777	575.660232	437.6795227	380.5700987
Gglean008216	0.425706	0.147446	0.424116	0.409452
Gglean008217	5.88978	5.3401	3.82225	4.02396
Gglean008218	0	0	0.77547	0
Gglean008219	0	0	0	0
Gglean008220	0.0528081	0	0	0
Gglean008221	27.139171	26.86019	17.18994152	17.18483979
Gglean008222	3.186359837	2.809916	6.5275294	5.885253
Gglean008223	3.186359837	2.809916	6.5275294	5.885253
Gglean008224	0.460654	0.616162	0.533531	0.381141
Gglean008225	0.703161	0.159733	7.63E-06	0.895675
Gglean008226	129.805	351.506	0.884215	0.923702
Gglean008227	13.73520701	11.99185505	12.457214	13.24663301

Gglean008228	63.98770566	67.95633	90.8703	101.32934
Gglean008229	63.98770566	67.95633	90.8703	101.32934
Gglean008230	38.16720233	39.27494639	72.43029577	76.44923573
Gglean008231	5.22E-07	2.02E-11	8.87E-05	4.31E-05
Gglean008232	0	0	0	0
Gglean008233	0.669809	2.75217	1.92361	0.061667
Gglean008234	37.5736	44.862	28.1382	26.8192
Gglean008235	23.42312	24.78415	22.69372	19.66834
Gglean008236	0.165295	0	0	0.0660691
Gglean008237	0	0	0	0
Gglean008238	0	0	0	0
Gglean008239	22.70167	18.55484	22.27531	19.62622
Gglean008240	10.7908	12.0853	5.75749	4.4512
Gglean008241	2.860084047	2.868307994	3.746424	4.683364987
Gglean008242	0	0	0	0
Gglean008243	4.2944	4.71505	1.59344	1.07949
Gglean008244	190.4887743	204.6032986	169.0996076	154.4422524
Gglean008245	47.12908306	47.02873203	47.76158082	44.32118216
Gglean008246	1.77749	0.383901	0.466929	1.27684
Gglean008247	0.315313	0	0	0
Gglean008248	0	0	0	0.555036
Gglean008249	47.12908306	47.02873203	47.76158082	44.32118216
Gglean008250	56.3573	56.3492	39.1068	39.1826
Gglean008251	31.22375	32.72305	35.09803	33.82234
Gglean008252	3.5269078	4.004300859	2.429304	1.9771905
Gglean008253	14.25176211	14.89339297	21.39060897	23.99368223
Gglean008254	40.23174255	36.45766563	34.720197	37.40720563
Gglean008255	125.76543	124.65128	147.83854	140.94047
Gglean008256	14.92269	19.617936	30.3959	30.25142
Gglean008257	47.66204622	47.95823307	33.68106828	28.71383085
Gglean008258	0	0	0	0
Gglean008259	20.1884395	22.34327457	18.34467854	17.28737205
Gglean008260	20.1884395	22.34327457	18.34467854	17.28737205
Gglean008261	18.02867	15.25267001	10.847968	16.90430854
Gglean008262	18.02867	15.25267001	10.847968	16.90430854
Gglean008263	0	0	0	1.62907
Gglean008264	55.95820448	56.4116251	42.74711469	40.52544923
Gglean008265	11.42763	11.54367	6.76346	7.71274
Gglean008266	167.9071486	169.9241873	215.6785625	290.8321308
Gglean008267	0.144188	0	0.117357	0.139431
Gglean008268	0.346016	0.177118	0.325896	0.44362
Gglean008269	0	0	0	0
Gglean008270	0	0	0	0
Gglean008271	0	0	0	0

Gglean008272	0.183324	0.38774	0.150794	0
Gglean008273	0	0	0	0
Gglean008274	0.0866756	0.1334	0.035135	0.0417163
Gglean008275	0	0	0	0
Gglean008276	0	0	0	0
Gglean008277	0.0293763	0.0600575	0.0237337	0.0565003
Gglean008278	0	0	0	0
Gglean008279	0	0	0	0
Gglean008280	0	0.191939	0	0
Gglean008281	0.416553	0	0.103211	0.0611987
Gglean008282	0	0	0	0
Gglean008283	0	0	0	0
Gglean008284	0.51986	0	0	0
Gglean008285	0.503522	0.407652	0.1614016	0.091587066
Gglean008286	0.163176	0	0.131959	0.103747
Gglean008287	0	0	0	0
Gglean008288	0	0	0	0
Gglean008289	47.58067	45.4642	39.09056	36.20258
Gglean008290	79.7261	70.6133	45.9481	44.0296
Gglean008291	39.41909234	38.25708	32.468938	29.65076568
Gglean008292	39.41909234	38.25708	32.468938	29.65076568
Gglean008293	33.272236	33.04633377	30.533129	27.833243
Gglean008294	33.272236	33.04633377	30.533129	27.833243
Gglean008295	464.7428	469.5435	363.7481	363.659
Gglean008296	464.7428	469.5435	363.7481	363.659
Gglean008297	2.97841	1.38923	1.13565	1.34845
Gglean008298	2.60E-05	2.53E-05	0.903642	1.55E-05
Gglean008299	1574.467	1555.092	437.361	356.393
Gglean008300	52.66273095	49.81654621	35.1997598	30.52206387
Gglean008301	78.7398	76.2641	53.00948	54.2815
Gglean008302	0.649734	0.477278	0.679341	0.357754
Gglean008303	3.47058	0	0	0
Gglean008304	0	0	0	0
Gglean008305	19.82532	17.88359	20.35611	22.34014
Gglean008306	148.2414231	146.2019337	133.5708074	137.5296299
Gglean008307	47.51811	58.05620007	73.02822642	61.6634148
Gglean008308	130.66922	130.81992	138.36743	79.23338
Gglean008309	2.86617	3.03402	2.616680149	3.87474
Gglean008310	0	0	0	0
Gglean008311	6.632779767	6.627948849	8.697641133	12.7818799
Gglean008312	147.26982	142.05941	91.73813	83.42518
Gglean008313	37.51322	37.17239867	33.33485802	40.85285064
Gglean008314	23.984694	23.914485	22.81599	21.222807
Gglean008315	5.28874	3.484308	2.848421	2.856904

Gglean008316	28.94926	28.3051	19.00017	16.38766
Gglean008317	70.6897	81.5234	73.0724	56.3223
Gglean008318	42.91921	44.9405084	57.2002704	67.04509
Gglean008319	33.9484	33.29234	28.3791	27.13817
Gglean008320	23.60319	25.56379	21.84254	21.75237
Gglean008321	0.027379	0.0559828	0.154433	0.0261406
Gglean008322	0	0	0.0453151	0.0537043
Gglean008323	0.499007	0.53817	0.206765	1.48183
Gglean008324	0	0	0	0
Gglean008325	31.5611	32.68814	30.15791	27.68843
Gglean008326	1.133215	1.011062	2.325481	2.308765
Gglean008327	37.95346	57.1298	17.5576	13.52775
Gglean008328	16.50402614	16.09333	11.33335728	8.870366
Gglean008329	16.50402614	16.09333	11.33335728	8.870366
Gglean008330	11.5396	11.7689	11.8377	11.8615
Gglean008331	16.50402614	16.09333	11.33335728	8.870366
Gglean008332	16.50402614	16.09333	11.33335728	8.870366
Gglean008333	0.446096	0.251557	0.234656	0.384555
Gglean008334	3.999514	4.884329	5.840117	5.809129
Gglean008335	19.826	21.25304656	27.93801247	26.57875677
Gglean008336	35.68074738	32.1168608	39.2938204	40.19039677
Gglean008337	35.68074738	32.1168608	39.2938204	40.19039677
Gglean008338	7.663736917	7.840164062	9.206346952	10.35943428
Gglean008339	4.78911	2.57243	4.01808	6.96089
Gglean008340	0	0.0280986	0	0
Gglean008341	0	0.0206172	0	0
Gglean008342	38.845	39.0604	21.61656	17.9999
Gglean008343	40.85942	39.4152	23.92131	20.37189
Gglean008344	1.921745867	1.376796953	2.7267375	3.2760634
Gglean008345	22.45493	22.32764	31.13743	44.5706
Gglean008346	55.664357	54.66945574	45.764448	46.57781753
Gglean008347	55.664357	54.66945574	45.764448	46.57781753
Gglean008348	0	0.0235465	0	0
Gglean008349	0	0.0235465	0	0
Gglean008350	22.68703	22.35673	24.61949	22.41935
Gglean008351	10.87861827	9.803148042	6.169806489	5.997985586
Gglean008352	0.387726	0.253186	0.146445	0.123547
Gglean008353	0.319446	0.184179	0.293578	0.275004
Gglean008354	1435.667395	1518.507611	959.0880121	919.3347839
Gglean008355	12.37300809	13.08583724	18.10808675	21.3204726
Gglean008356	3.34675	3.30577	6.79951	3.9231
Gglean008357	10.05220875	11.35999623	13.70422735	16.29726234
Gglean008358	10.05220875	11.35999623	13.70422735	16.29726234
Gglean008359	10.05220875	11.35999623	13.70422735	16.29726234

Gglean008360	52.081272	50.12855	25.801632	24.240976
Gglean008361	81.4485	82.2997	56.18705	51.0785
Gglean008362	5.84547	5.95267	6.64664	5.38725
Gglean008363	2.9457	2.60453	2.41416	1.76182
Gglean008364	0.813309	0.345367	0.399918	1.11449
Gglean008365	0	0	0	0
Gglean008366	12.2240957	7.9493271	11.831629	11.796815
Gglean008367	0	0	0	0
Gglean008368	0	0	0	0
Gglean008369	31.637673	29.161549	20.87453	25.3514657
Gglean008370	31.637673	29.161549	20.87453	25.3514657
Gglean008371	48.1611	45.4069	50.4598	44.3178
Gglean008372	0.67374361	0.9377701	0.521033138	0.621608126
Gglean008373	309.0995	321.0146	318.834	298.833
Gglean008374	7.04619	5.97849	5.70711	4.52953
Gglean008375	0	0	0	0
Gglean008376	3.84823	3.23465	3.78299149	6.20885
Gglean008377	3.84823	3.23465	3.78299149	6.20885
Gglean008378	12.91698	12.09421	15.26389	19.1368
Gglean008379	16.2024	10.4809	11.0917	14.963
Gglean008380	10.6607	6.26003	6.75038	6.74169
Gglean008381	31.99700492	29.95313	37.50880081	35.9559553
Gglean008382	33.706446	37.9090878	20.494548	17.0693482
Gglean008383	33.706446	37.9090878	20.494548	17.0693482
Gglean008384	33.706446	37.9090878	20.494548	17.0693482
Gglean008385	85.608175	87.910415	59.994228	57.88788045
Gglean008386	85.608175	87.910415	59.994228	57.88788045
Gglean008387	36.66247	36.09486	29.50654	29.35231001
Gglean008388	19.6504	20.1273	9.91772	9.06808
Gglean008389	2.60242	2.96775	0	15.178
Gglean008390	22.75578	18.1643	26.57675	29.85435
Gglean008391	26.56591	46.29025	7.62893	8.82197
Gglean008392	45.0938	46.2103	29.1058	24.485322
Gglean008393	16.75677	17.01057	18.54461	21.36792
Gglean008394	18.1369627	21.08402308	39.87797417	39.3333316
Gglean008395	18.1369627	21.08402308	39.87797417	39.3333316
Gglean008396	7.64948371	6.891877256	10.41389158	8.223423175
Gglean008397	43.201361	40.95861091	32.47484	30.76760922
Gglean008398	382.7588194	389.009807	534.103361	560.0440027
Gglean008399	25.557937	24.3958	25.203239	23.697054
Gglean008400	0	0.190899	0	0
Gglean008401	0.049356669	0.0508004	0	0
Gglean008402	0.049356669	0.0508004	0	0
Gglean008403	17.80464	18.26667	11.44478	9.0613

Gglean008404	18.79081236	17.6054	30.78610009	33.63360056
Gglean008405	0.326344	0.351852	0.270864	0.971113
Gglean008406	0.342819	0.264971	0.046219	0.164463
Gglean008407	3.57283	3.23931	3.56813	6.35483
Gglean008408	15.76666471	14.3521954	15.911354	15.248637
Gglean008409	92.7828	103.225	105.332	97.6219
Gglean008410	23.4712	21.2225	18.1294	16.6731
Gglean008411	1.58981166	2.762417	1.765879	4.06838
Gglean008412	46.6626	49.0453	38.0953	40.0522
Gglean008413	43.75093166	43.95434463	44.76072151	46.31442113
Gglean008414	43.75093166	43.95434463	44.76072151	46.31442113
Gglean008415	31.5212	29.2897	10.9943	7.416
Gglean008416	9.79664	0	0	0
Gglean008417	9.48377	7.33915	4.4776	4.10256
Gglean008418	29.28594	29.51901401	25.81678	24.077868
Gglean008419	29.28594	29.51901401	25.81678	24.077868
Gglean008420	16.33729	19.40851	7.42125053	7.727911
Gglean008421	82.45939	97.912009	76.28412	81.41611
Gglean008422	82.45939	97.912009	76.28412	81.41611
Gglean008423	171.9412303	170.6508311	168.642744	140.913892
Gglean008424	171.9412303	170.6508311	168.642744	140.913892
Gglean008425	10.95094074	8.99079	3.968300015	2.79023001
Gglean008426	0.251772	0	0	0
Gglean008427	53.36527167	57.495351	28.58339438	24.58446984
Gglean008428	0	0	0.0744407	0
Gglean008429	32.729388	37.659883	55.43752	59.59833
Gglean008430	13.42195543	13.77657079	10.7490277	10.12792661
Gglean008431	5.14785	6.71204	8.481075	10.720344
Gglean008432	167.68261	179.0274	139.65743	144.446
Gglean008433	0.994874	0.965317	2.469735	3.02813
Gglean008434	80.78467	81.56358091	49.65236	42.633328
Gglean008435	0.5899181	1.363157	1.277068	2.22694
Gglean008436	1.930510013	1.98562002	2.0057	3.938220023
Gglean008437	14.68976923	14.44988065	18.29261726	28.20102524
Gglean008438	0.0274884	0	0	0.026356
Gglean008439	90.9692	94.0139	61.6526	52.9646
Gglean008440	10.25058	9.79749	7.34363	7.27589
Gglean008441	31.1041672	31.29381	43.83973	50.63324
Gglean008442	0.965759	2.626936	1.496527	2.008464055
Gglean008443	113.746383	128.6212942	97.162467	87.0144023
Gglean008444	2.2844405	2.022721	1.416969	1.64334
Gglean008445	2.2844405	2.022721	1.416969	1.64334
Gglean008446	13.08515	13.24972	23.83881	21.42194
Gglean008447	0	0.581106	0	0.357814

Gglean008448	82.4295	85.105	57.2666	52.2286
Gglean008449	0	0	0	0
Gglean008450	44.731988	52.743641	25.466512	25.48871516
Gglean008451	13.1235	14.4428	22.042	30.6733
Gglean008452	44.731988	52.743641	25.466512	25.48871516
Gglean008453	44.731988	52.743641	25.466512	25.48871516
Gglean008454	44.731988	52.743641	25.466512	25.48871516
Gglean008455	44.731988	52.743641	25.466512	25.48871516
Gglean008456	44.731988	52.743641	25.466512	25.48871516
Gglean008457	47.415475	47.26746	68.1660983	66.98214567
Gglean008458	0	0	0	0
Gglean008459	0	0	0	0
Gglean008460	0	0	0.0344688	0
Gglean008461	0	0	0	5.80202
Gglean008462	0.0690222	0	0	0
Gglean008463	0	0	0.292272	0.23102
Gglean008464	39.2646395	43.44644083	64.79026662	70.29730583
Gglean008465	5.872866077	5.973826958	5.899573312	8.727457306
Gglean008466	5.872866077	5.973826958	5.899573312	8.727457306
Gglean008467	0	0	0	0
Gglean008468	24.72141644	23.90638672	20.6351444	24.5572125
Gglean008469	24.72141644	23.90638672	20.6351444	24.5572125
Gglean008470	0.682687	7.47E-07	5.43E-05	0.241518
Gglean008471	204.17772	185.546898	78.73904	33.19386
Gglean008472	256.2888971	277.7263375	69.99269078	51.7560048
Gglean008473	256.2888971	277.7263375	69.99269078	51.7560048
Gglean008474	31.0049514	40.75148315	4.784848895	3.864176151
Gglean008475	137.2458	130.9941	148.4675	164.8478
Gglean008476	23.83898	23.380406	19.528817	18.522096
Gglean008477	44.977	44.1961	17.647	14.3899
Gglean008478	6.06807	7.70936	2.74285	4.87363
Gglean008479	50.3402	52.7148	42.8487	39.29144
Gglean008480	50.3402	52.7148	42.8487	39.29144
Gglean008481	1.071625219	1.150150556	1.418684093	2.187318031
Gglean008482	14.39946578	14.93188046	7.7175882	8.208084718
Gglean008483	14.39946578	14.93188046	7.7175882	8.208084718
Gglean008484	14.39946578	14.93188046	7.7175882	8.208084718
Gglean008485	28.714	18.5524	28.2875	24.8104
Gglean008486	80.23135	78.31741	65.751	66.47214
Gglean008487	7.81698	7.68871	3.92933	4.04305
Gglean008488	0	0	0	0
Gglean008489	57.026403	48.01910072	70.583933	70.365123
Gglean008490	57.026403	48.01910072	70.583933	70.365123
Gglean008491	57.026403	48.01910072	70.583933	70.365123

Gglean008492	57.026403	48.01910072	70.583933	70.365123
Gglean008493	57.026403	48.01910072	70.583933	70.365123
Gglean008494	57.026403	48.01910072	70.583933	70.365123
Gglean008495	57.026403	48.01910072	70.583933	70.365123
Gglean008496	57.026403	48.01910072	70.583933	70.365123
Gglean008497	57.026403	48.01910072	70.583933	70.365123
Gglean008498	6.94986132	7.828892406	2.101676398	2.503979701
Gglean008499	6.94986132	7.828892406	2.101676398	2.503979701
Gglean008500	25.413	24.3738	10.6006	13.7939
Gglean008501	21.9998	22.321	30.4997	22.1194
Gglean008502	0	0	0	0
Gglean008503	3.282090028	1.312338475	3.404456218	3.944770129
Gglean008504	0	0	0	0
Gglean008505	0	0	0	0
Gglean008506	9.06273	12.34312492	1.195302	1.249422
Gglean008507	0.04494	0	0	0
Gglean008508	22.243086	23.382541	22.720838	24.29265806
Gglean008509	22.243086	23.382541	22.720838	24.29265806
Gglean008510	0.003852926	0.00028616	0.87357839	0.001478476
Gglean008511	0.003852926	0.00028616	0.87357839	0.001478476
Gglean008512	0	0	0	0.188727
Gglean008513	0	0	0	0
Gglean008514	0	0	0	0
Gglean008515	15.3731	20.2527	20.0502	19.9808
Gglean008516	3.65929	2.89652	6.94475	6.72005
Gglean008517	72.9401	60.3079	55.096	57.5622
Gglean008518	9.57772	10.27112252	9.419119	10.05157751
Gglean008519	25.06704	28.6253199	50.3238217	55.72194384
Gglean008520	31.55766147	27.42340648	38.48832	44.14901
Gglean008521	0.117874	0.0814741	0.12797	0.265152
Gglean008522	0.281096	0	0.465801	0.835741
Gglean008523	105.2642641	127.452662	174.2672644	197.4330306
Gglean008524	69.64510469	100.0324718	69.00810724	72.8096903
Gglean008525	15.96198218	14.16432199	15.5607873	14.03550813
Gglean008526	0.0731834	5.55E-06	0.0764997	0.214474
Gglean008527	0.0918841	0	0.242589	0
Gglean008528	0	0	0.720063	0
Gglean008529	39.8493998	46.14468	55.11567672	60.96275385
Gglean008530	39.8493998	46.14468	55.11567672	60.96275385
Gglean008531	27.867118	29.16332228	15.6681063	12.05909885
Gglean008532	27.867118	29.16332228	15.6681063	12.05909885
Gglean008533	6.51469	11.7428	12.4693	15.3262
Gglean008534	0	0	0	0
Gglean008535	1.706211123	1.228883389	2.2775218	2.28531287

Gglean008536	0	0	0	0.10784709
Gglean008537	5.86942	4.01042	7.36603	5.75795
Gglean008538	6.948737	6.156106	4.8972838	4.629259
Gglean008539	20.264571	19.493207	32.46195462	34.55419387
Gglean008540	0.0413015	0	0	0.0794015
Gglean008541	0.183145	0.0473996	0.148983	0.0440751
Gglean008542	0	0	0	0.300613
Gglean008543	0	0	0	0
Gglean008544	0	0	0	0
Gglean008545	0	0	0	0
Gglean008546	12.7407873	12.54366	21.0921	22.87378
Gglean008547	0.311013546	0.710205619	0.228511342	0.86787617
Gglean008548	0	0	0	0
Gglean008549	9.215603414	9.48545376	9.8641534	12.86958981
Gglean008550	113.7201261	127.3112074	109.1344474	112.3133449
Gglean008551	328.8881891	332.1781973	139.2885751	83.51909992
Gglean008552	1.404380249	0.951373037	1.433256124	1.032572047
Gglean008553	0	0	0	0
Gglean008554	0.839791	0.222993	1.71267	2.05622
Gglean008555	190.1706	343.8832	353.5322	470.3066
Gglean008556	190.1706	343.8832	353.5322	470.3066
Gglean008557	3.53342	3.92212	5.35406	5.81609
Gglean008558	0	0	0	0
Gglean008559	0.572001	0	0	0.185936
Gglean008560	10.59468825	12.25784995	10.88907914	10.53676506
Gglean008561	10.59468825	12.25784995	10.88907914	10.53676506
Gglean008562	10.59468825	12.25784995	10.88907914	10.53676506
Gglean008563	0	0	0	0.0723663
Gglean008564	0	0	0.190496	0
Gglean008565	0.0521261	0	0	0
Gglean008566	0.503627201	0.299424676	0.1931416	0.402389799
Gglean008567	0	0	0.308	0
Gglean008568	1.39713	1.2264	1.56141	1.46585
Gglean008569	6.7256852	7.968837521	10.47608122	11.53236244
Gglean008570	40.5134	40.3549	23.6779	21.1975
Gglean008571	28.28845	26.78948	31.54534	28.42212
Gglean008572	15.284059	15.133381	13.795227	14.79325
Gglean008573	0	0	0	0
Gglean008574	3.75259	2.93332	3.89993	5.02377
Gglean008575	0	0	0	0
Gglean008576	11.56807809	7.949497546	7.032449	5.305748965
Gglean008577	11.56807809	7.949497546	7.032449	5.305748965
Gglean008578	1.41427	0.821279	3.62154	3.8529
Gglean008579	55.4836	59.44966	34.16263	22.944493

Gglean008580	55.4836	59.44966	34.16263	22.944493
Gglean008581	55.59691	53.31764	39.75145	37.86074
Gglean008582	6.280391234	5.057651067	8.348984533	9.397453
Gglean008583	6.280391234	5.057651067	8.348984533	9.397453
Gglean008584	0	0	0	0
Gglean008585	0	0	0	0
Gglean008586	22.15150348	24.85382331	33.745826	38.788929
Gglean008587	22.15150348	24.85382331	33.745826	38.788929
Gglean008588	30.18890011	33.839616	47.60699139	58.19884
Gglean008589	3.700628466	3.791329731	4.562139	5.51201032
Gglean008590	34.1174031	37.20571617	62.58737503	58.52160485
Gglean008591	3.80335	2.25551	1.55954	2.3479
Gglean008592	11.2389	12.764	4.16306	6.41522
Gglean008593	79.58211762	81.25216775	91.893907	84.95533711
Gglean008594	1.87298	1.341381	1.802824	1.593267
Gglean008595	0.656238254	0.428489528	0.185255102	0.957778577
Gglean008596	0.656238254	0.428489528	0.185255102	0.957778577
Gglean008597	0.565577931	0.798721	0.759182777	0.990388074
Gglean008598	0	0	0	0.100587
Gglean008599	0	0	0	0
Gglean008600	0	0	0	0
Gglean008601	18.120546	12.511031	18.61546	29.94134
Gglean008602	18.120546	12.511031	18.61546	29.94134
Gglean008603	0	0	0	0
Gglean008604	51.1229	49.0627	29.0774	25.9102
Gglean008605	0.381495	0.684589	1.08583	1.74544
Gglean008606	16.43439303	14.215592	11.589949	11.338191
Gglean008607	0	0	0	0
Gglean008608	0	0	0	0.025095882
Gglean008609	4.8711961	6.1072258	5.56561308	6.046532018
Gglean008610	0.365020578	0.2113156	0.177690853	0.105373671
Gglean008611	40.7627	35.7297	20.58431	17.49707
Gglean008612	5.179433204	7.232263	11.20652507	12.76731218
Gglean008613	10.93914	11.06594	10.427432	15.38831
Gglean008614	10.91603	10.55117973	11.612559	13.47688
Gglean008615	35.97138296	36.53613002	20.532858	17.799892
Gglean008616	16.34769	19.84271	14.58722	14.56881
Gglean008617	8.84508	8.45204	4.17682	4.34758
Gglean008618	10.7674	14.97973	17.47275	18.76733
Gglean008619	14.00298461	12.81572592	5.65940631	8.37691586
Gglean008620	16.1121	9.85998	5.43913	6.82383
Gglean008621	43.627	46.1496	28.60245	25.94927
Gglean008622	10.75783206	11.396787	17.439732	20.70447088
Gglean008623	12.82115568	12.08196961	16.894064	18.790525

Gglean008624	0	0	0	0
Gglean008625	0.590363	0	0	0
Gglean008626	34.29797375	39.81238747	53.89255672	51.09081513
Gglean008627	34.29797375	39.81238747	53.89255672	51.09081513
Gglean008628	0	0	0	0
Gglean008629	1.98908	1.64007	0.411227	0
Gglean008630	2.31E-39	0.689912	0.115088	0.136328
Gglean008631	0.100959	0.208045	0.165263	0.293456
Gglean008632	147.8748	154.1285	184.9902	244.489
Gglean008633	55.68766117	63.03576166	46.951532	67.96439434
Gglean008634	552.7316	469.4608	471.0547	532.7181
Gglean008635	26.69411209	29.45220968	16.96677184	18.82356449
Gglean008636	4.402766	5.727166	9.792907	9.691595
Gglean008637	4.402766	5.727166	9.792907	9.691595
Gglean008638	44.58691388	40.87179476	38.172152	27.09233313
Gglean008639	42.84111302	38.12128925	46.65771817	61.5069208
Gglean008640	6.370871745	7.527001	7.90759217	9.353747
Gglean008641	0	0	0	0
Gglean008642	0	0	0	0
Gglean008643	0	0	0	0
Gglean008644	0.108982405	0.09614118	0.0455272	0.814415754
Gglean008645	0.108982405	0.09614118	0.0455272	0.814415754
Gglean008646	0.108982405	0.09614118	0.0455272	0.814415754
Gglean008647	0	0	0	0
Gglean008648	60.09104	67.23332	16.73322	14.58294
Gglean008649	17.97341	19.75621	9.3796	8.30385
Gglean008650	0.242224	0.250637	0.197374	0
Gglean008651	13.84671	10.64858	7.92477	7.22354
Gglean008652	111.671171	94.080511	92.865157	102.455475
Gglean008653	130.178182	141.2537441	117.8222467	103.7856056
Gglean008654	50.864792	54.135005	47.33565	43.265626
Gglean008655	16.73094635	15.32004878	12.73329058	13.85468107
Gglean008656	0.346048733	0.958010605	0.892547212	1.225520587
Gglean008657	3.43781	5.19432	5.764402153	9.46398
Gglean008658	63.93893881	73.72097582	69.61800221	68.12175503
Gglean008659	21.5289	18.906	29.0276	42.672
Gglean008660	0	0	0	0
Gglean008661	0	0	0	0
Gglean008662	5.91079	6.48616	4.6616	3.85238
Gglean008663	8.51172	9.59532	12.72495	15.69098
Gglean008664	17.74511499	19.33256626	10.7444721	9.688864
Gglean008665	48.20996	46.403685	62.53185	63.29238
Gglean008666	0	0	0	0
Gglean008667	39.94455293	57.04557215	53.04981812	79.77494806

Gglean008668	184.3897	166.57222	258.74005	287.2267
Gglean008669	0	0.054926373	0	0
Gglean008670	16.41665	14.64832	32.38041	34.77929
Gglean008671	1.181374821	0.948172	1.155907	1.137214814
Gglean008672	1.24574	2.025513184	1.045154	0.538423478
Gglean008673	96.4556	95.1575	81.6946	70.848
Gglean008674	65.851195	79.73969	77.35559	78.95516
Gglean008675	65.851195	79.73969	77.35559	78.95516
Gglean008676	0.57666655	0.558668844	0.438399817	0.521317395
Gglean008677	0.292221612	0.115342892	0.125111128	0.140965995
Gglean008678	0.209545	0.183031	0.169868	0.202754
Gglean008679	6.30059	7.00148	11.43552	11.9728
Gglean008680	1.376715737	2.936386	2.12838959	3.082678009
Gglean008681	1.376715737	2.936386	2.12838959	3.082678009
Gglean008682	2.240604013	2.69842	3.288879	4.596335
Gglean008683	170.0871467	164.0103157	194.0631323	178.5148574
Gglean008684	170.0871467	164.0103157	194.0631323	178.5148574
Gglean008685	7.07333	6.417263572	10.234495	11.5166516
Gglean008686	7.07333	6.417263572	10.234495	11.5166516
Gglean008687	197.59161	183.0649	277.2273	328.3615
Gglean008688	17.8949523	18.506213	25.981061	30.3123257
Gglean008689	17.8949523	18.506213	25.981061	30.3123257
Gglean008690	2.235326	1.973594	2.802597	3.74372
Gglean008691	0.581394589	0.80519952	1.003930108	0.981682421
Gglean008692	6.298233006	6.645668421	8.238610357	9.626814438
Gglean008693	6.298233006	6.645668421	8.238610357	9.626814438
Gglean008694	3.892500354	2.934512	4.556824	2.293179
Gglean008695	13.0221316	14.82431	4.45060075	4.092673
Gglean008696	13.0221316	14.82431	4.45060075	4.092673
Gglean008697	18.02787	18.968451	4.73889514	4.25765759
Gglean008698	18.02787	18.968451	4.73889514	4.25765759
Gglean008699	28.9341	32.7057	23.64244	22.10492
Gglean008700	0.770621	0.771215	1.386913	2.53447
Gglean008701	20.04117	22.04178	9.23013	9.04645
Gglean008702	74.88752008	73.07888959	45.799766	42.77327601
Gglean008703	74.88752008	73.07888959	45.799766	42.77327601
Gglean008704	74.88752008	73.07888959	45.799766	42.77327601
Gglean008705	26.544755	27.92288734	24.73105269	20.42572206
Gglean008706	36.4224	35.5299	21.70337	20.9154
Gglean008707	26.544755	27.92288734	24.73105269	20.42572206
Gglean008708	12.05767	12.290487	14.371128	14.645467
Gglean008709	7.736904316	7.87301	7.224314	9.163200672
Gglean008710	189.9466	225.9899	84.66879	67.6459
Gglean008711	25.34675	22.73177	14.64193	15.50768

Gglean008712	0	0	0	0
Gglean008713	11.78174	11.433326	11.17360947	11.94193993
Gglean008714	0.638254688	0	0.0562179	0.559305012
Gglean008715	0.638254688	0	0.0562179	0.559305012
Gglean008716	0.08417495	0.043371669	0.074994568	0.0926269
Gglean008717	0.16883613	0.12912168	0.2446044	0.315325135
Gglean008718	0.16883613	0.12912168	0.2446044	0.315325135
Gglean008719	2.106016	2.305327	1.214794	1.25343
Gglean008720	0	0	0	0
Gglean008721	0	0	0	0
Gglean008722	19.34231073	17.91788604	3.651857109	3.481145786
Gglean008723	37.271366	41.53614402	19.239	17.037511
Gglean008724	37.271366	41.53614402	19.239	17.037511
Gglean008725	2.71062	1.91687	2.3869	1.84646
Gglean008726	36.3233857	36.96128046	30.017789	29.39154984
Gglean008727	11.00880334	10.271563	13.81563088	16.07049822
Gglean008728	0	0	0	0
Gglean008729	51.76168476	50.13941633	48.23014	44.38876
Gglean008730	51.76168476	50.13941633	48.23014	44.38876
Gglean008731	59.4079	61.0156	76.4843	83.6947
Gglean008732	117.6979	118.73067	123.2864	132.8111
Gglean008733	0.075343	0.114464	0.0303385	0
Gglean008734	16.10787	17.00407	20.64059	16.95368
Gglean008735	23.58742839	21.2112234	24.34091	30.61831
Gglean008736	2.435904	2.330257	2.186987	2.439731492
Gglean008737	2.435904	2.330257	2.186987	2.439731492
Gglean008738	0.354182	0.373539	0.721839	0.171933
Gglean008739	2.078038722	2.429793878	2.613454916	2.146525
Gglean008740	8.641324002	5.9302842	3.033702245	3.324658569
Gglean008741	8.641324002	5.9302842	3.033702245	3.324658569
Gglean008742	3.705919965	9.17221	4.26451	6.55276
Gglean008743	17.02206403	18.45933436	10.76501661	10.40673187
Gglean008744	25.7871	29.2054	18.5339	19.6317
Gglean008745	19.49313	18.43679	20.87882	22.38147
Gglean008746	53.37853945	50.72874848	55.23611011	60.54537831
Gglean008747	4.63051	5.72091	6.8942	5.27793
Gglean008748	168.095	812.211	638.727	920.081
Gglean008749	15.67069	16.971547	16.36363	16.438721
Gglean008750	26.45889806	28.44893418	48.07803697	45.874937
Gglean008751	26.45889806	28.44893418	48.07803697	45.874937
Gglean008752	0	0	0	0
Gglean008753	0	0	0	0
Gglean008754	0.0861995	0	0.069413	0
Gglean008755	0	0.146906	0	0.135246

Gglean008756	0	0	0	0
Gglean008757	0	0	0.0673085	0
Gglean008758	0.336981	0.348437	0	0.162389
Gglean008759	0	0	0	0
Gglean008760	3.32578	5.10038	7.19794	8.45118
Gglean008761	36.15767	42.56097	38.67434	56.752647
Gglean008762	4.15476839	3.491231909	5.374109986	6.055973
Gglean008763	3.951350903	3.60178359	2.071455379	2.29665762
Gglean008764	133.4419	132.7569	31.329087	23.60216
Gglean008765	7.16773	9.36131	12.63704	11.60759
Gglean008766	37.58925238	39.39153766	27.24609263	23.04532812
Gglean008767	37.58925238	39.39153766	27.24609263	23.04532812
Gglean008768	3.03218	2.66732	1.64662	2.25904
Gglean008769	18.19612	19.03637	16.26109	17.33699
Gglean008770	18.19612	19.03637	16.26109	17.33699
Gglean008771	0	0	0	0
Gglean008772	0	0	0	0
Gglean008773	20.03926709	21.120422	21.53242449	22.06564745
Gglean008774	6.58947	10.34161	9.37526	11.873
Gglean008775	0	0	0	0
Gglean008776	79.6153	77.6619	60.1407	60.184249
Gglean008777	20.09202	19.95951	18.26414	17.02441
Gglean008778	20.09202	19.95951	18.26414	17.02441
Gglean008779	6.89E-10	0.00120453	2.78E-38	6.73E-27
Gglean008780	89.2162	82.0632	85.5883	107.7
Gglean008781	32.97617	33.892052	8.661183473	6.179129
Gglean008782	0	0	0	0
Gglean008783	0	0	0	0
Gglean008784	0	0	0	0
Gglean008785	0	0	0	0
Gglean008786	5.91102	6.03758	4.70525	3.99102
Gglean008787	62.6007	61.5263	58.2061	62.6465
Gglean008788	28.936955	22.479236	36.683836	44.471576
Gglean008789	0	0.069272575	0	0.032171405
Gglean008790	19.72025	15.32453	10.60581	8.361154
Gglean008791	133.0036	137.80341	87.51884	100.8891
Gglean008792	133.0036	137.80341	87.51884	100.8891
Gglean008793	1.401894	2.294823	1.864231	2.427708
Gglean008794	14.76744	12.8305	9.69621	9.93899
Gglean008795	102.9069641	88.78102195	99.03703116	113.77208
Gglean008796	102.9069641	88.78102195	99.03703116	113.77208
Gglean008797	13.90621	17.8204	6.62988	4.87663
Gglean008798	4.39E-05	9.53E-08	5.82E-07	0
Gglean008799	78.91968	86.66436	36.854765	32.665341

Gglean008800	0	0	0	0
Gglean008801	0	0	0	0
Gglean008802	0	0	0	0
Gglean008803	0	0	0	0
Gglean008804	0.166434	0.174682	0	0
Gglean008805	4.11458	7.0521	6.81877	5.11484
Gglean008806	0	0	0	0
Gglean008807	0.068758	0	0.0564963	0
Gglean008808	0	0	0.0594478	0
Gglean008809	0	0	0	0
Gglean008810	2.735050485	2.35404318	3.012920553	3.947111004
Gglean008811	0.674771	1.082364142	1.4757106	0.795370889
Gglean008812	0.674771	1.082364142	1.4757106	0.795370889
Gglean008813	24.16610649	24.9482	22.35265398	24.118046
Gglean008814	12.42383585	10.20214847	10.58578518	10.3206098
Gglean008815	12.42383585	10.20214847	10.58578518	10.3206098
Gglean008816	53.92351	60.7838	61.02805	61.2992
Gglean008817	0	0	0	0
Gglean008818	0	0	0	0.0783472
Gglean008819	0	0	0	0
Gglean008820	0	0	0	0
Gglean008821	5.107418081	5.827143908	8.092366	8.26693858
Gglean008822	27.07179	29.24984	39.5278	49.4212
Gglean008823	0	0	0	0
Gglean008824	0	0	0	0
Gglean008825	0	0	0	0
Gglean008826	0	0.0693426	0	0.0633296
Gglean008827	0	0	0	0
Gglean008828	45.40431128	38.93251	61.074362	63.986721
Gglean008829	45.40431128	38.93251	61.074362	63.986721
Gglean008830	22.828913	25.13362181	40.239683	42.432794
Gglean008831	22.828913	25.13362181	40.239683	42.432794
Gglean008832	22.828913	25.13362181	40.239683	42.432794
Gglean008833	12.556735	12.93409016	18.598368	21.152225
Gglean008834	12.556735	12.93409016	18.598368	21.152225
Gglean008835	23.3196	25.8818	6.62322	6.21858
Gglean008836	16.265628	16.381079	22.55359867	26.544291
Gglean008837	0	0	0	0
Gglean008838	55.9803	56.505	43.7599	39.4432
Gglean008839	64.5684	59.43335	49.15008	43.159
Gglean008840	29.74252447	31.80496856	27.491271	24.88723277
Gglean008841	29.74252447	31.80496856	27.491271	24.88723277
Gglean008842	32.36972	35.68265	19.34618	17.203
Gglean008843	2.34419	0.0088351	1.17226	0.683357

Gglean008844	50.97752	49.51202	34.67572	30.6309
Gglean008845	29.7434	24.9704	15.0104	11.0559
Gglean008846	18.29283	20.76799	28.8238	30.13171
Gglean008847	18.29283	20.76799	28.8238	30.13171
Gglean008848	12.45746098	12.55365835	7.30387991	6.147547959
Gglean008849	39.8728	32.6846	35.425889	49.125
Gglean008850	39.8728	32.6846	35.425889	49.125
Gglean008851	7.82295	8.895218	4.207463	0.270663
Gglean008852	0	0.267946	0	0
Gglean008853	1.974900737	1.652810886	2.978200627	2.906600644
Gglean008854	34.71009	37.93397391	62.29742	27.5733201
Gglean008855	34.71009	37.93397391	62.29742	27.5733201
Gglean008856	9.372827698	9.14534	5.785255935	7.355244
Gglean008857	9.372827698	9.14534	5.785255935	7.355244
Gglean008858	8.82442	6.496491625	5.189354	4.481731362
Gglean008859	32.51277246	32.76955033	31.03727453	28.77201207
Gglean008860	175.4942	143.7689	100.1993	97.0739
Gglean008861	2.671600786	2.579369876	2.903229901	3.27537627
Gglean008862	11.47317174	10.73321	14.8278	18.11134
Gglean008863	30.2914	27.29431	31.7764	45.6725
Gglean008864	65.010556	59.14596603	75.011795	83.36179972
Gglean008865	65.010556	59.14596603	75.011795	83.36179972
Gglean008866	33.40261717	30.6623811	49.43691742	56.73222545
Gglean008867	118.0776454	131.6381145	81.21706246	78.0636141
Gglean008868	6.65837	7.087203	4.193194434	4.687924
Gglean008869	4.906283	5.64992	7.26676	9.760128
Gglean008870	138.8183	131.9256	118.8611	153.7838
Gglean008871	9.362654	9.87662	6.435826	5.739959027
Gglean008872	10.6176	9.09132	7.39591	7.32283
Gglean008873	111.079079	129.323891	66.281228	66.304615
Gglean008874	111.079079	129.323891	66.281228	66.304615
Gglean008875	0	0	0	0
Gglean008876	3.48743	3.0961	3.37991	3.53547
Gglean008877	3.48743	3.0961	3.37991	3.53547
Gglean008878	61.3067	65.92132	27.95581	28.38006
Gglean008879	19.87358	18.62451	12.575911	11.35285
Gglean008880	5.461594	5.941552	3.168500947	3.287047
Gglean008881	12.065697	12.055532	24.87199	21.67475
Gglean008882	41.98565285	38.365854	12.45840131	6.376065821
Gglean008883	0	0	0	0
Gglean008884	100.6650787	101.5092434	126.3871281	192.4011118
Gglean008885	1.64E-106	2.13E-45	8.67E-36	2.038515337
Gglean008886	1.64E-106	2.13E-45	8.67E-36	2.038515337
Gglean008887	0.973925744	1.41917161	1.138483432	1.595206

Gglean008888	0	0	0	0
Gglean008889	0	0	0.045899042	0
Gglean008890	0	0	0	0
Gglean008891	3.1757	2.75893	6.09743	6.93745
Gglean008892	10.74199929	8.83387259	6.556169257	7.572702854
Gglean008893	10.74199929	8.83387259	6.556169257	7.572702854
Gglean008894	0.08724451	0.08840469	0.320166397	0.14165123
Gglean008895	40.78863	43.66729	33.042444	34.876352
Gglean008896	6.81386	8.48555	10.6822	9.14492
Gglean008897	7.438071048	8.88009389	4.471856773	4.461879458
Gglean008898	26.72136608	26.70594484	30.24315629	33.53811182
Gglean008899	26.72136608	26.70594484	30.24315629	33.53811182
Gglean008900	26.72136608	26.70594484	30.24315629	33.53811182
Gglean008901	0.0728434	0.0382424	2.42E-84	0.2051628
Gglean008902	2.56134	0	3.19113	3.81481
Gglean008903	0	0	0	0
Gglean008904	18.22894	13.50295	12.42979	11.46755
Gglean008905	125.4300944	121.2430863	40.77243141	18.9053368
Gglean008906	76.54292	77.45839002	100.1774	91.20704181
Gglean008907	76.54292	77.45839002	100.1774	91.20704181
Gglean008908	28.20850356	30.19261108	51.92850597	52.08980911
Gglean008909	49.6555	50.1098	30.1668	19.9718
Gglean008910	2.74672	2.988121	3.867360029	3.24887
Gglean008911	47.56961478	45.21967819	28.12291382	26.0342305
Gglean008912	12.56564	16.40459	14.23519	13.96066
Gglean008913	27.9193	26.1502	11.47819	10.85278
Gglean008914	13.2058	16.1597	22.822	31.9751
Gglean008915	0	0	0	0
Gglean008916	15.76937	13.0262	11.63037	8.16957
Gglean008917	15.76937	13.0262	11.63037	8.16957
Gglean008918	24.66041	23.027638	34.27155	32.8464
Gglean008919	92.58297	103.51702	110.54704	117.82712
Gglean008920	46.3135	44.1998	43.8262	34.9747
Gglean008921	7.3216	4.60551	7.91256	12.0079
Gglean008922	0	0	0	0
Gglean008923	44.97819	46.3299	10.38586	7.62376
Gglean008924	44.97819	46.3299	10.38586	7.62376
Gglean008925	35.90102462	30.13329974	24.36811468	21.96924895
Gglean008926	38.19390233	39.11063742	50.49190079	51.64750072
Gglean008927	38.19390233	39.11063742	50.49190079	51.64750072
Gglean008928	14.92994	19.0714225	28.99213008	32.212948
Gglean008929	14.92994	19.0714225	28.99213008	32.212948
Gglean008930	28.83108	29.53569	37.101632	46.99782
Gglean008931	19.275	24.8133	26.5267	26.5895

Gglean008932	314.6580993	318.8030269	346.4619089	398.7029708
Gglean008933	44.2638	42.6434323	44.17637	45.85903
Gglean008934	0	0	0	0
Gglean008935	25.18943	21.5148	41.6552	44.32469
Gglean008936	35.80734388	31.03216319	50.0636523	57.32043
Gglean008937	6.21623	8.67988	10.62767	14.15008
Gglean008938	730.41769	717.48881	517.83534	452.99038
Gglean008939	3.50E-104	2.02E-121	0	7.42E-29
Gglean008940	58.7624	61.5196	43.5008	50.1291
Gglean008941	0.270676	0.768410724	1.103447	0.4959038
Gglean008942	42.9419628	44.03894488	37.333954	35.70563396
Gglean008943	40.90831	42.99227	28.03249	25.11116
Gglean008944	44.50361319	45.76682018	22.34370093	18.71314744
Gglean008945	44.50361319	45.76682018	22.34370093	18.71314744
Gglean008946	451.5007374	392.7463976	339.5561798	341.46434
Gglean008947	7.8043927	32.6165087	2.007638779	12.0521237
Gglean008948	70.044648	73.60804	78.92334	93.47092
Gglean008949	70.044648	73.60804	78.92334	93.47092
Gglean008950	31.36306	37.58226	46.71796	30.04012
Gglean008951	31.36306	37.58226	46.71796	30.04012
Gglean008952	63.97601258	66.45339518	39.97847785	32.33533714
Gglean008953	63.97601258	66.45339518	39.97847785	32.33533714
Gglean008954	30.3817	32.9123	19.9742	16.9084
Gglean008955	0	0.392291014	0	0
Gglean008956	63.99628777	66.14353713	92.35919573	87.18279008
Gglean008957	0	0	0	0
Gglean008958	0	0	0	0
Gglean008959	29.6573	31.278712	36.354334	36.4744
Gglean008960	29.6573	31.278712	36.354334	36.4744
Gglean008961	75.601002	74.510525	92.925648	89.464482
Gglean008962	27.953794	29.438844	22.28272	25.259677
Gglean008963	17.690125	17.97613198	9.64935438	7.985535606
Gglean008964	52.81881	53.74541	50.84331	52.74451
Gglean008965	30.42619	30.7157	27.3397	28.82219
Gglean008966	30.42619	30.7157	27.3397	28.82219
Gglean008967	30.42619	30.7157	27.3397	28.82219
Gglean008968	0.378594	0	0	0
Gglean008969	28.147626	30.69801103	28.02698405	28.764659
Gglean008970	12.32558	14.28737	13.44366	20.31296
Gglean008971	0	0	0	0
Gglean008972	0	0	0	0
Gglean008973	25.22579	21.67403	19.30129	21.48732
Gglean008974	0	0	0	0
Gglean008975	0	0	0	0

Gglean008976	0	0	0	0
Gglean008977	0	0	0	0
Gglean008978	0	0	0	0
Gglean008979	0	0	0	0
Gglean008980	0	0	0	0
Gglean008981	0	0	0	0.754519
Gglean008982	0	0	0	0
Gglean008983	0	0	0	0
Gglean008984	0	0.446751	0	0
Gglean008985	0	0	0.297633	0.53003
Gglean008986	6.08E-230	0.1812552	0.5159902	0.128876
Gglean008987	13.73787	14.37461	8.53225	7.63774
Gglean008988	25.166	24.0146	15.9248	24.0516
Gglean008989	95.66238	109.2178261	91.4799	102.40704
Gglean008990	12.4938	38.2268	39.9084	38.6655
Gglean008991	3.390530787	2.946310539	1.6391986	1.744921215
Gglean008992	49.67446	52.56248	34.95221	28.2957
Gglean008993	15.425144	26.579557	10.23998	9.62701
Gglean008994	13.996955	13.56216791	14.2204762	13.18000208
Gglean008995	28.6163	36.86091516	35.60265	40.67703
Gglean008996	0	0	0	0
Gglean008997	4.554532	4.400005	2.94237	2.359870366
Gglean008998	221.7957	321.989	11.1034	9.9288
Gglean008999	0	0	0	0
Gglean009000	2.716248	3.25839	6.06701	6.76746
Gglean009001	51.988	71.0939	56.6252	53.9919
Gglean009002	0.848729	1.39295	1.0555	1.26574
Gglean009003	0.0809313	0.0830516	0.261119	0.154679
Gglean009004	22.7386	19.58798	11.06928	13.42804
Gglean009005	0.864066	0.952064012	1.28327	1.27332
Gglean009006	1.28298296	1.776997842	2.236404001	1.978522162
Gglean009007	16.9761	17.1311	13.1129	14.5903
Gglean009008	33.66361677	34.1001098	13.43958015	11.25819812
Gglean009009	77.71729	82.293829	61.13368858	54.121369
Gglean009010	77.71729	82.293829	61.13368858	54.121369
Gglean009011	77.71729	82.293829	61.13368858	54.121369
Gglean009012	77.71729	82.293829	61.13368858	54.121369
Gglean009013	2.60304	3.45253	2.32491	6.11197
Gglean009014	878.3903087	872.7747146	1319.697108	1411.441206
Gglean009015	878.3903087	872.7747146	1319.697108	1411.441206
Gglean009016	3.50295	4.41162	4.84097	3.56433
Gglean009017	0	0	0	0
Gglean009018	57.6102	61.6092	50.717	46.1278
Gglean009019	77.65750312	79.77380373	61.86750339	59.87030317

Gglean009020	45.24067	51.2884	68.4281	57.41885
Gglean009021	13.618755	12.78915	12.80914525	15.05333306
Gglean009022	0.551647	0.473718	0.333638	0.549209
Gglean009023	8.8118175	9.184941	9.877794	11.62578
Gglean009024	8.8118175	9.184941	9.877794	11.62578
Gglean009025	0	0	0	0
Gglean009026	0	0	0	0.0704622
Gglean009027	1.41698	3.9751	2.47686	2.97409
Gglean009028	40.66549	38.56485	41.69949	43.19231
Gglean009029	10.827135	10.5053266	7.7365	6.795497
Gglean009030	2.66271	3.60927	1.97975	1.70683
Gglean009031	0.000443022	0.559958	1.87E-05	0.000351501
Gglean009032	4.27831	4.18556	4.30735	6.61982
Gglean009033	0.524915	0.136485	0	0
Gglean009034	0	0	0	0
Gglean009035	0	0	0	0
Gglean009036	18.54408937	21.66792558	11.75471	14.51359005
Gglean009037	18.54408937	21.66792558	11.75471	14.51359005
Gglean009038	10.26101001	9.812494	19.394993	17.49971
Gglean009039	186.1198858	155.1103762	418.267417	447.0200375
Gglean009040	31.318	6.45477	11.92384	41.73116
Gglean009041	31.1726609	32.11162742	39.46811736	34.97986143
Gglean009042	0	0	0	0
Gglean009043	29.735638	33.40088	23.7489	22.6643
Gglean009044	0	0	0.067117168	0.079513439
Gglean009045	0.107916419	0.300858186	0.177079661	0.525439579
Gglean009046	4.461635924	4.277439519	8.16528	8.119887605
Gglean009047	5.3626	4.193682885	6.063369	8.85397
Gglean009048	0	0	0	0
Gglean009049	0	0	0	0
Gglean009050	25.12317	22.92088	37.2696	45.2438
Gglean009051	3.00622	2.687641	2.032286	2.017392
Gglean009052	569.7347927	526.590669	362.2301345	348.726282
Gglean009053	0	0	0	0
Gglean009054	103.4212201	86.40803501	61.87482982	59.95382262
Gglean009055	103.4212201	86.40803501	61.87482982	59.95382262
Gglean009056	5.360550409	5.203134928	7.618480525	9.465256028
Gglean009057	0.65595287	1.054723	1.044441	1.546501
Gglean009058	0.65595287	1.054723	1.044441	1.546501
Gglean009059	23.808425	24.38333	25.61981	24.32795
Gglean009060	0	0	0	0
Gglean009061	20.453255	24.46783	17.873093	14.82454
Gglean009062	0	0	0	0
Gglean009063	13.41221924	9.959284182	19.84399	28.65821

Gglean009064	8.57755	8.94282	10.9001	18.0286
Gglean009065	0.534288341	0.787504294	0.4832112	1.039893184
Gglean009066	1.3714115	0.960799197	1.120834362	0.905591813
Gglean009067	1.3714115	0.960799197	1.120834362	0.905591813
Gglean009068	32.21573	28.01855	30.67743	30.18995
Gglean009069	0	0	0	0
Gglean009070	202.69134	197.5664091	191.461802	263.54264
Gglean009071	0.357482	5.28E-05	3.70E-05	0.000208216
Gglean009072	0	0	0	0
Gglean009073	0	0.35429	0	0
Gglean009074	1.063043	1.137782	1.463185	1.759229
Gglean009075	1.454865052	1.748392313	2.542147088	3.093392473
Gglean009076	89.0472015	97.14515396	71.842219	77.2554994
Gglean009077	10.431846	11.637088	8.61444	7.810104
Gglean009078	0	0	0	0
Gglean009079	2.155602	2.313125	5.112292	3.075973
Gglean009080	0.0461357	0.0315403	0.0248693	0.117687
Gglean009081	0.231379	0	0.379869	0.112445
Gglean009082	0.0453592	0	0.334601	0.221334
Gglean009083	38.77502	36.6595	18.24047	14.45638
Gglean009084	0	6.28833	0	0.802352
Gglean009085	38.77502	36.6595	18.24047	14.45638
Gglean009086	7.11239	7.63152	7.09335128	9.64
Gglean009087	0	0.174654544	0.06881211	0.08153764
Gglean009088	0	0	0	0
Gglean009089	8.15398	8.18639	9.91686	9.82109
Gglean009090	167.6148209	123.0205225	107.5699775	123.421021
Gglean009091	26.68152	33.52404	15.32315	13.65289
Gglean009092	68.60206189	60.20128295	71.65348186	101.0541639
Gglean009093	4.764581	4.558981	6.078576	6.7645273
Gglean009094	2.91358	1.35808	2.2245	3.60759
Gglean009095	1.487540827	1.353372138	2.023706329	2.598353756
Gglean009096	2.14E-06	4.80E-06	4.23E-05	5.28E-05
Gglean009097	5.53015292	4.72480028	6.071881834	7.47466998
Gglean009098	0.95875269	0.960044	5.558530812	5.756083
Gglean009099	2.446316	2.029222	2.016694	1.3615836
Gglean009100	0	0	0	0
Gglean009101	0	0	0	0
Gglean009102	0	0.141135	0.089332	0.211613
Gglean009103	0.142802	0.434134	0.310553	0.409275
Gglean009104	79.62806976	84.35429267	61.01761759	57.28180528
Gglean009105	13.9146	17.102	10.6228	8.76818
Gglean009106	111.6063	111.4404	95.6256	85.7135
Gglean009107	0	0	0	0

Gglean009108	3.89477	4.18005	7.84232	7.19248
Gglean009109	8.82436	10.1757	10.1881	9.1936
Gglean009110	1.718272	1.183775	3.034075	2.214619
Gglean009111	0.801483	0.65775	0.194444	0.768887
Gglean009112	0	0	0	0
Gglean009113	2.374457	2.757065	3.794363	4.323375
Gglean009114	0	0	0	0
Gglean009115	0.831748	2.05803	2.47396	1.84686
Gglean009116	0	0	0	0
Gglean009117	0	0	0	0
Gglean009118	0.0343726	0.0349052	0	0
Gglean009119	0	0.104991	0.0822522	0
Gglean009120	177.701	170.824	208.899	311.631
Gglean009121	9.86E-06	0	1.83E-18	1.33E-06
Gglean009122	3.77918	3.75082	1.96764	2.09107
Gglean009123	402.5396462	406.5653877	416.5486608	392.2154603
Gglean009124	71.13952	66.9126609	75.67985984	71.30697928
Gglean009125	0	0	0	0
Gglean009126	59.8717	53.578313	70.867075	68.835624
Gglean009127	10.4136	11.90100002	16.6578	18.0656
Gglean009128	4.55742	3.910649921	3.87175	2.1351422
Gglean009129	13.142201	12.7060605	16.60046	16.61714
Gglean009130	24.3504	41.0059	4.69335	5.84393
Gglean009131	15.77662	12.977829	23.48483	26.87451
Gglean009132	94.0781	98.1095	139.5181	160.3353
Gglean009133	26.72378088	25.650765	40.271622	43.24816
Gglean009134	0	0.53512	0.790666	1.40545
Gglean009135	0.307299899	0.306570529	0.617664682	0.541433559
Gglean009136	0	0	0.055657683	0
Gglean009137	0	0	0.055657683	0
Gglean009138	0	0	0	0
Gglean009139	1.164625864	2.813593866	1.565677871	1.989459881
Gglean009140	1.164625864	2.813593866	1.565677871	1.989459881
Gglean009141	0.639244679	0.545238	1.221726	1.356911
Gglean009142	0	0	0	0
Gglean009143	0	0	0	0
Gglean009144	0	0	0	0
Gglean009145	0	0	0	0
Gglean009146	0.0857091	0	0	0
Gglean009147	39.714032	39.357357	29.783222	31.4553974
Gglean009148	39.714032	39.357357	29.783222	31.4553974
Gglean009149	0	0	0	0
Gglean009150	4.611932336	2.673257673	2.621779189	2.284293175
Gglean009151	4.611932336	2.673257673	2.621779189	2.284293175

Gglean009152	8.278331022	8.133031053	5.417417094	4.695210003
Gglean009153	0.123386518	0.049732734	0.076683274	0.046035374
Gglean009154	0	0.0457545	0	0.0839446
Gglean009155	0.038693046	0	0	0
Gglean009156	0.0813978	0.255168	0.0660996	0.157003
Gglean009157	0.27203	0.291527	0	0
Gglean009158	26.6886	23.03159	17.83808	14.40085
Gglean009159	25.30113	22.90061	14.67354	14.04846
Gglean009160	9.327313	9.953093	8.605427	9.95018
Gglean009161	1.883306	1.826724	1.906463	3.76481
Gglean009162	10.057462	9.601379	13.674238	17.275232
Gglean009163	65.359084	78.2231	26.210432	24.335081
Gglean009164	12.9636	15.6669	11.806	16.0241
Gglean009165	57.69576	63.8593	61.22832549	62.56157
Gglean009166	75.04849	74.6971	66.58775	69.09526
Gglean009167	8.77488	7.62241	5.91562	4.90397
Gglean009168	52.221049	60.50553	47.26537	46.4020564
Gglean009169	26.045387	24.7344759	28.722354	26.6543
Gglean009170	26.045387	24.7344759	28.722354	26.6543
Gglean009171	26.045387	24.7344759	28.722354	26.6543
Gglean009172	54.83767	61.839	45.135379	44.012
Gglean009173	54.83767	61.839	45.135379	44.012
Gglean009174	44.6695	93.8162	115.842	133.387
Gglean009175	4.76455	6.02007	8.62607	11.0117
Gglean009176	29.9425	20.7011	11.1034	10.6824
Gglean009177	70.9062	83.7796	44.77186	17.67248
Gglean009178	27.4941861	24.7256	34.34761976	29.135998
Gglean009179	18.48900442	19.81067	30.68686	34.8119
Gglean009180	0	0	0	0
Gglean009181	12.92961044	13.84203265	24.05345946	25.57084911
Gglean009182	21.523519	19.64446	20.622709	20.63367
Gglean009183	69.794958	71.92270042	39.96676067	38.58046699
Gglean009184	5.687031375	5.071534688	3.209730559	2.631910749
Gglean009185	5.687031375	5.071534688	3.209730559	2.631910749
Gglean009186	1.81276	2.99521	1.12458	0
Gglean009187	39.15660325	36.07009066	49.15071206	51.75507108
Gglean009188	32.85136	37.1759	37.05632	39.7498
Gglean009189	11.11067	15.16437	9.30567	11.92253
Gglean009190	32.73923	33.35218	35.87133	34.28153
Gglean009191	76.31512013	88.786776	125.895248	125.03125
Gglean009192	0.272939	0.302401	0.332757	0.658099
Gglean009193	1.3437058	0.6109322	0.458233424	0.427692345
Gglean009194	7.986442	8.68088	12.55375	14.13413
Gglean009195	16.75669	16.76627	13.63733	12.85812

Gglean009196	35.91356455	34.888786	29.90001765	27.14925636
Gglean009197	97.0617	105.5225	63.8952	53.434
Gglean009198	22.86926	26.63751	24.68091	25.31415045
Gglean009199	0.937888	0.962003	0.170397799	0.438994257
Gglean009200	190.1911	208.5991	119.5718	92.3531
Gglean009201	0.2848141	0.255403671	0.595424527	0.816211021
Gglean009202	35.39369003	36.15899643	27.70295329	30.91746
Gglean009203	4.98059774	3.901005282	7.005744679	7.409626373
Gglean009204	4.98059774	3.901005282	7.005744679	7.409626373
Gglean009205	177.884113	201.8273295	189.8131	206.0068446
Gglean009206	292.279	299.163	279.946	322.745
Gglean009207	58.84952	52.24464	56.937	59.68796
Gglean009208	0	0	0	0
Gglean009209	0.0416578	0	0.0671645	0.0397973
Gglean009210	0	0	0	2.14002
Gglean009211	2.348573081	2.395181436	2.298607918	1.11459876
Gglean009212	8.893948	8.52718	5.552353584	5.75316
Gglean009213	0	0	0	0
Gglean009214	0	0	0.245773	0
Gglean009215	0.178104	0.138401	0.0724235	0.0430023
Gglean009216	0.488453	0	0	0
Gglean009217	0.121232	0.187527	0.0489883	0.11608
Gglean009218	0.0795258	0	0.130268	0.154127
Gglean009219	9.19742	6.75313	5.11114	4.45347
Gglean009220	0	0	0	0
Gglean009221	0	0	0	0
Gglean009222	0	0	0	0
Gglean009223	0	0	0	0
Gglean009224	0.112493	0.172675	0.349228	0.2159
Gglean009225	0	0	0	0
Gglean009226	0	0	0	0
Gglean009227	0.0605623	0	0	0
Gglean009228	0.0476622	0.143838	0.190821	0
Gglean009229	0.415938	0.32863	0.257997	0.508533
Gglean009230	0	0	0	0
Gglean009231	1.2341	1.31365	0.611473	0.973658
Gglean009232	0.0339754	0	0	0
Gglean009233	0	0	0	0
Gglean009234	5.26218	3.78281	0.37803	1.81536
Gglean009235	0	0.068254523	0	0
Gglean009236	79.89171	85.9286	55.71478	47.09987
Gglean009237	30.22538566	31.98640169	10.70775	9.312440717
Gglean009238	0.047950929	0.097970673	0.077278381	0
Gglean009239	0	0	0	0

Gglean009240	0.848371	0.947615	4.22814	4.23162
Gglean009241	31.9246	30.4252	19.09561	19.57441
Gglean009242	0.06921154	0	0	0
Gglean009243	50.41295554	49.3912354	44.30436064	48.0374471
Gglean009244	50.41295554	49.3912354	44.30436064	48.0374471
Gglean009245	50.41295554	49.3912354	44.30436064	48.0374471
Gglean009246	37.13451	53.94222	53.3944	71.26388
Gglean009247	13.996557	14.493271	5.68779683	4.752755051
Gglean009248	68.877904	68.23612	60.5629	59.92763
Gglean009249	37.1002	35.263	31.9619	31.5814
Gglean009250	52.1501188	53.299758	16.699752	14.90800256
Gglean009251	0	0	0	0
Gglean009252	161.915	223.351	148.081	408.766
Gglean009253	20.2642	18.5027	16.7087	16.058
Gglean009254	30.63945882	31.07341949	29.33618	25.6704471
Gglean009255	133.645	135.771	86.4471	87.341
Gglean009256	36.72230084	38.41484694	24.92280477	21.571655
Gglean009257	21.50075	20.04413	12.93916	11.601184
Gglean009258	1.410988	0.9975883	0.842478089	0.825884
Gglean009259	60.270227	58.62890362	51.91587446	48.48654901
Gglean009260	60.270227	58.62890362	51.91587446	48.48654901
Gglean009261	60.270227	58.62890362	51.91587446	48.48654901
Gglean009262	5.95547	6.117045	1.881326	3.237169
Gglean009263	29.03803	27.50352	33.28208	34.30639
Gglean009264	5.95547	6.117045	1.881326	3.237169
Gglean009265	71.84004	78.29000933	74.725854	70.59212
Gglean009266	71.84004	78.29000933	74.725854	70.59212
Gglean009267	18.49703	24.51707	5.46162	4.56068
Gglean009268	22.6707321	23.0834458	7.66655	6.855434138
Gglean009269	8.62807316	9.071456	5.215861	4.423432
Gglean009270	20.37925	20.94746	14.45631	14.45961
Gglean009271	0	0	0	0
Gglean009272	0.264667	0.559865	0.457545	1.284883
Gglean009273	34.43866	35.17614	26.45109	25.6477189
Gglean009274	0	0	0	0
Gglean009275	0	0	0	0
Gglean009276	0	0	0	0
Gglean009277	17.043299	15.131	16.415526	19.398282
Gglean009278	20.267384	19.51404	14.18794	14.99989
Gglean009279	2.18382	1.62284	1.27875	2.17786
Gglean009280	50.4301733	52.7083	48.7608	45.7693
Gglean009281	20.4158	19.4486	10.4257719	10.3879
Gglean009282	7.16273	7.26363	5.77527	6.73436
Gglean009283	15.408679	17.828579	7.047422	4.921097

Gglean009284	0	0	0	0
Gglean009285	79.0304	76.9685	19.2253	14.2636
Gglean009286	7.23139	6.73903	9.32575	8.24339
Gglean009287	0.134087	0	0	0
Gglean009288	8.6023	8.95434	11.9658	12.58663
Gglean009289	0	0	0	0
Gglean009290	698.30764	748.33449	710.092	1226.07109
Gglean009291	11.51021137	11.17738672	16.36045754	20.34074951
Gglean009292	0.319592	0.0835393	0.130479	0.232194
Gglean009293	0.506773	0.210591	0.4982	0.394492
Gglean009294	0.38864	0.722909	0.380036	0.452861
Gglean009295	0.179615	0.184891	0.0724069	0.0856771
Gglean009296	22.993255	24.038043	20.17505	21.208025
Gglean009297	21.203176	18.66685166	21.84691074	22.1803114
Gglean009298	27.21995	21.82513	19.07716	18.831
Gglean009299	13.02268281	12.68348518	16.72102304	20.55881215
Gglean009300	13.02268281	12.68348518	16.72102304	20.55881215
Gglean009301	13.02268281	12.68348518	16.72102304	20.55881215
Gglean009302	0	0	0.196619	0
Gglean009303	0.758048926	0.0603278	0.112582	0.0947013
Gglean009304	0.2504482	0.134642336	0.360436311	0.293605798
Gglean009305	0.2504482	0.134642336	0.360436311	0.293605798
Gglean009306	0.2504482	0.134642336	0.360436311	0.293605798
Gglean009307	11.5591	19.87345	12.09233	13.73795
Gglean009308	1.8848267	1.992625471	3.102379687	3.684247
Gglean009309	1.735165811	0.928926869	4.839949935	4.577831316
Gglean009310	6.42925	7.05732	4.90561	5.24509
Gglean009311	33.11755519	35.834551	21.523979	18.141849
Gglean009312	13.18986501	15.59801721	21.72401689	22.65801743
Gglean009313	28.27035	27.73764	16.57083	14.78685
Gglean009314	39.94399	41.928871	37.075443	38.01092
Gglean009315	39.94399	41.928871	37.075443	38.01092
Gglean009316	39.94399	41.928871	37.075443	38.01092
Gglean009317	0	0	0	0
Gglean009318	0.317337	1.69E-05	0.102872	0.00030582
Gglean009319	0	0	0	0
Gglean009320	0	0	0	0
Gglean009321	0	0	0	0
Gglean009322	16.1607	16.97157	19.04495	16.03623
Gglean009323	0	0	0	0
Gglean009324	0	0	0	0
Gglean009325	0	0	0	0
Gglean009326	0	0	0	0
Gglean009327	0	0	0	0

Gglean009328	2.13E-06	6.59E-06	3.52E-06	2.11E-06
Gglean009329	0.951011	0.886866	0.55661	0.444424
Gglean009330	0	0	0	0
Gglean009331	0	0	0	0
Gglean009332	0	0	0	0
Gglean009333	0	0	0	0
Gglean009334	0	0	0	0
Gglean009335	0	0	0	0
Gglean009336	0	0	0	0
Gglean009337	0	0	0	0
Gglean009338	0	0	0	0
Gglean009339	0	0	0.0701677	0
Gglean009340	0	0	0	0
Gglean009341	309.2768	316.8221	309.2854	366.6112
Gglean009342	309.2768	316.8221	309.2854	366.6112
Gglean009343	309.2768	316.8221	309.2854	366.6112
Gglean009344	1.96542	2.92192	1.63672	7.19075
Gglean009345	2.2563	1.66625	6.23244	5.99318
Gglean009346	33.206	33.9448	17.6226	14.6271
Gglean009347	0	0	0	0
Gglean009348	0.526731	0.335147	0.383384	0.261753
Gglean009349	0	0.585733	0.446389	0
Gglean009350	0	0	0	0
Gglean009351	0.117608	0.242403	0.0796281	0.0749546
Gglean009352	29.20121	30.37452	30.43089	33.40534
Gglean009353	29.20121	30.37452	30.43089	33.40534
Gglean009354	1.65169	1.46793	1.40966	1.61043
Gglean009355	18.2821	16.56213	11.38005	10.45497
Gglean009356	19.0991	20.6971	10.5237	9.31078
Gglean009357	1080.09668	1140.967505	674.2365114	643.9764136
Gglean009358	2.590159	2.4107401	2.836104	2.793954668
Gglean009359	14.127454	14.28348042	8.686481409	8.078289
Gglean009360	25.52286	22.57104	13.32286	13.90154
Gglean009361	14.4547	18.0877	22.329	27.8071
Gglean009362	73.32155	77.02096	57.85537	51.63259
Gglean009363	6.413570026	10.07493	2.502530031	3.261663
Gglean009364	6.413570026	10.07493	2.502530031	3.261663
Gglean009365	6.413570026	10.07493	2.502530031	3.261663
Gglean009366	26.77303	26.79253	16.120256	9.273274
Gglean009367	17.34011	19.85353001	21.55465	23.35192
Gglean009368	17.34011	19.85353001	21.55465	23.35192
Gglean009369	0	0.115526	0.177654	0.105513
Gglean009370	0.34523	0.237952	0	0.222245
Gglean009371	0.41179	0.869984	0.51153	0.608778

Gglean009372	131.0605782	131.5851018	111.0569159	104.7497137
Gglean009373	39.26395	43.60724	34.06369	31.40652
Gglean009374	14.57370716	15.27505419	17.96501084	16.90572859
Gglean009375	29.79262	26.26787	12.72827	9.89364
Gglean009376	29.79262	26.26787	12.72827	9.89364
Gglean009377	37.3552824	39.19945	47.38235	45.13992
Gglean009378	1.20356	1.96492	2.34291	3.63957
Gglean009379	10.42143	12.09256	6.92448	6.6748
Gglean009380	21.7621	21.72412	16.61944	17.60856
Gglean009381	52.51760198	46.3574	23.44890374	21.35744
Gglean009382	52.51760198	46.3574	23.44890374	21.35744
Gglean009383	16.6253	21.565	10.2277	6.55898
Gglean009384	1.36347	1.3043	2.24361	1.96195
Gglean009385	1.36347	1.3043	2.24361	1.96195
Gglean009386	0.705264	0	0	0
Gglean009387	0	0	0	0
Gglean009388	0	0	0	0
Gglean009389	0	0.13027	0.205538	0
Gglean009390	11.84713	11.90318	2.75243	1.330645
Gglean009391	0.805586	1.37417	1.96266	1.77819
Gglean009392	0.606467	0.216549	0	0
Gglean009393	0	0	0	0
Gglean009394	1.906988065	2.047634121	1.86435851	1.628183205
Gglean009395	0	0	0	0
Gglean009396	0	0	0.0257848	0
Gglean009397	0.0649562	0.0671677	0	0.0624939
Gglean009398	12.9869	12.7759	17.4192	17.4973
Gglean009399	2.47413	1.30371	0.868628	2.76016
Gglean009400	8.04955	8.24737	9.1188	14.19699
Gglean009401	58.43656	58.13419	36.32975	30.46169
Gglean009402	48.04660053	52.786675	68.756258	72.3267
Gglean009403	48.04660053	52.786675	68.756258	72.3267
Gglean009404	2.201498	1.974667	0.655778	0
Gglean009405	19.16366528	16.60501927	16.87531562	17.68392668
Gglean009406	42.07945	40.82492681	66.96429561	71.01202223
Gglean009407	0	0	0.319894	0
Gglean009408	0	0	0	0
Gglean009409	37.69087691	37.31071684	44.34108532	64.79653504
Gglean009410	0	0	0	0
Gglean009411	5.99197	13.5291	24.2561	5.76683
Gglean009412	0.114815058	0	0.093252586	0
Gglean009413	0.0665904	0.0691256	0	0
Gglean009414	105.3013016	104.7152323	75.38108	71.991897
Gglean009415	0.695671	0.121528	1.50378	1.3472

Gglean009416	0	0.116475	0	0.216804
Gglean009417	0.336096	0.234819	0	0.984958
Gglean009418	4.01365	4.23561	3.54787	3.12998
Gglean009419	0.120824	0.249408	0.0977498	0.349734
Gglean009420	0	0	0.0287641	0
Gglean009421	0	0	0	0
Gglean009422	0	0	0	0
Gglean009423	17.49538	21.20879	7.94841397	12.62534
Gglean009424	17.49538	21.20879	7.94841397	12.62534
Gglean009425	17.49538	21.20879	7.94841397	12.62534
Gglean009426	0	0	0	0
Gglean009427	6.01835	7.22243	3.411049501	3.311196
Gglean009428	1.73491	0	0	0
Gglean009429	8.05E-06	0.209963	0.0823757	0
Gglean009430	1.14E-138	2.19E-189	0.100095	0.120289
Gglean009431	0	0	0	0.213274
Gglean009432	26.9305	10.547	20.9448	31.5764
Gglean009433	106.73577	103.41921	200.58049	216.5894
Gglean009434	192.4454	202.4751	66.0953306	54.07328
Gglean009435	43.83578192	48.2793961	32.30187998	28.67900813
Gglean009436	43.83578192	48.2793961	32.30187998	28.67900813
Gglean009437	38.09218	34.02129	29.01024	25.12165
Gglean009438	0.3600223	0.1926953	0.296200496	0.44592
Gglean009439	4.687148	4.973279	6.5924	5.998015
Gglean009440	4.687148	4.973279	6.5924	5.998015
Gglean009441	0	0	0	0
Gglean009442	0.342693	0.0708076	0.168239	0.066156
Gglean009443	3.654556682	4.313738364	2.817259296	3.164832046
Gglean009444	3.654556682	4.313738364	2.817259296	3.164832046
Gglean009445	3.654556682	4.313738364	2.817259296	3.164832046
Gglean009446	3.654556682	4.313738364	2.817259296	3.164832046
Gglean009447	48.04024861	52.81422728	32.21899265	30.4742689
Gglean009448	48.04024861	52.81422728	32.21899265	30.4742689
Gglean009449	7.200441	7.940681	7.246851	5.891703
Gglean009450	36.37745	34.22136	3.003158	0.308266
Gglean009451	0	0	0	0
Gglean009452	12.353833	13.91706833	19.511296	18.83808776
Gglean009453	12.353833	13.91706833	19.511296	18.83808776
Gglean009454	6.145409952	5.961653	4.175416003	3.389903
Gglean009455	33.037169	37.26185	53.65466	54.25261
Gglean009456	22.71536995	24.54728725	17.66793293	15.02787508
Gglean009457	1.30019	2.12973	1.35585	2.59985
Gglean009458	2.192965	1.78244339	1.960738	2.837087
Gglean009459	33.6992	38.5238	15.7558	12.9436

Gglean009460	32.3943	37.0063	16.437	12.9695
Gglean009461	20.4658846	21.79960214	14.39121008	13.5531032
Gglean009462	15.20936	12.5512	21.76701	26.85986
Gglean009463	30.2981326	32.71545306	29.13635575	27.80042475
Gglean009464	30.2981326	32.71545306	29.13635575	27.80042475
Gglean009465	145.90676	171.94089	54.169633	58.5051
Gglean009466	39.71662	47.37933	40.74085	39.34921
Gglean009467	39.71662	47.37933	40.74085	39.34921
Gglean009468	45.6353	43.1203	40.5159	35.6444
Gglean009469	0.775440681	2.52390428	1.345490211	1.029040818
Gglean009470	0	0	0	0
Gglean009471	0.76601008	1.032629836	1.392977538	1.992734966
Gglean009472	0.76601008	1.032629836	1.392977538	1.992734966
Gglean009473	30.661	29.3657	26.5519	27.3126
Gglean009474	0.637459	0.403403	0.575656	0.305419
Gglean009475	1.61207	1.46654	1.13373	1.57638
Gglean009476	6.967305	10.07493	4.4434966	3.434341
Gglean009477	25.73481	25.18659	19.32316	19.42704
Gglean009478	14.78044752	14.8235436	14.6002144	14.30254158
Gglean009479	14.78044752	14.8235436	14.6002144	14.30254158
Gglean009480	6.516684135	5.991134915	5.899079329	6.150938
Gglean009481	40.67971829	41.39987241	45.525784	48.7221801
Gglean009482	40.67971829	41.39987241	45.525784	48.7221801
Gglean009483	13.0472	24.8693	7.54759	9.34827
Gglean009484	97.1089027	96.48376464	92.39803653	73.82312048
Gglean009485	0.334792	1.41312	0.13571	0
Gglean009486	27.89942	128.0096	27.28651	143.14856
Gglean009487	27.89942	128.0096	27.28651	143.14856
Gglean009488	0	0	0	0
Gglean009489	0	0	0	0
Gglean009490	1.36277	1.57591	0.729954	0.581179
Gglean009491	0	0	0	0
Gglean009492	0	0	0	0
Gglean009493	0	0	0.0292945	0
Gglean009494	0	0	0	0
Gglean009495	0	0	0	0
Gglean009496	0	0	0	0
Gglean009497	0	0	0	0
Gglean009498	0	0	0	0
Gglean009499	0	0	0	0
Gglean009500	0	0	0	0
Gglean009501	3.593192636	6.208161	5.04765	3.9441846
Gglean009502	3.88314	4.41156	6.05426	6.5659
Gglean009503	3.029482051	2.357734	2.936880922	3.1468963

Gglean009504	0	0	0	0
Gglean009505	0	0	0	0
Gglean009506	28.93654253	32.31346209	21.78838147	22.58705345
Gglean009507	0.0966784	0.0993868	0.4117024	0.56211479
Gglean009508	1.681904165	0.6747448	1.273206421	2.3205655
Gglean009509	8.128426	10.310061	8.141138	12.271002
Gglean009510	0	0	0	0
Gglean009511	30.1681	29.47069	28.33101	26.44931
Gglean009512	59.778	50.5171	51.7104	86.4789
Gglean009513	1981.618	2074.265	1320.797	1268.155
Gglean009514	85.697	80.3962	49.6452	45.903
Gglean009515	0	2.81595	0	2.39383
Gglean009516	37.3497	31.9242	23.1297	23.9373
Gglean009517	18.0492	10.4428	0	0
Gglean009518	0	0.668716	0.493953	1.77232
Gglean009519	25.09148235	22.70633224	33.95641442	35.28366785
Gglean009520	25.09148235	22.70633224	33.95641442	35.28366785
Gglean009521	56.41006809	60.41122	52.28849532	55.50229032
Gglean009522	23.44195866	23.31934313	18.82411687	17.4940554
Gglean009523	1.8555	2.290287	1.388580236	1.035937875
Gglean009524	154.9121721	168.279018	88.717745	59.1350205
Gglean009525	1.41188	2.67112	4.10331	3.84352
Gglean009526	15.257387	16.51898726	7.39132	6.652056
Gglean009527	81.20553	70.25914	69.74088	41.14662
Gglean009528	52.0046	59.346	60.5713	69.7567
Gglean009529	2.018734	1.284595318	2.086941698	3.059996
Gglean009530	2.018734	1.284595318	2.086941698	3.059996
Gglean009531	2.018734	1.284595318	2.086941698	3.059996
Gglean009532	0	0	0	0
Gglean009533	104.0592252	137.971843	139.461383	121.799695
Gglean009534	104.0592252	137.971843	139.461383	121.799695
Gglean009535	104.0592252	137.971843	139.461383	121.799695
Gglean009536	13.18160874	12.41727648	4.46367055	3.474936024
Gglean009537	13.18160874	12.41727648	4.46367055	3.474936024
Gglean009538	28.0867486	29.334183	31.50161216	31.5917515
Gglean009539	0	0	0	0
Gglean009540	23.7188	30.1517	11.0987	9.16644
Gglean009541	0.980701	1.17597	1.34543	1.40553
Gglean009542	95.48197698	95.29992403	127.4783842	116.9667124
Gglean009543	38.81985	35.55000374	55.308856	59.867291
Gglean009544	0	0	0	0
Gglean009545	0	0	0.13478	0
Gglean009546	0	0.514896	0.20034	0
Gglean009547	0	0	0	0.303448

Gglean009548	0.638049	1.22539	1.64778507	2.05431
Gglean009549	0.932964	0.291866708	1.297402	0.822335
Gglean009550	0	0	0	0
Gglean009551	0.548962269	0.537441	0.455605203	0.725102493
Gglean009552	0	0	0	0
Gglean009553	15.38558136	15.3409	8.278317493	7.0312865
Gglean009554	15.38558136	15.3409	8.278317493	7.0312865
Gglean009555	17.96040587	21.34271527	25.02531121	29.23843351
Gglean009556	17.96040587	21.34271527	25.02531121	29.23843351
Gglean009557	0	0	0.0855159	0
Gglean009558	0	0	0	0.288137
Gglean009559	3.31717771	4.386289884	1.15318349	1.05628131
Gglean009560	0.0405089	0	0	0
Gglean009561	0	0	0.112009	0.0441988
Gglean009562	0	0	0	0
Gglean009563	5.277144	4.359022147	7.8844013	9.117355
Gglean009564	0	0.154609	0	0
Gglean009565	88.58143	87.20913414	87.17599	85.6916
Gglean009566	43.04142874	44.00228422	28.8475673	26.99096929
Gglean009567	43.04142874	44.00228422	28.8475673	26.99096929
Gglean009568	43.04142874	44.00228422	28.8475673	26.99096929
Gglean009569	0.291224	0.453324	0.550648	0.61432
Gglean009570	31.1438	30.581	26.5015	27.0131
Gglean009571	0.000292693	0.208023	0.101658	7.90E-07
Gglean009572	96.1395	90.0817	124.299	113.054
Gglean009573	19.28815	19.763163	11.34052	8.568704
Gglean009574	16.554	13.62771	14.9965	11.91854
Gglean009575	25.29329	27.85666	11.34703	10.42014
Gglean009576	17.21819569	15.124063	11.9591995	8.079522062
Gglean009577	42.93274512	36.6817313	33.231689	33.28404234
Gglean009578	0	0	0	0
Gglean009579	0	0	0	0
Gglean009580	86.25099252	81.427493	65.00392855	61.57831112
Gglean009581	1.255032	2.15605	1.135598	1.137755347
Gglean009582	0	0	0	0
Gglean009583	21.84873	25.44784	21.73234	29.44916
Gglean009584	21.84873	25.44784	21.73234	29.44916
Gglean009585	21.84873	25.44784	21.73234	29.44916
Gglean009586	117.8977	121.53417	132.6509	151.5538
Gglean009587	43.4965	42.2657037	40.34732966	39.50871353
Gglean009588	111.03	106.519	71.5689	60.5526
Gglean009589	5.388994687	6.365250982	7.540332518	9.96472996
Gglean009590	7.307179729	7.1029	8.569451021	9.91491
Gglean009591	20.75149	19.05217	40.3614	41.9185

Gglean009592	24.55639757	28.70023078	25.07850885	41.7406
Gglean009593	60.6478457	58.71066062	22.34918705	21.68662105
Gglean009594	8.1589558	7.442703	11.391747	13.68073
Gglean009595	7.06184	11.83017	14.28909	16.56421
Gglean009596	6.719359104	6.264454675	7.995844	8.762771373
Gglean009597	6.719359104	6.264454675	7.995844	8.762771373
Gglean009598	0	0	0	0
Gglean009599	24.1075	24.1035	6.26683	6.00141
Gglean009600	7.34791	8.17509	5.5064	4.42656
Gglean009601	0.0948297	0.0329544	0.103304	0
Gglean009602	0	0	0	0
Gglean009603	1.91661	2.13603	1.38625	1.27687
Gglean009604	0.134298	0.233408381	0.054666671	0
Gglean009605	1.04017	0.85504	0.84445	0.820508
Gglean009606	0	0	0	0
Gglean009607	18.262	16.0883	22.059	21.7784
Gglean009608	66.6862	59.4751	65.3999	63.6547
Gglean009609	24.358327	24.615506	19.59606	19.236259
Gglean009610	2.39188	2.38758	2.05273	1.5059
Gglean009611	1.78846	0.609488	0.823964	0.296568
Gglean009612	1.20616	0.495299	0.229337	0.866148
Gglean009613	6.859116	6.308476	22.67976	19.541096
Gglean009614	23.60932	16.87405686	24.50536	24.170461
Gglean009615	14.4529033	17.26430005	17.346988	16.70886376
Gglean009616	0.366751	0.222962	1.00462	0.993762
Gglean009617	45.60474	43.98206	39.20499	39.70426
Gglean009618	71.6463	65.5391	47.4182	45.8024
Gglean009619	11.60549338	13.4203447	9.91917	8.286457066
Gglean009620	46.88615	47.78853	18.20635	14.24414
Gglean009621	3.042938	3.464546	5.15262	5.76858
Gglean009622	94.89	114.982	92.0352	80.9458
Gglean009623	41.21560288	37.67695883	44.21359184	40.61443521
Gglean009624	162.6507	157.0401	91.0879	90.15322
Gglean009625	0.114422	0.0584938	0	0
Gglean009626	0	0.0249251	0	0
Gglean009627	0	0.0249251	0	0
Gglean009628	0.465106	0.163856	0.126168	0.149332
Gglean009629	0	0	0	0
Gglean009630	0	0	0	0
Gglean009631	6.124051	5.476341	6.223529	5.78623676
Gglean009632	6.124051	5.476341	6.223529	5.78623676
Gglean009633	6.061758	6.494075	4.348136	4.69719
Gglean009634	3.92303	2.92546	2.27985	1.95478
Gglean009635	36.94868	35.61436	22.95185	20.81135

Gglean009636	2.655836632	1.031875634	0.555369661	1.265861002
Gglean009637	2.655836632	1.031875634	0.555369661	1.265861002
Gglean009638	1.92144	1.95006	1.53248	1.22068
Gglean009639	8.13349	6.52312	14.3577	8.65954
Gglean009640	0	0	0	0
Gglean009641	0	0	0	0
Gglean009642	0	0	0	0
Gglean009643	0.230772	0.159343	0	0
Gglean009644	0	0.149626	0.0585325	0
Gglean009645	1.474232809	1.417033142	0.193896111	0.275172723
Gglean009646	10.10902	11.21034	15.47241	22.90978
Gglean009647	0	0	0	0
Gglean009648	0.0789232	0	0.0798073	0.0566668
Gglean009649	0	0	0	0
Gglean009650	14.887818	10.95920064	8.682361	7.776461216
Gglean009651	0.93565	0.824957	0.648622	0.128733
Gglean009652	0	0	0	0
Gglean009653	276.979	190.991	187.577	256.349
Gglean009654	18.80586	18.12042	14.38843	14.913886
Gglean009655	0	0	0	0
Gglean009656	0	0	0.0419382	0
Gglean009657	0	0	0	0
Gglean009658	0	0	0	0
Gglean009659	0.0800815	0.0833838	0	0
Gglean009660	60.90585	69.1912875	63.86061	63.32311
Gglean009661	5.273906	4.04272471	3.477714	3.867039032
Gglean009662	2.46372	2.37961	4.38897	6.51785
Gglean009663	86.73944	103.2613	75.787	66.3208
Gglean009664	0	0	0	0
Gglean009665	15.630062	16.31958934	12.8058203	9.8371603
Gglean009666	414.6072	426.7538	146.40086	120.29605
Gglean009667	32.79900115	37.0027003	21.90900761	17.23385106
Gglean009668	108.812283	112.9578035	100.1368417	92.16615039
Gglean009669	14.28256	15.68183	6.36829	5.23749
Gglean009670	43.062757	44.180064	52.290698	52.14515
Gglean009671	1.625	1.23624	1.56846	0.931236
Gglean009672	0	0	0	0
Gglean009673	0.660645	0.3254	0.23287	0.786556
Gglean009674	37.01826651	36.66820483	32.08917787	29.17807065
Gglean009675	101.237252	91.22231953	95.46619639	103.5956814
Gglean009676	101.237252	91.22231953	95.46619639	103.5956814
Gglean009677	0	4.22E-05	0	0
Gglean009678	10.6523	11.034	8.15829	7.23737
Gglean009679	0.0485137	0	0	0

Gglean009680	0.261678	0.915201	0.886642	1.25416
Gglean009681	0.3088827	0.2081229	0.75977702	0.908849271
Gglean009682	19.52313006	18.43898272	18.60049	30.86507
Gglean009683	71.02656	69.540927	14.68689	15.80871
Gglean009684	15.7877	16.5087	77.2875	113.618
Gglean009685	0	0	0	0
Gglean009686	11.246371	11.00784	7.546116	5.205497
Gglean009687	13.57668	13.77497	19.54401	17.57742
Gglean009688	181.2377869	151.0144581	238.1596013	397.3514353
Gglean009689	25.394704	26.20764381	25.87488299	28.364697
Gglean009690	34.2497	29.97	27.6325	48.7329
Gglean009691	30.40348075	23.741518	36.4953291	38.457913
Gglean009692	30.40348075	23.741518	36.4953291	38.457913
Gglean009693	41.97851446	40.57731043	50.40037	58.25984058
Gglean009694	74.0724	66.8342	54.6652	52.3667
Gglean009695	14.84068	13.71017	8.11317	8.071898
Gglean009696	0.14385992	0.522224115	0.233071722	0.259040867
Gglean009697	0.14385992	0.522224115	0.233071722	0.259040867
Gglean009698	44.04009	43.93833	27.45712	26.300188
Gglean009699	96.0191331	103.868837	70.548568	61.553339
Gglean009700	34.5943	30.5169	3.78995	3.45058
Gglean009701	8.453669	8.219771	7.740781	8.616504
Gglean009702	29.24086	23.76498	20.2733	21.10016
Gglean009703	16.09044	14.87279	7.47108	6.86425
Gglean009704	58.21156604	61.08857673	77.048401	70.028758
Gglean009705	44.98	42.8387	56.9491	52.5035
Gglean009706	18.62165177	19.72408475	14.57452621	13.95139693
Gglean009707	108.7931	106.0343	109.5548867	110.9105
Gglean009708	28.0234	31.1237	31.3069	27.37243
Gglean009709	24.55390027	26.496412	26.428949	35.252755
Gglean009710	0.422343	0	0	0
Gglean009711	0.422343	0	0	0
Gglean009712	25.46618363	23.78120633	24.9663351	27.16768748
Gglean009713	419.0257355	450.28463	180.520423	154.1189661
Gglean009714	419.0257355	450.28463	180.520423	154.1189661
Gglean009715	419.0257355	450.28463	180.520423	154.1189661
Gglean009716	419.0257355	450.28463	180.520423	154.1189661
Gglean009717	1.50516	0.019836	0.634803	4.03702
Gglean009718	9.31204	7.332724	7.93339	5.974994
Gglean009719	51.95523346	55.73187752	51.35893677	46.327316
Gglean009720	0	1.06451	0.813640116	6.479486
Gglean009721	6.479991804	5.150091317	5.114997656	8.293445486
Gglean009722	32.66720858	31.14517029	25.69326185	23.28659722
Gglean009723	9.88951	8.83701	9.69687	12.88445

Gglean009724	1.79197	2.143769	2.855995	2.93924
Gglean009725	162.652	221.265	273.053	304.62
Gglean009726	104.160229	109.8034588	80.37518945	76.32643172
Gglean009727	11.79965	11.50633	4.79971	3.990660643
Gglean009728	32.53907384	31.25741644	35.72131061	35.975538
Gglean009729	32.53907384	31.25741644	35.72131061	35.975538
Gglean009730	59.74385715	65.397571	35.26400947	30.86771952
Gglean009731	59.74385715	65.397571	35.26400947	30.86771952
Gglean009732	59.74385715	65.397571	35.26400947	30.86771952
Gglean009733	15.07564	18.12997	28.90586	27.66911
Gglean009734	19.07552468	23.88408176	10.2550271	10.43424254
Gglean009735	19.07552468	23.88408176	10.2550271	10.43424254
Gglean009736	19.07552468	23.88408176	10.2550271	10.43424254
Gglean009737	12.7700542	13.15223851	20.47837409	22.97094431
Gglean009738	12.7700542	13.15223851	20.47837409	22.97094431
Gglean009739	0	0	0	0
Gglean009740	65.21486	67.80000598	51.5548689	41.96613
Gglean009741	18.91	23.1857	20.4765	19.1626
Gglean009742	352.38751	322.38339	452.74562	599.57811
Gglean009743	17.76434255	24.21233326	16.16599793	18.29819634
Gglean009744	6.23737	6.54816	6.88879	9.55838
Gglean009745	2.247912004	2.259324987	2.380202668	2.463061
Gglean009746	0	0	0	0
Gglean009747	0	0	0	0
Gglean009748	0.354282113	0.128117343	0.30694059	0.567929782
Gglean009749	0.0594858	0.0638766	0.000548787	0.337753
Gglean009750	10.3532414	9.508656157	12.10361114	16.184251
Gglean009751	10.3532414	9.508656157	12.10361114	16.184251
Gglean009752	99.61421	102.47885	95.39675	92.53295
Gglean009753	12.1153	11.8686	8.16967	8.12508
Gglean009754	0	0	0	0
Gglean009755	23.1248924	27.6662597	43.9462459	43.719883
Gglean009756	23.1248924	27.6662597	43.9462459	43.719883
Gglean009757	0	0	0	0
Gglean009758	0	0	0	0
Gglean009759	17.6391	14.9151	13.4496	11.7398
Gglean009760	0	0	0.0928336	0
Gglean009761	0	0	0	0
Gglean009762	0	0	0	0
Gglean009763	0	0	0	0
Gglean009764	12.427293	13.352125	13.22990001	11.869422
Gglean009765	128.38877	129.166443	91.99054	91.43002
Gglean009766	161.6746	256.6501	337.234	359.1558
Gglean009767	6.30485	8.21644	10.883638	12.04031

Gglean009768	6.30485	8.21644	10.883638	12.04031
Gglean009769	0.115031	0.205762	0.0698154	0.166273
Gglean009770	0	0	0	0
Gglean009771	0	0	0	0
Gglean009772	0.0821867	0.0560704	0	0.0261418
Gglean009773	0	0	0	0
Gglean009774	0	0	0	0
Gglean009775	20.092148	18.60102298	15.586954	15.425436
Gglean009776	0	0.745464	0.552499	0
Gglean009777	0	0	0	0
Gglean009778	0.0378479	0	0.0620356	0.0369159
Gglean009779	0	0	0	0
Gglean009780	0.152777	0	0	0
Gglean009781	0	0	0	0
Gglean009782	0.0348801	0.017545	0.0558435	0.033082
Gglean009783	0	0	0	0.108667
Gglean009784	0	0	0	0
Gglean009785	3.99282	27.48892	2.29661	1.89656
Gglean009786	0	0	0	0
Gglean009787	0	0	0	0
Gglean009788	40.11721	63.43298	36.09489	46.38706
Gglean009789	0	0.164445	0	0
Gglean009790	3.13E-05	6.23378	5.61849	2.16721
Gglean009791	15.10975	13.88038	16.21822	18.92574
Gglean009792	10.86519	9.002408598	13.08564	13.48727
Gglean009793	43.122492	50.447256	30.187305	27.222758
Gglean009794	33.91152	36.9282	27.3581	25.04979
Gglean009795	1.39686	9.53289	2.6226	7.80084
Gglean009796	0	1.94321	0	0.200275
Gglean009797	1.637953	8.21612	4.792591	5.763465
Gglean009798	1.637953	8.21612	4.792591	5.763465
Gglean009799	0	1.066552313	0.0840228	0.379194
Gglean009800	0	0	0	0.18132
Gglean009801	0	0	0	0.0247261
Gglean009802	6.5192	8.08118	3.45506	1.96823
Gglean009803	3.0209	5.19238	5.50186	7.43375
Gglean009804	2.184091761	3.000900159	3.7393949	3.689663001
Gglean009805	21.9813	25.6225	27.9259	26.0804
Gglean009806	0	0	0	0
Gglean009807	11.80796819	7.592242244	13.5425513	13.83141165
Gglean009808	0	0	0	0
Gglean009809	0	0	0	0
Gglean009810	0	0	0	0
Gglean009811	0	0	0	0

Gglean009812	0	0	0	0
Gglean009813	47.10001534	43.906725	45.03853004	38.07363073
Gglean009814	5.94154	4.53251	6.485168	8.42472
Gglean009815	99.18849	91.756149	129.51581	135.29724
Gglean009816	17.2658	14.4187	11.1265	13.2771
Gglean009817	0	0	0	0
Gglean009818	13.94571409	12.63570403	21.75330529	22.18980334
Gglean009819	53.2484	37.4031	10.6208	19.7321
Gglean009820	26.68454	26.16555	14.0823	15.50525
Gglean009821	7.02891	7.26571	11.41324	12.6327
Gglean009822	28.04879335	27.318493	36.09880006	36.24165045
Gglean009823	28.04879335	27.318493	36.09880006	36.24165045
Gglean009824	0	0	0	0
Gglean009825	6.45014	6.3818	3.2171	3.81287
Gglean009826	0.351472	0	0.0478053	0
Gglean009827	0	0	0	0
Gglean009828	0	0	0	0
Gglean009829	21.32472614	20.47094814	16.78600335	14.38222159
Gglean009830	0.144259	0.0551149	0.1028	0.0520751
Gglean009831	0	0	0	0
Gglean009832	0	0	0	0
Gglean009833	0	0	0	0.0407744
Gglean009834	0	0	0	0
Gglean009835	0	0	0	0
Gglean009836	0.0187577	0	0	0
Gglean009837	0	0	0	0
Gglean009838	0	0	0.037239	0.0443479
Gglean009839	0	0	0	0
Gglean009840	0.0174105	2.78362	1.57617	1.95003
Gglean009841	27.99196554	18.053152	21.14346482	31.36219043
Gglean009842	27.99196554	18.053152	21.14346482	31.36219043
Gglean009843	27.99196554	18.053152	21.14346482	31.36219043
Gglean009844	1.953251	2.407256075	3.19858	3.416429
Gglean009845	25.65119145	29.21290627	17.80381249	19.64564
Gglean009846	10.6375	13.7122	0.451276	0.247487
Gglean009847	53.75083283	42.41905001	1.524654618	0.953372
Gglean009848	36.96119	42.670552	42.11435	43.26127
Gglean009849	62.03389	68.81921	32.0614	28.31621
Gglean009850	14.10316	15.17122	19.56243	25.92197
Gglean009851	25.60206323	27.52609448	34.75080841	33.91607264
Gglean009852	0	0	0	0
Gglean009853	6.35E-06	9.66E-06	2.68E-05	6.16E-05
Gglean009854	0	0	0.465364	0
Gglean009855	2.90407	4.69969	4.83134	4.59636

Gglean009856	0	0	0	0
Gglean009857	0	0	0	0
Gglean009858	0	0	0	0
Gglean009859	0	0	0	0
Gglean009860	0	0.365498	0.475758	0.340544
Gglean009861	42.7592387	38.5335403	70.8801596	66.18076373
Gglean009862	42.7592387	38.5335403	70.8801596	66.18076373
Gglean009863	0	0.0628324	0.586848	0
Gglean009864	0	0.0628324	0.586848	0
Gglean009865	10.99326	11.03462	16.6046	20.23973
Gglean009866	0	3.67221	0	0
Gglean009867	78.27561	80.48916	51.173211	56.22732
Gglean009868	1.270814064	1.621319473	1.2818133	1.405065008
Gglean009869	1.270814064	1.621319473	1.2818133	1.405065008
Gglean009870	0.0762961	0.11903	0	0.110474
Gglean009871	118.143	134.156	135.262	222.185
Gglean009872	11.46808	15.9450742	20.8830991	18.812524
Gglean009873	11.46808	15.9450742	20.8830991	18.812524
Gglean009874	16.22118	23.506858	15.15298	6.968610007
Gglean009875	16.22118	23.506858	15.15298	6.968610007
Gglean009876	0.115056934	0.2479485	0.330536	0.163068201
Gglean009877	12.3949	13.617	8.50301	7.77951
Gglean009878	4.84E-12	3.75E-12	1.37E-08	0
Gglean009879	0.37054	0.0970672	0	0.0897757
Gglean009880	0	0	0	0
Gglean009881	12.96146	9.87626	7.72649	6.94749
Gglean009882	31.58267	37.76443	33.17044	33.79839
Gglean009883	41.10196	45.580074	31.3870205	25.05779789
Gglean009884	0.498563	0.810307	0.606419	0.997602
Gglean009885	8.422500001	7.029682751	10.28254139	15.11525044
Gglean009886	8.422500001	7.029682751	10.28254139	15.11525044
Gglean009887	17.1313	21.9966	12.1367	11.6554
Gglean009888	10.47945	1.68102	0.935907161	6.384859
Gglean009889	10.47945	1.68102	0.935907161	6.384859
Gglean009890	17.78123	18.39476	17.346573	15.06133
Gglean009891	1.26458	1.00915	0.913034	0.354189
Gglean009892	42.15687	43.18081	19.91129	31.58087
Gglean009893	0	0	0	0
Gglean009894	0	0	0	0
Gglean009895	2.8975	8.421	11.6963	13.2724
Gglean009896	5.684887775	6.636667004	8.327179231	9.687433277
Gglean009897	0	0	4.47479	0
Gglean009898	0	0	0	0
Gglean009899	0.0318195	0	0.0257971	0

Gglean009900	10.3913	10.5225	10.0723	10.0481
Gglean009901	23.81194016	30.83671061	42.19335251	40.142173
Gglean009902	23.81194016	30.83671061	42.19335251	40.142173
Gglean009903	23.81194016	30.83671061	42.19335251	40.142173
Gglean009904	1.51994	2.65378	3.93252	5.6448
Gglean009905	0.531549	0.223978	0.655269	0.524967
Gglean009906	31.14686386	29.53294781	27.83130542	15.47484767
Gglean009907	31.14686386	29.53294781	27.83130542	15.47484767
Gglean009908	59.38387	49.76584	36.75975	35.94637063
Gglean009909	0	3.01E-17	0	8.71E-09
Gglean009910	0	0	0	0
Gglean009911	1.7888	6.44038	2.00004	2.63658
Gglean009912	121.8806535	110.9881733	99.3062617	85.67346997
Gglean009913	102.21812	114.738288	71.92475085	64.17943
Gglean009914	8.91392	7.52026	9.57282	12.168
Gglean009915	0	0.0732202	0	0
Gglean009916	2.64806	0	0	0
Gglean009917	0.0412878	0	0	0
Gglean009918	0	0.0605646	0.0474921	0
Gglean009919	5.787299	5.569936	4.072357	4.152643
Gglean009920	31.467194	30.99772025	29.3173227	26.03572073
Gglean009921	3.227383432	3.646570313	4.922564001	5.454736924
Gglean009922	0	0	0	0
Gglean009923	0	0	0	0
Gglean009924	0	0	0	0
Gglean009925	0	0	0	0
Gglean009926	0	0	0	0
Gglean009927	24.33726924	19.09047771	40.8306003	57.68423543
Gglean009928	24.33726924	19.09047771	40.8306003	57.68423543
Gglean009929	108.01712	106.22136	116.9265	101.96957
Gglean009930	87.45107194	80.40709	87.43156314	113.0256409
Gglean009931	21.386384	23.717435	13.019744	11.94752
Gglean009932	19.0887	28.3686	9.13447	7.40863
Gglean009933	34.69538	29.03748	28.56797	26.19298
Gglean009934	0	0	0	0
Gglean009935	1.128247	1.399272	1.536684	2.544399
Gglean009936	52.01912609	52.62140139	60.08199926	55.93779193
Gglean009937	0	0	0	0
Gglean009938	38.55650776	41.09875399	28.0144737	27.34896325
Gglean009939	38.55650776	41.09875399	28.0144737	27.34896325
Gglean009940	0.868218433	0.483556356	0.796674219	1.242458942
Gglean009941	1.17E-147	0.0973223	0.1948529	0.120724
Gglean009942	0	0	0	0
Gglean009943	0	0	0	0

Gglean009944	3.10E-20	4.07E-17	1.20E-20	1.10E-21
Gglean009945	5.07585	7.98918	9.8023	8.87898
Gglean009946	3.106258	4.800146	6.588572	8.183596
Gglean009947	21.229964	22.960333	20.42911	20.054319
Gglean009948	12.81919297	14.013607	17.84708101	21.88328765
Gglean009949	34.97065849	34.195	43.94123	52.0977
Gglean009950	55.68324	63.7576	45.0358	40.85815
Gglean009951	41.3276	40.2586	36.1279	32.3457
Gglean009952	37.345154	41.05077477	38.60561974	37.19550104
Gglean009953	37.345154	41.05077477	38.60561974	37.19550104
Gglean009954	56.03443	56.7233473	38.69145	36.65518
Gglean009955	56.03443	56.7233473	38.69145	36.65518
Gglean009956	16.66789	16.13232	22.18857	31.4762
Gglean009957	8.52596	13.062	18.5769	10.9603
Gglean009958	30.55493	27.69011	28.49227	40.29017
Gglean009959	30.55493	27.69011	28.49227	40.29017
Gglean009960	64.14270472	56.06057898	76.94184675	84.79585078
Gglean009961	64.14270472	56.06057898	76.94184675	84.79585078
Gglean009962	64.14270472	56.06057898	76.94184675	84.79585078
Gglean009963	0	0	0	0
Gglean009964	0	0	0	0
Gglean009965	44.91065	49.89605	29.98579	29.13168
Gglean009966	0	0	0	0
Gglean009967	0	0	0	0
Gglean009968	5.08283	5.67786	0.000288068	3.17836
Gglean009969	23.11273	19.942601	19.203951	19.70210623
Gglean009970	2.76901	4.45531	5.35567	5.86712
Gglean009971	0.541247	0	0	0.54238
Gglean009972	166.6161957	155.8321652	113.4067971	103.2639517
Gglean009973	166.6161957	155.8321652	113.4067971	103.2639517
Gglean009974	0.129595	0	0.0348334	0
Gglean009975	0	0	0	0
Gglean009976	0	0	0	0
Gglean009977	0	0	0	0
Gglean009978	0	0	0	0
Gglean009979	1.67E-16	3.56E-63	0.084908463	1.86E-14
Gglean009980	16.26448	18.98105	5.37315	4.915
Gglean009981	9.52571521	10.68294863	9.69011496	8.944934922
Gglean009982	29.463347	36.11251951	44.032436	38.59702004
Gglean009983	10.27403925	11.9134737	12.23916397	13.50272339
Gglean009984	0	0	0.0436691	0
Gglean009985	0	0	0.0436691	0
Gglean009986	0	0	0	0
Gglean009987	8.44E-06	0	0	0

Gglean009988	2.35E-16	0.584434	1.08E-12	5.12E-05
Gglean009989	14.17140002	13.15480014	8.031529	8.061590038
Gglean009990	0.53557925	0.542183159	0.571619038	0.8590847
Gglean009991	0	0	0	0
Gglean009992	52.73726	60.23532	35.16637302	36.076908
Gglean009993	8.572296	6.33155	7.327341	7.24199
Gglean009994	32.04517	29.946279	40.019662	35.510577
Gglean009995	9.971788	12.89202	9.921397	10.73535
Gglean009996	0.446065164	0.15792016	0.251834259	0.242713718
Gglean009997	14.88436478	15.99427499	6.360862	4.924262
Gglean009998	81.4021	77.9889	80.9016	80.0597
Gglean009999	1.143659	1.986894	1.552690262	1.73854
Gglean010000	1.143659	1.986894	1.552690262	1.73854
Gglean010001	0	0	0	0.0977475
Gglean010002	1.34357	4.31E-05	0.312905	0.00653135
Gglean010003	0	0	0	0
Gglean010004	0	0	0	0
Gglean010005	36.9497707	39.22586955	48.82142335	51.0283788
Gglean010006	41.28931	42.27786	11.291727	8.219921
Gglean010007	15.883765	18.5173	18.5957	21.76128
Gglean010008	15.883765	18.5173	18.5957	21.76128
Gglean010009	15.883765	18.5173	18.5957	21.76128
Gglean010010	16.2622	18.517	15.0099	15.9319
Gglean010011	26.812156	28.8844787	28.72852071	29.99946947
Gglean010012	32.49103376	32.04754921	44.16982223	54.10812493
Gglean010013	0.277233	0	0.453972	0.816895
Gglean010014	74.898908	89.405687	116.82627	126.29161
Gglean010015	74.898908	89.405687	116.82627	126.29161
Gglean010016	1.81652	2.83022	2.27173	3.40837
Gglean010017	0	0	0	0
Gglean010018	15.51251061	15.96075198	23.15711079	25.84981155
Gglean010019	0	0	0	0
Gglean010020	0	0	0.284398	0
Gglean010021	0.132884	0.295233	0.170045	0.247269
Gglean010022	150.9702	160.3855	107.0959	80.9295
Gglean010023	150.9702	160.3855	107.0959	80.9295
Gglean010024	0.0409162	0.0421115	0.0663377	0.0392795
Gglean010025	26.03662268	25.885055	17.15038082	15.383638
Gglean010026	0.307772	0.802073	0.251986	0
Gglean010027	44.25059	44.99780104	10.69870075	4.859060764
Gglean010028	5.58593	4.76872	2.73185	2.74931
Gglean010029	5.33897	5.90226	1.884723	1.21135451
Gglean010030	101.85397	110.3278873	54.364328	54.27279
Gglean010031	0.122919	0.13304	0.202507	0

Gglean010032	0	0	0	0
Gglean010033	0	0.215934	0	0
Gglean010034	20.36222324	20.63783603	21.653276	24.995683
Gglean010035	111.017	108.035	89.0334	79.7917
Gglean010036	67.02988	71.82516	75.92362	77.13068
Gglean010037	41.9417	40.03434	30.56068546	27.484243
Gglean010038	59.86862036	66.16014637	57.93869	57.5912828
Gglean010039	42.48326	42.05809651	100.7377598	102.4760188
Gglean010040	42.48326	42.05809651	100.7377598	102.4760188
Gglean010041	0.274000193	0.108487589	0.397809	0.4098783
Gglean010042	108.8038832	95.36753004	105.3369834	106.19673
Gglean010043	108.8038832	95.36753004	105.3369834	106.19673
Gglean010044	33.52827	34.30097	54.37703	56.75012
Gglean010045	0.1934957	0.466065018	0.35837	0.976269
Gglean010046	66.98422909	79.688771	97.230944	104.133727
Gglean010047	0.397963	1.34141	0.130306	0.23375
Gglean010048	515.5039	542.7678	775.9445	977.4388
Gglean010049	2.1725818	2.731495	3.1319	2.247111
Gglean010050	2.1725818	2.731495	3.1319	2.247111
Gglean010051	29.947	29.43984	26.44374	25.74955
Gglean010052	49.98292871	47.93497	48.63877002	51.26342323
Gglean010053	15.7919	15.8947	20.278483	21.626427
Gglean010054	25.12387059	26.22061015	15.37344587	12.82900784
Gglean010055	25.12387059	26.22061015	15.37344587	12.82900784
Gglean010056	25.12387059	26.22061015	15.37344587	12.82900784
Gglean010057	81.0344	93.0111	29.28069	23.42215
Gglean010058	38.27363958	37.30332949	40.05592333	46.25021618
Gglean010059	18.711981	19.040814	27.2399538	33.859496
Gglean010060	12.68042	11.90531	18.09733	22.55003
Gglean010061	0.469724358	0.321691175	0.506467338	0.719778638
Gglean010062	0.469724358	0.321691175	0.506467338	0.719778638
Gglean010063	0	0	0	0
Gglean010064	0	0	0	0
Gglean010065	0.601931384	1.289743775	0.803446999	0.974121686
Gglean010066	0.601931384	1.289743775	0.803446999	0.974121686
Gglean010067	24.9269	17.4616	18.7506	25.2296
Gglean010068	34.6223302	34.237712	50.50167344	59.57696207
Gglean010069	0	0	0	0
Gglean010070	15.06465	13.20269	9.78527	9.07917002
Gglean010071	12.32740879	10.29501443	6.295942516	5.889951656
Gglean010072	8.041021	7.44495	4.428054	3.980311463
Gglean010073	8.041021	7.44495	4.428054	3.980311463
Gglean010074	0	0	0	0
Gglean010075	0.0728861	0	0	0

Gglean010076	37.3066594	37.49100816	44.53516177	45.645337
Gglean010077	37.41598592	40.31227569	27.95158165	25.96653042
Gglean010078	2.410607137	2.370918845	2.4456215	3.076454693
Gglean010079	15.13011206	18.12880295	8.94738734	9.019804465
Gglean010080	15.13011206	18.12880295	8.94738734	9.019804465
Gglean010081	10.24996	9.43633	11.76543	11.43979
Gglean010082	93.17328	85.09416	80.9431	73.37112
Gglean010083	4.14349	4.18498	5.25392	5.81573
Gglean010084	0	0	0	0
Gglean010085	0	0	0	0
Gglean010086	0.141958681	0.269202	0.1140433	0.096315501
Gglean010087	0.198562	0	0.325997	0
Gglean010088	0	0	0	0
Gglean010089	12.9824491	12.11113003	8.20905619	6.1649169
Gglean010090	1.139810065	1.068260041	0.47873202	0.428540176
Gglean010091	0	0.0742308	0.0294161	0.0348413
Gglean010092	0	0	0	0
Gglean010093	0	0	0	0
Gglean010094	0	0	0	0
Gglean010095	0	0	0	0
Gglean010096	19.15925288	20.7171797	30.27384355	31.80877125
Gglean010097	19.15925288	20.7171797	30.27384355	31.80877125
Gglean010098	19.15925288	20.7171797	30.27384355	31.80877125
Gglean010099	2.583251	2.726534	1.775374	1.263210411
Gglean010100	31.77107206	34.69450837	19.10300445	18.25296009
Gglean010101	0.4804737	0.303153263	0.13359182	0.116941108
Gglean010102	0.278992	0.370449	0.309603	0.641846
Gglean010103	0.249812	0	0.050339	0.059767
Gglean010104	45.204315	52.08891	57.14095	48.29681
Gglean010105	1410.46	1411.097	890.541	840.972
Gglean010106	113.83995	123.9168632	133.3393434	121.6341653
Gglean010107	53.22158	62.73178	43.22002857	39.58369231
Gglean010108	159.432784	165.98798	68.02987042	62.510853
Gglean010109	0.106708	0.222808	0.345629	0.102146
Gglean010110	231.9672	214.9546	280.1695	439.982
Gglean010111	12.08049	11.9855	5.294576	6.06447
Gglean010112	26.03231823	31.871573	23.76686204	27.68157429
Gglean010113	0.127213	0.200795	0.051784	0.122778
Gglean010114	0	0	0.309191	0.550173
Gglean010115	18.110829	20.06409	11.36805	12.07334
Gglean010116	23.35004	23.93545	18.72187	17.12435
Gglean010117	14.47985253	15.21870266	15.28237481	16.14458952
Gglean010118	0.700135097	0.786738531	1.096528721	1.3481381
Gglean010119	7.88E-06	0.757978	0.21808	0.937648

Gglean010120	0.191114	0.403104	0	0.184802
Gglean010121	23.7524501	24.42268209	18.83125904	17.57575802
Gglean010122	51.7782634	51.58800258	49.30923099	41.30154728
Gglean010123	2.448422058	3.822162	2.001516234	4.09810325
Gglean010124	1.822134573	1.862938371	2.538374	3.658179271
Gglean010125	2.21741	1.228409026	1.5581758	2.699469
Gglean010126	0.00671269	0.0925979	0.0108375	0.00774232
Gglean010127	0.00671269	0.0925979	0.0108375	0.00774232
Gglean010128	0	2.10E-183	0.0304965	0.058263
Gglean010129	19.14991	19.42856	12.42959	10.42878
Gglean010130	17.011929	15.88593587	10.239792	13.052331
Gglean010131	17.011929	15.88593587	10.239792	13.052331
Gglean010132	0.0465768	0.28904	0.113929	0.315825
Gglean010133	0.396904284	1.005368668	0.914798914	0.80647987
Gglean010134	1.06195	2.3538	2.64803	3.16782
Gglean010135	1.06195	2.3538	2.64803	3.16782
Gglean010136	0	0	0	0
Gglean010137	16.747	20.725	3.40058	2.488
Gglean010138	0	0	4.71783	0
Gglean010139	6.78982	4.976603195	8.304653308	11.288854
Gglean010140	7.568102	8.647962056	7.59129	6.21911
Gglean010141	7.568102	8.647962056	7.59129	6.21911
Gglean010142	6.495854468	8.281834	5.011202	6.034957766
Gglean010143	23.40394	27.1831	27.427	28.1794
Gglean010144	1.29674	2.02198	1.65515	1.49066
Gglean010145	0.230081	0.299905	0.235629	0.2236
Gglean010146	0	0	0	0
Gglean010147	21.94597686	21.09871442	41.38209341	48.997658
Gglean010148	32.00535	35.55996	30.02378	28.46291438
Gglean010149	46.514253	50.90094	25.72489	19.30837
Gglean010150	0.667037	0.391436	0.233421	0.0927402
Gglean010151	46.514253	50.90094	25.72489	19.30837
Gglean010152	46.514253	50.90094	25.72489	19.30837
Gglean010153	3.826279	3.57034	2.50513615	4.52602
Gglean010154	0	0.876121	0	0
Gglean010155	0.0434262	0.177128	0.0350992	0
Gglean010156	6.34137	5.08119	4.64107	8.10635
Gglean010157	0	0.223167	0	0.104714
Gglean010158	0	0	0	0
Gglean010159	0	0	0	0
Gglean010160	19.2065	20.9915	20.0673	17.7529
Gglean010161	75.061334	59.85324	45.90934	44.03990001
Gglean010162	0.47443	1.08853	2.89259	1.96682
Gglean010163	0.0597199	0	0	0

Gglean010164	12.74185	15.52342221	8.622245	7.01098
Gglean010165	0	0	0	0
Gglean010166	0	0	0.03492037	0
Gglean010167	0	0	0.03492037	0
Gglean010168	0	0	0	0
Gglean010169	0	0	3.12103	3.70404
Gglean010170	0.625731502	0.286632215	1.269388	0.97199611
Gglean010171	7.17665	5.46116	0	0
Gglean010172	7.31982	9.67004	11.31867	11.59074
Gglean010173	1.614963	1.988523	2.48867146	2.154904
Gglean010174	5.647949827	5.99326	8.366933	9.74664801
Gglean010175	5.647949827	5.99326	8.366933	9.74664801
Gglean010176	19.66287	18.51650068	15.841185	19.892888
Gglean010177	21.39787	20.16007018	16.37943445	17.89642
Gglean010178	29.26339515	30.726704	15.134474	14.29643823
Gglean010179	3.138267631	3.342470072	3.314428098	3.800680027
Gglean010180	0.120076	0.184311	0.0484046	0.172478
Gglean010181	2.39E-05	0.336998	2.41E-05	9.11E-06
Gglean010182	2.320513697	2.111160561	3.270807	3.588707737
Gglean010183	14.7443564	18.39741782	16.07946295	16.29380532
Gglean010184	24.6117	29.677	11.90556	9.50246
Gglean010185	1.803504	0.358965724	2.878288	0.506786472
Gglean010186	22.6095721	22.558216	13.6161123	11.543809
Gglean010187	41.07198326	42.16697592	50.73980384	48.44605593
Gglean010188	15.08997	12.83912	2.794648	2.705519
Gglean010189	38.57073744	40.69639266	31.79149214	29.91228981
Gglean010190	23.02598694	23.80974863	30.14883344	27.4560493
Gglean010191	23.02598694	23.80974863	30.14883344	27.4560493
Gglean010192	23.02598694	23.80974863	30.14883344	27.4560493
Gglean010193	0	0.0640216	0.0500668	0.41335706
Gglean010194	187.845189	175.0326573	281.5588729	374.365
Gglean010195	46.860274	47.30800664	47.06516964	47.78525
Gglean010196	13.03121	11.200408	12.46104	11.96515
Gglean010197	172.6411728	198.7292118	96.91535003	85.63342194
Gglean010198	172.6411728	198.7292118	96.91535003	85.63342194
Gglean010199	0	0	0.71461	0.213216
Gglean010200	0	0	0.210707	0.250801
Gglean010201	0.146929	0.22785	0.208194	0.316589
Gglean010202	0.468542	0.585781	0.0759753	0.0899665
Gglean010203	0	0.0624933	0.146339	0
Gglean010204	0	0	0	0
Gglean010205	0	0	0	0
Gglean010206	3.53061643	3.262188273	5.895977724	5.974177528
Gglean010207	3.53061643	3.262188273	5.895977724	5.974177528

Gglean010208	22.10453317	21.95894566	22.348248	23.23821409
Gglean010209	0	0	0	0
Gglean010210	57.8940206	56.06254	81.88001	80.20608
Gglean010211	57.8940206	56.06254	81.88001	80.20608
Gglean010212	80.6091	111.7552	40.0613	37.1611
Gglean010213	5.49E-28	2.57E-19	5.48E-19	9.24E-08
Gglean010214	9.85976	9.60907	4.517318	3.83992
Gglean010215	9.85976	9.60907	4.517318	3.83992
Gglean010216	1.03188	0	0	0
Gglean010217	198.11214	205.80848	106.99155	111.54886
Gglean010218	0	0	0.236345	0.186418
Gglean010219	0	0	0	1.48523
Gglean010220	38.3305	37.2208	22.1234	16.8373
Gglean010221	28.53821457	28.4829237	28.6859707	27.76884893
Gglean010222	0.165882235	0.222667174	0.153775424	0.161950449
Gglean010223	21.507914	20.59936	25.208246	28.690679
Gglean010224	0	0.416892	0.317934	0
Gglean010225	25.148587	27.946772	37.623766	42.89181
Gglean010226	0.468203	0.249575	0	0
Gglean010227	0	0.255479	0.202714	0.60107
Gglean010228	0	5.54123	0	0
Gglean010229	0.0826626	0	0.0667049	0
Gglean010230	0	0	0	0
Gglean010231	20.627178	21.448786	23.609916	23.34689
Gglean010232	0	0	0	0
Gglean010233	19.83886	20.65352	23.5493	23.14176
Gglean010234	0	0	0	0
Gglean010235	1.487527	1.040138867	0.68385607	0.476035
Gglean010236	43.9054236	43.613719	54.26609	50.191154
Gglean010237	43.9054236	43.613719	54.26609	50.191154
Gglean010238	0	0	0	0
Gglean010239	48.0677	36.7429	73.8309	95.7357
Gglean010240	34.77105	30.73955	19.36984	16.56758
Gglean010241	0	0	0	0
Gglean010242	0	0	0	0
Gglean010243	0	0	0	0
Gglean010244	0	0	0	0
Gglean010245	0.460892427	1.02241	0.359401982	0.516897275
Gglean010246	0	0	0	0
Gglean010247	0	0	0	0
Gglean010248	0	0	0	0
Gglean010249	25.9507	29.6816	36.0538	23.1773
Gglean010250	0.106442	0	0	0
Gglean010251	0	0	0	0

Gglean010252	0	0	0	0
Gglean010253	0	0	0	0
Gglean010254	0	0	0	0
Gglean010255	0	0	0	0
Gglean010256	0	0	0	0
Gglean010257	0	0	0	0
Gglean010258	0.189027	0	0	0
Gglean010259	0	0	0	0
Gglean010260	0	0	0	0
Gglean010261	0.0556495	0.352597	0.13665	0.268447
Gglean010262	0	0	0	0
Gglean010263	0	0	0	0
Gglean010264	0	0	0	0
Gglean010265	0.210462948	0.582318783	0.6264711	0.39228086
Gglean010266	0.210462948	0.582318783	0.6264711	0.39228086
Gglean010267	0	0	0	0
Gglean010268	2.29797002	2.213400154	1.748830297	1.247410099
Gglean010269	0.059018	0.0612131	0.288028	0.340957
Gglean010270	0	0.094405705	0.0552328	0.044539149
Gglean010271	0	0	0	0
Gglean010272	234.286904	248.350057	292.2148751	324.636933
Gglean010273	234.286904	248.350057	292.2148751	324.636933
Gglean010274	0.8599663	1.297116	0.343773351	0.23300545
Gglean010275	20.39488493	20.42705589	15.5260719	16.483227
Gglean010276	0.127536	0.0327186	0.0514236	0.0306814
Gglean010277	22.62391144	23.14799024	16.80304222	15.0549939
Gglean010278	22.62391144	23.14799024	16.80304222	15.0549939
Gglean010279	74.8543	75.1627	70.1836	69.1849
Gglean010280	14.5503	18.481	19.2468	24.7719
Gglean010281	121.8296097	124.437287	117.20422	116.6954851
Gglean010282	0	0	0	0
Gglean010283	0.617527086	1.237658067	1.935762961	2.174172404
Gglean010284	3.63034	2.52934	2.96915	0.1971
Gglean010285	12.04754348	11.16013754	8.53870445	10.49597808
Gglean010286	0.391716446	0.283328828	0.158299616	0.213795944
Gglean010287	17.64359	27.09427	26.49395	33.33739
Gglean010288	0.115144619	0.059377447	0.0449074	0.212316176
Gglean010289	0.97180907	0.926781	1.0919583	1.089863825
Gglean010290	0	0	0	0
Gglean010291	0	0.2838235	0.278586	0.800723
Gglean010292	54.59928243	26.37136675	30.16075817	10.08665539
Gglean010293	54.59928243	26.37136675	30.16075817	10.08665539
Gglean010294	54.59928243	26.37136675	30.16075817	10.08665539
Gglean010295	10.9514077	8.392570222	6.217356	7.5540194

Gglean010296	10.9514077	8.392570222	6.217356	7.5540194
Gglean010297	17.12462	13.59292297	9.994942315	9.931433
Gglean010298	17.12462	13.59292297	9.994942315	9.931433
Gglean010299	0	0	0	2.87363
Gglean010300	30.45480263	24.19727933	19.45372554	18.05898391
Gglean010301	5.44721	4.64443	3.02701	3.08608
Gglean010302	0.248187115	0.382285484	0.327685707	0.747087566
Gglean010303	0.800925211	0.647534796	0.53547417	1.11744
Gglean010304	0	0.210533	0	0
Gglean010305	3.40242	5.10111	2.00814	2.01893
Gglean010306	3.3741827	4.55527	3.844443	4.77765962
Gglean010307	0	0.0394574	0.0620173	0.257485
Gglean010308	0.000170714	0.000554971	0.442452	0.523895
Gglean010309	0.630503	0	0.205482	0.122526
Gglean010310	2.926947473	5.45893673	7.947387089	9.973855742
Gglean010311	0	0	0	0
Gglean010312	0	0	0	0
Gglean010313	0	0	0	0.4977
Gglean010314	22.66331	24.87866	24.30532	11.64855
Gglean010315	28.61214	25.8389	16.82945	14.36507
Gglean010316	28.61214	25.8389	16.82945	14.36507
Gglean010317	27.87458	28.55925	20.32827	17.70923
Gglean010318	0	0	0	0
Gglean010319	0	0	0	0
Gglean010320	0	0	0	0
Gglean010321	0	0	0	0
Gglean010322	0	0	0	0
Gglean010323	0	0	0	0
Gglean010324	0	0	0	0
Gglean010325	0	0	0	0
Gglean010326	0	0	0	0
Gglean010327	0	0	0	0
Gglean010328	0	0	0	0
Gglean010329	0	3.57601	5.06437	0
Gglean010330	6.20403	0	4.97042	0
Gglean010331	0.987196	0	0	0
Gglean010332	0.698267	1.08951	0.170211	0.693955
Gglean010333	0	0	0	0
Gglean010334	30.9503	40.1213	36.3992	48.4845
Gglean010335	0	0	0.202885	0
Gglean010336	0	0	0	0
Gglean010337	0	0	0.20553	0
Gglean010338	0.237491254	0.548877377	0.072285794	0.0786914
Gglean010339	0	0	0	0

Gglean010340	0	0	0	0
Gglean010341	0	0	0.049032	0
Gglean010342	0	0	0.0939439	0.0560291
Gglean010343	0	0	0.0950121	0.226012
Gglean010344	0	0	0	0
Gglean010345	0	0	0	0
Gglean010346	0	0	0	0
Gglean010347	0	0	0	0
Gglean010348	0	0	0	0
Gglean010349	0.593176199	0.286772046	0.2022345	0.133608824
Gglean010350	0.102266	0	0	0
Gglean010351	0	0	0	0
Gglean010352	0	0	0	0
Gglean010353	0	0	0.387105	0
Gglean010354	0	0	0	0
Gglean010355	0.100263	0	0	0
Gglean010356	0	0.174584	0	0
Gglean010357	0	0	0	0
Gglean010358	1.07091	1.50811	1.268	1.21903
Gglean010359	0	0	0	0
Gglean010360	0	0	0	0
Gglean010361	0	0	0	0
Gglean010362	0	0	0	0
Gglean010363	0.130664	0.136096	0	0
Gglean010364	0	0	0.0958968	0
Gglean010365	0	0	0.197079	0
Gglean010366	0	0	0	0
Gglean010367	0	0	0	0
Gglean010368	0	0	0	0.458248
Gglean010369	0.207551	0.390126	0.235825	0.158928
Gglean010370	0.105322	0.0538001	0.0860325	0.0512527
Gglean010371	0	0	0	0
Gglean010372	0	0	0	0
Gglean010373	0	0.395335	0.101126	0
Gglean010374	0	0	0	0.249554
Gglean010375	0	0.0843449	0	0
Gglean010376	0	0	0.187225	0
Gglean010377	0	0	0	0
Gglean010378	0	0	0	0
Gglean010379	0.045113222	0	0.0726262	0.0431215
Gglean010380	0	0	0	6.32975
Gglean010381	0	0	0	0
Gglean010382	0	0	0	0
Gglean010383	0	0	0	0

Gglean010384	0	0	0	0.0681283
Gglean010385	0.726489	0.379885	0.297128	0
Gglean010386	0.0942637	0	0	0.0927861
Gglean010387	0.150561	0.0765937	0.121263	0.0720767
Gglean010388	5.788522296	5.672571739	5.412170397	4.997853182
Gglean010389	0	0	0	0
Gglean010390	0	0	0	0
Gglean010391	0.540809	0.142134	0.111574	0.133213
Gglean010392	0	0	0	0
Gglean010393	0	0	0	0
Gglean010394	0	0.123087	0	0
Gglean010395	0	0	0	0
Gglean010396	0	0	0	0
Gglean010397	0	0.199545	0.152792	0
Gglean010398	0	0.04550357	0.01778626	0.083924014
Gglean010399	0	0	0	0
Gglean010400	0.383468	0.561999	0.757314	0.822371
Gglean010401	0	0	0	0
Gglean010402	0	0	0	0
Gglean010403	0	0	0	0
Gglean010404	0	0	0	0
Gglean010405	0	0	0	0
Gglean010406	0	0	0	0
Gglean010407	0	0	0	0
Gglean010408	0	0	0	0
Gglean010409	0	0	0	0
Gglean010410	0	0	0	0
Gglean010411	24.93874766	22.00452	18.19030582	17.18928006
Gglean010412	7.558086381	7.5952694	8.979141001	9.34813
Gglean010413	3.275376061	4.1303356	4.512934	5.306323493
Gglean010414	0.427498846	0.356376892	0	0.121178622
Gglean010415	0	0	0	0
Gglean010416	0.339674	0.599072	0.940964	0.882692
Gglean010417	0	0	0	0
Gglean010418	7.02305927	6.644110607	11.747912	13.701754
Gglean010419	0	0	0	0
Gglean010420	0	0	0	0
Gglean010421	0	0	0	0
Gglean010422	59.24872	51.30131	47.355257	45.736872
Gglean010423	12.4583	9.54503	17.3128	23.6575
Gglean010424	0.829979313	0.73288	0.541302	0.894464
Gglean010425	0.829979313	0.73288	0.541302	0.894464
Gglean010426	10.23243729	9.5718528	8.6041855	7.433243255
Gglean010427	2.870904	3.525308	1.842305	1.575279

Gglean010428	0	0	0	0
Gglean010429	17.458858	18.46448	29.60706	34.53005
Gglean010430	17.458858	18.46448	29.60706	34.53005
Gglean010431	17.458858	18.46448	29.60706	34.53005
Gglean010432	0	0	0	0
Gglean010433	0	0	0	0
Gglean010434	0.382300788	0.534901302	0.788306939	0.592833687
Gglean010435	0.7448701	1.242293757	1.779122748	1.1846708
Gglean010436	0.8274517	1.09307	1.18483	1.935881
Gglean010437	0.8274517	1.09307	1.18483	1.935881
Gglean010438	0.7448701	1.242293757	1.779122748	1.1846708
Gglean010439	0.7448701	1.242293757	1.779122748	1.1846708
Gglean010440	17.86435	17.59412	20.28372	19.9136
Gglean010441	25.81307	24.481406	28.18381	29.10032
Gglean010442	23.85925387	25.10423	26.20337704	24.596184
Gglean010443	41.4695	40.9112	64.0237	65.2743
Gglean010444	60.6725	60.2214	56.1489	64.8493
Gglean010445	65.65726	67.98064	61.97897	61.44348
Gglean010446	44.1105	48.05093146	48.9373	50.7383
Gglean010447	44.1105	48.05093146	48.9373	50.7383
Gglean010448	31.92345979	27.04292175	45.65278747	57.19169215
Gglean010449	93.44644552	96.7259868	106.216072	109.7672228
Gglean010450	1.566582053	1.047259801	1.630406202	0.722680237
Gglean010451	4.873853744	4.356557203	5.439555425	6.216176849
Gglean010452	4.84652246	5.811615	6.890802169	8.818588355
Gglean010453	0.730132117	0.290148209	1.008021949	0.769804285
Gglean010454	57.67010007	74.34971046	79.795587	111.000711
Gglean010455	0	0	0	0
Gglean010456	0.121952	0.376437	0	0
Gglean010457	7.85492	10.10873	0.063087388	0.07492234
Gglean010458	9.666516255	9.231872736	12.10551587	12.75517165
Gglean010459	21.943	17.9665	27.6589	38.8765
Gglean010460	33.20163	31.22689	24.23530564	27.92328787
Gglean010461	36.1583874	34.812315	40.096513	43.33291548
Gglean010462	36.1583874	34.812315	40.096513	43.33291548
Gglean010463	0	0	0	0
Gglean010464	0.822578475	1.091206128	1.729492	1.8125485
Gglean010465	0.822578475	1.091206128	1.729492	1.8125485
Gglean010466	0	0	0	0
Gglean010467	0	0.23094059	0.039852833	0.143207012
Gglean010468	7.94557547	6.87673867	4.827404	5.3102094
Gglean010469	4.11648261	3.7074	2.71737126	2.31137
Gglean010470	4.11648261	3.7074	2.71737126	2.31137
Gglean010471	4.11648261	3.7074	2.71737126	2.31137

Gglean010472	0	0	0	0
Gglean010473	88.30614767	85.280564	70.81351092	87.60086
Gglean010474	22.141502	30.83583	12.491447	14.833367
Gglean010475	6.4913	8.77932	1.24049	0.889013
Gglean010476	0.0140528	0.0143031	0.0112596	0
Gglean010477	4.6707935	5.1395051	8.90531161	8.416317
Gglean010478	4.6707935	5.1395051	8.90531161	8.416317
Gglean010479	7.77141	7.28337	12.6244	13.64795
Gglean010480	75.85456337	79.57366676	62.83080083	64.15677223
Gglean010481	21.202379	17.30320115	15.6983	12.59991209
Gglean010482	9.26216	13.6928	6.30537	9.6993
Gglean010483	0	0.353513349	0	0
Gglean010484	0.838849988	1.275476225	2.040619155	2.54726497
Gglean010485	5.89411	5.95572	2.97183	2.40314
Gglean010486	0.371199	0.448223	0.55038	0.652449
Gglean010487	32.84730434	34.69889847	33.660333	34.71295316
Gglean010488	12.7896	10.9297	15.6608	22.9062
Gglean010489	0	0	0.267971	0
Gglean010490	0.364851453	0.922919519	1.127863	1.299262
Gglean010491	0.176074	0.134117	0.39079	0.294851
Gglean010492	0	0	0	0
Gglean010493	24.70449154	27.36710718	27.95360905	34.68637909
Gglean010494	4.82861	4.379980001	3.835283424	7.23705
Gglean010495	4.82861	4.379980001	3.835283424	7.23705
Gglean010496	0	0.476135	0	0
Gglean010497	0.85067561	0.92891567	1.57732752	1.753841028
Gglean010498	0.0366616	0.0437605	0.0346386	0.0672689
Gglean010499	4.311141	4.096688	4.378132	3.849605
Gglean010500	94.58612338	96.04218629	48.19423871	46.99371896
Gglean010501	0	0.137122	0.036694658	0.198149038
Gglean010502	2.93939	0.396103	0.828501	3.08549
Gglean010503	18.8892156	18.496168	17.631034	15.771558
Gglean010504	0	0	0	0
Gglean010505	8.039693046	7.28147328	10.23679777	14.24582135
Gglean010506	8.039693046	7.28147328	10.23679777	14.24582135
Gglean010507	11.53631412	11.73106	12.11468421	14.12400251
Gglean010508	11.53631412	11.73106	12.11468421	14.12400251
Gglean010509	22.9319	24.9829	17.205	17.4401
Gglean010510	20.29056596	24.03155589	12.22606276	10.01560563
Gglean010511	1.04286646	1.39874778	1.30209181	0.9437156
Gglean010512	1.04286646	1.39874778	1.30209181	0.9437156
Gglean010513	6.92605	6.33488	9.40928	7.48554
Gglean010514	80.63834295	81.94721007	65.41896	69.261288
Gglean010515	80.63834295	81.94721007	65.41896	69.261288

Gglean010516	80.63834295	81.94721007	65.41896	69.261288
Gglean010517	1.17408	1.23105	3.21514	3.05938
Gglean010518	101.60745	101.76598	165.17643	180.36814
Gglean010519	101.60745	101.76598	165.17643	180.36814
Gglean010520	0.212259	0.601359942	0.30798357	0.102911167
Gglean010521	0.212259	0.601359942	0.30798357	0.102911167
Gglean010522	24.32179834	1.750453333	1.926356472	1.9249466
Gglean010523	24.32179834	1.750453333	1.926356472	1.9249466
Gglean010524	0.497159	0.903359	1.21293	2.28632
Gglean010525	0	0	0	0
Gglean010526	75.67103904	78.4177	66.40715208	61.81140016
Gglean010527	0	0	0	0
Gglean010528	0	0	0	0
Gglean010529	0	0	0	0
Gglean010530	7.264412656	6.362790997	7.770611218	7.270044
Gglean010531	7.264412656	6.362790997	7.770611218	7.270044
Gglean010532	2.41998197	2.194111153	1.018638246	1.472875551
Gglean010533	2.41998197	2.194111153	1.018638246	1.472875551
Gglean010534	0.158061772	0.3837763	0.54883612	0.800472
Gglean010535	407.455	476.224	257.2765	229.9249
Gglean010536	0	0	0	0
Gglean010537	0	0.105661	0	0
Gglean010538	0.156741	0	0	0
Gglean010539	0	0.0446039	0.0349577	0
Gglean010540	0	0	0	0
Gglean010541	0	0	0	0
Gglean010542	0	0	0	0
Gglean010543	0	0	0	0
Gglean010544	20.17411274	20.23825	29.65244942	33.9728856
Gglean010545	0	0	0	0
Gglean010546	0	0	0	0
Gglean010547	0	0	0.0688235	0
Gglean010548	0	0	0	0
Gglean010549	0	0.0443468	0	0.124177
Gglean010550	0	0	0	0
Gglean010551	113.1324	109.9605	157.865466	141.756407
Gglean010552	53.0035	48.5164	51.0291	61.7535
Gglean010553	32.4783	33.8368	10.6648	8.5856
Gglean010554	85.0191496	89.80755085	64.34903585	72.22305506
Gglean010555	46.2026	45.7505	27.3251	21.9603
Gglean010556	85.0191496	89.80755085	64.34903585	72.22305506
Gglean010557	0.37607	0.13167	0.51228	0.1212
Gglean010558	9.72188	9.15953	10.47574	9.77966
Gglean010559	21.58854	22.90881	54.5467	43.09944

Gglean010560	0	0	0	0
Gglean010561	38.05700468	36.66876202	35.075822	34.06794496
Gglean010562	0.0434925	0	0.0354828	0
Gglean010563	15.10157226	16.294281	23.79159	25.691921
Gglean010564	0	0	0	0
Gglean010565	0.809839	1.44445	1.10508	2.65002
Gglean010566	1.259946	0.881653	2.3539476	2.9833507
Gglean010567	118.182	122.851	96.9833	93.3869
Gglean010568	0.624645081	0.799036006	1.159532863	1.75823387
Gglean010569	14.0472486	13.35993	18.800317	17.52956624
Gglean010570	11.38884051	12.50076088	17.62204766	20.66764483
Gglean010571	3.6129	3.65686	5.99995	6.14231
Gglean010572	143.4056	140.3667	101.3142	87.9655
Gglean010573	20.0939969	20.420613	14.354779	13.58506
Gglean010574	0.00196586	0.00956651	0.0580712	0.0100578
Gglean010575	6.93613	7.87162	7.98378	11.21955
Gglean010576	24.24131	22.94376	11.53062	11.50504
Gglean010577	10.29377099	12.16459234	5.418163133	6.136369005
Gglean010578	10.29377099	12.16459234	5.418163133	6.136369005
Gglean010579	40.54678446	39.50134	24.89998	22.62497062
Gglean010580	9.08618	11.3204	5.71881	5.53714
Gglean010581	145.5666	139.6003	86.1091	79.5628
Gglean010582	0	0	0	0
Gglean010583	1.34951637	1.328533	2.070069	1.903566364
Gglean010584	91.70637117	78.94214082	57.49345468	56.98405328
Gglean010585	22.94622	27.17885	26.31332	27.59687
Gglean010586	252.895	251.854	274.341	302.83
Gglean010587	43.33098686	45.65508	11.492349	8.847088
Gglean010588	43.33098686	45.65508	11.492349	8.847088
Gglean010589	11.29095	12.69495	8.77178	7.46227
Gglean010590	3.13154	3.24893	2.17459	1.54586
Gglean010591	19.50542	22.38911	8.78731	8.19484
Gglean010592	86.75138031	88.06737	19.34882964	15.52225134
Gglean010593	2.095923	4.782903949	3.308922	5.470276
Gglean010594	43.861	39.3952	45.3445	38.7289
Gglean010595	1.6083092	1.36278471	2.371017511	2.471771003
Gglean010596	43.45529304	43.57054603	38.3782971	37.37002284
Gglean010597	49.452	51.49126	57.78083	58.32477
Gglean010598	23.259	24.627667	15.7299926	15.392997
Gglean010599	0	0	0	0.0419374
Gglean010600	41.797	35.9956	31.6621	31.1283
Gglean010601	32.9944	11.163	0.00150716	0.0590043
Gglean010602	3.47E-05	0.000335411	2.49E-06	0.051159
Gglean010603	12.1222	13.4666	12.2314	15.5079

Gglean010604	3.38993	0	2.84466	8.47046
Gglean010605	37.5915	31.4937	43.9485	79.9533
Gglean010606	182.42328	99.33855647	198.27685	237.2461199
Gglean010607	6.3556026	5.550795019	4.379723062	6.04885341
Gglean010608	70.7110201	83.95685	112.53636	131.14478
Gglean010609	0	0	0	0.0331073
Gglean010610	85.50729326	90.837338	42.50127859	30.119635
Gglean010611	85.50729326	90.837338	42.50127859	30.119635
Gglean010612	0.195907767	0.360982726	0.191309739	0.150860145
Gglean010613	0.113509	0	0.273525	0.108571
Gglean010614	24.8952455	25.90904054	18.12474954	14.87972001
Gglean010615	24.8952455	25.90904054	18.12474954	14.87972001
Gglean010616	21.683439	24.764835	14.6196809	13.49146
Gglean010617	21.683439	24.764835	14.6196809	13.49146
Gglean010618	29.47614593	28.62822621	26.63203094	24.68644851
Gglean010619	29.47614593	28.62822621	26.63203094	24.68644851
Gglean010620	29.47614593	28.62822621	26.63203094	24.68644851
Gglean010621	0	0	0	0
Gglean010622	5.38141	3.81251	3.14348	1.99703
Gglean010623	2.74226	3.82211	6.43E-07	3.05E-05
Gglean010624	0.0575275	0.0598079	0	0
Gglean010625	0.370418137	0.806814	0.25774	0.393754
Gglean010626	0.370418137	0.806814	0.25774	0.393754
Gglean010627	14.8763	28.5135	2.71063	0.97274
Gglean010628	139.03084	130.70658	65.41463	44.54978
Gglean010629	49.18009096	47.99298886	46.19213801	46.48313976
Gglean010630	49.18009096	47.99298886	46.19213801	46.48313976
Gglean010631	0	0.0908244	0	0.0838511
Gglean010632	0	0.178305	0	0.082783
Gglean010633	48.77436	56.447941	31.57737	28.54590011
Gglean010634	0.34353	1.10431	1.11677	2.01571
Gglean010635	11.539883	9.692660411	7.400502	6.5722337
Gglean010636	11.539883	9.692660411	7.400502	6.5722337
Gglean010637	0.561100208	1.142264	0.977255	0.881983
Gglean010638	11.92846848	14.03040914	16.51053411	21.42232028
Gglean010639	12.27739373	16.66622	18.283363	23.54765
Gglean010640	58.04817	57.86714	52.1556	46.55124
Gglean010641	58.04817	57.86714	52.1556	46.55124
Gglean010642	14.3871	13.92732	8.906132458	10.04067
Gglean010643	36.66084	42.09652	25.73884	22.28373
Gglean010644	15.76436	14.69827	10.17162	8.73595
Gglean010645	15.00465	16.58738	8.39346	6.21026
Gglean010646	9.05913	13.854259	19.49846	21.76737
Gglean010647	45.48401	41.539586	37.96926031	37.08099

Gglean010648	13.46990622	14.59941113	12.54197252	11.804476
Gglean010649	0	0	0	0
Gglean010650	8.32558	8.45674	8.13771	7.31126
Gglean010651	8.24957	6.8331	4.8695	4.95773
Gglean010652	0	0	0	0
Gglean010653	51.82548	53.83628	35.61769	37.04585
Gglean010654	17.69758	19.057892	5.835108	4.364104
Gglean010655	16.1719	15.3133	6.28277	4.03534
Gglean010656	1.344837003	1.495546013	1.85957082	1.907519687
Gglean010657	1.344837003	1.495546013	1.85957082	1.907519687
Gglean010658	30.14542	33.27266	31.48924	31.22231
Gglean010659	30.14542	33.27266	31.48924	31.22231
Gglean010660	40.30033	38.89885	46.12035	53.5364
Gglean010661	16.52163419	15.491056	24.629647	24.36921957
Gglean010662	30.73197	28.030039	36.95847	37.43924
Gglean010663	2.46091	3.08686	2.87221	3.97946
Gglean010664	11.3168	11.4041	6.8823	6.84961
Gglean010665	47.274601	50.52213371	68.179305	77.360497
Gglean010666	49.138896	48.23703449	56.62668877	54.19361508
Gglean010667	14.47774	13.68143	12.14001	14.29712
Gglean010668	4.6347601	5.191026594	7.903899051	8.517687
Gglean010669	0.0852124	0	0	0
Gglean010670	98.3336	107.4681	74.9536	68.3856
Gglean010671	253.3446	231.0016	601.081	655.125
Gglean010672	0.112891866	0.036724495	0.216624504	0.060446893
Gglean010673	19.09659888	17.30443743	31.0480882	30.3522335
Gglean010674	11.031425	14.926501	5.247828	4.427663624
Gglean010675	248.3308	224.9596	203.3439	203.587
Gglean010676	248.3308	224.9596	203.3439	203.587
Gglean010677	1.37473	2.4114	0.668412	2.09561
Gglean010678	4.53565	5.49202	5.41199	5.84344
Gglean010679	69.22014	74.09906	67.91758	96.11195
Gglean010680	34.0600133	30.20539624	24.46249442	23.25361602
Gglean010681	23.69688	23.265195	22.67324	23.03891
Gglean010682	134.1052746	123.2739431	158.782475	155.7554046
Gglean010683	37.221172	43.13885202	43.94704	42.79966
Gglean010684	0	0	0.0952936	0
Gglean010685	3.31585	3.90616	1.46518	1.73028
Gglean010686	3.486762	3.500899648	5.805702	5.781412472
Gglean010687	8.2608	6.86294	8.47324	9.78553
Gglean010688	0	0	0	0
Gglean010689	65.1835	59.1525	46.6651	40.5505
Gglean010690	8.019020502	7.382768749	10.08068003	12.22528024
Gglean010691	45.8513	36.3605	51.2019	72.101

Gglean010692	8.40549	0	0	0
Gglean010693	0	0	0	0
Gglean010694	0	0	0	0
Gglean010695	35.87138	43.48215	24.67007	20.89642
Gglean010696	28.02237241	41.64036401	36.85407399	44.3865054
Gglean010697	56.4046	64.4755	27.91135	28.37855
Gglean010698	1.92773828	2.216547211	2.967068388	3.767305776
Gglean010699	3.448422644	4.415639	6.983881	7.30056
Gglean010700	16.54191	15.63525	12.0263755	14.1853
Gglean010701	0.685913	0.486982	0.863131	0.88122659
Gglean010702	7.60394	7.676490015	9.304068	12.53992
Gglean010703	22.986769	21.78433	33.09996	39.0223
Gglean010704	5.1118	3.3289	5.64444	6.42486
Gglean010705	6.805917	8.070614	9.095017	11.95594
Gglean010706	16.9530002	15.61590009	9.957690617	7.341830061
Gglean010707	16.3936306	13.8654248	23.10947209	26.77312269
Gglean010708	0	0	0	5.84138
Gglean010709	5.24819196	6.203830022	6.810226948	9.568541001
Gglean010710	14.4514	11.2908	14.4519	19.9544
Gglean010711	11.922	8.97521	10.2751	13.5785
Gglean010712	4.0188	3.736184	4.55663	7.84669
Gglean010713	77.44192311	99.20115553	68.02358095	73.462207
Gglean010714	10.207366	13.019	10.010432	8.13287
Gglean010715	17.56047	18.12748	14.38433	13.505011
Gglean010716	23.5597	24.2164	21.4131	21.4909
Gglean010717	24.86723	27.97358411	24.23782044	28.66902611
Gglean010718	24.86723	27.97358411	24.23782044	28.66902611
Gglean010719	47.9551	41.9984	26.222	22.0437
Gglean010720	0.4986431	0.53218464	1.09049687	1.35528732
Gglean010721	32.39372827	32.25224011	47.7243893	52.13459173
Gglean010722	111.2362	115.265	107.8109	110.5088
Gglean010723	6.02266	4.5732	3.74345	5.11935
Gglean010724	62.20992	61.3279058	36.90931	33.33185009
Gglean010725	62.20992	61.3279058	36.90931	33.33185009
Gglean010726	62.20992	61.3279058	36.90931	33.33185009
Gglean010727	149.20773	158.61347	181.96988	263.01655
Gglean010728	69.11775	57.42867	45.02525	35.12332
Gglean010729	69.11775	57.42867	45.02525	35.12332
Gglean010730	20.44978	20.24729	26.54342	24.0532
Gglean010731	34.1	28.467072	30.151743	27.229282
Gglean010732	1129.210049	1088.110036	702.7790285	715.5550166
Gglean010733	1129.210049	1088.110036	702.7790285	715.5550166
Gglean010734	40.5152	41.0917	34.0255	33.8562
Gglean010735	12.916055	16.024151	23.33785	21.97871077

Gglean010736	12.916055	16.024151	23.33785	21.97871077
Gglean010737	25.41160183	27.66257708	38.42169087	48.65520748
Gglean010738	46.96275549	49.62484695	47.57345029	61.29470729
Gglean010739	46.96275549	49.62484695	47.57345029	61.29470729
Gglean010740	4.276985	3.812162	5.91539	6.547243
Gglean010741	4.276985	3.812162	5.91539	6.547243
Gglean010742	102.7934137	103.5617559	119.8215005	128.6161739
Gglean010743	30.86536	27.74392438	30.49012471	32.205925
Gglean010744	32.9159	33.8056	29.00985	32.8313
Gglean010745	52.8104	44.8132	47.4832	47.052
Gglean010746	31.71886191	32.754919	33.2386803	32.932174
Gglean010747	28.20442708	23.750484	29.1527394	28.4172931
Gglean010748	28.20442708	23.750484	29.1527394	28.4172931
Gglean010749	28.20442708	23.750484	29.1527394	28.4172931
Gglean010750	61.826	70.6342	42.3485	40.65443
Gglean010751	18.03247	18.4686	12.482714	11.56563
Gglean010752	0.510677	0	0	0
Gglean010753	15.85673	16.02041	13.92214	13.50349
Gglean010754	20.13287049	20.12956	14.7183	17.228238
Gglean010755	20.13287049	20.12956	14.7183	17.228238
Gglean010756	32.07428	32.32094	12.38834192	11.47101608
Gglean010757	55.57802347	48.3274	32.81046348	32.08894936
Gglean010758	55.57802347	48.3274	32.81046348	32.08894936
Gglean010759	3.024048079	2.902966038	3.483460604	3.651655722
Gglean010760	192.9443	215.4072	202.773	199.2511
Gglean010761	15.42168158	16.59276474	17.44044531	16.46234054
Gglean010762	135.6705723	126.22811	97.7149126	93.51925681
Gglean010763	18.0418	18.2663	15.3688	13.5791
Gglean010764	29.489	21.8509	11.3753	11.917
Gglean010765	137.8873	155.3624	173.7694	206.278
Gglean010766	176.959	159.288	144.137	172.777
Gglean010767	58.27177594	42.73589717	40.51079443	33.05783592
Gglean010768	9.269492	8.381696	8.42758	9.30662
Gglean010769	43.87733	42.41108	13.652214	10.33529669
Gglean010770	125.1162	121.4318	89.3219	71.4801
Gglean010771	12.71909	18.17551	18.39056	20.4284
Gglean010772	16.94845	17.88719	9.80365	8.82941
Gglean010773	2.743273269	3.264361363	3.036940002	4.674871748
Gglean010774	5.80641976	7.05823	9.96952	13.65319
Gglean010775	8.0865	6.92315	2.50716	3.20563
Gglean010776	14.02847005	12.89484184	15.86034273	16.31444023
Gglean010777	23.32187335	26.49105345	26.11868903	25.57315506
Gglean010778	982.508	1035.602	638.306	628.109
Gglean010779	11.9904	12.6691	8.93397	10.2824

Gglean010780	50.468774	54.11017051	49.36496775	46.03587415
Gglean010781	50.468774	54.11017051	49.36496775	46.03587415
Gglean010782	19.8848	22.6674	11.8759	12.2577
Gglean010783	11.6828	9.88651	7.02057	7.03162
Gglean010784	50.468774	54.11017051	49.36496775	46.03587415
Gglean010785	0	0	0	0
Gglean010786	14.9618	16.41762	7.24208	6.45228
Gglean010787	39.19089141	37.47670435	62.2355032	72.10820875
Gglean010788	39.19089141	37.47670435	62.2355032	72.10820875
Gglean010789	18.00338	20.84382	19.32895	20.695002
Gglean010790	18.00338	20.84382	19.32895	20.695002
Gglean010791	18.00338	20.84382	19.32895	20.695002
Gglean010792	16.89624242	16.73051336	6.395204634	5.875626713
Gglean010793	0	0	0.40129	0
Gglean010794	3.938795996	4.366021306	4.439313142	5.318271452
Gglean010795	3.938795996	4.366021306	4.439313142	5.318271452
Gglean010796	0.613437	0.67414	0.0329175	0
Gglean010797	122.7260736	129.3104253	156.1545753	155.6919634
Gglean010798	122.7260736	129.3104253	156.1545753	155.6919634
Gglean010799	0.153562204	0.238476863	0.3854798	0.403003559
Gglean010800	92.3396	83.898	76.3394	75.1661
Gglean010801	36.04345	40.49336139	34.52549032	33.204824
Gglean010802	0.523426181	1.083186561	0.8947175	0.87182339
Gglean010803	0.523426181	1.083186561	0.8947175	0.87182339
Gglean010804	96.476022	117.68201	146.76813	178.85952
Gglean010805	61.57557	64.4085	65.22647	112.33476
Gglean010806	3.49869	3.53275	4.79531	6.58454
Gglean010807	61.57557	64.4085	65.22647	112.33476
Gglean010808	25.92749	23.59205	14.87806	14.62147
Gglean010809	0	0.229691	0	0.211054
Gglean010810	26.56877	28.5909	11.33534	10.0209
Gglean010811	23.38910891	23.64066558	23.0345485	24.85831138
Gglean010812	29.70710528	27.79037892	19.18265628	16.25649599
Gglean010813	7.468704104	9.20598	6.973860175	7.81065
Gglean010814	5.80641976	7.05823	9.96952	13.65319
Gglean010815	41.781599	45.839242	27.864208	25.7445458
Gglean010816	35.40408	43.35559	38.96033	38.14032
Gglean010817	78.9486	84.0912	67.3668	60.2187
Gglean010818	10.49797	8.93328	4.07341	5.17057
Gglean010819	53.80738	49.97882	38.12245	35.3844
Gglean010820	53.80738	49.97882	38.12245	35.3844
Gglean010821	20.20725	25.7668	11.0303	7.69904
Gglean010822	21.95211	19.27278	11.46953	10.81872
Gglean010823	125.848236	139.3521029	73.53300171	55.73060403

Gglean010824	45.94823921	38.11324634	25.61990251	25.10510498
Gglean010825	25.75961	22.60312	20.62452	18.90266
Gglean010826	22.39569	22.79228	23.45665	19.44991
Gglean010827	54.54813	53.02911	46.71228	38.935433
Gglean010828	0.901675	0.00063545	1.26397	5.96E-05
Gglean010829	9.72408758	9.367610139	6.243762628	5.016918317
Gglean010830	30.230655	25.64582238	46.403359	46.6558999
Gglean010831	41.65928027	46.07460792	36.02486359	35.11770004
Gglean010832	22.74194	20.297144	16.03703	15.39295
Gglean010833	27.96247	24.77472	30.68762	32.1799
Gglean010834	2.058500012	1.869663636	3.146224801	2.9270055
Gglean010835	35.62615	33.50996	16.96962	12.44124
Gglean010836	0.541879	1.44218	1.56023	0.266875
Gglean010837	0	0.387896	0	0
Gglean010838	17.78572108	18.79760599	23.69884231	28.9647284
Gglean010839	66.1219	63.45878	48.45889	39.7207089
Gglean010840	14.4889	14.6338	10.9049	10.0763
Gglean010841	82.798677	82.48508	66.8028	62.5355
Gglean010842	65.5063103	65.210464	52.52748905	48.76155566
Gglean010843	65.5063103	65.210464	52.52748905	48.76155566
Gglean010844	9.637521116	9.7451104	16.063102	15.26172226
Gglean010845	20.9352	25.9523	9.03917	9.68099
Gglean010846	34.61381	63.86038	46.49817	36.11509
Gglean010847	0.112101	0.116504	0.0925572	0
Gglean010848	28.80242	32.63422001	35.75395	36.03183002
Gglean010849	28.80242	32.63422001	35.75395	36.03183002
Gglean010850	34.06577004	41.49643	52.33674	51.7946936
Gglean010851	34.06577004	41.49643	52.33674	51.7946936
Gglean010852	16.64609	18.89186	10.50312	9.55427
Gglean010853	0	0.0541272	0	0
Gglean010854	0.046464146	0	1.83E-86	0
Gglean010855	42.6348	45.4652	12.03703	9.50296
Gglean010856	0.1217547	0.369681191	0.464361	1.007912
Gglean010857	1.597334391	1.887106	2.48063	3.056177
Gglean010858	59.13488	21.10909	28.56501	35.87536
Gglean010859	59.13488	21.10909	28.56501	35.87536
Gglean010860	61.252651	57.560657	62.998028	62.930583
Gglean010861	20.91234	17.036561	28.381	33.72004
Gglean010862	14.4632	15.6031	11.6523	9.72851
Gglean010863	1190.11206	1390.856055	801.2890392	794.4880386
Gglean010864	10.789619	11.69366779	15.10350588	18.30418122
Gglean010865	0	12.056	4.28196	20.2654
Gglean010866	3.394791793	2.82753302	3.958499958	4.695033262
Gglean010867	3.394791793	2.82753302	3.958499958	4.695033262

Gglean010868	54.9292	53.1977	39.5186	39.1136
Gglean010869	393.4169	280.0032	221.6096	309.8155
Gglean010870	3.972883	5.375526	3.186462	3.7121791
Gglean010871	33.43597922	34.93483	23.455866	22.41453
Gglean010872	9.00722	8.733	7.13704	8.18358
Gglean010873	23.6138041	22.5121617	30.94648594	36.193504
Gglean010874	489.451125	407.682771	559.975148	868.19364
Gglean010875	0.279806	0.11496	0.676294	0.376547
Gglean010876	10.197	7.43161	10.285	15.6851
Gglean010877	19.10099	15.06206	20.94656	16.22045
Gglean010878	19.10099	15.06206	20.94656	16.22045
Gglean010879	88.01452162	99.69268959	131.1297497	147.9065229
Gglean010880	88.01452162	99.69268959	131.1297497	147.9065229
Gglean010881	14.13466	13.58957	13.18695	16.88532
Gglean010882	145.3460814	155.782286	160.9112736	179.7685654
Gglean010883	0	0	0	5.84138
Gglean010884	0	0	0	0
Gglean010885	26.848718	26.865801	39.9427007	44.775357
Gglean010886	50.2459522	53.03031	31.0738634	25.95587
Gglean010887	39.9284633	49.65053164	37.26737357	34.470953
Gglean010888	2.01594	2.38539	0.777698	1.48016
Gglean010889	0	0	0	0
Gglean010890	10.70405006	9.1371363	12.75199377	18.663266
Gglean010891	10.70405006	9.1371363	12.75199377	18.663266
Gglean010892	18.4975748	11.7542521	14.443173	93.2111181
Gglean010893	18.4975748	11.7542521	14.443173	93.2111181
Gglean010894	16.61635	12.99008	12.52963	13.01257
Gglean010895	120.05643	116.2566995	83.82388399	79.660247
Gglean010896	4.029002882	4.90142563	5.798981	6.677979233
Gglean010897	4.029002882	4.90142563	5.798981	6.677979233
Gglean010898	0	0	0	0
Gglean010899	0	0	0	0
Gglean010900	0	0	0	0
Gglean010901	51.13866167	49.18174008	53.20610242	51.0607448
Gglean010902	0	0	0	0
Gglean010903	3.969085	5.381507	9.22615088	9.608560001
Gglean010904	112.7000026	106.4006885	59.548549	57.10909
Gglean010905	3.658386	3.638669	4.774287	7.422624872
Gglean010906	111.6547691	112.9650959	127.9004829	118.6011575
Gglean010907	111.6547691	112.9650959	127.9004829	118.6011575
Gglean010908	0	0	0	0
Gglean010909	40.1661	39.8532	39.5888	35.9542
Gglean010910	34.4998	30.2181	17.88414	22.9854
Gglean010911	34.4998	30.2181	17.88414	22.9854

Gglean010912	98.16640002	105.296596	65.5430008	60.6138001
Gglean010913	24.39728207	21.9895778	22.73374	26.24442933
Gglean010914	16.58293	17.26742	16.97847	18.11991
Gglean010915	16.58293	17.26742	16.97847	18.11991
Gglean010916	33.9799	32.2676	25.5492	31.2107
Gglean010917	31.32707	29.73357	21.12669	16.67576
Gglean010918	31.32707	29.73357	21.12669	16.67576
Gglean010919	5.54168	6.07451	6.02655	12.0323
Gglean010920	33.89301	44.1492	23.16767	24.62103
Gglean010921	33.89301	44.1492	23.16767	24.62103
Gglean010922	8.113631471	6.612207	10.801932	15.03325
Gglean010923	44.92985	45.07216	43.48156	48.93333
Gglean010924	51.99852	53.01295	38.84121	39.30532
Gglean010925	51.99852	53.01295	38.84121	39.30532
Gglean010926	0.210421	0.124333	0.26854	0.202049
Gglean010927	47.11589	50.3298	39.62223	42.12182
Gglean010928	0	0	0	0.265029
Gglean010929	0.0884249	0.0915659	0.356658	0.169209
Gglean010930	2.616526856	3.458077	1.275471022	1.294246844
Gglean010931	1.572821	1.253400637	2.414983	1.91688904
Gglean010932	6.509086	5.827775	3.238451	2.970933965
Gglean010933	246.611	222.357	299.544	416.046
Gglean010934	0	0.0916877	0	0
Gglean010935	0	0	0	0
Gglean010936	0.161781	0	0	0
Gglean010937	0.921531	0	0	0
Gglean010938	0	0	0.129545	0
Gglean010939	2.366431805	2.6545362	2.80691261	3.0761995
Gglean010940	10.603553	14.12320549	8.721671	8.31386
Gglean010941	19.52911134	19.9111	18.053462	17.10049
Gglean010942	19.52911134	19.9111	18.053462	17.10049
Gglean010943	18.569358	16.270963	23.852166	20.308729
Gglean010944	48.21788	44.074212	33.791834	33.03466078
Gglean010945	17.42979958	19.9438693	13.44989651	14.51651856
Gglean010946	2.506016391	2.142777941	4.799737183	4.562551312
Gglean010947	0.494018507	0.197198338	0.342814669	0.522652357
Gglean010948	0.494018507	0.197198338	0.342814669	0.522652357
Gglean010949	43.1439445	47.75787805	29.8153888	23.37108695
Gglean010950	43.1439445	47.75787805	29.8153888	23.37108695
Gglean010951	46.2733	45.1122	38.1081	39.391
Gglean010952	0.784443	0.485183	0.822	0.925363
Gglean010953	25.17582774	24.7697806	25.684197	23.711315
Gglean010954	195.06285	200.91166	290.6743678	315.2863758
Gglean010955	195.06285	200.91166	290.6743678	315.2863758

Gglean010956	83.03129	88.17603	46.70145	38.8307052
Gglean010957	0.413199094	0.277205526	0.669966253	0.370972005
Gglean010958	0.413199094	0.277205526	0.669966253	0.370972005
Gglean010959	10.09998973	9.783937061	15.37214018	20.157804
Gglean010960	10.09998973	9.783937061	15.37214018	20.157804
Gglean010961	0.108915608	0.02227743	0.087334527	0.062303856
Gglean010962	0.108915608	0.02227743	0.087334527	0.062303856
Gglean010963	1.35013	2.44302	2.91794	2.46785
Gglean010964	15.50507984	17.16695454	27.23564951	26.02982432
Gglean010965	15.50507984	17.16695454	27.23564951	26.02982432
Gglean010966	22.607252	31.290311	34.24191	29.160258
Gglean010967	22.607252	31.290311	34.24191	29.160258
Gglean010968	36.14378784	39.9404561	63.9530687	60.9465529
Gglean010969	38.42431	31.74511	35.078834	32.874617
Gglean010970	38.42431	31.74511	35.078834	32.874617
Gglean010971	0.337423	0.189829	0.307997	1.301027
Gglean010972	0	0	0	0
Gglean010973	12.10222	11.25159	24.5586	23.97224
Gglean010974	8.637851	7.71136	3.502590254	3.143375
Gglean010975	30.95807204	32.45469	50.21921	52.94181
Gglean010976	11.386	9.34236	7.6601	9.0449
Gglean010977	1.11472	1.8756	2.09257	1.75671
Gglean010978	9.59054	9.54136	4.53328	4.25625
Gglean010979	21.40462	19.39302	18.68787	19.98849
Gglean010980	282.5317667	297.5736188	311.3901768	350.0891529
Gglean010981	0	0	0	0
Gglean010982	31.4273	32.3631	24.12349	21.32773
Gglean010983	67.25868801	63.8894207	82.04092918	80.18448548
Gglean010984	50.4155	47.8145	23.40888	29.963
Gglean010985	13.80274	16.17103	11.818046	13.88687
Gglean010986	13.80274	16.17103	11.818046	13.88687
Gglean010987	19.1398	18.4679	11.8538	10.7844
Gglean010988	1.469716527	2.289740532	0.291630099	0.5443675
Gglean010989	47.6908	46.9293	42.5651	40.0422
Gglean010990	23.50984395	25.76974754	20.41387512	18.8167466
Gglean010991	20.40790402	23.65257233	20.61094122	21.78430187
Gglean010992	20.40790402	23.65257233	20.61094122	21.78430187
Gglean010993	41.91511	36.81418279	64.23033902	68.40411502
Gglean010994	41.91511	36.81418279	64.23033902	68.40411502
Gglean010995	41.91511	36.81418279	64.23033902	68.40411502
Gglean010996	95.36180133	95.81341129	121.0649782	142.6428029
Gglean010997	95.36180133	95.81341129	121.0649782	142.6428029
Gglean010998	95.36180133	95.81341129	121.0649782	142.6428029
Gglean010999	0.117975	0.24451	0.04787	0.0568276

Gglean011000	0.059333	0.0613544	0.336726	0.0570973
Gglean011001	0	0	0	0
Gglean011002	5.4044744	5.650089647	3.240602617	5.254652278
Gglean011003	33.19153	33.05791	29.939995	27.551824
Gglean011004	0	0	0	0
Gglean011005	28.43119969	29.12675241	39.587392	44.96186
Gglean011006	0.993126	1.030320023	0.5297964	0.110773
Gglean011007	0.39096	0.277046	0.343205	0.33189
Gglean011008	4.115599804	3.676240934	6.80419	6.885442
Gglean011009	0	0.221611	0.172048	0.409188
Gglean011010	4.115599804	3.676240934	6.80419	6.885442
Gglean011011	15.2613	17.1331	12.2443	12.9892
Gglean011012	3.153112	2.687128	5.088626	6.030625
Gglean011013	61.59523	73.73862	93.6187	100.399
Gglean011014	15.43798278	16.87676969	10.927889	11.01461
Gglean011015	0	0	0	0
Gglean011016	59.45017	58.700224	38.037761	33.912057
Gglean011017	59.45017	58.700224	38.037761	33.912057
Gglean011018	18.27287	20.423041	20.01889	32.67589
Gglean011019	31.6259	33.7818	25.9429	27.2236
Gglean011020	8.56	7.51022	5.368399553	3.848773791
Gglean011021	87.4668	86.9666	70.8696	73.9678
Gglean011022	0.183277	0	0.0986605	0
Gglean011023	6.2630195	5.7102734	4.747399	3.6218074
Gglean011024	20.85304	28.29957	16.6002	29.28127
Gglean011025	0	0.000326584	0.124071	0.2197
Gglean011026	35.67	36.7575	24.964	25.0084
Gglean011027	10.69187	12.82008	8.29239	7.97277
Gglean011028	3.88195	2.83406	3.1857	3.76953
Gglean011029	81.4391	86.9558	49.59471	46.10526
Gglean011030	8.396095834	8.941757813	5.228821773	4.785200625
Gglean011031	14.73542	17.30906	8.81055	8.58513
Gglean011032	64.17247304	60.18269251	47.927187	42.485222
Gglean011033	181.61378	199.9110351	126.876728	116.9220396
Gglean011034	44.24391	37.227075	37.8994716	35.17366
Gglean011035	20.90740818	20.86824	23.95202725	33.392071
Gglean011036	20.90740818	20.86824	23.95202725	33.392071
Gglean011037	16.02125903	14.6011615	12.72477822	10.21973367
Gglean011038	16.02125903	14.6011615	12.72477822	10.21973367
Gglean011039	16.02125903	14.6011615	12.72477822	10.21973367
Gglean011040	69.69642122	76.37918921	70.0905	79.756984
Gglean011041	6.782725	7.26041	3.813072256	4.600461
Gglean011042	69.69642122	76.37918921	70.0905	79.756984
Gglean011043	0	0	0	0

Gglean011044	0	0	0	0
Gglean011045	1.44121	1.1869	0.300796	0
Gglean011046	8.378794842	10.65467892	9.7153202	12.56890013
Gglean011047	0.280921	0.465011	0.47945	0.567067
Gglean011048	12.47844	14.158861	28.890713	29.27283855
Gglean011049	12.47844	14.158861	28.890713	29.27283855
Gglean011050	87.31391	66.48959	50.556834	37.85607714
Gglean011051	0	0	0	0
Gglean011052	40.981492	33.45069218	31.86566488	33.43240332
Gglean011053	40.981492	33.45069218	31.86566488	33.43240332
Gglean011054	0	0	0	0
Gglean011055	0.872077	0	0.720355	1.72066
Gglean011056	1.26101	1.83359	2.04884	2.80706
Gglean011057	0.444169	0.661835	0.537815	0.282682
Gglean011058	36.64122122	31.25560443	35.97045942	38.05257315
Gglean011059	36.64122122	31.25560443	35.97045942	38.05257315
Gglean011060	51.4536	62.5530002	78.3221	99.5326
Gglean011061	3.903141245	4.522231065	5.956860408	6.737077
Gglean011062	3.903141245	4.522231065	5.956860408	6.737077
Gglean011063	3.903141245	4.522231065	5.956860408	6.737077
Gglean011064	6.83499383	8.296344	9.343658301	9.821827
Gglean011065	6.83499383	8.296344	9.343658301	9.821827
Gglean011066	46.2501089	51.40277152	42.63109	38.93253774
Gglean011067	10.4878	8.1219	12.0383	16.0459
Gglean011068	30.8291	28.5352	26.4195	27.9043
Gglean011069	0.364088099	0.615857833	0.945822421	0.6024459
Gglean011070	35.997903	38.43754116	78.2406622	75.76078557
Gglean011071	35.997903	38.43754116	78.2406622	75.76078557
Gglean011072	4.52027	6.61671	6.67982	7.15977
Gglean011073	0.422985	0.197528	0.343249	0.036977
Gglean011074	18.8188	19.57929	7.88818	7.2909
Gglean011075	9.683816	11.25792	9.08407	7.58707
Gglean011076	5.7398	4.65042	2.99842	3.37663
Gglean011077	0.383834	0	0.159517	0
Gglean011078	36.9001	36.7838	20.02455	16.475805
Gglean011079	0.202632	0.0887523	0.046842	0
Gglean011080	4.363909966	5.98470679	6.190878008	8.225086
Gglean011081	4.363909966	5.98470679	6.190878008	8.225086
Gglean011082	0	0	0	0
Gglean011083	0	0	0.587547	0
Gglean011084	1.455451399	2.441945754	2.660211103	3.614304886
Gglean011085	0	0	0	0
Gglean011086	17.09682562	19.9432807	15.5515981	15.87001733
Gglean011087	6.089251	6.850716367	7.868549	6.520016

Gglean011088	43.4391355	33.81477075	20.43800828	22.63865323
Gglean011089	43.4391355	33.81477075	20.43800828	22.63865323
Gglean011090	43.4391355	33.81477075	20.43800828	22.63865323
Gglean011091	33.7793957	35.17742488	60.01980649	65.12422725
Gglean011092	17.09856757	19.71190083	11.18884	11.82152949
Gglean011093	0.142571	0.154562	2.499684	1.998280226
Gglean011094	0.873473	0.519526	0.922806	1.10047
Gglean011095	52.70407	56.0925	53.87206183	50.86429
Gglean011096	19.63496737	20.6678177	7.267758	6.262366085
Gglean011097	0	0	0	0
Gglean011098	4.63561	4.62181	6.66664	7.56452
Gglean011099	7.435113	6.012865229	11.24591248	10.66391
Gglean011100	45.26247	44.90919	33.51628	35.96344
Gglean011101	0	0	0	0
Gglean011102	30.01751	34.16819	5.646131	4.355691
Gglean011103	35.6141	33.3409	24.8455	23.02966
Gglean011104	66.02443	71.8557	82.0862	93.1399
Gglean011105	10.32528988	10.21494	16.136625	16.3489309
Gglean011106	10.32528988	10.21494	16.136625	16.3489309
Gglean011107	7.598657834	7.0198392	4.755688978	5.405825307
Gglean011108	0.0383716	0.0395619	0	0.0370837
Gglean011109	9.69258	10.4168	8.93152	8.96799
Gglean011110	0	0	0	0.636342
Gglean011111	21.04653756	20.71909548	34.98950901	33.98158477
Gglean011112	35.8278	36.08335	21.25063	23.1043
Gglean011113	35.8278	36.08335	21.25063	23.1043
Gglean011114	27.16561	33.945822	22.05604	21.961152
Gglean011115	0	0	0	0
Gglean011116	0	0	0	0
Gglean011117	0	0.346879	0	0.477677
Gglean011118	12.76184	10.028996	9.937	9.2156
Gglean011119	16.9272	22.13733	29.07619	26.51689
Gglean011120	16.9272	22.13733	29.07619	26.51689
Gglean011121	12.59294	10.26554464	15.17174	18.34921
Gglean011122	1453.476	1538.939	218.5713	72.1326
Gglean011123	0	0	0	0
Gglean011124	31.62983057	35.34884	45.30163	58.690022
Gglean011125	31.62983057	35.34884	45.30163	58.690022
Gglean011126	12.267	12.5566	19.2851	19.7879
Gglean011127	34.572323	33.690249	49.21682	65.45861
Gglean011128	66.6886	61.9959	30.04128	27.63135
Gglean011129	21.09107789	25.496296	21.36267179	24.68953
Gglean011130	21.09107789	25.496296	21.36267179	24.68953
Gglean011131	19.6632164	16.43707043	19.6242343	22.53315687

Gglean011132	19.6632164	16.43707043	19.6242343	22.53315687
Gglean011133	0	0	0	0
Gglean011134	56.16092062	43.24682387	28.89607346	33.05055409
Gglean011135	56.16092062	43.24682387	28.89607346	33.05055409
Gglean011136	0.545441	0.141756	0.332503	0.394886
Gglean011137	120.59124	130.20617	101.09608	110.1649
Gglean011138	39.9425	39.9658	43.2938	44.0219
Gglean011139	5.221584258	3.881746433	3.537446	2.816524392
Gglean011140	13.52131057	13.506924	5.728568	5.2319708
Gglean011141	10.171061	10.660179	6.410353	5.49127
Gglean011142	0	0.200117	0.154837	1.10608
Gglean011143	0.637706135	0.60639162	0.662993	0.2480082
Gglean011144	7.32706	6.12308	3.21598	3.4748
Gglean011145	0	0	0	0
Gglean011146	0.254994	0.329033	0.519753	0.369613
Gglean011147	144.9793	138.33869	126.96849	128.37116
Gglean011148	144.9793	138.33869	126.96849	128.37116
Gglean011149	1.53651	0.539406	0.973685	1.16169
Gglean011150	0.435766	0.16841	0.219952	0.312641
Gglean011151	40.39201538	45.77784	63.87431	71.34001
Gglean011152	43.575545	44.57364699	43.72864534	44.12788005
Gglean011153	30.46574	24.51127	17.04546	15.63186
Gglean011154	0.250955	0.456576842	0.148377	0.145773
Gglean011155	0.106613	0	0	0
Gglean011156	44.550533	49.709232	26.568149	23.66958991
Gglean011157	25.79179652	24.90808195	33.06538838	35.38351383
Gglean011158	12.7592	13.5157	12.5652	9.48492
Gglean011159	0.893332036	1.207308271	1.307761778	1.623864495
Gglean011160	1.64E-18	0	8.80E-08	0.0812078
Gglean011161	3.67115	3.48539	5.02232	2.94559
Gglean011162	5.11965	4.92208	4.80616	3.77615
Gglean011163	286.9902	259.221	248.2638	235.8325
Gglean011164	286.9902	259.221	248.2638	235.8325
Gglean011165	0.741626098	0.806385313	0.62254203	0.93760409
Gglean011166	5.68792	7.5265	1.29952	0.386105
Gglean011167	4.07365176	3.919798083	6.289971055	6.408001
Gglean011168	4.07365176	3.919798083	6.289971055	6.408001
Gglean011169	35.40262152	32.67382034	36.43458511	42.66964874
Gglean011170	0.192984954	0.299643399	0.156700261	0.347749
Gglean011171	13.6082	13.6954	6.28404	7.22942
Gglean011172	1.225231	0.265768	0.826330792	1.910337
Gglean011173	32.228571	34.14582879	39.809957	38.96481
Gglean011174	0	0	0	0.067194
Gglean011175	0.190997	0.158613	0.298836	0.102775

Gglean011176	0	0	0	0
Gglean011177	5.89263	3.067450136	13.7284	16.1992
Gglean011178	0	0	0	0
Gglean011179	0	0	0	0
Gglean011180	0	0	0	0
Gglean011181	2.92369	3.88569	1.99095	0.94943
Gglean011182	0.159332	0	0	0
Gglean011183	13.1464	14.0782	20.1337	19.524
Gglean011184	92.24279633	93.44413418	76.22424671	65.4980796
Gglean011185	92.24279633	93.44413418	76.22424671	65.4980796
Gglean011186	92.24279633	93.44413418	76.22424671	65.4980796
Gglean011187	0	0	0	0
Gglean011188	46.4019	44.5393	19.09705	14.48403
Gglean011189	29.2075	28.9302	17.68919	13.93142
Gglean011190	73.4162	69.4496	63.1513	57.2828
Gglean011191	213.528	212.879	257.039	254.276
Gglean011192	13.019935	12.56475	15.49011	18.28213
Gglean011193	0.0425067	0.0578298	3.97E-65	0.0391804
Gglean011194	60.09594254	61.29554094	29.98035332	26.37964
Gglean011195	0	0	0	0
Gglean011196	0	0	0	0
Gglean011197	0.452924	0	0.124392	0
Gglean011198	40.89331	38.62297	31.299363	28.97356
Gglean011199	86.5717	101.997	93.9783	69.6069
Gglean011200	129.7264	146.2472	93.2567	78.1003
Gglean011201	67.18404	60.42748	65.0148	66.01668
Gglean011202	24.881	23.91528	13.9633	13.94844
Gglean011203	53.1688937	55.29520057	43.9437856	40.8666845
Gglean011204	11.925404	11.74477	12.469376	11.429336
Gglean011205	11.925404	11.74477	12.469376	11.429336
Gglean011206	0	0	0.43385	0.350321
Gglean011207	0.161016	0	0.132056	0
Gglean011208	1.475592098	2.324047649	2.323280038	3.459288671
Gglean011209	10.26029791	9.326625275	15.60576338	17.08166286
Gglean011210	11.209079	10.43385718	8.8330963	8.845529
Gglean011211	11.209079	10.43385718	8.8330963	8.845529
Gglean011212	41.17384953	44.407037	26.31275	22.941615
Gglean011213	34.616827	27.258531	21.954677	22.012257
Gglean011214	34.616827	27.258531	21.954677	22.012257
Gglean011215	30.297897	27.334994	16.919806	15.80275
Gglean011216	1.532199698	1.650217987	1.238386483	1.190742955
Gglean011217	58.61184289	63.96904704	58.305077	51.40745209
Gglean011218	41.32614657	43.15585	50.29437959	48.94311727
Gglean011219	44.63116717	45.85586424	49.91877164	50.59155656

Gglean011220	34.559741	38.101506	19.304044	18.56890005
Gglean011221	0.407449	0	0	0
Gglean011222	0.16624	0.145988	0.0574419	0.181119
Gglean011223	0	0	0.086447425	0
Gglean011224	9.318240733	9.620347402	13.14506103	15.555944
Gglean011225	9.318240733	9.620347402	13.14506103	15.555944
Gglean011226	4.043669025	3.614317814	2.010171251	1.690822039
Gglean011227	0.705503	0.60316	1.29238	1.40607
Gglean011228	3.4434	2.74152	3.33096	2.47136
Gglean011229	21.38101143	17.05212102	14.511023	19.685763
Gglean011230	25.32034543	25.35886	34.39447	37.65883
Gglean011231	22.9097	28.119	22.0924	20.0356
Gglean011232	13.9596811	14.39525207	12.30554153	11.962085
Gglean011233	80.61875208	79.4679532	73.04955092	76.110444
Gglean011234	80.61875208	79.4679532	73.04955092	76.110444
Gglean011235	22.13163	26.12725	12.03944	9.99267
Gglean011236	13.78434	15.87685	12.27199	12.06908
Gglean011237	930.927	961.398	611.617	582.198
Gglean011238	72.97742	94.00359	97.57837	88.60125
Gglean011239	0	0.123472	0.402484	0
Gglean011240	41.6018954	42.33823919	30.91680011	25.98717553
Gglean011241	41.6018954	42.33823919	30.91680011	25.98717553
Gglean011242	41.6018954	42.33823919	30.91680011	25.98717553
Gglean011243	41.6018954	42.33823919	30.91680011	25.98717553
Gglean011244	3.15608	1.18081	0.855392	0
Gglean011245	21.51984	24.1535	4.85568484	4.73641
Gglean011246	264.2619	210.4446	151.0062	152.9983
Gglean011247	11.37330791	6.731991902	9.92156536	12.53903301
Gglean011248	11.37330791	6.731991902	9.92156536	12.53903301
Gglean011249	35.89807705	34.2891742	32.971077	31.4333942
Gglean011250	0	0	0	0
Gglean011251	0	0	0	0
Gglean011252	0	0	0	0
Gglean011253	0	0	0	0
Gglean011254	70.90527881	74.87273651	116.3053365	111.7245433
Gglean011255	70.90527881	74.87273651	116.3053365	111.7245433
Gglean011256	2.361186	1.701933432	2.264480813	3.321875068
Gglean011257	4.121789	3.334362	3.931967001	3.9708088
Gglean011258	8.83475	9.12180002	15.99747	15.27579
Gglean011259	17.21907	19.00445	13.92138	15.88345
Gglean011260	12.06494653	12.755947	15.9942691	16.88759001
Gglean011261	12.06494653	12.755947	15.9942691	16.88759001
Gglean011262	62.4345	56.9071962	41.728032	39.60475009
Gglean011263	76.8465431	75.00200624	55.1151416	54.368975

Gglean011264	76.8465431	75.00200624	55.1151416	54.368975
Gglean011265	100.65285	107.5188	65.75045	57.75921
Gglean011266	3.937318	5.93365	7.272001	8.85772
Gglean011267	47.35158	49.59785	64.06592	53.21585
Gglean011268	44.88821	45.7363	35.52397	32.51664
Gglean011269	0.524649	2.047735	7.11687	3.74384327
Gglean011270	0.524649	2.047735	7.11687	3.74384327
Gglean011271	29.1241	30.2588	23.4874	22.3673
Gglean011272	74.5071	69.4081	39.04756	36.83014
Gglean011273	74.5071	69.4081	39.04756	36.83014
Gglean011274	18.38656	14.20232319	10.78082397	11.13408623
Gglean011275	15.403379	13.41935791	10.933835	11.04109353
Gglean011276	12.202345	12.215995	18.364617	15.884671
Gglean011277	0.708585831	1.292242	0.183604601	0.136038547
Gglean011278	17.28107601	21.89181371	19.27561575	20.3294535
Gglean011279	0.230825	0.203746	0.242047	0.2869
Gglean011280	8.76538	11.52389	5.62674883	6.970976072
Gglean011281	18.63678	19.4396957	27.57195433	30.09459908
Gglean011282	0.564483	0.355499	0.254031	0.0903669
Gglean011283	14.33228228	12.3696625	21.2797	21.333585
Gglean011284	35.55714	36.55869	46.2915	61.4931
Gglean011285	26.6202	4.28449	9.10604	10.7277
Gglean011286	0	0	0	0.03882
Gglean011287	336.1112	385.19139	274.43768	258.50441
Gglean011288	336.1112	385.19139	274.43768	258.50441
Gglean011289	110.6059	80.5096	110.7452	110.2438
Gglean011290	20.99623	16.66424	12.19229	17.12291
Gglean011291	2.571730184	3.136561	2.680979019	3.05687
Gglean011292	0	0	0	0
Gglean011293	831.865	809.604	1233.67	1517.69
Gglean011294	17.02799	16.75281	26.11078	30.1358
Gglean011295	65.13776	70.94765	99.23107	89.79962
Gglean011296	390.73911	405.08279	335.0597	307.94763
Gglean011297	390.73911	405.08279	335.0597	307.94763
Gglean011298	0	0	0	0
Gglean011299	0.158846	0.0828847	0.129941	0
Gglean011300	3.152594625	4.220468355	4.55153	6.23561
Gglean011301	23.46828	27.273024	36.44017	36.514647
Gglean011302	260.7421	341.4877	121.6801	111.905
Gglean011303	30.6114	28.0757	14.1456	15.5857
Gglean011304	11.21706426	11.31257701	7.560773555	8.200359585
Gglean011305	71.393243	71.47915	53.10143	51.50737
Gglean011306	12.99268	18.38077	5.49872	3.73071
Gglean011307	19.323216	21.68411155	12.35241	11.95281845

Gglean011308	47.63438392	45.609365	43.34022597	46.13764338
Gglean011309	31.53777141	31.90922556	27.59725198	25.12734171
Gglean011310	24.00461	22.14447	16.47693	18.14969
Gglean011311	31.6343	29.8842	31.3505	31.864
Gglean011312	93.3691	96.3914	59.7668	50.1094
Gglean011313	0.157061	0	0.321257	0.152868
Gglean011314	0.000813935	0.150838	0.372835	2.54892
Gglean011315	0.76224	0.000209447	0.297631	0.00057329
Gglean011316	0.571345	0	0.708077	0.563298
Gglean011317	0	0	0	0
Gglean011318	0	0	0	0
Gglean011319	0	0	0	0
Gglean011320	0	0	0	0
Gglean011321	1.00281	2.47557	3.14643	3.74278
Gglean011322	34.58827594	34.94955515	66.171784	65.577938
Gglean011323	34.58827594	34.94955515	66.171784	65.577938
Gglean011324	0.16019901	1.242716642	0.65277388	1.857727732
Gglean011325	0.831259	0.359866	0.596155	0.926855
Gglean011326	0.115189	0.158009	0.0937965	0.148975
Gglean011327	2.080276371	1.5062791	1.375262192	1.084778737
Gglean011328	0.000124505	0.48362454	0.471898245	0.37121325
Gglean011329	2.25E-17	1.93E-07	1.18E-15	2.56E-14
Gglean011330	7.7055	4.90228	3.51328	3.76833
Gglean011331	7.76748	7.00057	6.88218	7.25152
Gglean011332	24.567884	24.46959	24.57845	25.282697
Gglean011333	21.043	17.36386	19.998882	20.6087
Gglean011334	47.1885	39.0231	32.9868	40.4723
Gglean011335	32.624735	27.50084	32.48224019	33.281245
Gglean011336	0.525902443	0.367994302	0.336883853	0.303719943
Gglean011337	0	0	0	0
Gglean011338	26.45234716	27.3344432	29.16651422	27.55917596
Gglean011339	7.41287	9.628691	10.939937	17.016578
Gglean011340	10.25453267	11.403537	14.2875258	18.136157
Gglean011341	1.470008	2.322669	1.997472	2.131303009
Gglean011342	195.9621	197.9168	195.1748	188.1604
Gglean011343	7.180094	9.20938	6.18778118	8.375326
Gglean011344	7.180094	9.20938	6.18778118	8.375326
Gglean011345	0.188243424	0.042092	0.09968309	0.090507119
Gglean011346	1.172727212	0.877168865	1.836873556	2.732567321
Gglean011347	1.172727212	0.877168865	1.836873556	2.732567321
Gglean011348	1.172727212	0.877168865	1.836873556	2.732567321
Gglean011349	0.0319353	0.000179067	0.00290811	1.96E-13
Gglean011350	0.204242347	0.068023952	0.166934149	0.15819127
Gglean011351	0.0502532	0.0518883	0.122928	0.194652

Gglean011352	0.244940757	0.507805196	0.164597462	0.194363521
Gglean011353	60.692358	54.5560096	45.576421	41.5573858
Gglean011354	60.692358	54.5560096	45.576421	41.5573858
Gglean011355	0.00423719	0.00209478	0.000967769	0.00050267
Gglean011356	1.56420266	1.343366384	1.670780229	2.565563014
Gglean011357	128.757268	111.315531	303.25809	319.072814
Gglean011358	69.0084	88.1163	60.1153	40.8408
Gglean011359	3.619673509	3.456301047	5.15959176	4.108649203
Gglean011360	22.95226408	27.00315247	32.70813452	38.14693196
Gglean011361	22.95226408	27.00315247	32.70813452	38.14693196
Gglean011362	22.95226408	27.00315247	32.70813452	38.14693196
Gglean011363	14.1787	13.1312	13.0123	12.4266
Gglean011364	13.659479	15.073854	15.99913942	17.857878
Gglean011365	13.659479	15.073854	15.99913942	17.857878
Gglean011366	28.27910281	31.37988	32.101973	30.50775758
Gglean011367	75.637151	73.8607272	46.23787	41.58524
Gglean011368	591.5435	581.5888	500.0477	436.9258
Gglean011369	32.701138	40.40667844	27.3525586	23.0640548
Gglean011370	8.199113	7.015912	7.2827096	7.139261671
Gglean011371	39.7374	36.42578	26.94158461	24.6153268
Gglean011372	39.7374	36.42578	26.94158461	24.6153268
Gglean011373	10.541542	11.73004042	6.0313674	5.412920133
Gglean011374	63.77248	50.54768	41.98683	36.64336
Gglean011375	11.44915	9.83707	8.22709	7.42194
Gglean011376	141.04915	150.0061854	99.43475708	96.327541
Gglean011377	10.455622	7.70635946	13.09812	15.91294
Gglean011378	21.01259	24.332134	33.19954	34.21521
Gglean011379	9.14196	8.747175391	3.947487	2.259815
Gglean011380	0.03702	0.0769448	0.0602972	0
Gglean011381	0	0	0.0598205	0
Gglean011382	5.25259	3.2347	4.19815	4.1015
Gglean011383	38.9156	29.0844	29.2171	23.112
Gglean011384	0	0	0	0
Gglean011385	28.60311	26.0539	38.36089	33.89753
Gglean011386	0	0	0	0
Gglean011387	11.01685976	9.8922408	12.60622496	12.66959028
Gglean011388	21.3845	21.9784	19.5665	19.3609
Gglean011389	23.9083	24.0048	21.5837	20.6737
Gglean011390	2.6377302	2.3769432	1.8148928	1.2752105
Gglean011391	5.64395424	7.96699862	5.595712	7.393567
Gglean011392	1.60361	2.35275	3.08E-05	0.000220741
Gglean011393	0	0	0	0.355564
Gglean011394	0	0	0	0
Gglean011395	14.42174083	14.50897	10.81655117	10.23543408

Gglean011396	23.7868	24.670527	31.910123	35.48726502
Gglean011397	23.0346	23.0677	17.977	15.7029
Gglean011398	23.0346	23.0677	17.977	15.7029
Gglean011399	0.578762	0	0.478945	0
Gglean011400	20.46017	18.498515	12.298578	10.5818501
Gglean011401	20.46017	18.498515	12.298578	10.5818501
Gglean011402	8.44444	7.15829	5.41656	4.65196
Gglean011403	48.96221	54.18376	43.61978	41.80476
Gglean011404	145.8613	133.9114	147.363	201.2509
Gglean011405	18.02333143	16.58624	20.27356865	18.5490172
Gglean011406	18.02333143	16.58624	20.27356865	18.5490172
Gglean011407	25.46131	25.2222	18.88648	21.30156
Gglean011408	167.340575	165.6035111	141.572281	141.542409
Gglean011409	4.71358	2.58173	1.44995	2.89322
Gglean011410	26.90738	25.55285	22.25302	26.24212
Gglean011411	0.0679685	0.0350934	0.0274691	0
Gglean011412	3.667873343	3.930580452	5.666161	6.99359702
Gglean011413	30.696341	36.454804	8.8138297	12.013283
Gglean011414	39.1077	37.476	27.1427	23.77634
Gglean011415	106.9972	105.4582826	77.0522	62.113012
Gglean011416	106.9972	105.4582826	77.0522	62.113012
Gglean011417	106.9972	105.4582826	77.0522	62.113012
Gglean011418	19.248323	17.31075	18.60195	29.57285
Gglean011419	19.385101	19.298221	17.106954	19.328823
Gglean011420	34.9026814	35.04282306	35.89239194	52.34884
Gglean011421	0	0	0	0.0990628
Gglean011422	1.2049008	1.642795	1.23941	1.5917123
Gglean011423	0	0	0	0
Gglean011424	4.3235554	6.651593	9.599042	10.863933
Gglean011425	1506.95	1504.12	959.204	897.704
Gglean011426	79.8973	84.9703	51.89152	46.28170003
Gglean011427	0	0.09453	0.0370989	0.131487
Gglean011428	20.023322	21.76809	15.337142	13.139671
Gglean011429	20.023322	21.76809	15.337142	13.139671
Gglean011430	0.0514486	0	8.25E-08	0
Gglean011431	52.3683	55.231	25.634	21.1982
Gglean011432	42.0597	41.1015	30.7361	28.43
Gglean011433	24.6413886	27.13958561	19.460761	22.31138908
Gglean011434	24.6413886	27.13958561	19.460761	22.31138908
Gglean011435	3.45722	6.65108	8.78466	4.86594
Gglean011436	265.674	234.365	326.572	326.624
Gglean011437	5.50265	4.47279	5.56908	7.23755
Gglean011438	24.6413886	27.13958561	19.460761	22.31138908
Gglean011439	27.843302	32.07902617	21.71126485	20.04975965

Gglean011440	27.843302	32.07902617	21.71126485	20.04975965
Gglean011441	27.843302	32.07902617	21.71126485	20.04975965
Gglean011442	27.843302	32.07902617	21.71126485	20.04975965
Gglean011443	62.33948743	56.99020018	50.186705	58.14238323
Gglean011444	62.33948743	56.99020018	50.186705	58.14238323
Gglean011445	5.64587	3.5450602	3.7644	5.1517338
Gglean011446	5.64587	3.5450602	3.7644	5.1517338
Gglean011447	4.478086	4.88986	4.547005986	4.133016022
Gglean011448	3.92233	5.99177	7.85251	8.60139
Gglean011449	0	0	0	0
Gglean011450	0.595188	0.937337	1.070577	1.164025
Gglean011451	2.910252194	2.67737028	4.362458565	4.306379214
Gglean011452	2.910252194	2.67737028	4.362458565	4.306379214
Gglean011453	16.319	23.7100433	31.60996	37.32362
Gglean011454	16.319	23.7100433	31.60996	37.32362
Gglean011455	0	0	0	0
Gglean011456	0	0	0	0
Gglean011457	17.8206	26.6149	38.5009	29.6827
Gglean011458	0	0	0.427326	0
Gglean011459	0	0	0	0
Gglean011460	0	0	0	0
Gglean011461	0	0.240969	0	0
Gglean011462	21.07422	20.115306	28.234091	26.561665
Gglean011463	0.066333932	0	0.120416592	0.031952107
Gglean011464	0	0	0	0
Gglean011465	6.31501596	6.799391894	3.825039	6.09409
Gglean011466	13.613258	16.28926	11.89651	9.35772
Gglean011467	0	0	0	0
Gglean011468	3.91728	3.013894	2.981997	3.195936
Gglean011469	40.69585824	43.63180228	9.410509538	6.919680153
Gglean011470	27.277	28.0529	16.981	14.5693
Gglean011471	0.150915627	0.052465988	0.062954338	0.122665984
Gglean011472	58.26952	60.43576	46.7194	49.58483
Gglean011473	58.26952	60.43576	46.7194	49.58483
Gglean011474	19.37498	16.77823	19.46011	17.55344
Gglean011475	14.1315	13.8814	12.5678	11.4971
Gglean011476	1.344295	1.469492	1.568316	0.477850629
Gglean011477	20.4041	18.16237	15.74928	16.88187
Gglean011478	42.7189	48.6716	53.6875	50.7281
Gglean011479	0.867054	0.733607487	0.48475	0.563387
Gglean011480	1.455565	0.542862	0.939988	0.998979
Gglean011481	0	0	0	0
Gglean011482	0	0	0	0.103725
Gglean011483	0	0	0.0590621	0

Gglean011484	0	0	0	0.0584321
Gglean011485	0.168477	0.112546	14.1446	10.1969
Gglean011486	5.87E-31	3.51E-24	0.01868358	1.24E-36
Gglean011487	5.87E-31	3.51E-24	0.01868358	1.24E-36
Gglean011488	0.273708781	0.569245606	0.524446542	0.795575483
Gglean011489	0	0	0	0
Gglean011490	0.0199567	0	0	0
Gglean011491	0.668671	0.699247	0.277566	0.330012
Gglean011492	0	0	0	0
Gglean011493	0.166226	0	0	0
Gglean011494	2.012104434	0.5853784	1.234713231	1.550871
Gglean011495	2.012104434	0.5853784	1.234713231	1.550871
Gglean011496	2.012104434	0.5853784	1.234713231	1.550871
Gglean011497	47.97102	49.77922	52.16597	54.29208
Gglean011498	31.3672	28.6706	30.3944	33.6528
Gglean011499	4.167845543	3.838162026	5.255999647	5.310523167
Gglean011500	25.88042	28.64051	22.39826	22.86116
Gglean011501	20.90276423	23.62742985	18.3534949	19.9357065
Gglean011502	95.1001	102.1463	52.90485	47.9325
Gglean011503	0	0	0	0
Gglean011504	0	0	0	0
Gglean011505	24.92658	31.0987	26.20869	30.28704
Gglean011506	22.15337	22.849337	32.5792	29.08439
Gglean011507	22.15337	22.849337	32.5792	29.08439
Gglean011508	3.36684	13.339575	7.843978	3.466452
Gglean011509	0	0	0.694442	0
Gglean011510	16.2656	19.377	9.74727	8.4029
Gglean011511	60.63495	70.8678	65.2769	82.09497
Gglean011512	80.34802528	84.11543	63.98504055	57.20538013
Gglean011513	6.32120403	5.134119391	13.13844292	14.9221
Gglean011514	21.46549119	21.53649126	18.46883323	16.92500275
Gglean011515	1.19528	1.26782	0.56631	1.34733
Gglean011516	7.923521293	7.6369476	9.819860802	14.13116
Gglean011517	8.325205577	8.922316331	9.246875735	12.917043
Gglean011518	0	0	0	0
Gglean011519	11.54170032	9.753108234	6.168010318	5.868640374
Gglean011520	11.54170032	9.753108234	6.168010318	5.868640374
Gglean011521	0.000131362	0.000198674	0.966777	0.317498
Gglean011522	0	0	0	0
Gglean011523	0	0	0.053885	0
Gglean011524	32.20782685	32.88955413	27.57823443	28.64394985
Gglean011525	18.908384	18.987379	26.375227	31.36923907
Gglean011526	18.908384	18.987379	26.375227	31.36923907
Gglean011527	0.617316	1.026773	0.516851	0.528454

Gglean011528	51.32156238	49.57772578	79.337392	76.08071563
Gglean011529	25.8983	25.0151	35.7542	37.9137
Gglean011530	43.93115	38.97134	36.29372	33.63752
Gglean011531	27.7265	31.1938	26.0606	26.7619
Gglean011532	6.97276	5.42761	4.36813	6.29247
Gglean011533	63.92605204	59.21200278	89.33731351	84.08795126
Gglean011534	63.92605204	59.21200278	89.33731351	84.08795126
Gglean011535	63.92605204	59.21200278	89.33731351	84.08795126
Gglean011536	5.62272	7.42731	8.34895	9.25395
Gglean011537	152.75612	161.26885	81.127313	65.49492628
Gglean011538	152.75612	161.26885	81.127313	65.49492628
Gglean011539	0.4785797	0.1663712	0.163944	0.501686167
Gglean011540	0	0	0	0
Gglean011541	0.572265	0.7379218	0.9902831	0.926556
Gglean011542	0	0	0	0
Gglean011543	0.572265	0.7379218	0.9902831	0.926556
Gglean011544	0	0	0	0
Gglean011545	0	0	0	0
Gglean011546	0.790996	0.688921	0.217151	0.386999
Gglean011547	6.549615	9.671124	9.367926	11.883584
Gglean011548	8.938414	11.279007	15.80797021	18.146953
Gglean011549	0.440465068	0.631861685	0.630904	1.427961474
Gglean011550	0.294730465	0.155435	0.621127	0.143256634
Gglean011551	11.24059	10.34899	8.405579	7.24571
Gglean011552	11.24059	10.34899	8.405579	7.24571
Gglean011553	11.24059	10.34899	8.405579	7.24571
Gglean011554	3.115501	4.323345	7.563049807	8.967131
Gglean011555	0	0	0	0
Gglean011556	10.2187	10.3368	9.52282	9.29477
Gglean011557	27.9419	30.8093	23.618	23.50684
Gglean011558	50.9627	52.2077	12.42193	9.78898
Gglean011559	0.261096	0.191559	0.0303943	0
Gglean011560	0.337934	0.243191	0.232764	0.1415272
Gglean011561	0	0	0	0
Gglean011562	0	0	0	0
Gglean011563	0	0	0	0
Gglean011564	0	0	0.051660739	0
Gglean011565	0	0	0	0
Gglean011566	0	0	0	0
Gglean011567	0	0	0	0
Gglean011568	0	0	0	0
Gglean011569	0	0	0	0
Gglean011570	30.34020001	34.1038	34.2032	33.9223
Gglean011571	0	0	0.0578228	0

Gglean011572	0	0	0	0
Gglean011573	0	0	0	0
Gglean011574	0.414063191	0.6792001	0.332130937	0.45573
Gglean011575	0.743875909	0.641939026	0.391219344	1.028161
Gglean011576	0	0	0	0
Gglean011577	21.8405406	18.8890139	20.92948982	21.065591
Gglean011578	21.8405406	18.8890139	20.92948982	21.065591
Gglean011579	21.8405406	18.8890139	20.92948982	21.065591
Gglean011580	21.8405406	18.8890139	20.92948982	21.065591
Gglean011581	0	0	0	0
Gglean011582	35.85223973	39.95849	33.41695612	31.18001149
Gglean011583	0.195618	0.385329	0.533963	0.3877598
Gglean011584	0.195618	0.385329	0.533963	0.3877598
Gglean011585	0.1725	0.0589061	0.278261	0.109938
Gglean011586	73.777	54.9589	45.9651	93.5605
Gglean011587	23.482653	23.81341066	32.61819465	42.75339
Gglean011588	8.892881101	8.855565	7.865294	7.337347
Gglean011589	4.72815	10.6558	9.44307	13.28899
Gglean011590	18.60735	20.962267	11.0633193	9.45195
Gglean011591	0	0	0	0
Gglean011592	0.883286	0.750908	0.505211	0.448709
Gglean011593	0	0	0	0
Gglean011594	0	0	0	0
Gglean011595	0.885268	1.902551	2.059447	2.64948
Gglean011596	3.14782	2.83416	2.81032	4.64668
Gglean011597	29.07638495	29.89171638	7.930935387	6.777464364
Gglean011598	0	0	0	0
Gglean011599	0.397622	0.429162	0.651737	1.56546
Gglean011600	109.16776	117.83326	122.7087006	141.9161988
Gglean011601	12.71459	13.96528	10.40597	10.65586
Gglean011602	7.028008	8.60018	9.05581	9.37109
Gglean011603	0	1.0156	0.268128	0.31867
Gglean011604	48.6276	53.9015	34.58209	32.9867
Gglean011605	8.00552	9.18531	8.59281	9.00187
Gglean011606	0	0	0	0
Gglean011607	0	0	0	0
Gglean011608	0	0	0	0
Gglean011609	0.345596	0.143734	0.112465	0.200823
Gglean011610	0	0	0	0
Gglean011611	135.3320007	156.998962	111.2929934	101.430033
Gglean011612	127.7626	150.5708	74.0176	69.8408
Gglean011613	127.7626	150.5708	74.0176	69.8408
Gglean011614	13.290843	13.304914	14.407704	13.292392
Gglean011615	31.95031	37.80793	31.89482	34.10068

Gglean011616	8.410011123	8.7177378	15.07374	18.57221334
Gglean011617	1.022098304	1.2097843	0.720647244	1.183889
Gglean011618	0	0.528409	0	0
Gglean011619	1.459444272	1.189938	1.42982746	2.005199
Gglean011620	4.280230617	9.2768	5.660876	8.34855
Gglean011621	4.280230617	9.2768	5.660876	8.34855
Gglean011622	19.548001	17.06543	14.43139	14.28307
Gglean011623	0	0.2734348	0	0
Gglean011624	9.50841637	9.334626	13.70552624	14.892737
Gglean011625	0.95898	0.227535	6.36E-06	0.188479
Gglean011626	0.228791	0	0.12502	0.0743349
Gglean011627	1.442243859	1.571628111	1.2938	1.81777554
Gglean011628	6.58443	0	3.63397	0
Gglean011629	6.75267	7.84181	6.00209	5.33484
Gglean011630	218.1948	182.390695	217.09695	275.5549
Gglean011631	51.58421	53.66328269	29.94679591	25.93203
Gglean011632	13.58991778	38.01012255	33.3847405	42.394091
Gglean011633	6.205907	4.629026367	3.053417458	3.204562454
Gglean011634	64.03472006	67.56982375	74.2398	70.28045746
Gglean011635	2.84273	5.35145	6.37003	5.74722
Gglean011636	5.32311395	5.426127819	3.886037892	3.566838684
Gglean011637	0	13.2683	0	0
Gglean011638	0.164773	0.169816	0.0665548	0.159403
Gglean011639	3.026	3.82632	5.38635	6.04785
Gglean011640	4.80005	5.17076	3.47119	7.13147
Gglean011641	0.0372034	0.000643475	2.19E-06	0.481447
Gglean011642	15.727454	13.43316	15.23968	13.073293
Gglean011643	0	0	0	0
Gglean011644	0.519119	0.363628	0.988201	1.00897
Gglean011645	1.906166918	1.642179506	2.045225068	2.310152603
Gglean011646	1.906166918	1.642179506	2.045225068	2.310152603
Gglean011647	41.7050655	41.73421	51.97268	59.97375
Gglean011648	41.7050655	41.73421	51.97268	59.97375
Gglean011649	0.850555	3.20392	3.00242	3.3365
Gglean011650	39.6149	35.8586	48.213	67.248
Gglean011651	42.78206	42.65824123	45.61670116	43.07428601
Gglean011652	5.94134	6.53093	6.94084	8.22804
Gglean011653	2.2205	2.15258	5.0421	4.44376
Gglean011654	5.86128	9.60823	10.9285	13.8266
Gglean011655	0	0	0.572827	0
Gglean011656	0	0	0	0
Gglean011657	0	0	0	0
Gglean011658	10.5804	12.494	5.4168	3.99636
Gglean011659	20.7744	36.6924	14.1495	26.0345

Gglean011660	12.7201	8.07531	18.7965	36.4595
Gglean011661	0.157889	0	0	0
Gglean011662	0.0444954	0.137091	0.0359106	0.0426154
Gglean011663	87.8938	102.475	47.22198	38.00178
Gglean011664	7.655777	9.424753	3.012343405	2.386651452
Gglean011665	7.655777	9.424753	3.012343405	2.386651452
Gglean011666	35.21987	40.88645	16.915296	13.237271
Gglean011667	58.00301	58.20002	58.84462	61.10896
Gglean011668	180.8054	194.226	243.56358	298.15948
Gglean011669	180.8054	194.226	243.56358	298.15948
Gglean011670	0	0	0	0
Gglean011671	0.039361136	0.137033658	0.213804385	0.063455192
Gglean011672	84.1646154	85.09045478	83.6699091	83.99768525
Gglean011673	84.1646154	85.09045478	83.6699091	83.99768525
Gglean011674	12.89107051	15.25535658	8.267246533	8.3561796
Gglean011675	12.89107051	15.25535658	8.267246533	8.3561796
Gglean011676	12.89107051	15.25535658	8.267246533	8.3561796
Gglean011677	24.9885	17.5061	12.843	9.87778
Gglean011678	0.6550442	0.775516874	0.44610577	0.6990175
Gglean011679	0.169297	0.206395	0.1687996	0.316884
Gglean011680	20.2597461	19.000783	15.9007085	18.86453455
Gglean011681	8.75366	11.8668	19.2048	21.4889
Gglean011682	2.689367682	2.0888466	2.129121079	2.9820657
Gglean011683	0.0301048	0.156784	0.0243976	0.0288363
Gglean011684	6.60719391	8.039729	11.47921004	12.71754082
Gglean011685	4.906967	4.794241	5.1426	7.83598
Gglean011686	0.386394212	0.287744872	0.541572027	0.588851355
Gglean011687	8.415909011	7.24179044	11.39685703	12.92475366
Gglean011688	5.51294	0.878038	3.26919	0.785369
Gglean011689	3.96568	1.41411	3.06921	3.0968
Gglean011690	2.60716	3.311	0.913441	1.42158
Gglean011691	0.0359669	0.0370923	0.203992	0.172433
Gglean011692	28.8342	22.8884	16.5674	16.3805
Gglean011693	16.936082	16.56545	10.378668	9.422156
Gglean011694	16.936082	16.56545	10.378668	9.422156
Gglean011695	77.9535	63.2793	73.2	68.6693
Gglean011696	2.562611	3.193170754	1.9419288	1.065516408
Gglean011697	2.562611	3.193170754	1.9419288	1.065516408
Gglean011698	4.745766	5.786941975	3.439218	3.803271148
Gglean011699	2.74867	0.841056	1.265884411	1.893979
Gglean011700	0.046775187	0	0.037103599	0.044191919
Gglean011701	0.046775187	0	0.037103599	0.044191919
Gglean011702	0.020827728	0.0543346	0	0.020175711
Gglean011703	0	0	0	0.092352814

Gglean011704	68.3293	76.44232	41.15269	33.39779
Gglean011705	24.95522	27.8535	7.09906	5.69255
Gglean011706	24.95522	27.8535	7.09906	5.69255
Gglean011707	36.4632	35.8187	46.6992	43.1032
Gglean011708	21.56231185	22.26947	28.115251	41.322939
Gglean011709	0.1301301	0	0.137278724	0.070161665
Gglean011710	11.537085	12.6642127	7.975873	7.33618
Gglean011711	12.4273	11.2196	9.2751	7.87402
Gglean011712	14.31620402	13.55667913	20.43461426	23.688683
Gglean011713	14.31620402	13.55667913	20.43461426	23.688683
Gglean011714	24.7656	11.265	21.5917	28.3936
Gglean011715	2.373703	2.80593608	1.173951655	1.6808404
Gglean011716	2.373703	2.80593608	1.173951655	1.6808404
Gglean011717	32.1886	33.08787211	25.99718	22.037663
Gglean011718	0	0	0	0
Gglean011719	1.75986	1.27752	1.22390184	2.33919
Gglean011720	2.115310966	1.717569577	3.571181501	3.860626631
Gglean011721	0.0751114	0.0813227	0.2425331	0.283619
Gglean011722	26.26281656	27.97676892	22.84236036	24.0611229
Gglean011723	0	0	0	0
Gglean011724	0.101654	0	0	0.0989753
Gglean011725	0	0	0	0
Gglean011726	0	0	0	0
Gglean011727	0	0	0	0
Gglean011728	0	0.158342	0	0
Gglean011729	0.307079	0.315989	0.249563	0.49533
Gglean011730	0	0	0	0
Gglean011731	0	0	0	0
Gglean011732	76.34940001	91.69044272	71.19230917	61.68530187
Gglean011733	31.18479618	51.6793	11.261189	6.784181
Gglean011734	31.18479618	51.6793	11.261189	6.784181
Gglean011735	90.55671	89.3127	20.16842	14.07373158
Gglean011736	90.55671	89.3127	20.16842	14.07373158
Gglean011737	0.048542	0	0	0
Gglean011738	0	0	0	0
Gglean011739	0.10134	0	0.0550039	0.0325079
Gglean011740	28.15361742	30.14775006	45.180882	47.19471633
Gglean011741	37.96016019	43.23823	23.58562	22.29104
Gglean011742	0	0	0	0
Gglean011743	0	0	0	0
Gglean011744	78.28373082	75.7784939	85.26686804	88.31986364
Gglean011745	2.640541	3.87257	3.23666	4.073161
Gglean011746	0.326607	0	0.267184	0.158805
Gglean011747	0.830627	1.016988	1.612281	2.090643

Gglean011748	0	0	0	0
Gglean011749	15.065	15.57605	6.67837	5.98936
Gglean011750	21.386926	21.132595	14.862793	15.551585
Gglean011751	73.79654	84.07221	25.89743	20.98736
Gglean011752	26.873254	31.7228722	24.28780815	23.09750693
Gglean011753	0	1.46415	0	0
Gglean011754	73.0605	80.648	60.7526	57.5092
Gglean011755	52.669913	53.875299	60.846238	69.528878
Gglean011756	241.96969	279.550189	304.60914	339.422
Gglean011757	241.96969	279.550189	304.60914	339.422
Gglean011758	22.2456	27.64557	12.75662	9.79983
Gglean011759	0	0.241838	0	0
Gglean011760	0	0	0	0
Gglean011761	47.83716	52.37172	39.01986	38.60294
Gglean011762	6.928162261	6.903227029	10.93902002	13.92404621
Gglean011763	6.928162261	6.903227029	10.93902002	13.92404621
Gglean011764	28.48579226	26.36299893	22.96855962	20.00641151
Gglean011765	147.48983	145.19922	146.70031	161.09751
Gglean011766	147.48983	145.19922	146.70031	161.09751
Gglean011767	0	0	0	0
Gglean011768	21.47278534	20.354972	22.8667584	24.09692005
Gglean011769	15.69737076	15.2842	25.11038123	27.98985002
Gglean011770	0	0	0	0
Gglean011771	9.3115347	9.723108229	8.591082607	7.350843486
Gglean011772	9.3115347	9.723108229	8.591082607	7.350843486
Gglean011773	2.03E-11	8.81E-25	2.15E-54	2.22E-08
Gglean011774	0	0	0	0
Gglean011775	4.3933	2.9634	2.839082	2.031061
Gglean011776	0	0	0.050007125	0
Gglean011777	22.109459	21.89873937	38.79878202	47.77639176
Gglean011778	44.549	35.02383	36.13624	40.69244
Gglean011779	0.643079	0.305221	0.949519616	0.873598
Gglean011780	19.63820839	19.08039292	18.9624952	19.0705906
Gglean011781	19.63820839	19.08039292	18.9624952	19.0705906
Gglean011782	19.63820839	19.08039292	18.9624952	19.0705906
Gglean011783	2.71E-06	1.36E-05	0.0554114	0.0219232
Gglean011784	1.05975	1.44859	0	0.0810811
Gglean011785	102.09	109.239	101.721	110.106
Gglean011786	25.7474	28.4478	32.7652	33.9711
Gglean011787	19.37072	18.02268	15.09317	15.45573
Gglean011788	143.868	154.729	226.834	250.037
Gglean011789	22.1188	27.6322	14.4903	16.0664
Gglean011790	113.0695	116.3461	76.9119	71.9301
Gglean011791	5.71845	6.27295	3.9406	3.5054

Gglean011792	3.630208712	4.262303114	3.553819	3.7802004
Gglean011793	3.630208712	4.262303114	3.553819	3.7802004
Gglean011794	11.875139	14.48342035	24.871615	27.45790527
Gglean011795	2.491272	1.729600322	3.227610814	4.198448092
Gglean011796	0	0	0	0
Gglean011797	0.124748	0.0258389	0.020234	0
Gglean011798	8.32413	6.02192	10.84614	13.1421
Gglean011799	483.7717115	480.2970018	649.4454422	841.25551
Gglean011800	483.7717115	480.2970018	649.4454422	841.25551
Gglean011801	160.578037	162.992631	34.97532738	30.61402434
Gglean011802	3.69424	2.02251	3.29389	2.1688
Gglean011803	3.69424	2.02251	3.29389	2.1688
Gglean011804	0.409973125	0.212130096	0.415322613	0
Gglean011805	0	0	0	0
Gglean011806	0	0.100803981	0	0.031772987
Gglean011807	4.606691162	5.409123648	5.937625	6.982813155
Gglean011808	4.606691162	5.409123648	5.937625	6.982813155
Gglean011809	0	0	0	0
Gglean011810	18.644271	18.970777	30.908477	32.261128
Gglean011811	3.707927562	4.730054712	4.207517704	5.148515043
Gglean011812	0.451386735	0.561241003	0.380915168	0.324014193
Gglean011813	0.347156	0	0	0
Gglean011814	132.30195	111.30384	160.23119	245.08727
Gglean011815	5.35279	4.4887	5.33094	5.90482
Gglean011816	84.13897	84.83698	52.83597	41.617485
Gglean011817	70.97668327	80.13353252	52.62363603	47.08443184
Gglean011818	0.0833237	0.0432325	0	0
Gglean011819	0	0	0	0
Gglean011820	0	0	0.105586	0
Gglean011821	0	0	0.00944141	0
Gglean011822	0	0	0	0
Gglean011823	0	0.362913	0.276733	0.662379
Gglean011824	0	0	0	0
Gglean011825	0	0	0	0
Gglean011826	0	0	0	0
Gglean011827	0	0.327135	0	0
Gglean011828	0	0.262034	0	0
Gglean011829	0	0	0	0
Gglean011830	0	0	0	0
Gglean011831	0	0	0	0
Gglean011832	0	0	0.01620552	0
Gglean011833	44.62523973	45.41755026	33.913154	27.374257
Gglean011834	0.0739363	0.229283	0.240025	0
Gglean011835	0	0	0.219645	0

Gglean011836	12.116996	14.37608164	17.48791599	21.719984
Gglean011837	4.583151247	6.989731079	2.041200699	2.833040987
Gglean011838	0	1.488856	0.695731497	3.336898147
Gglean011839	0.124883	0.0432164	0.101592	0.401256
Gglean011840	0	0	0	0
Gglean011841	0	0	0	0
Gglean011842	0	0	0	0
Gglean011843	0	0	0	0.592793
Gglean011844	0	0	0	0
Gglean011845	0	0	0	0
Gglean011846	0	0	0	0
Gglean011847	0.0533344	0.0540814	0	0.0770134
Gglean011848	0	0	0	0
Gglean011849	0	0	0	0
Gglean011850	0	0	0	0
Gglean011851	0	0	0	0
Gglean011852	0	0	0	0
Gglean011853	0	0	0	0
Gglean011854	2.91E-06	0	0.238238	0.180013
Gglean011855	23.514097	23.835177	20.770788	19.385167
Gglean011856	19.8229	19.3927	47.3791	53.3204
Gglean011857	12.41503781	9.79182529	6.268975001	6.257675396
Gglean011858	5.21016	5.76273	4.61996	3.76588
Gglean011859	56.40303303	57.345506	50.368159	47.18186054
Gglean011860	0.0410818	0.042775	0.0998927	0
Gglean011861	0	0	0.0205701	0.0972549
Gglean011862	118.3518379	131.7833181	124.4637348	133.7948057
Gglean011863	76.676714	81.15420406	68.71526195	63.13645063
Gglean011864	0	11.0449	0	0
Gglean011865	43.9413	56.9838	91.973	96.66
Gglean011866	0	0	0	0
Gglean011867	10.3139	10.5499	6.52262	5.21127
Gglean011868	0	0	0	0
Gglean011869	0	0	0	0
Gglean011870	0	0	0	0
Gglean011871	0	0	0	0
Gglean011872	15.27954006	17.04864173	23.99450101	25.8165427
Gglean011873	0	0	0	0
Gglean011874	84.57617408	91.43893959	83.34087	77.17429517
Gglean011875	84.57617408	91.43893959	83.34087	77.17429517
Gglean011876	84.57617408	91.43893959	83.34087	77.17429517
Gglean011877	4.27684	5.6481	4.27037	4.15221
Gglean011878	13.521229	10.86683002	21.62924022	12.0750004
Gglean011879	3.29288	2.368378	2.06594	2.210032

Gglean011880	0.132613	0.0682808	0.05347	0.126262
Gglean011881	0	0	0	0
Gglean011882	41.93892449	35.56803706	63.93253391	72.32582792
Gglean011883	0	0	0	0.745277
Gglean011884	0	0	0.0633867	0
Gglean011885	0	0	0	0
Gglean011886	0	0	0	0
Gglean011887	0.538178	0.6549861	0.214342	2.23535
Gglean011888	0	0.0924847	0.0725917	0
Gglean011889	0	0	0	0
Gglean011890	0.115179	0.119507	0	0.110532
Gglean011891	0	0	0	0
Gglean011892	0	0	0	0
Gglean011893	0	0	0	0
Gglean011894	0	0	0	0
Gglean011895	2.47708	0.896343	1.50468	1.56059
Gglean011896	0	0	0	0
Gglean011897	0	0	0	0
Gglean011898	0	0	0	0
Gglean011899	0	0	0	0
Gglean011900	0.1996469	0.34697	0.33240198	0.2760174
Gglean011901	3.291274	5.186105	4.289015	7.235933
Gglean011902	3.291274	5.186105	4.289015	7.235933
Gglean011903	3.291274	5.186105	4.289015	7.235933
Gglean011904	1.2857354	0.872028668	1.74749091	1.791960802
Gglean011905	3.473992	4.005497513	4.474796501	5.721920142
Gglean011906	0	0	0	0
Gglean011907	0.533042	1.39852	0.897847	1.10124
Gglean011908	0.159952	0.27242	0.0859033	0
Gglean011909	0.797496	0.451087692	0.899122	1.449994
Gglean011910	0	0	0	0
Gglean011911	25.3516	18.9733	14.7332	17.6464
Gglean011912	0	0	0	0
Gglean011913	0	0	0	0
Gglean011914	0	0	0	0
Gglean011915	0	0.133153	0	0
Gglean011916	0	0	0	0
Gglean011917	0	0	0	0
Gglean011918	0	0	0	0
Gglean011919	0	0	0	0
Gglean011920	1.51575	3.12486	1.14525	1.04868
Gglean011921	0	0	0	0
Gglean011922	0	0	0	0
Gglean011923	0	0	0.0541585	0

Gglean011924	0	0	0	0
Gglean011925	0	0	0	0
Gglean011926	0	0	0	0
Gglean011927	0	0	0	0
Gglean011928	0.128923	0	0	0.125622
Gglean011929	0	0	0	0
Gglean011930	0	0	0	0
Gglean011931	0	0	0	0
Gglean011932	0	0	0	0
Gglean011933	0	0	0	0
Gglean011934	0	0	0	0
Gglean011935	42.49848	39.57523	29.32339	29.02836
Gglean011936	17.2397	15.2817	18.516	25.3672
Gglean011937	16.90634616	18.175972	34.0360249	33.50602056
Gglean011938	0	0	0	0
Gglean011939	0	0	0.0530702	0
Gglean011940	3.4911733	4.073703	4.2816295	5.345486
Gglean011941	0	0	0	0
Gglean011942	0	0	0	0
Gglean011943	0.80377	0.777546	0.250914	0.059545
Gglean011944	7.27631	7.51724	2.8306	1.68193
Gglean011945	0.59455	0.699542	0.160737	0.0759566
Gglean011946	0	0.0407601	0.0635613	0
Gglean011947	0	0	0	0.0521265
Gglean011948	0.508366	0.566247	0	0
Gglean011949	0	0	0	0
Gglean011950	19.9835462	19.8488062	21.50662385	30.08695494
Gglean011951	7.79656	6.44693	2.31839	3.46689
Gglean011952	0	0	0	0
Gglean011953	57.20228	58.863689	36.01965433	31.32944
Gglean011954	14.292	14.8113	9.84982	11.189
Gglean011955	1.515543	1.563838543	2.836297088	3.107474
Gglean011956	60.72668791	61.895312	58.7562852	60.9122574
Gglean011957	19.47017201	21.88490413	22.90150002	23.13316036
Gglean011958	46.4774	44.54971	46.6817	44.6354
Gglean011959	43.5182	35.8217	31.9146	37.1068
Gglean011960	11.29911	8.857013	8.370073	9.76175
Gglean011961	1.35422408	1.352062	1.621499947	1.043988409
Gglean011962	1.35422408	1.352062	1.621499947	1.043988409
Gglean011963	6.89375	5.938657	9.71399	11.09334
Gglean011964	25.7952	28.2167	35.1815	36.9305
Gglean011965	14.33861947	14.42729766	11.0534807	11.89038138
Gglean011966	1830.4401	1683.8572	1693.5972	1625.7995
Gglean011967	13.11806	11.90857	7.12457	7.2042

Gglean011968	5.09291	6.11474	5.68087	9.43478
Gglean011969	1598.550076	1771.220106	1032.640085	982.4810714
Gglean011970	1.05772	0.505460251	0.373795454	0.573199
Gglean011971	67.731082	68.5377	60.428753	57.66272
Gglean011972	24.11022232	26.32294	28.0843	27.0051055
Gglean011973	0	0	0.111625	0.0660933
Gglean011974	18.2275	15.6847	11.0613	11.1469
Gglean011975	1.38876	1.71273	1.99328	0.252239
Gglean011976	35.11971	39.12157	35.55375	38.34399
Gglean011977	49.99075	52.99543	32.72959	30.31349
Gglean011978	49.99075	52.99543	32.72959	30.31349
Gglean011979	50.0947	66.5073	38.30726	51.3665
Gglean011980	45.6969	52.0782	44.7349	42.9432
Gglean011981	0	0	0.0674698	0
Gglean011982	0.081894992	0	0.0383938	0
Gglean011983	11.463276	23.91405	45.6038	42.53239
Gglean011984	0	0	0	0
Gglean011985	3.72462	2.89856	3.56849	4.45472
Gglean011986	43.50558185	45.92828027	39.61971283	44.37084136
Gglean011987	16.28408	15.48746	27.01908	28.9537
Gglean011988	0	0	0	0
Gglean011989	0	0	0.0269262	0
Gglean011990	1.20344	1.73236	0.937283	1.03573
Gglean011991	0.0442388	0	0	0
Gglean011992	0.125388	0	0.101949	0.241956
Gglean011993	0.143321469	0.25252795	0.180521645	0.157601917
Gglean011994	5.34937	17.8602	13.346	18.2756
Gglean011995	0.464903	1.32	0	0
Gglean011996	0	0	0	0
Gglean011997	41.18711	39.06688	42.79217	43.4304
Gglean011998	24.7696565	25.24268989	30.53753036	27.84104758
Gglean011999	24.7696565	25.24268989	30.53753036	27.84104758
Gglean012000	0	0	0	0
Gglean012001	6.05065	3.75859	3.62376	7.80173
Gglean012002	0.895587	0.566107	0.587096	1.04987
Gglean012003	0.56134	0.707576	0	0.216490274
Gglean012004	0.56134	0.707576	0	0.216490274
Gglean012005	78.23059	73.135484	88.185128	86.51944
Gglean012006	78.23059	73.135484	88.185128	86.51944
Gglean012007	78.23059	73.135484	88.185128	86.51944
Gglean012008	0	0.0991574	0	0
Gglean012009	0.102701547	0	0.1771636	1.3784657
Gglean012010	0.102701547	0	0.1771636	1.3784657
Gglean012011	46.384895	48.43297	45.713736	73.321988

Gglean012012	0	0	0	0
Gglean012013	0	0	0	0
Gglean012014	0	0	0	0.343858
Gglean012015	0	0.0939765	0	0
Gglean012016	127.761	94.4842	58.8452	151.476
Gglean012017	45.80715501	43.79616	20.799387	17.921533
Gglean012018	2.62906	2.99721	2.05725	1.87359
Gglean012019	2.08E-05	4.45E-22	7.34E-21	6.18E-17
Gglean012020	0.234534	0.161268	0	0
Gglean012021	0.00391754	1.27985	1.26615	0.277039
Gglean012022	74.80443243	73.50757078	76.402201	73.06708984
Gglean012023	152.9098	161.4956	120.6894	109.5047
Gglean012024	47.118373	50.57224528	39.52300487	36.22252615
Gglean012025	0	0	0	0
Gglean012026	54.28397982	53.74209124	84.9486118	95.71154612
Gglean012027	54.28397982	53.74209124	84.9486118	95.71154612
Gglean012028	54.28397982	53.74209124	84.9486118	95.71154612
Gglean012029	37.36106288	37.09349863	35.06878632	35.24554594
Gglean012030	0	0	0	0.032600169
Gglean012031	0	0	0	0
Gglean012032	0.0371946	0	0	0
Gglean012033	48.0453	48.4254	43.0524	49.1638
Gglean012034	73.7784	71.6974	23.63432	20.46691
Gglean012035	9.18945	8.65465	4.26745	5.00662
Gglean012036	31.7767	33.3463	48.9915	50.4076
Gglean012037	29.78068	35.08395	19.596798	14.43995206
Gglean012038	9.63653	10.75409	16.71311	17.20221
Gglean012039	8.125867	9.19121	8.32326	8.14043
Gglean012040	25.16293714	27.21826218	31.66976864	38.39858396
Gglean012041	25.16293714	27.21826218	31.66976864	38.39858396
Gglean012042	4.64422	3.37943	1.99466	1.07491
Gglean012043	11.78848196	11.7851455	11.42425	12.12876
Gglean012044	0	0	0	0
Gglean012045	0.457542	0.24576	0.190186	0
Gglean012046	0.272320508	0.376919927	0.805212389	1.2604246
Gglean012047	0	0	0	0
Gglean012048	0.272320508	0.376919927	0.805212389	1.2604246
Gglean012049	0	0	0	0
Gglean012050	12.6135394	12.71317484	17.1632891	15.70282125
Gglean012051	6.62566	6.06503	11.9946	6.50475
Gglean012052	0.202371203	0.136159202	0.192932658	0.327653068
Gglean012053	21.85760373	24.25969702	23.70083574	21.65032676
Gglean012054	21.85760373	24.25969702	23.70083574	21.65032676
Gglean012055	0	0	0	0

Gglean012056	0	0	0	0
Gglean012057	0	0	0	0
Gglean012058	0.0801526	0	0.13067	0.541341
Gglean012059	16.90634616	18.175972	34.0360249	33.50602056
Gglean012060	32.34027082	33.09873506	33.61438201	30.83425
Gglean012061	43.34116368	47.48484446	39.50040336	32.38537368
Gglean012062	4.25733	5.77634	5.8673	10.1672
Gglean012063	24.66978	21.35902	20.04024	12.38279
Gglean012064	0.171975	0.309346	0.104663	0.0415053
Gglean012065	8.45207	6.6666	5.82625	6.64412
Gglean012066	0.248726	0	0	0
Gglean012067	0	0.0991247	0	0
Gglean012068	0	0	0	0
Gglean012069	0	0	0.116103	0
Gglean012070	0.254094465	0.074041688	0	0.0485726
Gglean012071	0	0	0.0874741	0.0517604
Gglean012072	48.9782	57.1908	44.2931	48.4967
Gglean012073	55.2913	80.9029	57.583	58.3427
Gglean012074	45.7309	50.6568	43.5948	37.8736
Gglean012075	0	0	0	0
Gglean012076	0	0	0	0
Gglean012077	165.734295	166.4993449	106.2396053	86.46483942
Gglean012078	18.94739647	19.35117383	19.29011298	19.6126131
Gglean012079	2.50332	2.728454	4.25953	2.244037
Gglean012080	23.18332	11.5024052	26.0977	29.86038
Gglean012081	15.900503	18.240532	24.697016	29.909886
Gglean012082	4.24488	0.000609504	7.64749	9.54768
Gglean012083	5.0548406	6.00178	2.529427	3.031717
Gglean012084	29.0622	28.4001	27.9657	22.4037
Gglean012085	90.4693	90.7218	72.9773	71.1394
Gglean012086	0	0	0.0492494	0.0292306
Gglean012087	28.48238525	32.08246552	8.824291668	6.507426696
Gglean012088	73.1095	72.894	60.52604	64.1243
Gglean012089	295.962	325.1	227.932	242.562
Gglean012090	8.97809	7.40315	8.78369	16.50384
Gglean012091	8.97809	7.40315	8.78369	16.50384
Gglean012092	23.38229	24.85865	31.52267	41.59794
Gglean012093	1.976019277	3.220573594	3.554156894	4.385057825
Gglean012094	19.24529	12.034775	14.4280262	16.0401
Gglean012095	0	0	0	0
Gglean012096	0.230659	0.119797	0.0941359	0
Gglean012097	0.156342	0.323149	0	0
Gglean012098	6.12E-06	0.143976	0.229725	0.651552
Gglean012099	1.77123	0.829787	0.540098	0.778606

Gglean012100	3.5305738	2.782986	1.757807	2.152357172
Gglean012101	0	0.0204106	0	0
Gglean012102	0	0.0133289	0.0105715	0
Gglean012103	13.71313587	11.97043865	13.23550862	13.20076635
Gglean012104	8.754409513	9.292809209	15.6544162	16.90573374
Gglean012105	17.558952	15.1241438	18.4043956	21.84116
Gglean012106	4.28923	4.91263	0.643394	0.0634179
Gglean012107	0.07884943	0	0.064004963	0
Gglean012108	50.69800014	52.39620056	29.9539	23.41790005
Gglean012109	50.69800014	52.39620056	29.9539	23.41790005
Gglean012110	50.69800014	52.39620056	29.9539	23.41790005
Gglean012111	13.4737	12.499	8.32823	7.61309
Gglean012112	0	0	0	0
Gglean012113	0	0	0	0
Gglean012114	5.11961	4.99663	5.97939	8.06687
Gglean012115	1.780233	1.406368	1.628753	3.16873
Gglean012116	0	0.170310042	0.133660154	0.105460495
Gglean012117	1.63751	2.61808	3.12243	2.39941
Gglean012118	17.47296982	18.19406607	27.56110043	30.21032425
Gglean012119	0	0	0	0
Gglean012120	0	0	0	0
Gglean012121	0	0	0	0
Gglean012122	0.174015	0.133055	0.212438	0.210878
Gglean012123	0	0	0	0
Gglean012124	3.386465	2.643566626	2.348629	1.750621
Gglean012125	3.19137	3.17959	1.912773	2.089875
Gglean012126	0.0799676	0.0406703	0	0
Gglean012127	0	0.0399138	0.0105579	0
Gglean012128	10.960784	13.0312759	17.443462	21.73569
Gglean012129	1.33451408	0.8754916	1.246250478	1.634265
Gglean012130	0	0	0.421307	0
Gglean012131	64.59027145	71.85647563	58.02186314	88.6092735
Gglean012132	11.2228688	13.123871	10.80271301	12.661859
Gglean012133	19.7773	20.3371	29.5094	38.4845
Gglean012134	6.133183538	7.286270491	9.279404768	7.79166153
Gglean012135	4.74811	4.23307	0.921789	1.9955
Gglean012136	0	0	0	0
Gglean012137	8.187320713	9.577279853	7.195223441	7.665830805
Gglean012138	11.11531	10.7277984	3.992770695	2.80975
Gglean012139	14.08704	11.16024	8.86828	7.79205
Gglean012140	42.849973	41.436839	26.60738	26.951677
Gglean012141	42.849973	41.436839	26.60738	26.951677
Gglean012142	3.077221602	4.262214187	4.841352	6.266942001
Gglean012143	97.4414	99.5764	16.4227	14.1058

Gglean012144	49.98894606	48.78432295	57.176113	58.48073418
Gglean012145	49.98894606	48.78432295	57.176113	58.48073418
Gglean012146	49.98894606	48.78432295	57.176113	58.48073418
Gglean012147	54.960383	64.443255	81.04335438	81.6077144
Gglean012148	54.960383	64.443255	81.04335438	81.6077144
Gglean012149	43.83845	51.29589	55.67318	55.818235
Gglean012150	43.83845	51.29589	55.67318	55.818235
Gglean012151	13.1085	9.79106	8.34102	8.6193
Gglean012152	7.5936	7.43701	6.86204	5.92346
Gglean012153	0	0	0	0
Gglean012154	0.0453594	0	0	0.0438357
Gglean012155	0.0453594	0	0	0.0438357
Gglean012156	0	0.325195	0	0
Gglean012157	0	0	0	0.047275534
Gglean012158	0	0	0	0.047275534
Gglean012159	0	0	0	0
Gglean012160	0	0	0	0
Gglean012161	0	0	0	0
Gglean012162	0	0	0	0
Gglean012163	45.99932072	44.17259	58.40997	67.57318
Gglean012164	1.141664882	0.980372667	1.045535409	1.099936515
Gglean012165	1.91764902	2.063574384	2.787504653	3.700623981
Gglean012166	1.91764902	2.063574384	2.787504653	3.700623981
Gglean012167	0.440303	0.130200063	0.1625647	0.451601
Gglean012168	0.440303	0.130200063	0.1625647	0.451601
Gglean012169	65.7125	66.7939	85.9266	89.2552
Gglean012170	17.35382	15.735689	12.669034	12.242613
Gglean012171	11.0493164	14.023577	23.9265	22.29241029
Gglean012172	18.50366209	23.8216696	17.47712697	20.241425
Gglean012173	0.429914226	0.331341791	0.470448501	0.232385296
Gglean012174	0	2.00E-115	1.70E-12	0
Gglean012175	29.29137	29.486741	20.460838	20.15917
Gglean012176	29.29137	29.486741	20.460838	20.15917
Gglean012177	152.7100542	169.855559	172.8398213	176.160142
Gglean012178	152.7100542	169.855559	172.8398213	176.160142
Gglean012179	0.668817	0	0.838576	2.34242
Gglean012180	1.694551981	1.387549403	1.10947132	1.228480504
Gglean012181	1.05985	1.3059	1.11133	1.22301
Gglean012182	0.908651	0.730873	5.77E-07	0.630556
Gglean012183	0	0	0	0
Gglean012184	0	0	0	0
Gglean012185	0	0	0	0
Gglean012186	0	0	0	0
Gglean012187	19.55426	17.75414388	31.24552	26.81221001

Gglean012188	19.55426	17.75414388	31.24552	26.81221001
Gglean012189	22.8305	25.1653	28.4136	35.5992
Gglean012190	91.88807819	93.66219698	76.00969645	73.72500993
Gglean012191	91.88807819	93.66219698	76.00969645	73.72500993
Gglean012192	34.55081818	35.55029084	45.48683447	45.90526947
Gglean012193	20.59458855	23.5293297	17.7361186	17.14562511
Gglean012194	0	0	0	0.159543
Gglean012195	0	0	0	0
Gglean012196	0.544396	0.143392	0	0
Gglean012197	0.230476	0.119964	0.189382	0.22484
Gglean012198	19.60890105	21.71517667	12.7229122	16.25406744
Gglean012199	2.13763	2.58821	2.49269	1.59402
Gglean012200	3.7851	3.67514	3.65183	3.36742
Gglean012201	0	0	0	0
Gglean012202	134.8971	149.3997	21.2793	17.94309
Gglean012203	48.87974	56.907719	25.87860911	27.87756
Gglean012204	0.0425328	0.1777731	0.069321974	0.0818861
Gglean012205	20.47585193	19.44345709	29.14390241	31.48327637
Gglean012206	939.363	1001.852	633.973	591.649
Gglean012207	3.32137	1.67652	5.2237	5.64139
Gglean012208	36.75516465	43.8627576	63.66938	71.629629
Gglean012209	36.75516465	43.8627576	63.66938	71.629629
Gglean012210	8.42737	10.7643	1.82E-104	0.0160018
Gglean012211	55.43774411	61.36149642	67.465093	58.66982736
Gglean012212	0.271278	0.393685	0.0882999	0
Gglean012213	32.8745	35.72847	26.47502	18.953799
Gglean012214	45.7942001	43.5998414	21.278859	18.329673
Gglean012215	45.7942001	43.5998414	21.278859	18.329673
Gglean012216	45.7942001	43.5998414	21.278859	18.329673
Gglean012217	46.9005	45.3867	49.4993	50.7734
Gglean012218	367.39372	350.69069	346.22874	488.01333
Gglean012219	12.39226692	10.77984236	26.73680916	27.31290411
Gglean012220	137.27	141.293	189.558	226.297
Gglean012221	50.7834	49.1419	37.8943	37.3246
Gglean012222	20.346	21.3009	18.798	17.7737
Gglean012223	41.76934	47.48392	51.2117	56.24563
Gglean012224	16.6825	21.4796	30.1055	31.9771
Gglean012225	0.0366001	0.10274268	0.0295363	0
Gglean012226	0.0366001	0.10274268	0.0295363	0
Gglean012227	0	0	0	0
Gglean012228	0	0	0	0
Gglean012229	8.676423996	12.67194561	8.722835436	12.00381816
Gglean012230	8.676423996	12.67194561	8.722835436	12.00381816
Gglean012231	0.175281	0	0	0

Gglean012232	0.0761092	0.156693	0.123194	0
Gglean012233	65.08344	70.75042	81.84742	94.50961
Gglean012234	48.8923	62.6487	12.4576	11.2636
Gglean012235	7.48061	7.74351	10.1687	14.9495
Gglean012236	30.31434653	32.62036	16.973208	13.27109324
Gglean012237	30.31434653	32.62036	16.973208	13.27109324
Gglean012238	4.843615	3.990370223	5.14677	4.57677
Gglean012239	11.026803	12.145488	6.99617	7.76747
Gglean012240	2.128763045	2.23929335	2.332110655	3.826265
Gglean012241	0.393664397	0.291751652	0.504895466	1.092600218
Gglean012242	2.94966	1.85527	0.459136	1.8805
Gglean012243	0	0	0	0
Gglean012244	15.4344	19.7994	13.9979	14.9285
Gglean012245	21.212987	20.49314007	16.62299642	15.76103128
Gglean012246	1.621678349	2.216884553	3.9938181	4.47262133
Gglean012247	0.414801	0.949325	0.270407	0.959722
Gglean012248	0.0864358	0	0.0700417	0.0826882
Gglean012249	8.491679992	7.590891734	13.59173805	13.02910902
Gglean012250	0.149093	0.102716	0.0806346	0.570913
Gglean012251	0.206883	0.16043	0.126095	0.149007
Gglean012252	3.016750245	3.488640155	2.665970489	2.268870193
Gglean012253	55.0613	55.8162	74.1958	106.719
Gglean012254	1036.56	894.825	1144.95	1486.93
Gglean012255	0	0	0	0
Gglean012256	7.14305	6.94892	6.40073	14.9597
Gglean012257	21.6826	18.8825	20.0605	21.8304
Gglean012258	0	0	0	0
Gglean012259	0	0	0	0
Gglean012260	0	0	0	0
Gglean012261	0	0	0	0
Gglean012262	0	0	0	0
Gglean012263	0	0	0	0
Gglean012264	0	0	0	0
Gglean012265	16.46197	13.12641	14.48746	12.0598
Gglean012266	28.6985	27.5445	29.758	26.547
Gglean012267	0.185013	0.190544	0	0.088908
Gglean012268	94.0749	83.41038	54.02988	42.25152
Gglean012269	33.12426	33.60976	55.65549	56.59488
Gglean012270	0	0	0	0
Gglean012271	0.796823	0.929805	0.810497	0.957321
Gglean012272	4.24822	4.09558	2.81268	1.23764
Gglean012273	46.67326	47.407898	33.86416267	32.24952786
Gglean012274	22.03669	17.67715	2.82921	2.547786
Gglean012275	598.2788	526.6303	877.3731	922.0503

Gglean012276	13.77022	14.10052	9.50481	10.80627
Gglean012277	40.6998125	41.657534	26.512708	26.57111293
Gglean012278	40.6998125	41.657534	26.512708	26.57111293
Gglean012279	40.9506	41.1164	41.4446	38.0462
Gglean012280	45.43357005	47.16127	44.71940475	43.80088437
Gglean012281	45.43357005	47.16127	44.71940475	43.80088437
Gglean012282	79.0697	73.7408	48.5582	37.13419
Gglean012283	79.0697	73.7408	48.5582	37.13419
Gglean012284	24.94964125	27.83245	22.79456877	22.18311003
Gglean012285	145.9736	128.4482	88.9365	79.8915
Gglean012286	85.3825	88.42	93.0024	94.7866
Gglean012287	44.14480636	54.3894339	68.093301	72.664583
Gglean012288	44.14480636	54.3894339	68.093301	72.664583
Gglean012289	0	0	0.351764	0
Gglean012290	0	0.253423	0	0
Gglean012291	6.67E-05	5.83E-07	0.0128248	2.20E-07
Gglean012292	0	0	0	0
Gglean012293	2.55683	3.34369	3.25372	1.98677
Gglean012294	70.44094	64.30394	63.46912	66.14207
Gglean012295	30.3955	30.14096	30.3685	24.64697
Gglean012296	13.04572	13.35319	16.8694	19.1843
Gglean012297	221.65042	234.7546648	144.25402	121.6823951
Gglean012298	25.34783813	24.90729493	24.48553266	25.68509
Gglean012299	6.174637158	5.371	3.10315	6.28319
Gglean012300	12.38103	14.66564	9.65086	8.86669
Gglean012301	37.75829002	36.954369	31.51254885	31.33609695
Gglean012302	37.75829002	36.954369	31.51254885	31.33609695
Gglean012303	1.78686	2.45951	0.740108	0.44137
Gglean012304	21.1394	22.2446	13.9769	13.6553
Gglean012305	43.32812	37.05014	21.36057	22.23296
Gglean012306	43.32812	37.05014	21.36057	22.23296
Gglean012307	0	0	0	0
Gglean012308	2.45607	2.09943	1.91892	1.52213
Gglean012309	2.05592	0.367949	0.281078	0.673322
Gglean012310	0	0	0	0
Gglean012311	13.31208	14.44512	8.66565	8.31979
Gglean012312	15.3596	16.9406	3.37918	2.82433
Gglean012313	32.3717	30.442	18.287	15.1323
Gglean012314	11.9943	11.192	9.42228	8.72694
Gglean012315	11.9943	11.192	9.42228	8.72694
Gglean012316	18.67003	21.38053	4.591814	3.877979136
Gglean012317	16.00492	12.12143	9.2223	9.19905
Gglean012318	9.291915	11.7010011	18.14104645	22.249567
Gglean012319	62.169583	68.10116008	95.92504	114.4207101

Gglean012320	62.169583	68.10116008	95.92504	114.4207101
Gglean012321	22.0743	20.5529	13.9914	13.8581
Gglean012322	21.653823	21.851005	20.723	19.6175542
Gglean012323	11.7309	18.9046	24.1816	24.2689
Gglean012324	31.27582	26.5513	37.11475	38.17047
Gglean012325	0	0.161696	0	0.150536
Gglean012326	36.1115332	36.05189	29.84495	26.974232
Gglean012327	48.11749	54.683	66.0482	81.6531
Gglean012328	45.66955213	46.39980531	56.2270217	50.88369939
Gglean012329	45.66955213	46.39980531	56.2270217	50.88369939
Gglean012330	11.8623	22.13676	22.56638	26.274
Gglean012331	96.76111	141.234983	18.6213193	23.5446856
Gglean012332	96.76111	141.234983	18.6213193	23.5446856
Gglean012333	14.4623	15.1654	9.5326	10.0838
Gglean012334	14.86613396	12.6173897	13.86466772	15.65410002
Gglean012335	14.86613396	12.6173897	13.86466772	15.65410002
Gglean012336	23.17950678	23.88123201	37.78648701	44.37736234
Gglean012337	23.17950678	23.88123201	37.78648701	44.37736234
Gglean012338	6.281736522	4.598500312	11.576388	9.4418454
Gglean012339	47.164299	54.324002	44.993906	43.272115
Gglean012340	38.14421	44.93767	29.95261	25.9733
Gglean012341	61.201147	65.89211569	42.70723562	39.78618796
Gglean012342	61.201147	65.89211569	42.70723562	39.78618796
Gglean012343	2.67962	3.13008	2.38377	2.74624
Gglean012344	0	0	0	0
Gglean012345	20.721864	18.717062	13.433243	12.02768118
Gglean012346	27.68619	25.59015	26.29264	23.7696
Gglean012347	28.3321	31.982	29.8091	28.2065
Gglean012348	15.10685	15.21376	16.6627	18.99717
Gglean012349	10.751	11.5334	0.243427	0
Gglean012350	6.36506	11.5517	1.05382	0
Gglean012351	49.1456783	54.15612438	69.09741652	73.55547
Gglean012352	49.1456783	54.15612438	69.09741652	73.55547
Gglean012353	0	0	0	0
Gglean012354	23.0408652	31.4345952	30.848731	42.4215332
Gglean012355	24.084868	30.933049	44.375444	51.265101
Gglean012356	24.084868	30.933049	44.375444	51.265101
Gglean012357	0	0	0	0
Gglean012358	32.655	29.97057	30.00486	33.80477
Gglean012359	111.569	98.8888	70.2367	75.1785
Gglean012360	1.534	1.62453	1.4044	0.834349
Gglean012361	73.43805	85.10272	85.84794378	106.9133
Gglean012362	22.33225573	23.336393	19.70527514	18.65029
Gglean012363	22.33225573	23.336393	19.70527514	18.65029

Gglean012364	20.65421	24.56025	40.2112	41.306467
Gglean012365	20.65421	24.56025	40.2112	41.306467
Gglean012366	18.8853354	24.07681472	32.03969937	36.89003632
Gglean012367	24.7181	25.75529002	30.12490237	31.58213
Gglean012368	97.4886	100.9417383	114.1905735	105.94946
Gglean012369	24.313545	26.173947	16.367733	15.27563
Gglean012370	16.12367	13.6768	16.69739	18.43713
Gglean012371	4.4194	3.14349	4.68921	4.33126
Gglean012372	30.9149	32.891	29.81038	38.8642
Gglean012373	13.19458599	14.679872	27.01826	29.98868867
Gglean012374	11.23290592	10.39223481	6.543681025	6.082804205
Gglean012375	0.844672219	0.590099541	0.541753407	0.565292394
Gglean012376	19.905	19.1228	23.5305	23.1096
Gglean012377	77.5353	75.6639	105.1787	122.9152
Gglean012378	13.3329	14.8265	9.41399	8.92265
Gglean012379	15.71014	16.1849	9.97984	9.69768
Gglean012380	19.39303	18.42203	19.03297	21.32151
Gglean012381	4.1801	2.39455	1.73474	0
Gglean012382	22.34452278	26.2625098	24.00416155	22.98629068
Gglean012383	13.01783	14.21079	9.72243	11.72656
Gglean012384	0.108542822	0	0	0
Gglean012385	25.5542	27.2146	14.1348	12.53490008
Gglean012386	9.134509867	8.445606	13.74484	13.11752
Gglean012387	40.85173465	46.24150252	20.91330588	16.3361898
Gglean012388	13.26680803	13.33726803	19.79137575	20.81698458
Gglean012389	0	0	0	0
Gglean012390	0.220063025	0.3571352	0.525345908	0.916126279
Gglean012391	39.624803	44.77142	15.063032	11.563446
Gglean012392	97.35494	105.55089	126.50246	135.95886
Gglean012393	30.16665915	28.59900281	20.711372	21.12974558
Gglean012394	30.16665915	28.59900281	20.711372	21.12974558
Gglean012395	32.517985	32.791066	27.14657	24.6574138
Gglean012396	0.333673	0	0	0.16228
Gglean012397	7.06259	7.67387	7.98163	5.6254
Gglean012398	36.16127858	34.6969	35.849521	37.402312
Gglean012399	14.6728	15.7372	8.39144	7.40859
Gglean012400	28.13194	31.08168403	19.21574	17.29361101
Gglean012401	7.601644627	6.66656414	8.003813767	9.215772
Gglean012402	16.14252571	15.8325293	16.86659937	17.68843039
Gglean012403	90.1919	95.80628	29.55794	24.27992
Gglean012404	249.008	226.146	105.346	86.5639
Gglean012405	16.094878	14.265105	15.377672	16.80751805
Gglean012406	16.094878	14.265105	15.377672	16.80751805
Gglean012407	3.305697468	3.246674567	4.938786525	6.183483317

Gglean012408	96.28428	95.79652	100.33127	112.897931
Gglean012409	96.28428	95.79652	100.33127	112.897931
Gglean012410	177.542849	182.5161	186.4061804	169.5337
Gglean012411	177.542849	182.5161	186.4061804	169.5337
Gglean012412	10.89379	11.08424	10.51001	19.03561
Gglean012413	1.393566051	1.500219262	1.39232316	1.952988603
Gglean012414	31.77723	31.29841	35.31866	37.01158
Gglean012415	55.99946604	63.09399328	56.95033138	55.6331342
Gglean012416	10.79813	13.83542	7.21015	5.29037
Gglean012417	37.68819	33.60156362	29.04967	27.08104
Gglean012418	0	0	0	0
Gglean012419	0	0	0	0
Gglean012420	3.606271	2.988652106	4.151851	4.91782
Gglean012421	10.2265112	10.06424951	14.26944806	15.91502509
Gglean012422	0.185859214	0	0.07752447	0
Gglean012423	5.346972499	5.870861	4.981218713	4.478184831
Gglean012424	37.25893	41.72994	33.09416	32.9863
Gglean012425	31.08834	31.41624	14.78917952	15.442398
Gglean012426	31.08834	31.41624	14.78917952	15.442398
Gglean012427	21.09588	20.407351	18.799758	18.567419
Gglean012428	30.71228	27.71505	20.11001197	16.96280346
Gglean012429	30.71228	27.71505	20.11001197	16.96280346
Gglean012430	50.9542521	56.54244371	15.93627445	16.38884741
Gglean012431	1.56332	1.6279	1.64398	2.54926
Gglean012432	0	0	0	0
Gglean012433	0	0	0	0
Gglean012434	0	0	0	0
Gglean012435	355.147	342.245	367.068	346.88
Gglean012436	0	0	0	0
Gglean012437	115.8236994	156.3891	92.298582	82.1582295
Gglean012438	115.8236994	156.3891	92.298582	82.1582295
Gglean012439	3.607498024	5.139453035	2.785239036	3.370488407
Gglean012440	2.42257522	2.537701637	2.172239126	2.694014567
Gglean012441	2.42257522	2.537701637	2.172239126	2.694014567
Gglean012442	84.508294	76.12235	114.9959219	132.323262
Gglean012443	5.782918525	6.674899624	4.19032195	10.01510012
Gglean012444	3.003554	1.965619	3.36877	0.0157986
Gglean012445	39.069633	34.2086	21.311388	23.587138
Gglean012446	32.60613414	30.808839	18.96244774	17.93027175
Gglean012447	24.98325	26.65419	17.60347	16.86668
Gglean012448	155.7875	137.2397	76.96867	68.91638
Gglean012449	5.9287563	5.196053322	6.643460133	7.804222001
Gglean012450	5.9287563	5.196053322	6.643460133	7.804222001
Gglean012451	0.939297054	0.564848062	1.184833056	1.913451643

Gglean012452	0	0	0	0
Gglean012453	1.02194	0.844092	0.826125	0.452865
Gglean012454	0.0559274	10.14922	0.210596198	0.285985
Gglean012455	12.16817	10.14922	7.17214	5.98147
Gglean012456	0	0	0	0
Gglean012457	31.534732	46.39135	40.31164	34.60426
Gglean012458	31.534732	46.39135	40.31164	34.60426
Gglean012459	79.9054303	87.1184539	93.85956738	93.11331987
Gglean012460	164.4375	166.6555	59.45310214	61.940695
Gglean012461	73.0059	85.5007	54.674	51.5957
Gglean012462	39.2303468	44.3050089	44.0082123	51.46579804
Gglean012463	2.86414213	2.5820242	1.88783925	2.34647061
Gglean012464	2.86414213	2.5820242	1.88783925	2.34647061
Gglean012465	8.250815	6.28297	9.598122793	11.2461238
Gglean012466	0.276178	0.0812612	0.225429	0.26649
Gglean012467	7.976945831	7.427839293	11.70002956	11.71702726
Gglean012468	178.74423	156.25359	86.721843	69.82433
Gglean012469	285.218304	252.054373	271.49883	324.156
Gglean012470	84.5548	96.0471	60.418	50.0366
Gglean012471	3.99952	4.65713	4.59254	9.16129
Gglean012472	308.6967	261.6607	290.51247	357.1526
Gglean012473	308.6967	261.6607	290.51247	357.1526
Gglean012474	308.6967	261.6607	290.51247	357.1526
Gglean012475	22.4949	31.28162474	37.43903	45.88496203
Gglean012476	13.8925	5.25408	26.1392	70.4491
Gglean012477	112.71754	112.6619097	165.27644	168.398573
Gglean012478	112.71754	112.6619097	165.27644	168.398573
Gglean012479	0	0	0	0
Gglean012480	0	0	0	0
Gglean012481	16.0803	15.3138	13.8605	13.2488
Gglean012482	0	0	0.0674662	0
Gglean012483	50.08943	47.51399	52.330835	45.80098
Gglean012484	39.10069	36.80212	41.57599	41.80589
Gglean012485	7.09486	7.02777	9.02124	10.2393
Gglean012486	0	0	0	0
Gglean012487	22.43565302	23.538735	13.19155629	12.48239589
Gglean012488	6.0536	5.94871	9.03181	8.60827
Gglean012489	22.620328	26.91440969	31.37565888	35.959764
Gglean012490	11.2452	12.7151	13.6064	22.4586
Gglean012491	0	0	0	0
Gglean012492	272.149199	262.422031	242.747589	427.105973
Gglean012493	272.149199	262.422031	242.747589	427.105973
Gglean012494	13.97590905	16.14674	18.94356839	23.30708898
Gglean012495	0	0	0.15693	0.0928134

Gglean012496	0.95174041	0.4052327	0.393683096	0.408577
Gglean012497	49.29506911	50.23109814	45.181717	41.8154548
Gglean012498	23.16822536	25.5980067	20.81417922	19.96286417
Gglean012499	3.608470503	5.164274359	2.853928825	4.293138476
Gglean012500	3.608470503	5.164274359	2.853928825	4.293138476
Gglean012501	16.9267	24.4029	9.6752	9.60398
Gglean012502	21.26341	19.888155	23.804419	32.406085
Gglean012503	2.33063515	2.84632913	3.285397219	4.04958239
Gglean012504	0	0	0	0
Gglean012505	15.69410057	16.2284	14.5385	14.5392
Gglean012506	15.69410057	16.2284	14.5385	14.5392
Gglean012507	0	0	0	0
Gglean012508	42.46343452	44.01747618	33.51152452	33.34371311
Gglean012509	42.46343452	44.01747618	33.51152452	33.34371311
Gglean012510	14.16036	16.43937	11.18085	13.09195
Gglean012511	15.162127	13.70272629	18.99753122	19.03272737
Gglean012512	19.6654526	19.643164	29.444791	32.20251705
Gglean012513	29.6931749	31.0672611	16.34896	18.9029853
Gglean012514	8.758865389	7.884839	9.5755	13.92906
Gglean012515	272.149199	262.422031	242.747589	427.105973
Gglean012516	0	0	0	0
Gglean012517	0	0.298951	0	0
Gglean012518	0.088349273	0.484904939	0.0486209	0.112782161
Gglean012519	0.088349273	0.484904939	0.0486209	0.112782161
Gglean012520	27.18763	23.03586	27.1318	35.18789
Gglean012521	3.9148734	6.19837	2.49856	2.156420001
Gglean012522	1.35336	1.21668	1.55758	1.02214
Gglean012523	13.50025132	12.95800477	14.18580101	11.518441
Gglean012524	2.873584323	2.983271633	4.249939537	4.63268116
Gglean012525	132.12937	133.847803	102.678029	95.10145429
Gglean012526	76.289994	75.525451	71.510442	78.403041
Gglean012527	42.9586	40.9091	33.7907	32.3642
Gglean012528	26.74656	30.66576	34.33631	32.45339
Gglean012529	60.01167	61.68108	48.70144	46.46033
Gglean012530	8.942528411	8.766292786	12.9975732	12.7082643
Gglean012531	8.942528411	8.766292786	12.9975732	12.7082643
Gglean012532	1.590946	0.929061342	1.018493732	0.946348
Gglean012533	1.590946	0.929061342	1.018493732	0.946348
Gglean012534	1.590946	0.929061342	1.018493732	0.946348
Gglean012535	3.38E-05	0.00753933	0.757462	0.288209
Gglean012536	0	0	0	0
Gglean012537	1.99757	1.37541	1.25339	1.27318
Gglean012538	6.41372	5.1968	4.80636	6.00903
Gglean012539	32.742686	37.83323	16.08810959	11.532885

Gglean012540	32.742686	37.83323	16.08810959	11.532885
Gglean012541	8.90741	8.92202	9.56185	9.36659
Gglean012542	90.92544	89.81639	113.64489	125.91148
Gglean012543	1.66902	1.6483	1.21969	1.18916
Gglean012544	55.6850545	62.95710176	77.52506193	87.40168719
Gglean012545	34.0093	38.48169	32.10869	34.71515
Gglean012546	75.62888	87.30143	67.816134	69.9086
Gglean012547	0.32584348	0.3762949	0.563574	0.365792
Gglean012548	0.32584348	0.3762949	0.563574	0.365792
Gglean012549	0	1.23778	0	1.0857
Gglean012550	9.740087	8.59722	6.80039	7.3191
Gglean012551	0.277091785	0.171138827	0.18061796	0.106030412
Gglean012552	0.277091785	0.171138827	0.18061796	0.106030412
Gglean012553	0	0	0	0
Gglean012554	0.0789881	0.247762	0.257459	0
Gglean012555	3.15993	2.96413	4.99525	3.81016
Gglean012556	11.6743	7.02148	19.6547	21.6096
Gglean012557	0.145499	0.149564	0.117742	0
Gglean012558	30.97144	30.11718	49.70068	52.42353
Gglean012559	0	0	0	0
Gglean012560	0	0	0	0
Gglean012561	1.986798123	1.718855465	2.765018493	2.77081
Gglean012562	41.3124	40.23267	68.36921	75.8014
Gglean012563	25.3471	23.42764	14.894785	12.724811
Gglean012564	0	0	0	0
Gglean012565	0	0	0	0
Gglean012566	0.526765305	0.4373548	0.620974016	0.516254462
Gglean012567	24.878	27.061	19.1519	22.3664
Gglean012568	2.31566	2.50856	3.82224	6.10539
Gglean012569	36.70458	40.45018	24.43761	22.95504
Gglean012570	16.10919	13.75164	12.17815	9.62021
Gglean012571	16.10919	13.75164	12.17815	9.62021
Gglean012572	20.05057985	23.19562784	14.49564223	12.89427327
Gglean012573	35.6967392	28.07811	42.90167668	50.41890964
Gglean012574	35.6967392	28.07811	42.90167668	50.41890964
Gglean012575	35.6967392	28.07811	42.90167668	50.41890964
Gglean012576	48.66266279	42.14243882	37.77020798	41.73296091
Gglean012577	48.66266279	42.14243882	37.77020798	41.73296091
Gglean012578	43.3573	43.1309	30.7126	28.0331
Gglean012579	11.0542	11.205107	16.38797	19.93681
Gglean012580	26.53998264	27.29197	43.032623	48.32318071
Gglean012581	21.14791	18.23343807	27.07058	31.9729731
Gglean012582	11.77589542	14.12925366	2.857637266	2.653023855
Gglean012583	49.48762	53.55886	46.1182	50.08057

Gglean012584	49.48762	53.55886	46.1182	50.08057
Gglean012585	1.638804269	1.400554	2.016274	2.732237133
Gglean012586	28.33212259	34.40516	44.9297	54.88652
Gglean012587	0.475081939	0.644611044	0.587613997	0.697322081
Gglean012588	32.16282595	34.4173004	22.1036076	22.54643182
Gglean012589	35.1115547	35.597829	27.44792291	22.90982602
Gglean012590	49.65616	45.70771464	51.8645	53.85054717
Gglean012591	33.2272	36.2634	25.78984	25.81949
Gglean012592	44.768001	44.17508156	88.64519	87.25089133
Gglean012593	44.768001	44.17508156	88.64519	87.25089133
Gglean012594	141.55	150.176	94.9089	81.4475
Gglean012595	27.734201	26.866272	26.592457	26.8365929
Gglean012596	0	0	0.0750819	0
Gglean012597	0	0	0	0
Gglean012598	17.050725	18.1658501	9.289403379	9.081029459
Gglean012599	24.2667	31.2234	6.88284	6.30116
Gglean012600	19.396371	21.28991	23.03501	23.03632
Gglean012601	80.25834076	83.50116532	68.06466	64.67670886
Gglean012602	0.0327495	0.101334	0	0.06245941
Gglean012603	13.9139	15.1924	15.22	14.7498
Gglean012604	31.91200761	33.83301227	30.09847652	31.18541536
Gglean012605	31.91200761	33.83301227	30.09847652	31.18541536
Gglean012606	31.91200761	33.83301227	30.09847652	31.18541536
Gglean012607	16.39321888	16.9928667	26.4480842	31.20700511
Gglean012608	24.1219	27.3314	19.8769	16.6508
Gglean012609	1.60E-05	0.000102985	0.841375	0.144808
Gglean012610	81.829826	80.5250249	66.22295411	70.25697714
Gglean012611	81.829826	80.5250249	66.22295411	70.25697714
Gglean012612	0.0821516	0.0857095	0.0330321	0.0390805
Gglean012613	56.72951	55.96531	73.4053	76.29979
Gglean012614	0.224944	0.0290372	0.446977088	0.609466
Gglean012615	7.70425799	10.7055238	14.28775256	16.383362
Gglean012616	7.70425799	10.7055238	14.28775256	16.383362
Gglean012617	7.70425799	10.7055238	14.28775256	16.383362
Gglean012618	30.16463346	28.05406575	24.22842382	22.58947638
Gglean012619	198.331037	215.8330286	102.0620597	83.07281874
Gglean012620	9.551215365	8.025224828	5.294422	6.461401466
Gglean012621	76.10668574	83.64106305	65.07433	54.70605972
Gglean012622	31.3393	28.1276	17.1603	22.2083
Gglean012623	0	0	0	0
Gglean012624	0	0	0	0
Gglean012625	37.57353984	40.25228632	26.59832594	21.55092963
Gglean012626	238.3986	232.4914	330.9017	378.8187
Gglean012627	28.58243	29.38742	29.22448	28.98724

Gglean012628	28.58243	29.38742	29.22448	28.98724
Gglean012629	15.465914	16.381784	17.93201	16.75399331
Gglean012630	15.465914	16.381784	17.93201	16.75399331
Gglean012631	52.45233005	45.2626179	48.29159	43.32834022
Gglean012632	2.495807194	2.2357106	2.9263572	3.834013276
Gglean012633	2.495807194	2.2357106	2.9263572	3.834013276
Gglean012634	17.08215	18.27404	20.23519	22.58634
Gglean012635	1002.349	1025.629	668.987	680.749
Gglean012636	28.589994	31.351825	24.02927	26.866381
Gglean012637	28.589994	31.351825	24.02927	26.866381
Gglean012638	18.47480205	17.01573	15.252435	17.143644
Gglean012639	33.82761647	36.56141142	24.82615546	21.34576581
Gglean012640	27.60824431	34.51964103	16.95665821	14.81406661
Gglean012641	6.380534844	4.394644263	5.539254296	6.567825685
Gglean012642	6.380534844	4.394644263	5.539254296	6.567825685
Gglean012643	29.71748	32.18285	24.4904	26.55692
Gglean012644	29.71748	32.18285	24.4904	26.55692
Gglean012645	51.6347	53.2598	40.0695	36.8691
Gglean012646	44.60227565	44.9519629	44.92405727	38.04545805
Gglean012647	12.46	18.0774	6.16897	6.56505
Gglean012648	17.97283601	21.84315851	20.33080312	19.07900224
Gglean012649	30.1132	30.7603	42.2839	49.9588
Gglean012650	114.027	104.347	75.0131	79.0668
Gglean012651	122.64872	117.76904	118.45155	121.71006
Gglean012652	0.0574541	0.059498	0.0937726	0.222173
Gglean012653	27.9923	21.906	28.0753	26.5452
Gglean012654	39.55105	39.96174	15.61601	9.566721126
Gglean012655	127.81134	129.0470049	128.2043384	130.3832596
Gglean012656	0	0	0	0
Gglean012657	0	0	0	0
Gglean012658	51.05094	53.01164	37.75072707	34.56793101
Gglean012659	123.4764097	123.072514	126.79157	125.5936783
Gglean012660	42.619161	48.1594983	37.316911	36.932467
Gglean012661	42.619161	48.1594983	37.316911	36.932467
Gglean012662	42.619161	48.1594983	37.316911	36.932467
Gglean012663	20.2429573	19.27901246	29.4677312	29.03171762
Gglean012664	3.48085	3.33185	3.71312	7.48999
Gglean012665	14.95943	15.59717	9.845327	8.113755
Gglean012666	65.86130306	86.145443	86.30883217	111.9189261
Gglean012667	16.19436	19.566308	28.11183	30.11532
Gglean012668	8.58191	8.71663	9.68652	10.777229
Gglean012669	0.101277	0.0528489	0.16532	0.0976291
Gglean012670	0	0	0	0.031592341
Gglean012671	0.208201	0.140372	1.73E-06	6.70E-06

Gglean012672	39.13097	42.2328222	31.6097	28.271472
Gglean012673	39.13097	42.2328222	31.6097	28.271472
Gglean012674	13.0791557	15.47992401	29.22726017	29.62335311
Gglean012675	13.0791557	15.47992401	29.22726017	29.62335311
Gglean012676	1.122854418	1.382037144	2.102532	2.318890518
Gglean012677	2.016314381	2.548126946	3.224894364	4.01179604
Gglean012678	0	0	0	0
Gglean012679	104.1841	93.323	92.3367	90.0461
Gglean012680	8.27994	10.43711	7.02462	9.08074
Gglean012681	167.312886	153.836311	117.21483	105.1426769
Gglean012682	40.55118816	42.85483	39.466711	39.24835
Gglean012683	0.173491	0.419832	0.187519	0.222766
Gglean012684	20.67374	21.70405	16.57968	15.51211
Gglean012685	215.35779	235.3638	167.49682	153.63962
Gglean012686	215.35779	235.3638	167.49682	153.63962
Gglean012687	2.23336	0.349391	1.170370118	0.835609
Gglean012688	0.470439	0.244352	1.3345	1.01482
Gglean012689	0.270911	0.559975	0.768263	0.616004
Gglean012690	34.9372	35.86879	18.36556	15.96017
Gglean012691	9.496522	7.72599	9.04578	9.800198
Gglean012692	0.0520271	0.101755	0.105039	0.0742657
Gglean012693	9.82E-237	0.235662	0.131872	9.28101999999
Gglean012694	45.90874	48.241671	39.170428	39.464908
Gglean012695	46.6674	36.7803	22.8026	25.7227
Gglean012696	7.76791	6.01747	4.34089	4.13451
Gglean012697	25.5132	23.62406	18.8757	17.50844
Gglean012698	132.45768	113.43502	98.07899	120.6331
Gglean012699	85.7968	91.55562	91.64525	102.01834
Gglean012700	0.244374	0	0.197047	0.116983
Gglean012701	2.23083000125	0	0	0
Gglean012702	2.23083000125	0	0	0
Gglean012703	0	0	0	0
Gglean012704	0	0	0	0
Gglean012705	0.0200031	6.79E-33	4.87E-07	1.87E-59
Gglean012706	239.6007316	252.3028491	204.8987806	192.6055751
Gglean012707	239.6007316	252.3028491	204.8987806	192.6055751
Gglean012708	239.6007316	252.3028491	204.8987806	192.6055751
Gglean012709	25.046	29.7973	48.5427	52.7481
Gglean012710	55.263296	54.5137454	14.10127625	8.818439089
Gglean012711	18.9151	19.7512	23.9264	27.2518
Gglean012712	135.4482	137.3646	110.697	95.9444
Gglean012713	39.4681	47.3235	29.4841	24.4553
Gglean012714	15.7141	16.003	16.9374	17.1194
Gglean012715	16.64685308	15.9655311	20.8182684	20.72274

Gglean012716	0.257137065	0.184984637	0.16321495	0.087133422
Gglean012717	0	0	0	0
Gglean012718	64.1317	68.4738	43.76	33.8586
Gglean012719	0.600307	0.281152	1.68603	1.32288
Gglean012720	0	0	0	0
Gglean012721	1.24492	0.427625	0.134208	0
Gglean012722	57.7928	57.35673	36.80588	29.83884
Gglean012723	1.160641503	0.738062527	1.1577266	1.129522389
Gglean012724	0.229193432	0.424437238	0.314548975	0.425902447
Gglean012725	0.511169351	0.779677591	0.576091336	0.468340104
Gglean012726	2.5561	3.15E-16	1.30E-08	7.54549
Gglean012727	0.107993453	0.043300229	0.018297239	0.021453108
Gglean012728	2.01515475	2.412896875	2.81162956	3.558572589
Gglean012729	2.01515475	2.412896875	2.81162956	3.558572589
Gglean012730	3.39416	3.52704	5.83902	6.68997
Gglean012731	0.020304391	0	0.01631383	0.019291238
Gglean012732	0	0	0	0
Gglean012733	19.21995384	19.18482624	14.46136057	13.34315797
Gglean012734	21.96144	22.088546	17.68591	15.24936593
Gglean012735	0.103352	0	0.02854809	0
Gglean012736	0.103352	0	0.02854809	0
Gglean012737	84.01780718	101.0618654	42.63668	40.94382534
Gglean012738	84.01780718	101.0618654	42.63668	40.94382534
Gglean012739	26.052609	27.75689827	29.82962556	27.10143715
Gglean012740	26.052609	27.75689827	29.82962556	27.10143715
Gglean012741	4.88351	7.27252	9.01171	12.74827
Gglean012742	4.88351	7.27252	9.01171	12.74827
Gglean012743	0	0	0	0
Gglean012744	0.86552	0.20361	0.0960666	0.472437
Gglean012745	10.084978	11.779433	8.10576	7.395354154
Gglean012746	0	0	0	0
Gglean012747	0	0	0	0
Gglean012748	0	0	0	0
Gglean012749	20.74348	17.60406272	15.05023	15.01968
Gglean012750	22.1393	25.6583	13.0732	11.21186
Gglean012751	0	0	0	0
Gglean012752	0.0949999	0.358209	0.128905	0.152456
Gglean012753	33.7625	24.8615	23.0207	27.0375
Gglean012754	20.37590715	19.13372546	20.8690517	19.95190851
Gglean012755	0	0	0	0
Gglean012756	0	0	0	0
Gglean012757	21.75897002	23.96513	33.27683	39.19513
Gglean012758	0.127565	0.133474	0.208661	0.123714
Gglean012759	333.40824	438.3401059	15.60629	16.84979301

Gglean012760	33.60925	34.4289	34.257348	32.11116161
Gglean012761	33.60925	34.4289	34.257348	32.11116161
Gglean012762	0	0	0.144335	0
Gglean012763	7.74549	9.21241	8.10059	12.3013
Gglean012764	2.336597408	2.338869358	3.126274948	3.96151985
Gglean012765	2.336597408	2.338869358	3.126274948	3.96151985
Gglean012766	8.190399075	7.229856998	9.625757911	10.441909
Gglean012767	8.190399075	7.229856998	9.625757911	10.441909
Gglean012768	46.21495605	42.78327591	34.81674151	31.12206996
Gglean012769	46.21495605	42.78327591	34.81674151	31.12206996
Gglean012770	46.21495605	42.78327591	34.81674151	31.12206996
Gglean012771	46.21495605	42.78327591	34.81674151	31.12206996
Gglean012772	4.87921	4.87348	4.94346	8.8895
Gglean012773	16.28647	13.04163	14.78498	15.29088
Gglean012774	0.904311	1.1620854	0.696837476	1.295370828
Gglean012775	0.904311	1.1620854	0.696837476	1.295370828
Gglean012776	82.87051196	89.16850361	114.5211728	148.4561596
Gglean012777	82.87051196	89.16850361	114.5211728	148.4561596
Gglean012778	16.66865183	16.2235809	25.04788265	30.0205426
Gglean012779	4.134997106	4.737074499	6.308630152	7.356073001
Gglean012780	4.134997106	4.737074499	6.308630152	7.356073001
Gglean012781	20.9961	22.6617	12.4707	9.63585
Gglean012782	6.38425	5.00986	3.67003	5.59095
Gglean012783	0.070702001	0.438780083	1.034815	0.549809
Gglean012784	8.45633	8.79368	8.37051	8.4157
Gglean012785	129.2751411	105.91201	39.32292	29.76264424
Gglean012786	129.2751411	105.91201	39.32292	29.76264424
Gglean012787	129.2751411	105.91201	39.32292	29.76264424
Gglean012788	59.93875	55.42102	55.227005	58.80937
Gglean012789	76.36852	72.60747	79.75852	74.70206
Gglean012790	76.36852	72.60747	79.75852	74.70206
Gglean012791	16.619656	18.08679	20.30998	28.30384
Gglean012792	16.51703171	14.19117998	16.88208564	17.143935
Gglean012793	53.9681	58.71344514	37.7986	33.72933925
Gglean012794	58.98908	60.55635	80.1527	88.0151
Gglean012795	150.167	163.218	117.062	106.794
Gglean012796	22.57839341	20.83727512	33.55214	35.16012516
Gglean012797	98.0347	68.6984	139.0163	209.247
Gglean012798	1.15823938	1.0734521	0.91975278	0.543364809
Gglean012799	58.63129	70.421	5.22205	3.74189
Gglean012800	2.4464	1.68179	4.1473	3.83117
Gglean012801	27.73085	24.95454556	18.46219907	17.51333963
Gglean012802	48.7267	42.37248	59.70465	77.1162
Gglean012803	48.7267	42.37248	59.70465	77.1162

Gglean012804	17.100753	19.43131184	2.848358465	2.326601
Gglean012805	1.0685258	1.352544175	0.757463054	0.530355421
Gglean012806	0	0	0.169396	0
Gglean012807	1.156861317	0.891915072	0.310384164	0.672693
Gglean012808	1.156861317	0.891915072	0.310384164	0.672693
Gglean012809	7.15E-26	0.00124829	2.16294	2.47E-16
Gglean012810	3.80377	4.18563	2.125486	2.49287
Gglean012811	0	0	0	0
Gglean012812	0	0	0	0
Gglean012813	0	0	0	0
Gglean012814	0.2023078	0.2061905	0.2034596	0.229283
Gglean012815	2.89336	1.45784	4.27309	2.80996
Gglean012816	0	0	0	0
Gglean012817	0.0696655	0	0.0566369	0.135165
Gglean012818	10.4483479	11.8639513	19.130671	20.99964275
Gglean012819	0	0	0.456121	0
Gglean012820	36.28777793	39.43363503	30.76663313	30.97089103
Gglean012821	20.31355	18.11338	10.224001	10.34334452
Gglean012822	48.36442	42.28693	36.61629	34.88031
Gglean012823	0	0	0	0
Gglean012824	0	0	0	0
Gglean012825	5.067678319	4.819354	8.89462	12.56179958
Gglean012826	41.71806792	40.9593308	73.20158311	69.85039485
Gglean012827	41.71806792	40.9593308	73.20158311	69.85039485
Gglean012828	41.71806792	40.9593308	73.20158311	69.85039485
Gglean012829	7.992721711	7.497969221	4.298637515	3.727088157
Gglean012830	7.992721711	7.497969221	4.298637515	3.727088157
Gglean012831	277.314621	267.508168	347.921891	527.608875
Gglean012832	29.40238	32.50353	28.51676	23.75056
Gglean012833	0	0	0.0529197	0
Gglean012834	31.14902	33.693419	24.90295	21.23782
Gglean012835	47.19871	48.08362	35.44748	34.45599
Gglean012836	16.31478197	17.30743255	26.970862	25.7459636
Gglean012837	16.31478197	17.30743255	26.970862	25.7459636
Gglean012838	0.0207116	0.0213635	0.0167538	0
Gglean012839	0.0207116	0.0213635	0.0167538	0
Gglean012840	48.43179016	45.94496085	50.99976875	51.43684044
Gglean012841	0.239180007	0.195462189	0.154179368	0.411408687
Gglean012842	35.66764	31.668949	35.948324	35.386737
Gglean012843	61.0431095	67.73275739	56.01616528	57.04619201
Gglean012844	6.703057021	7.523544998	2.825069679	2.937076118
Gglean012845	133.192	151.4522	189.009	239.4425
Gglean012846	133.192	151.4522	189.009	239.4425
Gglean012847	79.8552	114.741	191.964	209.728

Gglean012848	117.699	112.899	212.268	232.452
Gglean012849	261.3076	278.2421229	397.2364	504.8282
Gglean012850	32.48542674	29.530829	38.07257	33.54325459
Gglean012851	18.07218	17.32882	16.0131077	18.40961823
Gglean012852	16.700165	15.39389	15.881538	15.753507
Gglean012853	10.10645388	8.201713077	16.779126	16.86554402
Gglean012854	0	0	0	0
Gglean012855	52.27818061	55.4279402	25.97599	26.84341
Gglean012856	52.27818061	55.4279402	25.97599	26.84341
Gglean012857	135.6412332	137.850397	98.36902152	97.191066
Gglean012858	135.6412332	137.850397	98.36902152	97.191066
Gglean012859	135.6412332	137.850397	98.36902152	97.191066
Gglean012860	8.083152	3.92647	6.39728	7.61721
Gglean012861	0	0	0	0
Gglean012862	12.35061526	10.86100005	6.728341985	7.503892
Gglean012863	8.608965	9.70227	9.437255	9.71491
Gglean012864	0.478929	1.15652	1.41342	0.771253
Gglean012865	87.32784	89.12882	90.22771	93.23528
Gglean012866	56.33687022	64.46355073	54.70110297	48.25899866
Gglean012867	15.10327	14.413	26.45376	22.02974
Gglean012868	79.76074984	84.32094295	135.3661944	130.8179471
Gglean012869	400.92392	341.11475	370.45888	401.86246
Gglean012870	400.92392	341.11475	370.45888	401.86246
Gglean012871	26.3805	17.9809	12.3245	15.2031
Gglean012872	0.289403	0.750682	1.16516	0.552956
Gglean012873	13.24169968	10.26755724	8.825614318	7.306031037
Gglean012874	0	0	0	0
Gglean012875	13.24169968	10.26755724	8.825614318	7.306031037
Gglean012876	0	0	0	0
Gglean012877	171.787608	170.9327167	226.192709	273.0945106
Gglean012878	171.787608	170.9327167	226.192709	273.0945106
Gglean012879	21.288011	35.75551897	42.18960082	57.895799
Gglean012880	21.288011	35.75551897	42.18960082	57.895799
Gglean012881	26.73855841	26.26241398	43.873818	51.30354827
Gglean012882	26.73855841	26.26241398	43.873818	51.30354827
Gglean012883	382.2705684	400.43102	202.8548534	166.97044
Gglean012884	382.2705684	400.43102	202.8548534	166.97044
Gglean012885	0	0	0	0
Gglean012886	13.08893608	11.54723633	17.3114318	18.16195109
Gglean012887	30.76308	42.64066	38.31145	45.30066
Gglean012888	25.99548	30.37045	19.25955	19.24211
Gglean012889	100.5124313	103.095617	81.97559822	71.083627
Gglean012890	88.18674768	89.66755923	64.01794049	58.59401013
Gglean012891	4.915153	8.779431	2.637797566	1.872677757

Gglean012892	4.915153	8.779431	2.637797566	1.872677757
Gglean012893	43.62223	42.10104	43.88657	46.33265
Gglean012894	27.689435	31.955	6.802397	5.4019786
Gglean012895	27.689435	31.955	6.802397	5.4019786
Gglean012896	10.55219481	9.48762016	13.80016306	17.05129512
Gglean012897	4.646956325	5.051725	6.023877267	8.138617057
Gglean012898	4.646956325	5.051725	6.023877267	8.138617057
Gglean012899	4.646956325	5.051725	6.023877267	8.138617057
Gglean012900	1.03393	0.731419	1.10188	1.51664
Gglean012901	13.08603	13.984769	28.26312	26.16466
Gglean012902	17.009771	20.460113	16.986057	20.775584
Gglean012903	0	0	0.136813	0
Gglean012904	9.201533048	9.845373	16.616053	16.090761
Gglean012905	0.0810264	0	0	0
Gglean012906	0	0	0	0
Gglean012907	15.59906	16.83891	12.37626	11.46558
Gglean012908	0	0	0	0
Gglean012909	0	0	0	0
Gglean012910	0.135421	0.140454	0.109798	0.130309
Gglean012911	0	0	0	0
Gglean012912	0	0	0	0
Gglean012913	0.384386	0.540684	0.478608991	0.47520308
Gglean012914	0	0	0	0
Gglean012915	1.902246784	1.53803511	1.163915436	1.284052655
Gglean012916	2.143785493	3.071267901	1.035650541	0.742075274
Gglean012917	2.143785493	3.071267901	1.035650541	0.742075274
Gglean012918	5.648013872	4.99892947	3.39921472	5.366890356
Gglean012919	65.9945	63.2181	175.4897	171.5631
Gglean012920	12.7323	12.9427	13.9829	11.0357
Gglean012921	0.119409	0.0405843	0.129031	0.420629
Gglean012922	0	0.397899	0	0
Gglean012923	0	0	0.0680036	0
Gglean012924	0	0	0	0
Gglean012925	67.52678	66.18854	82.8247	90.9725
Gglean012926	67.52678	66.18854	82.8247	90.9725
Gglean012927	11.15676	11.54648	13.43731	12.26255
Gglean012928	142.80569	102.853343	143.6463772	175.95489
Gglean012929	142.80569	102.853343	143.6463772	175.95489
Gglean012930	0.192666	0.200293	0.547744	0
Gglean012931	53.6072	40.1268	59.1405	58.906
Gglean012932	4.778687724	6.682477	7.434173	9.08613958
Gglean012933	4.778687724	6.682477	7.434173	9.08613958
Gglean012934	0.061665	0.127226	0	0.0592242
Gglean012935	6.16769	5.711794	8.330874	9.95873

Gglean012936	18.09278618	14.48747541	10.05605235	8.18827052
Gglean012937	0	0	0	0
Gglean012938	33.799022	31.2055876	17.8792399	13.8342037
Gglean012939	102.274	108.42	111.908	110.699
Gglean012940	29.47219	29.883	15.96537	16.18064
Gglean012941	35.69173	36.21131	2.125397	0.291333
Gglean012942	1245.583335	1296.954839	795.8541	756.2312
Gglean012943	2.851203803	2.675752337	3.550126171	5.242979483
Gglean012944	2.43587	2.72257	4.18942	4.99572
Gglean012945	16.87311141	15.5185083	21.56009636	26.37373008
Gglean012946	2.468949542	3.719432943	4.119175529	5.753881268
Gglean012947	15.4895	16.6945	29.3666	33.1766
Gglean012948	3.74253	3.17511	2.75293	2.62409
Gglean012949	0	0	0	0.308776
Gglean012950	3.18221	2.65661	3.09152	4.54094
Gglean012951	0	0	0	0.139037
Gglean012952	1.44283	1.90833	0.136995	0
Gglean012953	161.97856	172.41045	162.54681	153.31268
Gglean012954	35.2884247	38.67176	22.08098404	20.74941552
Gglean012955	4.76597	3.21622	4.43896	6.71266
Gglean012956	0	0	0	0
Gglean012957	31.16672205	30.55991367	26.0566384	25.46787713
Gglean012958	57.2434	62.5634	45.93109	44.862425
Gglean012959	57.2434	62.5634	45.93109	44.862425
Gglean012960	0	0	0	0
Gglean012961	23.84263916	22.78385402	23.64616581	22.81453364
Gglean012962	0	0	0	0
Gglean012963	39.910995	56.34362	22.842928	21.656631
Gglean012964	39.910995	56.34362	22.842928	21.656631
Gglean012965	0.0591014	0.182787	0	0
Gglean012966	0.345018	0.25372	0.322101	0.0955719
Gglean012967	6.400411051	6.491299287	5.143119615	5.09619596
Gglean012968	6.400411051	6.491299287	5.143119615	5.09619596
Gglean012969	553.362	463.971	470.189	729.286
Gglean012970	25.38402127	25.9273	45.915112	47.046882
Gglean012971	47.6118	47.7025	55.7386	99.3186
Gglean012972	12.74557	15.53661	11.71666	10.85511
Gglean012973	18.5124	20.297	33.9063	35.4514
Gglean012974	38.6127	38.2031	42.9733	38.9452
Gglean012975	0	0	0	0
Gglean012976	8.744850904	5.3656648	10.23467083	11.14392246
Gglean012977	11.34842462	11.088645	13.919271	13.72382416
Gglean012978	0	1.58448	0	0
Gglean012979	38.15018551	35.37143	28.485135	28.19857

Gglean012980	38.15018551	35.37143	28.485135	28.19857
Gglean012981	38.15018551	35.37143	28.485135	28.19857
Gglean012982	0.513978004	0.215773152	0.517444776	0.456968404
Gglean012983	108.613	81.6935	116.555	133.685
Gglean012984	790.1467	611.0708	577.5075	859.7879
Gglean012985	86.8276	77.44988	58.16551	50.37601
Gglean012986	16.46616	13.8729	12.60934	12.89114
Gglean012987	91.3571	92.7266	38.8215	40.1001
Gglean012988	12.46428	11.40172	18.48134	18.21636
Gglean012989	339.8465046	340.392139	312.9150259	360.0362149
Gglean012990	75.30670554	81.2943038	87.72970043	89.640492
Gglean012991	16.78666	16.77367	16.41199	13.60291
Gglean012992	16.78666	16.77367	16.41199	13.60291
Gglean012993	0	0	0	0.0554791
Gglean012994	0.609239	0.253242	0.396985	0.354298
Gglean012995	79.81598	84.75915	70.18938	68.17688569
Gglean012996	141.178	109.056	24.951	12.1398
Gglean012997	32.0629	30.529	36.17342	34.49892
Gglean012998	0	0	0.0889427	0
Gglean012999	161.8632	143.6042	115.7991	234.9424
Gglean013000	7.965537072	7.327833947	12.0552011	14.65175673
Gglean013001	0	0	0	0
Gglean013002	17.0429	15.52324	9.807463272	10.744506
Gglean013003	25.50833	27.53315	13.23866	12.71863
Gglean013004	11.463755	12.715092	11.490846	11.02339
Gglean013005	111.651752	113.4623691	197.8576292	231.674602
Gglean013006	46.57356916	45.74638247	32.456999	28.75071165
Gglean013007	6.40615	7.4354	10.36535	11.66342
Gglean013008	24.2741	21.52041	22.03282	20.74433
Gglean013009	72.70935	70.71967	89.75355	118.43453
Gglean013010	3.57144	9.48883	0.991894	0
Gglean013011	606.7824	377.71799	524.342093	626.3428757
Gglean013012	606.7824	377.71799	524.342093	626.3428757
Gglean013013	0.642513	0.295422	0.344836	0.620088
Gglean013014	23.33322	21.70659	16.70416	19.65204
Gglean013015	30.90455	31.31851	28.05846	26.36089
Gglean013016	6.205774	7.064622	4.59588931	4.533781142
Gglean013017	111.474	116.112	157.879	193.394
Gglean013018	0	0	0	0
Gglean013019	3.08412	4.6646	3.53592	2.31955
Gglean013020	68.19550123	75.57140304	71.20170537	55.80550272
Gglean013021	0.5671101	0.6166106	0.4515487	0.5537377
Gglean013022	0.5671101	0.6166106	0.4515487	0.5537377
Gglean013023	0	0	0	0

Gglean013024	0	0	0	0
Gglean013025	0.947477	0.755503	1.431735524	1.785142
Gglean013026	3.102979	3.304253	2.514285	3.138938
Gglean013027	0	0	0	0
Gglean013028	0	0	0	0
Gglean013029	0.168406	0	0	0
Gglean013030	0	0	0	0
Gglean013031	0.44374	0.130753	0.516359	0.244728
Gglean013032	0	0	0	0
Gglean013033	0	0	0.0766138	0
Gglean013034	0	0	0	0
Gglean013035	0	0	0	0
Gglean013036	1.4363	1.36993	2.56757	3.05827
Gglean013037	0	0	0	0
Gglean013038	0.243848	0.519745	0.200625	0
Gglean013039	0.0769082	0.1049386	0.062029	0
Gglean013040	41.41045	44.61862	40.01393	40.63485
Gglean013041	53.39693	65.06231	73.0767686	97.46369
Gglean013042	53.39693	65.06231	73.0767686	97.46369
Gglean013043	15.21631	14.84378	21.687797	24.31135
Gglean013044	4.55994	5.67006	2.730287024	1.951050079
Gglean013045	0	0	0	0
Gglean013046	58.16130871	62.5208368	48.19634039	42.9829398
Gglean013047	20.74528007	21.72805007	18.9882357	18.9172203
Gglean013048	0	0	0	0
Gglean013049	0	0	0	0.231798808
Gglean013050	0	0	0	0
Gglean013051	0	0	0	0
Gglean013052	21.27594	17.892992	11.79143879	12.4284697
Gglean013053	17.620727	17.345537	22.543158	22.834371
Gglean013054	9.23006	9.731528	9.959989	10.85666
Gglean013055	316.4158838	346.3823213	293.7100374	308.53248
Gglean013056	0.540958	0.422602	0	0
Gglean013057	29.84508001	30.56729455	34.29085012	32.55482912
Gglean013058	0.0164604	2.25E-13	4.39E-08	0.195192
Gglean013059	0.303788908	0.412339202	0.506089382	0.546751693
Gglean013060	18.21543	13.21043	9.57462	8.26511
Gglean013061	74.75404	79.108815	97.72003575	95.08295007
Gglean013062	74.75404	79.108815	97.72003575	95.08295007
Gglean013063	1.430484617	2.818087126	2.896558437	3.360218312
Gglean013064	1.430484617	2.818087126	2.896558437	3.360218312
Gglean013065	0	0	0	0
Gglean013066	4.83992	4.06166	5.74253	7.28095838
Gglean013067	0.0534302	0.108968	0.0216041	0

Gglean013068	0	0	0	0
Gglean013069	3.447698871	3.037123439	5.281987531	4.566739
Gglean013070	37.54910666	37.025702	24.12436352	22.85785644
Gglean013071	0	0.640904	0	0
Gglean013072	20.94109	18.2695	15.92397	17.67507
Gglean013073	11.2407	14.1316	11.8187686	11.83994
Gglean013074	21.41526	31.4053	29.1712	31.8111
Gglean013075	67.76598093	82.05329864	42.72459956	37.48734253
Gglean013076	67.76598093	82.05329864	42.72459956	37.48734253
Gglean013077	67.76598093	82.05329864	42.72459956	37.48734253
Gglean013078	5.40973	2.34609	2.088100081	6.059320042
Gglean013079	0	0	0	0
Gglean013080	19.37502	20.13564	18.79446	17.17895
Gglean013081	9.171991485	9.29685721	7.582864006	7.777092827
Gglean013082	9.171991485	9.29685721	7.582864006	7.777092827
Gglean013083	160.19465	151.3151	39.5913	32.064852
Gglean013084	28.8666	27.31493	26.107	28.61948
Gglean013085	0	0	0	0
Gglean013086	6.26141	6.95724	9.32886	11.14403284
Gglean013087	1.03908	0.280762	1.07847	0.772396
Gglean013088	0	0	0.115353	0
Gglean013089	3.90E-115	0.31233	3.29E-114	0.372473
Gglean013090	14.23491	13.57504	5.99357	6.2964
Gglean013091	43.860081	43.198548	8.795267	6.518449
Gglean013092	0.340171	0.7287821	0.277598	0
Gglean013093	41.8767	44.4536	25.1675	21.50739
Gglean013094	18.4151	18.4521	12.8238	12.3643
Gglean013095	16.41284996	15.63732535	23.53389815	26.55614024
Gglean013096	58.12423	66.31632	15.330938	9.966719
Gglean013097	9.73414	11.2698	2.57556	0.279254
Gglean013098	101.579072	100.085428	72.243932	61.247949
Gglean013099	0	0	0	0
Gglean013100	88.31659	80.138355	67.228683	61.230973
Gglean013101	31.5059942	28.82726272	42.394097	41.0332126
Gglean013102	37.68805273	38.57036112	36.77027693	37.56198321
Gglean013103	170.4900068	132.2780078	266.2420063	353.0610083
Gglean013104	17.719099	17.0043152	23.54421635	29.91369197
Gglean013105	11.74609	15.88846	11.0237	10.46818
Gglean013106	63.3981	66.8668	56.7064	52.3723
Gglean013107	0	0	0	0
Gglean013108	0	0	0	0
Gglean013109	0	0.10322	0	0.0962498
Gglean013110	3.206221	4.00278991	4.449453518	3.500108575
Gglean013111	0.29831454	0.337048636	0.051835036	0.122500401

Gglean013112	3.11182	2.58443	2.71696	2.74368
Gglean013113	1.047521069	1.099132157	1.9228101	1.787470603
Gglean013114	0.128994	0.217161	0.104269	0.0414815
Gglean013115	0	0	0	0
Gglean013116	0	0.039132079	0	0
Gglean013117	0.039937	0	0	0.0383417
Gglean013118	0	0	0	0
Gglean013119	0.0630403	0	0.103618	0
Gglean013120	0	0	0	0
Gglean013121	3.27558	3.39498	4.28631	3.78615
Gglean013122	29.31244	30.3024	40.03234	47.12853
Gglean013123	0.358367	0.123503	0	0.172707
Gglean013124	3.74966	8.78715	1.10691	1.29002
Gglean013125	0	0	0	0
Gglean013126	18.92564	19.77289	15.17112	14.95813
Gglean013127	0.824120929	0.703552747	0.55422666	0.76254454
Gglean013128	0	0	0	0
Gglean013129	0	0	0	0.186843
Gglean013130	0.612246505	0.63786605	0.4346202	0.6180037
Gglean013131	0	0	0	0
Gglean013132	20.75312	22.20759141	19.40226519	18.08258381
Gglean013133	48.31934713	50.31720603	82.176709	87.73087906
Gglean013134	4.0391542	5.740489256	5.544676188	7.914995445
Gglean013135	4.0391542	5.740489256	5.544676188	7.914995445
Gglean013136	418.106721	459.873952	665.301008	810.88873
Gglean013137	418.106721	459.873952	665.301008	810.88873
Gglean013138	418.106721	459.873952	665.301008	810.88873
Gglean013139	0.398698064	0.391070255	0.107407372	0.659686107
Gglean013140	0	0	0	0
Gglean013141	0.176172	0	0.143456	0.169958
Gglean013142	0.10691	0.0369875	0.115818	0.0342331
Gglean013143	21.04400106	18.7561	26.07151014	34.20690058
Gglean013144	0.0908082	0.187026	0.0732573	0.0867041
Gglean013145	0.29592864	0.268929475	0.660715785	0.379027658
Gglean013146	0.29592864	0.268929475	0.660715785	0.379027658
Gglean013147	0.0700814	0	0	0.100799
Gglean013148	0	0	0.0272993	0
Gglean013149	0	0	0	0
Gglean013150	0	0	0	0
Gglean013151	0	0.00970903	0	0.00904057
Gglean013152	29.54500601	26.83834676	25.7890294	25.66193225
Gglean013153	163.2746483	169.6902937	138.56474	125.7134629
Gglean013154	31.52951	32.72162	29.74727	29.66918
Gglean013155	0	0	0	0

Gglean013156	0.50841	1.38161	1.23981	1.23086
Gglean013157	0.0507519	0.064633644	0.051091282	0
Gglean013158	0	0	0	0
Gglean013159	0.0346803	0.0355764	0.0840398	0
Gglean013160	13.555773	13.741863	13.402427	14.853959
Gglean013161	0	0	0	0
Gglean013162	10.13846669	9.720931428	11.23976887	10.77392488
Gglean013163	35.441697	36.40882	32.166293	32.18349
Gglean013164	38.7576	39.7821	24.3922	20.66766
Gglean013165	1.210273	1.320792	1.731343	3.275939
Gglean013166	1.210273	1.320792	1.731343	3.275939
Gglean013167	0	0	0	0
Gglean013168	0	0	0	0
Gglean013169	0	0	0	0
Gglean013170	14.38821921	14.24401088	15.86500799	23.54777849
Gglean013171	14.38821921	14.24401088	15.86500799	23.54777849
Gglean013172	0	0	0.395642	0
Gglean013173	42.82164593	48.48044085	41.59036535	39.097037
Gglean013174	1.02269157	0.390036	0.897496592	1.089312403
Gglean013175	6.39217	6.09013	6.633901662	17.86036
Gglean013176	16.69121	15.98134	27.28727	31.40117
Gglean013177	19.3651081	19.68834066	26.34861975	31.5850468
Gglean013178	189.6606873	196.3234796	295.2587589	302.326523
Gglean013179	0	0	0.106008	0
Gglean013180	0	0	0	0
Gglean013181	0	0	0	0
Gglean013182	8.581728418	7.862640371	15.404203	15.89061723
Gglean013183	0	0	0	0
Gglean013184	0	0	0.0441173	0.0520485
Gglean013185	0	0	0	0.050758848
Gglean013186	0	0	0	0
Gglean013187	7.19467078	10.01493	13.84592057	15.805275
Gglean013188	0	0	0	0
Gglean013189	0	0	0	0
Gglean013190	0	0	0.274307	0
Gglean013191	7.18629	6.08484	10.4248	11.0211
Gglean013192	0	0.297488	0.680985	0.270639
Gglean013193	0	0	0	0
Gglean013194	0	0	0.0626193	0
Gglean013195	0	0	0	0
Gglean013196	0	0	0.132552	0
Gglean013197	0.253988	7.20E-06	8.52E-05	0.310794
Gglean013198	0.000399462	0.000218825	1.11705	5.71E-05
Gglean013199	30.55622	28.36809	18.39637	14.47113

Gglean013200	0.581459	0.484782	0.568318	0.67123
Gglean013201	0	0	0	0
Gglean013202	16.48022993	17.14378676	24.57837204	26.33151007
Gglean013203	0.0520619	0	0	0
Gglean013204	41.08057267	44.286838	40.6730771	46.50647979
Gglean013205	1.5351	1.86059	1.93508	2.86886
Gglean013206	0	0.192127	0	0
Gglean013207	1.350317698	1.0458342	1.350408377	2.08565546
Gglean013208	39.32708903	39.10640911	25.11359394	25.7553351
Gglean013209	4.00415	3.55645	3.12109	3.088517
Gglean013210	4.00415	3.55645	3.12109	3.088517
Gglean013211	39.32708903	39.10640911	25.11359394	25.7553351
Gglean013212	44.86425398	40.92409696	41.920332	43.471844
Gglean013213	3.54436	4.77396	3.6469	3.07883
Gglean013214	28.42775	30.72571	22.30698	25.24442
Gglean013215	402.346399	334.416683	305.8544471	316.40813
Gglean013216	402.346399	334.416683	305.8544471	316.40813
Gglean013217	84.90066835	85.27291	85.57791144	95.9751067
Gglean013218	27.1895	29.6895	37.0971	45.2413
Gglean013219	1.126145	0.718756744	0.856986209	0.8800094
Gglean013220	28.989	24.5562	16.1671	15.599
Gglean013221	0.0952802	0.703246	0.0699049	1.50968
Gglean013222	4.40535	5.67465	1.50615	1.6479
Gglean013223	6.02503	5.81233	3.89969	3.7346
Gglean013224	1.855102173	2.322254	2.521298	3.782680439
Gglean013225	0	0	0	0.207915222
Gglean013226	0	0	0	0.207915222
Gglean013227	1.664843136	0.841832955	2.140943451	1.235705054
Gglean013228	1.664843136	0.841832955	2.140943451	1.235705054
Gglean013229	1.664843136	0.841832955	2.140943451	1.235705054
Gglean013230	115.868	128.421	77.4207	72.8817
Gglean013231	28.08319	18.81721	24.82462	25.47754
Gglean013232	98.6229	120.5277	38.61611	37.48432
Gglean013233	1.3968	0.831631	0.647254	0.576734
Gglean013234	13.4438161	13.03688882	19.6858062	23.10073455
Gglean013235	0.168956	2.13E-05	0.273727	0.0782474
Gglean013236	53.255	56.7492	28.08599	25.51459
Gglean013237	3.40E-18	1.42123	2.77E-11	0.550584
Gglean013238	65.8726	65.6675	48.31172	48.36862
Gglean013239	12.15361	13.04964385	7.27866145	6.440815757
Gglean013240	0	0	0	0
Gglean013241	20.9366	19.0429	16.7545	16.5083
Gglean013242	130.08922	117.30651	145.89431	164.77537
Gglean013243	130.08922	117.30651	145.89431	164.77537

Gglean013244	0	0	0.14918	0
Gglean013245	0	0	0	0
Gglean013246	0.178969	4.37E-05	4.72E-06	0.39609
Gglean013247	0	0	0	0
Gglean013248	4.30598505	2.65560695	3.772318	4.288336
Gglean013249	0.906850048	1.491711324	2.029611598	2.154192854
Gglean013250	0.213434	0.236663	0.257913	0.591324
Gglean013251	0.1606157	0.236553962	0.421293357	0.308746491
Gglean013252	7.78327	4.867543	8.4671	12.3402
Gglean013253	17.75351	19.90406	12.61715823	11.61079
Gglean013254	47.6632	46.998	30.3651	29.533
Gglean013255	39.88015	45.68854	49.89524	51.54425
Gglean013256	2.8715083	2.733342627	5.7602228	6.31294076
Gglean013257	2.8715083	2.733342627	5.7602228	6.31294076
Gglean013258	16.59033842	15.98661151	11.540888	10.68572263
Gglean013259	42.39538	57.65825	50.48772	56.29936
Gglean013260	38.24333	39.124882	13.62697	14.2623
Gglean013261	78.89083	88.3752	56.66772	49.56542
Gglean013262	38.24333	39.124882	13.62697	14.2623
Gglean013263	27.25839266	36.56725327	50.15379	52.344467
Gglean013264	27.25839266	36.56725327	50.15379	52.344467
Gglean013265	27.25839266	36.56725327	50.15379	52.344467
Gglean013266	30.52244667	32.7735667	29.18929505	28.5616623
Gglean013267	30.52244667	32.7735667	29.18929505	28.5616623
Gglean013268	0.857133	2.90199	0.719075	0
Gglean013269	10.60706466	11.395777	9.493365622	10.74943008
Gglean013270	0	0	0	0
Gglean013271	0.461783	0.194679	0.680375	0.2521126
Gglean013272	24.4773	6.2544	20.1674	15.933
Gglean013273	35.09292276	43.770573	38.66217	38.5678
Gglean013274	35.09292276	43.770573	38.66217	38.5678
Gglean013275	9.270263664	7.318472	12.71754862	16.213485
Gglean013276	17.28408	31.02339	36.1219	43.4094115
Gglean013277	52.2954	50.2881	22.0777	17.9406
Gglean013278	396.451	400.296	129.251	123.258
Gglean013279	6.198111254	8.557723671	14.67040771	14.23575044
Gglean013280	0	0	0	0
Gglean013281	0	0	0	0
Gglean013282	0	0	0	0
Gglean013283	0	0	0	0
Gglean013284	0.915697	0.581878	0.150388	0.443256
Gglean013285	0.776716	1.1541	1.40442	1.2575
Gglean013286	11.02884875	10.98033897	10.100535	9.2842663
Gglean013287	38.66572037	38.13397984	32.43285181	32.9932299

Gglean013288	22.16663582	21.8316911	17.55346674	16.47389177
Gglean013289	58.11192	54.02604	76.68278	106.98644
Gglean013290	44.347459	42.100565	33.30064517	30.721484
Gglean013291	44.347459	42.100565	33.30064517	30.721484
Gglean013292	62.21295	65.7464	70.3762	67.536
Gglean013293	2.070110224	2.34149022	2.137390094	2.811710004
Gglean013294	2.8889	2.84274	3.60951	3.58581
Gglean013295	8.16305	6.45614	9.01813	8.50906
Gglean013296	19.22244887	19.54806389	9.533720087	9.476267126
Gglean013297	1.189064301	0.937250079	1.428717173	1.112508131
Gglean013298	9.227376	9.24322	10.16761	8.854086
Gglean013299	24.55129843	24.39092503	21.77486412	23.41787053
Gglean013300	24.55129843	24.39092503	21.77486412	23.41787053
Gglean013301	0.102420199	1.2401455	0.248750981	1.067484028
Gglean013302	0.102420199	1.2401455	0.248750981	1.067484028
Gglean013303	364.6625	315.6622	296.6096	352.1617
Gglean013304	25.65014	30.0288	15.6283952	13.0220941
Gglean013305	47.0532	49.64938	39.50709	32.30970001
Gglean013306	47.0532	49.64938	39.50709	32.30970001
Gglean013307	77.845648	90.818149	54.5771003	52.7695
Gglean013308	4.95003	2.37172	4.90388	4.28847
Gglean013309	0.381665	0.268753	0.720399	0.613735
Gglean013310	1.61888	1.20639	2.82119	2.89792
Gglean013311	17.4520209	19.64574731	19.19030043	17.58309748
Gglean013312	17.4520209	19.64574731	19.19030043	17.58309748
Gglean013313	2.19E-06	1.33E-06	7.41E-06	0.0935577
Gglean013314	0.0984207	0.0142751	0.0220333	7.52E-05
Gglean013315	0	0	0	0
Gglean013316	0	0	0	0
Gglean013317	0	0	0	0
Gglean013318	0.025697	0	0	0
Gglean013319	0	0	0.157515	0
Gglean013320	0.322171	0	0	0
Gglean013321	2.01686	1.09066	2.81403	3.6505
Gglean013322	1.540250554	1.514720737	1.861240177	1.8370807
Gglean013323	0	0	0	0
Gglean013324	0.147891	0.190506	0.178465	0.10573
Gglean013325	0	0	0	0
Gglean013326	0	0	0.100978	0.241449
Gglean013327	50.27519	55.72722	77.44431	101.94359
Gglean013328	0.6728886	0.339482	0.141490262	0.423214
Gglean013329	289.9583118	272.774636	304.5242587	366.3691521
Gglean013330	5.53753	8.37087	7.44093	10.1816
Gglean013331	69.81845804	71.20325218	84.07900753	77.9302513

Gglean013332	0	1.53361	0.237105	0.281895
Gglean013333	27.14732687	29.50528081	20.05220836	17.7832578
Gglean013334	35.42562947	31.81316792	18.36942424	16.37920335
Gglean013335	35.42562947	31.81316792	18.36942424	16.37920335
Gglean013336	3.448977	2.46199	3.962919	2.38495
Gglean013337	9.54386	10.1215	12.5944	15.5452
Gglean013338	22.07856	22.65045892	21.03446989	26.0210008
Gglean013339	21.08303248	21.89077618	18.259994	17.08009738
Gglean013340	0.518019	0.994557	1.46363	1.74136
Gglean013341	98.509	94.2809	108.6944	98.9639
Gglean013342	27.80871548	29.59861451	26.02908746	23.90826171
Gglean013343	27.80871548	29.59861451	26.02908746	23.90826171
Gglean013344	38.12874172	41.17655947	54.9263438	61.92156502
Gglean013345	0	0	0	0
Gglean013346	38.12874172	41.17655947	54.9263438	61.92156502
Gglean013347	38.12874172	41.17655947	54.9263438	61.92156502
Gglean013348	7.3621513	8.208384214	10.27294038	13.66765394
Gglean013349	14.0371	13.549	7.53398	7.06088
Gglean013350	26.5768	28.8654	16.74646	16.13687
Gglean013351	8.53228	8.24293	7.06159	6.86783
Gglean013352	422.67	373.008	130.894	103.161
Gglean013353	68.1056	64.0384	30.6203	24.4813
Gglean013354	61.6423	78.17644	45.97374	43.80807
Gglean013355	0	0	0	0
Gglean013356	0	0.100727	0.0787654	0
Gglean013357	0.0636511	0	0	0
Gglean013358	0	0	0.100127	0
Gglean013359	15.7933	15.81589	11.62633	12.37979
Gglean013360	0.650231	0.466778573	0.571210307	0.293972
Gglean013361	1.06359	1.80631	2.58733	2.11529
Gglean013362	0	0	0	0
Gglean013363	0.278986	0.863144	0.4721018	0.3747551
Gglean013364	61.4881	61.5861	27.2151	21.2279
Gglean013365	1.598206176	1.536464737	1.421399533	1.070113534
Gglean013366	1.598206176	1.536464737	1.421399533	1.070113534
Gglean013367	18.37248519	16.83671758	25.4807102	25.18502708
Gglean013368	0	0	0	0
Gglean013369	18.37248519	16.83671758	25.4807102	25.18502708
Gglean013370	2.826956	3.222152814	3.689654234	6.44943
Gglean013371	0	0	0	0
Gglean013372	55.31745	60.13123	81.1747227	116.2490667
Gglean013373	0.889206009	1.563373532	1.006465001	0.906589398
Gglean013374	1.942475	2.5127472	3.078198876	1.595067437
Gglean013375	62.71297	67.75991	57.60734	59.91499

Gglean013376	62.71297	67.75991	57.60734	59.91499
Gglean013377	45.82852224	46.78427	55.946259	60.04272
Gglean013378	0	0	0	0
Gglean013379	0	0	0	0
Gglean013380	0	0	0	0
Gglean013381	0	0	0	0
Gglean013382	0	0	0	0
Gglean013383	0	0	0	0
Gglean013384	0.108741	0	0	0
Gglean013385	0	0	0	0
Gglean013386	47.18648019	47.63368119	49.24872908	46.62182631
Gglean013387	39.49321	39.1243	31.09579685	30.414967
Gglean013388	111.54689	108.621	95.9728	94.776441
Gglean013389	20.36897	23.63015	5.610134	5.146965
Gglean013390	22.41256163	23.4135386	23.56124262	23.93605837
Gglean013391	0	0	0	0
Gglean013392	4.027800094	5.359160138	2.379400992	1.543990299
Gglean013393	43.479605	40.65744	22.75623711	21.96695668
Gglean013394	43.479605	40.65744	22.75623711	21.96695668
Gglean013395	46.47978523	45.084665	26.56094578	18.06579607
Gglean013396	0	0	0	0
Gglean013397	66.96047	67.880381	44.125846	47.42148378
Gglean013398	0.699033	0	0	1.37954
Gglean013399	42.18848	45.53691	23.406274	21.758512
Gglean013400	11.915532	9.57850547	11.49893467	12.598564
Gglean013401	144.6825	143.7428	101.1903	101.144
Gglean013402	27.0559	31.962	17.157	7.1712
Gglean013403	40.57766	43.199834	71.957564	66.384771
Gglean013404	3.19933	2.215679088	1.2847966	1.086291961
Gglean013405	35.67625	32.35613	26.18088	26.94437
Gglean013406	0	0	0.09389017	0
Gglean013407	6.68182309	7.998190414	11.15010263	11.48581634
Gglean013408	31.13430932	30.43359355	63.82492626	74.52027835
Gglean013409	304.3857	322.7203	392.6148	597.166
Gglean013410	0.30054	0.930585	0.103555	0.245066
Gglean013411	0	0.858812	0	0
Gglean013412	1.06961	1.38344	0.972777	2.25908
Gglean013413	0	0	0	0
Gglean013414	0	0	0	0
Gglean013415	0	0	0	0
Gglean013416	0.086028	0.132646	0	0.0819719
Gglean013417	0	0	0	0
Gglean013418	0.991561671	0.742583903	0.937936208	0.699582001
Gglean013419	0.991561671	0.742583903	0.937936208	0.699582001

Gglean013420	0	0	0	0
Gglean013421	0	0	0	0
Gglean013422	0	0	0	0
Gglean013423	1.1946	1.44244	1.38	2.04755
Gglean013424	13.36387349	12.10634456	15.76717705	18.14055102
Gglean013425	0.163488	0	0	0
Gglean013426	33.501352	34.842746	24.2023	22.27781
Gglean013427	36.53598	32.07109	28.50284	25.08486
Gglean013428	0.07496516	0.618962027	0.374833192	0.505664837
Gglean013429	0.0407877	0.0841194	0	0.117195
Gglean013430	0.599298	0	0.350615	0.331045
Gglean013431	0	0	0	0.137199
Gglean013432	0	0	0	0
Gglean013433	0	0.106788	0	0
Gglean013434	0	0	0	0
Gglean013435	0	0	0	0
Gglean013436	19.0393735	16.77468108	15.4270662	17.33301712
Gglean013437	45.720884	80.89269888	71.049891	108.0708603
Gglean013438	45.067329	46.441536	41.010289	42.658236
Gglean013439	2.51901	0.595853	2.51105	2.74509
Gglean013440	28.44021814	33.55367099	16.47954343	13.35445097
Gglean013441	1.863344647	2.224664	2.27553382	7.43029
Gglean013442	1.863344647	2.224664	2.27553382	7.43029
Gglean013443	2.461917605	2.277066	0.871786523	1.132746424
Gglean013444	0	0	0	0
Gglean013445	30.63671748	29.68524124	29.87216065	26.36162509
Gglean013446	0	0	0.403617	0
Gglean013447	33.12906	29.00102465	22.4462588	19.51424
Gglean013448	1.04632	2.724000387	1.1723008	2.78437013
Gglean013449	0	0	0	0
Gglean013450	200.948632	165.41167	117.6780162	115.4630137
Gglean013451	0.256584	0.561332	0.495795	0.982399
Gglean013452	0.256584	0.561332	0.495795	0.982399
Gglean013453	28.55187559	27.41966188	44.27659358	51.12256518
Gglean013454	28.55187559	27.41966188	44.27659358	51.12256518
Gglean013455	28.55187559	27.41966188	44.27659358	51.12256518
Gglean013456	3.022174808	2.265328341	1.854334	1.440677899
Gglean013457	540.515	549.762	633.905	811.802
Gglean013458	0.195753	0.407675	0.481401157	0.568549708
Gglean013459	0	0	0	0
Gglean013460	0.225288801	0.0782188	0.1359158	0.318799
Gglean013461	0	0	0.13054	0
Gglean013462	32.3589	31.7503	36.8767	45.1052
Gglean013463	0	0	0	0

Gglean013464	1.906655582	2.611528348	2.256070792	3.706755859
Gglean013465	11.00067103	13.0006641	16.68188101	17.5383186
Gglean013466	11.00067103	13.0006641	16.68188101	17.5383186
Gglean013467	14.046422	13.44918866	21.79545	23.87057923
Gglean013468	3.43366	4.40691	4.90654	5.00431
Gglean013469	0	0	0	0
Gglean013470	9.96559	10.33852	13.6517	15.30638107
Gglean013471	7.39761	9.46544	12.5858	14.0376
Gglean013472	18.307966	18.25886009	27.57407	30.4104
Gglean013473	47.104471	50.95407	39.67211391	35.389133
Gglean013474	47.104471	50.95407	39.67211391	35.389133
Gglean013475	47.104471	50.95407	39.67211391	35.389133
Gglean013476	47.104471	50.95407	39.67211391	35.389133
Gglean013477	0	0	2.07652	0
Gglean013478	0	0	0	2.45773
Gglean013479	0.970249	0.887332	1.322500486	1.438441049
Gglean013480	0	0.165429	0.382142	0
Gglean013481	0	0	0.236508	0
Gglean013482	877.472	825.502	1145.04	1541.21
Gglean013483	3.32734	0	13.4887	6.35896
Gglean013484	4.167108	6.785586	25.283897	13.069327
Gglean013485	0	0	0	0
Gglean013486	30.0688	25.8439	33.6421	41.9268
Gglean013487	0.749785738	1.704399148	1.519080007	2.116363211
Gglean013488	0.197166283	0.498761	0.416896761	0.31984368
Gglean013489	1.68E-07	1.80E-07	1.07E-06	2.10E-06
Gglean013490	0.105148503	0	0.196938392	0.069984698
Gglean013491	0.105148503	0	0.196938392	0.069984698
Gglean013492	0.039392212	0.026763796	0.074002463	0.105684891
Gglean013493	0.275687233	0.42870728	0.698011216	0.563461838
Gglean013494	0.366281431	0.207759107	0.740437592	0.3940588
Gglean013495	0.366281431	0.207759107	0.740437592	0.3940588
Gglean013496	0.45046072	1.2001578	1.050587124	1.146247864
Gglean013497	1.6824647	1.8467187	1.976623	2.567906
Gglean013498	0	0	0	0
Gglean013499	0	0	0	0
Gglean013500	5.46031	3.3284	5.59197	5.35469
Gglean013501	7.721837093	5.9275581	7.195089	10.26748129
Gglean013502	7.721837093	5.9275581	7.195089	10.26748129
Gglean013503	7.721837093	5.9275581	7.195089	10.26748129
Gglean013504	17.31416	16.525231	16.961947	14.213
Gglean013505	0	0	0	0.141515
Gglean013506	40.54404	38.40218	24.48651	21.734341
Gglean013507	6.552188	6.324418	9.125725	10.103508

Gglean013508	9.45638	6.325074	3.381365	6.380125
Gglean013509	9.45638	6.325074	3.381365	6.380125
Gglean013510	0.614309	2.7429	0	0
Gglean013511	0	0.0252355	0	0
Gglean013512	0.177715	0.183243	0.14427	0.171613
Gglean013513	0	0	0	0
Gglean013514	0	0	0	0
Gglean013515	0	0	0	0
Gglean013516	0.422605	0.791162	0.951318	1.37715
Gglean013517	0	0	0	0
Gglean013518	52.03402	66.79730305	81.579342	95.89210286
Gglean013519	43.94453	42.49269	29.25538	32.03399
Gglean013520	43.94453	42.49269	29.25538	32.03399
Gglean013521	1.39539	1.384545	2.5737802	2.18358
Gglean013522	5.177782	14.19379	16.31015	4.35672
Gglean013523	0.357055	0.368516	0	0
Gglean013524	0.08439	0.0870448	0	0.0811992
Gglean013525	0	0	0	0.110907
Gglean013526	0	0	0	0
Gglean013527	0.175165573	0.230912617	0.327982093	0.345178292
Gglean013528	0.128143	0	0.103779	0.306922
Gglean013529	33.54883	38.71653	33.46401	32.87708
Gglean013530	33.54883	38.71653	33.46401	32.87708
Gglean013531	0	0	0	0
Gglean013532	0	0	0	0
Gglean013533	0	0	0	0
Gglean013534	0	0	0	0
Gglean013535	0	0.109059	0.167824	0
Gglean013536	0	0	0	0
Gglean013537	0	0.102623	0	0
Gglean013538	0	0.212086	0	0
Gglean013539	0	0	0	0
Gglean013540	47.28105	40.99623	61.38487	60.4347
Gglean013541	0.0479871	0.049276	0.0771107	0
Gglean013542	0	0	0	0
Gglean013543	1.31657	1.22728	0.767829	0.649233
Gglean013544	0	0	0	0
Gglean013545	0.350388	4.84E-05	0.000817868	0.000165483
Gglean013546	0	0	0	0
Gglean013547	0	0	0	0
Gglean013548	0	0	0	0
Gglean013549	41.1698	45.9317	22.9467	20.7405
Gglean013550	0.307363	0.330577	0	0
Gglean013551	0.0453226	0.3422169	0.228473105	0.778555

Gglean013552	0.0453226	0.3422169	0.228473105	0.778555
Gglean013553	117.3982641	126.8207553	146.4971438	149.8589924
Gglean013554	117.3982641	126.8207553	146.4971438	149.8589924
Gglean013555	117.3982641	126.8207553	146.4971438	149.8589924
Gglean013556	9.30972	8.94097	9.70916	10.7053
Gglean013557	1.513459951	1.344180918	1.390408	1.912789
Gglean013558	2.30131	1.95715	2.40488	2.40231
Gglean013559	2.21941	13.9573	8.69397	6.72636
Gglean013560	9.8431	9.92121	11.1309	11.0655
Gglean013561	299.1892428	261.365082	434.512586	444.756734
Gglean013562	299.1892428	261.365082	434.512586	444.756734
Gglean013563	299.1892428	261.365082	434.512586	444.756734
Gglean013564	11.0189	10.1196	8.76467	11.0311
Gglean013565	13.5726	14.0356	21.0917	7.19778
Gglean013566	8.382553	5.596891	5.012348	5.054544
Gglean013567	86.34527195	87.96741	49.526784	47.07942405
Gglean013568	86.34527195	87.96741	49.526784	47.07942405
Gglean013569	1.02E-06	1.87E-06	4.45E-06	1.67E-07
Gglean013570	0	0	0	0
Gglean013571	0	0	0	0
Gglean013572	3.137847748	3.516622406	3.314378423	6.339159
Gglean013573	3.137847748	3.516622406	3.314378423	6.339159
Gglean013574	4.020312	3.024150254	2.49097466	1.616681508
Gglean013575	2.991302244	4.10505	8.450726414	4.91545
Gglean013576	0.027074897	0.083958749	0.0218701	0.025777748
Gglean013577	0.428302244	0.446254481	0.103041705	0.342338508
Gglean013578	15.608636	9.177676	21.409295	27.29319
Gglean013579	0.150364236	0	0.061195505	0
Gglean013580	10.52401	10.11228	12.4983	12.27739
Gglean013581	0	0	0.10679	0
Gglean013582	2.66388	3.94218	5.22073	6.23002
Gglean013583	0	0	0	0
Gglean013584	0.149283	0.202843	0.160849	0.143122
Gglean013585	0	0	0	0
Gglean013586	5.77795	4.27681	3.82449	0.00312586
Gglean013587	8.790653677	7.000390645	12.00030431	15.10154379
Gglean013588	0.0346562	0	0	0
Gglean013589	0.0346562	0	0	0
Gglean013590	0	0	0	0
Gglean013591	25.8452838	28.93720545	31.547934	34.19635173
Gglean013592	91.83002	119.21687	70.4762	79.55025
Gglean013593	2.31E-07	5.90E-05	1.96E-05	1.135
Gglean013594	0	0	0	0
Gglean013595	0	0	4.60957	5.47509

Gglean013596	1.67704	0.791838	2.20994	2.50605
Gglean013597	41.18107455	40.12174903	43.11391299	42.4749018
Gglean013598	41.18107455	40.12174903	43.11391299	42.4749018
Gglean013599	33.9901106	32.69112935	36.93029742	42.08482334
Gglean013600	497.47184	479.7313034	509.72344	443.040513
Gglean013601	0.980502	0.8189734	0.641374	0.374342
Gglean013602	20.243101	18.31252985	25.65573187	25.74900515
Gglean013603	54.14982219	52.52873015	114.9017412	114.006126
Gglean013604	34.791	33.8235	31.4155	30.0324
Gglean013605	10.6556	4.0368	3.30691	3.09021
Gglean013606	10.7345	6.282463567	7.02627	9.25347
Gglean013607	17.1543	14.251	11.574	11.3336
Gglean013608	156.544567	145.77411	108.3453921	91.43599117
Gglean013609	156.544567	145.77411	108.3453921	91.43599117
Gglean013610	156.544567	145.77411	108.3453921	91.43599117
Gglean013611	99.5048	104.287	42.6603	35.4939
Gglean013612	0	0	0	0
Gglean013613	0	0	0	0
Gglean013614	1.224646616	2.370286352	1.874341562	1.058382253
Gglean013615	1.224646616	2.370286352	1.874341562	1.058382253
Gglean013616	49.68194256	51.76916433	39.21694307	37.4592612
Gglean013617	49.68194256	51.76916433	39.21694307	37.4592612
Gglean013618	17.25925	15.1358	21.09028	29.72317
Gglean013619	0	0	0	0
Gglean013620	0	0	0	0
Gglean013621	0.262639	0	0	0
Gglean013622	0	0	0	0
Gglean013623	0	0	0	0
Gglean013624	0	0	0	0
Gglean013625	0	0	0	0
Gglean013626	0	0	0	0
Gglean013627	1.69537	1.80311	1.0348	2.45733
Gglean013628	0.200311	0	0	0
Gglean013629	1.444958	0.984761	0.0860286	0
Gglean013630	1.97807	0	0	0
Gglean013631	0	0	0	0
Gglean013632	0	0	0	0
Gglean013633	0	0	0	0
Gglean013634	0	0	0	0
Gglean013635	0	0	0	0
Gglean013636	0	0	0	0
Gglean013637	0	0	0	0
Gglean013638	0	0	0	0
Gglean013639	0	0	0.056457	0

Gglean013640	0	0	0	0
Gglean013641	0	0	0	0
Gglean013642	0	0	0	0
Gglean013643	0.0813905	0	0.0330513	0.117143
Gglean013644	34.216894	31.4695417	48.09686	60.50871
Gglean013645	0	0.0606522	0.0476364	0.0562719
Gglean013646	0	0	0	0
Gglean013647	0	0	0	0
Gglean013648	0	0	0	0
Gglean013649	0	0	0	0
Gglean013650	3.56817021	4.945022	4.061102	3.877682555
Gglean013651	2.492559	2.476853	3.675622	4.130318
Gglean013652	3.14889198	4.054504867	4.295266951	5.22208841
Gglean013653	12.15349421	11.55494032	10.80030101	7.319401537
Gglean013654	12.15349421	11.55494032	10.80030101	7.319401537
Gglean013655	0	0	0	0
Gglean013656	11.43029229	11.83470002	7.4722384	7.619759585
Gglean013657	0.638784	0.226083	1.92164	0.208024
Gglean013658	20.05686099	22.02171561	30.87440331	35.55001328
Gglean013659	20.05686099	22.02171561	30.87440331	35.55001328
Gglean013660	20.05686099	22.02171561	30.87440331	35.55001328
Gglean013661	57.029745	62.74075109	57.40304021	55.227956
Gglean013662	57.029745	62.74075109	57.40304021	55.227956
Gglean013663	37.85531	50.3361	48.94173	44.41391
Gglean013664	0.831223	0.526427	0.258116	0.239562
Gglean013665	2.005222545	1.455697773	2.822437221	4.91696004
Gglean013666	9.63086426	13.99783073	4.942930278	6.344387498
Gglean013667	0.1599615	0.199568268	0.088146011	0.104102961
Gglean013668	41.3363	39.8427	45.1748	47.8897
Gglean013669	1.43275	1.70032	1.16517	1.48855
Gglean013670	48.6721	50.66948	53.79878	56.3263
Gglean013671	0.394750052	0.861707	0.325573	0
Gglean013672	0.394750052	0.861707	0.325573	0
Gglean013673	0	0	0.376614	0
Gglean013674	24.033998	24.6576452	22.766141	28.00972
Gglean013675	24.033998	24.6576452	22.766141	28.00972
Gglean013676	23.50731	24.17282	19.78828	26.80826
Gglean013677	23.50731	24.17282	19.78828	26.80826
Gglean013678	10.405046	11.814045	6.5981	4.340487054
Gglean013679	67.23621	79.87077	60.41767	59.53047
Gglean013680	0.596995	0.511619	1.0537	1.53341
Gglean013681	6.18729	6.0047	7.76978	8.45781
Gglean013682	3.92968	4.286038	3.374505	6.529963
Gglean013683	32.05551504	31.66190463	35.13523965	34.91358043

Gglean013684	18.193919	18.66714	19.574863	23.771113
Gglean013685	0	0.307576	0.234886	0.281517
Gglean013686	40.91855278	34.109373	29.0321395	27.53313407
Gglean013687	72.51369558	81.92977	125.2047277	150.8496497
Gglean013688	13.6039566	15.786868	19.0054339	21.44998
Gglean013689	13.6039566	15.786868	19.0054339	21.44998
Gglean013690	6.429951	29.356906	15.13505	23.100926
Gglean013691	0.446371065	0.531842	0.647571289	0.450597482
Gglean013692	3.034783	2.619177326	2.652210569	16.554791
Gglean013693	3.034783	2.619177326	2.652210569	16.554791
Gglean013694	23.6506193	24.9352462	31.1474399	37.745706
Gglean013695	0.00431359	0.0562736	0.0450765	0.0204739
Gglean013696	0.0196504	0.0594053	0.0314568	0.0185957
Gglean013697	0.0469679	0.0158406	0.0880324	0.0297349
Gglean013698	0.1261192	0.094774722	0.1687957	0.1517227
Gglean013699	0.1261192	0.094774722	0.1687957	0.1517227
Gglean013700	36.46381938	44.0194572	30.13875442	26.03632891
Gglean013701	36.46381938	44.0194572	30.13875442	26.03632891
Gglean013702	49.609221	51.18317126	51.008547	47.93833035
Gglean013703	31.4507	32.0509	26.625	27.7725
Gglean013704	67.98997207	80.90577	37.9218077	32.32677
Gglean013705	103.403	103.708	124.167	151.955
Gglean013706	20.11452	25.10884	13.67529	11.85245
Gglean013707	1633.95836	1574.47239	1046.59069	972.79144
Gglean013708	23.7011748	24.34440584	16.87277943	16.841852
Gglean013709	23.7011748	24.34440584	16.87277943	16.841852
Gglean013710	42.4227	39.1635	25.9888	19.6473
Gglean013711	30.88636	32.83657	34.4221	32.73865
Gglean013712	25.855148	15.31111	2.76561	18.954689
Gglean013713	1.84453	1.74551	2.1976	2.16524
Gglean013714	43.765902	46.451922	57.932271	58.01688
Gglean013715	43.765902	46.451922	57.932271	58.01688
Gglean013716	20.3902	20.3517	15.6308	14.5106
Gglean013717	47.4402	61.68938	40.20527	37.50642
Gglean013718	4.705129	5.095493	2.74348	2.3192
Gglean013719	8.314363	8.305104775	4.508680661	3.991279
Gglean013720	3.690332	4.439757	7.336105	8.065316
Gglean013721	14.566224	13.53937192	22.70513017	27.637978
Gglean013722	14.566224	13.53937192	22.70513017	27.637978
Gglean013723	6.67916	6.17624	4.24031	3.81515
Gglean013724	0	0	0	0
Gglean013725	0	0	0.0261045	0
Gglean013726	7.649608943	4.88907715	6.391127528	6.388038119
Gglean013727	7.649608943	4.88907715	6.391127528	6.388038119

Gglean013728	100.43	86.5029	100.808	99.0074
Gglean013729	86.659207	90.21893351	109.322969	111.076258
Gglean013730	86.659207	90.21893351	109.322969	111.076258
Gglean013731	27.11854	29.199697	39.98965	38.756584
Gglean013732	0.1869065	0.4990196	0.514165	0.806873
Gglean013733	24.76450052	25.595167	41.384595	43.556329
Gglean013734	3.76759	3.221160025	3.13654	3.00908
Gglean013735	3.76759	3.221160025	3.13654	3.00908
Gglean013736	0	0	0	0
Gglean013737	22.8031	22.6026	23.0459	33.5217
Gglean013738	0.124443	0.38663	0.201719	0.120713
Gglean013739	57.63419339	44.98182096	66.20709157	73.993755
Gglean013740	57.63419339	44.98182096	66.20709157	73.993755
Gglean013741	57.63419339	44.98182096	66.20709157	73.993755
Gglean013742	0	0	0	0.207321
Gglean013743	0	0	0	0
Gglean013744	0.483814	0.000847427	1.03172	0.000131272
Gglean013745	47.7137754	53.736288	41.691755	39.2572409
Gglean013746	8.878373314	8.915389	10.263561	8.804556
Gglean013747	6.70238	4.28405	5.66713	6.86823
Gglean013748	11.5374	11.8258	6.95525	5.67159
Gglean013749	13.33748101	15.67006	17.054574	17.751359
Gglean013750	13.33748101	15.67006	17.054574	17.751359
Gglean013751	59.0914	65.6684	36.54123	29.78253
Gglean013752	42.5855	47.0923	47.0317	45.3561
Gglean013753	256.837842	268.9746017	273.0806018	278.707202
Gglean013754	256.837842	268.9746017	273.0806018	278.707202
Gglean013755	0	0	0	0
Gglean013756	5.357701001	5.12665	6.125560393	7.240386861
Gglean013757	77.59008278	81.7991444	126.4135519	138.4511536
Gglean013758	77.59008278	81.7991444	126.4135519	138.4511536
Gglean013759	0.331663	0.157176	0.468872	0.367192
Gglean013760	16.04259856	15.8308854	11.04017813	9.976850354
Gglean013761	16.963481	18.604797	18.09152118	16.81348314
Gglean013762	5.73681	4.58244	6.96067	2.99196
Gglean013763	0	0	0	0
Gglean013764	0	0	0	0
Gglean013765	17.5281	20.0727	26.0357	24.2475
Gglean013766	39.99231722	43.18824195	49.67594274	48.12752945
Gglean013767	21.00837423	23.08053002	33.21953675	38.42777016
Gglean013768	30.533211	34.833773	28.742029	27.201205
Gglean013769	0.213329	0.110854	0.173585	0.309671
Gglean013770	26.634868	30.46081	19.75243	17.6223252
Gglean013771	285.3954073	272.1689276	239.6525551	222.6329176

Gglean013772	121.24264	132.71752	108.60934	94.40312
Gglean013773	0.0262478	0	0	0.100549
Gglean013774	6.752362	5.397242561	3.326887057	2.810127725
Gglean013775	114.3089291	102.6727234	106.7510453	96.31551021
Gglean013776	6.1596569	7.53599847	6.955181131	7.639454825
Gglean013777	4.407966001	5.457678178	5.985841	7.546909514
Gglean013778	4.407966001	5.457678178	5.985841	7.546909514
Gglean013779	0	0	0	0
Gglean013780	0	0.0818256	0.0635876	0
Gglean013781	0	0	0.233339	0
Gglean013782	0	0	0	0
Gglean013783	3.082263	3.40164	4.503076	4.136318
Gglean013784	0	0	0	0
Gglean013785	0.126853	0.130833	0.132019	0.138864
Gglean013786	8.990201	8.922793239	11.638573	15.64684315
Gglean013787	8.990201	8.922793239	11.638573	15.64684315
Gglean013788	19.84005399	18.04603344	30.58758192	31.26963709
Gglean013789	85.29082988	86.64298182	105.9984981	99.76333386
Gglean013790	0	0	0	0
Gglean013791	116.9036	112.8834	106.5086	117.9019
Gglean013792	25.7771353	28.10417	7.732141	6.016037
Gglean013793	0.112376	0	0.0457228	0
Gglean013794	14.25657	11.832756	10.75521319	12.77170331
Gglean013795	6.5085728	7.298105377	9.73578611	12.08663725
Gglean013796	2.63864	4.44788	7.07326	6.89826
Gglean013797	9.1460499	11.41736636	8.550276833	7.80096323
Gglean013798	0.230734	0	0.280446	0.222072
Gglean013799	0	0	0	0
Gglean013800	29.964585	28.138715	25.5835	23.387305
Gglean013801	9.4267992	9.244464672	14.37670515	15.71795472
Gglean013802	0	0	0	0
Gglean013803	50.155824	52.821894	51.123438	50.58172
Gglean013804	15.71286	15.26376	13.63766	12.88409
Gglean013805	3.27127	6.63289	7.3094	3.7275
Gglean013806	842.45096	924.3474	483.9977	411.464953
Gglean013807	68.292229	67.28067243	75.88354207	78.78725055
Gglean013808	68.292229	67.28067243	75.88354207	78.78725055
Gglean013809	68.292229	67.28067243	75.88354207	78.78725055
Gglean013810	19.53448746	18.97338072	26.94447776	31.61871758
Gglean013811	39.4462	38.53154	25.78195	26.75623
Gglean013812	47.17472	46.68491	23.98985	19.89751
Gglean013813	0	0.637103	0	0
Gglean013814	51.58639	59.275323	48.307497	42.89404
Gglean013815	2.10887	2.6783	5.176088	6.6824

Gglean013816	16.74764	14.76027	17.26072	17.09561
Gglean013817	33.617889	38.4799383	34.733449	34.00787202
Gglean013818	33.617889	38.4799383	34.733449	34.00787202
Gglean013819	33.617889	38.4799383	34.733449	34.00787202
Gglean013820	8.421731001	12.2646411	14.27440111	15.934837
Gglean013821	8.421731001	12.2646411	14.27440111	15.934837
Gglean013822	10.8794	13.10573	7.05105	5.9077
Gglean013823	60.178587	62.03989	49.88668	49.21128
Gglean013824	9.55796	8.246215	5.690170031	6.580884
Gglean013825	4.645092073	6.434687	8.17573	9.000281
Gglean013826	12.65865	11.71318	11.815072	12.24147
Gglean013827	103.07268	126.26253	132.22877	133.33714
Gglean013828	808.7900856	909.0360066	794.2722603	707.2801576
Gglean013829	0	0	0	0
Gglean013830	20.668861	24.71214	20.70297	23.38388
Gglean013831	33.73583456	37.70617	32.99827779	32.2279744
Gglean013832	39.580362	39.257498	21.876673	18.97536111
Gglean013833	33.326	31.7291	24.1116	44.2116
Gglean013834	204.45	200.157	211.987	231.487
Gglean013835	0	0	0	0
Gglean013836	39.376684	38.69465	36.09914	37.07566
Gglean013837	26.05339	30.54557	22.650424	27.50373
Gglean013838	53.77174	57.61895	54.48652	50.13839
Gglean013839	51.50787611	62.08141055	50.52173175	51.8369311
Gglean013840	50.99915594	54.66349149	51.38428506	66.70033727
Gglean013841	18.38621	16.73647	32.88985	32.97401
Gglean013842	28.57062	31.52819	31.18077	27.464904
Gglean013843	28.676756	27.42451	26.25893	22.672261
Gglean013844	28.676756	27.42451	26.25893	22.672261
Gglean013845	28.676756	27.42451	26.25893	22.672261
Gglean013846	21.649392	21.85641625	17.87039156	17.05637927
Gglean013847	11.49996918	10.964417	3.198704662	3.499316887
Gglean013848	11.49996918	10.964417	3.198704662	3.499316887
Gglean013849	65.13181662	58.16188568	40.73160938	33.469961
Gglean013850	0	0	0.181589	0.108279
Gglean013851	0	0	0	0
Gglean013852	0	1.51E-06	0.56349	1.23E-06
Gglean013853	14.6683	13.9598	6.89865	5.31215
Gglean013854	0.203995	0.652158	0.498207	0.197514
Gglean013855	0.652886	2.984565186	2.762071347	1.134067695
Gglean013856	0.652886	2.984565186	2.762071347	1.134067695
Gglean013857	27.613956	26.17303414	18.058303	16.78464188
Gglean013858	36.4364	39.58276	32.95612	34.294264
Gglean013859	2.626314002	2.675382	3.59661	4.093119007

Gglean013860	27.8752	29.4887	11.4513	9.42424
Gglean013861	39.43656689	40.21739863	41.18037594	40.31170962
Gglean013862	39.43656689	40.21739863	41.18037594	40.31170962
Gglean013863	158.73498	178.2481	126.13361	118.32473
Gglean013864	86.99892446	89.34881376	52.477913	47.61268243
Gglean013865	86.99892446	89.34881376	52.477913	47.61268243
Gglean013866	17.70760394	17.67178664	22.66540744	25.40454642
Gglean013867	17.70760394	17.67178664	22.66540744	25.40454642
Gglean013868	9.311835	14.8043	15.216426	18.812229
Gglean013869	0	0	0	0
Gglean013870	162.35773	164.87943	127.45767	111.67721
Gglean013871	0.0252945	0.0385043	0.0356133	0.0240763
Gglean013872	0.333794	0.378635	0.590641	0.618546
Gglean013873	99.55915	104.72	74.92098	60.057619
Gglean013874	8.38325	8.07733	9.97436	10.328
Gglean013875	0	0	0	0
Gglean013876	7.1583	6.87433	2.333549	2.199714
Gglean013877	8.32182	11.10822	13.58018	12.763183
Gglean013878	0	0	0.454337	0
Gglean013879	16.31348	14.15712	12.05559	12.23524
Gglean013880	33.44003	27.568372	40.017256	52.14153
Gglean013881	441.4907	578.59979	771.687219	864.92159
Gglean013882	0	0	0	0
Gglean013883	1.7031	0	1.87507	0.559177
Gglean013884	33.94396	37.69436	24.36694	22.11506
Gglean013885	33.94396	37.69436	24.36694	22.11506
Gglean013886	4.59243	3.593030241	2.436398066	3.038926735
Gglean013887	10.047227	10.990297	15.208633	18.248401
Gglean013888	31.965077	32.3339	45.046343	52.7853
Gglean013889	2.30507	2.58996	1.57004	1.17729
Gglean013890	47.39295	49.02477667	57.10647	55.84335
Gglean013891	47.39295	49.02477667	57.10647	55.84335
Gglean013892	16.0655006	14.8777043	20.86908901	15.7330473
Gglean013893	28.487457	31.68658	28.140573	26.57736
Gglean013894	12.790917	14.48626	12.21701	12.624116
Gglean013895	26.57276364	26.34776116	24.34613854	22.40194624
Gglean013896	62.53085	59.564233	42.95639	43.9867
Gglean013897	0	0	0	0
Gglean013898	125.4295	129.6458	105.142	96.1036
Gglean013899	2.425200855	3.376623024	2.686019636	3.471574227
Gglean013900	0	0	0	0
Gglean013901	38.441968	41.236189	54.663665	54.393013
Gglean013902	29.15104	28.08793	64.72633	65.04923
Gglean013903	29.15104	28.08793	64.72633	65.04923

Gglean013904	0.0881222	0.136594	0.215437	0.0851907
Gglean013905	61.0183	58.518	35.8391	31.4227
Gglean013906	9.079886	10.97854842	12.18844	11.64055473
Gglean013907	9.079886	10.97854842	12.18844	11.64055473
Gglean013908	9.079886	10.97854842	12.18844	11.64055473
Gglean013909	9.079886	10.97854842	12.18844	11.64055473
Gglean013910	32.36354142	37.83527325	28.200626	27.21733103
Gglean013911	47.74368	45.496467	94.335139	87.849659
Gglean013912	47.74368	45.496467	94.335139	87.849659
Gglean013913	1.184975102	1.160950937	2.169266244	2.053986
Gglean013914	27.211016	29.399416	19.829781	22.97262003
Gglean013915	27.211016	29.399416	19.829781	22.97262003
Gglean013916	9.691069	9.809772	7.816128188	6.530078845
Gglean013917	30.36328124	31.36569398	33.61720171	32.53820802
Gglean013918	0	0	0	0
Gglean013919	2.34112	1.33051	3.91254	3.51225
Gglean013920	14.90390679	20.674234	20.6011002	21.03319941
Gglean013921	14.90390679	20.674234	20.6011002	21.03319941
Gglean013922	14.90390679	20.674234	20.6011002	21.03319941
Gglean013923	7.511855713	6.867434412	8.25893883	8.06689466
Gglean013924	0	0	0.0117569	0
Gglean013925	13.81743769	14.7846563	5.55279758	3.642850532
Gglean013926	8.8872	7.19643	6.63222	4.69698
Gglean013927	0.560039537	0.306305422	0.485739903	0.761427561
Gglean013928	0	0	0	0.0586713
Gglean013929	0	0	0	0
Gglean013930	27.4630071	36.52016296	41.59566742	45.58310387
Gglean013931	27.4630071	36.52016296	41.59566742	45.58310387
Gglean013932	0	0	0	0
Gglean013933	2.930091	2.105998	2.893446	3.92049
Gglean013934	2.930091	2.105998	2.893446	3.92049
Gglean013935	1.580730004	1.756836	2.391795639	3.14178
Gglean013936	32.4856	31.36905	29.343	26.41363
Gglean013937	48.58307	48.64562	54.01031	56.88753
Gglean013938	0	0	0	0
Gglean013939	7.22916	0	0	0
Gglean013940	4.72485	5.02887	6.69523	8.81171
Gglean013941	0	0	0	0
Gglean013942	0	0	0	0
Gglean013943	0	0	0	0
Gglean013944	0	0.128249	0	0.116685
Gglean013945	0.0566572	0	0	0
Gglean013946	0	0	0	0.0334717
Gglean013947	55.3954814	53.29710722	36.721036	33.77244394

Gglean013948	16.155662	17.26358023	7.278217634	6.139964
Gglean013949	55.3954814	53.29710722	36.721036	33.77244394
Gglean013950	55.3954814	53.29710722	36.721036	33.77244394
Gglean013951	55.3954814	53.29710722	36.721036	33.77244394
Gglean013952	55.3954814	53.29710722	36.721036	33.77244394
Gglean013953	55.3954814	53.29710722	36.721036	33.77244394
Gglean013954	5.381259	4.431944783	6.538826841	7.04081
Gglean013955	1.960286469	2.285088	3.848564583	3.87821946
Gglean013956	0.0543784	0.2818365	0.574581669	0.320649263
Gglean013957	15.694953	18.13312	25.0623456	35.46283
Gglean013958	8.90152	8.73885	11.08826	13.02395
Gglean013959	15.52097	17.16580015	23.958334	26.91428
Gglean013960	16.0840066	18.71825671	17.92943028	18.9997993
Gglean013961	9.6502	10.22147	14.99887	16.94454
Gglean013962	117.31661	131.004447	152.2665508	151.5166754
Gglean013963	117.31661	131.004447	152.2665508	151.5166754
Gglean013964	117.31661	131.004447	152.2665508	151.5166754
Gglean013965	117.31661	131.004447	152.2665508	151.5166754
Gglean013966	117.31661	131.004447	152.2665508	151.5166754
Gglean013967	0.529475	0.705668	0.979768	0.775103
Gglean013968	5.52424	6.09913	12.00155	12.47415
Gglean013969	0	0	0	0
Gglean013970	1.21929	0.658637	3.78535016	1.509570185
Gglean013971	40.89588238	42.36858438	57.59729	63.92325435
Gglean013972	40.8295	43.4187	26.63961	19.81981
Gglean013973	54.3878	55.2262	47.9025	46.1325
Gglean013974	18.43035941	19.522169	14.281358	12.65264903
Gglean013975	19.2650226	23.0760222	25.951895	21.509594
Gglean013976	2.06176	2.68332	4.43534	3.60916
Gglean013977	3.76965	4.89405	6.36324	7.70607
Gglean013978	31.33055	32.3945	27.15692	25.07075
Gglean013979	0	0	0	0
Gglean013980	17.99911016	18.81950321	19.3145303	17.66769542
Gglean013981	12.43127217	11.92820616	21.52487419	23.21446001
Gglean013982	69.21850488	76.50572808	84.99860871	90.48461269
Gglean013983	32.818893	35.24637	15.29904	11.09589223
Gglean013984	0	0	0	0
Gglean013985	19.928068	20.693593	16.994897	14.251621
Gglean013986	44.29954	51.39407	83.17153	96.21156
Gglean013987	27.6081	27.9908	28.0057	26.0079
Gglean013988	32.04396312	32.90700725	36.986963	39.26604802
Gglean013989	26.221459	29.90444713	27.845433	27.27658249
Gglean013990	2.215413	1.163957	0.957777	1.584012
Gglean013991	0	0	0	0

Gglean013992	15.250423	17.8630231	16.749361	16.75885199
Gglean013993	15.250423	17.8630231	16.749361	16.75885199
Gglean013994	4.785496001	4.946258	3.560087	3.075735444
Gglean013995	87.3123	85.3907	90.9157	94.8669
Gglean013996	0	0	0	0
Gglean013997	9.867163	11.576254	7.5142415	8.189566
Gglean013998	9.867163	11.576254	7.5142415	8.189566
Gglean013999	9.867163	11.576254	7.5142415	8.189566
Gglean014000	46.5764	62.42	5.79411	19.4643
Gglean014001	1.847496694	0.817158222	1.135931169	2.92916083
Gglean014002	1.847496694	0.817158222	1.135931169	2.92916083
Gglean014003	1.829155	2.186172611	2.661414203	2.459486
Gglean014004	30.3569203	34.04418548	47.18141645	57.25254
Gglean014005	35.98284	38.24258	28.73339	28.06171
Gglean014006	329.2481549	315.9647886	233.1056083	215.6592764
Gglean014007	36.26639	35.17794	27.69406	26.64719
Gglean014008	36.26639	35.17794	27.69406	26.64719
Gglean014009	34.631206	35.36213989	31.14490341	33.78912
Gglean014010	66.4907	62.3992	61.3384	63.2875
Gglean014011	53.88872	52.93916	44.08706	42.23528
Gglean014012	24.41986	25.44449	19.12799	18.63152
Gglean014013	50.259011	58.93973	53.16128	52.31699
Gglean014014	50.259011	58.93973	53.16128	52.31699
Gglean014015	3.548172077	3.478717196	4.557407512	4.583275577
Gglean014016	4.92584	0.842920251	3.88372	3.1999
Gglean014017	2.90E-06	0.375363	6.67E-05	0.518807
Gglean014018	282.689425	297.071131	209.119142	183.129006
Gglean014019	282.689425	297.071131	209.119142	183.129006
Gglean014020	126.0833	117.7148	88.2814	85.829
Gglean014021	32.17831056	27.703045	21.29186422	18.48848425
Gglean014022	19.205766	19.073577	23.428205	25.141534
Gglean014023	11.43562331	12.9081924	12.8245761	15.57801913
Gglean014024	11.43562331	12.9081924	12.8245761	15.57801913
Gglean014025	1.222796357	2.969577	3.333678789	4.137800411
Gglean014026	1.222796357	2.969577	3.333678789	4.137800411
Gglean014027	94.455	94.2073	102.3466	121.1751
Gglean014028	66.914183	67.06061801	54.08489703	46.75842194
Gglean014029	8.704386	10.69616665	9.432050998	10.55906551
Gglean014030	7.081261	6.326288	9.66616	10.05592243
Gglean014031	15.585694	11.80656778	13.733272	12.9561096
Gglean014032	15.585694	11.80656778	13.733272	12.9561096
Gglean014033	10.2572	11.13683	12.47718	11.20848
Gglean014034	41.02896	48.17059	32.59714	25.5847
Gglean014035	41.02896	48.17059	32.59714	25.5847

Gglean014036	48.92332	54.37974	52.26309	41.20482
Gglean014037	130.046562	103.4850133	138.370111	164.1390847
Gglean014038	24.82194	25.37292	23.24436	20.06436
Gglean014039	24.82194	25.37292	23.24436	20.06436
Gglean014040	0.191503281	0.177516796	0.223063987	0.197250229
Gglean014041	48.165	38.5462	41.3335	39.7206
Gglean014042	5.209037	7.37478	6.119366	7.902628
Gglean014043	13.782483	14.27141971	12.58582059	11.62713201
Gglean014044	20.916591	23.317541	12.068929	10.7463
Gglean014045	25.77876701	26.7636368	28.250296	28.79913374
Gglean014046	0.959562	0.180415	0.140815	0.332521
Gglean014047	0	0	0	0
Gglean014048	8.271342	8.82686	12.11943	14.4895
Gglean014049	8.271342	8.82686	12.11943	14.4895
Gglean014050	1.027035	1.078614	1.182239	1.958568
Gglean014051	1.97787	3.33143	5.0477	3.64103
Gglean014052	1.97787	3.33143	5.0477	3.64103
Gglean014053	0.200951	0.068946576	0.162982196	0
Gglean014054	107.4020574	114.2866396	115.62133	194.9383595
Gglean014055	38.99705	42.79582	23.7647	24.42711
Gglean014056	0	0	0	0
Gglean014057	9.928771	9.663790763	11.60951669	14.04693955
Gglean014058	2.87217	3.28084	14.3682	7.14964
Gglean014059	0	0	0	0
Gglean014060	0.147842	0.0764516	0	0
Gglean014061	0	0	0	0
Gglean014062	97.901695	99.12086	63.93559	60.36447
Gglean014063	97.901695	99.12086	63.93559	60.36447
Gglean014064	97.901695	99.12086	63.93559	60.36447
Gglean014065	119.7789322	111.254452	79.6233921	106.0899416
Gglean014066	119.7789322	111.254452	79.6233921	106.0899416
Gglean014067	7.1783	8.90411	7.07539	7.44744
Gglean014068	50.3444716	50.86217715	50.66604826	50.27104
Gglean014069	7.090074542	5.94774714	9.170213293	10.73589032
Gglean014070	14.5056	15.275	0.405519	0
Gglean014071	8.461404445	9.755788239	12.7483757	14.23067049
Gglean014072	19.1753	22.7947	26.0145	28.4725
Gglean014073	0	0	0	0
Gglean014074	28.31668	28.304536	38.06134	36.36220977
Gglean014075	265.4539	282.16679	186.0664	154.52137
Gglean014076	13.08223	15.15527	11.72499	11.48703
Gglean014077	0.121055105	0	0.126266304	0.074333294
Gglean014078	0.0110549	5.29E-05	2.40E-16	1.48E-09
Gglean014079	22.2796	21.5546	12.3755	10.9637

Gglean014080	4.474549118	4.297093924	4.624186717	5.432474173
Gglean014081	61.70765895	60.66724047	57.38897626	52.05002325
Gglean014082	61.70765895	60.66724047	57.38897626	52.05002325
Gglean014083	110.5050519	127.8745499	93.83706	89.08013
Gglean014084	110.5050519	127.8745499	93.83706	89.08013
Gglean014085	0.0368988	0.0378989	0.0887803	0
Gglean014086	0	0	0	0
Gglean014087	14.72951142	14.16878	8.907507488	8.869864902
Gglean014088	0	0	0	0
Gglean014089	0	0	0	0
Gglean014090	1.5194	1.08096	1.84851	2.496572
Gglean014091	33.89	26.364	15.041	14.6645
Gglean014092	0	0	0	0
Gglean014093	12.8053	14.326996	18.2152	20.9561
Gglean014094	73.2379516	79.06295204	132.0422252	145.8742424
Gglean014095	0	0.744939	7.34E-05	0.33983
Gglean014096	3.14828	5.49141	5.94957	5.69863
Gglean014097	2.064642	1.72475	2.137239	3.261131838
Gglean014098	0	0	0	0
Gglean014099	25.323197	24.985461	15.554055	16.229258
Gglean014100	0	0	0	0
Gglean014101	289.1479	254.0174	340.9181	436.1528
Gglean014102	2.349136	1.424445	2.209516	3.946263
Gglean014103	0	0	0	0
Gglean014104	0	0	0	0
Gglean014105	7.891254581	8.587463	10.6957455	13.13708
Gglean014106	0	0	0	0
Gglean014107	0	0.0201679	0	0.0189272
Gglean014108	29.7086	31.0095	32.0425	35.2696
Gglean014109	7.218754549	7.334586201	9.708611983	11.41134729
Gglean014110	0	0	0	0
Gglean014111	13.1563	9.99679	18.1935	17.2776
Gglean014112	289.33	266.4971	470.2271	456.5891
Gglean014113	3.44402	0.576178	1.6115	2.10383
Gglean014114	9.57969	11.53128	15.37519	14.44538
Gglean014115	0	0	0	0.0415532
Gglean014116	0	0	0	0
Gglean014117	128.6370594	129.79184	181.100299	206.6386572
Gglean014118	2.207963861	1.81575773	1.716714563	1.527603
Gglean014119	0	0	0	0
Gglean014120	6.07307	4.95654	7.62364	7.923850002
Gglean014121	3.23415	3.61604	2.81104	2.36709
Gglean014122	0	0	0	0
Gglean014123	0.120639	0.062359	0.146853	0.0580014

Gglean014124	16.05908	13.588805	16.47624334	21.780346
Gglean014125	1.522222407	1.650330383	1.423749349	1.192277847
Gglean014126	1.522222407	1.650330383	1.423749349	1.192277847
Gglean014127	0	0	0.0249404	0
Gglean014128	35.50948364	31.30836856	29.8975356	31.00458885
Gglean014129	35.50948364	31.30836856	29.8975356	31.00458885
Gglean014130	12.57027072	12.33359117	6.831507378	7.268583577
Gglean014131	0.555040996	0.2645197	0.920829	1.039261
Gglean014132	0.555040996	0.2645197	0.920829	1.039261
Gglean014133	14.0312	14.4514	14.9596	14.5025
Gglean014134	51.20792	51.37911228	34.74299957	36.211024
Gglean014135	14.0312	14.4514	14.9596	14.5025
Gglean014136	14.0312	14.4514	14.9596	14.5025
Gglean014137	14.0312	14.4514	14.9596	14.5025
Gglean014138	40.93699402	46.24737871	40.617927	36.16468262
Gglean014139	39.415383	38.070991	34.002572	38.25387004
Gglean014140	13.9096302	14.48844519	18.9678681	24.26156
Gglean014141	43.8287	41.5995	24.11335	28.4835
Gglean014142	4.351005	6.83779	6.520203	9.60184
Gglean014143	20.84769	16.78188	17.21516	16.40359
Gglean014144	8.77733	9.43604	3.80272	3.29332
Gglean014145	32.86539	33.12536	32.1556	34.6598
Gglean014146	104.65685	100.57687	56.66921	50.96245
Gglean014147	104.65685	100.57687	56.66921	50.96245
Gglean014148	5.97887	5.81438	3.70004	3.82714
Gglean014149	21.1089	21.7849	17.6973	19.1433
Gglean014150	21.08424	21.6824	10.82458	15.28297
Gglean014151	165.6326482	172.6101473	106.2372209	79.11492015
Gglean014152	165.6326482	172.6101473	106.2372209	79.11492015
Gglean014153	3.544776424	3.1814169	2.214314165	1.45794638
Gglean014154	3.544776424	3.1814169	2.214314165	1.45794638
Gglean014155	7.784609	6.87876	4.57565	3.951186
Gglean014156	11.0637714	7.722827185	7.0222693	6.692507142
Gglean014157	4.79786	0.49020367	2.61208	0.26574
Gglean014158	4.79786	0.49020367	2.61208	0.41171061
Gglean014159	373.3538862	420.5158601	245.5160856	181.227323
Gglean014160	50.7612	51.3261	39.0812	36.6798
Gglean014161	44.23017446	50.6820635	42.27717715	37.83812104
Gglean014162	44.23017446	50.6820635	42.27717715	37.83812104
Gglean014163	3.736196	5.888742009	7.0807518	6.242525
Gglean014164	4.82209	4.92539	6.02133	6.85245
Gglean014165	19.52583	21.01562	8.4847	7.10518
Gglean014166	16.40492	13.99595	9.13532	8.99531
Gglean014167	3.050539655	3.336296	5.93586	5.80727

Gglean014168	13.92711036	14.47778399	22.50359904	25.06868062
Gglean014169	13.92711036	14.47778399	22.50359904	25.06868062
Gglean014170	8.036384408	7.200491002	8.437953	12.573948
Gglean014171	14.28481054	12.04956263	18.99547733	21.50064002
Gglean014172	18.590482	18.36219698	14.5257786	13.75246646
Gglean014173	24.030862	22.925784	20.453642	24.18088
Gglean014174	24.030862	22.925784	20.453642	24.18088
Gglean014175	0	0.0741972	0	0
Gglean014176	29.0928	30.317	11.18878	10.02877
Gglean014177	18.96876362	22.873544	10.05785548	10.42961679
Gglean014178	48.34614814	40.79717966	29.58593608	26.62593259
Gglean014179	45.52302019	42.89244	38.56975201	37.123374
Gglean014180	1.3258	1.40169	1.97449	2.56603
Gglean014181	61.11069	64.15713	92.55894	96.45464
Gglean014182	13.96778	13.53996	16.15271	28.82786
Gglean014183	15.4668	14.4261	13.6421	11.1745
Gglean014184	12.0207	13.72	8.21299	6.5121
Gglean014185	20.62466771	16.30629	15.63741718	13.3927402
Gglean014186	20.62466771	16.30629	15.63741718	13.3927402
Gglean014187	24.2692	15.59184	18.7079	17.93315
Gglean014188	22.38867	20.8623	14.25904	13.02832
Gglean014189	0	0	0	0
Gglean014190	6.91109	6.35873	8.49811	8.74828
Gglean014191	88.4603	93.3904	29.3202	34.1309
Gglean014192	1.423294162	2.1309745	2.22749	2.645797
Gglean014193	4.145371569	5.358675	4.10337	1.812937178
Gglean014194	4.145371569	5.358675	4.10337	1.812937178
Gglean014195	55.6594	51.0037	16.4929	20.3778
Gglean014196	23.8416038	25.31432	23.911398	23.48164767
Gglean014197	60.38653	61.88427	42.35317	53.63399
Gglean014198	15.96846	16.96075	16.76704	16.55672
Gglean014199	75.4703	76.0972	38.1776	37.0009
Gglean014200	0.145517569	0.205302	0.424350394	0.415214136
Gglean014201	0	0	0	0
Gglean014202	0	0	0	0
Gglean014203	0	0	0	0
Gglean014204	59.9341	65.4735	69.8141	94.1533
Gglean014205	0	0	0	0
Gglean014206	67.2118	86.49	63.7008	130.747
Gglean014207	0.733869	0	0	0
Gglean014208	37.0581	20.70345755	40.5805	53.8794
Gglean014209	0	0	0	0
Gglean014210	0	0	0	0
Gglean014211	0	0	0	0

Gglean014212	8.410383801	10.14928605	6.350812	5.216927955
Gglean014213	0	0	0	0
Gglean014214	0	0	0	0
Gglean014215	0	0	0	0
Gglean014216	0	0	0	0
Gglean014217	0	0	0	0
Gglean014218	1.148761	1.627531	2.09973	1.934392
Gglean014219	0.145483	0.0495236	0.117967	0.0466156
Gglean014220	15.15464682	16.8624798	11.827631	14.199887
Gglean014221	104.5114	99.8297	112.0546	119.1215
Gglean014222	104.5114	99.8297	112.0546	119.1215
Gglean014223	27.70564419	29.43989662	24.86368094	25.9148125
Gglean014224	27.70564419	29.43989662	24.86368094	25.9148125
Gglean014225	8.723877	7.368314	4.794316177	4.1030412
Gglean014226	59.96397003	66.62490358	49.69578504	45.68759068
Gglean014227	59.96397003	66.62490358	49.69578504	45.68759068
Gglean014228	1.34E-05	5.57E-05	1.97E-05	9.93E-05
Gglean014229	59.85778973	62.01169	36.552419	32.594468
Gglean014230	0.0603321	0	0	0.116107
Gglean014231	0.0383426	0	0.0156605	0.0185195
Gglean014232	11.0004	11.7163	11.586	11.8824
Gglean014233	0.028841	0	0.0232849	0
Gglean014234	0	0	0	0
Gglean014235	0	0	0	0
Gglean014236	0	0	0	0
Gglean014237	35.81975642	38.13195117	62.7962603	61.17865072
Gglean014238	35.81975642	38.13195117	62.7962603	61.17865072
Gglean014239	3.09203	2.89474	1.84146	2.45574
Gglean014240	24.8833	23.0305	17.8789	21.2903
Gglean014241	7.657664287	8.831406189	11.34840935	13.808591
Gglean014242	15.6728	17.3449	22.5711	26.7796
Gglean014243	9.775434912	9.576329327	8.58243695	8.64818697
Gglean014244	9.775434912	9.576329327	8.58243695	8.64818697
Gglean014245	28.968028	25.742442	27.4618	25.477221
Gglean014246	22.83962236	22.21323728	32.61657032	39.85518688
Gglean014247	22.83962236	22.21323728	32.61657032	39.85518688
Gglean014248	6.60551	9.53731	5.46575	13.0556
Gglean014249	0.040671378	0.013312289	0	0
Gglean014250	0.040671378	0.013312289	0	0
Gglean014251	0.420548	0.2548195	0.46830623	0.86909
Gglean014252	0.420548	0.2548195	0.46830623	0.86909
Gglean014253	0.236293	0.360603	0.288533	0.456131
Gglean014254	0.726124	0.700299	0.099567	0.0656084
Gglean014255	0	0	0	0

Gglean014256	0	0	0	0
Gglean014257	1.40838	1.76217	0.778686	0.465814
Gglean014258	40.09231378	39.52215974	64.1406432	70.91511185
Gglean014259	6.2246471	7.226898	7.479841	10.062017
Gglean014260	6.2246471	7.226898	7.479841	10.062017
Gglean014261	2.35665	3.11711	0.321361	0.509354
Gglean014262	7.70E-05	2.33E-05	0.000117092	2.41E-05
Gglean014263	0.307404	0.295164991	0.293408	0.557147
Gglean014264	539.80214	625.061413	693.3710887	675.8911415
Gglean014265	0.763377	0.986895	0.541295	0.92465
Gglean014266	0	0	0	0
Gglean014267	0	0	0.152879	0
Gglean014268	0	0	0.145061	0
Gglean014269	0	0	0	0
Gglean014270	0	0	0	0
Gglean014271	0.153118272	1.95E-07	0.322726485	0.429557547
Gglean014272	0	0	0	0
Gglean014273	2.8198683	2.934659975	0.0182375	0.440792
Gglean014274	2.8198683	2.934659975	0.0182375	0.440792
Gglean014275	8.21404	6.05631	2.99732	4.32651
Gglean014276	3.58E-05	1.75E-05	0.00286844	0.00013732
Gglean014277	0.729891	0.460517	0.193423	0.509919
Gglean014278	10.780602	10.48470771	10.10792	10.84542846
Gglean014279	140.076592	144.7140189	99.88786967	92.26032353
Gglean014280	140.076592	144.7140189	99.88786967	92.26032353
Gglean014281	1.19316	2.06402	1.12285	2.34613
Gglean014282	1.19316	2.06402	1.12285	2.34613
Gglean014283	0	0	0.163797	0.194755
Gglean014284	18.09992169	23.78239844	31.64805491	34.93947935
Gglean014285	76.390909	69.9956116	47.718908	54.888582
Gglean014286	28.08788046	28.75827276	25.39166303	23.42232322
Gglean014287	21.90303504	24.0142021	12.92443964	12.32293853
Gglean014288	21.90303504	24.0142021	12.92443964	12.32293853
Gglean014289	9.42113	8.88486	11.07412	14.99905
Gglean014290	2.672729	3.87122299	2.532638505	3.799894464
Gglean014291	2.672729	3.87122299	2.532638505	3.799894464
Gglean014292	2.672729	3.87122299	2.532638505	3.799894464
Gglean014293	0	0.808238	0	0.188112
Gglean014294	0	0	0	0
Gglean014295	0	0	0	0
Gglean014296	0	0	0	0
Gglean014297	0	0	0	0
Gglean014298	0	0	0	0
Gglean014299	0	0	0	0

Gglean014300	0	0	0	0
Gglean014301	6.98314	7.43413	9.03178	7.76522
Gglean014302	6.397378905	5.925480076	5.754914672	4.730669919
Gglean014303	80.7095	87.5776	49.61091	42.8449
Gglean014304	5.943939889	5.442596112	5.578184652	6.000977863
Gglean014305	0.00391049	4.46E-05	7.61E-05	4.75E-05
Gglean014306	0.00136741	0.000962533	5.46827	1.33482
Gglean014307	17.52219	14.857091	16.3007332	15.62732353
Gglean014308	17.52219	14.857091	16.3007332	15.62732353
Gglean014309	1.80458	1.64427	1.45348	1.58778
Gglean014310	14.21885	12.508	30.14882	24.02024
Gglean014311	0.699464	0.207899	0.0981247	0.781348
Gglean014312	0	0	0	0
Gglean014313	20.46028628	19.69827272	30.14947356	31.5961097
Gglean014314	0	0	0	0
Gglean014315	0.3236282	0.358075468	0.697762	0.4366
Gglean014316	0.129008	0	0.0524056	0.123985
Gglean014317	51.69864009	41.94953111	65.93799187	74.37604591
Gglean014318	51.69864009	41.94953111	65.93799187	74.37604591
Gglean014319	51.69864009	41.94953111	65.93799187	74.37604591
Gglean014320	51.69864009	41.94953111	65.93799187	74.37604591
Gglean014321	17.04777356	19.30801266	30.00400149	26.89516872
Gglean014322	17.04777356	19.30801266	30.00400149	26.89516872
Gglean014323	3734.56199	3565.22503	1474.105418	1356.079902
Gglean014324	0.117197	0	0	0
Gglean014325	743.0694	413.0529	631.1713	712.5115
Gglean014326	0.101693	8.08E-07	0.960971	1.88E-16
Gglean014327	3.951134	4.052218648	3.7432372	4.994776
Gglean014328	11.04131	12.88731	3.751559	4.362195
Gglean014329	11.04131	12.88731	3.751559	4.362195
Gglean014330	52.24962	48.14809	37.60191	33.93344
Gglean014331	6.879394198	7.459915375	9.458313139	12.28233161
Gglean014332	37.7688	39.62111	50.56359	62.78712
Gglean014333	3.775263834	3.590978313	2.047516317	1.774456762
Gglean014334	149.2878	164.0688	108.3494	94.7686
Gglean014335	467.694677	460.18642	302.3839531	271.3344979
Gglean014336	1.260867	0.729467	1.15347	0.783469
Gglean014337	3.518846	3.692549	5.474181	5.277133
Gglean014338	68.9612	66.2201	59.5873	52.39056
Gglean014339	68.9612	66.2201	59.5873	52.39056
Gglean014340	0.1100789	0.173371208	0.112447308	0.2029571
Gglean014341	5.68282	2.20334	3.24335	2.56471
Gglean014342	57.7799	63.8063	69.2443	70.743
Gglean014343	16.25645	22.91304	17.59797	17.7005

Gglean014344	16.25645	22.91304	17.59797	17.7005
Gglean014345	7.0179264	7.685325162	12.057619	13.04085287
Gglean014346	5.408969989	9.334537	5.29825338	6.53724
Gglean014347	5.408969989	9.334537	5.29825338	6.53724
Gglean014348	6.30391	4.90923	6.20509	4.25446
Gglean014349	167.5136	190.4884	76.4255	62.594
Gglean014350	8.959772003	10.44236	5.480729179	4.679153837
Gglean014351	8.959772003	10.44236	5.480729179	4.679153837
Gglean014352	8.959772003	10.44236	5.480729179	4.679153837
Gglean014353	6.2632	7.28392	11.5115	14.0689
Gglean014354	221.5590505	227.78797	247.68721	243.7418
Gglean014355	16.974777	16.0675201	16.14108308	11.684128
Gglean014356	16.974777	16.0675201	16.14108308	11.684128
Gglean014357	21.50474872	15.08092022	17.4864665	19.1900828
Gglean014358	21.50474872	15.08092022	17.4864665	19.1900828
Gglean014359	21.50474872	15.08092022	17.4864665	19.1900828
Gglean014360	57.56887	51.38215	86.38976	125.6245
Gglean014361	0	0	0	0
Gglean014362	22.9512	23.438	32.0023	37.1301
Gglean014363	0.103781467	0	0	0
Gglean014364	15.10459414	15.75032906	9.689421767	9.749068787
Gglean014365	108.297	118.0682	70.702	65.9434
Gglean014366	13.33731	17.13612	15.98299	22.17573
Gglean014367	18.67514923	21.7993033	23.63457024	23.74034709
Gglean014368	3.28E-05	1.44E-05	0.487911	8.42E-05
Gglean014369	348.023	416.418	688.549	729.062
Gglean014370	534.998	543.419	932.729	942.341
Gglean014371	36.115132	39.980764	36.8044879	36.16318368
Gglean014372	36.115132	39.980764	36.8044879	36.16318368
Gglean014373	42.31990022	48.50346332	61.96404359	68.23778791
Gglean014374	0.1148683	0.117565015	0.164354	0.0711969
Gglean014375	2.39015415	2.56382089	2.65485012	3.641364461
Gglean014376	15.66328	17.38912	6.98423	7.58489
Gglean014377	31.15431687	28.9547876	44.4614375	49.83908
Gglean014378	112.711284	110.3993884	71.8279479	71.6614303
Gglean014379	112.711284	110.3993884	71.8279479	71.6614303
Gglean014380	22.766992	34.2309	26.65269	26.6545166
Gglean014381	28.88412838	25.30584525	23.07450598	35.34701
Gglean014382	5.62325	3.29685	6.70431	6.99444
Gglean014383	27.22475255	22.7101897	18.20003889	16.98135782
Gglean014384	0.00016914	0.000101392	0.145106	0.000247422
Gglean014385	0.268885188	0.104403296	0.276098189	0.525898872
Gglean014386	5.93116	3.79541	5.10346	12.1381
Gglean014387	14.540761	13.691443	9.8877829	11.39439

Gglean014388	1.873812	2.40962836	2.898055601	3.778418
Gglean014389	0	1.37877	0	0
Gglean014390	0	0	0	0
Gglean014391	0	0	0	0
Gglean014392	395.332	368.554	550.571	695.574
Gglean014393	40.24000546	41.53586393	26.70861812	25.44555881
Gglean014394	29.30635588	30.61604283	16.387556	15.92867944
Gglean014395	71.49951	74.1513	48.3258604	41.64893
Gglean014396	71.49951	74.1513	48.3258604	41.64893
Gglean014397	13.81268	14.57552	14.45759	14.895941
Gglean014398	6.106952017	5.534137384	3.692207277	4.103572
Gglean014399	36.95807197	45.32390098	34.47479065	34.01531267
Gglean014400	4.66024	2.20237	1.90101	3.53609
Gglean014401	0	0	0	0
Gglean014402	14.11375672	17.90182	5.73812988	4.420630008
Gglean014403	464.033	472.001	356.948	395.83
Gglean014404	7.685883	7.778158	2.1037546	2.80028
Gglean014405	15.11698	13.09967	19.86028	22.4388
Gglean014406	0	0.408308	0.156666	0.374299
Gglean014407	107.208967	94.974137	68.62039172	73.4585281
Gglean014408	0.193418	0.321627	0.283006	0.1341508
Gglean014409	107.208967	94.974137	68.62039172	73.4585281
Gglean014410	63.5613	71.9969	36.1614	34.3334
Gglean014411	2.548109	3.880774	3.46617	4.87336
Gglean014412	0.0830716	0.350744	0.203391	0.0802696
Gglean014413	0	0	0	0
Gglean014414	0	0	0	0
Gglean014415	14.0989034	16.17680333	7.064992921	6.412212744
Gglean014416	0.686398606	0.349235	0.347118	0
Gglean014417	4.44E-14	0.308751	0	8.86E-29
Gglean014418	6.25879	6.89666	12.5683	14.0094
Gglean014419	0	0.6736324	0.53803723	0.638615179
Gglean014420	0.0499744	0.103501816	0	0.0956662
Gglean014421	19.08251031	18.18594202	12.5180793	11.46219111
Gglean014422	14.158156	15.87477778	1.22593607	1.033182994
Gglean014423	15.3375	16.2255	12.9408	12.8982
Gglean014424	51.65585535	47.75698202	41.30174882	41.22184507
Gglean014425	51.1518	49.7961	49.2705	41.2522
Gglean014426	2.907356399	2.777927273	1.97206029	1.948922368
Gglean014427	3.818821972	3.814482	4.698860317	4.222558401
Gglean014428	234.5116	226.3042	283.9165	477.7461
Gglean014429	9.671514135	7.011627817	10.28670007	11.25530993
Gglean014430	56.74303	54.04108	43.10832	39.3665
Gglean014431	56.74303	54.04108	43.10832	39.3665

Gglean014432	56.74303	54.04108	43.10832	39.3665
Gglean014433	15.22195	17.101624	8.9843653	8.1861408
Gglean014434	0.97609	1.55838	1.86387	2.46392
Gglean014435	2.64194	2.4036	4.14399	1.7455
Gglean014436	71.7019788	65.4014776	98.4901022	99.946062
Gglean014437	71.7019788	65.4014776	98.4901022	99.946062
Gglean014438	2.51E-66	2.50E-25	0.950999	1.19E-77
Gglean014439	3.18799	2.90363	5.49577	4.18516
Gglean014440	71.7019788	65.4014776	98.4901022	99.946062
Gglean014441	71.7019788	65.4014776	98.4901022	99.946062
Gglean014442	71.7019788	65.4014776	98.4901022	99.946062
Gglean014443	71.7019788	65.4014776	98.4901022	99.946062
Gglean014444	71.7019788	65.4014776	98.4901022	99.946062
Gglean014445	71.7019788	65.4014776	98.4901022	99.946062
Gglean014446	9.22713	9.4249214	8.28603	7.930641
Gglean014447	5.466872676	8.480442857	11.777586	14.06542912
Gglean014448	14.56908	15.1387	2.79004	2.474891
Gglean014449	62.6633	75.9691	56.4786	113.2078
Gglean014450	76.89623	53.885393	38.350348	56.62613353
Gglean014451	0	0	0	0
Gglean014452	0.228310403	0	0.942356561	0.746837
Gglean014453	9.608280037	9.52524574	7.563162	5.87493
Gglean014454	9.608280037	9.52524574	7.563162	5.87493
Gglean014455	2.833	3.21029	5.48473	11.2875
Gglean014456	5.78601	0.806091	5.92688	3.73472
Gglean014457	0.478825	0	0.646556	0.460121
Gglean014458	22.2928	23.3536	14.9872	13.5962
Gglean014459	312.037483	320.2214284	462.212956	455.264229
Gglean014460	64.00445901	63.7486732	51.02175959	46.85543806
Gglean014461	8.103016	8.44335	8.57567	8.05636
Gglean014462	2.218508	2.243887	3.441285	4.941332
Gglean014463	16.22655	29.21386	17.35715	16.9072412
Gglean014464	12.4242	14.09561	14.13237	25.26184
Gglean014465	54.3796	64.86987	15.63088024	14.00381
Gglean014466	11.89082985	12.454911	7.11237	7.667826
Gglean014467	6.94856	7.08611	7.11792	8.3593
Gglean014468	6.94856	7.08611	7.11792	8.3593
Gglean014469	18.24436	16.85241305	23.53621661	22.88346
Gglean014470	18.24436	16.85241305	23.53621661	22.88346
Gglean014471	67.5250533	66.2636826	81.49318	94.0368522
Gglean014472	37.29	43.37600008	56.7035	66.1161
Gglean014473	7.28059801	7.67733	7.037552	7.0968901
Gglean014474	0	0	0.0480926	0
Gglean014475	10.5802	11.8554	9.68766	6.96613

Gglean014476	8.608594936	9.682942748	12.2282637	13.590887
Gglean014477	9.4902	9.87887	4.17588	3.76781
Gglean014478	4.286316975	5.729766353	2.390072167	2.496926461
Gglean014479	0.361865	1.04394	0.99437	1.24506
Gglean014480	17.2501	20.4166	30.8913	32.0327
Gglean014481	0.849674	2.68899	1.03474	4.13151
Gglean014482	2.64924	2.21652	2.66847	7.79952
Gglean014483	0	0	0	0
Gglean014484	1.98E-06	0.000287689	2.93E-05	5.76E-05
Gglean014485	0	0	0.0777883	0
Gglean014486	0	0	0	0
Gglean014487	0	0	0	0
Gglean014488	0.104206	0	0.337223	0
Gglean014489	0.58124431	1.026910647	1.108002717	2.053888486
Gglean014490	0.374694415	0.774040761	1.288939322	0.449164706
Gglean014491	0	0.670697	1.00042	0
Gglean014492	19.758322	17.41879	13.63870001	32.14676
Gglean014493	44.06639481	40.999859	35.49810767	31.902784
Gglean014494	2.926	2.39673219	3.864234747	4.479095208
Gglean014495	0	0	0	0
Gglean014496	13.3696	15.247	10.5784	8.55719
Gglean014497	3.00951	2.68381	2.3766	3.59496
Gglean014498	0	0	0	0
Gglean014499	0	0	0	0
Gglean014500	0.071251	0.0367435	0.0575316	0.101881
Gglean014501	0	0	0	0
Gglean014502	0	0	0	0
Gglean014503	0	0	0	0
Gglean014504	0	0	0	0
Gglean014505	0	0	0	0
Gglean014506	0	0	0	0
Gglean014507	0	0	0	0
Gglean014508	0	0	0	0
Gglean014509	3.01885	4.75199	3.14428	3.74815
Gglean014510	2.70786	0	8.99772	5.36288
Gglean014511	4.06179	3.02516	3.9365	4.69252
Gglean014512	54.5317223	51.94956874	37.1235365	32.78638823
Gglean014513	54.5317223	51.94956874	37.1235365	32.78638823
Gglean014514	39.80359	37.02539	38.886049	36.77641
Gglean014515	77.18885006	81.94966731	65.95912346	59.11849569
Gglean014516	77.18885006	81.94966731	65.95912346	59.11849569
Gglean014517	44.97972	50.68721	33.87455	25.97917
Gglean014518	1798.676	1788.572	1142.135	1049.018
Gglean014519	71.16532	74.74584	67.74615	56.644346

Gglean014520	8.35354	8.45607	3.90135	4.10888
Gglean014521	19.94282587	21.80870324	19.30982115	18.58402203
Gglean014522	83.5775	91.3748	119.075	111.114
Gglean014523	7.577958281	8.488814782	10.92712913	12.74643205
Gglean014524	7.577958281	8.488814782	10.92712913	12.74643205
Gglean014525	10.801511	11.84676051	13.64415543	18.50729795
Gglean014526	10.801511	11.84676051	13.64415543	18.50729795
Gglean014527	41.90253689	39.90328034	52.70594379	45.39295727
Gglean014528	41.90253689	39.90328034	52.70594379	45.39295727
Gglean014529	7.1715972	7.39603	8.6427827	10.061263
Gglean014530	526.33933	604.8836	652.47037	732.83388
Gglean014531	0.0846057	0	0	0
Gglean014532	36.73604	38.09367003	41.624295	43.9531
Gglean014533	8.212770434	7.754875357	9.576967	12.46869469
Gglean014534	14.99566859	14.03161814	12.015681	12.27685698
Gglean014535	165.8752045	177.416303	157.9767923	165.4464117
Gglean014536	165.8752045	177.416303	157.9767923	165.4464117
Gglean014537	165.8752045	177.416303	157.9767923	165.4464117
Gglean014538	0.156262	0	0	0.0499481
Gglean014539	25.28215567	27.68845326	13.67009105	11.56559716
Gglean014540	165.29991	145.21529	37.46662	17.45563064
Gglean014541	31.77626658	33.262423	28.753347	27.77336563
Gglean014542	114.04997	106.44397	75.09149	76.74794
Gglean014543	301.672	326.218	186.2673	184.4427
Gglean014544	83.8427	92.2111	112.6998	114.9891
Gglean014545	15.5587	9.88083	6.5289	14.4574
Gglean014546	17.82265216	20.68929153	13.76363022	10.51266802
Gglean014547	15.78833974	15.614281	12.8838109	10.740221
Gglean014548	94.741734	95.47301	88.63328005	82.19678012
Gglean014549	94.741734	95.47301	88.63328005	82.19678012
Gglean014550	2.801665	2.1815036	2.807555674	3.673819001
Gglean014551	2.801665	2.1815036	2.807555674	3.673819001
Gglean014552	21.37442	26.29235	39.8146	40.3768
Gglean014553	129.1589485	131.8697131	93.1903872	85.15785
Gglean014554	14.42221969	14.14591292	8.980974708	7.473937579
Gglean014555	0.0173788	0.0178683	0.0281427	0.033283
Gglean014556	0	0	0	0
Gglean014557	0	0	0	0
Gglean014558	0	0	0	0
Gglean014559	0.384135926	0	0	0
Gglean014560	0.6143506	0.560203969	0.534991976	0.68686517
Gglean014561	0.6143506	0.560203969	0.534991976	0.68686517
Gglean014562	1.171305	1.163432	0.728205791	1.4099
Gglean014563	0	0	0	0

Gglean014564	33.31696	25.95008	39.00058	54.39194
Gglean014565	0.194830624	0.813290169	0.39332216	0.419105514
Gglean014566	5.71992	4.49129	4.89507	6.00225
Gglean014567	0	0	0	0
Gglean014568	0	0	0	0
Gglean014569	0.583803203	1.208131901	1.346690196	0.476475405
Gglean014570	0	0	0	0
Gglean014571	0	0	0	0
Gglean014572	0	0	0	0
Gglean014573	0	0	0	0
Gglean014574	0	0	0	0
Gglean014575	0	0	0	0
Gglean014576	0	0	0	0.0274113
Gglean014577	0	0	0	0.0636965
Gglean014578	0	0	11.7593	0
Gglean014579	0.142837	0.223612	0	0.275943
Gglean014580	5.04369	7.71578	11.0187	13.9255
Gglean014581	34.7241739	32.083848	50.44686	63.417498
Gglean014582	0	0	0	0
Gglean014583	34.7241739	32.083848	50.44686	63.417498
Gglean014584	0	0	0	0
Gglean014585	34.7241739	32.083848	50.44686	63.417498
Gglean014586	8.522313	8.49319	9.12532	19.05996
Gglean014587	8.1151	9.95187	13.4175886	12.5495
Gglean014588	13.66262	16.06045	10.62554	9.73333
Gglean014589	0.182173	0.383553	0.196612	0.522286
Gglean014590	39.997921	42.325812	37.73613916	41.99911
Gglean014591	0.0317511	0.163594	0.334594	0.456254
Gglean014592	17.8943	20.9907	37.4996	39.6268
Gglean014593	45.4058	52.121	23.758	18.9773
Gglean014594	23.81884	21.33268	22.87137	22.52867
Gglean014595	0.66383	2.097540179	1.641068255	0.670577038
Gglean014596	0.66383	2.097540179	1.641068255	0.670577038
Gglean014597	0	0	0	0.333055
Gglean014598	0	0	0	0
Gglean014599	35.68681	34.41329	15.30615	12.5404
Gglean014600	44.1072	52.0441	40.9555	43.2258
Gglean014601	123.36743	128.23572	58.773946	49.58099
Gglean014602	0	0	0.107305653	0.189286072
Gglean014603	0	0	0.107305653	0.189286072
Gglean014604	0	0	0.107305653	0.189286072
Gglean014605	0	0	0.014499647	0.069275081
Gglean014606	0	0	0.014499647	0.069275081
Gglean014607	39.4565068	42.54896451	35.09216852	32.48899717

Gglean014608	0.35607	0	0.295759	0
Gglean014609	7.478658357	8.348432	6.931898089	10.84553
Gglean014610	7.478658357	8.348432	6.931898089	10.84553
Gglean014611	7.478658357	8.348432	6.931898089	10.84553
Gglean014612	0.00167844	0.495845	0.0167548	0.50466
Gglean014613	0	0.091044362	0	0
Gglean014614	52.784568	52.2309915	56.7823015	58.383039
Gglean014615	3.154866257	3.816458252	2.60591868	2.496327252
Gglean014616	3.154866257	3.816458252	2.60591868	2.496327252
Gglean014617	57.41318	55.13162	33.659226	40.08995
Gglean014618	57.41318	55.13162	33.659226	40.08995
Gglean014619	57.41318	55.13162	33.659226	40.08995
Gglean014620	1.466604496	0.191616475	0.591112062	0.526556575
Gglean014621	0.118545	0.12188	0.383639	0.227665
Gglean014622	1.07095372	0.39838074	0.955296041	0.985057258
Gglean014623	53.46229395	48.59860018	96.111734	96.741631
Gglean014624	0	0	0	0.118673796
Gglean014625	0.223709587	0.104009517	0.23185259	0.097516775
Gglean014626	0.223709587	0.104009517	0.23185259	0.097516775
Gglean014627	0.223709587	0.104009517	0.23185259	0.097516775
Gglean014628	0.113288124	0.10530928	0.018319379	0.03321246
Gglean014629	0.113288124	0.10530928	0.018319379	0.03321246
Gglean014630	0.113288124	0.10530928	0.018319379	0.03321246
Gglean014631	0	0	0	0
Gglean014632	0	0	0	0
Gglean014633	9.252938	7.6308902	6.2472905	5.061657
Gglean014634	9.252938	7.6308902	6.2472905	5.061657
Gglean014635	0	0.120359737	0.0643877	0.0762731
Gglean014636	18.40179006	21.68074032	29.1858493	32.40463
Gglean014637	133.7001	117.1852	83.5285	75.0878
Gglean014638	0.0320178	0.065755993	0.026009113	0
Gglean014639	0	0.0823894	0	0
Gglean014640	0	0	0	0
Gglean014641	46.263345	44.787216	48.3876913	43.030914
Gglean014642	0	0	0	0
Gglean014643	5.87557	5.0677	3.67487	3.35799
Gglean014644	18.64796305	17.66468527	27.8089607	31.89921498
Gglean014645	18.64796305	17.66468527	27.8089607	31.89921498
Gglean014646	7.081030226	8.666813847	6.892426	11.181194
Gglean014647	7.50E-14	1.52E-06	2.80E-05	2.47E-07
Gglean014648	52.2505	53.99296	41.05934	36.97239
Gglean014649	47.88471247	49.460264	53.463945	43.28626601
Gglean014650	47.88471247	49.460264	53.463945	43.28626601
Gglean014651	47.88471247	49.460264	53.463945	43.28626601

Gglean014652	47.88471247	49.460264	53.463945	43.28626601
Gglean014653	0.149389847	0.25124603	0.265098	0.581917606
Gglean014654	0	0	0	0
Gglean014655	0	0	0	0
Gglean014656	3.0027378	3.370127892	4.26847	3.6539
Gglean014657	3.0027378	3.370127892	4.26847	3.6539
Gglean014658	10.97335859	11.42692964	15.53966974	19.16637343
Gglean014659	21.54046263	22.90886259	19.66458569	18.66057085
Gglean014660	21.54046263	22.90886259	19.66458569	18.66057085
Gglean014661	0.0389731	0.0807507	0.0945714	0.0372448
Gglean014662	0.145033	0	0	0
Gglean014663	0	0	0	0
Gglean014664	6.44912	6.52968	5.64924	5.45555
Gglean014665	1.0045	1.51E-16	8.55E-43	4.58E-74
Gglean014666	0.228984	0.233675	0.231522	0.220267
Gglean014667	0.228984	0.233675	0.231522	0.220267
Gglean014668	9.83587	8.00257	10.4878	9.40578
Gglean014669	0	0.517319	0	0
Gglean014670	47.9846	48.0085	69.0296	70.1935
Gglean014671	0	0	0	0
Gglean014672	70.775836	93.169321	85.162939	73.184857
Gglean014673	15.199	18.1854	11.5646	11.458
Gglean014674	6.259788	7.96670247	9.030931	9.70181642
Gglean014675	0.169368	0.403412	0.163756	0.310837
Gglean014676	4.42351	6.27451	6.905	7.73496
Gglean014677	25.60474647	28.66507807	35.59203637	46.74474473
Gglean014678	97.1742	95.994204	121.67606	191.9570365
Gglean014679	2.67E-121	2.52E-117	5.44E-274	0
Gglean014680	2.03223	4.50373	3.60988	2.82057
Gglean014681	6.69011	7.15725	11.2464	12.6231
Gglean014682	27.81466	25.73556	16.22835	13.60543
Gglean014683	1.865696	1.997250135	1.674894336	1.317703
Gglean014684	18.56332	15.08208	23.80876	26.56231
Gglean014685	0	0	0	0
Gglean014686	0	0.0572405	0.133146	0
Gglean014687	1.328469794	1.170822587	1.500249836	1.612110051
Gglean014688	0	0	0	0
Gglean014689	0.269535711	0.19043742	0.613911098	0.437346355
Gglean014690	0.269535711	0.19043742	0.613911098	0.437346355
Gglean014691	0.641382	1.002288	0.960577	1.416678
Gglean014692	0	0	0	0
Gglean014693	0.464125	0.499388	0	0.227703
Gglean014694	36.611756	37.845742	51.006528	59.7457922
Gglean014695	1.0880171	1.739550398	2.086209812	2.769417512

Gglean014696	24.69669366	25.754181	29.67799572	32.4653577
Gglean014697	0.14125	0.00070174	1.30454	1.18E-05
Gglean014698	0.129539	0	0	0.12669
Gglean014699	49.115	46.65847	79.4653	80.0703
Gglean014700	28.5634	30.0026	31.5549	60.204
Gglean014701	38.0388	34.29952	23.36964	21.79075
Gglean014702	163.17283	179.3257	113.358	106.03299
Gglean014703	10.68673791	10.64390055	11.706699	11.80877585
Gglean014704	20.86964943	24.72367617	21.79472264	19.50108953
Gglean014705	0	0	0	0
Gglean014706	0	0	0	0
Gglean014707	20.86964943	24.72367617	21.79472264	19.50108953
Gglean014708	42.44612	43.02139027	30.74040016	26.57923002
Gglean014709	6.48521	6.583685	9.257107	11.66533786
Gglean014710	42.6791	45.9141	15.23589	11.45104
Gglean014711	0	0	0	0
Gglean014712	3.496989668	4.140617	2.7067611	3.31093226
Gglean014713	0	0	0	0
Gglean014714	0	0	0	0
Gglean014715	4.81447	4.466576	3.184354	4.23371
Gglean014716	0	0	0	0
Gglean014717	30.3128218	23.49894049	18.4580719	16.53530537
Gglean014718	0	0	0	0
Gglean014719	13.78777	13.04402765	15.85113294	19.59119003
Gglean014720	23.17131	20.99023	21.98023	24.67374
Gglean014721	110.0740027	125.3850224	155.8610094	154.6880089
Gglean014722	1.17685	0.946439	2.45288	2.17048
Gglean014723	131.119	126.703	110.36	96.71
Gglean014724	10.36948	13.980059	4.498313	8.899835
Gglean014725	32.80894	35.05522	25.16109	23.32169
Gglean014726	32.80894	35.05522	25.16109	23.32169
Gglean014727	0	0	0	0
Gglean014728	0	0.123273	0	0
Gglean014729	29.5539	36.1968	24.39522	24.75426
Gglean014730	25.2873	27.2369	20.5781	19.5804
Gglean014731	32.955614	31.399341	27.764977	26.513545
Gglean014732	7.560340554	9.582198126	5.834012618	5.170957931
Gglean014733	0	0	0	0
Gglean014734	0.84441	1.03426	1.70148	1.76247
Gglean014735	0	0	0	0
Gglean014736	0.379454815	0.629958194	0.847800619	0.47116475
Gglean014737	0.379454815	0.629958194	0.847800619	0.47116475
Gglean014738	0.257059	0	0.733277	0.37611
Gglean014739	4.5288345	1.918219441	3.040532809	3.039776975

Gglean014740	4.5288345	1.918219441	3.040532809	3.039776975
Gglean014741	4.5288345	1.918219441	3.040532809	3.039776975
Gglean014742	125.3094	118.7691	125.4086	124.847
Gglean014743	44.11378	46.0375	41.7189	36.61057
Gglean014744	16.47635001	18.486271	6.460829546	5.665661406
Gglean014745	1788.47628	1747.01964	1167.63566	1100.739007
Gglean014746	14.55138	14.98756	24.106964	26.065373
Gglean014747	88.77758578	85.59437818	140.0275629	115.6572029
Gglean014748	0.118195	0.0616377	0.193264	0
Gglean014749	0	0	0	0
Gglean014750	0	0	0	0
Gglean014751	0	0.0818321	0	0
Gglean014752	0	0	0	0.02578089
Gglean014753	30.13722599	27.55993675	31.87954646	30.08699175
Gglean014754	30.13722599	27.55993675	31.87954646	30.08699175
Gglean014755	68.7234	67.6135922	59.2859	48.05587
Gglean014756	42.05727	45.59045	51.83	50.76426
Gglean014757	8.02809896	7.59968085	11.8199	12.34226476
Gglean014758	8.02809896	7.59968085	11.8199	12.34226476
Gglean014759	81.68487	85.5859	38.53656	28.52905
Gglean014760	2.97688	2.808244	1.913072	2.012325
Gglean014761	83.50765	114.59253	73.95809	77.72703
Gglean014762	0	0	0	0
Gglean014763	0	0	0	0
Gglean014764	0.649447	0.440524	0.637359	0.240539
Gglean014765	47.14592328	48.85934645	51.99937993	47.73029264
Gglean014766	21.60947	17.94962594	30.33062841	30.275122
Gglean014767	29.9810608	32.630706	45.264495	50.48357
Gglean014768	98.6351	102.5690018	115.658	114.1245
Gglean014769	15.187159	16.569788	16.152634	16.22227201
Gglean014770	15.187159	16.569788	16.152634	16.22227201
Gglean014771	15.187159	16.569788	16.152634	16.22227201
Gglean014772	29.9218006	29.09420074	36.06318	37.365598
Gglean014773	0.820886	1.121560939	0.602505	0.81633446
Gglean014774	13.777168	16.623546	3.44367	3.597801
Gglean014775	22.889487	25.36848	16.31218	12.98502
Gglean014776	13.564113	15.8659	14.262743	14.41413
Gglean014777	533.8963592	577.11058	700.9573072	914.4173378
Gglean014778	55.02313654	60.67498	81.87421091	85.87270969
Gglean014779	5.625522808	5.667839787	5.593318334	9.317754657
Gglean014780	5.625522808	5.667839787	5.593318334	9.317754657
Gglean014781	0	0	0	0
Gglean014782	0	0	0	0
Gglean014783	0	0.128889	0	0

Gglean014784	0	0	0	0
Gglean014785	0	0	0	0
Gglean014786	21.80707	28.29873	11.4467	10.19124
Gglean014787	200.47244	245.41946	276.78267	270.15994
Gglean014788	200.47244	245.41946	276.78267	270.15994
Gglean014789	26.0326	26.4644	16.3742	15.17743
Gglean014790	20.15575799	20.09445032	27.09724082	28.17113688
Gglean014791	0.143452107	0.0496064	0.42767213	0.229901122
Gglean014792	173.0437	140.3547	93.9909	94.48250002
Gglean014793	1879.174	2498.86	1153.948	1031.643
Gglean014794	7.985008909	9.658453382	4.528917118	3.8826005
Gglean014795	1.18759	1.88986	2.24969	2.82776
Gglean014796	0.988016	0.907442	2.09675	1.94209
Gglean014797	43.4324	49.0365	45.7871	42.2585
Gglean014798	28.85038	32.36918	23.19344	20.70246
Gglean014799	67.39590601	76.0637947	48.28216586	46.35318939
Gglean014800	67.39590601	76.0637947	48.28216586	46.35318939
Gglean014801	0	0	0	0
Gglean014802	67.39590601	76.0637947	48.28216586	46.35318939
Gglean014803	67.39590601	76.0637947	48.28216586	46.35318939
Gglean014804	10.7267	10.6786	11.3082	16.469
Gglean014805	0.521046	0.983507	0	0
Gglean014806	65.7652	77.41	57.6716	60.7923
Gglean014807	18.4562797	18.5963374	28.30509919	33.0627129
Gglean014808	0	0	0.214656	0.127567
Gglean014809	15.055701	14.257359	6.1104211	4.521102
Gglean014810	0	0	0	0
Gglean014811	15.82080004	17.71255369	19.51724572	20.22823343
Gglean014812	83.6567	81.41365	83.79808	79.64802
Gglean014813	0	0.564959	0.218581	0.129172
Gglean014814	29.78398	24.91524	25.74143	25.21763
Gglean014815	47.76283	47.1926	44.8428	51.0428
Gglean014816	47.76283	47.1926	44.8428	51.0428
Gglean014817	1.488022229	0.809515	1.84532	2.315865081
Gglean014818	1.488022229	0.809515	1.84532	2.315865081
Gglean014819	0	0	0	0
Gglean014820	0	0	0	0
Gglean014821	0.1257388	0.104260325	0.0273415	0.096805002
Gglean014822	54.73373707	67.944815	41.770027	36.61154752
Gglean014823	2.425243988	3.42946351	2.466472792	1.888649
Gglean014824	2.97364	2.81054	2.555052614	1.35068
Gglean014825	49.8679	50.9298	40.14543	40.06002
Gglean014826	49.8679	50.9298	40.14543	40.06002
Gglean014827	11.8700071	9.4997723	14.26673317	17.1950068

Gglean014828	3.20780014	3.419425	2.338820707	2.621080119
Gglean014829	0.934319	0.615396003	0.947188	0.833248
Gglean014830	0.0438033	0	2.83E-110	0
Gglean014831	69.6104	63.3803	48.7742	34.9384
Gglean014832	20.8326	24.2406	22.3024	23.7878
Gglean014833	89.801522	90.83645783	75.15763344	65.6428912
Gglean014834	0.489457646	0.39176544	0.53154635	0.30092831
Gglean014835	43.44557602	46.63647561	72.16394803	76.9970884
Gglean014836	43.44557602	46.63647561	72.16394803	76.9970884
Gglean014837	43.44557602	46.63647561	72.16394803	76.9970884
Gglean014838	53.4721	55.0815	49.9347	48.3965
Gglean014839	0	0	0	0
Gglean014840	0	0	0	0
Gglean014841	73.12544077	75.14506644	103.96279	102.60927
Gglean014842	0	0	0	0.243049
Gglean014843	9.63218	10.1488	7.02468	6.26686
Gglean014844	9.72776	10.6799	6.35023	6.41209
Gglean014845	106.235	99.1955	102.538	91.0993
Gglean014846	22.522148	21.2693762	24.24318444	20.15934023
Gglean014847	22.522148	21.2693762	24.24318444	20.15934023
Gglean014848	10.6368	13.9591	6.56665	5.285
Gglean014849	11.6113	12.7181	5.84953	5.30738
Gglean014850	4.31515	4.725195	7.25582	7.79759
Gglean014851	0.239385526	0.5151557	0.1922555	0.252205
Gglean014852	0.239385526	0.5151557	0.1922555	0.252205
Gglean014853	53.35415	63.65499276	38.70388084	35.11047055
Gglean014854	5.543260433	5.391364606	4.722552386	5.882452002
Gglean014855	5.543260433	5.391364606	4.722552386	5.882452002
Gglean014856	0.757836043	1.158380002	1.164920031	1.28057
Gglean014857	2.411975823	2.027719358	0.76108475	0.891373107
Gglean014858	2.411975823	2.027719358	0.76108475	0.891373107
Gglean014859	0.279338	0	0.0573879	0
Gglean014860	0.047238	0	0	0
Gglean014861	81.98414	68.65753	152.7081	132.99863
Gglean014862	81.98414	68.65753	152.7081	132.99863
Gglean014863	81.98414	68.65753	152.7081	132.99863
Gglean014864	30.89773967	32.67045504	31.00215501	32.53338821
Gglean014865	8.54504	7.22968	9.30989	12.10144
Gglean014866	0	0	0	0
Gglean014867	7.979907	10.91507	4.76062	4.64544
Gglean014868	5.14609	4.85664	3.8024	4.01816
Gglean014869	4.077860513	2.66609	3.80138	2.42788
Gglean014870	54.46861	61.0663	43.89022	42.22224
Gglean014871	58.5506153	62.13653769	61.35798923	61.08362309

Gglean014872	0.769785614	0.96212977	0.496103	0.554388
Gglean014873	1.98458	12.3678	0.407681	0
Gglean014874	219.0765	223.742	247.8964	276.3165
Gglean014875	22.0926	22.3126	15.3781	14.9645
Gglean014876	1.38599	3.86429	1.14533	4.38506
Gglean014877	2.3167143	1.736403	1.556436	0.575844
Gglean014878	12.634207	12.029622	17.971798	25.997096
Gglean014879	0.868717	0.128155	0.102191	0.243589
Gglean014880	18.78233	16.27192	12.80865	13.7698
Gglean014881	14.14908	14.68864	10.14011	7.43462
Gglean014882	138.74959	146.73326	143.81723	146.15893
Gglean014883	14.80817165	16.51061025	11.60782897	10.0911654
Gglean014884	14.80817165	16.51061025	11.60782897	10.0911654
Gglean014885	0.802038	0.780059	1.05428	1.45005
Gglean014886	0.120341	0.0624558	0.0488217	0.115259
Gglean014887	4.10425	3.263406948	2.023423815	1.732110147
Gglean014888	5.70744	4.14592	6.85417	4.65863
Gglean014889	0	0	0	0
Gglean014890	0	0	0	0
Gglean014891	8.635048	7.710678	11.465491	14.4732
Gglean014892	0	0	0	0
Gglean014893	0	0	0	0
Gglean014894	0	0	0	0
Gglean014895	250.74092	254.83241	221.93392	224.63319
Gglean014896	250.74092	254.83241	221.93392	224.63319
Gglean014897	0	0	0	0
Gglean014898	11.98323	11.50463	5.112253	4.60205
Gglean014899	22.3353	26.5965	13.0614	12.8388
Gglean014900	35.23125102	35.95692879	30.45044949	31.26897029
Gglean014901	48.9935	61.2026	5.51575	2.00273
Gglean014902	39.80632761	44.20738	25.362908	21.3843376
Gglean014903	0	0	0	0
Gglean014904	12.62414	15.35769	6.65348	5.63541592
Gglean014905	12.62414	15.35769	6.65348	5.63541592
Gglean014906	0	0	0	0
Gglean014907	0	0	0	0
Gglean014908	3.43286168	2.72515002	3.708581	3.823039
Gglean014909	0	0	0	0
Gglean014910	22.779	23.0354004	14.13890497	11.82400391
Gglean014911	12.3049	16.6236	27.0148	18.5628
Gglean014912	67.40461218	65.478579	51.45160454	49.3706033
Gglean014913	67.40461218	65.478579	51.45160454	49.3706033
Gglean014914	67.40461218	65.478579	51.45160454	49.3706033
Gglean014915	0.459748	0.117678	0.651357	0.33345

Gglean014916	0.0720055	0	0.581045	0.557777
Gglean014917	0	0.471017	0.859329	0.642264
Gglean014918	0.143024	0.319909	0.43704	0.465594
Gglean014919	4.288429	3.600074425	3.063866	2.514089115
Gglean014920	8.72711	7.713766	6.192970787	5.649989
Gglean014921	45.89796104	44.678881	41.80266582	38.69431501
Gglean014922	59.4977654	57.51551101	98.330764	103.493752
Gglean014923	37.4615	42.5169	32.0523	27.4615
Gglean014924	8.31981	4.98439	3.95932	4.09003
Gglean014925	3.440656	2.203463	4.715163	5.884542
Gglean014926	0	0	0	0
Gglean014927	42.472129	45.8966	35.885227	35.169281
Gglean014928	79.7807942	82.49788753	87.8246843	96.00930993
Gglean014929	79.7807942	82.49788753	87.8246843	96.00930993
Gglean014930	1.99E-41	9.42E-14	4.49E-62	1.12817
Gglean014931	0.879581	1.14864	0.899309	0.802924
Gglean014932	8.71302	10.21512	2.967341	2.544847
Gglean014933	223.181	203.105	301.254	322.445
Gglean014934	0.296956	0	1.2305	0.885731
Gglean014935	312.316	283.333	270.958	409.137
Gglean014936	0	0	0	0
Gglean014937	0.0373057	0.0770093	0.150757	0.0713629
Gglean014938	0.000291377	0.531096	0.0677126	0.0995613
Gglean014939	7.02E-05	0.000118963	0.000222653	0.382978
Gglean014940	0.28496	0	0	0.136682
Gglean014941	7.5337	7.39729	4.66124	3.86356
Gglean014942	6.371634	5.86997	3.503908	3.41851
Gglean014943	2.622875605	5.335564	2.880333	1.818142342
Gglean014944	2.622875605	5.335564	2.880333	1.818142342
Gglean014945	2.622875605	5.335564	2.880333	1.818142342
Gglean014946	0.776618	0.382434	0.269448	0.461263
Gglean014947	0	0	0	0
Gglean014948	0	0	0	0
Gglean014949	8.75838654	12.61680267	17.40655837	13.888162
Gglean014950	8.75838654	12.61680267	17.40655837	13.888162
Gglean014951	17.86418505	21.06370413	27.14053071	28.62192116
Gglean014952	0.525469	1.231097	1.178462	0.380368
Gglean014953	25.87082118	26.85098614	26.75462554	26.78655153
Gglean014954	200.634603	167.8283	265.236352	291.452259
Gglean014955	200.634603	167.8283	265.236352	291.452259
Gglean014956	200.634603	167.8283	265.236352	291.452259
Gglean014957	200.634603	167.8283	265.236352	291.452259
Gglean014958	200.634603	167.8283	265.236352	291.452259
Gglean014959	16.484653	17.7532	14.247669	13.7514

Gglean014960	51.52792281	51.25501829	69.1916099	73.84234549
Gglean014961	22.9933	22.86835	15.387225	17.82970306
Gglean014962	0.807682	0.910922	0.685014	0
Gglean014963	7.4032439	7.355186	5.249453	5.1174212
Gglean014964	1.34985	4.10E-05	0.750633	0.300962
Gglean014965	39.62981807	40.86016316	29.24663177	25.63877054
Gglean014966	11.43799747	11.70840674	5.628107788	4.139245
Gglean014967	11.43799747	11.70840674	5.628107788	4.139245
Gglean014968	0.0674474	0.0345643	0	0.0323359
Gglean014969	0.074992	0.232265	0.0613682	0.0728515
Gglean014970	3.29613	4.30491	4.76481	5.99266
Gglean014971	11.679493	14.37711019	17.3240003	16.770698
Gglean014972	33.460996	30.17967	34.521636	39.61643242
Gglean014973	30.87485	30.20187	13.75104	13.933285
Gglean014974	0.177518366	0.0864296	0	0.32134353
Gglean014975	0.455190178	0.547049	0.830479851	1.373723498
Gglean014976	0.6454998	2.583792901	1.648354644	2.719752684
Gglean014977	0	0	0	0
Gglean014978	0	0	0	0
Gglean014979	29.3036103	31.249526	27.92930007	25.57520241
Gglean014980	0	0	0	0
Gglean014981	20.60839	17.21181	31.05168	34.74945
Gglean014982	2.096177	1.70670953	3.53953	3.89847
Gglean014983	0	0	0	0.0335479
Gglean014984	5.25159	7.97041	3.36217	2.73761
Gglean014985	1.6154	1.28247	2.07697	1.8259771
Gglean014986	0	0	0	0
Gglean014987	34.64411	30.91542	20.2977	15.597852
Gglean014988	16.70211001	16.71565436	31.37460202	32.53401252
Gglean014989	17.2038741	15.971635	22.573945	23.56177001
Gglean014990	33.1303	41.507	40.6615	46.454
Gglean014991	16.7665	12.6141	8.5777	9.18243
Gglean014992	25.99476586	25.307379	28.47603	26.77904864
Gglean014993	0	0	0.352028	0
Gglean014994	3.31356	3.50609	4.84816	5.72003
Gglean014995	0.100122	0.154329	0	3.59E-50
Gglean014996	2.91036	5.8418	6.14416	2.33616
Gglean014997	243.807	169.601	149.139	221.501
Gglean014998	103.7576464	93.79038388	155.2739957	158.2847274
Gglean014999	103.7576464	93.79038388	155.2739957	158.2847274
Gglean015000	0	0	0.0469836	0
Gglean015001	86.5619	71.9243	20.9674	9.88863
Gglean015002	7.305224433	9.133082893	1.542295787	1.428205306
Gglean015003	33.952594	31.448456	36.329469	37.127331

Gglean015004	18.1824	16.1952	7.48114	6.90255
Gglean015005	21.8992854	19.88079974	30.29528481	29.02027
Gglean015006	4.18533477	4.844741513	5.983781126	7.036101965
Gglean015007	2.252668392	2.473355	3.2297556	2.440416941
Gglean015008	2.984886	2.89367439	3.247781	4.616774
Gglean015009	0	0.0639804	0.0996221	0.10615
Gglean015010	0	0.0510215	0.0796081	0.0603049
Gglean015011	67.862621	78.14312	61.31755	79.9294
Gglean015012	67.862621	78.14312	61.31755	79.9294
Gglean015013	16.62937	17.72863173	15.37061	15.50611
Gglean015014	16.62937	17.72863173	15.37061	15.50611
Gglean015015	30.5789	34.673	33.5659	34.4234
Gglean015016	0	0.0257567	0	0.0961244
Gglean015017	11.6402	11.629085	13.910254	14.95418
Gglean015018	17.27668	15.024234	19.617169	21.700106
Gglean015019	17.27668	15.024234	19.617169	21.700106
Gglean015020	20.94789	22.189756	11.70005	11.79285
Gglean015021	0	0	0	0
Gglean015022	6.23E-07	1.61E-05	0.121312	0.324969
Gglean015023	6.50E-07	3.25E-06	0	0
Gglean015024	0.389121	0.8654	0.620649	0.625332
Gglean015025	0.0877628	0	0.214798	0.0845763
Gglean015026	23.81731815	24.54399183	16.9586759	16.00352664
Gglean015027	12.116346	12.52501	8.435961	8.05066
Gglean015028	0.274994	0.287319	0.224367	0.532383
Gglean015029	0	0	0	0
Gglean015030	50.37052056	43.44813236	55.21589056	52.14644434
Gglean015031	0.0102323	0.0510195	0.0411372	0.0197351
Gglean015032	0	0	0	0
Gglean015033	15.7063	16.870923	9.536412743	7.663892151
Gglean015034	28.44708515	25.703968	60.29973	52.706224
Gglean015035	5.81137	7.748820911	8.067473623	13.11890846
Gglean015036	19.99569	16.94865	7.77642	5.515586
Gglean015037	32.79325912	33.39996785	32.06653045	29.84938339
Gglean015038	32.79325912	33.39996785	32.06653045	29.84938339
Gglean015039	43.13647396	42.44144827	45.01797069	47.76483731
Gglean015040	30.9497	20.18859	31.2872	18.72933
Gglean015041	16.6047	17.2175	12.4737	12.6892
Gglean015042	157.426	147.194	60.5298	51.6999
Gglean015043	29.680936	30.6609	14.471242	14.766587
Gglean015044	29.680936	30.6609	14.471242	14.766587
Gglean015045	29.680936	30.6609	14.471242	14.766587
Gglean015046	0	0	0	0
Gglean015047	17.121303	16.833887	26.75380768	28.75195172

Gglean015048	0.096754	0.3361701	0.527369023	0.239547028
Gglean015049	0.096754	0.3361701	0.527369023	0.239547028
Gglean015050	8.49064314	9.02580459	13.06207812	13.93593683
Gglean015051	0	0	0	0
Gglean015052	0	0	0	0
Gglean015053	114.704	154.076	76.1946	84.5044
Gglean015054	42.294493	53.65286	11.991904	13.00239
Gglean015055	18.276689	18.929616	23.103522	26.545681
Gglean015056	21.96351	24.2687	14.64839	12.63778
Gglean015057	15.5856	16.6554	8.17379	4.7223
Gglean015058	56.15457	57.2846	57.43683	57.06379
Gglean015059	27.8799	31.3909	6.28318	4.35804
Gglean015060	0.000858539	0.000138047	0.884819	0.0256556
Gglean015061	3.599651618	3.16826658	4.0577956	4.377394417
Gglean015062	3.599651618	3.16826658	4.0577956	4.377394417
Gglean015063	3.599651618	3.16826658	4.0577956	4.377394417
Gglean015064	3.599651618	3.16826658	4.0577956	4.377394417
Gglean015065	3.599651618	3.16826658	4.0577956	4.377394417
Gglean015066	27.6643	29.279419	43.48956	43.30652
Gglean015067	27.6643	29.279419	43.48956	43.30652
Gglean015068	145.26796	173.645965	165.8683543	193.0979848
Gglean015069	0.147321	2.24967	1.59127	1.26084
Gglean015070	79.96442761	83.95262447	79.61506105	116.7772511
Gglean015071	77.1418	59.8505	66.1546	89.7606
Gglean015072	79.96442761	83.95262447	79.61506105	116.7772511
Gglean015073	2.86676	1.69927	0.679538	0.242218
Gglean015074	24.1641	25.4213	14.67953	12.9324
Gglean015075	0.56936	0.590444775	0.707291955	0.68357837
Gglean015076	8.92872	9.173787073	11.97932486	13.890538
Gglean015077	8.92872	9.173787073	11.97932486	13.890538
Gglean015078	0	0	0	0
Gglean015079	32.2351	38.8157	23.16866	24.3185
Gglean015080	71.75053092	72.4440764	95.59350183	102.3563652
Gglean015081	71.75053092	72.4440764	95.59350183	102.3563652
Gglean015082	6.74134	7.689220162	6.7373503	20.30535
Gglean015083	0.42401144	0.404564707	0.529057719	0.695750379
Gglean015084	0	0	0	0
Gglean015085	0.0859947	0.0439841	0	0
Gglean015086	0	0	0	0
Gglean015087	0	0	0	0
Gglean015088	0.68578092	0.329557075	0.817653698	0.660734047
Gglean015089	0.257203	0.275082	0	0
Gglean015090	7.49867	7.96782	6.43109	7.7487
Gglean015091	4.16169	2.75251	5.15074	5.55327

Gglean015092	20.60504079	20.9750319	16.13283794	14.40403295
Gglean015093	59.98462358	69.68180163	63.79873865	55.72286
Gglean015094	8.63376458	10.61672775	9.50741	9.94249
Gglean015095	21.08250497	27.53276989	12.34220596	12.43515589
Gglean015096	111.314523	127.33014	72.46344068	66.37185045
Gglean015097	111.314523	127.33014	72.46344068	66.37185045
Gglean015098	80.150919	71.47760652	77.234992	82.5925088
Gglean015099	80.150919	71.47760652	77.234992	82.5925088
Gglean015100	9.91104	8.142175	15.83626	15.72135
Gglean015101	43.14118	43.51058	27.95818	24.11571
Gglean015102	231.3776	203.3109	217.1355	234.9186
Gglean015103	231.3776	203.3109	217.1355	234.9186
Gglean015104	0	0.163145	0	0.153687
Gglean015105	23.3373	24.698	8.31119	7.12608
Gglean015106	1.07632	0.287991	0.222779	0.529981
Gglean015107	163.766713	151.16812	157.1968074	151.1311083
Gglean015108	3.3727135	2.412630571	1.860316362	1.57196711
Gglean015109	0.661293	0.379416	1.539	1.47386
Gglean015110	15.71112063	15.89240456	17.45502506	23.89332603
Gglean015111	21.12699277	21.647141	13.98405	12.81689058
Gglean015112	56.5126317	61.3642597	31.22878942	26.2124562
Gglean015113	11.7234291	13.23655512	22.890183	25.258099
Gglean015114	11.7234291	13.23655512	22.890183	25.258099
Gglean015115	22.25136261	22.22284198	11.16798811	11.95859459
Gglean015116	35.087258	35.2165	24.54715	21.6479689
Gglean015117	3.261743744	2.172407297	1.545310912	1.118227
Gglean015118	3.261743744	2.172407297	1.545310912	1.118227
Gglean015119	0.05571212	0	0.04527604	0
Gglean015120	0.1938488	0.226446199	0.470936504	0.939962843
Gglean015121	0.1938488	0.226446199	0.470936504	0.939962843
Gglean015122	21.697043	20.245182	21.645247	22.796454
Gglean015123	5.629009327	6.333831	4.395791521	3.722671553
Gglean015124	123.0291024	130.9920363	99.15852326	88.23749268
Gglean015125	218.6627	235.825	248.166	234.4288
Gglean015126	28.60864229	29.62716186	27.17537743	25.08215227
Gglean015127	28.60864229	29.62716186	27.17537743	25.08215227
Gglean015128	0	0	0	0
Gglean015129	85.93290409	86.81904	26.8064562	20.59789768
Gglean015130	64.72983521	64.5807844	39.41079	37.62989899
Gglean015131	64.72983521	64.5807844	39.41079	37.62989899
Gglean015132	30.08985332	23.47445707	29.22547162	34.61232554
Gglean015133	27.039561	27.499798	14.50451202	13.261595
Gglean015134	27.039561	27.499798	14.50451202	13.261595
Gglean015135	0	0.0652275	0	0

Gglean015136	33.2086	37.7639	20.73364	21.6446
Gglean015137	19.61354	18.3285	16.35532	17.40432
Gglean015138	32.4411022	30.73177833	32.50008701	32.51545035
Gglean015139	7.05151	8.48067	4.89771	4.02435
Gglean015140	44.39860432	47.83019579	54.5129783	52.55288446
Gglean015141	86.5727	55.435	61.1855	23.8686
Gglean015142	25.465	29.23227	15.01069	12.489693
Gglean015143	182.646	187.87	141.428	137.723
Gglean015144	45.910054	50.692692	36.106281	31.036013
Gglean015145	32.729826	28.725969	29.324111	37.989562
Gglean015146	60.83364	59.55938	45.83713	41.26609
Gglean015147	60.83364	59.55938	45.83713	41.26609
Gglean015148	18.62485	19.36875977	18.88484983	24.1376997
Gglean015149	18.62485	19.36875977	18.88484983	24.1376997
Gglean015150	59.792104	71.11659	82.13082	86.42415
Gglean015151	35.10523	33.24042	29.3906	42.271774
Gglean015152	45.37	36.19777	42.7271	66.4657
Gglean015153	8.203535306	5.68952	8.704063	13.96624251
Gglean015154	73.557889	88.39798	46.8297	35.95697
Gglean015155	51.6005	58.2819	50.8073	56.2765
Gglean015156	46.69885	50.90734	40.20351	41.33496
Gglean015157	11.0495	14.1047	3.49901	2.45334
Gglean015158	14.63653432	16.19622091	19.63317097	23.98212424
Gglean015159	73.99741	65.99763	70.60583	74.86849
Gglean015160	33.58786	28.25126	27.96105	21.68373
Gglean015161	74.2926	70.7018	74.4735	66.8688
Gglean015162	55.914589	58.77960128	28.03490069	24.85900072
Gglean015163	5.71063	4.16488	4.915689	4.75548
Gglean015164	33.95166452	33.51667302	41.53084889	44.98419807
Gglean015165	33.95166452	33.51667302	41.53084889	44.98419807
Gglean015166	33.95166452	33.51667302	41.53084889	44.98419807
Gglean015167	0.479214	1.06776	0	0
Gglean015168	3.2782	2.86278	3.66346	7.30036
Gglean015169	0	0	0	0
Gglean015170	42.97153877	48.81567407	27.3982156	28.15499411
Gglean015171	42.97153877	48.81567407	27.3982156	28.15499411
Gglean015172	30.22627	31.8332	35.10326	34.34866
Gglean015173	16.56741694	17.0528732	17.1485658	15.99604933
Gglean015174	16.56741694	17.0528732	17.1485658	15.99604933
Gglean015175	0.018388402	0	0	0
Gglean015176	0.018388402	0	0	0
Gglean015177	0	0	0	0
Gglean015178	0	0	0	0
Gglean015179	1.290651713	1.587755932	1.770596048	3.2632248

Gglean015180	74.7086	54.7336	24.74185	41.13272
Gglean015181	74.7086	54.7336	24.74185	41.13272
Gglean015182	2.32684	1.10708	1.70923	2.04285
Gglean015183	4.831315898	1.868283788	2.902460719	1.416800702
Gglean015184	0	0	0	0
Gglean015185	26.92855722	26.54581723	23.65117726	21.94162744
Gglean015186	26.92855722	26.54581723	23.65117726	21.94162744
Gglean015187	26.92855722	26.54581723	23.65117726	21.94162744
Gglean015188	47.2054	48.3806	50.003092	49.5928
Gglean015189	121.133	103.496	111.353	105.207
Gglean015190	49.59880062	45.37953376	42.66813	40.167
Gglean015191	2.599311205	1.773732555	1.285192708	2.035116301
Gglean015192	2.599311205	1.773732555	1.285192708	2.035116301
Gglean015193	171.388	215.093	568.405	657.694
Gglean015194	25.289907	25.4755357	23.596649	20.89873444
Gglean015195	5.3408453	5.122687	10.630212	11.35329179
Gglean015196	8.217724	11.50782223	19.031532	17.6711
Gglean015197	0	0	0	0
Gglean015198	16.1486	19.0994	3.95085	3.6692
Gglean015199	4.82E-05	2.36101	3.26E-56	0.64158
Gglean015200	368.92412	367.51093	475.96524	604.558
Gglean015201	0.617197	4.78406	0.510081	0
Gglean015202	157.173	180.485	34.9868	30.1706
Gglean015203	17.37726409	15.63216372	13.38279991	13.68916001
Gglean015204	19.90835812	17.50679341	24.01752647	29.50965219
Gglean015205	0.751401	1.212069246	1.400487	0.250534
Gglean015206	0.751401	1.212069246	1.400487	0.250534
Gglean015207	0.2585997	0.1016222	0.364763053	0.336856
Gglean015208	0.0344628	0	0.0555962	0.0330003
Gglean015209	0	0.113568	0	0
Gglean015210	1.120392791	1.05925023	0.692027	0.87686004
Gglean015211	23.81581564	23.84956	25.575835	25.08663938
Gglean015212	16.28078	17.56348	13.38684	18.56194
Gglean015213	16.28078	17.56348	13.38684	18.56194
Gglean015214	8.518699567	9.569	11.60015	15.64574
Gglean015215	69.68750047	64.33127061	82.41701316	78.11010575
Gglean015216	59.458828	53.07186	38.56219	37.40661046
Gglean015217	1.940353113	1.65597582	1.937823256	2.478615197
Gglean015218	4.765917284	5.990703472	5.685376388	8.21306441
Gglean015219	0	0	0	0
Gglean015220	1.0366157	0.963084	0.1685104	1.650064261
Gglean015221	0	0	0	0
Gglean015222	0	0	0.016871442	0
Gglean015223	0	0	0.016871442	0

Gglean015224	0	0	0	0
Gglean015225	0	0	0	0
Gglean015226	51.5119	59.4232	39.6822	35.3793
Gglean015227	59.7175	64.8653	44.5238	37.1455
Gglean015228	23.65401	21.1552	24.30281	25.17915
Gglean015229	35.78603801	37.94124678	61.42757525	70.69671254
Gglean015230	1.257988	1.3320675	0.999503	1.665276
Gglean015231	12.1777	11.368	17.536	16.864
Gglean015232	35.689431	31.796883	32.152582	31.81713085
Gglean015233	35.689431	31.796883	32.152582	31.81713085
Gglean015234	35.689431	31.796883	32.152582	31.81713085
Gglean015235	67.62195	69.56659	111.6311	102.39491
Gglean015236	0.102677	0.107593	0	0
Gglean015237	0.972318	0	0	0
Gglean015238	22.24871	24.21383	33.86147	35.08868
Gglean015239	22.24871	24.21383	33.86147	35.08868
Gglean015240	0	0.0745963	0	0
Gglean015241	25.35822167	27.8256335	18.52394879	16.97969776
Gglean015242	25.149348	28.185676	28.873473	26.5937761
Gglean015243	1.21677	1.46921	1.49042	0.911853
Gglean015244	0	0	0.348504	0
Gglean015245	51.18341924	55.45404566	46.25326318	43.04940203
Gglean015246	29.86491	31.4241	35.2685	40.8659
Gglean015247	29.86491	31.4241	35.2685	40.8659
Gglean015248	30.37319883	32.529975	42.93019449	48.82489653
Gglean015249	43.4064	30.71180031	25.73740283	25.00360505
Gglean015250	43.4064	30.71180031	25.73740283	25.00360505
Gglean015251	110.17565	111.38887	82.61891	67.05631
Gglean015252	6.213586173	6.708271999	7.133395573	13.01012799
Gglean015253	6.213586173	6.708271999	7.133395573	13.01012799
Gglean015254	6.102621505	6.036946352	7.948556558	9.14456725
Gglean015255	21.3486638	20.62157016	26.39033928	23.84159836
Gglean015256	0	0.11428518	0.0506648	0.01498164
Gglean015257	0	0.11428518	0.0506648	0.01498164
Gglean015258	32.194025	32.749905	32.5940107	30.9829266
Gglean015259	32.194025	32.749905	32.5940107	30.9829266
Gglean015260	485.2881005	438.7258006	502.1753	572.839
Gglean015261	485.2881005	438.7258006	502.1753	572.839
Gglean015262	51.82537	42.04915867	55.4628825	54.141572
Gglean015263	502.6243912	556.0307797	332.30692	301.9588
Gglean015264	10.82194839	12.03069737	15.20524	15.62775
Gglean015265	93.5889	93.8151	75.3	71.5113
Gglean015266	61.557905	59.940242	46.613066	47.406883
Gglean015267	21.44283	20.177094	30.57921	47.75963

Gglean015268	21.44283	20.177094	30.57921	47.75963
Gglean015269	33.14806812	30.82410156	53.95489421	57.28871115
Gglean015270	70.9506	70.8839	40.0104	33.7078
Gglean015271	70.9506	70.8839	40.0104	33.7078
Gglean015272	40.44887722	45.1702746	28.831174	25.35250818
Gglean015273	0.473357	0	0	0
Gglean015274	0	0	0.22362	0
Gglean015275	34.5079	30.26759	28.06744	30.00777
Gglean015276	34.5079	30.26759	28.06744	30.00777
Gglean015277	51.75005786	49.88312896	48.35976	44.80129621
Gglean015278	2.39602	2.5753	0.875019	0.520667
Gglean015279	5.47093	6.60035	3.219111	3.132343
Gglean015280	312.625	292.712	263.654	264.716
Gglean015281	13.15854044	5.6148413	18.05882477	30.10505
Gglean015282	160.4739	180.5376	98.898	85.4559
Gglean015283	244.59892	238.69664	25.567486	25.20822
Gglean015284	0.294903	0.251076372	0.41533	0.1838437
Gglean015285	0	0	0	0
Gglean015286	0	0	0	0
Gglean015287	59.41321	62.1062	44.83148	44.29977
Gglean015288	49.6165	52.31527	72.37100019	87.73133
Gglean015289	5.236779	6.11262	8.40915	10.72891
Gglean015290	5.236779	6.11262	8.40915	10.72891
Gglean015291	49.6165	52.31527	72.37100019	87.73133
Gglean015292	14.078322	18.515492	6.86438	6.543506
Gglean015293	29.60502	28.9428509	28.3835272	28.235889
Gglean015294	27.102996	28.27482727	19.94504482	16.88974855
Gglean015295	43.55614	48.444826	56.908747	77.146034
Gglean015296	0.956455	1.2114	1.93E-06	0
Gglean015297	22.3623	34.5451	24.1128	41.9563
Gglean015298	0	0	0	0
Gglean015299	14.88279	15.46294	13.54617	12.99686
Gglean015300	29.5029	31.43747	16.842688	15.1912
Gglean015301	29.5029	31.43747	16.842688	15.1912
Gglean015302	36.80477	44.23426	9.410863839	8.374687
Gglean015303	0	0	0	0
Gglean015304	8.080264	6.043211832	4.315322778	3.760688964
Gglean015305	27.491953	26.019354	32.395293	31.246647
Gglean015306	41.19475	37.94146	30.4118	37.4185
Gglean015307	41.19475	37.94146	30.4118	37.4185
Gglean015308	24.1051	22.6115	15.3689	14.1677
Gglean015309	41.19475	37.94146	30.4118	37.4185
Gglean015310	167.4602	172.1271	200.9074	268.5043
Gglean015311	167.4602	172.1271	200.9074	268.5043

Gglean015312	7.39454	7.91857	6.43066	5.55857
Gglean015313	48.5583	45.85573	43.9473	40.80835
Gglean015314	0.316434	0.664075	0.945847	0.611213
Gglean015315	0.887474	1.86201	3.623820586	2.37515
Gglean015316	8.010751678	9.450201	6.907442	5.745674665
Gglean015317	18.92191	32.7934	24.95329	18.64651
Gglean015318	47.53762	51.000886	31.0985028	31.44769
Gglean015319	47.53762	51.000886	31.0985028	31.44769
Gglean015320	47.53762	51.000886	31.0985028	31.44769
Gglean015321	100.8404898	77.55869165	113.9662208	112.0266017
Gglean015322	100.8404898	77.55869165	113.9662208	112.0266017
Gglean015323	18.14471	17.08582	17.7403	20.60015
Gglean015324	49.123626	51.91161365	58.86269661	59.709918
Gglean015325	14.92860583	16.595447	27.240417	28.533861
Gglean015326	8.92373901	9.97576432	15.73689069	16.34189714
Gglean015327	8.92373901	9.97576432	15.73689069	16.34189714
Gglean015328	0	0	0	2.69527
Gglean015329	0	0	0	0.228155
Gglean015330	0.303103	0	0.0838327	0.0991277
Gglean015331	0.243677	0	0	0
Gglean015332	11.564019	14.59867	14.64484	16.358908
Gglean015333	0	0	0.0921878	0
Gglean015334	8.38408	6.66967	11.3294	12.8191
Gglean015335	0.0609639	0	0	0
Gglean015336	5.62217	8.64182	12.4975	10.6452
Gglean015337	1.60E-15	1.51678	3.18E-19	4.46207
Gglean015338	0	0	0	0.405894
Gglean015339	0.240426	0.125058	0	0
Gglean015340	0	0	0	0
Gglean015341	0	0	0	0
Gglean015342	0	0	0	0
Gglean015343	22.02201087	23.1448249	41.17667	44.58597431
Gglean015344	44.9121	56.0849	17.5713	16.9594
Gglean015345	23.774154	24.57504	14.01435	9.982189
Gglean015346	0	0	0.31348	0
Gglean015347	0	0	0.063277479	0
Gglean015348	0.154868	0.0797117	0.250091	0
Gglean015349	0.08053278	0	0.114338988	0.130776143
Gglean015350	8.94714	6.24643	1.19359	1.7792
Gglean015351	44.85658	32.69645	24.81976423	29.98714
Gglean015352	44.85658	32.69645	24.81976423	29.98714
Gglean015353	3.1014764	7.848816	4.173549	7.936252
Gglean015354	3.1014764	7.848816	4.173549	7.936252
Gglean015355	0.679152	0.139687	0.879749	1.193465

Gglean015356	46.04836	51.78866	35.99574	35.09436
Gglean015357	148.31595	164.1108	102.34733	89.73284
Gglean015358	6.231051298	7.069254	6.778653	9.033784032
Gglean015359	147.4480947	154.64652	98.45296901	83.316919
Gglean015360	147.4480947	154.64652	98.45296901	83.316919
Gglean015361	0	0	0	0
Gglean015362	16.335792	14.840253	26.38846	23.84793
Gglean015363	17.74394919	16.546461	9.55505625	8.82409
Gglean015364	28.840209	29.77805105	8.362344504	6.91516254
Gglean015365	0	0	0	0
Gglean015366	19.2102	16.851137	29.890223	30.167064
Gglean015367	0.114451	0	0	0
Gglean015368	0.441194	0.262859224	0.540353363	0.749222
Gglean015369	0.441194	0.262859224	0.540353363	0.749222
Gglean015370	0.370747	0.15218	0.239574	0.49682
Gglean015371	1153.08686	1187.24281	787.11128	748.2079
Gglean015372	41.1197	49.9445	40.2072	38.99
Gglean015373	0	0	0	0
Gglean015374	67.24864504	77.83953029	67.16652012	56.13345129
Gglean015375	67.24864504	77.83953029	67.16652012	56.13345129
Gglean015376	59.35664	54.841581	41.986752	46.390356
Gglean015377	59.35664	54.841581	41.986752	46.390356
Gglean015378	173.53663	181.6257383	143.6327369	122.443244
Gglean015379	0.30011	0.73322	0.393888	0.789783
Gglean015380	18.79646	21.03289	18.0843	19.00001
Gglean015381	18.79646	21.03289	18.0843	19.00001
Gglean015382	15.44176	14.57820735	23.45488953	27.13152509
Gglean015383	2.38118	0	3.91466	0
Gglean015384	0	0	0	0
Gglean015385	0	0	0	0
Gglean015386	24.66711	22.23738	14.10599	14.46958
Gglean015387	7.816496362	7.34361418	7.519252557	10.73381148
Gglean015388	7.816496362	7.34361418	7.519252557	10.73381148
Gglean015389	0.0880889	0.447689	3.17E-07	7.91E-08
Gglean015390	0.949432	0.706058	0.212127	0.301025
Gglean015391	22.8875329	25.388585	32.966924	28.4263514
Gglean015392	33.36501735	32.20114623	33.65153006	34.30208091
Gglean015393	8.96951	9.47247	4.80446	3.34033
Gglean015394	7.25836	8.21221	3.22016	2.25994
Gglean015395	98.71981406	117.318012	94.33291953	87.5739596
Gglean015396	98.71981406	117.318012	94.33291953	87.5739596
Gglean015397	0.833045	0.824185	1.37405	1.1051
Gglean015398	3.02543	2.18703	2.74444	2.94149
Gglean015399	51.102406	50.941431	60.23801	59.731458

Gglean015400	51.102406	50.941431	60.23801	59.731458
Gglean015401	51.102406	50.941431	60.23801	59.731458
Gglean015402	20.2539	15.60508	11.31553	10.71245
Gglean015403	11.69626	10.72191	13.96277	17.10234
Gglean015404	2.370036	3.9508	4.96399	5.66689
Gglean015405	2.940879	5.75458	3.304231623	6.962057
Gglean015406	3.08403	2.795651	2.185693	2.047412
Gglean015407	0.957742378	1.261482936	0.690153471	1.113615565
Gglean015408	0.957742378	1.261482936	0.690153471	1.113615565
Gglean015409	0.361106	0.275943	0.0963205	0.276328
Gglean015410	14.058203	13.04734	8.52035	8.482035
Gglean015411	21.28631	20.66018227	20.83655	23.55510637
Gglean015412	21.28631	20.66018227	20.83655	23.55510637
Gglean015413	15.44835	15.97719	24.9659	25.10818
Gglean015414	0	0	0.7484	0.897668
Gglean015415	86.4161	74.0521	165.954	177.3462
Gglean015416	0	0.123173	0	0
Gglean015417	2.174204121	3.615746084	1.386663406	2.953019874
Gglean015418	2.174204121	3.615746084	1.386663406	2.953019874
Gglean015419	10.71879	10.81166	5.82865	3.625445
Gglean015420	10.71879	10.81166	5.82865	3.625445
Gglean015421	0	0	0	0
Gglean015422	10.9297	8.57754	6.68588	5.56836
Gglean015423	0	0	0	0
Gglean015424	0.3858901	0.241213751	0.0268412	0.742263232
Gglean015425	0.3858901	0.241213751	0.0268412	0.742263232
Gglean015426	15.5865	16.5119	21.5257	24.818
Gglean015427	0	0	0	0
Gglean015428	0	0	0	0
Gglean015429	0	0	0	0
Gglean015430	0	0	0	0
Gglean015431	0.0908457	0.515783435	0.0471316	0.21688
Gglean015432	1.125076036	0.8497166	0.5634553	0.272615
Gglean015433	1.125076036	0.8497166	0.5634553	0.272615
Gglean015434	0	0	0	0
Gglean015435	0	0	0.526063	0
Gglean015436	2.783686	3.063538	3.087724	3.577637
Gglean015437	23.6026	23.8517	12.8994	9.84145
Gglean015438	10.16870018	10.28100006	13.1022258	18.86080007
Gglean015439	0.166901	0	0	0.080843
Gglean015440	2.1975047	3.80855393	1.6333383	1.392046418
Gglean015441	4.730484	5.622692555	5.045406	4.996267
Gglean015442	0.740188	0.465345	0.871042	1.30949
Gglean015443	0	0	0	0

Gglean015444	0	0.114242	0	0
Gglean015445	23.21532	24.422384	27.951314	28.63586
Gglean015446	0.104006	0	0.0268801	0
Gglean015447	1.01786	1.32239	0.640676	0.867865
Gglean015448	0	0	0	0
Gglean015449	1.35741	1.82882	1.27331	1.37168
Gglean015450	0.561013	0.68492	0.648674	0.972008
Gglean015451	0.596117901	1.4016698	1.380549	1.514443
Gglean015452	0.596117901	1.4016698	1.380549	1.514443
Gglean015453	0.741981	2.29575	1.09004	0.961046
Gglean015454	0	0	0	0
Gglean015455	5.118481347	6.041001327	6.59283173	10.12213842
Gglean015456	0	0.274649	0	0
Gglean015457	35.477645	33.1147	33.356745	32.2322
Gglean015458	12.0599	11.48153	8.24055	8.43616
Gglean015459	0.399997	0.286025	4.95E-84	1.05E-125
Gglean015460	0.2218757	0.236447	1.450022	0.748199
Gglean015461	0.114533	0.0595682	0.697336	0.441459
Gglean015462	31.26	36.33432	30.959183	25.34363
Gglean015463	31.26	36.33432	30.959183	25.34363
Gglean015464	31.26	36.33432	30.959183	25.34363
Gglean015465	11.909738	14.11352	11.779363	9.96729001
Gglean015466	1.50808	0.653038	0.838434	0.0900874
Gglean015467	21.33381197	23.66304613	40.44696055	39.75007002
Gglean015468	0	0	0	0
Gglean015469	0	0	0	0
Gglean015470	0	0	0	0
Gglean015471	0.157638	0	0.0427054	0.0505178
Gglean015472	0	0	0.0398934	0
Gglean015473	0.0753366	0	0.0609448	0.0721254
Gglean015474	5.24474	3.58704	3.32262	2.72881
Gglean015475	0.144694	0	0	0
Gglean015476	0	0	0	0
Gglean015477	0	0	0	0.60778
Gglean015478	0.136958	0	0	0
Gglean015479	0	0	0	0
Gglean015480	0	0	0	0
Gglean015481	0.11978219	0.538515	1.22414578	2.347542
Gglean015482	0.093803	0.0981822	0.154093	0
Gglean015483	0	0.15584	0	0
Gglean015484	44.56586588	43.27263882	64.1640547	68.55474161
Gglean015485	4.25921	4.49144	2.95337	2.626
Gglean015486	34.2210452	42.1298473	62.4332147	64.4515906
Gglean015487	34.2210452	42.1298473	62.4332147	64.4515906

Gglean015488	49.02804248	54.33097	64.54717344	60.93846204
Gglean015489	8.08489	11.8677	3.93203	4.3554
Gglean015490	49.837681	49.63254	45.54785059	45.82619
Gglean015491	49.837681	49.63254	45.54785059	45.82619
Gglean015492	231.90817	219.54114	289.67893	268.83475
Gglean015493	6.5441	6.26818	5.42528077	6.75174
Gglean015494	0.38975	0.201902	0.158913	0.189774
Gglean015495	0.747528	0.930371	1.34153	1.00876
Gglean015496	11.33436	14.24286	22.8751	27.99434
Gglean015497	11.33436	14.24286	22.8751	27.99434
Gglean015498	0	0	0.0275788	0
Gglean015499	1.75563	1.14701	0.628025	0.262468
Gglean015500	18.46770839	18.03065641	13.22652036	12.05025692
Gglean015501	0	0	0	0
Gglean015502	14.79291196	12.36974361	16.08465464	20.74875838
Gglean015503	0.0062196	1.71E-05	3.46265	0.00353932
Gglean015504	18.57661834	19.43114704	10.46402252	8.320866012
Gglean015505	614.0267	645.757031	242.64208	193.2423552
Gglean015506	142.1648218	119.921191	149.612166	138.28794
Gglean015507	2.08435	2.48013	2.19983	1.83604
Gglean015508	142.1648218	119.921191	149.612166	138.28794
Gglean015509	142.1648218	119.921191	149.612166	138.28794
Gglean015510	54.90031941	60.23773	66.60065	62.54747
Gglean015511	7.86684	11.4523	0.619853	0.133673
Gglean015512	57.0483092	55.52201	39.51429	33.7273
Gglean015513	28.11574676	28.82276485	33.9941753	44.82459774
Gglean015514	28.11574676	28.82276485	33.9941753	44.82459774
Gglean015515	0	0	0	0
Gglean015516	0	0	0	0
Gglean015517	0	0	0	0
Gglean015518	47.07615412	43.83946044	41.64150179	32.81700568
Gglean015519	9.854	5.42489	8.90758	16.0177
Gglean015520	0.116085	0	0	0
Gglean015521	0	0	0	0
Gglean015522	0	0	0	0
Gglean015523	75.90367253	80.164066	50.4188254	47.13078609
Gglean015524	75.90367253	80.164066	50.4188254	47.13078609
Gglean015525	75.90367253	80.164066	50.4188254	47.13078609
Gglean015526	26.32242	24.2553	18.3339448	16.406066
Gglean015527	0.00944571	1.44617	1.79436	1.20499
Gglean015528	242.40414	246.8811	285.0595	290.4419
Gglean015529	19.9299	21.5818	10.6696	16.3046
Gglean015530	0.785014	1.13194	0.467553	0.0446966
Gglean015531	58.91406	61.40921	44.48278	39.86935

Gglean015532	2.37583	2.44682	2.7258	3.35693
Gglean015533	1.4638921	0.520474	0.937844	1.9034874
Gglean015534	1.4638921	0.520474	0.937844	1.9034874
Gglean015535	24.0487	25.34346	6.21269	5.7214
Gglean015536	22.7035	25.1899	38.6298	44.4936
Gglean015537	30.83496	29.834471	26.069109	22.513959
Gglean015538	29.90319138	32.517271	40.059069	46.19975
Gglean015539	29.90319138	32.517271	40.059069	46.19975
Gglean015540	29.90319138	32.517271	40.059069	46.19975
Gglean015541	0	0	0.769446	0
Gglean015542	14.93604	14.10823	11.37406	10.50097
Gglean015543	46.72281	48.5455	46.85039	44.22025
Gglean015544	74.7272	72.79096	38.819231	35.51758001
Gglean015545	58.21379	64.47156	51.2672	43.90525
Gglean015546	3.565194	2.71223146	2.252632667	3.02371
Gglean015547	48.371233	50.99966	30.66347	24.60441
Gglean015548	48.371233	50.99966	30.66347	24.60441
Gglean015549	17.68376	21.67586	4.950628	3.959911
Gglean015550	16.6163	13.9174	10.8878	6.37521
Gglean015551	60.2209	64.0952	39.08162	39.42994
Gglean015552	34.5177	47.7618	29.1638	40.4156
Gglean015553	8.462402182	7.998911375	13.21090591	13.91498165
Gglean015554	3.42456	2.79292	4.28921	4.70052
Gglean015555	100.0645517	106.065896	125.4770017	132.2896647
Gglean015556	0	0	0.0246722	0
Gglean015557	0	0	0.0780215	0
Gglean015558	0	0	0	0
Gglean015559	0.308044398	0.557937988	0.433238523	0.328133108
Gglean015560	42.8171	52.63212	76.90755	60.3244275
Gglean015561	42.8171	52.63212	76.90755	60.3244275
Gglean015562	33.07269186	31.88126	37.8	39.7954
Gglean015563	1.383881184	1.66175701	2.3889774	2.855293711
Gglean015564	91.7311	84.952	73.5928	69.8298
Gglean015565	22.28165	24.1752	31.54224	30.84321
Gglean015566	0.625801	0.869387	0.980591	0.903018
Gglean015567	1.48289	1.27791	2.40496	3.00227
Gglean015568	0	0	0	0
Gglean015569	7.9781	7.56912	10.5451	13.2273
Gglean015570	26.7922	33.9474	28.19635	24.89117
Gglean015571	0	0	0	0
Gglean015572	0.518653	0.905418	0.759587	1.60392
Gglean015573	156.31214	146.04281	93.24314	90.4193
Gglean015574	0	0.0740349	0.174561	0
Gglean015575	28.0883	26.0736	23.5214	24.1151

Gglean015576	61.37717	54.85355	69.40043	75.4189
Gglean015577	61.37717	54.85355	69.40043	75.4189
Gglean015578	19.4626	22.2459	17.6465	16.1078
Gglean015579	12.338639	14.573126	9.773494312	8.898853984
Gglean015580	27.4514	31.2261	14.6861	11.8253
Gglean015581	31.358957	28.26518795	19.01471	19.1137139
Gglean015582	57.83327	68.10992	62.81478	63.42472
Gglean015583	27.2789572	27.435319	21.36964363	19.441731
Gglean015584	1.43975	0.741417	1.44858	2.158216
Gglean015585	21.9355	30.4638	8.61136	13.2855
Gglean015586	0.991344	1.5437	1.315207	1.16989
Gglean015587	49.25942076	51.76140471	64.16562033	75.67975566
Gglean015588	82.043347	93.78487	33.052433	29.935529
Gglean015589	20.76411132	23.35702799	20.41851031	21.04213074
Gglean015590	29.95544432	35.47044298	14.87345272	10.68238896
Gglean015591	29.95544432	35.47044298	14.87345272	10.68238896
Gglean015592	81.00203	96.28841	60.1100787	57.05876
Gglean015593	81.00203	96.28841	60.1100787	57.05876
Gglean015594	81.00203	96.28841	60.1100787	57.05876
Gglean015595	74.36422442	75.882275	49.0565395	45.826663
Gglean015596	74.36422442	75.882275	49.0565395	45.826663
Gglean015597	74.36422442	75.882275	49.0565395	45.826663
Gglean015598	74.36422442	75.882275	49.0565395	45.826663
Gglean015599	41.73298	42.96589473	25.27578585	24.33036623
Gglean015600	41.73298	42.96589473	25.27578585	24.33036623
Gglean015601	16.0501	16.34215	18.32512	21.56181
Gglean015602	56.904986	58.704887	59.052685	62.703294
Gglean015603	29.2281	27.1873	21.6425	21.4635
Gglean015604	0	0	0	0
Gglean015605	20.11572629	21.67454867	15.94067282	13.95791102
Gglean015606	20.11572629	21.67454867	15.94067282	13.95791102
Gglean015607	6.28009	6.50159	8.34632	8.12187
Gglean015608	27.3818	26.8417	21.7784	18.4923
Gglean015609	37.45096	40.20777	22.81229	18.61575
Gglean015610	0.000440537	9.02E-05	0.00166768	0.000365165
Gglean015611	30.5224	32.346	35.9776	30.7895
Gglean015612	179.5731	185.1319	176.186	178.0176
Gglean015613	179.5731	185.1319	176.186	178.0176
Gglean015614	0	0	0	0
Gglean015615	0	0	0	0
Gglean015616	19.5980402	20.43620058	24.0411189	30.9018482
Gglean015617	6.51205	6.31427	6.15779	9.82828
Gglean015618	127.87605	169.29571	104.61803	111.41614
Gglean015619	10.35882	8.00868	5.9944	5.14641

Gglean015620	88.5236	97.4218	135.666	168.1495
Gglean015621	88.5236	97.4218	135.666	168.1495
Gglean015622	0.0777711	0	0	0
Gglean015623	0.0325041	0.0339867	0.0264048	0
Gglean015624	0.474089	0	0.39256	0
Gglean015625	1.77155	4.1453	6.43283	7.7459
Gglean015626	0	0	0	0
Gglean015627	10.13596	9.16976	6.09925	4.556988
Gglean015628	0	0	0	0
Gglean015629	0.337707	3.28479	3.95162	1.01079
Gglean015630	28.72445075	29.7192064	21.4766477	21.20707691
Gglean015631	28.72445075	29.7192064	21.4766477	21.20707691
Gglean015632	0	0	0	0.172837
Gglean015633	8.20686	5.7068	10.5257	8.13901
Gglean015634	289.807	263.176	611.606	659.04
Gglean015635	210.136	189.215	225.406	189.445
Gglean015636	1.07E-49	1.31E-20	4.37E-15	9.52E-58
Gglean015637	0	0	0	0
Gglean015638	32.4045	30.2782	32.5108	31.4208
Gglean015639	52.2719	49.3412	50.0386	54.0762
Gglean015640	170.9869	158.2756	191.6819	205.1379
Gglean015641	12.28221	16.41878	3.477754	2.893984179
Gglean015642	75.1109	75.8373	61.1361	57.4693
Gglean015643	1.4581	1.97063	0	0.72161
Gglean015644	74.256851	73.8909484	64.0764213	71.794809
Gglean015645	74.256851	73.8909484	64.0764213	71.794809
Gglean015646	21.40507828	21.765725	17.08459452	17.73070899
Gglean015647	5.264688	5.33893	2.280843	2.522788
Gglean015648	5.264688	5.33893	2.280843	2.522788
Gglean015649	48.67631361	52.19013516	56.92923699	53.90403223
Gglean015650	0.0979996	0.0335311	0.0792236	0.0315104
Gglean015651	50.7454	44.4708	32.71428	31.47534
Gglean015652	0	0	0	0.0708668
Gglean015653	53.88207	57.64171	54.90205	54.58661
Gglean015654	107.9499	121.032	56.75	68.816
Gglean015655	24.9318	22.5613	35.425	39.5601
Gglean015656	14.292211	15.83881	13.90933	15.261811
Gglean015657	9.005198384	8.308442655	9.22518559	8.537509496
Gglean015658	0.0124693	0.451704	0.947615	1.33565
Gglean015659	47.6422	49.8022	35.7444	31.1326
Gglean015660	12.6537	14.9765	9.74442	9.79988
Gglean015661	20.767575	19.40172	22.627708	22.672881
Gglean015662	9.65005	6.70484	15.45585	21.29636
Gglean015663	76.0943	70.1912	64.1736	68.9262

Gglean015664	12.7763	10.2724	15.9941	16.2625
Gglean015665	46.4136	33.1274	71.5543	63.4869
Gglean015666	105.8274	112.9281	138.071	141.5893
Gglean015667	0	0	0	0
Gglean015668	34.62980368	40.6080522	51.67170894	46.28281155
Gglean015669	0	0	0	0
Gglean015670	26.968715	28.736391	10.810866	8.4077825
Gglean015671	26.968715	28.736391	10.810866	8.4077825
Gglean015672	2.68421721	2.868746288	3.846271871	4.451132246
Gglean015673	26.93289	21.30007	35.31095	33.9606
Gglean015674	0.0893894	0.0924819	0	0.171379
Gglean015675	5.08419506	3.508193837	8.081965	6.703165
Gglean015676	4.95537	5.74665	1.780546	1.711789
Gglean015677	22.6077	25.73354	29.39569	28.27022
Gglean015678	27.57273174	29.16588377	36.15449366	46.43864737
Gglean015679	9.85604	10.3234	5.8526	4.76313
Gglean015680	0	0.234744	0.180881	0.216302
Gglean015681	0	0.501874	0	0
Gglean015682	0	0	0	0
Gglean015683	11.8215	12.0996	8.19254	7.2139
Gglean015684	8.834252	11.85025	7.804586	6.397272974
Gglean015685	73.154766	78.116947	41.8194	37.19489
Gglean015686	73.154766	78.116947	41.8194	37.19489
Gglean015687	106.4172019	109.5273021	114.299209	102.8273025
Gglean015688	25.531226	25.557171	27.412078	22.40921
Gglean015689	25.531226	25.557171	27.412078	22.40921
Gglean015690	25.531226	25.557171	27.412078	22.40921
Gglean015691	61.9388	69.3002	53.4229	54.0755
Gglean015692	25.2328	25.7689	21.9736	23.8637
Gglean015693	0.151348058	0	0	0
Gglean015694	0.151348058	0	0	0
Gglean015695	13.5851	14.3836	13.7577	14.7591
Gglean015696	2.951981	2.481194	1.518702	1.3011003
Gglean015697	14.461924	13.943456	21.765473	26.530138
Gglean015698	16.602871	16.677117	13.274629	13.887672
Gglean015699	114.96288	119.40304	78.20443557	69.51168761
Gglean015700	18.78225	19.6547	15.664052	14.972168
Gglean015701	18.78225	19.6547	15.664052	14.972168
Gglean015702	21.13355823	20.83940783	23.388418	22.643458
Gglean015703	27.0781	26.3685	16.9719	16.2916
Gglean015704	48.2169	33.4994	38.9149	45.3302
Gglean015705	14.79942	15.7791	7.339824	8.32066
Gglean015706	4.749562554	3.015588081	6.086192221	8.277514636
Gglean015707	3.065327	5.223549	3.971498	5.867852

Gglean015708	3.065327	5.223549	3.971498	5.867852
Gglean015709	3.17758	7.83864	8.17087	4.48259
Gglean015710	75.97103007	84.04084915	76.57477763	70.51334527
Gglean015711	19.1627	22.073	15.6776	14.7921
Gglean015712	16.78583	16.11765	13.07843064	13.57452
Gglean015713	11.4873	9.09856	6.79627	6.50247
Gglean015714	9.04917	9.19681	4.57304	8.50544
Gglean015715	3.0652868	2.68255	1.869404	2.074678
Gglean015716	3.0652868	2.68255	1.869404	2.074678
Gglean015717	8.84249	9.64552	7.180952783	6.647379269
Gglean015718	0	0	0	0
Gglean015719	0	0	0	0
Gglean015720	0	0	0	0.118816
Gglean015721	0	0	0	0
Gglean015722	0	0	0	0
Gglean015723	0	0.322526	0.12541	0
Gglean015724	0.0581016	0	0	0
Gglean015725	0	0	0	0
Gglean015726	0	0	0	0
Gglean015727	0	0	0	0
Gglean015728	2.37337	0	0	0
Gglean015729	71.90383	84.34957	40.27524	36.644087
Gglean015730	0	0	0	0
Gglean015731	0	0	0	0
Gglean015732	0.03936409	0.020324815	0	0.018784572
Gglean015733	0	0	0	0
Gglean015734	0.000201623	0	1.35E-06	1.78E-07
Gglean015735	0	0	0	0
Gglean015736	0.184948	0	0.22381	0.352923
Gglean015737	0	0	0	0
Gglean015738	1.64189	0.848715793	2.962447856	3.499350485
Gglean015739	1.45982	0.463104	1.57116	1.1434
Gglean015740	59.612	65.7134	75.7827	81.259
Gglean015741	18.49601	16.06128	16.42343	16.15252
Gglean015742	28.4701	30.9646	11.6253	10.5195
Gglean015743	0.7632195	1.019892	1.543194	1.236001
Gglean015744	18.6464	21.7395	11.2908	9.04238
Gglean015745	22.795	23.2512	24.7427	23.2508
Gglean015746	1.9405491	1.414192523	2.222088	2.288375
Gglean015747	1.9405491	1.414192523	2.222088	2.288375
Gglean015748	1.9405491	1.414192523	2.222088	2.288375
Gglean015749	1.9405491	1.414192523	2.222088	2.288375
Gglean015750	21.769938	15.38941	26.025541	38.63172
Gglean015751	13.51347	27.13385	15.15014	24.35909

Gglean015752	8.574407	16.70359	7.1844	7.06555
Gglean015753	0.502329	0.446489916	0.222313346	0.746114408
Gglean015754	42.1232	38.4466	40.0929	59.0062
Gglean015755	12.00283458	12.58257744	14.6137908	19.06760481
Gglean015756	1.14323	1.48553	0.974582	0.950943
Gglean015757	74.5797664	77.85481	83.57255018	76.57013
Gglean015758	74.5797664	77.85481	83.57255018	76.57013
Gglean015759	41.527077	43.74777	31.72223	29.20877
Gglean015760	13.87762	11.59657	9.9593	10.98323
Gglean015761	6.92488	5.45051	2.73857	2.29362
Gglean015762	0	0	0	0
Gglean015763	0	0	0	0
Gglean015764	96.18870905	78.32495023	113.2593935	144.2231844
Gglean015765	96.18870905	78.32495023	113.2593935	144.2231844
Gglean015766	4.821101795	5.647918484	7.343657561	9.86167928
Gglean015767	4.821101795	5.647918484	7.343657561	9.86167928
Gglean015768	0	0	0	0
Gglean015769	0	0	0	0
Gglean015770	3.558074	4.167927351	6.77974329	6.113283102
Gglean015771	7.540836	8.074384	5.373714	4.273904
Gglean015772	1.17439941	0.18646362	0.8508173	0.97104007
Gglean015773	1.17439941	0.18646362	0.8508173	0.97104007
Gglean015774	6.93025304	7.5380918	10.58156065	10.47609037
Gglean015775	0.1747	0	0	0
Gglean015776	33.9048	27.2321	34.0659	50.3924
Gglean015777	2.424547951	3.059790486	5.869640361	5.778340434
Gglean015778	2.424547951	3.059790486	5.869640361	5.778340434
Gglean015779	7.452498	8.68005	7.4531025	9.27333
Gglean015780	22.38141	22.1987385	18.848564	19.87577857
Gglean015781	0.206681	0.214372	0.333345	0
Gglean015782	24.7670767	23.04611	14.54275	11.912864
Gglean015783	24.7670767	23.04611	14.54275	11.912864
Gglean015784	0	0	0	0
Gglean015785	0	0.0344904	0.0539134	0.0318663
Gglean015786	0	0.487263	0	1.19E-05
Gglean015787	0.793217158	0.357627946	0.646495005	0.330000057
Gglean015788	0.793217158	0.357627946	0.646495005	0.330000057
Gglean015789	0	0	0	0
Gglean015790	0	0.232771	0.360311	0
Gglean015791	0	0	0	0
Gglean015792	25.12851	22.18184	23.06966	21.76135
Gglean015793	25.12851	22.18184	23.06966	21.76135
Gglean015794	116.54268	116.286286	32.356481	29.155119
Gglean015795	10.06229868	11.09819551	15.24962204	17.50795743

Gglean015796	0.0466038	0	0.112223	0
Gglean015797	1.44E-05	0.00100741	4.83E-06	2.46E-05
Gglean015798	43.0526	28.93032	40.79222	47.8509
Gglean015799	2.053303	3.07033	3.38242	3.794567
Gglean015800	0	0	0	0
Gglean015801	0.451984	1.10662	1.05963	0.820207
Gglean015802	0	0.246445	0.0964706	0.0571143
Gglean015803	0	0	0	0
Gglean015804	0	0	0	0
Gglean015805	18.1975	18.726455	33.6363076	33.99141115
Gglean015806	105.033	99.763	160.974	208.742
Gglean015807	20.3821	19.1742	9.25182	5.77875
Gglean015808	0	0	0	0
Gglean015809	0	0	0	0
Gglean015810	0	0	0	0
Gglean015811	0	0.0862435	0	0
Gglean015812	0	0	0	0
Gglean015813	5.434078614	5.244207205	5.0478345	4.8036668
Gglean015814	0	0	0	0
Gglean015815	0	0	0.161526	0
Gglean015816	0	0	0	0
Gglean015817	0	0	0	0
Gglean015818	0	0	0.241056	0.143878
Gglean015819	39.76020865	39.418396	38.85006	51.74155202
Gglean015820	39.76020865	39.418396	38.85006	51.74155202
Gglean015821	39.76020865	39.418396	38.85006	51.74155202
Gglean015822	86.90106	90.9113	80.6862	68.306
Gglean015823	63.57198	69.47065	63.5442	58.45849
Gglean015824	1.31703	6.73669	2.14392	1.93076
Gglean015825	29.931241	25.626958	26.76179	23.488751
Gglean015826	29.931241	25.626958	26.76179	23.488751
Gglean015827	45.6623	38.9832	30.28217	31.93129
Gglean015828	27.39362027	30.07408789	30.2380357	32.1395714
Gglean015829	27.39362027	30.07408789	30.2380357	32.1395714
Gglean015830	35.3903	39.3402	50.9772	54.2132
Gglean015831	29.0309	29.5778	17.1079	15.5549
Gglean015832	32.13309576	32.88064294	18.82684142	14.6655994
Gglean015833	15.12365	17.77194	13.379196	11.98472
Gglean015834	177.01083	185.2437	192.01043	180.87817
Gglean015835	7.06E-05	0.451492	2.41132	8.07055
Gglean015836	2.14E-05	2.11E-05	0.00173334	1.68E-05
Gglean015837	56.4322	61.228	18.60839	13.77795
Gglean015838	18.1474	17.7302	11.3786	9.50291
Gglean015839	21.3607763	21.38761935	15.66684092	17.64522682

Gglean015840	24.3029	27.2645	17.5039	16.8086
Gglean015841	33.29104	35.08524	42.02995	40.13144
Gglean015842	33.29104	35.08524	42.02995	40.13144
Gglean015843	40.62267	37.25203923	19.92584	19.158708
Gglean015844	30.14539	33.6857	28.46141	25.10736
Gglean015845	36.49762	38.1883	33.52147	34.02534
Gglean015846	29.57439489	31.705768	30.09190402	27.14899827
Gglean015847	20.893553	19.79520211	37.19023348	36.2646663
Gglean015848	0.111634	0.0386687	0.150295	0
Gglean015849	71.78867	67.8698	67.47093	62.78718
Gglean015850	51.7401	56.8255	49.8682	46.5978
Gglean015851	1.994225787	1.534405283	0.894305127	0.506314401
Gglean015852	87.59919071	87.29448276	95.95873689	100.2256849
Gglean015853	87.59919071	87.29448276	95.95873689	100.2256849
Gglean015854	21.069	25.2259	14.6233	12.1772
Gglean015855	19.760871	20.401159	18.582497	18.018238
Gglean015856	0	0	0	0.0259054
Gglean015857	6.572731	6.308904	10.157154	10.55141758
Gglean015858	0	0.126267	0	0
Gglean015859	0.141673	0.0586413	0.0229053	0.135278
Gglean015860	0	0	0	0
Gglean015861	0	0	0	0
Gglean015862	0.703848	0.58266	0.81976	1.15622
Gglean015863	0	0	0	0
Gglean015864	0	0	0	0
Gglean015865	1.46536	5.57688	3.72113	3.08309
Gglean015866	33.01487936	35.6018454	27.052547	24.74704864
Gglean015867	33.01487936	35.6018454	27.052547	24.74704864
Gglean015868	0	0.0513645	0.201976	0.143328
Gglean015869	32.986	34.6224	17.9675	18.1017
Gglean015870	42.59815	48.57515	32.056994	28.020255
Gglean015871	224.5123064	232.9534999	200.0944631	188.5289058
Gglean015872	33.58017249	35.74523701	50.8002519	52.45130687
Gglean015873	33.58017249	35.74523701	50.8002519	52.45130687
Gglean015874	21.86563	22.4466	11.87582	10.62616
Gglean015875	24.10697238	22.91486	19.77932	19.459798
Gglean015876	30.058737	42.03845	37.674566	32.746847
Gglean015877	0	0	0	0
Gglean015878	35.379369	43.69325	63.93666	70.6565
Gglean015879	3.005917	4.276862772	3.588826533	5.576476211
Gglean015880	4.443027243	5.061250614	6.342028214	7.857978113
Gglean015881	21.53978981	21.35738922	27.25419611	30.2809553
Gglean015882	0	0	0	0
Gglean015883	20.65152	20.57249	14.98595	16.34543

Gglean015884	29.56269	29.77464	18.266432	14.46503
Gglean015885	393.75334	424.3793146	273.21635	278.07256
Gglean015886	52.83427002	53.16821767	48.730453	46.58703074
Gglean015887	52.83427002	53.16821767	48.730453	46.58703074
Gglean015888	15.35248911	16.76542596	13.33161687	11.88538224
Gglean015889	0	0.294387	0	0
Gglean015890	0	0	0.716505	0
Gglean015891	222.907	216.428	230.675	329.969
Gglean015892	9.043484	11.68196249	11.359794	15.666123
Gglean015893	9.76501	6.42787	9.94334	13.9853
Gglean015894	9.043484	11.68196249	11.359794	15.666123
Gglean015895	0	0	0	0
Gglean015896	0	2.0176	2.239	0
Gglean015897	0.000163191	0.000239437	0.2541883	0.403815
Gglean015898	0.000163191	0.000239437	0.2541883	0.403815
Gglean015899	2.1872	0.514338	2.59869	0.954136
Gglean015900	62.345871	49.352383	65.24144	53.05801607
Gglean015901	62.345871	49.352383	65.24144	53.05801607
Gglean015902	3.031607	4.164363	6.743365	5.475488
Gglean015903	0.354404	0.0759669	0.0599755	0
Gglean015904	76.75569	73.95759	49.5186	47.79177
Gglean015905	76.75569	73.95759	49.5186	47.79177
Gglean015906	76.75569	73.95759	49.5186	47.79177
Gglean015907	76.75569	73.95759	49.5186	47.79177
Gglean015908	0.99789	1.12066	4.06785	2.92248
Gglean015909	0	0	0.072422454	0
Gglean015910	16.29354	14.320109	11.71069	8.93621
Gglean015911	16.29354	14.320109	11.71069	8.93621
Gglean015912	28.68132	32.73606	33.89982	34.31789
Gglean015913	25.20150846	28.99034	34.43063	44.50483
Gglean015914	0	0	0	0
Gglean015915	0	0	0	0
Gglean015916	51.84059	52.93264	57.8693	51.13
Gglean015917	19.34891	15.91146	32.95427	33.44666
Gglean015918	19.34891	15.91146	32.95427	33.44666
Gglean015919	100.86024	101.81438	113.63154	104.89734
Gglean015920	2.035085322	1.066488	1.669629267	1.499114566
Gglean015921	11.6049488	12.92474624	9.69723984	9.311956971
Gglean015922	72.0045	58.7654	68.94726	67.6533
Gglean015923	55.20079	60.09747	32.33081	27.67169816
Gglean015924	30.6437	29.1725	15.0941	13.729
Gglean015925	12.5481	22.48308	2.98843	1.00344
Gglean015926	17.83646967	23.29679743	11.15495977	8.539353094
Gglean015927	21.938	31.0339	32.2638	38.3533

Gglean015928	26.958828	28.33145754	28.689611	29.87434
Gglean015929	19.45126	16.70009	20.85314	22.63136
Gglean015930	36.576029	34.819868	33.63072198	33.7398357
Gglean015931	1961.444	1606.743	2210.64	3238.698
Gglean015932	5.492386372	5.59343894	6.357802196	8.534721
Gglean015933	0	0	0	0
Gglean015934	0	0	0.040084	0.0949592
Gglean015935	1150.206	1169.532	744.9748	738.1116
Gglean015936	72.4833	75.7163	66.6264	69.9104
Gglean015937	35.81833	39.378667	21.6042664	19.956786
Gglean015938	196.6338685	168.5105708	204.8640939	277.3599141
Gglean015939	196.6338685	168.5105708	204.8640939	277.3599141
Gglean015940	15.91282536	17.11031	14.375052	13.66199
Gglean015941	75.11299585	78.20154765	66.919934	59.09711633
Gglean015942	36.186654	40.37299243	47.25031151	50.16872858
Gglean015943	36.186654	40.37299243	47.25031151	50.16872858
Gglean015944	36.186654	40.37299243	47.25031151	50.16872858
Gglean015945	11.27493	11.16198	28.96345	31.9092
Gglean015946	50.669123	48.17110001	45.553462	48.0567
Gglean015947	50.669123	48.17110001	45.553462	48.0567
Gglean015948	199.43708	206.94832	173.09986	154.22038
Gglean015949	105.0122182	119.329785	79.3255577	75.62611
Gglean015950	22.45089999	19.91399728	34.796492	40.3567657
Gglean015951	39.74362	39.91562	41.23703	42.26743
Gglean015952	55.90822	52.8136	46.08175	40.158056
Gglean015953	55.90822	52.8136	46.08175	40.158056
Gglean015954	55.90822	52.8136	46.08175	40.158056
Gglean015955	79.42344951	87.696085	111.072013	87.20870392
Gglean015956	79.42344951	87.696085	111.072013	87.20870392
Gglean015957	79.42344951	87.696085	111.072013	87.20870392
Gglean015958	79.42344951	87.696085	111.072013	87.20870392
Gglean015959	26.43748299	26.67301859	39.56917916	44.3507128
Gglean015960	0.110564	0	0.179263	0.213098
Gglean015961	18.47575266	19.84021	28.3662	30.685705
Gglean015962	27.24187	29.90166	27.49565	27.70555
Gglean015963	55.9958504	61.91182526	59.39576093	57.21949341
Gglean015964	37.0422258	38.994694	24.34146392	23.61922904
Gglean015965	37.0422258	38.994694	24.34146392	23.61922904
Gglean015966	37.0422258	38.994694	24.34146392	23.61922904
Gglean015967	12.466441	11.455861	12.500276	10.976308
Gglean015968	54.28086606	55.3983	56.34483421	55.34572616
Gglean015969	457.271941	400.3996027	934.0861772	833.9511996
Gglean015970	0	0	0	0
Gglean015971	0	0	0	0

Gglean015972	0	0	0	0
Gglean015973	4.5244574	3.964157	5.728706	6.666208327
Gglean015974	19.26472	20.12256	11.85705	10.81739
Gglean015975	13.2749	10.0588	13.5956	17.2448
Gglean015976	0.622525	0.000875785	0.000332454	0.00110079
Gglean015977	31.68801	31.42503258	48.41412451	60.0209
Gglean015978	31.68801	31.42503258	48.41412451	60.0209
Gglean015979	117.7903362	102.1643673	190.8144125	200.8717099
Gglean015980	27.4726	28.7606	68.15705	77.79379
Gglean015981	117.7903362	102.1643673	190.8144125	200.8717099
Gglean015982	1.32E-136	2.76E-200	0.0170034	2.34E-215
Gglean015983	0	0	0	0
Gglean015984	20.21612336	23.142208	23.508095	24.42810464
Gglean015985	52.09764	55.43013	55.91173	51.75861
Gglean015986	0.254197	0.486193	0.623465	0.738096
Gglean015987	4.276717	1.862995915	2.63397874	2.966644921
Gglean015988	4.276717	1.862995915	2.63397874	2.966644921
Gglean015989	15.74312912	15.68034414	31.14926925	28.80982757
Gglean015990	0	0	0	0
Gglean015991	2.180118	2.067920243	3.002610581	4.522971124
Gglean015992	2.180118	2.067920243	3.002610581	4.522971124
Gglean015993	10.11367769	10.34738356	17.44963834	22.40017658
Gglean015994	11.306	13.2231	10.9458	12.4308
Gglean015995	5.77803	4.01225	6.00728	10.2909
Gglean015996	55.24676	60.41146	46.5834	53.79414
Gglean015997	31.66969439	34.828856	47.18881559	46.70083769
Gglean015998	26.39061	27.84328	22.61787	21.82077
Gglean015999	8.679624253	7.68994	8.57988	6.713823261
Gglean016000	0	0	0.416662	0
Gglean016001	14.00173755	12.58536318	23.3413815	21.78068
Gglean016002	13.10085	11.98954	20.77748	21.72212
Gglean016003	8.40684	9.76453	7.68645	14.6303
Gglean016004	31.59365	24.23463	19.8969	18.12705
Gglean016005	31.59365	24.23463	19.8969	18.12705
Gglean016006	6.3086	6.38282	7.08374	7.92008
Gglean016007	31.419168	29.69509	21.6143724	20.608047
Gglean016008	31.419168	29.69509	21.6143724	20.608047
Gglean016009	31.419168	29.69509	21.6143724	20.608047
Gglean016010	125.196	128.954	38.982	26.1778
Gglean016011	7.022800752	8.2442155	4.895169	4.604291532
Gglean016012	7.022800752	8.2442155	4.895169	4.604291532
Gglean016013	0	0	0.244236	0
Gglean016014	20.381279	22.08075559	28.84112	34.748597
Gglean016015	25.37275563	26.90829053	25.24595193	23.03024311

Gglean016016	6.386976	9.773292045	6.398220951	6.36778947
Gglean016017	18.2664098	21.3746221	5.961751	4.841689619
Gglean016018	1.82503	0.751924	1.17492	0.819415
Gglean016019	10.02613	10.85628	17.92147	17.69642
Gglean016020	196.4803	224.1364	168.4611	132.0469
Gglean016021	17.7701	19.9268	17.6521	18.3733
Gglean016022	1.57133	1.93642	2.9975	2.84222
Gglean016023	103.5851	118.0572	153.7231	146.8631
Gglean016024	0	0	0	0
Gglean016025	45.4526	66.8331	67.8919	94.6322
Gglean016026	19.98594	24.37675	16.05948	14.5549
Gglean016027	132.6464	135.4093	117.98485	134.292
Gglean016028	19.33789476	15.611517	19.49510499	22.88868027
Gglean016029	19.33789476	15.611517	19.49510499	22.88868027
Gglean016030	19.33789476	15.611517	19.49510499	22.88868027
Gglean016031	37.44596	41.41804	37.41366	36.964604
Gglean016032	50.22659328	49.61430548	22.27685	20.91821
Gglean016033	74.4226	86.0146	18.3345	13.67871
Gglean016034	25.18137	25.38790526	17.82982	16.02582
Gglean016035	15.7658	15.873	8.07287	6.63021
Gglean016036	0	0	0	0
Gglean016037	9.232269	7.522663	9.03038	8.66954
Gglean016038	67.87740947	70.89441175	66.33650403	61.22861241
Gglean016039	9.890908	9.1034627	11.55748	17.63688
Gglean016040	9.890908	9.1034627	11.55748	17.63688
Gglean016041	25.70009	27.47194	20.65627	19.23412
Gglean016042	10.3495	11.0142	13.652	15.64
Gglean016043	68.02275387	82.6042567	79.61236775	76.50215535
Gglean016044	21.31061983	26.32906599	33.92748633	31.89092238
Gglean016045	21.31061983	26.32906599	33.92748633	31.89092238
Gglean016046	123.6529466	123.85971	117.708011	129.562436
Gglean016047	2.488107739	2.777859	1.3258902	1.3529021
Gglean016048	0.3077	0.157452	0.165779	0.196371
Gglean016049	9.279726198	8.604766108	4.778495943	6.302917066
Gglean016050	9.279726198	8.604766108	4.778495943	6.302917066
Gglean016051	4.246257316	4.791886371	2.320173002	1.357093646
Gglean016052	4.246257316	4.791886371	2.320173002	1.357093646
Gglean016053	4.246257316	4.791886371	2.320173002	1.357093646
Gglean016054	4.246257316	4.791886371	2.320173002	1.357093646
Gglean016055	99.2956	102.8	73.3219	60.652
Gglean016056	17.2781	16.2185	9.72844	5.60721
Gglean016057	218.593	205.982	239.206	317.1278519
Gglean016058	6.8284	8.58036	11.797	16.126
Gglean016059	23.48164	20.58088	27.4451	45.1299

Gglean016060	33.0461	35.0385	23.2855	21.4804
Gglean016061	15.67830176	14.43484619	8.576127	6.0824059
Gglean016062	47.956045	48.03932	33.451813	33.94652
Gglean016063	0	0.0450849	0	0
Gglean016064	0.146947	0.592528	0.152835	4.26E-60
Gglean016065	0.146947	0.592528	0.152835	4.26E-60
Gglean016066	0	0	1.73589	4.13453
Gglean016067	26.3384951	27.922947	48.23097237	46.90489412
Gglean016068	0	0	0	0.303005
Gglean016069	12.2828	14.1759	9.19911	7.69016
Gglean016070	24.39291585	24.95587176	13.96899631	10.160011
Gglean016071	24.39291585	24.95587176	13.96899631	10.160011
Gglean016072	64.6625	74.6547	36.31117	33.42736
Gglean016073	1.48074	1.54362	2.17183	2.63804
Gglean016074	49.8275122	47.85937	51.10634	53.97894
Gglean016075	29.66364	28.598093	33.721908	33.05053
Gglean016076	29.66364	28.598093	33.721908	33.05053
Gglean016077	23.34213231	25.62940968	19.37035935	17.76221953
Gglean016078	8.37776	8.10995	6.11064	4.05427
Gglean016079	20.3796	25.6169	22.5987	20.293
Gglean016080	8.82443	11.8281	7.11689	6.89651
Gglean016081	8.87891	7.99911	10.56054	8.02814
Gglean016082	0	0.0499349	1.99E-07	0.0153255
Gglean016083	27.30405	30.12136	19.530203	18.4808
Gglean016084	9.83018	13.8602	2.95391	4.47929
Gglean016085	1.198608331	3.000810326	3.017472446	3.305004
Gglean016086	3.23249	1.18896	3.99608	5.86995
Gglean016087	1.505512174	1.631188496	2.068039904	2.332156
Gglean016088	74.0076	79.2691	49.0873	45.8297
Gglean016089	1.447839263	1.111303041	1.248100376	1.5955463
Gglean016090	1.447839263	1.111303041	1.248100376	1.5955463
Gglean016091	35.1993	32.7463	29.4386	28.8239
Gglean016092	0.000148236	2.74452	2.03109	1.25E-09
Gglean016093	14.61985	14.61322	6.931753	6.998265
Gglean016094	7.73467511	10.67632	11.49656208	14.54021094
Gglean016095	7.72E-264	2.57E-124	3.72E-129	0
Gglean016096	7.72E-264	2.57E-124	3.72E-129	0
Gglean016097	51.17920001	52.90301212	47.20008986	42.24999001
Gglean016098	19.50771	19.68625	26.83692	23.31384
Gglean016099	2.20E-19	1.91E-14	0.0500577	0.155914
Gglean016100	24.4704	26.9166	17.13053	14.00711
Gglean016101	38.53283	36.50783	39.4696	47.71244
Gglean016102	38.53283	36.50783	39.4696	47.71244
Gglean016103	32.1023129	32.37479974	27.96495378	30.37367233

Gglean016104	0	0	0	0
Gglean016105	5.799476	2.915855185	5.02203	7.770703642
Gglean016106	32.79684872	33.6743491	35.78153062	38.80155123
Gglean016107	211.2107	230.2403	160.472	151.397
Gglean016108	211.2107	230.2403	160.472	151.397
Gglean016109	0.344086	0.359136	0.61332	0.197958
Gglean016110	7.537085	4.702102	8.157524	8.053631021
Gglean016111	7.2459	9.56272	12.096828	14.27074
Gglean016112	0	0	0	0
Gglean016113	0	0.24399	0.114848	0.045269
Gglean016114	13.90909476	18.78755331	22.8772726	31.86275697
Gglean016115	13.90909476	18.78755331	22.8772726	31.86275697
Gglean016116	176.941519	170.0074508	119.459001	112.292001
Gglean016117	30.2755	30.2168	21.1611	15.023
Gglean016118	46.1023	49.9255	29.9921	29.009
Gglean016119	24.5322202	22.730423	29.50913	25.38157
Gglean016120	24.5322202	22.730423	29.50913	25.38157
Gglean016121	34.4551	41.3465	24.1824	20.7588
Gglean016122	95.28210299	91.852507	104.710342	110.3861119
Gglean016123	84.4271	82.7616	78.1397	70.5983
Gglean016124	40.4192	47.8846	54.0667	62.9218
Gglean016125	9.826702	10.255048	17.019945	16.13163711
Gglean016126	45.9841008	52.7622821	66.8942676	87.280845
Gglean016127	0	0	0	0
Gglean016128	0.0541809	0.111263	0.0438653	0
Gglean016129	0	0	0	0
Gglean016130	0	0	0	0.536172
Gglean016131	0	0	0	0
Gglean016132	24.95126781	25.61628568	25.13213486	26.16568446
Gglean016133	24.95126781	25.61628568	25.13213486	26.16568446
Gglean016134	1.031623324	1.062020039	0.322989519	0.221741329
Gglean016135	0.657068	0.538178	0.117769	0
Gglean016136	7.911171	9.230859	15.745794	14.150486
Gglean016137	21.04393	26.13133	26.75213	30.4165
Gglean016138	211.441838	209.1667699	191.7959543	187.6475168
Gglean016139	211.441838	209.1667699	191.7959543	187.6475168
Gglean016140	0	0.165521	1.46329	1.04E-45
Gglean016141	48.45177	51.39151	49.73917	48.76692
Gglean016142	48.45177	51.39151	49.73917	48.76692
Gglean016143	35.32559	36.48982	28.32136	27.73875
Gglean016144	33.81427	38.84295	28.36959	33.52465284
Gglean016145	22.35435	21.17364	13.286391	12.52338
Gglean016146	58.0982	51.0103	51.127	46.483
Gglean016147	863.0033	979.2685	37.40666	40.12288114

Gglean016148	33.94622655	36.94992583	25.80913257	21.48603123
Gglean016149	33.94622655	36.94992583	25.80913257	21.48603123
Gglean016150	24.7985	26.4164	16.7136	14.0442
Gglean016151	24.7985	26.4164	16.7136	14.0442
Gglean016152	28.01353	27.48228	24.905713	24.17752
Gglean016153	18.7948816	18.55401427	28.41011858	24.71364218
Gglean016154	84.4189	77.79268	66.352328	66.8122
Gglean016155	0.968383	1.173741	1.614929	1.8105
Gglean016156	0.968383	1.173741	1.614929	1.8105
Gglean016157	35.587	33.9548	23.35727	23.14657
Gglean016158	233.05	211.65	289.632	250.2
Gglean016159	8.080582688	8.028509415	12.45328939	12.03854881
Gglean016160	36.83091189	39.85582824	28.41501491	28.64176991
Gglean016161	24.09525217	25.71482758	31.93158038	36.16220013
Gglean016162	55.01710194	62.64580769	76.39210322	78.75140444
Gglean016163	42.3572	45.6241	41.0083	40.9076
Gglean016164	70.18083	64.43503	163.57333	183.06169
Gglean016165	70.18083	64.43503	163.57333	183.06169
Gglean016166	14.18886488	15.25716769	20.4436881	20.88668676
Gglean016167	53.133673	56.908693	36.000676	32.771158
Gglean016168	53.133673	56.908693	36.000676	32.771158
Gglean016169	1489.306	1526.769	985.815	976.591
Gglean016170	0.182315	0.116649	0.252642	0.333304
Gglean016171	34.1706776	29.5512898	75.26418202	72.15686004
Gglean016172	34.1706776	29.5512898	75.26418202	72.15686004
Gglean016173	34.1706776	29.5512898	75.26418202	72.15686004
Gglean016174	0.246314	0.916591	4.59E-23	1.086455
Gglean016175	0.246314	0.916591	4.59E-23	1.086455
Gglean016176	19.6152	22.5061	8.9862	7.92799
Gglean016177	8.003165641	7.013601865	4.882621404	7.474947468
Gglean016178	8.003165641	7.013601865	4.882621404	7.474947468
Gglean016179	0.489375421	0.537332155	0.396304376	0.4428622
Gglean016180	17.44547004	19.87355021	15.26546001	15.58319032
Gglean016181	17.44547004	19.87355021	15.26546001	15.58319032
Gglean016182	5.93265	3.6399	9.96036	7.1726
Gglean016183	164.7846	182.6747	109.8197	105.2162
Gglean016184	132.3925021	140.2068025	88.77830174	85.42969186
Gglean016185	15.44444922	18.74229283	21.71709	24.46277
Gglean016186	35.9011	46.5884	20.2239	15.062
Gglean016187	75.9908	79.4857	67.0798	69.1512
Gglean016188	75.9908	79.4857	67.0798	69.1512
Gglean016189	75.9908	79.4857	67.0798	69.1512
Gglean016190	7.349476	6.62997832	5.94005705	6.62185589
Gglean016191	0	0	0	0

Gglean016192	104.058	115.751	51.3207	42.6724
Gglean016193	74.25584475	77.36994931	86.88570633	76.23030508
Gglean016194	94.06271059	87.11897683	23.44978	14.436382
Gglean016195	36.18263	41.06415	37.16199	34.14776
Gglean016196	36.18263	41.06415	37.16199	34.14776
Gglean016197	23.598655	24.44453208	54.928215	59.4778
Gglean016198	11.2178	12.63713	14.39372	15.741225
Gglean016199	68.97549209	63.17342822	61.51906002	54.45788274
Gglean016200	17.6616	18.4348	13.262	14.0882
Gglean016201	44.4675015	46.6655162	42.338703	46.93712
Gglean016202	44.4675015	46.6655162	42.338703	46.93712
Gglean016203	38.3334	35.969	25.7264	32.428
Gglean016204	466.475	463.76	555.275	647.57
Gglean016205	46.65667869	39.72268258	45.96079751	43.8382386
Gglean016206	46.65667869	39.72268258	45.96079751	43.8382386
Gglean016207	26.2012	22.18354	30.3388	29.7217
Gglean016208	0	0	0	0
Gglean016209	0	0.165521	1.46329	1.04E-45
Gglean016210	3.262265	2.515601	2.5764	1.821247
Gglean016211	3.262265	2.515601	2.5764	1.821247
Gglean016212	67.254705	69.88684	47.000245	40.637917
Gglean016213	89.6785	86.4218	61.71901	60.63092
Gglean016214	0.4747367	0.416426427	0.655081186	0.847511787
Gglean016215	8.906858555	9.50844364	7.379486006	7.01227846
Gglean016216	0	0	0	0
Gglean016217	0	0	0	0
Gglean016218	0	0	0	0
Gglean016219	3.78208	4.57057	0.00232386	5.69725
Gglean016220	0	0	0	0
Gglean016221	0	0.213276	0	0.193884
Gglean016222	0	0	0	0
Gglean016223	0.193063832	0.049688952	0.039152072	0.092568918
Gglean016224	0.193063832	0.049688952	0.039152072	0.092568918
Gglean016225	0.118073657	0.151584677	0	0.072028904
Gglean016226	0	0	0	0
Gglean016227	0.184244147	0.207906625	0.658223	0.41697413
Gglean016228	0.184244147	0.207906625	0.658223	0.41697413
Gglean016229	0.0272159	0	0.0444223	0.052779
Gglean016230	0.688477226	0.600271695	0.669497084	0.677595542
Gglean016231	0.688477226	0.600271695	0.669497084	0.677595542
Gglean016232	0	0.09522643	0.0323504	0.2666896
Gglean016233	34.0961	44.1015	48.9478	44.3903
Gglean016234	9.708879236	9.477606323	12.40896456	14.28460002
Gglean016235	15.2764224	16.885708	25.23705955	30.9913187

Gglean016236	15.2764224	16.885708	25.23705955	30.9913187
Gglean016237	15.2764224	16.885708	25.23705955	30.9913187
Gglean016238	0.213499	0.738955	0.520683	0.752375
Gglean016239	0.13261	0.362283	0.355811599	0.0847623
Gglean016240	0.419722	0.460021	0.346027	0
Gglean016241	0.340323	0.179614	0.276381	0.499308
Gglean016242	0.103338	0.106671	0.084026	0
Gglean016243	0	0	0	0
Gglean016244	0.20121	0.238068	0.0926992	0.273929
Gglean016245	0	0.0662442	0	0.0935279
Gglean016246	0	0.0662442	0	0.0935279
Gglean016247	0.0973285	0.101168	0.315531	0
Gglean016248	2.84699	1.92389	1.26505	0
Gglean016249	71.94	71.9136	2.34648	0.222465
Gglean016250	45.46206	40.80619	51.43264	72.59098
Gglean016251	45.46206	40.80619	51.43264	72.59098
Gglean016252	57.01822283	51.95362932	45.16886948	45.97624329
Gglean016253	0	0	0	0
Gglean016254	0	0	0	0
Gglean016255	0.179084	0	0	0
Gglean016256	0	0	0	0
Gglean016257	0	0	0	0
Gglean016258	0	0	0.0561824	0.0664317
Gglean016259	0.440965	0.76757	0	0
Gglean016260	58.7498	56.4781	2.35394	0.0451005
Gglean016261	0	0	0.291156	0
Gglean016262	1.47E-05	8.33E-06	0.062817	0.0744986
Gglean016263	0.932803	1.70439	0.0998011	0.215983
Gglean016264	0	1.68797	0	0.745514
Gglean016265	0.061248	0.0633782	0	0.177108
Gglean016266	0	0	0	0
Gglean016267	0.125136	0	0	0.0400714
Gglean016268	0	0.0317173	0	0
Gglean016269	0	0	0	0
Gglean016270	0	0.488335	0	0
Gglean016271	76.26264326	70.74754438	67.904792	57.283426
Gglean016272	76.26264326	70.74754438	67.904792	57.283426
Gglean016273	18.79208092	20.962	25.78715017	31.7962347
Gglean016274	18.79208092	20.962	25.78715017	31.7962347
Gglean016275	7.607303	9.41469	11.41847	14.13874
Gglean016276	198.497144	198.2848	147.2988	168.41914
Gglean016277	198.497144	198.2848	147.2988	168.41914
Gglean016278	65.98077378	60.47588	51.87493375	62.63866379
Gglean016279	8.8205	9.23594	12.7409	15.5932

Gglean016280	0.0444527	0.0458837	0.0360647	0.128593
Gglean016281	0.04697879	0.220027137	0.295085008	0.319673793
Gglean016282	0	0	0	0
Gglean016283	0	0	0	0
Gglean016284	19.34605269	13.72376474	11.8272362	13.7195138
Gglean016285	0.825900547	0.513033847	0.430466033	0.747489925
Gglean016286	73.7658	76.4239	54.43466	47.77501
Gglean016287	2.76901642	4.233856089	4.497599651	3.973434852
Gglean016288	2.76901642	4.233856089	4.497599651	3.973434852
Gglean016289	3.51353	3.2917	3.70598	2.95707
Gglean016290	2.76901642	4.233856089	4.497599651	3.973434852
Gglean016291	54.6624	53.0882	50.21689	47.7217
Gglean016292	9.455269512	12.50385419	4.43103705	3.810163916
Gglean016293	19.60318888	20.44068669	32.93774586	36.341707
Gglean016294	0	0	0	0
Gglean016295	0	0	0	0
Gglean016296	0	0	0	0
Gglean016297	14.9288	15.2497	14.1766	13.1427
Gglean016298	21.85972	20.92716	24.75561	26.04995
Gglean016299	0	0	0	0
Gglean016300	2.583876938	2.338684436	3.387153546	3.654824621
Gglean016301	35.44077	32.79479	29.56776	33.10683
Gglean016302	1329.221	1425.344	877.5257	824.797
Gglean016303	0.138103	0.146167	0	0
Gglean016304	26.7625	27.6652	36.7303	36.2639
Gglean016305	6.06509	6.69529	14.88185	10.88196
Gglean016306	53.83449	41.46000003	51.59237	65.9039003
Gglean016307	274.66158	287.0762351	213.50233	187.64014
Gglean016308	0.714542	0.653396744	0.373823	0.908535
Gglean016309	1.984026552	2.171572	3.437746633	4.412235
Gglean016310	1.62566926	1.461887885	2.48002	2.591478
Gglean016311	0	2.98E-113	4.51E-169	1.62E-305
Gglean016312	13.128423	14.8152124	19.12786578	22.7861441
Gglean016313	0	0	0	0
Gglean016314	5.63927	5.51876	2.1215	3.16131
Gglean016315	118.317	118.432	87.2204	58.2894
Gglean016316	321.692	302.061	100.754	87.5766
Gglean016317	24.7586414	22.381454	31.974011	30.13031591
Gglean016318	11.50820779	11.662893	11.28779818	10.34013787
Gglean016319	51.356	57.3146	61.5425	60.224
Gglean016320	31.38605697	33.97989	67.32180712	70.04691595
Gglean016321	0.261393299	0.066418594	0.2317456	0.166823397
Gglean016322	160.404	161.079	100.902	99.0046
Gglean016323	26.085	28.754	18.80008	17.34615

Gglean016324	17.60866381	17.4520143	26.93496668	29.50712517
Gglean016325	0	0	0	0
Gglean016326	47.2038	48.16933	34.738067	34.401783
Gglean016327	0.864103	1.15399	1.21848	1.37397
Gglean016328	0.749119	0.73486	1.05385	1.28046
Gglean016329	42.737	29.3879873	27.29898	26.20713
Gglean016330	78.00798	73.3752731	69.34156131	71.74888
Gglean016331	78.00798	73.3752731	69.34156131	71.74888
Gglean016332	78.00798	73.3752731	69.34156131	71.74888
Gglean016333	0.4313318	0.127191104	0.225184622	0.409163
Gglean016334	0.4313318	0.127191104	0.225184622	0.409163
Gglean016335	17.71050637	19.33844	12.08836	8.744487
Gglean016336	0	0	0	0
Gglean016337	1.49986	1.414530382	0.953575129	1.03363
Gglean016338	1.14971	1.92972	1.37082	1.97057
Gglean016339	0.51805	0.445108	0.420175	0.583421
Gglean016340	32.36487337	27.86469	36.08320969	49.06300309
Gglean016341	3.921801	6.71198	8.2394	8.2800908
Gglean016342	4.9585	5.73339	5.79067	7.94997
Gglean016343	49.49700466	50.846335	76.745623	65.204439
Gglean016344	0.169482685	0.115155994	0.239119599	0.310433105
Gglean016345	40.8911	39.09492003	64.53047	75.27505
Gglean016346	0.0609881	0.0626439	0.0494767	0.0585906
Gglean016347	0	0	0	0
Gglean016348	114.597529	113.933617	51.7966196	49.1184974
Gglean016349	42.26531	43.18502	45.54695	47.88075
Gglean016350	14.5488	16.1675	8.53784	7.10277
Gglean016351	114.597529	113.933617	51.7966196	49.1184974
Gglean016352	39.77949	41.37819	32.92717	28.21907
Gglean016353	0	0	0	0
Gglean016354	30.8144	28.70336	34.1328	32.64596
Gglean016355	7.543694	7.616670552	8.73174053	7.7795861
Gglean016356	3.147916924	2.748469	1.82337	2.504949
Gglean016357	30.7193	35.0494	8.10398	7.02353
Gglean016358	10.45384	9.85259	7.11704	7.03723
Gglean016359	108.0759	110.8943	55.44677	41.85139
Gglean016360	14.72004715	13.48798422	19.24145516	15.43266173
Gglean016361	89.00679	92.40945902	124.5012148	113.2842384
Gglean016362	89.00679	92.40945902	124.5012148	113.2842384
Gglean016363	1013.73	739.175	443.392	385.291
Gglean016364	11.781723	10.543	8.5774	10.8402
Gglean016365	38.993271	37.53998097	22.75237386	21.45682553
Gglean016366	0	0	0	0
Gglean016367	23.92679	25.504268	39.192153	43.396031

Gglean016368	0	2.94528	0	5.06718
Gglean016369	37.2859	40.1329	30.2545	33.5865
Gglean016370	0.893331	0.798626	1.44998	2.09152
Gglean016371	3.022112	1.938629	3.904227	4.314983
Gglean016372	185.3054762	186.9197905	245.2659666	270.1186669
Gglean016373	185.3054762	186.9197905	245.2659666	270.1186669
Gglean016374	41.02309	38.82973	25.50085	22.17939
Gglean016375	41.02309	38.82973	25.50085	22.17939
Gglean016376	17.3855	18.9281	9.3539	7.11417
Gglean016377	363.4	402.6869	196.285	157.5133
Gglean016378	35.04248	37.53434	35.25004	29.91138
Gglean016379	35.04248	37.53434	35.25004	29.91138
Gglean016380	35.04248	37.53434	35.25004	29.91138
Gglean016381	0	0	0	0
Gglean016382	0.773191	0	1.30817	0.780661
Gglean016383	0	0	0	0
Gglean016384	0	0	0	0
Gglean016385	0	0	0	0
Gglean016386	26.70501	28.93222585	20.90882003	22.03221
Gglean016387	16.38161	15.015873	24.78305514	25.42171772
Gglean016388	81.2713	69.0389	53.1658	60.7386
Gglean016389	31.2948	34.8932	10.50759	11.23550003
Gglean016390	0.672623	0.726088	1.67093	0.667513
Gglean016391	610.916	711.3296	579.9771	700.3338
Gglean016392	21.9873	19.977	17.5405	9.928
Gglean016393	0.78013	0.949249	1.636678	1.606784
Gglean016394	13.78477188	9.018312	14.299	16.99974
Gglean016395	19.686896	23.742868	9.591290071	7.337300001
Gglean016396	4.317898014	4.727386421	5.42031076	7.31025937
Gglean016397	60.32196	65.55878	23.93034	18.81999
Gglean016398	60.32196	65.55878	23.93034	18.81999
Gglean016399	0.00107345	0.000654927	0.00172372	9.61E-05
Gglean016400	86.51419	92.36392	69.3587	63.60625
Gglean016401	86.51419	92.36392	69.3587	63.60625
Gglean016402	98.8529	94.8205	85.1726	77.4549
Gglean016403	2.97172	1.77769	1.8474	1.10791
Gglean016404	359.4467	405.5389	292.5698	313.8635
Gglean016405	19.50389	20.18088	21.86627	20.37536
Gglean016406	1020.9898	1022.491	871.634356	772.4131442
Gglean016407	9.61178	10.14287	5.60153	5.33797
Gglean016408	24.38905994	31.08205	31.11541	29.69492104
Gglean016409	80.800436	65.197769	62.880619	70.12291621
Gglean016410	0.434866	0.404725	0.20888517	0.680966578
Gglean016411	0	0	0	0

Gglean016412	17.32846	17.13299	6.1501	4.70156
Gglean016413	47.97586	56.4927558	33.88029677	29.693565
Gglean016414	8.11508	6.79816	10.4492	7.93259
Gglean016415	0.593524	1.404198	1.553865	1.54137
Gglean016416	42.76865301	42.33868	33.027283	31.616753
Gglean016417	0	0	0	0
Gglean016418	0	0	0	0
Gglean016419	11.695858	15.039595	20.4830964	26.921769
Gglean016420	0	0	0	0
Gglean016421	0	0	0	0.043823885
Gglean016422	0.596692877	0.631849178	1.1221081	1.98292962
Gglean016423	0.596692877	0.631849178	1.1221081	1.98292962
Gglean016424	42.94851	46.13674	38.78548	36.03983
Gglean016425	9.73E-27	9.21E-22	7.27E-19	0.00160278
Gglean016426	15.365	17.9992	11.6434	10.2731
Gglean016427	13.66266587	14.33704167	21.24655375	28.15834726
Gglean016428	13.66266587	14.33704167	21.24655375	28.15834726
Gglean016429	0.46304	0.400589	0.652478	0.535321
Gglean016430	47.5901	47.823616	49.1721434	49.84033
Gglean016431	59.3365	61.11206	59.9256	58.075
Gglean016432	30.62956	26.71185	28.83138	30.41971
Gglean016433	38.25472	37.26367	36.12539	33.73158
Gglean016434	28.94801158	29.50224458	30.1004	32.1627
Gglean016435	8.088390374	12.01249	8.27291021	9.2522774
Gglean016436	16.08994	14.16288	14.51125	12.91514
Gglean016437	33.78597	29.816798	51.3177	62.51229
Gglean016438	51.77175	56.49478	49.64148	49.59468
Gglean016439	51.77175	56.49478	49.64148	49.59468
Gglean016440	51.77175	56.49478	49.64148	49.59468
Gglean016441	23.57489	27.02157	13.60976	12.53389
Gglean016442	50.87289	53.29977	36.186531	34.28898
Gglean016443	14.97980979	22.03527	25.65388	22.90560176
Gglean016444	180.454	156.272	118.228	130.77
Gglean016445	0	0	0	0
Gglean016446	79.5119	56.3018373	50.71925	80.1601
Gglean016447	72.574	81.1989	114.734	129.88
Gglean016448	29.80056	30.33092	18.02044	17.2593847
Gglean016449	0.110829622	0.157562263	0.174882467	0.142512293
Gglean016450	59.8519	63.5422	94.9588	101.7875
Gglean016451	47.99065	41.63398	34.4308663	33.18859646
Gglean016452	23.52648	24.34731	25.460842	37.45625
Gglean016453	18.16474828	15.320335	14.3548	16.0863162
Gglean016454	18.16474828	15.320335	14.3548	16.0863162
Gglean016455	19.19279558	17.33161971	25.69736997	31.9226

Gglean016456	24.32603	28.839935	18.60105	21.03179
Gglean016457	1.56E-05	4.92E-06	0.677601	0.741433
Gglean016458	14.435	15.366	7.15083	7.2425
Gglean016459	73.83860021	69.88860241	74.33070111	107.2689028
Gglean016460	19.1327	22.2454	3.93766	3.73311
Gglean016461	1139.8015	1081.0584	727.06584	691.67289
Gglean016462	37.09724	39.13309103	38.95617673	36.1688603
Gglean016463	0	0	0	0
Gglean016464	3.189447009	3.060219983	3.6323458	4.132737605
Gglean016465	3.189447009	3.060219983	3.6323458	4.132737605
Gglean016466	24.63229967	22.70111645	16.59931519	15.42904497
Gglean016467	24.0714	20.7362	9.21219	8.28852
Gglean016468	106.38872	108.335	66.13167	61.54691759
Gglean016469	29.83630001	26.95530005	35.738265	41.353931
Gglean016470	12.47619133	12.86613126	5.190414446	5.242470121
Gglean016471	37.11185	36.31001	63.52442	62.85345
Gglean016472	20.605348	13.35158	18.353644	12.610965
Gglean016473	6.81289	6.147056	9.91141	14.87588
Gglean016474	34.62010148	35.34550048	43.30192	46.05650051
Gglean016475	1.89568	2.40195	1.05715	0.943617
Gglean016476	56.18668	51.80016	57.55926	56.50402
Gglean016477	9.924998927	9.45668516	10.89643	11.25335057
Gglean016478	0.266345341	0.272407001	0.499533	0.44236757
Gglean016479	31.1746	27.252	28.1463	24.8115
Gglean016480	66.62424826	66.48059027	100.9921156	83.85954252
Gglean016481	66.62424826	66.48059027	100.9921156	83.85954252
Gglean016482	46.600343	53.04656	70.943981	66.43075
Gglean016483	46.600343	53.04656	70.943981	66.43075
Gglean016484	0.407101	0.445006	0.335921	0
Gglean016485	0	0	0	0
Gglean016486	24.16174	23.15728	15.27198	16.61903
Gglean016487	24.16174	23.15728	15.27198	16.61903
Gglean016488	103.28838	99.55134	42.85828	35.46774
Gglean016489	103.28838	99.55134	42.85828	35.46774
Gglean016490	10.212023	8.625707566	9.89515	11.00396
Gglean016491	32.79335	33.88705	44.84915	41.34837
Gglean016492	215.6237526	219.9254238	236.4024381	326.3377184
Gglean016493	420.3994	445.4434	279.063	271.1125
Gglean016494	3.1321	4.77808	6.13365	7.43987
Gglean016495	147.8336049	147.1781526	126.7667577	124.7320249
Gglean016496	147.8336049	147.1781526	126.7667577	124.7320249
Gglean016497	15.1926	15.5204	5.1572	3.8939
Gglean016498	5.38928	5.16859	9.62761	13.6001
Gglean016499	47.62411815	44.556276	20.20953031	20.19535538

Gglean016500	47.62411815	44.556276	20.20953031	20.19535538
Gglean016501	12.7125	11.4645	13.106	11.8295
Gglean016502	0	0	0	0
Gglean016503	0	0	0.189944	0
Gglean016504	0	0	0.121444	0
Gglean016505	36.71724967	40.90697796	27.56078461	22.46292933
Gglean016506	30.55261717	31.95202701	21.669697	19.42600346
Gglean016507	0	0	0.138706647	0.057493295
Gglean016508	20.62303031	20.06449538	18.82305562	17.63084496
Gglean016509	27.1483	33.5498	18.0284	16.6679
Gglean016510	0.0717875	0	0.233916	0.0692481
Gglean016511	175.745	177.101	98.6936	95.5979
Gglean016512	20.75951	20.23824	15.49618	15.89127
Gglean016513	0.0677836	0	0	0
Gglean016514	4.362582	4.96828	6.816308	5.630704718
Gglean016515	23.89347	19.51909	56.75783	64.87326
Gglean016516	8.121023	7.461814	6.21035	6.853727
Gglean016517	123.7905	151.1337	78.7244	67.6782
Gglean016518	95.00719	91.49348	105.72923	134.42801
Gglean016519	95.00719	91.49348	105.72923	134.42801
Gglean016520	75.86746713	81.94729238	112.4026745	136.02431
Gglean016521	85.805053	87.55584136	41.66233	35.495216
Gglean016522	85.805053	87.55584136	41.66233	35.495216
Gglean016523	85.805053	87.55584136	41.66233	35.495216
Gglean016524	42.4992	42.5668	22.7276	19.8824
Gglean016525	15.32242	14.07183	15.40364	15.06872
Gglean016526	1.585093774	1.25385625	1.75922502	2.690687
Gglean016527	46.81006	46.44913	37.5673	36.375692
Gglean016528	46.81006	46.44913	37.5673	36.375692
Gglean016529	33.71676	33.80462	46.55275	53.18165
Gglean016530	0.1440873	0.067988	0.206173	0.5468164
Gglean016531	309.371	301.526	43.383	13.9719
Gglean016532	93.79414	94.68648	92.36686	84.12116
Gglean016533	80.50268545	84.271252	49.30037645	44.16144166
Gglean016534	80.50268545	84.271252	49.30037645	44.16144166
Gglean016535	0	0	0	0
Gglean016536	0	0	0	0
Gglean016537	68.9732	67.5414	75.4366	58.3817
Gglean016538	52.66212288	57.8132321	63.097688	73.09761
Gglean016539	0	0	0	0
Gglean016540	0	0	0	0
Gglean016541	0	0	0.0444728	0
Gglean016542	0	0	0	0
Gglean016543	0	0	0	0

Gglean016544	0	0	0	0
Gglean016545	0	0	0	0
Gglean016546	0	0	0	0
Gglean016547	0	0	0	0
Gglean016548	0	0	0	0
Gglean016549	31.94026	30.95231	15.39388	12.750849
Gglean016550	1.382022814	2.149414	1.957171893	2.265488481
Gglean016551	0.0696298	0.218108	0	0.202403
Gglean016552	3.988490002	3.69356	5.747920005	6.94057
Gglean016553	0.236525	0	0.0955815	0
Gglean016554	0	0	0	0
Gglean016555	0	0	0	0
Gglean016556	0	0	7.08943	8.3171
Gglean016557	4.1577908	3.443182	4.5226925	6.72807
Gglean016558	0	0	0	0.163827
Gglean016559	0	0	0	0
Gglean016560	0.114439	0.117696	0.186258	0
Gglean016561	0	0.255634012	0	0.05659389
Gglean016562	0	0.255634012	0	0.05659389
Gglean016563	0	0	0	0
Gglean016564	0	0	0	0
Gglean016565	1.5919	1.02328	0.787172	0.628444
Gglean016566	0	0	0	0
Gglean016567	0	0	0	0
Gglean016568	0	0	0.146691	0
Gglean016569	0	0	0	0
Gglean016570	0.122943	0	0	0
Gglean016571	0	0.106382	0	0.0999975
Gglean016572	0	0	0	0
Gglean016573	0	0	0	0
Gglean016574	0	0	0	0
Gglean016575	0	0	0	0
Gglean016576	0	0	0	0
Gglean016577	0	0	0.0292585	0
Gglean016578	0	0	0	0
Gglean016579	0	0	0	0
Gglean016580	0	0	0	0
Gglean016581	0	0	0	0
Gglean016582	0	0.029851168	0	0
Gglean016583	0.975582	0.530659	1.21577	0.96949
Gglean016584	0.485184923	0.861907057	0.498556	0.551887538
Gglean016585	1.610493269	1.943334765	1.686705802	2.129674305
Gglean016586	1.610493269	1.943334765	1.686705802	2.129674305
Gglean016587	0	0	0	0

Gglean016588	0	0	0	0
Gglean016589	0	0	0	0
Gglean016590	0	0	0	0
Gglean016591	0.260575	0.335695	0.732572	0.375328
Gglean016592	19.60358996	17.19576401	23.829593	24.99201955
Gglean016593	12.64194	12.207474	9.756723	10.524439
Gglean016594	138.6	144.224	62.0019	57.4882
Gglean016595	64.5124692	66.76647187	66.93118135	64.77123031
Gglean016596	18.98246	19.66751	27.62738	28.67743
Gglean016597	1.1058369	1.2074372	2.43957466	2.357918
Gglean016598	1.1058369	1.2074372	2.43957466	2.357918
Gglean016599	19.68906946	19.33752	30.65020009	34.2665
Gglean016600	10.93494	13.84532	17.33516	18.27405
Gglean016601	15.2002	16.5571	9.58731	8.73755
Gglean016602	73.60732	75.03431	59.51854	58.78735
Gglean016603	20.37809	21.28979748	28.21597	25.73861
Gglean016604	18.20238008	15.93853566	9.9669496	9.509841654
Gglean016605	53.56974773	59.10178204	80.00286441	69.62437172
Gglean016606	53.56974773	59.10178204	80.00286441	69.62437172
Gglean016607	0	0	0	0
Gglean016608	50.41761	52.71577	57.2623757	57.906712
Gglean016609	16.67698304	15.72227	17.88491864	18.87222157
Gglean016610	54.8995	54.2858	41.0887	35.1976
Gglean016611	29.644648	28.393653	22.317984	21.039923
Gglean016612	0	0	0	0.0798155
Gglean016613	289.3487	277.1863	229.1112	270.4488
Gglean016614	0	1.13982	0	0
Gglean016615	0	0	0	0.0434981
Gglean016616	0	0	0	0.0658201
Gglean016617	2.46E-06	0.291344	0.110677	1.88E-07
Gglean016618	0	0	0	0
Gglean016619	0	0	0	0
Gglean016620	0	0	0	0
Gglean016621	0	0.276895	0	0
Gglean016622	0	0.0438143	0	0.0411552
Gglean016623	0	0	0	0
Gglean016624	0	0	0	0
Gglean016625	0	0	0	0
Gglean016626	0	0	0	0
Gglean016627	0	0	0.709403	0
Gglean016628	0	0	0	0
Gglean016629	0	0	0	0
Gglean016630	0	0	0	0
Gglean016631	198.8114	220.6605073	119.588437	124.2997427

Gglean016632	0	0	0	0
Gglean016633	0	0.250185	0	0
Gglean016634	8.063534082	7.364657871	13.35237413	13.67287465
Gglean016635	80.7141	52.1121	37.3448	43.335
Gglean016636	41.95742	34.07502	51.2299	47.178622
Gglean016637	41.95742	34.07502	51.2299	47.178622
Gglean016638	21.4718	19.1906	13.4114	9.71114
Gglean016639	93.09756	98.03351	108.93127	114.70577
Gglean016640	16.93167542	18.84047	14.35465	15.41027
Gglean016641	16.93167542	18.84047	14.35465	15.41027
Gglean016642	16.93167542	18.84047	14.35465	15.41027
Gglean016643	9.645020301	8.08528766	12.92597834	13.33534963
Gglean016644	15.20162308	14.50102738	16.407909	17.41511545
Gglean016645	3.294866	2.173207737	3.489466	6.876866
Gglean016646	47.29509943	50.37116544	63.00496602	81.97223539
Gglean016647	8.19377	8.753253881	2.890905	1.744772717
Gglean016648	35.2141	39.9826	28.6653	25.5681
Gglean016649	29.458867	30.71340407	34.302131	31.13311464
Gglean016650	17.9383	22.5949	16.6435	15.5323
Gglean016651	0	0	0	0
Gglean016652	17.69766265	16.54456435	20.4990531	21.67433786
Gglean016653	0	0	0	0
Gglean016654	26.1363	19.2285	31.743128	36.1199
Gglean016655	0	0	0	0
Gglean016656	1.08743	1.14214	0.177353	1.27045
Gglean016657	37.2438	40.996	20.2919	17.4616
Gglean016658	29.95099142	37.3707231	30.04638168	26.55063827
Gglean016659	43.12839433	47.72400018	47.03081706	47.4263717
Gglean016660	1478.272713	1615.596004	1057.05107	961.749186
Gglean016661	15.2298	14.3675	9.60412	9.76994
Gglean016662	9.64031	9.23001	10.39359	12.88076
Gglean016663	46.46813	43.35038898	68.79371	66.92147
Gglean016664	11.735554	10.474518	12.656044	14.347901
Gglean016665	18.88364344	18.81530607	19.51807215	26.89434676
Gglean016666	14.910791	14.898764	20.451279	23.30566874
Gglean016667	0.222188	0	0.36617	0
Gglean016668	22.28093	26.61494	28.599085	33.762211
Gglean016669	22.28093	26.61494	28.599085	33.762211
Gglean016670	0	0	0	0
Gglean016671	126.8979	111.7893	141.8579	107.9892
Gglean016672	0	0	0	0
Gglean016673	16.77138627	18.15083562	10.70415014	8.650515312
Gglean016674	20.75173873	20.19010259	38.34291223	38.8954037
Gglean016675	9.8048062	10.3577862	3.8344867	3.1574138

Gglean016676	9.8048062	10.3577862	3.8344867	3.1574138
Gglean016677	0	0	0	0
Gglean016678	18.939891	18.211249	21.625475	19.906516
Gglean016679	18.939891	18.211249	21.625475	19.906516
Gglean016680	0	0	0	0
Gglean016681	38.30723267	39.80472255	45.96579128	53.67764812
Gglean016682	113.90325	122.6358203	164.8949525	181.91206
Gglean016683	113.90325	122.6358203	164.8949525	181.91206
Gglean016684	1.12706	0.22562	2.77E-05	2.49E-06
Gglean016685	21.38210093	25.07244985	25.14373337	29.40858059
Gglean016686	14.04288306	14.25786665	16.20716929	20.91226225
Gglean016687	0	0	0	0
Gglean016688	0	0	0	0
Gglean016689	81.70415	77.605497	101.612782	103.042538
Gglean016690	62.06956	68.2708	65.0117	68.61059
Gglean016691	0	0	0	0
Gglean016692	0.054613718	0	0.061814585	0
Gglean016693	7.40932831	6.6930362	10.94684913	12.32699608
Gglean016694	0.139015	0	0	0
Gglean016695	0	0	0	0
Gglean016696	1.019542814	1.638303	3.734941	3.795299
Gglean016697	31.9388	29.6618	44.6531	58.9359
Gglean016698	0	0	0	0
Gglean016699	0	0.0638222	0	0
Gglean016700	0	0	0.0897453	0
Gglean016701	0	0	0	0
Gglean016702	0.9977521	1.275969	0.353380326	0.381838016
Gglean016703	3.58837	4.033006	16.215364	4.737221
Gglean016704	3.58837	4.033006	16.215364	4.737221
Gglean016705	0	0	0	0.0987113
Gglean016706	0.641041	1.261841237	2.344789666	2.2256237
Gglean016707	0	0	0	0
Gglean016708	0	0	0	0
Gglean016709	0.946967742	1.511747	1.393997	2.544617
Gglean016710	0	0	0	0
Gglean016711	0	0	0	0
Gglean016712	0	0	0	0
Gglean016713	0	0	0	0
Gglean016714	0.152996	0.0948364	0.0743687	0.0586659
Gglean016715	0	0	0.026001	0.024829107
Gglean016716	8.03324	9.44267	13.07254	11.17682
Gglean016717	0	0.2402	0.127093	0.15095
Gglean016718	2.693072	2.274043	3.360295	4.034
Gglean016719	25.05399	25.19746	35.94745	33.77294

Gglean016720	24.70075	28.42944	26.29256	30.1018
Gglean016721	22.44277834	19.27376711	17.74039157	19.01782212
Gglean016722	0.000251974	0.223136	0.000111892	0.250916
Gglean016723	0	0	0	0
Gglean016724	5.291267289	4.952092188	9.420069352	9.844206
Gglean016725	8.37366	9.07771	7.35086	5.663437
Gglean016726	163.0069	149.8867	164.1125	214.4483
Gglean016727	0	0	0	0
Gglean016728	0	0.0408265	0.0651657	0.0772704
Gglean016729	0.606183	1.68676	3.1485	3.70146
Gglean016730	13.538927	13.08328222	7.729697	6.841260953
Gglean016731	13.538927	13.08328222	7.729697	6.841260953
Gglean016732	13.538927	13.08328222	7.729697	6.841260953
Gglean016733	13.538927	13.08328222	7.729697	6.841260953
Gglean016734	0	0	0.426449655	0.211676875
Gglean016735	0	0	0.426449655	0.211676875
Gglean016736	0	0	0	0
Gglean016737	0.634432	1.41929	2.1171	3.16078
Gglean016738	0	0	0	0
Gglean016739	0.1309153	0.1546913	0.1492531	0.107943849
Gglean016740	2.095842905	2.812252455	2.347647806	2.020632993
Gglean016741	1.26592568	1.58413232	1.588051687	1.369362426
Gglean016742	0	0	0	0
Gglean016743	0	1.00535	0	0
Gglean016744	0	0	0	0
Gglean016745	0	0	0	0
Gglean016746	0	0	0	0
Gglean016747	0	0	0	0
Gglean016748	3.084089	2.130220038	3.583790051	3.891950167
Gglean016749	10.5955	13.3743	13.9096	22.6458
Gglean016750	25.13112	20.96454	19.62167	19.19935
Gglean016751	0.636395	1.050687	1.303248	1.681542
Gglean016752	15.04779	17.95759	7.14784	6.363794
Gglean016753	0.331829	0.0979417	0.384455	0.363898
Gglean016754	6.52723	5.89252	6.16432	8.50063
Gglean016755	151.9354	152.7614	93.2949	86.4928
Gglean016756	17.952399	20.196863	15.46366	16.369868
Gglean016757	0.98605	1.29568	1.22295	1.22381
Gglean016758	79.114	82.2494	57.3552	79.913
Gglean016759	1842.137	1521.7768	1640.6818	2462.575
Gglean016760	1.798710001	0.469127511	2.383610287	3.62318
Gglean016761	1842.137	1521.7768	1640.6818	2462.575
Gglean016762	0	0	0	0
Gglean016763	12.31275	23.21842	3.75306	4.946979

Gglean016764	0	0	0	1.0323
Gglean016765	0	1.23753	0	1.10027
Gglean016766	0.471974	0	0.12833	0.304585
Gglean016767	0	0	0	0
Gglean016768	1.55E-10	0.301176	0.115317	9.46E-10
Gglean016769	0	0	0	0.135993
Gglean016770	0	0	0	0
Gglean016771	0	0	0	0
Gglean016772	0	0	0	0
Gglean016773	0	0	0	0
Gglean016774	0	0	0	0
Gglean016775	59.907	53.6758	48.7658	47.2706
Gglean016776	11.8966	15.0175	9.62351	12.2575
Gglean016777	45.1706	45.7535	42.2945	50.128
Gglean016778	92.9362214	83.62281839	75.6940089	83.91351549
Gglean016779	92.9362214	83.62281839	75.6940089	83.91351549
Gglean016780	60.29170946	62.61953048	40.28583052	34.16946597
Gglean016781	10.1203	10.3181	10.7049	15.4214
Gglean016782	262.134	278.849	245.208	442.085
Gglean016783	11.6419	46.0055	48.8352	71.3292
Gglean016784	0	0	0.0398342	0.0477033
Gglean016785	0.0900753	0	0.143764	0
Gglean016786	0	0	0	0
Gglean016787	420.185	339.981	401.109	580.111
Gglean016788	23.46814	27.64982	23.20165	25.29257
Gglean016789	55.993405	57.404888	82.572435	94.451474
Gglean016790	32.6796	32.9987	23.9014	5.31784
Gglean016791	42.05557	44.61334124	20.10537726	16.32840001
Gglean016792	42.05557	44.61334124	20.10537726	16.32840001
Gglean016793	15.88480946	15.75542211	8.2074171	10.15616
Gglean016794	143.8578	132.9431	129.8553	123.1895
Gglean016795	42.92644016	46.1189758	54.31415	53.64746
Gglean016796	108.6075	123.2251	144.6221	200.1741
Gglean016797	0	0	0.0476749	0
Gglean016798	42.3448	46.61825	32.966	33.7537
Gglean016799	0.000594212	0.000250411	1.8386	0.00011624
Gglean016800	62.85461312	60.982523	36.64146	32.013637
Gglean016801	62.85461312	60.982523	36.64146	32.013637
Gglean016802	62.85461312	60.982523	36.64146	32.013637
Gglean016803	0	0	0	1.5704
Gglean016804	13.50302	14.54045	6.35735	4.754373
Gglean016805	27.5633	26.825	24.95191	39.5605
Gglean016806	7.45967	6.95840735	9.12799	22.5639
Gglean016807	7.45967	6.95840735	9.12799	22.5639

Gglean016808	68.029088	64.18541	56.38298	60.01833
Gglean016809	68.029088	64.18541	56.38298	60.01833
Gglean016810	15.65612	15.91543	15.55746	15.235048
Gglean016811	19.588316	18.31184459	8.242731068	7.55858
Gglean016812	90.82751819	97.00715586	131.7349823	144.9264607
Gglean016813	90.82751819	97.00715586	131.7349823	144.9264607
Gglean016814	8.21112	8.35564	7.05083	8.44184
Gglean016815	58.544555	54.33411	47.24477	49.3132
Gglean016816	16.597678	15.687525	26.222346	27.525722
Gglean016817	0	0	0	0
Gglean016818	0	0	0	0
Gglean016819	4.295663	5.95366496	7.891403	7.80179
Gglean016820	4.295663	5.95366496	7.891403	7.80179
Gglean016821	0	0.498001	0.379346	0
Gglean016822	7.17811	5.22079	12.1373	9.97272
Gglean016823	19.172132	17.7036004	30.228085	31.33860028
Gglean016824	50.7871698	51.86614828	44.11410189	40.53810776
Gglean016825	50.7871698	51.86614828	44.11410189	40.53810776
Gglean016826	0	0	0	0
Gglean016827	14.66994	16.02359908	8.089959	6.9759359
Gglean016828	28.24641548	28.539026	14.325837	12.98569527
Gglean016829	1.239168	0.747257263	0.683665	1.733234
Gglean016830	8.14711	7.3151	11.3093	9.71151
Gglean016831	6.2402546	5.793826	5.223392745	5.030467
Gglean016832	29.25434014	29.79882556	26.27841977	24.31787819
Gglean016833	0.170167541	0.217245441	0.325903517	0.304979163
Gglean016834	62.935259	67.369622	59.178454	59.400114
Gglean016835	0	0	0	0
Gglean016836	0	0	0	0
Gglean016837	62.564713	64.507952	54.075568	58.852475
Gglean016838	3.64E-05	2.98E-05	0.000220677	1.85E-05
Gglean016839	0.29017	0.149178	0.234549	0.555588
Gglean016840	36.87915	36.36204	14.22362	11.86338
Gglean016841	0.829191	1.23834	0.66826	1.22595
Gglean016842	50.17127822	51.131217	59.75549417	51.29443826
Gglean016843	50.17127822	51.131217	59.75549417	51.29443826
Gglean016844	0	0	0	0
Gglean016845	20.95518	15.80554	14.32579	12.24094
Gglean016846	25.38902	23.64919	19.52785	19.8642
Gglean016847	38.9789	37.72866	38.2617	40.9441
Gglean016848	25.351406	30.17214	39.64689	50.50165
Gglean016849	53.98494291	61.92879242	22.91638612	15.41281
Gglean016850	33.88199232	22.43007282	26.9300621	26.824769
Gglean016851	19.09837	11.114	14.75616	10.5424824

Gglean016852	15.7391585	17.1108124	19.25751683	22.13324394
Gglean016853	25.6673	29.421	7.68521	6.12053
Gglean016854	65.62903318	77.01486766	56.95630412	50.19447336
Gglean016855	65.62903318	77.01486766	56.95630412	50.19447336
Gglean016856	59.57597722	69.77342519	61.60868576	63.75593872
Gglean016857	17.4094	31.1408	26.3229	25.3148
Gglean016858	23.458	27.9286	19.1544	20.0816
Gglean016859	33.3573	29.11005	28.4171	26.65823
Gglean016860	62.95932	70.4934	10.99436	10.51142
Gglean016861	51.1077	50.2864	50.308	50.4878
Gglean016862	24.26048	28.83343	18.5761	16.029094
Gglean016863	24.26048	28.83343	18.5761	16.029094
Gglean016864	3.300837	2.760729	4.67679042	4.80673975
Gglean016865	16.96392	10.97908846	22.4705006	31.8815088
Gglean016866	5.76327	7.862	7.64502	12.3445
Gglean016867	0.132992351	0.3073618	0.0772306	0.385317
Gglean016868	2.3743	1.986367	2.386848512	1.884639
Gglean016869	4.373834392	4.386139	8.90153	8.3474
Gglean016870	0.413532	0.631266	0.44235	0.45341
Gglean016871	0	0	0	0
Gglean016872	0	0	0.017860515	0
Gglean016873	0.158484	0.109617	0.129106	0.0509049
Gglean016874	9.79096	9.27019	10.9793	14.9077
Gglean016875	5.42283	8.21715	3.07987	0.631516
Gglean016876	56.8022	62.53689	36.55348	33.5742
Gglean016877	28.02502391	29.44832678	14.29780716	12.01574508
Gglean016878	0	0	0	0
Gglean016879	12.8534	25.6764	54.6641	62.6584
Gglean016880	0	0	0	0
Gglean016881	135.1787484	136.2541628	143.92074	148.7547558
Gglean016882	33.95808	28.76034	26.64348	26.88458
Gglean016883	33.95808	28.76034	26.64348	26.88458
Gglean016884	49.02337798	45.60416267	41.02378667	56.86613248
Gglean016885	49.02337798	45.60416267	41.02378667	56.86613248
Gglean016886	20.52139	14.51929	27.7081	35.9767
Gglean016887	0	0	0	0
Gglean016888	14.8948	11.0984	8.63776	6.40533
Gglean016889	118.21136	126.0752	116.269647	130.748392
Gglean016890	118.21136	126.0752	116.269647	130.748392
Gglean016891	18.87822	18.28868	6.86386	6.03347
Gglean016892	0.02358033	0	0.111038172	0.0218795
Gglean016893	0	0	0	0
Gglean016894	0	0	0	0
Gglean016895	16.27191	13.40806	10.2359	9.03462

Gglean016896	30.80652	27.7172	12.22404	7.25824
Gglean016897	17.369558	21.2803	18.01700319	18.25660066
Gglean016898	4.26132	3.40515	5.48469	4.03536
Gglean016899	22.709442	24.12971	25.7846	28.85932
Gglean016900	0	0	0	0
Gglean016901	99.898	107.3588	59.0539	47.00669
Gglean016902	28.58581	30.19584	25.01379	25.61858
Gglean016903	28.58581	30.19584	25.01379	25.61858
Gglean016904	10.507849	11.012886	11.422614	11.046359
Gglean016905	11.5823	10.5113	16.7534	18.8885
Gglean016906	8.310599671	9.169900001	4.884270251	4.457172925
Gglean016907	8.310599671	9.169900001	4.884270251	4.457172925
Gglean016908	17.09342821	16.61746555	13.32595943	12.271139
Gglean016909	17.09342821	16.61746555	13.32595943	12.271139
Gglean016910	0	0	0	0
Gglean016911	0	0	0	0
Gglean016912	51.39869	69.65816	104.39806	98.14286
Gglean016913	51.39869	69.65816	104.39806	98.14286
Gglean016914	59.968	56.804	93.4692	90.4962
Gglean016915	14.792913	14.58234219	13.60533872	11.7360511
Gglean016916	2.658976265	2.596710747	2.432570359	2.526803449
Gglean016917	158.28	140.315	166.646	294.377
Gglean016918	23.38906	22.12047	26.04184	34.60627
Gglean016919	5.589399516	5.098796653	4.57250622	4.044614789
Gglean016920	5.589399516	5.098796653	4.57250622	4.044614789
Gglean016921	0	0	0	0
Gglean016922	24.5588	27.3167	14.3652	13.0237
Gglean016923	0	0	0.048302406	0.081309991
Gglean016924	0	0	1.03811	0
Gglean016925	2.57749	2.02834	3.12813	3.59766
Gglean016926	122.4535	107.49876	132.6903	138.0138
Gglean016927	0.135162	0.194572	0.0439403	0.0781782
Gglean016928	0	0.143102	0.111964	0
Gglean016929	0.744616	0	0	0
Gglean016930	56.74369	55.671	53.85544	46.33728
Gglean016931	0	0	0	0
Gglean016932	16.9182105	17.216615	25.82537522	30.31425506
Gglean016933	16.9182105	17.216615	25.82537522	30.31425506
Gglean016934	16.9182105	17.216615	25.82537522	30.31425506
Gglean016935	79.23724753	62.61361215	66.479776	63.94474272
Gglean016936	79.23724753	62.61361215	66.479776	63.94474272
Gglean016937	79.23724753	62.61361215	66.479776	63.94474272
Gglean016938	79.23724753	62.61361215	66.479776	63.94474272
Gglean016939	32.51481	31.52156	28.00592	28.79365

Gglean016940	15.7498	17.2726	15.7496	15.7556
Gglean016941	28.9844	26.9507	12.69151	12.66958
Gglean016942	54.7607	49.3872	33.0103	27.763
Gglean016943	6.139308	7.0139	2.7890841	2.582505
Gglean016944	11.46268111	10.803383	14.75279405	18.80983268
Gglean016945	38.80121	38.89016	18.21923	17.90645
Gglean016946	83.29539086	94.74704729	73.11783951	56.71254942
Gglean016947	14.8704	14.8606	15.6591	12.8206
Gglean016948	1.245016	1.391421706	2.157083176	2.7241034
Gglean016949	21.3728	20.19118	21.331808	22.46018138
Gglean016950	21.3728	20.19118	21.331808	22.46018138
Gglean016951	0	0	0	0
Gglean016952	24.2174	11.0748	43.219	27.863
Gglean016953	115.44	91.8178	84.9837	205.096
Gglean016954	16.5111	16.8011	9.47806	8.02514
Gglean016955	41.30394	42.0635	33.85288	31.62717
Gglean016956	25.76553	25.50735237	33.18392696	41.042897
Gglean016957	42.1093	40.02511	74.9205	86.4119
Gglean016958	42.1093	40.02511	74.9205	86.4119
Gglean016959	260.4111	254.6975	176.5372	183.4816
Gglean016960	13.34282	13.12181	9.36395	9.33015
Gglean016961	0	0	0	0
Gglean016962	239.109	195.076	185.371	310.961
Gglean016963	0	0	0	0
Gglean016964	52.88104564	54.373049	53.11335399	51.30363249
Gglean016965	64.81978381	59.514671	71.154339	109.642772
Gglean016966	38.10088	40.62587	30.47198	32.01129
Gglean016967	88.64270348	116.7230066	102.6725159	104.3449229
Gglean016968	0.20635	0.43763	0	0.607412
Gglean016969	0	0	0	0
Gglean016970	24.557185	27.41161832	14.50736705	13.12180869
Gglean016971	14.181137	13.18854844	14.49815731	16.74815529
Gglean016972	0	0	0	0
Gglean016973	176.2906477	189.15671	329.6567086	326.3222055
Gglean016974	0	0	0	0
Gglean016975	1.9795298	2.1179879	4.1253798	4.8058407
Gglean016976	10.08126	9.55825	16.40845	18.63999
Gglean016977	23.4338875	22.137282	28.74233548	34.3782359
Gglean016978	3.05656462	3.72688218	2.685534	4.040324
Gglean016979	5.495523	7.59925425	10.20494562	12.904291
Gglean016980	35.79250233	22.87169963	30.78820236	47.31929873
Gglean016981	35.79250233	22.87169963	30.78820236	47.31929873
Gglean016982	1.032821	0.515249235	1.6882075	1.705765
Gglean016983	40.19338196	40.131526	47.821382	66.26993

Gglean016984	37.4265	41.515	26.0496	20.363
Gglean016985	30.1791	30.3115	23.7879	25.178
Gglean016986	63.09744638	53.63465252	78.78054057	87.60939
Gglean016987	63.09744638	53.63465252	78.78054057	87.60939
Gglean016988	111.855	106.2617	111.1117	122.8718
Gglean016989	9.757880349	8.81084696	6.468574871	7.639635563
Gglean016990	44.74401459	39.15492545	60.21190679	60.19249
Gglean016991	16.27432	17.12907016	5.603687619	5.848255911
Gglean016992	14.43598	10.77023	5.44	6.94302
Gglean016993	79.2372	80.40974	79.26924	87.82537
Gglean016994	9.40213	9.69646	7.30189	6.57809
Gglean016995	28.085685	26.309814	32.16429	28.88609
Gglean016996	28.085685	26.309814	32.16429	28.88609
Gglean016997	0.00126267	7.73E-08	0.812205	0.18547
Gglean016998	28.085685	26.309814	32.16429	28.88609
Gglean016999	25.79823921	34.15541124	39.6353	40.61011391
Gglean017000	48.8532	43.5671	36.9463	39.0664
Gglean017001	0	0	0	0
Gglean017002	0	0	0	0
Gglean017003	18.0750304	19.2867083	28.7081824	30.39550355
Gglean017004	18.0750304	19.2867083	28.7081824	30.39550355
Gglean017005	119.042245	143.0788698	75.840142	66.498732
Gglean017006	17.3183	15.8416	15.929	16.9977
Gglean017007	0	0	0	0
Gglean017008	42.83460171	47.719344	55.3723099	55.3923602
Gglean017009	0.223887	0	0	0
Gglean017010	34.94384043	37.74582875	29.64645267	27.73766798
Gglean017011	0	0	0	0
Gglean017012	0	0	0	0
Gglean017013	0	0.00391022	1.57E-06	0
Gglean017014	0	0.00391022	1.57E-06	0
Gglean017015	0.123842	0	0.024886	0.0295215
Gglean017016	11.04830469	9.003846407	15.12543519	16.14872
Gglean017017	4.89322	6.2781	9.25702	10.3548
Gglean017018	1127.733	1189.8324	728.2828	750.876
Gglean017019	4.824644	4.924805	3.6342043	3.049521
Gglean017020	116.858	103.698	68.83	68.3378
Gglean017021	5.019473838	5.7247676	8.0150558	9.071711
Gglean017022	23.32001301	25.25315773	24.1619634	25.00700724
Gglean017023	68.2114	71.8098	45.5826	41.1251
Gglean017024	36.1175	39.2991	8.96036	7.63819
Gglean017025	70.255644	82.820332	105.2225786	118.647636
Gglean017026	70.255644	82.820332	105.2225786	118.647636
Gglean017027	9.18596	8.07524	10.0462	8.47718

Gglean017028	13.9505	15.22831	17.22958	18.29642
Gglean017029	0	0	0	0
Gglean017030	0	0	0.215465	0
Gglean017031	8.13E-05	0.00354159	5.73E-05	0.725417
Gglean017032	1.433587	0.5510427	0.565803	0.306829
Gglean017033	0.0991743	0.203578	0	0
Gglean017034	1041.232	1132.868	716.605	659.3782
Gglean017035	12.93725	15.849884	12.317316	12.207386
Gglean017036	7.48217	6.33939	9.837179688	10.48647213
Gglean017037	45.39591994	45.47570563	36.39236344	31.07355389
Gglean017038	45.39591994	45.47570563	36.39236344	31.07355389
Gglean017039	0	0	0	0
Gglean017040	27.51747	29.27337058	26.37457	25.74072731
Gglean017041	1.39118512	2.378590279	2.099259742	2.406458013
Gglean017042	71.24605453	81.83406949	122.267948	109.7233807
Gglean017043	71.24605453	81.83406949	122.267948	109.7233807
Gglean017044	0	0	0.631437	0.756694
Gglean017045	64.24444516	65.90415069	52.52001499	47.0845413
Gglean017046	36.037008	33.295828	21.351806	19.68605453
Gglean017047	120.9655	111.5379	74.0563	77.6112
Gglean017048	120.9655	111.5379	74.0563	77.6112
Gglean017049	97.9594	93.59584	83.2246	90.10442
Gglean017050	165.98159	168.48115	152.30241	144.02718
Gglean017051	0	0	0	0
Gglean017052	0.0355365	0	0	0
Gglean017053	0.156935	0.0818142	0.0635352	0.0758467
Gglean017054	46.5683	61.174	31.1247	30.5485
Gglean017055	134.9182382	140.1291271	130.8274423	115.8318043
Gglean017056	77.3598	79.43884	74.71189148	71.70265
Gglean017057	77.3598	79.43884	74.71189148	71.70265
Gglean017058	0	0	0	0
Gglean017059	0	0	0	0
Gglean017060	31.03787244	26.3259909	40.67298653	44.30842047
Gglean017061	41.503734	46.73972071	39.26426109	36.10614548
Gglean017062	0	0	0	0
Gglean017063	10.369365	9.742629128	18.0652036	19.63097389
Gglean017064	0	0	0	0
Gglean017065	24.8826525	26.096985	29.248117	26.67748
Gglean017066	471.9198	519.5073	1112.845	1204.825
Gglean017067	1.593142894	1.465340892	1.448582051	1.842263593
Gglean017068	1.593142894	1.465340892	1.448582051	1.842263593
Gglean017069	20.734315	19.150012	5.91739343	5.325664
Gglean017070	22.04110009	23.79956	17.320285	18.52210009
Gglean017071	67.68132366	66.42687901	67.59371108	65.1148554

Gglean017072	67.68132366	66.42687901	67.59371108	65.1148554
Gglean017073	67.68132366	66.42687901	67.59371108	65.1148554
Gglean017074	358.1463	301.3702	687.5164	669.9472
Gglean017075	55.20280518	57.81452151	82.95812623	93.16942083
Gglean017076	202.7958835	210.1882803	234.24712	270.659678
Gglean017077	11.27135	16.21437	20.45907	22.77021
Gglean017078	8.93403	6.652229	13.814653	15.17191072
Gglean017079	14.654075	13.794432	10.007955	9.082923
Gglean017080	49.83	53.00076	39.68337	34.80792
Gglean017081	45.20601937	35.11469399	60.495408	63.3816119
Gglean017082	31.213101	31.51182	20.352103	18.4529
Gglean017083	44.425689	42.31070732	39.5115075	38.84942783
Gglean017084	44.425689	42.31070732	39.5115075	38.84942783
Gglean017085	13.27495519	9.938949141	7.748020002	7.392116
Gglean017086	36.85596	35.28449	28.17761	22.94574
Gglean017087	0.14002	0	0	0
Gglean017088	65.5466	58.5837	90.193	112.723
Gglean017089	0.321198	0.055595	0.346979	0.102616
Gglean017090	0.24556759	0.264918552	0.33180704	0.981182862
Gglean017091	0	0	0	0
Gglean017092	0	0	0	0
Gglean017093	0	0	0	0
Gglean017094	1.258	2.11703	0.526124	1.25147
Gglean017095	103.7684785	95.85528799	48.69141449	35.7194773
Gglean017096	103.7684785	95.85528799	48.69141449	35.7194773
Gglean017097	103.7684785	95.85528799	48.69141449	35.7194773
Gglean017098	0.103623	0	0	0
Gglean017099	4.628536	4.393441	5.264086	7.890414
Gglean017100	0	0	0.055125282	0
Gglean017101	0.548904	0.531794	0.753279	0.895584
Gglean017102	0.664382	0.167332	0.878762	0.0150549
Gglean017103	267.0598	269.5543	264.1095	255.8628
Gglean017104	23.94729001	25.17800804	9.784696215	7.624847
Gglean017105	0	0	0	0
Gglean017106	0	0	0	0
Gglean017107	0	0	0.25845	0
Gglean017108	0	0	0	0
Gglean017109	1.72196	1.09459	0.562293	1.35218
Gglean017110	0	0	0.0318681	0
Gglean017111	23.498554	23.11598	15.12421	12.81465
Gglean017112	0	0	0	0
Gglean017113	0	0	0	0
Gglean017114	3.70127	6.96223	8.44869	6.97832
Gglean017115	5.75031	8.35784	12.5138	14.2883

Gglean017116	10.4076	10.4685	16.7439	20.0072
Gglean017117	0.956977	1.46929	0.643877	1.60629
Gglean017118	0.605652	0.34713	0.601598	1.10305
Gglean017119	89.62937575	93.17818617	75.99685101	70.42017873
Gglean017120	89.62937575	93.17818617	75.99685101	70.42017873
Gglean017121	0	0	0	0
Gglean017122	0.804029001	1.252926	0.257095015	1.012233
Gglean017123	0.804029001	1.252926	0.257095015	1.012233
Gglean017124	0.804029001	1.252926	0.257095015	1.012233
Gglean017125	0	0	0	0
Gglean017126	0	0.059879	0.235507	0.279421
Gglean017127	2.788712244	1.58473796	2.989171548	4.764619045
Gglean017128	59.14989	61.02651	45.3533	45.88308
Gglean017129	59.14989	61.02651	45.3533	45.88308
Gglean017130	41.4042	42.3975	64.0377	76.0494
Gglean017131	10.5205	9.90332	8.84348	10.445
Gglean017132	0	0	0	0
Gglean017133	54.77496006	53.442926	61.13842846	63.53730995
Gglean017134	1.859685	1.736362241	1.899793	2.561064407
Gglean017135	4.547739117	3.725080775	3.0645714	4.356775061
Gglean017136	4.547739117	3.725080775	3.0645714	4.356775061
Gglean017137	4.547739117	3.725080775	3.0645714	4.356775061
Gglean017138	44.5043	48.4302	3.949626	3.92076
Gglean017139	44.5043	48.4302	3.949626	3.92076
Gglean017140	0	0	0	0.177935
Gglean017141	0	0	0	0.191055
Gglean017142	0	0.200031	0.312343	0.370981
Gglean017143	0	0.32574	0.250682	0
Gglean017144	1.16E-18	6.11E-06	0.557076	0.193961002
Gglean017145	1.16E-18	6.11E-06	0.557076	0.193961002
Gglean017146	0	0	0.0676251	0.160208
Gglean017147	2.59E-05	1.05651	2.97E-05	0.000187951
Gglean017148	0	0.102875	0.242288	0
Gglean017149	0.0361803	0	0.0293378	0
Gglean017150	22.70039	26.1107701	32.62424992	46.02654813
Gglean017151	6.723086569	12.42616624	9.9599057	16.45474412
Gglean017152	4.69009	3.62532	2.99459	2.8195
Gglean017153	0	0.429175	0	0.337451
Gglean017154	0	0.1707004	0	0.335949874
Gglean017155	2.274469764	3.049407586	2.783166246	3.531546315
Gglean017156	2.274469764	3.049407586	2.783166246	3.531546315
Gglean017157	13.87689	10.1695	6.39607	20.78057
Gglean017158	0.756292	0.911701	0.140821	0.669225186
Gglean017159	44.75970009	51.34871323	46.76430797	47.06999227

Gglean017160	44.75970009	51.34871323	46.76430797	47.06999227
Gglean017161	10.42111182	3.21904972	11.03712145	7.032241582
Gglean017162	0	0	0	1.5287
Gglean017163	5.94041	6.97697	7.28882	11.0397
Gglean017164	0	0	0	0
Gglean017165	6.20E-05	5.42E-06	0.117942	0.92538
Gglean017166	0.000627959	0.000837329	1.08069	8.53E-05
Gglean017167	116.0220622	119.8674303	53.3435154	51.52089843
Gglean017168	1.788862061	1.390779203	1.881292	1.194703778
Gglean017169	0.538965	0.285699	0.659193	0.394375
Gglean017170	0.187616884	0.267918108	0	0.276654068
Gglean017171	0.57347631	1.495463	1.180310562	0.704608576
Gglean017172	5.05118	7.36407	2.89841	2.32543
Gglean017173	19.45645	7.14293	10.64068053	20.73677
Gglean017174	19.45645	7.14293	10.64068053	20.73677
Gglean017175	0.839107881	1.4959973	1.167669224	1.566671369
Gglean017176	0.839107881	1.4959973	1.167669224	1.566671369
Gglean017177	3.62899004	4.583030326	3.453020064	3.27621007
Gglean017178	3.62899004	4.583030326	3.453020064	3.27621007
Gglean017179	10.5121	5.90927	0	0
Gglean017180	0	0.090451485	0	0.124871286
Gglean017181	0	0	0	0.067757
Gglean017182	17.45688	19.01596	22.75741	36.46739
Gglean017183	298.452	243.084	130.385	112.415
Gglean017184	50.5905	32.1604	33.7902	25.522
Gglean017185	50.5905	32.1604	33.7902	25.522
Gglean017186	21.5352	18.534	12.2059	12.5414
Gglean017187	4.853755	3.152223	0.764068	0.873647
Gglean017188	12.644562	16.98951	17.863219	15.589707
Gglean017189	12.644562	16.98951	17.863219	15.589707
Gglean017190	7.271622	6.112303895	2.830771	0.478097318
Gglean017191	0	0	0.972535	0.388859
Gglean017192	3.37E-37	0.555256	5.45E-07	9.77E-08
Gglean017193	74.7544	81.2384	112.9517	130.7315
Gglean017194	0.0789861	0.0824122	0	0
Gglean017195	0.138966	0	0.0562486	0.0671325
Gglean017196	0	0	0	0
Gglean017197	2.68688	3.96425	3.56329	4.52965
Gglean017198	0.135648	0	0.109777	0
Gglean017199	0	0	0	0
Gglean017200	0	0	0	0
Gglean017201	2.524376	2.65946	3.867473	3.532256
Gglean017202	0.300694086	0.67275086	1.135957	0.831061208
Gglean017203	0	0	0	0

Gglean017204	0	0	0	0
Gglean017205	0	0	0	0
Gglean017206	0	0	0	0
Gglean017207	0.225677576	0.362953918	0.439339777	0.2293875
Gglean017208	0.225677576	0.362953918	0.439339777	0.2293875
Gglean017209	0	0	0	0
Gglean017210	0	0.147402	0	0
Gglean017211	0	0	0	0
Gglean017212	0	0	0	0
Gglean017213	0.386704	0.416869	0.315435	0
Gglean017214	0	0	0	0
Gglean017215	0	0	0	0
Gglean017216	0.153252	0.209925	0.155252	0.077810183
Gglean017217	0	0	0	0
Gglean017218	0	0	0	0
Gglean017219	0	0	0	0.0636199
Gglean017220	0.153252	0.209925	0.155252	0.153865
Gglean017221	0	0	0	0
Gglean017222	0	0	0	0
Gglean017223	0.200415	0.522318	0.324936	1.06438
Gglean017224	195.7170224	200.443931	18.29142202	10.49918349
Gglean017225	195.7170224	200.443931	18.29142202	10.49918349
Gglean017226	0.197163	0.412203	0.161404	0.0952337
Gglean017227	0	0	0	0
Gglean017228	0	0	0	0
Gglean017229	1.35546	0.900555	0.394543	0.654987
Gglean017230	0	0	0	0.0702759
Gglean017231	40.777	37.7467	30.51387	27.69845
Gglean017232	12.665755	15.58456	11.77703775	12.365421
Gglean017233	12.665755	15.58456	11.77703775	12.365421
Gglean017234	10.33315181	11.70193732	14.40565315	13.39959243
Gglean017235	0	0	0	0
Gglean017236	0	0	0	0
Gglean017237	0	0.090451485	0	0.124871286
Gglean017238	0	0.090451485	0	0.124871286
Gglean017239	0	0	0	0
Gglean017240	178.08356	180.51705	23.878	8.67989
Gglean017241	25.93117385	29.40492	31.430664	34.45622
Gglean017242	0.204763	0.0707625	0.1653	0
Gglean017243	0.151488	0	0	0.0727166
Gglean017244	113.577505	117.2463287	106.31978	107.7939715
Gglean017245	113.577505	117.2463287	106.31978	107.7939715
Gglean017246	16.71015	17.77588	17.670228	18.69786272
Gglean017247	397.461506	383.092966	440.356675	533.189762

Gglean017248	68.05565127	75.2236389	89.99500803	78.88946494
Gglean017249	9.033580864	10.82081487	12.83515568	20.81581989
Gglean017250	0	0	0	0
Gglean017251	0	0	0	0
Gglean017252	8.82888818	6.939884643	9.902177163	8.70011891
Gglean017253	0	0	5.42576	0
Gglean017254	0	0.495338	0.897185	0
Gglean017255	0	0	0	0
Gglean017256	0	0	0	0
Gglean017257	2.383471	2.153479784	2.080707	2.347732133
Gglean017258	0.524402	1.73761	0.437658	2.09953
Gglean017259	11.9199579	13.21271768	15.27412145	14.98726699
Gglean017260	277.49541	280.75706	261.51173	307.82703
Gglean017261	22.89312	27.10953	24.67225	24.60906
Gglean017262	12.957711	12.23848524	10.580404	8.643543465
Gglean017263	16.27564	17.356691	18.15316	17.065975
Gglean017264	0.380047	0.386991	0.476055	0.767661
Gglean017265	0	0	0	0
Gglean017266	0	0	0	0
Gglean017267	8.723476	4.31104	9.25136	10.15732
Gglean017268	34.32442507	31.77582398	28.91550902	26.70282341
Gglean017269	0	0	0	0
Gglean017270	0	0	0	0
Gglean017271	26.31906593	24.45579986	20.338977	27.56348949
Gglean017272	20.621463	20.861897	31.569689	32.88789
Gglean017273	1760.343	1899.316	1205.164	1094.617
Gglean017274	0	0	0	7.30626
Gglean017275	43.025503	41.518712	41.089429	48.717596
Gglean017276	25.91987	26.30979	11.59717	12.325233
Gglean017277	25.91987	26.30979	11.59717	12.325233
Gglean017278	23.10085	24.36231	24.97481	22.87893
Gglean017279	19.6701	23.208	24.6927	28.1743
Gglean017280	3.0636789	3.800284603	9.25694	7.08543
Gglean017281	3.0636789	3.800284603	9.25694	7.08543
Gglean017282	56.02630536	58.68540595	83.11970665	96.48211254
Gglean017283	7.99736855	8.6639832	8.417090058	7.41689997
Gglean017284	0	0	0	0
Gglean017285	12.4923241	11.71826626	12.35301935	14.807968
Gglean017286	12.4923241	11.71826626	12.35301935	14.807968
Gglean017287	0	0	0	0
Gglean017288	34.9486	33.1688	8.09455	20.6428
Gglean017289	277.0323035	309.3316666	254.6314997	256.4718795
Gglean017290	7609.765205	6720.778078	8425.057643	8915.235903
Gglean017291	7609.765205	6720.778078	8425.057643	8915.235903

Gglean017292	7609.765205	6720.778078	8425.057643	8915.235903
Gglean017293	58.9976	42.711	2.9336	0.332741
Gglean017294	0	0	0.035137433	0.08348045
Gglean017295	0	0	0.035137433	0.08348045
Gglean017296	27.202462	26.09836	20.11457	19.55893
Gglean017297	161.3643395	160.4739898	86.05992636	81.7347906
Gglean017298	42.1072	44.9757	31.91067	33.44558
Gglean017299	4.51663	5.10203	5.34402	4.5564353
Gglean017300	0	0	0	0
Gglean017301	0	0	0	0
Gglean017302	0	0.0443916	0	0.041513
Gglean017303	33.82219	37.44747	41.6452654	51.90116
Gglean017304	33.82219	37.44747	41.6452654	51.90116
Gglean017305	33.82219	37.44747	41.6452654	51.90116
Gglean017306	0	0	0	0
Gglean017307	0	0	0	0
Gglean017308	0	0	0	0
Gglean017309	0	0	0	0
Gglean017310	490.55452	541.326	490.71638	410.7222
Gglean017311	18.56628	12.60897	23.3525	21.7332
Gglean017312	0.0580082	0.0589311	0.138802	0.108129
Gglean017313	0.0580082	0.0589311	0.138802	0.108129
Gglean017314	0	0	0	0
Gglean017315	0	0	0	0
Gglean017316	0	0	0	0
Gglean017317	0	0	0	0
Gglean017318	0	0	0	0
Gglean017319	0	0	0	0
Gglean017320	0	0	0	0
Gglean017321	0	0	0	0
Gglean017322	0	0	0	0
Gglean017323	0	0.162966	0	0
Gglean017324	0	0	0	0
Gglean017325	18.63451515	19.27371588	19.53639522	20.400903
Gglean017326	25.0658	29.0355	8.34392	8.70287
Gglean017327	0	0	0	0
Gglean017328	0	0	0.0399085	0.047229
Gglean017329	0	0	0	0
Gglean017330	0	0	0	0
Gglean017331	0	0	0	0
Gglean017332	0.0335171	0.104225	0.0542561	0.0640456
Gglean017333	0	0	0	0
Gglean017334	0	0	0	0
Gglean017335	15.21808	16.26427	16.98843	21.63705

Gglean017336	0	0	0.8681	0
Gglean017337	3.241227123	2.859382191	5.86612635	5.717691145
Gglean017338	1.29307	2.27517	1.54161	1.11127
Gglean017339	0	0	0	0
Gglean017340	0	0	0	0
Gglean017341	29.08143	37.79293	35.49939	36.53339
Gglean017342	0	0	0	0
Gglean017343	121.5405944	132.2115789	64.2998801	55.84059
Gglean017344	3.51273	2.24176	0.230642	2.41164
Gglean017345	24.477038	20.903311	17.333908	15.430207
Gglean017346	8.016335658	7.789928586	12.47105477	13.10418785
Gglean017347	0.651235277	0.625844627	1.202494	0.997025119
Gglean017348	0	0	0	0
Gglean017349	24.36115	20.294714	24.389104	20.21833
Gglean017350	24.36115	20.294714	24.389104	20.21833
Gglean017351	86.5072	85.0289	40.1812	33.0674
Gglean017352	31.614716	36.20464129	22.56959828	21.45659916
Gglean017353	31.614716	36.20464129	22.56959828	21.45659916
Gglean017354	31.614716	36.20464129	22.56959828	21.45659916
Gglean017355	31.614716	36.20464129	22.56959828	21.45659916
Gglean017356	34.70358	33.46274	50.33643	55.18634
Gglean017357	21.076	25.0125	15.4564	12.7828
Gglean017358	0	0	0	0
Gglean017359	38.11395608	36.04431514	61.1011364	77.668672
Gglean017360	195.326	149.497	141.241	113.122
Gglean017361	48.56945	57.59654	14.82486	12.77481
Gglean017362	6.12343	16.62469	0.992052	0.2972776
Gglean017363	74.4535	77.37324	58.1231	52.9349
Gglean017364	3.95949974	3.42970073	4.587193103	4.656855537
Gglean017365	10.20022443	8.784539385	12.4068687	14.17115064
Gglean017366	10.20022443	8.784539385	12.4068687	14.17115064
Gglean017367	4.484511561	3.931090693	3.888191351	3.561307536
Gglean017368	0.0940925	0.153655008	0.159351	0.122048
Gglean017369	20.98503001	19.2001	6.094079	5.558017627
Gglean017370	20.98503001	19.2001	6.094079	5.558017627
Gglean017371	228.93957	226.9741	130.712973	118.90692
Gglean017372	67.8904	68.676404	59.39798	57.83538
Gglean017373	21.52761427	22.62242062	25.29408722	21.76241827
Gglean017374	120.801	118.816	114.088	118.95
Gglean017375	5.83448286	5.565205868	8.307768296	9.69563961
Gglean017376	3.581106173	2.100587609	4.306056	5.331138
Gglean017377	10.7373	8.12777	17.0338	14.3769
Gglean017378	1.416733	1.56815	3.03996	2.000890536
Gglean017379	0	1.72894	0	0

Gglean017380	0	0	0	0
Gglean017381	0	0	1.60245	0.961808
Gglean017382	0.904177	3.17046	4.969831921	4.52409
Gglean017383	0.904177	3.17046	4.969831921	4.52409
Gglean017384	0.790767959	0.731858966	0.987029	0.927287437
Gglean017385	46.50367	52.34027	42.94082	42.42175
Gglean017386	10.1113	9.67811	12.03123	13.71037
Gglean017387	17.50284	19.23847	15.16728	14.86244
Gglean017388	48.98276029	99.20671951	93.728092	132.1641215
Gglean017389	48.98276029	99.20671951	93.728092	132.1641215
Gglean017390	0	0	0	0
Gglean017391	11.99745703	13.440922	3.747993415	3.714512
Gglean017392	11.99745703	13.440922	3.747993415	3.714512
Gglean017393	316.7342553	354.2544299	150.293	133.8642194
Gglean017394	13.50002	12.28694144	17.60988891	19.24915568
Gglean017395	104.7593315	117.761174	29.76596917	24.97786541
Gglean017396	104.7593315	117.761174	29.76596917	24.97786541
Gglean017397	104.7593315	117.761174	29.76596917	24.97786541
Gglean017398	2.34153	2.72728	2.59087	2.97322
Gglean017399	1317.604	1350.58	857.532	821.137
Gglean017400	202.2329	165.0387	202.147	182.1473
Gglean017401	23.1961797	30.397825	25.3634954	32.9411314
Gglean017402	23.1961797	30.397825	25.3634954	32.9411314
Gglean017403	0	0	0	0
Gglean017404	0.565677	1.05011	1.13366	0.943309
Gglean017405	14.041285	18.87216	18.54006	23.20401
Gglean017406	2.70521	3.01487	3.55142	3.48817
Gglean017407	0.887283269	2.964382	1.225402698	0.144402287
Gglean017408	0.887283269	2.964382	1.225402698	0.144402287
Gglean017409	24.45006	38.27523	34.45091	49.01092
Gglean017410	24.45006	38.27523	34.45091	49.01092
Gglean017411	0.424127	0.72924	0.459261	0.849887
Gglean017412	0	0	0	0
Gglean017413	0.280444	9.40E-05	0.00655421	0.0526202
Gglean017414	0.280444	9.40E-05	0.00655421	0.0526202
Gglean017415	1.973085001	2.304351328	2.84749233	4.816442
Gglean017416	0	0	0	0
Gglean017417	0	0	0	0
Gglean017418	22.7637	20.107	8.74325	9.42416
Gglean017419	0.156206	0.484236	0.631996	1.194
Gglean017420	43.9537	38.26624	48.9662	70.7641
Gglean017421	23.8481	0.00927958	13.0262	20.1169
Gglean017422	4.38444	3.916005	2.36226971	2.484509
Gglean017423	4.38444	3.916005	2.36226971	2.484509

Gglean017424	9.382805	9.738898759	6.582763	5.72573
Gglean017425	43.58635	48.25630001	21.179375	23.102949
Gglean017426	118.2236	126.625	134.1049	123.8514
Gglean017427	3.37697	0.000624907	3.84E-05	0.000515709
Gglean017428	6.01743	5.41866	4.7058	5.99684
Gglean017429	0	0	0	0
Gglean017430	23.93061	24.71282	23.248974	22.6049
Gglean017431	23.93061	24.71282	23.248974	22.6049
Gglean017432	1.1639	0.655052	0	0.0568228
Gglean017433	9.616419393	10.512205	6.287994	5.631985
Gglean017434	87.40763	113.3026	68.04255	45.99509
Gglean017435	87.40763	113.3026	68.04255	45.99509
Gglean017436	18.206871	18.218806	22.192449	21.576068
Gglean017437	5.94905	6.79332	9.31285	10.9753
Gglean017438	0	0	0	0
Gglean017439	0	0	0	0
Gglean017440	95.16138644	108.4891041	77.08627323	72.6377633
Gglean017441	2.68512	11.819	6.66462	4.16495
Gglean017442	1.64236	0.0110957	0.000152784	0.596618
Gglean017443	19.22406515	22.13091124	32.916785	40.08093387
Gglean017444	0	0	0	0
Gglean017445	0.631880759	0.812412997	1.1332035	1.816611377
Gglean017446	53.65719264	57.44180156	44.06237562	41.16417142
Gglean017447	53.65719264	57.44180156	44.06237562	41.16417142
Gglean017448	62.24392	46.34456	29.898101	27.249279
Gglean017449	62.24392	46.34456	29.898101	27.249279
Gglean017450	62.24392	46.34456	29.898101	27.249279
Gglean017451	2.50101	7.08212	1.03564	2.4806
Gglean017452	0.461195379	0.208052096	0.460608345	0.600192536
Gglean017453	0.461195379	0.208052096	0.460608345	0.600192536
Gglean017454	19.49231	18.69021	16.11596	16.50176
Gglean017455	5.5190451	11.35143	9.37248	5.229161733
Gglean017456	0.0612263	0.317931	0.0998045	0.0590119
Gglean017457	0	0	0	0
Gglean017458	26.0895	20.9338	21.2818	24.2249
Gglean017459	16.93940496	17.91475567	36.55709713	34.97549223
Gglean017460	1.85633	0.815804	0.617701	0
Gglean017461	0	0	0.0467297	0
Gglean017462	49.89019	61.04151018	45.174452	36.609477
Gglean017463	2.95453	3.10616	5.3452	4.6783
Gglean017464	28.4395	46.7202	66.5867	69.4365
Gglean017465	0	0	0	0
Gglean017466	14.2137	18.5215	25.5639	31.4796
Gglean017467	49.5425	55.0073	57.9463	57.929

Gglean017468	37.9583	35.8018	27.2253	25.9907
Gglean017469	35.19807714	36.54799296	28.14630196	24.67526823
Gglean017470	12.292541	13.851589	22.48109	24.72066
Gglean017471	0	0	0	0
Gglean017472	206.9007628	242.2186092	278.8631658	349.0376835
Gglean017473	206.9007628	242.2186092	278.8631658	349.0376835
Gglean017474	206.9007628	242.2186092	278.8631658	349.0376835
Gglean017475	0	0	0	0
Gglean017476	38.704974	39.754386	25.078571	20.68465
Gglean017477	1.72883	1.45262	2.93336	2.36532
Gglean017478	8.90841	14.34352	12.54846	15.38709
Gglean017479	63.7962	71.0982	53.4466	49.7385
Gglean017480	59.90275426	61.81500558	40.67094556	35.81529816
Gglean017481	51.06075	59.57747	54.27409	56.48253
Gglean017482	23.078464	22.41135945	14.47838173	12.35531999
Gglean017483	23.078464	22.41135945	14.47838173	12.35531999
Gglean017484	4.59545	4.39616	1.11481	0.995829
Gglean017485	0	0	0	0
Gglean017486	111.90109	111.5640689	66.893597	60.45505357
Gglean017487	0	0	0	0
Gglean017488	8.180453884	6.189161	12.28352	15.095791
Gglean017489	31.94660244	29.594386	40.386378	54.041983
Gglean017490	0	0	0.21527	0.513806
Gglean017491	0	0	0	0
Gglean017492	0.231091	6.78E-05	0	0
Gglean017493	35.6538	30.44092	33.65557	53.57421
Gglean017494	19.181191	21.076521	21.040636	22.708352
Gglean017495	7.604072955	10.46245438	10.51493488	9.518207215
Gglean017496	7.604072955	10.46245438	10.51493488	9.518207215
Gglean017497	7.604072955	10.46245438	10.51493488	9.518207215
Gglean017498	4.37E-07	0.337478	0.218101	1.33E-05
Gglean017499	0	0	0	0
Gglean017500	0	0	0	0
Gglean017501	0.518364	0.248019	0.741279	0.914629
Gglean017502	29.463347	36.11251951	44.032436	38.59702004
Gglean017503	2.52722	3.21099	4.21465	2.47251
Gglean017504	39.99231722	43.18824195	49.67594274	48.12752945
Gglean017505	85.29082988	86.64298182	105.9984981	99.76333386
Gglean017506	83.01618646	93.00498636	99.88920204	127.8022746
Gglean017507	81.98414	68.65753	152.7081	132.99863
Gglean017508	43.70666	38.8466	37.97205	34.63415
Gglean017509	14.94311906	14.91026332	11.09350023	9.701770179
Gglean017510	54.28397982	53.74209124	84.9486118	95.71154612
Gglean017511	51.18341924	55.45404566	46.25326318	43.04940203

Gglean017512	267.461819	274.300328	289.079427	267.645534
Gglean017513	16.416142	15.555056	23.1391	23.36824
Gglean017514	97.1089027	96.48376464	92.39803653	73.82312048
Mite-foot_newGene_1	0.201793776	0	0	0.06485222
Mite-foot_newGene_10	15.4815	16.6951	2.47482	2.95749
Mite-foot_newGene_1000	1.981951	2.142625	3.58594	2.77742
Mite-foot_newGene_1002	0	0	0	0
Mite-foot_newGene_1003	0.630582	0.578354	1.25662	0.649345
Mite-foot_newGene_1004	0.190129	0.0869934	0.214537	0.218134
Mite-foot_newGene_1005	0	0	0	0
Mite-foot_newGene_1006	58.7464	55.56467	38.606048	34.78271
Mite-foot_newGene_1007	25.61389646	20.83241711	31.475662	35.7087021
Mite-foot_newGene_1008	0	0	0	0
Mite-foot_newGene_1010	0.0837828	0.172238	0.203462	0.0803556
Mite-foot_newGene_1011	23.42305353	23.33109146	34.47124878	39.21538751
Mite-foot_newGene_1012	0.0662474	0	0.106985	0.0315308
Mite-foot_newGene_1013	2.09347	2.2974	1.83959	3.05853
Mite-foot_newGene_1015	19.99027	17.297958	9.2369774	11.165983
Mite-foot_newGene_1016	26.4159	19.7498	14.6612	11.7861
Mite-foot_newGene_1061	0	0	0	0
Mite-foot_newGene_1062	0	0	0	0
Mite-foot_newGene_1063	0.2472091	0.1856935	0.396737	0.219311
Mite-foot_newGene_1064	0	0	0.224771	0.26807
Mite-foot_newGene_1065	12.22441016	10.7744873	7.490949345	5.228884524
Mite-foot_newGene_1067	1.45824	0.827798	1.37537	1.9444
Mite-foot_newGene_1068	0.0511881	0.155615	0.348066	0.0729046
Mite-foot_newGene_1069	6.13974	4.82927	3.65712	2.90984
Mite-foot_newGene_1070	7.181424	5.85088	3.275545	3.411649
Mite-foot_newGene_1071	1.85E-47	1.72E-30	4.49E-62	5.18E-62
Mite-foot_newGene_1088	1.948418	1.300147	1.904635	0.363817
Mite-foot_newGene_1089	0.159728	0.109536	0.128616	0
Mite-foot_newGene_1090	1.47938	0.733643	1.20511	1.08821
Mite-foot_newGene_1094	1.1524676	0.71668969	0.739523728	0.482762
Mite-foot_newGene_1095	0.99925	1.19133	1.11768	0.62582
Mite-foot_newGene_1098	0	0	0	0
Mite-foot_newGene_1099	3.108021	2.33043	0.627166995	1.13388451
Mite-foot_newGene_11	174.5261003	168.24687	201.3758	212.67603
Mite-foot_newGene_1100	0.920188	0.609408	0.778409	0.973238
Mite-foot_newGene_1101	38.20906	39.3263	65.55889	67.75798
Mite-foot_newGene_1102	6.467229129	7.167519142	5.332989	4.406595783
Mite-foot_newGene_1103	0.7757141	0	1.271816728	1.52018644
Mite-foot_newGene_1104	0.245262	0.0687527	0.0792241	0.302271
Mite-foot_newGene_1105	2.310899	1.784616	2.021392	2.006453
Mite-foot_newGene_1106	0.0375648	1.28E-08	0.0275794	0.0376087

Mite-foot_newGene_1107	16.951851	12.94604	10.47886	9.133508
Mite-foot_newGene_1108	81.204857	82.020501	72.298614	77.177686
Mite-foot_newGene_1109	0.103489	0.107079	0.0839854	0.199124
Mite-foot_newGene_1110	0.500963	0.49613	0.662937	0.556796
Mite-foot_newGene_1111	45.3371	46.9503	38.8276	34.7321
Mite-foot_newGene_1112	10.9044	11.6839	13.734	17.8811
Mite-foot_newGene_1113	11.6368	16.3005	20.7771	25.008
Mite-foot_newGene_1114	13.8975	11.5722	9.41668	18.7336
Mite-foot_newGene_1115	6.30351	5.9318	9.46165	9.00487
Mite-foot_newGene_1116	0.074315	0.154742	0	0
Mite-foot_newGene_1117	3.852581	3.822603	4.38115	7.303608
Mite-foot_newGene_1118	0	0	0	0
Mite-foot_newGene_1119	3.8154	2.34604	2.85708	2.21647
Mite-foot_newGene_1132	1.15309	0.835307	0.522222	0.289801
Mite-foot_newGene_1140	0.999017	1.43741	0.820587	2.62005
Mite-foot_newGene_1141	0	0.106083	0.0832901	0.0985901
Mite-foot_newGene_1142	0	0	0.101290749	0.0372323
Mite-foot_newGene_1143	0	0	0	0
Mite-foot_newGene_1144	0.112466098	0	0	0
Mite-foot_newGene_1145	274.096	270.708	356.482	407.911
Mite-foot_newGene_1146	7.160729	8.786603	7.30968	9.519424
Mite-foot_newGene_1148	0.80499	1.62607	1.91979	4.83698
Mite-foot_newGene_1149	3.01871	3.32242	1.67465	1.19452
Mite-foot_newGene_1150	29.60687598	31.95281303	43.04402816	46.21790897
Mite-foot_newGene_1151	23.3645	24.4444	22.47863	20.40924
Mite-foot_newGene_1152	0	0	0	0
Mite-foot_newGene_1180	6.90729992	6.011039714	9.522302823	10.2395042
Mite-foot_newGene_1189	0.695881	0.730377	0.402662	0.284252
Mite-foot_newGene_1190	0	0	0	0
Mite-foot_newGene_1191	0.545726	1.03282	0.896259	1.063
Mite-foot_newGene_1192	4.48267	3.67994	7.13107	9.23824
Mite-foot_newGene_1193	67.1884	70.7205	75.3609	77.9453
Mite-foot_newGene_1194	34.21482	27.4512	25.55720104	19.6563024
Mite-foot_newGene_1195	0	0.897147	0.651268	0
Mite-foot_newGene_1196	7.41925	8.55657	8.64322	8.97475
Mite-foot_newGene_1198	1.412197	1.725343	1.190534	1.59837
Mite-foot_newGene_1199	0.133669	0	0	0.0637656
Mite-foot_newGene_12	0	0	0.113712	0
Mite-foot_newGene_1200	0.0215696	0.0222665	0	0.0206898
Mite-foot_newGene_1201	1.977428	1.53595	1.110861	2.367266
Mite-foot_newGene_1218	7.84381	6.40877	5.1091	4.29021
Mite-foot_newGene_1220	0	0	0	0
Mite-foot_newGene_1221	6.60673	4.42296	6.84721	10.1265
Mite-foot_newGene_1222	20.3657	18.0842	11.5726	11.9574

Mite-foot_newGene_1223	0	0	0	0
Mite-foot_newGene_1224	11.31627137	10.88709172	22.4791074	30.415519
Mite-foot_newGene_1225	13.4332	14.4209	15.8252	19.4213
Mite-foot_newGene_1226	2.89798	4.77417	2.02366	1.45456
Mite-foot_newGene_1243	0.367068	0	0.0421711	0.0997186
Mite-foot_newGene_1244	0.993097	0.312368	0.403926	0.0951889
Mite-foot_newGene_1245	2.35399	1.03131	1.10902	0.595444
Mite-foot_newGene_1246	6.75851	7.164782	4.220116	4.76666
Mite-foot_newGene_1247	1.46092	1.8729	1.13483	0.90459
Mite-foot_newGene_1249	0	0	0	0
Mite-foot_newGene_1250	8.407095	7.178014	7.64408	6.565065
Mite-foot_newGene_1251	1.2233	1.32551	3.22E-06	3.79E-06
Mite-foot_newGene_1252	0.0826155	0	0.0334456	0
Mite-foot_newGene_1254	0	0	0.380991	0
Mite-foot_newGene_1273	0.435742	0.310316	0.668873	0.386352
Mite-foot_newGene_1276	0	0.0988753	0	0.0910058
Mite-foot_newGene_1281	4.13158	3.31512	2.37737	2.32374
Mite-foot_newGene_1282	98.643	90.0851	143.985	265.167
Mite-foot_newGene_1283	0.987904	0.781955	1.44023	2.20411
Mite-foot_newGene_1284	17.32530645	14.9094176	26.905374	24.235699
Mite-foot_newGene_1285	2.8166	1.96875	2.99501	2.80968
Mite-foot_newGene_1302	0	0.1274051	0	0.16643572
Mite-foot_newGene_1303	0.0458407	0.192631	0.0376764	0
Mite-foot_newGene_1304	0	0.0648207	0.100933	0
Mite-foot_newGene_1305	17.2625	16.0486	14.5347	16.1064
Mite-foot_newGene_1306	0	0	0	0
Mite-foot_newGene_1307	24.45182057	19.09081922	18.898668	19.95056638
Mite-foot_newGene_1308	0.399336	0.415819	0.108358	0.153526
Mite-foot_newGene_1309	34.86794	34.92962	53.52306	60.518
Mite-foot_newGene_1310	3.51558	2.39717	2.49831	5.02819
Mite-foot_newGene_1311	1.76237	0.947265	2.48007	2.08115
Mite-foot_newGene_1321	4.056719588	3.225901471	2.709841672	2.587082521
Mite-foot_newGene_1323	1.61838	1.29374	0.448782	0.57624
Mite-foot_newGene_1324	0.659115	0.815472	0.721968	0.284116
Mite-foot_newGene_1325	3.40192	3.57945	5.27371	3.12641
Mite-foot_newGene_1326	7.75289076	12.195456	7.192693166	5.943579157
Mite-foot_newGene_1327	0.564121	0.524583	0.756824	1.52877
Mite-foot_newGene_1328	2.18938406	2.541562	2.708385	2.07065704
Mite-foot_newGene_1329	0.0555324	0.172114	0.136251	0
Mite-foot_newGene_1330	0.236838	0.820851	0.637147	0.75383
Mite-foot_newGene_1331	35.3109	43.76029945	19.4447185	17.2367934
Mite-foot_newGene_1332	0	0	0	0
Mite-foot_newGene_1333	0	0	0	0
Mite-foot_newGene_1334	0	0	0	0

Mite-foot_newGene_1335	0.932484	0.873476	0.163087	0.120002
Mite-foot_newGene_1336	0.582216	1.10407	0.550903	0.839448
Mite-foot_newGene_1337	2.15786	1.40682	1.20937	0.524355
Mite-foot_newGene_1338	7.38219	6.71478	5.84049	5.410337
Mite-foot_newGene_1339	0.282475	0.0980667	0.154669	0.182412
Mite-foot_newGene_1345	8.68994	6.72471	10.14863	6.67877
Mite-foot_newGene_1346	1.64153	1.46872	2.14663	1.78059
Mite-foot_newGene_1347	0	0	0.174489546	0.415973721
Mite-foot_newGene_1350	29.004663	28.849668	17.019413	14.30907
Mite-foot_newGene_1351	0.984534	1.411888	1.554856	1.256894
Mite-foot_newGene_1352	0.23298	0.126451	0.248098	0.339712
Mite-foot_newGene_1353	0.228	0.332733	0.223816	0.0877323
Mite-foot_newGene_1354	2.464207891	2.440353453	2.487665	3.375491
Mite-foot_newGene_1355	2.432819	2.862416	1.361216	0.843342
Mite-foot_newGene_1356	0.637708	0.34671	0.64736	0.577402
Mite-foot_newGene_1357	0.643977	0.816657	0.3776652	0.503391
Mite-foot_newGene_1358	1.76385	2.02368	2.35227	0.713451
Mite-foot_newGene_1359	1.0714567	1.437436	1.074638457	0.799614878
Mite-foot_newGene_1360	1.10608	1.08752	1.07537	0.371359
Mite-foot_newGene_1361	2.15964	1.82374	2.17328	1.38807
Mite-foot_newGene_1362	0	0.086005002	0	0
Mite-foot_newGene_1363	0	0.085402	0	0.0796484
Mite-foot_newGene_1364	32.9983712	34.319068	46.52307	53.00339091
Mite-foot_newGene_1365	1.92528975	1.662528297	2.179176303	2.01294624
Mite-foot_newGene_1366	5.54648	3.94094	2.332608	2.418611
Mite-foot_newGene_1367	0.626053	0.747939	0.479451096	0.3944734
Mite-foot_newGene_1368	49.399296	50.051039	51.39300908	47.3226527
Mite-foot_newGene_1369	32.9945	30.891	30.0396	27.6411
Mite-foot_newGene_137	38.0407	38.0949	56.7355	69.3357
Mite-foot_newGene_1370	85.9022	87.7405	81.305	74.9811
Mite-foot_newGene_1371	3.81035	4.97329	8.41605	9.65957
Mite-foot_newGene_138	0	0	0	0
Mite-foot_newGene_139	0.353701	0.731627	0.649385	0.673652
Mite-foot_newGene_14	0.422219	0.107694	0.216254	0.361251
Mite-foot_newGene_140	4.006897	3.88934	0.781437	0.375932
Mite-foot_newGene_1406	2.7005	2.51238	0.726945	0.943485
Mite-foot_newGene_1408	3.33177	3.22636	4.050696273	5.131708927
Mite-foot_newGene_1409	1.88785	1.83555	3.66055	2.01992
Mite-foot_newGene_141	0.2489649	0	0	0
Mite-foot_newGene_1410	21.20172367	22.790208	18.69048421	21.63138068
Mite-foot_newGene_1411	9.214016	9.05707	3.771967	2.769696
Mite-foot_newGene_1412	303.95836	318.21199	227.24449	198.10024
Mite-foot_newGene_1413	0.21797	0.399663	0.313213	0.231214
Mite-foot_newGene_1414	0	0	0	0.185345

Mite-foot_newGene_1415	5.69908	5.46381	7.48958	6.81625
Mite-foot_newGene_1416	0	0	0	0
Mite-foot_newGene_1417	97.5462	90.6061	70.0592	68.1658
Mite-foot_newGene_1418	13.8018	6.1125899	16.7592442	17.2487259
Mite-foot_newGene_1419	0	0	0	0
Mite-foot_newGene_142	0.510299	0.538595	0.32662	0.169427
Mite-foot_newGene_1420	2.65309	2.78885	2.92207	3.97588
Mite-foot_newGene_1421	0.146891	0.15326	0.239929	0.286717
Mite-foot_newGene_1422	18.6731	18.2624	21.6027	31.1901
Mite-foot_newGene_1423	0	0	0.0423548	0
Mite-foot_newGene_1424	15.7854	16.4232	24.8056	26.6633
Mite-foot_newGene_1425	1.586185559	1.627326102	2.326770517	3.162203627
Mite-foot_newGene_1426	0	0	0.309672	0.146446
Mite-foot_newGene_1427	0	0	0	0
Mite-foot_newGene_1428	34.61512	31.09343416	40.87424	39.83242
Mite-foot_newGene_1429	155.4194	173.7158	169.2264	192.2792
Mite-foot_newGene_143	2.0226	1.79679	1.10433	1.25453
Mite-foot_newGene_1430	0	0.0908484	0.0710089	0.25273
Mite-foot_newGene_1431	43.5598	45.7961	55.9023	57.7654
Mite-foot_newGene_1432	2.94688	2.47025	4.80025	5.65361
Mite-foot_newGene_1433	4.58776	4.06684	4.13074	3.43661
Mite-foot_newGene_1434	0	0	0	0
Mite-foot_newGene_1435	6.55797	8.64696	17.9101	11.0527
Mite-foot_newGene_1436	0	0	0	0
Mite-foot_newGene_1437	35.19039	32.86066	48.65337	59.20638
Mite-foot_newGene_1438	0	0	0	0
Mite-foot_newGene_1439	0	0.075495	0.0295088	0.0348696
Mite-foot_newGene_144	0.151905	0.0939944	0.564053	0.274927
Mite-foot_newGene_1440	0.209826	0	0.569819	0.201365
Mite-foot_newGene_1442	0.0641319	0.0673319	0.0526862	0
Mite-foot_newGene_1444	0.121524	0	0	0
Mite-foot_newGene_1445	1.73673	0.768395	0.601279	0.59333
Mite-foot_newGene_1446	13.92506	13.17923965	8.392170672	9.790093309
Mite-foot_newGene_1447	0	0	0	0
Mite-foot_newGene_1448	0.0338909	0.105202	0	0
Mite-foot_newGene_1449	0	0	0	0.107127
Mite-foot_newGene_145	0	0	0	0
Mite-foot_newGene_1450	0.2534769	2.58E-164	5.70E-130	7.40E-150
Mite-foot_newGene_1451	959.36645	893.11644	1262.01768	1218.57851
Mite-foot_newGene_146	728.337	724.252	664.746	1159.319
Mite-foot_newGene_147	1.58958	2.62441	0.641095	0.931285
Mite-foot_newGene_148	2.490945	0.286547	3.01881	3.75784
Mite-foot_newGene_1486	185.192	184.685	166.823	199.466
Mite-foot_newGene_1487	0.244359	0	0.345614	0.233631

Mite-foot_newGene_1488	2.619041288	2.61006294	2.584683025	2.874398165
Mite-foot_newGene_1489	5.97657	5.71096	10.5223	11.454882
Mite-foot_newGene_149	0.169428	0	0.13841	0
Mite-foot_newGene_1490	1.38258	1.05918	0.455471	0.685455
Mite-foot_newGene_1491	0.899	0.745895241	1.033393262	0.840217562
Mite-foot_newGene_1492	50.8331	47.45398	56.62863	56.08512
Mite-foot_newGene_1493	0.051905	0.0385733	1.50E-10	6.01E-18
Mite-foot_newGene_1494	0	0.164454	0.258092	0.152141
Mite-foot_newGene_1495	0.0546139	0.113918	0.44397	0.315554
Mite-foot_newGene_15	0.672035	0.276239	0.863902	1.41402
Mite-foot_newGene_150	1.11E-10	0.00563893	1.45E-09	5.63E-11
Mite-foot_newGene_1508	0.366583	0	0.420703	0.716186
Mite-foot_newGene_1509	1.400958	0.764077	0.691597662	1.101024
Mite-foot_newGene_151	0.70488	0.20884	0.489817	0.0966964
Mite-foot_newGene_1510	102.654	117.241	105.476	136.101
Mite-foot_newGene_1511	0.0781431	0	0	0
Mite-foot_newGene_1512	0.118816085	0.121117205	0	0
Mite-foot_newGene_1513	0.125012	0.0655054	0	0
Mite-foot_newGene_1514	12.0368089	12.218351	18.420385	22.43107
Mite-foot_newGene_1515	0	0.274858	0.211398	0.885417
Mite-foot_newGene_1516	0	0	0	0
Mite-foot_newGene_1517	52.87417712	53.62028329	54.67812352	49.55469591
Mite-foot_newGene_1518	0	0	0	0
Mite-foot_newGene_152	1.9794	1.96355	0.734422	1.55559
Mite-foot_newGene_1528	1.17	3.20907	4.6484	3.93449
Mite-foot_newGene_1529	5.93271145	5.673597468	3.595039	1.6993322
Mite-foot_newGene_153	0.0583145	0	0	0.05565564
Mite-foot_newGene_1530	1.35678	1.60745	3.11211	3.00149
Mite-foot_newGene_1532	0	0	0	0
Mite-foot_newGene_1533	45.8276	46.6156	31.18198	28.5895
Mite-foot_newGene_1534	0.079892922	0.181863136	0.320279468	0.210162875
Mite-foot_newGene_1535	16.207	32.54164	52.7028	46.8945
Mite-foot_newGene_1536	0.104824	0	0.169162	0.0994563
Mite-foot_newGene_1537	71.6102194	66.41639021	71.83796797	67.5345877
Mite-foot_newGene_1538	0.238454492	0	0.19375933	0.426404
Mite-foot_newGene_1539	0	0	0	0.177105
Mite-foot_newGene_154	49.5921	51.5599	84.9627	89.7453
Mite-foot_newGene_1540	12.7717	12.4486	12.3829	13.3432
Mite-foot_newGene_1541	16.3534	14.5743	18.857	21.0578
Mite-foot_newGene_155	0	0.13802	0.108248	0.191332
Mite-foot_newGene_1555	0	0	0	0
Mite-foot_newGene_1556	1.553569	1.003908	2.047191	2.52309
Mite-foot_newGene_1557	1.28173	1.586938	0.303072	0.260924
Mite-foot_newGene_1558	3.78408	3.74248	2.62686	2.33268

Mite-foot_newGene_1559	0.584376	0.437011	0.67656	0.319126
Mite-foot_newGene_156	35.02887041	34.439323	53.59251584	63.25079896
Mite-foot_newGene_1560	0.116284	0	0	0
Mite-foot_newGene_1561	1.03825	0.714207	0.296593	0.17618
Mite-foot_newGene_1562	0.525176	0.952025	0.21344	0.379598
Mite-foot_newGene_1563	14.99973	19.187965	22.79988	30.40235
Mite-foot_newGene_1564	0	0.0695164	0	0.032529
Mite-foot_newGene_1565	0.57547	1.4221	0.467067	0.221789
Mite-foot_newGene_1566	0	0.139917	0	0.12876
Mite-foot_newGene_1567	3.494961503	3.32433796	4.533505487	3.543994
Mite-foot_newGene_1568	5.18235	6.97254	11.14382	12.20265
Mite-foot_newGene_157	0.242942	0.099967	0.511279	0.186459
Mite-foot_newGene_158	0.423366	0.107972	0.288229	0.56927
Mite-foot_newGene_1586	0	0	0	0
Mite-foot_newGene_1587	9.99259	9.02198	13.50258	15.81499
Mite-foot_newGene_1588	0	0	0.0478277	0
Mite-foot_newGene_1589	0.0371687	0	0	0.035130056
Mite-foot_newGene_159	37.04965	36.97035	57.29915601	63.84626246
Mite-foot_newGene_1590	0.158794	0.262602	0.0774484	0.180808
Mite-foot_newGene_1591	0	0	0	0
Mite-foot_newGene_1592	1.39126	0	1.52615	0.916015
Mite-foot_newGene_1593	0	0	0	0
Mite-foot_newGene_1594	4.1290101	2.6374902	3.818676079	3.97395
Mite-foot_newGene_16	2.00746	0.910123	2.16087	1.90377
Mite-foot_newGene_160	0.282043	0.300719	1.27333	0.554919
Mite-foot_newGene_161	0	0.266879	0	3.96E-78
Mite-foot_newGene_1616	4.28312	4.29235	6.3627	6.92345
Mite-foot_newGene_162	55.474584	59.47640796	32.2815194	28.538577
Mite-foot_newGene_1620	0.0997934	0.692956	0.4457	0.183271
Mite-foot_newGene_1621	6.09745	5.45266	0.877659	0.615102
Mite-foot_newGene_1622	54.4512	49.9876	111.537	122.901
Mite-foot_newGene_1623	8.61989	10.8568	4.20226	3.44477
Mite-foot_newGene_1624	0.609231	1.42351	0.000244108	0.30347
Mite-foot_newGene_1626	2.1451125	0.493049	4.63632174	4.22779144
Mite-foot_newGene_1627	0.679923718	0.937605	0.236411494	0.2256296
Mite-foot_newGene_1628	2.86367	2.51738	2.86079	2.63114
Mite-foot_newGene_1629	178.449362	201.3124692	264.128989	248.409481
Mite-foot_newGene_163	10.8512	5.19083	6.23863	9.18899
Mite-foot_newGene_1630	0	0	0	0
Mite-foot_newGene_1631	1.46202	3.4023	0.277969	0.174155
Mite-foot_newGene_1632	0	0	0	0
Mite-foot_newGene_1633	0	0	0	0
Mite-foot_newGene_1634	4.80475	4.85055	1.94247	2.42203
Mite-foot_newGene_1635	0	0	0	0

Mite-foot_newGene_1636	45.275521	46.50642049	62.1062168	46.8677
Mite-foot_newGene_1637	6.29437	6.1637	8.03735	7.69491
Mite-foot_newGene_1638	12.08922663	13.50707645	13.08842998	13.21253889
Mite-foot_newGene_164	1.57645	6.74156	4.7363	6.07731
Mite-foot_newGene_165	4.94765911	7.045922152	1.2787592	1.254531722
Mite-foot_newGene_1658	205.609	233.019	182.953	234.842
Mite-foot_newGene_166	9.81918	11.1436	10.4228	9.84758
Mite-foot_newGene_1660	0.695207	0.830608	1.01256	1.589943
Mite-foot_newGene_1661	2.59244	4.38553	2.74695	3.09306
Mite-foot_newGene_1662	14.64863	17.26759	21.66729	24.27277
Mite-foot_newGene_1663	2.11004	2.12309	1.78581	2.00869
Mite-foot_newGene_1664	0	0	0.0547841	0
Mite-foot_newGene_1665	2.72974	0.711349	0.332174	0.131403
Mite-foot_newGene_1666	0.237302	0.277228	0.333265	0.423585
Mite-foot_newGene_1667	0.827296	0.7913	1.24087	1.17899
Mite-foot_newGene_167	48.3876	45.6644	44.4661	45.3869
Mite-foot_newGene_1674	0.719173	1.25137	0.349157	0.278695
Mite-foot_newGene_1675	0	0	0	0
Mite-foot_newGene_1676	9.929417	4.622128	11.517641	15.839331
Mite-foot_newGene_1677	0	0	0	0
Mite-foot_newGene_1678	1.08993	0.463442	0.288909	0.226976
Mite-foot_newGene_1679	7.916007	8.636790658	2.47445898	1.941674863
Mite-foot_newGene_168	14.11090162	17.018261	19.146908	18.8950019
Mite-foot_newGene_1688	0	0	0	0
Mite-foot_newGene_1689	0.243917	7.98E-07	0.00457691	4.04E-06
Mite-foot_newGene_169	1.25309	1.71379	1.60343	2.24966
Mite-foot_newGene_1690	0.956775	0.705931	1.37095	0.776886
Mite-foot_newGene_1691	0	0.128658	0.0977893	0
Mite-foot_newGene_1692	0	0	0.440439	0
Mite-foot_newGene_1694	0.514375	0.664484	0.721944	0.835688
Mite-foot_newGene_1695	0.0496866	0.102987	0.0400872	0
Mite-foot_newGene_1696	0.445041	0.52303	0.0911296	0.108073
Mite-foot_newGene_1697	4.13052	4.24563	4.06988	3.64773
Mite-foot_newGene_1698	3.36538	2.96244	0.890875	1.02258
Mite-foot_newGene_1699	0	0	0	0
Mite-foot_newGene_17	42.10476332	39.35727259	49.79123766	51.6898267
Mite-foot_newGene_170	131.5792646	146.5059998	116.5870413	108.4156286
Mite-foot_newGene_171	8.63226	8.38427	3.28886	2.190515
Mite-foot_newGene_1715	6.59418	5.93056	2.72987	2.50273
Mite-foot_newGene_1716	0	0	0.0493486	0
Mite-foot_newGene_1717	0.271727	0.597943	0.8675	0.617357
Mite-foot_newGene_1718	1.73667	2.64958	2.18547	4.44422
Mite-foot_newGene_1719	3.503445601	2.87144042	2.259141085	2.902600935
Mite-foot_newGene_172	6.969738	1.6151553	1.5709	4.879845

Mite-foot_newGene_1720	0	0	0	0.16172
Mite-foot_newGene_1721	11.825	10.7382	18.6441	23.4551
Mite-foot_newGene_1722	4.096699	4.1389387	1.831894	1.679895812
Mite-foot_newGene_1723	0	1.71755	0	0
Mite-foot_newGene_1724	0.243098	0.100677	0.316926	0.140548
Mite-foot_newGene_1725	69.6495	70.7993	32.0355	26.8341
Mite-foot_newGene_1726	0.466867	0.360895	0	0
Mite-foot_newGene_1727	0	0	0	0
Mite-foot_newGene_1728	0	0	0	0.0647069
Mite-foot_newGene_173	32.99805	34.03438302	39.44514633	33.68894884
Mite-foot_newGene_1743	0.301956	0.208789	0.040462	0.143166
Mite-foot_newGene_1744	114.072	124.194	76.6064	62.9051
Mite-foot_newGene_1745	0	0.104816	0.0544062	0
Mite-foot_newGene_1746	21.1441	21.5105	26.6778	28.8651
Mite-foot_newGene_1747	28.89324	26.95868	46.06482	47.4221
Mite-foot_newGene_1748	0	0	0	0
Mite-foot_newGene_1749	0	0	0	0
Mite-foot_newGene_1750	0.458533	1.25872	0.616291	0.587165
Mite-foot_newGene_1751	24.0445	26.1129	22.7127	22.0888
Mite-foot_newGene_1752	0	0	0	0.0346066
Mite-foot_newGene_1753	0.47378	0.12226	0.385198	0.342606
Mite-foot_newGene_1754	38.16895	32.43827	41.29236	43.80596
Mite-foot_newGene_1755	135.426	124.705	59.4703	61.7255
Mite-foot_newGene_1756	0.702834	0.600507	1.13308	0.633115
Mite-foot_newGene_1757	5.46094	4.84621	5.21187	6.48546
Mite-foot_newGene_1758	29.98400234	27.648616	10.80148234	9.386161924
Mite-foot_newGene_1759	4.020497	2.47271	1.810846	2.270391
Mite-foot_newGene_176	13.8983	13.16971	11.70057	11.35413
Mite-foot_newGene_1760	0.185708	0.10141538	0.377365433	0.715928568
Mite-foot_newGene_1762	40.6043	37.0492	31.1927	33.6097
Mite-foot_newGene_1763	7.92732	6.07806	9.95855	13.3336
Mite-foot_newGene_1764	235.951	186.355	242.849	299.539
Mite-foot_newGene_1765	21.0408	15.3426	12.0279	9.47667
Mite-foot_newGene_1766	0.442524	0.106566	0.249752	0.424613
Mite-foot_newGene_177	19.62978079	19.63106	11.0448	9.363454352
Mite-foot_newGene_178	0.112459	9.81E-08	0	5.57E-06
Mite-foot_newGene_1789	0	0	0	0
Mite-foot_newGene_1790	0	0	0	0
Mite-foot_newGene_1791	8.93854	10.53509	11.18786	19.10315
Mite-foot_newGene_1792	0.721834	0.339095	0.500935	0.694991
Mite-foot_newGene_1793	0	0	0	0
Mite-foot_newGene_1794	18.71544	18.48427	20.75559	22.84273
Mite-foot_newGene_1795	0.563192	0.235211	0	0.539917
Mite-foot_newGene_1796	60.87449	57.381	68.5196	90.35838

Mite-foot_newGene_18	2.501607898	1.944805726	3.139258	3.99096876
Mite-foot_newGene_180	227.686	215.882	184.625	378.932
Mite-foot_newGene_1806	0.780349	0.672686	1.63766	1.37996
Mite-foot_newGene_1807	5.88459	5.12296	9.28586	10.3465
Mite-foot_newGene_1808	2.47095	2.7014	3.34797	4.46157
Mite-foot_newGene_1809	21.958667	20.67363236	35.66165494	38.16099614
Mite-foot_newGene_181	0.0733575	0.101115	0.0198848	0.0234475
Mite-foot_newGene_1810	0.677321	0.553601	0.890231	0.863778
Mite-foot_newGene_1811	0	0	0	0
Mite-foot_newGene_1812	16.75441	16.97463	26.39654	25.49112
Mite-foot_newGene_1813	0.777024	0.74467	0.0614376	0.0543315
Mite-foot_newGene_1814	1.185121521	1.246791628	0.474578	0.3967992
Mite-foot_newGene_1815	0	0	0	0
Mite-foot_newGene_182	0	0	0	0
Mite-foot_newGene_1828	2.591086	2.306132	1.234576393	1.20659
Mite-foot_newGene_183	0	0	0.401403	0.11986
Mite-foot_newGene_1831	0	0	0	0
Mite-foot_newGene_1833	0	0.123991	0	0
Mite-foot_newGene_1834	4.83413	4.01983	6.34456	6.46798
Mite-foot_newGene_1835	0.0952635	0.0980538	0.0766222	0.0905255
Mite-foot_newGene_1836	0	0	0	0
Mite-foot_newGene_1848	0.142272	0.147242	0.68842	0.20334
Mite-foot_newGene_1849	613.317	640.305	433.169	425.642
Mite-foot_newGene_1850	0	0	0	0
Mite-foot_newGene_1851	0.0594387	0.0618983	0.0968444	0.172347
Mite-foot_newGene_1852	5.27E-45	0.07796315	3.92E-08	1.08E-12
Mite-foot_newGene_1853	1.2136	1.8116	1.39263	0.709736
Mite-foot_newGene_1854	1.01009	1.41182	1.18298	1.68523
Mite-foot_newGene_1855	0.32852452	0.346208	0.440146	0.958761
Mite-foot_newGene_1856	1.874979	2.784605	3.652646	3.578974
Mite-foot_newGene_1857	1.71889	0.86021	2.36725	2.31929
Mite-foot_newGene_1858	6.040319	6.25712	4.172112282	3.821802
Mite-foot_newGene_1859	0	0	0	0
Mite-foot_newGene_1860	52.95579	58.92332	18.37413	15.49473
Mite-foot_newGene_1861	0	0	0	0
Mite-foot_newGene_1878	0	0	0	0
Mite-foot_newGene_1879	0	0.0897853	0.0562205	0.149288
Mite-foot_newGene_1880	0	0.883702	0.139149	0.331175
Mite-foot_newGene_1881	12.6822	13.4694	7.13727	7.13277
Mite-foot_newGene_1882	56.5739	51.1554	34.5239	31.3185
Mite-foot_newGene_1883	0.110625	0.173265	0.135183	0.106551
Mite-foot_newGene_1884	1.58179	0.408949	0	0.472428
Mite-foot_newGene_1885	0	0	0	0
Mite-foot_newGene_1886	2.831668889	3.675798	4.369698092	5.914941

Mite-foot_newGene_1887	851.498	744.879	701.103	1266.82
Mite-foot_newGene_1888	0	0	0	0
Mite-foot_newGene_1889	35.18371	33.50391	55.92155	54.33796
Mite-foot_newGene_1897	20.85211	20.28727	25.94238	30.40345
Mite-foot_newGene_1898	0	0	0	0
Mite-foot_newGene_1899	18.2921	18.665	17.7419	18.1172
Mite-foot_newGene_19	11.7309	10.6532	7.72501	8.00052
Mite-foot_newGene_1900	48.9369	42.1901	27.8513	25.8158
Mite-foot_newGene_1901	0.767956	0.502039	1.05422	0.117398
Mite-foot_newGene_1902	11.3379	14.757	6.71621	7.04144
Mite-foot_newGene_1903	0	0	0	0
Mite-foot_newGene_1904	0.50384903	1.046709098	0.047425006	1.345673
Mite-foot_newGene_1905	499.0922607	522.5519395	639.434108	852.172012
Mite-foot_newGene_1906	0.695326	0.840056	2.09174	2.01275
Mite-foot_newGene_1907	0.655912468	0.685549859	0.7303732	0.696442652
Mite-foot_newGene_1908	0.2557374	0.243135	0.506546	0.0606389
Mite-foot_newGene_1909	45.77892634	42.76167872	32.00949094	36.43379895
Mite-foot_newGene_1929	1.236321	1.549892	0.4137556	0.4116742
Mite-foot_newGene_1930	0.229658	0.241089	0.18835	0.148177
Mite-foot_newGene_1931	0.155527	0.16356	0.127307	0.376212
Mite-foot_newGene_1932	4.03476	3.142820689	6.60136794	6.9750235
Mite-foot_newGene_1933	0.0881376	0.212369	0.142149	0
Mite-foot_newGene_1934	0.513248	0.353044	0.137577	0.162702
Mite-foot_newGene_1946	31.8882	34.604	41.7303	53.6465
Mite-foot_newGene_1948	0.403292	0.208875	0.360713	0.523497
Mite-foot_newGene_1949	23.0223	24.7915	21.8071	19.482
Mite-foot_newGene_1950	0	0	0	0
Mite-foot_newGene_1951	0.067094485	0	0.0926071	0.09136989
Mite-foot_newGene_1952	0.240902	0.000152146	0.364904	0.672108
Mite-foot_newGene_1953	0	0	0	0
Mite-foot_newGene_1954	1.608582	1.235538	1.053641783	0.76987142
Mite-foot_newGene_1955	0.422864	0.0732212	0.171139	0.135183
Mite-foot_newGene_1956	0	0	0	0
Mite-foot_newGene_1961	3.62538	2.617474	1.538039	1.816656
Mite-foot_newGene_1962	0.706921105	1.373881001	0.707056656	0.216527485
Mite-foot_newGene_1963	23.52902	20.14002	15.22816	12.57718359
Mite-foot_newGene_1971	14.7137	13.7891	5.63423	4.9073
Mite-foot_newGene_1972	0	0	0	0
Mite-foot_newGene_1973	0.585738779	0.279948736	0.999260711	0.694545362
Mite-foot_newGene_1974	4.183349	6.955852	7.688584	9.008288
Mite-foot_newGene_1975	0	0	0	0
Mite-foot_newGene_1976	0.035827	0.0731524	0.0868749	0.0343496
Mite-foot_newGene_1977	0.690149	0.719703	0.592488	0.263018
Mite-foot_newGene_1978	1.836019	1.798059166	2.472926	2.16162176

Mite-foot_newGene_1979	0.436293	0.180711	0.409559	0.24961
Mite-foot_newGene_1994	30.22766	32.21828	41.74414	41.54289
Mite-foot_newGene_1995	0	0	0	0
Mite-foot_newGene_1996	0	0	0	0
Mite-foot_newGene_1997	0.869847	1.08294	0.425813	1.11887
Mite-foot_newGene_1998	32.8943	33.2792	20.2835	21.7876
Mite-foot_newGene_1999	1.09105	2.12E-06	3.01E-07	0.51917
Mite-foot_newGene_2	80.9849	70.8145	87.6329	125.921
Mite-foot_newGene_20	23.9429	19.2966	20.4513	21.1233
Mite-foot_newGene_2000	0.76646	0.780187	0.458343	0.490897
Mite-foot_newGene_2001	20.42138	18.88006	17.09264	15.02899
Mite-foot_newGene_2002	0.857619	0.650846	0.688772	0.596754
Mite-foot_newGene_2003	0.0833749	0.34736	0.271672	0.402793
Mite-foot_newGene_2004	6.07696	4.74738	5.06644	5.92178
Mite-foot_newGene_2005	7.21421	7.03192	7.38986	10.9071
Mite-foot_newGene_2006	0	0.12936	0.0337643	0
Mite-foot_newGene_2007	11.17543147	12.082663	18.1764446	22.09742759
Mite-foot_newGene_2038	2.7228	2.55716	2.51034	3.34899
Mite-foot_newGene_2039	4.32521	3.54886	1.41692	0.975207
Mite-foot_newGene_2040	0.537737	0.676117	0.170441	0.0900374
Mite-foot_newGene_2041	0.385234	0.420939	0	0.381737
Mite-foot_newGene_2042	4.7242	3.03293	2.17665	2.80401
Mite-foot_newGene_2047	39.3978	37.2878	17.0574	17.9471
Mite-foot_newGene_2048	0	0	0	0
Mite-foot_newGene_2049	0.492928	0.712776	1.69E-05	0.0214012
Mite-foot_newGene_2050	13.99809501	13.1475019	23.14112476	24.17467658
Mite-foot_newGene_2051	219.4223475	180.289692	221.6513772	195.0687286
Mite-foot_newGene_2052	49.07769428	43.55059321	53.31537611	70.931831
Mite-foot_newGene_2053	1.20234	3.54E-10	0.0173453	0.000170032
Mite-foot_newGene_2054	0.84028	0.503842	0.695482	0.690814
Mite-foot_newGene_2072	0	0	0	0
Mite-foot_newGene_2073	0.320117	0.376125	0.700811	0.557073
Mite-foot_newGene_2079	0	0	0	0
Mite-foot_newGene_2080	0	0	0	0
Mite-foot_newGene_2081	0.549546	0.984864	0.573666	0.40205666
Mite-foot_newGene_2082	3.58793	4.35858	5.80244	6.62569
Mite-foot_newGene_2083	46.6357	47.609	65.1672	81.2158
Mite-foot_newGene_2085	0	0	0.117323	0.139593
Mite-foot_newGene_2086	37.619928	29.954804	16.18400518	8.84332166
Mite-foot_newGene_2087	0.00105002	0	0.163443	0.0777648
Mite-foot_newGene_2088	0	0	0.0273555	0
Mite-foot_newGene_2089	6.67818	6.58247	5.67055	8.23927
Mite-foot_newGene_2090	0.28187887	0.140066682	0.266650233	0.378676489
Mite-foot_newGene_2091	0.195813	0	0.158822	0

Mite-foot_newGene_21	0	0	0	0
Mite-foot_newGene_2101	5.19133	4.59006	2.62465	2.1532
Mite-foot_newGene_2102	0.0599975	0	0.048702	0
Mite-foot_newGene_2116	0.557516	0.590515	0.564785	0.761235
Mite-foot_newGene_2117	0.595919	0.357021	0.740138	1.16366
Mite-foot_newGene_2118	0.913678	0.552833236	1.568198	1.970993
Mite-foot_newGene_2119	0.773311586	0.461804343	0.402132596	0.261009931
Mite-foot_newGene_2120	0	0	0.550449	0.328971
Mite-foot_newGene_2121	0.413643	0.934983	0.133123	0.157594
Mite-foot_newGene_2122	4.2658	3.33147	2.66273	2.30943
Mite-foot_newGene_2123	4.674572	4.428913	5.040032	5.939232
Mite-foot_newGene_2124	1.37969	1.15794	0.221541	1.31746
Mite-foot_newGene_2125	2.74584	2.66582	1.28687	1.34227
Mite-foot_newGene_2126	1.280121363	1.189164658	0.92136075	0.686628489
Mite-foot_newGene_2127	0	0	0.0755268	0.0888917
Mite-foot_newGene_2128	1.54913	1.54073	1.90995	1.55217
Mite-foot_newGene_2129	6.819795	4.102504	6.1520782	7.61138296
Mite-foot_newGene_2142	0	0	0	0
Mite-foot_newGene_2143	1.49217	3.41213	0.121092	0
Mite-foot_newGene_2144	0.372913	0.196712	0	0
Mite-foot_newGene_2145	2.61336	3.31823	3.12618	2.94334
Mite-foot_newGene_2146	2.7519232	1.94977	1.822641014	1.456968988
Mite-foot_newGene_2147	7.29526	5.9679	10.0921	12.7135
Mite-foot_newGene_2148	0.089667322	0	0	0.086175234
Mite-foot_newGene_2149	52.62724	52.84867	36.03531	33.35206
Mite-foot_newGene_2150	0.546763492	0.486691	0.484319	0.274952
Mite-foot_newGene_2151	0.115702	0.847311	0.568219	0.797047
Mite-foot_newGene_2161	2.86126	3.31727724	1.002197	0.810812078
Mite-foot_newGene_2162	0.258682	0.559679	0.401306	0.224452
Mite-foot_newGene_2163	10.3628	8.90788	6.42814	5.9496
Mite-foot_newGene_2165	1.32443	0.935386	0.537642	0.521379
Mite-foot_newGene_2166	0.155697	0.216207	0.590174	0.04971
Mite-foot_newGene_2167	5.76157	10.098135	16.6583683	14.98624
Mite-foot_newGene_2168	0.092102	0.0949035	0.59907	0.0443773
Mite-foot_newGene_2169	4.22082	4.68119	11.2009	8.20964
Mite-foot_newGene_2170	0	0	0.103621	0
Mite-foot_newGene_2181	1.02438	0.936625	0.515804	0.169209
Mite-foot_newGene_2182	19.78849044	18.315825	34.682716	31.07354673
Mite-foot_newGene_2183	0.0281189	0.0583517	0.0914591	0.0810847
Mite-foot_newGene_2184	4.16445	4.46097	7.15943	8.05147
Mite-foot_newGene_2194	2.47819	3.27969	2.21251	1.68536
Mite-foot_newGene_2195	0.243177	0.421979	0.131632	0.0780545
Mite-foot_newGene_2196	0	0	0.0745749	0
Mite-foot_newGene_2197	0	0	0	0

Mite-foot_newGene_2198	0	0	0	0
Mite-foot_newGene_22	0	0	0	0
Mite-foot_newGene_2201	0.0928403	0.0971132	0.152956	0
Mite-foot_newGene_2202	16.01501	15.21077	20.648092	27.30465
Mite-foot_newGene_2203	45.3599	45.55419	33.45463	31.01637
Mite-foot_newGene_2213	15.22735439	14.93311397	23.14754759	26.79649658
Mite-foot_newGene_2214	0.785855	0.68518	0.533259	0.0417863
Mite-foot_newGene_2215	34.1117	31.0451	33.33635	29.73538
Mite-foot_newGene_2216	0.176523	0.146973	0.228438	0.302828
Mite-foot_newGene_2217	0	0	0	0.0539103
Mite-foot_newGene_2218	0	0	0	0
Mite-foot_newGene_2219	1.22187	0.939692	1.01974	0.865554
Mite-foot_newGene_2224	46.5864	47.9495	40.7911	47.5262
Mite-foot_newGene_2225	67.3181	59.4661	57.1089	64.2033
Mite-foot_newGene_2226	0.265404	0	0.322406	0.574231
Mite-foot_newGene_2228	0	0.0823737	0	0
Mite-foot_newGene_2229	10.6023	5.2687	9.50072	11.8238
Mite-foot_newGene_2230	0	0	0	0
Mite-foot_newGene_2231	0	0.0250905	0	0.000570535
Mite-foot_newGene_2232	0	0	0.108819	0.045031384
Mite-foot_newGene_2233	0	0	0	0
Mite-foot_newGene_2234	6.529706	4.95691	5.210546	4.38964
Mite-foot_newGene_2235	2.12095	2.07851	1.4975	1.74607
Mite-foot_newGene_2236	1.15328	1.44822	0.13395	0.236621
Mite-foot_newGene_226	0.111319437	0.172660096	0.069931062	0.332052535
Mite-foot_newGene_2262	23.44841	18.44905	26.27835	42.1311
Mite-foot_newGene_2263	0.0440304	0.0459275	0.0354149	0.0835282
Mite-foot_newGene_2264	0.109241	0.805762	0.4453	0.747027
Mite-foot_newGene_2267	678.493	620.685	911.466	1143.32
Mite-foot_newGene_2268	3.50319	2.37454	3.77125	3.53692
Mite-foot_newGene_2269	47.1195	49.9413	23.2774	21.3288
Mite-foot_newGene_227	0.58876	0.563171	0.702548	0.415087
Mite-foot_newGene_2270	0	0	0	0
Mite-foot_newGene_2275	0.149298	0.766686	0.481224	0.28668
Mite-foot_newGene_2276	0	0	0	0
Mite-foot_newGene_2277	0	0	0	0
Mite-foot_newGene_2278	0.579453	0.455384	0.357114	0
Mite-foot_newGene_2279	0.0999923	0.206235	0.0809855	0
Mite-foot_newGene_228	0.255368	0.545429	2.52238	2.512
Mite-foot_newGene_2282	0.0952433	0	0.153471	0
Mite-foot_newGene_2283	0.320196486	0.3894733	0.5453515	0.545733052
Mite-foot_newGene_2284	0	0	0	0
Mite-foot_newGene_2285	6.722574322	5.94748522	1.782553641	2.260409532
Mite-foot_newGene_229	43.5905	43.2189	37.3426	33.5864

Mite-foot_newGene_2296	8.49617	6.629724	10.53435	10.715989
Mite-foot_newGene_2297	0.134555	0.343916	0.490542	0.451196
Mite-foot_newGene_2298	0.69373	1.12135	1.06875	0.599351
Mite-foot_newGene_2299	44.4841	45.0542	76.6117	95.6886
Mite-foot_newGene_23	0.203376	0.210787	0.219491	0.129415
Mite-foot_newGene_230	0.642389	0.685534	0.351323	0.406117
Mite-foot_newGene_2300	9.82655	10.0047	12.6703	17.3871
Mite-foot_newGene_2301	0.52502697	0.641085038	0.825056	1.159513418
Mite-foot_newGene_2302	8.115283	8.186534	9.6572	10.65318
Mite-foot_newGene_2303	0.129875056	0.0450623	0.158402114	0.125067053
Mite-foot_newGene_2304	5.263378481	5.834649901	4.147809511	4.926538052
Mite-foot_newGene_2305	0.58176	0.595467	0.100748	0.159251
Mite-foot_newGene_2306	24.6557	29.5418	30.9007	39.241
Mite-foot_newGene_2307	2.25449	2.52051	0.348034	0.309765
Mite-foot_newGene_231	1.40076007	0.959373	2.294667019	2.048128868
Mite-foot_newGene_232	0	0.100212	0	0
Mite-foot_newGene_2321	1.15094	0.850569	0.760844	0.752916
Mite-foot_newGene_2322	33.48354873	29.764048	20.73821576	22.60719765
Mite-foot_newGene_2323	0.17233	0	0	0
Mite-foot_newGene_2324	0.320013026	0.372601155	0.340766126	0.306441094
Mite-foot_newGene_2325	12.569384	13.21978775	9.665188063	9.709678233
Mite-foot_newGene_2327	1.55073555	1.547213906	1.359081	1.401734229
Mite-foot_newGene_233	1.525937	0.993927	0.179953799	0.050522801
Mite-foot_newGene_234	0	0	0	0
Mite-foot_newGene_235	11.38524659	10.17144203	15.18056	15.58403
Mite-foot_newGene_2350	24.19496	23.807206	36.201953	44.235013
Mite-foot_newGene_2351	41.3748	45.1807	62.2981	55.8105
Mite-foot_newGene_2352	48.91042961	50.68932986	27.26621473	23.66082777
Mite-foot_newGene_2353	3.511054	3.616171	5.80934	8.89486
Mite-foot_newGene_2354	1.97E-08	1.17E-52	0.000855137	0.515358
Mite-foot_newGene_2355	1.367948	1.318050911	0.437175018	0.422479945
Mite-foot_newGene_2356	0	0	0	0
Mite-foot_newGene_2357	0.179224	0.24949	0.19324	0.170527
Mite-foot_newGene_2365	0.674744	0.38548	0.945729985	1.0780835
Mite-foot_newGene_2366	4.29047	2.912002	4.36753	2.997998
Mite-foot_newGene_2367	1.773584213	1.537019407	2.69283935	3.087848525
Mite-foot_newGene_2368	2.7496	0.284362	3.343448	2.464157
Mite-foot_newGene_2369	0.050619393	0.089293	0.0940278	0.1440448
Mite-foot_newGene_237	1.097083	1.391722947	2.258366	2.241291539
Mite-foot_newGene_2370	9.74761	8.97893	4.37695	4.55362
Mite-foot_newGene_2371	41.2719697	44.64499063	71.716719	72.9123
Mite-foot_newGene_238	0	0	0	0
Mite-foot_newGene_2382	20.7358	27.6551	19.9647	22.2668
Mite-foot_newGene_2383	100.37	108.041	88.5231	72.2442

Mite-foot_newGene_2384	4.09984	15.2888	0.0769144	0
Mite-foot_newGene_2390	0.452263	0.58448	0.548874	0.943057
Mite-foot_newGene_2391	3.404551188	4.0425084	3.766814281	4.660723094
Mite-foot_newGene_2392	42.8595	43.7207	48.1326	56.3711
Mite-foot_newGene_2393	9.41514	9.58675	12.54949	14.06019
Mite-foot_newGene_2394	21.26853	22.73952	14.87485	11.57878
Mite-foot_newGene_2395	0	0	0	0
Mite-foot_newGene_2407	8.77735	8.70216	13.0955	13.4327
Mite-foot_newGene_2408	1.613883	0.312205425	1.498187	0.767107269
Mite-foot_newGene_2409	7.29823	5.312853	5.047949	4.986977
Mite-foot_newGene_2410	6.475774899	4.196548663	5.463334218	3.863189816
Mite-foot_newGene_2411	24.6042	22.6152	9.39106	7.96403
Mite-foot_newGene_2412	0	0	0	0
Mite-foot_newGene_2413	0	0	0	0
Mite-foot_newGene_2414	1.660259	3.441157	1.84937	2.942271
Mite-foot_newGene_2415	53.71357055	56.08043457	32.7027025	24.67402235
Mite-foot_newGene_2416	57.0738996	61.924722	81.7248168	87.7245043
Mite-foot_newGene_2417	2.204509585	1.2685132	2.576423102	3.74692457
Mite-foot_newGene_2418	0	0	0	0
Mite-foot_newGene_2419	11.78281	11.51369	7.87565	6.74217
Mite-foot_newGene_2420	0.365874	0.22266	0.296644	0.028551
Mite-foot_newGene_2421	148.3042539	143.79694	104.0599952	94.8769727
Mite-foot_newGene_2422	0	0	0	0
Mite-foot_newGene_2423	93.62685393	93.57076452	137.0711206	123.0565873
Mite-foot_newGene_2424	27.00383872	29.383491	15.04876174	15.418887
Mite-foot_newGene_2425	49.9597	61.7744	29.0346	32.2093
Mite-foot_newGene_2426	20.122578	18.5733812	12.278868	13.99943052
Mite-foot_newGene_2427	0.941899	1.02097	0.34097	0.101257
Mite-foot_newGene_2428	0.3028253	0.868115	0.615856	0.583432002
Mite-foot_newGene_2457	1.03989	0.612038	0.644432	0.4506144
Mite-foot_newGene_2458	22.6896	28.7644	47.8138	47.9487
Mite-foot_newGene_2459	1395.91	1347.93	1142.34	1055.88
Mite-foot_newGene_2460	0	0	0	0.0967088
Mite-foot_newGene_2461	14.79404	17.7374078	24.8930159	27.420722
Mite-foot_newGene_2462	35.2182	23.5331	22.3095	30.7867
Mite-foot_newGene_2477	0	0.0689823	0.108463	0
Mite-foot_newGene_2478	0	0	0	0
Mite-foot_newGene_2479	0.600869	0.436481	0.676723	0.231176
Mite-foot_newGene_2480	0	0	0	0
Mite-foot_newGene_2482	0.039373	0.0358748	0.0470957	0.219485
Mite-foot_newGene_2483	0	0	0	0
Mite-foot_newGene_2484	0.804713	1.148066	1.002846984	1.459749
Mite-foot_newGene_2485	0	0	0	0
Mite-foot_newGene_2489	83.6381	75.7675	83.6098	114.578

Mite-foot_newGene_2491	0	0.110844	0	0
Mite-foot_newGene_2492	3.36652	3.73617	4.89892603	7.12037
Mite-foot_newGene_2493	3.137	29.3754	8.05777	27.8529
Mite-foot_newGene_2494	0.77639	0.556549	0.500532	0.433385
Mite-foot_newGene_2495	0.0658482	0	0	0
Mite-foot_newGene_25	6.05216	7.50074	11.0543	12.8953
Mite-foot_newGene_2504	6.28346	2.0688	4.01497	6.54107
Mite-foot_newGene_2505	45.3278	48.0125	26.6153	24.2307
Mite-foot_newGene_2506	1.500223362	0.992760034	1.459096221	1.273080817
Mite-foot_newGene_2507	1.02291	0	0	0
Mite-foot_newGene_2508	0	0	1.46309	1.7563
Mite-foot_newGene_2509	0	0	0.0936218	0.110316
Mite-foot_newGene_2514	1.14778	0.17156	0.934086	1.11028
Mite-foot_newGene_2516	46.9856	42.1222	41.0858	43.3432
Mite-foot_newGene_2517	1.706043	1.63911	0.956953734	1.114031
Mite-foot_newGene_2518	1.88773	0.605268	0.811385	0.982902
Mite-foot_newGene_2519	0	0.220239	0.0579422	0
Mite-foot_newGene_2520	5.025601443	3.721908322	4.454357	4.427459917
Mite-foot_newGene_2521	5.93664	3.95433	6.03784	6.45266
Mite-foot_newGene_2522	0	0	0	0
Mite-foot_newGene_2523	0	0	0	0
Mite-foot_newGene_2524	0.0573746	0	0	0
Mite-foot_newGene_2525	0	0	0	0
Mite-foot_newGene_2527	0	0	0	0
Mite-foot_newGene_2528	0	0	0	0
Mite-foot_newGene_2540	4.61005	5.80637	4.13227	1.90879
Mite-foot_newGene_2541	0.31266	0	0.770729	1.53825
Mite-foot_newGene_2542	16.5666853	17.7301582	27.1750074	28.9695599
Mite-foot_newGene_2543	46.53223	45.99268	40.89434	42.28297
Mite-foot_newGene_2544	4.86157	5.56619	6.11683	7.77915
Mite-foot_newGene_2545	42.6527	40.6457	47.1083	50.0671
Mite-foot_newGene_2546	0	0	0	0
Mite-foot_newGene_255	0.232556	0.11177	0.0752259	0
Mite-foot_newGene_2551	21.1381	18.9406	14.0185	13.9626
Mite-foot_newGene_2552	8.861078	8.40516	7.345537	6.950684
Mite-foot_newGene_2553	1.573190129	1.568760021	2.394850076	3.9949764
Mite-foot_newGene_2554	1.68469	1.49679	1.51675	1.67605
Mite-foot_newGene_2555	3.074442748	2.986908062	1.435209599	1.791236784
Mite-foot_newGene_256	1.13085	0.839977	1.43638	0.826694
Mite-foot_newGene_2561	1.31824	0.9145	0.545066	0.573759
Mite-foot_newGene_2562	34.1588	42.4847	45.7812	40.8203
Mite-foot_newGene_2563	1.03387	0.591573	0.359918	0.607514
Mite-foot_newGene_2576	0	0.0692494	0	0
Mite-foot_newGene_2577	0.53739	0.388186	0.349908	0.465517

Mite-foot_newGene_2578	4.32887	4.24924	4.94902	8.06977
Mite-foot_newGene_2579	0.29428	0.206787	0.14065	0.000119537
Mite-foot_newGene_2583	0.104302	0	0.0841738	0.0998179
Mite-foot_newGene_2584	0	0.0712175	0.111526647	0.104740636
Mite-foot_newGene_2585	2.90856	3.14216	4.35163	1.72626
Mite-foot_newGene_2586	0	0	0	0
Mite-foot_newGene_2587	0.0808125	0.331809	0	0.309878
Mite-foot_newGene_2588	0.469464	0.45908	0.702696	0.588539
Mite-foot_newGene_2599	5.03E-06	0.2393	0.114665	0.000414759
Mite-foot_newGene_26	0.0103102	0.00164522	0.855152	0.4282
Mite-foot_newGene_2600	0.0796751	0.915889	0	0.385209
Mite-foot_newGene_2601	31.1913	31.5974	34.1599	52.023
Mite-foot_newGene_2602	0	0	0	0
Mite-foot_newGene_2603	0.224427	0	0.548287	0
Mite-foot_newGene_2604	27.0995	23.2507	29.4768	38.7313
Mite-foot_newGene_2605	52.31095358	53.44320116	13.92200222	12.57790008
Mite-foot_newGene_2606	1.149195	0.96033203	0.965587	1.326491
Mite-foot_newGene_2607	0.30206	0.374464	0.114475	0.198557
Mite-foot_newGene_2608	2.42456	2.53453	2.04382	4.00765
Mite-foot_newGene_2609	0.251158	0.134871	0.103996	0.123265
Mite-foot_newGene_2610	132.3912671	140.7900549	223.9233572	239.8784719
Mite-foot_newGene_2617	83.3784	121.597	171.881	177.428
Mite-foot_newGene_2618	0	0.0781451	0.0911245	0.285487
Mite-foot_newGene_2619	1.96503	1.516516	1.453054477	2.422667
Mite-foot_newGene_2620	0.0611421	0.0318195	0.0993398	0.351506
Mite-foot_newGene_2621	0	0	0	0
Mite-foot_newGene_2622	10.01838	9.562676031	6.043209286	5.759867862
Mite-foot_newGene_2623	13.2853	9.51327	14.8284	14.5987
Mite-foot_newGene_2624	15.87979	14.8161	24.93665	26.515183
Mite-foot_newGene_2625	0	0.0308276	0.0965187	0.0284756
Mite-foot_newGene_2626	0.366084	0.381768	0.427801	0.31012
Mite-foot_newGene_2627	0	0	0	0
Mite-foot_newGene_2628	0.215097	0.468905	0.598731	0.331607
Mite-foot_newGene_2629	0	0	0	3.40247
Mite-foot_newGene_2630	15.52296586	14.33988456	11.55126584	10.66657303
Mite-foot_newGene_2632	0.13399813	0.043703321	0.034480448	0
Mite-foot_newGene_2633	8.94582	7.09731	10.12904	14.77908
Mite-foot_newGene_2635	0.0597244	0	0.098057	0.0576167
Mite-foot_newGene_2636	18.529	18.1284	15.5626	15.0743
Mite-foot_newGene_2658	1.4117	1.67238	1.3879	1.00702
Mite-foot_newGene_2659	0.680639	0.749627	0.687527	1.49656
Mite-foot_newGene_266	9.61395045	9.9300579	13.05621482	17.88167
Mite-foot_newGene_2660	0.351003	0.602595	0.560058	0.444717
Mite-foot_newGene_2663	0	0.038452	0	0

Mite-foot_newGene_2664	1.69493	1.22292	0.679553	0.74334
Mite-foot_newGene_2665	6.59642	4.827	4.74001	5.28486
Mite-foot_newGene_2666	5.07E-12	6.39E-16	8.38E-06	7.07E-08
Mite-foot_newGene_267	0.410909848	1.024678863	0.2685486	0.1983053
Mite-foot_newGene_2676	22.1311	21.8017	32.3917	39.4623
Mite-foot_newGene_2689	26.6446924	27.50312655	39.5074172	42.93312998
Mite-foot_newGene_2698	38.4462	35.7672	35.9014	38.6425
Mite-foot_newGene_2699	1215.44	1064.591	964.79	1678.068
Mite-foot_newGene_2700	0	0	0	0
Mite-foot_newGene_2701	5.161571019	4.612345764	8.694074693	8.431175805
Mite-foot_newGene_2708	12.42047521	10.95651824	4.033911	8.357347174
Mite-foot_newGene_2709	36.096285	40.091953	42.04741	48.74881
Mite-foot_newGene_2715	0.148847	0.0768409	0	0.0708719
Mite-foot_newGene_2717	277.7921674	290.765535	256.5090821	301.1111577
Mite-foot_newGene_2718	17.86313	17.81559	13.1606	12.28032
Mite-foot_newGene_2719	6.532947607	7.648154278	7.828351	9.24988394
Mite-foot_newGene_272	3.73678	3.23637	2.01067	1.61344
Mite-foot_newGene_2720	0	0	0.0939942	0
Mite-foot_newGene_2721	55.5653	54.5993	33.5143	29.4055
Mite-foot_newGene_2722	1.38374	1.3171	0.186051	0.333859
Mite-foot_newGene_273	15.3988	19.0156	12.415	13.0623
Mite-foot_newGene_2731	0.786383	0.402876	0.677909	0.161916
Mite-foot_newGene_2738	3.864967559	2.531070713	2.572929774	2.161522685
Mite-foot_newGene_2739	0	0	0	0
Mite-foot_newGene_274	20.72953277	27.36814204	19.18540319	20.50687549
Mite-foot_newGene_2740	0	0	0	0
Mite-foot_newGene_275	0	0	0	0.020272
Mite-foot_newGene_2751	0.745419	0.583565	0.60994	0
Mite-foot_newGene_2755	0.130152	0.270137	1.05724	0.12573
Mite-foot_newGene_2756	0.165942	0	0.178811	0.105937
Mite-foot_newGene_2757	0.251333	0.608896	0.511301	0.321553
Mite-foot_newGene_2758	0.192459	0.130952	0.259605	0.584191
Mite-foot_newGene_276	0.961608	0.691005	0.415165	0.734567
Mite-foot_newGene_2766	0	0	0	0
Mite-foot_newGene_2767	0	0.0409186	0.0637488	0
Mite-foot_newGene_2768	0	0.10565	0.204722	0.33783
Mite-foot_newGene_2769	20.656	18.0046	25.3031	28.0376
Mite-foot_newGene_277	5.76208	7.32931	3.17371	1.64954
Mite-foot_newGene_2770	0.0633927	0.19748	0.257531	0.272911
Mite-foot_newGene_2771	6.65625	7.49121	4.77494	8.98475
Mite-foot_newGene_2772	2.92449	1.32322	3.47128	4.67293
Mite-foot_newGene_2773	0	0	0	0
Mite-foot_newGene_2774	585.603	507.938	795.18	1054.82
Mite-foot_newGene_2775	0	0	0	0

Mite-foot_newGene_2776	0	0	0	0
Mite-foot_newGene_278	5.9275051	7.5910952	0.8145957	0.61378727
Mite-foot_newGene_2789	0.0565388	0	0	0
Mite-foot_newGene_279	66.2279	54.9962	64.9652	79.0587
Mite-foot_newGene_2790	85.75321732	104.1610721	75.40868646	70.73939297
Mite-foot_newGene_2791	76.3234	72.60579	44.98893	38.77693
Mite-foot_newGene_2792	0.0931474	0.256388544	0.219493056	0.217840588
Mite-foot_newGene_2793	2.582012	2.483281	3.578723	4.041111
Mite-foot_newGene_2794	7.69147	9.12266	2.36008	2.16043
Mite-foot_newGene_2795	7.43E-07	6.68E-05	0.182982	0.0707348
Mite-foot_newGene_2796	0.152619	0.283762	0.32314	0.205731
Mite-foot_newGene_2797	56.5234	56.6802	52.5386	56.0897
Mite-foot_newGene_2798	0.48950322	0.351251494	0.656959105	0.772492
Mite-foot_newGene_2799	0	0	0	0
Mite-foot_newGene_28	1.34E-32	1.19E-14	0.942298	2.11E-38
Mite-foot_newGene_280	12.51009	12.83433093	7.24746	4.46651
Mite-foot_newGene_2800	0.472887	0.381356	0	0.151783
Mite-foot_newGene_2801	0.362706	0.537028	0.882568	0.943342
Mite-foot_newGene_2802	2.40036761	1.532735939	2.690543699	3.201558319
Mite-foot_newGene_2804	3.98416	3.11116	5.56393	6.53259
Mite-foot_newGene_2805	0.0684653	0.0235342	0.0368635	0.0217437
Mite-foot_newGene_2806	0	0	0	0.216055
Mite-foot_newGene_2807	1.52746	1.19411	1.7268	1.4814
Mite-foot_newGene_2808	0	0	0	0
Mite-foot_newGene_2831	0.481408	0.301939	0.0939267	0.277934
Mite-foot_newGene_2832	0.223728	0.103537	0.396936	0.287366
Mite-foot_newGene_2833	332.355	317.381	214.68	214.76
Mite-foot_newGene_2834	1.0205	0.949539	0.857577	1.06362
Mite-foot_newGene_2835	0.220445	0.10171	0.138907	0.164063
Mite-foot_newGene_2842	0.316779	0.465832	0.143172	0.125584
Mite-foot_newGene_2843	7.690708	7.097229392	9.745240327	9.198663
Mite-foot_newGene_2844	0.32793	7.72E-14	3.60E-17	3.14E-11
Mite-foot_newGene_2845	0	0.0660532	0.206917	0.0615175
Mite-foot_newGene_2846	0.654785	0.90062	0.863322	0.452719
Mite-foot_newGene_2847	0.585194	0.405608	0.389919	0.299995
Mite-foot_newGene_286	0	0	0	0
Mite-foot_newGene_2860	0	0	0	0
Mite-foot_newGene_2861	1.11234	1.25311	0.512939	0.190196
Mite-foot_newGene_2862	0	0	0	0
Mite-foot_newGene_287	0.269217	0.552316	0.549739	0.130992
Mite-foot_newGene_2870	0	0	0	0
Mite-foot_newGene_2871	17.74126897	18.252053	29.79227124	31.60863
Mite-foot_newGene_2872	0	0	0	0
Mite-foot_newGene_2873	0	0	0	0

Mite-foot_newGene_2874	0	0	0	0
Mite-foot_newGene_2876	0	0.0779546	0	0
Mite-foot_newGene_2877	9.96843	8.33146	14.2153	14.6256
Mite-foot_newGene_2878	1.415509	1.157211	0.613597	0.63543
Mite-foot_newGene_2879	0.428887	0.111178	0.563489	0.358798
Mite-foot_newGene_288	1.43872	1.611	3.42652	2.49421
Mite-foot_newGene_2887	0	0.31443	1.09E-05	0.382205
Mite-foot_newGene_2889	1.39772	1.479285	1.96847	2.18224
Mite-foot_newGene_289	9.39588	8.84581	11.2527	10.7302
Mite-foot_newGene_29	2.12659	2.48277	2.31651	2.07029
Mite-foot_newGene_290	4.610368	5.28868	1.055752	0.716868
Mite-foot_newGene_2903	0.414522	0.351377	0.397162	0.433869
Mite-foot_newGene_2904	3.36138	3.24515	5.41312	6.22694
Mite-foot_newGene_291	127.45054	117.1809961	207.56099	209.19079
Mite-foot_newGene_2910	0	0.0514334	0	0
Mite-foot_newGene_2911	0	0.10389	0	0
Mite-foot_newGene_2919	1.396715	0.883818	1.992915	1.517052
Mite-foot_newGene_2920	0.0334774	0.174925	0.0272811	0.0322938
Mite-foot_newGene_2921	0.181837	0	0.135803	0.154172
Mite-foot_newGene_2928	8.23547	8.77003	1.65882	2.65555
Mite-foot_newGene_2935	1.249371	1.085697	1.586038	1.136706
Mite-foot_newGene_2936	0	0	0.0912659	0.218111
Mite-foot_newGene_2937	3.566065931	3.647050942	2.319579672	2.793358578
Mite-foot_newGene_2938	1.3449	1.48368	0.361342	0.17051
Mite-foot_newGene_2939	0.5226318	0.770789	0.805161	0.975482
Mite-foot_newGene_2940	0.335223	0	0.182414	0.108127
Mite-foot_newGene_2941	1.04311	1.63437	1.03099	1.02171
Mite-foot_newGene_3	0	0	0.241431	0.28782
Mite-foot_newGene_30	34.48878	42.3559	16.9094702	17.06505
Mite-foot_newGene_305	3.225248	3.077063	2.467368	2.229623
Mite-foot_newGene_306	0.964314	0.631907	0.63918	1.09513
Mite-foot_newGene_309	10.251	9.14952	7.28801	5.70735
Mite-foot_newGene_32	0	0	0	0
Mite-foot_newGene_320	1.24589	2.01717	0.642167	0.411472
Mite-foot_newGene_321	45.3349	49.2932	45.9286	43.8042
Mite-foot_newGene_322	0	0	0	0
Mite-foot_newGene_323	2.362856882	2.196626	0.231482886	0.309320129
Mite-foot_newGene_324	0.650371	1.89269	2.02079	2.22296
Mite-foot_newGene_329	0	8.79E-45	0	0
Mite-foot_newGene_33	0	0	0	0.0574398
Mite-foot_newGene_330	0.0296134	2.54E-07	0.0639402	0.100504
Mite-foot_newGene_331	0.442174	0.27939	0.515588	0.424945
Mite-foot_newGene_332	0	0	0	0
Mite-foot_newGene_34	1.9737	7.43985	0.74976	1.93373

Mite-foot_newGene_342	0	0	0	0
Mite-foot_newGene_343	8.93956	9.13301	14.8482	17.5517
Mite-foot_newGene_344	0.318474	0.207306	0.55057	0.192257
Mite-foot_newGene_347	0.98196	6.47E-96	2.25E-20	1.37E-298
Mite-foot_newGene_348	23.07313	21.52385	29.33718	29.29765
Mite-foot_newGene_349	0	0	0	0
Mite-foot_newGene_35	0	0	0.185609	0.221563
Mite-foot_newGene_355	0	0	0	0
Mite-foot_newGene_356	66.036	69.224	64.38	64.9926
Mite-foot_newGene_357	3.588613233	2.813998691	3.021431179	3.140384556
Mite-foot_newGene_358	7.08106	5.43282	3.91809	1.87562
Mite-foot_newGene_359	1.76696	1.33382	2.11898	1.85915
Mite-foot_newGene_36	6.21E-09	8.14E-08	0.734322	7.16E-08
Mite-foot_newGene_360	23.42935416	24.20670915	39.12434855	42.63642328
Mite-foot_newGene_362	0.639481	0.502838	0.946276	0.695725
Mite-foot_newGene_363	0.0750338	0.426114	0.457174	0.324476
Mite-foot_newGene_364	67.06475065	75.04511285	65.55461024	55.77659162
Mite-foot_newGene_365	11.6913	11.9434	8.30376	8.29437
Mite-foot_newGene_366	0.653269	0.734246	0.424684	0.30072
Mite-foot_newGene_367	0	0	0	0.22712
Mite-foot_newGene_37	23.58989	20.614212	22.715511	24.90290847
Mite-foot_newGene_370	6.02404	4.4192	5.90841	10.5348
Mite-foot_newGene_371	0.349803	1.10302	0.284164	0.169494
Mite-foot_newGene_372	14.47714756	14.50129367	18.49890402	19.3067345
Mite-foot_newGene_373	0	0.174254	0	0
Mite-foot_newGene_375	18.1628551	18.75728	18.622562	17.2442397
Mite-foot_newGene_38	0	0	0	0
Mite-foot_newGene_381	8.534578753	6.902932917	8.246948728	9.893990258
Mite-foot_newGene_382	0	0	0	0.088062
Mite-foot_newGene_383	4.17703	2.04604	3.09365	5.93377
Mite-foot_newGene_384	4.095432888	5.475713	4.61577	7.39519204
Mite-foot_newGene_389	0	0	0	0
Mite-foot_newGene_39	16.6227	12.1445	17.3717	19.2019
Mite-foot_newGene_390	0	0	0	0
Mite-foot_newGene_391	0	0	0	0
Mite-foot_newGene_392	0.227874922	0.44014995	0.47755511	0.383138131
Mite-foot_newGene_393	0	0	0	0
Mite-foot_newGene_4	3.361297409	3.67920816	2.856304613	2.908207833
Mite-foot_newGene_40	0	0.318049243	0.730315707	0
Mite-foot_newGene_400	0.161338561	9.17E-06	0.406157258	0.419522975
Mite-foot_newGene_409	0.271061	0	3.87E-07	0
Mite-foot_newGene_41	0.0385972	0	0.0312551	0.0738108
Mite-foot_newGene_410	0.7490011	0.831062	0.566787727	0.697513541
Mite-foot_newGene_415	5.33974	2.24133	3.96E-05	4.14E-06

Mite-foot_newGene_416	0.042895098	0.073925818	0.103979403	0.065200495
Mite-foot_newGene_417	0	0	0	0
Mite-foot_newGene_42	22.24784481	22.65042607	36.77078675	42.12731674
Mite-foot_newGene_428	11.6904	14.1205	15.6377	21.2408
Mite-foot_newGene_429	89.8476	83.6873	112.705	128.6228
Mite-foot_newGene_43	3.40469	2.6493	2.41506	2.58018
Mite-foot_newGene_430	3.22861	5.0661	1.47516	1.5459
Mite-foot_newGene_431	0	0.437093	0	0
Mite-foot_newGene_432	0	0	0	0
Mite-foot_newGene_436	0.204647	0.780046	0.468132	0.260043
Mite-foot_newGene_437	0	0	0	0
Mite-foot_newGene_44	0	0	0.47617	0.376771
Mite-foot_newGene_446	0.496344	0.368335	0.547443	0.374065
Mite-foot_newGene_447	5.50264	4.51499	2.68247	3.09908
Mite-foot_newGene_448	2.43443	1.42932	1.05582	1.64722
Mite-foot_newGene_449	22.8226	15.2218	12.1025	13.8387
Mite-foot_newGene_45	2.26206	1.76827	3.288249	6.45958
Mite-foot_newGene_457	4.65508	5.24701	8.21244	6.34636
Mite-foot_newGene_458	5.88430941	5.404545	7.4635469	5.901992
Mite-foot_newGene_459	0	0	0	0
Mite-foot_newGene_46	15.54544525	28.21842927	25.69433456	37.73696345
Mite-foot_newGene_461	0	0	0	0
Mite-foot_newGene_462	0	0	0	0
Mite-foot_newGene_47	0	0.291294	0	0
Mite-foot_newGene_472	0.35972	0.30756	0.582328	0.265603
Mite-foot_newGene_473	1.00224	0.98333	1.59094	1.70638
Mite-foot_newGene_474	0	0.104205	0.321357	0
Mite-foot_newGene_475	20.8978	21.8562	36.8157	52.9277
Mite-foot_newGene_476	4.07365	6.92333	3.8608	3.123
Mite-foot_newGene_478	5.78686	3.25527	6.56649	10.6869
Mite-foot_newGene_479	0	0	0	0
Mite-foot_newGene_48	1.78156	1.56416	2.28428	1.77051
Mite-foot_newGene_480	0.664661	0.242992	0.153776	0.181814
Mite-foot_newGene_481	93.81398	84.8771	98.1313	103.57527
Mite-foot_newGene_482	0	0	0	0
Mite-foot_newGene_49	0	0	0	0.0274906
Mite-foot_newGene_5	30.5715	29.2145	22.5465	23.7936
Mite-foot_newGene_50	8.21176	10.1898	10.5164	10.1673
Mite-foot_newGene_503	96.304612	123.637923	6.709382	3.953905
Mite-foot_newGene_504	1.46908	3.19725	4.06761	1.62152
Mite-foot_newGene_505	0	0	0	0
Mite-foot_newGene_506	0	0.141863137	0	0
Mite-foot_newGene_508	4.6046	5.30519	6.92876	6.52705
Mite-foot_newGene_509	0.930958	0.602354	0.955774	1.35681

Mite-foot_newGene_51	10.05471011	11.859588	11.463959	13.461853
Mite-foot_newGene_510	6.830841567	7.416921389	7.901101065	8.008301238
Mite-foot_newGene_511	0.256353945	0.139520668	0.262221	0.2118987
Mite-foot_newGene_512	0.0550953	0.0562734	0.133212	0
Mite-foot_newGene_52	0	0	0	0
Mite-foot_newGene_520	0	0	0	0
Mite-foot_newGene_521	0.303038	0.26718	0.367063	0.268272
Mite-foot_newGene_522	0.0344671	0.0929419	0.000156004	0.134205
Mite-foot_newGene_528	0.331531	0.614181	0.267752	0.252946
Mite-foot_newGene_529	3.11302	3.12499	0.253502	0.0379166
Mite-foot_newGene_53	12.7378	12.14992	15.70035	17.87974
Mite-foot_newGene_534	11.90201	15.40295	20.44153	17.70919
Mite-foot_newGene_535	22.521	20.3377	30.6073	43.8631
Mite-foot_newGene_54	1.054831841	1.672858752	1.6039192	2.405025233
Mite-foot_newGene_541	47.13332603	43.92226536	72.23540349	71.2453763
Mite-foot_newGene_542	23.7009	24.7539	35.9199	40.7553
Mite-foot_newGene_543	227.403	215.081	210.554	207.319
Mite-foot_newGene_546	0.212424	0.29634	0.234011	0.619388
Mite-foot_newGene_547	1.969200725	1.971555342	1.745998489	1.674989847
Mite-foot_newGene_549	6.692924146	6.461059757	10.361963	11.93362779
Mite-foot_newGene_55	0	0	0	0
Mite-foot_newGene_550	0.246253	0.222701	0.875366	0.354982
Mite-foot_newGene_551	0.386295	0.513673	0.279252	0.440658
Mite-foot_newGene_552	0	0	0	0
Mite-foot_newGene_553	9.15831	9.92779	12.41283	15.64236
Mite-foot_newGene_554	0.569746	0.528035	0.776127	0.449266
Mite-foot_newGene_555	3.4024471	2.71636437	3.698571449	3.644260715
Mite-foot_newGene_556	0	0	0.229417	0.272884
Mite-foot_newGene_557	0.67074375	0.7002027	0.83343536	0.981850637
Mite-foot_newGene_558	0.153821	0.20948	0.205926	0.146272
Mite-foot_newGene_56	0	0	0	0
Mite-foot_newGene_561	0.339209	0.404133	0.312227128	0.3492142
Mite-foot_newGene_562	0.366517	0.508251	0.644381	0.409054
Mite-foot_newGene_563	1.50971	2.44508	2.25553	3.1777
Mite-foot_newGene_564	1.13714	1.43239	1.8588	1.4437
Mite-foot_newGene_565	0.480451	0.543945	0.228541	0.23095
Mite-foot_newGene_566	0.556703	0.61659	0.272987	0.107131
Mite-foot_newGene_567	0.603957	0.0624394	0	0.0290385
Mite-foot_newGene_57	6.490190467	8.101309885	2.309770852	2.352995
Mite-foot_newGene_58	0	0	0	0
Mite-foot_newGene_59	0	0	0.0395387	0.0937298
Mite-foot_newGene_6	0	0	0	0
Mite-foot_newGene_60	11.0417	12.7967	7.33307	10.1773
Mite-foot_newGene_606	0.460659	1.1995	0.134313	0.158782

Mite-foot_newGene_607	3.43E-09	4.435	4.69E-06	6.75E-05
Mite-foot_newGene_61	66.31453177	83.58292044	29.82152631	20.34006647
Mite-foot_newGene_62	37.11353654	38.98829	15.28851091	14.936074
Mite-foot_newGene_621	0	0	0	0
Mite-foot_newGene_622	0	0	0	0
Mite-foot_newGene_623	0.205945085	0.232827	0.723075	0.6461848
Mite-foot_newGene_63	1.11665	1.19391	1.66308	1.82355
Mite-foot_newGene_631	38.074	33.9069	37.8616	55.8656
Mite-foot_newGene_633	0	0.88429	0.67117	0
Mite-foot_newGene_637	0.301061	0.559683	0.256457	0.2016
Mite-foot_newGene_638	2.6101	1.90639	0.870571	6.97E-06
Mite-foot_newGene_639	0	0	0	0
Mite-foot_newGene_64	0.0213134	0.108362	0.278984	0.289769
Mite-foot_newGene_640	44.8789	38.61068	49.88912	52.2153
Mite-foot_newGene_641	51.2906	54.2766	51.003	51.0249
Mite-foot_newGene_642	0	0	0	0
Mite-foot_newGene_643	5.23007	5.94432	4.23844	3.83679
Mite-foot_newGene_646	48.41626	60.75203	73.3899	60.7823
Mite-foot_newGene_647	1.53E-08	2.20E-10	1.43E-08	3.14E-07
Mite-foot_newGene_65	0.0855877	0.0891671	0.281265	0
Mite-foot_newGene_652	1.27173	1.56342	1.12391	0.666739
Mite-foot_newGene_656	0.893088	0.31502	1.3354	0.724711
Mite-foot_newGene_657	0	0.644467	0	0.472346
Mite-foot_newGene_661	30.6319	28.5951	35.6771	36.0887
Mite-foot_newGene_664	0.629592	0.655301	0.170232	0.201645
Mite-foot_newGene_665	15.3503	16.0224	8.67737	5.10866
Mite-foot_newGene_666	0.85815	0.350146	0.372385	0.117458
Mite-foot_newGene_667	6.53268	3.8365	8.34317	6.03554
Mite-foot_newGene_668	11.629481	11.02491978	10.936893	10.372153
Mite-foot_newGene_669	0.164942	0.459294	0.178884186	0.318433
Mite-foot_newGene_670	1.39278	1.48103	1.24817	1.16197
Mite-foot_newGene_671	0	0.201731	0	0
Mite-foot_newGene_672	8.36582	11.469338	7.62386857	11.26746332
Mite-foot_newGene_673	8.251641	10.996145	7.4364475	7.810424081
Mite-foot_newGene_674	6.04526	5.99141	1.68493	0.869863
Mite-foot_newGene_675	0.722845	5.50505	0	0
Mite-foot_newGene_7	0	0	0	0
Mite-foot_newGene_705	0.253026	0.842293	0.246621	0.0972032
Mite-foot_newGene_706	3.018366	3.73926	1.474834	1.021297
Mite-foot_newGene_712	0	0	0	0
Mite-foot_newGene_713	0	0	0	0
Mite-foot_newGene_714	0.84955	1.10552	0.934386	0.980261
Mite-foot_newGene_717	0.727227	0.595905	0.337964	0.449692
Mite-foot_newGene_718	0	0.0275723	0	0

Mite-foot_newGene_719	1.23E-05	0.0962756	0.277211	2.26E-05
Mite-foot_newGene_726	0	0	0	0
Mite-foot_newGene_733	0.2159	0.457966	0	0
Mite-foot_newGene_736	8.96815	8.70868	16.2541	17.6923
Mite-foot_newGene_737	0.53908	0.379004	0.43989	0.173342
Mite-foot_newGene_742	1.243746374	2.343053872	1.840676928	2.217962686
Mite-foot_newGene_743	14.88823	15.236557	24.73372	26.47251
Mite-foot_newGene_746	0.160199	0	0	0
Mite-foot_newGene_747	2.08212	1.54944	0.361608	0.431898
Mite-foot_newGene_748	22.04032472	25.12928619	18.938458	16.85634296
Mite-foot_newGene_749	1.65654	2.34898	1.35969	1.43874
Mite-foot_newGene_750	0	0	0	0
Mite-foot_newGene_751	0	0	0	0
Mite-foot_newGene_753	60.45806	60.1987	62.4401	103.527
Mite-foot_newGene_754	3.890375	3.53018	2.2475	2.18784
Mite-foot_newGene_755	9.7902	7.59805	5.90497	6.0489
Mite-foot_newGene_756	3.11242	2.02291	1.5198	1.43983
Mite-foot_newGene_757	0.331302	0.517143	0.313343	0.265089
Mite-foot_newGene_758	70.4969303	70.44529681	63.503637	59.44659018
Mite-foot_newGene_779	0	0	0	0
Mite-foot_newGene_781	0.0948534	0.10032	0.0775535	0.0915877
Mite-foot_newGene_789	2.00767	1.7679	1.62555	1.57793
Mite-foot_newGene_793	4.59372	3.80473	6.13157	6.76743
Mite-foot_newGene_796	0.061308	0	0.400917	0
Mite-foot_newGene_797	1.27513	0.671529	1.56178	2.17347
Mite-foot_newGene_798	34.3887	24.7906	46.017	66.4868
Mite-foot_newGene_799	0	0.0846259	0	0.0780121
Mite-foot_newGene_800	1.27044	1.45088	1.97847	1.99096
Mite-foot_newGene_801	0.676032	0.351488	1.10377	1.80574
Mite-foot_newGene_802	12.5399	15.969	21.3586	17.4421
Mite-foot_newGene_803	0.922939	0.428264428	0.895893335	0.790854058
Mite-foot_newGene_804	0	0	0	0.173041
Mite-foot_newGene_805	0.0744488	0.15517	0.241048	0.141963
Mite-foot_newGene_806	0	0	0	0.158353
Mite-foot_newGene_807	0	0	0	0
Mite-foot_newGene_808	0.263614	1.53E-18	1.18E-12	0.314604
Mite-foot_newGene_809	0.879068	0.668309	2.03661	0.31438
Mite-foot_newGene_810	5.5819	5.1614	8.0363	9.51106
Mite-foot_newGene_811	4.37849	4.56664	6.37103	12.8148
Mite-foot_newGene_812	0.05926	0	0.0479614	0
Mite-foot_newGene_813	7.1492591	7.343182423	4.815384821	4.232632381
Mite-foot_newGene_814	19.7707037	20.37956115	34.68067019	34.28243744
Mite-foot_newGene_815	0	0	0	0.0784107
Mite-foot_newGene_816	0.6164	0.550156	0.617485	0.271251

Mite-foot_newGene_817	2.64852	3.9295	5.99926	3.58728
Mite-foot_newGene_818	1.37595	0.000233774	0.739734	0.000413462
Mite-foot_newGene_819	17.04149	17.935615	20.79626	22.27244
Mite-foot_newGene_820	10.78103996	11.934232	13.72911193	12.05470121
Mite-foot_newGene_821	0.369030014	0.429293922	1.71566712	1.027470057
Mite-foot_newGene_822	13.9097	8.34459	19.7249	20.95270026
Mite-foot_newGene_824	0.73962	0.738958	0.246486	0.507485
Mite-foot_newGene_832	1.6160802	1.243	1.2350717	1.0269217
Mite-foot_newGene_840	0	0	0	0
Mite-foot_newGene_846	0	0	0	0
Mite-foot_newGene_847	5.480414	4.561990806	3.544490097	2.92430485
Mite-foot_newGene_848	0.280141	0	0	0.0893178
Mite-foot_newGene_854	9.59551	8.62814	6.66699	4.42216
Mite-foot_newGene_855	28.5995	29.9551	21.3335	20.4223
Mite-foot_newGene_856	4.625053943	3.071184114	2.934271633	3.177152615
Mite-foot_newGene_857	0	0	0	0
Mite-foot_newGene_858	1.25867	0.245725	0.399896	9.00E-05
Mite-foot_newGene_859	0.01737772	0.1263822	0.07061442	0.0331635
Mite-foot_newGene_860	0.0475366	0.0497869	0	0
Mite-foot_newGene_861	0	0	0	0
Mite-foot_newGene_862	4.20236	3.76147	2.35724	1.86971
Mite-foot_newGene_863	0.284272	0.133931	0.313628	0.347328
Mite-foot_newGene_864	0	0	0	0
Mite-foot_newGene_865	0	0	0	0
Mite-foot_newGene_866	59.4609	51.93222	61.23661	61.0294
Mite-foot_newGene_868	0	0	0	0
Mite-foot_newGene_869	5.7632	7.73471	7.73411	10.9219
Mite-foot_newGene_895	18.21099	17.73702284	14.17246	10.3154025
Mite-foot_newGene_896	1.79625	2.55176	0.79755	0.906924
Mite-foot_newGene_9	8.18487	7.17986	11.3624	13.467
Mite-foot_newGene_903	2.36072	2.36972	2.63702	3.3617
Mite-foot_newGene_904	0.631297	0.4	0.451558	0.330982
Mite-foot_newGene_905	0	0	0	0
Mite-foot_newGene_906	6.10E-08	3.42E-115	0.459649	7.63E-71
Mite-foot_newGene_907	1.781819	1.895256	2.629632	2.513409
Mite-foot_newGene_908	0	0	0	0
Mite-foot_newGene_909	0	0	0	0
Mite-foot_newGene_910	5.623486429	5.810926406	7.645370844	8.317997617
Mite-foot_newGene_911	8.42E-34	2.34E-157	1.78E-70	6.90E-132
Mite-foot_newGene_912	49.2211	49.575265	90.91613	84.4196
Mite-foot_newGene_913	0.460751	0.796111	0.0630582	0.299614
Mite-foot_newGene_928	1.1139289	0.181256508	0.918218	0.796532
Mite-foot_newGene_929	0.100559	0.104002	0	0
Mite-foot_newGene_936	0	0	0.304869	0

Mite-foot_newGene_942	0.304166	0.400454	0.16336	0.0966916
Mite-foot_newGene_944	0.0778111	0.0198374	0.0470194	0.0743625
Mite-foot_newGene_946	0	0	0	0
Mite-foot_newGene_947	0	0	0	0
Mite-foot_newGene_949	0	0	0	0
Mite-foot_newGene_951	0	0.0904608	0	0.170204
Mite-foot_newGene_952	39.1464	43.31219	26.39933	22.42381
Mite-foot_newGene_953	0.411548698	0.573104641	3.51E-07	3.92E-06
Mite-foot_newGene_954	7.63343	7.64283114	1.331054399	1.084550921
Mite-foot_newGene_955	3.60498	4.81093	4.81795	7.58746
Mite-foot_newGene_963	5.11E-06	4.36E-08	2.31E-07	0.167799
Mite-foot_newGene_964	0.834327	0.394345	0.493283	0.255153
Mite-foot_newGene_965	0.476423	0.5429	0.155299	0.0460013
Mite-foot_newGene_966	0.954583	1.12066	0.539658	0.755601
Mite-foot_newGene_974	0	0	0	0
Mite-foot_newGene_975	1.986361	1.532839	1.311365	1.421512143
Mite-foot_newGene_978	9.16838	5.64675	2.27181	4.41493
Mite-foot_newGene_981	10.78760865	11.55397	14.54455	18.0849
Mite-foot_newGene_982	4.70492519	4.353639	6.08848257	4.63300181
Mite-foot_newGene_983	1.39092	2.53756	3.97533	3.34296
Mite-foot_newGene_984	0	0.0440949	0	0
Mite-foot_newGene_985	10.93303	9.51475	16.09084	16.59258
Mite-foot_newGene_986	0.0844708	0.219408	0.285116	0.1247
Mite-foot_newGene_987	0.711725	1.13455	0.432792	1.02031
Mite-foot_newGene_988	1.091716	0.814295305	0.5989406	0.420445476
Mite-foot_newGene_989	0.64668524	0.766143487	0.9725608	0.9749288
Mite-foot_newGene_990	136.9035546	135.5884781	115.0138575	101.7233885
Mite-foot_newGene_991	23.9409	24.2448	14.3728	12.8937
Mite-foot_newGene_992	0.671086138	0.500894079	0.982234114	0.990600226
Mite-foot_newGene_993	2215.939	2357.085	1642.27	1471.703
Mite-foot_newGene_994	0	0	0	0
Mite-foot_newGene_995	0.466527	0.364784	0.803258	0.503188
Mite-foot_newGene_996	5.405688	4.798337734	3.188786	2.488026081
Mite-foot_newGene_997	3.99704	3.1365	4.29328	6.75572
Mite-foot_newGene_998	22.9555	24.4686	8.38953	7.66334
Mite-foot_newGene_999	0.118721	0.139576	1.84E-122	4.48E-107

larva	larva	nymph	nymph	adult
54.82731	60.29024	67.73645	69.9777	141.834
20.68486	20.08571316	36.045085	24.42648298	33.2353
20.68486	20.08571316	36.045085	24.42648298	49.3141
109.051021	114.19299	65.10723	68.66315	79.23974
109.051021	114.19299	65.10723	68.66315	12.046
13.71840327	12.7193343	15.339256	13.1888913	0.303117
13.71840327	12.7193343	15.339256	13.1888913	3.19887
10.07511638	11.258599	8.845856413	8.869220223	4.639157829
10.07511638	11.258599	8.845856413	8.869220223	4.639157829
35.91872	40.77593	174.8176	198.4586	65.256
21.19552	22.55178	22.31751	21.4225311	31.3337
64.28379	59.30396	12.15542	23.383467	2.587580029
0	0	0.890488	1.33E-06	3.74E-07
0	0	0	0	0
68.26102697	63.81487076	22.51864725	27.32060387	2.674462289
1.903066903	1.270690004	1.7728439	2.692456	36.70059
6.67677125	4.771931214	12.13287078	14.7364005	20.9844221
5.111632001	6.070958	7.161365712	7.12418	29.06919625
15.78298	21.37441	26.8468	25.7896	28.891
7.128243	11.0548001	15.494776	16.36871963	40.2038
7.1298	8.30023	15.06617	13.74278	7.22545
44.95863	29.28425	74.615756	56.93299	14.705046
44.95863	29.28425	74.615756	56.93299	14.705046
0.655954	0.569509	1.09436	1.4755	0.452823
21.709037	24.68301	19.74492	20.44438	5.17369
27.11370484	29.92272211	16.6686862	18.27098	10.75710272
27.11370484	29.92272211	16.6686862	18.27098	10.75710272
7.975827575	8.876905	4.40625372	4.592478856	0
1.353779	1.292034	2.586786	2.749312	19.74077
6.324362	6.96123	9.10581	10.3396	23.07558333
6.324362	6.96123	9.10581	10.3396	8.29745
0	0.525609	0	0.323906	5.8502905
5.54908	9.22876	13.1234	14.1813	5.8502905
181.15117	176.029164	18.641279	66.94720748	26.2603
0.5214114	0.430509	1.138439	0.779667	0
1.073755	0	7.97662	17.61344	13.05372
1.549545	1.036696	5.88987	7.13512	2.80728
0.384224	0.568658	2.68434	2.42431	2.45792
1.8686	1.42635	1.45125	1.7841	1.46098
0.626792	2.54897	1.50226	2.05713	1.01875
0.949642	1.76553	1.08553	1.67922	0.73833
18.8539945	16.233485	14.74307052	15.375345	26.39967
18.8539945	16.233485	14.74307052	15.375345	4.829754119

18.8539945	16.233485	14.74307052	15.375345	4.829754119
0	0	0	0	0
0	0	0	0	0
0	0	0.0810042	0	0.103272
28.00042413	26.65740273	26.40059471	26.32706273	20.087647
45.994971	47.43682	57.57462	60.37037	35.7252
45.994971	47.43682	57.57462	60.37037	35.7252
32.46876926	35.21004857	24.72793156	25.99824082	35.93541013
42.39156138	42.3637876	31.50341405	35.32131635	12.359655
68.04252	75.18855	56.98977	60.35515	58.41407
34.864164	39.144681	41.497815	39.660246	49.6786
0	0	0	0	0
0.001458	4.60782	0.0395259	1.40629	5.11E-05
0.34084697	0.192479613	2.957241984	3.887734857	0.877694017
0.34084697	0.192479613	2.957241984	3.887734857	1.198856357
27.63551	23.54959	18.85251	20.51231	7.44658
3.360606	2.955914	7.14356164	6.7763267	8.499417644
32.93602567	31.37419429	34.623904	36.4492002	44.5454803
11.1379	9.97597	7.04117	7.20029	4.24E-06
2.92261316	1.039104763	43.39930207	101.9560029	94.26040771
20.53867	20.81997	19.03017	15.352574	49.87381812
741.0516919	796.07346	1191.433371	1134.343904	1279.387
0	0	0	0.430522	2.90452
6.85169	9.40408	14.5691	11.5097	58.5344
0	0	2.73105	14.2289	362.504
29.758672	32.78766	23.72106833	20.10428791	42.66731
10.71003	9.892392	5.901776	6.652544	2.51568822
33.74761	32.46074	31.481177	33.630821	67.6597
595.072282	566.02528	769.796712	863.82777	1277.502
4.449912913	4.864602898	5.188205882	5.4361781	38.2861
5.85139	8.06714	12.9665	13.409	20.457
9.251810001	8.29665	6.457801	5.698609	0
9.251810001	8.29665	6.457801	5.698609	3.906962
45.7083169	37.904759	107.73582	116.106535	19.95473
45.7083169	37.904759	107.73582	116.106535	41.585566
45.7083169	37.904759	107.73582	116.106535	41.585566
1.604825382	1.717808003	0.772495	3.427532	3.0250432
1.604825382	1.717808003	0.772495	3.427532	3.0250432
0.334371	0.0705654	0.703784	0.419502	3.73929
19.3894	15.4445	17.4452	14.9859	7.51111
5.89E-12	0	0.221881078	0	4.29147
12.934929	19.73044	11.8953574	13.26463618	39.50126
12.934929	19.73044	11.8953574	13.26463618	39.50126
0	0	0	0	0

13.861	8.21827	10.2363	12.8329	0.585317
10.6982	12.2944	16.3751	13.2935	37.2767
41.835	38.5824	11.43295	17.29028	27.738578
228.24211	217.631	200.289346	195.639	152.972
2.49503	2.74344	5.30046	4.51513	41.697
64.8232	34.5517	14.0189	11.2	0.376272
90.7878	96.0798	31.7694	25.0766	0
233.089	191.146	68.1375	134.504	0.954942
576.602	322.004	220.95	248.783	11.2509
0	0.114897	0.0564086	0.453868	0
84.3988593	77.5037465	64.0653929	55.0439355	0.0966068
84.3988593	77.5037465	64.0653929	55.0439355	1.75318
27.6106	27.2632	96.7477	71.7281	36.144
84.3988593	77.5037465	64.0653929	55.0439355	10.805095
84.3988593	77.5037465	64.0653929	55.0439355	10.805095
0.00840318	0.0508045	0.426572	1.41697	5.628851829
0.091032763	0.038833961	3.316269626	8.744522	5.628851829
0.091032763	0.038833961	3.316269626	8.744522	5.628851829
74.7521413	71.9091898	52.5154425	49.36532025	24.7817031
74.7521413	71.9091898	52.5154425	49.36532025	17.576274
21.50601593	18.38204282	15.66993677	15.73509324	2.95884
21.50601593	18.38204282	15.66993677	15.73509324	133.1177012
21.50601593	18.38204282	15.66993677	15.73509324	133.1177012
0.350043	0.0880953	5.9654	11.6526	133.1177012
8.78E-08	0.132127	2.02E-07	0.0736442	1.29188
0.078351	0.0398411	0.0773888	0.0521527	0
7.255	6.3262	12.3506	11.7726	43.73075
3.47104002	4.0388781	7.700258	9.65427	16.25961477
31.8972938	27.50109978	19.15634207	15.07223147	0.751965
31.8972938	27.50109978	19.15634207	15.07223147	4.99584
17.4287071	16.29909014	11.8609243	11.22391936	2.845483984
1.395599097	1.669973269	1.06374235	0.671618739	0
17.59152	15.5659665	19.57681	20.03089	59.4779
38.449093	41.700049	40.50960337	42.707131	38.99565
38.449093	41.700049	40.50960337	42.707131	38.99565
279.5928	309.59194	220.56636	158.48395	12.87275511
279.5928	309.59194	220.56636	158.48395	12.87275511
0.000316118	0.000203734	5.72E-06	7.51E-06	0.751186
11.617653	14.504975	13.678097	13.055284	22.96673362
11.7530634	13.45072427	5.86859381	5.60052603	0.335042
11.7530634	13.45072427	5.86859381	5.60052603	0.549341
11.7530634	13.45072427	5.86859381	5.60052603	15.663
14.86712	18.13489	10.31765246	8.581358988	0
1.026973	1.29315	0.884838	1.076627	0.848782

14.88527478	13.84740203	11.4604889	11.47886422	1.0921
59.0487	73.0734	93.3019	73.1346	156.525
74.88890018	77.099333	87.986804	86.01363702	299.432
9.52179	9.63739	11.0458	7.98744	12.6164
17.51662	15.7957406	25.5352094	22.17836	37.46772524
4.61512	4.18538	8.36902	7.92006	50.498349
7.5848	10.08427	7.67656	6.78572	1.58E-06
7.5848	10.08427	7.67656	6.78572	3.15511
24.405381	23.54798683	30.48818383	26.42120794	110.82671
45.93033821	45.1819253	23.7913807	27.17878848	10.43620031
0	0	0	0	24.18481716
5.0580845	4.462816194	4.582030016	5.890333099	64.57104
0	0	0	0	0
0	0	0	0	0
10.8913307	10.56050287	19.46760733	16.36462974	12.34703001
10.8913307	10.56050287	19.46760733	16.36462974	12.34703001
5.8526628	5.010498695	16.5840606	18.96182255	12.658501
5.8526628	5.010498695	16.5840606	18.96182255	12.658501
9.910620302	10.39455636	18.38536652	14.96932278	60.9507
0	0.057557646	2.459892608	7.370713	3.649719
0	0	0	0	0
6.319799831	6.5973129	22.85510593	15.34877497	30.35787
9.092802643	8.452276974	27.7299035	24.86490548	13.85700554
5.28553197	5.26538365	9.501153499	9.662338508	1.795919457
7.739047	8.15802	9.73696	8.832099	118.8691
0	0	0	0	0
0	0	0	0	0.104658
0	0	0	0	0
0.605760685	0.1164	0.358736548	0.334837776	0.436444692
0.605760685	0.1164	0.358736548	0.334837776	0.436444692
3.275084	4.144983998	6.05558	5.606319248	63.03441
2.44734	2.99462	4.05022	1.5526	39.7481
5.399676	6.835916678	4.611712095	5.439299623	0.0611821
5.399676	6.835916678	4.611712095	5.439299623	2.711027553
1.187845218	0.907565696	13.119559	22.68596545	123.0192785
27.9569	27.4551	31.7623	32.0469	119.619
0	0	0	0	0
1.21065	1.38685	1.18484	2.36702	5.59791
8.687608342	9.473879549	15.66711486	27.076083	58.74564711
4.830043	5.135986	6.374174	5.814231224	3.583763
4.830043	5.135986	6.374174	5.814231224	3.583763
0.151455	0.254731	0	0.133295	69.1312
26.62072	29.0768	12.46906	14.25917	9.24949
0	0	0	0	0

1.28722	0.347451	3.45E-05	3.107	2.19E-12
0	0	0	0	2.119
1.47334	0.138526	1.18531	4.89E-05	2.63499
16.04484	14.36205	22.1414	15.15374	52.99147
2.545642	1.942948028	5.239662	3.563768	24.21780995
4.529144118	3.478181	8.517525701	10.44522304	6.105444959
751.35	836.606	1180.747	1191.253	984.521
1.27592	1.625	1.80326	2.35108	6.95502
14.59060257	16.92372	9.759863	12.2759	23.7837
18.49667	20.86343	14.7755	14.39504	41.70932238
53.34302	53.56409	52.70900333	50.97824478	35.05299
6.21406	5.91513	5.64203	6.59858	7.40104
1.37044	1.71926	5.21661	2.68992	6.239618
0	0.757768	0.477489	0	36.8477
27.6129	21.90203	20.9026	16.56924	4.32281
67.24873381	66.61518944	89.4803284	86.670078	37.51378509
67.24873381	66.61518944	89.4803284	86.670078	43.8245
13.49647	8.95867	14.54807	5.2164614	16.32453643
13.49647	8.95867	14.54807	5.2164614	16.32453643
28.759469	28.33970068	49.41228931	57.37835693	136.8082
22.97854424	18.91989781	14.93998602	16.345231	6.13963
12.26907	11.69175	5.94168605	5.631240187	3.085410379
33.852367	35.71113	30.22739446	29.6444332	20.78210769
98.3479	61.2474	48.46642	44.721	25.1695
16.548628	16.204864	17.88896832	16.24390155	26.91346
28.15748	25.93384	23.5622	24.11781	87.52541
16.6946	17.50671	27.17712	27.38799	87.52541
19.51931426	23.2746488	16.61192601	12.19263466	78.90823
25.17836	24.7464017	22.52564064	22.06491468	36.49472
5.886834	5.70035849	8.821883	8.537260047	11.52428527
5.886834	5.70035849	8.821883	8.537260047	11.52428527
5.886834	5.70035849	8.821883	8.537260047	11.52428527
34.925784	36.942913	44.193484	50.75724	51.1861
91.1298	90.8105	65.722	62.6583	116.6593
10.61638501	9.707333	4.584387319	5.671340023	124.3058266
10.61638501	9.707333	4.584387319	5.671340023	1.66876
20.02802454	19.60753939	19.60214	24.4148	8.783450608
0.368796	0	0	0	0
0	0	0	0	0
0	0.136744	0.128922	0.527008	0.159931
0	0	0	0	0
5.68036078	5.5930474	1.794509	2.528824	1.30196
66.43660001	59.96430005	53.24551091	37.7584421	16.6894
66.43660001	59.96430005	53.24551091	37.7584421	14.48619

28.4142	31.5687	34.6366	31.5846	36.0167
15.414904	15.6328815	14.959904	14.82672096	33.55099
7.082979176	7.195841144	9.890551	9.70911	42.959
5.41072	5.95094	9.43302	6.93542	46.5178
14.6705	19.8262	10.7024	13.3681	2.94483
42.21862215	41.23882867	26.90373522	30.11878832	7.152776654
0	0	0	0	0
0	0	0	0	0
13.2944598	14.0066726	3.8293329	5.546294808	12.844577
1.57E-10	0.000197476	2.62E-05	0.699417	0.00151051
77.372835	81.06721	29.94493	38.23914335	39.90977
77.372835	81.06721	29.94493	38.23914335	39.90977
134.3610581	121.9141286	24.94275922	25.7911574	2.21278
6.07326	6.957298	9.49827	9.60714	90.1428
79.93730714	81.24157749	101.8527478	96.43739546	517.318
250.89049	265.3369	219.63879	187.28642	274.0620014
0	0	6.26906	14.2845	31.1545
40.2233	32.2233	117.87	114.213	104.972
7.61515	5.30985	11.6472	18.7694	3.83966
465.145	300.474	919.927	911.296	379.088
4.30286	4.815903836	4.63346	4.958448289	1.10271
18.69200311	17.27212911	13.3791111	15.0360381	4.467511534
14.0901	10.9407	13.282	6.38341	36.5282
312.26629	284.4878	637.4609	489.7205	283.895
312.26629	284.4878	637.4609	489.7205	1.80095
49.950948	49.091851	59.671707	57.422023	37.111017
106.2812	72.9123012	310.6798	310.7487	35.68761284
11.72444	7.4677	73.0016	84.42663	10.591615
106.2812	72.9123012	310.6798	310.7487	37.3450804
4.937827981	4.63768282	8.132942	7.260682211	3.564484763
0	0	0	0	0
30.77947	32.4331	22.01715	20.24559	13.69051125
30.77947	32.4331	22.01715	20.24559	13.69051125
6.93E-21	1.18E-145	1.79E-51	5.27E-195	0.164432
0	1.09689	0	1.38256	1.29875
28.466185	30.8984315	4.114137861	9.63142	2.176225888
5.43769	5.23991	8.15663	6.66239	11.1831
5.122594532	5.813043907	8.925139	7.140305985	31.16850146
5.122594532	5.813043907	8.925139	7.140305985	31.16850146
170.22493	155.2541	426.87384	536.72035	34.3225
170.22493	155.2541	426.87384	536.72035	388.124
6.23234445	7.4399639	1.411212773	2.089755678	0.643981
0.4847715	0.64643468	0.604069001	0.859874722	0.225978
4.25210661	4.599765263	4.181866479	6.3615848	30.778209

4.25210661	4.599765263	4.181866479	6.3615848	30.778209
4.25210661	4.599765263	4.181866479	6.3615848	30.778209
7.07233539	8.738773266	7.218685312	6.705570785	2.35395
1.436441	1.05868379	1.519545649	2.269614131	0.187263
1.436441	1.05868379	1.519545649	2.269614131	1.077100599
1.436441	1.05868379	1.519545649	2.269614131	1.077100599
30.49392945	26.95352349	49.10718393	42.168933	48.8749
1066.423402	1017.36652	958.56769	907.77556	621.55
15.0912	15.0724	13.5037	13.4656	24.305
33.87348	32.29591	35.3448647	35.96923	63.1626
22.235779	24.094598	30.115178	24.946563	36.98881099
22.235779	24.094598	30.115178	24.946563	59.5242
17.462679	19.48378	22.55740563	17.95808	48.7215
17.462679	19.48378	22.55740563	17.95808	48.7215
13.74243072	13.78786701	16.76897	16.25271422	97.4652
1066.423402	1017.36652	958.56769	907.77556	5.450091
1066.423402	1017.36652	958.56769	907.77556	5.450091
794.068	1021.75089	699.9526822	548.06876	318.8211776
794.068	1021.75089	699.9526822	548.06876	271.983
794.068	1021.75089	699.9526822	548.06876	271.983
4.36931	3.37853	4.50609	4.17473	2.16134
53.3822	57.78226	41.59558	42.4975	68.7035
82.12116421	90.69277047	84.85779629	76.28231034	105.51302
0	0	0.256576	0	0
0	0	0	0	0.540389
0.454002085	0.431552657	0.517127875	0.976741365	0.280378
7.51675	7.95985	42.8749	40.978	85.7811
0	0	2.889007178	5.105194277	2.621187232
67.9396179	83.696733	101.822063	79.209912	154.963
80.529386	76.02914911	91.42683916	73.26496792	66.29605
28.85997	28.87866	19.61006	20.21498	6.231080389
0	0	0	0	0
0	0	1.86425	3.92921	7.99872
38.99560884	39.64410531	46.02340332	50.9495597	56.0016
0.368207	0.186839	1.72229	2.79335	4.43167
93.40717	56.761096	132.4898	49.233	116.214
0	0	0	0	0
34.54681	36.26464	27.87986	26.87555	25.44014
0.11503199	0.05647742	0.985803685	1.5466961	0.662137
11.39610406	12.31494489	8.001741849	10.81410928	5.25272
19.51370682	16.57484827	13.241592	15.179636	14.7266
0.352884	0.232178	0.677013	0.461531	10.82539
34.07835	33.308665	38.267366	32.04471	59.713
45.36425634	41.027076	16.82816618	20.31371131	7.70323

1.24358913	0.916455205	3.276254372	4.60493866	1.85905
12.4617408	12.374757	9.087123525	10.61317	0.183484
7.571976631	7.85737308	11.29310586	11.13591829	24.50851
22.22492	24.15755	26.50291	25.55832	59.71880683
5.390580018	5.581349748	5.473311098	4.208613146	4.220030036
1.66153	1.67411	1.92671	2.97821	2.15833
0.1179518	0.175990126	0.4073379	0	12.31862213
0.1179518	0.175990126	0.4073379	0	12.31862213
44.84002	50.07433	40.87915	47.03043	111.0353
32.71093	36.92503	72.20909	71.37587	69.4545
29.760556	33.96035	40.95410001	37.95736	34.12190278
29.760556	33.96035	40.95410001	37.95736	34.12190278
0.130316	0	0	0	2.87728
42.30671498	36.76507133	10.65166	12.46037	0
42.30671498	36.76507133	10.65166	12.46037	2.00227
78.58540241	88.75665	93.59599754	84.85254808	42.6486
78.58540241	88.75665	93.59599754	84.85254808	52.1526
22.474728	23.31223549	21.55775502	20.744321	21.1954
777.061	791.275	107.9429	196.4799	7.49815
0.0774883	0	5.747420007	11.63331061	13.77874
0	0	0.204632	0	0
0	0	1.61382	1.68102	1.03126
0	0.156251	0.149597	0.304954	2.262096
0.868927	2.19E-07	0.362509	0.972859	9.90E-20
5.13E-08	2.40E-05	3.89E-11	5.38E-07	3.13E-05
83.8662	98.1337	57.2968	78.5207	87.8264
21.09671996	25.84180652	23.43864535	22.44915158	5.17E-05
29.12495	29.15527	27.61286	25.35841	55.4115447
0.00231043	3.25159431	4.51715775	7.78337	6.65E-26
39.69104722	45.3146835	41.47326326	40.086316	168.0480358
24.85883593	31.12508616	15.15620821	11.64591146	1.02554
26.040027	25.04718014	24.07451854	21.02921	46.79405
0.83824	0.222055	0.849399	0.914775	0
31.451427	31.827115	30.032972	29.5645894	40.5124957
31.451427	31.827115	30.032972	29.5645894	40.5124957
0	0	0	0	0
46.69000097	46.9457	33.68100002	35.6914	13.06405
0.184843	0.372119	0	0.485535	19.325813
0.0425894	0	0	0	16.6314
56.4050992	61.37808168	68.73675407	70.26635003	229.3944717
56.4050992	61.37808168	68.73675407	70.26635003	229.3944717
24.3631	24.386	29.1712	23.6755	37.6528
531.504	341.427	166.354	217.063	5.02766
0.0752068	0	0	0	0

14.0728	11.3869	10.805	10.1823	6.14126
23.64427423	24.828564	20.03112491	19.48161973	43.12577837
145.0239	142.3983	174.1609	147.8672	95.3832
11.322	12.6695	17.5343	12.9607	18.0866
71.18571393	78.52364736	53.41164089	38.49995059	119.6091238
71.18571393	78.52364736	53.41164089	38.49995059	119.6091238
85.2035	89.0892	94.8727	116.0517	106.3742
27.36098952	26.6291323	26.74924434	29.339108	39.64216
35.0024627	36.08812813	28.93649256	32.2084075	8.66301
58.971947	59.52344	63.612496	64.031681	15.42248
0.64538	1.67E-05	1.2236	1.1343	3.42974
8.269099665	7.11909488	8.404332	5.629522217	1.4048
500.608	573.704	2126.874	1480.778	80.7661
7.623114795	8.032781	8.95154	7.5311	14.45714
7.623114795	8.032781	8.95154	7.5311	14.45714
19.3014	24.3861	30.3114	24.7964	39.2035
11.8576	14.26443	11.68089	14.75576	35.54858397
1.578530015	1.410930042	2.724800013	3.526940002	0.480916
59.17791	71.37099	40.41853631	39.60157	199.9912473
13.93192	15.27885	16.70753	16.03932	4.785
0	0	0	0	0
42.81787919	40.91557	49.30625	55.30116742	97.384149
9.81625	9.42529	18.3334	13.77291	30.9327
0	0	0	0	0
90.45510479	81.63560063	89.84773858	84.85492172	189.0344
61.0785	75.3427	103.443	87.1897	141.45
0.157203	0	0.148173	0	0
0.635899295	0.856729	1.569717	0.55323913	0.603788
18.4256856	15.77156765	13.4083551	13.42574847	4.535054087
0	0.626668	1.14003	0.797078	0.778043
5.318847	2.421091	14.68726	7.357991	9.98799
0.2855695	0	1.779145	1.745371105	4.301988444
0.5421688	0.72476755	1.445737	1.934396	16.52878
0	0	0	0	0
39.121248	45.44714518	31.67780819	32.793779	9.18338
76.9428	79.0764	11.7139	14.8212	183.004
36.64873	40.62729	45.87254	40.45214	19.6738
36.64873	40.62729	45.87254	40.45214	69.95491228
2.807811424	3.796520705	5.386475022	4.338200001	34.74521195
0.22267	0.250407	0.0735971	0.0983104	0
0.85874723	0.59420149	0.62463039	0.964641408	0.299767
17.8789	13.1929	23.2898	19.2048	10.2291
14.9828	14.8748	18.0169	18.0394	5.28931
67.5149	66.64536	5.753108	13.77037249	3.57317

32.21085	33.17827	30.01329	28.338154	33.4885
83.08540701	89.208261	71.47175	62.353056	36.695419
83.08540701	89.208261	71.47175	62.353056	36.695419
67.27738208	67.9044	80.7454415	78.22693	59.502284
67.27738208	67.9044	80.7454415	78.22693	59.502284
57.2178	63.1467	31.2416	34.2097	69.2347
24.15986	24.97691	31.51657	31.81407	40.39402
0.633624559	0	8.63467	9.465536	6.88073
36.36242	41.3648	24.83053	23.79621	17.12469101
99.49481	112.83018	192.06004	141.03618	66.4627
12.64473	13.571235	12.173499	8.083299763	16.1535
355.9043619	236.5241067	53.68831457	62.78064677	15.38423
1.065710135	1.744376015	36.0672	83.8597	5.95E-05
1.065710135	1.744376015	36.0672	83.8597	5.95E-05
1.065710135	1.744376015	36.0672	83.8597	0.5844
1.065710135	1.744376015	36.0672	83.8597	75.1339
0	0	0.423903	0.288354	0.821038
0.158699	0.575081	4.55E-07	1.93E-07	8.37389
0.567424	0	47.6604	107.825	80.2964
0	0	0	0	0
1.1343	0.259288	47.2437	118.006	143.634
0.249733	0.0853608	1.42136	2.15697	1.3058
10.4865241	12.810225	12.065828	11.934602	17.564999
3.783381	21.976842	0.553144	2.610559	18.95227
15.67045	19.78944701	15.907894	20.90991	39.10731959
15.67045	19.78944701	15.907894	20.90991	39.10731959
53.6827544	60.82591428	83.27163828	92.13340445	51.90898347
8.317226464	8.14942563	11.29714403	10.77840002	14.04791128
0	0	0	0	0
8.7853	10.51815	12.40463	10.4295	6.10859
0.2323078	0.6943257	0.111334433	0.053744911	51.26775001
0.2323078	0.6943257	0.111334433	0.053744911	51.26775001
0.10206	0	0.197557	0.402986	0.123577
372.7536	389.6442	36.93986	159.5901	5.97876
4.53246	6.01192	7.44728	3.58274	69.1361
120.1301145	141.1173554	79.99070453	85.02142637	135.591
0	0	0	0	0
1.73446	2.22231	2.45444	2.95463	6.28847
32.40295213	32.62335252	22.84440286	25.498723	11.29641563
32.40295213	32.62335252	22.84440286	25.498723	11.29641563
82.54405832	83.3402439	66.70492	84.604142	26.03023
82.54405832	83.3402439	66.70492	84.604142	26.03023
18.41357	18.87262	24.47511	18.86671	45.7197
6.82432	5.183378	7.09684	6.00336	58.7295

12.9653	14.4991	18.9932	12.9368	41.5703
30.00077	28.611888	28.23532	29.80483	46.361948
274.2484679	313.2533993	499.6207691	495.2093516	633.0555
11.63	11.3809	15.1028	13.7384	35.2086
22.99315581	15.694985	33.86274	22.33971	50.043975
34.3281	33.5638	38.86980131	29.9588	54.087
22.99315581	15.694985	33.86274	22.33971	50.043975
9.278296	9.06295121	11.12454998	10.24135809	10.0453
28.089	35.7509	41.7757	31.8248	191.4481
7.21873	7.92647	13.1913	13.31508	10.216013
0.485518	0.0399345	0.1867947	0	0.93934
23.44265	26.52500779	19.7948402	19.68186769	16.3381
23.44265	26.52500779	19.7948402	19.68186769	10.5202
21.040493	22.18107147	19.80247688	18.01467935	29.7284
652.6281056	689.2377168	1031.880707	1003.082133	1180.320262
50.3453	51.6367	64.3591	40.765	90.3037
0.000346477	0.000164242	0.000145814	0.000488779	4.1317
0.140798	0.27828	0.268749	0.180904	561.788
2255.468	1318.533	12237.12	5319.45	21.8832
952.012	403.456	1766.64	915.365	6.59593
149.935	45.2358	412.122	160.571	0.316315
2.396369	3.014568	9.75652	5.79131	0.404391
16.46440585	15.29212	14.26423003	13.586596	4.586332
61.371817	22.927215	15.67796	19.51273	2.39717
148.3895	95.2777	143.2221	97.5152	6.22838
19.77758222	21.65744103	59.5338329	34.88662487	24.56586
5.523417	5.893556	5.213851	5.486176	1.53635
117.681	149.1031	380.534	393.331	319.96072
11.0905	9.98886	15.9594	12.6399	8.05504
2.2042778	3.691009095	4.562596375	3.37184	6.89129467
6.407680229	8.120685	9.8295538	9.34324	2.026197448
6.407680229	8.120685	9.8295538	9.34324	2.026197448
0	0	0	0	0
27.19489	30.8916	33.86912	23.18783048	92.8396
27.19489	30.8916	33.86912	23.18783048	12.833128
27.19489	30.8916	33.86912	23.18783048	33.15472
2.22378	2.69146	4.56365	3.56786	7.72002
139.619486	147.802784	149.168186	133.5441318	77.0029
0.186828	0	0	0	0
10.8459	12.0539	27.6495	38.6794	2.54224
21.7739018	20.98068861	21.348614	21.32507596	8.0029609
21.7739018	20.98068861	21.348614	21.32507596	8.0029609
12.679918	13.66687164	10.84248	11.73152074	8.392591729
9.759847326	11.88379995	9.149385	8.391203108	7.49126

18.05172133	22.777865	22.199699	17.75572442	3.518162
0.151811	0	0	0	3.82284
0	0	12.6095	26.0554	44.938
0	0	0	0	0
0.988886	0.252312	7.84544	14.2512	9.83202
2.81162457	3.121993168	4.929951	5.4214	17.113155
0.104619	0	0.153208	0	7.5478808
18.1119	19.64572	12.97452	14.96143	3.224106542
19.53134335	19.337239	14.07242254	15.27534707	14.92644344
9.88554	7.98496	9.48034	9.16069	37.56490346
0.246047	0.740391	0.914519	0.823645	0.166219
0.0197413	0	1.031833528	2.342977	0.890843085
0.0197413	0	1.031833528	2.342977	0.890843085
12.14275084	15.021318	5.274840661	5.176944045	39.7349
7.486036	7.309664	4.684036	5.838293	2.305433609
117.54306	132.04777	101.733355	58.57954263	23.90074
1.76E-09	1.05E-10	1.46E-08	3.00E-09	6.73E-07
34.25910254	33.62980333	23.8894	22.2307	4.090760002
0	0	0	0	0
1.660152377	3.187662224	4.000436421	3.5998732	78.4003
17.10209018	19.18386423	23.64561	18.70417021	63.846
19.6419	22.3501	23.8983	19.2426	50.68141029
30.3055	30.735	27.5067	26.4978	39.51
9.94181	8.807749	10.56176	10.701689	27.2957
0	0	0	0	0
19.85852314	15.2056985	25.49118135	15.706139	55.49790421
18.36917	19.89747	28.5689	26.97539	23.3983
26.27752	30.09568375	26.797901	28.5717488	33.2858
26.27752	30.09568375	26.797901	28.5717488	34.0159
38.49797	35.82624	46.37422	42.10703	45.6899
90.923237	117.382854	90.357841	104.647077	110.4500093
7.9243	8.51184	10.4489	9.60732	58.9946
16.4787	17.05212	26.31747	21.52177	68.4204
0.888919	1.33858	2.04473	1.13521	7.03298
2.110624	3.659119	3.271387	3.032733857	13.8225
0	6.37E-29	0.204509	5.48E-35	7.22E-06
0	6.37E-29	0.204509	5.48E-35	7.22E-06
7.8659	8.73577	13.59118	14.29073	22.6534
6.525362726	9.239755669	13.17031725	13.66126694	43.89067909
17.4284708	14.95902496	22.15641496	18.4563164	46.83754322
13.78685	11.35815269	27.3277	22.7443	3.13889
13.78685	11.35815269	27.3277	22.7443	15.8314
5.57448	2.036912	47.0509	53.0911	49.5866
30.421763	30.2510681	30.96513179	39.10595601	15.11600772

30.421763	30.2510681	30.96513179	39.10595601	15.11600772
0	0	0	0	0
0	0.242925	0.231512	0	0
103.4051276	96.96806153	115.20989	150.8665966	91.1885
13.53818702	15.52351402	18.0211248	20.41647602	88.5246
13.53818702	15.52351402	18.0211248	20.41647602	22.16241341
19.38076578	22.56383323	30.62812386	29.12733483	245.66289
19.38076578	22.56383323	30.62812386	29.12733483	245.66289
0	0	0	1.7419	1.91787
39.41376044	37.893307	36.91503	39.45858914	114.64485
128.3603974	130.8839572	149.2277593	117.5565727	45.1624
128.3603974	130.8839572	149.2277593	117.5565727	45.1624
128.3603974	130.8839572	149.2277593	117.5565727	45.1624
128.3603974	130.8839572	149.2277593	117.5565727	9.431862749
128.3603974	130.8839572	149.2277593	117.5565727	23.57054
128.3603974	130.8839572	149.2277593	117.5565727	54.610285
128.3603974	130.8839572	149.2277593	117.5565727	54.610285
128.3603974	130.8839572	149.2277593	117.5565727	54.610285
128.3603974	130.8839572	149.2277593	117.5565727	18.38885
128.3603974	130.8839572	149.2277593	117.5565727	25.50439
128.3603974	130.8839572	149.2277593	117.5565727	25.50439
128.3603974	130.8839572	149.2277593	117.5565727	13.21542942
128.3603974	130.8839572	149.2277593	117.5565727	13.21542942
128.3603974	130.8839572	149.2277593	117.5565727	12.36240851
128.3603974	130.8839572	149.2277593	117.5565727	15.42276456
128.3603974	130.8839572	149.2277593	117.5565727	23.14141849
128.3603974	130.8839572	149.2277593	117.5565727	23.14141849
128.3603974	130.8839572	149.2277593	117.5565727	23.14141849
128.3603974	130.8839572	149.2277593	117.5565727	21.34584269
128.3603974	130.8839572	149.2277593	117.5565727	21.34584269
128.3603974	130.8839572	149.2277593	117.5565727	21.34584269
128.3603974	130.8839572	149.2277593	117.5565727	21.34584269
128.3603974	130.8839572	149.2277593	117.5565727	21.34584269
128.3603974	130.8839572	149.2277593	117.5565727	4.07778
10.46134194	10.83928508	20.31923041	20.82527625	105.65389
18.20303041	17.16682	19.539812	24.378713	10.035592
4.384523608	6.38641	5.791406	4.084817866	5.2503765
66.1876	112.68246	195.5103	213.1004	26.0396
66.1876	112.68246	195.5103	213.1004	39.6607
22.65262	21.5127	13.085186	14.47438358	4.84283
22.65262	21.5127	13.085186	14.47438358	4.84283
22.65262	21.5127	13.085186	14.47438358	4.84283
19.6227	6.19896	50.3745	35.9526	106.227
0	0	0	0	10.86840832

97.6898	102.127	104.116	111.239	196.884
15.27900714	19.79491657	15.0167151	14.2277098	30.59483
15.27900714	19.79491657	15.0167151	14.2277098	30.59483
0	0	0	0	30.59483
19.70922	20.03253	23.09327	16.82574	87.0861
18.29818	14.25006	28.85557	24.32184	5.62612
18.29818	14.25006	28.85557	24.32184	7.5725
38.76379	42.81654	47.13767	38.56464	83.75030733
38.76379	42.81654	47.13767	38.56464	83.75030733
14.152557	15.88519	4.245821	7.15892	44.86649041
0.270760143	0.406047129	0.038104793	0	44.86649041
0	0	2.11404	4.89989	3.29334
0	0	0	0	0
23.76221	21.951183	27.85937	28.7290815	95.9161
7.82699	6.82112	13.7067	10.2237	3.72291
552.6119	547.0516	726.4491	660.3786	301.303
8.993778868	8.24657549	11.43143359	10.1045	41.5631
1.74E-05	5.41516	3.14619	3.27501	3.43231
29.45358	26.39556	29.50659	21.36565181	68.14614745
0	0	0	0	0
12.10741986	14.1480846	11.95501516	13.65231051	300.9017
0.038569469	0.155084682	0.60512239	1.389004741	13.0567
0	0	0	0	0.0925032
1.0495	0.887415	1.481993	1.973202	17.67006
13.554109	15.830336	14.794875	15.44306439	15.77607
13.554109	15.830336	14.794875	15.44306439	15.77607
13.554109	15.830336	14.794875	15.44306439	15.77607
14.38605937	14.41740595	28.09680486	28.57772619	39.132959
14.38605937	14.41740595	28.09680486	28.57772619	39.132959
8.41064	9.17992	22.4748	24.856	3.29002
15.02090289	13.68884151	10.9379929	12.3304721	6.50222
93.42285	86.98469	93.0102	87.43313	64.3047
11.48724859	12.13406578	10.25773037	12.26495887	19.13821029
23.12019969	22.34352516	30.35555044	31.29053328	95.58930452
11.58321129	8.662114003	6.973586898	7.414520404	0.593008
11.58321129	8.662114003	6.973586898	7.414520404	2.259762
0.682077532	1.19234317	0.429851	0.677075215	0.425821
0	0	0	0	0
83.742571	90.966961	92.13951	81.221455	38.92468
4.23359	3.75045	3.28685	2.78353	1.1466
83.742571	90.966961	92.13951	81.221455	49.673681
5.51374	4.07219	10.9682	13.4747	9.35354
6.2135134	6.37203695	5.680667511	4.181789	0.496851
8.596683352	11.33639856	12.62136884	10.73979823	0.862556995

2.547569	2.367647	2.725496	3.51669	0.825163274
22.39943271	22.38464929	15.15578614	17.55177559	10.10357
22.39943271	22.38464929	15.15578614	17.55177559	10.10357
22.32412	20.40228	24.16085	19.10531	75.4291
7.45096673	7.54611	5.80513	6.23725	3.23454
64.781748	112.4711624	306.8346387	307.0019627	109.10502
6.13729	6.236010982	13.63967258	14.4506	2.245474288
17.65185	14.19387	8.672847	12.472595	3.502033962
33.18756258	31.36654554	19.06003326	20.62552903	23.54098
13.8667	14.6381	4.24334	6.76965	0.269555
31.68525	31.54459	7.886556	13.08228	1.652066
17.37021	16.25945	12.56726	16.70247	30.46849
34.2714	37.9908	52.8414	45.6011	83.8245
7.93805	7.58777	14.909	12.951252	44.7687
3.93774	2.73011	6.86801	5.5321	16.175
2.49E-05	0.330539	0.526562	1.31495	1.78062
37.464053	46.36861	70.67482	62.232448	131.3039
21.2933	20.3058	17.4504	18.0491	44.552
27.2682	25.15895	25.42819	26.531161	8.288192182
1.589147557	0.421541456	1.624139789	1.179316983	0.383438
2.06146	2.05433	3.29632	2.09587	2.84985
33.25558	38.73883	30.83137	25.90006	48.349
0	0	0	0	0
0	0	0	0	0
59.9831	71.6636	61.0871	61.4887	106.9511
313.9974	246.506	122.0463	104.6728	8.1885
46.92373408	35.26980177	29.66052	32.25215248	6.435119
46.92373408	35.26980177	29.66052	32.25215248	6.435119
10.1745	9.34237	11.90770045	9.97920327	26.090904
0.457890983	0.781661126	0.205705	0.3456308	30.24570409
14.87772044	14.4000491	16.95889967	17.6048541	18.3249191
14.87772044	14.4000491	16.95889967	17.6048541	18.3249191
0	0	1.01744	0	1.27726
6.84944	6.43345	10.0708	8.93435	32.8175
0.00231043	3.25159431	4.51715775	7.78337	6.65E-26
0	0	0	0	6.65E-26
42.514555	28.48751149	54.34155	50.73215	26.454852
16.1317	18.0437	18.2427	13.9966	20.2515
42.514555	28.48751149	54.34155	50.73215	26.454852
15.78870567	18.162317	13.71030463	15.0618108	23.53437111
9.23526	11.14516	9.53386	6.10239	2.30813
12.5095	14.066	15.1419	15.1314	23.5365
15.15987	15.51056	15.08484971	17.27153	58.4611
28.30197649	24.81895274	25.1047443	27.68674688	34.843966

0.25930639	0.072434854	1.524648653	2.10903472	0.82803065
20.8805	28.2783	25.7837	24.7119	29.9121
0.306768	0	5.50238	15.7927	8.53306
0	0	0	0.209455	11.5393
14.02495	12.98397308	11.951538	12.68485989	4.19567
0	0	0.269937	0	0
13.43656481	12.6003069	4.745569296	5.052281882	26.18702
4.05302	4.05107	6.16938	7.08601	39.48638683
19.0474965	19.5104473	21.20539539	22.13629266	39.48638683
144.513035	153.1730625	150.0800252	137.2020612	126.2500133
206.445623	148.1236437	163.68642	205.2467465	159.0922
0.867986	0.551097	1.902576	1.057145053	0.844353
37.13426131	35.95110878	29.3525007	29.42156252	21.93708
37.13426131	35.95110878	29.3525007	29.42156252	21.93708
15.59710027	20.10643174	26.69029919	18.24221018	5.073969
15.59710027	20.10643174	26.69029919	18.24221018	1.535699187
15.59710027	20.10643174	26.69029919	18.24221018	20.5632189
0.55643	0.323878	1.508239	1.05938	6.74083
3.016544872	2.493702788	10.85382911	14.14960411	6.86933
19.51696	24.99681	31.19993	31.98584	87.8245
28.7418	16.2239	37.7246	33.2839	47.1643
0	0	0	0	0
17.78900017	16.42245	13.27487	13.62737	35.00170001
48.676719	51.718012	31.328115	35.90429	36.31065
1.96789	1.27165	0.891453	1.97088	0.236012
69.57292	56.945702	43.10839809	45.6175709	348.918
0.275591	0.364632	0.384471	0.596618	12.1072
9.276035055	10.5573338	7.44145281	8.877924267	9.720100298
9.276035055	10.5573338	7.44145281	8.877924267	9.720100298
0	0	0	0	0
0	0	0	0	0
0	0	0	0	0
0.733543	0	4.743638	8.98071	5.93636
13.4764	13.476123	21.959117	16.850538	61.57859
9.388960383	11.18032616	15.85139634	11.56525565	61.4743
36.662572	40.24986	36.53641	32.704131	65.12807
225.0100063	207.6049059	65.3281059	80.463036	4.5388
0	0	0	0	3.38605
0	0	0	0	0
2.731624	3.24205	2.079288	2.508810415	2.489353516
0	0	0	0	0
0	0	2.19751	3.074479	1.913061
5.746156	4.03033	10.99723	12.14229	4.11197
54.47534	69.03014	96.3473	94.4934	170.2587

8.696117898	8.375428972	6.846626111	7.12613154	5.449230321
80.30706	73.32981	27.573023	28.87772	20.17302
26.90177	28.76377	21.259121	23.09082585	28.30377
8.89E-07	1.92E-05	5.37E-06	9.66E-14	56.66640372
3.44306078	4.766491	8.020368	6.933444013	12.66232527
3.44306078	4.766491	8.020368	6.933444013	12.66232527
25.01869	22.44412	31.0423	33.6568	63.2022
2.741294	2.69004	2.740344	2.31706	108.9066
69.485	76.8278	67.2596	65.997	127.241
5.83456961	5.081870006	7.834179224	9.38414002	2.650913225
5.83456961	5.081870006	7.834179224	9.38414002	2.650913225
0.362773	0.181999	0.702184	1.17181	5.40075
122.8774	99.5372	106.235176	74.077405	1.257941
122.8774	99.5372	106.235176	74.077405	58.721181
122.8774	99.5372	106.235176	74.077405	58.721181
241.179	292.614	217.577	142.86	532.79
2.05883	0.513317	0	0	0
7.60901	6.378957393	5.955571	6.276166	4.321965726
198.0445	156.2812	86.1485	89.807	7.403
21.181387	25.850331	20.744004	19.978779	35.48114
21.25151	17.78266	22.78017	20.02055	34.9862
17.7263966	18.19318841	29.4661483	23.1912126	95.3091
8.92366	8.36699	14.14311	11.623009	15.5007
8.92366	8.36699	14.14311	11.623009	21.187442
8.92366	8.36699	14.14311	11.623009	21.187442
0.126756	0.253722	0.122775	0.821448	64.4264
0.9720978	0.952766993	2.713055075	5.085685	224.11497
33.7090775	31.433869	25.7564333	26.26592536	9.115100872
7.393626749	8.763416144	5.197992827	4.942987492	1.775932334
0.615864	0.282445333	10.47847	16.86793	8.26907
0.302856	0.262155	0.0230136	0.495589	0.000709409
0	0.333984	0	0.0884214	43.84468
5.633652	5.969928	6.88224	8.62048	2.931723
44.6412	48.6191	45.1939	37.5138	61.5827
25.4171	24.7323	9.6907	6.42777	20.7935
0.0964842	0	1.802322399	4.39055851	4.671390482
1.095010418	1.278446	2.307762	2.435714	6.78039
1.095010418	1.278446	2.307762	2.435714	1.72712
11.92739	5.84842	11.26102	11.73832	4.571379441
72.283702	69.33802246	43.80530186	51.0331419	29.80429
72.283702	69.33802246	43.80530186	51.0331419	29.80429
5.72842	6.127517	7.7839	7.06454	5.229868197
28.92400357	29.11726981	30.47604632	34.43832975	76.70701391
4.77228215	3.50936789	1.913776138	2.969211095	0.840745503

4.77228215	3.50936789	1.913776138	2.969211095	0.840745503
69.78280703	62.60586	39.01834236	43.173103	0
69.78280703	62.60586	39.01834236	43.173103	9.37609
69.78280703	62.60586	39.01834236	43.173103	20.0721
12.8907	11.6552	16.9442	13.493	52.2739
0	0	2.419041688	3.489794704	1.14155
0.677661	0.831829	0.787254	0.893159	0.842811
0.280402	0.269943	1.78809	4.1154	230.137
0	0	0	0	0
27.5433	26.1574	18.8086	20.1015	5.88514
31.99642	32.50641	45.49742	45.4243	112.79905
44.34773044	37.43660284	56.63999131	41.96646345	65.1133
41.604289	31.50031	16.44271	13.27341	1.8553802
41.604289	31.50031	16.44271	13.27341	1.8553802
41.604289	31.50031	16.44271	13.27341	3.007454
5.35055	5.90874	3.67433	4.836453	15.6868
1.862766	1.811829	0.444074	0.256244	100.8536756
0.189688068	0.028561929	1.122243093	3.015429733	0.999880011
0.189688068	0.028561929	1.122243093	3.015429733	0.999880011
89.7585	86.9829	142.0745	155.2626	112.8908
0	0	0	0	0
0	0	0	0	0
1.15946	0.169417	12.5352	21.4548	17.0618
46.04481	50.06864202	36.87797	50.48412	47.23385
1031.23	729.98	346.772	464.317	7.87171
446.4516	922.932	3.685046	4.44398	5.66601
1667.09	1132.8	515.446	577.52	7.33938
0.242989	0	0	0	1.37116
0	0	0	0	0
0.354395	0.0363985	0.214301	0.0802773	2.57431
29.41876	27.77932	20.31662	22.76293	5.99048415
0	0	0	0	0
67.35722605	87.859391	56.11091779	52.93766668	69.9329
4.4953	4.60977	4.56371	4.74319	19.08962
29.231646	26.647085	31.220548	34.161273	14.2224
27.4349	15.7143	54.853	50.9292	766.166
7.53023	7.519484	10.78334605	8.98459	34.87341492
63.47326	55.8135	111.48474	106.15338	143.5639
165.5201	148.50655	189.10681	154.32956	163.829
8.41435	8.51707	14.559	11.65324	47.63452
25.45877573	27.31182157	25.03326961	26.5073236	14.32667
0	0	0.166049	0.0557668	0.0502919
185.1513431	170.9600586	160.129538	159.0911263	54.3381
4.19294	4.14242	6.08469	4.75419	24.8151

0	0	0	0	1.10E-06
72.1820055	69.0274122	74.1524767	79.658654	90.04361436
72.1820055	69.0274122	74.1524767	79.658654	90.04361436
0.174507	1.22083	0.488131	0.672902	1.21124
50.16397	45.56911	29.48663	36.32822	9.824921688
49.75866	45.23064	7.167098	13.637867	1.814741722
8.719123284	7.822003147	18.449291	30.22432089	8.91501
1.066354863	0.28329259	12.697722	24.90063288	47.53383412
1.066354863	0.28329259	12.697722	24.90063288	47.53383412
0	0	2.24376	0	0
2.90645	3.64566	2.80087	4.07592	3.30345
16.34979	14.134474	26.693244	24.29379	53.13671992
10.643862	6.10629	19.9246	23.432486	0
74.92796378	73.4401451	88.81735995	108.8084819	37.5264
74.92796378	73.4401451	88.81735995	108.8084819	20.6999
21.7674	14.8554	20.8632	24.0916	5.08158
37.97402679	41.967015	62.01171	58.20625457	84.4321
37.97402679	41.967015	62.01171	58.20625457	84.4321
2157.442154	2123.849374	2438.8554	1907.006309	310.0011453
193.1318034	134.840595	143.0820612	107.508929	15.3007
193.1318034	134.840595	143.0820612	107.508929	8.13127
31.883	25.5368	8.90761	6.56411	0.848537
193.1318034	134.840595	143.0820612	107.508929	19.857287
193.1318034	134.840595	143.0820612	107.508929	0
42.9021	35.7055	100.862	81.167	69.951
4.95E-05	1.18E-05	2.09E-05	8.23E-05	2.51E-05
15.3382019	14.528736	24.154004	34.125111	32.077594
15.3382019	14.528736	24.154004	34.125111	32.077594
15.3382019	14.528736	24.154004	34.125111	32.077594
6.08089927	6.081561849	11.37205767	9.08513783	39.85217219
6.08089927	6.081561849	11.37205767	9.08513783	39.85217219
500.7625552	561.4801836	736.0191063	737.811232	721.744
8.50762	6.5321	15.00832	21.75911	38.0392
66.95424	79.98467	74.17326	75.87934	20.3704
66.95424	79.98467	74.17326	75.87934	143.5742
12.16606	13.54434	17.40179	13.59526	58.4127
321.33951	409.5667	382.5407579	292.7671825	601.2354714
271.5539	287.7534	261.6164	236.9225	323.6301905
271.5539	287.7534	261.6164	236.9225	27.81402553
53.12782738	51.36803271	28.48732975	34.77959812	20.89818614
53.12782738	51.36803271	28.48732975	34.77959812	20.89818614
59.54445	58.988729	46.587737	45.58727	23.32484794
1.24314	0.189416	1.15946	0.982420794	0.171978
0.385952	0.387662809	0	0	31.86538133

0.725799273	0.608053	0.362681	0.879376	0.263274
0.400066753	0.728845603	0.800459905	0.142999499	0.0439758
0.670247961	1.228467633	1.415683	3.21197	0.373547
0.620807	0.000421124	0.546717	3.19035	2.8492
62.54013495	65.53091	62.2268076	66.0945124	76.40638908
62.54013495	65.53091	62.2268076	66.0945124	76.40638908
62.54013495	65.53091	62.2268076	66.0945124	76.40638908
39.65804	37.45646	40.615215	45.078657	75.54164887
27.552277	30.08866812	36.495241	31.87069001	62.61211
107.589331	127.4585738	106.7494454	109.841738	41.4653
64.8033	50.76371	10.75116	12.083254	0.615222
18.60951	18.31802088	13.56594628	14.76124552	4.471011837
0.544943	1.90155	3.07019	1.0512	0.962014
0	0	0	0	0
33.57607591	29.01764868	25.49292792	25.20780141	3.75819
33.57607591	29.01764868	25.49292792	25.20780141	3.75819
83.711629	80.04355	65.61155	64.54094	19.73476
0.17999898	0.834606581	0.316797545	0.09437379	0
20.64202781	19.68215	14.81974578	14.55166	4.611892
4.373147	6.1255687	14.68221985	15.09800695	2.59528
0.171325	0.174788	0	0.151784	0.282304
26.01742	29.59797	34.73211	26.76028	74.2426
0	0	0	0	2.248765
0	0	0	0	0
0.279896267	0	3.345300558	7.696077146	4.16168
0.279896267	0	3.345300558	7.696077146	4.16168
0.286748438	0.088888856	0.637608896	1.184567018	0.979954638
17.15943011	13.62428554	44.90333211	31.78520388	5.194593
0	0	0	0	0
1.855980387	1.724049757	1.68931105	1.055724312	0.597839
18.7702	12.7292	10.1532	0.00277984	0.653111
646.023	710.722	1051.692	1013.111	1015.956
2.096358	2.732695017	2.192258976	2.138991265	2.676811315
20.53963568	22.38778535	19.392888	17.86962898	7.928934
0	0	0	0	0
226.31326	238.6464	179.94403	193.46836	56.11654
226.31326	238.6464	179.94403	193.46836	56.11654
1.02280259	1.1066772	2.119541	1.828215477	1.041028098
1.02280259	1.1066772	2.119541	1.828215477	1.041028098
8.99628	10.99215	12.1547	10.53207	43.16686
6.475980654	7.409770953	6.222453	6.66570219	1.59077
1.326100203	1.114591111	0.109661	0.292918	0
24.5767	25.2103	23.4387	20.8453	40.88040376
83.22731273	63.5289702	28.4699083	35.5204108	0.605652

83.22731273	63.5289702	28.4699083	35.5204108	2.14447
120.4709	124.9947	129.4058	125.1302	94.6072
6.016843	5.3421175	8.130111694	9.134189745	28.37800456
74.65936	71.01707	28.41713	34.40916	47.50980835
35.60192291	41.585201	17.63284038	18.95988781	3.261195492
35.60192291	41.585201	17.63284038	18.95988781	3.261195492
3.71568	4.7005	9.09846	8.19045	33.4223
24.45021895	23.75539	15.48638119	17.35234424	7.42918
30.2084	20.064	34.9045	37.0287	2.40E-22
36.91323623	37.3409	33.40769	33.51294852	34.29331761
8.0433	9.23724	2.59373	4.3868	5.082536023
3.28755	2.5600778	2.9506409	2.2899538	0.707264
14.487478	13.206451	10.283916	9.137386	7.383702175
14.487478	13.206451	10.283916	9.137386	7.383702175
14.487478	13.206451	10.283916	9.137386	1.68666186
5.118357	6.617836	17.229607	26.57421	26.172
5.118357	6.617836	17.229607	26.57421	37.42611452
0	0	0	0	0
2.14053	1.21529	8.56269	10.6307	9.50776
2.366135	3.86178095	6.487355	5.329939	6.82419
2.366135	3.86178095	6.487355	5.329939	5.10861
0	0	0.372924	0	0
19.56594904	22.519251	22.49034953	20.4454046	17.74748
1162.455	757.511	390.8349	469.682	16.8792
0.377299	0	0	0.483403	0.45913
17.63662	15.88132	21.0117	22.23943	53.8009
11.24889547	9.140204425	21.87121819	26.01566648	10.90109726
11.24889547	9.140204425	21.87121819	26.01566648	10.90109726
9.18594162	6.18515506	49.73905	31.32590273	21.9835
9.18594162	6.18515506	49.73905	31.32590273	21.9835
13.426666	14.259829	12.17315553	11.20087406	3.736004683
2.612740687	1.305494633	4.237774905	4.7123025	1.953785819
2.612740687	1.305494633	4.237774905	4.7123025	1.953785819
0.000496294	4.13802	2.88639	2.69639	0.000686105
0	0	0	0	0
24.24887	21.28242678	14.04497	14.95548	2.699114302
4.666181	5.177799	4.655864	3.377872	0.199069
360.92	279.296	156.631	243.815	18.5713
110.7515374	121.739066	98.616201	121.0401142	25.92550824
110.7515374	121.739066	98.616201	121.0401142	323.14
326.2477	351.3632	358.203	275.8171	377.508
120.0031	106.6034	111.4575	122.218	64.128668
277.375	296.8354	21.51414	49.68673	5.33145
11.61056	12.32655	11.73181	10.90855	59.51681417

37.0734349	36.214705	19.962563	19.9214146	2.58141
4.86252	4.38237	9.06813	9.19249	3.87146
1.260855	2.548178856	0.51603968	0.564219812	189.25206
8.90234	4.9656502	5.554416	8.221176	1.295108666
8.61092	9.12172	6.114444	6.844829	8.19153
10.27517162	32.35305247	57.086113	29.05664154	36.21
15.33251	11.91994	8.73709737	16.231	8.425394
0	0.525016	0	0	1.89471
0.137193	0	7.925123	8.6678923	19.61517
0.997788	0.702656732	0.0976184	0.358484	1.262688
26.96999571	25.03912969	47.96060089	61.19075462	77.47753
4.45747	1.48114	9.97646	5.38434	11.8947023
37.78736359	41.80532019	30.22112819	28.78027112	50.322
37.78736359	41.80532019	30.22112819	28.78027112	50.322
37.78736359	41.80532019	30.22112819	28.78027112	50.322
33.74761	32.46074	31.481177	33.630821	32.00431471
8.25127	9.36593	7.30686	7.81205	10.0705
29.42773	28.62567	21.47872	20.068337	10.051593
48.25095871	46.75538785	38.7575653	43.8306601	41.1404
83.55052149	76.33736499	56.79400244	57.02458742	3.798403056
83.55052149	76.33736499	56.79400244	57.02458742	3.798403056
83.55052149	76.33736499	56.79400244	57.02458742	14.63742
83.55052149	76.33736499	56.79400244	57.02458742	14.63742
11.78495	10.16563	6.871579	8.41095	1.94379
23.2212	23.3519	19.4531	17.6136	26.0129
11.7543959	10.88583523	11.4760086	12.441875	3.819295102
11.7543959	10.88583523	11.4760086	12.441875	0.493992
0.983333914	0.925893	0.418451	1.04003	0.0709375
2.01237	1.46559	3.79431	4.02736	2.66544
113.383	91.3922	100.56	127.172	4.88507
43.20722203	40.8186604	22.2536574	24.67211985	16.59105021
43.20722203	40.8186604	22.2536574	24.67211985	16.59105021
2.2849	2.32018	2.51878	3.97438	2.2205
5.87897	8.025240806	5.863390008	5.918770223	0
188.295	194.414	236.526	182.278	105.0849
71.637959	73.715424	59.63200001	62.9913227	107.7206654
71.637959	73.715424	59.63200001	62.9913227	107.7206654
8.63856373	6.80845479	6.201483965	8.402409386	5.770171
0.107728	0.192145	0.310971	0.166858	0.0768415
11.9992886	12.8020633	10.75496833	8.88394141	55.8264
57.34881988	54.19592795	11.222161	17.07850511	1.155466
12.25244	10.67385	18.86364	14.72378	19.05139
0	0	10.6186	15.633	30.05961
0	0	0.457809	0.618931	0.407593

0.17119512	0.1913134	0.553811114	1.044535011	3.072581848
52.5831	57.1949	137.94	157.589	64.0072
201.1459187	209.4260059	15.20721851	35.09180343	102.2708103
33.6164141	35.4008073	33.391759	39.655236	47.7276
3.4917	3.28006	1.4362	1.860910002	208.632
3.44251	4.58546	7.61284	4.42492	7.04912
6.55102	6.74871	4.79989	4.08787	33.77087
6.684169811	7.52991172	9.288281809	8.444828826	19.97394
0	0.234168	0	0	0.271274
4.49043	5.668290529	4.344775022	5.985176781	2.14403
79.23985439	93.83296985	72.33966457	63.64504	60.25267338
102.721	111.406	51.51224731	66.57755243	11.9129
3.665076	3.62658067	3.469265239	4.208136666	2.34474
3.67E-13	1.66481	0.00032141	3.00E-15	0.0084603
10.1805	13.39	11.7362	9.49216	18.3583
3.68295	3.62713	3.7802	3.60693	13.55088
0.105201	0.106514	0.156405	0.278269	0
56.9069	54.50170011	42.4669	41.0742	26.86781
0.692313	0.771999	0.0681242	0.457669	38.67287
112.33355	114.5	76.48606	75.42454	45.71277
49.252526	52.33577	51.3488	46.586568	61.2938
18.16738401	18.8101209	17.37808837	18.26318512	58.6438
18.16738401	18.8101209	17.37808837	18.26318512	58.6438
0	0	0	0	0
0	0.672099	0	0	0
0	0	0	0	0
0	1.29938	0	0	0
11.3147	9.90214	1.48722	2.05974	1.700650039
18.9778932	17.79479702	15.3769323	13.08094571	16.40475
2.54E-24	1.04E-29	1.57E-74	4.66E-25	2.538590124
0	0	0	0	0
0	0	0	3.38E-06	3.42E-06
0	0.245517	0	0.320454	1.9171
66.16750447	55.3186206	63.52325116	53.71491515	51.8495
4.210706	4.253187423	6.522839654	6.156249997	31.9756
0	0	0.878266	2.00484	4.9848
8.23012	14.97365	6.28926	18.85301	41.33688
968.931	1039.529	1657.156	1793.989	1606.401
72.1837874	72.79017158	97.14068	96.857769	31.34021093
20.42	22.2936	10.7039	8.39704	0.506555
20.34952	14.71486	9.62079	12.4935	4.55973
22.501387	29.08902515	53.06023333	78.59606188	55.39529
22.501387	29.08902515	53.06023333	78.59606188	55.39529
22.501387	29.08902515	53.06023333	78.59606188	8.00077

21.70813	28.6805	29.0703	23.95647	78.4781
35.96877328	45.05896982	62.99156	51.3946274	165.4451
7.203776559	5.81587	2.0094	2.40307	1.20664
8.506271912	6.262158454	4.12534123	3.043170649	1.139386726
7.82E-06	0.479833	2.3212	4.79E-05	1.32436
10.55925	11.06893293	8.118622083	9.073570456	1.90689
4.16917	4.99879	7.56715	7.71442	19.2324
10.55925	11.06893293	8.118622083	9.073570456	2.60864
1.47345	0	0	0	0
1.42895	0.636297	0.559038	0.753382	0.465497
6.955679	6.344910937	11.86416	9.18357	27.29731
16.79346	16.270959	15.17429	18.73264	43.77620868
16.79346	16.270959	15.17429	18.73264	43.77620868
48.11151891	57.15690092	38.7004	37.8455	40.30364793
64.8564	46.52404	160.6551	156.1771	121.1596
27.98309447	33.587117	34.98356232	33.187176	30.35014316
27.98309447	33.587117	34.98356232	33.187176	2.90094
29.44641783	27.68442647	19.13141566	18.80211657	3.28519
0.3700194	0.071016107	2.305345	4.461190344	2.191965439
0.3700194	0.071016107	2.305345	4.461190344	2.191965439
5.058822	6.483609	2.709857	2.70184003	1.09224
2661.2	1851.348	320.798	321.555	13.8585
37.63223	38.97194	47.5871	45.21867	51.07659
13.582766	10.034747	3.088336	4.99164	0.399119
1.337013	1.218873	0.163415	0	161.3016
1.9630431	1.912011	3.313906903	2.468607669	1.87517
20.60381	18.29460402	31.8597	25.81740613	58.19252861
15.21984	16.404572	17.076464	17.669551	23.24989
45.77732286	49.94571	48.44372601	54.18773161	44.48962186
8.5469	10.1655	7.20348	5.92364	3.30587
501.03338	469.1877	391.10938	281.06903	53.37811
21.85034	25.23060001	20.636321	20.802079	22.3542296
8.14668	8.59767	12.4581	12.1111	33.0819
2.13E-08	1.25E-07	5.74E-07	1.11E-07	0
1.284309688	5.805594682	0	1.035396424	248.932
0.34446552	1.14215282	0.16568604	0.180829147	337.4171341
18.98407	15.22125	12.93531	11.04674	4.22772
2.810701	2.54244	1.86052	1.799780235	1.30902
3.37187	1.76642	4.25505	4.778	1.80395
24.76131062	27.2663095	32.33895769	32.99879223	43.44107734
0	0	0	0	0
24.76131062	27.2663095	32.33895769	32.99879223	43.44107734
14.09939579	12.57320072	13.13643703	11.54907007	36.80500144
14.09939579	12.57320072	13.13643703	11.54907007	3.39769

546.2654	523.00751	734.13286	586.2003	302.09938
9.18806	7.72402	9.91224	9.19375	10.62177
0	0.44565	0	0	0
66.76337297	71.275492	76.854153	66.69169003	0.665809
66.76337297	71.275492	76.854153	66.69169003	115.51775
66.76337297	71.275492	76.854153	66.69169003	115.51775
27.25646	23.47223	26.46959	27.5319	69.21623
0	0.220832	0.413166	0	5.72489
0	0.151297	0	0	2.62201
0.77652	0.54834686	0.392564762	0.33898694	4.92311
4.84855	5.26309946	7.72640393	7.564745	8.88565
0.0379958	0	0	0	6.75552
25.189999	23.000093	46.899153	43.62447	26.2099
4.428802	4.690709	8.41264	5.26898	21.95775
32.99451	34.7431	94.5812	67.8813	54.68791693
3.43406	3.74453	2.24294	2.43269	12.07037386
5.24492	4.93994	8.64345	8.32761	12.07037386
0.680756	0	23.1405	53.3779	49.4878
0.259478	0	20.0012	46.8169	71.0409
18.97868	19.218284	17.92020324	17.318729	21.32009
51.8831	58.1006	42.59538	41.53048	109.9780372
6.849998	5.65504	5.85474	8.02091	9.50564
31.20191573	25.96669228	18.74860687	22.1149977	39.67105
12.50560329	12.06063421	9.899620023	11.00851367	1.76461
12.50560329	12.06063421	9.899620023	11.00851367	3.334447
119.7892	114.6852	86.8403	102.5268	8.767767
203.01792	189.40893	234.64814	197.66886	86.3997
65.23097171	70.97011522	70.51542656	73.64558849	64.75650992
65.23097171	70.97011522	70.51542656	73.64558849	64.75650992
53.5036925	50.41286186	46.6106076	46.83454376	75.83161553
11.179256	9.73891	13.81328	13.83974	12.95627
74.89329	71.82622645	58.24153	59.21286	70.6558
30.271	32.3104	39.5561	29.4828	63.1711
50.45268	56.01746	37.36551	40.72124	52.01580719
0.412259	0	0	0	0
2.531557154	2.993246	1.676685094	2.355348351	44.10428276
13.86978	17.5323	21.17761	20.15798	3.72059
5.10862	5.385963	7.774395	6.847547	20.56648
5.10862	5.385963	7.774395	6.847547	20.56648
7.32808	9.29463	11.69508	10.0368379	33.8752
24.6964606	24.49201822	25.79507901	27.16559439	4.26199
24.6964606	24.49201822	25.79507901	27.16559439	24.34262065
24.6964606	24.49201822	25.79507901	27.16559439	2.19835
161.542	174.521	47.1197	65.6653	13.6009

0	0	0.492512	0	0
77.0657	70.3463	53.4175	50.9096	14.315
0.132338346	0.072760042	0.811490214	2.166364192	0.836709518
3.240873	4.76221	8.27244	8.2861	23.4526
1057.136	1026.162	497.6102	662.849	302.72
120.279	113.195	79.7334	76.599	19.59880001
39.39322552	36.55088	73.82665	68.17871	94.7616
22.8834	22.0282	17.6759	15.9551	6.39118
2.656640401	4.019217003	4.315690994	5.451218051	0.524158
1.147546	1.163919	1.4108163	1.428232229	0.612474
0.741487	0.250132	0	1.29494	0.282947
66.26884471	53.786872	36.74492733	43.48170081	6.789018
30.115989	36.49124	25.35951	25.1828	11.1601
30.115989	36.49124	25.35951	25.1828	45.2824
3.81084	4.12336	6.77965	5.12397	25.0262
82.410091	79.7780754	118.997097	110.8444645	217.0117
82.410091	79.7780754	118.997097	110.8444645	19.6356
82.410091	79.7780754	118.997097	110.8444645	0.0688023
82.410091	79.7780754	118.997097	110.8444645	26.5917
41.3536	45.49959	29.33557	32.83291	28.84646
41.3536	45.49959	29.33557	32.83291	1.19987
54.07583127	42.66806145	41.28195969	42.07027775	14.574892
0	0	0	0	0
0	0	0	0	0
0	0	0	0.427493	0.0968084
0.0747513	0	0.0756407	0	0
0.138537	0	0	0	0
0.611885275	0.420923066	0.407113159	1.015927081	0.102394
5.084417	9.772242	7.222938	7.033113237	0.491102
0.205378	0.0346009	0.204072	0.271657	0.207335
0	0	0	0.116824	0
0.358018	0	0.791302	1.41434	0.41597
0	0	0	0	0
0.727293	0.784699	0.862222	0.4720733	0.429647
46.657618	37.1480731	39.94902	56.3971	6.382672557
46.657618	37.1480731	39.94902	56.3971	6.382672557
15.61485444	17.85040093	21.38013119	18.26525346	39.168964
7.049739443	5.759527021	6.004486574	11.66382727	19.4024
0.0493001	0.033582	0.213421	0.834833	0
9.795862389	9.807163806	12.476408	11.058841	24.8657
11.51524	8.627374	13.849027	16.941502	39.7709
11.51524	8.627374	13.849027	16.941502	1.49536
0.0717278	2.42E-06	0.285862	0.194927	9.38658
0	0	0	0	1.57614

0	0	0	0	0.365622
0	0	0	0	1.098783491
3.170587	3.617297	3.499622	3.0571235	118.49385
45.67946	47.76089	45.2235	40.706471	40.20543791
45.67946	47.76089	45.2235	40.706471	9.22901
11.53505101	15.15788	18.56237	15.482613	50.29904582
0.802646	0.40572	2.51257	3.381736532	1.2419199
0.802646	0.40572	2.51257	3.381736532	1.2419199
0.0858905	0.276824893	0.66928763	1.3588435	0.942264
13.80671939	15.27904665	6.933697363	7.472622252	3.002030985
61.929068	57.31546	90.44904	82.15309209	90.9721
10.46184	17.32583	8.187244	12.83136311	5.8528
10.46184	17.32583	8.187244	12.83136311	2.20737
19.8566	20.3628	24.4204	27.0151	3.89825
10.92689789	12.71515576	18.72309218	20.96077	90.9721
224.4417548	202.3048545	231.4375023	210.43454	72.79781
3.931189	3.445118109	6.455517	5.264169	15.399139
87.495	98.03	101.808	88.4884	109.539
187.9379022	140.0759568	109.7023806	133.2427493	3.06406
110.6047565	107.52972	79.45628	81.03134336	22.74635
1.86724	0.413679	14.87923	35.0984	16.08592
0	0	0	0	0
0	0	0.228366	0	0
140.9020056	137.1690077	124.1810024	115.3090039	55.1579
89.988	90.7356	108.2843	120.133	96.3329
19.50872	22.9132	22.7022	22.07866	36.70764
21.15179	23.24662	16.93309	16.3975	28.99992
12.82103	12.34544	11.33565	12.55273	24.48088
13.96497696	14.85203742	13.96365897	14.14055488	13.16745501
24.30014044	24.73089022	16.6129564	13.87391009	26.33867
2.68237	1.72423	9.77237	14.6851	6.56273
37.34864	38.91357	31.77017	28.68368	30.3413
27.84990271	27.60220093	40.89730107	43.63690143	3.64809
0.142582	0	2.60211	4.6184	1.05434
0.154586	0.0778844	0.915178	1.62164	1.15675
0	0	0	0	0
0	0	0	0	0
9.27571548	10.30082404	14.20953662	9.666384015	30.43806
15.420721	14.71872	16.17062	13.42122	24.33760101
0.590204	0.199979	0.577123	0.388155	0.480716
19.85771007	18.06039023	20.333619	20.81713508	8.119051566
94.607	88.0608	100.4118	88.9217	88.6422
14.33773919	12.71746867	11.54872589	11.65522649	18.57442014
1.198331279	0.931249268	1.493411497	1.324964411	0.0625355

4.454358609	5.107397	6.648604845	8.69699	4.03108
6.41979	5.051759111	8.578943372	9.631984	1.75911
0	0	0	0.655133	0
0	0	0	0	0
38.6371	44.6671	34.01108	35.05121	44.8687
52.4685	53.3685	37.9303	35.0073	12.72964
36.2715	34.2507	28.4842	30.9809	83.5357
3.57E-05	1.55698	1.22515	1.43491	2.92E-06
1.099631	2.226803	2.610675	2.019875	1.62061225
15.13396	16.62851	19.51741	17.34821	24.4201
21.45081	24.81094923	50.60852765	46.83930117	22.98930679
102.4566991	87.48271692	24.99132081	31.98509851	2.831527
102.4566991	87.48271692	24.99132081	31.98509851	2.831527
0.292613	0	0.176547	0.5559	0.0694526
27.88955104	25.82553549	19.92820016	24.24781608	2.51542
27.88955104	25.82553549	19.92820016	24.24781608	9.728597087
47.4984	47.3648	41.0306	33.8201	49.5032
6.66931	4.8444	8.44359	7.74157	4.736911114
0.965361	0.912377	1.74967	1.2028033	1.17536
17.15873568	18.008632	34.66600524	30.2067199	21.87646
1.69243	0.572504	3.83847	2.2214	2.14577
0.278517	0.839831	0.268643	0	0
0.275812	0.3327574	0.900082716	0.606840459	0.230958
0.520582	0.634312	0.601536997	1.144759437	0.668363668
1.872873	2.078400276	5.101381	3.494444	1.99720147
81.32592516	72.98153978	110.4724063	100.6936756	40.775
81.32592516	72.98153978	110.4724063	100.6936756	45.9003
81.32592516	72.98153978	110.4724063	100.6936756	33.564252
9.6032	9.38027	9.99575	9.25592	28.72463
0.38679	2.44E-39	0.607307	0.682175	1.06431
30.468865	26.61196786	46.65851028	39.81716879	18.42371371
30.468865	26.61196786	46.65851028	39.81716879	2.4167
30.468865	26.61196786	46.65851028	39.81716879	18.63632
155.1052899	148.3190509	104.1380587	100.2300488	44.84795
15.47504	10.3222	54.40621	59.10545	27.1473104
5.60965	7.54961	5.16206	9.00004	14.23829
5.887985	4.668831614	2.553063	2.44416	2.151541985
5.887985	4.668831614	2.553063	2.44416	2.151541985
20.56999	14.9993	70.4353	59.3271	27.6898
1.84125	1.68373	1.96341	1.77287	0.000477734
4.3993859	4.675712	6.410034183	5.203059934	16.0624
10.70956386	11.15466634	7.776797498	8.751028321	5.289542646
0.627365545	1.291947	0	0.276077065	156.9359025
0.310022	0	0	0	0.703719

1.256286495	1.969720876	3.215770578	2.090625346	5.83950196
17.20537	16.99601749	45.66744	31.23512	56.960851
0	0	0	0	0
20.3253403	19.43000706	13.58120483	15.43999611	3.248155
6.94219	4.79395	0.803383	2.40943	0
0	0	0	0	0
18.1753	13.8513	30.9259	23.1374	19.9534
4.093332899	4.44065	2.72646707	7.15398	10.1589
1.979431	10.292732	2.51674	4.0207271	766.5893139
59.8512241	51.22482	106.63025	91.48268	94.78125
59.8512241	51.22482	106.63025	91.48268	94.78125
0	0.0407872	0.0803633	0.0538029	0.0481478
0	0	0	0	0
184.4019855	225.4758596	286.8933498	271.2956968	398.5862813
0	0	0	0	3.73037
9.05879771	8.217806864	10.882015	10.99318627	6.22122
28.29036184	36.982967	16.97545129	17.78563107	8.297557
160.8334805	148.1852027	126.3998597	146.68235	31.64145
160.8334805	148.1852027	126.3998597	146.68235	31.64145
46.43687914	45.20430091	49.54184374	48.53067883	42.27285
0	0	0	0	0
0	0	0.0280438	0	0
46.242009	45.487758	31.535152	32.346537	0.332538
46.242009	45.487758	31.535152	32.346537	45.82937
46.242009	45.487758	31.535152	32.346537	0.126142
46.242009	45.487758	31.535152	32.346537	45.82937
13.314152	14.447089	17.70474843	17.75773061	46.24447
0.151515244	0	0.312633293	0.500114	0
0.151515244	0	0.312633293	0.500114	0
91.48431	75.76074	4.585403678	19.53087	2.229030557
8.197981299	6.610743729	9.21638914	8.079983902	18.027281
1705.1137	1929.9695	3146.8173	3124.4502	1701.08
17.848811	14.58625	32.99223	36.79161	86.49504
2.952123	3.353186653	4.16314331	4.092242	27.92804
8.41467	8.1319	42.4316	69.3097	1492.76
56.27889576	56.670505	65.965102	44.84454	106.2824
0.484905	0.237764	0	0	0
9.743250002	8.879160007	7.96354	8.84302	46.16620005
9.743250002	8.879160007	7.96354	8.84302	46.16620005
55.48220014	50.66108753	64.49098	65.98832004	26.1533
3.79207	4.37853	5.28385	6.44191	63.46320167
55.48220014	50.66108753	64.49098	65.98832004	63.46320167
822.9286	939.0078	1389.4538	1265.3763	1160.300875
5.67796	5.92093	9.947054	7.89833	66.528

0	0.0388729	0	0	1.88699
3.95213	6.78848	4.81796	3.13288	1.06552
0.736715	0.2332203	0.498819	0.424532	1.04454
11.46500716	9.530009607	17.08438727	13.4272792	3.47E-05
11.46500716	9.530009607	17.08438727	13.4272792	48.21426
0	0	0	0	0
0.000113436	3.18E-05	8.99E-05	3.57E-05	7.66E-05
124.141682	112.771549	125.769643	108.744161	79.6881
124.141682	112.771549	125.769643	108.744161	25.43729
124.141682	112.771549	125.769643	108.744161	34.6103
0.854654	0.145993	0.846158	1.5296	1.866072289
0.403954	0.275335181	1.469012151	1.37457	1.866072289
0.085191689	0.125131	0.3244161	0.4027814	0.925317
0.144916	0.0981027	0.669123	0.770721	0.93151
20.89784	17.88144	23.38419	18.138315	26.86868298
39.1222	32.42338	47.1228	37.96629	52.9283
3.567969	3.67426	7.22999	3.451661141	11.7426
23.83884	22.01368	18.49728	20.73066	24.61611
14.60664437	14.42001592	17.92221927	14.45631875	44.43594
14.60664437	14.42001592	17.92221927	14.45631875	10.72910032
14.60664437	14.42001592	17.92221927	14.45631875	10.72910032
12.8868	11.568	17.7325	13.5599	33.4539
7.27663	7.90707	8.05131	7.58516	17.7888
53.93588	55.42082	62.75665	59.47169	150.6139
53.93588	55.42082	62.75665	59.47169	150.6139
123.4595038	130.2841821	135.1341082	139.5600147	48.88923554
123.4595038	130.2841821	135.1341082	139.5600147	48.88923554
25.01773	28.99245	27.52486	24.51309	36.2436
11.52806067	15.61919408	14.47543	15.38273065	14.04768
24.77055	24.89609314	27.580852	26.384829	11.087279
27.317123	24.75267	24.89132	24.84877	17.65476
0.235214004	0.092296062	1.394461657	3.18892419	2.209756
51.8785	48.5938	32.48947	35.8674	64.8466
51.8785	48.5938	32.48947	35.8674	28.8456
28.52973001	19.18592322	18.92046	25.2709	5.87972
28.52973001	19.18592322	18.92046	25.2709	12.3796
26.48322	31.51473	28.9252	29.1371	76.0853
0.625458	0.639532	0	0	0
16.36067888	15.86397641	24.47309758	17.61284483	18.7586
16.36067888	15.86397641	24.47309758	17.61284483	22.35610586
8.975583743	9.792120531	14.989287	15.50344195	43.255439
1.43253	0.423322	7.0089	11.0348	3.9058
11.088822	15.781124	7.76671	8.229812	0.954337
11.088822	15.781124	7.76671	8.229812	0.954337

11.088822	15.781124	7.76671	8.229812	1.262045277
1.84102	2.03048	4.44836	3.35041	2.1314
24.40283052	22.54859085	17.84650802	19.13143246	5.525590654
10.7265	11.3982	13.8729	14.6128	28.09341
15.75882536	14.750178	24.578378	25.44338001	17.71229
21.619449	20.92147	31.16638	21.97183	30.1025
21.619449	20.92147	31.16638	21.97183	32.46906994
0	0.0494646	0.194922	0.713439	0.289612
0	0	0	0	0
8.74093	9.951961	17.54808275	14.87070425	17.10367
2.107096364	2.6044007	2.259423586	2.3839	4.629923
15.98211778	9.667478	23.93612027	22.60740653	161.9036677
15.98211778	9.667478	23.93612027	22.60740653	161.9036677
43.41460607	37.86793892	42.904192	53.542621	18.2753
43.41460607	37.86793892	42.904192	53.542621	30.4784
0	0	0	0	0
38.52193193	37.738325	32.67181539	31.68285296	45.45927
43.1033	37.685196	27.736599	26.52995	11.33082
43.1033	37.685196	27.736599	26.52995	2.39745
43.1033	37.685196	27.736599	26.52995	2.563
0	1.19591	0	0.511433	0
83.78814242	88.55636368	66.30386982	67.97042512	56.866052
0	0.453967	0	1.16885	0
7.033778344	4.36366929	13.03526169	13.07455142	17.96723
7.033778344	4.36366929	13.03526169	13.07455142	17.96723
30.19074	37.1401	45.8573	41.2467	28.0889
1.347327457	1.727142	1.552844105	1.113199997	2.08026
4.76E-06	1.95E-05	8.80E-06	0.979783	0
2.10335	1.961147	1.665539	4.18334	4.008584539
17.96017	16.5545	15.17174	13.35302	34.9015
0	0.0913322	0	0	1.18879
16.34737	19.2731	19.90119	14.93577	41.13861874
0	0.254541	0.40924	0.110905	0.592854
4.500671	1.148138768	1.752614	2.710035086	0.276938
0	0	0	0	0
0	0.144545	0	0.0945195	0
0.0925992	0	0	0	0
0.243479	0.190727	0.0796993	0.283444	0.06556
0	0	0.0801146	0.0538272	0
0	0	0.0635045	0.0853495	0
0.189276	0.0766787	0.222023	0	0.0911257
0.0395214	0.039917	0.0386986	0	0
0	0.0372027	0.108813	0.145796	0.13467
10.03592	10.8247	13.18095	14.11117	34.56309

11.0813	9.85625	7.44645	6.31973	2.08613
14.445016	14.966628	29.240441	26.32329902	60.83980347
6.79221	9.33223	5.3725	6.87201	34.7541
34.19776	42.59086	49.08647	41.94536	48.36172
32.092651	27.8397	19.911907	22.0044	9.26221
24.40283052	22.54859085	17.84650802	19.13143246	0
67.317672	59.98997459	39.603962	46.71330866	13.19556
28.625832	24.495611	19.191046	21.33060171	8.252196054
0.163873	0	0	0	0
2.605366357	2.726808228	2.0256542	1.962757936	0.45349
13.42227102	14.0478812	9.62710025	8.6895028	5.256837
3.544245594	2.352646	3.968482	3.539125	4.684
18.17008	26.1489	36.1885	33.9329	43.2409
13.16700409	11.66170215	7.771251207	7.441837494	0.232439
59.5778	46.5315	56.0014	53.7121	77.7953
0	0.193639	0	0	0
0.0971843	0.193845	0.185953	0.375753	0
19.541574	21.35339	17.05443419	19.27799	12.73955
10.1352	11.30207	10.1089	10.04735	13.1513
2.41614	3.07218	1.12491	0.888571	99.8363
114.6341938	114.2933044	177.1064	168.9375144	142.863
5.39405	3.78397	7.97148	6.42794	23.23138
21.95149	24.94677	27.88523	25.04387	41.8747
3.44699	3.98846	8.74375	10.35582	5.807813637
8.778290002	8.015412081	7.9739303	10.599196	43.2339
104.7600804	100.49949	76.28436107	91.70857664	4.71834
104.7600804	100.49949	76.28436107	91.70857664	4.71834
104.7600804	100.49949	76.28436107	91.70857664	4.46786
104.7600804	100.49949	76.28436107	91.70857664	37.9953
40.894043	39.85723783	37.96377723	43.63377828	62.0069
40.894043	39.85723783	37.96377723	43.63377828	69.64514038
6.68486	6.65568	1.40499	3.00906	0.93596
0	0	0	0	0
30.77051	25.09004	34.49743	37.60381	19.97119331
22.67266741	26.50705001	35.05828203	33.05564	112.5973
22.67266741	26.50705001	35.05828203	33.05564	112.5973
236.295	219.715	197.575	171.961	112.678
69.67509998	91.15371764	93.0871676	86.74383472	112.305
18.55677159	19.69338919	25.005427	30.45263059	37.7675
84.65649326	82.08981735	55.26275707	65.15781	36.41612
0.564626	0.326646	0.553879	0.284902	0.0220422
42.86364542	50.39665	49.95228404	49.94309385	36.81336052
6.552362528	6.648156	10.886881	12.0309021	20.13335
6.552362528	6.648156	10.886881	12.0309021	20.13335

12.5356	12.431	11.794	11.8961	27.1946
28.03436641	28.87125443	30.51356565	34.3433116	41.90851524
26.578	19.51462	8.96237	13.23344	3.519702027
26.578	19.51462	8.96237	13.23344	3.519702027
21.2175699	25.694404	25.574459	21.82321923	0.675821
21.2175699	25.694404	25.574459	21.82321923	44.2072
8.20974	5.76867	51.24635	76.9475	82.73254
14.1312	11.17918	37.5294	45.3163	35.580144
29.86012	27.30923271	22.60018	20.78422726	5.238421072
0	2.28E-07	0	0	0
0.308211	2.05969	0.198912	0.253582	6.16E-05
0	0	3.61387	4.89719	12.6841
0.30571	0.441059	0.515104	0.569663	0
3.34E-06	1.16E-05	8.13E-06	6.29E-06	0.116908
27.57105	34.42462	21.47618	22.325	43.75121
25.38979	19.89381942	26.51148448	21.80003389	16.22751
11.761209	21.61952	17.08397	27.31801	4.455678
84.692	81.131	85.2357	78.4271	1474.06
3.72494	2.00774	2.43747	3.39033	22.6639
8.62279	7.87933	10.69314	11.03857	4.95126
43.1952	39.7745	33.0042	30.0201	14.241
0.304137	0.0617489	0.660631	0.403589	0.151024
8.487882168	9.861584151	14.0704841	12.75969312	18.3426
32.23810231	28.06940275	20.81050211	24.31480278	11.53394339
29.34900447	33.02761303	28.6717071	26.90430986	34.28500665
9.40E-06	0.618822	7.06E-06	1.71E-15	6.39E-05
0	0	0	0	0
47.030515	46.03764	5.025648	9.76740532	0.877105
0.722952	0.737515	2.82812	3.83106	0.467785
0.669838	1.35783	2.90138	1.74651	2.85202
9.069029784	10.170373	10.24518172	9.972785621	0.263023
9.069029784	10.170373	10.24518172	9.972785621	16.835767
76.348285	79.3947567	113.4785406	97.87084	143.74616
76.348285	79.3947567	113.4785406	97.87084	280.9779
0	0.123179688	2.94687	5.50533	10.0717
0.0591463	0	3.45263	10.642	10.2464
0.285327	0.349228	0.111992	0.153122	0.0675498
0	0	1.68674	4.65243	14.3107
0	0	2.67112	7.24308	19.5737
0.115862	0	2.12708	2.79054	7.56467
16.469848	17.56690391	19.41926316	18.48712736	45.10151
9.23043	8.46721	11.371547	11.1511	28.2724
0.490249	0.679205	0.259024	0.276313	125.1185
210.72267	201.432218	192.64616	187.44433	114.6095

4.887180097	5.110914009	3.383684007	2.029106	1.821309634
4.887180097	5.110914009	3.383684007	2.029106	1.821309634
3.364321	4.181681	5.84699	4.807752653	0.867457229
2.42819	1.75931	0.183	0.247533	0.368578
2.44354	2.52593	1.27201	0.457034	0
1.61929	1.4648	0.316081	0.437198	0
0	0	0	0	0
0.112619	0	0	0	0
4.5184	3.91886	1.14941	1.14191	0
2.5047	1.49375	0.393869	0.269958	0
2.20497	1.87698	0.396514	0.864098	0
31.52281239	33.25583	14.63529364	16.28831897	3.281389966
0	0	0.0743165	0.204388	0.099926
9.130006	5.296844334	9.0177621	9.384894	20.62410414
9.130006	5.296844334	9.0177621	9.384894	20.62410414
9.130006	5.296844334	9.0177621	9.384894	20.62410414
0	0	0	0	39.46828
21.88204247	25.74306099	17.91813002	19.42665461	39.46828
21.88204247	25.74306099	17.91813002	19.42665461	39.46828
43.17702062	44.61215103	23.58833557	26.33463077	11.59319
19.27751	20.26954	20.95705	17.90476	33.07778
9.40116	8.66816	11.68918	9.24923	22.7324
4.391500482	4.801603859	5.410773094	6.3140874	350.143
0.111459224	0.252639667	1.6116781	2.077977486	4.73641
88.30750436	81.85050295	124.443486	75.89211	239.3993
6.13845794	5.29379	9.32346	9.652860449	13.58796482
101.2744	106.9086	156.8941	125.171	150.362
17.2817	16.9765	17.1284	13.287	36.05391071
27.0997	24.4234	19.2988	25.3361	36.05391071
19.2697	22.8748	30.1807	29.7053	94.0931
3.4875352	3.8945162	6.10774	4.228822134	2.12858
14.265378	13.64102055	22.60680874	18.76878945	8.405410255
0	0	0	0	0
2.16101496	1.028333224	6.139099181	5.149926802	0.233277
2.16101496	1.028333224	6.139099181	5.149926802	0
17.46704	19.42777	13.56186	8.71748	1.19904
17.46704	19.42777	13.56186	8.71748	14.9888
1.94609	1.12699	8.5425	15.4634	8.78165
13.653274	16.46384958	10.881364	11.14842437	0.342854
13.653274	16.46384958	10.881364	11.14842437	2.130304134
16.75073	20.68333	33.6987	11.93693	31.3249
14.97106667	12.87591822	29.30383024	22.63104408	9.067914857
10.09343901	10.16982702	14.41044274	13.65166793	4.93286365
1.076257003	0.852858	3.53716614	2.776085716	21.314897

1.076257003	0.852858	3.53716614	2.776085716	21.314897
1.076257003	0.852858	3.53716614	2.776085716	1.9951
3.4159	2.68333	0.361292	0.987138	0.451586
78.10582989	72.8372279	48.14589966	51.3261062	0
78.10582989	72.8372279	48.14589966	51.3261062	60.1363
28.924	33.1572	26.9344	25.7287	46.2614
45.8185	52.30893	49.18858	42.85397	20.23673
45.8185	52.30893	49.18858	42.85397	43.7468
0.199263	4.56E-29	3.06807	10.28	21.4301
16.86918536	13.06280595	19.27894	16.774668	82.70546535
45.38003277	46.87354901	43.48146183	39.59105212	62.61042346
20.24114826	33.7884837	23.86912526	18.61397387	3.8488
1.976271545	2.474383362	3.950578958	3.304812055	127.7372
19.03628	20.30166	23.99152	20.02261	42.76763
48.85657	51.40573	15.162394	18.322533	1.620154317
6.80472	7.37087	17.80036	15.57248	13.19455
28.65065372	24.24775551	16.81067481	14.69453736	10.23215
28.65065372	24.24775551	16.81067481	14.69453736	10.23215
26.1326193	23.91103418	18.272531	17.484089	5.602442146
0	0	0	0	0
0	0	0	0	0
0	0	0	0	0
0	0	0	0	0
623.5183797	569.3211556	803.8272272	685.8744579	474.918
5.360430756	6.040665304	3.672233154	3.709634489	2.0821379
0.512121827	0.041034308	1.47065051	4.1782535	2.114096
0.510224399	0.215753634	1.184923184	1.881082	0.419982
39.63591	43.00559	53.69059	59.37389	47.31566
39.63591	43.00559	53.69059	59.37389	47.31566
0	0	0	0	0
0	0	0.337847	0.467373	0
11.3047	20.7647	17.58486	12.75943	36.5778
0	0	0.616023	1.1171	0
35.64515	37.483749	44.42875	43.26225	91.996374
35.64515	37.483749	44.42875	43.26225	91.996374
2.210841	1.464582	5.383693	8.408030548	5.75756
1.928693687	2.17513045	3.33452172	5.73744249	5.857609239
7.451094	9.902409	12.06838	10.791434	30.83887
111.9352549	85.63555876	26.68273121	34.49199642	2.7843
0	0	0.252772	0.22727	0
0	0	0.362344	0.327921	0
0.283742	0.5677163	0.742790075	0.739488245	0
47.4788	44.5048	26.3333	25.3034	1.49927
0	0	0.149516	0	0.393378

42.1548	41.5106	32.1542	30.7554	25.43321
68.532061	67.56817084	73.86368647	72.24791349	78.5154
20.1392	18.9156	22.5835	22.51118	58.3062
0.820763876	2.89219643	1.444934	1.406782	7.110861149
0.583804342	0.56065	3.001614349	2.180464047	2.89822
142.7378964	151.9240218	90.26922	105.8728	546.1371996
13.31771927	13.67316801	13.93451261	11.55975509	27.4245
13.31771927	13.67316801	13.93451261	11.55975509	9.95891
4.77095	3.53339	5.07541	4.81853	1.80111
1.633213	0.8965494	0.998762	0.855720421	0.240617
12.75449973	13.76545	13.54423	14.02157	50.5938
0.88799	1.01918	1.63528	0.880126	0.752842
1.982949092	2.840659	3.598035	3.729441751	1.91779418
0.252381	0.0668083	9.07E-07	0.0885252	0.000702631
0.391915	0.779234	1.27936	0.69304	1.39771
0	0	0	0	0
52.00589417	38.96720661	121.8304129	102.3077849	8.489451986
52.00589417	38.96720661	121.8304129	102.3077849	8.489451986
11.95567	9.876972	18.8401664	14.05425306	26.46558255
0	0	5.33E-06	9.83E-10	0.000166595
0.0554776	0.396283	2.78983	6.66944	2.48149
0.855089	1.4119	0.980519	1.16507	31.716
3.29373	1.95345	5.47058	5.64713	1.23437
31.87046	30.33736281	21.90658	23.02533006	7.544453661
0	0	0	0	0
86.6933	101.368	117.97	97.3263	72.0231
24.0271	23.5817	29.3195	31.8597	39.2924
0.585627977	0.620246	0.87854	1.020053	2.239684946
12.90699171	13.15096321	26.90694	27.5977	2.925173137
13.745313	13.58465	14.63369	12.414943	27.25710271
123.182554	121.231054	115.5420459	106.5790179	106.025
13.19097	17.010008	30.83698	21.2012971	49.834635
13.19097	17.010008	30.83698	21.2012971	49.834635
59.79045762	68.5487676	36.5449051	31.271566	17.8308
59.79045762	68.5487676	36.5449051	31.271566	17.8308
11.6495	12.219	21.9837	18.6388	11.3324
0.0703858	0.285047	1.4536	1.96196	2.44131
104.89952	103.00797	85.67471434	71.622575	31.13015
59.2243	41.69343	17.13186	23.00245	1.87907
10.61716031	9.6396008	13.599708	10.97900596	30.24879
10.61716031	9.6396008	13.599708	10.97900596	30.24879
37.08771231	28.63020946	22.22058	27.38454489	6.999751066
0.0980516	0	0.582967	2.61456	0.237996
2.22218	1.68378	0.508162	0	1.80203

1.469762	1.896326	1.212998	2.002775	1.45209
0	0	0.427411	4.41135	2.58398
0.052774854	0.1913878	0.664890156	2.240416313	2.128205574
89.99587174	88.3057352	47.26687279	49.36124301	12.809905
89.99587174	88.3057352	47.26687279	49.36124301	12.809905
0	0	0	0	0
0	0	0	0	0
0	0.0743069	0	0.097755	0
0.136267	0	0.0665819	0	0
0.0746395	0.0760128	0.150542	0	0
0	0	0	0.161812	1.45896
3.910061	4.95419	8.01376	9.63462	22.344491
53.265637	44.439034	23.93162	34.6856797	29.02469
53.265637	44.439034	23.93162	34.6856797	29.02469
1.8127158	1.191218	3.038897	2.879306485	18.1231
17.3312	15.6117	16.6675	16.0305	39.786
7.62630661	8.021496138	10.85679531	9.36158863	41.96922
2.468658331	2.777454092	4.027746	4.0510988	7.56276
19.29964	20.68684	19.04969	17.16934	86.3564
22.25227345	17.8274	36.3668	53.91028	182.6531
18.24453	15.47672	31.12990346	46.36958214	43.5827
18.24453	15.47672	31.12990346	46.36958214	17.93287127
68.347626	77.78638	7.05705	9.937913453	2.346185399
1.196053387	1.199602022	0.96675611	1.126109	3.780937954
4.863166898	3.88042178	7.190240763	6.279304875	1.778267321
0	0.2164	0	0	0
0.033599	0	0.0337319	0.181937	0
21.904	20.8358	11.7191	12.813	3.83413
5.083135	7.655514117	7.315394041	9.511732	49.0593
207.08666	225.72802	246.88432	239.4287	399.119
0	0	0	0	0
50.25054224	67.39268364	60.40036858	48.26335286	174.1291898
4.35E-05	1.83E-05	1.17E-05	1.60E-05	16.4062
58.7517	58.1037	66.11692	76.66325	137.189
31.60736	25.28294	46.17335	36.247143	11.90610182
32.8631087	33.759518	28.0347714	30.9728496	24.5824
120.795	99.4575	63.4531	77.7076	3.17043
241.50247	275.3969106	248.4566227	179.15492	25.96131
124.2861784	117.6982047	87.33569511	88.83894742	13.80479939
23.8912	24.06653	30.6674	22.78818	50.46916653
6.35681	4.26412	5.45187	6.27528	20.9136
2.249599077	2.068874	4.350125	3.172506	32.18031
9.0363	3.068945	22.55761	11.67468	0
1.12421	0	12.0096	16.1341	9.13983

2596.636297	1446.15526	5977.726945	3279.167173	16.164
0.150381881	0.328828	0.1192103	0.778962	2.5285
10.35630001	10.50805897	9.694509	7.354050436	3.82151
7.594186792	8.767755	10.98959	9.641107607	26.35377
10.000927	11.192435	14.989304	16.83284073	58.05607
0.443035403	0.561661165	0.046100131	0.181265145	112.871
0.443035403	0.561661165	0.046100131	0.181265145	249.891
12.95613	14.15744	19.27599	19.47743	46.3528
10.135704	10.4047	17.16617	12.61833388	29.3175
62.12125	48.9103	57.087071	57.13619	82.4288
12.54927	14.342207	14.88821	15.44145	179.36991
15.17319	15.39554459	20.20863918	25.67154036	21.9473
0.187931	1.587786	0.431884	0.582085	8.472644573
12.116772	14.19178753	17.38285595	13.828164	28.8556
12.116772	14.19178753	17.38285595	13.828164	1.92095
10.62345	8.751501	6.172967	4.65901	0.352467
10.62345	8.751501	6.172967	4.65901	0.630243
6.543976095	6.361388618	7.340357247	6.791439113	36.1568
0	0	0.0339876	0	0.82579
0	0	0	0	0
12.27367192	9.925792421	14.509742	14.742869	3.70609
24.61651917	28.80804404	19.493084	14.18029552	35.7841
7.911729	7.895320594	13.79447513	10.8931934	23.1827
0.118988	0.11821	0.116149	0.155189	0.44625
18.67847	17.04696	16.35983	16.16604	39.99290001
4.9403114	5.04732	2.888595	3.10384	1.713341222
5.09E-132	0.0734997	3.57048	4.97E-11	2.05E-05
0	0	0	0	0
43.6155	45.200155	41.711345	42.953481	112.03942
45.522378	49.14770229	43.22489166	51.17549	76.57303126
45.522378	49.14770229	43.22489166	51.17549	76.57303126
0	0	1.813839334	3.847964391	1.220698975
0.549834602	0.628162872	0.045390005	0.102454283	197.5003
0.0584824	0	1.282883122	3.552209	1.732862193
0.0584824	0	1.282883122	3.552209	1.732862193
0.547288093	0.073778669	2.444415002	4.38574	1.502165367
25.07464598	13.9577017	16.80160023	24.27728	5.89648
19.23802	22.17754	23.59625	16.97821	58.56326
19.23802	22.17754	23.59625	16.97821	58.56326
86.13385741	78.42346381	121.640939	103.090334	267.1771
86.13385741	78.42346381	121.640939	103.090334	267.1771
10.38047505	10.971269	13.08820674	10.79386326	58.75437
198.5125948	190.1205551	174.4660048	142.75679	64.7494
0.48226	0.315235	0.457635722	0.0498099	2.46778

0	0	0.0995154	0	1.21729
16.44400883	11.69699889	18.5288215	22.84545486	4.901780107
188.519622	157.3590101	88.83214166	95.90962669	8.636008158
188.519622	157.3590101	88.83214166	95.90962669	8.636008158
188.519622	157.3590101	88.83214166	95.90962669	8.636008158
0	0	0	0	0
29.120023	28.69204445	23.703927	26.00009116	9.573044822
0.0849161	0	1.1852	1.36616	1.85197
2.732873873	3.175810638	3.82024593	4.490637834	17.67899939
2.732873873	3.175810638	3.82024593	4.490637834	17.67899939
0.489433629	0.250269091	1.46193046	0.833544663	5.0151916
22.85833	28.8864	30.5502	28.3247	66.73159
0.120515	2.21008	14.0588	1.17854	0.000770298
3.161811962	6.16204	7.7135929	6.135700059	19.53243155
3.161811962	6.16204	7.7135929	6.135700059	19.53243155
7.34509	8.56407	12.92739	10.21284442	19.90398
7.74482	11.0142	15.1423	17.253	44.5951
41.95071755	37.83760748	20.51925916	19.19839752	30.315
0.295532677	0.2678863	0.1704039	0.38992	25.15896
52.3912	50.88905339	61.215317	58.53485065	41.53785
6.841982	7.454285	10.388922	8.5705	43.62190301
145.959042	150.6522	132.5751923	132.2470241	136.0527
145.959042	150.6522	132.5751923	132.2470241	136.0527
0	0	0	0	1.61998
29.25593	33.5322	38.3798	35.8321	121.5672
44.252657	37.803038	25.306925	25.859131	72.0756
44.252657	37.803038	25.306925	25.859131	12.85972
2.34706	2.27980291	1.35178241	2.860081	77.0048
2.469410484	2.877581135	0.678959345	0.796717036	47.6849253
31.06706	32.0637567	28.91543	29.5207368	48.98233516
15.7685	15.1785	22.8069	21.5843	30.5888
0.0679335	0	0.06738	0	0.0820544
0.861656	0	0.278503	0.380713	0
0	0	3.1374	0	0
0	0	0	0	0
2.243778671	1.871795138	1.4475282	1.784054205	0.894480079
31.52034	48.8145	36.1811	27.06162	43.47474
31.52034	48.8145	36.1811	27.06162	43.47474
0.00321083	0.000315039	0.00688642	0.000893072	0.000556412
18.18506432	16.029638	16.51859	16.09600357	6.75913
36.0722	31.3031	74.9869	46.4611	16.34501015
67.92717	74.3895	70.12823	75.77443	23.35616
0.066805	0.132494	0.127303	0.256229	4.66056006
0.000600411	1.27E-05	0	0	0

7.610563	9.46969	9.80306	9.60199	2.900253754
64.108169	79.927191	113.873769	97.792649	88.44614
64.108169	79.927191	113.873769	97.792649	88.44614
2.026175	2.199461	1.894947	1.988512	221.541
589.0840145	473.0724253	183.4662963	191.4886303	8.75929
0.5461531	0.721364654	0.523879823	0.417308601	1.235790535
114.57251	100.95073	133.73444	91.25654	0.658863351
114.57251	100.95073	133.73444	91.25654	75.7725
9.614254	9.394792	16.25739	11.38207	26.83625
61.4407	57.42521	63.81433	60.13951	41.214899
720.7739503	491.1271302	84.50485026	99.69148036	0.0371127
720.7739503	491.1271302	84.50485026	99.69148036	6.281080606
382.116	319.777	68.2617	76.4609	6.44634
5.04369	3.2571	0.289505	0.636833	2.269270465
5.96615	4.69891	18.4772	17.893	7.08628
1.12E-10	0.0514253	0.277224002	0.346679	2.26E-05
27.44698414	27.11103	38.95232591	34.86413	31.8973
13.36293	13.05047	15.28617	12.12786	39.65464
12.56989	7.600374	20.66381	18.57362	4.45854
12.56989	7.600374	20.66381	18.57362	2.52945
12.45883	10.164085	14.74776	16.859424	26.3704
3.431727	6.11901	0.521464	0.442175	682.076
40.50512	38.78445	37.75388	38.39437	20.92444
20.36223	21.44145	19.50537	17.624153	6.530927403
9.24716	10.6503	12.29724	14.25954	23.53798241
0.256783575	0.0920397	0.126448314	0	0
0.256783575	0.0920397	0.126448314	0	0.453263
69.60641375	74.757853	67.21868839	70.91427171	15.77431
34.852107	36.58813816	35.55387938	35.02440395	48.119662
21.79296	15.6662653	58.72305986	43.05784002	6.303364561
0.946354	0.000952119	0.548326	0.00644456	1.03658
113.0215003	95.65743046	158.8522919	155.7241135	82.18303422
113.0215003	95.65743046	158.8522919	155.7241135	145.4741173
68.417386	63.48459	72.65127	61.76916	44.9771
1.85792	1.3273433	1.668501773	0.325122	0
22.4245281	19.96589677	14.72785245	17.458067	3.6906
11.5962	10.926808	9.20425872	7.650249	2.61723663
16.60871	16.6113	17.53558	17.57892	78.0420117
11.3012	10.75346	18.5346	13.86747	18.6886
138.1601441	172.9667362	84.39772527	75.94033863	234.4250462
0	0	0	0	0
44.0399	45.4773	51.5716	41.5758	36.1815
15.045056	16.525879	15.031598	16.566604	29.10031
3.9412204	3.778670508	17.41312201	24.89882076	16.80248519

0	0.00982579	1.77E-06	6.48E-05	16.80248519
136.05709	152.3994	244.32765	195.38256	52.2645
1.95699	0	0	0.561692	0.515244
25.17533726	24.58608458	21.48890741	25.68529554	10.5536
0.80521	0.3803278	0.366465	0.334460091	0
24.32460517	26.86943648	29.05177938	24.34662565	6.664431073
0	0	3.2849	0	0
29.02463799	28.60452	175.605	139.1511	80.03256006
3.876685859	4.213488	2.570728106	4.02178536	1.36092
136.9305	131.8586051	152.469001	128.6318	72.6413
45.9065	45.0297	24.6234	25.5455	48.1829
6.86831	7.80654	6.16616	5.07542	15.1523
18.37329	19.03911	16.04011	18.48213	5.221600181
96.43488093	112.30492	110.06644	103.36015	43.70461
96.43488093	112.30492	110.06644	103.36015	43.70461
60.446879	51.58688042	62.09829926	56.6650061	11.65492
4.761736	4.344551592	2.47840859	3.688498557	0.874435
11.15858771	8.95548574	28.30512	28.25586	15.184949
5.33518066	4.773280187	7.821940137	18.78618	25.98491801
5.33518066	4.773280187	7.821940137	18.78618	2.98069
0.673909	0.331199	1.197451	2.0961731	2.99228
5.488285159	4.731353943	4.117023982	5.935829653	2.447628
10.485918	10.02783716	14.1810097	15.55609092	24.624159
10.485918	10.02783716	14.1810097	15.55609092	24.624159
0.145757	0	0	0	0
0	0	0	0	0
9.006702649	8.416477481	13.00978109	12.08190991	27.42655416
32.70677006	38.39192912	31.95985233	37.62366	68.6574
0	0	0.0427542	0	0.10461
0	0	0	0	0
48.8479	54.0686	56.4112	41.0952	80.1807
20.43312	20.13254	35.405518	28.26776	46.93549
0	0	0	0.401485	0.351358
0.36307	0.384898	0.359098	0.3429	0.144879
75.19683	71.470723	50.965797	55.94926784	0.9918
0.912329	1.50033	0.761879	0.841828	0.83055
75.19683	71.470723	50.965797	55.94926784	24.68
75.19683	71.470723	50.965797	55.94926784	24.68
20.8535	14.3332	14.5979	16.8717	22.2063
11.95235	10.61115	8.22436	8.65176	2.34695
20.751149	16.37450818	13.368835	15.74888449	6.61379
25.21884451	27.035316	33.67126	33.44685974	47.1724
25.21884451	27.035316	33.67126	33.44685974	25.94582
1.21561	0.109246	8.623290098	10.862516	10.85846

1.21561	0.109246	8.623290098	10.862516	10.85846
0	0	0	0	0
1.112491825	1.231041498	0.624557331	0.46863308	0
1.112491825	1.231041498	0.624557331	0.46863308	0.517627476
1.112491825	1.231041498	0.624557331	0.46863308	0
0.270362057	0	3.853768	8.495404013	5.356636
65.1686	59.29795	47.0488	47.42224	12.8392547
65.1686	59.29795	47.0488	47.42224	0
16.87725	14.20701	15.03488	16.7811	47.1238
13.87770323	13.923216	12.55642	13.81009	1.06388
13.87770323	13.923216	12.55642	13.81009	7.42455
27.66792707	28.95511594	17.09864854	15.86340962	3.5103
19.68306373	18.504568	18.35719708	19.184396	14.64079
0.11830935	0.056644778	6.229252	12.591474	22.22739
0.198686	0	12.25975879	28.64919917	62.089725
14.91631013	13.25672158	24.73335211	28.17809	21.55682169
14.91631013	13.25672158	24.73335211	28.17809	21.55682169
123.6126839	100.1055181	89.92178	109.6435602	44.097217
123.6126839	100.1055181	89.92178	109.6435602	44.097217
18.26754338	15.930722	18.12548388	15.210644	5.20721648
18.26754338	15.930722	18.12548388	15.210644	5.20721648
32.13455143	32.549294	31.614423	33.1281849	47.76964
32.13455143	32.549294	31.614423	33.1281849	47.76964
2.115578352	5.379419808	7.052684184	7.238997867	145.8884
2.115578352	5.379419808	7.052684184	7.238997867	145.8884
32.8674343	35.5729469	23.083624	25.12585158	36.849796
15.16298	13.05857	7.36569	6.982905086	3.452893127
4.803965156	3.093401055	5.949842286	7.603331304	69.689389
4.803965156	3.093401055	5.949842286	7.603331304	4.7097
4.803965156	3.093401055	5.949842286	7.603331304	69.689389
0	0	0	0	0
0.325997	0	0	0	0
0.280759	0.353187	0.479148	0	0.16337
0	0	0	0	0
2.360102	2.91799	0.333259718	0.75738	0.40736
0	0	0	0	0
0	0	0	0	0
7.877876953	7.023612125	10.86227926	8.279633065	1.93348
3.740493	3.424581795	5.045686	4.685651	4.20317
14.2513	15.6498	19.1906	18.2547	19.3259
2.68601	3.29609	0.331195	0.597911	0.134519
2.448722	1.431906227	1.552941378	1.761891508	0.203095
8.90657	11.4741	15.4605	14.2496	35.675
24.8150179	29.3517899	30.09024851	29.5275176	107.8133

0	1.70E-131	0	0	7.16E-16
10.67792629	10.9146181	12.3206103	11.74561943	34.10429207
1.29313	1.95E-06	1.5059	4.55079	50.8417
0.000252056	9.02712	15.4349	10.1926	4.42E-05
0.0477841	0.0476832	0	0	0.914333
9.28092	11.00635	11.59517	8.89292	43.27596
99.19283958	86.58404132	82.859038	83.42406051	124.2383
1356.832309	986.515611	169.480103	153.6815044	3.95717
2380.757	1677.4162	288.8305	282.46779	14.992166
276.231	196.6958	36.17816	38.19651	7.21666
168.48292	112.57139	13.548919	14.68930164	3.459741
384.1021007	264.766253	52.98793484	39.66071296	5.282690054
46.5643804	40.78096128	28.1702361	32.06227548	10.594153
125.2235981	137.653831	37.320536	49.729397	49.198713
125.2235981	137.653831	37.320536	49.729397	3.43031
125.2235981	137.653831	37.320536	49.729397	1.826635792
56.78556513	56.90459337	53.5471786	47.76338907	6.196613
56.349371	61.26771	16.811425	24.406476	10.02123
0	0	0	0	0
2.591779547	1.894470344	3.578875645	3.702895479	1.125856064
24.14589	28.26293742	19.259814	19.47289059	36.0516
24.14589	28.26293742	19.259814	19.47289059	17.3121
1.67602	1.69725	2.06853	2.2196	0.861028
0.640497226	0.695777361	0.478879374	0.405039604	61.14292181
0.729752992	1.435989	0.15739	3.67E-36	123.757098
4.1742664	4.36088	9.717412331	8.054387645	75.52037282
18.670067	19.80660011	8.166182	8.392656	0.961284035
18.670067	19.80660011	8.166182	8.392656	0.961284035
18.670067	19.80660011	8.166182	8.392656	3.425291543
18.670067	19.80660011	8.166182	8.392656	3.425291543
18.670067	19.80660011	8.166182	8.392656	3.425291543
40.5503	40.6696	97.2876	88.523	113.0187498
1.98408	0.000654448	0.00107154	0.290523	113.0187498
0	0	0	0	0
2.273668	2.56991	3.93499	3.79715	24.9418
13.81146532	16.44739571	17.90286296	14.914357	18.2255
0	0	1.04405116	2.815735	2.381589
11.971176	10.88510156	11.80481719	11.82585001	34.92468102
2.75626	1.206660987	2.4126263	1.763372579	36.64103
2.75626	1.206660987	2.4126263	1.763372579	36.64103
17.98473814	18.61772651	19.78531235	21.95786292	53.49592
22.1195	28.6894	29.7881	16.7759	1.68E-05
90.511397	89.180659	48.91487957	59.79246413	11.86310199
39.1286	35.3619	52.642	38.4194	51.7145

90.511397	89.180659	48.91487957	59.79246413	11.86310199
90.511397	89.180659	48.91487957	59.79246413	11.86310199
90.511397	89.180659	48.91487957	59.79246413	11.86310199
2.472871	2.777871012	1.92500353	2.298202	0.263779
44.413826	42.23562	37.04083004	40.69991034	26.36528
7.263789	8.070329868	11.13713407	9.634126629	59.85850475
0	0	0	0	0
30.8244	42.87417	14.14354	16.05056	20.46185
1.85047	1.23919	0.950443	0.977033	49.4005
0.311255029	0.398933459	0.077844995	0.156510545	22.82706
1.445420524	2.114330037	0.249283689	1.56002344	361.986
2.754095	2.944328	2.083199	1.844303	1.01476
0	0	0	0	0
29.03152936	25.29082981	16.5208805	18.646459	4.382137934
29.03152936	25.29082981	16.5208805	18.646459	4.382137934
0	0	0	0	0
16.18295	13.95604	2.994543	2.601925	0
16.18295	13.95604	2.994543	2.601925	0
4.941235801	4.185593023	21.15003233	22.24989586	13.82215951
2.150683	0.935375	5.94983	4.0246	0.259825
6.167591	6.024684	11.54665032	9.022435232	3.110624869
3.022504465	3.176753279	3.533708654	5.47948244	31.53905
0.13334049	0	3.49365	9.13942	5.88774
1.511393	1.61157	2.911779907	3.470539102	19.13161
2.48202	2.203342391	7.21409	5.64941	16.75873
21.4082	20.68794	20.77084	18.10983	49.13355
27.9307	30.2291	56.3507	45.0635	94.6459
9.814931	9.609213	17.346707	12.630505	29.79253712
2.949038	2.340609895	4.93621527	5.47864	2.27843
0	0	1.646753932	4.615708	1.21222
0	0	1.646753932	4.615708	0.333641
74.80311954	74.78315976	84.46627633	94.42412129	108.27967
46.5496	47.9263	111.6562	111.9393	85.9419
0.965081	1.87937	4.91192	1.79086	6.74988
52.875091	74.166589	145.59742	129.23452	13.2301
12.52136264	14.86586909	19.39518307	17.82347842	62.3337696
0	0	0.348161	0	0
23.5990269	25.4581816	25.008731	24.849939	25.22550547
23.5990269	25.4581816	25.008731	24.849939	25.22550547
0	0	0.855852	0.443541	0.715627
0	0	0	0	0.191459
11.43797574	11.80808	7.46286	7.69527	1.664001582
0	0	0	0.196086	0
0.284395	0	0	0	0

3.62273696	4.132707456	5.000596111	5.332263133	80.686867
0.598579	0.305979	6.67001	17.6563	14.6672
0.936994487	1.433712439	1.062798101	0.949831491	0.659671
59.03762	53.8782	80.74435811	78.9344	347.97
59.03762	53.8782	80.74435811	78.9344	23.8808
17.39129	16.11597	17.04759	18.58992	4.414723523
127.0455326	114.2427077	126.7669363	160.4925696	71.92464215
127.0455326	114.2427077	126.7669363	160.4925696	71.92464215
127.0455326	114.2427077	126.7669363	160.4925696	71.92464215
124.714	100.801	13.3241	10.5043	2.22867
120.5685	125.3928	123.502	93.4257	68.636
40.048546	37.91224678	29.32931584	35.24193403	13.3672271
0	0	0	0	0
0	0	0	0	0
0	0.891303	0	0	0.566236
21.83236	20.19135	32.3051	32.64	44.3481
21.83236	20.19135	32.3051	32.64	52.60245
66.21554	51.60729	71.64306	46.16519	100.802
66.21554	51.60729	71.64306	46.16519	55.69560427
9.61E-06	1.86E-05	0	5.47E-07	0.000155197
11.43386512	15.20970778	17.01081215	11.75602573	78.48222
0.809413	0.6866668	0.8730427	0.929957	0.532158
17.03050084	21.56888338	29.3654209	27.25838736	1.67529
17.03050084	21.56888338	29.3654209	27.25838736	67.5745
46.20980454	50.95040547	81.635371	66.58232904	39.42202
38.37088798	33.44429707	25.50281071	29.20074976	20.1631079
171.2024	206.7463	92.4557	82.536	211.1652
14.15155606	13.45379275	18.27328	15.373002	29.42438
78.35636609	74.376988	52.0845014	50.6094463	53.15408723
61.13016387	57.9663621	46.1021592	48.78971201	10.51825
59.72815006	57.08721673	49.08701159	46.61343898	26.06841772
24.0973	22.0429	8.70093	8.84832	0.503532
0.825248645	0.737569803	0.156273421	0.399190418	183.7901
0.041544053	5.41E-10	3.864141	7.408154295	6.520003187
27.03208	22.19777	44.70477	30.19487	99.4335
27.03208	22.19777	44.70477	30.19487	99.4335
15.83416002	23.81881004	25.61313029	24.50296002	31.93826672
1.58E-05	5.38E-05	4.02E-05	1.16E-05	10.6719
0.0912801	0.271729	0.262102	0	0.108067
14.070495	12.078639	22.89416	37.566461	4.359105
14.070495	12.078639	22.89416	37.566461	420.256
14.59002	12.54348	12.86434	19.21091	9.40739
3.154875946	3.678710524	3.37062805	2.982801685	30.12844204
0.113393243	0.330022	0.137124828	1.380811	50.797115

10.86328534	13.89876	18.7895	16.069239	62.1233
10.4572	10.919436	17.564392	11.5654	22.3179
4.1072	4.02108	6.06091	5.41053	24.7732
7.74885	8.8183	9.82665	9.9402	24.5959
20.5438	18.8752	31.6138	24.559	68.1937
31.06267	35.29698	37.00605	36.3431	48.4218
4.864640879	5.182246	6.00192	6.253242	28.91868
3.042856205	3.147017	4.882798495	10.065305	14.6411
0.703661	0.711949	0.135178	0.366904	0.467303
3.042856205	3.147017	4.882798495	10.065305	2.047868
2.86186	2.48635	2.560988	1.80991	0.550386
44.88099456	49.71447104	17.5204025	19.58273876	1.799896
44.88099456	49.71447104	17.5204025	19.58273876	1.799896
11.01041192	8.504048023	11.55835638	12.66476073	9.669772285
4.90929	6.91756	8.82703	5.66002	20.1507
21.3342	27.6533	47.2368	52.7434	49.8895
25.75334753	25.24550832	25.10074932	32.39376576	8.352032
25.75334753	25.24550832	25.10074932	32.39376576	8.352032
1.68388	1.17302	1.40474	1.36459	0.516978
4.321392316	4.268381794	3.211144835	2.28290243	0
0.457063	0.56604	0.807979	1.3582401	11.27402
15.336262	16.31098	44.93314	37.90736	11.27402
2.89649	2.64064	1.23232	1.39993	0.304681
1.957570249	1.917953358	5.090624042	3.58970111	2.166070424
22.68196661	20.42560463	14.2065216	17.76450341	4.398209711
0	0	0	0	0
0	0.0874928	0	0	4.29435
0	0	0	0	0.363911
0	0	0	0.0496875	3.40095
0	0	0	0.0212241	1.70258
0.148885	0	0	0	1.23763
0	0	0	0.110144	1.44047
0	0	0	0	0
0	0.0439478	0.256825561	0.230203438	0.655775524
6.622212	8.83149	10.09304578	17.46533	1.15249
22.97201384	24.14532673	24.39362435	24.51538909	24.66120211
30.95017034	27.50790585	18.45709858	20.33710835	5.329551523
0.246682	0	0.236115	0.630789	0
0	0	0	0	0
88.712	127.3986	53.69757	52.3019	8.193075684
88.712	127.3986	53.69757	52.3019	8.193075684
0	0.0730459	0	0	0
0	0	0	0	0
0	0	0	0.200778	0

0	0	0	0	0
0	0	0	0	0
0.214216	0.0716013	0.345633	0.186203	0.245721
0	0	0	0	0
0	0	0	0	0
0.0672974	0.0681493	0.0656244	0	0
0.0695345	0	0	0.0882452	0
0	0	0	0	0
0	0	0	0	0
0	0	0	0	0
0	0	0	0	0
58.2334	61.2491	102.9027	102.8039	46.0187
1.80864649	1.63436633	3.32473536	6.6052836	45.2867
0	0	0	0	1.53241
0	0	0	0	2.11772
0.107314	0	0	0	0.501716
0	0	0	0	1.01759
0	0	0	0	3.74428
0	0	0	0	0.788063
0	0	0	0	0
0	0	0	0	0
0	0	0	0	0.220949
0	0.0949174	0.372478	1.37333	0
0	0	0.439257	1.03541	0.269055
8.92E-05	0.101636	5.96E-07	0.272528	0.597252
45.022384	49.37688	45.511664	35.750489	50.7793
0.0410004	0	0.0804044	0.107072	0.194222
0	0	0	0	0
2.16E-46	4.58E-08	0	1.19E-10	0
0	0	3.61574	8.46334	14.1647
31.6155	13.989318	20.936243	19.65604169	0
31.6155	13.989318	20.936243	19.65604169	8.43567
6.06925	6.242560145	10.04361477	9.67173	3.59793
6.06925	6.242560145	10.04361477	9.67173	27.24787
1.61377	1.18173	2.30221	2.26374	7.25496
38.92935444	41.167325	30.971738	24.96676317	2.676391721
38.92935444	41.167325	30.971738	24.96676317	663.702
13.625286	13.5982719	26.09167	27.335097	31.09051471
15.65508	14.62092	22.73992	13.85369	41.2621
25.49831	29.86083202	35.880305	35.762782	44.853863
2.860795179	2.8908064	6.677403953	6.310401549	0.0804405
2.860795179	2.8908064	6.677403953	6.310401549	2.3387
0.044428	0.0856411	0.245803	0.381234	75.05061
24.7769	32.8401	25.1455	23.5864	46.72557

9.16547	9.38009	10.20277	8.70134	18.823783
41.59918	38.026003	27.59555	30.83364	19.2489633
41.59918	38.026003	27.59555	30.83364	19.2489633
41.59918	38.026003	27.59555	30.83364	19.2489633
62.608	63.2395	76.3472	72.9643	142.055
59.7974189	73.46836	94.7744382	86.07174	124.4586
6.84854122	6.36207624	6.088492975	5.928954209	2.760534413
6.84854122	6.36207624	6.088492975	5.928954209	2.760534413
6.84854122	6.36207624	6.088492975	5.928954209	2.760534413
32.25580773	35.27100744	33.68320984	32.16501131	60.45860738
28.85648	26.06171	24.27899	23.47444	188.6082
5.93508	10.67163	15.04601	12.95109	4.098668445
7.3905	3.398429	9.95633	7.06114	4.54235
22.1213	20.79618	40.7187	33.825	17.70478
28.40306	37.54512	76.87624	88.95137	49.4424
3.814196723	4.140154117	5.168879934	5.269341537	15.55613
0.516992	0.392512	0.357317	0.30842	0.431296
0.733107	0.00330533	0.000544222	0.0348212	3.80E-06
16.794662	17.077363	18.685436	22.69213	72.03652088
38.63938	38.55964	36.170357	34.80187	33.44604
39.345	46.4049	52.3498	53.75235	73.59215
0.71349	0.235323013	3.626031	6.90687	3.28846
0	0	0	0	0
166.007	194.25865	165.2432	143.0588	53.52835832
45.124799	64.2815092	48.92390692	41.78760657	49.78308
12.2017	9.77574	12.3765	13.0523	9.41374
23.123175	23.219464	18.1733813	18.36312	14.09585
78.83999052	77.50936	55.16058	64.62089326	33.6767
16.7858	17.5282	25.6012	18.8838	35.7824
3.300013	3.676018287	10.3141	6.937708	32.2212
43.9837	39.8335	17.25828	21.06932	2.40616
9.36831	4.14256	1.90411	4.83663	0
4.66922	4.75185	10.5581	4.93247	34.9048
5.400879	4.74501	6.53696267	6.524192	14.64543
24.635141	23.21301571	17.78300501	17.702165	10.8303
24.635141	23.21301571	17.78300501	17.702165	4.28095
0.894472	2.026055	5.35422	4.89579	1.51238
61.83876557	59.63615504	59.1159222	60.10983867	36.63914821
0	0	0	0	0
18.8623	16.7675	32.0939	28.851	34.1103
18.888036	27.07479	15.8965	21.11379	61.45281551
58.0432	69.4536	95.7462	75.9642	145.5644
19.5188	17.952	29.3912	41.9325	78.2934
10.88381739	11.28890729	15.12112306	19.93203693	15.74407912

10.88381739	11.28890729	15.12112306	19.93203693	15.74407912
1.34133	0	0.823083	0.574231	0
91.648922	84.59525538	177.2792082	188.2493703	181.564
56.792	42.0798	36.9959	50.7868	128.147
21.47208996	24.07591846	17.88451837	22.99964	26.333987
11.37971034	7.194133601	36.648566	44.32235545	17.63670136
11.37971034	7.194133601	36.648566	44.32235545	17.63670136
1.675712955	1.53857	1.66523	2.183611193	2.225493
1.675712955	1.53857	1.66523	2.183611193	2.225493
0.217621	0.27189	7.35E-06	6.33E-06	0.000104387
0.217621	0.27189	7.35E-06	6.33E-06	0.000104387
0.18705	0	0	0.241111	0
24.1668278	22.6090192	16.48484562	16.55954836	4.158401
3.438789002	3.79789072	2.059328911	2.800761	0.3202
58.5904	58.8992	44.795	35.5971	14.19610615
0	0	0.266928	1.97423	1.4906
0.869436	1.13586	1.14323	1.08124	1.31476
13.96272725	14.18287	19.37768	16.250233	86.0193
0	0	0	0	0
0	0	0	0	0
0	0	0	0	0
12.7509	11.49383	12.700445	14.34991	21.73112
12.7509	11.49383	12.700445	14.34991	21.73112
0	0	0	0	0
0	0	0	0	9.782230092
8.3185823	9.089463023	16.23139	18.8068712	12.833874
8.3185823	9.089463023	16.23139	18.8068712	12.833874
0.893797053	0.931178647	1.414123955	1.576291916	1.55005
0	0	0	0	0.171281
6.33955	6.01219	11.5758	14.4136	31.9401
0	0.101484	0	0	1.49496
0	0	0	0	0
0.102436793	0.247599	7.567647383	20.21790207	23.42039765
0.102436793	0.247599	7.567647383	20.21790207	23.42039765
0.165858	0	17.3482	46.7259	58.9176
22.52403	20.21715	22.3786	18.82025	39.37175
22.52403	20.21715	22.3786	18.82025	39.37175
119.1429286	103.67734	69.73058726	62.50948548	9.791663213
119.1429286	103.67734	69.73058726	62.50948548	9.791663213
0.124589	0	0	0	0
0.0259152	0	0.0257869	0.0345676	0
15.02889	16.74163	17.32947	16.05566	31.82314
3.028366523	3.879790892	1.500221865	1.765485594	194.348
1.094453	1.757320187	0.0993689	0	194.348

8.5415	7.72819	13.94579	14.73908	18.9704
0	0	0.577514	0	0
21.840277	23.34684267	21.86316478	18.5016054	46.71704
33.19607	32.01652	23.76051	24.9252	54.03108
0	0	0	0	0
0.778167	0.786322	1.44692	0.926149	0
29.1616711	24.5833251	96.374921	115.15265	135.18274
1.570624336	1.016246897	3.820030608	4.842303579	3.23671728
20.99059538	25.64185494	28.8649237	27.549974	40.791
20.99059538	25.64185494	28.8649237	27.549974	74.53722134
20.99059538	25.64185494	28.8649237	27.549974	74.53722134
128.3550591	123.6552503	136.79161	119.29732	52.87124279
0	0	0	0	0.114441
0	0	0	0	0
31.93367	32.88917	31.63864	31.28048	43.14335
6.14609	8.17961	6.77196	7.48382	2.165517
131.4339818	127.7135439	97.95369647	95.660244	28.01146
131.4339818	127.7135439	97.95369647	95.660244	11.89991065
131.4339818	127.7135439	97.95369647	95.660244	11.89991065
14.242871	19.86072	11.200515	11.717112	2.817215588
14.242871	19.86072	11.200515	11.717112	2.817215588
0.0772868	0	0.0734418	0.197609	0
9.913424	6.771582	5.802245	5.733933975	1.33327285
195.26831	199.79682	197.68666	163.8453945	144.5086
107.0029145	118.9883596	120.88401	111.0176009	28.9306
169.99056	128.46977	77.41561	58.30416	11.4304
0	0	0	0	0
0	0	0	0	0
0	0	0	0	0
10.17619113	4.72249014	4.051032167	4.281972328	0.750327
10.17619113	4.72249014	4.051032167	4.281972328	7.71E-06
119.4238	120.274	136.8661	124.8326	110.836
12.47363	1.6183	3.2619	7.03912	2.08991
46.91709475	42.83061163	22.43495313	27.16500678	5.763671
46.91709475	42.83061163	22.43495313	27.16500678	5.763671
14.30876359	14.71369411	19.011089	15.27682538	19.068555
1.01026	0	0	0.437545	0.397686
0	0.636446	0	0	0
0	0	0	0	0
22.20805315	20.70967205	23.000848	19.71500203	61.49169
16.40722	20.15543	11.36735	11.52342	3.418020724
19.43088509	20.08741668	25.77718	27.25678944	44.515
205.672363	196.2048352	181.5534761	194.3012563	54.86802
205.672363	196.2048352	181.5534761	194.3012563	6.33601

96.7024347	99.1826265	9.3687666	15.59279841	2.3166
2.786872	2.295388038	2.183578	1.95003	0.676582724
0.325093	0.588006	1.14996	0.47966	5.50417
0	0	0	0	1.39082
5.05204	5.40113	6.43234	6.11077	6.13385
10.73958108	12.01805	17.96774	16.786794	66.2324
10.73958108	12.01805	17.96774	16.786794	66.2324
10.73958108	12.01805	17.96774	16.786794	66.2324
37.15884695	36.71079459	40.67804371	41.389782	53.75571118
37.15884695	36.71079459	40.67804371	41.389782	53.75571118
6.03E-07	1.21E-06	2.78E-06	1.52E-05	3.68757
30.0189	32.11501	30.55156	25.22677	52.5415
22.829599	25.649528	36.073252	35.9352223	66.1941
9.3043	8.70332	12.4389	10.7318	19.8127
0.784140556	1.124886522	0.955109014	0.95398159	43.51544
0	0	5.63265	10.193	14.4983
39.3279	37.8478	37.437	39.9652	49.7947
3.20428	2.64557	3.406556	2.980434813	9.33061
17.403314	16.58752605	19.0475139	13.1132544	9.07123
99.2787	121.9138	71.4757	65.902	107.9838
4306.819145	4091.628807	4762.281971	3936.75689	44.000995
4306.819145	4091.628807	4762.281971	3936.75689	44.000995
26.4641	24.4265	17.1705	18.444	5.367572689
28.720081	29.025127	18.97338	23.5658	1.76255
56.53004	57.80453	41.5834	44.18126	40.88250582
56.53004	57.80453	41.5834	44.18126	40.88250582
28.720081	29.025127	18.97338	23.5658	3.19725
14.27659	11.99225	8.437108	9.978861	347.408306
0	0.647035	0.201653	0	1.42081
9.81458	10.07525	15.24949	11.99079	10.4958
35.744537	34.467671	29.03014	26.03937	6.69938
35.744537	34.467671	29.03014	26.03937	1.12405
0.438762	0.438773	0	0.287808	13.9206
0.114225	0	0	0	40.8182
13.065096	13.511842	10.594202	13.95297838	5.909395338
0.535405	0.531827	0.823598	1.28501	2.46788
4306.819145	4091.628807	4762.281971	3936.75689	729.341
22.07746413	15.91110122	8.271896673	12.72258	0.84835254
16.63147	19.72446	18.69883	15.52797	12.89594194
6.52279	7.50141	12.4122	11.3045	61.8131
8.33207	10.48363	11.91326	12.16717	16.53224
1.604099115	2.130723	4.60455	4.216745	1.118141387
0.139193	0	1.9226	0.557934	0.995727
0	0	0	0	0

5.100931	5.041530622	10.66913	15.121082	41.1843
5.26296	3.96985	9.77271	9.06014	27.15344
45.01172421	40.30101498	41.65035968	39.656179	17.1358
45.01172421	40.30101498	41.65035968	39.656179	9.53545
0.699796	0.732163	0.99511	1.673461	0
7.13143	9.44226	11.33136	9.239496	30.313538
44.29326	42.086999	40.48701	38.80516	21.68022
13.32853	18.32796	13.76469	20.26751	1535.772137
5.78003	5.93061	11.1167	7.21125	16.2964
11.438	11.9102	18.293	19.815	45.809
33.2264	41.6888	12.05585	16.81913	35.2829004
712.882	807.063	1180.24	1159.47	1210.66
1.029295	1.00837	1.497448	0.963611	15.5665
0	0	0	0	0
14.94313	16.48518	17.44246	15.54716	45.8402
10.4153	10.4253	9.58676	6.49499	0.000432862
14.15883	12.21219	12.175336	11.371221	33.180629
14.15883	12.21219	12.175336	11.371221	33.180629
12.57822	13.79417	11.56338	10.43679	45.88231013
0	0	0	0	0.942879
6.97874	9.15045	11.52481	7.22198	81.1919
58.6668014	57.34850751	65.77404	55.63131	144.944
11.3125	13.1669	13.1588	15.5446	4.07112
59.45232361	61.105899	83.519692	67.9803	155.857
59.45232361	61.105899	83.519692	67.9803	5.82894
59.45232361	61.105899	83.519692	67.9803	16.457
10.81607	11.087534	19.7101	20.45592	34.96745
11.20769351	10.74191602	10.10092198	11.06343072	37.05768029
0.72194	0.287465	0.276103	12.967	11.6061
620.8210011	376.7529421	516.9810052	548.796715	118.38923
8.31140344	8.102073459	6.839811286	6.536705	3.962393178
5.218790833	5.683999422	8.4129459	7.151629778	2.96117
7.27624	8.5125	10.9935	8.25465	9.05434
67.82999566	73.92146471	54.39597002	50.83062573	48.73943
67.82999566	73.92146471	54.39597002	50.83062573	17.2608
86.70743746	109.6981367	78.95426456	62.55621943	12.598844
35.17163	32.39948	26.02916	24.90541	4.830820488
0.922073	0	32.273	68.6956	61.5652
10.78189	10.13117	10.90403	10.04093	35.4872
2.821984	2.788312	7.836697	7.030281529	7.77881
0	0.0845725	0	0	0.407386
63.22390083	57.8763736	24.409401	22.54474337	1.80129
63.22390083	57.8763736	24.409401	22.54474337	15.8268611
3.11115	2.489672	1.055644	1.002824301	35.276

9.97437	8.84795	11.04753	12.72688	22.54293088
60.3025	57.0318	44.4525	40.2525	20.63494
7.33978	8.43973	8.6106	7.68634	20.63494
33.44389	35.64753792	24.14558063	30.29333958	56.5211
45.2768	47.11253	33.92759	36.9805	12.59911
0.564366	0.698215	4.728388	1.076174	51.8904
0.564366	0.698215	4.728388	1.076174	51.8904
0.0999266	0	0	0	2.048296412
1.73724	1.52921	1.77348	1.35119	1.18398
1.5017	1.17624	0.943801	0.540386	0.556822
0	0	0	0	0
0	0	0.571281	0	3.13658
0	0	0	0	0.378179
0	0	0	0	0
0	0	0.128478	0	0
0	0.312047	0	0	0
0	0	0	0	0
0.0244593	0	0	0	2.58723
0	0	0	0	0
2.21411506	2.435100915	2.962689	1.536490357	34.87780261
2.21411506	2.435100915	2.962689	1.536490357	34.87780261
0	0	0	0	0
2.31E-06	0.269189	1.09E-06	3.80E-07	0.467026
0	0	0	0	0
6.65206	5.53553	15.27801	13.65566	1.51622
0	0	0	0.12844	0.230336
20.95298718	19.5442016	30.70613116	32.73366309	59.14662002
6.77527	8.91648	9.62797	16.4775	1442.702
47.8969	50.94005	66.19333	68.50994	68.0549
47.8969	50.94005	66.19333	68.50994	24.3074
1.01018	2.04301	0.463985	2.5873	1.13938
23.20605	23.6536	24.80555	26.17496	37.6717
40.3748	42.63486	38.56896	36.58640738	79.5012
55.4815	68.2504	56.0843	58.441	123.528
0	0.0674053	0	0.0874302	0
11.071508	11.475706	13.397698	10.49619901	35.269033
28.08156	27.99852	20.09266518	17.13412	7.52734
0.7535278	0.640078601	0.24577725	0.563849	0.0840111
0	0.416895	0	0	0
0.727584	0.11646	0.187898	0.202386	0
57.60476785	64.53456604	60.84025686	46.18375936	40.411586
5.345827	5.05256244	14.32545	20.5468	25.17050192
5.68088	4.93466	9.24411	7.73599	56.44890432
0	0	33.1795	62.7949	56.44890432

0	0	2.71478	2.89445	0.953493
13.92836	14.40316	12.12492	10.83083679	3.803515211
0	0	0	0	0
0	0	0	0	0
0.15197	0.1296434	0.322937	0.367192	0.154968
0.153111137	1.35E-24	0.08600604	0.134693486	0.952192876
0	0.768534	0	0	1.938243
0.889298051	0.390609004	0.593053304	1.074112	0.418044
3.273003	4.287678	2.619781	3.391201989	5.027624
0.367767	0.750927	0.360225	0.245146	0
0.262594	0.0439453	0.0855808	0.0573261	0.208499
0.898158001	1.5752504	1.67746	0.996242	1.66067
0.100121	0.204435	0.397107	0	0
0.598832	0.916082	0.735328	0.999835	0.349692
0	0	0.254987	0.000275037	0.418169
0.14528	0.073625	0.0728242	0.291883	0
171.466	91.8252	220.836	324.573	17.7881
16.71191	18.67413	10.59724	10.27918	2.4375
0.36343	0.161881	0.391366	0.470362	0.0940486
17.29836325	14.6994561	22.23933448	19.34554449	2.41227
0	0	0	0.067451	0
69.702247	61.126084	75.916021	58.78357018	16.5461
0	0	0	0	0
0	1.03383	0	0	0
0	0	0	0	0
1.45841	1.05824	6.36284	5.57853	5.16542
4.19883	2.71976	4.39786	3.31786	1.37884
6.3254	7.37517	7.41889	7.7867	34.911
0	0	0	0	0
0.0618536	0	0	0.161405	0
0.786568	1.055611099	0.766874235	0.181105016	0.103682
0.125515	0.0639713	0.804577	0.167125	0.232119
1.136539	2.337268	0.713344	0.549286706	0.179599
1.136539	2.337268	0.713344	0.549286706	0.401471
0	0	0.0857096	0.114995	0
0.29531	0.149397	0.361057	0.0970146	0.088222
0.370972	0	0.283269	2.25605	0
0.228646	0	0	0	0
0	0	0	0	0
0	0	0	0	0
0	0.261238	0.18959	0.256241	0.603161
0.191326	0	0	0	0.495271
0.2412454	0.091789283	0.477033461	0.285251	1.143391
2.614485	2.745829	2.805710302	2.97189	2.2214006

0.11799	0	0	0.154615	0
0.375852	0	0	0.463928	0
0.374648	0	0	0.166614	0
0	0.0833226	0.320084	0.216527	0
0	0	0.135128	0	0.0563552
0	0	0	0.10528	0
0.0903373	0.0928477	0	0	0
0	0	0	0	0
0	0	0	0	0
0	0	0.291283	0.404652	0
0	0	0	0	0
0.104707	0	0	0	0.0631574
3.563587009	4.359730004	5.263780012	5.50622	1.7595
0	0.265754	0.344062	0.174489	32.8438
0	0	0	0	0
11.11526	9.96772	14.59844	13.05733	32.39643097
128.442	130.17	80.7105	91.5682	67.3909
1.57316	1.16141	4.9315	7.93435	6.71559
58.179248	54.92839	57.43224003	50.3301329	11.785446
0.863010464	0.777551413	2.864945007	3.42273938	19.21716498
6.495041	7.86565	5.656061	6.547236	1.232263847
10.904616	6.24196122	7.62021174	8.64870607	1.24078977
16.5984	18.6813	20.5855	16.3762	34.8083
21.4546	24.76	20.66283	13.79154	17.09623
0	0	0	0	0
9.3097	10.07039	8.84061	10.76111	15.16163242
17.6084	11.8106	19.072	0.181738	72.7191
350.703	332.5471	360.63657	388.053	252.7023
0	0.0731771	0.212421	0	0
8.95408	6.33111	12.00392	10.50126	33.5759
0.580724	1.40128	0	0.151048	168.6908
0	0.971438	0.118359	0	168.6908
0.541343	1.40466	0.378533	1.49607	187.263
1.26919	1.37215	0	0.13736	232.472
64.9425	47.2358	22.5765	17.3323	1.40383
1.35452	2.0114	0.907561	0.9792	0
10.722796	14.69031116	24.61507487	26.86767694	7.47423
0	0	0	0	2.73314
4.76094	5.673688	3.4846138	3.321829	0.946987261
4.76094	5.673688	3.4846138	3.321829	0.946987261
86.417	35.5881	111.269	86.4797	609.736
24.3037	13.6764	46.58002	35.79969	0.535982
241.324	75.343	91.2173	118.701	15.2393
24.3037	13.6764	46.58002	35.79969	3.19419

4.76094	5.673688	3.4846138	3.321829	0.946987261
8.618980316	6.758072713	6.839680343	7.350341359	4.333450502
8.618980316	6.758072713	6.839680343	7.350341359	4.333450502
0	0	0	0	0
0.0573242	0.116328	0.62063	0.530809	0.137092
14.556715	13.45525254	9.165433	9.970242141	2.914800791
0	0	0.656177	0.795007	19.06480002
12.380081	12.06056	8.52299	7.700976	2.18602
1.66946	0.119499	11.3791	26.3701	13.059
1.301774	1.122218	1.7942535	2.400865	13.42392
12.37498137	13.06441049	22.05161	28.24694805	56.315931
23.540675	23.22651006	43.793954	45.0952	18.250492
8.50126	8.00231	8.7866	8.64642	42.7836
57.3207	31.63221	6.645861	3.463753	4.19138
4.579826576	5.394609	10.62267	6.75058	23.90793
4.579826576	5.394609	10.62267	6.75058	23.90793
12.8499	17.0983	1.9742	2.17558	0.223338
16.69528603	14.52840422	18.46511433	16.93040554	55.44130675
167.181009	160.197385	133.615301	140.04088	19.672059
109.866	94.2496	25.5034	18.0603	0.930981
1.241116	1.0925399	1.624093024	1.65464819	3.13760419
3.33554	2.43866	1.23402	2.68789	0.919426
0.342714	0.147326	0.217254	0.12921	0.238608
6.58038	6.84503	10.5163	6.56234	5.86231
34.1648907	35.48479078	31.54788976	34.73578866	76.9586
34.1648907	35.48479078	31.54788976	34.73578866	6.09E-05
18.67655554	22.8512549	23.97319	21.98002053	25.91140407
32.5881	32.3839	22.9299	25.7218	34.2938
72.04000003	71.87560093	43.67510029	27.60900145	2.547448065
72.04000003	71.87560093	43.67510029	27.60900145	2.547448065
72.04000003	71.87560093	43.67510029	27.60900145	45.7088
0	0	0	0	0
21.63546	19.70562945	17.41347969	16.4337415	13.33565614
13.64843	12.44713	14.86929	19.44565	12.2481
3.570524847	8.02171755	11.77791597	20.47298978	0
3.570524847	8.02171755	11.77791597	20.47298978	1.496992
3.570524847	8.02171755	11.77791597	20.47298978	0.858989
3.570524847	8.02171755	11.77791597	20.47298978	46.355985
1.1488685	1.4869045	2.176075856	11.59851	46.355985
338.518181	332.619283	315.683456	309.055555	0.12411
0.555454303	0.383079366	4.112245073	7.41198	2.044989
18.06162	16.82465	26.80312	23.99546	37.8765
0	0	0	0	0.215035
5.13081	3.00026	8.70712	10.7392	13.0385

1.55018	1.44175	3.43659	2.80457	6.05501
2.4041	1.13613	4.36358	7.15166	4.63483
29.985692	27.18400698	25.2049882	24.98560064	20.96434
45.20461	38.70822	14.99312	20.92606	138.6295
22.84260784	25.034353	34.28276875	28.23136015	65.992
14.63952061	14.503088	21.56092721	20.55465646	5.875298528
14.63952061	14.503088	21.56092721	20.55465646	8.307469227
14.63952061	14.503088	21.56092721	20.55465646	8.307469227
7.0514508	6.25268123	10.9469225	7.863021224	6.373850084
7.0514508	6.25268123	10.9469225	7.863021224	6.373850084
7.0514508	6.25268123	10.9469225	7.863021224	6.373850084
10.547676	11.042883	14.78648271	12.035267	14.75652
0.129647	0.0873228	2.207376587	4.35899	3.720795192
0	0	1.94353	2.68579	0.478715
6.773137	5.864203	7.807628	5.748714	3.88368
37.4784	38.53705	35.5998	33.22688	57.4812
0	0	0	0.142689	0
14.53213201	15.65708371	20.31763281	24.9077198	11.44294906
14.53213201	15.65708371	20.31763281	24.9077198	11.44294906
14.53213201	15.65708371	20.31763281	24.9077198	6.610959802
14.53213201	15.65708371	20.31763281	24.9077198	6.610959802
14.53213201	15.65708371	20.31763281	24.9077198	13.73472782
14.53213201	15.65708371	20.31763281	24.9077198	13.73472782
20.16435	18.30144	23.9567	20.556	94.3547
4.62E-134	5.64E-10	8.30E-48	2.47E-204	1.10E-05
4.62E-134	5.64E-10	8.30E-48	2.47E-204	1.10E-05
4.593015853	3.460628138	17.27917	18.37949	18.79534
0.39708	0.134675215	6.38806	18.74622	21.90208
0.392908779	0	3.93421122	6.7051	4.6544
0.557455	0.923654	20.5308	44.0856	30.7563
0.015032433	0.279469512	0.000274064	5.40E-06	66.2561
0.015032433	0.279469512	0.000274064	5.40E-06	175.091
0	0	0	0	0.0687621
0.129264	0.0323905	0.0626805	0	0
0	0.423967	0.102054	0	1.88E-06
0.249345901	0.0800146	2.861973	8.03133	3.95778
0.86496827	1.087641463	2.468574	2.953163	0.996147188
556.3909	610.6115	607.1962	483.6937	484.215
0	1.08278	0	0	0
54.08474736	46.0456023	24.58789744	25.99881302	2.876125952
667.38262	657.221759	555.45843	478.326541	81.75432
0	0	0.763852	1.90275	0.891215
9.38645777	9.396669	16.008529	14.1365255	18.395034
9.947549	9.439687	20.268558	13.69646	14.460049

9.947549	9.439687	20.268558	13.69646	14.460049
8.400056	7.422744	12.42333	9.71997	31.189131
30.5202	31.2593	45.30919	37.51162522	39.87552313
25.0405	21.7033	33.9503	23.1024	32.5307
12.28105553	12.41385165	11.97446326	12.32423818	21.6295
0	0	0	0	0
0	0	0.11674	0	0
18.74253	17.0492018	13.64329072	13.44171	4.88905
208.4475025	191.8566075	140.3400142	154.3561172	40.69294
3.542177	5.399694	7.93982	7.951960368	56.72831068
1.662270061	1.711116771	4.259772541	3.435904855	35.25924625
89.66095312	81.73386114	62.20337907	56.13229	35.295817
0	0	0	0	0
38.2953799	32.59732153	28.07035434	26.82764562	16.80181
0	0	0	0	0
178.7763422	141.1211551	64.61674315	75.74739456	17.39275066
178.7763422	141.1211551	64.61674315	75.74739456	41.6312
0.075078	0	1.48943	1.85779	2.1625
51.17274592	49.33194493	44.73227797	38.8401946	99.34029776
51.17274592	49.33194493	44.73227797	38.8401946	99.34029776
41.7145	49.02074	37.10777	34.63005	49.5072146
57.13733555	42.75185328	76.38104229	64.535294	16.70283232
8.58273	10.50206	13.07944	14.37884	22.686
25.08643	24.26281	1.483384	2.906275	0.9627
54.52505	54.59509	104.09175	95.02934	92.34484
0	0	0	0	0
42.87044	41.78785	89.5594	64.5203	220.099
1.288310333	0.297384024	0.094452989	0.127971	0
8.824625604	8.672657658	5.223048969	4.399925718	1.8997729
35.95330759	34.55184829	39.1683138	33.18309704	13.09432387
13.74118347	19.0447	7.34552	8.250128673	43.7574
13.74118347	19.0447	7.34552	8.250128673	55.4316
0	0	0	0	0
0.514075486	1.481734	0.933177947	1.910222001	0.851185553
0.514075486	1.481734	0.933177947	1.910222001	0.851185553
67.24363196	70.0965775	46.179654	44.54087748	58.5067
67.24363196	70.0965775	46.179654	44.54087748	58.5067
30.01295	24.47171	33.383	34.38044	86.382
7.846985	9.19346	18.26609	13.15801127	55.10128065
7.846985	9.19346	18.26609	13.15801127	55.10128065
32.48851	34.07345	37.0692	39.27158	49.37189062
38.5798	41.416	42.3071	36.0142	58.71382
0	0	4.82847	6.58627	13.4807
15.15877	14.2733	14.24191	10.33042	68.1964

9.31033	8.96327	15.4055	14.1354	35.2072
5.40863	8.31877	6.15705	6.03207	7.01252
10.5251	11.8602	10.4574	10.9641	19.43769
18.02693	19.46309	22.72313	18.96306	37.7281
30.4461	24.3973	13.09265	17.03082	1.84757
1022.1005	993.6794	498.2589	499.5226	104.3579
28.26143	23.75371333	21.32915	29.65191	2.46556
28.26143	23.75371333	21.32915	29.65191	6.535845511
0.495480838	0.800899344	0.254698366	0.433502761	127.899782
36.9155	35.06442	22.94692	22.45135	23.43098118
10.009449	12.47324	17.64435	14.4624558	32.1309221
10.009449	12.47324	17.64435	14.4624558	32.1309221
1.80181	1.37604	4.1767	7.7853	3.34276
13.55954	10.9209	11.5597	10.19857	8.68E-05
62.92331	67.3859	48.21452	45.5627	93.28883
13.55954	10.9209	11.5597	10.19857	13.0668
92.55232	82.44485	63.26707744	72.043776	67.43805676
2.20488	1.69593	2.66893	2.38769	14.6498
92.55232	82.44485	63.26707744	72.043776	68.70389
3.3963	4.66759	5.14531	4.62687	23.78126
10.81432637	13.802008	15.30478	16.33719	34.85596097
7.94383731	8.42865	11.19810551	11.98717	111.4602
0	0	0.23485	0	7.53147
57.17786486	56.63313342	43.26590402	49.96185051	26.65543
61.18106696	66.67343052	56.01694274	54.58714117	14.40248223
61.18106696	66.67343052	56.01694274	54.58714117	14.40248223
61.18106696	66.67343052	56.01694274	54.58714117	14.40248223
61.18106696	66.67343052	56.01694274	54.58714117	9.213184965
61.18106696	66.67343052	56.01694274	54.58714117	14.55310331
61.18106696	66.67343052	56.01694274	54.58714117	11.86285
61.18106696	66.67343052	56.01694274	54.58714117	11.86285
61.18106696	66.67343052	56.01694274	54.58714117	11.86285
61.18106696	66.67343052	56.01694274	54.58714117	10.93461
61.18106696	66.67343052	56.01694274	54.58714117	2.71534
61.18106696	66.67343052	56.01694274	54.58714117	14.05171495
61.18106696	66.67343052	56.01694274	54.58714117	14.05171495
12.77174377	10.63229178	25.63323431	25.84199432	28.58176
6.24433617	6.12502637	16.7011639	11.65029614	12.192743
6.24433617	6.12502637	16.7011639	11.65029614	12.192743
0	0	0	0	0
150.121484	89.95822674	45.01799	37.97529	0.899406
2.683736	2.321136207	4.947107637	8.859494377	10.38177
2.683736	2.321136207	4.947107637	8.859494377	10.38177
3.23664024	2.859431797	11.62488	16.43396	26.83003

0.157136	0	0	0	0
55.7168562	55.934497	63.8707	57.92178142	57.80028
55.7168562	55.934497	63.8707	57.92178142	57.80028
25.44441509	28.333868	34.51822818	35.29330013	69.39980567
4.192139	2.369269	7.05667	5.068063	1.05686
9.872483477	9.428474135	6.536060511	8.002323948	1.54603
0.278055	0.283168	0.137345	0.832923	66.6713
13.336812	13.16158886	13.34909	8.037734062	1.04283
38.25622285	38.227879	33.12898864	35.001185	49.537642
64.3325803	81.294113	84.2205062	70.402413	9.26039723
22.4275995	14.8857779	15.00795091	18.76003149	0
22.4275995	14.8857779	15.00795091	18.76003149	0.0751433
22.4275995	14.8857779	15.00795091	18.76003149	4.27189
17.14648	19.560442	14.406867	15.92222	0.747725
28.7723	31.8753	29.1507	27.762	178.663
15.06842152	15.72788656	13.90699699	10.05983631	2.28913
1.543175496	1.69848234	2.246646613	1.778451074	60.653247
32.2001	25.1648	26.9828	27.3684	18.74710288
0.182153	0	0.30222	0	17.07135
12.9564765	12.75510284	25.45822727	21.62510965	17.07135
8.63772	9.64983	4.06257	1.97718	1.42192
22.07618552	20.62900001	15.49273678	19.81946295	67.91432
0.249792	0.167966	0.643781	0.216061	0.591328
3.335028212	4.9668138	3.6515257	3.2861824	0
3.335028212	4.9668138	3.6515257	3.2861824	0
6.82556	7.316182344	11.41907	13.6333	11.7838
4.932739611	7.967685894	6.566762399	6.109934	0
4.932739611	7.967685894	6.566762399	6.109934	2.62842
1.190159368	0.878629367	1.091056308	1.463323273	0.537346
7.310252808	5.82111107	7.011846832	8.061953015	3.982705
4.14265	2.801256	3.558250066	3.873515	0.937203
4.14265	2.801256	3.558250066	3.873515	0.937203
135.3404681	129.8812834	150.1082613	120.2958696	31.02328688
135.3404681	129.8812834	150.1082613	120.2958696	10.8613
70.39133042	71.03265153	93.82774196	94.93945713	133.70959
9.98841	8.88938	10.12257	10.67264	27.9956
41.5262304	35.18807095	23.55580631	24.7035344	3.487445
41.5262304	35.18807095	23.55580631	24.7035344	5.98647128
0.328701	0.217901	0.524545	0.280818	11.9867
17.62420399	16.60144754	11.33711333	8.8214531	5.55937
17.62420399	16.60144754	11.33711333	8.8214531	5.55937
12.57098	12.82465	19.05788	13.25153	72.232
6.74185406	8.13932303	11.2681547	9.8444947	2.32767
6.74185406	8.13932303	11.2681547	9.8444947	26.795354

6.30518	6.152531	9.84437	8.043153	43.7514
59.94661	56.82357	40.335115	48.16759	20.09598
7.48374	8.7306	10.3286	9.42153	38.84025546
11.76073	13.57019	15.18762	13.75149	28.742
116.9387	111.6359	137.1854	109.3633	48.2169
3.97421	2.44284	1.3979	4.29534	1.47372
3.794031806	3.17109865	23.080368	22.57675033	26.34032
14.0925	15.8159	15.26	16.0863	41.2633
2.04173	1.01101	2.00256	0.0302621	0.800846
4.278576579	4.927234	3.393308387	3.528574705	1.450220153
25.15287741	23.036045	15.19999	18.0407	4.317611
9.472076111	12.1099257	19.76363298	18.10527042	17.02666621
4.8261381	5.102014432	8.72122019	7.86519	11.689696
2.43791152	1.874367401	2.2663847	2.97664217	0.413662
19.972	22.8162	44.3394	45.8008	73.4292
5.556628	5.791052	9.758340019	7.396802816	3.33092
5.556628	5.791052	9.758340019	7.396802816	18.0808
0	0.093525366	0	0.0408647	4.09621
34.93301	32.674	29.19214	29.23696	9.66269
14.84082	14.37255	14.15082	11.9342	13.42477
2.44378	0.579782	22.7159	35.2491	18.9397
3.08606	2.4762	4.39519	6.3781	38.0869
28.34140384	26.2907	47.52398583	43.76847229	37.133169
28.34140384	26.2907	47.52398583	43.76847229	37.133169
30.7622141	23.54066964	5.615058503	6.536828338	0.735930337
0	0	0	0.138583	1.88311
3.44819	2.91859	1.25969	2.66471	0.144407
40.34708	41.27741	39.22984	40.33836	53.420269
105.014	88.9011	16.9154	22.3339	0.847268
34.01058932	41.03125003	42.6438927	45.81370525	37.12545682
50.94787	50.47497886	47.533651	42.353363	11.747729
2.749087	2.381749	4.72135	5.6744948	61.769788
4.783228481	3.294560644	5.684486	6.848383637	42.43997
4.783228481	3.294560644	5.684486	6.848383637	42.43997
0	0	0	0	0.205379
0.027135038	0.027325774	0.106967598	0.52257792	8.53838
3.959663006	2.898688836	11.35964	11.54442	0.95435
3.959663006	2.898688836	11.35964	11.54442	2.77971
16.72714	21.19259	21.2293	20.9372	271.6001
22.8022086	22.1352079	29.25137817	30.3811991	30.142352
16.28134992	17.87142391	23.92879324	19.1964208	99.31529793
0.0537389	0.000101543	6.87E-05	0.000238978	6.78E-05
17.62264	22.1482	24.6865	23.4569	41.23692423
16.73001316	20.55986901	16.74653921	17.14048867	31.8975

0	0	0	0	0.0838422
47.153202	46.22940586	52.03882222	48.8284	21.42130038
0.161306	0	0.316328	0	0.189328
0	0	0	0	0.083753
0	0	0	0	0.223248
5.96799	4.33489	15.9537	32.0701	12.0667
54.21233	56.05345007	36.37460005	38.14814	75.15730664
54.21233	56.05345007	36.37460005	38.14814	75.15730664
2.44911	2.6523	1.63576	5.11136	6.25249
1305.777	1326.023	1551.245	1294.534	874.445
168.793474	163.50508	86.912218	88.57814	26.26325
1.411662	2.130025	4.245922	2.62712	1.915351958
11.9429	11.7993	11.943219	11.982295	27.47096
0	0	0	0.236084	0.20217
0	0	0	0	0
3.575286	2.10678323	5.799044003	5.77324	28.96906
3.575286	2.10678323	5.799044003	5.77324	28.96906
77.70767	79.84654	101.08854	73.95132	77.0899
2.166171115	2.42930045	3.636821	2.801069848	6.71299222
16.54773	20.71923	27.5937	26.1289	57.52858
7.161233543	7.836931275	8.673581635	7.30545682	18.96371366
214.529	237.661	177.947	153.494	27.9143
24.74618	25.42205	31.01792	41.761	35.46116
443.8951	415.9919	549.016	458.4778	411.9508
10.023901	13.41842714	16.99684	13.440401	55.94666
611.259639	539.293804	476.61924	452.0153988	26.0117
611.259639	539.293804	476.61924	452.0153988	216.2983
9.863331	9.028652123	9.809441018	11.48947922	6.56852
0.459670023	0.183331992	9.14152	16.81068	20.1577
0.502651	0.378747	14.8313	27.4777	29.8407
57.27982801	54.98435316	50.95124288	51.17145459	49.568
57.27982801	54.98435316	50.95124288	51.17145459	33.58925002
17.39914	15.99924	20.57396	17.4182	28.72896
56.45623285	59.20407078	63.7252657	76.2608374	105.09691
0.000157991	0.00109975	5.06E-05	5.81E-05	0.000498955
139.89821	133.39314	141.80672	158.38471	144.5741
17.58228474	14.6330734	7.395469692	8.540760593	1.324840526
35.2078	36.21754737	17.3804	22.82192	20.93747
35.2078	36.21754737	17.3804	22.82192	20.93747
0	0	0	0	0
0	0	0	0	0
0.0681307	0	0.0666478	0	0
24.79119075	23.42651017	43.87052	35.90350847	100.6727
16.88730912	14.16139	20.76302	23.57828	69.7476

16.08288	18.08707	23.1791	20.7269	19.18525
7.06778	7.16906	5.83782	5.22058	18.0753
30.4728	34.9853	22.48917	20.45469	6.141594039
9.55652	11.627815	21.13676	25.032672	31.226579
0	0	2.03363	0	0
1.89396	2.15612	3.69402	6.12734	75.6143
13.04818	12.97718	21.51026	21.83835	35.3369
6.42653	6.25998	6.42768	8.09183	21.8763
17.3692	16.25106	25.75809	20.03200331	30.3755
17.3692	16.25106	25.75809	20.03200331	84.4069
17.3692	16.25106	25.75809	20.03200331	382.803
14.4895618	16.8396588	9.680420421	8.886010305	10.27578049
1.149457983	2.343343258	4.577701751	2.327741333	1.968811197
1.149457983	2.343343258	4.577701751	2.327741333	1.968811197
5.9329	11.6959	8.41013	6.7351	6.43E-05
162.073118	142.5963681	251.477655	270.71397	61.71904593
162.073118	142.5963681	251.477655	270.71397	61.71904593
162.073118	142.5963681	251.477655	270.71397	157.1151
19.317657	21.18237375	17.10274	13.63465536	0.16621
19.317657	21.18237375	17.10274	13.63465536	3.930895346
22.082642	24.903819	20.81651898	19.32085244	3.396889343
10.6075	13.4824	13.8135	11.7942	43.1646
23.318	34.6257	38.3447	32.2107	53.4084
45.4502	47.73	50.4219	49.643	54.4301
34.904011	32.28359	21.08569	22.72516	4.363054689
4.72862	2.89509	8.68225	7.12324	4.93427
1.01062	1.02444	0.683027	0.409579	0.820552
10.574	15.8524	13.9393	12.3143	0.000256377
8.53793	10.22368	8.30511	8.62386	34.78501
14.33281	12.89983	11.980073	11.66562	4.468669
32.2001	25.1648	26.9828	27.3684	0
0	0.045587	0.0878507	0.235165	0.108843
42.64782291	39.5939141	31.47427515	25.31878544	7.522321452
0	0	0	0	0
7.80849	6.64077	12.16751	18.35118	51.4201
5.09252	4.377216346	7.00822	10.04523	5.99552
0	0	0	0	0
0	0	4.93832	0	1695.11
50.3649	32.2318	259.96	97.5101	348.934
0	0	0.181821	0	0.450088
54.28128835	56.42387955	34.69664304	30.6856159	4.03709
54.28128835	56.42387955	34.69664304	30.6856159	9.698141026
52.06255	38.184485	42.03528	35.787421	12.71377
52.06255	38.184485	42.03528	35.787421	12.71377

10.17124859	11.7020729	15.1670994	16.5366972	42.31215
28.9065	30.84365	27.21983	24.82751412	58.2129
0	0	0.927244	0.420799	0.723701
0	0	1.9114413	2.325025	0.505823
2.023261354	1.961530007	5.460751	3.750547245	3.276621203
2.023261354	1.961530007	5.460751	3.750547245	3.276621203
0.987990519	0.923609	1.65710315	3.142462	6.69032
2.202384324	1.65957201	5.619583	4.90480414	3.870398693
2.202384324	1.65957201	5.619583	4.90480414	3.870398693
9.50911	7.69031	14.65387	23.25647	37.64275
26.560743	31.83608111	27.76227994	26.15276393	30.74433
7.03713	8.66072	2.83916	3.526358	0.886916
0.870559	1.60425	0.758083	1.23723	181.848
15.060962	13.395016	14.194998	11.26932991	45.22976
40.5043	37.0926	41.8941	36.197	67.4914
0	0	0	0	0
18.47502468	17.86171007	21.36832162	20.80883882	67.753001
146.4337	153.9458	54.97555	66.5593	47.97122
24.06888823	24.92101772	33.3403046	35.352438	38.58661
4.792265681	6.389515071	8.939377733	5.875588287	6.774802085
12.19518	14.66978	18.86427	16.48343	18.5081
2.45901	4.9632	5.70792	4.71611	5.64492
43.22161971	45.47244475	42.29842054	38.63743018	64.698577
14.37037146	16.12409304	18.07256489	17.37846	29.0419902
14.37037146	16.12409304	18.07256489	17.37846	15.47110137
14.37037146	16.12409304	18.07256489	17.37846	29.0419902
12.97723	13.56751	14.84876	13.79234	20.5691
0.0997675	0	2.42062	8.26795	4.79618
1.677860173	1.147002881	16.52838456	11.08535667	17.28489792
1.677860173	1.147002881	16.52838456	11.08535667	17.28489792
7.34058	5.51737	4.39287	3.57007	0.274657
17.84581672	18.38010294	19.41846419	20.25893343	46.20050045
17.84581672	18.38010294	19.41846419	20.25893343	46.20050045
20.58453874	15.27715016	16.91776555	19.00961	30.72818
50.2922	57.2267	68.8166	52.4344	78.5448
9.857603093	12.742293	15.80552548	11.96775425	65.9446
9.857603093	12.742293	15.80552548	11.96775425	4.80351
4.81877583	5.87141845	6.958515271	5.747333311	30.894397
9.776787627	9.029612113	14.603411	16.8676	138.1597
0.9230249	3.08446	0.629196	0.9658625	175.0676
10.55736	12.471583	2.588515733	2.999515759	66.584
20.853289	21.39928847	27.38662489	23.53934306	111.0301
58.94654476	54.66855536	36.10261667	40.58594754	13.79849346
58.94654476	54.66855536	36.10261667	40.58594754	13.79849346

24.662893	20.4892002	7.785532	11.14940011	1.923800758
3.303885	4.642479	8.755856	6.79387	27.07976424
1.344879426	1.923671464	1.657096601	1.984681467	5.21147
0.457779	0.514352	0.0503217	0	91.5998
2.049365	4.311	2.024579	4.84577	380.4922
5.6719	4.2446	8.65948	7.96322	11.5543
15.14171695	11.28952905	6.328387815	9.602086768	2.326727989
15.14171695	11.28952905	6.328387815	9.602086768	1.31352
1.79799	0.548524	1.04978	1.44686	0.656304
16.366108	18.0083798	9.185941796	11.19805184	0
1.62408	2.54645	2.99812	1.18754	0.557033
16.366108	18.0083798	9.185941796	11.19805184	0
16.366108	18.0083798	9.185941796	11.19805184	0.596132
16.366108	18.0083798	9.185941796	11.19805184	6.071841206
9.495432524	11.92167858	9.94884	8.77699183	4.56437
7.61366	2.35912	0	2.01651	0
0	0	2.06687	6.73039	4.75543
32.1396117	31.95664538	11.36210402	11.61260335	0.422294
6.229892354	8.931922745	2.572684	4.511189	9.8833208
6.229892354	8.931922745	2.572684	4.511189	9.8833208
1.41912	1.7772	0.677835	1.39211	0.816495
0.271505	0.136599	1.52447	2.13827	0.711209
0.03857165	0	0.628951	0.716011	0.247044
0.0521118	0.105853	0.309651	0.138515	0.187877
1.571090194	1.185831599	2.70767648	4.248002557	0.521778
1.571090194	1.185831599	2.70767648	4.248002557	0.486382
10.06745	8.09065	1.215017	1.977346	0.455184
25.34603	22.67534	4.60581	7.19337	0.447976
4193.198	3132.321	825.483	957.068	20.1692
4193.198	3132.321	825.483	957.068	46.067
1.0517373	5.32E-05	5.85E-05	1.18651	1.278703932
0.215485	0.213409	0.797655	0.273045	102.328
11.38446	11.98465	18.39264	15.85711	47.5267
0	0	0.165207	0	0
87.56375	97.70895	68.97842212	72.29680196	0.000502204
0.320098	0.0803118	12.9004	30.0042	31.1967
205.3056	180.7096	11.85391	49.21677	3.82657
8.593352	3.656847	8.428924	4.149027	68.465
0	0.249716	0.181117	0.244038	1.755800291
64.7842	65.9957	103.197	112.942	104.209
0	0	0	0	0
0	0	0	0	0
0	0	0	0	0
0.973553468	0.799490533	1.676860863	0.854259389	0.0840054

3.10476	2.49608	5.52757	4.4295	3.43145
3.84484547	3.621069	7.525658625	6.52913	2.827237077
0.434246213	0.168004773	0.256356542	0.485413329	0.289929045
0.203108	0.203488	0.386308	0	0
4.372790977	5.316172	4.63037	8.66604	9.167865
10.7362212	10.925192	10.55728	12.58437	41.2498
73.143526	72.73364	52.588551	61.17243399	49.33662048
37.7421475	39.325061	28.49186	27.36650112	1.60577
27.36692	22.915059	27.8471945	26.339161	51.9299
37.7421475	39.325061	28.49186	27.36650112	5.30052
1775.837393	1047.697264	620.541311	700.97245	19.6359
26.34924509	26.71326673	36.61825294	30.10032233	49.6971
26.40632	25.42759	30.7444	32.83129	45.495
8.935149404	8.697805002	11.56994695	10.516578	40.68411
0	0	0	0	0
0	0	0.295457	0.397752	0.213839
0	0	0	0	0
0	0	1.302792474	1.978235	1.676762579
0	0.0707886	0.278634	0.0929414	0.170705
0	0	0	0	0
0	2.53E-05	0.285524	0.574512	0.398272
0	0	0	0	0
0	0	0	0	0
0	0	0	0	0.0976321
0	0.782749	0	0	0
0.119625	0.123359	0.238507	0	0.137138
0	0	0	0	0
9.690547355	9.95955278	11.51219067	10.81203625	20.901693
0	0	0	0	0
0	0.111237	0.00217273	3.72E-06	0.000909595
32.15145534	30.41182216	28.60951157	29.81531902	39.31992035
13.37114	13.21103	20.43091	16.26572	34.69685
13.37114	13.21103	20.43091	16.26572	34.69685
48.39588	50.33095	61.3202	51.3117	76.7134
28.4418	35.3602	49.7912	40.0057	187.496355
0.4355156	0.481362035	1.03806904	1.141963	0.858666
0.614332	0	0	0.771479	0
15.0841	16.4976	33.027	36.2906	53.8119
10.5474	13.0657	5.76309	10.5434	2.94465
17.617451	15.994164	24.883349	24.04738	23.9261
0	0	0	0	0
0	0	0	0	0
0	0	0	0.136732	0
0	0	0	0	0

0.0397313	0.0407865	0.199549	0.108679	0.0951848
0	0	0	0	0
0	0	0	0.0785124	0
0	0	0	0	0
3.92265	4.88307	7.94899	4.35842	0.000167185
3.20116	4.10591	7.42388	4.88007	3.63566
10.65474	11.74091	30.4815	20.84527	8.90094
0.0850602	0.170889162	0.811677	1.9780008	0.259046355
0.0850602	0.170889162	0.811677	1.9780008	0.259046355
4.236879896	3.1191517	6.826897975	5.004507588	8.496508065
0.204339	0.415149	0.583363	0.815706	0.485563
6.48E-06	0.186893	0.443011	0.57606	0.000149977
10.7776	10.7571	23.5752	21.437	10.4825
7.701830807	8.26360346	8.098769001	8.2757547	11.83978
0	0	0	0	2.28455
2.271398797	1.7198832	1.589471635	1.055182106	0.164252
2.271398797	1.7198832	1.589471635	1.055182106	0.614338
6.63346	7.529373	13.99109	13.81237	37.9537
0.98282	0.726567	0.809141	1.38142	0.273303
0.799508	0	1.58963584	0.68105	2.439485865
0	0	0	0	0.000595303
17.53827	21.57173	20.16874	18.108071	42.10796
35.54935805	37.17889	33.22091235	32.95694971	36.07454
32.28051204	30.15945902	28.16404482	24.042	26.62573914
32.28051204	30.15945902	28.16404482	24.042	49.8507
6.375598	6.09176	9.51859	6.71996	15.6903
63.01325	64.94398	80.86265	72.40942	30.385245
0.420527	0.4058776	0.497004	0.49346234	1.13794
18.27038	19.84613	60.82908	60.00257	57.1183
0.214564	0.0433014	0.170552	0.0570088	0.562918
0	0	0.975736	3.65049	15.3235
6.18913	6.19134	16.9748	18.1327	32.0317
0	0	0	0	0
0	0	0.0613716	0.0811446	0
0	0	0	0.334349	0
0.220505	0	0.382878	0.800869	0.134532
525.036981	533.6474417	661.1269666	520.7170518	5.136368446
525.036981	533.6474417	661.1269666	520.7170518	5.136368446
525.036981	533.6474417	661.1269666	520.7170518	247.015
0	0	0	0	0
5.42553	6.28032	4.155195	6.311448	5.7787
12.596	14.5524	19.2185	16.5193	93.0228
30.5542	29.2008	34.794	33.0129	4.88034
6.382824986	4.502079459	4.714592039	4.078578271	20.13949

6.382824986	4.502079459	4.714592039	4.078578271	0.448062
0	0.063161	0.061741	0.0822878	14.42408747
0	0	0.0931049	0	0.615742
0	0	0.18989	0.516876	0
0	0	0.0917603	0	0.0554186
0	0.0480021	0.0469942	0.0627338	1.20414
0	0	0.0551762	0.0745933	0
0.41673	0.453736	0.666435	0.764235	0.531527
0	0	0	0	0
57.82612	54.99379	34.31186	33.10459	7.31273
9.21424	9.5717	9.58017	11.52057	32.84857
92.86955001	85.22200032	15.61430559	22.1059882	2.152171974
92.86955001	85.22200032	15.61430559	22.1059882	2.152171974
20.12586	12.4992	10.62989	11.41971	0.282539
163.9307	151.0714	178.6359	192.1548	134.1891
0	0	0	0	0
28.675	30.4602	28.6146	30.0544	79.80091
3.47358	3.292176	5.05125	5.75888	13.7004
286.345	216.244	35.0733	28.5654	3.70723
87.1915	65.4731	16.4591	18.2775	3.6249
1581.97	1062.37	162.328	138.177	10.5643
0	0	0.79	1.80135	2.2619
24.077674	31.676205	41.026868	40.69186	31.48019
93.80726949	93.39973871	76.04963242	73.18678334	50.2055522
21.72758442	19.721487	16.48853931	15.17790282	1.97582
24.5783	24.0547	31.8954	34.3986	41.187029
13.995308	15.693333	16.96224189	18.0265045	39.34727782
28.4328	23.89595	29.67342	29.951365	49.1245
28.4328	23.89595	29.67342	29.951365	16.6256
269.2175	275.2459	275.3261	225.6863	169.3005
14.76947	13.58898	11.11058	11.03536	3.53748
0	0	0	0	0
0.0847128	0.174309519	0.5243062	0.1437208	0
0.0847128	0.174309519	0.5243062	0.1437208	0.0670255
16.88715	16.6128143	14.09949816	14.552865	64.0108197
16.88715	16.6128143	14.09949816	14.552865	4.23629
16.88715	16.6128143	14.09949816	14.552865	5.03517
751.632	717.723	963.96	796.891	430.361
544.5572965	518.2763147	354.69673	337.08512	199.8255
24.56190001	23.43300201	18.47162148	19.45951228	20.74936
38.6323	39.40812	39.55123	45.012247	45.48425
15.5673	12.1055	1.22492	1.11089	0.83124
23.8324	27.0641	20.52351	16.7589	44.8652
25.96938	29.59405	26.26021	19.16714839	51.736

1.736958	1.930117	3.76166	3.84825	57.76641936
0.287823153	0.182080266	0.05234018	0.176630055	16.6254559
0.287823153	0.182080266	0.05234018	0.176630055	16.6254559
0.225087	3.01E-06	0.203555	0.36404	0.313739
0.63959	0.571188	1.67E-05	0.000110979	1.51E-05
0.464317	0.0974641	0.520231	0.0854724	0.494815
6.13687	5.81039	9.34851	7.5824	34.40718137
197.16132	180.14884	133.17075	121.06897	27.42757
0	0	0.125603	0	27.42757
197.16132	180.14884	133.17075	121.06897	27.42757
3.24468	2.17418	4.02973	3.51989	14.3041
0	0	0	0	0
0	0	0	0	0
0	0	0	0	0
0.416911	0	0	0	0
0	0	0	0.15356	0
0	0	0	0	0
0.153406	0	0	0	0.0615652
0	0	0	0.179275	0.0833442
0	0	0	0.113673	0
0	0	0	0	0
0	0	0	0	0
0.0976054	0	0.0479009	0.129297	0.115102
1.701065	2.060532	1.601580541	2.0544	1.52382
4.08E-06	8.24E-06	2.54E-07	5.74E-06	0.401448
0	0	0	0	0
0	0	1.976368	2.08492511	4.49531
0.78086	0.783415	0.247844	1.54196	0.462168
1.4363	1.33631	1.23247	1.75233	0.271385
0	0	1.976368	2.08492511	2.004211084
0	0	0	0	0
0	0	0	0	0
0	0	0	0.232874	0
0.115519	0	0.148439	0.0991601	0.181734
0.0220332	0	0	0.0293138	0
0	0	0	0	0
0.108575	0	0.107694	0.292434	0
0.142508	0.143397	0	0	0
0	0	0.123368	0.0822795	0
0.0873652	0	0.0285632	0.114149	0.108252
0	0	0.0856235	0.959768	0.24252
0	0	0	0	0
4.76734	6.1929	9.58692	6.48038	16.2257
5.49174	5.65568	20.9885	9.12343	22.6128

36.79037	33.6595	27.1445	35.33818	53.2321
1.499954425	1.204407239	2.087756529	1.534327932	31.7706
1.499954425	1.204407239	2.087756529	1.534327932	31.7706
16.1982025	18.9144122	29.84074331	29.7775025	50.65310715
75.63634303	80.29766268	55.55672864	52.2776	43.62609274
46.68132	39.93457	32.69902	36.21363514	26.72627
7.788505	10.17467	9.816007692	9.10648866	14.0973
17.67124173	20.042727	20.703797	19.03169004	40.1867
1.0393	1.06518	0.986253	2.28288	0
34.69546754	28.64716	15.6221151	18.38175401	0.973996329
0.0194527	0.0193389	0.137226	0.1174293	2.515959544
0.0194527	0.0193389	0.137226	0.1174293	2.515959544
46.4526	44.7613	60.943	48.254	27.4455
4.743245	4.41837563	6.062517	7.28864505	27.23372
16.66363	19.37535	24.8707	20.95122	6.49042
2.668396	3.348875	1.081828047	0.8322867	202.526
2.668396	3.348875	1.081828047	0.8322867	166.69793
0.379155	0.1270305	1.193937	1.180386	0.660144
17.9314	26.1606	19.2843	19.9937	54.46474055
9.63203386	16.675652	15.96112039	14.08436332	30.5213
5.87631	6.37654	11.41604	7.59083	36.0155
34.29964343	37.254363	32.43912005	33.02613075	39.31276
1.56368	1.2346	2.68584	3.50631	18.5455
7.570651422	7.014641017	8.145197991	8.791122874	10.58335519
7.570651422	7.014641017	8.145197991	8.791122874	10.58335519
7.570651422	7.014641017	8.145197991	8.791122874	10.58335519
29.49643	31.87603	33.68253	29.43595	117.6520354
0	0	0	0	0
0	0	0	0	0
0	0	0	0	0
0	0.512211	0	0.220612	0
0.308251967	0.19426707	2.905227709	5.241129671	2.514692993
0	0	0	0	0
87.45160056	84.93610543	58.2289	61.70430057	29.06430078
1.612020588	1.624290132	2.490400158	2.507260008	21.642655
6.3207	14.1193	19.669	17.7847	15.5896
640.9152119	715.4900735	999.0540907	943.3866078	1107.678
0.5341321	0.4090576	0.563771	0.0379272	0.587719
0.5341321	0.4090576	0.563771	0.0379272	0.73692
0.5341321	0.4090576	0.563771	0.0379272	1.87544
1.84E-99	8.91E-83	3.35E-35	4.60E-121	0.669119
19.7808	20.0857	25.8526	31.4149	26.9705
4.13516	3.58223	4.85785	4.87642	4.43321
0.0295901	0.148292	0	0	0.033856

1.24399	1.25928	6.30095	2.14356	1.41933
0	0.300213283	0	0	0.134764
23.0451451	25.03877154	24.24177528	28.154416	10.7124
23.0451451	25.03877154	24.24177528	28.154416	17.1568
0.00103172	0.0876969	3.55367	4.24613	6.1765
47.97260247	38.58460346	77.99099	84.28627842	57.6478
1.648887	1.16667	1.4638	2.24993	1.50977
4.83965	6.49037	5.14508	4.1882	24.386
14.806628	11.81740048	2.173250748	2.007101101	0.310073
9.29704	8.36894	1.34784	1.94107	0
1024.85	854.551	145.459	183.566	13.8124
133.9487148	91.36982436	46.04900122	33.67631248	3.0407
133.9487148	91.36982436	46.04900122	33.67631248	75.93042
133.9487148	91.36982436	46.04900122	33.67631248	75.93042
133.9487148	91.36982436	46.04900122	33.67631248	0.532511
133.9487148	91.36982436	46.04900122	33.67631248	1.34127
1.807547522	2.076879	3.242482	2.593935304	1.895570197
3.45639612	4.023995907	4.950338034	2.569372488	34.80944
40.4127	41.7987	60.5358	74.2417	267.252
0	0	0	0	0
0.921363	0.604197	1.99056	2.5148	36.9053
0.340453948	0.076569672	2.4954083	6.319626779	2.5386118
0.340453948	0.076569672	2.4954083	6.319626779	0.535942
0.108078477	0.06757626	2.2134986	4.022126033	2.368983471
0.146810012	0.239654747	2.428316148	5.473167129	3.708455861
0.146810012	0.239654747	2.428316148	5.473167129	3.708455861
245.1488	253.9807	159.1684	123.3405	87.81092
23.89698412	26.05629078	1.86954706	5.106748474	8.09067
0	0	0	0	0
0	0	0.422895	0	0
8.069673311	7.95966113	8.844525706	9.263132842	46.95883
1.279211629	1.083766162	1.363122018	2.095169	15.7525894
0.0627244	0	0.0307365	0	0.0375155
0	0	0	0.389902	0
0	0	1.07402	0.37349	0
0	0	0	0	0
0	0.0852914	0	0	0
0	0	0	0	0
0	0.138392	0.802837	0.54196	0.332639
0	0	0	0.203446	0
0	0	0	0	0
0	0	5.25	0	0
0	0.469184	0	0	0
0	0	0	0	0

0	0	0.758393	0.685007	0
0	0	0	0	0
10.933319	6.91951908	85.07443029	53.81441294	28.7504
57.84081	47.3691	26.25854	27.76747	10.78218108
30.5168	27.666	25.5885	17.7341	13.4374
57.84081	47.3691	26.25854	27.76747	10.78218108
5.0007	9.61545	17.95721	23.47411	269.377
5.0007	9.61545	17.95721	23.47411	239.868
0.0710483	0	0.1391	0.279804	0.0849638
0.0579515	0	0	0	0
0.462625	0	0	0.591843	0
0.6410511	0.277038751	0.312113	0.265067093	0
0	0	0	0.415383	0
0	0	0	0	0
0	0	0	0	0
0.150277	0	0.0955419	0.193122	0.0599543
0	0	0	0.251566	0
0	0.13662	0	0	0
0.180919	0.182683	0.177455	0.236828	0
0	0	0.241993	0	0
0	0	0	0	0
0	0	0	0	0
0	0	0	0.108793	0.196049
0	0	0	0	0
0	0	0	0	0
0	0	0	0	0
0	0	0	0.248944	0
0	0.104561	0.0690579	0	0
0	0	0	0	0
0	0	0	0	0
0	0	0	0	0
0.0726289	0.220699	0.036116	0.0482538	0.0454565
0	0	0	0	0
0	0	0.171436	0.23124	0
0	0	0.0627183	0.167347	0.301442
0.187958	0.187578	0.180586	0	0
0.375515	0.376591	0	0	0.935954
0	0.167379	0	0.644871	0.196991
0	0	0	0	0
0.451261	0	1.65E-07	4.34E-07	1.06E-06
0	0	0	0.387559	0
0	0	0	0	0
0	0	0	0	0
0	0	0.797471	0	0

3.78442	0.805748	10.8854	2.62867	4.28511
2.456181	3.05533	4.16597	3.218489	1.47043
34.818	24.9749	41.9809	44.0069	172.019
0.0956377	0.418391	4.62599	9.23656	9.48801
14.7951	15.1364	23.4821	19.8198	6.00464
48.78860249	48.93340762	39.89780777	36.37460693	20.09820222
0	0	0	0	0
123.2635663	79.12339184	33.58163142	44.87704613	1.808599
32.19571133	33.60191655	23.23401586	23.82120569	9.195691528
39.65354	37.42776	31.02662	31.02433	27.24245
39.65354	37.42776	31.02662	31.02433	27.24245
11.2701	12.6928	13.5386	10.6415	2.44286
1.8621	1.68285	2.83954	2.49115	2.25507
0.0934522	0.0957698	0	0	0
78.728176	76.37592	54.05832339	58.18097301	34.59699744
51.09194	53.977213	65.59527	51.28522	99.3499
51.09194	53.977213	65.59527	51.28522	16.68697021
64.73216434	68.02886764	56.932225	55.76142197	73.36967866
2.854820024	4.420801177	8.43242	5.620474886	4.859947263
12.02369	14.03287	15.89045	14.9203	95.9746
4.473278	4.802806	5.947625588	4.768258	3.16318
112.9338335	119.1656742	98.995035	92.69780145	62.39573462
35.846582	44.877649	4.293880578	7.579288	11.26979301
2.615710549	2.7158612	4.422592221	3.39758919	88.30546163
2.615710549	2.7158612	4.422592221	3.39758919	88.30546163
7.423919	3.682409366	11.8730274	8.661984723	4.665920577
7.423919	3.682409366	11.8730274	8.661984723	1.80563
0	0	0	0.572154	0
42.91498528	41.82407872	42.05538282	43.082217	40.25517123
42.91498528	41.82407872	42.05538282	43.082217	40.25517123
8.3786289	5.821783982	15.33392619	14.80505475	3.59351
18.426472	15.787978	4.324480661	7.802818495	1.56722
2.467737822	1.605993237	3.920582	3.748572878	46.2136
610.3213254	475.4431838	123.759654	136.7629888	9.21993
610.3213254	475.4431838	123.759654	136.7629888	0.706892
610.3213254	475.4431838	123.759654	136.7629888	11.078357
1.299282007	0.335092957	16.0429901	32.15043577	31.78883
25.00958402	26.35024514	29.3071149	26.69264375	39.24330557
0.206603	0.210624	5.49472	6.65009	3.17892
3.90178	7.22284	12.45817	9.28237	4.11416
54.35115	47.439257	75.05003062	62.54826995	31.4388
6.91005	7.38578	0.715719	1.549955	0.110344
6.94E-07	2.82E-22	0	3.72E-06	0
131.68781	139.61118	135.68946	145.798098	223.3390458

31.1012055	36.162592	31.51582032	33.34537	37.88891
2.71809	3.24786	1.56285	0.979865	203.349
9.561831088	10.485676	7.300355669	6.062915635	2.742072164
23.734058	11.78076667	31.54082692	24.930079	10.93031859
23.734058	11.78076667	31.54082692	24.930079	10.93031859
95.9792	87.4928	16.60993	32.6964	0.745422
6.385983121	5.89556884	10.22246839	9.353753	25.43151376
18.5961	15.9669	18.9934	17.7977	30.0988
19.9833473	23.96274788	26.6291285	24.37580072	15.45835209
19.9833473	23.96274788	26.6291285	24.37580072	15.45835209
19.9833473	23.96274788	26.6291285	24.37580072	18.7067
27.10354816	27.14171958	16.52808951	17.1808185	47.252105
24.034194	21.744357	17.73266876	19.4405053	0.758783
24.034194	21.744357	17.73266876	19.4405053	1.56122
24.034194	21.744357	17.73266876	19.4405053	5.9438051
12.456856	14.221476	9.895897	10.843752	4.116249
31.30779684	67.43631	76.69835	59.3572	5.24392979
16.7337404	17.46553641	27.748386	25.94495313	80.15365
4.3567711	3.587844582	2.142146608	2.669933358	0.933530391
1.43004088	1.481965803	1.0020709	0.91430981	0.334544
88.1289704	43.58513	43.19816349	48.7372	2.63708
24.1406	18.4012	177.522	159.353	67.5258
8.50881	9.95481	29.8224	29.23041	67.1811
3.591849868	4.321017589	0.977079977	1.91758016	0.288667
16.413599	16.53142895	25.12347852	19.78591035	23.51604
38.556316	39.08909	41.05752	42.76012	49.79503
52.763856	50.59908876	19.22429	23.05745142	0
52.763856	50.59908876	19.22429	23.05745142	0.341978893
52.763856	50.59908876	19.22429	23.05745142	0.341978893
52.763856	50.59908876	19.22429	23.05745142	1.963811074
4.864173224	5.806877	12.18578	9.735241	18.45721795
19.69331228	27.5949695	42.06713	34.19496922	81.6147
0	0.153164	0.146193	0	0.878327
0.162529	0.216935	0.574885	1.25175	0.190289
50.38096001	52.68257	53.41881	57.063646	35.560828
29.80432925	31.41177	13.20978	17.17937	18.07554231
32.7759	30.89265	21.87531	23.06371	49.96136
9.17581	10.10977	19.0211	12.37209	118.3792
18.842638	14.23833134	7.099985355	9.869288	0.415052
96.5444	109.0538	65.92795	67.77802	143.033
92.21331323	101.0370164	65.47090793	64.84460515	88.64000863
25.620092	27.8519	29.45521486	21.23137699	5.7681
19.96475	23.5596	29.58795	28.915979	15.40779
19.96475	23.5596	29.58795	28.915979	15.40779

19.96475	23.5596	29.58795	28.915979	81.8323
19.96475	23.5596	29.58795	28.915979	32.6092
0	0	0	0	0
0	0	0	0	0.141304
12.92845	12.12998	18.18523	18.21533	31.92449786
37.848498	39.5364	61.1848	56.8039	30.9149
19.24677	13.27405	12.06552	10.95695	2.82131
21.946344	23.66844	19.864594	19.326594	20.4884
0.795573	0.648026	2.058722	2.363844	13.49704397
31.08954	33.33356	42.6876	41.5609	68.1221
34.56990801	37.25346655	33.70126789	38.09559044	19.2256138
36.73872629	35.90573373	33.83213496	36.13223	34.4471255
35.839469	34.783764	35.780278	37.64729	25.12326
35.839469	34.783764	35.780278	37.64729	25.12326
35.839469	34.783764	35.780278	37.64729	25.12326
26.544	36.0823	21.6258	23.3052	23.8696
6.748714	7.628746561	6.725750044	5.35952948	32.934754
2.391726	0.780479	0	0.333347	0
0.649602024	0.543947	0	0.080574	0
9.42467	8.557706	7.4820947	8.331354068	5.37223991
25.66741	23.62778	21.02823	21.14479	9.358551138
0.217495	0.301935	0.105403	0	1.73075
5.143811	5.125247	4.755417048	5.419654	7.842748
16.34399734	9.877709095	22.77611792	19.23938825	26.2749
16.34399734	9.877709095	22.77611792	19.23938825	6.11772
20.060737	11.38199314	37.201806	22.03109044	4.865371
1.11317	1.81661	9.01E-07	0.131254	126.473
1.0272	0	7.04722	32.7734	40.905
17.228	18.45892	11.518296	13.95041	4.527388944
56.2948	56.2898	45.5759	49.9108	152.2929
0.169761397	0.121332	2.703092	4.371969	1.9457033
0	0	0	0	0
13.82478378	14.74834056	12.93156026	13.394204	19.04993
1978.6292	1923.6444	137.32222	269.41517	24.99730591
2.99308	3.29661	5.59615	5.21076	30.7753
17.39978116	20.716734	20.22705661	20.8156	32.58866
0.819079	3.66905	1.661409	0.851329	0
64.8169	63.0811	61.9749	53.0629	63.3723
0.461777	0	0.770575	0	0.206578
49.59139387	54.4406352	70.50402904	71.74717967	73.57593366
49.59139387	54.4406352	70.50402904	71.74717967	73.57593366
0.313901	0.124595	3.98398	7.72986	4.05664
4.301674	4.819257	6.704843	10.45994777	14.5164
0	0.407776	0	0	0.874614

0.12489	0	0	0.158601	0.282626
0	0	0	0.186456	1.03018
7.18274	6.28016	5.17216	3.68267	75.527
21.64035373	21.09026205	14.98092075	13.76110645	4.091409586
57.014752	62.770404	128.40475	83.124091	148.353272
26.871761	29.7600193	68.12132521	66.9334478	8.960259256
26.871761	29.7600193	68.12132521	66.9334478	8.960259256
132.3016	150.7767	95.0845	100.7525	112.1124
0.158046	0	0	0	0.000918739
0.219243393	0.485175	0.758917	0.871378	0.296866
0.219243393	0.485175	0.758917	0.871378	0.306401
36.24266787	37.34934849	30.69932964	33.93261303	131.5576121
36.24266787	37.34934849	30.69932964	33.93261303	131.5576121
13.2607374	15.68138	16.986	13.93395	49.93481853
13.2607374	15.68138	16.986	13.93395	49.93481853
8.55932	8.06894	8.34934	8.18333	44.91382404
19.206592	21.833476	19.593621	19.124047	35.9771
25.1062774	23.39578634	21.05640605	21.08481339	29.42915
25.1062774	23.39578634	21.05640605	21.08481339	29.42915
17.94266217	17.13497957	12.66550761	13.49791105	4.77093
227.8465	235.2748	209.7904	148.1608	104.1385
143.72232	138.4110863	129.0146421	110.6998146	26.8016
143.72232	138.4110863	129.0146421	110.6998146	19.7366139
143.72232	138.4110863	129.0146421	110.6998146	19.7366139
0.902451	0.262743	0.518551	0.770855	1.82021
24.99562	31.30198	40.67994	27.51432	173.672
0	0	0	0	0.119259
0	0	0.335498	0	0
27.73979609	27.96650032	27.57227	23.22163696	28.42141
15.05065763	16.0084935	15.96651692	13.221993	17.46924346
1.05861	1.07041	2.03729	0.463131	36.8528
0	0	0	0	0
0	0	0	0	0
202.3089131	159.4421442	57.88793483	66.58276648	3.72565
0.276124	1.42427	1.43475	1.06075	1.17634
0	0	0	0	0
52.217851	45.13335	33.794185	34.29737	7.339013357
48.01469821	55.23594238	57.39052094	53.94109583	140.9476
46.10534	43.46151	48.76326	43.35691	59.35241445
46.10534	43.46151	48.76326	43.35691	59.35241445
19.92703374	18.40572781	18.00637222	15.37546128	43.240204
0	0	0	0	0
38.42121	46.4535	43.32218	27.83573	7.865200326
6.14078	5.564980008	6.41529	5.35229	6.95109

0	0	0	0	0
17.966747	17.96537433	19.07661	18.19619	2.572538507
17.966747	17.96537433	19.07661	18.19619	2.572538507
1.498705	1.360554876	1.604450646	1.927722074	26.12216
79.16514	72.08333	113.0658	129.30498	119.09244
8.07243841	9.6542	8.564019001	9.210686	25.64138
20.90649415	21.13580972	29.31918398	27.34021471	86.4579
18.83471742	17.86625736	12.96043012	12.202412	4.357142598
0.333512	0.839346	0.485983	0.437255	116.62
16.6213842	12.67691	21.706678	24.8959968	0.000277274
16.6213842	12.67691	21.706678	24.8959968	42.57090676
16.6213842	12.67691	21.706678	24.8959968	42.57090676
25.98967	25.84058009	26.33787007	25.63696	36.302
25.98967	25.84058009	26.33787007	25.63696	8.74661
3.28131	3.98621	5.10849	2.57203	0.91281
18.95518113	18.17978779	21.2897601	17.9406	71.2066
23.40920162	19.30880314	17.42490083	18.54500094	2.332390699
1.230853001	1.067349002	1.319608822	1.758744502	1.602175024
7.8943126	7.627103112	12.804249	11.6922909	16.468903
57.9692784	58.01929274	95.04253559	91.88356	49.40712604
57.9692784	58.01929274	95.04253559	91.88356	6.931223116
26.15	29.28811	27.2499	27.1427	35.1174
7.74522983	10.548289	7.18749117	4.722694222	3.966258
7.74522983	10.548289	7.18749117	4.722694222	3.966258
0	0	0	0	0
0.296464893	0.472203673	1.229096	1.864919249	0.834452688
180.4838	185.1951	218.4741	159.5832	155.0645
0	0	0	0	0.186731
2.298262	4.25126758	2.336219	2.921961	361.955
18.64531	21.73077	23.07606	23.3324	61.81037
0.115382	0.097625127	0.474855011	0.816205196	12.5842
0	0	0.0487235	0.131634	0.983558
1.700473829	1.50082097	6.961524746	12.18655655	6.48804
0	0	0	0.105814	0.187784
0.219254	0	0	0.140261	0.771523
26.79535166	26.91782175	25.32696047	22.11734163	48.20676127
26.79535166	26.91782175	25.32696047	22.11734163	48.20676127
28.2873	28.45475	25.424015	28.55876	77.6492
2.45545	4.62811	0.640748	0.339515391	465.60556
0	0.0759019	0.0741788	0	0
156.72669	121.86836	123.139898	138.44022	40.16991904
156.72669	121.86836	123.139898	138.44022	40.16991904
156.72669	121.86836	123.139898	138.44022	40.16991904
0.895411	0.486189	0	0	0.114892

0.518484768	0.570356	0.998319391	1.719410384	1.907310277
1.41394	1.20211	3.10745	4.12181	0
0	0	0	0.124227	0.264602
55.22683194	41.85992954	32.57436154	38.159415	63.2095
55.22683194	41.85992954	32.57436154	38.159415	63.2095
0	0.052763865	0	0	5.51104
1.17176	0.00521545	2.36749	3.03061	3.10679
32.380017	37.93667201	30.245393	27.36275206	35.83086
0	0	0	4.8374	0
30.77111	32.35336	17.807395	19.81768	6.879474267
30.1326	30.8887	29.8977	21.57623	3.11044255
13.29713	12.36568	17.73909	15.5567687	44.10372073
8.690193	7.46132	9.64755	9.433197	22.6949
8.690193	7.46132	9.64755	9.433197	1.760852673
53.95469	56.012	50.9583	47.96776	56.1549
50.33598	43.36725	51.21284	53.07368	35.1815
0.0410144	0.492858	0.160623	0.0538397	0.048192
0.421560488	0.246695779	0.321653881	0.250956	0.0481819
27.45709809	28.27081	39.78930784	40.79474945	37.71200001
27.45709809	28.27081	39.78930784	40.79474945	37.71200001
1227.25476	1213.8321	1534.84495	1058.73718	178.7581382
1227.25476	1213.8321	1534.84495	1058.73718	178.7581382
14.3873	15.0023	17.39025	14.3244	9.13647
27.81961241	29.3743531	38.31153008	27.86281	62.5939
0	0	1.79936	3.38608	2.37915
65.87388789	73.51341108	75.14049235	67.5735266	360.6623105
65.87388789	73.51341108	75.14049235	67.5735266	360.6623105
0.346113	0.1807036	12.685322	27.896727	14.39740319
13.47186	10.42013	16.8049688	22.13188	253.9277
30.23175547	33.323488	42.3375	39.12271104	78.73283199
0	0	1.65207	6.08913	5.63541
0.305379	0	4.3074	11.0328	11.5438
0	0	1.78202	5.72658	5.52551
17.52130737	20.79887019	13.78205	15.32152545	18.8179914
17.52130737	20.79887019	13.78205	15.32152545	18.8179914
1.114904576	0.262740241	6.422597311	13.759444	8.557976
0	0	2.93231	10.248	2.407406
32.153952	29.11250002	24.452672	24.05170007	12.44140003
0	0	0	0	0
1.845	2.95712	1.9159	1.09143	217.407
26.59908	25.97409	29.52963	22.98450177	51.12579
0	0	0	0.149711	0
26.67483	24.0191	15.77597	17.878596	4.686634
0.848658805	0.179603364	2.415039797	4.6092516	1.09466

0.848658805	0.179603364	2.415039797	4.6092516	1.4988079
0.848658805	0.179603364	2.415039797	4.6092516	1.4988079
0.353272	0.176241	0.662713	0.692205	1.58858
8.64489	6.20264	33.8274	18.1179	1.38576
0.788456	0.192141	0.554269	0	2.02025
3.0133	1.21528	2.19038	2.32872	2.98701
0	0	0	0	0
0.120591	0	0	0	0
0.155486	0	0.154337	0	0
0.083375	0	0.0810559	0	0
0.980797	1.17047	1.517291	1.1231269	0.304
0.426012	0	0.386469	0	0
0	1.14238	0	0.733704	0
0.146748	0	0.14384	0.195725	0
0.230288	0	0	0	0
0.745831546	0.617376915	0.829386418	0.951104876	0.721921782
0	0	0.745556	0	0
0	0	0	0.425813	0.187449
0.496292	0.167963	0	0	0
37.05186148	36.87457801	27.30359867	25.43694657	17.22917
0	0	0	0	0
0.15096	0	0	0.395919	0
0.306065	0.615529	0.591595	0.802181	0.88373
0	0	0	0	0
0	0	0	0	0.186848
0	0	0	0	0
16.127	13.035	5.75505	3.91689	2.81913
0.150221	0	0.725502	0.390136	0.35307
29.38326861	25.35678988	35.52678636	44.8713116	29.39371
29.38326861	25.35678988	35.52678636	44.8713116	29.39371
21.1339	19.631	32.0508	26.1019	17.4141
0	0	0	0	0
0	0	0	0	0
0	0	0	0	0
0.0768383	0	0.0764288	0.20466	0.094423
0	0.122699	9.69802	19.3534	20.2916
151.70156	179.39099	108.7060657	79.12282837	76.7453
0.0619288	0	2.3028	6.27904	3.323034
11.94327136	12.25563406	14.71304	13.87115122	57.1306
171.3688785	176.2690524	168.2038082	194.6474571	227.7737
64.22432612	70.41032	76.233845	63.04512	128.899
26.67328643	30.33210306	33.76829832	35.60975644	41.0532
118.1446	105.9192	22.96879	41.3647	18.75672
1.67763	2.612825	0.398864	1.63573	305.5158

0.0515822	0	0	0	0
6.96669	5.49877	5.116	2.69447	8.59872
6.296596137	6.829572785	8.571713383	7.793169	50.593
23.50994902	23.95241652	22.73051167	26.75902925	35.4433
56.2923	45.6125	9.87422	14.45913	2.896859
0	0	0	0	0
64.284713	61.778602	44.187978	49.261	15.64340002
10.4183	12.0111	15.4488	12.2521	27.8133
2.07758	2.39957	2.35288	2.41108	1.46696
0.136068281	0.172292865	0	0.286507348	26.3334
12.73704	11.15619	20.58694	23.08842	151.3539
36.5845	34.6824	33.9115	28.096	78.34471264
25.4595	24.1986	45.1055	40.7835	21.2893
58.4843	57.74389	54.8648	51.3176	58.1266
2.624386	2.645226	4.124783	3.087427851	22.02572727
2.624386	2.645226	4.124783	3.087427851	2.86829
28.79577	33.3922	40.5462	33.88971	398.1909
25.5456	28.6333	45.9701	57.5399	18.8312
2.52968	2.82134	4.60117	3.98119	12.74315
8.420147	8.881622228	8.91123079	8.843652	4.73058
39.78968	41.67691	69.22948	65.375386	39.40983
39.78968	41.67691	69.22948	65.375386	39.40983
0.0015573	0.000150458	0	1.20E-06	26.5879
7.868653509	7.907353536	9.673828515	9.251131719	41.5891
0	0	0	0	0.221635
0.2409127	0.381782	0.246876	0.927693	61.34348
0.0622577	0	0.180276	0.160788	1.3414
1.317505564	1.078496518	0.5637122	1.0467339	97.07694
1.317505564	1.078496518	0.5637122	1.0467339	97.07694
26.42539072	24.68891549	16.74179168	19.067764	29.63299
682.3808	745.9132	1097.54742	1119.31365	1364.870022
170.541663	160.7447	224.45066	204.6171604	114.982
170.541663	160.7447	224.45066	204.6171604	20.63042013
0	0	0	0	0
18.7771147	20.63132573	18.95589085	19.9724637	32.756898
100.1147105	99.86817001	113.5916142	117.5319233	141.7271
41.00123	49.29465337	24.67673	32.53115	31.9707514
41.00123	49.29465337	24.67673	32.53115	31.9707514
0	0	0	0	0.64842
20.548743	18.93078523	14.59521	15.84037	22.60382
12.59896	14.93154791	19.67454	14.38298623	3.97E-05
12.59896	14.93154791	19.67454	14.38298623	3.89276
24.43886	28.84791	28.057067	23.27318184	5.12E-05
24.43886	28.84791	28.057067	23.27318184	28.81834

24.43886	28.84791	28.057067	23.27318184	28.81834
9.909790048	9.97970182	10.82501152	11.45150568	3.730911483
8.51526	5.82829	2.169358609	3.29752	0.0594271
3.34572	3.86159	1.28085	1.63626	0.0723127
11.548084	9.251899	6.73502031	7.471311662	1.456410164
0	0	0	0	0
0.266345	0.131323	0.502767	0.847295	0.157469
9.24E-07	0	0.886297	2.95238	5.384
0	0	0.0746651	0	0
0	0	0	0	0
0	0	0	0	0
0	0	0	0	0
0	0	0	0	0
0.36217	0	0	0	0
0	0	0	0	0
0	0	0	0	0
0	0	0	0	0
0	0	0	0	0
0	0	0	0	0
0	0	0	0	0
0	0	0	0	0
0	0.178453	0	0	0
0.219467019	0.273186008	0.1315918	0.255119	0
0	0	2.36377	6.69834	4.34E-06
0.0101201	0.199854	2.05001	3.80E-05	8.34E-05
1.09681	0.371205	21.0283	39.8345	27.0564
0	0	0.948147	4.05368	6.62638
0	0	0.846673	3.4999	2.93498
0	0	14.2326	26.714	27.0183
0.545652	0.123462	14.5652	30.5187	33.152
1.18801	0.5122587	16.10405	30.1017	19.5983
60.95051483	65.00520664	91.90314211	84.43429004	84.75167
8.659288	7.4089409	13.3700411	12.57381892	2.997478595
4.438780261	4.378851293	7.412989155	6.41061	51.23951018
13.50056	17.22769	13.21168	12.78201	34.79637
0	0	0	0	5.86776
9.38749	7.00374	5.91354	12.2624	0.198724
6.54E-05	0.000703887	9.85E-19	9.57E-08	0.359626
2.17983	2.54336	5.1647	5.79929	58.4982
77.02206	81.82844	101.004948	90.78352224	415.7919
2.532086224	2.045619385	5.269543	3.676599363	18.88036
0	0	0	0	0.256933
16.08719	18.806899	11.57206832	9.823586	1.5420742
16.08719	18.806899	11.57206832	9.823586	1.5420742

16.08719	18.806899	11.57206832	9.823586	1.5420742
5.779976	5.864877	9.40747	8.90607	55.2877002
5.779976	5.864877	9.40747	8.90607	55.2877002
38.94259	41.92219	30.3835	27.41978	6.43534
14.47835	16.83262	22.56505	20.50819	45.99951
79.529904	74.394175	64.90616596	60.37198033	14.11470538
34.77894	39.70176	16.50234	19.34273	36.01264
34.77894	39.70176	16.50234	19.34273	36.01264
0.288434	0.146035	1.13855	5.01189	8.87664
0	0	1.02825	1.72542	1.5566
1.433876515	1.365187097	2.482892851	2.541640281	1.547156013
1.433876515	1.365187097	2.482892851	2.541640281	1.547156013
0	0	0	0	0
0	0.0590947	0	0	0
0	0	0	0	0
0	0	0	0	0
0.036231	0.0362975	0.0358516	0.0474588	0
0	0	0	0	0
0.072020725	0.082049128	0.039938935	0.266829885	0.0286891
0	0	0	0	0
0	3.22599	0	0	0
0	0.2824	0	0	0.317863
0	0	0	0.466933	0
0.405383054	0.374330443	0.261844774	0.620350787	0
0.582832339	0.700392991	0.93322507	1.121225103	0.186856
0.229315	0	0	0.148369	0.136356
0	0	0	0	0
0	0	0	0	0
0.459461	1.00337	1.00577	0.633131	2.02531
0	0	0.0543115	0	0.576406367
0	0	0	0	0
0	0	0.163505	0	0
0	0	0	0	0
0	0	0	0	0
0	0	0	0.0667849	0
0	0	0	0	0
0	0	0	0	0
0.478614	0	0	0.617684	0
0	0.265871	0.492339	0.6778	0
0.299555	0	0	0	0
0	0	0	0	0
0	0	0.101057	0	0
0.150773217	0.331998157	0.292911	0.195602955	0.0615537
0	0	0	0	0

3.496693207	3.166008	3.729180191	3.934958699	0.9517857
0	0	0	0.199585	0
0.0701672	0.0469314	0.0229279	0	0
0	0	0	0.0393773	0
0.0195235	0	0.0560101	0.0253757	0
1.853444	2.199447	1.611624267	2.46224	0.806726106
0.0156158	0.0156423	0	0.0202991	0.424522
4.285481073	2.5434216	24.075067	48.94637	4.00496
27.3555	33.5433	33.679	33.9813	58.3134
39.18946	42.94572	42.3625627	37.42257	93.6543
39.18946	42.94572	42.3625627	37.42257	12.08340844
3.398940932	7.375661028	6.28528507	6.832957597	24.04197114
3.398940932	7.375661028	6.28528507	6.832957597	24.04197114
3.398940932	7.375661028	6.28528507	6.832957597	24.04197114
13.7363	9.97552	15.6169	15.8484	15.9392
14.927059	13.5172442	13.74553275	14.91027405	29.17928
12.60204	11.88069507	17.92884	11.67136	6.55146
0.0879046	0	0.0851262	0.114317	0.721926
29.7169	26.7613	35.1597	25.7686	57.7565
52.87829817	52.579936	42.96336657	46.0406857	29.85468333
9.549978	10.424746	12.2555	10.02343	25.6987
0	0	0	0	0
37.490925	34.513649	24.93365	26.665648	8.33958
6.33862	6.21532	10.36823	7.01838	5.07681
19.88138045	19.27429418	27.53038528	22.01875526	56.45121989
19.88138045	19.27429418	27.53038528	22.01875526	56.45121989
0.248053	0.404217	0.703361	1.33727	6.79E-10
180.4883741	188.4903964	205.6706119	220.8664345	153.57996
180.4883741	188.4903964	205.6706119	220.8664345	28.59180182
0	0	0.66786	1.35498	14.2884
24.66981173	24.50211401	26.36461013	25.81641127	47.45797603
17.53843892	22.21764227	26.41760367	22.10300626	35.43791678
12.5046	17.54283	15.77535	11.76177	41.3444
11.3735	10.5797	1.21309	0.416589	0.38759
0	0	0.449731	1.23018	0.528063
17.35542533	18.26679187	18.62440942	22.79641191	55.85800245
17.35542533	18.26679187	18.62440942	22.79641191	55.85800245
137.305	119.139	110.463	91.8903	35.4892
89.393937	100.196015	122.787012	84.38682	234.126
52.7507	54.3091	42.2118	37.2687	7.08679
9.696294655	14.21333092	19.96425532	14.47072913	44.336575
82.677	106.3188	120.125	121.366	26.55
450.0344	585.485	1020.536	853.657	212.733
8.022364002	8.522066978	15.2824482	22.80484514	7.631409947

0	0	0	0	0
24.59231523	21.47712046	16.23083402	21.23133612	4.977273294
24.59231523	21.47712046	16.23083402	21.23133612	4.977273294
37.26302	42.05222	137.7817	83.141	30.81831283
37.26302	42.05222	137.7817	83.141	30.81831283
0.710778	8.38543	9.98387	3.36416	0.000553914
0	0.0266428	0	0	0.192226
0	0	0	0	0
0.587981878	0.334149717	0.137036387	0.192485407	0.0867925
0.587981878	0.334149717	0.137036387	0.192485407	0
30.3615	32.0234	25.5463	23.52826	25.68109349
71.92658403	71.23074	133.8160025	121.9717116	60.5164492
2.997598266	2.234587974	1.884198138	1.2732789	0.202245
39.18984	37.44758	43.99889	41.52751	56.82198989
337.158428	351.670562	392.2951849	410.11787	121.95267
18.04122	20.6627	19.47507	18.35555	32.139
2.395400888	1.782802383	29.50269896	58.20619008	50.3666
127.9177002	157.1372529	163.60364	147.9063403	45.34005173
127.9177002	157.1372529	163.60364	147.9063403	412.1583
13.54310577	15.02817863	17.94323172	15.33706041	34.4245
528.872	536.724	535.775	490.793	990.57
0.730651	0.854426	6.52E-06	1.18403	0.174696
0.582748	0.585608	0	0.750414	0.351809
0	0	0	0	0
28.72472	30.39826	35.21474	35.859159	23.90926
17.38346185	19.06812	24.90148	26.51437	93.61519373
18.73050873	17.89772434	31.51488762	32.91728214	38.1956
22.34439869	20.55661653	17.38278251	18.96649754	3.82004502
22.34439869	20.55661653	17.38278251	18.96649754	3.82004502
9.8135	8.77954	8.20961	7.80216	1.23879
28.24878	27.639284	57.116495	62.785165	25.67726
0	0	0	0	0
91.264921	81.511035	55.310645	66.8153778	13.31411
32.5434261	31.04260412	32.88675266	34.91428501	27.78680419
32.5434261	31.04260412	32.88675266	34.91428501	27.78680419
1.33786	1.92808	3.13308	5.71258	40.8998
10.52665778	9.8467065	13.28023129	10.61998287	85.87830959
40.57613132	49.09243643	26.10797175	22.7760921	16.1638282
40.57613132	49.09243643	26.10797175	22.7760921	16.1638282
3.645762	4.46953104	5.881548	11.18328608	241.709
5.80097	4.4633	7.31234	5.45013	7.81065
1.39901	1.41505	1.58477	1.45109	0.645495
41.43843408	52.45888	55.85650435	50.7923645	173.1899
94.129245	115.5859959	159.41071	133.08923	472.8591

82.47912	83.7793	33.85847	31.26389	1.445355217
0.187537564	0.065740198	11.07772931	11.246162	9.486018685
0.187537564	0.065740198	11.07772931	11.246162	9.486018685
0	0	0	0	0
27.02901834	24.728987	30.989029	34.206133	41.70747
3.0522	1.65788	2.52659	2.44224	7.18749
6.90272	6.33897	10.7658	7.0208	20.1004
2.064666	0.45539344	43.7587	91.5174	78.6613
3.28933	4.21667	4.69249	5.56104	1.28249
3.28933	4.21667	4.69249	5.56104	13.2342
1.426951887	1.264691727	2.633885	3.609115	9.53623
4.178722	4.409832	13.569057	11.968744	5.59667
0	0	0	0	0
8.7011	12.0568	14.1938	12.2287	6.17396
0	0	0	0	0
0	0	0	0	0
0	0	0	0	0
0	0	0.192756	0.263186	0
0	0	0	0	0
0.0717479	0.363468	0	0.28268	0.352434
0	0	0	0	0
0	0	0	0	0
0	0	0	0	0.0796195
0	0	0	0	0
0	0	0	0.168856	0
0	0	0	0	0
0	0	0	0	0
0	0	0.314382	0.648906	0
24.58862568	21.59488619	5.51896231	12.23426746	0.398037
24.58862568	21.59488619	5.51896231	12.23426746	0
24.58862568	21.59488619	5.51896231	12.23426746	0.39421
0.638368423	1.561913867	1.39611	1.997920314	0
0.13143	0	0	0	0.162341
10.16542	10.73707	15.31776	15.09953	36.2323
10.16542	10.73707	15.31776	15.09953	36.2323
12.94375286	15.58454072	26.65036414	21.21892065	19.78706
15.443363	14.37916	19.209185	15.633357	38.49487
31.36059621	30.25543	21.799755	22.36745	14.83609
77.4512	68.8993	83.6301	96.1868	287.275
45.83758351	44.05646	33.29854343	36.30918	21.45135
27.33755	28.56819	25.0380319	24.29427225	40.383262
676.25605	741.08256	1092.47718	1081.88361	1208.84
2.753601	3.14893	5.33195	7.634741	35.46522
2.753601	3.14893	5.33195	7.634741	35.46522

9.39948	10.0129	16.2515	11.3545	42.7106
51.66035	49.27292	51.80755	57.8505	62.38683017
51.66035	49.27292	51.80755	57.8505	62.38683017
52.3911	55.8577	50.5394	50.7026	150.662
13.2539	14.7751	13.7788	14.2724	16.1338
71.59522976	69.50218146	61.50631575	67.58495199	23.77011
36.6837	37.1329	34.0072	34.9689	34.3988
0.691249828	1.437728	0.171973816	0.064630707	413.8530068
0.691249828	1.437728	0.171973816	0.064630707	413.8530068
1.696721854	2.3348	8.44947	7.8078	5.83895
61.8256	70.48339	90.24965	87.11432	115.11617
61.8256	70.48339	90.24965	87.11432	115.11617
36.66440083	36.453267	47.4065382	43.419988	60.6562
0.560213909	0.892640386	0.4232	0.579435367	0
35.92736249	36.83942001	25.40572879	29.81795028	23.56264063
240.266	209.186	333.696	301.665	73.6278
11.35996	9.764039	13.548621	12.72019001	27.8375
54.57882805	33.89394241	92.15554139	115.034886	16.63823
54.57882805	33.89394241	92.15554139	115.034886	16.63823
54.57882805	33.89394241	92.15554139	115.034886	16.63823
7.858604575	5.554253304	15.4662971	20.33412883	9.606108
282.967	261.083	334.683	293.907	201.446
97.56949632	101.2210489	93.70765434	104.5803447	54.83321622
29.4931288	31.03686646	41.35596	52.1047	44.91583
29.4931288	31.03686646	41.35596	52.1047	44.91583
20.87004	22.707104	21.081662	22.510379	33.2197
10.7467	11.00303	11.10137	11.0706	16.62794
45.3891	48.4357	34.9763	43.5304	88.1415
0	0	0	0	0
57.42397064	58.48499989	40.1854793	43.7815351	62.26284
12.0718	13.1177	8.02596	10.4649	20.50663114
24.0475	19.50810011	5.87998	5.75241	0
17.88906	16.59992	26.61545	22.10984	30.86
25.785484	25.62766537	14.84656	11.033769	1.16063
14.68528	14.67599696	13.47392499	13.37201	2.04869865
8.797394588	9.206626	7.229488532	6.761185	62.580775
4.527899	4.23678	5.201062	5.576086	38.075584
65.99773733	65.56425973	83.83594237	80.05566406	119.196769
25.22191375	25.73241257	26.68483197	23.77761327	24.709787
143.2098	146.2624	8.23152	43.0297	2.73894987
6.81348	5.77262	2.432340162	2.38194	0
30.6383504	29.10479	24.8917539	21.49792	45.7393
30.6383504	29.10479	24.8917539	21.49792	57.97690804
6.104508	9.321423	10.2381	9.013523	6.462555766

6.104508	9.321423	10.2381	9.013523	6.462555766
80.22753705	68.460671	123.8517122	141.2919223	52.804135
0.0144665	0.157156	4.90E-14	8.00E-13	2.90E-13
20.05747814	23.109659	31.2956079	32.3666042	73.483898
20.05747814	23.109659	31.2956079	32.3666042	75.99194
20.05747814	23.109659	31.2956079	32.3666042	0.694465
4.01178827	3.959499979	9.553891009	11.75587607	45.8945
50.19961	49.41696	57.4004	68.501613	1.370683364
50.19961	49.41696	57.4004	68.501613	82.3516
14.74785	16.80589	22.36384	21.0844	41.678
85.17030104	72.70081246	59.98018914	62.638763	51.0788
18.75908	20.20963803	23.90841	21.79464	25.07699
112.00509	69.552001	114.504736	95.948861	29.58701971
112.00509	69.552001	114.504736	95.948861	29.58701971
22.01345	25.03034	17.4655	15.56126	7.77169
117.568176	102.433162	91.39001293	82.71127312	2.2356723
117.568176	102.433162	91.39001293	82.71127312	6.666789365
117.568176	102.433162	91.39001293	82.71127312	6.666789365
117.568176	102.433162	91.39001293	82.71127312	0.0615383
0.0209582	0.114069	0.175449	0.243779	0.0163313
0	0.0750168	0.148286	0.0990353	0.0289976
0.0476707	0.0973917	0.239541	0.255294	0
0.103679	0.0907664	0.179304	0.0995785	0.0868984
0	0.0795473	0.154285	0.103832	0.0879166
0.138525	0.0469533	0.138917	0.124623	0.0519124
0.0215931	0.0881735	0.152053	0.101636	0.0248461
0.119666	0.132874	0.162692	0.27681	0.0758583
117.568176	102.433162	91.39001293	82.71127312	0.437113347
117.568176	102.433162	91.39001293	82.71127312	0.437113347
117.568176	102.433162	91.39001293	82.71127312	0.437113347
117.568176	102.433162	91.39001293	82.71127312	0.559096
117.568176	102.433162	91.39001293	82.71127312	1.78794
117.568176	102.433162	91.39001293	82.71127312	4.71785
117.568176	102.433162	91.39001293	82.71127312	12.5483
63.438728	80.66109	66.85244248	68.59518	62.71237
11.30910102	10.62049831	15.076089	13.52856075	41.6827111
26.64305	32.78479	36.92214	29.02139	56.024
26.64305	32.78479	36.92214	29.02139	56.024
37.41623907	41.04157237	39.05721346	44.74190614	28.03747397
37.41623907	41.04157237	39.05721346	44.74190614	28.03747397
37.41623907	41.04157237	39.05721346	44.74190614	66.1872158
37.41623907	41.04157237	39.05721346	44.74190614	66.1872158
8.190040182	9.121496963	3.47186663	4.1341436	18.02549
531.8237	547.9415	72.25079	142.28336	14.8129

16.107533	18.457317	19.3291223	16.61027	66.73008889
16.107533	18.457317	19.3291223	16.61027	66.73008889
0.101957	0.102073	0	0.268654	0.117029
5.92755564	4.929773964	9.610247022	10.26715626	56.67210087
5.92755564	4.929773964	9.610247022	10.26715626	56.67210087
9.13029	7.72512	7.33406	9.53419	11.01885
70.11669	70.06662	69.08372	71.43554	38.99095434
32.432808	41.4221516	43.77437004	36.29589441	0
32.432808	41.4221516	43.77437004	36.29589441	6.76463
32.432808	41.4221516	43.77437004	36.29589441	9.28866
30.9216	24.26909	21.88222	24.6651	12.13783695
68.50456938	76.19676833	87.606649	96.21397606	147.3101
4.322384239	2.765336874	5.892013798	8.000918246	5.291165339
4.322384239	2.765336874	5.892013798	8.000918246	5.291165339
14.121046	15.72928609	19.89916438	15.80705993	29.08276
0.125100386	0.042182573	1.235490784	3.508305319	1.94398
0.125100386	0.042182573	1.235490784	3.508305319	1.179323676
0.125100386	0.042182573	1.235490784	3.508305319	1.179323676
32.81848	27.35504	38.81968	30.77654	54.44380746
32.81848	27.35504	38.81968	30.77654	54.44380746
8.326547	4.129862401	25.77770514	26.34833816	3.854942623
18.34915001	17.62681512	18.18411165	15.36270697	4.075634
1290.062083	1526.38594	1452.052177	1116.92668	1362.93
13.5527	17.6017	19.4163	24.1129	59.551
12.7572	10.8578	8.99704	11.8831	0.183014
1290.062083	1526.38594	1452.052177	1116.92668	54.20449
452.483	302.853	45.0712	45.0179	2.52062
5.285927	5.928459138	7.261439	9.693703525	6.812254
24.936438	19.3997	23.469	29.18445	20.24560156
29.546563	21.992956	33.650376	35.29666	124.91971
29.546563	21.992956	33.650376	35.29666	124.91971
29.546563	21.992956	33.650376	35.29666	124.91971
21.610791	22.08253492	29.28160935	21.9294677	64.2247
9.56402216	9.1985713	13.72057333	13.08673907	11.171474
10.36282195	7.97742187	13.66101681	10.94820248	2.359923492
61.76302522	56.07416266	21.52171701	23.66120649	1.633220861
38.03365556	25.11201608	4.069425807	4.448408356	0.34125
6.197039292	6.265905	4.15702458	3.817682266	1.191482976
1.152896002	1.323858615	3.945182309	4.958024722	35.79782986
6.058849133	5.630981281	9.209523975	8.943281	27.81415
10.093656	8.455668562	13.090747	13.106502	15.227579
12.7617	9.37785	13.8149	12.2215	7.50888
10.093656	8.455668562	13.090747	13.106502	15.227579
0	0.0421087	0	0.108395	0

0	0	1.26174	0.864172	0.75239
13.28992	13.91407	15.7607	12.15022709	47.0915486
8.7637	9.07224	11.9918	10.6636	37.8282
14.33358615	14.26102168	18.5074976	18.17734729	6.009886
14.33358615	14.26102168	18.5074976	18.17734729	31.2867362
23.83480458	20.788737	18.26006046	17.9782339	8.8301051
23.83480458	20.788737	18.26006046	17.9782339	8.8301051
3.696414	4.793731625	5.96254055	5.9954918	9.01786
17.393726	15.60920022	6.774450683	9.133166	1.71419
24.31198	23.89281	27.39858339	29.06901	61.84533
0	0	0.680532	0	4.38069
0	0	0	0	0
38.98474	32.50799	26.20782	31.2617	32.013
53.12797542	48.46808325	34.32994015	34.68383857	5.251915521
53.12797542	48.46808325	34.32994015	34.68383857	5.251915521
24.06141	24.50162	10.96260746	13.267344	3.060791082
0	0	0	0	0
24.2662115	27.45639509	23.62452	27.56806001	48.21699733
316.6646	325.586411	62.4884179	98.02595	13.78540864
22.2563	24.2198	27.4908	22.2017	43.0666
95.31594517	84.99297452	65.016227	73.28718	1.91137
95.31594517	84.99297452	65.016227	73.28718	3.2806
188.4896	189.6163	236.512	204.7144	108.76
221.10615	209.7649411	200.9996	210.7546239	201.8182
31.82205347	31.96405087	30.03867496	29.18097013	32.79926754
31.82205347	31.96405087	30.03867496	29.18097013	32.79926754
60.1969	66.9181	89.052	73.0782	132.0149
0	0	0	0	0
0	0	0.0526004	0.0355134	0
0	0	0	0	0
2.392751864	1.643903583	6.207123408	4.656478334	0.907877588
0.078748213	0.407896123	0.685110008	0.152817996	0.099935
39.66250002	36.13630001	57.192427	53.716022	62.6164
346.0912	345.6669	421.9511	343.9048	138.2398
1.47187	1.22193	6.92096	9.66542	2.63658
17.763175	21.21341372	27.070377	33.12542	73.88486
78.68083197	107.7189758	46.78144272	43.88683462	14.16036417
0.48847293	0.426681	0.32496824	0.2176	43.51250074
19.86952492	20.03360648	31.27650361	22.96983827	29.22346
13.67784	12.901736	18.12002939	15.76518	32.960626
30.6730614	30.00769621	23.36478892	23.88407053	80.17296
30.6730614	30.00769621	23.36478892	23.88407053	80.17296
84.2186176	80.64063506	74.80478559	78.148857	49.3383076
9.39562	8.51821	5.80475	5.59335	0.810614

154.75007	134.29189	125.20783	95.4251	65.477062
154.75007	134.29189	125.20783	95.4251	65.477062
3.158672614	1.656743036	1.573264068	2.443144845	0
23.14799678	20.90362113	25.86098719	23.730032	15.08975
0	0	0.114247	0	4.2887
17.400325	17.807502	11.46917382	10.7247375	25.01922
33.10687081	33.97741091	24.25094266	28.5591434	21.455407
38.4899	40.114	30.7907	30.8634	5.30517
64.9617	54.9439	109.043	100.44	71.5614
25.51059	27.565314	30.111579	29.806395	61.45400125
11.45389	12.66469	12.98249	13.20951	10.28262084
32.765	30.2657	21.61503	23.05987	5.681035855
25.72071209	24.75173673	24.65123928	24.07431115	29.31828371
53.3532	55.6161	57.0207	57.2939	51.0135
21.02962	18.38517	19.23019	14.2083	51.99159
20.29944	20.9045	24.34727	26.73993	38.8647
20.3998	23.0873	24.3712	28.4787	57.9981
110.6638	113.1637	77.6311	83.2511	39.1818
22.90221	23.40894	37.1211	24.9997	127.947
0	0	0	0	0
235.8931419	249.5956303	215.8888033	146.7184435	90.3950285
13.8546	13.1734	8.12816	8.03351	20.9655
21.33084301	21.7901384	19.17745401	20.80106762	22.187895
113.15737	85.279853	51.7327682	62.210337	5.562388
113.15737	85.279853	51.7327682	62.210337	5.562388
259.163	265.861	321.361	316.187	340.766
32.530363	33.69357741	33.13426614	31.37827572	86.07973
54.9582	47.1907	16.7082	15.4945	3.25937
20.00172801	19.0443057	15.39855672	16.808869	1.398264
20.00172801	19.0443057	15.39855672	16.808869	20.20421
21.6810886	20.89668486	31.58812921	24.52446243	29.1013
14.94862813	17.60655582	14.74407108	13.6871984	16.816892
2.829643	2.454528001	1.974014948	2.280808459	5.398614
0	0	0	0	0
0.168066	0	0	0	0
850.655	535.0376	686.467	642.7341	179.3694
38.4070001	32.63783465	32.46784395	49.50663775	33.822552
11.428682	10.4316113	13.313365	12.396579	34.27623748
31.9973	26.1308	29.0855	20.55787	106.128
8.300556	9.829584	19.27673	13.943564	4.82136
8.300556	9.829584	19.27673	13.943564	10.2653
8.6664	9.30816	7.73811	7.48678	23.748
92.129506	95.500266	101.2236967	96.12751179	15.06627332
92.129506	95.500266	101.2236967	96.12751179	15.06627332

92.129506	95.500266	101.2236967	96.12751179	15.06627332
92.129506	95.500266	101.2236967	96.12751179	97.34123405
11.48589843	10.697329	11.85710333	13.21748522	43.1648
12.714628	14.22968	19.75483	14.45169	40.31078
91.6425	99.2307	86.3118	84.3085	191.101
73.30351	85.04206	85.502201	85.16795	68.86907
34.01655914	36.4115	30.14847438	31.72670593	28.863475
34.01655914	36.4115	30.14847438	31.72670593	28.863475
28.09557005	28.41937079	33.89361248	36.3309338	86.0257
81.509517	78.79082672	82.697671	72.74918485	186.90994
17.933747	21.0531	19.833359	22.214299	56.85113
34.09570126	35.053644	38.191204	35.8965	43.434706
34.09570126	35.053644	38.191204	35.8965	43.434706
183.95346	216.1606	45.74087377	92.32760183	27.654992
11.08304533	14.84520022	9.952700677	12.30761555	6.48053
11.08304533	14.84520022	9.952700677	12.30761555	14.9974
11.08304533	14.84520022	9.952700677	12.30761555	291.572
155.921894	129.9503726	75.87192619	111.9803206	2.66611
155.921894	129.9503726	75.87192619	111.9803206	16.727994
1.26693	0.254058	0.645038	1.5241	0.298812
4.758056046	2.006009837	1.930550131	2.851433559	0.0797651
18.2635	17.3157	21.6978	20.6477	45.15673
254.55513	242.26692	196.766672	178.504336	42.4762
113.1868501	130.7606599	104.0816264	87.61454448	66.5778
29.51109928	30.66679127	29.06435263	31.19345903	32.5572335
29.51109928	30.66679127	29.06435263	31.19345903	32.5572335
68.3441	59.9977	43.3164	41.0998	13.32185
75.6914	71.3926	63.578	55.2638	127.7485
196.4642379	214.8452228	275.935466	247.8068093	127.7485
19.4748	23.0358	17.3626	15.3868	51.2307
58.796025	53.116292	53.62693	41.80759997	43.52954
58.796025	53.116292	53.62693	41.80759997	43.52954
0	0	0	0	0
0	0	0	0.806551	0.233677
18.326931	14.684482	21.192535	16.462937	95.7865
6.47044	5.550416	16.50214	13.42178	13.14179
24.15505	17.813351	26.41735	26.974524	5.39228
13.7059	8.1455	14.9121	7.05336	0.429153
7.574521	10.33387	9.86501	8.173781	1.76079
0	0	0	0	0
0	0	0	0	0
0.491748	0.296685	0.201524	0.389717	0
0	0	0.119297	0	0.575165
0	0	0	0	0

0.154177	0.12349	0.242291	0.121471	0.108591
0	0	0.163554	1.10049	0.217678
0	0	0	0	0.588358
0	0	0	0.141896	0.123072
0	0	0	0	0
0	0	0	0	0
0	0.293348	0.289712	0	0
0	0	0	0	0
0	0	0	0	0
0	0	0	0	0
0.2724	0	0	0	0
0	0	0.500316	0	0
0	0.802468	0	0	1.37939
0	0.874449	0	0	0
0	0	0	0	0
0	0	0	0	0
0	0	0	0	0
22.24471	19.5651	14.00516	14.86469	2.422122661
0	0	0.155694	0	0
0.8479	0	0.797025	0	0.484128
0	0	0	0	0
0.0442169	0	0	0.292469	0
0.101383	0.708313	0.193094	0.661279	0
0.860287	0.215997	1.19774	1.35835	5.63323
0	0	0	0	1.60242
0	0.100486	0.195334	0	0
0	0.0919964	0	0.362246	0
9.877231	10.542188	7.867616	6.440680159	17.5788
16.15181	17.82064	18.55052	17.03538	61.39709467
0	0	0	0	0
34.48058645	36.61915	29.5142	29.913498	31.28283
34.48058645	36.61915	29.5142	29.913498	30.5286
4.41947	2.4359349	6.326172424	6.282993463	2.94826403
0	0	0.0885821	0	0.227956
0	0	0	0	2.74477
25.19669	22.15726	18.49279	17.22606	27.48433763
41.2952	23.5968	97.514	124.35	42.7697
73.0257	47.3844	190.083	238.401	68.4327
0	0	0	0	0
0	0	0	0	0
0	0	0	0	0
0	0	0	0	0
0	0	0.108009	0	0
0	0	0	0	0

0.125261821	0.318064592	0.2516807	0.192122691	0
0.125261821	0.318064592	0.2516807	0.192122691	7.24973
0.696214815	1.968779459	1.24446032	4.08368639	7.881939
0.696214815	1.968779459	1.24446032	4.08368639	7.881939
0.131317	0.258614	0	0	0
11.9576	10.6049	8.62567	9.4396	3.20051
0	0	0	0	0.243397
0	0	0	0	0
0	0	0	1.061085057	1.034302
0	0	0	0	0
0	0	0	0	0
0	0	0.0855226	0	0
0	0	0	0.490743	0
0	0	0	1.34635	0
0	0	0	0	0
0	0	0	0	0.123067
0	0	0	0	0
0.0599728	0.0615257	0.0600099	0.322214	0.446021
0	0	0	0	0
0.787978	0.896149	0.680251	0.651026	0.903843
4.35208216	3.1239151	3.677559847	3.151751111	2.104919
106.5208	99.9432429	45.07124	49.981649	18.32296699
106.5208	99.9432429	45.07124	49.981649	97.24759
0.778712439	1.587015451	0.416682	0.407857723	185.7082
0.551491	0.484275	8.37E-19	0.274993	4.40651
2.75127	1.75436	0	0.191137	0.180567
275.547295	313.96319	275.7130148	194.6162348	189.253
71.48652	69.51819	23.72498	32.23675	9.49248
176.9017	184.1398	236.4287	163.8324	206.1462
5.62467	7.45067	6.95224	5.63308	65.3172
58.05659432	65.22264	62.78302	61.29035	26.29654687
31.27033198	28.70029269	13.43049004	15.7470027	2.19533
43.131623	39.249905	27.668515	33.416903	9.339917901
7.53088	9.37904	4.2217883	6.67407	232.8115
0.393099	0.377988	0.13659064	0.1855649	0.496917
38.9407	43.3406	63.8561	51.9755	116.84955
101.44502	104.9760164	79.10001492	72.02172617	123.4250104
67.1885	60.2059	93.1028	58.5336	35.9664
5.4985689	5.835453254	2.7440154	4.0905942	20.510929
4.905549	4.96415	4.656097995	4.152085948	0.751315
16.04883245	17.23655029	16.71423491	15.80908148	38.478
224.3851	181.7101	287.519	261.6374	264.26
17.803848	20.58636729	11.363505	11.49158363	28.952
22.0057664	24.76082258	16.43374679	17.60181916	15.16468

667.4667	722.8451	1153.088	1132.2522	1081.79
7.515720179	7.669103268	12.6140898	10.84233136	2.18005
7.515720179	7.669103268	12.6140898	10.84233136	5.53333
2.668358145	1.481587925	5.194659962	7.558809028	4.05807
2.668358145	1.481587925	5.194659962	7.558809028	1.03E-05
8.164876187	6.117777	9.3989717	6.9067743	225.8730107
8.164876187	6.117777	9.3989717	6.9067743	225.8730107
8.164876187	6.117777	9.3989717	6.9067743	225.8730107
8.164876187	6.117777	9.3989717	6.9067743	225.8730107
1.124392	0.43412	0.555911461	0.686642	0
35.60713336	37.09494681	54.85908414	48.26655014	25.863524
0	0	0	0	1.6019
0	0.497712	0	0.42962	49.0144
0	0	0	0	1.61733
18.76015	16.52934	19.29066	16.9243	36.67043
5.80374	8.1155	10.84445	9.00497	34.5492
6.917414	7.08837905	11.53717	11.526915	38.6531
4.05404	3.95576	6.37505	7.59186	64.5612
30.10463368	29.9333065	37.28498024	40.56398162	145.04517
24.81913551	26.02020639	20.84475486	19.7634891	7.49718
1.295474333	1.2174903	3.341981	3.219066	0
1.295474333	1.2174903	3.341981	3.219066	0.478832
0.119089	0	0.647449731	2.290406006	3.53167
3.3240142	0.923186282	32.48852956	39.56443918	63.93072074
32.3513	42.5996	39.5184	37.5298	9.19054
1.51E-05	2.28E-05	2.10E-06	0.000224165	2.09938
50.2887	30.4367	76.8952	42.6078	0
8.624875265	7.471902124	10.71506714	6.719060249	64.85672
189.9857	224.0188	123.3072	130.9356	5.98502902
0.421711997	0.328990901	0.605219621	0.18486769	0.0568891
0	0	0	0	0
0	0	0	0	0
0	0.368162	0.287719	0.19204	0.176857
25.70950527	10.18080256	106.5880216	100.2610391	20.71900892
9.96E-06	1.42E-05	1.32E-05	1.54E-05	0.0878018
0.678162061	2.124300511	2.33911	2.110226	2.58725
0.678162061	2.124300511	2.33911	2.110226	5.95287
40.7924	27.32144	169.4449	158.1499	4.7859
4.11366	4.10931	3.59426	3.754922663	3.775364
0	0	0	0	0
0	0	0	0	0
6.356912609	7.78656311	4.790765	5.2202905	7.049081393
6.356912609	7.78656311	4.790765	5.2202905	7.049081393
0.151528	0	0	1.719520066	6.82948116

38.18260963	57.75508901	226.349984	205.8721266	1.571882755
38.18260963	57.75508901	226.349984	205.8721266	71.4023
4.48146	4.53886	3.769946	3.61361	0.757383307
22.94985149	23.798661	24.99232784	21.49161306	24.38374993
7.108631	5.960428	10.29971	7.07276	3.25785
9.35345	8.79094	13.9699	15.0071	42.1872
7.108631	5.960428	10.29971	7.07276	7.39339
11.412184	8.53168493	14.813197	15.06883463	30.49313
11.412184	8.53168493	14.813197	15.06883463	30.49313
0	3.48608	0	0	0
0	0.0269242	0	0	0
0	0	0	0	0
0	0	0	0	1.32571
1.45548	0.0882061	2.62352	1.30905	0.000114219
2.49792	0	8.8136	31.7044	9.00941
2.34417	0	4.36257	10.0967	5.75268
403.2123	216.4359	345.5371	263.558	7.92931
0.247078	0.245274	0.297776	2.10169	46.1668
5.73902	3.58288	3.41824	4.47313	0.181424
17.9313767	18.13004315	12.03177353	10.087479	3.069784001
29.65429446	28.91826809	36.15113053	28.73116574	12.33853
55.25138552	57.51196474	45.6260887	43.61412271	3.24202
55.25138552	57.51196474	45.6260887	43.61412271	18.76067
55.25138552	57.51196474	45.6260887	43.61412271	34.57055
55.25138552	57.51196474	45.6260887	43.61412271	34.57055
9.513271759	9.46542	15.48101	16.1169214	27.69454
0	0	0	0.480817	0
62.3492	58.1725	50.4965	53.7277	41.1724
0	0.360769	2.78616	6.86427	5.73772
3.13473	2.91641	3.98906	3.22929	6.29848
8.60E-05	1.11E-05	3.00E-07	0.0821948	0.00139359
0.000244426	7.45E-05	0.00829171	1.37769	2.59E-05
1.6128	1.34288	2.04366	1.67502	3.76197
0	0	0	0	0.272284
19.3342	19.616	17.6977	16.8388	18.9671
66.62995596	59.66024093	42.57399318	45.30403	3.78326
66.62995596	59.66024093	42.57399318	45.30403	10.74597
111.36856	83.52557	58.86099	61.89554	3.785379
1.65784	3.45177	3.39666	6.11658	0.82334
34.91686261	39.8171774	22.13893149	23.42564018	54.33017
12.191673	12.109469	17.48063458	19.28500001	44.92332
19.0281154	19.63290054	15.793608	17.2496174	7.47104
6.45364	4.68673	9.0612	7.16258	25.65243
0	0	0	0	0

0	0	0	0	0.239733
0	0	0	0	0
0	0	0	0	0
46.97321148	48.663402	40.429016	46.4411514	21.04070117
0	0	0	0	0
0	0	0	0	0
0	0	0	0	0
0	0	0	0	0
0.198006	0.197599	7.90905	14.0917	10.6959
1.08721	0.930403	1.50923	0.81802	0.560399
0	0	0.167673	0.226878	0
27.158729	26.85863	21.58827	22.454011	6.695243004
0	0.10252	1.93971	3.95369	1.68179
2.20E-05	4.46E-05	2.48E-05	2.01E-05	6.38E-05
0	0	0	0	0
6.326087016	5.9410663	3.590958206	4.916015206	2.846378
9.311023679	9.291796217	6.07840563	9.625461459	4.174927886
9.311023679	9.291796217	6.07840563	9.625461459	4.174927886
22.39388403	20.5985	14.0386396	18.22375486	11.936
0.11228	0.224422	0.492492	0.662348	0
20.1468	24.44428	23.98636	30.2825	39.5953
0	0	0	0	0
0	0	0	0	0
0.256926	0.769392	0.329291286	0.308562423	0
0.570548374	0.674266646	0.509019095	0.29011778	1.170666169
0.353547	0.240332	0.234183	0.637367	0
40.10583715	44.680006	46.578221	44.724815	57.897
21.2714852	19.60971411	20.645607	21.99794973	0
21.2714852	19.60971411	20.645607	21.99794973	22.74176204
21.2714852	19.60971411	20.645607	21.99794973	22.74176204
21.2714852	19.60971411	20.645607	21.99794973	22.74176204
21.2714852	19.60971411	20.645607	21.99794973	3.6894
0.517263223	0.592497083	2.44415	1.71969996	0.933182
0	0	0	0	0
0	2.39998	2.16145	0	0
0	0	0	0	0
1.870310029	1.490092873	3.091204335	3.006070051	1.184871
3.54473403	4.03751533	4.80700286	4.16898334	42.98885
0	0	0	0	0
0	0	0	0	0
0	0	0	0	0
0	0	0	0	0
0	0	0.0579298	0.0776988	0.176544
0.108147	0.348161	0.209273	0.0698341	2.15676

2.791122	3.14578	4.97101	4.09312	3.88325
30.5099	27.214	42.8449	44.9163	49.2377
1.121133109	1.2142242	0.969386524	0.686591024	65.1235
0.109481	0.110104	0	0.143489	24.758592
0.117216	0.237761	0.0580539	0.155678	24.758592
9.6197	12.32109	14.88326	14.23896	58.5069
18.41052	20.12644	15.539435	16.7235763	45.2991
13.611074	14.49311	18.29517	17.429536	100.7975
0.312668	0.960515	0.602725	0.828801	4.77062
0.61776	0	5.03054	1.6265	2.87706
0	0	0	0	0.74967
0	0	0	0	2.39605
13.74210924	15.429383	11.00051855	11.10395488	5.438842415
13.74210924	15.429383	11.00051855	11.10395488	5.438842415
16.42142	18.06969	18.33231	19.09683	30.06082
28.70260851	29.75055963	35.3439863	36.49644406	40.34494828
91.997262	89.1171281	78.95210902	85.190145	115.4809
0	0	0.726037	1.4924	0.879465
13.60844	14.08421	7.00186	8.5298	3.87519
18.82694142	17.741552	24.89189	21.37334415	28.65540079
30.82045713	28.88841317	22.72264897	24.15880567	20.143834
207.4319	184.6408	62.396898	77.82477	0
207.4319	184.6408	62.396898	77.82477	15.87848
53.83964	54.25818	54.50193	58.976943	72.4756382
53.83964	54.25818	54.50193	58.976943	72.4756382
52.22668	53.36156	47.15334	39.35278	65.4344
0.110149	0.333586	0.106835	0.437523	0.130081
15.31268588	15.5796956	10.27161296	10.13932882	2.310589478
15.31268588	15.5796956	10.27161296	10.13932882	2.310589478
15.31268588	15.5796956	10.27161296	10.13932882	2.310589478
20.08304	21.4485393	22.404296	19.35693	21.74304
19.6359	18.6008	20.7743	15.5054	2.57978
114.5087237	118.8677606	118.7824062	105.7164566	0.818495
114.5087237	118.8677606	118.7824062	105.7164566	61.1625
15.31789296	17.69848002	16.52340048	16.1383737	25.35701317
2.56728	2.25405	2.307	1.11528	6.86432
50.06151013	48.56926066	55.72888863	57.4408595	37.64550288
50.06151013	48.56926066	55.72888863	57.4408595	37.64550288
50.06151013	48.56926066	55.72888863	57.4408595	36.5787
1.479454	1.322803	1.428498	0.922194	0
0.326355	0.0819951	0.709435	0.844013	0.665055
30.031	42.2432	39.9438	30.1064	64.5249
31.02930233	34.13390384	28.65750157	25.63510373	54.80360803
40.94167183	43.58975352	30.39098006	28.31993555	13.756676

5.58942	4.17404	6.89484	4.74903	0.000352773
38.117068	33.28756231	28.82435011	37.61892448	53.8517
279.8524	278.8276	306.2309	230.9469	215.0632
151.492	146.089	203.782	163.746	81.0093
8.58209	9.10048	11.72489	8.95811	52.343
23.8012	32.5858	30.6681	17.3133	33.2401
52.30655539	60.66437182	77.11606988	72.29762498	9.42722
52.30655539	60.66437182	77.11606988	72.29762498	36.27526718
52.30655539	60.66437182	77.11606988	72.29762498	88.2967547
64.106	73.26995	43.2997	36.58691	6.118446297
27.22645	22.05052	2.037146	4.143431	0.405906
23.53388502	22.78330505	21.81212609	22.64427505	33.3561208
121.3985676	92.89163703	51.394154	60.58952	3.724912126
121.3985676	92.89163703	51.394154	60.58952	3.724912126
121.3985676	92.89163703	51.394154	60.58952	5.408302452
336.52148	284.4883182	412.9039	330.30945	288.649
7.83238	6.33465	10.4782	10.1801	33.5295
0	0	0	0	0
23.0751418	23.16217	17.8421657	20.4524143	15.74860237
23.0751418	23.16217	17.8421657	20.4524143	15.74860237
43.87920219	42.89560695	60.693346	65.1025762	6.060197796
50.00976631	48.12076071	66.01435494	55.69239571	62.76914
0.449024	0.666567	0.32365	0.144038	205.6257
25.28151973	29.82859263	21.6250128	21.07892198	19.581706
363.775	363.239	427.287	504.531	518.154
27.26148322	29.814974	24.617064	18.31150085	2.37014
16.59163	17.95654	26.02507	29.28384	17.6628
16.59163	17.95654	26.02507	29.28384	63.7717
14.295752	16.57744	26.68701815	24.421401	44.96097
14.295752	16.57744	26.68701815	24.421401	44.96097
0	0	0	0	0
45.45036	40.63009824	14.462025	16.43547669	2.78897
7.24071	3.52431	30.3402	40.7678	78.3547
2.42E-05	2.78E-06	1.86E-06	4.19E-06	4.20E-06
1.7056	3.40642	1.30312	1.10232	1.59477
21.2178	21.7446	12.087269	13.1154	4.85701
51.53792	52.23233	50.29116389	41.69941373	58.9814
5.9482	8.00097	7.62951	8.28449	28.8796
0	0	2.17705	14.8302	231.081
58.5982	56.14746	63.63948	70.34777	71.04528
10.9299	10.02236	14.07214	12.68126	41.7744
13.26782	15.51973	17.45468	16.96101	88.17770035
31.397139	31.096271	23.384555	22.597671	9.90489
1.66152	0.74853	3.6074	2.0162	0.596545

7.4044	8.78879	6.17738	6.65972	7.38E-05
1.638119	1.413808	1.32873608	2.084126356	3.044678741
13.42657516	12.36410279	13.29690603	12.22368799	24.0751
8.76670663	7.434460367	9.00617	8.130374	14.71947234
865.56	955.689	1539.519	1396.731	1162.27
13.09402	19.26926	34.0243	22.43748	47.8284
11.2783	11.6401	18.6781	13.962	39.0561
20.44379	23.6901	21.23086	12.72514	2.0431
49.56873701	43.454067	41.2280865	42.408591	33.7662267
49.56873701	43.454067	41.2280865	42.408591	33.7662267
20.55008	15.09966	8.47329	7.965008	2.405419994
56.86740232	59.67206024	52.33200167	53.0774146	10.04313187
107.5766	98.1418	7.55451	30.2525	1.674703901
0	0	0	0	0
141.0465243	132.354259	94.2539707	73.8768499	26.03890525
14.73411711	15.24844203	25.28002805	17.390305	44.14985
56.66826826	45.268639	76.3018689	70.29792532	57.78827
4.410483	3.52817	13.93314	9.06511	2.67261
1060.286974	1125.211018	84.57162429	168.6115099	19.9423851
281.5820636	318.887867	17.2860147	35.72485228	5.479280221
26.66592115	23.29742161	10.79157886	11.15572361	1.069068051
4.7548	4.51046091	8.546906	8.699772256	22.43101025
7.82464	10.1956	11.3539	11.5366	4.01811
1.68437	2.05192	2.63501	1.45379	1.17552
10.179231	10.13104	13.1453928	12.45115199	40.81835953
17.35952796	23.22892111	6.866144469	6.550300788	23.6239259
9.037	8.89609	11.1693	11.1304	4.57872
33.54098696	40.1776	35.59806397	37.41496485	61.26867
42.073583	37.72144	4.766387	11.15732505	0.496105
7.595859053	6.687071	11.96411597	10.138136	47.2900355
20.11465	13.67325	13.33250478	15.06773	15.01630103
595.8411062	320.2359237	149.4132473	261.5724167	2.44723
0.341393	0.485623	1.14931	1.99615	0.44617
59.5237	56.8177	40.6226	46.2288	10.6193
4.87111	4.54313	4.72994	4.71538	2.90833
4.476020347	4.029453	7.723358333	6.225724392	17.45559754
4.476020347	4.029453	7.723358333	6.225724392	17.45559754
30.5479	30.2424	23.3882	26.7657	64.6367
98.356419	81.96536373	46.74961506	52.0348264	18.577144
98.356419	81.96536373	46.74961506	52.0348264	0
98.356419	81.96536373	46.74961506	52.0348264	6.938724455
98.356419	81.96536373	46.74961506	52.0348264	6.938724455
0	0	0	0	0
7.46539	5.39724	8.55432	4.07393	0.493349

12.99028	13.16359	13.282228	14.47226	29.46483
54.8212	43.869	16.3838	21.4469	1.81286
24.4586	25.2362	27.3156	28.5211	32.1076
2.60722	3.80565	3.52186	3.26235	4.04608
141.6487	136.5034	139.42154	144.1639	91.2601
10.21892	10.13381	15.55706	15.8449	41.1162
116.64949	144.79808	502.7129	271.32683	29.94001654
38.60706	50.54502	54.45280211	54.50240239	3.45907
38.60706	50.54502	54.45280211	54.50240239	32.183
0	0	0	0	0
5.50238	5.78314	5.487575	4.492644	1.742843122
0	0	0	0	0
33.59221	37.75308	21.17385	20.555581	19.607984
36.67561	38.70221	27.2253	26.90142008	30.23380999
29.1637	26.9029	31.7751	27.0577	51.2307
72.9718	79.2885	82.1857	79.28	170.6125
7.16305	9.22484	9.94805	9.70681	30.8859
9.09292	12.4016	13.3807	9.44786	26.2053
24.08891398	21.69773471	3.5338744	6.972193945	1.80123
92.412	72.80599	34.99023	41.19916	2.73604
32.96853	35.2321417	30.21281	32.384082	42.07181836
99.44619	121.01948	77.39268	79.41816188	167.251
253.033	210.607	322.832	253.788	202.055
19.08042101	17.30807	22.58082074	20.7381709	47.72830012
19.08042101	17.30807	22.58082074	20.7381709	47.72830012
24.5016	24.3229	24.299	28.1845	81.8001
0.359607	0.073155	3.3558356	5.074632862	4.63361
21.595234	23.76143	30.86182	39.29131322	53.4253
21.0334	20.3058	27.4608	20.0117	77.813
735.021	778.437	1141.85	1131.62	1547.12
15.442	9.04457	15.0831	5.65271	50.3597
11.95778169	9.853073	28.67143775	25.138247	14.98615443
5.73E-08	5.67E-69	0.00470629	6.11E-16	3.15E-05
21.091	16.37692	23.7873	16.75546	24.6295
3.14242	4.39741	3.39224	2.46653	7.75805
10.08212	10.49782	13.69993	16.65053	61.365
15.82621	17.35926	17.05354	14.4595	38.38297
69.70530519	56.37130923	72.93180926	73.6646054	35.5773
69.70530519	56.37130923	72.93180926	73.6646054	102.25402
0	0	0	0	0
3.61783	1.28817	5.56066	3.08963	18.5151
1643.93	1764.31	2195.71	1455.53	378.362
27.29326	26.03146	23.43021	25.43987	27.78456
78.219794	70.4055371	49.85002527	49.26613362	11.38658325

5.01862	4.49384	1.590331038	2.0264089	1.10171
21.51438	27.14953	23.3686513	26.39047	40.53759
0.373984	0.573121	0.653529	0.507111	0.667368
0	0.18153	0.641005	0.546873	0
0	0	0	0	0
672.004	466.214	228.2287	311.431	5.25849
2.382371	3.80294	6.30423	3.284065	0.951789
5.48997	5.21905	6.31445	5.37113	28
16.36418	18.87102	13.70836	15.70483	28.4546
11.47500919	13.5676528	17.12217346	15.12355151	39.4962
31.64312	30.20762	37.70209	31.64333	8.96597
31.64312	30.20762	37.70209	31.64333	52.28473
2.40121	2.21753	3.85248	3.14954	21.7773
9.877116	10.48513	8.433949413	11.583448	0.000627231
26.30120007	24.11281193	16.54340003	17.9727	3.67487225
100.0944648	92.84629324	71.8729272	78.0059577	16.98090564
0.071287082	0.072698598	0.047199255	0.126297832	0.929785
8.78473	6.66649	0.800955	0.0576112	7.14E-06
8.84547	11.2477	14.1259	10.5092	57.327
3.902440204	3.684187	2.726941	2.560405	2.31376
2.326022	2.0670658	3.17602	3.473640206	1.705244
2.326022	2.0670658	3.17602	3.473640206	5.7809
0	0	0	0	0
20.68031	19.625272	14.3942202	13.210677	3.689679
24.3183	17.85706	25.2857	32.9193	36.904
91.522325	81.51404	40.68233	39.403569	0.633287
91.522325	81.51404	40.68233	39.403569	13.29734
26.1488	30.3035	28.3898	30.8311	34.56815797
0	0	0.40287	0.83844	719.684
8.99202	8.674915529	12.80051359	10.672569	45.6130308
6.41963	7.12262	9.02909	11.20933	26.44121
90.8516	82.4113	46.0349	44.0558	3.58261
0	0.292627	0	0	3.083171231
6.47446	5.37875	2.78028	1.872943	3.083171231
0.41918	0.425096	1.64078	0.656583	0
6.443994458	5.46254904	6.186726	5.500832132	5.046240788
0.38297	0.436657043	0.376913	0.454781705	1.6102
1.286626	0.87298539	0.669741181	0.7070018	40.8348
56.1983	47.38611	11.66322	16.10096	2.682417411
6.82548	5.087543	52.91213	36.39465	55.0623
6.18E-05	0.178812	0.000556561	0.000286353	6.63031
1.788531	1.465579	1.578346	1.699082	1.58376741
16.98234071	10.80819639	23.79739241	19.88695803	5.47501
2.40994508	2.203011884	4.252604401	4.925329542	109.9467

4.91271	4.71334	4.78589	3.719666064	40.5775
18.21957	14.72299	17.81415	16.60689081	36.0433
21.71865	20.68733	15.383627	18.1622	19.8205
0	0	0	0	0
40.35886	43.37096	77.667593	75.48517	80.1364
40.35886	43.37096	77.667593	75.48517	80.1364
206.306753	235.940492	357.88133	308.12846	88.1893
63.64084	82.4759	89.22087	74.88525	17.42855
63.64084	82.4759	89.22087	74.88525	17.2232
49.45773384	49.28315568	48.07163108	49.20961874	63.2679
6.80944	7.28946	11.027	10.1293	25.5265
16.82476414	16.3027154	15.387724	14.22539838	10.54306
0.579949	0.673861	0.450697	0.556387	18.2764
3.58E-13	0.00742456	6.15E-19	1.82E-09	0.00129565
4.916534	6.585181	7.272014304	5.94602034	28.30765
3.1506043	3.185741369	5.092847729	4.666266509	170.04024
62.21724926	65.54187664	60.01955523	60.4395609	170.04024
62.21724926	65.54187664	60.01955523	60.4395609	170.04024
62.21724926	65.54187664	60.01955523	60.4395609	20.8192
2.8451	3.44064	4.88758	5.30183	31.2107
13.05379859	13.17999965	15.88654561	13.25351683	8.054358
13.05379859	13.17999965	15.88654561	13.25351683	8.054358
0	0.0723596	0.495403	1.89338	0.986067
0	0	0	0	0
0	0	0.118297	0	0
7.30904	6.94186	2.69347	2.56403	0.665155
0	0	0.0575436	0.0766142	0
0	0	0	0.378384	0
0	0.193587	0.0943148	0.253076	0.115964
0	0	0	0	0
0	0	0	0	0
0.111405	0.227466	1.61844	2.31235	0.520679
0.111405	0.227466	1.61844	2.31235	0
5.86898	6.63188	11.4386	8.03245	23.5001
6.604133734	6.523364305	6.805050021	7.251018364	6.505641233
0.935296	0.94169	0.927497	1.23703	13.9436
1.17912	1.4401	1.393051327	1.73179	0.262055
0.0919187	0	0	0.120181	0
0	0	0	0	0
0	0	0	0	0
1.766602	1.498331	2.842046	1.74406556	0.956045
0	0	0	0	0
0	0.0416132	0	0.0546609	0
0	0	0	0	0

0	0	0	0	0
0.0641554	0.0637559	0	0	0.143196
0.573274882	0.21914925	7.41819187	14.885632	18.94097663
21.46814943	23.3791836	20.39917923	22.48717981	57.10765
0.815125	0	0.499892	0	0.627136
0.287977	0.144098	0.422415	0.282302	0.820991
0.632592	0.56437	0.97703	0.370227	1.83399
5.85751	10.43047	1.7724	3.08605	2.395409662
0	0	0	0	0.176793
0.102866	0.032555	0.3124665	0.931822	2.25413
41.13156475	40.19235312	31.65621448	38.77858896	43.78531
0	0	0	0	0
0.080983	0	0	0	0
0	0	0.20609	0.275424	0
0	0	0	0.376938	0
0	0	0	0	0
11.2765018	9.473292	4.554860637	4.676942308	1.26863084
25.077687	21.845843	34.459831	27.46884	43.654344
86.3057	56.26669	50.22556	53.6103	48.108505
86.3057	56.26669	50.22556	53.6103	48.108505
61.90888	56.951529	36.149251	35.166414	65.9203
561.2655	616.8073	948.7108	920.595	1043.4242
1.605909836	1.485535708	0.7587625	1.02600147	0.163299
0	0	0	0	0
5.74982	7.97771	10.5469	8.31131	174.041
2.117594816	2.275416985	2.33952823	2.66413173	1.042276866
0.218413	0.221053	0.213271	0.286188	0.132013
1.592016818	1.261641282	4.309683919	3.879181449	5.38017
789.922	909.321	1226.84	1186.76	237.656
20.1309	21.6695	26.918	24.4698	38.9444
44.0118	35.4446	20.75165	16.3225	1.61886
380.59	366.3624	471.971	377.638	366.696
0.5555926	0.267226297	5.05528	8.12104	5.35309
13.710268	13.08627215	9.368021	10.54311801	2.791752904
0	0	0	0	0.0725963
0	0	0.189784	0.257297	0
0.887932	1.4576	0	0	0.00123166
0	0	0	0	0
0	0	0	0.152831	0
0	0	0	0	0
0	0	0	0	0
0	0	0	0	0
0	0	0	0	0
0	0	0	0	0

0.579276475	0.540093997	0.847411148	1.87044472	0.489485583
0	0	0.039317	0	0
0	0	0	0	0
0	0	0.536011	0	0.381113
0	0.1639	0.185022	0.0357763	0.0327932
0	0.0319376	0.0310133	0	0.0385612
18.32877342	18.70579784	24.82757249	20.74353696	82.86513
18.32877342	18.70579784	24.82757249	20.74353696	82.86513
6.391569	6.72979	8.895157	8.348091	22.16023616
0.375358278	0.105252931	1.707445	2.681426	0.65258
27.74955207	44.183388	5.340160876	4.913573342	4183.699
11.087961	9.743278	14.91286964	15.60031679	14.8478
11.087961	9.743278	14.91286964	15.60031679	17.9974
93.9887	106.483	70.2179	65.7333	27.135641
6.78359	6.474456863	18.856851	19.22795	46.1852
5.016983496	4.342212797	19.38264954	21.23962	15.30695
32.56752	29.08929	40.74397	47.50565	149.9612
0.59724	0.665935	0	0	28.2855
12.8882	14.2246	22.9545	22.4262	7.30773
48.13497692	43.15878046	30.32392391	33.60113022	10.10680932
44.8985064	47.00994129	31.46808679	38.24189224	20.8699
16.8543	16.8155	45.9852	40.1954	84.92840014
0.040002	0	0	0.0525909	0.18897
17.6204	24.6021	40.0392	31.6726	9.68044
1.60851	0.00527075	4.63074	7.84055	6.9208
0.81393111	0.170176174	3.678384	7.32624	2.46684
0	0	0	0	161.407
0.0228434	0.0232741	0	0	20.1665
0	0	0.20103	0	0
0	0	1.427149365	5.671117	7.813335
0.0523896	0.212057	0	0	0.0626686
0	0	0.0454829	0	0.107488
0	0	0	0	0.25763
0.377636996	0.32672792	0.248044557	0.497045679	0.0428918
0.163597	0	0	0.21768	0
0	0.166319	0	0	0
0.144978773	0.432060174	0	0.744813	0
0	0	0	0	0
0	0	0	0	0
0	0	0.106947	0	0
0.59104394	0.746984177	0.638673927	0.72631343	0.260206
0	0	0	0	0
2.132672105	0.877968298	1.148775817	0.611973669	1.444782345
9.418865	9.325477188	9.980122179	8.90227275	12.75956088

9.418865	9.325477188	9.980122179	8.90227275	12.75956088
36.19822594	35.31923536	45.57540946	42.30210692	22.20158436
36.19822594	35.31923536	45.57540946	42.30210692	22.20158436
47.70513	44.36653	32.35131	32.198385	5.925177117
47.70513	44.36653	32.35131	32.198385	7.77605
0	0	0	0	0
0	0	0	0	0
0	0	0.0983846	0.669531	1.42257
0	0	0.0914422	0.247755	0
0	0.318639	0	0	0
0.3418	0	0.32528	0	0
0	0	0	0	0
0	0	0	0	0
0	0	0	0	0
0.237866	0.311435	0	0	0
29.29892519	26.829909	18.257892	18.694991	6.357541214
29.29892519	26.829909	18.257892	18.694991	6.357541214
13.79872201	16.05413986	16.17635605	20.75144801	6.938443053
0	0	0	0	9.65086
0	0	0	0	0
0	0	0	0	0
0	0	0	0	0
0	0	0	0	1.29923
17.33347491	15.349351	46.018557	32.65726	28.67703244
17.33347491	15.349351	46.018557	32.65726	28.67703244
1.338881966	0.740999859	1.2553074	1.481551	1.407726933
0	0	0.178624	0.120611	0.320774
0	0.357711	0	0	0
0	0	0	0.406323	1.79928
0	0	0	0	0
0	0	0	0	0.206475
0	0	0	0	0
570.8777	426.699	249.2871	240.1953	11.41161887
13.8601	13.927	25.1564	17.502	56.1659
18.75287	18.37128	20.32403	18.81617	37.0229
20.89898299	17.18626359	20.93267745	21.17332066	19.33990802
0	0	0	0	0
0.0845454	0	0	0.167532	0.0494552
0.0500487	0	0	0.079378121	11.54425
12.6105263	11.7197312	15.44656	15.55544842	44.32962316
12.6105263	11.7197312	15.44656	15.55544842	44.32962316
924.851	958.194	1355.53	1438.35	1460.05
43.5454713	42.53479	40.06561	47.355037	48.3145
6.989308	8.21004996	11.50057882	9.945927023	28.603173

782.29749	847.90738	1224.8113	1190.608074	2434.55
0.842232927	0.794625758	1.589807012	1.432505492	17.5543
57.04893	52.72399	57.60673	60.4773	49.86242
100.0742	71.6857	116.3296	94.5336	147.1017468
4.245680383	3.100380874	5.9257174	8.31709923	8.807446568
0	0	0	0	0
71.5809308	78.21204	67.88332	61.60822	137.3398
0.714334498	0.200927601	2.53115001	2.863890824	0
0.714334498	0.200927601	2.53115001	2.863890824	3.88789
10.31936474	9.750877061	16.990936	47.911529	0.276913
10.31936474	9.750877061	16.990936	47.911529	1.271620308
10.31936474	9.750877061	16.990936	47.911529	48.0092
3.204145164	2.221978711	12.70253872	19.44633572	54.41657
18.094422	17.09570437	2.867100734	5.844721916	0.252111
0	0.375189	1.82239	6.40153	1.99016
0	0	0	0	0
0	0	0.515388	1.41883	0.627071
0	0	0	0	0
6.46431	4.52922	25.2575	30.2837	17.2072
16.4498076	16.91687238	20.78461933	23.06371503	34.77097587
4.204916	3.294917869	8.566692	6.0602539	7.11666
4.286422	5.843999	3.482278	3.108153244	3.818817
16.90550917	19.96171685	15.5085076	15.77749095	15.72693033
16.90550917	19.96171685	15.5085076	15.77749095	15.72693033
15.25094	21.58591	21.83456	18.70365	72.60176576
5.341005947	5.696426088	7.675606505	7.481561004	34.07466888
5.341005947	5.696426088	7.675606505	7.481561004	44.723953
17.50568	17.55735538	16.4141971	16.50774495	9.246328277
25.07125005	22.49080217	32.04939003	32.75546784	39.8589
0	0.110889	1.21961	5.42088	3.28754
2.1721325	2.608976142	4.86566726	4.781800517	22.89565805
2.1721325	2.608976142	4.86566726	4.781800517	22.89565805
2.1721325	2.608976142	4.86566726	4.781800517	22.89565805
0	0	0	0	0
0	0	0	0	0
12.503455	12.284796	13.433159	10.845475	11.046511
0.454066	0	0.108168	0.145654	0.409587
0	0	0	2.72439	0
51.9181	51.28383	20.03478	20.11264	1.41719
64.98260277	66.10440454	18.252895	23.11551016	16.85347
64.98260277	66.10440454	18.252895	23.11551016	16.85347
19.119909	20.30529	18.85121	17.94281	50.33669
207.7725235	198.6243962	243.9661209	144.0393794	25.84688999
20.59198294	19.74665003	24.8713	25.735239	37.93118938

28.024838	28.71863953	26.09748007	26.005533	10.5795406
9.74644	10.6938	19.4682	12.6844	40.00590203
19.18205	15.39178	21.61426	19.21406	54.35784092
0	0.221454	0	0	0
26.553466	28.14629093	29.430019	26.27413784	62.25872985
67.131704	70.460803	69.0119004	73.6465	50.78590147
42.0753	45.0415	58.9436	48.9594	77.6328
5.49437	4.634322	0.852866022	2.439308	0.473675594
27.0841437	30.46296322	34.120168	30.61206168	74.454691
35.9426649	32.82476935	32.55623113	38.93647493	12.25000107
11.50945	13.83547	16.84475	13.01448	15.56090829
17.153817	8.459251	1.604626119	3.14306916	2.183903
17.153817	8.459251	1.604626119	3.14306916	2.183903
17.153817	8.459251	1.604626119	3.14306916	2.183903
17.153817	8.459251	1.604626119	3.14306916	1.11209
8.88347907	10.86681356	9.112563917	8.418542694	38.650528
0	0	0	0	0
1.33891	0.975434	0.6472559	0.0980269	0
0	0	0	0	0
59.15075072	50.2472711	53.23086928	45.452806	16.93417
59.15075072	50.2472711	53.23086928	45.452806	16.93417
28.17880177	28.719202	27.85170117	27.870246	24.365051
72.8081	72.01618	68.49833	67.80345	240.6639
1337.33976	1329.311	1550.25236	1231.973576	493.598
91.777806	83.785784	81.69674629	86.2436231	70.05297
0	0	0.289959	0.5918	49.98251888
18.79149289	19.45048186	18.30181137	20.22468	26.64972
88.242533	97.35855	70.314453	70.183105	14.27469439
88.242533	97.35855	70.314453	70.183105	14.27469439
88.242533	97.35855	70.314453	70.183105	84.83497
22.76939362	26.8748	28.41643	27.71885281	44.4046054
32.55596691	31.2090317	27.63709616	30.52557355	83.7934
0	0	0	0	0
4.82833	5.03398	4.4015	3.7814	223.633
267.29389	285.6725	418.1056	518.2508	223.633
34.673445	28.974998	24.714793	21.82856	4.277500001
18.34665534	21.05195341	23.8135574	19.75621723	110.0460173
0.178393	0.231221	0.672752	0.785763	9.187012
1.283439	1.055768	3.39713	2.33483	0
46.58024678	35.88229836	38.87073232	40.85224372	20.19773491
52.44043591	58.38121316	41.24405202	40.96710466	99.7496
0	0	0.240255	1.29002	0
50.3325	49.0634	40.5071	37.3344	48.4141
48.63256462	61.51527915	38.59472455	42.47945549	31.45298421

9.69239114	5.888177144	8.26362459	9.05386666	30.50972
0.313546672	0.104094303	0.271935164	3.937073802	8.346903655
5.067655013	4.556680946	8.237699004	6.260314392	13.40382232
5.067655013	4.556680946	8.237699004	6.260314392	13.40382232
60.34667	61.395831	45.03112	45.7842585	23.13199157
60.34667	61.395831	45.03112	45.7842585	23.13199157
93.67957341	89.15208241	85.59141172	89.97709868	73.87736158
0	0	0	0	0
16.2714	14.0578	16.78742703	16.94910392	53.14667729
24.90352	32.42245634	26.521049	27.04627708	10.78826493
24.90352	32.42245634	26.521049	27.04627708	10.78826493
0.862467366	0.950429434	0.936039345	1.736264	0.492705
3.6972964	2.719182	6.206887	5.00387	31.0125
3.97693	4.436075	6.206887	5.14945716	3.63363
0	0	0	0	0
0	0	0	0	0
0	0	1.30922	3.28242	1.85652
11.6207014	9.208134067	7.689110083	8.262211213	4.644171
11.6207014	9.208134067	7.689110083	8.262211213	4.644171
11.6207014	9.208134067	7.689110083	8.262211213	4.644171
2.52972	7.06373	7.93194	2.44404	8.85E-05
685.01211	472.82147	231.38766	294.46441	9.344091
6.452173	7.36256	9.266454	6.672335	0.14777
6.452173	7.36256	9.266454	6.672335	29.146
5.980615659	5.66839952	10.18791024	7.72405705	118.9727037
35.56359015	38.57711036	28.50627055	30.32787	44.9328
35.56359015	38.57711036	28.50627055	30.32787	44.9328
23.57563	22.81931403	14.14988452	17.84230519	3.189945941
34.1431135	39.23165	29.66644959	28.77558842	51.49234
5.53873	4.97621	5.35464	7.41393	2.96759
8.004663	7.1025355	15.52917	13.07884	30.7115
30.9771	28.15166	38.91275	31.09407	60.098
7.251383368	7.545544887	12.2964706	11.31823156	8.793618433
7.251383368	7.545544887	12.2964706	11.31823156	8.793618433
0.592581045	0.75751162	0.885989746	1.232932517	0.468352
0	0	0	0	0
0	0	0	0	0
0	0	0.674621	0	0
0.941101	0.317054	0.290913	1.20092	2.80619
0.116456	0	0	0.459852	0
0.0669535	0.272153	0	0.0895313	0
0	0	0	0	0
0	0	0	0	0
7.888988768	9.406448001	9.779675	8.31358	16.993041

7.888988768	9.406448001	9.779675	8.31358	16.993041
103.335768	96.73559	35.20188705	32.13966767	1.703831
8.05443	6.93951	7.50639	5.12239	21.09994
0	0	0	0	3.24002
0	0	3.644858	20.41458	408.309
28.05362454	26.17400864	22.6145737	15.08432381	3.26292
5.91E-05	1.37E-05	0.000353673	1.78E-05	17.19965
0.69141756	0.933758	1.073172787	0.438749385	2.85804
25.017047	27.46934	38.90905	39.219976	24.856358
0	0	0	0	0
14.43277	14.87787014	14.4375797	13.21217289	4.724444747
73.134619	64.302651	90.866905	69.1342	16.80913
73.134619	64.302651	90.866905	69.1342	0.484749
0	0	0	0	0.375822
0	0.132905	0	0	0.161072
0	0	0	0.12119	0.117839
7.22E-10	0.124917	3.31961	7.96902	1.7107
0	0	0	0	0
0	0	0	0	0
6.02182	7.7578	8.87669	8.93124	10.0038
49.221212	54.34615602	56.185703	49.95159	37.49048902
49.221212	54.34615602	56.185703	49.95159	46.26639054
49.221212	54.34615602	56.185703	49.95159	46.26639054
6.165106	5.500299	7.174053045	4.848902224	2.0915605
6.165106	5.500299	7.174053045	4.848902224	2.0915605
0.357747051	0.3829425	0.62117604	0.377858662	0.525685
0	0	15.2565	63.6416	746.786
0.398894	0.815568	11.84564	32.69028	163.555
0	0	0	0	0
0	0.253686876	0.0619697	0	16.90568
0	0	0	0	0
0	0	0.645896	1.51247	0.423432
58.0173982	53.771501	41.14408586	50.69111253	33.30651001
58.0173982	53.771501	41.14408586	50.69111253	33.30651001
58.0173982	53.771501	41.14408586	50.69111253	33.30651001
58.0173982	53.771501	41.14408586	50.69111253	33.30651001
0.176096	0.354234	0.422835	0.459222	1.758050005
19.96632	22.04201	24.18574	22.25927	19.53701404
3.69E-05	3.44E-05	1.01E-06	0.0322573	4.32E-05
0.425200618	0.352893862	0.196204853	0.46909937	0.0653982
0	0	0	0	0.0479026
0.0840243	0.0853044	0.166161	0.112181	0.0505234
0	0	0	0	0
0	0	0	0	0

0	0	0.111203	0	0
20.28781037	19.09170085	15.1864297	16.47012212	1.947732
20.28781037	19.09170085	15.1864297	16.47012212	1.947732
118.0695211	116.2432388	160.41714	121.0852271	87.72118
48.11223	52.337063	67.0837	57.85713	55.3267735
2.26248	3.28045	6.51885	5.99729	25.347
27.4159875	28.37981303	21.35219101	24.160161	37.79821
27.4159875	28.37981303	21.35219101	24.160161	37.79821
32.581013	29.53855	17.59067	17.20654	6.19512
24.32132101	25.1309861	23.31186219	20.117765	0
24.32132101	25.1309861	23.31186219	20.117765	22.21710787
24.32132101	25.1309861	23.31186219	20.117765	0.918012
49.35845171	48.89798579	55.47086859	51.12987023	148.58758
41.6982	85.198	178.186	171.059	1491.61
724.03587	799.17343	1122.180117	1068.772915	924.5076
547.7843	371.5584	210.7967	273.6917	5.372998
9.74545	12.3262	14.2451	16.5636	16.7879
0	0	0	0	0
20.85654015	18.57903657	14.18514096	16.67638648	0.25441
0	0	0	0	0
20.85654015	18.57903657	14.18514096	16.67638648	25.7790703
2.438008204	1.926036782	3.0553508	4.020126233	10.843977
2.438008204	1.926036782	3.0553508	4.020126233	10.843977
0	0	0	0	0
0	0	0	0	0
0	0	0	0	0
0	0	0	0	0
0.434683	0	0.143858	0.191221	0.18078
35.37244958	35.97424948	48.73575447	44.03307277	51.89527
17.26167	14.42264	12.98518	14.05202	8.21706
10.48677	11.810801	14.4174	12.25865011	36.6519
25.80224312	26.78117957	22.88138	30.82054	58.8738
0	3.33337	6.76919	1.05718	50.2326
38.349223	41.147192	33.44070378	34.69956	27.48981
38.349223	41.147192	33.44070378	34.69956	19.79443873
38.349223	41.147192	33.44070378	34.69956	19.79443873
76.5737	56.08085	65.8809	49.90927	7.651635
30.1164619	33.8100212	4.908577977	7.219286	0.996526
30.1164619	33.8100212	4.908577977	7.219286	0.916444
30.1164619	33.8100212	4.908577977	7.219286	3.02539
6.38268	14.7175	2.15071	4.41005	719.687
1.715809187	1.994961369	4.11435146	4.171731022	52.36812
22.66375699	21.00060012	16.86850865	19.76999995	8.30019145
0.0468802	0.0962888	0.139776	0.0629993	0

69.150787	67.099988	58.09178014	58.12961	59.72141048
7.46534	8.85375	6.5392	8.07628	3.991101435
11.972036	11.17629815	11.89339011	14.444512	56.26102318
1.92E-55	1.04E-06	0.257762	0.914178	0.000143886
19.106393	18.49631277	14.674826	16.37054254	8.5046
4.309073	2.81503	7.2903434	4.932081	4.503236924
48.85594	50.15941	78.03016	67.79019	121.49
22.659664	24.21539	26.6667	27.234946	67.769
22.659664	24.21539	26.6667	27.234946	67.769
0.0492511	0	0.588518003	1.842437863	1.241266446
36.8979715	40.74352386	32.06597921	34.8518532	38.808536
0.473473	1.045252	1.486443	1.029625	0.721256219
0.901494115	1.354873	1.425052615	1.662317	3.532894
9.62678	7.4400639	8.43244	7.73231	13.80135
378.419993	376.707817	526.0089795	427.96899	231.288
378.419993	376.707817	526.0089795	427.96899	2.773419
31.65361	34.616859	34.45780324	35.70890416	30.33640165
26.4611	31.9512	28.5754	22.3349	101.7345
684.416	690.394	1050.216	1032.51	1027.49
25.27241188	29.4789141	18.92700146	21.27464985	11.99219
5.130531	4.684552014	5.731487	4.953254	4.79057
48.7858	40.185	24.195308	24.4544	8.566504923
48.7858	40.185	29.430019	26.27413784	8.566504923
132.962	137.357	114.265	87.698	64.9428
0.428847525	0.532179061	4.73645361	9.365956534	7.953340885
0.428847525	0.532179061	4.73645361	9.365956534	7.953340885
31.67335	39.26725	31.21361	32.07405	16.62989
13.9106433	14.07104	10.714375	11.51950434	4.321969309
10.82878041	12.39614356	20.16952995	16.47962989	11.398494
27.30325279	26.826579	37.02810434	32.23554608	0.732522
27.30325279	26.826579	37.02810434	32.23554608	0
27.30325279	26.826579	37.02810434	32.23554608	63.33751
0.479267	0.557979	1.9485649	2.98315	64.01342
41.809094	45.59365	36.04104	32.33387	8.57149
41.809094	45.59365	36.04104	32.33387	8.817651092
27.70088	26.9025	23.1437	20.72652	10.22434
11.594043	12.691736	14.04799906	12.4162034	4.931215967
11.594043	12.691736	14.04799906	12.4162034	4.931215967
0	0	0.211272	0.288143	1.28264
7.57786555	8.87633	8.239717	6.54932396	14.69406
7.57786555	8.87633	8.239717	6.54932396	14.69406
13.031567	12.63068701	15.90609706	15.080315	41.86760246
0.376895	7.25248	3.33315	0.172959	2.98E-05
8.60834021	10.30501301	22.58365	14.82223505	83.9158

7.32842	7.64512	3.987279	4.11935	2.213372
0.0842473	0.083645	0.020581	0.109277	0
0	0	0	0	0
0	0	0.142206	0	0.176411
0.122982	0	0.117072	0	0.296276
0	0.217051	0	0	0
0	0.0221211	0.128981	0.0297155	0.025245
0	0	0	0	0.147691
0.155447	0	0.291773	0	0.176155
0	0	0	0	0.0364202
0.429737	1.34582	0.462175	0.84608	0.902637
0	0	0	0	0
0.0383541	0	0.0741247	0.203007	0.0452778
0.0482436	0.0488429	0.094789	0.0634731	0
0.0987436	0	0.0950668	0	0.240674
0	0	0	0	0
0	0	0.0327782	0	0
0	0	0	0.0872734	0.161547
0	0	0.0244091	0	0
0	0	0	0	0
0	0	0	0	0.147407
0	0	0	0	0
6.5186	7.09681	15.868	22.8818	0
0.179434	0	0	0	0
0.318718	0	0.776311	0	0
0	0	0	0	0
369.6420976	208.52877	324.3792155	316.170218	97.074769
0	0	0	0	0
0	0	0	0	0
0.111663	0.113019	0	0.147105	0
1.264602587	1.583161145	1.757570089	1.253763034	1.188937
0	0	0	0	0
0	0	0	0	0
0.138452	0.269915	0	0.346263	0
0	0	0	0	0
0	0	0	0	0.384963
144.0211142	110.8207088	28.10349421	30.50873277	0.958417
2.842690008	2.707586076	5.247166958	4.708984005	68.2657
9.12728559	11.79114722	13.41548596	10.76805941	90.6974
5.872237002	5.379249	2.158041644	2.210802	0.517962662
17.6203	21.7054	18.7764	13.8104	35.59317952
36.03505	40.19064	39.77624	38.46086	24.4306
99.3775	91.575864	375.69765	643.25751	692.64899
0	0	0	0.42104	0

20.88527797	25.05430179	31.16099641	23.23341	31.50062
53.4588	58.2755	57.2063	56.0405	127.5415
1656.281728	1967.083553	2643.907204	2585.19964	2675.685
1656.281728	1967.083553	2643.907204	2585.19964	2675.685
0	0	0	0	0
2.9668983	2.236852	1.269373004	0.9822009	0.159526
0	0	0	0	0
0.146055	0.587144	0	0.191688	0
0	0	0	0	0
0	0	0	0	0
0	0	0	0	0
0	0	0	0	0
0	0	0	0	0
0	0	0	0	0
0	0	0	0	0
0	0	0	0.438762	0
0.313167	0.0784564	0.344701	0.569868	0.74041
0	0.0291651	0.0285911	0	0.0345754
0	0	0	0.168759	0
66.83229876	73.31212525	57.71220474	62.12637465	22.593958
66.83229876	73.31212525	57.71220474	62.12637465	22.593958
66.83229876	73.31212525	57.71220474	62.12637465	22.593958
0.154745	0.156941	0	0	32.1071
0.619177	0.752931	0	0	112.632
0.315596	0.599329	0	0	98.3963
0.296222	0.898099	0	0	123.564
0	0	0	0	0.150468
0	0	0	0.229446	0.208998
0	0	0	0.162947	0
0	0	0	0	0
0	0	0	0.0535749	0
0	0	0	0	0
0	0	0	0	0
0	0	0	0	0
0	0	0	0	0
0	0	0	0	0
0	0	0	0	0
0	0	0.295864	0	0
0	0.017118	0.00837599	0.0113497	0
0	0	0	0	0
0	0	0	0	0
0	0	0.274163	0	0
0	0	0	0	0
0	0	0.0988953	0	0

0	0	0	0	0
0	0	0	0	0
0	0	0	0	0
30.5713	26.3972	91.5453	115.238	35.2479
0.0724328	0.217503	0.282447	0.0952919	0.256411
0	0	0	0	0
0	0	0	0	0
0.0790459	0	0.0393041	0	0
0	0	0	0	0
0	0	0.0434332	0	0.0543934
3.52987	5.45242	3.79841	3.33041	0.579189
4.21702	7.42726	15.6611	11.8596	12.3079
27.67988365	25.549519	54.16444819	44.39599458	44.1870101
27.67988365	25.549519	54.16444819	44.39599458	44.1870101
0.369217	0.180934	2.77367	2.55762	0.40654
0	0	0	0	0.221711
0	0	0	0	0
0	0	0	0	0
0.384453	4.97E-06	0.240271	0.564257	1.69E-06
0	0.317353	0.461562	0.209148	0.0944199
0.151712	0	0.151082	0	0
0	0	0.606049	0.613951	0.194488
0.325533	0.3113604	1.752586	4.25913	120.5934
4.97E-06	1.01198	0.271009	5.40E-06	0.161866
14.26546556	15.3861638	42.016846	42.15137	15.119939
12.87087384	12.53783543	19.87093785	21.560879	71.89679
0	0.0983048	0.188242	0	0.239681
5.63576	4.42377	8.96958	6.97096	35.9654
717.922	772.854	1165.423	1106.763	1084.964
0	0	0	0	0
0	0	0	0	0
0	0	0	0	0
5.06738	1.298252	20.3259	25.3822	19.3844
35.14489	32.20165	46.16048	36.432914	33.7693
35.14489	32.20165	46.16048	36.432914	89.4848
10.32970691	11.65235	15.511105	15.50260913	27.0622
117.5823153	132.3063951	430.978626	405.7534791	0.462274
117.5823153	132.3063951	430.978626	405.7534791	18.228095
117.5823153	132.3063951	430.978626	405.7534791	168.374
117.5823153	132.3063951	430.978626	405.7534791	41.84555
0.165933	0	1.155920176	2.668180214	0
0	0	0	0	0
0	0	0	0	0
0	0	0.376326	1.95E-05	0.134471

0.35178352	0.07212522	4.05157	5.24177	4.458876
176.8979	162.1577	12.32142	38.87215	1.545677645
26.705222	24.55302039	29.94902	35.93231	25.44394
24.75809278	19.7548	23.72262	19.02591	24.13240649
0.15775	0.318159	0.0386128	0	33.5378
5.18564	6.41425	6.78083	5.71721	24.645849
11.2908	12.0492	13.317	13.0194	24.645849
0.105781	0.477636	0.262529	0.278933	1.84637
4.46008	4.82209	8.34298409	11.95640101	15.70202
0	0.302359	0.297585	0.595049	0.180952
0	0	0.496683	0.22356	0
0	0.481663	1.75191	6.66503	4.30019
0	0	0	0.457716	0.257893
0.0597855	0.0977258	0.184133	1.56E-05	25.6838
0	0	0.0780299	0	3.1387
0	0	0	0	0
0.22242	0	0.430821	2.03123	1.05243
0.260281	0	1.46997	4.07367	2.11589
93.4097	95.5888	39.0495	43.5193	5.22586
59.58228	50.1502	62.52324	76.47617	80.314185
1.323428603	1.750056764	0.398598918	1.645442	1.131808
0.478381	0.477263	0.397536	0.716649	12.0973
0	0	0	0	0
0.152412	0.0774385	1.36281	2.64163	0.991764
4.44525	3.246868	5.573425063	3.241523	5.21266
4.44525	3.246868	5.573425063	3.241523	4.723132645
10.55525	7.08775	12.97343	14.22704	7.61396
27.10251	29.24257543	34.575281	32.93785869	127.6816
0.308537	0.262166	0.0510922	0.344288	0.182561
3.624752	6.8633	1.58855	2.675285	1.398137
10.14386953	11.71083114	13.50369913	13.93560001	28.92434
30.03369	29.46589	29.54543	31.121576	53.6092
21.71611233	21.50529125	26.04318074	23.77831497	4.57021
21.71611233	21.50529125	26.04318074	23.77831497	18.9742
54.03304383	52.47659909	10.95360643	20.46536404	10.36999
0.449318	0	3.6419	6.59672	2.92947
2.85501	2.69889	6.60803	7.26267	44.6783
0	0.0592573	0	0	1.26213
0	0	1.72E-06	0	7.94E-08
0	0	0	0	0
11.1676	8.90467	8.86151	11.5844	3.31386
0	0	0	0.489834	0
40.73414022	55.75486	179.6066658	167.6100466	33.58237045
0.0785725	0	8.036515	13.125428	13.967844

55.8355	36.7332	58.8614	37.0176	68.4652
60.13171599	53.665474	35.803603	47.00174096	9.189852655
0.524703	0.11782	0.515875	0.688282	0.284481
0.178412	0.0912501	0.044211	0	0.0543653
15.30426309	17.318201	28.08549	27.94263	18.72384126
2337.78921	1854.24262	498.82643	492.60121	18.4059
10.96769	12.5709	10.007278	11.49307	11.75160579
5.902289	7.460527179	9.855738825	9.378382	29.58864
30.71351	27.61833	41.4509	31.8173	24.9217
30.34984	30.569084	33.469561	36.51119	42.550374
30.34984	30.569084	33.469561	36.51119	42.550374
3.417319	4.71798	5.81139	5.93918	3.907387
0	0	0	0	0
69.71086171	75.42500828	46.67326311	46.5685914	10.74509
69.71086171	75.42500828	46.67326311	46.5685914	6.255258019
0	0	2.53E-06	0	0.581674
21.131222	22.33426207	19.20168749	18.93969364	40.91594
54.680096	55.74270683	43.8123872	53.44263	73.2497
80.647	67.5149	383.766	286.025	3.92923
41.0044	65.9371	60.7356	92.4115	96.5296
1.949762145	1.962201413	2.116030793	2.07365112	0.791094841
0.0659409	0	0	0	0
2.27E-05	3.33E-05	3.16E-06	3.37E-06	3.72E-06
612.0696466	655.5985432	932.1682929	703.9201949	1333.279
146.42002	108.13706	50.82961	67.21818492	5.760343547
146.42002	108.13706	50.82961	67.21818492	5.760343547
95.77398	71.93263	139.15748	152.7716459	507.7
38.52097	27.065473	40.89064	38.28895	19.55476874
1.11508	0.539195	1.653296	1.338146036	3.377963315
0	0.456947	0	0	0
5.2036865	3.701889162	4.037350031	4.20059	1.323531787
0	0	0	0	0
91.83627335	91.83140561	46.054578	52.01156322	19.81158181
60.78255237	53.54873097	36.50286	37.03448044	4.92867
60.78255237	53.54873097	36.50286	37.03448044	51.83641431
329.856313	313.5961074	349.4332448	333.142126	204.0338
0.212989	0.107393	0.349087	0.0468409	0
3.732926973	4.64638	1.750200743	4.10235	0
12.159027	16.355071	11.751134	7.525330041	20.65037
0.691593	0.206105	5.42E-05	0.000127468	7.72E-05
16.928411	15.107302	18.86072	18.91347	18.7609
0.0383019	0.0773179	0.15086	0.0505523	0
0	0	0	0	0
0	0	0	0	0

6.373400842	6.512871092	5.183888717	5.182552091	2.821564
11.92253	13.76381014	15.32214021	10.61242003	2.607192
11.92253	13.76381014	15.32214021	10.61242003	0.0852769
27.0947	28.22859	23.31245	25.973095	47.8076
7.30547	4.64182	4.93455	2.59717	2.10059
2.63616	1.275417306	1.014770118	1.68071	53.24835
9.230229	9.240322	6.1875607	7.498540463	2.093498
2.952133588	3.701384	4.74728588	4.89734783	25.2992
1.54023	0	2.98757	3.06188	0.92365
1.8902	2.86915	2.94637	2.79462	0.288059
1.2624274	0.67101143	0.1562532	0.20913028	63.699629
21.86948346	22.74196822	26.90459552	23.84223343	14.12548
0	0.0944418	0.45727	0.123128	0.110672
0	0.0601069	1.73376	2.91045	1.74142
0.08566371	0.028934833	2.341866	3.61428	2.12811
0.634453857	0	8.532989395	14.82888212	14.1271
33.97451116	28.63180001	11.7787363	11.82769	1.13491
29.69295782	29.42446913	29.25537	32.65294007	19.73076
6.350728512	7.376063921	10.84341319	8.537067474	38.41564344
6.350728512	7.376063921	10.84341319	8.537067474	38.41564344
20.821961	27.16908284	82.65406276	63.80021451	70.96072
0	0.0454063	5.81482	14.9821	14.6668
0	0.192775	0.0931382	0	0
0.31814404	0.0428399	0.2375695	0.1049996	0
0.806015	0.826712	0.409009	0.170523	0.249995
0	0	0	0	0
10.01931912	12.62788629	9.563903001	7.80669498	3.429604
20.62032739	19.89041822	23.47750543	24.00614716	38.16937
20.62032739	19.89041822	23.47750543	24.00614716	38.16937
106.856707	107.5575703	139.3393349	151.4821	94.036
4.322906934	4.678484623	8.406560778	8.833693425	3.687608306
0.0477805	0	0.425719	0.70239	1.28453
7.00963	3.73927	10.93286	12.95959	12.982
0	0.2573	0	0	0
0	0	0	0	0.0457792
0.631624	0.157085	0.66107	1.22612	0.188866
0.285025	0.077065	3.28748	3.00849	0.250743
0.0503848	0	1.700180411	2.379279986	3.117979
67.0858	73.7642	89.5234	76.4238	0.0019321
17.4135	17.7041	21.3408	16.1284	10.8181
11.3638	15.6124	17.2867	19.8248	40.0694
86.71814814	86.0061383	67.9558318	69.66369704	61.90603
86.71814814	86.0061383	67.9558318	69.66369704	61.90603
576.201	611.354	923.724	843.203	1210.666

8.75378	8.70102	8.39293	7.629441698	22.39422211
33.71166288	37.04902637	43.58075836	35.55773922	52.5706
33.71166288	37.04902637	43.58075836	35.55773922	21.96095153
8.178251	5.960482055	1.128068953	1.5210762	0.0933137
18.55746936	17.68551809	17.32587123	16.62928355	10.73213
18.26525147	19.24633	13.91765881	14.608717	3.58771
18.26525147	19.24633	13.91765881	14.608717	5.59737
17.01127913	17.8717402	14.94973758	20.18778967	15.6925987
17.01127913	17.8717402	14.94973758	20.18778967	15.6925987
0	0	0.400316	2.13315	4.83646
0.107494849	0.03567974	0.698134	1.2686314	0.413777
0	0	9.8114	14.62699	17.99046
0	0	0	0	0
0	0.0985489	0	0.12879	0
0	0	0	0	0.553247
0	0	0	0	0
0.0447556	0	0.0873155	0	0.583989
44.50820239	42.05153874	34.60062687	36.24711034	23.72663444
0.641388474	0.3923136	7.506475666	17.96761081	18.0923685
0.641388474	0.3923136	7.506475666	17.96761081	18.0923685
0.719821	0.289751	0	0.562089	0.17804
0	0	0	0	0.465564
0.150126	0.742137	0	0	0
1.02489	0.260968	0.499715	0	0.155763
11.07336	12.39105	20.92581	15.75428	72.1545
0.561482	0	0	0	0
17.58204	21.2623	30.49914	24.0401	7.01011
0.329099	0.480083	0.300901	0.445406371	42.52938
1.225962873	1.421098336	0.944703	0.601016577	0.165892
33.6879	28.5569	7.39257	11.86957	1.9184
16.19809	17.80049	17.19507	18.35168	26.36851
0	0.0212054	0.433813	1.54731	1.090576875
0	0	0.206485	0.554013	0.396129
0	0	0	0	0
25.36787	17.99145	28.2947	19.61984	12.8481122
0	0	0.462185	0.635358	0
44.30381378	44.88542445	49.17008034	45.28274	75.37056
6.76665	10.0044	13.8917	8.99034	7.08029
10.19482	9.88188	18.10796	16.51658	20.30373493
0.699619	1.019138	1.703059	0.893847	0.381595
4.632543605	4.035740134	0.289836138	0.505290948	0.0728977
38.147885	44.01417	78.651652	43.104922	48.6019
0	0	0	0.521608	0
0	0	0	0	0

0.706388	0	0	0	0
84.7002	89.916	17.313	22.4014	5.06604
1.071918353	1.326568483	1.876154049	3.089496208	0
1.071918353	1.326568483	1.876154049	3.089496208	0.667775
28.5068074	26.89634824	20.60245379	22.56162088	17.131151
28.5068074	26.89634824	20.60245379	22.56162088	17.131151
0	0	0	0	0
0	0	0.6117	0	0
0	0	0	0	0
0	0	0.227173	0.0616861	0
2.0575	2.16951	2.04593	2.01016	0.900058
0	0	0	0	0
0	0	0	0	0
0	0	0	0	0
0	0.0871352	0	0	0
0	0.453685	0	0	0.501647
1.42E-07	0.0876581	8.07E-08	3.02E-07	0.0961705
0	0	0	0	0
0	0	0	3.75373	0
0	0	0	0	0
0	0	0.0836309	0	0
14.0450939	13.83020561	8.184903778	7.48695017	7.28646
0.879142	0.860036	99.4295	251.015	204.434
270.7755	284.116	451.07	348.399	384.15
10.71359	14.21267	11.31154	11.29397	28.6841
0.335318	0.504164	0.15957	0.64665	0.396027
9.36398	6.27419	11.4026	9.00905	38.3397
12.60183	15.81531	11.84406	7.81033	19.3315
91.1834048	84.6768193	71.98928282	60.55996212	71.3256
0	0	0	0	0
4.306335	3.593545	7.29503	8.532898	83.0751
22.19593	22.58854	15.8024034	15.5406	3.86562
28.0451	29.3434	25.4053	26.2147	39.8811
0	0	0	0	0
0.315599717	0.471146557	0.301464949	0.50684076	0.0878663
0.259174045	0.093437414	0.128227988	0.220933202	0.0992032
0	0	0	0.102505	0
0.700758	1.33939	0.941506	1.43398	0.442249
0.0581186	0.0590147	0	0	0
0.318026	0	0	0	0
0	0	0	1.54494	0
0	0	0	0	0
0	0	0.349047	0.968454	0
0	0	0	0	0

0	0	0	0	0
0	0.212437	0	0	0
0	0	0	0	0
13.32884387	14.05682011	8.536364002	11.206698	3.04452142
29.03142	38.44859	49.98115	41.26825	87.45486
727.09643	788.32887	1249.85896	1149.68219	1051.23
0	0	0	0	0
0	0	0.438941	0.295825	0.0891428
0	0	0	0	0
21.0034	24.4443	9.57759	10.5562	16.4198
5.768786819	7.697816651	17.30904553	8.962120865	13.92912118
5.768786819	7.697816651	17.30904553	8.962120865	13.92912118
21.34587	17.6186	18.12511	16.69535	23.32911153
23.77757732	24.691221	32.58911684	36.51188491	73.0018
0	0.640334	0.301943	0	0
0.308672004	0.412668	0.254519737	0.3086957	0
0.308672004	0.412668	0.254519737	0.3086957	0.207281
8.62057	6.457518	11.985702	12.617714	13.808797
0	0	0	0	0
0	0	0	0	0
3.236193	5.310866	4.223217	3.086044	3.59379
0.316641	0.402488	0.375085	0.794916	0.42756
0	0	0	0	0
0	0	0	0.158811	0
42.46709	47.91098	42.77814	39.60124	46.31408
11.80380229	12.15780317	16.90848841	10.15029058	3.394935
3.506920059	3.421880673	0.550773731	0.830012984	2.0079094
3.142311134	4.076099679	6.709958607	6.948794054	18.759965
3.15266598	4.913760549	4.569017	4.427090825	12.58051227
0	0	0	0	0
0.67945188	0.53698547	0.50142692	0.62651615	0.184216
170.1534	159.72	181.7176	132.7434	79.951
9.498285	9.40279	11.755662	9.395915	25.2082
0	0	0	0	2.93737
33.0164	32.7452	43.7877	35.6841	64.2691
13.8094901	16.122803	9.989440341	6.87398147	11.23558994
13.8094901	16.122803	9.989440341	6.87398147	11.23558994
18.5716	15.7538	18.321	17.8801	3.52782
0.147918	0	2.84583	3.62485	0.545612
15.64585	11.345267	41.9811391	35.988874	37.00663
5.40111	4.67127	1.08669	2.4126	4.52E-05
0.529506248	0.4119576	1.407312165	1.768061	11.3976
2.09329	2.27799	3.67448	3.54353	3.14203
3.456536982	2.423121932	2.048814431	4.70661094	7.82179

3.456536982	2.423121932	2.048814431	4.70661094	0.554941
166.68757	158.721404	94.673112	91.34097	48.767296
16.89410851	15.90108	21.6985782	23.47208658	74.2882
32.4318	34.1766	47.0643	36.9059	66.5636
29.27458074	35.64340864	32.55130544	37.56032686	18.4189163
29.27458074	35.64340864	32.55130544	37.56032686	18.4189163
29.27458074	35.64340864	32.55130544	37.56032686	18.4189163
1.511159	1.131648	0.294397	0.5755417	0.16845
50.8083	47.5921	10.3752	8.83177	0.714621
16.073805	15.73621	21.141479	19.610521	30.445638
16.8940092	14.9990895	7.704514	10.0170039	0.561819
0	0	0.390578	1.67253	1.33346
10.1695	9.98239	13.072	15.8252	23.2818
44.9043	47.186	120.097	111.548	56.0557021
14.8405855	13.5726987	7.446067	7.745664103	1.064626695
0	0	0	0.04960931	1.32398
15.250031	12.136241	29.40748	36.50523	40.5938
10.39490086	11.031829	11.904732	11.93263784	23.41372001
10.39490086	11.031829	11.904732	11.93263784	23.41372001
1.57005	3.77E-05	2.96E-05	1.71E-07	8.14E-05
0.903924354	0.0570471	5.301133769	9.860922939	4.11374
111.8631	120.5361	64.2995	70.0716	286.6336
1.85E-07	0.0305792	0	0	2.71659
999.5992	540.2607	1697.748	1112.2156	10.694
0.251171811	0.152625778	4.475920244	9.99055	4.81886
0	0	0	0	0
0.618883	0.0365512	15.80723	25.35207	34.3583
2.42599	1.214858	3.68814	2.120304	0.192654
2.06196	3.12138	2.91561	3.79881	9.92195
23.135092	26.172954	24.32114	24.83255	62.66337478
0	0	0	0	0
2.04595	2.05252	2.76497	0.968239	4.16057
12.58270872	13.086317	18.230842	16.80690594	13.97023
4.561526885	4.8252617	8.29417883	9.123475388	10.53693
25.79666068	19.75898316	6.698524002	9.231933208	0.320593
0.0498422	0.100347	0.097605	0.261646	0.410606
3.13E-05	1.83555	2.27E-05	0.214002	32.9617
8.922875	8.871682	9.291095155	8.645173	2.652463536
7.35974	6.35187	10.26112	9.794586	11.6702415
0	0	3.07212973	4.60652	3.397819769
11.74924466	13.396764	13.36813081	13.42780399	24.626501
70.04174006	42.57785178	27.59933	37.27134274	37.85373
0	0	0	0	0
10.10880074	12.8226606	10.558664	10.95562024	30.90508

0	0	1.53238	1.32452	4.91E-05
0.149115	0.0301748	2.128433896	4.609543033	2.07413
0	0.0312383	9.18E-30	2.11E-17	4.07E-07
0.128117	0.0320767	2.030450727	3.661104316	1.826822533
0	0.048497369	1.180582	2.37107	1.0060032
0.174742	0	0.335407	0.454681	0
1.10616305	1.267684	0.899775403	1.632201	0
0	0	0	0	0.339412
14.24689044	15.0238963	13.15430356	16.01381373	18.31740049
0.986862	1.2118	0.478627481	0.566271	2.17352
0	0	0	0	76.24093476
50.1156	56.4417	57.4896	61.1967	76.24093476
0	0	0	0	0
0	0	0	0	0
0	0	0	0	0
0	0	0	0	0
0	0	0	0	0
0	0	0	0	0.268218
0.0835429	0	0	0	0
0	0	0	0	0
0	0.22205	0.761525	0.725483	1.19011
5.36614	8.9003	6.1433	6.54436	2.31323
5.59439	3.62765	10.3342	8.87969	3.22886
0	0	0.0513932	0	0
0.483492	0	0.788109	0	0.786567
0	0	0	0	0
0	0	0	0	0
5.45708	3.49755	6.93169	1.50102	3.68925
0	0	0	0.0762082	0
0	0	0	0.0431756	0
0	0	0	0	0
0	0	0.109959	0	0
0	0	0	0	0
0	0	0	0	0
0	0	0	0	0
0	0	0	0	0.272567
0	0	0	0	0
0	0	0	0	0
0	0.17481	0	0	0.104005
0	0	0.138341	0.187832	0.165812
0.350261	0	0	0.898461	0.393532
0	0	0	0	0
0	0	0	0	0
0.436246	0.445505	6.7456	6.06358	2.9142

0	0	0	0	0
3.63096	4.050359	5.00441	5.411517097	7.6711
13.68086994	10.43960111	13.74187836	14.49149293	7.004235302
0.093954968	0.02323247	2.641841474	3.478717525	2.181308943
0	0	0	0.0770958	0
0	0	0.15663	0	0
0	0	0	0	0
0	0.240893	0.22443	0	0
0	0	0	0	0
0	0.0782572	0.225426	0.71258	0
0	0	0	0.212158	0
0	0	0	0	0.121731
0	0	0	0	0
0.11039	0	0	0	0.258582
0	0	0	0	0
0.100519	0	0.0980299	0	0
13.59852473	14.06879	17.72007603	16.88939297	54.6215
0	0.065147182	1.77081764	1.280527898	2.09714
0	0.065147182	1.77081764	1.280527898	0.0742723
2.745156445	3.04307807	5.415706929	4.709306223	34.74767656
2.745156445	3.04307807	5.415706929	4.709306223	34.74767656
218.66442	226.812528	128.03141	75.62864	28.7869
218.66442	226.812528	128.03141	75.62864	6.663943
6.66547	4.38213	2.138854	1.7861	78.8652
377.278	309.662	137.594	188.823	9.53975
360.327	269.236	135.514	216.811	11.0198
0.465626	0.32656	0.0373069	0.0506418	67.76886
0	0	0	0	0.467411
26.8970449	35.47186814	25.89181203	32.410772	68.478048
0	0.0479879	2.26501	11.7899	353.245
0	0	0	0	0.100517
0	0	0	0	0
0	0	0	0	0
37.9456	31.7492	26.7188	29.6017	79.445
2.051972858	1.458086	5.015269005	2.670331026	0.593586
42.915794	42.829913	38.59075387	36.59561739	51.88167
2.81963	2.15632	7.57E-09	1.03E-65	573.735
37.08025	45.11941112	40.763624	49.25347	106.432
88.51196605	83.19600906	59.96342946	63.77611019	67.91938
55.9312	67.6964	78.9667	70.6561	60.6038
0.205103	0.105909	0	0.140007	0.250154
0.856169	0.0316431	0.221464	7.06E-13	56.4843
3.001644613	4.14934	2.910381593	5.862042	8.057519526
0	0	0	0	0

0	0	0	0	0
1.67E-30	2.49E-12	1.13599	0.655321	0.270857
39.39840185	44.56770263	33.49840323	33.1515036	58.61023
65.89415574	57.21731137	49.3009119	42.48983293	4.58868
0.766341168	0.825501	0.670749	0.864677539	16.148216
11.57124231	11.286427	10.44409445	7.337056531	2.634568719
26.676874	22.29330721	6.199632397	9.672875181	0.417164
0	0	0.038131	0	0
0.218915127	0.0401043	0.786939706	1.667178	0.543503
0	0	0.328251	0.219695	0.396581
8.18118	2.74693	4.44292	6.78976	8.68856
0.76213	1.68989	0	0.341613	159.876
0.000107351	7.62E-07	1.92E-06	3.08E-05	0
0	0	0.495663	0.337935	2.42318
0.325257	0	0.0640302	0	0.157516
9.268766	7.2240289	9.946003597	7.466884	19.11627
6.769910452	7.306161193	3.049429859	3.479301	2.037334656
0.186557	0.185687	0	0.469427	0.422904
0.210561	0.107522	0	0.422136	32.66459
0	0	0	0.263406	0.748693
0	0	0.780175	2.088831202	1.010191963
40.6740394	41.55110462	39.774998	39.44180648	24.67209
0	0	0	0	4.170882031
4.354605	2.902869891	2.63494	3.54309	0
32.5647567	29.87063612	27.88878494	30.39974	23.6632
10.47394387	11.47712579	11.70938211	13.06971	61.4597
147.68959	158.76721	159.22789	134.569271	155.36128
147.68959	158.76721	159.22789	134.569271	155.36128
0	0	0	0	0
0.496345935	0.372416117	0.4424424	0.3606769	1.00563
10.888929	8.968858	14.283693	8.84339696	37.64397
13.68796249	15.7669143	14.913651	14.71832272	11.858865
13.68796249	15.7669143	14.913651	14.71832272	0.0699809
13.68796249	15.7669143	14.913651	14.71832272	1.71078
34.06231884	38.26633676	43.28711388	38.82028408	80.39492654
24.02269	30.73194	28.84378	26.25985	27.56766
0.407945	8.42E-13	0	0	9.08E-05
13.20200522	14.05250769	11.54016115	10.88512023	39.15254018
7.0024884	7.90986427	10.56353792	7.0521223	1.258679995
0.725950479	0.42114	13.79532	33.0657	54.18559
34.6136	34.10310095	25.66083	25.57905	54.18559
47.8591	55.2575	68.9707	57.7148	99.8567
4.479463761	4.313111035	3.88632767	4.890474	5.91539
0	0	0	0.0632209	0.0580352

0	0	0.293949	1.3948	0.464374
0	0	0.063428	0	0.0267049
0	0	0	0.174125	0
0	0	0	0	0
0	0	0	0	0
0.253604	0.685749	0.247301	0.708847	0.174515
0	0.785192	0	0.331654	0
0	0	0	0	0
0	0	0	0	0
0.0993354	0	0.19668	0	0.242774
0	0.0701748	0.0681375	0	0.0804434
0	0	0	0	0
0.561596	0.760717	2.01639	1.72276	0.690623
0	0	0	0	0
19.43803	21.56433	15.00005	16.10283	12.732001
19.43803	21.56433	15.00005	16.10283	12.732001
0	0	0	0	0
0	0	0	0	0
0	0	0	0	0
2.503294793	2.091175289	2.356730003	1.763277154	0.673188
2.503294793	2.091175289	2.356730003	1.763277154	0
0	0	0	0	0
0	0	0	0	0
0	0	0	0	0
0.318745	0	0	0	0
0.141079	0.141917	0	0	0
0.331677	0	0.605481	0	0
57.025	52.5413	22.27719	25.95305	31.81391
1.43802	0.728168	0.654183	0.919655	0
0	0	0	0	0
0	0	0.256595	0	0
3.54228	2.533880023	4.49194	2.58821	1.35462
61.33703	56.01512	4.47139946	11.06243	1.19597
0.445149	4.74E-05	8.29E-29	2.35E-09	0.163793
61.33703	56.01512	4.47139946	11.06243	1.78525
5.256191	6.196797	8.01563	6.78503	23.78622
0.783822	1.60613	4.11942	0.443456	13.0202
56.5758	65.1229	57.4653	50.2892	138.232
129.784	154.26	56.0134	53.452	66.9253
7.53404	6.82876	10.2481	8.49909	2.26487
3.63172	4.6733	8.45579	8.75999	54.9431
4.6693095	7.055514	5.69935017	4.655371	2.68399
0.662003	0.832674	0.324617	1.222984	89.0553
85.88017817	93.94551	98.395311	82.4760184	124.6440033

85.88017817	93.94551	98.395311	82.4760184	1054.770034
0.156333774	0.538156472	0.434882119	1.331925	0.704388
3.83906257	3.707154661	3.96843801	3.580040017	27.74752
6.69802	6.67999	10.7861	10.4459	35.5576
31.186316	34.52005	35.94689	32.6527058	42.03024
0.930394	0.473539	0.0924178	0.248206	691.529
11.8855	6.48825	42.9586	75.7526	159.98
35.0610657	32.98867833	36.18033924	34.1955801	139.82329
35.0610657	32.98867833	36.18033924	34.1955801	24.7305
0.996086	1.099826036	5.13419	2.395446	5.42775
0	0	0	0	0
9.645666358	9.590527506	15.05878027	14.9255614	92.81647611
9.645666358	9.590527506	15.05878027	14.9255614	92.81647611
0.126803	2.67E-06	5.57E-06	6.05E-05	0.336545
33.33518214	30.2899294	25.09207	33.36395	116.5266015
33.33518214	30.2899294	25.09207	33.36395	116.5266015
10.6793653	10.209726	13.88635	14.14632	37.0867
462.545	517.748	769.913	691.233	835.653045
230.12408	198.39029	156.89197	149.69361	59.84723
230.12408	198.39029	156.89197	149.69361	94.2353
0	0	0	0	1.83745
11.53032924	11.87848	19.93751041	19.636867	9.121849359
11.53032924	11.87848	19.93751041	19.636867	9.121849359
0	0	0	0	0
37.39337	36.345568	28.38143801	31.1737678	8.192981674
0.492291	0.167696	0.161683	0.437768	0.190525
0.151866	0.15526	0.598765	1.63641	0.183874
0.512118033	1.107949245	2.098877883	3.167774	1.08411859
0	0	0.0503179	0.0684629	9.174291
12.83527679	14.4029291	13.64995164	12.87872775	22.23672
27.83329	25.43819	18.73945	16.85043	6.18507
0	0	0	0	0
18.86519	18.46076	19.55162	23.16525	41.66484
41.90531	42.52384	39.78116	36.46301	100.06121
16.77115	17.390832	15.904449	16.37559503	20.74980606
86.23832583	74.63456419	23.563481	28.21821679	3.989928144
33.53372322	31.55640221	53.90029904	62.15756799	70.9728551
33.53372322	31.55640221	53.90029904	62.15756799	70.9728551
86.682932	57.761841	28.073763	36.313512	0.640857
338.728	250.888	136.763	173.419	5.18295
31.8451	29.93393	33.24255	30.58962	65.1619
17.4862	17.262	19.6517	13.5828	140.0138
7.56424	12.8729	9.63936	10.1233	140.0138
0	0	0	0	0

0.0621214	0.0630412	0.185049	0.248978	0
8.566383243	8.999021	15.41862258	18.68481812	38.53099
16.6478	19.15017	15.40941	15.76753	15.18
21.94718	24.14332	25.35057	27.41051	40.3194
655.1118	759.8771	1161.3564	1185.1905	936.5
34.00011	39.0637	43.544	81.3619	31.41898
18.08398	22.944	20.14593	18.80337	20.0397
0.295931299	0	0	0.198075	16.03065
0.739839	0.446449	1.25818	1.05001	0.26601
0.148853	0.0608999	0.32581	0.358578	0.178817
6.92549	6.07197	11.8715	12.1588	3.51277
0.0952751	0	0.0474102	0.0638481	0
2.391050436	3.360216553	4.156868909	2.766871	5.7309997
0.265799	0.000821847	0.0744477	0.000183474	0.361872
0	0	0	0	0
23.00668	15.893013	2.445908	1.667252	0
23.00668	15.893013	2.445908	1.667252	0.18285
45.35231573	47.83940548	31.83078407	32.52906901	8.177381802
0.0800847	0.985685	0.000203199	0.371183	15.9617
20.46104062	20.41169	29.07942915	33.97216166	94.674857
20.46104062	20.41169	29.07942915	33.97216166	94.674857
20.46104062	20.41169	29.07942915	33.97216166	94.674857
20.46104062	20.41169	29.07942915	33.97216166	94.674857
8.18845	8.67873	14.6246	13.9475	37.6064
0.267425	0.511896	2.929877833	7.52047	3.18777
8.548160054	8.339140375	2.352618223	2.903965198	7.853506
8.548160054	8.339140375	2.352618223	2.903965198	7.853506
499.045659	444.5519416	1023.11465	1071.0705	368.51769
21.530217	18.31774175	15.10385073	16.840957	8.714961988
23.13492575	24.28476273	18.365524	18.82945802	32.85323
2.884205	1.936522	0.921032567	2.520119	2.86213
138.228529	151.9675155	620.9883437	436.727963	73.2574
10.1116	9.50656	18.6824	15.4648	56.3112
138.228529	151.9675155	620.9883437	436.727963	0.800707
138.228529	151.9675155	620.9883437	436.727963	22.34328
0.2561344	0.008084984	0.105364	0	9.24E-05
0.2561344	0.008084984	0.105364	0	9.24E-05
0	0	0	0	34.89228315
0.041121645	0.070533104	0.057423533	0.67814258	34.89228315
0.041121645	0.070533104	0.057423533	0.67814258	34.89228315
0.041121645	0.070533104	0.057423533	0.67814258	34.89228315
0.206708577	0.396434868	0.154924956	0.393611552	38.91270583
52.82906612	56.281748	37.869993	28.98121	44.68395
6.806830457	6.254733333	3.924660486	4.407615207	13.664967

10.5495	10.14128	16.5122	13.47055	53.9104
4.421812	4.569886003	5.452395051	5.29608	3.04782
4.421812	4.569886003	5.452395051	5.29608	1.31578
0.51741474	0.666308498	8.746441	16.201866	19.73856
0.51741474	0.666308498	8.746441	16.201866	19.73856
45.592806	44.7986	37.479279	37.09985	53.2795
17.96028	15.1301145	14.564327	15.04288	12.59127716
5.90896982	7.08584	9.6232	8.358091734	28.45091496
99.59080968	88.09951311	57.73250504	59.3865043	19.20310106
42.11201	38.544158	76.771858	98.60883	36.98052
0.0488187	0.141355262	0.5361232	1.440135795	0.681109
20.33583586	13.396664	4.443735881	9.0763598	0.459639
0	0	0.987403	2.18114376	1.1113374
0	0	0	0	0
104.3998	75.2713	386.38	518.0937	372.17634
0.840678	0.422049	1.93207	2.1285	1.25912
1.18445	0.60439	3.1493	2.393	5.7401
106.6708	56.42812	83.27567	90.9997	38.545853
0	0	0	0	0
0	0	0	0	0
6.39712	5.30173	2.977911	1.98562043	4.95E-05
10.90549693	11.83887569	17.64223228	13.54335547	69.54612665
3.44748	0	4.43396	14.8332	75.1927
20.0361839	22.0398247	21.2956048	22.31111884	35.5145
20.0361839	22.0398247	21.2956048	22.31111884	31.5031
61.56093681	61.554963	78.33134544	71.81839453	1.64755
61.56093681	61.554963	78.33134544	71.81839453	44.73314005
16.251263	17.28206	14.598966	17.33729	33.190572
27.02230863	24.50328965	32.38517916	33.30440764	8.85174
32.07935	33.8366	28.768555	30.76067	2.57457
0	0.203072	0.587088	0.791348	0.940783
25.327798	15.805931	12.10547	14.90899	0.696273
0	0	2.42108	2.53729	2.19633
27.150194	33.369911	38.1278091	30.75811351	34.2597
19.18870897	19.39880592	24.89842311	22.49090203	46.63140558
7.42646	7.67878	9.50568	7.62857	30.6844
91.843477	84.36821586	10.47747109	23.01532414	2.25454
0.882772	1.18587	0	1.5237	3.79145
0.359642	0	0	0	0
2.49756	2.24476	1.86801	3.0252	142.67
8.223629	8.06182	10.80332	9.1505	10.80085
0.0937003	0.280657	0.0907891	0.122825	0.112464
0	0	0	0	0
0	0	2.421847	6.68251	10.6035

56.6602684	55.77945232	70.859613	60.22923905	68.07931
3.890668418	3.363898446	5.45867	5.6159882	64.57305448
55.5391245	54.46003563	62.25973215	59.28106744	138.957
0.073294191	0.061967163	0.0239172	0.095095891	1.545172
0	0	0	0	0
0.212333846	0.107153275	1.68176671	4.958210677	2.240588
0	0	0	0	0.909515
0.0446589	0	0.442174	0.600562	0.66067458
3.212797828	1.890749	12.71945	16.80082565	16.74267
0	0	0.13276	0	0.16145
0.460466	0.117378	4.60043	7.0028	4.52086
11.7885	5.5182	2.39486	1.11633	2223.73
32.73244	28.50722	11.47133134	11.6439604	1.18868
28.55975174	31.68738	29.0872	34.66909	53.2041
9.558408143	9.183463	4.600025	5.062729272	1.82005957
78.87395	38.62296	74.02005	83.018	179.9661
78.87395	38.62296	74.02005	83.018	179.9661
34.27696	44.09293	21.220232	18.74262	34.43427
5.671238	4.99152	8.12548	5.536491264	2.03384
657.3668543	730.3496212	1084.789196	1033.848737	1326.349
0	0	0	0	0
33.67993	33.9043	6.74248343	7.3317	0.744831
0.238	0	0.218808	0	5.87643
0	0	0	0	9.76763
36.50283286	26.90536957	168.0411331	235.3650419	159.8895943
0	0	0	0	5.68307
394.777	350.292	923.131	1110.808	1169.47077
1.57E-05	0.178426	0.279257	8.17E-05	36.16
0.435658	4.07E-90	1.51E-34	2.22E-28	6.24505
0	0	0	0	0
91.8749	103.9532	90.1512	88.28580039	52.59928
0.237717426	0.159989427	0.3164906	0.322792	19.31671
5.058512913	6.126828095	8.361193426	8.03532846	41.22253
2.41317977	1.541802055	3.255835765	4.034022821	37.2223
0.50651067	0.74408673	0.577022513	0.548966708	99.312027
160.53501	190.97078	175.31488	136.35398	239.76021
12.420498	12.577805	15.37171	13.154891	28.792327
3.89421	3.80144	2.04049	2.83164	0.92266
40.33243327	42.09209048	26.8123554	27.21802712	0.888045
0	0	0	0	0
40.33243327	42.09209048	26.8123554	27.21802712	5.219871451
70.41020239	158.396322	270.659277	192.04589	47.8298
49.26832681	55.06020901	71.43246401	63.68547	8.78179
49.26832681	55.06020901	71.43246401	63.68547	50.452588

8.362205	9.656984514	20.14679	18.55477328	113.7511
8.362205	9.656984514	20.14679	18.55477328	26.508
25.9586596	27.291833	22.03178793	20.55431499	39.9765
163.5193415	145.8468698	98.39431013	101.2613961	69.7756045
163.5193415	145.8468698	98.39431013	101.2613961	69.7756045
35.489876	35.94805848	40.61315554	44.9277941	70.34607603
35.489876	35.94805848	40.61315554	44.9277941	70.34607603
28.391139	24.3634	74.96488	46.70625	168.0981
28.391139	24.3634	74.96488	46.70625	168.0981
40.54983663	57.54699442	60.27940195	65.6969328	58.58374006
40.54983663	57.54699442	60.27940195	65.6969328	58.58374006
20.01630408	17.6959837	11.5937302	13.654003	4.157221687
0	0	0	0	0
0	0	0	0	0
0	0	0	0.162473	0
0	0	0	0.0551664	0
0	0	0	0	0
0.078985	0	0	0.208255	0.0948759
0.0169629	0.0697245	0.0339839	0.0459109	0
0	0	0	0	0
0	0	0	0	0
0	0	0	0	0
0	0	0	0	0
0	0	0	0	0
0	0	0	0	0
0	0	0	0	0
0	0	0	0	0
0	0	0	0	0.081945
0.0585653	0.473807205	0.448162884	0.020757612	0.075505201
0.0585653	0.473807205	0.448162884	0.020757612	0.075505201
0.100759	0.0990182	0.239413	0	0.301249
0	0	0	0	0
0.0323757	0	0	0.0441639	0
0	0.103142	0	0.406038	0
0	0	0	0	0
0.151221	0	0.143204	0	0
1.676940576	1.405166	0.735250793	0.36652173	0
3.54895	2.92642	3.44452	3.38346	0.252847
0	0	0	0	0
0	0	0	0	0
0	0	0	0	0
0.183197	0.2959	0.144929	0.244576	0.0452973
0	0	0	0	0
0	0	0	0	0

0	0	0	0	0
0	0.0721947	0	0	0
0	0	0.0513738	0	0
0.200503	0.257848	0.535178	0.743988	0.0697242
4.619958	4.9065	3.018371	3.708233	4.37051
3.267896836	3.8769228	6.77229354	7.041360295	176.6761
0.325339043	0	1.295869276	3.716272	3.138101531
0.1310119	0.191508747	4.003294	7.515399444	3.138101531
0	0	1.572857597	3.235991486	1.394560222
114.13126	114.84967	79.60906	78.04676	15.29990106
50.17950219	59.9596034	32.74716	31.23369	52.3363
7.586039	7.678302	12.621142	11.170819	24.44088
30.8931298	38.94288965	42.1199726	38.2749297	158.3088
0.185723	0.421237	1.56222	3.64926	0.932801
9.0837	9.1315	11.13582	8.57985	22.6038
0	0	8.07E-05	1.25611	0.000136706
0	0	0	0.441609	0.775765
0.278336797	0	2.149601332	5.177103087	4.208012128
17.7923248	19.18366784	11.32073251	13.06459119	7.57418633
37.4076235	32.70070973	24.93211616	29.97856047	69.95466
3.11124	2.91532	4.01035	5.53639	0.723462
23.014587	25.47057	14.56853	14.50368	1.824465
0.726367	0.548626	1.05469	0.239114	0.214986
0	0	0	0	0
60.4054	76.74410002	34.35551604	36.4736481	98.58171971
0	0	0	0	7.6568
4.574194643	4.619653766	5.161064	5.681250312	0.977029861
4.574194643	4.619653766	5.161064	5.681250312	1.879594224
10.0403133	10.79720981	17.441582	16.70453897	58.53730794
0.200307	0	0	0	0.232003
0.854398	1.286682425	0.8249678	1.16295621	2.83E-24
15.931744	14.20427262	8.396595766	9.293368432	2.03108
102.00214	108.2903076	126.99597	114.70715	84.58866
174.310265	166.773611	201.96675	179.26849	69.83676342
174.310265	166.773611	201.96675	179.26849	69.83676342
28.82064	29.2808	21.3237	23.83876	27.6881148
28.82064	29.2808	21.3237	23.83876	27.6881148
26.4469751	33.48392618	25.34533435	27.544615	56.9321
47.06660651	48.6727898	42.12864021	43.68921886	15.83775
109.53335	104.480635	158.9649	147.4749	168.3133
27.35407445	35.377351	30.10245	24.8276189	48.72040942
40.798936	39.6454	71.5013	51.1999	129.769
77.1041	69.856	52.0434	51.3357	19.12370136
9.55975	8.50588	14.4237	9.51292	27.5892

10.36609168	9.789416354	17.94507	13.4587017	53.311035
32.62965	34.48499	35.431609	33.13259	57.7891
32.62965	34.48499	35.431609	33.13259	21.14475132
0	0	0.253743	0	0.161538
31.70219	34.85776791	31.65454	33.92707841	62.96515692
7.95437	4.9701	11.1366	9.0953	12.26569
20.7073	20.7676	25.0753	19.0355	4.852
14.86084125	12.865204	24.038648	21.85152	38.374
14.86084125	12.865204	24.038648	21.85152	22.05662
72.97809	67.88163	51.614739	50.578291	10.181546
0	0	0	0	0
7.95437	4.9701	11.1366	9.0953	12.26569
49.527588	58.17702	45.92202	45.48739691	64.3416
20.022093	22.38157	173.1815391	193.0973194	49.05376092
19.78126868	22.41946365	17.63164884	18.61280002	15.1536
1.56E-70	0	4.35931	9.36696	10.4001
1.40E-05	0.151426	1.03169	2.52963	2.25E-06
34.61147729	35.0024288	39.66412115	38.25618512	58.99250973
34.61147729	35.0024288	39.66412115	38.25618512	58.99250973
26.7183	14.55155058	25.81254	23.90685	91.27040415
52.913	51.9207	41.6346	45.8656	52.75090224
25.6863	26.0861	38.0469	32.0622	76.3826
4.062401419	3.321145329	5.675066203	5.600897	2.328711
605.98567	459.707873	209.02194	251.475791	6.16177
605.98567	459.707873	209.02194	251.475791	12.1151
605.98567	459.707873	209.02194	251.475791	9.82543
0.379745	0.160273	0.358681	4.01E-06	58.914
3.970024149	4.6715	4.39522	1.038526	2.638678
87.5904	89.7157	96.8567	94.481	21.454
13.8254	12.1649	10.4498	12.5091	40.1687
8.325660803	7.30306794	8.467521015	6.624903109	25.8642
50.0210005	48.2992243	46.61992	46.881489	17.17188
49.9445	50.9157	49.5523	48.3452	91.1516
2.52241	3.3330878	5.16670714	4.046087	16.07689
0.354684	0.59355	1.547015	4.2505	21.7111
13.0477	16.9014	16.9175	15.6871	30.1589
18.9597692	18.042592	19.810001	25.713606	36.6143
36.82585	37.4704	37.301316	38.48690549	58.34165799
0	0	0	0	166.645
5.82339	7.49552	10.5035	6.79297	28.2581
37.36286	35.34175	75.2791	53.012	148.8445
16.268753	23.74809	34.895802	41.7983529	1.821533
16.268753	23.74809	34.895802	41.7983529	11.497846
16.268753	23.74809	34.895802	41.7983529	11.497846

7.25821	6.768114	7.058577	5.834859	19.88883554
64.84920002	70.70876	42.12796241	45.727956	25.69187
18.9597692	18.042592	19.810001	25.713606	25.69187
0	0	0.27419	1.46045	1.15258
99.36783	157.77348	201.64111	214.51091	118.7602
0.209549	0.695513	0.0336851	0	82.8585
28.720729	22.440499	17.052882	22.92479	7.475600792
3.641558	3.241365	2.908935	4.350837	1.359943029
3.88442	4.459728	3.551434	2.94773	372.28624
10.47226	11.80101	12.24808	10.48986	19.70103
67.2848	63.04578	39.8006779	40.6317	12.45431493
20.463643	21.31346	31.7233	24.56209	40.7662005
6.40911	2.045576	19.5462	21.01832	20.787009
20.05638	18.234065	30.52638	41.811051	97.20775093
20.05638	18.234065	30.52638	41.811051	97.20775093
0.16876	0	0	0	0
13.87379194	14.2541537	20.52440248	17.65887544	23.63224
42.387069	40.46589	50.04264	42.32022	16.34500709
0.222943	0.112009	6.01854	10.6846	5.79682
42.387069	40.46589	50.04264	42.32022	16.34500709
33.26807271	29.44017	21.43240373	21.70269387	34.5249
0	0.186004	0	0	0
0	0.186004	0	0	20.578
0	0	0	0.199088	1.19366
4.541339231	5.571720727	5.834799634	4.869918339	36.10774
4.541339231	5.571720727	5.834799634	4.869918339	3.53275
0.41259046	0.0826182	0.823948	1.835060054	0.711762
54.311566	53.39271	24.516774	29.426588	2.964952129
19.230177	14.235743	70.98581	72.89599	56.52752
9.621088491	12.66033771	18.02451695	13.43621231	62.25878358
36.41184	28.66291	29.74338	39.06231	57.93466
0.14512889	0.0583703	1.213766121	1.646264378	1.510161027
0.543736	0.180496	0.350414	0.939757	0.496535
0.98752	0.71324	2.20856	4.99937	108.105
18.4404	19.0907	28.2635	37.4653	49.01235182
29.8697	27.6462	38.598	30.8973	49.01235182
17.69696533	20.2223434	23.46770223	23.80101692	62.22155
30.16263134	28.97144004	19.751416	22.531003	0.997272549
30.16263134	28.97144004	19.751416	22.531003	0.997272549
0.274916	0	0	0	0
0	0	0	0	0
68.216494	68.734571	62.66629	75.691074	150.1581
14.07887144	14.35845453	22.52364777	19.16048591	44.474309
71.76848822	72.2963255	103.5633791	88.54891587	36.42730337

71.76848822	72.2963255	103.5633791	88.54891587	36.42730337
71.76848822	72.2963255	103.5633791	88.54891587	193.1012
0	0.272692	0.392202	2.2874	34.25711
16.0049	17.6339	22.0284	17.9145	1.79388
29.45801085	34.01020349	30.211	26.66660729	30.57505089
0	0	0	0	0
0	0	0	0	0
14.14824179	15.53342398	26.68046876	20.20671426	16.19423942
54.311566	53.39271	24.516774	29.426588	2.964952129
0	0	0	0.300278	0.271099
0	0	0	0	0
0	1.15E-58	4.59E-08	0	0.0222891
367.479	341.386	447.017	361.333	169.652
19.52006	19.481568	26.5281	17.36858	46.691084
19.52006	19.481568	26.5281	17.36858	173.0131
48.6009	46.8195	61.2973	52.7565	92.8597
0.598337	1.20696	0	0.627149	70.1789
0	0	0	1.09311	0.971741
0	0	4.79098	0	1.71662
0.681352	0.210664	1.59729	0.830748	0.837015
3.43408	3.83296	7.40229	4.55047	5.92259
4.97051	4.12704	11.0661	10.2369	9.62399
42.387069	40.46589	50.04264	42.32022	39.3984
159.6512	167.869	125.0698	110.5448	273.0810045
12.828852	13.332139	10.702275	13.74279	17.49636
22.89802	25.43218	22.39968	20.00699	4.16729259
7.51377	9.27948	9.93174	9.33604	22.0305
1.01E-247	0	0.556436	4.36E-69	0.0666389
2.79052	3.26637	4.43879	4.062094	34.5524
6.6251253	7.42353	9.153107	11.218359	15.59055
6.6251253	7.42353	9.153107	11.218359	3.1104
0.113359	0	2.57393	3.4507	1.43198
2.88E-179	0	1.48E-36	1.7026105	3.83269
126.99662	119.48878	139.52416	151.51814	77.2217
7.97443	5.20334	4.44197	3.530836	2.748922
17.261104	13.6086	19.573459	22.78602	50.05980914
54.47169	62.47604	48.56827	46.25635	18.16119367
54.47169	62.47604	48.56827	46.25635	18.16119367
54.47169	62.47604	48.56827	46.25635	74.169011
1.59787	1.40703	1.45915	0.871148	0.845065
26.7931	36.7806	47.5373	44.7049	168.3221
98.54306	108.52187	129.41704	105.93415	152.29
40.7471	47.813	29.2278	27.2318	79.4542
60.0283	58.434	71.9432	64.9812	42.19347

3.584428	3.794605	5.734317	5.380611	37.9278
63.9508	68.6311	65.2074	75.3093	67.774
6.87976	12.2524	5.9542	1.34987	2.32899
13.2991781	10.34161	10.862611	8.714984496	1.6212504
13.2991781	10.34161	10.862611	8.714984496	1.6212504
12.707879	12.72166	9.24886	6.531579	1.01147
12.707879	12.72166	9.24886	6.531579	3.27479
12.74609407	11.98228	8.125750967	9.85032704	4.02842
12.74609407	11.98228	8.125750967	9.85032704	4.02842
75.93461884	73.67273199	51.42666395	53.04059544	46.978903
25.82756224	27.428364	26.743582	31.48827391	19.96639
0	0.132401	0.251348	0.170789	2.41231
2.03E-05	7.70E-09	2.37E-05	1.43E-05	0.0149823
72.0757567	77.25738144	70.6976117	66.561079	101.8214089
72.0757567	77.25738144	70.6976117	66.561079	1.06147
36.03429	31.464442	20.30840016	22.663576	5.47703
15.17194	13.05672	10.53117	9.99057	3.157621979
15.17194	13.05672	10.53117	9.99057	0.188726
22.94816953	20.67385703	23.810443	22.23716234	6.70571
11.936081	18.251267	26.41134	24.94891	22.6805
4.432147684	3.212331	5.454242	6.955544	9.1373
4.432147684	3.212331	5.454242	6.955544	9.1373
4.589700027	3.885013925	7.39818625	12.1433204	30.817972
0	0	0	0	0
51.3794	54.406	52.7482	53.7342	53.5974
25.4412289	24.932338	21.02179957	21.840876	13.37095
15.0394	15.6945	18.3473	14.4535	49.8976
128.06805	152.83607	141.20385	139.31986	139.2015
17.80266572	22.73542	26.09893281	25.37817876	0.0196696
17.80266572	22.73542	26.09893281	25.37817876	2.7138
17.80266572	22.73542	26.09893281	25.37817876	74.5459
53.4843111	54.25374999	33.29206253	29.13223387	12.52720023
19.19412	18.66895	27.22948812	24.01105	33.35471293
0.972041	1.31151	3.57958	2.44039	4.44922
13.66378737	14.38217522	12.33749361	13.7542699	15.2523129
5.207618139	5.047420127	7.992589662	8.422781	46.82725
0.319217	0.443923	0.308221	0.535291	0.255765
36.363965	38.1867965	35.36452674	39.17874564	45.35711418
12.4291658	12.993685	13.8388025	12.728427	19.4003
2.25628	2.29255	2.01628	3.07259	6.01966
21.80482	20.46175	20.52432	20.48499	8.737094506
42.392733	42.95388944	56.90618145	39.15142721	40.0453
33.40218416	37.09237271	47.98521927	40.09266332	170.4896
33.40218416	37.09237271	47.98521927	40.09266332	170.4896

33.40218416	37.09237271	47.98521927	40.09266332	7.727237639
4.2604895	4.573782	4.206811088	4.296709	17.2737443
12.94139	10.003038	27.03724	20.8419	13.4761
42.1558381	35.63726337	23.53040941	26.11168327	4.0064217
14.285252	17.50333657	26.257849	17.845931	25.4996329
14.285252	17.50333657	26.257849	17.845931	25.4996329
14.285252	17.50333657	26.257849	17.845931	44.6597
4.33592	6.670785188	8.744707	8.86104	21.53754
4.33592	6.670785188	8.744707	8.86104	21.53754
0.0617067	0.0314403	0.0922678	0.287587	0
0	0	0	0	0
44.05531598	42.97775552	20.56957685	22.48820549	38.2058075
20.36616301	19.58195	19.317713	19.58073562	18.93795
20.36616301	19.58195	19.317713	19.58073562	18.93795
42.58043	42.18337	40.19462	43.990226	23.21226002
35.1803	35.7958	54.2368	52.3176	173.837
33.1229	36.50319	30.12876	25.3297	40.43182
0	0	0	0	0
0	0.0688238	0.0654423	0	0
0	0	0	0	0
0	0	0	0	0
0	0	0	0	0
0	0	0	0	0.180652
7.964432	10.5379888	13.23110255	17.107792	0.688047
7.964432	10.5379888	13.23110255	17.107792	9.55505
0.224397	0.150851	0	0	0
0	0	0.177761	0	0
261.879	127.831	170.801	209.273	219.655
0.221932	0	0	0	0
0	0	0	0	0
0.107517	0	0	0	0
0	0	0	0	0
0	0	0	0	0
6.34762	4.58524	5.4171	5.61705	1.34056
0	0	0	0	0
0	0	0	0	0
0	0	0	0	0
0.0158596	0	0.0311474	0.021005	0.0383934
0	0	0	0	0
0.131426	0.177631	0.40132	0.330939	0.245527
0	0	0	0	0
0	0	0	0	0
0.812791	0.811661	1.25064	0.684011	0.317274
15.965312	17.60266	16.406173	14.16135	1.985752

16.989391	17.36794451	25.71506035	30.7397648	62.829329
16.989391	17.36794451	25.71506035	30.7397648	18.5833
0	0.0514455	0	0.133738	0
135.70797	133.8019	83.46593	81.41838	1.75972
135.70797	133.8019	83.46593	81.41838	21.01626
52.17881	57.02671	72.82485	49.6583	181.864
31.2914	39.9676	25.4456	26.1908	31.0177
19.0475	23.1308	35.4858	34.0502	16.90481
44.57447172	45.34816382	38.18417505	39.16619435	27.06782379
32.93282451	30.60409739	23.511435	27.60332	9.02601
8.27498	4.52772	6.49426	9.09985	4.49794
10.6285	12.2107	17.2205	12.0873	11.28089
43.5956	35.66	106.456	78.8715	19.0711
0.672408	0.505292	0.654289	0.659983	0.996997
0.0799355	0.161724	0	0.106352	0.194676
142.7784	135.6309138	117.8216001	108.0632	59.3549
21.045021	19.64072	21.14998442	22.08953	27.13817838
21.045021	19.64072	21.14998442	22.08953	27.13817838
9.673234733	8.4342167	16.01354395	15.79700563	6.54741
53.7963	35.6538	27.2625	31.255	5.40276
15.89404	17.541473	14.85980761	15.90306136	7.7124688
5.265484928	6.358493	4.08800111	3.623206	1.49089
80.118147	93.90597971	89.7701488	87.92738	80.86069
80.118147	93.90597971	89.7701488	87.92738	80.86069
6.27861	4.66253	11.7877	7.81998	2.68735
14.126477	16.91490071	1.304950831	3.753738	0
10.52734	9.049955	10.58218	9.29557	5.82878376
4.966720859	6.179917514	5.385495178	7.528720705	2.88887124
0	0	0	0	0
97.46300032	106.7315006	89.67780148	90.01640237	55.82127136
97.46300032	106.7315006	89.67780148	90.01640237	31.4722
1.896133457	2.052411	14.10336	17.01197	2.3828163
27.9296	25.6669	44.3344	62.9418	7.96516
0.00281479	0	0.252732	0.00314986	0.513708
2.70323	1.92019	0.890793	1.46111	1.94633
183.87114	187.504614	254.81912	202.05842	197.32228
183.87114	187.504614	254.81912	202.05842	197.32228
489.39382	350.3140397	401.71167	356.95105	21.5044
4.01317	3.795303	1.431326502	1.47431039	555.304
45.78208	36.57998	36.54017	26.25615	20.82841705
45.78208	36.57998	36.54017	26.25615	59.7370135
45.78208	36.57998	36.54017	26.25615	59.7370135
4.39319	3.37365	2.19776	2.38118	198.702
3.619502122	3.338142743	1.7693716	1.658642383	198.702

242.356	344.532	983.081	841.597	40.5619
22.32682	19.31913	14.60662	15.399568	3.261703147
22.32682	19.31913	14.60662	15.399568	3.261703147
132.4104596	126.3461626	169.2294415	159.2420261	54.36448
77.1703	73.47	120.588	78.7806	121.025
12.8102	14.5122	13.8643	13.0463	0.000683318
28.68693224	26.85925665	14.3030305	15.65380631	46.70181691
5.51508	5.33675	1.16655	2.29565	140.658
8.08E-26	0.000475207	0.0186882	8.95E-15	2.29E-05
62.96079	63.83196	43.45834	48.80593	31.78184
66.7626	67.3895	137.3217	126.8404	139.848
6.284460503	5.50558974	4.870787557	5.985550456	2.512694669
0	0	0	0	0
11.24480775	11.81535488	9.846802	9.854615987	4.713231
345.235488	315.089032	128.045327	167.79714	6.0346
5.481114686	5.288042	9.161774	3.93100812	52.97369914
1.54E-05	2.75E-05	9.80E-06	2.72E-05	0.487421
0	0	3.99112	7.88621	3.83185
7.38128	6.610304692	3.20060738	3.040134065	1.469320591
0.780386	1.993500317	13.18893807	22.65799783	15.53672
0.961722761	0.61278412	0.420694917	0.940494504	1.172402
30.01228532	28.99961	92.65770129	100.0277638	18.50229
16.632783	15.908589	24.37796	21.92292137	78.002
4.570799828	3.858867528	8.610542075	6.111694456	0.86082
40.47054	40.03116	66.16729	46.21361	204.833
11.79040735	10.67801498	9.199301	9.162923988	3.0953053
11.79040735	10.67801498	9.199301	9.162923988	3.0953053
0	0	0	0	0
0.335111	0.428624	0.248241	0	0.098135
0	1.51944	0	0	0
534.0371	524.5342	33.90742	119.3613	8.158226215
5.390939	6.169295	9.01148	10.04999	43.78633
95.89568	104.472665	126.30664	118.347668	92.8127
35.58782339	34.72280912	32.0476097	37.5243914	12.67753
0	0	0.895071	0	0
723.733	812.283	1228.22	1147.251	809.575
26.53115193	27.38557	32.84221869	29.540898	61.63178
4.32760707	4.633058802	1.3268064	2.049040442	0.9188441
5.396746064	7.110739973	6.73140489	4.188313431	7.70823
124.5438	140.265	114.9754	102.8604	42.6545
27.0651	28.1338	20.1502	20.2788	42.9046
33.55080625	30.1693227	27.76311803	27.23039428	12.33387
33.55080625	30.1693227	27.76311803	27.23039428	12.33387
0	0	5.984599	14.457626	17.7134

59.11024391	59.78343404	67.86881415	74.41253302	64.207687
59.11024391	59.78343404	67.86881415	74.41253302	64.207687
3.39495	3.08891	6.85511	5.35064	2.6952786
61.63035579	53.97805702	36.13669055	46.23783247	11.56356
0.7079387	0.2135498	5.1215913	8.323353	6.188435
9.56641	9.27596	11.1261	5.89157	5.62379
5.46869	4.90710466	9.378683599	8.55470816	36.55224
20.07194447	19.863455	23.38614089	20.36992504	21.7971
20.07194447	19.863455	23.38614089	20.36992504	55.77966502
28.92300475	29.942749	39.85034	36.360465	38.85414
28.92300475	29.942749	39.85034	36.360465	38.85414
4.299567682	4.20222	2.08044	1.729243	0
0.185112	0.12626124	3.09523	5.25894	5.76423
4.299567682	4.20222	2.08044	1.729243	1.59188
41.189807	39.41293	40.54254	39.07195	48.5734
0	0	0	0	0
0.997148997	0.808198401	2.021316729	1.8085917	1.192213881
16.81694	13.54008	2.478465	5.22713	1.405721
3.4810363	2.41049145	4.69013129	4.072907402	60.2743
302.306	307.007	354.696	287.248	91.3029
16.39152002	18.27800024	18.23409	20.10923007	0.179014
16.39152002	18.27800024	18.23409	20.10923007	38.18944911
10.33514568	10.37313049	11.8102935	12.14811021	22.106066
17.5392	16.0461	24.5572	20.704	33.9048
17.5392	16.0461	24.5572	20.704	25.1065
16.4945	17.1886	25.0543	16.6804	77.4523
6.50677	7.20307	12.53206	16.45458	13.6137
9.90607	10.88467	4.0725	5.20108	0.935102532
260.819978	250.79734	58.668039	92.1703	18.56591
0	0	0	0	0
14.2278	9.25804	15.4436	17.1533	28.7248
65.10504	66.68419	88.49811	80.80169	21.145179
8.03446	13.8553	12.5952	14.531	51.208
11.3993	20.2463	11.7512	13.6491	127.811
8.48637	8.00091	17.2536523	12.5926869	44.65821
8.48637	8.00091	17.2536523	12.5926869	44.65821
4.777892612	6.960984	4.719321513	3.720189301	1.76514
65.57052001	58.31384525	9.437885054	16.85539331	17.78395
19.29397	20.8623	30.2737	22.711	25.2427
523.992	514.436	684.477	579.82	307.011
81.6655	69.8769	142.8368	108.0646	98.9391
120.36793	107.19023	113.98814	115.157895	30.434585
9.81523755	15.25382487	14.60905035	13.48421115	6.96788
9.81523755	15.25382487	14.60905035	13.48421115	0.62864

0.9240927	1.90687631	0.921236625	0.9878181	148.9368066
27.2183	20.36380003	12.23030009	13.55490049	3.47956
27.2183	20.36380003	12.23030009	13.55490049	0.174271
27.2183	20.36380003	12.23030009	13.55490049	0
8.06539	9.82638	10.3338	10.3612	12.2505
47.708318	51.7590658	41.447455	41.118593	106.1700213
47.708318	51.7590658	41.447455	41.118593	13.1306
20.61239	20.2486	22.78407	23.74043	36.4408327
0.256927	1.02407	0	0.323778	0.58847
0	0	0	0.601406	0
0	0	0	0	0.331249
0.44955193	0.287395935	0.406427059	0.290328554	0.420723465
0.44955193	0.287395935	0.406427059	0.290328554	0.420723465
0	0	0.675366	0	0.271063
0	0	0	0	0.162394
0.0620628	0	2.81083	5.87079	7.4804
0	0	0	0	0
48.6375	61.3919	77.8078	68.8102	169.0581724
48.24600353	50.42563	33.87087	32.58756597	5.48105
4.449893	3.700043073	2.419518013	2.245182923	0.420612
38.59071954	41.18888706	39.41783115	43.3274385	56.57232256
0.413264	0.297974	1.044457	0.389683	3.28916995
0	0	0	0	0
35.36547888	37.37432008	45.88819623	39.04179279	28.59452944
20.084271	24.90406118	27.76397829	28.36840794	26.58792
2.127215	2.60634	1.714446	2.328751	3.589057
0	0	0	0	0
0.319872542	0.110764625	2.143044	2.468554084	1.636246595
22.823295	24.49762704	19.914827	20.04935468	8.154937362
22.823295	24.49762704	19.914827	20.04935468	8.154937362
26.42889	28.63168	20.3258386	23.88361	38.013547
26.42889	28.63168	20.3258386	23.88361	38.013547
49.971698	45.26755256	35.45771051	38.17564501	9.31515
568.523	644.768	346.262	289.208	83.3468
35.5648	39.593009	50.451051	55.73888	136.5907
19.7091901	18.90339429	10.90716	13.90511	3.266096852
5.134673226	4.350148608	7.012603287	8.12659445	2.265149013
105.7833	77.0974	55.60939	55.8983	14.50920099
57.63040403	51.79740453	32.57270214	32.85610301	6.76259234
1.598961582	1.913306909	2.572531974	2.71865901	15.70620411
3.33848	2.83676	10.6902	7.6623	3.30141
42.00459	35.291664	43.01623	36.75392394	40.57622
105.6820144	127.5110318	98.37401077	85.65421581	126.30101
54.29229339	59.27708459	68.2605691	62.95252671	149.661

0.372097	0.408992	0.214338	1.09E-16	2.04E-06
28.44869597	28.80149735	26.715321	28.27881915	41.08294501
1.477100197	1.845980245	6.93839137	7.373892017	0.384227526
10.59407	9.59219	67.5096	63.1389	13.199888
0	0	0	0	0
24.866656	23.26009	24.30418	26.517291	31.7504
46.1245	41.7383	49.0854	35.6424	35.556
62.6964972	65.82810053	51.852289	52.4318586	27.96459044
62.6964972	65.82810053	51.852289	52.4318586	27.96459044
120.1129165	114.0484506	71.25727197	88.48496033	63.10141173
33.28160094	31.58020065	23.22000065	28.43220077	28.57016
2.26249	0.757895	4.12524	0	1.82921
83.37840625	77.56751237	61.05340485	62.77010798	77.75190279
636.384	699.957	1051.938	960.7	1120.949
55.2114	43.9099	14.252	30.2288	2.34832
84.0433	71.6509	40.6647	46.7685	3.02031
0	0	0	0	0
31.96357	37.7776	23.71802	18.01462	1.905358903
104.6628573	87.07233618	53.73325866	56.60890908	9.188320439
104.6628573	87.07233618	53.73325866	56.60890908	9.188320439
6.29234	7.64946	7.37473	7.44958	1.77271
43.38584697	41.785947	32.067892	25.9980969	7.83528
18.71481	21.72707	39.7604	31.93741	63.39467
13.34261	13.75763	16.72104	20.33499	22.4106
203.1247988	164.6149474	70.180978	96.07874923	6.081879135
53.96384	50.29468	65.77252	70.2251	56.3779
53.96384	50.29468	65.77252	70.2251	56.3779
21.7203	23.6233	23.692	26.3945	58.7854
24.777718	25.24127268	24.87921035	27.70698563	105.84253
49.4564	46.7453	45.0319	48.0135	10.78870283
79.61693	88.6676152	82.438036	78.00232	102.0621
41.103789	42.3571941	31.410685	31.31729894	5.434460109
107.720821	95.69415008	54.92941809	64.9280055	24.97641449
66.499414	60.264733	45.17035034	52.250888	0.808496
66.499414	60.264733	45.17035034	52.250888	17.29592
66.499414	60.264733	45.17035034	52.250888	17.29592
1.40372	0.463163	3.13315	3.64058	75.9544
0.415149328	0.753678665	0.500182538	0.753206191	75.9544
5.54892	5.67486	7.95572	7.99918	0.766018
0.0974185	0	1.1908	2.22455	0.191247
11.7715	14.3602	13.3721	15.3795	21.8106
30.4466677	39.352153	35.70816	30.08463	37.29629245
30.4466677	39.352153	35.70816	30.08463	37.29629245
79.60378014	72.99813548	63.91022671	67.05084172	72.99284598

25.435323	31.435153	16.399297	14.344693	15.7515
2249.45	1683.98	278.478	421.297	23.7916
66.36427	77.43447	105.26005	84.75155	161.156
7.4204259	8.833519632	11.41740597	9.6230392	28.4685
85.00217	88.47595	100.66675	91.0601613	42.1738
85.00217	88.47595	100.66675	91.0601613	42.1738
85.00217	88.47595	100.66675	91.0601613	76.4665
226.0716	252.7409	133.9216	121.63319	29.61811396
68.562	73.3429	83.5488	71.2317	55.1508
336.543	393.954	208.2928	185.889	30.51037
85.00217	88.47595	100.66675	91.0601613	8.18E-06
85.00217	88.47595	100.66675	91.0601613	4.22359
0.0730302	0.0738234	0	0.09602	5.12751
2.36395	0.642782	3.28316	4.11997	2.72246
10.6917036	14.76480189	9.536201692	11.33860083	71.679
0	0	0	0.776816	16.659
0.00123374	0.335391	3.0237	0.00662414	2.47304
4.056073	4.416922	6.46948	6.94434	35.0372
0	0	0	0	0
6.37753	6.2575	5.12051	4.65569	3.31806
47.3672	51.0642	59.0373	56.2266	723.702
2.68371	9.23675	2.4802	2.59919	3.742650385
2.68371	9.23675	2.4802	2.59919	3.742650385
100.4451	96.19317721	116.787	125.54164	186.9662444
59.4799312	58.792588	3.4590373	5.0918493	1.99491
4.23263	4.27711	4.80863	4.16933	31.6633
2.612141	2.222438581	3.852644245	3.200260106	1.006705698
2.612141	2.222438581	3.852644245	3.200260106	1.006705698
10.49575	10.95439	11.13563	10.22356	4.17242
10.6827	13.748484	16.40814	13.75833	34.027
15.54589	19.967672	19.279346	26.20062	50.36900022
15.54589	19.967672	19.279346	26.20062	50.36900022
306.633402	290.3155	294.0593	221.094616	54.40478
12.58140567	14.25130022	19.38667693	20.29788784	5.73195
171.9288	165.8558	177.8178	165.2944	68.16481
4.52E-05	0.870053	0.133774	0.250037	0.320258
26.707783	24.133337	20.618338	21.78094062	22.027414
0	0	0	0	0
56.4136	51.3543	81.8374	96.94117	56.22076
16.319588	16.69842	16.87129	17.22115	25.59315282
22.8645	14.6062	47.3016	44.5577	24.8353
8.058401	10.18453506	3.883589734	7.346642	197.83071
0	0	0	0	0
0	0	0	0	0

0.159987	0.405494	0.436297195	0.1062	0
5.34184	8.52915	4.90909	2.96539	0.904365
19.317	17.3697	10.6575	17.0904	21.6337
25.40952	24.5216	17.71926	19.397114	6.279956471
0	0	0	0	0
3.73955	4.04089	6.73216	7.58023	2.76635
205.8251	248.22	420.14	354.878	444.784
7.05513	5.63211	6.31157	7.35094	4.05044
43.2391	52.9264	81.0461	75.0209	217.193
719.749	793.897	1250.855	1218.89	1345.54
0.256044458	0.486499	1.946412	2.60409166	2.672546
0.256044458	0.486499	1.946412	2.60409166	2.672546
9.3761491	8.16340006	4.477423061	8.06812	2.391849548
3.500211645	3.874647253	5.501580894	4.399125	16.68088
16.27725453	20.78600441	21.886509	19.61901	51.51979642
28.34389	27.3655021	20.99968003	17.66047209	13.05567796
15.934	19.5494	19.3545	10.2926	49.8768
0	0	0	0	0
0.24506	0.248669	0.209035	0.511667	0.0430581
0.250508	0.0857564	0	0.11228	0
0	0.0824607	0.237111	0.321026	0.0981014
0.0676934	0	0	0.0446498	0.041862
0	0	0.177211	0.0945173	0
42.03907882	34.77993487	17.5094204	21.2667565	0.252776
42.03907882	34.77993487	17.5094204	21.2667565	0.165208
0.644943	0	0	0	0
42.03907882	34.77993487	17.5094204	21.2667565	2.421614766
42.03907882	34.77993487	17.5094204	21.2667565	2.044748268
42.03907882	34.77993487	17.5094204	21.2667565	2.239031
42.03907882	34.77993487	17.5094204	21.2667565	2.239031
6.936595345	6.923816254	6.026557092	4.825062091	1.215080312
4.96295	4.47715	7.94874	8.3662	67.2849
10.08345002	9.677316	24.60361	25.32018505	16.50712019
54.3898	39.7487	8.35903	10.42	0.465407
28.2997	16.5106	11.8058	15.8478	0
29.3523	24.8768	3.02934	6.30599	0.950445
13.1876	7.66597	5.07888	8.32087	0.368296
4.628120234	4.591231	0.83137345	1.185130469	0.411569
0.576812143	0.527515509	2.54421771	3.530525025	2.029529263
13.52868	13.449502	15.86615433	12.71977875	28.99769
28.58285614	27.7669155	26.5101142	24.65560467	21.571734
28.58285614	27.7669155	26.5101142	24.65560467	21.571734
55.718	34.5636	62.6757	41.5031	0.60544
0	0	0	0	0

12.3848	8.02668	6.64819	11.5203	1.69499
0	0	0	0	0
28.9927845	24.08747951	19.43649143	20.71790263	10.78900106
28.9927845	24.08747951	19.43649143	20.71790263	10.78900106
0	0	0	0	0
12.32061	8.35778	20.34465	11.44311	63.6142
33.32186	35.77639	50.7259	48.75458	71.60688715
31.43442	32.93134	42.6198	35.83325	64.7866
171.3957	177.7513	168.7609	162.4427	171.6884
95.520496	90.252409	91.48787926	110.6371427	106.8341019
95.520496	90.252409	91.48787926	110.6371427	106.8341019
32.27969259	30.64167221	22.60651228	26.00384763	286.9452
1.600612	1.36712	2.284549	3.87933	67.3863
37.93852725	39.75382	56.2063	54.86086485	40.677
11.274	8.95625	4.91924	5.45837	0.401671
0	0.110474	0.611563	1.29819	0.839958
62.67669775	75.504974	73.98705859	84.26503167	47.76071107
44.8122893	49.0355555	100.1762629	98.859324	82.5371
147.178876	158.4576639	258.8289423	212.51984	531.218
29.8769	37.57407	43.06381	45.42264	45.26169629
172.55084	202.7976561	77.36212962	89.0978609	103.6005
0.276250011	0.600554	0	0.291680203	96.6116
0	0	0	0	0
0	0	0	0	0
75.39585536	76.62112044	43.19183246	48.43576345	59.92692689
75.39585536	76.62112044	43.19183246	48.43576345	3.52649
5.126471786	5.107796	14.52808	13.291722	14.67572134
23.2646	24.4188	25.325	26.497	55.4807
0	0.433882	0	0	0.515642
14.8920318	15.3208705	26.0223568	22.41272381	61.06064
14.8920318	15.3208705	26.0223568	22.41272381	37.24239
14.8920318	15.3208705	26.0223568	22.41272381	17.10816
0	0	0	0	0
275.4489443	248.0202601	38.1941129	74.6461189	5.293824
275.4489443	248.0202601	38.1941129	74.6461189	0.255017
19.08193	19.4032	40.9722	39.9003	14.1051
38.48611159	34.05391555	24.71603624	25.00545605	0
38.48611159	34.05391555	24.71603624	25.00545605	6.181943628
0	0	0	0	0
173.5731321	144.161967	84.9843501	92.06166731	14.0736616
3.524568	3.062096	6.82331	10.69902	5.379291088
59.232	71.5224	65.0621	61.3397	21.6344
2.686034	3.097703	6.67978	4.81395	8.29721
6.70469	6.070053	7.690285958	9.257	40.527106

0	0	0	0	0
3.26138	2.90948	5.1069	4.0941	53.51230572
59.1322	79.5737	90.6449	78.1802	151.9473
21.90417	25.74723	15.42722	15.418357	51.2376
0.396469	0.130097	0.49715	0	0
0	0	0.30029	0.203345	0.376288
0	0	0	0	0
1.76535	1.33366	4.41015	6.075291424	5.25385
6.786277	6.74094	10.54229363	9.005601	37.3017
6.481658	6.27471	10.213181	12.36254	3.20728
57.58605764	60.78889041	57.69808854	61.91082676	33.2907
57.58605764	60.78889041	57.69808854	61.91082676	91.427246
57.58605764	60.78889041	57.69808854	61.91082676	91.427246
47.3212	49.787605	46.008301	47.05115219	13.135
47.3212	49.787605	46.008301	47.05115219	96.71414
554.8972	628.531	914.362	882.458	681.4992452
0.314544	0.281545598	0.655290483	1.2798068	0.576791
46.63690847	58.38732735	67.96061029	59.89771454	149.4499
41.4186	38.8699	65.9852	61.9855	71.2328
26.312846	27.03665	24.12955	23.307371	10.373037
75.443308	74.481683	11.709143	21.855132	3.588885
0	0	2.89678	6.23989	3.99714
0	1.38277	0	0	0
0.109299	9.09E-05	0.387566	1.39E-05	1.14103
0	0	0	0	0
5.674045603	18.55249697	25.53164089	27.81552489	11.94373982
5.674045603	18.55249697	25.53164089	27.81552489	11.94373982
0	0	0	0	8.25088
6.812504	7.378535	9.826984	8.83533011	3.81087
6.812504	7.378535	9.826984	8.83533011	68.78055
92.89723992	91.21971635	35.97028083	45.29658261	1.08324
92.89723992	91.21971635	35.97028083	45.29658261	284.8666
74.890997	65.48532	52.00634	58.3967505	0
74.890997	65.48532	52.00634	58.3967505	17.121962
84.937455	82.72769	21.530124	35.443698	2.96516
45.2548	55.55485	42.98037	40.75585	94.0601
14.73627327	13.316293	24.73819128	43.7564541	28.9259
31.2235	34.05848	21.8026	21.896319	0.0854806
5.09063538	4.183399	5.80117	5.780380973	35.5075
2.80715	3.84788	3.27942	1.91984	1.82565
99.27491341	100.636781	15.13320588	17.43848575	2.13889
99.27491341	100.636781	15.13320588	17.43848575	3.23278
1.464120871	0.74894552	1.241773937	2.519300052	10.331347
8.122163818	9.442088859	7.002234372	6.719982148	2.47616

29.52553	29.82412	23.0113	18.04638	5.302431192
21.2637	9.48747	9.11962	13.6556	74.3414
307.2922033	314.6101157	378.34424	305.84305	134.3281372
38.4973	38.93475	33.77286	36.39208	38.87868
2.772557	3.117583	7.17694	5.976598	44.29382
8.61473539	7.28327	12.02322	12.463793	5.80372
32.3113	37.8991	19.3492	19.8141	8.2664
42.86973552	50.08846512	78.596112	70.97340149	26.23116314
42.86973552	50.08846512	78.596112	70.97340149	2.89788
42.86973552	50.08846512	78.596112	70.97340149	26.23116314
2.64453813	2.812109199	3.270694848	3.202213568	13.103865
185.382	118.342	49.6287	59.4783	4.13202
2.392781499	1.91615132	2.514459	1.711720122	0.225444
0	0	0	0	0
0	0	0	0	0
0	0	0.203832	0	0
0	0.0831641	0.0411112	0.109582	0
41.02355	47.72806	45.09992	37.15148	44.4679
13.11980442	17.10101349	14.481466	14.27338446	18.04398273
13.11980442	17.10101349	14.481466	14.27338446	31.24679945
14.40051219	16.54769736	22.07489149	22.73271	31.24679945
34.9897	33.53412	46.51947601	42.68373	55.6128
0.363878	0.238628	0.372161	0.564005	1.57E-06
62.419	53.94472	37.36395	36.40667	9.43046
23.85540111	25.97632256	19.74557418	21.57157043	36.89490805
843.4152848	773.6821226	43.62371443	183.0870245	6.390991727
0.228498	0.540727	0.225188	0.402729	0.0925242
103.097	60.2446	21.9317	22.9471	1.32058
39.12287	40.213781	43.08817884	37.68737	30.0571
22.99399	23.39673	30.22848	29.76763	6.43587
635.435	677.214	1057.149	995.852	808.388
72.248999	62.754246	45.892243	47.820136	12.25916
72.248999	62.754246	45.892243	47.820136	12.25916
72.248999	62.754246	45.892243	47.820136	12.25916
66.4018	69.2889	60.7021	61.4808	26.9637
18.249297	17.86736038	12.54063031	14.2875206	3.89408
4.802326	4.6818091	6.795563	15.21599	23.0185
27.6364	45.4493	8.636380719	12.35819	2.528289633
34.72197	38.354	29.62641	25.99801	18.81411
34.72197	38.354	29.62641	25.99801	18.81411
27.69045037	28.12451166	27.82499969	26.66135402	38.9689
49.5657743	50.29386401	39.4079549	41.78987674	53.6134
1.28121	0.526032	7.71695	15.1255	39.012
56.1617	54.35748	3.166306	19.53855	0.250836

47.10853785	46.80288509	57.32583999	44.97639247	87.02177742
0.504579	1.25194	0.135583	0.668926	102.086
93.30847604	90.8345957	74.43909752	78.97536899	103.99947
93.30847604	90.8345957	74.43909752	78.97536899	103.99947
0.331419	0.416457	0.60876458	0.641814748	11.66691
20.78285	17.774186	10.78776	13.52869	3.373544
45.43108	44.0062	87.72227	65.07042	16.8414
0	0	0.211643	0.0947581	3.316649102
12.1039	11.3286	35.5112	29.4632	30.6964
10.815	10.56064	6.538448	8.882941	17.03733437
27.9843	31.4426	22.1834	29.4827	5.47581
49.705535	49.832325	33.1983497	38.14252634	9.386065
56.68203046	55.48946479	34.20438947	38.715097	31.64746591
56.68203046	55.48946479	34.20438947	38.715097	31.64746591
56.68203046	55.48946479	34.20438947	38.715097	31.64746591
20.60567193	22.5064418	24.814246	22.80546	34.16865796
17.20426	24.42983	17.00205	15.96373	8.09305
55.12983144	50.62942982	28.37879518	33.84804031	23.635855
20.24157	19.74035	15.8247	17.61005	3.93556
36.60843018	36.68872	25.80207225	27.87309704	9.5086677
36.60843018	36.68872	25.80207225	27.87309704	1.087776
16.0571	17.3032	10.3943	7.26503	4.02E-05
54.0692	46.8832	56.0484	58.1026	47.9818
3.34883	3.13261	5.47941	7.18162	90.971
41.74568873	29.59986976	2.009236055	8.915703186	0.516051208
41.74568873	29.59986976	2.009236055	8.915703186	0.516051208
16.29545505	17.829288	15.39607042	14.351041	17.20206288
9.15796	8.50784	19.775	20.4659	40.773
6.46193	5.18921	9.39106	8.7317	20.5098
0.983686	1.8209	1.312292	1.66084384	17.0517
12.05190781	11.262235	9.60320121	11.26639335	0
12.05190781	11.262235	9.60320121	11.26639335	3.906867286
2.958175	2.385707374	2.95193938	4.298634924	2.201193717
1.13E-76	2.20E-53	1.78E-38	2.56E-78	6.71E-08
89.12208636	70.28145149	24.29155421	36.17929261	1.737220217
89.12208636	70.28145149	24.29155421	36.17929261	1.737220217
52.2405	47.7983	92.404	79.474	10.5429
0.168985	0.393355	0.107701	0.364788	0.0654873
6.127783345	3.651903362	18.483796	18.0460312	15.52707
17.57377305	15.09028666	13.15408549	14.10034472	7.69635483
0	0	0	0	0
39.63787566	33.05995469	39.44375503	32.10333964	11.78306852
0	0.827658	0	0.530268	0.488627
0	0	0	0	0

13.21666	15.04182308	11.63011999	13.26274592	4.003912635
13.21666	15.04182308	11.63011999	13.26274592	4.003912635
13.21666	15.04182308	11.63011999	13.26274592	4.003912635
6.17465	8.03718	9.07516	7.20617	24.6814
76.0434	56.5928	9.83066	10.89056	0.659432297
42.5915481	45.623205	40.03312495	41.47894806	80.72040331
43.83910535	49.49471223	43.74540838	44.82400617	80.72040331
0.992433	0.421987	2.02396	2.52917	1.26848
0.308518	0.247377	2.58544	3.45109	1.78976
0	0.0725578	0.211354	0.668962	0.691078
14.95905128	8.49847	69.95131726	96.25968998	44.57614388
0.787937	0.158901	11.7541	13.9684	11.4514
0	0	0	0	0
51.41454899	53.27792245	55.43105862	56.89904639	47.25658
51.41454899	53.27792245	55.43105862	56.89904639	47.25658
852.241	964.685	1413.165	1407.606	787.6
0.553064033	0.334837783	0.582085	0.386125	0
133.85724	134.9208	150.49757	122.0238451	125.57738
39.84728	48.9577	40.59148	33.54997	70.37582
11.04304821	11.60534789	12.22096317	10.38124426	24.170994
11.04304821	11.60534789	12.22096317	10.38124426	24.170994
41.37632	40.20487	24.50251	19.58946	4.9216891
25.3577	30.3255	27.9665	17.2715	37.6454
7.65955	8.39247	14.19335	10.5892	48.90341853
14.8699	13.3954	20.0945	17.8854	52.9918
20.4297	21.4221	31.5368	40.831	94.9853
1.148119005	1.00502	2.477129	2.816161	12.75521485
1.148119005	1.00502	2.477129	2.816161	12.75521485
62.1185	52.8301	53.2602	57.7344	50.4982
28.85941656	33.88897411	22.9475284	26.74052506	3.68743
28.85941656	33.88897411	22.9475284	26.74052506	4.233174
222.4570609	217.5215864	217.4889906	195.152576	45.76000716
0	0	0.314382	0	0
0	0	0	0	0
6.48001	6.045920003	13.56601426	20.07341	19.28490001
4.63529	5.49843	6.56255	5.53697	22.4927
0	0	0	0	3.11967
0	0	0	0	8.26074
0.0407717	0.286792	0	0	10.1582
0	0	0.0383196	0	0
0	0	0	0	0
0	0	0	0	1.3977
2.149674436	2.859591298	6.35115	3.755082781	65.121
0.108507235	0.331484546	1.939735337	3.458312498	2.684578069

9.09389	11.32816	17.45234	18.27753	77.1565
0	0	0	0	0
1.319328	0.458433881	10.37317779	20.68039	11.6237358
0	0	3.90E-05	0.367948	2.72E-05
17.83133	19.638165	19.57239	17.67231	56.57394
7.693524797	8.417925087	12.63305346	11.73378	9.414744
7.693524797	8.417925087	12.63305346	11.73378	41.04002
0	0.059901564	1.750022	2.898251129	3.200966
0.323723	0	61.1927	115.074	164.788
0.21102266	0.035397016	1.258824188	2.489607	0.832477
0.124934885	0.029795252	1.845808	3.286987	1.729444
0	0	0	0	0
0	0	0	0	0
20.22021181	21.7773131	22.52459505	23.95151062	5.100011
0.477146084	0.471026011	0.750716	0.290425	0
67.75443717	82.9230116	95.7739447	78.31982138	13.53938
0.45760496	0.332457717	0.63138862	0.2630619	36.116643
0.45760496	0.332457717	0.63138862	0.2630619	36.116643
44.5958	48.007	47.0252	59.8083	49.9381
11.66085	11.432598	13.85172473	10.94237141	5.13117
11.66085	11.432598	13.85172473	10.94237141	6.00998
45.98082522	54.64510577	12.54951266	15.39274018	0.149443
45.98082522	54.64510577	12.54951266	15.39274018	1.493892
0.128739	0.256628	0.310385	0.166343	20.205
0.268625	0.0676539	0.741146	0.539892	0.172839
1.08271	0.00157964	1.53213	0.638352	4.10318
0.0398222	0	0	0	4.49096
0	0	0.111091	0	0.506475
33.87209714	30.72463147	22.08961	23.2616039	8.57003
0	0	1.09108	2.62017	1.7626
0	0	0.212062	0	0.537101
0	0	0.527975	0.477157	0.423348
0.328141	0.331111	0	0.435347	0
25.34717635	23.25877979	17.32639457	21.1969	4.509838857
0	0	0	0	0
0	0	0	0	0
0	0	0	0	0
24.8292	17.4917	60.5034	53.7605	28.8244
14.6042	16.7292	22.1337	23.154	62.448
98.3428	96.71901	112.1082	89.81787	211.2297893
0.6046898	0.3972721	9.194966341	23.0527756	28.152232
0.6046898	0.3972721	9.194966341	23.0527756	28.152232
12.7698	11.8492	30.9085	30.7852	40.5943
12.7698	11.8492	30.9085	30.7852	1.38628

3.2491	5.39816	9.02873	7.18817	5.43224
0.0410871	0.211191	0.165556	0.0555542	0.0510302
0.39446	0.7119	0.539406	0.1025	0
4.242375	4.843971262	15.747434	15.3556674	8.741445
4.242375	4.843971262	15.747434	15.3556674	8.741445
0	0	0	0	0.451073
24.21675	24.337479	19.67408	27.97545	31.59335
0.0968652	0.0987723	0	0	0.0591606
0.071752	0	0.638412125	1.616200249	2.083206322
12.371289	13.378614	14.684121	9.73234	25.6586
45.79590388	44.32500175	61.06100341	53.24800104	54.1744
1.05839	0.380717255	1.401135079	1.4475556	0.322902
1.05839	0.380717255	1.401135079	1.4475556	10.65054357
0	0.180664	0.1766	3.54239	2.24221
0	0	0	0	0
1.426731013	1.9746362	1.0854625	0.922592839	0.385524
81.99268709	109.8467566	90.37675681	76.8804113	45.32221
46.88894557	59.59391135	58.17368986	50.90611156	61.61150559
3.43231	5.38627	6.97729	7.95136	1.86857
0	0	0	0	0.921467
0	0	0	0	0.716815
0	0	0	0	0
0	0	0	0	0
0	0.055963232	1.50414	4.80227	7.17259
0.744271	0.74241	0.709232	0.961016	0.215215
0.830348163	0.590196	0.330649	0.3748869	0
58.0157282	51.21053109	29.34762944	38.43818478	0
58.0157282	51.21053109	29.34762944	38.43818478	0.548264
0.185118	1.49447	0.180683	1.46961	0
2.00E-128	0.180171	0	0.236952	0.210016
58.0157282	51.21053109	29.34762944	38.43818478	0.393844
2.09396	0.987979	0.480157	0.433342	0
0.419471	0.949797	0.609937	1.10338	0.246311
0.544345	0.30465	0.177366	0.318979	0
0.268205	0.270573	0.390356	0.529386	0
1.71754	1.48806	1.04653	0.882193	0.151788
1.53832	1.19611	0.820694	0.440833	0
1.31929	0.96077	0.550402	0.497598	0
1.3305	0.297473	0.143216	0.582666	0.179715
1.29933	0.388233	0.742451	0.333369	0.149546
1.82548	1.3408945	0.941975	1.509704	0.399714
58.0157282	51.21053109	29.34762944	38.43818478	0.773587
58.0157282	51.21053109	29.34762944	38.43818478	0.686811
58.0157282	51.21053109	29.34762944	38.43818478	10.42917809

39.1706	8.33594	291.617	286.105	439.295
17.99579692	12.68739042	15.56855976	15.34442664	6.748133
17.99579692	12.68739042	15.56855976	15.34442664	6.748133
12.9523	13.8055	19.4198	11.757	3.10366
0	0	0.105639	0.0702614	0
0	0	0	0	0
93.74376053	88.7491949	49.68698401	46.18822624	7.849761592
12.62777987	10.49133289	18.76577203	15.16534313	6.79572
20.54167892	16.54605725	102.139285	194.9193377	34.25860245
20.54167892	16.54605725	102.139285	194.9193377	34.25860245
7.600882	10.0586	10.46961	6.02285	7.608142828
7.600882	10.0586	10.46961	6.02285	7.608142828
17.92604842	12.62601021	20.3903008	26.97683398	3.9812332
17.92604842	12.62601021	20.3903008	26.97683398	3.9812332
6.59189	3.29275	34.0133	27.969	18.5190239
0	0	0.734912166	6.474000049	1.526600365
0	0	0	0.177187	0
0	0.0985184	0.187349	0	0
0.283512	0	0	0.370656	0.688145
0	0	0	0	2.63E-05
0.0640141	0	0	0	2.63E-05
2.78363	3.47651	8.34935	8.70552	3.71148
0	0	0	0	0.186195
0	0	0	0	0.500029
0.0905738	0.305869	0.0903849	0.443662	9.58204
3.55799	0	61.9675	75.1111	155.066
0	0	1.54623	4.27843	28.75712
0.134953	0.029831	1.407742422	4.030737003	0.964291163
0.134953	0.029831	1.407742422	4.030737003	0.964291163
56.5203	59.30136	56.834	47.291	96.19113101
56.5203	59.30136	56.834	47.291	96.19113101
2.659186	2.480839	4.24992	5.373108	51.98820967
11.22316	8.92773	14.38307	14.74679	189.4866
151.39534	151.64584	184.43265	155.4327	227.228
35.427236	35.015242	26.604668	28.484305	69.770633
35.427236	35.015242	28.0362	31.9359	69.770633
46.2344	59.3695	64.7092	71.97793	130.7879
0	0.559476	0	0	0
0.454041	0.227569	0.414334	0.289856	0
4.37109	5.37185	4.82465	2.49529	0.347868
2.90591853	3.39465	3.084397994	3.831723	2.954917572
2.02878129	2.50267	6.65314	4.415122414	3.447263352
22.9705	7.69912	28.0362	31.9359	37.92330176
22.9705	7.69912	28.0362	31.9359	37.92330176

3.24532	3.74891	6.01037	7.03164	48.6684
2.42307	2.40019	2.38108	2.11366	1.00389
21.95091113	18.88048544	14.98312762	17.2749798	0.486778
0.522786	0.857985	0.384909	0.342783	0.155123
0.091114	0	0.143375	3.10E-85	0.317544
0.145217	0.0586438	0.115296	0.191915	0.140103
17.85927	17.8492537	25.30734021	29.31848777	86.09430378
108.087184	87.56701	157.73257	190.03296	443.6785079
9.82851	15.4995	28.8746	19.8787	82.04635439
7.415958009	7.542792	13.60618056	11.69052	16.698887
5.94399	5.70205	7.7106	6.569681	14.00112
0	0	0	0	0.338697
0	0	0	0	0
0.0986235	0.302075	0.486149	0.267675	0
0	0	0	0	0
0	0	0.662353	0	0
0.439888	0.176461	0.732798	0.462544	1.4663
1.29E-06	0	0	0	0
0	0	0	0	0.163839
0	0	0	0	0
0	0	0.536036	1.95105	1.59525
0	0.637945	4.00677	4.81107	8.77194
79.2924	74.06389	74.4814	80.9519	138.302
79.2924	74.06389	74.4814	80.9519	18.6253
30.3333	29.2356	29.6979	37.5237	45.26925
32.249405	38.57416641	61.63828968	66.23431966	83.23153519
0	0	0.715847	2.20458	0
0	0	0	0.235018	0
0	0.283716	0.184799	0.124639	0.229572
19.53854679	19.33611262	48.2820287	42.75162848	14.2734818
0.462293	0	0.211256	0.582721	20.5313
0.0362794	0	0	0	1.15775
0.0370972	0	0	0	1.03882
41.88598	41.11484	4.51864	10.16381	1.35361
0.164409155	0.183644417	0.242514373	0.256473381	34.3558
0	0	0	0	0
0	0	0	0	0
0.326594	1.20511	0.460519	0.311811	2.23191
22.3817	28.7514	14.47412	18.14022	31.0532
1.105135343	0.88921562	3.990677078	4.0823775	8.47143
1.105135343	0.88921562	3.990677078	4.0823775	1.897317786
273.88921	264.807808	227.4017648	248.2349085	108.092
4.2043	3.69174	7.28288	3.37752	6.3245
0	0	0	0	0

0.0374438	0.0378299	0	0.398537	0.528430322
0	0.0362734	0	0.0475335	0.0430681
0.207123	0	0	0.281211	0.993612
56.254	61.2776	39.6238	52.392	1154.16
10.5044	10.3058	26.2462	24.5385	177.047
101.4906792	79.947417	29.55003419	35.86526773	2.07320262
0.6996243	0.721146757	2.556237864	2.026737206	0.228564
4.59603	6.8499	3.90268	5.89597	251.075
0.473018	0.437058	0.231125	0.414832	0.335395
0.737403	1.1139854	0.0875999	0.467738	125.6710075
0	0	0	0	0
0.171371	0.251221392	0.781712	0.375328	0.187062
0.171371	0.251221392	0.781712	0.375328	0.187062
0	0	0	0	0
0	0	0	0	0
0.29864	0.217028	0.422238	0.398767	0.402696
0	0	0	0	0
2.251718	2.036212803	4.222043324	4.63891	1.554059655
72.5237649	60.85557904	147.69911	138.3768547	77.55253
72.5237649	60.85557904	147.69911	138.3768547	77.55253
72.5237649	60.85557904	147.69911	138.3768547	47.0241
13.90753	11.65729	15.19332	15.37997	31.05962161
0.540426	0.356056	2.14E-17	0.0624459	7.98265
0.181137	0	0.706255	0.238953	0.2158
0	0	0	0	0
48.97502343	52.301073	47.05954318	44.03167481	37.76186
337.325865	305.4998066	430.4138725	341.8370922	232.64
62.96189079	57.18340211	62.1559055	62.9368974	59.3049
0.046900963	0.046422856	0.090261191	0.4249211	5.91999
17.67448386	21.21968388	31.3618013	30.89401947	28.20488
12.50302001	8.273653387	31.641582	40.44296692	13.0111
12.50302001	8.273653387	31.641582	40.44296692	4.04107
0	0.169756	0	0	0
15.3142	12.5545	21.6122	17.8256	59.5414
0	0.115588631	0	0	4.784443963
68.81020019	56.58223939	48.89280961	58.62847958	13.812967
7.889331321	8.825299	6.723064	7.197885	7.866966
82.57405368	86.35084638	73.65862894	71.39528203	72.17154
0.521544	2.455934	0.806468	0.956273	0.757817
33.25850722	34.22561042	34.56495926	32.51572267	38.9855483
0	0	0.43948	2.08119	52.74721261
492.509028	383.0930194	1468.682052	1562.382031	969.999
0	0	0	0	0
0.0986541	0	0.22432	0.0862809	0.0799483

0	0	0	0	0
0.0521911	0.10516	0.356645	0.273314	0.364267
0	0	0	0	0
0	0	0.230007	0	0
0	0	0	0	0
0	0	0	0	0
0	0	0.534944	0.364881	0.105511
6.28073	4.03387	1.36625	0.740614	0.348015
0	0	1.09618	0	0
0.143661	0	0.196456	0.226205	0
0.189802183	1.31E-06	0.323412854	0.082292288	0
0.189802183	1.31E-06	0.323412854	0.082292288	0.303917
0	0	0	0	0
0	0	0	0	0
0	0	0	0	0.0950763
0	0.0845523	0.0824474	0.0370124	0
0	0	0	0.72254	0
4.330327247	4.60273998	6.395776459	5.397334073	7.50529
0	0	0	0	0
0	0	0	0	0
0.0924891	0	0	0.122967	0
0.234934837	0.039659327	0.228064755	0.565860058	1.0266636
0	0	0	0	0
0.0398566	0.0523948	0.0377323	0	0.0885444
4.20332	4.36073	1.45537	1.08423	0.048294
40.34280328	33.61067203	25.57954435	28.229827	5.561481212
1.74521	1.12069	2.6329	2.64062	3.21152
1.468986212	1.535726	2.068279	1.794336021	3.311338145
4.709392	5.7752789	10.78407204	11.68054928	107.1291
10.4243	11.93615	9.04996	7.07189	11.7883
0.221263057	0.933904719	1.662911708	3.072612804	13.63428402
1.97454977	1.67794	2.32669	2.244327	32.5754
7.679625638	9.75788	4.813921	7.9125	4.440794
8.019223	9.427628277	13.855126	9.666525	35.17294332
8.019223	9.427628277	13.855126	9.666525	35.17294332
14.90056	19.20988	21.00225	19.49996	731.4
66.2991	50.7296	88.0051	105.811	186.105
13.5132	13.2197	12.928	7.34	1.84437
9.309817008	8.765353	20.194169	32.700827	38.7535
9.309817008	8.765353	20.194169	32.700827	14.001034
29.03881375	27.11584669	18.68067	20.1258928	5.618098967
0	0	0	0	0
0	0	0	1.8671	1.83726
0	0	0.326349	0	3.51185

0.534684	0.178414	0	0.461552	7.81439
0	0.213192	0	0	90.46583178
0	0	0	0	2.27017
211.8861488	200.07463	397.4205031	502.8264354	514.3193
0	0	0	0	0.383952
99.06467353	84.25544162	191.4476011	264.0078959	1433.1704
2.17315228	2.427519766	1.949125687	2.251029645	123.33
63.0115	54.411	89.2733	60.4306	29.8733
10.02694	13.076719	13.74894	11.302453	86.5666
13.24472	14.95445	16.69672	11.97044	29.3555
13.24472	14.95445	16.69672	11.97044	21.19035
65.41403	67.96294	72.19427	53.52002	55.53849127
0.39543	0.404432	0	0	0
7.793	7.75259	9.62978	8.23562	32.2443
43.23453	35.691753	19.16796544	21.73399749	1.21856002
43.23453	35.691753	19.16796544	21.73399749	1.21856002
49.647063	58.379934	37.12042	31.8534159	31.61702
8.38467	8.2895	13.5171	10.56113	35.94551296
132.7653503	144.9312072	145.812512	112.7501001	138.038
237.92781	219.9268	250.1527	253.0792	117.6830076
0	0	2.71425	2.86922	4.16914
3.89697	6.00731	8.64293	4.85198	0.280277
4.472797207	5.317944461	6.668031027	5.556352225	14.40120306
0.472035	0.948166	1.30789	0	0
246.7463	246.9746	213.7174	208.8757	261.878
1.69945	1.90127	6.40617	4.52319	2.14379
84.828169	91.5146218	116.9627795	90.03328	45.3631
14.16376461	13.778125	16.10528537	14.06361112	22.51193552
0.16546	0	0.151195	1.4301	146.339
30.64690327	31.021008	27.50870704	26.76050941	43.08900581
2.55272	1.7903	3.23782	2.32695	0.00291557
4.393954	4.03168634	7.156467	6.19895	86.7673187
20.45560865	26.4524	46.43353063	41.3474	47.9024
0.231869	0.969419	0	0.316280932	116.21338
39.9304	34.1505	16.82479	20.15678	3.04745
162.2703966	214.8521124	287.9020056	227.04883	27.84737
30.54357	28.25591	26.8189	23.74785	7.3418
59.13461722	45.77541536	43.80498	42.65859	9.29172
0	0	0	0	0
4.18193	6.255976	1.237215268	2.621223	3.30115
113.230678	101.6138025	148.535856	139.086897	22.19365
37.65356	45.9989	68.96	46.7266	68.89574
2.028435	1.941583	2.89372	2.73144	123.4121
3.812217	4.227732	8.965208	9.123699	67.86983

22.55209057	23.57216822	22.83064341	27.11455421	17.331915
22.55209057	23.57216822	22.83064341	27.11455421	17.331915
22.55209057	23.57216822	22.83064341	27.11455421	17.331915
25.167	28.4602	36.2659	35.3878	68.2969
0	0.218252	0	0.565639	5.79673
11.978334	13.486573	13.396781	12.06069579	3.13664
11.978334	13.486573	13.396781	12.06069579	58.8994
1.791371396	0.754899	0.727578	0.592348458	0
100.018875	99.0852758	80.92366075	65.32913661	13.8499242
100.018875	99.0852758	80.92366075	65.32913661	13.8499242
7.441781	7.97551797	16.399012	11.63207	20.74645
0	0	1.44954	2.55403	5.08608
3.7574318	3.446331	8.269287658	10.308883	65.34764
24.71851	28.45392212	30.77299024	28.36053048	76.32162
25.81309	27.12544	29.54937	24.21262	101.9693
0.493816313	1.181788142	0.372605057	0.349077426	308.7540144
0.493816313	1.181788142	0.372605057	0.349077426	308.7540144
11.84583	13.28691	18.85273	15.83943	25.221
11.77407	19.2946	24.8677	25.061	6.54852
3.102188	4.51633	28.158751	23.693048	6.12267
195.345	257.883	224.51	174.31	23.6513
195.345	257.883	224.51	174.31	49.40789
26.66488	38.735693	29.89192	28.92772	8.99048
26.66488	38.735693	29.89192	28.92772	0
53.62489	57.52743	74.17619	82.8659	67.85255
20.390381	27.558064	9.958006	12.307363	7.68874
0	0	0	0	0
0	0	0	0	0
13.45950475	14.2258046	33.64535062	33.42671088	131.7170086
0	0	0	0	0
0	0.108627	0.104399	0.566731	0.733305
31.85763334	41.97098384	49.96065	41.82672652	70.5373
26.81819952	28.597126	31.064661	35.85570432	38.53341143
26.81819952	28.597126	31.064661	35.85570432	66.604
3.572847	4.553232548	27.301842	18.628275	2.70917
0	0	0	0	0
0.546869	0.532699	0.51797	0.461561	0.334767
14.291969	12.98544	18.6563342	27.77081143	16.30460073
4.589767055	5.80952419	6.934959409	6.12387	37.744372
3.73697	3.34767	5.50536	7.98671	87.4942
3.17044	3.30905	5.64996	4.50371	15.1368
27.82597	29.09813	25.94398	26.60935	46.7693
0.000692122	2.37E-06	5.14E-05	0.42233	0
0	0	0	0	0

0	0	0	0	0.192247
40.49112	36.45576	45.74427	33.75705	0.000114658
40.49112	36.45576	45.74427	33.75705	71.7291
30.749496	32.78016456	27.30693065	25.59146046	26.16538
2.320732528	2.85056	3.457653	3.56758	25.48444
2.320732528	2.85056	3.457653	3.56758	25.48444
73.02324117	54.60335242	162.7636503	189.2891111	50.9472
110.64316	111.20228	51.16602	57.043	35.7787
0.211676	0.242073	0.303632	0.152136	751.251
29.8914	33.7874	23.7558	21.8861	68.77093106
111.358892	107.360424	90.5162639	91.89881	108.4656
30.04077	31.77798	21.9089	23.13728	21.18202
38.95312	40.24158	52.6883	43.18788	40.94179
4.6841	2.82407	6.40506	5.77975	15.8985
28.49287	25.32958	2.9345191	5.30482	0.763489
19.1755	15.1559	28.4551	24.8713	43.31763
2.66408	1.95113	11.812	20.8111	43.31763
28.21414	34.59738	54.28459	46.73765	49.0191
30.4713	37.2387	35.9024	30.5914	105.4853
84.0367	88.2736	80.07618	85.43871	99.5201078
8.033888	7.68054	7.0421624	5.940677	28.00498
8.18827	8.68826	9.55248	9.10764	23.9123
5.88049	6.189660001	6.255067	6.1422775	3.13395
5.88049	6.189660001	6.255067	6.1422775	7.68777593
3.047237336	4.060884	3.707313773	3.616191417	43.14817
0.321188	0.573520065	0	0.058033173	62.5526
114.7408	111.1427	141.2812	121.0451	104.76
67.71866018	70.4085311	17.1948986	26.04731496	0.223009
38.96877456	37.52984949	19.16435441	17.69024371	58.471926
38.96877456	37.52984949	19.16435441	17.69024371	58.471926
43.43795	45.59889	24.883954	30.996762	63.476881
1.62245	2.04746	1.79954	2.11302	44.86308
10.68364	12.21	21.78327	22.77874	36.5035
44.796	49.27506	20.18088	23.8965	45.69005211
18.842	18.8518	29.7102	22.5636	54.1381
25.51497171	28.31989044	29.88119905	23.393056	11.7841005
25.51497171	28.31989044	29.88119905	23.393056	11.7841005
19.01378	18.85972071	28.71204617	33.10195178	23.07634904
0	0	0	0.486982	0
0.174322	0.116805	0.0284788	0.0762926	0.172384
14.53014363	13.87154344	10.06575306	12.50990441	2.41774
14.53014363	13.87154344	10.06575306	12.50990441	3.75832
23.57273	19.85374	42.4137	43.8909	34.2394
7.43264	8.40892	12.0932	9.83666	120.764

117.997	141.9951	206.5612	192.0967	565.274
66.8144	85.4167	113.685	93.9946	189.207
19.53211	21.8854	13.569607	14.97828	8.66135
19.53211	21.8854	13.569607	14.97828	8.66135
4.5777	4.08729	5.415637	6.58824	8.68701
4.44946	2.41912	4.11554	6.45302	8.23058
179.58759	191.98563	207.36358	176.79975	183.272
23.02268	25.0817	32.7542	25.105	54.7806
30.53196	31.76748	32.943756	29.33669	33.03616
42.8485	43.0763	40.1776	39.8749	3.786427
0	0	0	0	0
13.74136259	10.92013	15.06305873	12.86288	23.7026
741.877833	766.8973728	1134.669901	1117.676249	1266.970282
0	0	0	0	0
0.0785643	0.159046	0.155626	0.10424	0.192676
0.183558	0	1.02278	3.48025	3.77499
0.0497696	0	4.37713	11.6745	9.7582
0.0686154	0.139271	0.269952	0.181985	0.25273
22.20731	22.38651	26.1564	26.09497	10.2017
0	0.083982	0	0	3.35776
0	0	0	0	3.12401
0.0930631	0.307664466	5.172494	6.445917645	4.0465078
0.50286984	0.113132	1.833969	0.815654	0.378887
0	0	0	0	0
0	0	0	0	0
1.23E-05	2.28E-05	4.88E-06	0.373043	8.40E-06
1.833145	1.156620369	4.566434	2.857311453	19.72711573
0.811326371	0	2.039650314	3.434442711	2.05657
0.419535	0.56670222	0.42611399	1.782976728	9.03397
0.112297005	0.092790202	2.543815	6.89028	43.9372
0.096393362	0.0669407	2.97417	6.76442	4.81398
1.31030029	0.659484011	6.425296	12.62603	6.71111
4.01893	5.93591	7.76644	5.44439	15.36578034
6.070471161	6.876782728	8.4146533	9.421807673	63.02083395
6.070471161	6.876782728	8.4146533	9.421807673	63.02083395
29.275447	35.607852	32.30022618	30.249068	20.549124
29.275447	35.607852	32.30022618	30.249068	20.549124
29.275447	35.607852	32.30022618	30.249068	22.9201
29.275447	35.607852	32.30022618	30.249068	22.1454
64.8613	56.2557	49.9279	54.5842	40.8668
69.46222	62.50005	45.2957	51.79599	13.04964323
69.46222	62.50005	45.2957	51.79599	13.04964323
26.16357	20.21184	38.28922	41.88819	59.26492
11.7776	11.2657257	1.471199	3.558551	0.46794

20.27094	21.67933444	11.168274	11.43805428	2.584192
2.039796	0.647575131	5.21887	6.088190599	13.75798
0	0.323024	0	0	0.183218
0.704529	0	0	0	0.204316
0	0	0	0	0
0	0	0	0	0
0	0	0	0	0
0	0	0	0	0
0.22289	0.0449759	0.131225	0.117728	0.0536612
0	0	0	0	0
0	0	0	0	0
0	0	0	0	0
0.336726	0	0.0834179	0.2265	0
0	0	0	0	0
0	0	0	0	0
5.347673273	2.964534	7.839951295	10.2158057	7.216060866
32.20484	27.62276	31.2543	28.14217	61.4384
0	0	0	0	0
0	0	0	0	0
4.364507	4.062556	4.240505	4.398497	9.40771
0	0	0.321801	0.291627	0.129678
0	0	0	0	0
0.27166237	0.099160384	0.598247422	0.697002973	0.836039
0.345150263	0.09763828	1.019481722	1.01280427	0.584038216
0.345150263	0.09763828	1.019481722	1.01280427	0.584038216
0.202577	0	0.194958	0.531624	0.765285
0	0	0	0	0
0	0	0	0	0
24.82260005	29.58503	32.15835162	29.22568013	30.9397
4.83972	7.00519	9.48938	9.5841	54.4856
9.17638	12.5087	11.6926	18.2975	12.7663
27.79026534	29.2155	41.54669	34.62321	72.7276
10.8725092	10.0679495	10.943807	8.52537	16.662163
10.8725092	10.0679495	10.943807	8.52537	0.589678
10.8725092	10.0679495	10.943807	8.52537	16.662163
0.17059	0.112869	0.764278	1.65467	3.76879
9.203041159	11.90472	13.962192	12.49177795	15.1705
60.8198	33.5264	23.9533	22.8711	4.57138
10.9978	12.295	18.3819	15.0574	35.7601
1.14553	0	0	0	0
41.7463	32.6739	40.6814	26.7992	5.04163
0.162830802	0.624099	0.0540869	0.660873	0.516521
267.15348	168.243135	264.87421	248.22488	36.0257
0.791644	0.733423	1.04175	0.958204	1.10866

3.56496	2.32647	2.80194	3.26156	7.56858
0.543051	0.269285	1.39143	2.43321	3.82328
0	0	0.0343812	0	5.38443
2.746428164	3.304177141	6.546977	5.54069	7.407946691
22.2155	32.70653623	28.994827	23.97948	4.598318592
13.23118	13.13678	17.03063	13.73569	30.7126
96.8959	103.78	50.3594	52.4977	73.3627
3.577382	3.304332	6.62422	3.679738998	2.508532
3.577382	3.304332	6.62422	3.679738998	2.508532
2.57062	3.99939	5.24451	5.79701	2.75858
0	0	0	0	0.270733
1.598033001	2.799008	1.944159	2.61663	25.27981827
83.63896	77.17333188	66.15043	64.970525	19.8375
15.10077898	12.4808515	15.97369981	16.22092565	9.57301
53.0728	12.3464	35.8621	35.7285	2.07926
2.247020424	1.297138833	4.0448988	3.312976	2.832611
148.96781	245.1385	309.4485	267.7737	23.9382
10.2532	5.23274	1.48396	2.99553	8.2065
0	0	0	0	4.21386
0	0	0	0	0
0	0	0	0	0
149.0042246	143.8982262	31.19121679	40.72413339	45.52091425
122.6403	121.654	149.0839	128.8219	226.2352
28.57833844	29.8150287	25.88990705	25.12409944	21.8743
9.33897031	10.842221	15.35757	12.8173	28.1844
5.38097	3.5097	17.0336	14.4991	15.9831
8.2391	9.92497	15.9068	12.5283	17.1701
71.99185348	65.245421	16.35282961	30.65377999	3.430456
110.0652	105.8608	145.5417	107.4427	248.8905107
1.7842	1.78831	1.42754	1.60307	0.713523
0.498687	0.0504025	0.0995935	0.198228	0.058772
23.5269	22.72405	26.77416	19.35287	67.5326
212.601332	159.238191	135.23598	117.7029	9.923870004
5.66700657	4.832389	0.6541786	1.073065134	0.137573
12.43515	6.958642	9.40081	9.466383	2.580405419
84.11496762	104.1226847	128.6343693	100.6597798	189.8871
0.793858	1.36553	2.62719	1.60838	13.0739
20.13115	20.35802	20.26008826	18.92963014	31.2044
60.49235364	58.20846129	81.96497268	91.72021508	172.4045
176.9953	139.5113	46.1566	44.5144	7.62813
1069.42	760.609	450.784	576.765	13.7826
0.563265	0.285325	0.216132	0.0735932	0.266714
10.7946	9.20332	2.63571	1.46646	0
274.5099162	212.4945008	159.2887038	155.8237207	4.3138

1936.751	1345.578	650.214	807.261	12.2063
1.224319242	1.660178	1.186665269	0.990416676	0.192137
9.795216552	10.27589444	16.8670857	14.41675521	27.63166
37.6512203	39.10150565	41.38640686	45.86120011	25.35990888
1.835317357	1.83500757	1.662030193	2.034223	1.748484712
1.835317357	1.83500757	1.662030193	2.034223	1.748484712
0.021332226	0.02170313	1.15606087	2.238836657	1.09627829
2.645572885	5.722347	1.546388441	1.546474364	0
2.645572885	5.722347	1.546388441	1.546474364	0.259925
10.68695699	11.310676	7.829416104	9.903244	7.9288
12.04688885	12.09063819	18.595367	16.17944198	20.93817
5.296743874	5.10524	6.55435	6.01018	2.8502
5.296743874	5.10524	6.55435	6.01018	0.635384
26.10701132	23.914707	22.49647	21.28160102	23.9399
58.1765	55.0744	6.55525	18.21933	1.40957
52.460888	56.85305	53.37962	54.00944988	78.0413458
52.460888	56.85305	53.37962	54.00944988	78.0413458
0	0	0	0.0912647	0
14.96211212	15.33451861	11.25834851	12.93822154	23.175915
2009.819533	1962.537205	1999.6176	1561.984998	337.7743143
2009.819533	1962.537205	1999.6176	1561.984998	337.7743143
2009.819533	1962.537205	1999.6176	1561.984998	337.7743143
3.554072341	5.064	6.6043118	5.664516508	33.93497
6.05321	4.72238	6.66648	5.67058	47.1488
0.571476	0.166293	0.241344	0.545107	0.198837
81.6702	75.29188	33.70772	35.61326211	4.868630218
1.822489294	1.373787477	3.598772309	6.290389295	1.405132339
18.29890641	17.512007	13.100469	14.392052	3.269756034
26.00149925	28.35706238	29.23465146	35.93673292	5.054942173
24.22087	24.042881	27.53657	25.39013867	128.478
0.135645	0.172208	0.168554	0.090039	0.0421839
0	0	0	0	0
0.109783123	0.476753	0.308246861	0.137013694	0
7.205741	6.95038	12.73060002	5.63506	5.6215029
1.20748	0.958597	0.266311	0.722657	10.6405
0	0	0	0	0
38.465892	43.585253	49.154284	47.7851933	10.95526732
0.732411	0.244679	9.37018	15.7409	12.5176
27.243106	27.240869	22.458795	25.32932443	136.6039713
27.243106	27.240869	22.458795	25.32932443	136.6039713
27.243106	27.240869	22.458795	25.32932443	136.6039713
318.52919	304.45217	31.64541826	44.32287665	8.70245
72.81310711	61.74168511	119.1793622	117.9284331	73.954169
22.62036129	20.0485689	16.858361	16.03910382	6.378697333

22.62036129	20.0485689	16.858361	16.03910382	6.378697333
22.62036129	20.0485689	16.858361	16.03910382	1.819466158
11.194999	12.7514	13.009706	11.79917426	26.50307556
11.194999	12.7514	13.009706	11.79917426	4.08827
1.59575	1.85709	1.13614	1.62434	1.01322
0.0757141	0.0363753	0.0520157	0.0934096	45.97371
10.7712432	9.956924204	6.594385534	7.658530906	2.636531598
45.97663	41.290324	46.51462198	30.200928	39.63207162
1.10819127	0.58780244	3.697208318	8.642038418	2.258090042
1.10819127	0.58780244	3.697208318	8.642038418	2.258090042
1.10819127	0.58780244	3.697208318	8.642038418	1.238770198
1.10819127	0.58780244	3.697208318	8.642038418	1.238770198
0	0	4.14567	3.62394	3.98526
31.2731	38.1522	21.33175	23.5885	56.4378
31.8105	34.4027	40.372	35.5655	132.145
7.040863143	7.980740818	12.4331723	9.542421198	20.80830974
118.5902047	80.58126167	691.0061096	526.0462282	68.61292
26.02201	22.9815	138.9156	94.2499	20.93587
9.682238	9.91711211	45.40022	38.97009579	17.89840152
8.981204	8.707245627	27.44156773	22.60337698	32.28458
8.981204	8.707245627	27.44156773	22.60337698	32.28458
0	0	0.169282	0.231081	0.411793
61.86085	53.783196	115.0787	111.06353	27.17721016
61.86085	53.783196	115.0787	111.06353	18.97638
28.4118	20.8481	9.51916	14.3021	5.05E-05
37.4888	31.8998	20.1615	16.9439	1.51571
8.25891	7.47209	4.39567	4.52492	0
0.764296	2.82205	0.731642	0.329377	0.289924
1.623264	2.686323	1.9192992	3.880935	1.08636
32.4678	31.1776	23.12201	27.17254	7.510900002
4.62016	2.46431	2.79183	3.02E-06	1.52152
5.760255	6.155737	3.874293	5.691632137	1.828611413
54.41213029	63.8574916	27.82144	30.80904	13.80944364
0	0	0	0	0.402174
0.103247659	0.10839467	1.217681582	2.277454945	1.71422
0	0	0	0	0
4.973517258	3.115839	13.7920012	17.409961	3.835059816
14.694779	15.761826	11.308423	13.6702317	33.3319
14.694779	15.761826	11.308423	13.6702317	0.44119
9.03966	3.90382	14.8338	14.6602	34.25747
14.0346	19.6352	20.5825	16.4697	34.25747
82.51852668	80.87823	75.80961029	80.05385094	51.75786717
37.9114	36.6511	46.3162	36.9911	57.5153
19.07089421	17.501045	18.19203	21.14966808	34.1791

4.72E-05	1.73E-16	2.12E-18	3.52E-39	3.70E-11
7.847858726	6.756872353	4.20935	4.24028	0.758526
12.8026	12.2547	13.8062	13.6059	15.7544
0.402932	0	1.39427	3.73777	3.3475
15.07688	13.1894	23.92684	18.54087	61.3495
3066.205	2979.278	3057.394	3366.947	2030.763
21.6264	26.79	45.1281	35.8973	37.8267
83.11979	78.85098	71.809082	61.195236	60.45892
25.4413	23.33238	36.60264	34.74654	87.98477
0.808756	0.818858	0	0.207046	131.3757
22.188552	23.920274	29.66021	31.874834	31.16321
55.22819872	45.17189	82.1803979	79.47753037	73.143
7.134390055	5.568470001	4.700700277	5.06266	1.675180578
50.93971542	81.25272451	25.85796645	39.38922453	6.473
7.791228846	7.367928856	11.50850625	10.59096047	15.8492024
7.791228846	7.367928856	11.50850625	10.59096047	15.8492024
18.95086	23.59684	26.51382	21.21438	52.8102
91.940799	86.71179	96.788201	94.97353	94.64132006
2.354321003	1.906241071	7.556660764	4.450121167	2.430420744
2.81E-05	0.165395	6.89E-05	0.00033261	2.39121
35.45416	36.21724385	34.98789719	35.92843	28.169
1.498116767	1.215515378	1.131422852	0.99415318	0.357864
67.7417	33.6246	43.0662	47.7633	5.25353
30.13585	23.04953	11.00729	14.26896	72.6903
2.895746	3.282928	4.948308307	3.32899225	39.8061
9.01636	10.0365	18.76462	13.42383	7.94427
26.080075	23.66746	21.19985758	24.4055	16.765466
0.175077	0.746574	0.849246	1.0483	3.74505
1.936779	2.109025	2.08167841	2.77508	0.678882
4.25375	5.02031	6.43782	6.79061	3.5637
15.32049	13.65117	15.54251	11.63909	13.4139
12.9643	20.227823	5.14027	25.68906807	20.1612
21.3619	19.9097	24.58252	21.28246	7.28713
0	0	0	0	0
76.36550261	69.34023801	49.38492301	51.96337422	17.740438
76.36550261	69.34023801	49.38492301	51.96337422	17.740438
127.927546	135.3523278	116.0176502	97.7797157	25.42518
127.927546	135.3523278	116.0176502	97.7797157	25.42518
19.21869242	23.92339804	26.45406525	26.7503676	69.20185664
3.36071	2.695592091	4.696273622	6.055113205	4.028172784
21.80819	39.991	43.664	10.57476	29.1141
40.32308179	39.03797531	46.09382669	39.82435865	69.9922
40.32308179	39.03797531	46.09382669	39.82435865	27.29624
40.32308179	39.03797531	46.09382669	39.82435865	27.29624

3.820603	4.360719723	6.07766	3.788017	23.5429
0	0.147253	6.79234	9.01091	6.63987
32.851768	36.2310163	33.360136	24.257255	3.09544976
2.32417	2.890032	7.437234	2.95264	16.6222
1.65781	1.99331	2.98422	2.04473	2.00691
0	0	0	0	0
0	0	0	0	0
0	0	0.437598	0	0
4.63266	3.94435	3.8882	4.16969	0.995972
0	0	0	0	0
0	0	0	0	0
0	0	0	0	0
0.225648	0.0759256	0.443304	0.503553	0.437609
0	0	0	0	0
0.153248	0.102584	0.100098	0.134757	0
0.101182	0.101483	0.247417	0	0.120571
43.525511	42.923317	42.939356	43.611607	81.121191
43.525511	42.923317	42.939356	43.611607	81.121191
210.673555	211.11604	100.528523	93.71089	134.05142
24.43782	23.27245558	18.24514	18.09038945	5.118424244
24.43782	23.27245558	18.24514	18.09038945	5.118424244
68.83829	60.56487	7.649372	14.19858991	2.183246038
504.80078	405.09179	53.51329138	93.009753	6.73443
50.7924	33.9662	14.0384	12.972	0
38.65283954	38.05590673	30.3022787	28.34900002	91.6926
13.79880354	11.90052813	12.80018343	12.72956039	17.0192
4.19817	6.90433	6.22917	7.69445	12.0442
12.200567	13.64261	20.37779	15.95141624	92.11331072
2869.39	2084.95	363.821	308.188	10.6653
171.569	94.5454	34.4918	5.31669	1.85225
2.18673	2.06151	0.0775538	0.208698	364.473
4.073320506	4.418190766	8.9025063	11.26460128	40.983006
61.14362	70.5195	70.29652	70.07703	91.24004
25.81128	23.97568258	32.32136361	28.77285752	35.4810901
25.81128	23.97568258	32.32136361	28.77285752	35.4810901
0.0456243	0.0926296	0.179187	0.395797	2.44299
461.2946115	451.6916272	599.5975909	547.1474959	270.8567
15.0297406	16.787255	15.20053089	11.52093	58.91118985
0	0	0	0	58.91118985
21.26365923	23.230192	22.20062053	21.04606545	44.7209
21.26365923	23.230192	22.20062053	21.04606545	44.7209
43.4639	28.0432	2.49899	12.4828	2.56731
3.74778	4.75422	5.94623	3.74759	62.4122
4.12874422	3.475446	7.760903631	5.602878502	16.75764

160.9532051	156.6483517	103.34569	107.14505	84.3983
29.92620035	31.91726417	23.15746248	29.384694	22.82974162
29.92620035	31.91726417	23.15746248	29.384694	22.82974162
29.92620035	31.91726417	23.15746248	29.384694	22.82974162
663.4942906	673.9472545	586.5572201	466.5837472	157.8605544
12.786	11.6803	28.5508	25.06	15.2983
3.105719	3.712667247	2.996023239	11.170189	35.5682179
3.105719	3.712667247	2.996023239	11.170189	35.5682179
3.105719	3.712667247	2.996023239	11.170189	35.5682179
232.44	308.89883	447.436	435.535	184.3302
16.82796792	18.52382309	19.29534751	20.47164607	53.74152
7.014619298	7.139562272	7.053088509	6.516288561	13.27953
7.56373545	6.412054	7.89036	8.24715	6.565048
42.91724862	45.08422187	52.20448429	48.34266	94.53632397
0	0	0	0	0
35.19039	38.25112	2.998695	8.21722	10.0093
10.99004	11.072165	11.47806635	11.970695	27.03579
2.442365	2.546779	6.94558	6.50437	1.77351
5.75999	6.94623	5.44641	5.15159	1.01249
24.723773	24.92865	21.39502	24.64612	21.1679039
0.352993	0	10.5906	21.372	19.6854
12.02688	11.7778	10.62742	11.62055	6.618977
148.3019281	151.4696014	144.8095555	136.5201709	131.7550491
53.79623	60.3346	60.4724	52.56136	19.25395
53.79623	60.3346	60.4724	52.56136	19.25395
0	0	0	0	0
0.489074472	0.071188315	3.143514713	2.539918127	1.575185215
0.306805	0	11.7292	28.3535	45.074
63.4583	53.962909	26.219675	28.095371	1.70245
28.30758	24.21526	18.40512	16.11591	10.73927
28.30758	24.21526	18.40512	16.11591	121.952
29.02728	27.24438	25.14969	23.98458	61.6312
13.59622	20.08165	16.1543	15.82462	35.315599
41.97676	45.28812	58.579481	43.52696798	142.547
0	0.160956	0.0527131	0.494189	20.8947
1.07076	0.000240673	1.88778	4.71062	2.67008
1.551370904	0.903664	1.552424	1.175870049	2.702624528
32.3105	16.3474	11.199	14.5109	1.63312
126.5977	102.0732	40.85914	47.9133	3.94019
416.5458	299.4206	51.06628	58.32555	4.14004
25.6892	31.1675	55.2328	49.6993	20.1081
29.78986	32.10897992	44.608041	40.36762838	12.4407
63.0213052	65.1532	35.429863	44.91433686	19.74640881
63.0213052	65.1532	35.429863	44.91433686	19.74640881

112.8585	101.6888	8.305397445	17.904151	1.754330807
10.72341311	10.45299557	9.469769585	8.952211338	2.3868
54.198005	52.78075251	8.915873688	17.3055528	4.61488
53.22248	53.41076	61.92683	77.28565	56.64553
5.70995	5.4695	8.24374	6.5831	16.3104
90.96021997	86.56782652	45.162833	48.57420178	44.829566
29.81027379	34.09612526	29.2939679	33.4058059	82.1178
14.12034	13.71733	15.12462	15.73277	32.94737
0	0	1.84E-06	8.47E-06	5.00E-07
0	0	0	0.914021	0
0	0.199929	0.0964399	0.0656654	0.113503
0.243101	0.243903	0.232722	0	0.269105
11.56540926	10.92872314	7.108801753	6.644241	2.346510017
52.103	41.6726	52.2664	48.0063	20.0467
6.68791269	8.602691	7.73038	5.133980544	1.358499912
8.59676	6.36483	5.79891	4.115704	2.45348
53.98766423	54.8237448	53.76085083	53.82248608	79.44117
38.07733376	34.62372885	43.2694937	47.60586052	73.4713
0.217631	0	0.933187	1.91019	1.51808
6.91791	8.61059	4.0325382	3.734449	1.99505
15.0426	17.5464	24.5812	18.0946	30.629
6.067649	6.92516423	9.281691875	7.56349068	11.48835614
13.96195163	17.39275125	16.21699861	16.98246815	14.01602
6.767356	8.01233	7.302193026	6.032057686	2.17195
13.910124	14.18432327	16.70125291	14.725764	10.2087205
14.51434	16.25031	20.84904	19.71002	62.51879
0.801018793	0.550288007	2.293455559	3.026353	35.36907
211.0865	216.1934	83.9239	113.1799	6.8536
29.81867119	28.36131489	24.02436787	23.94488594	49.62001906
17.142895	21.33069	15.70497	14.952401	31.396554
13.20642	12.28985	17.06787	12.14728	21.35690671
4.8082834	4.907694	4.7762385	6.55842598	30.2341
0	0	0.0620333	0	0
0.225965	0	0.431124	0	1.63984002
0.399584	0	5.72E-09	3.06E-06	8.26E-08
0.257645	0.253188	0	0	0
1.239842806	1.874940221	1.473163	2.40429892	2.2954
0	0	0.385807	0	0.235606
0	0	0	0	0
0	0	0	0	0
0	0	0	0	0
0	0.144376	0	0.184517	0.168586
0.743931	0.467398	0.2542849	0.311141	66.761
5.98229	7.3325	8.90209	7.32012	49.9927

30.24653	33.33636	58.51492	46.95863	20.95344
20.53052	15.4446	16.41356	16.73731	2.55688
87.6120075	94.10796603	70.01878896	65.7611975	38.7583
87.6120075	94.10796603	70.01878896	65.7611975	95.67695409
0.284211251	0.291506	14.50764	32.1514	42.9386
2.310389254	3.102205662	3.935401219	3.571500734	4.649949
5.383675302	6.101538471	4.540017225	5.146566101	0.875514599
0.237144	0	0.585006	0	0.288852
1175.5	1306.4	512.199	537.717	8.4094
30.44286	24.309808	6.552108137	6.763024	0.432071
0.0359829	0.093732612	2.450055464	4.611951	1.924855397
0.0359829	0.093732612	2.450055464	4.611951	1.924855397
1.32898	2.15767	1.31192	1.15707	0.248119
7.50954385	9.396638234	12.902092	14.19011	39.4250021
0	0	0	0	0.615393
11.29411593	12.9693252	13.6520338	12.69293123	46.409611
7.896056053	9.365727	9.12317	10.021387	36.3024
2.079152	2.35509	1.616595	1.197466	0.224431
2.079152	2.35509	1.616595	1.197466	0.899631
0.416518	0.700412	0.068976	0.548923	53.4426
81.0109	140.3817	59.351	50.1335	5.04466
4.244793657	5.17368	4.46891359	5.81079	9.963828
11.96773	12.37169	9.91774	8.71639	51.6142
122.51081	117.27088	130.96646	88.5845	55.43886
23.7905	26.07256	23.55019	23.68761	7.029751696
0	1.62163	0	0.518474	0
23.7905	26.07256	23.55019	23.68761	7.029751696
30.76699	27.17017	78.7843	70.138	356.8761
30.349736	36.68113706	28.05893197	26.716269	30.176604
30.349736	36.68113706	28.05893197	26.716269	30.176604
13.8114	9.97766	16.035	8.83593	10.8951
1.269928	1.290888	1.775355	2.145362	26.757
35.78049662	31.66910002	26.77983702	31.28721534	36.92372336
4.171684041	4.069489145	7.826691	6.143155364	7.2511
4.171684041	4.069489145	7.826691	6.143155364	7.2511
0	0	0	0	0
45.6737484	44.27359	37.481373	35.228543	17.48708138
78.5569	20.6618	76.3929	40.5542	23.5282
18.84362001	14.51065255	12.69711314	11.68177196	3.273311
18.84362001	14.51065255	12.69711314	11.68177196	3.273311
29.81356	33.92061	39.71351	34.2376	120.117
3.5135	4.40269	4.43272	4.65537	3.49222
18.28038072	16.455652	18.6255001	18.295928	59.24426
18.28038072	16.455652	18.6255001	18.295928	0.717977

18.28038072	16.455652	18.6255001	18.295928	0.100867
3.113294778	1.375770249	6.207046	5.315160559	19.6513
7.719823	8.014558	7.96176	5.691451086	7.57316
17.95848	17.30897	20.36647	20.06421	38.1794
20.351427	19.58918799	14.90605144	15.28799256	3.181404875
7.798492434	4.86363	14.90426048	18.83383263	7.365057255
0	0.928414	2.1611	0	0.576982
80.61608025	71.41816758	58.97382055	61.36163259	16.28778
13.97944704	11.07163214	24.594534	18.268933	7.050876946
125.46732	129.31365	10.68447516	34.70326	2.304155001
224.8010067	217.2060036	161.6570065	149.9740033	35.02500138
40.124427	47.56581	42.17664	33.82154003	63.93777
18.25466	15.14997	16.28091	12.3783	90.9944
7.818569	8.969839	9.19409	6.885263079	11.185481
0.392183	0.787301	1.84056	1.01059	0
0.263961	0.174591	0.251062	0.344736	2.72188
0.525035001	0.650023	0.6883339	0.890558862	0.205434
17.4776195	17.2762093	13.75449345	14.55407858	5.572916916
0.110331	0.337326068	2.950594922	7.502591	12.66578214
13.464733	9.254881001	7.427401004	9.530272	2.061799102
10.20827071	9.488094053	8.069929175	5.411102236	5.139866473
10.20827071	9.488094053	8.069929175	5.411102236	5.139866473
0.112671	4.26497	3.70072	1.45726	5.43046
0.60974	0.914233	1.49096	1.57994	2.74106
100.5315174	113.7895452	53.88404939	50.90593627	5.33614
0	0	0	0	0
0.703363	0.709092	1.10428	2.40145	1.39457
0	0	0	0	0
0	0.209927	0	0	0
0	0	0	0	0
2.13032	2.43873	0.324221	0.811051	0.592363
0	0	0	0	0
0.0214982	0.0216165	0.0105473	0.0716253	0.0388881
0.820399	0.690678	0.470872	0.811669	20.919653
0	0	0	0	0
0	0	0	0.100085	0
0.138695	0.115009	0.209325	0.281828	0.207473
0	0	0	0	0.118464
0	0	0	0	0
0	0.0453567	0.307851	0.118699	0.157615
1.16311	0.859287	3.21332	4.33437	1.39679
0.0768343	0	0.150293	0	0.0921554
0	0.111304	0	0	0
1.40E-07	6.82E-08	0.175557	1.55E-07	0.340041

46.44323	42.91935	92.8716	97.30543	81.2803
37.2019	41.0482	41.0191	45.9026	65.10168547
57.25192	55.10588	30.9111	33.80496635	7.786720813
0	0	10.8111	23.8281	13.2453
0.333096	0	0.95096	1.28249	0
9.6662836	9.415567272	8.693401352	9.962870833	0
9.6662836	9.415567272	8.693401352	9.962870833	1.415640188
0	0	0	0.349064	0
61.32105191	59.25208526	68.05405568	74.67044555	60.3863
61.32105191	59.25208526	68.05405568	74.67044555	60.3863
0.147452	0	8.70319	22.7807	15.1223
85.94913919	74.85116	20.91342218	26.89170125	0.637451
9.669478	9.239762877	12.46559454	13.0568408	21.52831
11.05622	11.00041	14.2311	12.53065	25.5757
0.4740044	0.434823247	0.459457	0.123418633	118.37853
0	0	0.174025	0.239304	0
0.365016	0	0	0	0
0.920514363	0.8990342	2.393535016	1.748772525	0.157826
0.920514363	0.8990342	2.393535016	1.748772525	0.193525
15.36724705	18.44557226	18.0421194	14.72808538	41.458938
2.488210791	3.494341728	8.373809855	10.18727	51.05871142
2.488210791	3.494341728	8.373809855	10.18727	51.05871142
2.488210791	3.494341728	8.373809855	10.18727	51.05871142
12.128505	11.34347536	14.48161292	14.18462505	44.55758
25.12969	17.79319	17.70388	15.96563	3.232200506
0	0	0	0.68917	0
22.657768	24.016476	12.9271213	10.657581	4.95892
28.907088	28.16320474	37.163556	33.17764	22.794734
2602.6	2711.21	163.0469	461.3171238	12.165
0.617749964	0.382159656	2.348088232	2.405494996	1.0576
31.6805732	27.69190812	10.978975	15.5076917	13.81357
3.354116346	3.942533764	2.4472063	3.849355	1.852564
0	0	1.80371	0	0
429.1273	477.2358	32.21366	59.73632	8.69193
1.63326476	1.370381022	0.125078183	0.222124	0
34.17099	33.75363	27.13866	25.98103	45.196
35.27724	31.7470782	34.97317	34.20205	85.7365
54.99991561	55.06220426	51.81441371	46.73242606	70.32607371
54.99991561	55.06220426	51.81441371	46.73242606	2.957685
282.5251675	354.1730693	556.5756809	574.4098751	248.1700049
282.5251675	354.1730693	556.5756809	574.4098751	248.1700049
8.26535	6.70108	3.96564	5.66043	0.858993
7.22489	7.1816	8.62032	9.22628	89.0046
23.49512	29.83902	37.44189	31.7912	48.98923

21.1182193	20.536629	19.282564	18.87936456	5.584273592
52.69409	50.34786	42.1573	41.57215	40.81229317
12.40339	12.42867	18.17061	19.42793	30.55725
118.42508	99.655373	122.27839	110.48738	22.348092
118.42508	99.655373	122.27839	110.48738	26.59333409
30.47078315	26.865594	47.3151816	37.61737185	3.537630318
30.47078315	26.865594	47.3151816	37.61737185	3.537630318
4353.7	2713.58	1956.56	2423.84	50.2923
14.89695	11.00941	29.94272	26.6436	7.96857
0	0	0	0	0
24.410821	20.138682	14.518688	15.42329882	5.841898
7.35301	6.51495	5.57418	5.79916	2.22113
116.91469	102.989	65.16207	56.10759	6.101830698
0	0.425416	0	0.543067	0
0.111489	0	0.213111	0	0
116.91469	102.989	65.16207	56.10759	6.101830698
6.55762	6.810294	7.640008	6.557219	2.431894
4205.6803	2926.8793	1052.0382	1429.84499	18.14514
2248.21	1465.42	1003.83	1300.04	16.3238
291.752491	181.371062	173.400665	205.130674	3.23479
3034.673953	1807.381889	1006.289333	1324.465201	2.48191
12.922262	13.07430116	19.32210158	21.78030125	7.607462697
118.42508	99.655373	122.27839	110.48738	2.68075
9.65287	9.88551	14.2813	12.6871	40.9982
9.526686	14.91253754	20.87049956	16.77227597	5.34982
0.259242	0.203334	0.178459	0.319482	1.1623
1.282280039	0.933139058	13.11275302	26.56202423	31.01769177
36.7046	37.4261	79.1561	62.2273	34.418
26.8273	27.0755	31.8805	23.5193	67.0708
184.9823023	169.0004098	120.3372265	134.21447	36.76529
184.9823023	169.0004098	120.3372265	134.21447	36.76529
9.720490352	10.92251	9.472710691	11.120927	0
9.720490352	10.92251	9.472710691	11.120927	18.7723
67.010049	84.88177	15.4432	13.322045	3.221152884
67.010049	84.88177	15.4432	13.322045	3.221152884
471.610482	515.77828	772.61687	745.00627	873.47
471.610482	515.77828	772.61687	745.00627	48.64357867
6.95092	7.53763	9.43341	10.72308	14.57646
1.27E-17	1.31E-38	0.0682399	9.06E-216	0.380834934
0.732	0.634238	3.420256	7.0079	4.1541
0	0	0	0.163286	0
25.30212272	23.64149004	16.50592202	18.67852044	4.400052
10.70675	7.522003	9.41974	7.119439	423.8906
18.99658	17.57056	43.3649	34.89231	6.128170368

66.67993786	65.80943605	64.88037248	61.96334144	30.690793
246.1022	270.7678	171.5991	168.6706	230.3676
33.00672	28.16774	18.07258	18.5382	6.93506
82.32285315	96.85868342	93.90060328	83.48324614	286.7172641
82.32285315	96.85868342	93.90060328	83.48324614	286.7172641
20.40700646	18.230728	13.197428	12.50930434	4.412261978
43.4466553	42.27966842	7.56779724	12.48461	2.409863343
84.4444	84.61252	135.88676	91.52648	96.28513571
84.4444	84.61252	135.88676	91.52648	75.28285613
84.4444	84.61252	135.88676	91.52648	75.28285613
0	0	0.380816	1.698213	0.246057
11.2555	11.2386	11.8448	6.75931	3.89304
8.01471	9.93077	10.44163	7.53587	9.00851
3.488211	3.889744	5.83250936	5.047723	11.4369
49.5598	16.2882	63.2763	32.265	254.098
933.8866	1048.8391	1460.177	1338.103	1407.38
21.75597342	18.305829	36.79242514	31.6443376	46.3852
21.75597342	18.305829	36.79242514	31.6443376	46.3852
22.4651	22.8829	18.0869	19.9968	24.2721
79.94207001	75.07410813	53.08205552	56.50637086	5.946067
0	0	0	0	0
0	0	0	0	0
30.09253	39.42799	34.4515	36.31732	56.5795
0.49906	0.334876	1.3573	1.52736	0.965314
0	0	0	0	0
2.53898	0	9.97033	23.2262	7.13433
5.98096	4.12233	7.91933	6.96035	25.01656
0.453760233	0	1.068714	3.423651	1.790430275
0	0	0	0	0
46.42555	51.8463	53.12731546	38.23244307	4.696399
0	0	0	0	0
0.0426467	0	0	0	0.0503699
0	0	0.881922	0.29936	0
0	0.775939	0.983738	0.666122	0.622451
0	0	0	0	0.426047
0.345733	0.174878	0.93366	0.801356	22.8937
0	0.238289	0.11446	0.31133	24.7938
0	0	0	0	0.470016
0	0	0	0	0
0	0	0	0	0
8.27177	0	1.83225	0	0
0.408384	0	0.504086	0.267166	0.131544
0	0	0	0	0
0	0	0.607582	0.855049	0

0	0	0	0	0
0	0	0	0	0
0	0	0	0	0
0	0	0	0	0.197225
0	0	0	0	0
0	0	1.33253	0	0
0	0	0	0	0
0	0	0.93494	0.856213	0
195.2352	183.2499	200.1974	186.7738	69.1076
34.46812	35.355266	38.33029	43.86875	61.96951
4.19319	4.40067	5.68242	5.13798	15.89242582
9.992031039	11.891127	16.19110353	15.6934327	31.50107
181.86304	169.1963	129.0507774	123.88077	43.521622
0	0	0	0	0
33.2217889	34.0504593	28.4190217	33.4335965	13.46446
33.2217889	34.0504593	28.4190217	33.4335965	13.46446
0	0	0	0	0
18.07178122	19.126984	18.633391	19.26414	68.805786
32.06736423	31.87184658	30.71997404	34.06944599	26.36598225
126.687	55.7832	69.1832	70.3472	9.79791
19.0298	19.61516	9.85213	7.976899	0.375589
0	0	0	0	0
7.25794	6.93424	5.36123	6.47584	1.744072036
7.25794	6.93424	5.36123	6.47584	1.744072036
0	0	0	0	0
0	0	0	0	0
10.482074	11.65167	8.839877	12.1724	2.589906
10.482074	11.65167	8.839877	12.1724	2.589906
0	0	0	0	320.936
0	0	0	0	38.6904
0.78278	0	0	0.348358	17.2629
57.321049	54.808203	18.3852682	24.0297564	3.82214
72.3196	79.2782	81.4463	96.8069	26.0408
59.3652	55.9958	51.9458	52.5953	10.1608
3.68312	3.92728	2.36947	2.72177	0.770668
0.631137	0.424946	1.23136	0.276503	0
0	0	0	0	0
0.366648	0.904436	0.676823	5.38369	5.22607
141.500995	167.170033	102.114774	95.888226	263.2019
1.02319	0.236497	1.44836	0.000685094	2.16655
1.934985	1.82953	9.67678	7.85659	0.275209
26.3613	21.9262	15.7205	20.6116	1.13331
7.1898027	7.287122596	6.326258056	6.846105	2.494408555
708.733852	777.1967006	1084.845533	1055.273755	994.123

15.938446	16.7233473	15.62253021	16.72596	40.28235
27.7983	26.0845	29.2582	24.1972	50.6943
54.4498476	58.55717963	39.26973733	43.99939	46.02354305
15.2760032	15.171048	22.945381	26.089699	7.22272
40.150339	44.05912	21.15374226	22.49897	43.4506
8.021378166	6.753516	7.552714293	11.154107	6.72482
0	0.700538	0.656328	0	0
38.0101	34.5225	21.0574	21.9261	57.70910333
151.0753	177.5205	156.929	93.98404	102.367
219.4761335	187.7662595	116.2114772	115.3960933	3.058393071
219.4761335	187.7662595	116.2114772	115.3960933	5.937862
1.722962	0.349814	4.307123241	3.806171972	0.424733
19.97588689	19.987405	25.41687147	21.30791606	44.79063383
2.4985252	2.733770903	2.893053241	2.039223603	1.18886029
1.368489193	0.685712283	1.374132159	7.082715	3.1921971
1.368489193	0.685712283	1.374132159	7.082715	3.1921971
0.273036509	0.165262712	1.132202098	1.155496186	0.49015
8.996011	12.7129573	19.18317467	21.24212438	37.164258
0	0	0.656830794	2.589836	0.563851749
0	0	0	0	0.392221
0	0	0	0	0
0.31287175	0.15692952	1.760723557	3.215324962	4.49153
0.187332	0	0.17894	0	3.081525282
0	0	0	0	0
0	0	0	0	0
26.53804	26.44949	31.75114	32.61714	57.9773
0	0	0	0	0
7.168532	6.07303	4.24542	6.06345	3.63346
1.98027	0	3.50242	2.52376	0
1.99875	2.40973	3.54214	4.20024	1.86524
0.455358	0.154997	0	0	0
8.0353662	4.073497569	4.8710239	8.769305	1.76499
12.78376	7.86036	43.3707	61.739	0
2.08285	3.59505	2.614	3.16167	0.931349
0	0	0	0	0
0	0	0	0	0
0.55589	0.344511	1.57771	3.56234	0.993788
5.36944	1.70078	45.2957	37.376	24.0213
36.5313401	38.51681977	54.38551	59.01103	111.247735
36.5313401	38.51681977	54.38551	59.01103	111.247735
36.5313401	38.51681977	54.38551	59.01103	51.4227
7.242499679	6.576401856	5.40275217	5.002876886	2.987017386
0	0	0	0	3.139010358
0.0152068	0.000176348	4.12E-07	0	23.0711

0	0	0.653515	0.293261	0.797992399
0	0	1.000028	1.37540364	0.797992399
0	0	1.000028	1.37540364	0.797992399
0.0972744	0.0490211	0.166916	0.19229	0.698830358
0.111858	0	0	0	0.773494
35.667552	33.28145121	21.2988506	23.45924418	8.568552
35.667552	33.28145121	21.2988506	23.45924418	8.568552
27.05701	33.07365	35.06738	24.928824	61.4687
27.05701	33.07365	35.06738	24.928824	61.4687
9.85013	6.66962	17.20899	22.67568	46.671
37.3462	28.55179	43.6973	37.5813	59.60087
4.625126	5.596382	2.893053241	2.039223603	0
2.4985252	2.733770903	2.893053241	2.039223603	0
64.691561	58.44517746	74.37459551	72.93668431	23.7514
18.71914266	15.9940321	15.34968202	10.56605286	8.86652
18.71914266	15.9940321	15.34968202	10.56605286	2.53125
27.671962	27.30961	21.251207	24.18784	19.6331
6.969099542	11.64549822	11.30054489	13.15657	8.724780043
90.1270345	85.4001078	61.27727069	67.0786619	21.7337
0	0	0	0	0.479144
20.64495193	11.600358	50.17916176	43.64249	13.82718
0.881593742	2.569523467	2.54762814	2.848055563	24.53520007
34.36043	32.19165	28.57606	25.26271	49.4274
12.0785	10.6079	7.32608	7.38864	1.71408
19.93782	23.57904	22.73209	19.00242	67.4788
107.80437	110.868111	86.3313139	76.4620784	136.7711
1.124124517	0.833849996	0.996831	0.765251	0.0476622
0	0	0	0	0
7.31784	10.72	5.74773	1.6086	1.2493
15.37837301	10.72534096	8.740172926	10.2714938	0.120588
7.135522897	6.88352417	11.51989834	11.95159825	50.20154
7.135522897	6.88352417	11.51989834	11.95159825	16.59323976
7.135522897	6.88352417	11.51989834	11.95159825	16.59323976
0.647325	0.186316	0	0.360873	0
2.40E-05	1.29E-19	5.68E-15	3.38E-06	1.20E-44
34.3272	41.1638	50.0739	37.79963	30.6117
0.004677848	0.301213268	1.25E-05	1.54E-06	0.0705031
11.776882	8.244668553	7.179039	5.37127567	6.107611563
11.776882	8.244668553	7.179039	5.37127567	6.107611563
9.13937	9.124914	7.77982	7.60951	3.06262147
8.46449	9.141733	8.466649	7.902338	9.54750228
0.498091	0.127295	0.246866	0	0.150618
53.6963	49.805	184.6422	199.065	339.196
0.132431	0.536492	0.259665	0	0.152834

0	0	0	0	0
1.90571	2.41977	3.77469	2.9134	1.857301
0.762652	1.28642	0.167213	0.450136	73.0095
9.493316006	11.08399409	8.12663293	10.38295496	1.65862
9.493316006	11.08399409	8.12663293	10.38295496	25.41357001
1.14838	1.07518	1.36424	1.2863	91.9171
13.0962	18.9279	4.31145	5.60222	91.9171
9.493316006	11.08399409	8.12663293	10.38295496	25.41357001
5.466261	4.088893215	10.74708	11.580182	4.53175
32.10462	33.90483	30.77607	26.06129	14.3383
34.65330532	38.71340486	10.77661249	13.15273667	1.851600786
34.65330532	38.71340486	10.77661249	13.15273667	1.851600786
11.5430419	11.9839342	5.631181	5.127760155	1.341480149
11.5430419	11.9839342	5.631181	5.127760155	1.341480149
17.05570482	15.15073757	10.55336372	13.68499325	4.107089
0	0	0	0	0
144.3701469	122.8798077	87.75413	88.20331387	12.931
472.19608	350.51496	165.66859	227.23283	7.587663659
472.19608	350.51496	165.66859	227.23283	7.587663659
4.21106	4.60869	4.28667	4.04773	0.79368
3.006123	3.881347	4.77278	3.25148	10.1442
2.81886	0	19.4048	0	12.1664
9.89374	6.830886	7.84753	8.33417	3.847943441
0	0	0.386941	1.06901	0
0.455913125	0.382347993	0.381111744	0.676258418	1.714034045
0.714366	0.719643	1.01963	0.732698	6.899810084
4.02234	3.83002	9.10003	8.47971	6.899810084
0	0	0.504651	1.49959	1.10957
0	0	0	0.11724	0
1.542894659	1.384223965	2.167457138	1.74371583	1.27963
0.383600946	0.5868328	1.0688304	1.1015396	1.125071
0.383600946	0.5868328	1.0688304	1.1015396	0
0.383600946	0.5868328	1.0688304	1.1015396	0
0.383600946	0.5868328	1.0688304	1.1015396	0.0652975
0.218435	0.163401	0.580407	0.427835	0.19202
0.903654	0.98857576	1.062118226	1.288518929	1.100758
0.903654	0.98857576	1.062118226	1.288518929	1.100758
0	0	0	0	0
21.12127738	26.9688822	13.133202	15.631333	8.597894131
21.12127738	26.9688822	13.133202	15.631333	8.597894131
28.61540001	29.1092	18.72028	20.61609944	5.97396724
6.32522317	5.206009384	3.416967098	4.368100861	16.96997
0	0	0	0	0
10.80870165	11.374224	8.092582442	11.35830849	12.30871638

11.60348385	16.35789	16.86987	14.49022383	37.19115
1.1434156	1.147505	10.421367	19.730703	21.42059
1.1434156	1.147505	10.421367	19.730703	21.42059
454.447477	453.109404	326.155699	319.17601	76.631136
0	0	0	0	0
12.025401	11.23111	12.88759	13.34627	5.617585
0.20890436	0.33098461	0.59178476	1.467414841	20.18447
0.20890436	0.33098461	0.59178476	1.467414841	20.18447
12.8511986	11.91653365	14.01781864	11.11938313	33.40920249
13.22817	13.60559	17.51911	13.11323	48.0366
18.8052	17.6954	32.909	19.8266	260.6446
137.90442	137.65782	151.2858338	181.1897782	48.1786
137.90442	137.65782	151.2858338	181.1897782	21.480424
27.14220022	33.6456095	44.19590018	46.12010137	75.1037
81.55155	83.20853	74.664	61.7252	96.8422
81.55155	83.20853	74.664	61.7252	96.8422
7.04895	8.54857	2.700317	1.36283	2.89009
5.40999	5.5346	7.02926	4.96048	3.34904
33.84099	33.542351	26.62989006	16.63730835	3.04610757
33.84099	33.542351	26.62989006	16.63730835	3.04610757
35.474976	35.8803638	63.81291	61.924575	53.1537
0.456496	0	0.674747589	3.97774	3.2859
7.905996695	9.25881599	3.8772761	6.078151643	0.364503
1.6438356	2.012716015	1.152787111	5.594435	0
0.326897	0	43.6745	84.8988	234.161
0.345565	0.688649	3.53661	5.92734	15.8438
0	0	0	0.0613122	0.223608
0	0	4.90485	13.0609	32.7696
2.509106	3.4559	4.731164	3.974808	43.1544
877.8324506	790.3181306	844.1440575	669.0100815	113.183237
2.6157278	2.0607507	1.089090112	0.702952	0.12102
2.552230652	2.155619523	0.353382795	0.504274186	0.33934
58.67875556	53.3082643	52.569614	55.136561	56.88193
7.414179528	8.882886615	11.29444605	10.7131825	17.3573
11.55423	11.87782	19.66521	20.01763	49.3614
11.55423	11.87782	19.66521	20.01763	49.3614
56.93087	53.275388	96.35460701	77.3159197	42.49390046
56.93087	53.275388	96.35460701	77.3159197	42.49390046
56.93087	53.275388	96.35460701	77.3159197	42.49390046
776.8414535	801.7224021	1190.608005	1219.937598	1009.907
0	0.772431	1.69668	0.989925	1.21899
19.657845	18.0948702	22.6899236	22.37719842	48.7608
0.0482074	0.0486368	0	0	0.0570521
4.09069	4.71691	7.04405	7.270693	13.57726

804.1197	880.13511	1334.6026	1244.5713	1204.7647
49.184638	43.2767699	73.3602983	53.82	84.845512
49.184638	43.2767699	73.3602983	53.82	84.845512
13.70120355	15.37987511	11.96256268	13.18434413	11.49040228
0.492587533	0.1043385	3.191735141	5.389501795	2.155076
0	0	0.269399	2.18606	1.02761
0.436507286	0.056094509	2.15957713	5.828598608	6.746651982
0.207081914	0.409217	0.2454135	1.07036224	0.491282
0.155517941	0	1.856524117	3.16219	1.802139811
0	0	0	0	0
14.502735	13.908757	9.15387	10.864727	2.14805
150.636864	141.2059889	137.7398145	142.3666061	96.316876
150.636864	141.2059889	137.7398145	142.3666061	96.316876
16.35495017	19.85613	17.68142229	17.25704825	31.81217405
27.34201	31.766355	33.50673	33.66594429	55.545
0.675986	0.932526	7.46407	8.48403	6.14568
3.340611931	3.756873018	3.6285168	5.413002	106.76885
7.6003	3.71067	15.12871	16.19819	347.6921054
50.25788491	58.8897311	90.99550534	94.20623328	347.6921054
50.25788491	58.8897311	90.99550534	94.20623328	347.6921054
1.027866	0.646524	0.9519638	0.60755	0
1.027866	0.646524	0.9519638	0.60755	0.300875
1.027866	0.646524	0.9519638	0.60755	0.217306
1.643758	0.972114647	0.6266408	0.761422271	0.42531
0.608171198	0.3575658	0	0	61.40254
11.64538554	13.372465	8.1081716	8.800552075	2.673890833
27.33762636	28.57917084	31.53170487	39.10975418	79.78973
13.58486	16.05133	16.52617	16.83831	64.9291
6.31745	5.0249	4.27475	4.04883	0.459257
19.441951	24.28675	24.70029749	24.412952	50.02051705
19.441951	24.28675	24.70029749	24.412952	2.50412
33.755	37.8686	50.6683	50.4277	90.1898
8.849397219	8.922937321	14.4092627	14.04143	29.49742193
182.153	141.04	49.0285	43.0464	8.46824
13.071724	12.282907	8.716682	9.534119449	3.043615575
25.80316	24.89588011	30.33547023	35.52003	9.27162
25.80316	24.89588011	30.33547023	35.52003	42.5914
125.78922	118.55781	20.15895	35.81338	9.31627
29.9759	29.6073	47.4858	42.1119	70.8138
41.98512059	43.75505925	32.1286071	35.73462686	6.795364
41.98512059	43.75505925	32.1286071	35.73462686	6.795364
41.98512059	43.75505925	32.1286071	35.73462686	18.23342
41.98512059	43.75505925	32.1286071	35.73462686	2.784340695
3.39784	3.453621108	3.728088	3.742	1.039950971

147.0615	148.19077	147.23737	162.11319	50.83580022
83.658735	83.431722	62.28348229	67.04790035	67.19414
83.658735	83.431722	62.28348229	67.04790035	67.19414
6.099712731	6.480515	4.663675	4.710711905	1.4233555
0.260222	0.36542	0.151572	0.618988	0.0600394
7.34534	8.68831	9.70278	11.77877	4.657583919
2.563179993	2.381944744	8.441479608	7.51769487	3.857
2.3092	2.15951	7.10875	9.39527	10.9015
0	0	0	0	0
4.25071	4.21724	6.38994	5.24073	1.85061
10.51501	9.400556	20.65915	15.68402	17.43889
8.30969	9.28695	10.8589	9.0534	17.43519437
25.12473815	24.96232133	24.06735435	23.852334	22.92475412
5.920810071	6.938540281	6.882060083	4.036050256	0.685642
5.920810071	6.938540281	6.882060083	4.036050256	0.417568
0.310651	0.307504	9.09E-05	4.15475	3.88059
36.1834	44.7191	32.3622	29.6353	61.3565
779.369	876.828	1297.85	1246.261	1295.687
14.98154	13.6417569	20.33932	19.405501	36.12381586
27.70354	25.44893959	34.92188595	34.63752671	47.0304
5.227685	3.93151328	8.38182	9.85761	6.197173
5.67E-20	3.32E-21	3.52E-29	9.48E-37	2.05E-06
3.347195	3.733409	4.52676	3.579013399	5.27644
4.28341	3.51436	4.4513	4.85087	122.97308
0.889327	2.04645	0.964382	2.11525	1.797112004
29.11218	24.82727	25.30649	25.293263	2.61787
3.58339	3.5186	6.48014	2.94338	10.4721
232.916	187.3759	175.1126	212.357	149.583
30.4744	22.2693	24.0949	23.8558	6.85011
26.23094579	27.11122658	40.98265563	39.49362439	104.3404677
0.364234067	0	10.6309	24.31819	19.8505
0	0	0	0	0
15.16368	15.35036	19.14449	19.05405	86.13303492
0.069839	0	1.06034	1.64731	2.44E-06
6.466760583	4.046936	7.305217342	7.481042997	3.321476
5.50357844	4.358858425	3.529247	4.194759064	0
5.50357844	4.358858425	3.529247	4.194759064	1.519568616
0.0548715	0	0	0	0
0	0	0	0.40417	0.359351
13.256998	13.62455115	15.22997315	16.434941	2.49974
13.256998	13.62455115	15.22997315	16.434941	95.4393
1.78385	2.50772	5.44827	3.79387	1.2959
3.20712	4.43348	4.76011	4.96231	36.86880004
0	0	0	0	0

33.376001	26.27887	15.65789318	19.93138	8.74498
15.2775201	15.788488	14.99547	15.83816	26.03649
118.8805	113.50981	97.86616	110.3941	60.640729
43.0268	39.9529	36.352	35.7965	29.8629
16.011355	15.88412333	16.15063086	17.80386146	24.5092916
21.74632	15.53915	25.78494	16.349341	20.537
37.52351	35.37274	34.16897	33.776956	40.73110188
12.64652637	15.78153707	22.75232193	20.37781144	56.2206
2.26703	0.248618	76.308	166.7739	101.259
0.173266258	0.187324463	2.5099681	4.241193	1.065599
0.173266258	0.187324463	2.5099681	4.241193	1.065599
0	0	0	0	0
0	0	0	0	0
20.891303	19.158835	24.434056	25.1584536	12.209593
27.9091	31.0517	21.68477	22.0428	45.4036
14.961967	18.29601984	23.82322048	29.06055953	11.686505
8.93161	8.19421	9.13074	12.8375	12.0607
13.447524	15.259446	25.146803	22.389154	21.54758
0	0	0	0	0
0	0	0	0.0647974	0.0293991
0.7907335	0.946554	1.512715	1.850036	19.0584
0.310087389	0.671306	1.026870197	0.575233078	1.2904937
0	0	0	0	0
0	0	0.19059	0.257359	0.333804
0	0.0773247	0.669933	0.604128	0.0905917
0.0947145	0.192618	0.185813	0	0
0	0	0	0	0
10.3143	7.21171	7.44171	9.16897	38.0706
2.630123245	2.266568705	4.30115641	3.955424519	13.3766
0.118902	0.121348	0	0.159498	0.789616
2.79304	3.68257	2.85378	2.87195	8.27377
0	0.0872063	6.56601	10.7167	10.7385
0	0	2.74909	5.23539	4.03264
0.375633	0	31.8989	69.2752	59.0043
0.401248	0.202853	336.7143	728.617	800.156
0.241828	0.566695	96.8053	181.974	115.168
0	0	57.32403014	115.6724281	38.490319
0	0	57.32403014	115.6724281	1.92281
0	0	57.32403014	115.6724281	38.490319
0	0	57.32403014	115.6724281	38.490319
0	0	4.37669	8.51649	4.08023
0	0	0	0	0
0.0898947	0	0	0.118019	0
0.918968	0.309061	0.372218	0	0.283563

0	0	0	0	0
0.223586	0.246251	0.218784	0.964961	0.0937624
0.223586	0.246251	0.218784	0.964961	0
3.10921	2.83885	1.57679	1.53614	0.489203
37.09938	34.02271	31.05071451	35.20376218	23.76502594
5.41243	2.62149	63.8958	72.6379	0
7.297088	6.12751	8.60976	7.790349496	1.01376
6.2704	4.399231	18.90579	21.62818469	27.25550642
0	0	0	0	0
21.6221632	24.19104196	28.68104709	31.53543467	43.91453
131.1751	122.9515014	118.2631034	122.0027011	57.77020184
56.3027	73.7428	231.133	115.758	28.92350116
0	0	0	0.285491	0
52.30323	54.36687	47.37121746	25.78383053	5.97032
45.49149	36.0631	49.6402	51.1227	238.794
4.52396	4.60518	9.90449	7.92165	30.74858
37.55827527	38.31825021	14.42609289	18.76274633	25.6464
0	0.196199	0.189728	0.0636567	0
37.55827527	38.31825021	14.42609289	18.76274633	0.288749
37.55827527	38.31825021	14.42609289	18.76274633	0.982041
7.553974535	6.687107556	4.51382152	4.96433372	1.799790541
3.896720134	4.15916	2.665635	3.243498	3.629830032
671.435249	617.371707	837.372582	607.20686	61.70569
671.435249	617.371707	837.372582	607.20686	2.21241
0.502001461	1.014131	1.533704	1.708658004	0
0	0	0	0	0
77.685	119.785	295.336	279.365	12.7907
23.681314	19.058278	23.352421	22.935511	3.51014
23.681314	19.058278	23.352421	22.935511	1.83725
23.681314	19.058278	23.352421	22.935511	38.80828
33.5671	32.8872	20.06681	23.0817	56.44135116
33.57304001	34.1463301	32.30459	33.783722	56.44135116
15.9884	15.8333	19.472	21.4274	10.605376
19.559661	15.838425	21.239115	21.8933611	59.5371
20.69199365	26.19511	20.68607	21.04409	42.49911557
5.785789057	6.10786457	12.98778108	11.9231623	41.91541323
17.93114	16.149961	19.883527	19.432338	130.8953
32.80431466	29.03604762	21.23057242	25.31130859	0.502584
32.80431466	29.03604762	21.23057242	25.31130859	0.81692
32.80431466	29.03604762	21.23057242	25.31130859	7.885775
26.465295	22.66281702	13.41664227	13.55936962	1.748510386
26.465295	22.66281702	13.41664227	13.55936962	5.157690147
30.6105	25.90505	34.1381	29.57044	50.93733923
5.19407	5.74246	13.17012	9.47674	46.0585

38.60192	33.87049	42.72398	34.16781	6.34455
17.87031622	17.16885121	29.64280609	27.26161557	16.83114344
0	0.0572389	0	0	1.05916
0.367505	0	0	0	1.04602
15.9145	21.2656	26.5448	22.4489	71.4208
60.053465	60.68952	57.744951	53.62071736	28.21796954
60.053465	60.68952	57.744951	53.62071736	28.21796954
2.058846	3.0742	0.239486947	0.752911	305.6028
105.160661	96.39911	101.3915077	101.3936093	110.0038542
30.44848	31.75953355	25.49799335	28.35555261	13.93034
0	0	0	0	0
25.16678451	26.07564489	25.98349101	26.48240975	43.20204
0	0	0	0	1.2753
23.109928	24.05031	26.384334	21.05688	35.3352
56.39951699	53.69496507	35.2899358	38.83725018	10.79521
26.547	31.0515	24.7961	26.7988	32.6316
33.17832	31.06802	64.46247	64.71768	38.24669
440.696	489.793	731.089	655.998	851.7090352
2899.54	2876.3	4039.32	3267.48	1516.54
27.73529	34.69888	22.40349	22.20837	4.517480901
27.73529	34.69888	22.40349	22.20837	4.517480901
6.340236507	7.226417106	10.4368174	11.12407643	11.042353
6.340236507	7.226417106	10.4368174	11.12407643	11.042353
38.87265347	34.27416553	24.64478	24.54547082	11.65012912
0	0	0	0	0
484.8612866	526.783593	780.5802543	710.4278619	1.47203
484.8612866	526.783593	780.5802543	710.4278619	773.984099
5.242709559	7.806787191	10.474868	11.17989	1.911812544
0.2940921	0.157148236	4.092212	5.420402926	0.681861345
0.2940921	0.157148236	4.092212	5.420402926	0.681861345
0.2940921	0.157148236	4.092212	5.420402926	4.890943
14.2085552	12.08878783	10.64930006	10.33068001	4.419781028
11.45341097	14.46579	12.45630237	10.61050733	40.79753
7.162325	7.01904	13.01084	9.066054	11.15588
44.1822	39.9536	58.9647	37.9261	96.0675
0.537554	0.273383	0.781469	0.354087	3.874400329
61.58604791	66.76061183	21.75308672	27.43827862	8.797230839
61.58604791	66.76061183	21.75308672	27.43827862	8.797230839
27.3138	34.3119	50.2165	35.7039	331.546
25.50039	23.91065256	20.1541	17.50527799	7.634752524
19.55536	22.93535	48.189	41.8722	25.18767
1.066635	0.8392183	2.897932737	1.899808	1.94144922
1.066635	0.8392183	2.897932737	1.899808	1.94144922
1.351317011	1.498407	2.004209	2.429182659	1.9649998

2.374762078	2.403046	3.962427	3.043528	52.1656
47.68593346	51.86503144	54.21533	49.46097605	19.26633733
47.68593346	51.86503144	54.21533	49.46097605	19.26633733
47.68593346	51.86503144	54.21533	49.46097605	19.26633733
19.18769	18.34331	18.20131	20.97402	105.2122
136.9342	144.3029	121.16384	121.928	223.3189
26.6964	29.2466	31.786	30.7093	51.8646
15.15660597	11.12438511	19.77951681	14.39038851	11.69072
8.814507096	12.24504718	17.704109	14.234662	3.005931255
14.5697	15.51799	19.08474	16.5344	76.5158
39.77041	42.9052	43.7467	46.0592	0.881549
39.77041	42.9052	43.7467	46.0592	191.05974
0.134629712	0.176963836	0.0445389	0.299109685	46.4026
1423.978	1113.997	680.7248	880.2866	8.67522
2.93315	2.63376	2.44695	2.64037	0.531484
1.14532	1.96877	2.941207	1.24781	0
1.3101	0.876415378	5.30369567	1.7005646	2.305548609
8.855139	8.343792841	12.78131601	14.20089553	36.05887103
33.698013	28.55650074	28.141822	28.033275	36.05887103
15.24868	16.11267	14.95018	16.3533	39.2315
28.63494784	29.1792106	22.95129342	23.89046087	22.52134
7.1377	7.25715	10.36388	8.94802	28.2535
12.8854	10.5673	23.4909	18.1908	38.8718
15.389098	17.951843	15.101897	15.244414	8.24399
54.29221097	54.46911155	33.82410812	35.87230718	81.34785
0	0.0517815	0.0503417	0.135097	0.183024
29.5298	27.56548	29.42664	25.81793	19.5162
0	0.661684	1.18969	0	0
0.3952288	0.0327912	3.648946	3.709768	2.872372903
4.636183964	4.229844055	6.41466	6.132739914	31.618617
18.443096	19.711499	13.36223788	19.310876	31.618617
0.326344	0	0	0	0
5.92E-11	0	2.03203	7.04328	9.02702
509.35658	545.81998	648.2243142	672.1381662	534.493
7.071490004	7.363305	6.894893	6.1954	33.34062
0.310656	0.236471	0.191667	0.0514349	0
2.0145891	2.833511	4.419186	4.172657	9.891986279
2.0145891	2.833511	4.419186	4.172657	1.341392963
11.615261	9.748406837	6.133383572	5.934912218	1.595772147
8116.1286	9973.0453	28863.786	16206.8709	243.217
8116.1286	9973.0453	28863.786	16206.8709	39.5537
30.448707	30.402347	25.6031	25.3134	25.3026
23.40891	22.64486	24.97927	28.13823	42.1451
31.21578131	30.314791	25.50008172	25.95236	6.370254055

0.167357256	0.378584669	0.607442585	0.650965209	0.136901
0	0	0	0	0
15.40563782	11.14127	12.945881	10.37639346	16.28557364
15.40563782	11.14127	12.945881	10.37639346	16.28557364
13.85203	15.51539	8.66334	13.4361	41.38466
89.5633	84.1919	63.8958	72.6379	35.277
230.3976062	233.9439	178.76412	169.8761	287.50713
0	0	0	0	0
35.06689639	35.55618276	23.60542845	21.30456268	3.983170517
35.06689639	35.55618276	23.60542845	21.30456268	3.983170517
0	0	0	0	0.116275
0	0	0	0	0.610388
0	0.111021	0	0	0
0	0	0	0	0
0	0	0	0	0
0	0	0	0	0
0	0	0	0	0
0.275662	0.329368	0.46967	1.09178	0.665451
0	0	0.144895	0	0.185692
0	0	0	0	0
0	0	0	0	0
0	0	0.233204	0	0.422102
23.76214	22.42579612	14.269879	16.2927077	6.356753569
5.48	8.64631	14.0618	13.757	5.98192
33.6928899	36.60921886	18.89023015	22.79037247	31.95134055
20.09603	18.80323	12.06606	11.758134	1.986088
1932.7	1560.22	747.877	767.141	30.8905
2.543715	2.151036	3.182	4.45489	29.4217
2.512926996	1.938245071	6.612687263	4.180590894	6.48241
2.512926996	1.938245071	6.612687263	4.180590894	10.6987
5.65337	7.2394	7.07811	7.70211	82.6932
1.378261	1.233884	1.662953	2.542528	1.08525
2.27894	2.40762	2.79805	1.85359	0.596985
22.455274	16.893176	19.345847	19.90163	5.474825396
22.455274	16.893176	19.345847	19.90163	5.474825396
43.24936009	56.299586	63.42732125	60.40226204	126.9577
23.7159783	23.26317231	23.98057073	22.94053838	57.97110482
34.78923699	39.9772092	44.25125	30.78896006	13.94898753
9.869668	13.15721	15.66942	9.15562371	27.8309
27.98717268	31.46803855	45.6033205	41.75053068	96.8011
45.98958694	34.92679248	38.07445116	32.47758	85.47
45.98958694	34.92679248	38.07445116	32.47758	59.58
27.3593	22.84276	21.5865	24.1066	8.649471276
52.2355	48.879492	38.336295	39.325854	28.87509

0.8940319	0.92872545	1.433586151	0.981951459	1.354585
3.82572	5.00642	7.01651	5.24965	6.4799
140.7409298	138.9305302	130.7857895	117.1696401	72.0942
5.263468144	5.111710018	6.60244	6.434033578	30.23696703
5.263468144	5.111710018	6.60244	6.434033578	30.23696703
10.40862	10.20611591	10.09515702	9.873984639	3.912653
2.175633106	2.389442	2.220618	1.65645013	0.539219
11.65404	11.23818	1.859157	2.238238	0.920196
0	0	0	0	5.193474
22.37326348	20.13368566	16.68387285	16.2366884	4.397077279
17.79755	26.24588	25.17485	26.6893	9.54154
0.251967	0.291583	0.0357021	0.143795	2.07626
4.87398	4.90522	7.47657	9.25462	26.151
54.46907	63.42883	51.85605	46.2116	69.4627
40.14485	37.16604	23.915304	28.475103	16.0301
33.32209	31.31775	18.61588639	17.964278	5.344494116
7.611016	7.193349	12.11578	10.263119	26.907638
5.83585	6.19451	6.38707	4.98239	2.05064
29.78182828	42.68563659	49.68000006	37.14324004	45.9849
29.78182828	42.68563659	49.68000006	37.14324004	85.9731
15.35986	10.93761	2.617814	5.233666457	0.0854422
11.62341	12.92275	18.95897	19.31829	50.4264
7.082720149	7.153767	4.348840676	5.824642	0.580142
0	0	0.987389	1.81878	1.4646
0.141546878	0.015566393	1.694800075	4.37697	5.51885
31.55955	39.17166	54.30938	34.69001	53.7519
187.65286	169.2723412	212.9927323	198.9196145	135.733
5.21426	4.69456	6.64981	5.0331	13.3136
43.83033327	42.90366806	43.24191959	46.83111789	87.9795
0.7157546	0.6482054	0.558122468	0.538984	3.34232
5.12928	2.72636	12.2154	12.5239	0.104297
0.153755079	0.565302993	0.505552312	0.2781254	0.581457
2.5863	3.22636	3.40948	4.096	29.148
60.3095	73.7394	77.8611	70.7079	73.4572
24.20465381	24.6960305	17.9122841	17.74964686	10.17157267
14.69039	14.241704	16.48466	15.096529	31.92444
853.989152	817.7007533	1312.014485	1223.377048	1037.176
853.989152	817.7007533	1312.014485	1223.377048	57.92962
56.5105	50.6538	78.1154	62.2216	47.7224
85.904394	81.22472	64.343439	69.25969	41.41812436
0	0	0	1.07039	0
0.129976	0.267272	0.37164	0.168775	0.143905
17.13417	15.85203	20.85853	18.68153	53.1791
0.096134854	0	0.873673586	3.574482118	8.91E-06

0.300864	0.0613173	0.599926	0.160882	0.0739658
0	0	0	0	0
0	0.252921	0.16229	0	0.0992934
35.61802241	33.25801973	26.016966	28.07440553	8.490542264
0	0.470378	0.303374	0	0.364519
9.33796	8.96431	13.43534	9.73811	16.44695
6.182767	5.97069	11.87420092	12.62936	91.9163
8.05329	10.079	10.6904	9.20662	7.46953
12.67832	12.73102	24.7234	22.50155	45.53691935
13.48721	12.67896	19.88016	16.91398	39.3426
23.03874	29.9582	66.2245	65.4473	35.55013
28.039653	30.6008707	27.8059	27.131066	69.2537
0.18639	0.252072	0.924639	0.45612	0.56137
6.324375398	6.86399906	7.060517383	6.754768463	47.86771
32.24697	33.38214	16.13608	19.59138	0
14.0186	12.1934	12.2154	12.5239	4.71292
6.962565	6.981649	5.453379	6.592431	1.82861
375.203	319.601	394.531	389.642	255.084
13.98554304	13.14470802	16.74773323	15.81038309	23.83656
1.621047	1.136273	7.00884	3.08952	1.04951
0.674263934	0.314308901	0.152607408	0.655754	0.425846
51.9952943	51.3327464	29.82209838	30.87275468	8.36536
13.4293	15.8886	30.1939	30.585	115.79
76.14805173	85.452975	38.68925	40.81397	142.2206
76.14805173	85.452975	38.68925	40.81397	142.2206
3.20006	4.61E-16	1.90744	0.86909	6.20781
1.0659	0.931934	0.887566	1.18742	0.394791
48.06580184	42.03866	21.51536291	22.7012781	6.4395586
7.75882	8.77145	7.55103	7.41874	26.779
124.9691	143.3694	97.4826	126.7455	2038.056
11.26536	12.32353	18.45018	15.10004	34.92923812
0.467092	0.284017608	6.808930355	13.966253	24.29650076
1.411852	0.60153782	21.78114	46.9464014	33.5448
18.97676478	17.5465317	14.400169	16.82196919	5.3577439
0	0	0	0	0
0.512816	0.206944	0.491926	0	3.656872
9.524002	10.922896	21.676967	16.084634	5.508685
154.24812	109.85348	22.180554	24.16886	3.729003
210.2710065	211.8932411	259.9259326	242.7696559	124.5073
33.77714	32.53311	30.91700001	32.64978	35.06961553
0.0791512	0.0403101	0.039239	0.105243	0.0476083
301.7520424	242.6491278	64.14140321	89.856571	5.53413
0.282171723	0.121263	1.711059	2.81938	247.4383
0.282171723	0.121263	1.711059	2.81938	247.4383

13.65903	6.11678	8.19750529	9.119442553	0.644717
41.93730048	39.99883383	36.62497766	38.26044528	11.3527296
15.28743929	13.24605597	10.68280904	8.270744436	0.70927
15.28743929	13.24605597	10.68280904	8.270744436	7.714912
15.28743929	13.24605597	10.68280904	8.270744436	7.714912
15.28743929	13.24605597	10.68280904	8.270744436	17.2673
15.28743929	13.24605597	10.68280904	8.270744436	109.87903
112.389574	119.1903103	99.244049	97.83576654	225.54099
112.389574	119.1903103	99.244049	97.83576654	225.54099
11.97739	12.69744	25.890543	32.904609	57.86618
11.97739	12.69744	25.890543	32.904609	57.86618
11.97739	12.69744	25.890543	32.904609	57.86618
41.08077	53.2698	51.8256	49.90144	73.44137
37.01425	28.34166	30.32725	33.47729	94.3045
37.01425	28.34166	30.32725	33.47729	94.3045
1.675228687	1.612979029	6.009034	8.23378521	8.20272
15.3015	14.579	12.0691	10.5304	2.57643
19.38154871	19.65602761	30.64455775	26.11400752	46.2252
28.17091	30.92559	26.14394	25.1918	45.0601343
14.007721	12.71461881	30.35271649	28.76528209	39.77741
18.174774	20.702314	19.735684	19.935926	30.1128
46.18237	50.11786	39.13889	35.485	95.45710445
31.38453506	28.92495685	22.91071751	24.83519273	8.195765512
31.38453506	28.92495685	22.91071751	24.83519273	8.195765512
15.70136	15.28876	19.09233	15.92062	50.4667
0.968248729	1.491586041	1.46207433	2.448343207	13.89053
0.968248729	1.491586041	1.46207433	2.448343207	85.62758
19.67684	16.47840003	28.103558	34.444627	7.786038094
12.91839	13.64748	30.651249	33.2902	28.11861
12.91839	13.64748	30.651249	33.2902	28.11861
18.37543	23.88725	15.51645	12.1313	21.2725
40.21777276	39.0601364	40.89875601	39.15269537	60.2012
5.87247	6.42002	6.85826	6.64777	29.7495
39.5274078	43.85806433	28.42901636	29.72491028	4.842363647
64.4299	42.7742	16.4842	26.5649	0.305971
42.76967934	40.36889285	8.195532606	10.11546214	3.64558782
0.0604588	0.0612458	0	0	0.146929
0	0	0.0267906	0.145718	0.189115
0	0	0.206315	0.139548	0.24665
0.0241623	0.0461939	0.898635	2.95443	16.6828
0	0.0879699	0.260353	1.9726	15.6682
0	0	0	0	0
3.070004	1.0498146	2.62483	4.331524	2.64341
0	0	0	0	0

2.020546707	4.15697395	8.348444211	10.73550321	3.41292
0	0	0	0	0
0	0	0	0	0
0.043434224	0.136802212	0	0.088948822	0
0.043434224	0.136802212	0	0.088948822	0
1.597577	1.642762668	2.819484	2.95888	1.17588
0	0	0	0	0
0	0	0	0	0
5.823712543	4.6971778	11.00914151	11.75034263	71.294
5.823712543	4.6971778	11.00914151	11.75034263	58.140863
32.84696	30.87048	43.0548	30.66264	89.5534
20.81460443	20.883287	19.2611298	20.06608376	70.97178264
334.99543	300.7165728	20.864176	96.199141	1.7262
334.99543	300.7165728	20.864176	96.199141	4.84523
494.0844195	554.994	787.7092	754.887938	774.7429366
0	0.311633	0	0	16.2811
0	0	0	0	0
8.5419935	11.66391	13.66755	15.23321	25.2201
7.72859748	8.326184	11.61046521	10.035942	32.7303
13.9705	10.11848995	20.09780011	14.67024052	2.580221311
13.430635	16.10989317	22.616897	19.672726	24.73911
0	0	0.86072	2.54288	4.31939
8.684254	8.568427766	11.40251491	9.425201	32.26973716
0	0	0.293856	0.651312	0
0	0	0.436031	0	0
38.63882	42.17864291	30.6140658	29.61536689	24.23840339
0.620443457	0.642813585	0.082737657	0.223597616	47.0761
253.8845	270.5765	301.9358	228.0192	49.40708
27.19285	33.99632	24.29381	23.18376	58.2473
22.54818	19.71016	10.24126	11.09892	32.506631
48.5897	42.03	55.7442	51.3782	98.91512
227.3219789	242.1141697	222.4625075	232.7796126	209.8167
0.804199	0.406686	1.14002	1.38159	1.055613
2.70376	3.32555	2.193	1.65568	10.8359
0	0	0	0	0
0	0	0	0	0
0.497914	0.506194	0.219662	0.369368	0
10.96954498	13.2404781	14.98629	22.17959	78.64096269
4.2473829	3.534248	2.586949566	2.560147074	0.628401701
4.2473829	3.534248	2.586949566	2.560147074	0.628401701
1.36153	1.11022	0.473535	0.543609	0.255176
1.76737	1.40292	0.0739761	1.14E-05	0.759464
0.403787	0	0.258038	0.173769	157.179
9.944757687	8.917733535	13.98511966	11.30809103	15.83268

119.361643	109.26603	66.50709	69.66135	10.33882
119.361643	109.26603	66.50709	69.66135	10.33882
199.4273982	197.133631	15.38458625	46.64706598	3.1101239
0.000440281	0.000164606	0.0041205	1.31498	2.30E-06
0	0	0	0	0
0.000232465	6.88E-05	2.02792	1.06336	0.000190154
21.49844	19.3317	20.25431	17.65522	47.74266681
22.86655	20.67973	21.44063	20.27355	59.007007
0	0	1.00432	0.272149	0.745791
0	0	0	0	0
0	0	2.1931	0	0
17.80842	18.27566	25.76362	18.06093	20.6402
3.67335	4.43287	6.27081	3.22154	8.67492
10.38547029	9.409339114	15.1393686	8.603250381	3.720748642
0	0	5.3008729	13.91544	13.503762
0.575473	1.05489	1.58591	1.12809	0.922693
108.6568097	120.61503	115.51238	109.8402369	108.97516
27.86279336	27.45462599	31.73407036	30.98579	27.88109849
0.917909	1.24327	1.77186	1.68532	3.59352
0	0	0	0	0
0	0	0	0	0
27.86279336	27.45462599	31.73407036	30.98579	6.79558
23.9174	25.3983	26.5788	32.3847	55.7321
25.57866	26.05783	24.1468	23.50955	40.0556
1.192978005	1.558557836	1.475576	1.666311082	16.594627
24.25474504	20.9263487	14.53178833	17.79928116	4.926603727
29.74366517	27.970425	34.68132	22.4979653	64.3558
139.57888	169.3120703	111.3963	110.03368	205.562016
36.15084	28.05211	20.24029	20.28642	11.69010022
23.99141941	23.99050619	21.55371362	24.50485484	12.13145737
0	0	0.17228	0	0
11.78978086	11.08055742	11.36179413	11.41897014	41.31962
11.78978086	11.08055742	11.36179413	11.41897014	41.31962
25.98796	21.09634	10.486679	15.76885276	45.09371533
25.98796	21.09634	10.486679	15.76885276	45.09371533
0	0	2.00411	3.75071	7.07816
34.00780239	34.51790104	31.06669751	32.71766854	23.72041675
6.47634	8.47248	10.65644	10.36967	50.17038
301.4085467	283.6929493	277.4841807	218.8669409	66.381
0	0.131243	0	0	0
0.0566657	0.113516	0.109048	0	0.133045
0	0	0	0	0
0.139486	0	0	0	0
0	0	0.0855138	0	0.318013

0	0	0.153923	0.630498	0
0	0	0	0	0
0	0.0441244	0.0432555	0.058388	0
0.0480166	0	0	0	0
0	0	0	0	0
0.217457	0.124444	0.0908896	0	0
0	0	0	0	0
0	0	0.131683	0	0
0	0	0	0	0
0	0	0	0.0921474	0
0	0	0	0	0
0	0	0	0	0
0	0.206694	0	0	0
0.134772214	0.099460231	0.0675239	0.139774487	0.139707
0.163142	0.166288	0.481125	0.939131	0.338206
0	0	0	0.356693	0.156428
0	0.386048	0	0	0
38.53475	39.02067	35.96766	36.61002	81.95145
30.7192	32.8544	39.9173	39.4443	79.042
22.05870563	25.30505015	21.91142	21.41530016	44.84342828
22.05870563	25.30505015	21.91142	21.41530016	44.84342828
52.59532328	57.111959	20.74306811	24.24345	2.33537
52.59532328	57.111959	20.74306811	24.24345	66.9508
269.575	296.6398	300.6063	360.997	745.511
269.575	296.6398	300.6063	360.997	745.511
0.0635021	0.0644171	0	0	24.7246
1.46E-05	2.61E-05	5.51E-06	1.38E-05	2.04E-05
138.6052	158.9345	204.5437	161.0829	238.256
21.90474726	20.07739589	21.31276838	21.49197409	19.15455
64.1822	68.06954	89.9388	69.1005	92.78028
0.822883	0.27169	0.266215	0.119411	0
0	0	0.653235	0	0
0	0	0	0	0
23.026087	23.243295	22.44789	22.40772	18.35436
115.51968	111.381728	99.2515	95.03704179	168.3513
78.41585003	100.18834	26.24820052	30.90409012	54.7343
12.702088	14.6965	16.68024	19.72683	7.95244
3.92933	6.05085	4.065493491	4.70025	1.69018225
0	0	0	0	1.259352113
35.17395599	27.80829866	3.74104843	8.492005489	0.645070198
78.75917	85.60095	84.543563	82.2913	139.187
42.71031318	41.80639103	51.28666	43.22593	40.34726
20.5217	19.131885	23.30051	22.61343	20.968969
1.076440437	0.9222141	1.109836	1.681610132	55.6975

10.31969	14.5103	16.43348	16.24776	28.46347
42.4442	44.5775	46.9992	45.2295	98.0918
159.0115657	152.76028	145.1151227	144.7095537	126.3073
29.11406	29.555132	30.5058	28.88628	28.7989163
22.43428	23.96148	24.13619	22.53815	19.06683084
0.677558	0.649181	1.45814	1.42197	0.246694
0.175047	0.176122	0.915836	0.614913	0.630834
0.863123	1.95569	1.64691	1.12735	0.994188
27.87821982	23.29089836	8.465297273	10.669953	0.223059
16.42333	14.79867	16.52313	16.11767	33.20868
4.76911	5.46093	3.828666	3.457444	0.796697588
8.30984	5.554231	10.3608	10.42242	130.9633
6.969191	9.373189936	13.663283	12.98832591	0.517882
6.969191	9.373189936	13.663283	12.98832591	0.23349
15.1641	12.3281	17.7285	15.1607	11.2179
6.969191	9.373189936	13.663283	12.98832591	17.03360005
6.969191	9.373189936	13.663283	12.98832591	17.03360005
0.23709	0.167216	0.164106	0.282883	0.491592
11.540388	10.3658715	15.48176615	12.542489	8.927181
25.251175	27.2497192	29.109364	29.608286	60.05215
55.80861407	45.56500853	128.915609	156.4479088	60.05215
55.80861407	45.56500853	128.915609	156.4479088	60.05215
34.568436	24.396188	27.67752	23.0290241	1.458802965
3.77122	7.86218	3.9307	4.70758	3.336267172
0	0	1.7734289	2.552659952	1.637405
0.043011329	0.04610743	3.041031	4.39398	1.91368
9.13926	10.39589	10.94505	10.7951	54.9121
12.22617	15.49297	14.40291	12.83647	34.1724
4.86047867	3.730073851	13.18955317	12.29610951	11.791358
110.5877	108.9753	140.636	150.668	55.06
41.027448	38.27657203	46.804993	47.36629	53.8004
41.027448	38.27657203	46.804993	47.36629	47.25691
0.02379498	0.062330267	2.282238029	5.84954	5.706207673
0.02379498	0.062330267	2.282238029	5.84954	5.706207673
20.42767	21.63176	18.71156	18.61791	26.4162
0.29669058	0.753586	0.158094701	0.172230397	226.7133
0.409904	0.144193	1.51878	3.08081	8.17032
0.4102684	0.621297	0.320312	0.1799708	0.144208
669.2953	773.5740861	1161.375485	1076.3794	1052.850575
35.77850621	33.97325274	28.1374677	26.34080919	1.76212
4.80443	8.40909	11.2104	9.62926	8.74612
29.50794318	22.14339071	43.42028363	32.01714255	10.3761047
29.50794318	22.14339071	43.42028363	32.01714255	10.3761047
29.50794318	22.14339071	43.42028363	32.01714255	10.3761047

16.98873	18.28061	25.4216	21.20051	66.01366
24.19707	29.36936	31.40907	29.22634	59.5128
4.64619	5.70666	3.27397	3.0935	6.70474
4.18759	2.9661	5.40699	8.05354	7.77697
1.06446	1.37871	3.66769	2.16974	3.15975
0	0	0	0.634804	0
19.9407283	18.70249287	19.46363944	17.80049937	3.315835252
0	0	0	0	0
0	0.0256393	0	0.067097	0
10.379896	11.112892	14.334106	11.266896	35.061
10.379896	11.112892	14.334106	11.266896	38.55091
30.44171	31.47576	33.93473	28.08545	48.8305
1.591635768	1.628323502	12.743234	25.55691	30.97842
215.2297	252.555	258.3122	248.079	539.438
3.379759	2.96844	15.08052	17.50923	108.4932
0	0	0	0	0
11.69213	7.00357	11.791	6.049036	2.232772562
11.69213	7.00357	11.791	6.049036	2.232772562
60.2766	56.0039	31.37211	30.0348	2.95428
12.3653	8.38207	21.1333	20.69	36.2395
5.83194	11.0972	11.6024	7.26837	0.00558506
38.971	34.1379537	28.625813	33.8321313	3.9533
12.643828	13.193826	16.009399	12.2635705	57.2955
12.643828	13.193826	16.009399	12.2635705	55.3712
12.643828	13.193826	16.009399	12.2635705	33.56125
47.47524	55.432614	53.22509084	50.12872473	42.18983
47.47524	55.432614	53.22509084	50.12872473	42.18983
30.4095	30.94658199	22.60784	26.4739	40.90429
5.77913	5.32329	8.52251	7.6692	35.0028
0.97391	2.88265	0	1.20401	0
53.84432	47.62633	57.91848	60.21084	43.79764
19.70696	27.75743	17.20098	26.8574	0
14.453346	15.80209	20.44142007	18.42804	48.8864
15.7043	18.63924	22.49118	18.35638	29.1822167
17.57561	20.2685906	20.6405625	17.2851728	3.667251884
17.57561	20.2685906	20.6405625	17.2851728	3.667251884
7.64822805	9.19230115	11.10972192	8.278448272	15.60273579
19.47884319	24.1093679	21.52744579	21.4041137	64.08692252
254.0424072	269.4894105	413.2146543	318.5362714	1350.787
15.684061	16.997681	14.12254597	15.669212	18.758465
0	0	0	0	0.0404391
0.106409	0.0536935	2.192831412	4.174126176	1.950915
0.106409	0.0536935	2.192831412	4.174126176	1.950915
5.36028	5.782447	7.29928	8.74832	7.84359

53.29201138	54.06850152	76.46651278	90.26872165	117.3230415
1.58704	1.57954	0.246513	0	2.51868
0	0.0955788	0.0927775	0.375077	0.0742486
160.83891	105.45434	82.06591	100.65479	15.5977
9.073959871	10.51424656	10.82989957	9.127355557	22.45421378
76.2886	101.166	77.4974	75.9479	76.3734
7.54971	9.61763	11.7577	12.0084	88.5606
7.783408	5.401761	14.19443	11.22568	5.04952
32.1095	30.74779	26.81481	29.33751	47.0577
46.35356094	44.56750275	47.75934053	54.45541094	45.32830435
46.35356094	44.56750275	47.75934053	54.45541094	280.6761354
6.23083	7.12837	8.46524	7.04702	0.000287702
0	0	1.18904	1.71445	0
1.90477	1.57909	5.64781	7.79883	76.66404
16.39871	17.0291084	20.7249	17.47239193	28.10056
16.39871	17.0291084	20.7249	17.47239193	28.10056
4.839901	5.219074	7.47393	5.229629	17.7361
52.66574	42.85698	46.6677	55.9692	5.93503
52.66574	42.85698	46.6677	55.9692	17.2342
98.44412455	101.3764021	67.72931106	74.47437995	91.16970554
98.44412455	101.3764021	67.72931106	74.47437995	91.16970554
2.110829129	1.690027221	2.755524405	2.918325	30.24628
0	0	0.218602	1.81313	0.421337
15.32867598	18.9185931	21.88060542	19.26287469	35.60873058
0.436601011	0.308931	2.341209	6.07853	3.218378764
71.85740001	69.69477798	33.51258898	39.59541116	11.01287
9.781604621	8.2577166	11.15856717	13.0874054	17.15736
5.067051006	6.14993012	5.37228065	4.91075	3.66091
152.65594	158.43839	185.9346151	183.8254729	145.17201
5.238698	7.557366	17.49532	15.95273	21.38945074
29.29811	39.549778	29.78188	27.53358563	27.22973645
6.74503	6.8515	16.32039	13.91418	7.921284
16.616	11.81640002	4.483760217	5.815790035	0.266526
67.82775	55.59331	15.78108	13.33027	0
0.0676216	0.061499462	0.956122	1.557188	0.710112279
39.6634	52.9129	48.6446	41.3365	3.42769
8.2045	7.4624	14.59199	12.64123	23.2718
90.8377	82.53138	57.72454	58.6205624	20.84449
1.08838	0.926019	0.4205469	0.763118	0.0685424
55.491371	62.90387	50.449897	49.57959	65.4174063
1.662827421	1.124272	1.618696	0.986313	0.36621
1.662827421	1.124272	1.618696	0.986313	0.849003
45.5164	42.90611	28.5763	27.4756	6.619336
0.329446	0.330812	0	0.429931	0.201156

34.2895	38.5744	42.3749	39.7301	94.8806
0	0.345812	3.58E-07	6.65E-129	94.8806
23.3679558	21.3039858	31.848615	24.024264	37.92669148
22.7643	17.8483	40.9064	50.5217	204.49
23.3679558	21.3039858	31.848615	24.024264	37.92669148
23.3679558	21.3039858	31.848615	24.024264	60.03300036
23.3679558	21.3039858	31.848615	24.024264	37.92669148
23.3679558	21.3039858	31.848615	24.024264	37.92669148
23.3679558	21.3039858	31.848615	24.024264	37.92669148
51.494171	50.463093	39.20926799	43.23558	19.677691
0	0	2.53498	11.5259	14.0956
0	0	0	0	0
0.747563	0.140236	10.2854	19.6217	10.7407
11.6992	18.9627	29.7564	49.1308	17.3336
0.809389	0.148439	0.216114	0	0
0.737331	0.250748	0.365175	0	0
64.75662	88.53344567	79.19821066	69.69146095	81.51278
11.03297202	9.755518002	9.129298552	11.66152501	1.00884
11.03297202	9.755518002	9.129298552	11.66152501	3.28453
0.265018	0.268317	0.346862	0.352013	0.104796
66.18575	61.31557359	95.9889419	87.89822827	111.94884
66.18575	61.31557359	95.9889419	87.89822827	16.8208
0.742864	1.6675	4.11E-06	4.92802	2.61E-06
11.03384	6.080199819	29.49201	20.19356	36.2978
1.18594711	1.40447642	2.306165059	5.033005853	106.6799
1.18594711	1.40447642	2.306165059	5.033005853	0
2.466130359	2.989343199	1.7937199	2.256714	6.50926
202.4158	201.5241	260.8968	199.6974	181.86663
12.801107	15.034596	16.823269	13.142315	4.81838
6.35954	8.02718	13.9553	11.5336	44.9883
0.193708	0.328112	0	0	96.8879
26.76689	30.986	27.11887	24.60065	56.06571756
26.76689	30.986	27.11887	24.60065	47.3771
1.258315606	1.202133982	2.3562675	3.69159152	2.275226
4.389279773	4.433663775	6.952785242	9.081394396	0.651876
4.389279773	4.433663775	6.952785242	9.081394396	15.53587174
4.389279773	4.433663775	6.952785242	9.081394396	15.53587174
10.4452	12.7603	15.1851	10.0409	8.6436
48.9817	52.01859	65.95844	64.631481	73.98431699
2.47829	2.29645	4.07263	3.30872	15.1684
0	0	0	0	0
84.7052555	78.9662385	79.48021354	90.00281241	1.71945
84.7052555	78.9662385	79.48021354	90.00281241	2.64197
84.7052555	78.9662385	79.48021354	90.00281241	1.36482

84.7052555	78.9662385	79.48021354	90.00281241	38.52335804
84.7052555	78.9662385	79.48021354	90.00281241	38.52335804
84.7052555	78.9662385	79.48021354	90.00281241	38.52335804
84.7052555	78.9662385	79.48021354	90.00281241	38.52335804
84.7052555	78.9662385	79.48021354	90.00281241	38.52335804
84.7052555	78.9662385	79.48021354	90.00281241	38.52335804
1.831594645	1.515304814	5.37069609	3.921694776	13.14113728
1.831594645	1.515304814	5.37069609	3.921694776	13.14113728
9.10783	11.8333	21.0062	13.7305	27.63842
10.1067	27.9879	6.25315	6.88082	0
0	0	0	0	0
8.390691431	7.15529261	9.755794741	10.18789064	4.41109
0.039974879	0	2.40428	3.633709	3.13288
0.0984962	0	0	0.132188	0
3.221693065	4.434023799	6.164735854	6.687214341	5.831903228
0	0	0	0.117461	0.112382
24.34018775	25.75544303	27.76392476	22.43727513	0.314999
24.34018775	25.75544303	27.76392476	22.43727513	28.87631062
5.32114	10.88532	12.34268	13.86357	29.86886
5.32114	10.88532	12.34268	13.86357	29.86886
0.175421	0.178204	1.37627	1.86068	1.65758
0.328938	0	2.04398	3.74445	3.119893388
0.374229	0.06655094	2.96266	7.05367	3.119893388
23.6087	21.3039	49.201	65.2521	23.546
3.2155	2.16764	2.68788	0.92834	0
43.6072	34.3062	91.4849	124.5737042	162.127
10.15595525	10.090707	15.75202557	14.87348277	21.66832
39.05541	35.2924091	28.66805	37.25922	4.514295288
29.135258	29.701418	30.9176191	29.28346287	57.39025
0.255228	0.0429565	0.169089	0.113455	0.0504642
0.222651	0	0.207638	0	0
266.0378819	252.763	158.8829519	167.6385837	49.5535
86.36214	84.6692159	77.2616368	77.7013967	117.1586
12.10451504	14.87238921	11.30285523	10.82538376	12.89374
0.000668811	0.394402	1.56E-06	0.000873979	0.276184
0.226276	0.525092	0.449074	1.25E-05	0.758295
0.74521	1.22457	0.233095	0.636601	1.13356
65.47453432	70.16097788	66.406206	67.88749	20.8998
65.47453432	70.16097788	66.406206	67.88749	59.8476
12.24661037	13.20679507	14.4240866	11.54192261	20.3873
12.24661037	13.20679507	14.4240866	11.54192261	30.25480237
72.7389	67.6246	161.056	196.554	100.2763
0	0	0.464331	0.938811	0.414814
5.486980949	4.1308898	3.846591159	4.891358474	1.733444373

0.241639878	0.113270146	2.255453794	2.918661245	2.800771
5.16092	7.19997	10.1294	19.8229	10.4472
2.68816576	1.995049768	2.350802385	2.913234204	94.2892
34.21952252	32.04837237	24.20402871	29.26151263	9.671057282
0.134172	0.225078	0	0.23373	0.108167
0.431238	0.388161	0.188695	0.565036	0.0582678
0.248914	0.500854	0.716852	0.643366	0
0	0	0	0	1.20492
0	0	0	0	0
0	0	0	0	0.339665
13.48031198	13.05354957	9.529577353	8.994606575	2.01123
1.636932438	1.363652387	1.436131868	1.463419739	0.618162548
0	0	0	0	0.394148
12.49866878	12.59176	15.02848044	15.894612	37.5015
91.13594435	87.58413	64.95156894	66.95764437	100.6761
7.521221029	9.900348746	13.96106041	11.48517853	44.15554847
2.614352302	2.236177164	2.106447537	2.674746634	9.26507
0	0	0	0.435454	3.64046
0	0.739453	0	0.473692	0
247.5026	227.2908	352.6944	324.5499	0
247.5026	227.2908	352.6944	324.5499	564.144
15.6984	10.3073	82.2995	72.5441	40.2597
0	0	0	0	0
0.332763	0.336713	0.162203	0	0.406405
11.08356379	15.80998028	19.24031464	20.53021194	86.63653978
11.08356379	15.80998028	19.24031464	20.53021194	86.63653978
11.08356379	15.80998028	19.24031464	20.53021194	86.63653978
0.0743399	0	1.07189	3.1535	8.41E-05
0	0.114974	0.997697	2.25248	1.55689
0.42781614	0	4.493887	10.71731	8.452244
0.156448311	0.106262091	1.911037	2.178932326	2.41369
0.573867	0.447811	1.28326	0.569208	30.5732
16.8266	13.9132	38.8944	52.5601	72.3895
14.94574122	14.80288174	17.71567415	16.18933433	24.5193
13.8371	15.3374	17.5291	16.5844	49.0582
25.12229	21.73321	32.76038	26.27854	74.229436
12.28458	12.110171	10.50391297	10.524779	13.2056
0	0	0	0	0
13.0949	11.4085	7.462	6.9949	0.415531
0	0	0	0	0
2.79076858	3.345447	8.53276038	7.258747186	2.2763
2.79076858	3.345447	8.53276038	7.258747186	84.7303
6.92374	6.70869	10.269	12.3181	4.5885
14.28802377	19.13153	18.43259	14.942994	118.8505

14.28802377	19.13153	18.43259	14.942994	118.8505
68.07512	61.330537	35.93034	41.37266	56.39441
10.565373	11.2179588	10.584101	14.615626	6.340517528
10.565373	11.2179588	10.584101	14.615626	6.340517528
0	0.0581538	0	0	0.0722188
0	0	0	0	0
37.2468083	34.04781251	23.37903563	20.85864612	5.323379177
37.2468083	34.04781251	23.37903563	20.85864612	5.323379177
57.08745	58.20727	39.84957	38.00074063	8.24567
13.38292	12.13697	8.65802	9.55988	5.085152
30.16472499	31.39594475	17.5216763	18.41705465	4.119375732
0.000383227	0.000130064	7.15E-05	0.000103211	4.39E-05
4.25875	4.54945	8.47307	6.84179	7.06486
55.3070061	57.10719061	42.4075748	44.75262801	25.76870376
6.46492	5.22637	16.95585	14.7414	12.78145
1.086305577	0.408307165	0.528255974	0.548815724	2.308748
1.086305577	0.408307165	0.528255974	0.548815724	2.911441
1.97616943	2.226308549	5.027457	18.90649436	3.53067
0.104426	0.429937	0.417986	0.702218	0.259523
0	0.0971264	0	0	0.111346
0	0	0	0	0
63.9225	52.00385	12.820161	12.171355	0
63.9225	52.00385	12.820161	12.171355	1.06225
0	0	0.728685	1.79411	0.00214508
2.97258	3.46517	1.45575	1.83509	139.343
0.286727	0.0966949	0.650979	0.375854	1.00755
8.316186	7.671508	10.1545615	9.487274066	10.521049
0	0	4.31024	9.36153	29.62668
0.11246811	0.028112769	0.938385469	4.09317789	29.62668
4.888606339	5.699884081	7.596601705	6.119371337	15.22335006
0.190297609	0.097493926	1.110887588	4.66304	35.72880387
11.26442	14.68374	21.09745	20.45884	42.4051
14.76231252	14.26951786	12.02623646	12.3713032	7.823692377
11.700026	11.525853	11.31694	10.948971	27.206
10.34097	11.2665	6.730583953	8.578773411	3.54412
12.244311	11.48800753	20.7213238	20.44740714	44.27419
11.77819	10.28374	13.26606	13.64676	20.87173851
2.5386	1.741235	6.41777	4.01683	37.24562
22.43715	28.84703	30.33149	27.89275	17.5153
2.80234848	3.198521469	1.15730965	2.07992782	132.908
0.518378	0.698621	0	0.228361	117.921
18.02112	18.220278	21.77957	20.26789	48.8352
26.70518	23.13312854	20.18462506	20.67868873	3.224557
35.06280072	31.75860003	22.23205001	21.08795	3.590023695

0.160140789	0	3.4534915	4.982752	1.839630702
0	0	0	0	0
29.47292371	39.22687562	33.44795593	26.79968031	27.4502
29.47292371	39.22687562	33.44795593	26.79968031	3.951282
0	0	0	0.48499	0
0	0	0.626352	0	7.09E-97
2.89005	3.73359	1.34831	5.97624	2.07E-08
1.55388	0.909273	14.4349	31.7662	93.4862
631.016	483.772	279.5355	266.5209	26.20244
109.610946	87.619434	118.3701296	100.1613102	78.3193122
948.823	851.665	1232.129	908.587	514.237
7.316541214	10.588543	12.81256626	11.43102752	15.3684
10.667476	10.979587	5.062213	4.139396	0.0554698
10.667476	10.979587	5.062213	4.139396	0.504616
15.14362822	13.81240002	12.69132748	12.6086437	5.88637296
83.22998366	68.68161868	39.54760214	42.52511409	17.93442011
18.145138	15.158129	18.140602	10.58820283	3.352711555
0.14527	0	0	0.181338	0
0	0	0	0	0
0	0.120222	0.231274	0	0.137825
1.886691366	0.20832977	1.2687723	1.681719982	0
1.886691366	0.20832977	1.2687723	1.681719982	0.776079
1.886691366	0.20832977	1.2687723	1.681719982	0.776079
0	0	0	0.660013	0
4.61628	4.447012557	7.909619	5.870544	98.3095
20.0361	16.738128	70.8855	73.68862	70.2168
0.231991	0.116328	0.111901	0.149578	2.706
1.07101	0.901727	0.354191	0.3248008	233.4596
164.4135325	174.69504	187.213174	162.443895	103.763
86.27521777	116.6086	84.61840641	72.33811732	70.44043
35.9356	36.957748	30.184586	26.7692	36.446242
12.70184929	14.51231651	15.71042244	14.04091782	49.7033
1.061784396	1.077354679	1.291915	0.549225461	0.195878
2.771809586	2.0188403	5.10841	1.817738857	3.160607728
46.238429	45.67558992	63.17467383	72.58115595	52.23788
70.0745	68.8778	9.74396	13.1217	1.84482
0.367668	0.122066	1.41461	3.34794	1.12502
0	0	0	0	0
3.52659	3.44049	2.61205	1.73337	23.9051
46.58176	42.85275	14.80302917	14.28867	3.16415522
8.1196645	9.429348583	11.18042568	10.98897426	37.72649098
58.69088	72.12305	62.66155	55.30087	98.92139
0.339253	0	0.0133513	3.88907	3.57649
177.6923585	144.7755087	323.7754128	337.013539	194.587

389.27387	356.2844831	989.1687	912.1825301	1534.59
2.583334	0.801745918	30.21202	29.3922	56.4292
52.0606	60.2426	10.34993	15.99666	3.40127
12.69059	5.03376	55.4627	66.7126	53.7292
9.21765	3.768992187	43.3317	42.87108	14.3923
55.8394	64.4656	68.7711	65.5062	126.1603
52.86416	55.46771	37.55987	34.88943881	21.24600116
52.86416	55.46771	37.55987	34.88943881	37.4931
1.761474045	1.277916317	5.552432229	8.768790446	2.0048928
0.286778883	0.05243622	1.9193004	2.837711006	1.7664959
0.661202	0	18.797634	38.1817	51.6533
8.67153	8.213817817	6.526554	8.918660062	7.164314154
2.790348411	3.546689826	7.770026	9.9249171	10.7765
2.790348411	3.546689826	7.770026	9.9249171	15.0745125
5.6067	3.5457748	7.37021	4.36957	15.0745125
133.5735636	129.5993332	95.48701544	111.8484053	56.04528256
133.5735636	129.5993332	95.48701544	111.8484053	56.04528256
25.9418496	25.81188494	34.75950894	33.97745164	17.30567263
25.9418496	25.81188494	34.75950894	33.97745164	17.30567263
373.56077	462.6812	267.9134	192.8563832	15.3701
57.014012	49.109678	31.85463	37.7285	20.56300222
57.014012	49.109678	31.85463	37.7285	20.56300222
28.7732	18.09535	67.9196	69.884	12.53242
1.32592207	1.68737849	0.933179311	1.24598365	0.284507
11.75385905	10.7318543	14.12584363	22.0862503	11.8119
11.75385905	10.7318543	14.12584363	22.0862503	3.470449179
2.17934789	3.369869	10.553241	10.386592	4.025422066
2.045692	1.809129	2.306819	1.86506	46.98017
2.045692	1.809129	2.306819	1.86506	46.98017
2.322271	2.141868	4.62052	5.12632656	46.72409
2.322271	2.141868	4.62052	5.12632656	46.72409
12.66475	13.80092	15.57977	12.81773	46.178
13.865437	12.561799	27.50523	28.31663	1.772776432
6.348743	7.9604906	10.318084	8.55928077	21.33578
22.74086081	25.72081615	27.66720905	27.57600098	11.2621
22.74086081	25.72081615	27.66720905	27.57600098	8.631592974
22.74086081	25.72081615	27.66720905	27.57600098	123.15535
23.296353	27.23554005	3.265071644	3.3847036	1.73327
10.34489	10.62664	21.46029	17.43958	90.7534
23.296353	27.23554005	3.265071644	3.3847036	1.73327
24.19684	22.24802	14.936432	16.072843	26.22338
8.583738	6.261373	7.04642	7.233398	23.29586988
46.0255	42.1328	75.7772	60.2049	123.268
2.022629	2.198367	1.260727	1.008412	64.4895

0	0.105128	1.814392481	5.1998	2.127703843
11.58559	10.145526	13.82892	10.71758	26.38066
4.936672042	5.638149096	7.975419	7.321259091	6.822360895
4.936672042	5.638149096	7.975419	7.321259091	6.822360895
1.045149862	1.470435133	4.685345497	2.718136958	16.39881169
0.75037356	0.700624203	1.187397916	2.13743708	1.209744
0.75037356	0.700624203	1.187397916	2.13743708	1.209744
3.60897	2.80251	5.84223	7.88717	10.83852
0	0	0	0	0
0	0	0	0	0
4.724977	4.477223823	3.739589	3.2041252	2.911441
12.264931	15.017567	18.60392001	17.07066	33.6514
12.264931	15.017567	18.60392001	17.07066	59.0086
1.00342	0.771669	1.26629	1.85533	1.06418
20.66969752	21.88370722	22.36007678	21.010804	20.95775471
30.1411557	28.13462081	32.6627137	35.351152	20.16789
1.778194293	0	3.498153824	5.144755265	5.67168191
29.20616116	33.9833291	38.56926681	42.59144472	63.7947
29.20616116	33.9833291	38.56926681	42.59144472	63.7947
75.985	80.3805	58.4062	58.6024	41.519
173.8927	172.731	226.317	180.25323	175.3989
0.0629631	0	0.572091	1.48029	0.21964
9.89393	13.35778	14.94832	12.78263	9.04249
38.552785	32.392757	28.25273	31.39971	11.24912867
9.294638	8.99171	24.65869	20.35139	6.1917149
9.294638	8.99171	24.65869	20.35139	6.1917149
0.801484	1.28729	10.5549	18.685	10.6377
6.588911	7.32325404	15.69721761	10.225524	4.62098
5.437681817	5.626602826	7.732956231	7.217410766	0
5.437681817	5.626602826	7.732956231	7.217410766	6.121271193
19.49972	16.59945	30.9405	24.00703	10.3278
7.019220732	6.849924003	10.32828722	10.06932237	33.5034
22.4854	21.1141	25.6867	24.2827	57.2459
18.44236	12.68446	17.31835	21.77508	57.2459
57.59800904	58.585027	64.7626752	66.48946118	73.26456521
3.51328	8.11389	6.07731	4.61164	3.16066
7177.9	12121.2	3518.58	2557.09	108.407
16.5863	17.09593	22.57892	22.19676	19.7421
79.39372352	92.06459	34.45480776	35.94944662	3.2042807
79.39372352	92.06459	34.45480776	35.94944662	1.623049329
0.570557	0.344851	3.11386	2.6892	1.23512
0	0	0	0	0
0	0	0.253939	0	0
0	0.149018	0.282267	0	0.70485

0	0	0	0.787488	0
0.171475	0.0872334	0	0	0.102863
0.174569	0.0885876	0.171145	0.344676	0.107867
0	0	0.79728	0	0
70.279	66.7957	213.674	185.823	197.7978
290.7626	270.7026	855.44	936.598	663.425
11.425869	9.625148	7.5833	8.063365518	2.865842381
0.192894387	0.099419373	0.0977937	0	25.88573
1.279667938	1.919524027	0.870175831	1.056015973	243.0311
39.7259	32.2193	70.4808	53.2992	5.94475
11.08582314	8.415188397	26.39144809	44.159723	98.71295003
11.08582314	8.415188397	26.39144809	44.159723	98.71295003
1.8924	2.39524	2.78739	3.63699	94.9609
11.014523	12.2828324	29.40247544	31.5577	94.9609
11.014523	12.2828324	29.40247544	31.5577	94.9609
0	0	0	0	0
0	0	0	0	0
18.97175	17.2409665	13.456667	16.643025	29.219543
9.85148	8.06393	7.33342	7.33069	8.57464
0	0	0	0	0
44.47140137	51.7994	47.8736	45.62552297	116.044
9.12896	8.879024	13.3362	12.38466	46.68255
9.12896	8.879024	13.3362	12.38466	46.68255
0	2.26E-29	0	0	1.5212
59.458	58.7368	123.286	115.33	118.563
1.94644788	2.622791922	4.562978	2.152862219	3.825855279
0.049458	0	1.48862377	2.60372	2.441094
0	0	0	0	0
0.257158599	0.123627291	2.347100867	3.905721377	0.427073
1.822692	0.343332667	36.4935	77.2094	69.5604
2.350422	2.603722	4.646772	5.902081	14.70253
62.6475	64.0294	78.1681	70.8892	118.7495
68.023736	51.40574	50.821682	55.775847	1.97457
0	0	0.643870779	1.99032762	0.757638936
8.27704	10.29105	11.63053	10.51764	2.8191531
124.543367	99.14655	47.65119	56.53956	1.66868
124.543367	99.14655	47.65119	56.53956	52.8573
1.5704216	2.26690025	1.882104	1.698588	0.566771
11.83466	11.91094	17.202799	13.7571	14.33188
121.6393767	127.8715425	159.2003372	140.0715843	82.7337
121.6393767	127.8715425	159.2003372	140.0715843	2.885914139
5.075818	4.30679532	5.137687168	4.891217017	7.79471
1.87E-05	0.185606	1.60E-06	3.20E-05	0
22.100871	25.15743	28.044571	22.974771	65.62663

0	0	0.410851	0	0
0	0.0738714	0	0.190042	0
0	0	0	0.253009	0
0	0	0	0	0
0.491597	0.335537	0.161829	0.658777	0
32.2779	20.7445	28.0393	21.9442	5.82589
0	0	0	0	0
0.0687963	0	0.268832	0	0
0.0759194	0	0	0	0
0	0.0842591	0	0.109827	0
1.804593069	3.024893397	2.207547081	1.7675243	0.807990333
0.262184403	0.731038896	0.359149103	0.9300549	0
0.262184403	0.731038896	0.359149103	0.9300549	0.540533
9.779959	9.084833337	10.17158001	9.835858815	47.37332
13.62783992	14.307267	22.05324153	18.93952325	15.14209
13.62783992	14.307267	22.05324153	18.93952325	15.14209
47.5925	47.2356	75.0094	63.5351	179.3552
0	0.169347	0.0825445	0.110208	0.100815
0	0	0.256859	0.11549	0
0.159311	0	0.158105	0.105289	0.0959013
0.046522454	0	1.30230902	3.397644	0.851597053
17.785402	15.978123	5.258984	8.070481616	2.126081351
93.6266	76.5185	198.5504	161.466	75.1605
0	0	1.20E-06	0.941465	0.88086
0	0	0	0	0
0	0	0	0	0.104192
0.415762	0.212592	2.57045	8.66634	4.70016
0.245107	0	2.58656	6.74949	5.13985
53.3627058	49.99114	44.1615069	42.63129889	12.5504
53.3627058	49.99114	44.1615069	42.63129889	39.6979
31.329732	27.633005	31.245922	25.38961786	0.470417
31.329732	27.633005	31.245922	25.38961786	1.63073
31.329732	27.633005	31.245922	25.38961786	32.90121383
18.40020116	17.68282416	24.051541	31.91414884	26.50786001
18.40020116	17.68282416	24.051541	31.91414884	26.50786001
2.75274	1.74511	4.33105	3.16985	39.22198
45.10944	42.281697	34.213429	35.17141227	6.74239
0	0	0	0	0
23.99544	24.1269	27.27816	27.86524	43.5964562
4.663276	3.791947	1.743105	2.101176	267.7313
17.89616074	18.38183677	18.83776489	18.90142625	24.494038
17.89616074	18.38183677	18.83776489	18.90142625	24.494038
11.86789508	10.72974114	13.64040699	12.12877559	42.6911
9.74E-05	0.0115276	6.82E-09	0.00141975	8.80818

35.59715	35.14808	42.13242	43.03143	59.8685
8.78033	7.63314	9.00037	10.8992	65.9639
23.226175	23.64361	21.894601	19.793808	14.99509
23.226175	23.64361	21.894601	19.793808	14.99509
5.437061183	5.803731089	10.08397003	8.447015883	35.03455
73.2663	36.8691	92.5616	56.5815	17.2634
73.2663	36.8691	92.5616	56.5815	1.64557
2.616667	1.434090002	3.022675	0.940337718	0.485574
0.2094	0	0.202027	0.273091	0.492274
9.820142	6.784292	13.63785	11.324742	6.422306481
42.289823	40.874001	46.3350212	54.18838005	70.38679
42.289823	40.874001	46.3350212	54.18838005	70.38679
0.889987473	1.868559626	0.355971081	0.503990915	102.1876
0.889987473	1.868559626	0.355971081	0.503990915	102.1876
2.49599956	1.618689466	6.80932	10.11072	56.18749
23.63444687	24.492417	22.53557239	24.52314236	23.68177527
8.55038	13.44549	1.757892	1.762453	1785.907
9.69381033	10.30302843	15.2954734	13.27206388	4.640207
26.24833	26.32065	20.98836443	23.4855976	18.72565048
82.341018	63.06664	24.3775	33.00638	1.53454
80.60595701	79.75181001	38.64372951	40.99871705	33.64305
80.60595701	79.75181001	38.64372951	40.99871705	10.07879
75.78589978	70.40944527	68.35026843	69.15277835	23.31532711
60.7579622	65.8142583	65.6688881	68.600423	82.79007
1.25297	1.010623003	1.55654458	1.219185086	39.07041
10.72723	10.59793	9.029	10.17421	2.699615
172.1134	154.6431	196.0149	170.9794	117.2749
5.714607	6.300248	8.35193078	8.34985	12.5267
0.60144	0.468648	0.261032	0.259985	111.18
84.75888755	87.38953136	139.8857782	128.2839015	222.796
84.75888755	87.38953136	139.8857782	128.2839015	18.25871699
0	0	0	0	0
6.87072	6.66007	3.9496	6.03094	0
6.87072	6.66007	3.9496	6.03094	0
29.500825	32.62827	24.0469	27.4795	92.0996298
9.583126	7.00355	13.68323	12.26795	63.6157
1.891831968	1.9633449	2.200133759	2.273618	11.09327
14.200537	18.956893	19.462864	24.427084	9.54854
1.634540362	1.555324507	2.732174795	2.143665072	1.80168
0	0.303846	0	0	0
365.541252	282.96972	179.8075607	190.9101472	11.24580293
9.24E-13	0.658024	0.177809755	1.05E-78	3.23E-05
9.24E-13	0.658024	0.177809755	1.05E-78	3.23E-05
3.309409	3.533481427	5.794358157	6.439196103	5.43282

0	0	0	0	0.256179
0.293316338	0.596239	4.020181	7.31628	3.270743
0	0	0	0	0
23.9664	19.1698	2.21342	5.79992	0.435539
7.06751872	14.65613833	22.22159321	34.38648064	2.36304
7.06751872	14.65613833	22.22159321	34.38648064	139.4304
1.734287	1.149216	2.646253936	1.951026036	0.444016
24.4321089	25.705746	47.806123	32.75564	78.9054
21.31800001	15.5548	8.20656	10.9729	0.557741
1.560610328	2.422487853	2.930140647	3.637319333	39.91872
51.70141867	58.08716621	49.58022501	53.1843177	44.04747978
51.70141867	58.08716621	49.58022501	53.1843177	44.04747978
51.70141867	58.08716621	49.58022501	53.1843177	44.04747978
0.627526	0.510138	5.29237	7.10892	3.821517
0	0.685984	0	0	5.80035
0	0	0	0	0
2.377766863	2.316231412	0.9450647	1.5060608	128.2687
0.770356052	0.78681183	1.778432282	2.511631226	41.4715
57.326413	53.589503	63.765791	59.68858369	21.717908
57.326413	53.589503	63.765791	59.68858369	19.99182
33.72980127	31.97810114	26.39890086	29.72990096	10.18660048
15.2422	18.263	12.9115	17.1513	7.13615
4.000797	4.49306	4.465560005	3.824570302	1.344531559
16.29372739	18.84200341	20.52502872	18.56001587	42.91966
19.38059	26.07047	28.07478	20.74619	11.82111
7.07309	10.42291	12.19035	10.85115	34.18584872
10.6878	12.9467	1.62612	1.19187	2.23555
0	0.109915	0.102277	0	0
5.172424	5.84673	5.685280025	8.39601	25.84343
5.172424	5.84673	5.685280025	8.39601	25.84343
22.35133	21.95942	14.52632	13.083518	8.34307
113.92428	113.4372	78.99766	83.08635	78.525102
13.6336	13.2856	9.71529	9.14981	3.239198
20.5683	23.6029	59.1914	56.0207	55.8866
0.253854	0	0	0	0
3.167210216	2.903816	5.199132	6.25281	60.25798
3.167210216	2.903816	5.199132	6.25281	60.25798
1.2281313	2.644113035	0.800486894	1.433874592	354.3971116
49.2297	50.73110001	38.00320126	37.72140197	21.00918
49.2297	50.73110001	38.00320126	37.72140197	51.47941837
32.07492	28.77471002	26.286078	19.02546	5.298666663
32.07492	28.77471002	26.286078	19.02546	5.298666663
89.51527	88.8355	86.94432	81.77139559	18.01933
31.4503	30.1138	54.2707	42.0136	40.1518

184.20635	171.7084832	166.4815027	134.5405664	71.6498
39.9834956	45.2562227	66.00397	67.37460994	91.97886
14.4459	10.3473	18.0688	25.5964	3.59744
19.76363	22.34787	19.045394	19.18902	82.41032
52.89947794	56.28030534	79.52481325	75.89459844	82.41032
36.9977	34.5154	44.9573	57.4765	16.6767
425.33844	452.71043	571.569643	581.03769	674.9238
0	0	7.32084	0	3.00E-15
33.12227	42.1173	49.9079	48.0819	113.2666068
5.01228	3.61476	0.55538339	2.17371	0
24.62041573	22.9962974	23.52441255	20.99562044	28.05493291
13.92948	14.62298	18.15111	17.1729	29.1244
22.87626089	21.19876663	19.32016427	20.42217601	3.34032
22.87626089	21.19876663	19.32016427	20.42217601	6.558933
481.0531	481.603187	454.2255	504.08549	549.9662744
3.027948892	2.313717	5.458451777	10.54921159	22.9478884
93.12003	93.154336	59.89434	55.99539	0.000973597
93.12003	93.154336	59.89434	55.99539	4.7962
15.383629	25.97205	26.03516003	28.91078	21.85551
15.383629	25.97205	26.03516003	28.91078	21.85551
31.456412	33.5232239	39.23673461	37.74061698	31.61696
31.456412	33.5232239	39.23673461	37.74061698	31.61696
16.6571	18.4118	27.299	22.0236	57.5033
0.0789184	0.338237	0.753053182	1.290007081	1.221111191
57.72140823	57.66846057	45.61053325	48.47833941	23.01940203
0.0951222	0	2.927443003	8.48503	3.805158288
0.0951222	0	2.927443003	8.48503	3.805158288
45.42788	36.394	30.51051	37.69752	33.41107
45.42788	36.394	30.51051	37.69752	33.41107
38.323607	41.06552	59.92446191	97.955185	68.1066
22.09935	22.99077	19.6794	17.96377	46.3433
3.168839	2.74805	4.478042706	6.84408	119.97599
37.80197	33.76801	44.85447	45.956	44.4136
25.01635	24.23898	29.08329	22.82083	3.46746
25.01635	24.23898	29.08329	22.82083	0.295774
25.01635	24.23898	29.08329	22.82083	3.82108
0.595717	0	0.866279	0.786316	0.723042
25.417619	27.132591	23.000045	21.372318	24.11937
32.7319	27.1802	76.615	55.2558	19.58607
0	0	0	0	0
0	0	0	0	0
17.92819	17.78458	16.8689	15.522441	23.4282
0	0	0	0	0
1.04471	0.425647	0.410779	0	0

0	0	0.406804	0	0
0	0	0	0	0
0	0	0	0	0
0.586394	0.20042	0.195313	0	0
0	0	0	0	0
0.905123	0	0.853522	0	0
0	0	0	0	0
0.196313	0	0.187559	0	0
0	0	0	0	1.39657
0.702621	0	0.674074	0.915362	0.202541
0.371623	0.103083	1.079447	0.40754	0
7.0968	5.29253	10.47799	8.82674	19.0986
11.5141	10.6558	13.4826	16.7165	38.7014
136.94768	127.062	229.54348	217.85408	190.121
15.4792	5.2843	5.29935	11.9287	0.990619
0.428220729	0.271248333	1.903998796	2.189270362	30.090206
18.38251	18.25746	22.32337	21.645508	29.1219
8.36931	7.594625	12.3386	10.5688	44.308
13.4345639	13.57317925	10.45883344	11.72777547	11.66545154
93.9117	89.4667	36.25297	47.26315	17.00354
0	0	0	0.494002	0
0.469482607	0.698188871	0.226849023	0.3486938	170.335
31.0706	20.954	8.34288	12.4805	61.4443
0	0	0	0	0
18.19142	16.8165	40.32175	53.0424	17.3592
2550.8	2120.21	1298.92	1355.92	35.6981
1.24365	0.312965	2.324	2.41681	1.78859
0.163976	0.494569	0.392538	0.322336	0.0991211
10.69395	13.78871	17.17801	15.33898	17.3691
2.2746	2.24633	1.82184	2.88182	2.369845
6.672456399	5.473593817	9.4179412	7.263184	5.698830485
9.10489	10.8868	11.0815	12.1894	25.09889
2.7999359	3.7372415	5.028704	6.285141	29.682491
149.6642804	144.7377011	172.5696687	241.1551703	79.1913
149.6642804	144.7377011	172.5696687	241.1551703	31.69464
149.6642804	144.7377011	172.5696687	241.1551703	31.69464
149.6642804	144.7377011	172.5696687	241.1551703	6.01911
911.435	679.297	651.679	812.925	24.3406
3970.723902	3391.497102	12525.8124	8630.962201	47.5805
3970.723902	3391.497102	12525.8124	8630.962201	1409.738
7.37019	12.3526	31.4077	27.6928	5.7387
1.48007	0.372076	2.7372	3.79404	37.263
33.4275	28.7995	42.9631	34.8529	55.5929
37.84940383	41.8929048	57.93280196	50.05050368	155.0784131

27.83566	45.2196	38.64588	25.0456	7.614562
21.033743	29.767687	23.861241	25.553959	34.13198
9.68313	5.43995	18.7663	16.7183	3.13464
15.50325188	16.97596834	17.71558522	15.23411175	44.38722
15.50325188	16.97596834	17.71558522	15.23411175	44.38722
0	0	0	0.732259	0
0	0.0794857	0	0	0
10.4562	9.24256	4.67028	3.71173	0
34.19339	37.94969	38.58575	34.37947	30.91975372
0.659164418	1.105618279	0.45826605	0.441093148	89.78745
1.63918	1.62108	2.86858	3.24543	7.69578
1.92025	0.84815	0.000128486	0.00170141	1.52E-05
8.2537	5.46979	7.45989	7.37856	0.336919
0.128422	0.130215	0.495973	0.832076	0.300361
0	0.317243	0	0	0
0	0.414047	0	0	0
10.852493	10.57657804	13.23584874	9.78093052	26.30612286
10.852493	10.57657804	13.23584874	9.78093052	26.30612286
10.4704873	9.292504312	5.759826708	7.799492166	9.967653
963.696	1071.563	52.72877	118.3676	9.967653
68.05904	46.63396	46.40412	47.35008	27.0947
17.11691888	23.50733497	18.57763991	15.08850849	5.472256605
0	0	0	0	0
30.66881	26.03005	34.756519	33.92242	15.78344
0.764563041	0.757662319	0.734489356	0.764997734	0
1.925511	1.965701635	5.201178	3.85509	3.112714
6.780244418	9.215963601	3.10949848	3.752502964	0.340105
5.693339	5.267396	5.40077	3.441588695	0.210638
0	0	0	0	0
0	0	0	0	0
42.6559	39.0554	28.71619	31.37452	7.975603
1.39339	1.046862644	3.370261	5.23063	10.4845
45.547419	54.90031384	31.61056151	40.96525614	1065.855
0	0	0	0	0
9.40739636	10.74890292	6.801257959	7.739758	182.888509
9.40739636	10.74890292	6.801257959	7.739758	182.888509
8.824306	8.042474147	5.254625981	7.1279204	2.026624991
2.33926	1.992648	1.784934914	1.497781971	0.0981413
2.33926	1.992648	1.784934914	1.497781971	0
18.90162832	19.04554402	18.75243268	20.591779	38.56518
0	0	0	0	0
15.574238	13.419669	15.406281	18.900645	13.07108
0	0	3.19717	4.83483	9.49129
54.06354	54.62409	8.29682132	15.00826878	1.14544

35.0531	30.1783	6.19109	5.74262	2.29997
2.185296568	1.760644	5.06769902	3.995449002	0.981038376
3.0759779	1.018474682	1.077972949	3.432052	12.64268617
3.0759779	1.018474682	1.077972949	3.432052	12.64268617
29.97808	31.83143	24.59859	27.3002153	32.5267
0	0	0	0.282409	0.257524
583.77887	309.18331	203.40806	248.14379	1.23686
2.15493	2.39729	1.2311	6.89E-05	0.134565
0	0	0	0	0
0.263665	0	0	0	0
24.5645	23.12348	5.83483	7.96834	2.28129
3.909916	4.133594	7.35823	11.23933	18.76937
77.32063895	87.93123568	59.7366914	64.80391025	42.152108
10.236354	9.781368	15.456987	14.99634125	14.508983
0	0	1.98294	1.88114	2.90107
4.9677	4.1289	20.35983	17.06852	567.451248
0	0.0502612	0.0162178	0.0876727	0.019749
0	0	0	0.145493	0.134089
0	0.18733	0.0893684	0.181207	0.162843
6.61159	8.35433	13.03659	8.55973	48.321869
0	0.501468	0.925929	0	1.165
6.61159	8.35433	13.03659	8.55973	48.321869
6.61165439	6.31402	13.06693	12.76872	55.5067
0.0637493	0.084120543	0.862312866	0.410054398	0
0	0	0	0	0
3.3738	4.15997	5.16492	4.8849	12.52406
76.75461493	95.47268203	80.78136503	71.75008297	163.3228
9.47415	9.09458	13.32968	19.10281	42.12236875
245.7504547	168.9376723	84.27606071	100.6514849	6.032027
35.865579	36.6653312	88.553662	84.615257	18.8526332
2.70229	2.55426	0.720442	3.43911	7.70E-06
3.073815902	3.304167388	3.327674541	1.936599753	0.42412
1.50E-05	0.217478	0.00155217	3.25E-05	0
13.18520151	10.60880255	8.82367214	10.02640421	2.90220076
10.67321379	2.736977159	20.11415167	5.574779738	9.21521497
5.51313	3.58146	3.909302779	4.69396	2.479197272
0	0	0.615771	2.10977	0
0	0	3.08697	5.33395	3.388359
2.45734	2.53967	1.721	2.35693	3.12222
7.47712	8.00994	6.76169	7.71385	10.0427
73.32556447	76.67100461	56.42147864	64.22227291	86.25497
7.51289	7.48805	13.5005	13.0284	18.8193
51.7942	61.6943	53.0503	53.7835	188.0615
0	0	0.878813	0	0

8.48215	9.65422	7.46757	6.14291	1.88221
8.87191	8.74983	5.35671	6.4924	4.96874
4.820969	3.921836	4.01269	3.212575	2.09443
0.321436	0.161233	0.312029	0.211443	1.20694
0	0	0	0	0
0.451773998	0	0	0	0
0	0	0	0	114.625
1.21656	1.54776	2.86741	3.51555	0.393179
0	0	0	0	0
0	0.390332	0	0	0
0.0371265	0.15128	0.329051	0.201573	0
0.374145	0.281777	0.182347	0.249282	0.120796
552.479	482.274	104.151	113.203	5.2811
3.80E-05	1.64E-09	1.69E-09	0.298337	0.706805
2.44705	2.66702	3.96163	4.42983	4.17501
325.3663111	390.2256084	246.5847469	243.43273	533.829
77.22449	79.04063146	67.7317	83.31651456	75.76378
0	0	0	0	0
86.40233	93.99393	45.59590038	43.1343	6.56103
21.8323	22.2785	21.0181	19.8872	6.3368023
0.301880995	0.223070451	0.0546315	0.293749	70.0567
20.190316	20.65227268	16.48681	17.43433	17.62636705
12.1273	11.1414	20.4294	14.7629	64.7525
20.18291	20.65523	15.08534	16.45613899	6.883758
232.3912	227.5351	251.5204	198.8657	131.042
37.18173	36.351483	27.385622	27.89238	6.467021
0.655449005	0.37391	0.126199	0	0
2.775886435	3.305283	5.095560856	6.353468	1.9908662
0.773302437	1.95331864	2.528120228	5.767013005	0.916551564
0.773302437	1.95331864	2.528120228	5.767013005	0.916551564
0	0	0.903863	0.365241	0.109879
4.281016228	4.543861079	6.083245181	6.682328158	3.48773
4.281016228	4.543861079	6.083245181	6.682328158	1.12212
2.483867553	3.189155821	10.8454663	8.022033172	7.73638
0	0	0.748528	1.890321517	1.093491602
0	0	1.024394953	2.14049595	0.992662
0	0	1.024394953	2.14049595	0.7414
0	0	2.114472408	5.110268	2.656103129
0.264914403	0	1.891775	2.234704662	1.3367912
16.20209868	17.10459602	16.807452	24.327077	31.7512364
16.20209868	17.10459602	16.807452	24.327077	31.7512364
0	0.222758	0.290852	0.292994	0.696597
0.547405802	0.993035506	0.703569324	0.617538341	1.6477
0.547405802	0.993035506	0.703569324	0.617538341	41.826179

0.692101364	1.009169875	0.0364402	0.44600356	81.04434
0.051737423	0.051416481	1.071389326	2.022597845	2.31261
0	0	0.183892	0.371873	0.218671
0.042871768	0	0.677964945	1.71544829	0.607472
0.167378	0.34021	0.575857	0.329728	0.298125
0	0	0.206608	0.280992	32.21905
1.554583	2.818142	0.656499	0.498117	300.5794
10.89826	15.18392	10.19218	11.46895	36.4106
6.93924	6.09845	12.20281	8.606475	17.110735
8.97781	6.9639	15.61815	10.17799	7.99836
40.83639	41.181078	33.663709	29.25676	49.81778
17.85312	17.331857	30.9805	19.14157	80.4671
14.9491	14.9086	19.5224	15.2628	22.62297
60.54068	61.0235	64.44272	66.8965	99.35658193
73.054	66.86683	50.53255	54.45663	187.4763801
2.224696	2.372717	1.94215	1.19862	1.73675
69.19422	66.856036	53.33088	57.554677	43.1043
19.37961	22.19855	16.91915	18.1419	11.916652
19.37961	22.19855	16.91915	18.1419	11.916652
19.37961	22.19855	16.91915	18.1419	11.916652
37.85302	31.38356	37.74744	33.2511	39.65504
37.85302	31.38356	37.74744	33.2511	9.04446
689.387	903.914	2520.45	1952.01	35.9626
125.654	128.594	31.0313	38.2897	6.44052
0.454081	2.52257	2.19851	2.96426	189.30179
7.080696	37.92811	23.9008	29.06416	69.2457
64.767486	81.25406479	44.93148119	50.76785	35.70537
53.80145	36.06469	16.88290499	17.08901933	0.589958
1.57404	0	13.4375	3.85785	6.13395
14.50243569	13.78446934	11.06988758	9.478108881	4.390599172
14.318716	14.93256373	12.68131895	14.288797	28.568511
4.676490424	6.82123497	5.328928025	5.8009683	431.5523
7.235626	5.825892867	8.386260018	9.447956621	12.25244
7.235626	5.825892867	8.386260018	9.447956621	1.69571
0.955315	0.323505	0.303231	4.18232	50.44037
73.28072095	74.76732015	41.65989752	48.14275968	50.44037
36.56938	41.18687	30.40404	26.12	33.4476
18.93684	13.67445	23.52814	21.5017	21.3175
34.37036	37.48412	50.19847	53.61627	49.41617
132.8046248	135.3778935	115.1636239	99.1762592	31.96257
0.71847	0.660531	0.358308	1.05327	0.366056
4.40678588	2.909219	7.694681	4.236112338	3.38143785
18.52144	20.51993	20.41747	19.64712	17.35767
10.78878	10.09803	14.50946	14.39279	34.89796

39.85086415	41.6521053	30.86375572	36.29309481	52.23
45.9751	51.7844	50.3559	43.181	77.4015
27.17770019	30.45008	19.05806001	19.07167	65.2712971
0.476729147	0.302385818	0.836356069	2.255801	2.44673
57.8281	65.4012	78.5694	65.7225	135.476
0.393878584	0.703404437	0.99909732	3.376064313	26.0955103
13.333934	15.39982512	24.05530884	20.54275	10.675555
11.25590677	10.17671118	8.885644108	9.04862162	3.179031952
11.25590677	10.17671118	8.885644108	9.04862162	3.179031952
194.6044426	179.6313007	222.0606697	252.2150003	194.918
194.6098	199.6533	206.72	143.4908	161.0248
38.13382	39.33604	41.738351	41.86745	28.98215615
0	0	0	0	0
0.132205	0.0442498	0.043791	0	0
0	0	0	0	0
1.197068453	0.638451668	0.5545431	0.796436057	0.669529
1.877110706	1.5173192	1.509253	2.235788269	58.96804379
0	0	0	0	0
0	0.516198	0	0	5.71407
0.234784	0.189849	0.138596	0.123689	0.226041
0	0	0	0.437384	0
0.0649041	0.263352	0.19294	0	0.0769277
0.0826489	0.0820679	0.0776467	0.524544	0
2.40041	3.26932	4.57568	7.94316	0
0	0	0	0	0
0	0	0	0.173454	0
0	0	0	0	0
0	0	0	0	0
0.896116	1.182	2.32337	2.9648	1.90255
0	0	0	0	0
0	0	0.0345877	0	0
0	0.11828	0	0	0
0	0.0493525	0.094894	0.128527	0.117363
0.834799	0.743929	0.720549	0.555876	0.36879
0	0	0.453311	0	0
9.12097	6.31446	7.21181	13.5631	35.2963
0.0369604	0	0.109401	0.0976621	0
0	0	0	0	0
105.76	48.3946	553.825	276.721	15.7183
0	0	0.707316	1.068863966	0.454716
25.54046	33.22823	29.26238	31.12912	9.697199
5.595821	5.445847	8.644535	8.141039426	35.54320754
0.307262448	0.355435331	2.088702503	3.569186146	2.24466
16.3194	18.2621	88.739	104.81	302.042

73.9705	99.3258	147.26	140.664	266.543
9.93713	11.85748	14.19097	11.41427	28.8824021
0	0	1.102592313	2.84571846	26.59037
47.940378	49.07662838	47.01502056	48.42807703	79.5041
47.940378	49.07662838	47.01502056	48.42807703	79.5041
47.940378	49.07662838	47.01502056	48.42807703	70.8221
154.4659	184.9935	183.0038	143.1932	22.1074
3.9458147	4.826251327	7.289354435	6.7113674	38.67531107
66.3632	68.9423	80.7757	75.4841	64.31743137
24.5955	25.355	24.0725	25.9062	31.4273
3.5112444	3.8805704	3.5424583	5.3430551	132.0294
0.0359092	0	0.457488	1.84048	1.31111
189.943	104.722	68.1994	129.904	33.3638
11.762	15.7771	18.4933	19.676	25.4802
24.11204111	22.45245	23.06259	24.3363927	33.3608
73.0296	90.4934	75.5791	66.9316	245.81
19.45489354	17.71394765	17.25555747	16.85773797	16.60948
6.84343	9.43549	9.84056	9.4834	47.44063
0.209155087	0.1546398	0.068491584	0	16.34344828
50.842038	45.41676	55.27438972	68.61136501	96.1236
50.842038	45.41676	55.27438972	68.61136501	69.90132001
50.842038	45.41676	55.27438972	68.61136501	69.90132001
1.107284	1.206757	1.214241	2.119576	17.1959
28.17419	26.33519	26.20936	22.26976	42.5749
1.107284	1.206757	1.214241	2.119576	9.93259
45.86446785	43.5305744	41.73162518	43.01591673	57.17897663
45.86446785	43.5305744	41.73162518	43.01591673	57.17897663
2.64814	1.543919742	1.9915345	2.424291902	70.07489
4.968535295	5.244427678	9.064979	8.21322207	48.45779
0.564660328	0.681325082	1.986189185	3.561370973	66.339
9.60134	8.80205	10.49328	8.74632	22.8893
0	0	0	0	0
0.878663	0.7931403	1.80156	4.90916	22.03
16.47745	20.00058	14.71274	13.400449	22.03
0	0	0	0	0
0	0	0	0	0
0	0	0.122547	0	0
18.1966	17.277801	18.0424	14.327638	23.95509
9.445154	10.22295	11.90244	9.859444	11.29508
5.8725	5.08554	6.59475	4.72485	0.936291
43.1584163	41.6424	38.7648	38.077339	71.04772625
3.47028	3.49433	4.27276	5.90919	82.9989
6.48534	6.70683	7.55744	6.10611	14.82578
3.963776	3.5517873	6.456066	5.330982	17.165

0	0	0	0	0
5.65728	4.876	9.7563	7.41923	41.49900023
24.0533	20.8852	113.748	89.7808	20.967
0	0	0	0.366488	2.092890205
19.69252	22.126	97.71194	66.01137	25.03544
0	0	0	0	0
3365.7141	2789.5425	664.18687	769.64494	68.5217
29.65816973	31.18221742	42.95179	38.85228149	13.34157681
2.38721	1.68468	4.52	3.01786	1.25857
0.410551	0.311504	0.301242	0.267003	0.487646
0.683609	0.769619	1.56975	2.5062	1.39742
0.182481	0.278692	0.356247	0.840858	0.672958
20.974354	19.2622	26.086951	21.735883	103.79794
18.48203773	19.21587862	17.64306699	15.67022251	31.2533
27.25995	23.90079	29.66911	25.12522	19.55248407
126.8068745	143.16671	229.043634	175.979523	36.5888
126.8068745	143.16671	229.043634	175.979523	10.53079108
126.8068745	143.16671	229.043634	175.979523	10.53079108
0	0.230663	0.333856	0.754955	0.129017
9.31E-07	1.33E-06	1.73334416	5.001361045	4.633241
0.233224063	0.13771658	1.636470977	3.799042	6.055772
0.233224063	0.13771658	1.636470977	3.799042	6.055772
0.233224063	0.13771658	1.636470977	3.799042	0
6.66864	6.85399	3.63898	4.995126	2.292395
2.778627612	4.089033	2.459770978	4.397624	1.16671
128.3493032	78.46986739	546.8175	560.5003465	1021.608
5.90863	5.04652	5.97781	5.22816	11.617
15.55205386	18.13762147	22.47475388	20.13766894	45.7021
40.60862156	36.12835868	29.91951431	31.03116803	8.344070109
9.41705	11.68352	12.25243	11.45656	26.6672
30.06369	31.525406	42.66068987	33.95139974	33.63943
30.06369	31.525406	42.66068987	33.95139974	5.45731
30.06369	31.525406	42.66068987	33.95139974	80.5945
0	0.0521595	0	0.136789	0
3.03E-06	0.97839	4.28E-06	1.37E-05	6.03E-07
0	0	0	0	0
0	0	0	0	0
0	0	0	0	0
18.32595	24.4479	46.3395	46.8239	9.04255
0	0	0	0	0
0	0.0581093	0.018854	0.0509521	0
0	0	0	0	0.155847
0	0	0	0	0.0706131
0.283438	0	1.85464	3.02126	1.37657

7.45E-07	5.21E-05	0.0624989	0.338781	5.70E-06
0	2.61E-05	0.19666	1.31474	11.4264
0.116225317	0.477108458	1.9788811	1.853616	0.558596
0.175048	0	2.2796774	8.2967269	3.4543377
0	0	0	0	0
0	0	0	0.142714	1.939612
0	0	0	0	0
0	0	3.33072	8.27333	6.43811
0	0	0	0	0
0	0	0	0	0
0	0	0	0	0
0	0	0	0	0
0	0	0	0	0
2666.064	1689.374	976.348	1733.899	28.7663
2666.064	1689.374	976.348	1733.899	12.8754
2666.064	1689.374	976.348	1733.899	4.45314
50.299	39.9498	45.0961	74.1752	1.8745
14.2011	6.1078	17.4466	35.5599	3.13777
8.19616	10.1936	12.5166	10.5066	64.1903
0	1.0337	0.922463	1.29917	0
0.228432	0.604467	1.03389	0.785947	0
0.757796	1.49766	0	0.480991	0
0	0	0	0	0
0.1073	0.345077	0.763114	0.534408	0
108.15366	75.83487	44.42919	57.06862	1.61E-05
108.15366	75.83487	44.42919	57.06862	12.2849
1.99928	2.14399	1.71608	1.25356	3.33562
6.45656	7.61656	9.17045	10.23982	35.4735
9.49849	10.9072	13.6075	10.3926	22.5188
625.461832	677.2436529	1065.594716	1003.133486	890.0088849
10.713193	8.848084	21.85743	22.058168	9.77814
0.766726302	1.083616988	0.393970979	0.426292177	168.9545
3.98597	3.752667	9.53508	10.40596	99.9033
32.8031	34.608	155.326	130.283	43.2611
41.78633	55.74563	53.17124	42.5095	114.8724
4.016658	2.835717843	1.166576102	3.493699437	2.30052
4.016658	2.835717843	1.166576102	3.493699437	2.30052
4.016658	2.835717843	1.166576102	3.493699437	3.57917
8.803196	9.590261	16.21324	11.450249	20.4782
22.374542	21.39226	14.33336789	14.89640328	10.13674346
22.374542	21.39226	14.33336789	14.89640328	10.13674346
0.105711	0.211468	0.3079	0.411315	0.128476
0.112607	0.112668	0.107191	0.286536	0.665521
9.30294	8.24687	8.52695	10.6458	2.49753

81.31753201	83.30034126	91.63647405	80.28793476	83.86901
19.51531	22.68631	31.75463	25.53473	41.8255
9.711433	9.110605168	5.013791783	5.675553275	1.557530678
6.164787	7.17449	11.54651	15.725936	31.21219
6.164787	7.17449	11.54651	15.725936	31.21219
34.52505103	34.00472885	41.27120033	45.093412	89.4335
9.26522	13.4395	22.7737	23.7118	10.9337
5.95368	5.82884	6.81636	6.46675	8.82734
16.073215	14.11988	15.18746	17.18126	12.67734
17.07039	15.28096	24.4923	16.99941	61.10310006
17.07039	15.28096	24.4923	16.99941	61.10310006
6.96616	5.39422	11.0464	5.88594	26.3793
3.73714	2.018529015	2.006754	3.689996	1.66047
3.73714	2.018529015	2.006754	3.689996	2.2855
0	0	0	0	0
0	0.0703337	1.37274	2.83466	2.93567
0.277834	0	6.88374	13.148	15.9246
2.20685	1.47966	1.97416	2.61849	0.809353
0.0399259	0.137044816	0.265735	0.213259	15.1578
0.673992	0.614205	1.8729	0.883881	0.743842
0.354128	0	0.502544	0.670964	6.84766
0	0.557695	0	0	1.30272
6.14047172	5.584059057	12.17461838	14.00603167	9.45604
0	0	0	0	0
0.206498	0.103016	0.166263	0	0.529813
0	0.133723	0.39584	0	0
36.9061	23.6823	5.30199	6.88676	0.511079
5.25449	4.40077	0.446204	0.61207	0.185727
37.62957	30.38032	10.44543	9.41761	0.869256
19.80973	25.14642	37.64657	29.59355	199.6976
64.992983	62.205346	53.20530047	52.23600047	23.00620019
64.992983	62.205346	53.20530047	52.23600047	23.00620019
1.235577	0.489216	9.63033	9.96123	1.78482
21.14863978	20.490321	22.66133	22.80245064	32.94993755
116.1429001	111.3916253	61.40236	72.01917	106.9049454
0	0.30468	1.14283	1.16438	1.00518
0	0	0.759663	4.6835	2.97355
110.8975163	87.80274	28.353982	32.306787	8.45003
9.08869	8.65982	6.244291028	12.05620071	218.48355
386.585	126.791	1374.13	782.49	0
1.512805491	0.540482	5.345561	14.126982	3.899661577
0.138491	0.0707504	1.794143523	3.273931183	1.932270967
48.49670222	56.8680096	53.4848478	49.33964263	157.077
1.03271	1.60877	3.75611	3.30101	0.942697

0	0.438863	0.104812	0.850177	0.126991
0.445198	1.10607	2.4309	2.14062	0.512854
5.36091	4.31841	2.853	5.79723	5.598837216
0.11696	0	0.111386	0.607001	5.598837216
0	0.0773702	0	0	0
0	0	0	0	0
0	0	0.383952	0.259448	4.33109562
6.608266	5.900680439	11.522219	12.581543	24.16420395
6.608266	5.900680439	11.522219	12.581543	24.16420395
6.608266	5.900680439	11.522219	12.581543	24.16420395
1.01841	1.00152	1.78469	0	2.56259
2.298696398	2.6537599	4.97061	4.188868	13.4424
0.0235842	0.498389	0.000149447	1.9072	50.0609
5.74E-06	0	1.16E-05	6.51E-05	1.52E-05
0.362218	0.1389	1.28173	2.9782178	2.182277935
0	0	15.5425	30.0069	56.8117
93.2676	81.35370015	116.189873	120.627581	150.083
194.78986	203.41709	150.28924	141.8965	43.1333
23.48950027	24.29193044	35.43780019	29.151918	81.4663
19.94314873	20.2522787	24.90439655	24.80695622	41.47649
19.94314873	20.2522787	24.90439655	24.80695622	39.16982
19.44906	17.6891	24.94413	19.70761	56.639838
0.920617597	0.650341	0.68579661	0.37446698	0.0480984
8.423551	8.351326635	5.28639	6.547235	3.374752313
8.423551	8.351326635	5.28639	6.547235	3.374752313
0	0	0	0	0
0.0723091	0.144823	0.0694008	0.279082	26.97697384
9.0258807	7.3043454	23.8769095	24.2490856	26.97697384
9.0258807	7.3043454	23.8769095	24.2490856	7.19848937
9.0258807	7.3043454	23.8769095	24.2490856	3.105522
9.0258807	7.3043454	23.8769095	24.2490856	3.105522
14.7008846	14.24216	17.400512	27.07508817	49.18465335
14.7008846	14.24216	17.400512	27.07508817	49.18465335
6.70222229	7.320586	8.454201	15.35392	13.66818292
0.0809304	0.240497	0.177360042	0	2.859112743
0	0	1.06322	0	0
16.37026195	19.54833353	10.325926	10.0313319	4.448518866
16.37026195	19.54833353	10.325926	10.0313319	4.448518866
1.033641	1.235148	1.49311386	1.467984918	68.06733615
34.55496	44.83831	28.08324	29.361329	11.42126
8.756076648	10.84926344	15.48388804	13.87261984	13.4631
4.62327	2.8368	10.1033	9.7894	2.60095
7.794815	8.11977	6.590158	7.58727	15.04879
7.97836	8.11081	9.78418	7.60679	28.8677

7.88665	8.76981	10.9201	7.78241	28.2973
9.620829577	10.48829059	13.2775459	11.14221811	41.76871
39.79173	40.00952518	4.799943704	2.449988913	37.1389
30.99149962	33.09455034	26.34539266	27.2621609	47.5204
30.99149962	33.09455034	26.34539266	27.2621609	47.5204
49.04894	47.58662505	32.930625	31.91645	12.2584
36.01664	42.09295	37.20121	40.03927	47.36414876
36.01664	42.09295	37.20121	40.03927	47.36414876
24.735	28.434	29.7745	23.1448	85.8153
9.184428056	9.080598799	14.74764327	19.53991752	11.3084
0	0	0	0	0
3.211241736	3.305315924	5.830048	5.639983662	2.48585
3.211241736	3.305315924	5.830048	5.639983662	5.509974
17.7502	18.9405	20.0798	17.9258	30.6551
0.865392	0.769002	0.810053	0.701579	0.183567
1.82699	2.84289	4.72859	5.01672	2.14572
4.016658	2.835717843	2.065952	3.493699437	1.7935036
11.47919	11.87616	13.29076	12.01848	50.6663
13.3332422	14.14550146	13.47646314	15.18426623	18.04754
13.3332422	14.14550146	13.47646314	15.18426623	1.966402603
5.385666	5.616002836	5.808755	5.17228741	18.31771
48.308997	45.529048	45.732284	43.14294221	53.35302
48.308997	45.529048	45.732284	43.14294221	35.72052
6.69591	9.51462	6.1975	3.91204	29.6314
54.21984	53.66890868	43.6865584	44.501003	20.77354
0.727368	0.737056	8.24345	14.8968	12.4883
13.27096	18.45863	23.64581	24.28974	190.93802
13.27096	18.45863	23.64581	24.28974	190.93802
0.0977593	0	4.27734	2.74995536	4.86177
0.0977593	0	4.27734	2.74995536	2.765212
0.860015	0.14583	0.840969	0.955075	0.169119
0	0.0442075	0	0	0
0.136772	0	0.227603	0.666992	0.339679
0.192602	0.052005637	4.0432318	8.28296597	9.2798
0	0	1.277478651	3.649591858	1.190310516
0	0	1.277478651	3.649591858	1.190310516
0.188446655	2.27E-07	1.860655303	3.15599846	1.289280933
0	0	0	0	0
0	0	0	0	0.425456
0	0.186477	0	0	0.108773
0	0	0	0	0
1.8581604	3.281223185	3.7014871	2.105496496	11.3624361
7.9134	8.19262	7.74621	7.68919	1.48696
2.769693799	2.237614807	1.054850496	1.716153439	1.68801

0	0	0	0.0902042	0.0413254
0	0	0	0	0
29.88349775	26.88291927	27.08395489	25.11548752	39.0732
1.448709	1.766934436	1.781310744	1.432833436	0.325145
3.774234437	2.532650628	7.286107467	6.908243352	2.14327
13.050974	9.890852606	42.59903	29.965338	3.420164
0	0	0.566429	1.15393	0.339922
16.04903	17.62333	22.52926	17.79904	58.6934
40.6966	45.9849	49.4259	52.9891	103.8719
1232.522	1371.149	2136.485	1969.045	2227.73
24.27023	32.0765	38.8246	33.8305	52.0666
8.12145	12.1285	12.2444	11.1876	12.1395
2.08119	3.15879	1.02064	1.94717	75.5388
25.7375	21.3444	17.7583	20.9848	11.0097
5.26755	7.44591	3.48966	2.15124	2.30402
36.58096352	40.90674	75.92875671	69.81683	34.99848
36.58096352	40.90674	75.92875671	69.81683	34.99848
40.876	39.4773379	38.10280503	39.5891125	44.47151559
12.40360942	13.59353053	12.66362017	13.19771023	29.8642277
1.98E-07	0.101063651	0.130735	0.187936	2.08623
23.18663348	31.69156267	51.46185784	44.51518309	58.2
6.08816	9.07834	24.1915	24.089	7.30197
3.803153	3.866671087	6.022158	4.216145898	14.019788
57.40254	52.43677	74.5675	78.3455	65.55049096
21.67725	25.3492	29.95031	33.8978	96.8391
0.711415012	0.6373741	2.049186517	2.100552607	5671.816
0.711415012	0.6373741	2.049186517	2.100552607	5671.816
0.711415012	0.6373741	2.049186517	2.100552607	5146.18
0	0	0	0	0
80.94414399	63.18121888	52.65663668	60.80085088	34.06443
80.94414399	63.18121888	52.65663668	60.80085088	34.06443
80.94414399	63.18121888	52.65663668	60.80085088	34.06443
2.527781926	2.982570184	4.074192433	4.3416963	12.21506537
2.527781926	2.982570184	4.074192433	4.3416963	12.21506537
33.4412193	29.42038031	31.68064305	36.241832	36.636313
0	0	0	0	0
6.66051	6.82123	10.9277	10.1214	41.8083
0.531243	0.610131	1.23724	2.29807866	32.31530001
71.59611372	77.7340434	60.50330935	65.65671412	27.966255
32.54758948	33.207573	25.74185925	20.68639	28.683276
0	0	0	0	0
0	0	0	0.567529	0
0	0	0	0	0
13.49559	6.567297	49.9718	54.488	16.94194861

38.20040238	32.06950202	173.5900026	154.4300075	17.20500324
2.268156	1.99191	1.55998	1.879777	2.3885298
0	0	0	0	8.886183
59.110531	22.204097	348.79039	304.92393	28.37007
0	0	0	0	0
5.848795123	6.8939679	9.808173	8.178150548	17.90761
5.848795123	6.8939679	9.808173	8.178150548	11.9408
34.66025807	31.3004052	19.45677922	20.97753299	5.84581
34.66025807	31.3004052	19.45677922	20.97753299	5.84581
0.210509	0	3.80935	10.3675	0.424130162
0	0	1.61969	5.43472	0.424130162
2.33496116	2.041324951	4.013858794	3.40632737	0.953271253
0.130803	0	0.0854559	0.229153	0.0523973
0.0478688	0.144997	0.0469145	0.12599	0
0	0	6.1778	11.6919	19.9795
19.1032001	15.33342215	2.124590485	4.204181668	0.280277
0	0	0.118639	0	0
76.2918	75.763715	57.44622	57.769913	18.67060422
16.45369488	18.38281	17.295169	17.910986	33.51107
16.45369488	18.38281	17.295169	17.910986	33.51107
16.45369488	18.38281	17.295169	17.910986	33.51107
1.13316	0.721174	1.33994	1.41531	0.134827
20.5651	21.761	21.1273	19.1198	40.3985
5.81E-35	7.95E-82	0.0166788	0.0738275	5.12E-09
114.087	161.7	114.09	95.1878	165.271
6.505620003	6.20077613	10.33762	12.71698	32.12457
7.34009	6.98877	13.20486	10.80371	24.58564
5.618871	6.222575	8.28281	7.599928	15.353124
6.436692777	4.355968	8.158679	8.561095149	42.83011658
41.3737709	45.82441528	62.24858	52.44643914	60.2691
0	0	0	0	0
0	0	0	0	0
10.39840351	10.16385628	7.5358359	7.793325389	6.005451579
0.593667343	1.092872	0.362286992	0.393430653	4.26026761
0	0	0	0	0.596185
69.6542	49.5736	77.7498	69.6538	13.87681347
69.6542	49.5736	77.7498	69.6538	13.87681347
69.6542	49.5736	77.7498	69.6538	49.9756
265.494	251.268	327.3096	249.1205	118.7713
30.19616944	32.3863777	39.66363855	39.68270779	67.119
50.3455	56.4737	55.901	42.7659	93.7003
8.156167402	7.453362902	8.301477431	10.82757831	6.208505005
17.31261099	23.95956475	29.57820379	28.02498	7.81757087
18.00893	20.7753	16.94306	18.2873	9.1823

71.88414942	59.22944717	18.042328	20.72233015	1.3058
35.711032	35.82427	35.33290698	37.77735	54.1386983
18.663092	19.12995	7.890863599	8.190014971	3.670939
1.848184	1.961439	0.4700135	0.6796421	0.478671
25.98538814	25.95304985	24.46695654	26.52195076	3.28088
25.98538814	25.95304985	24.46695654	26.52195076	8.902796124
0	0	0	0	0
3.73918	2.68028	6.45074	6.18279	37.2932
1.48467	3.98193	1.06E-13	2.76E-25	0.000148807
0.17785047	0.211851	0.624112	0.868341225	0.33692
0	0	0	0	0.678194
0	0.279973	0	0	22.6433
2.011526881	2.51739267	4.794127	4.612983679	2.225666677
4.06E-06	0.204805	0.348195	4.67E-06	8.12687
0	0	0	2.79E-06	1.13474
52.6433	38.1488	23.8404	14.3867	1.8967
45.3066	47.1554	34.5881	33.2555	0
24.297279	23.01581293	20.995464	25.302924	21.084314
0	0.353689	0	0	56.478
1.64E-05	0	0.152318	0	56.478
0.164527	0.220932	0.161373	0	4.27075
3.664943237	2.743427246	1.329284106	2.047795275	1.08437
21.19733	23.56141	14.99707	19.26456	19.5719
13.781905	10.85752124	15.42376	13.00714615	22.53832
0.49497	0.0692936	0.462298	1.08857	2088.08
40.3901	43.25544	68.60007	47.8055504	83.3531
31.3939	31.3325	35.6262	29.7168	66.5756
7.104719357	7.432550648	7.331297985	9.737245831	17.87596625
10.62886	13.56627	13.73722	13.7475	64.2257
6.216557	6.95923	9.74433	5.09113302	4.10132
60.3462	63.5682	116.6371	104.2485	146.098
33.9871876	31.5078877	19.73077433	22.3012646	34.74919298
66.6032	60.17283	91.1217	72.44595	179.673
0.2221912	0.0513454	5.009859224	6.496242698	4.57657
0.349683045	0.326101	2.568662	4.500473561	1.622310257
0.349683045	0.326101	2.568662	4.500473561	1.622310257
0	0.30209	0	0	0
0	0	0.257148	0	0
0	0	0	0	0
22.71982	19.98167	42.76862	41.46426	25.0347
22.71982	19.98167	42.76862	41.46426	16.1363
5.079287	5.553830487	6.5488056	8.156727064	27.52547
0.462587	0.354527	0.227116	0.457771	52.8961
1.103774	0.953244	0.34781	0.123024298	249.4031

0.308702138	0.3290411	0	0	39.1564846
0.308702138	0.3290411	0	0	39.1564846
0.671307	0.899441	0.771435	0.886801	0
0.00181746	0.000363784	2.99958	1.58128	8.84973
0	0	0	0	0.0803481
0	0.20608	0	0.13272	0
0	0	0	0	0
0	0	0.0770181	0.206111	0.36647
0.222685	0.372275	0.570847	0.289051	0
0.249356137	0.050138702	0.178786142	0.323539166	0.525503
47.5111	39.85077	3.902424095	7.04353	0.270489145
0	0	0	0	0
0	0	0.105095	0.168327	0.722944
0	0	0	0	0
1.127101314	0.712404942	0.0466616	0.625778095	115.13695
0	0	0.253721	0	0.926504
0	0.0932536	0	0	0.0538621
188.502	181.633	73.2852	107.669	165.639
10.645014	11.96440631	13.683407	11.293823	32.28678
0.113955	0	1.54357	4.44185	1.7572
0	0.0565058	0.110027	0	0
0	0	0	0	0
0	0	0	0	0
0.165788	0	0.328149	0	0.984542
45.82986168	43.72480508	50.37129078	41.79516716	66.60987
9.72315	8.207402	2.326706762	3.033174	0.491383
17.172	13.974	1.11153	2.91681	0.518097
42.12763	40.53306	24.049086	26.73506351	8.888670938
0	0	0	0	0
9.234281	10.43568905	8.395474012	9.237788	13.867359
57.5463	54.4566	81.0898	70.3013	180.3463
10.68820414	12.32911501	21.9910503	16.7133659	67.4831
88.90610163	93.76344146	101.2716376	89.13385242	184.3822
3.421971	3.03454	5.51505	6.096066	19.3311
29.23424	28.88919	24.524406	24.963373	29.88684
1.40301	0.652438	1.0446	0.707056	0.328002
0	0	0	0	0
2.78E-06	0.0958808	0.407279	0.56737	0.000499454
31.59361427	29.965672	20.30488454	23.89008237	26.39435
111.8922482	109.8643521	140.4703371	130.0807357	132.040057
111.8922482	109.8643521	140.4703371	130.0807357	13.279376
8.14E-05	0	3.76096	3.51824	3.02549
0.364269	0.515399	7.45E-09	1.17E-06	95.0766
0	0.106679	0.104061	0	0.362905

0.00163479	0.000306367	2.38E-06	0.231296	1.16997
1.065581386	1.093112477	2.267429	4.144061	8.687586
29.68115979	21.66303	15.88334263	17.81630116	0.798458
4.767361	5.03245	8.849032	6.27478	86.7257
20.4712	31.2196	75.4575	84.2225	897.09
0	0	0	2.12646	0
3.158622864	2.64814	5.864747	5.616674887	24.79804
15.37854	17.294873	10.95851493	11.6541	2.990745
617.5005449	446.0709285	63.49842356	89.39900501	9.4617
47.35368	45.440901	45.796323	43.57538	85.23998
66.1444	31.9308	22.5163	29.1131	1.27047
35.84763191	28.07466234	94.76165196	119.0423116	113.7369576
35.84763191	28.07466234	94.76165196	119.0423116	113.7369576
219.2590369	215.3003001	456.866855	485.7238925	220.6537
41.9764	50.3168	53.4263	42.6864	60.5374
2.960602	2.801195	7.56875	10.94991	139.2999
3.249361447	2.09041071	5.535838041	6.702310594	4.233838052
3.249361447	2.09041071	5.535838041	6.702310594	4.233838052
23.13764	24.06743	22.78767	23.75496	27.04318
55.8599397	68.7176888	41.5173958	38.560374	43.30345
0.569571	1.023850001	0.986021	0.6675694	27.8424
6.065509	6.733439	7.602	9.89019	9.24669
21.43722	22.80979	28.16806	27.58457	51.951724
8.96765	9.20635	10.98797	11.25938	31.926654
47.853796	43.01283865	35.52660476	39.94318	13.35745
68.5166	72.1794	29.6039	36.7763	5.29946
11.42874837	11.70791583	13.13549831	12.89024612	9.00815
52.8104	56.51879	68.94345	51.72852	119.311
19.77944	23.4964	21.29197	20.63431	33.8511988
58.638237	59.21403	40.584803	37.52886	9.002622
0.0562969	0.158286	0.00134996	11.9426	20.04371
0.0562969	0.158286	0.00134996	11.9426	20.04371
25.052039	23.76159619	23.261237	22.622973	42.2493
134.446618	140.141052	132.1762034	116.1165284	38.4781
134.446618	140.141052	132.1762034	116.1165284	3.222720215
134.446618	140.141052	132.1762034	116.1165284	208.7800984
134.446618	140.141052	132.1762034	116.1165284	208.7800984
1.53E-06	0.0470921	0.597812	0.0251847	28.4644
3.881894	3.508146	7.19909	7.824923	51.89423
35.53991011	35.873735	36.60523251	40.43509778	54.25857475
11.89446	7.18556	14.8225	18.33824	82.2787
22.98272	23.06366	7.694853515	9.337688837	2.1779
20.88757072	26.084011	14.11747762	16.17191955	3.1257122
61.07215	48.33117	12.74375515	14.053197	2.40962

3.9608	3.141935	2.37068	2.28445	0.311531
735.945	675.816	52.5369	240.925	10.0206
53.40985631	55.11464945	46.85735587	38.14607474	28.32373339
3.863625	3.28417	7.45058	7.62523	28.9669
30.684253	26.54000975	31.851064	32.75141	33.24529459
30.684253	26.54000975	31.851064	32.75141	33.24529459
24.38441187	22.567637	28.5568759	27.28765403	21.4114
24.38441187	22.567637	28.5568759	27.28765403	6.74222
24.38441187	22.567637	28.5568759	27.28765403	60.4405
22.85946	28.18304	24.16401	21.58499	4.912788661
8.56514288	9.89790919	10.99448609	14.1053741	18.14307993
8.56514288	9.89790919	10.99448609	14.1053741	18.14307993
8.56514288	9.89790919	10.99448609	14.1053741	23.56032543
26.56996032	25.04662431	13.26861018	15.58238075	4.468288575
26.56996032	25.04662431	13.26861018	15.58238075	4.468288575
0	0	0	0	0
28.2063	28.746784	26.63036	27.11402	21.64756118
7.56863	8.1532	9.13087	7.29701	9.09E-06
514.14894	520.55636	453.02444	395.24632	197.8718
19.20063044	16.40267562	18.13624619	17.63053382	16.51476
6.73397	8.00324	8.20914	6.34294	3.44708
5.922455893	6.837564088	7.335702334	6.26168079	2.436695392
0	0	0	0	0
0	0	0	0	0
3.267585	2.51172816	4.188821	3.169380331	1.56109
1.73386	0.88599	3.39868	1.08076	0.000220237
35.56141942	31.363878	8.464673303	13.51427849	0
35.56141942	31.363878	8.464673303	13.51427849	3.79E-07
93.3527	91.59593	67.58183	68.88485	78.50927
8.79684	7.38237	11.9057	9.83622	22.9378
0	0	0	0	0
41.3089955	33.632057	26.503354	27.5609643	11.9988798
41.3089955	33.632057	26.503354	27.5609643	11.9988798
0.12653	0	1.23368	4.67626	2.15112
0	0	0	9.27414	0
42.791	32.5703	12.7712	6.20468	0.757927
0.116119	0.117058	1.14106	3.03529	2.193522747
0	0	0	0	0
0	0	0	0	0
0	0	0	0	0
7.914244	6.9602	7.94591	8.812667	9.28583
81.72336	82.64571	66.25682	55.93475	86.10467
674.759	839.12	3620.348	2792.206	49.3991
13.79472	8.445912	11.67696	10.84244	4.2761

13.79472	8.445912	11.67696	10.84244	5.71684
0	0	0.0293921	0	0
0	0	0	0.425441	0.387344
0.441450395	0	0.942031503	3.048994	0.915015
0	0	0	0	0.0351102
0.127085	0.043253	2.18153	2.48711036	1.991680544
0.151091	0	2.00149	4.49588	5.35738
18.19385	20.440867	26.11126978	22.17936	43.41226104
0	0	0	0	0
0	0	0	0.0777607	0.20856
0.0382018	0.0740595	0.0722972	0.143426	0
0	0	0	0	0
0.419019	0	0.268989	0	0.164911
0	0	0	0.353154	0
0.247024	0.360887	0.241066	0.350513	0.358389
0.0511784	0.0260648	0.0255473	0.0692991	0
0	0	0	0	0
0.883716	1.37349	1.31707	1.43162	0
0	0	0	0	0
0	0	0	0	0
131.543	102.135	391.3894	354.4416	270.8973
0.16305	0	2.08467	3.81459	3.17971
0.000180001	1.76E-05	2.56E-05	2.23E-05	1.42E-06
25.30486	22.36262	22.11198	20.702835	2.721347632
12.7936783	13.43799325	6.1794123	6.2771714	10.09411689
24.445347	23.10284	23.443597	21.333465	41.095012
11.17115	10.28412	10.91669	13.41374	34.4330233
2.3561	10.5512	4.99474	3.90043	13.0373
4.96346	13.4477	7.81686	4.44557	18.8986
15.56486	37.4795	17.895642	12.32999	10.7797
15.56486	37.4795	17.895642	12.32999	31.6194
2.227271	5.539364	4.04561	3.16276	12.35435
0	0	0	0.2248	96.6766
0.819073	0.0278586	30.3824	75.059	68.7805
1.16296	2.33257	1.77838	0.888779	21.1435
9.637	6.32929	23.1348	22.2777	0
1.707324459	2.314926761	5.90745791	6.143814737	2.931901
42.3481	46.353	36.9576	37.7958	33.9629
0	0	0	0	0
16.67450418	21.85996464	42.97309519	37.52915802	35.16651247
0.0424137	0	0	0	0
0	0	0	0	0
0	0	0	0	0.129318
2.744600134	0.568638	65.67892	46.16717	97.72428

0	0	0	0	0
29.4346175	33.0065921	52.638028	44.1219423	115.0491
8.33614	8.45913	6.91611	6.22721	1.29963
129.35004	130.142679	46.12085006	57.22572	15.68801024
8.47881	6.61724	12.2514	10.3446	40.6601
0	0	0	0	0
14.35592766	13.31340576	10.68480549	12.11750197	3.35142
0.709777	0	0	0	285.313
0.988603006	1.484043	0.200630506	0.77844581	151.359
17.65862	16.39892	11.3834	11.65246454	3.17346
30.896936	27.9385645	24.38283918	26.4584336	16.146266
30.896936	27.9385645	24.38283918	26.4584336	16.146266
0	0	0	3.6658	0
1.63546	1.0035	1.29982	1.76759	36.9519
0.118479	0.120788	1.66832	6.66507	3.16867
0	0	0.186977	2.84934	1.53E-07
0	0	1.67517	3.59425	1.96195
16.265372	16.45039983	18.26597441	19.54475468	23.70743
0	0	0.0188583	0.45075	3.97859
0	0	4.60403	15.496	438.628
0.0227604	0	2.11552	15.6262	174.891
0	0	0.000548559	0.000173716	3.34E-05
0	0	2.73388	12.2882	81.1348
0	0	2.75385	13.8862	131.48
0.0200931	0	0.46804	1.49434	10.4372
0	0	0.691001	2.07311	17.9763
0	0	0.290248	1.43533	4.92534
0	0	0.0862959	0.576808	7.28615
0.122072	3.20824	2.20462	0.000581024	7.4008
65.43308	48.261831	18.029097	49.5699908	35.97825756
65.43308	48.261831	18.029097	49.5699908	2.40084
65.43308	48.261831	18.029097	49.5699908	35.97825756
10.71506	7.640234995	6.415926	9.62261733	8.687527
33.36966981	35.45532647	50.59913213	55.25537412	25.81182514
0.219207	0.0868477	0.512681	0.339947	1.80733
7.708011	5.722274684	9.165126574	7.004200694	6.045091726
55.55902143	53.23864884	67.5585205	72.41625004	56.35550729
9.626088	10.50468204	13.6459	12.760045	316.4034319
36.25219	36.21452	29.23215	32.3725	13.131553
27.62115818	29.03279704	26.52093377	24.28267201	43.1236
0	0	0	0	0
9.74E-06	1.27E-05	1.12E-05	0.763878	1.02E-06
0	1.06976	0.338928	0.916967	0
10.1162	11.8963	8.34945	5.75162	3.38075

0	0	0	0	0
0	0	0.179417	1.75528	13.54663
0	0	0.42032	0	0.56692
0	0	0	0	13.54663
0.340648	0.114287	0.111607	1.04432	0
70.0750242	75.4818739	68.0606797	86.7805341	0.371474
70.0750242	75.4818739	68.0606797	86.7805341	0.100673
0.308474	0	8.99882	14.39244	0.335208
0.308474	0	8.99882	14.39244	0.335208
16.81544	13.30002	10.28125	8.50225	5.670206978
0	2.52423	1.11489	0	1.54102
100.74195	88.97809	100.5367452	84.54682438	17.43803
1.395653	1.156407797	1.392126213	1.30613362	0.848493021
1.395653	1.156407797	1.392126213	1.30613362	0.848493021
0.320195	0.203343	0.158273	0.0532544	0.0953084
126.632	92.2381	21.3443	17.4741	2.69332
25.403734	32.97918	26.78004	21.93033	1.99049
25.403734	32.97918	26.78004	21.93033	53.75529
5.070636099	4.638129	12.95345002	25.26938	41.23851187
5.070636099	4.638129	12.95345002	25.26938	41.23851187
0.272281006	0.2081244	0.727049916	2.876988132	20.33190114
4.50405	6.10024	4.87765	4.03529	10.7458
0	0	1.353244139	2.461933823	3.767342123
0.469133	0	0.281778	0.250838	0.453866
0	0	0	0	0
0.275070071	0.498587	0.072322811	0.048702122	100.2183
18.63469	15.56826	22.42072	15.353146	41.13374127
21.29331797	27.638749	30.68328221	34.62174136	26.64000721
1.91369	2.50117	5.23716	4.86483	1.85334
25.45181395	24.39996495	32.23349788	32.95340006	33.25756437
25.45181395	24.39996495	32.23349788	32.95340006	33.25756437
22.6529	17.7997	23.7119	16.0096	9.56992
0.56946325	6.668611	1.615352	0.152368	9.77261
0.56946325	6.668611	1.615352	0.152368	9.77261
9.68004	9.566536062	10.29497	11.8459	39.3345
0.141906	0.2841312	3.666538	5.40885	6.27591
20.22134	20.30924	21.34811	47.78866	83.80422951
0	0	0	0	0
0	0	0	0	0
40.3604	51.2639	126.116	112.964	38.107
11.94618961	12.50210659	9.22919271	9.451672087	2.714131323
0	0	0	0	1.26889
0.0685539	0	0	0	0.250491
0	0.0355995	0.0696569	0	0

12.8623	11.5311	15.2602	9.31933	12.6707
54.71611	66.40403001	12.0017687	17.223812	56.76865
54.71611	66.40403001	12.0017687	17.223812	56.76865
54.71611	66.40403001	12.0017687	17.223812	56.76865
10.9067	13.689	8.6232	11.0502	0.18845
1.33198	1.58406	3.6644	3.14124	0
17.12311542	16.87615749	13.4180792	14.367223	25.60922
17.12311542	16.87615749	13.4180792	14.367223	25.60922
2.500978	4.497808	0.066917748	0.044089083	717.3528
0.08628694	1.24E-13	1.046249	2.053534	1.77789
0	0	0	0	0.357342
1.72884	8.98644	2.80162	1.02431	15.93672
40.2187551	40.60284603	38.97321011	34.64122465	47.69891
18.03779	19.19360281	16.52895315	14.74121	53.60505
30.5573	25.0567	4.69551	15.6739	6.00E-05
0.0741072	0	0.0713948	0	0.0866243
0	0.390948	0	0	1.69847
0	0	0	0	0.725109
1.40247	0.491099	0.881205	0.711623	0.145726
2.568802	1.749599	3.93483	6.008888	33.93603
23.47560834	25.16641957	20.41141344	21.485294	53.89523343
6.819083038	7.242609513	10.63260279	7.861478337	6.091630916
0	0	0	0.185378	0
0	0	0	0	0
0	0	0	0	0
0	0	0	0	0
0	0	0	0	0
33.44992451	29.00591688	21.50418727	26.85614407	10.229535
33.44992451	29.00591688	21.50418727	26.85614407	10.229535
121.6071	135.6093	80.5398	72.03394	60.1798
209.2657099	181.12017	216.0783562	242.9731782	112.9368
9.62298269	9.74244	12.6476	10.07157511	48.7859
14.441	14.2388	39.9543	38.3985	30.223
18.6454327	21.94524	16.8623595	16.8608055	82.64371228
0.0230038	0	1.32131	3.77694	3.31422
2.638614	2.597393	4.66135	4.03411	4.03436
41.16696897	37.13073674	43.25417625	39.2389963	29.0942
0	0	0	0	0
52.63270784	48.6475118	62.95787131	69.48040828	56.20235689
52.63270784	48.6475118	62.95787131	69.48040828	16.857649
3.619103731	3.044245309	5.877560855	12.0590117	8.5936105
0.2188587	0.4939539	5.29846	8.159264	3.901347839
0	0	3.1379	4.629	4.50986
0	0	0	0	0

0.218145566	0.116139	6.78066	15.29768	14.91091434
2.70346	4.88306	4.1036	0.38652	0.552611
61.62761	52.79406	274.2341	291.1073	268.4785
13.821658	13.889968	15.743905	16.39662949	29.44665
33.80819595	25.74507483	39.0824777	34.86658086	25.00350539
43.66151	43.1924	34.7695	31.18147	15.7193
35.01436	41.93475	40.4464	34.22541973	44.51017
27.5389	33.0332	35.2428	31.9658	102.6196
22.1493513	21.33959858	19.35340212	17.31075349	14.11999
22.1493513	21.33959858	19.35340212	17.31075349	14.11999
21.70769	25.027774	30.50934	26.95238	108.89
21.70769	25.027774	30.50934	26.95238	22.5559
34.13216	31.6335	35.16696	30.77045	17.79867
21.6505	15.8875	30.4816	29.6773	43.9001
53.85738	46.40278	17.79646	21.88728	0.999704047
53.85738	46.40278	17.79646	21.88728	0.999704047
103.0293252	90.88937637	64.42798098	65.25092483	64.135978
103.0293252	90.88937637	64.42798098	65.25092483	64.135978
103.0293252	90.88937637	64.42798098	65.25092483	6.02541
0.729896	0.243949	15.8455	33.2788	26.3628
0	0	0.19762	0.268906	0
14.26697	16.59271	19.83271	16.200855	35.37846
0	0	0	0	0
0.278625	0	0	0.354884	0
0.0783798	8.46E-05	0.0972482	0	0.000168536
25.60249225	24.26629529	42.74619643	36.395424	50.3781
2.99912	4.50566	6.56195	4.25244	6.44637
0	1.14588	1.77096	0.49083	49.7442
72.25080212	73.27576543	82.35363016	88.11676011	0
72.25080212	73.27576543	82.35363016	88.11676011	76.03149
1.15218	0.143198	19.6623	34.7866	16.5771
0	0	7.7387	18.3786	17.9599
0.173718	0	11.2235	20.7507	21.924
0.1564991	0.609068116	0.899899	1.331783	0.203492
0.1564991	0.609068116	0.899899	1.331783	0.957757
1.156366222	1.23562161	5.721049	11.51866	18.2418
2.5259	2.445026	4.81879	5.86677	48.92542
3.990406	5.093117852	9.791457887	7.021796427	30.758376
35.45152098	31.84661769	22.86789269	20.85653341	1.84624
29.04708623	23.5799269	46.22321095	43.7047104	36.19227151
0.11125	0.226789139	4.23249	5.297397947	3.770088
0.11125	0.226789139	4.23249	5.297397947	3.770088
0	0	1.62664	4.25313	7.52544
0.14981	3.72E-05	7.92511	17.6814	24.4076

9.41E-20	5.74E-66	0	0.110486	6.44602
0.789427	1.33174	0	0.207968	176.92822
4.0674537	3.8268832	11.346973	12.5575157	5.531439573
0	0	0.617526	4.22528	19.0448
36.747582	36.508167	48.449011	36.45787	95.30130239
8.082126	8.27393	8.123762	8.73006	1.0754
25.03874142	22.23368352	24.0383509	23.36097429	15.790639
10.1923086	10.64414648	9.48308064	10.447604	10.02799
129.2991793	123.5092159	16.81375636	36.28253395	3.98013182
1.9917563	2.763636	4.87338764	6.25535844	23.94546
47.6901	42.2823	79.5495	60.2245	108.722
6.531801	7.217735	6.677461	6.135994	1.512793544
6.531801	7.217735	6.677461	6.135994	3.23794
0	0	0.0948568	0	0.116578
0.0356597	0.115705	5.12E-07	0.0640702	4.70021
0	0	0	0	0
0	0	0	0	0
46.18306565	42.34778252	34.1835759	33.68683044	11.36749
3.030896687	2.847644	5.034011891	5.73188	56.24633892
46.00521	39.58365	36.378684	32.61058	1.09658
46.00521	39.58365	36.378684	32.61058	214.5567562
46.00521	39.58365	36.378684	32.61058	3.27387
15.1489	13.4903	14.9102	8.64106	88.1363
22.50494415	22.4226135	21.42949693	20.01944311	27.976105
143.796805	124.33816	120.342061	109.2620414	73.5603
38.8019	33.5891	6.33464	8.3565	2.6009
139.39012	138.656454	115.20585	112.84371	68.17938
139.39012	138.656454	115.20585	112.84371	68.17938
9.65443	11.5594	20.6909	23.1895	11.0812
0	0	0	0	0
47.06930436	31.46550449	52.93990221	55.7398018	12.83450202
1.33546	1.1068	1.06686	2.26777	0
0	0	1.39161	3.02479	1.04236
0.3144508	0.33909	1.6640835	3.09551	0.996491
83.1105	85.8304	80.41546	75.43925	138.5335
83.1105	85.8304	80.41546	75.43925	138.5335
0	0.0435418	0.0424641	0	0.102643
13.646855	18.55885194	14.96325	15.67878065	214.6464
0	0	0.138971	1.5154	7.3505
9.358980635	6.911683912	13.970841	15.67850285	6.954657528
0.178983	0.179127	0	0	36.0368
0.9889075	0.566451	1.5219371	2.942591222	54.6068
36.14572104	44.47797	53.87931977	48.89457601	134.2831
5.01299	5.86748	19.0146	18.3247	12.4922

0	0.039943	1.243587462	1.663775394	1.498349103
6.68E-07	9.07E-07	5.85E-06	6.24E-06	7.09E-07
22.83491547	21.50855983	20.20842606	19.94187	29.37569793
66.0047	81.6594	67.7906	62.4853	176.0937
77.865588	94.40904	48.138052	48.15965	58.9972
21.091018	24.46861	28.70812	24.68317417	47.697257
22.13145	23.91511	19.81349	16.12635657	51.25162045
216.0912001	248.9226	32.61191689	50.07204849	5.327081568
216.0912001	248.9226	32.61191689	50.07204849	5.327081568
0.396073795	0.499112	2.10862	3.66518	2.02433
78.57028	84.72933	68.96246	69.13104	73.32295417
78.57028	84.72933	68.96246	69.13104	73.32295417
64.95277	63.01238	59.1123	60.13021	73.32295417
1.174966	1.88871	1.18547005	1.616312	0.312404
76.83591153	68.2386725	49.375387	55.996798	25.86443
0.333024	0.919521	3.01598	1.73162	1.09704
721.7002	664.57456	880.908	718.235	184.5332
9.49194	6.55859	18.3073	16.18432	6.001701908
9.49194	6.55859	18.3073	16.18432	6.001701908
18.3123	18.05282	23.86041	24.25176	25.7187
49.391323	45.15651874	43.505343	47.62013434	53.1939
12.1055	11.7019	9.93454	11.657751	14.53250039
8.759405211	11.17537136	19.08907115	19.55223133	31.40643
8.759405211	11.17537136	19.08907115	19.55223133	31.40643
8.759405211	11.17537136	19.08907115	19.55223133	31.40643
14.10057	15.29016	14.29174185	9.68142	72.0894
50.57931844	47.61817046	51.23542735	48.27181639	83.63780769
29.540708	24.9541038	38.12950366	48.92030764	17.98130861
14.35244	13.2605	8.184113	9.03086	3.43665
1.936771782	2.303403421	2.312359283	2.10321673	0.56443152
1.936771782	2.303403421	2.312359283	2.10321673	0
0	0	0	0	0.559368
0.942294	0	8.98809	16.2298	17.6878
2.343009822	2.09942146	4.244508602	5.604843112	2.341367
2.343009822	2.09942146	4.244508602	5.604843112	2.341367
98.6568	84.044	76.0446	70.2371	2.2757
56.37564468	55.089552	37.193038	43.3140475	11.98322049
0	0	0	0.415905	0.366004
6.11643	6.829790731	7.37759	7.808210008	109.01598
2.413786045	1.887290782	5.562978935	9.094327785	62.193413
2.790162354	2.873825	2.229779584	5.627445	66.3026
2.790162354	2.873825	2.229779584	5.627445	66.3026
0.160621	0	2.23516	4.63101	3.06855
0	0	0	0	0

33.70128051	34.136772	26.529657	25.96799486	24.786404
19.99416002	19.5768808	20.40405674	20.56119004	38.87770004
6.475770884	4.75201256	1.592333	3.070328457	0.07008
8.71725	7.848398283	7.184593	6.401286	12.96680598
8.71725	7.848398283	7.184593	6.401286	12.96680598
10.80858	14.04892	27.40719	29.8623	24.64048125
45.03818	50.07809	48.66748	53.694576	74.579998
3.26984	3.60178	3.20526	2.22913	0.88877
0	0	0	0	0
0	0.388571	0	0	0
0.368433537	0.390561124	0.145030645	0.543712	2.73561
0	0	6.67E-07	0.23193	0
0	0.0954972	0	0.2403	0
4.220652854	4.35888823	4.831516126	5.674847239	21.95663
0.280782519	0.2839035	0.275923029	0.478225087	8.617440082
0	0	0	0	0
0	0	0	0	0
0	0	0	0	0
0	0	0	0	0.0720184
0	0	0	0	0
23.55638293	19.50543153	13.018786	13.72303673	4.883166
23.55638293	19.50543153	13.018786	13.72303673	4.883166
23.55638293	19.50543153	13.018786	13.72303673	4.883166
0.24159597	0.222751104	0.139080142	0.238162762	11.66806
19.698847	21.68545734	26.54702457	20.9711639	67.23351
0.066566886	0.133795865	1.357621466	1.571943946	1.33456372
0.897376	0.300063	0.233739	0.103403	0.251965
0.481982	0.298143	0.114094	0	0
26.52333536	27.63461219	18.67291432	18.39651936	28.94903865
584.54	649.832	927.002	837.051	1000.652
123.6188	109.75404	96.18541052	112.2896	214.1028
27.7586	33.01514	34.39051891	38.396959	17.91433526
42.25021	51.00652	50.9707	31.18024	114.44508
0.112165	0	0.334793	0.452957	0
908.349	723.01	181.3563	207.199	14.18168
0.52389048	0.83722864	0.456043	0.523209524	87.1153
37.34012187	41.299029	41.1199179	42.52935411	56.0198
0	0.137258	0.403516	0.0896976	0.662078
0.163703	0.66229	0.475643	0.214008	0
8.59977	10.8235	13.20116	14.0911	41.3903
15.73688	16.60911	25.16954	18.102	43.4118
29.81927	28.309325	11.73433748	15.99477	4.887912079
2.030169721	1.993878	2.323107112	2.371931	1.478311858
1.26181	1.28823	1.08726	0.113632	1.83445

0	0	0	0.233329	0.400798
12.49668212	14.62463756	8.081247467	8.638334914	5.393922705
35.3768311	38.24334	30.37047	34.53144	16.31426
7.755102	12.80499056	19.422527	13.90307071	6.47806
8.791716551	7.45791124	5.64830842	4.129501	0.980159
4.52749	4.73009	3.57031	4.93675	2.120109146
0.1994001	0.1030337	3.67476	8.29089	2.645120208
0.1994001	0.1030337	3.67476	8.29089	2.645120208
0.0657876	0.236011	3.02843	4.266538	2.48934
8.566479	9.530025	29.25636	32.61682	79.09573122
43.9999	39.02274	126.4226	136.7128	59.0675
43.9999	39.02274	126.4226	136.7128	79.09573122
3.1873	2.01961	8.82414	9.54166	4.85908
6.110284842	5.173656688	9.959754736	10.57971532	2.82337373
18.8648	16.5638	34.7394	36.5472	2.82337373
18.8648	16.5638	34.7394	36.5472	12.4447
0	0	0	0	0
0.265868	0	0.334273	0.226706	1.61283
19.5218	1.95648	39.1625	43.7856	32.2772
21.96474235	26.48790066	19.00013957	18.12876181	6.612865969
10.664291	9.58626	31.235136	26.876564	18.05791602
10.664291	9.58626	31.235136	26.876564	18.05791602
20.93537	18.5227793	13.891349	19.4250511	33.03645
21.1081	24.599	50.0414	44.603	23.01326
0.750742	1.15946	1.59167	1.29839	1.05187
0.47413	0.179943	26.2812	62.4856	82.3417
0	0	0	0	0
77.26402189	76.65161668	16.12176379	17.83625653	2.168449
29.17015	26.69126	23.12166	22.737142	44.62075
19.28558	21.558058	28.282601	32.343547	23.36331
0.367029	0.0919419	0	0.353946	0.73300279
19.28558	21.558058	28.282601	32.343547	0.73300279
19.28558	21.558058	28.282601	32.343547	37.432
4.44517	6.85013	5.47538	3.094507788	1.08303
0	0	0	0	0
6.95651	3.2547	5.81869	8.69583	0.594005
43.2752	25.5137	15.31124	24.83347	0.619433
0	0	0	0.128475	0.23723
0	0	0	0	0
0	0	0	0.17903	8.9974
15.5132	17.7089	16.9614	17.2449	30.0357
4.315124	6.583096	0.666192217	0.384792	815.72162
1.89089	1.41088	0.714813	1.45163	0.361757
0	0.340282	2.620392825	5.775756	8.328624223

11.68881	8.36899	15.52264142	21.85964015	9.68079
0	0	0	1.06344	0
0	0	1.076240469	4.727781	5.611160662
0	0	1.076240469	4.727781	5.611160662
0	0	0	0	0
40.7105	25.5091	64.6379	101.479	59.0364
0.768226039	0.926283	0.749035643	0.815176221	0.347212
1.01352	0	0	0	0
9.28829	10.88721	19.36719	19.68941	9.44513
7.96891	9.48653	39.5423	28.4662	14.82181
23.987471	27.95781	48.4937436	46.549297	20.11714
23.987471	27.95781	48.4937436	46.549297	67.1088
16.965745	18.2875572	16.8086289	14.35440247	14.748893
16.201116	15.96903249	23.33981	25.05622934	45.08094
3.593072	3.863616971	6.07335	5.17594	82.65968
8.153277847	8.184786604	4.845920592	6.136507708	1.985371247
16.5071	14.5753	30.7473	21.5573	0.90656
0.000114138	0.000133462	6.85E-05	0.000805335	10.8323
4.715507	3.872779127	2.991575913	2.68203	0.594376
22.93489479	21.6210362	20.7659101	20.9032727	31.71534
8.84923	10.40106	12.48499	12.83487	26.85049
207.70049	286.6788	515.0449	436.1285	85.42793
0.939799158	0.322	0.0621456	0.830132	184.019595
35.21910749	35.96959486	24.06624	25.09638023	107.9276
6.331623	3.9032	10.91995	10.621676	5.96627
25.83696254	29.58244314	25.43210654	23.09534505	37.94241165
32.5708336	31.62812149	25.2600331	25.555046	25.32562569
32.5708336	31.62812149	25.2600331	25.555046	25.32562569
32.5708336	31.62812149	25.2600331	25.555046	25.32562569
1.017825033	1.321471	1.31560645	2.218094	0.727045
373.145271	303.720444	533.586155	338.763295	98.99564306
31.95805829	31.21351577	32.9336926	31.5445992	64.00589336
25.37248	29.5745	32.90127	22.18937	30.97628
66.70276	73.84988	95.65802534	81.26983	2.73975
66.70276	73.84988	95.65802534	81.26983	265.554
0.59743	0.202659	0.193833	0.264463	1.18476
0.383309	0	0.121847	0.823467	0.753243
0.16093	0.204096	0.15876	0.106584	0.094159
0.463064	0	0.368253	0.247397	0.112389
0.129249	0	0	0	0
0	0	0	0.312759	0.0924926
0	0.0356148	0.104669	0.0938393	0.0421233
8.089445502	7.221520477	12.84700347	14.69344228	11.34186306
8.089445502	7.221520477	12.84700347	14.69344228	11.34186306

19.391572	18.556766	20.0155692	21.75465215	140.0167
0	0	0	2.27671	0
159.69634	171.58163	34.46887	48.65437	4.51212
159.69634	171.58163	34.46887	48.65437	4.51212
66.7015	62.6475	80.3458	59.768	16.8294
11.8814	4.47157	7.4626	15.6089	0.00461839
2.397804	2.490514	5.32924	4.092731	16.87772
2.397804	2.490514	5.32924	4.092731	16.87772
0	0	0	0	0
83.17429138	77.077406	90.39760591	75.54007997	86.5372
0.883838	0.493254	1.62373	1.79996	0.558506
0	0.724091	1.30847	1.85542	0.849627
9.6937	12.6825	13.763	11.2582	2.65347
26.47720753	26.5509653	29.16311398	35.34734601	22.19028
0.119698412	0.397066385	0.110034	0.590356528	0.0360717
76.66668	72.1427	124.3039	100.6161	84.6888
0	0.571534	0	0.366139	0
47.62337	44.98189	31.50458	34.39761	12.72491197
0	0.187985	0	0	0
0.121693	0	0.46557	0.469202	0.422064
0	0	0	0	0
0.0820312	0	0.163595	0.110726	0.100769
0	0	0	0	0
20.30407	20.91149996	17.42478778	19.960014	10.46864414
0	0	0	0.319805	0
20.9571	23.52683	18.65509	20.23579	25.21282
0	0	0	3.81844	0
0	0	0.15379494	0	5.06285
45.411321	42.66257326	40.58307904	40.31394551	8.51256125
45.411321	42.66257326	40.58307904	40.31394551	8.51256125
1.26E-14	1.04E-10	6.69E-05	1.59E-06	0.620559
0.168244	0.169194	0	0.640292	1.31817
9.435786	10.365117	13.68257	12.342499	41.6074
0	0	0	0	0
0	0	0	0	0
0	0	0	0	0
0	0	0	0.157746	0
0.371525383	0.3147856	0.264379	0.687378	0
0.100482	0	0	0	0
0	0	0	0	0.0953065
0	0	0	0	0
24.0086	15.1367	43.5067	40.2	18.3546
0.106583	0	0.100052	0.135814	0.12143
0	0	0	0	0

0	0	0	0	0
0	0	0	0	0
0	0	0	0.659771	0
0	0	0	0	0
0	0	0	0	0
0	0	0	0	0
0	0.319704	0	0	0
0	0	0	0	0
0	0	0	0	0.145987
0.0600768	0.122701	0.0602194	0.0808917	0.0725868
0	0	0.352469	0.623479	0.366984
0.0597977	0	2.1239	5.32116	10.4898
0	0	0	0	0.336946
2.085148256	2.147107941	5.80963794	4.356165538	2.33201
2.085148256	2.147107941	5.80963794	4.356165538	5.598561935
0	0	0	0	0
1.143420226	1.201470035	1.44802682	2.137497	1.77917
0	0	0.12466	0	6.41933
0.516925244	0.334909984	4.19409	4.104148882	4.024076
0	0	3.01126	4.30881	2.28308
10.237569	11.81521304	2.674925001	4.121616	7.4966985
10.237569	11.81521304	2.674925001	4.121616	16.6286
0.377672982	0.637387814	2.116794633	3.31321041	2.32071
14.24159795	14.17804677	13.03249939	14.75358656	7.32863
0.388058	0.181195	7.42032	15.56765	15.65171
17.93218562	14.10641765	22.6667842	19.63801108	18.62515
17.93218562	14.10641765	22.6667842	19.63801108	14.0942
56.858	52.2416	66.5457	53.4342	52.3967
46.5714	25.9215	14.7506	19.5699	0
141.785125	141.072391	117.2550284	107.92275	33.5953227
0	0	0	0	0
1.128601622	1.782801168	0	0.164079	350.621162
1.06101	0.356624	1.00216	1.35014	350.621162
70.36511125	59.81595333	265.104447	266.2650971	79.5285
0.713884758	1.071109001	2.711352823	5.053649056	11.015167
109.907	98.2992	203.8818	224.2617	195.703
0.629412289	0.405656503	0.11228	0.150741284	0.280892
5.92343	5.226306	9.43752	10.731939	7.36474
0	0	0	0	0.563489
2.683992	2.190892	5.866072	4.04761	0.846139
6.006759336	7.062485261	7.842273123	8.98897894	3.624344346
6.006759336	7.062485261	7.842273123	8.98897894	174.8241003
6.006759336	7.062485261	7.842273123	8.98897894	174.8241003
0.95126713	0.998379176	0.444076134	0.14629426	97.55069

0.95126713	0.998379176	0.444076134	0.14629426	97.55069
0.850876908	1.132206012	0.025716544	0.231990903	221.9268
0.850876908	1.132206012	0.025716544	0.231990903	221.9268
1.49999	0	0	0	0.878866
2.079076621	3.0519109	0.783500284	0.458766042	403.9590104
0	0.378117	0	0.0704249	81.3191
1.38794318	0.984151777	2.952932318	2.154080467	2.531282673
1.847085	1.75134475	2.849158676	3.067075634	5.24636
0	0	0	0	0
0.386002	0.0964605	0.649281	0.249552	2.0666
8.21722593	8.124512	10.913275	8.808249	19.04563
3.14447	2.26629	9.31927	7.82813	3.87363
0.491872	7.92E-07	1.15E-06	1.12E-05	6.49E-05
0.725776	1.20902	3.50783	4.73458	9.56017
51.09385474	63.26547	165.7121352	132.8867	73.4793
0	0	0	0	0
0	0	0	0	0
0	0	0	0	158.00261
0.743141	0.774222	0.7078701	1.64344054	1.29176
16.59651101	18.01936	16.4422422	13.92726182	25.7914406
16.59651101	18.01936	16.4422422	13.92726182	25.7914406
13.24728	14.32994	15.58568	15.50698	37.7846
0	0	0	1.19223	0
0	0	0.469232	1.27E-47	2.60E-05
0	0	0	0	0
0	0	0	0	0
0	0	0	0	0
0	0	0	0	0.148518
0.164183	0.163138	0	0.424306	0
0	0	0	0	0
0	0	0.134124	0.0895285	0
0.450281	0	0	0.573294	0
0	0	0	0	0
0	0	0	0	0
0	0	0	2.45067	0
0	0	0	0	0
0.546578	0.177601	0.618084	0.714506	0.829463
0	0	0	0	0
47.0957	45.2237	48.3662	33.4854	1.22423
0	0	0	0	0
0	0	0	0	0
0.184989	0	0	0	0
0.029298435	0.048627989	0.0943414	0	0.186929
0	0	0	0	0

0	0.0806799	0	0	0
0	0	0	0	0
0.0563824	0.105558	0.0513327	0.0675504	0
0.109376	0.107906	0.306515	0.137327	0.123812
0	0	0	0.30626	0
0	0	0.523875	0	0.670415
0	0	0	0	0
0.209116	0	0	0	0
0	0	0	0	0
0.0750552	0.1363885	0.664875	0.245553254	0.289857
0.0530267	0	0.052262	0	0
0	0	0	0	0
0	0	0.428485	0.571402	0
0.794139	0	0.57377	0	0.244132
0	0	0	0	0
0.0978031	0.0961539	0.0910735	0.24697	0
0	0	0	0	0
0.035058	0	0	0	0
0.512616	0.656104	1.45017	0.497402	0.37127
0	0	0	0	0
0	0	0	0	0
0	0	0	0	0
0	0	0	0	0
0	0	0.114746	0	0
0	0	0	0	0.140085
0	0.195675	0	0.256022	0.245689
0	0	0	0	0
0	0	0	0	0
0	0.659413	0.917062	0	0
0.0428669	0.129719	0.0428273	0.0571304	0.158635
0.203208	0	0	0.0638957	0.121086
0	0	0	0	0
0	0	0	0	0
0	0	0	0	0
0	0	0	0	0.240424
0.0777852	0	0.154126	0	0.0968197
0.596923	0	0	0.529969	0
0	0	0	0	0
0	0	0	0	0
0.663114	0.272409	0.597488665	1.277733	0
0	0	3.8348	0	3.1015
0	0	0	0	0
0	0	0	0	0
0	0	0	0	0

0	0	0	0.299161	0
0.167014	0.492372	0	0.216146	0.196791
0.0903814	0	0.171053	0.226768	0
0	0	0	0	0
4.653625495	5.2134734	5.046363009	4.626469448	1.969377714
0	0	0	0	0
0	0	0	0.363048	0
0	0.129652	0	0	0.457179
0	0	0	0	0
0	0	0	0	0
0.123371	0	0.242873	0.162586	0
0	0	0	0	0
0	0	0	0.324853	0
0	0	0	0	0
0.072301915	0.216037633	0.16772076	0.161357036	0.701341535
0	0	0	0	0
0.305513	0.767602	0.295911	0	0
0	0	0	0	0
0	0	0	0	0
0	0	0	0	0
0	0	0	0	0
0	0	0	0	0
0	0	0	0	0
0	0	0	0	0
0	0	0	0	0
0.581569	0	0	0	0
0	0	0	0	0
15.52914704	13.29039271	28.6616531	21.04275616	166.57594
18.90791195	16.81664483	21.295639	22.8856071	8.10854
11.21687663	12.71739461	13.207467	9.880864094	2.789650206
0.532273	0.3564671	2.72988	4.974529216	2.474848
0.195756	0	6.70634	11.1906	5.46265
4.40108	6.15726	9.64671	14.7056	9.68621
0	0	2.820548	5.155649254	3.194092248
12.092357	9.678933911	7.796239487	10.00670862	2.818648175
0	0	0	0	2.19808
0	0	0	0	0
0	0	0	0	0.000438301
29.134871	30.4638449	37.226274	43.516297	47.0949
20.8344	22.0563	2.4255	6.35175	1.45328
1.474017016	1.472584	4.707165	4.861347	4.680772222
1.474017016	1.472584	4.707165	4.861347	4.680772222
19.61674	20.36527	24.38614	19.40835898	56.12357
4.751490422	3.697970355	21.63970006	21.14760011	8.75251

0	0	0	0	0.240836
58.30895	62.31041	28.23337	29.63702807	2.378346
58.30895	62.31041	28.23337	29.63702807	3.884141929
58.30895	62.31041	28.23337	29.63702807	3.884141929
0.122864	0	0.117453	0.325405	2.13509444
0	0	0	1.5726	4.203613
3.694409191	2.784687473	7.478316706	4.793072878	3.018012803
9.18035082	8.396933	20.19531	18.69976234	2.479260144
0.528079	1.0166843	0.616621	0.4023035	0.114443
0.528079	1.0166843	0.616621	0.4023035	0
9.18035082	8.396933	20.19531	18.69976234	9.130090812
9.18035082	8.396933	20.19531	18.69976234	9.130090812
24.41876	28.17441	38.07532	32.38658	24.574
23.99388	21.59388	16.42467	17.16335	5.421042693
16.79442023	15.609712	13.01233288	14.47791672	14.25375705
58.80914704	65.10082703	43.41793384	37.3708577	56.55838604
27.1985	39.6553	28.2774	16.3918	281.204
40.48719	44.60133	58.9332	48.70841	115.3107
41.3171	40.0128	43.4222	39.4032	49.4578
41.3171	40.0128	43.4222	39.4032	41.5784
90.60593446	87.3609052	40.55508685	52.4351864	5.81056
106.2278343	104.310551	81.6244231	88.27169392	71.97437152
2.075022932	1.187248841	2.444752414	2.749038584	18.12241481
9.809907	8.520746622	15.06405068	12.03651765	13.4901
10.29274495	10.22817916	6.22827322	8.259180862	2.304351868
1.11537	1.583604529	1.466206659	1.280532598	2.69973
47.37783	55.08565	53.853038	38.3231	11.54390024
0	0	0	0	0.951064
0	0	0	0	1.12468
0	0	0	0	1.70166
23.79974474	21.23351042	20.30427211	21.52157335	5.760341246
342.652	313.883	52.7287	97.8639	5.14856
27.65714	20.85700698	48.09597848	54.85113232	43.4579
42.044494	43.3573108	37.20692312	34.65805413	210.12876
42.044494	43.3573108	37.20692312	34.65805413	210.12876
0	0	0	0	0
5.538223921	5.018221389	13.64199	12.4517	6.3366005
5.538223921	5.018221389	13.64199	12.4517	6.3366005
0	0	3.853814442	9.005908031	11.794939
0.377177142	0.0544122	11.16040705	23.292648	25.418026
0.430493838	0.789037667	0.082011886	0.088487238	102.8368
0.305151341	0.640729	6.87946	14.0056	102.8368
0.305151341	0.640729	6.87946	14.0056	102.8368
0.305151341	0.640729	6.87946	14.0056	102.8368

0	0	2.58045	3.53456	8.96977
69.63409727	50.17813721	76.23057968	68.59673775	161.644078
57.091751	56.643041	27.109364	37.579222	7.467796172
1.08082	3.58787	5.69747	6.12213	5.21739
0.0158904	0.0644092	0.0316782	0.10795	0.0182337
5.439712033	7.293529711	10.95519488	18.42264891	9.952770134
5.439712033	7.293529711	10.95519488	18.42264891	9.952770134
4.81698	6.89275	4.77567	5.93693	6.38305
56.2581	60.91543	32.43087	35.41537	66.556872
1.585847906	0.902840717	1.82168262	3.330999379	187.12
1.82967	0	0	2.3023	45.5711
13.10968	8.29628	29.12058033	33.85397	36.172855
7.634435159	7.592609	9.663696719	9.66895	6.923141322
4.77576	2.738	5.2362	6.21542	7.30611
0.803132	0.603831	0.843298	1.30812	0.23902
57.03562509	57.321921	45.73364299	53.60484	76.0455398
206.816	181.659	30.6475	85.025	3.73903
53.0638	44.6643	8.0779	18.448	1.71949
0.716659	0.657273	0.545862936	0.769278	1.575627
0.329308	0.0937356	0.36222	0.368864	0.108756
0	0	0	0	0
45.05376305	43.1182116	47.31290281	45.82289062	50.12141
9.45405	7.19196	9.458091763	7.3916	64.24189
9.45405	7.19196	9.458091763	7.3916	64.24189
0	0	0	0	11.37042
2.592279682	3.613393727	5.021865617	5.961054864	1.744653087
0.1844455	0.226369	3.27355	6.66032	2.89973
4.042107	4.60021	4.825863	4.382906575	13.7676
61.68423565	56.22745	46.25964633	51.45569527	18.40631
0.562335016	0.166719023	10.380967	25.81611	18.78709
0.000339233	0.123846	0.162117	0	49.5583
12.74249	12.507832	14.8978	15.00984	14.94333
0	0	0	0	0
16.046278	12.87540612	13.03475236	13.08585055	1.64887
16.046278	12.87540612	13.03475236	13.08585055	3.219515695
16.17644	20.3969	11.35345	12.37006	139.03957
16.17644	20.3969	11.35345	12.37006	139.03957
11.6203	11.1602	14.3912	14.2686	20.95851
10.48900015	9.946968849	12.60423702	12.0973609	34.927
34.63337	28.78107	5.27095082	14.06841	1.13012583
34.63337	28.78107	5.27095082	14.06841	1.13012583
7.37106	6.24359	6.86604	4.84556	2.04312
54.360618	59.36218664	45.575015	52.01335	56.0211
54.360618	59.36218664	45.575015	52.01335	62.15610554

54.360618	59.36218664	45.575015	52.01335	62.15610554
4.48432	7.08348	17.6053	9.56357	0.212479
169.4700059	164.2904342	119.600211	128.275738	31.8922
169.4700059	164.2904342	119.600211	128.275738	0.729694
3.712605729	2.804222189	23.05171096	16.39410609	19.238604
3.712605729	2.804222189	23.05171096	16.39410609	19.238604
10.40499847	8.307222725	31.82497	19.48338	6.21869962
10.40499847	8.307222725	31.82497	19.48338	6.21869962
1.33196	1.09212	1.87351	1.55852	2.10648
43.9663	24.0206	230.245	223.337	74.036
67.96319	70.85722	85.02112	82.6621	42.44106
0	0	0.189249	0.516821	0.461695
0	0	0	0	0
0	0.0504255	0.0985694	0.100314	0.0607681
7.2835922	8.19591	7.668983	6.65252221	42.95712
7.2835922	8.19591	7.668983	6.65252221	1.67285
1.087210526	0.75638078	1.262115515	4.360428735	112.966845
1.087210526	0.75638078	1.262115515	4.360428735	112.966845
0.72979634	1.07556547	2.087611548	2.143197	4.29642
169.1879	203.3105	231.4779	198.4001	347.343
0	0	0	0	0
0	0	0	0	0
0	0	0	0	0.870942
0	0.0906865	0.17758	0.298066	0.0562052
0	0	0	0	0
0	0	0	0	0
0	0	0	0	0.151855
0	0	8.48255	23.0341	13.5378
35.33768	30.57545041	27.18073208	26.63305117	9.4616292
0	0	0	0	0
0	0	0	0	0
1.478449196	2.470293	0.570351	0.520653076	0
0	0	0.405651	0.852444	0.720132
0.278191	0.233575	0.319004	0.365957	0.0553376
0	0	0	0	0
84.04587351	95.16953468	86.58853961	76.17275113	17.94116
68.9082	53.6932	35.9173	33.0532	1.21089
5.03089	5.62423	8.56018	5.820805	50.0746
89.73690815	91.99977	85.02295	81.04862887	236.6353461
17.2739	18.6209	22.7982	20.1182	77.0277
89.73690815	91.99977	85.02295	81.04862887	236.6353461
0	0.068373	0.200646	0.0890462	0.081977
11.7351	12.54706	14.5336	13.27116	42.25729974
31.13402	58.7423	13.40872	16.53796	2.942147

0.471891	0.470286	1.30519	0.597618	1.12312
28.56749687	30.11117605	25.59152755	23.017932	75.53597844
0.41510192	0.0937685	6.99511	8.23885	11.8024
25.410611	23.736539	18.897934	19.87022837	6.858846091
0	0	5.78942	12.3428	10.2593
1.12348	1.37094	2.83237	2.38296	3.23657
7.03909	3.16058	12.01351	14.12463	13.5635
70.0905	77.1274	47.6184	49.2799	257.7515
6.040128911	4.144291332	0.429955451	0.936567	0
14.84302226	15.478882	10.848129	11.21105508	5.47872
49.23111512	40.72415912	38.16158731	38.71872722	6.66248
4.7654	4.66268	5.35162	5.162006	1.48947
43.9411	39.63678	60.7049	46.196	184.9399
8.044412176	9.71293	12.115441	12.25518857	36.80275
9.14E-05	0.000120775	0.000156978	0.000255471	274.6
10.20254	7.77825	11.57051	8.6481	8.08105
4.1473203	3.788999	7.78818	8.036220013	59.82516
6.284398844	4.458538427	8.13987386	6.205784683	16.59009755
6.284398844	4.458538427	8.13987386	6.205784683	16.59009755
18.58165	22.89867	28.98933	25.28269	70.6195
5.08059	5.69776	9.15429	9.2694	24.1193
50.5676	53.9339	68.6418	66.8845	641.21
0	0	0	0	3.77468
4.401981517	4.86003	4.12497365	4.727138	800.594
4.999489325	7.441511639	0.984734591	1.551840618	800.594
24.41926	26.06468	23.18702	21.74712	19.54724
414.311	417.31	541.993	422.798	244.998
4.429324932	3.674833	6.861355788	6.77637	60.90587915
4.429324932	3.674833	6.861355788	6.77637	60.90587915
6.76592	5.78858	9.00983	7.67756	20.077
0.362291	0.546725	0.0872601	0.117976	33.2653
3.58311	5.08397	5.22308	4.06064	23.2396
5.489039089	6.317455367	11.82835265	10.57692	77.3438
9.053355	9.021617	6.75577122	8.057964	1.89821
27.0869	32.6762	25.5076	26.7293	6.25006
3.514103666	3.116278887	4.84101894	4.934677939	1.730124992
29.80714071	28.59889115	29.11980122	27.5588911	38.41729
48.00283	46.46157	54.68027	59.73261	22.622262
5.2344237	5.679613	4.344563265	3.95661	2.533574168
0	0	0	0.231541	0
20.5828	21.7984	20.1961	20.2187	65.902
33.6728	72.5695	22.1673	22.4913	1.82833
9.65E-05	0.00360465	0.247615	7.84E-08	0.527304
33.6109	25.2094	4.92768	7.05342	0

8.70975	6.89258	3.80191	1.73514	0.554278
92.4193	78.7516	42.6171	38.3318	3.21041
726.126	625.4679	357.4110413	345.349	4.66259
18.18696792	22.12963407	27.54456491	29.36907078	3.90444
123.627276	122.326656	98.337284	75.3019143	16.03832
0.224949	0	1.90686	3.72357	1.94814
10.906011	16.58917835	14.167725	16.80278382	7.75661
10.906011	16.58917835	14.167725	16.80278382	3.44511
0.875682	0.873973	1.984018184	2.189659	0.252805
0.104399	0.103443	0.100195	0.136804	0
12.63742584	16.38968001	18.97220449	18.84153075	46.1
12.63742584	16.38968001	18.97220449	18.84153075	46.1
18.2889	18.902695	17.520889	19.231625	23.87851455
18.2889	18.902695	17.520889	19.231625	3.777251351
29.98005424	32.41548398	45.91304435	51.53806737	25.98785
29.98005424	32.41548398	45.91304435	51.53806737	25.98785
29.98005424	32.41548398	45.91304435	51.53806737	25.98785
0	0	0	0	0
1.08552	0.219963	0.635838	0.573782	43.5217
3.60172	1.09816	18.14	20.8026	0.0423949
0	0	0.628538	1.38353	0.354143
2.088348808	1.391982	5.938376	6.569434	3.934971
2.088348808	1.391982	5.938376	6.569434	3.934971
8.21563	5.90274	18.0044	20.6253	2.60624
14.865059	16.56489	17.00131	15.083197	50.78398
46.520615	44.89936899	36.162818	38.76738049	49.93544178
46.520615	44.89936899	36.162818	38.76738049	49.93544178
0.336349	0	0	0	0.202217
0.0826098	0.0839973	0	0	0
28.50564	29.00848159	29.53930223	30.13840004	56.34924023
6.42333	6.18788	21.3418	21.8495	35.2024
9.284983288	6.209228	13.787873	19.877078	39.00252685
9.284983288	6.209228	13.787873	19.877078	39.00252685
5.195989237	6.114333734	1.136056418	2.123065	0.320053
76.76961436	69.45760629	73.05700877	66.23560286	40.4710173
38.38169484	28.728321	115.2603445	85.59141144	30.15712
54.12851	47.39536	49.97948	46.61161	50.7652
54.12851	47.39536	49.97948	46.61161	45.24643663
7.204867524	8.363672	11.66674	7.337916462	12.235647
17.12322597	16.29079399	19.54377	17.02736007	47.64541073
6.14968	6.63017	8.33311	5.91075	27.370045
5.69167	6.63475	7.53461	8.36863	31.6948
12.525329	15.28181	16.26191	11.69121	16.17763
34.20737	38.22309	34.25023	35.73491	39.21288

7.36994261	7.098333918	8.519721393	6.918674277	25.68946184
0	0	0	0	0
5.51836	5.29002	3.87221	3.25087	1.07378
1.958808	2.6745	2.259359	1.135369	0.823664
0	0	0	0	0
25.77037	23.2332	29.90958	33.06207	145.035
3.358043	2.529921	4.850601	2.518982	11.109821
2.49986	3.32175	4.3112	2.21098	46.6816
3.37835033	3.706351783	5.76079613	5.434347794	9.889751
3.37835033	3.706351783	5.76079613	5.434347794	9.889751
31.58529	30.19924	29.95274	28.47238	16.82605
31.58529	30.19924	29.95274	28.47238	16.82605
72.98753	64.85532	48.7183	53.46707	137.6054
13.30264107	14.1343573	11.54694354	12.6488782	9.849108
24.263511	26.76419	17.332031	19.74717	91.00988
7.99131	6.19231	9.72256	7.29253	0.556211
5.24747	4.63149	6.14288	6.34941	29.5033
98.32738452	90.83894091	58.10413901	70.7745548	23.33142
44.54925644	45.38394733	35.69445487	40.7059381	36.107254
3.97834	4.94066	9.8415	5.88018	69.5265
3.90894985	4.624988189	4.1871887	2.823859433	1.23688
0.088722	0	0	0	0
68.2446	65.634	68.4066	49.90487	79.2226
1244.094	1341.832	91.4104	178.915	19.49203062
0.161399105	0.174724946	2.7258431	6.566028547	6.958813912
28.015565	24.49001354	36.88603696	37.36536903	18.11937603
4.196318	4.707353	4.431314	5.16379	14.96085
164.8938	178.2026	273.1945	211.3221	0.636065
164.8938	178.2026	273.1945	211.3221	1165.172
3.89E-05	0.528585	1.91E-05	1.21161	0.000120307
4.38685	4.4122	4.89747	4.8387	7.69377
243.4365	225.0247	42.02365	75.9582	1.48404
5.797675	6.456698	7.734203616	11.196304	214.5161
24.182486	24.019751	21.37713	22.758285	12.4693
117.3842433	116.34237	88.4929376	91.14843258	29.24154097
103.5917566	93.83301	92.27612134	72.7108755	123.6401
0	0.117887	0.0572802	0.0769097	0
0.569325	0.506957	0.560849	0.660285	14.9171
7.135604	9.045882	5.490431	6.112109312	0.953618741
6.79848	7.08325	8.91654	7.95276	10.8114
0	0	0	0	0
27.61856	27.2254	26.66083	28.03774	39.85457
22.782305	23.021709	16.812653	18.15981027	7.319363
67.9421	55.9529	72.4412	44.1334	1.54866

53.4014	23.5355	62.0264	21.5223	0
128.506	32.0736	43.1425	39.2353	10.3599
173.219	76.6087	135.222	135.332	5.64615
22.92834	32.70574	24.4392798	25.390138	10.0788
470.6217938	657.667621	689.9587737	562.053646	161.8715
19.58087	22.94536	26.11691	26.3348	11.87736
10.0237904	5.87243874	16.554107	15.8414855	17.22575
11.51621497	18.72654401	16.67925693	15.74075585	5.75161067
6.711865571	9.703215	16.1673	15.96718446	32.44004
1.81017819	1.646851	1.56733	1.038497039	0.597713
23.16730102	22.64243	5.7844739	9.30585	0.201308
56.79386	58.47389	21.0662	22.780814	2.472994
11.4072	12.4966	4.70999	5.04413	0.459298
31.10617	26.09683	5.120927097	10.177405	1.15213
2.379449	2.60271	1.66863	1.83711	2.23634
42.84479	38.470918	30.19798481	36.2373855	11.460155
0	0	0	0	12.13101
18.52356	14.59135239	7.276925	8.253820292	1.862711042
29.116	26.8182	12.477	14.2518	0.324533
21.0628	19.0605	7.68228	10.3871	2.27474
4.77632	2.42103	5.372320002	6.040662294	18.5886
92.70849	93.99317	113.14806	93.54201531	80.18404
10.879579	9.53097	12.914709	12.784797	35.10142
9.88724	10.48609108	10.09231493	10.90269	35.044383
15.6415	13.8325	22.0804	18.6616	33.3458
35.642132	35.32150103	24.325088	22.938207	62.2216
35.642132	35.32150103	24.325088	22.938207	4.816385924
9.36501	11.0315	13.6539	10.8263	44.58441745
3.3953648	3.95175475	1.812533773	1.709419307	0.954157123
56.60669555	54.91968	31.06145416	34.73445087	11.68546
77.15595909	76.15742189	87.4042	86.2749	137.8393132
3.22612	2.90701	3.37343	4.83756	30.5077
22.891536	31.85737	30.27525	26.16180492	0.46026
22.891536	31.85737	30.27525	26.16180492	5.02955
22.891536	31.85737	30.27525	26.16180492	10.66
746.4741	567.4064	197.1829039	223.1156063	13.51237567
23.034255	26.42592	36.4866702	41.38301	56.9667
23.034255	26.42592	36.4866702	41.38301	56.9667
20.70462	23.738	16.73654	17.45407	24.9794
15.760617	17.69638	20.911424	17.811845	69.3649
767.9170695	799.1293451	1224.981339	1091.500543	1060.500026
767.9170695	799.1293451	1224.981339	1091.500543	1060.500026
30.9106	35.7996	39.4039	28.2927	46.1042
29.793369	31.24514	25.13779	26.39384	10.47864237

29.793369	31.24514	25.13779	26.39384	10.47864237
86.396215	66.75006412	38.7218602	41.0358922	1.50967
54.90358066	59.0390103	53.03761778	57.33385797	56.9280402
54.90358066	59.0390103	53.03761778	57.33385797	22.42880459
6.823109	7.428405	6.757208	8.257612	1.443997746
6.823109	7.428405	6.757208	8.257612	1.443997746
228.1920302	210.9791402	272.58733	272.79345	126.4711
27.72138089	26.75573906	41.58416	38.87519872	90.9785
37.136	36.3431	34.6365	33.3331	36.57099
66.7237	62.2369	74.2344	69.791	71.0945
41.7394	45.28439	41.36547	41.39596141	44.847482
34.5207603	30.27109672	41.83890855	42.55863449	8.40987
34.5207603	30.27109672	41.83890855	42.55863449	70.66665
34.5207603	30.27109672	41.83890855	42.55863449	70.66665
30.2586	33.5442	39.93837	33.79478	88.5959
6.902399	7.82367	10.48833	8.90417	37.49285
0.659918	0.663324	1.26001	0.429019	0.369704
12.22573	12.1428	16.04122	11.86529	32.6567
24.04869679	18.831181	14.376978	11.80948071	1.47968
24.04869679	18.831181	14.376978	11.80948071	36.558378
4.133436097	2.990723672	7.440710156	12.42063	132.55661
27.002745	32.64403915	22.3723771	19.32873835	3.626933306
27.002745	32.64403915	22.3723771	19.32873835	37.953
5.133990721	5.076792526	5.099310964	5.036924077	18.5075
160.6595	178.8202	123.4345	109.82387	207.0268
6.586358038	7.380679	7.78905934	5.834996087	2.899901891
80.386114	91.33904	95.045057	89.2884	169.1717
13.1018	13.4446	16.5996	16.3833	34.468
4.7348	5.75195	6.9539	6.11186	50.0459
294.385	302.923	251.147	174.9768	140.4291434
215.903	196.053	268.549	239.519	192.155
30.70406936	30.89058299	32.89898831	27.19132757	88.8611157
7.584809	7.394987	8.6805	8.081761	27.34858543
3.6955039	3.512581644	7.032744243	6.541488	43.22809
53.0595	53.036	101.6619	75.4052	163.659
69.9407	99.4285	56.2938	51.4444	20.1052
7.69505	7.72848	10.754359	9.1074	22.91061
3.370641152	2.973631467	5.711134662	3.88045679	0.58461
11.418288	12.673228	6.72633	8.052557	8.70335
0.894318	2.37448	0.0061965	0.131503	53.5267
33.16021623	30.03864036	70.20578531	60.7792756	90.85426552
19.003383	19.17093254	16.94545698	15.20220156	20.91073
652.4918	719.8973	1050.001	1042.482	1019.810128
9.26489	8.25265	14.1901	12.6367	13.12148

29.560439	30.86577	40.55059395	36.000064	89.66726578
29.560439	30.86577	40.55059395	36.000064	6.40244
7.6982	9.24054	17.1658	15.8167	51.99240065
1.04276	0.862746	0.783863	0.989236	71.4969
29.560439	30.86577	40.55059395	36.000064	51.99240065
0	0	0	0	0
3.120447	3.10743	5.45554	5.48276	24.18626482
86.58770528	79.97718774	62.54500252	72.90835405	19.28520326
86.58770528	79.97718774	62.54500252	72.90835405	19.28520326
7.687146195	8.109174	9.34325488	10.025677	7.94E-05
7.687146195	8.109174	9.34325488	10.025677	7.94E-05
7.687146195	8.109174	9.34325488	10.025677	8.82828
1.371366	1.307932173	2.899293299	2.769193	19.47233164
0	0.356687	0	0	0
8.068244903	8.945414	7.728398477	8.045192647	2.5092683
8.068244903	8.945414	7.728398477	8.045192647	2.5092683
0.0221016	0	0.0976721	0.0592806	0
130.7442462	145.490497	93.1522381	94.13450947	113.338286
130.7442462	145.490497	93.1522381	94.13450947	113.338286
0.496450293	0.637674155	0.950516376	1.664766421	0.670766
75.8644	76.5203	78.3707	79.385	59.6689
20.63903815	21.38321	20.270669	19.77369008	18.069
3.594533683	4.253430294	5.047780005	3.36435	0.374318
3.594533683	4.253430294	5.047780005	3.36435	1.79169
186.8850187	184.4574906	95.48633605	71.9781938	10.26343
285.564	252.452	60.56618	57.78505	3.2135
11.9647	11.1424	2.15163	2.89226	0.582384
285.564	252.452	60.56618	57.78505	27.34508356
7.19638	8.42585	9.1127	9.09792	7.29086
1.08E-06	0	1.11E-06	9.27E-07	3.91E-05
6.06105	7.73188	8.816625	7.067935	18.9574
22.387399	22.01069032	26.3133	28.469277	16.77038397
17.550427	18.87261496	23.22105051	25.61720309	58.52312
6.540249264	8.610797774	10.5968	15.53369891	26.5356
11.418288	12.673228	18.9597	14.8604	8.70335
20.81454	21.482438	26.13106	22.87234	12.354796
21.21224546	18.40666766	26.26069	27.763763	38.29780802
50.2274	60.1121	53.007	53.2731	127.84
4.289	6.01954	6.49948	9.33612	58.9899
24.32103	26.07863	35.6798439	32.77761133	56.57990117
24.32103	26.07863	35.6798439	32.77761133	56.57990117
4.072133653	5.736271269	6.312908	6.211484812	53.53121645
9.51229	11.96993	17.32248	16.48657	26.324
21.81678	30.1575	41.2348	40.3955059	105.7540339

1.965041909	3.23542549	0.6420547	0.555436666	451.023688
11.51248	13.06968	15.29272	9.24052	47.82258
14.80967	18.05087	19.5592	15.56881	18.9985
47.447327	45.370595	56.4354	47.278786	61.85426
4.63E-05	5.21E-05	0.000213976	0.0709037	1.59E-05
3.837581522	4.763107636	6.886880635	7.294617122	20.52792
24.2914	24.19029	15.04151	18.26632324	8.479805498
32.9335	34.2441	29.15411	34.25771	67.32214
1.239994569	1.438229	0.240308314	1.05847244	257.19118
24.67658	24.66349	32.00679	30.87564	79.6029
3.237693937	3.608691352	2.649347	2.525731209	1.355364397
6.17878	8.56871	6.15942	5.58597	23.88483651
2.61377	2.17155	0	0.566742	0
43.0908	29.703	23.5234	38.523	2.58936
35.51752453	38.17820645	77.66805183	69.85147477	16.5299
26.01883086	27.14806	38.86375	31.1626565	71.2188
7.46607	7.36831	12.9697	11.4475	51.7669
59.4261	66.414	65.2821	56.5212	64.6985
36.95796517	39.608507	37.73445474	36.558124	37.11023534
36.95796517	39.608507	37.73445474	36.558124	37.11023534
9.604798677	8.547526675	6.658373	9.467014268	4.239965615
12.6285	13.2923	19.0706	17.1079	56.3608
30.25029	26.09972	18.614629	24.2662721	61.3569
0.438016	0.214602	0.102403	0	0.25367
28.56388	28.862633	20.2981	24.38604	27.47300001
28.56388	28.862633	20.2981	24.38604	27.47300001
75.49179898	83.6554251	32.60988	34.11097	3.97703
75.49179898	83.6554251	32.60988	34.11097	29.40058
6.763804796	7.34975	8.552615	8.6559213	20.5441
0.0297755	0.0301002	1.85739	3.79163	4.57979
1.284789	0.401028145	2.490619	5.3104	2.393638
3.6378918	3.571819	6.9914	6.85161	32.76898
6.44812	5.22293	11.38016	11.59247	7.16031
3.404402	3.568362718	3.883756	3.827410121	1.125350013
26.12178	30.133619	29.17745	36.40065	27.20906
26.12178	30.133619	29.17745	36.40065	27.20906
40.8113334	43.012484	43.146905	44.353669	63.8009
51.5794	53.522423	64.0778	47.54129	18.149
7.66978	6.44124	8.77211	6.63911	20.8701
574.08188	652.1521878	959.8071226	933.7057109	860.5485662
28.04636	25.48564	20.94483	23.21597	6.758040659
13.3251	10.0219	10.901	12.5044	8.57593
7.029704169	7.031101293	9.445956363	8.421258602	1.897173167
7.029704169	7.031101293	9.445956363	8.421258602	1.897173167

22.117	23.8395	33.9311	26.3082	162.547
339.3339	363.2505	209.4464	177.5183	324.574
4.274416	4.038534	6.473118	4.683704	2.375851728
15.4987	17.58038	18.09735	15.4457	38.7273
4.71317	4.40589	3.67363	3.3212	21.7908
63.47453221	55.590013	40.121585	40.94088709	9.16686
1214.15394	921.644971	270.73052	313.7501928	14.6935
0.579982	0.40677	1.55882	1.52047	1.11544
18.3636	14.5219	16.3465	11.9198	3.7479
11.88973	16.23963	11.70068	19.95551	39.6054932
11.88973	16.23963	11.70068	19.95551	39.6054932
351.9899263	360.09261	538.1247958	336.5646877	37.98195
351.9899263	360.09261	538.1247958	336.5646877	33.38044
16.01289	18.77459	13.73398	11.05879776	95.5671
219.3115916	227.4729918	210.5586559	171.9072608	125.1517
0	0	0	0	0
0	0	0	0	0
67.26717602	65.06720604	16.77526554	21.252497	2.205655324
11.21475655	11.71792836	21.54312327	15.90470829	59.91413387
28.535449	33.47297	24.471132	24.427578	44.32553847
0.611909	0.307046	0.196411	0.133789	44.4675
75.1848	122.961	74.0653	97.8574	23.3257
24.65082576	18.919797	10.26380432	18.74126406	0.465053
24.65082576	18.919797	10.26380432	18.74126406	1.972539
46.73432	63.964011	31.97838	26.130325	3.328347911
46.73432	63.964011	31.97838	26.130325	180.192
8.40136	7.76545	11.16075	7.70555	55.04809
68.038448	73.94321512	60.59806646	53.38665	140.28261
9.930442179	9.696949378	7.095345899	6.857488593	2.210922202
9.930442179	9.696949378	7.095345899	6.857488593	2.210922202
0	0	0	0	0.50919
0	0	0	0.382033	248.19
0	0	0	0	0
83.05975626	81.81862262	221.984737	178.8761471	73.8107
0	0	0	0	0
22.15877	22.15973521	34.878	31.93909	1.399463073
7.161819	9.7204798	15.62889	29.94565	27.59896
13.3823716	11.4217742	6.202540546	8.39902	3.191126455
39.977275	46.4580751	46.2704519	36.042387	19.59085758
39.977275	46.4580751	46.2704519	36.042387	19.59085758
0	0	0.598111	1.59076	2.16115
33.552	32.1902	31.0417	24.0384	31.757297
18.47467	21.23057	28.47594	26.45012	39.99966
18.47467	21.23057	28.47594	26.45012	39.99966

45.92363102	49.96736006	52.05511085	43.64570012	102.6725
26.54448844	27.28409695	31.0771971	31.8433875	45.9256
11.88794	14.276832	13.847945	12.22091467	31.13370694
11.88794	14.276832	13.847945	12.22091467	31.13370694
25.5873	22.9089	41.8057	31.6093	63.4652
13.66149	12.69001	19.75549	18.90609	14.57422385
13.66149	12.69001	19.75549	18.90609	77.4162
23.2087	19.0534	9.49868	9.31148	3.11425
31.06195	28.7894	67.02594	64.54915	25.36001
31.06195	28.7894	67.02594	64.54915	25.36001
33.02680383	28.10091424	14.92796909	16.24648858	7.18642
32.56755	36.77417	35.62434	37.303384	143.042
37.02487	34.62819	22.54968	25.78203	56.8124
37.02487	34.62819	22.54968	25.78203	39.08061
0.715228	0.424952	1.05395	1.01891	0.585549
39.84632	38.13931	30.34329	34.34187	45.34446056
0.915228	0	0	0	0.509695
0.0931606	0.0941481	0.0917162	0.1236	0
0.443463884	0.558725205	1.116160422	1.550202114	24.27054113
3.716761082	4.213030111	5.40754	7.430700154	6.311216
2.456385	2.603728	4.44963	3.8201395	31.78120994
212.729	244.269	408.721	300.395	55.1662
0.0867164	0	0.085839	0	0.106937
0	0	0	0	0
0	0	0	0	0
0	0.545714	0	1.40832	0
0.257017	0.0877194	0.169375	0	0
4.4997347	3.8190191	6.7294165	6.43242349	8.951535435
6.985553	7.09437621	7.83043972	8.37557583	8.854569
9.28226	10.62416957	10.48332	12.29621	68.32720447
9.28226	10.62416957	10.48332	12.29621	68.32720447
18.643373	27.709315	22.48499039	15.58811	17.22647
21.278758	20.324181	22.7006643	24.730819	47.83398
11.8349839	16.92702	13.64148325	12.97135015	21.72126
5.243841852	5.8271679	5.31234734	4.232557	1.20113042
1.658324279	2.75076	2.362378332	2.283845708	0.223286
1.658324279	2.75076	2.362378332	2.283845708	0.350234
12.49157536	14.75652271	11.69160128	11.63092411	25.5005236
12.49157536	14.75652271	11.69160128	11.63092411	25.5005236
28.3642	28.9129	42.1748	31.3546	55.3307
420.355	427.51	53.46403	84.4964	8.88076
28.8547146	28.1896029	42.9975847	64.97148163	38.71736163
288.54998	286.0205501	212.08707	194.98695	4.62838
288.54998	286.0205501	212.08707	194.98695	85.2948

22.86714915	30.25649	35.97003778	34.93544015	97.51133177
6.424628	4.63129242	25.51285271	27.1948	26.24040469
6.424628	4.63129242	25.51285271	27.1948	26.24040469
24.43990535	21.17826407	16.330371	14.88652356	1.94591
24.43990535	21.17826407	16.330371	14.88652356	4.377266598
0.263578604	0.234728272	3.134799129	4.7225132	3.110548594
0.263578604	0.234728272	3.134799129	4.7225132	3.110548594
4.82239	5.14587	5.7973	3.91315	1.93852
23.26231049	31.34467027	30.21956887	33.59149563	2.23258
23.26231049	31.34467027	30.21956887	33.59149563	2.23258
21.76157	24.47391	22.09390255	19.10355244	47.35840951
21.76157	24.47391	22.09390255	19.10355244	47.35840951
45.6866044	64.0700516	53.48307291	52.093327	26.54236867
34.313342	35.066457	40.21144	38.933772	57.2783
34.313342	35.066457	40.21144	38.933772	1.72472
34.33669	19.70679	20.98329	29.42963	1.1526
131.07	91.3439	130.972	98.1337	4.76745
20.31393	16.73498	20.80458	15.447232	8.452681
1.598254888	2.391034737	3.88919	5.038140037	26.45998
125.93381	121.44414	61.65943	70.23952	54.27272345
13.9489	7.65454	7.10962	13.4843	0.000260228
6.59199	6.04198	3.51364	2.83755	0.514162
2.75439	2.25182	5.57043	4.2703	27.8956
15.60064	15.31415	24.12431	23.25187	22.19631
419.7641162	409.570901	463.8871118	374.280683	257.5566
0	0	0	0	0
16.59867	19.09448	17.45854	17.99693	28.58134
57.39646303	62.05281286	37.18899393	37.67746345	105.40154
17.77701	16.25895	28.11494	23.41627	62.6916
9.44516022	14.79328	17.97848	22.72589	51.22056
9.44516022	14.79328	17.97848	22.72589	51.22056
7.641	7.11693	11.2408	10.7706	50.8829
0.722831522	0.715530877	0.726716339	2.859636744	6.065160067
23.32912	23.05065	30.27071	27.87291	39.6166
14.15312321	16.87563992	16.15758154	15.78895869	30.98948002
20.8159	15.4618265	21.81844618	16.127868	35.27327
20.8159	15.4618265	21.81844618	16.127868	6.66118
54.994345	50.48288	42.274322	44.27063	13.75446005
54.994345	50.48288	42.274322	44.27063	13.75446005
54.994345	50.48288	42.274322	44.27063	13.75446005
239.7207044	242.3476137	276.8696536	283.8585162	185.606237
239.7207044	242.3476137	276.8696536	283.8585162	185.606237
239.7207044	242.3476137	276.8696536	283.8585162	3.14148
0.562188	0.376783	2.43576	1.30636	0.528328

2.08581	1.14745	4.75518	2.15127	0.682686
0	0	0	0	0.5971
10.7620581	8.317837551	3.216459969	3.552968419	4.53295
17.224545	18.72767591	17.2955552	18.51979903	53.625723
0	0	0.115777	0.310926	0.558519
37.15763854	36.88397252	28.52437005	29.83584473	27.92454437
4.35571	4.363778	6.69966	6.169252807	1.80428
0.910329	1.27157	2.28849	1.67505	0.439411
8.639251956	11.164456	10.54653864	10.575957	2.3075497
0	0.573016	6.08377	7.23117	9.45536
8.639251956	11.164456	10.54653864	10.575957	2.3075497
9.41957	10.3913	12.5855	8.81938	16.6143
6.9091437	5.537988426	8.975084582	7.470905742	4.601402
74.64109	78.14382	143.7767	152.2682	110.3231
5.994513	4.794099415	3.390405318	2.554103005	2.132190845
0	0	0	0	0
38.16684029	40.32836284	39.36011819	41.71886816	121.3938179
38.16684029	40.32836284	39.36011819	41.71886816	121.3938179
86.43672	63.00749	18.493579	16.73537	2.651030043
16.0323	16.9726	15.6692	8.53341	53.3726
2.034367	1.772836143	2.105811931	1.789703168	0.900943
107.5394	103.2033	59.3033	74.203	75.01979
0	0	0.660879	1.4435	1.49567
0.2101017	0.2895616	0.409674237	0.048681746	96.4
15.16238	15.90534	14.15309	12.71282	18.9906
0.150098	0.00940268	7.38786	8.93901	8.02204
13.3012	15.7684	23.1751	16.0211	49.7766
5.535735	8.53204	12.35835	14.38587	24.24224
1.43119	2.30029	1.71892	0.447711	10.7286
52.59885	63.31239377	52.57694	51.11517	145.198178
0.396129189	1.546735	0.5603118	0.3300589	65.78296
5.6263	4.06974	10.13684	5.95375	39.7072
14.888379	18.938793	11.70116703	12.121757	34.40534
70.98485095	86.93136851	113.1321855	92.72038632	239.4602
22.47356601	24.3129201	30.67892635	25.4781151	59.4949
57.77202464	45.10422913	16.22474551	18.03827375	10.27483226
57.77202464	45.10422913	16.22474551	18.03827375	0.245568
6.7870326	5.385605878	13.15544632	8.575321677	7.225584
6.7870326	5.385605878	13.15544632	8.575321677	7.39864
6.7870326	5.385605878	13.15544632	8.575321677	9.52712
70.32845	69.62112	87.377581	67.58207503	51.3282
5.23949	5.3754	5.592930377	4.64213003	5.4217
70.32845	69.62112	87.377581	67.58207503	106.2181147
0	0	0	0	109.0283

0	0	0	0	35.988
0	0.262099	0	0	35.988
8.304948207	8.378478889	11.6053496	13.66676502	109.0283
1.16217	1.193521	2.043348	2.66128	2.53207
42.007907	42.88455	14.29992006	15.96344	2.126989
42.007907	42.88455	14.29992006	15.96344	6.27233
22.1163645	21.55913278	28.10373436	36.3232098	38.875
0.210959	0	8.60347	14.4547	6.80456
29.76876965	29.47269796	38.40281681	37.0029289	50.72427
29.76876965	29.47269796	38.40281681	37.0029289	50.72427
0.211357	0.215091	1.62773	0.276432	0.483335
0.499402	0.257881	0.747736	0.3346	0
4.71651	4.86934	8.79268	5.64517	3.25374
4.17326	5.97124	31.1487	24.9758	1.61263
29.11901553	26.76685084	42.03290617	32.45954604	39.2443
29.11901553	26.76685084	42.03290617	32.45954604	14.44484
139.268	159.1939	122.8125	67.6646	45.38228
22.97127214	18.98333735	24.77989299	20.927288	3.99033434
22.97127214	18.98333735	24.77989299	20.927288	3.99033434
22.97127214	18.98333735	24.77989299	20.927288	7.06709
22.82652636	24.46364003	25.15414659	23.18349885	6.224875653
22.82652636	24.46364003	25.15414659	23.18349885	6.224875653
69.80485329	86.38212578	103.369669	129.71603	87.18084255
37.8339	33.46	18.8263	24.1799	2.01671
26.5122	25.53216	34.3688	34.5688	77.3807
1.019655749	0.492100333	3.2764587	5.950441091	2.5140913
59.5266	73.38042	27.86395	28.565026	11.13480688
59.5266	73.38042	27.86395	28.565026	11.13480688
24.62137	24.42875	12.11801	10.2988	9.05447
0.169568	0.210587	0.659704	0.441497	0
6.31041	6.84703	8.5451	8.4838	7.1052
12.02626	11.40673	19.12631	15.34988	24.29549
0.170822	0.435467	0.639267	0.741819	88.2726
0.817525	0.649377	6.9492	17.9821	15.3681
10.205002	10.765491	10.057326	10.148458	44.0706
0.0920034	0.0610033	0.0588117	0.0397571	0.858593
10.05515	10.61431107	11.58808736	11.64417	14.0652539
10.05515	10.61431107	11.58808736	11.64417	14.0652539
0.318082	0	1.86723	3.61442	3.19938
0.421699	0	0	0.53759	0
15.394488	12.81071977	21.40639019	19.22896579	10.31124
0	0	0	0	0
6.68845809	6.69419252	8.66114109	7.502121186	6.91115
7.32914451	5.120028104	5.590276	7.709017956	9.24618

16.8254305	12.31988107	22.58204127	9.509776388	105.0381
16.8254305	12.31988107	22.58204127	9.509776388	34.1189897
16.8254305	12.31988107	22.58204127	9.509776388	34.1189897
61.05006369	55.18328	41.783485	42.35212394	15.00622
8.36025	7.80663	9.59815	12.06311	9.97679
1.55391367	0.698064	4.032171	13.47737	5.16312
0.611455	2.05979	0.577542	0.78632	7.28439
48.70157	54.52047	40.50996	39.71693	45.3793
1.538445837	1.820830023	2.304940313	1.837976335	71.720653
0.777452	0.357013	0.888833	1.65719	15.543
9.94149	9.43955	8.1479	10.05847	1.04117
4.93852687	6.80116	4.926056316	3.834299688	1.04512
30.750643	33.4932	24.76038842	27.07849	18.09728
0.446521759	0.232271513	6.346161	15.07101	18.3824
1.956052434	1.827514	3.946351	3.60789062	35.828706
20.01912	20.02157	20.8338	20.2819	24.5338
109.8095456	110.47785	90.19382571	83.70466464	100.0200186
19.13115	17.50362	18.823	18.69711937	1.14106
19.13115	17.50362	18.823	18.69711937	3.456895171
0.967185452	1.226485073	1.398248163	1.57025281	66.10077
0	0	0	0.105967	0.630016049
5.26195	7.23019	6.28753	12.802	40.1475
0	0.430075	0	0	0
35.42146668	32.76175286	32.78899165	19.58553966	5.8221
16.174926	16.925	20.47597	19.072707	38.06613111
16.174926	16.925	20.47597	19.072707	38.06613111
10.628157	11.53635369	7.126822	7.547675	6.346997705
0	0	0	0	0
0	0	0	0	0
1.42972	1.8575	2.44847	1.29147	8.23927
11.168968	12.94443	10.082251	9.515512	15.66078
35.52689	34.36631	27.2604	25.40125	49.00368
35.52689	34.36631	27.2604	25.40125	49.00368
28.0783	27.74765	21.07413	23.8826	4.40326
0.338379248	0.167652946	0.378579316	0.723635064	88.8132
0	0	0	0	0
36.16915024	33.66212002	23.49253	24.75474	8.114050021
36.16915024	33.66212002	23.49253	24.75474	8.114050021
4.68207	7.02552	4.18048	3.96579	2.06067
52.585007	50.59729679	52.344261	44.85714901	14.760114
5.121081	6.95947	2.897782	3.726111	182.1726
27.304426	28.81635266	32.9020607	31.57861003	21.09710694
27.304426	28.81635266	32.9020607	31.57861003	21.09710694
30.7233542	28.7376412	21.50385942	22.07632	6.548139846

30.7233542	28.7376412	21.50385942	22.07632	8.714587
0.0809188	0	0.164047	1.54068	0.705429
1.56312674	5.296931562	0.041514854	0.110847038	580.4898243
1.56312674	5.296931562	0.041514854	0.110847038	580.4898243
0.632049	0.570581	0.617824	1.20444	0.334919
115.41627	114.378892	148.99593	123.16198	109.3855
41.04551	37.47087	43.85082	41.94222	30.5544
0.495824201	0.49844196	0.662647861	1.265945281	73.37956
3.695581319	3.911053	5.250203102	5.473154954	60.96612741
4.177309	4.33465135	7.70954	7.091431464	60.96612741
0.351499	0.35542	1.36181	0.229593	1.02834
0.210207	0.427946114	0.390075234	0.539972	2.769774
0.665724	0.336534	0	0	51.5358
0	0	0	0	0
0.195617	0.065222	0.569573	0.16996	0.39211
85.09476	87.04699	93.74625	97.32634	41.67696
85.09476	87.04699	93.74625	97.32634	41.67696
0.819659	0.823551	6.33427	12.5886	8.2308
0.645525	0.946033	0.638741	0.388175	0.140741
166.4788147	158.07249	165.7644602	163.65247	60.8291
33.89249415	33.795735	29.39308085	34.67758	95.0867
0.718687428	2.044465725	0	0.095192232	166.2967645
0.196039	0.0553821	5.11101	14.30485	12.1838
1.43738	1.29272	46.0261	94.6506	118.166
17.206631	16.648851	24.61687	26.112188	46.91356
42.280834	40.36986	46.500781	37.4938	57.221011
28.459	25.4002	12.7007	11.3367	0.786047
6.315072902	6.492660408	13.83140441	18.662141	18.72423
0.091764	0	0.000160399	6.93E-07	0.759561
2.66624	3.16903	3.72037	2.58469	5.76653
2.54972	2.90523	3.73629	3.86386	7.46314
598.895	700.5326	384.9597	364.0935	122.2013
598.895	700.5326	384.9597	364.0935	308.312
3.699209137	2.360586631	4.941173949	4.485929394	2.695655208
0	0	0	0	0.302695
39.56161634	44.63693508	95.39658848	65.64755878	7.211246
39.56161634	44.63693508	95.39658848	65.64755878	7.211246
74.384486	83.41346947	113.7064068	114.2199743	74.7969
0.842625	0.3329406	1.200501351	0.949738318	1.100709733
4.34824	3.59713	6.0412	5.10434	38.4468
1.660642	0.578194	1.682572523	1.97098	1.730206122
25.5391767	24.97162087	10.64342935	13.33688687	2.245727
0.0768291	0.0776589	0.0374311	0.253733	0.0896288
0.457682	0.31715	0.41247	0.640112	0.440181

0	0	0	0	0
7.41168	9.26706	8.56464	3.86772	10.92660148
0	0.033308394	0.732170081	0.842588212	0.114284
0	0	0	0	0
0.167468	0	0.162282	0	0.194499
3.80201	3.99294	2.28708	6.17654	3.85716
0	0.428712	0.138277	0	0
10.8306	10.3497	8.70106	8.93806	2.23212
42.87344625	44.67440543	41.96042711	38.19847738	0.629922
42.87344625	44.67440543	41.96042711	38.19847738	6.07014
42.87344625	44.67440543	41.96042711	38.19847738	19.50858
0	0	0	0	0
5.274664	5.964	12.53498	14.31488	80.6446
8.31248	10.92216	11.59082	12.46226	13.2414
70.0405	77.1913	107.9142	83.3764	190.3599
214.084	262.453	178.914	168.843	177.092
14.59558	14.81931	15.36431	16.412945	16.51711
0.126974	0.0320191	5.66712	13.55647	22.2553
16.89469	17.21811924	12.51739165	10.6617354	7.406249
0	0	0	0	1.58095
0	0	0	0.251978	2.445170072
0	0	0.140218	0	1.87811
22.43067983	26.1088338	31.99809	28.56324569	95.71292063
61.3819	67.0803	113.18	90.4803	130.521
53.45	62.453	52.3166	47.144	65.3933
67.50025	56.98105146	85.6812	74.97527	74.8943
9.40902	8.3152	16.09813	13.67812	32.13719
32.54803	36.37752	32.9911	28.77609	38.254564
6.381373	7.942095005	6.356615848	5.13276794	28.228912
6.381373	7.942095005	6.356615848	5.13276794	28.228912
2.02624	0.170382	14.1946	21.3917	85.7346
3.0494	0.764711	27.9235	34.2531	153.1051
9.590706268	8.322339	12.71422988	11.603876	11.3971907
23.32926007	22.98690735	8.579446662	9.404823132	19.417
8.753377	7.89349	8.739958	8.664618	19.72506095
8.753377	7.89349	8.739958	8.664618	19.72506095
26.35544821	26.84141462	28.87119514	31.15082742	64.13297494
20.735322	21.51523	25.703896	28.15461	5.264228
20.735322	21.51523	25.703896	28.15461	5.264228
14.780948	13.934359	15.745885	15.133263	30.53893
0.89106841	0.610917593	0.812346494	0.893993345	23.034036
49.18635069	52.2194601	47.81093	48.80753	70.7602
37.17178646	37.99287643	26.92593698	29.1700098	12.265811
54.89945333	51.77589845	48.82982782	50.77425179	63.4820842

13.894093	14.14000002	18.862992	17.50105	69.57310353
0	0	0	0	0
0.104796	0.0264247	1.33177	2.1306	0.736676
0	0	3.943884961	8.76186	3.55505
20.04235	18.57943	15.806847	14.51210272	3.971181153
20.04235	18.57943	15.806847	14.51210272	3.971181153
3.3325647	3.053261537	4.81048803	4.677337654	1.364252599
0.204455	0.127183	1.43779	1.57705	7.30215
0.735533	0.43855	0.159443	2.46612	1.99385
17.260645	10.256305	5.395010487	6.259163197	98.08673
29.56717	27.21012	22.19195	20.7583	14.53151946
26.1957	28.7408	31.3864	28.9641	26.7715
7.036918582	7.365824899	10.99435886	8.699180767	25.50565
90.81504648	76.85779107	53.61728475	56.0778039	29.272302
90.81504648	76.85779107	53.61728475	56.0778039	29.272302
9.85697	10.67418572	13.445013	11.74979913	26.2328
8.777651	7.53814	9.54153	9.468028465	19.07074097
502.504	547.169	819.032	791.63	739.4031896
63.20675	62.01566	49.26244	49.58516	37.45967
7.55402	1.792415	18.63934	23.9086	8.51878
11.614749	12.41592389	12.57702266	11.29414076	23.42525032
11.614749	12.41592389	12.57702266	11.29414076	23.42525032
11.614749	12.41592389	12.57702266	11.29414076	23.42525032
11.614749	12.41592389	12.57702266	11.29414076	160.8935
0.585671	0	0.535719	1.50829	0
3.64601	3.991512542	5.2861	5.606467	53.4793
12.42205	20.44876	1.02633	0.951486	3638.340035
6.4098119	9.71816143	5.838574048	6.367655251	10.25218849
6.4098119	9.71816143	5.838574048	6.367655251	10.25218849
27.756646	27.319649	28.36389	29.96818	96.25673492
0	0	4.38772	7.07841	4.75377
0.199472	0	0.189816	0	0.462751
0	0	0	0	0
0	0	0	0.261568	0.233577
90.6871846	99.1367113	87.0891131	81.5694444	16.52235
90.6871846	99.1367113	87.0891131	81.5694444	16.52235
21.01437	12.59980442	24.0409085	33.64084	11.21233
1.98088	1.693911955	2.3419167	1.94757	5.03944
18.31592	18.7549	13.93235	14.74123	6.71361
9.40236	10.65172	7.65578	9.36571	63.1245
15.59918003	14.97280509	11.73805613	12.34615971	3.982860969
15.59918003	14.97280509	11.73805613	12.34615971	3.982860969
2.45245366	3.915846005	0.334194843	0.186475987	1773.4791
20.61299047	24.33506845	26.14167736	25.57904688	1773.4791

20.61299047	24.33506845	26.14167736	25.57904688	15.6851
44.44493	55.0323	52.94523	46.04708	106.76812
18.36113	21.6874153	56.74193	55.06679	23.48309
33.79323	52.84708	84.4449	59.03661	39.40172458
45.74954	47.05534	27.54513	31.14628	69.95568007
9.05216	9.60034	14.5715	6.47376	7.484457466
9.05216	9.60034	14.5715	6.47376	7.484457466
23.8183	22.44074	28.3878	23.3332	40.4363
19.95955	21.33891	25.61074	16.597279	72.38125576
19.95955	21.33891	25.61074	16.597279	72.38125576
9.52326919	7.849660351	13.76444923	15.60126999	62.05902401
1.49615531	1.971071104	3.733356	8.959770076	117.2967377
17.21313	21.82654	13.516758	10.02090141	3.513690496
0.090750084	0.034883609	0	0	1.996878153
27.13549291	23.58638828	24.8632686	26.99853	9.471600204
1.24914	1.90613	4.00621	3.15391	16.83642
9.058505	9.528793	19.68052	15.3709453	29.81102
20.22885	23.75341178	22.36463487	24.093708	16.103819
0.0341632	0.0344989	0.134254	0.089586	0.040487
22.43147309	22.00440547	17.522298	15.614025	8.243682766
90.4448	76.2088	97.5589	82.50895	45.76817
5.78166	2.92489	8.86813	7.28181	8.80379
0	0	0	0.0565578	0.0513671
208.766507	218.753883	181.9989905	164.240409	147.0024
208.766507	218.753883	181.9989905	164.240409	147.0024
175.8309	159.325	207.3514	161.5106	111.7415
9.74813	8.29428	13.42453	13.6296	17.07480804
2.21069438	1.660557919	1.2663438	1.122848866	0.0658154
0	0	0	0.419153	0
1828.73	1767	2059.51	1735.13	245.186
24.454336	29.54868	19.038928	16.47100223	5.632676002
33.98466	52.3033	41.77253	30.15062539	6.974709
249.613008	255.345842	201.3372	181.01259	210.46396
249.613008	255.345842	201.3372	181.01259	210.46396
0	0	0	0	0.489285
0.473568	0.237563	0.457756	0.511838	0
17.48087	16.23573	30.83896	30.13437001	27.60011214
23.10738	23.61582	20.585951	20.89373983	7.695358012
88.1315	115.3096	121.1012	104.3555	359.1332814
9.08428	13.1022	24.2578	14.351	29.7637
6.96378532	6.5091073	9.535060858	7.949531793	34.56671
43.87248	45.279622	49.3259	51.95458	46.6205
2.023985	1.63608163	5.62863	4.50658	22.4648
13.85108177	14.85490749	18.66780469	19.69891039	39.96556

41.575957	43.94506896	48.6475	47.23154	99.83953
15.06767748	14.93905702	10.79742573	10.30071726	4.10629
2.3144	2.732998	3.791075	5.90759	476.4276
35.2385	35.1562	17.2855	17.0949	43.40707
29.9786	32.69638	42.8566	40.6868	94.0288
0.159206	0.0796165	0.461421	0.308473	0.784587
0.00032218	0.0413123	0	0.201639	39.6907
0.051743	0.44656	0	0	0.00178322
0.698952	0	0.443643	0.299922	0.000149804
0	0	0	0	0
0.0917886	0	0	0.24742	0.114683
0	0	0	2.64126	0
0	0	0	0	0
12.7117	11.5004	22.8086	11.954	4.73942
39.43754562	36.85470018	28.65198522	26.80103001	10.2354
39.43754562	36.85470018	28.65198522	26.80103001	10.2354
242.5312956	176.7244667	74.30514675	95.23089872	2.770594378
1.51657	1.57413	5.9229	4.61037	1.00032
0.120324	0.0796196	0.764636	0.204466	4.358488
0.110407744	0.259150329	0.110303677	0.114389954	25.62970531
3.3632631	2.873502365	1.42660944	0.3457431	5.90E-06
7.60E-32	4.81E-37	4.28E-39	9.99E-24	0.480977
0.112008	0.623172	0.164722	0.368883	89.5541
4.81173	5.34539	6.81473	8.55502	16.0068
17.234727	20.87381383	18.3375334	18.803777	24.2463
21.75175	22.9459	26.8714	21.351339	27.71563
44.5361	33.9935	48.4809	48.9006	113.923
25.81898	23.42556969	29.290145	36.065791	92.8506
0.416928409	0.827323757	9.51319764	21.99212472	0.979034
0	0	0.170997	3.79E-09	0.488236
24.23541512	25.34841255	27.72911485	31.41444994	56.43602131
30.4796	21.9175	70.602298	64.346451	48.1359
21.06945	18.869211	11.93922216	11.88420214	2.01833
5.468218592	3.9825301	5.4394721	4.421568	0.917565
176.565	187.2681	203.9582	199.3825	208.3639
8.26956	8.305715034	16.82137	15.26561693	35.53855
8.26956	8.305715034	16.82137	15.26561693	35.53855
2.361327	1.203333595	6.75899444	8.52522	3.62646
4.086089858	3.506495008	3.732712103	3.677242581	0.747094926
4.086089858	3.506495008	3.732712103	3.677242581	1.283104881
4.086089858	3.506495008	3.732712103	3.677242581	0.830102577
0.000452153	1.89E-16	0.000712644	5.52E-48	2.60E-06
0.466616034	0.513046945	0.858784415	1.1669277	2.727840157
4.9805	2.41702	17.3474	17.6794	5.39633

0.997471935	0.917248	3.6484267	3.941894	1.056611145
24.86043	30.504203	30.26444501	31.86099	112.668
24.86043	30.504203	30.26444501	31.86099	23.3208
1.01E-17	4.03E-07	1.94E-11	6.84E-11	7.13E-06
1.114835847	1.52802218	0.725008217	1.002772417	0.374308
518.94939	566.10072	46.57541194	89.780709	11.316
37.5962	34.6632	42.7116	33.0817	75.3175
5.878209807	5.84925097	4.823929133	7.5005615	1.881079842
53.38456906	47.49654847	40.926004	34.497005	14.35166
53.38456906	47.49654847	40.926004	34.497005	14.35166
53.38456906	47.49654847	40.926004	34.497005	14.35166
11.347	13.5044	20.2434	16.4113	24.1174
27.20088398	28.940194	31.47655608	25.98015	31.184
27.20088398	28.940194	31.47655608	25.98015	28.59283
24.0117266	25.078926	21.77876068	23.16977409	11.73993767
30.84385457	35.09471417	28.64266081	26.2646802	53.31964176
263.2136	305.53361	157.33577	140.35665	311.80982
26.156619	24.5078684	46.96507849	50.71207683	97.677563
5.220831646	6.063558961	8.939595062	6.34700323	23.7165
25.28295	34.98331	43.82418	43.25785	35.51157265
25.28295	34.98331	43.82418	43.25785	55.9813
3.262325924	3.373347	6.3033	7.077396274	16.03863
1.995865	3.721122	0.911886267	2.673602	457.8143
3.769182	4.426427	7.28207	8.1634	61.95096
104.21147	115.48021	185.15	199.2541	182.9692
15.17872	13.428	15.19551	12.556477	10.6431
13.524727	16.45525	16.921	15.26504	5.86904
3.059630471	3.04544996	5.711176	6.643896	7.330998
0.233785	0.278305	0.350168	0.46949	0.281144
0.0388834	0	0	0	0
5.16943	4.61486	5.18729	4.644911	33.5906
10.0105	9.44105	9.95698	20.6957	20.9479
0	0	0	0	0.396503
35.79913	46.633	37.02823	34.15652	66.177
0	0	0	0	0
9.049183494	9.664699409	7.747555418	8.790385034	72.67091
9.20006	7.91029	11.2805	8.15037	44.67594
9.63278	11.7945	12.7918	12.8325	44.67594
1.0200355	0.9659532	3.133945	6.023929	2.684977387
10.50262373	10.59396	14.371595	11.89344906	16.12269989
1.00E-06	2.04E-06	0.0999215	1.47E-06	0.000197757
0.580409	0	0.57954	0	0.346665
0	0.120066	0	0	0
7.35654	7.58732	10.0020551	10.83219817	23.7656

47.13021	44.49473	39.56219	39.51216	15.44485
6.76879	7.814766	11.119735	8.40153	0.45349
6.76879	7.814766	11.119735	8.40153	49.88408749
0.80155	0	1.52764	0	0.48016
7.961038603	8.822636975	8.092575	9.178157	38.47062
7.961038603	8.822636975	8.092575	9.178157	43.61872
2.12231	1.91739	1.09412	1.56571	79.2515
26.86586	28.43729	25.18282	24.520972	79.2515
455.847	436.419	569.147	390.131	287.407
12.98431174	14.49064101	10.48763867	10.26160254	1.465234855
12.98431174	14.49064101	10.48763867	10.26160254	1.465234855
11.06264	10.80126	15.17867	14.86542	43.15196
126.3691129	121.581722	136.3484171	151.1502179	61.5061
0	1.26577	2.34152	1.0796	0.981685
22.75064	23.67079	28.4353	20.11114	20.50383
0	0.0723573	0.140562	0.142273	0.130457
13.20239856	14.7756443	39.21626198	34.09555	5.499668
7.754769	7.796612	12.720543	12.651027	29.7474
16.46141	19.9385	22.77407	19.24889	39.4586
39.65733	41.80113	60.66595	56.82052408	3.81E-05
39.65733	41.80113	60.66595	56.82052408	23.0905
39.65733	41.80113	60.66595	56.82052408	17.7855
60.58897	37.61044	57.52846	45.49099	9.50343
17.33635806	15.742087	19.71298	16.59959006	20.56838
70.28759761	40.912	39.0008759	36.15267196	1.78832
0	0	0	0.384711	0.231847
9.93349987	7.329371588	1.0877751	2.459687385	0.517469
0	0	0	0.935646	0.833931
9.7539485	9.64150508	9.952724	9.7871585	8.61026
794.427	854.454	1382.98	1331.28	1567.45
35.33840082	38.82796	45.84795092	42.88839	75.93175137
0.0963442	0.243591	0.282247	0.381705	0.113456
15.170492	16.978101	16.88801	14.467196	44.54537
15.170492	16.978101	16.88801	14.467196	44.54537
0	1.15E-07	2.82E-22	0	0.203396
12.04409	12.14476	14.58532	13.22694	39.7928
15.4975	16.0841	22.7422	19.0055	94.0219
27.79875569	34.003023	39.862215	33.4577603	9.373362009
27.79875569	34.003023	39.862215	33.4577603	10.07765
9.05124	6.0557	0.0127527	6.06759	0.00967365
195.003	225.176	335.609	270.107	649.395
8.43152	10.9209	20.9235	15.8	6.4248
27.79875569	34.003023	39.862215	33.4577603	10.07765
20.80610475	8.15272316	15.36657546	18.95628986	42.63967683

20.80610475	8.15272316	15.36657546	18.95628986	42.63967683
20.80610475	8.15272316	15.36657546	18.95628986	42.63967683
20.80610475	8.15272316	15.36657546	18.95628986	3.49E-05
40.780539	39.288291	34.108827	31.134479	11.359149
40.780539	39.288291	34.108827	31.134479	42.2694
3.293741189	3.568234	4.4957253	4.170389	9.08183
3.293741189	3.568234	4.4957253	4.170389	3.52E-05
2.836181	3.067356	5.033683	5.064873695	39.3367
0.000684609	0.000227076	0.000261008	2.64E-06	3.40435
0	0	0	1.56185	0
115.12123	95.74906	51.39918	62.74069	1.03689
4.618782867	6.28384369	5.531138013	4.676299895	1.370976523
4.618782867	6.28384369	5.531138013	4.676299895	1.370976523
60.71343	52.74689	80.3165	54.20602	24.61743127
60.71343	52.74689	80.3165	54.20602	24.61743127
0	0	0	0	0
0	0	0.21599	0	0
28.5858	25.8541	26.8319	22.9912	5.97707
5.65753	3.90405	17.6122	17.6117	17.6794
0	0	0	0	0
0	0	0	0	0
0	0	0	0	0.226623
30.2378	31.27231	15.9968	17.3057	4.724930441
0.231877835	0.210966645	1.680148379	3.564327895	1.634365908
0	0	0	0	0
3.038600878	1.499313977	1.272144488	1.792158206	45.1186
1.660951	0.787415	5.42969	8.42086	127.5529
0	0	2.00132	3.29163	2.38983
2.840021	2.831552	4.364852	7.4607	30.56917
3.423340104	2.734189	9.695710001	14.75420001	53.33016974
2.47046	2.60576	2.37672	2.66612	122.649
1.239912443	0.915754749	1.0682332	0.995064405	0.448267
30.943973	35.4435273	37.727062	31.527308	45.30493
30.943973	35.4435273	37.727062	31.527308	50.8681
9.64757	9.57957	15.1276	10.96279	46.0621
6.17948	6.16367	7.73575	6.93427	17.0024
3.041839	6.13487	3.042507066	0.353701963	1.21008
14.60083	13.06967	13.91329	12.58747	24.6992
40.6679	47.0124	31.9893	32.9228	39.7201
1.677843008	1.523143	4.779853	3.311272	1.309880628
11.25959	7.19746	45.04	60.8757	99.6469
0.062196	0	0	0	0.151266
0.05905	0	0.0588679	0	0.0706459
0	0	0	0	0

0	0	0	0	0
4.39877	1.58E-40	0.0396688	3.17E-118	9.17968
0.55006402	0.129497	4.241704	3.583202486	2.03864782
0.55006402	0.129497	4.241704	3.583202486	2.03864782
7.715263	6.842714	28.00681	29.73872	14.82051
0	0	0	1.28501	0
0.0648068	0	0	0	0
0.440045	0	0.270859	0.545819	0.481589
0	0	0	0	0
0	0	0	0	0.179765
0.543077	0.2813623	0.797786	0.253458	0
0.543077	0.2813623	0.797786	0.253458	0
0.543077	0.2813623	0.797786	0.253458	0
43.14747	44.04662	48.94807	45.5913	78.2959
26.6067	25.27576	26.87838	29.5141	26.2467
7.511075207	6.060169911	8.6463274	9.399227961	7.470267
10.87706	13.55421	14.494651	15.90083175	43.6271
15.89411645	14.7407015	16.98344054	17.30772907	16.14354576
25.0215	32.2416	35.574	30.1889	57.5135
0	0	0	0	0
0	0	0	0	0
36.7844	34.16070679	30.66122336	34.49070006	24.95294
21.27253	25.23635	30.241399	21.37438	0.097768
21.27253	25.23635	30.241399	21.37438	66.9737
6.978506	3.26623	1.778692	11.154037	13.1827
0.222759	0.22849	0.454798	1.37574	0.000162287
6.46931	7.58988	10.5597	9.95472	28.1029
82.2877	83.8078	165.7261	159.4661	70.1155
34.95164219	34.8487124	44.39276099	40.05273565	58.55681543
0.164085539	0.322655	0.228484744	0.229399575	0.397164
12.24278134	12.757298	18.61922212	23.98859005	13.52375
1.42688	0.98915	1.49552	1.27184	1.35758
13.08939345	15.02531	16.72614428	13.38917519	8.19409
26.72003537	36.042831	25.18924	23.08619	5.761360173
0	0	0	0	0
0.516063934	0.473090955	0.5130497	4.330739	8.43885
0.516063934	0.473090955	0.5130497	4.330739	85.87192
4.3573	3.82E-05	3.61191	1.62504	2.45054
0.366044	0	0	2.7667	1.57187
0	0.20687	0.201068	0.18015	0.164977
19.80564447	21.74100057	20.48470002	22.433527	36.18422054
57.8466	50.25284	46.35864	49.33486	14.397461
57.8466	50.25284	46.35864	49.33486	14.397461
1.281519	1.768283	1.436982	1.253342	1103.561

585.049534	521.3840628	36.14942431	141.073378	7.40101
23.73083	22.5141	22.04436	22.12865	31.5259
19.057203	23.09071	28.12535	24.39634	215.1573
28.4904	24.3906	26.3512	23.0867	44.5357
11.4444	10.5266	15.3034	11.1564	28.5519
115.184738	101.1619311	79.389515	88.08128519	31.456788
115.184738	101.1619311	79.389515	88.08128519	31.456788
115.184738	101.1619311	79.389515	88.08128519	31.456788
5.13974	4.82463	2.39427	1.61404	3.34461
34.730039	39.26095253	46.49225	43.18786	57.6106
34.730039	39.26095253	46.49225	43.18786	5.54385
0.2943515	0.387766	0.172129602	0.162717	0.0839053
0	0	0	0	0
0.39957426	0.52990839	0.73522895	1.695901048	0.448834
0	0	0	0	0
0.39957426	0.52990839	3.6942034	8.951642	6.782883
0.675094	0.0829589	5.13327	7.42599	5.00596
0	0	0	0	0.097927
0	0	0	0	6.094601432
48.165518	41.369156	105.038492	104.60152	63.79438745
53.82589	61.762111	62.62379	47.53128	41.93614
2.292170553	1.931942006	5.409047009	3.89655363	15.19170335
0.452848293	0.905753135	1.71688044	1.880760767	0.913004239
5.237372	6.6317	9.958288	7.6854349	4.85729
5.237372	6.6317	9.958288	7.6854349	4.65749
5.237372	6.6317	9.958288	7.6854349	25.333057
12.26843345	9.228886659	12.386013	10.89045148	2.63915
0	0	0.734454	2.08659	1.09179
5.56409	6.27096	6.53024	5.46521	17.7718
15.40495	16.62416	15.55087	16.72828	55.1549575
3.74035	4.11575	10.63729	11.47320002	51.2798
0	0	0	0.0498327	1.597316188
0.20775	0.164327	0.8822106	0.824038	0.0568142
0	0	0	0	0
0	0	0	0	0
0	0	0	0	0
0.341732685	0.048706349	1.312683173	3.351293	0.925819
0	0	0	0	0
0	0	0	0	0
0	0	0	0	0
0.325723	0.0547875	5.89799	10.3628	6.03425
0	0	0	0	0
23.773797	22.4268	13.00200002	14.57460001	6.36405
0	0.146135	0	0.192731	0

0	0	0	0	0
0	0	0	0	0
0.158392555	0.2163601	0.377040281	0.339913958	0.185127
1.813198239	0.394801837	2.68547552	1.98916	0
0	0	0	0	0
24.77592469	30.052164	26.56807777	29.24864913	46.81948
24.77592469	30.052164	26.56807777	29.24864913	46.81948
24.77592469	30.052164	26.56807777	29.24864913	0.0486604
24.77592469	30.052164	26.56807777	29.24864913	22.007727
0	0	0	0	0
28.834889	28.7912921	25.42568979	25.85185595	44.38904242
0.904921	2.4074036	1.45954	1.016778	0.515841
0.904921	2.4074036	1.45954	1.016778	0.319944
0.122809	0	0	0	1.28665
71.1802	28.5101	20.5592	26.0274	4.44578
43.1472751	41.3046699	32.70648541	30.88651395	22.74130003
7.578364609	7.631603	10.98913	12.230598	16.99782
36.6093	37.757	66.0167	53.3811	44.6609
7.28938	8.33059	11.07682	10.28292	24.7939
0	0	0.889969	2.47732	0.628951
1.29238	1.18259	2.03403	1.6118	0.470014
0	0	0	0	0
0	0	0	0	0
4.37476	1.914385	3.45914	6.99355	6.842287229
13.0212	14.4085	35.4166	25.0073	24.0604
3.601090259	4.38795392	9.287580501	9.930944142	81.6595
0	0	0	0	0
0	0.608285	1.41543	1.97431	23.0058
176.501151	174.0380301	180.8321234	139.7569308	124.4978
8.30284	7.88723	10.11004	8.00224	14.7556
14.11247	14.77186	24.23518	24.72237	25.6716
0.206064	0.400202	0	1.50403	0
18.015285	20.36848	19.39733	18.08896	30.873083
12.36472	12.84457	12.67338	10.99849	27.6514
0.508389	0	8.86322	24.5379	40.0081
1.13377	0.37618	24.6323	40.4029	36.5177
5.60505	0.236036	121.719	256.793	216.595
0	0.0680211	0	0	0.816852
0	0	0	0	0.187011
76.98474006	78.204359	70.7296314	64.9240313	43.019003
56.12832	55.7339	34.62713	31.45547	17.86044
56.12832	55.7339	34.62713	31.45547	17.86044
11.296129	12.304889	10.519889	9.674486469	15.63984
43.9961	42.267776	44.718971	38.48776	25.145688

38.56572	38.93364	11.99062	18.97521	2.184631
2.23928579	3.19376	3.9279	1.799080009	1.699481529
4.09251	1.580886	22.2161	14.28984	0.330172
11.90908	10.01014355	22.08285	11.68327	0.407801
15.63740103	18.6422011	18.00240133	14.07400052	6.97442
15.63740103	18.6422011	18.00240133	14.07400052	8.16205
10.84718	9.745326	14.61065	11.064497	27.71587
0.324273922	0.135947478	2.174365	4.138074338	2.083670011
12.84250777	12.61421129	9.580064044	10.47720455	44.48036046
0.10214	0.0519869	2.29E-06	0	2.48E-05
0	0	0.27919	0.184451	0
0.736243	0.540104	0.407918018	0.168777784	0.574389
0	0.815443	0	0	26.9536
3.96887	7.03268	6.17422	6.36902	19.66251927
300.9159001	297.522745	305.3404	237.756	300.4645
16.21538338	16.118141	21.66925539	19.424386	95.61342183
343.3378156	598.25707	348.222014	384.7328	41.6442
0.261296789	0.498447961	0.0287404	0	69.00278365
76.73674551	82.095752	76.87188464	77.54824815	50.25838
6.73852	10.2111	8.69611	4.18207	4.50023
2.964617616	3.174202803	3.465210005	3.739722965	1.07759
0	0	0	0	5.90105
0.259033	0.174513	1.33062	1.5974	2.28393
13.3796	18.4108	99.9406	58.7788	3.734027
23.53685	22.06569	45.2368	36.0294	3.30016
3.92E-06	5.61E-06	1.88219	0.342009	2.20183
12.8271191	11.23344878	12.15279	12.22072	35.99826009
0.000423995	6.28E-06	0.000334432	0.000334009	0.000190856
1.94468	2.43258	3.35334	3.94249	3.30035
4.8809814	4.01055362	8.846971255	9.279772583	2.465693
4.8809814	4.01055362	8.846971255	9.279772583	2.465693
57.93988	65.26702	92.535223	89.36205	243.522317
57.93988	65.26702	92.535223	89.36205	243.522317
2.20873	1.73724	1.04305	1.68533	0.832695
114.084	76.3363	46.6933	46.5636	4.15261
86.44774548	78.55480826	51.89151377	56.0815793	47.07160224
4.89152	5.44589	4.45823	3.36769	23.4436
0.493492	0.998965	1.57258	1.94801	0.568419
8.43387	10.5748	9.21208	6.19108	3.82103
0.817204	0	0	0	0
0.573486	0.575318	0	0	0
0	2.25814	0	0	0
3.5684	4.03037	4.60471	4.4487	19.1776
42.8372	30.8571	10.5766	7.62464	1.9075

71.381	41.0659	6.40372	13.99	0
0.275868	0	0	0.17734	0.167725
0	0	0	0.0591817	0.506701
20.44572346	24.55486746	26.57355092	19.68030146	81.7346
1.4295	1.6368	2.57446	2.81986	15.283243
1.4295	1.6368	2.57446	2.81986	15.283243
10.02093898	13.999036	12.353328	10.590408	13.0733
44.1358747	48.83537741	46.5707801	45.38051	52.79686
173.39277	175.82499	139.09223	138.3972	84.024217
173.39277	175.82499	139.09223	138.3972	84.024217
0	0	0	0	0
0.2710794	0	0.5527258	1.736730189	0.868732693
89.02263614	89.70340796	141.705069	135.4922416	105.8881819
89.02263614	89.70340796	141.705069	135.4922416	105.8881819
4.311571675	4.433227611	6.945513051	10.76539099	2.18698
4.311571675	4.433227611	6.945513051	10.76539099	22.2679
4.311571675	4.433227611	6.945513051	10.76539099	22.2679
0.775409	3.52303	0	0	446.017
1.280570811	0.9401579	2.94199696	4.783899006	7.53239
28.5012	23.45367	8.87022	11.39816	0.616298
21.18133618	22.9703824	13.511451	13.772104	0.889648
23.9356	26.4009	26.8741	30.4794	8.62037
6.781757	6.746124	5.76535686	6.93888316	1.70558096
0	0.0332616	0	0.262532	0
25.803404	22.4856433	15.1632289	17.64457837	0.787363
8.67293	8.675669	6.586883	7.268178	2.759750191
0.960717176	0.946324794	0.929665335	0.795454481	0.174896
131.48654	120.95416	56.7561528	75.14402091	2.87923
8.62836	1.52081	4.59009	3.22194	17.4422
3.6676	3.01991	5.70469	2.67558	0.905178
1.65642	2.19544	8.57769	6.51707	15.0882
0.488552	0.15171	0.336494	0.8513	0.436281
1.24332	1.82487	0.121689	0.0822896	115.249
1.650216	2.0589343	3.072044	2.847312	12.7903
1.650216	2.0589343	3.072044	2.847312	243.927
31.74304	30.49712	30.31505	30.32085	51.3837
1.37123478	1.394357217	1.585376	2.6396713	28.72510127
1.37123478	1.394357217	1.585376	2.6396713	28.72510127
0.12542791	0.348967004	0.429276	0.342189073	95.3037
2.2116	2.18762	3.36206	4.48906	68.57450014
0.027815316	0	2.292770761	5.091042062	2.519848096
0.027815316	0	2.292770761	5.091042062	2.519848096
0.113208848	0	1.550803	3.386787668	3.006658049
0	0	1.414982637	3.684987908	1.376088946

23.46198	25.56003	28.09781	28.19779	50.3591
2.702271	1.611758	4.52636	5.02365	0.672378
2.702271	1.611758	4.52636	5.02365	80.97075
36.3193	30.783	12.5162	20.6331	6.88102
89.66410295	76.94040358	93.796967	79.919295	51.7275
0.149242091	0.14762035	0.06981495	2.497588341	74.90410686
7.625430011	8.573084	5.28197205	5.64959609	23.0809
6.58184	7.01266	17.7255	14.7659	24.666
27.28861964	25.29778	19.49157331	21.4113166	10.2739055
27.28861964	25.29778	19.49157331	21.4113166	10.2739055
34.2992	18.4249	52.2053	45.5994	6.09146
0.30271174	0.361817263	0.205998846	0.47354246	28.914439
0.30271174	0.361817263	0.205998846	0.47354246	28.914439
15.01692176	16.41118977	21.68493985	20.990195	54.232288
0	0	0	0	0
3.96851	1.324520295	5.533794	9.44311	10.6394
2.575865006	2.492048	1.828733	1.001726	0.755107
13.4229	5.56363	66.4442	28.63493	3.71613
21.0796161	22.1167499	18.388094	14.59480526	27.83803633
0	0	0	0	0
0	0.103107	0	0.542407	0
0	0	0	0	0
0	0	0	0	0
0	0	0	0	0
0	0	0	0	0.848975
0.305041	0.204756	0.786706	0.13269	1.19758
0	0	0	0	0
0	0	0	0	0
53.915	57.04231	43.32247	44.01917	91.37430734
3.939945	4.143075	12.918198	6.400079535	60.944833
3.939945	4.143075	12.918198	6.400079535	60.944833
12.08767216	10.669571	16.13622811	21.40365861	37.11734
12.08767216	10.669571	16.13622811	21.40365861	37.11734
0	0	0	0	0.117796
0	0	0	0	0
1.056329	0.402684528	1.007446948	1.685589319	22.71645971
46.775366	46.761614	43.47672112	51.297648	22.71645971
20.750302	19.3223731	32.599834	27.311274	46.76229487
0.397855	0.0552337	1.3702072	1.519780801	0.920564
0.397855	0.0552337	1.3702072	1.519780801	0
156.995214	155.6737538	63.68118321	80.54775287	55.580501
18.643681	15.117659	33.9962	21.08742928	42.5757
0.566538	0.284275	0.135206	0.925949	0.835843
5.649179	14.27992	7.37045	3.784535	0.874202

0	0	0	0	0
1.568149	2.5729	3.88336	2.694406	31.0803
11.99699	13.48522735	15.18393462	14.6859787	27.390439
10.75853818	13.31124	10.17123501	10.40808499	51.99050997
21.91893641	18.40887001	22.01193567	25.00965946	45.21761
0	0	0	0	0
63.0529	60.683	82.1124	77.4903	166.722
67.02209501	61.00988	64.309448	55.90881096	20.07961
598.811647	620.997038	668.255734	452.0756479	37.812
598.811647	620.997038	668.255734	452.0756479	297.371
7.48009	8.382392	6.25593	5.0870265	6.0355709
0.182441	0.392548643	0.1778	3.32599	20.11494
0	0	0	0	492.018
51.0388	59.29386	135.7905	140.3334	91.0339
29.34758224	32.45847114	9.835914051	10.2272082	3.034672107
29.34758224	32.45847114	9.835914051	10.2272082	3.034672107
23.14931767	24.22212873	17.06415237	17.69162669	53.81141067
185.1028344	178.1170923	202.499856	173.0109134	15.3940203
185.1028344	178.1170923	202.499856	173.0109134	103.2835
0	0	0	0	0
30.92031119	27.76195704	31.40916503	29.40550192	14.39077
35.22496	32.81644	24.96428	26.63016	12.02773
0	0	0	0	0
7.700528763	10.02344539	9.041685524	8.441003489	0
7.700528763	10.02344539	9.041685524	8.441003489	3.684290484
0	4.01E-14	2.31E-28	0	0.215908
0	0	0	0.730489	0
0.109629	0.537247	0.115502	0.119621673	25.77179
0.145768368	0	2.453465982	3.504127174	1.252784472
66.44458814	61.23529254	7.858944017	23.32995456	3.9128
25.54270053	28.10389	23.89428439	19.79210128	41.7713
0.691526	1.407825	1.675492	1.06811	95.19919
19.11067745	18.83391357	17.49450507	14.49857725	21.53717558
19.11067745	18.83391357	17.49450507	14.49857725	4.932918
19.11067745	18.83391357	17.49450507	14.49857725	4.932918
0	0	0.36736	1.07641	14.395
0	0	0.0459905	0.123807	6.32941
186.008	158.113	177.135	152.293	91.7579
35.0956	35.6265	30.308	33.309	28.41203
7.82423	9.48788	10.26422	8.30066	58.1599
491.775	477.869	552.297	417.981	313.72
8.2715	11.5019	14.1115	16.165	14.5517
63.5878	76.773	75.6608	67.1721	242.4672
3.25679	3.6438	6.31911	6.6245	13.8109

4.530080001	4.678563	4.985399551	5.252942	7.366641
4.530080001	4.678563	4.985399551	5.252942	7.366641
68.73700245	62.9060013	7.356393404	18.1017481	1.872944
7.784640431	7.9569	2.7491317	5.59577	1.466551015
0	0.0706085	0.138393	0.093378	0
0.0549649	0.0557474	0.0812992	0.109812	0.0664739
9.86774	6.95998	11.3798	8.74531	6.80433
954.205506	929.18888	1016.80968	736.259122	159.62446
954.205506	929.18888	1016.80968	736.259122	159.62446
13.908217	10.61809508	23.24073822	16.51100624	122.78704
0.406334	1.37763	1.62059	2.823101	1.35354
0.406334	1.37763	1.62059	2.823101	9.07E-05
0.3342251	0.16885159	0.941739	1.837155895	11.26633
0	0	0.0749587	0.100143	0.18125
0	0	0.238542732	1.020618766	3.37243
6.587965001	5.895175	4.363370003	3.7622926	2.566230577
6.587965001	5.895175	4.363370003	3.7622926	2.566230577
0	0	0	0	0
24.798659	21.15433	15.99609	16.34583	5.388501134
3.772009981	4.579726463	5.1327253	5.698254236	6.055856
0.410523459	0.369772532	0.323783941	0.240133963	3.284270342
0	0	0	0	0
772.57902	627.10605	156.71858	209.89002	2.71779
12.3833	10.9402	15.2191	17.7462	4.91362
28.812433	35.43989	35.93752	33.21250316	53.919
37.93830037	44.80599623	35.8199411	31.15026339	100.113411
0	0	0	0	0
0	0	0	0	0
0.125139	0	0	0.317179	0
0	0	0	0.0329699	0
0	0	0	0	0
0	0	0.776848	0.34852	0.31872
0	0	0	0	0
0	0	0	0	0
0	0	0.0311528	0	0
0.715906	0	1.56031	0	0.546925
1.03876	0	3.18164	3.76856	1.18877
0.0787205	0	0.0769604	0.206961	0
0	0	0	0	0
0	0	0	0	0.450238
0.299602887	0.129684341	8.276923965	20.24340492	23.93390519
19.053647	18.46001819	25.335394	27.41988	29.7567
0.284912	0.123239	0.0400317	0.0534567	0.0940189
0	0	0.215719	0	15.2767

25.046473	23.701424	16.88133969	16.905441	4.958383479
4.255121395	6.841705	13.129848	13.25560541	11.1204087
4.85775	7.11673	6.12726783	8.33418	7.765128871
0.175172	0.21976	0.47731	0.34327	0.108675
0.045495	0	0.222245	0.117827	0.0551879
0	0	0	0	2.75218
0	0	0	0	0
0.236818	0	0	0	0
0	0	0.247105	0	0
0	0	0	0	0
0	0	0	0	0.533723
0	0.0554539	0.0268629	0.0360585	0.0673707
0	0	0	0.0477811	0.0421838
0	0	0	0	0
0	0	0	0	0
0	0	0	0	0
0	0	0	0	0
0.0631763	0.126418	0.183672	0.249573	0.0731693
0.266581	0.423181	0.474627	0.584035	0
16.908581	15.19568197	20.901788	17.48278651	32.884355
12.6523	11.8993	15.21	8.98974	47.8509
0.520675696	0.869698604	0.188288785	0.201883644	111.343703
1.38E-07	0.205853005	4.06E-07	0.117658776	67.1372
29.46746006	28.04647	20.16121682	21.77536	38.56078
0.181161	0.0461346	0.13419	0.300218	0.106161
0	0.198209	0	0	0
242.5447464	418.1003875	465.6113882	362.7116787	169.0522217
51.71928	59.14359	40.9716404	43.073275	69.59832824
0	0	0	0	0
120.499	95.4572	76.5484	77.8351	6.51E-07
0	0	0	0	0
3.26091	3.73391	3.28663	2.76107	9.96631
0	0	0	0	0
0	0	0	0	0
0.384693	0	0	0	0
0.644118	0	0	0	0
32.1392698	30.6254483	27.54690148	28.21322726	18.87158
0	0	0	0	0
83.23601298	74.00422463	51.70396384	51.7100227	39.000913
83.23601298	74.00422463	51.70396384	51.7100227	39.000913
83.23601298	74.00422463	51.70396384	51.7100227	39.000913
2.70244	2.59792	3.94535	2.46594	1.2281
124.068211	163.0915006	153.62873	180.6218	22.4689
0.1800672	0.258489	1.95914	3.79873	34.8337

0.423134	0.0718296	0.0694371	0.740518	0
0	0	0	0	0
53.21462577	50.69682529	34.2192112	36.17651354	7.99279
0	0.465484	0	2.3646	0
0	0.0860031	0	0	0.303654
0	0	0	0	0
0	0	0	0	0
1.024257	0.54525524	1.226107	1.401262705	1.04396
0.0875692	0	0.258369	0.693851	0.427697
0	0	0	0	0
0	0.123251	0.119613	0	0
0	0	0.696381422	1.31676559	0
0	0	0.696381422	1.31676559	0.449735
0	0	0	0	0
0	0	0	0	0
1.24819	1.10863	0.814536	0.633122	0.142899
0	0	0	0	0
0	0	0	0	0
0	0	0	0	0
0	0	0	0	0
2.198072251	1.322139879	6.754207786	10.38683	6.888961065
15.06437	18.64533	25.26767	22.9409	2.71963
15.06437	18.64533	25.26767	22.9409	7.97668
15.06437	18.64533	25.26767	22.9409	29.26080225
9.190249	7.0959734	10.158986	8.239240335	7.91717
31.06935	23.63446964	33.3076417	42.986307	8.068423411
0	0	0	0	0
0.905564	0.77296	2.32816	1.93126	0.00327127
0.737323	0.170172	15.5626	38.29	32.9687
2.104053461	2.88005339	2.201235853	2.253359	0.167404
0	0	1.62647	5.30404	0.705441
13.3128	11.1189	16.6696	17.8585	45.3641
0	0	0	0	1.27078
0	0	0	0	0
0.206729	0	0	0.272638	0
0	0	0	0	0
0	0	0	0	0
0	0.157842	0.077011	0.309372	0
0	0	0.111363	0	0
0	0.469663	0.434599	0.599615	0.568945
0.634385	0.983446	0.444147	1.06226	0.127055
0	0	0	0	0
0	0	0	0	0
0	0.0727694	0	0	0

0	0	0	0	0
0	0.156096	0.440365	0	0.186142
0	0	0	0	0
0	0	0	0	0
0	0	0	0.316368	0
0	0	0	0	0
0	0	0	0	0
0	0	0	0	0.444701
0	0	0.217016	0	0.261851
0	0	0	0	0
0.63079	0	1.87E-07	0.120222	1.19718
20.035954	22.2818	23.64161	23.25097	50.08619
71.1677	46.9189	37.476	44.4106	0.980964
23.77632977	29.1891428	12.35977094	12.36750271	0
0	0	1.92179	5.70438	2.32883
0	0	0.827809	4.91939	67.185
5.237908079	4.541649	8.2480903	7.872171726	5.715841984
0	0	0	0	0
0	0	0	0	0
0.191504	0	0.0637696	0	0
1.15961	1.17303	2.04178	0.940662	28.4281
0.170097	0.0433433	0.211885	0.681067	0.25607
0.0417347	0.168865	0.456484	0.94068	0.305875
0	0	0	0	0
0	0	0.324495	0	0
0	0	0	0	0
52.59239	43.48562	26.45007	29.67195	86.08081721
0.190245	0.852081	0.640075	0.123849	0.464853
0	0	0	0	0
15.6447508	17.80744325	19.24286279	21.2534484	85.52636
10.1532	12.5139	13.9623	9.43916	29.8125
3.84429056	3.5996341	3.878252	3.52555197	0.580079
59.56091774	61.63378982	55.07913331	51.55443383	52.73228979
23.35084	21.12502	23.316136	25.25964457	18.07739218
37.13341	35.51705	30.62373	33.05958	43.77499
31.3404	29.2197	32.8313	27.8762	14.96
7.722798623	9.0336836	11.390247	9.422572328	37.6709
0.7327972	0.5744832	0.789679022	0.4670674	0.0726587
0.7327972	0.5744832	0.789679022	0.4670674	0.243268
15.31556	14.97082	13.00212	13.76116	9.08093
30.1846	31.0315	22.0741	21.8125	22.57167
9.5145319	9.32574224	12.26849711	13.29163304	24.759016
1421.6836	1363.77	1132.28767	1400.22462	574.8325
1.744744	1.691491	2.58616	2.731979729	37.47118

5.22588	6.03413	6.908534	5.57412	4.53828
678.9121222	706.4920803	1014.970219	953.7363964	1455.400009
0.293840479	0.500184	0.3075747	0.233567	0.378633
31.158068	35.14312	46.325384	49.570062	128.51778
35.9796077	44.7996659	36.80400898	37.43167717	40.0961
0.15368	0.0777421	0	0.101022	0
0.495555	0.504114	0	0.132801	119.074
0.0886351	0.806659	0.0616225	0	30.9862
25.72684	28.02738	22.02859	21.932515	30.75076901
37.35757	43.65175	37.09528	34.18263	29.0075
37.35757	43.65175	37.09528	34.18263	0.981441546
56.3305	46.6588	65.18820002	52.477	58.47414242
43.8255	51.3831	34.5204	32.5396	44.9503
0	0	0	0.0572922	0.0518609
0.051288447	0.0521416	1.403300116	3.125080728	1.57204
484.84902	459.41587	1526.434875	1526.71235	630.2459866
0	0	4.06188	9.38317	14.49716
12.9993	10.2567	9.09588	8.63094	2.12823
33.000513	27.63296028	34.14183016	25.34506598	58.53565
57.56351	93.17455	46.44371	40.78716	35.8877
0	0	0	0	0
0.0352574	0	0.801058772	1.534822552	0.90694935
1.27615	1.29064	1.16571	1.38173	0.838545
0.0461657	0	0	0	0
0	0	0.368116	0	0.146628
0.810984401	0.69944918	0.924904883	1.191954771	0.471929
52.2998	37.0588	35.6065	26.6159	15.2029
0	0	0	0	3.52686
0	0	0.0612734	0.121597	0
25.99142	26.46024	33.55165	27.07585	65.8362
16.90512	18.74574434	13.579819	12.12346329	4.74509018
16.90512	18.74574434	13.579819	12.12346329	4.74509018
0	0	0.38192	1.0168	5.05321
26.2583	25.0551	44.0566	54.9951	41.33
2.68072	3.50384	7.38407	3.91935	3.88951
0.808063816	0.994636304	1.537739	1.834993769	1.019240119
0.808063816	0.994636304	1.537739	1.834993769	1.019240119
96.89817	91.72338	90.70592	91.47029	123.188212
96.89817	91.72338	90.70592	91.47029	123.188212
96.89817	91.72338	90.70592	91.47029	123.188212
0.157145	0.16061804	0.926836216	1.640943	0.289523
0.536615966	0.407103025	3.746242048	8.374391629	2.967165003
0.536615966	0.407103025	3.746242048	8.374391629	2.967165003
90.79329	83.89316	108.27523	117.01897	6.82375

0.143842	0.0974601	0.943889189	1.208091544	3.00753
0	0.0471887	0.0461586	0.0618775	0
0.183678	0	0	0	0.212397
0	0	0	0.126827	0.112488
199.251	179.037	178.763	147.089	627.306
9.563199142	9.91311994	11.88037288	10.3940835	20.61189
1.16866	1.47237	1.86364	1.26555	7.94351
0.11727	0	0	0	1.75E-12
0	0.32892	0	0.107633	0.297307
0.153502	6.74E-05	0.121279	0	0.000698549
73.03289884	82.10170775	82.45024761	78.33557	159.2842
88.5225	98.9606	99.3367	95.6119	268.6177
56.76062797	49.86490222	41.71874891	44.73406436	31.24677017
0	0	1.81043	3.20922	1.22079
112.1078718	105.1540092	66.95986253	70.35768045	2.85285
112.1078718	105.1540092	66.95986253	70.35768045	14.76844099
112.1078718	105.1540092	66.95986253	70.35768045	14.76844099
25.85209962	29.37913306	34.021579	39.77732	81.9846
0.755853709	0.315173923	17.32969654	45.0726104	53.3021
0	0.057356694	0.748777672	3.369856437	2.09951751
0.03872108	0.201304126	0.996408774	1.640949	0.602121
65.6167	70.3317	147.9066	146.7565	102.731
11.52348	10.84433	22.26847	15.6988	71.2816
0.920598	1.04507	0.501443	0.888992	32.8999
27.2359	27.3967	29.377	28.0142	27.621
11.07740544	10.60020686	15.70901104	13.89840775	41.67450001
8.41891	9.81071	7.10114	6.50947	13.01076
7.71153	7.72953066	9.572174	8.87815	3.82965
39.71299652	37.083987	48.58242923	62.48774888	1.926492973
39.71299652	37.083987	48.58242923	62.48774888	80.76849
0.907292	1.69613	2.15829	1.64054	1.11239
11.67933931	14.719719	15.14321559	15.25260645	16.50450899
0	0	0.178268	0	0
0.184962	0.364944	0.68512	5.77544	38.3545
4.550960082	3.573623343	6.470235686	6.872012602	4.14423167
0	0	0.391759	3.16153	2.18938
4.550960082	3.573623343	6.470235686	6.872012602	0
0.417902	0	15.4696	23.1605	15.8353
24.66941	25.18978741	11.715671	15.915161	3.1872038
17.4005	10.316	13.2148	17.7348	17.5467
0.208818329	0.2674301	9.45351	40.48675	293.469
23.32587532	24.86773563	29.48510344	30.45361353	0
23.32587532	24.86773563	29.48510344	30.45361353	26.8233
0	0	0	0	0

0	0	0	0	0
0	0	0	0	0
0.514718	0.701669	0.936766	0.461793	2.18375
23.77632977	29.1891428	12.35977094	12.36750271	10.487379
26.09434246	25.99792882	18.16968108	19.01184796	21.55539024
16.3543964	16.01522	16.79733	18.62062195	11.31412043
30.55035	32.77843	14.40278	15.81889	2.18733
4.0737998	5.75006082	6.944934195	8.18898	3.14E-05
0	0	0.0876635	0.0586997	0.0544073
9.73912	7.04949	6.73202	8.7666	9.82658
0	0	0	0	0
0	0	0.0905281	0.121022	0
0	0	0	0	0.0966097
0	0	0.126939	0.347755	0.152902
0.214612881	0.072857056	2.075519705	3.405834267	1.29809715
0.117294	0	0.0582764	0	0
28.3517	32.5594	33.0507	32.0925	59.0436
17.1344	9.84255	10.7325	14.545	29.1851
19.6029	18.9359	16.6584	17.2445	29.1851
0	0	0	0	0
0	0	0	0	0
26.94162054	28.23118	32.83533293	31.36979679	62.1911016
22.88174167	21.30519687	24.9136053	25.94181151	62.1911016
7.68602	9.36352	9.50135	7.45037	7.26484
80.6995	61.514	30.10648	38.23174	0
35.760649	30.33974	18.709847	21.706678	20.3685159
0.974122	5.16886	3.63659	1.43137	6.81745
1.580140001	1.24135	2.35307	3.12586	32.6287
21.1343	22.5938	21.7964	21.2152	60.1645
64.9488	74.8228	80.6047	71.0886	68.6307
0	0	0	0.0424761	0.431796
2.799988975	2.275036973	5.579721507	4.512849358	50.29449
73.0394	77.0296	70.4422	67.1563	130.8383
307.667	361.737	384.673	284.076	454.661
30.905	22.52134	14.54766009	12.83682	0.8857977
30.905	22.52134	14.54766009	12.83682	0.8857977
23.93492	28.48496	30.66532	23.01287	9.755663347
7.258682633	7.887733	16.96809347	17.4009	14.37864332
11.067797	10.47172	15.45258437	12.05015736	57.0528
0	0	0	0	0
0	0.0617436	0	0.0796296	1.2953
0.296644	0	0.430805	0	1.26098
0.897825	3.98E-06	2.54456	3.98675	6.4169
0	0.0506641	0.0489902	0.131428	26.9188222

0.114766327	0.315549158	0.367178484	0.486033339	26.9188222
0	0	0	0	0.0519947
0	0	0	0	0.101272
9.005418144	7.724034142	9.061379	6.706143821	36.23653239
9.781077485	9.908518791	17.40733108	17.12586177	11.40403
19.28518764	17.97155	15.32038032	17.02339816	6.265892
0.341168	0.204655	2.33478	5.77027	1.79584
0.17910782	0.128933846	1.194595	2.340196	1.05845
19.14244124	24.55356029	26.59215393	27.32200675	54.371733
19.14244124	24.55356029	26.59215393	27.32200675	54.371733
19.14244124	24.55356029	26.59215393	27.32200675	54.371733
4.60703	5.52649	6.47324	5.87914	18.9471
0	0.14384	0	0	0
0	0	0	0	0
18.4575	19.5116	30.2468	49.1353	59.1256
2.875645	2.999831	2.58115	3.261512	5.192411428
0.586375467	0.276861777	0.922915481	0.774963559	0.54527
8.0425	8.82009	17.9647	19.6412	8.99183
34.60946795	32.41945301	28.74773284	26.56102718	11.88972017
0	0.0971766	0	0	0.882405194
0	0	0	0	0
0	0	0	0	0
0.22947	0.226929	0.130244	0.351517	0.160222
0	0.000279734	0	0	0.20646
0.266094645	0.348082	0.1733659	0.237246418	42.7235281
0.2455281	0.2434854	0.0428924	0.0570051	35.2023
0	0	0	0	0.605422723
0.0140467	0	0	0	0.605422723
28.04494032	23.57186991	16.92133485	14.12591098	7.031573
2.861912431	2.973531	0.48729636	0.522829773	0
0.742051	0	0	0	22.2615
20.28155	18.6214492	44.96405016	53.56132	338.613
7.132504648	7.061439335	8.59110393	4.750010569	39.4307
37.4053	42.9272	45.2326	48.1129	90.7804
15.85498	16.267561	14.77454	14.49149816	9.05929
0.591574	0.000139586	0	0	94.7946
0	0	0	0	0
14.01277117	13.18356002	14.86132	11.57760676	22.09509104
1.349523029	0.74171091	1.21909741	1.99303999	42.0789
0.699657	1.314834	0.23875	0.386257	147.68516
15.113514	16.74129	19.803503	20.31149425	49.8828
15.113514	16.74129	19.803503	20.31149425	49.8828
4.355870218	4.583975407	2.465358875	2.016748881	0.434034
6.13577	7.39537	4.54674	5.98101	9.17111

89.61778772	82.82465011	68.0633561	72.51418898	0.00258091
89.61778772	82.82465011	68.0633561	72.51418898	60.16906938
89.61778772	82.82465011	68.0633561	72.51418898	60.16906938
90.9118203	75.86904831	49.75875809	59.45075463	1.98526
90.9118203	75.86904831	49.75875809	59.45075463	16.40949
55.86915	52.0761097	34.33642402	34.59634646	33.30888
55.86915	52.0761097	34.33642402	34.59634646	33.30888
2.40963	2.95431	1.03063	1.03823	66.4262
2.6268	3.38348	2.59625	2.75631	49.9444
0	0	0	0	0.138602
0.480029	0.339047401	0.9129495	0.88333	0
0.480029	0.339047401	0.9129495	0.88333	0.160392
0	0	0	0	1.1635
1.270899658	0.888544338	1.769519744	1.0183252	1.6428921
1.270899658	0.888544338	1.769519744	1.0183252	1.6428921
0	0	0	0	2.05095
0.178477531	0.104612297	1.7788701	3.139738598	1.653067
0	0	1.427953	2.030961247	1.653067
0	0	1.427953	2.030961247	1.086523186
125.9507487	113.1614023	69.25576	73.48271	45.02644
4.667341835	5.886710078	10.41334124	9.280110001	3.231137
4.454884157	5.787108054	2.7134276	3.587200342	1.17927536
4.454884157	5.787108054	2.7134276	3.587200342	1.17927536
1.169675	0.778351	7.22032	11.53847	1.2473
1.169675	0.778351	7.22032	11.53847	3.371771258
80.7385	83.5573	108.152	77.6508	64.598
11.156926	11.147575	12.4027	12.199123	15.7639
33.84987	46.24179	16.62282001	17.9523	23.48337
32.06340105	34.67225511	53.67112288	47.58877835	45.00077849
0.3966885	0.500289	0.917953	3.35816	2.50791
7.40E-15	0.00300313	0	0.00443427	8.02E-15
20.631686	23.418852	27.890246	29.41702959	40.843582
20.631686	23.418852	27.890246	29.41702959	33.9613
128.3870188	138.8062243	146.6349263	93.12145505	317.64689
128.3870188	138.8062243	146.6349263	93.12145505	317.64689
3.20352	2.16927	1.54597	2.17224	0
2.560221954	1.858501525	3.151188422	5.1221628	4.586091
1.66962	4.23083	6.3473	6.08444	6.47994
0	0	0	0	0.577448
0	0	0	0	0
0.061154192	0	3.801459	8.116002688	8.345764914
0.061154192	0	3.801459	8.116002688	8.345764914
0.061154192	0	3.801459	8.116002688	8.345764914
24.96647802	28.87206329	19.73800112	22.7690202	2.752887

24.96647802	28.87206329	19.73800112	22.7690202	2.752887
7.80462	9.37888	5.20898	3.16245	4.10704
56.2733731	55.35974	59.59630733	56.718565	177.8383
56.2733731	55.35974	59.59630733	56.718565	177.8383
41.63704242	43.8869264	59.1671975	68.7659148	53.72140205
19.3998996	17.82103476	14.34946448	15.11587011	22.324
0.398364	0.474519	0.380444	0.717081	0.095662
0	0	0	0	0.104162
0	0	0	0.174354	0
0.311527	0.513977	0.0982347	0.911753	1.2822
24.01936037	24.89554499	38.57690023	29.12452311	14.11042714
2.06503	2.11946	1.89761	1.5141	0.706741
6.6167	4.98383	13.2917	9.78546	7.41538
0	0	0	0	0
8.48892	6.49386	14.58906	8.6958	90.8326
16.09422667	19.7232101	19.77384	16.23777128	33.87308
0.263490747	0.177612737	0.391208219	0.452337	0.399242
27.78277862	28.14611511	22.76645002	25.092618	12.72864
479.449	555.235	803.375	721.487	887.8979
9.16759	9.09981	13.6988	18.9637	10.881
73.814423	65.2219368	43.85889	50.06056	2.244052
73.814423	65.2219368	43.85889	50.06056	14.35042
1.83228	0.891666	2.59E-13	0	34.28915118
31.81800082	31.05735464	20.67512371	23.73065427	16.50163196
0.111893	0.0566109	0.0551586	0.0740191	0.679792
9.8812775	14.54649	17.46845	22.51345	50.96058943
8.189095825	7.170374213	13.11577077	16.74925122	73.295874
8.189095825	7.170374213	13.11577077	16.74925122	73.295874
8.189095825	7.170374213	13.11577077	16.74925122	73.295874
28.153	31.4985	40.3602	28.9595	54.5559
1103.0805	812.189	369.06299	470.5295	24.105059
91.01914948	106.307926	10.44254067	16.47091917	2.049313642
377.985	371.53	494.399	350.152	195.372
36.294	32.3571	31.7127	35.1524	95.9972
12.2036	13.4277	13.8299	13.2328	20.8871
49.8236	45.93057962	37.579153	34.95477427	37.04060567
27.5356	28.1334	45.6209	42.3296	24.1143
0.0776416	0.145599	2.766575455	12.19374	67.09535
0.0776416	0.145599	2.766575455	12.19374	67.09535
0	0	1.472494535	1.864134849	1.19837
0	0	1.472494535	1.864134849	1.02622
39.72764728	40.61529482	14.46506	18.36197582	4.102166218
39.72764728	40.61529482	14.46506	18.36197582	4.102166218
0	0	0.175529	0.553325	8.94191

0	0	0	0.403354	3.465729177
132.64171	136.3631	113.14903	85.41598	82.47512
5.59475	6.5412	12.0861	8.11525	82.1407
37.675	37.5996	73.2819	116.89	100.767
6.922341	6.355335	9.94677749	8.503605	102.6691076
6.922341	6.355335	9.94677749	8.503605	102.6691076
2.524877	1.643486218	0.653509898	1.093284398	0.654346
13.36786	11.49371	21.82654	21.687032	49.8684
1.78362674	2.246237459	5.24482122	2.145688171	6.87831
2.999010623	4.130289	8.512091	10.74296	7.875964
0.254113	2.73E-05	0.21723	1.22E-06	7.79187
0.236585	0.237687	1.89869	5.16492	2.73474
10.0822	8.60921	9.88606	8.02741	37.1187
10.67947017	11.17548	13.08758654	11.44884172	20.8598
54.5219665	38.599052	200.6862418	230.8055328	104.05404
3.50813	3.7169	3.2833	2.52455	2.13535
2.45439	1.25374	8.09602	6.20919	11.6134
31.05579309	40.41711171	26.06520389	25.05041619	16.18520231
0.663737162	0.281429034	4.749889504	8.71239	3.491350993
0.168057	0.0570564	2.24437	5.28899	5.020527274
2.141790789	1.432141492	2.300970862	2.450250956	0.706528463
103.433	78.5961	31.3627	39.894	16.5598
3647.67	2424.26	1192.73	1636	25.9715
0	0	0	0	0
0.0834408	9.39956	5.5667	0.000493002	13.7498
17.1231	20.3955	19.8227	20.2728	20.1152
0	0	0	0	0
0	0	0	0.948475	0
0	0	0	0	0
0	0	0	0	0
0	0	0	0	0
0	0.12647	0	0.327804	0.155577
0	0	0	0	0
7.8888	8.5383	13.84282	11.41487	27.82132551
23.1045	18.29218	32.1267	26.1485	53.5746
0.190498	0	0.0927797	0.375901	0.221067
31.81286	37.69625	23.84419	29.84	29.87125
36.343174	35.857456	28.45549	26.58123	11.864
0	0.212948	0	0	0.259215
0.743821	1.34201	1.15068	0.843395	2.0522606
0.906466	0.00109237	3.32709	1.26028	5.57998
28.67209948	32.65077	31.74604	26.41869407	76.61554
1.265087	0.946081	9.09277	16.03146	4.75426
691.9112	644.9466	1269.2747	1247.0984	263.5682

5.878824	7.629618	3.107379	2.739182	1.337982
32.81234136	32.591845	29.2698314	27.85038515	10.4605
32.81234136	32.591845	29.2698314	27.85038515	42.6263
29.1549	27.5842	38.381	31.02946	57.7586
54.84935	51.20692	54.9257	46.630035	93.39734411
54.84935	51.20692	54.9257	46.630035	93.39734411
25.3563	26.3818	25.5805	24.5246	54.542555
25.3563	26.3818	25.5805	24.5246	54.542555
13.73212112	10.58286573	22.84794	25.24426	121.978
91.1311	79.4415	101.4144	98.6558	147.138
101.0927	92.6419	91.1207	120.8945	36.2059
70.4215267	66.42231986	62.64241529	68.17714955	35.4596
70.4215267	66.42231986	62.64241529	68.17714955	4.862078255
0	0	0	0	0
0	0	15.8432	27.9803	28.7959
0	0	8.37E-07	0.000469388	0.000182757
0	0	0.419596	1.67369	0.77798
0.859226	0.490768	0.470846	0.159204	29.1093
65.78446	54.25877	66.53226	50.74666	96.7545
19.205327	21.067337	17.41496	16.29561626	49.48071
22.59727354	20.494809	34.95633109	28.9726288	39.24617114
69.00927272	77.08833432	73.95937958	64.87422	119.5666383
23.8877203	22.73611258	38.51971524	36.01840805	72.96874564
1.259424216	1.916690016	1.403141034	2.515834363	96.1818
7.01382	7.11341	9.85023	8.4637	29.62556
33.76589949	28.670847	28.7512697	34.91073868	92.541
33.76589949	28.670847	28.7512697	34.91073868	92.541
1.26181	0	0.877845	0.402711	31.25648
10.9312	10.7338	8.05012	7.49821	31.25648
8.78059837	12.33078568	15.68034934	9.05155736	34.00411
8.78059837	12.33078568	15.68034934	9.05155736	34.00411
0	0	0	0	0.472159
1.70147	1.60632	1.66913	1.8516	1.91787
0.27543	0.829625	1.04534	0.713797	0.933151
0.165333006	0.132033722	1.954493375	2.925807817	1.535813113
6.99153	6.56174	10.10966	8.36251	25.9395
1.26845	1.21604	1.31614	0.790082	29.4579
7.90374	9.3491	10.5303	10.1308	37.5952
16.69711009	14.206	14.078	14.7946	0.237611
16.69711009	14.206	14.078	14.7946	0.361859
3.651700002	3.287480115	3.321251376	3.41122092	36.3808
0.695641765	1.000895105	0.60023319	0.538901894	134.89311
32.69309557	34.811942	46.70730724	43.1489845	19.567
99.71777118	92.11330451	100.367466	110.2520644	50.2552

99.71777118	92.11330451	100.367466	110.2520644	4.03318509
12.3884	15.1254	16.8098	15.0245	94.154374
15.1923	14.1879	14.41330328	16.879528	29.58223
10.4797	11.6572	6.68033	9.94334	2.02818
18.13136467	21.04319	18.06119	18.61264	45.56389
0	0	0	0	0.335798
28.6033	31.049945	27.1113001	24.623	45.36947912
180.39506	149.276774	89.8558	120.17618	17.1251
37.48688806	40.40606092	33.5096838	38.16541685	80.01500171
37.48688806	40.40606092	33.5096838	38.16541685	80.01500171
88.4912	87.8182	7.516229	25.25516	3.212493
29.02346	22.320185	29.55311494	33.07592274	33.8548
29.02346	22.320185	29.55311494	33.07592274	15.10246335
9.12422	11.3954	11.1577	8.53876	35.5023
12.95503	15.64786	17.18938	18.42076	32.5714
12.95503	15.64786	17.18938	18.42076	102.826
39.1007492	36.07526628	21.41884518	23.79669075	9.120615
39.1007492	36.07526628	21.41884518	23.79669075	9.120615
4.784777665	5.382701519	4.63320443	4.13006501	2.467618115
26.82075	28.04408	31.74333	35.14014	31.51902
15.043809	16.86884	15.193539	14.165008	39.10700523
30.38847823	33.1356797	35.66751628	31.82319332	75.62271
30.38847823	33.1356797	35.66751628	31.82319332	106.7583
3.32604	2.83043	5.00591	6.17886	24.7227
0	0	0	0	0
2.14478783	2.660723	2.2559496	2.175579469	136.7934
29.21027	28.12632	40.83193	39.23661	59.38823
33.4518	28.6158	42.0725	33.6507	20.6773
24.54177	26.6359	19.82015	19.77782	23.99435
0	0.387671	0	0.0848535	0.969342
0	0	0	0	0.697319
91.507619	90.418847	100.928235	112.112057	80.277497
91.507619	90.418847	100.928235	112.112057	80.277497
0	0	0	0	0.576432
41.34331506	47.296277	83.769284	69.77428	18.29079197
25.393823	30.10249594	21.874609	23.38830079	31.89266
25.393823	30.10249594	21.874609	23.38830079	31.89266
0	0	0	0	0
45.86549	41.55111	46.6992	51.17055	80.2593
60.6714	64.9901	77.1712	64.6349	70.01517
0.779146	1.73232	1.0586	1.42617	0.562333
147.5875412	145.5051462	145.7241204	124.0575953	82.56159528
12.4930673	13.42728302	16.01051287	21.57380833	37.97522
12.4930673	13.42728302	16.01051287	21.57380833	37.97522

29.67481059	25.82160715	17.79186758	20.282815	2.80E-06
29.67481059	25.82160715	17.79186758	20.282815	8.01556
72.7602948	56.28295033	16.30076302	22.2797536	2.708085074
23.7775	22.86407	19.79254	20.044176	8.68674
81.69254587	96.80532804	99.83328408	83.40521953	112.6500242
10.0757847	11.406747	10.5768091	9.576573	57.5125
17.7205	16.80988	13.98258	15.10428	42.9598
1.21344	0.642649	0.670315	2.74069	4.23E-06
163.723	154.1097	146.0716	125.6393	9.29702
24.31345	26.955841	20.5482	17.53664679	16.044883
0.733624215	1.635927841	0.728008714	1.088663622	44.86207
0.072247405	0.044651327	0	0	8.73529
17.2029	17.6798	20.552	19.1628	22.76702726
125.6477	122.3779	83.1143	78.6816	39.5953
7.82942	7.96657	15.3534	12.2526	17.5834
7.06074	7.89941	15.15478	14.7682	42.8519
24.23608	23.97886	18.32756	21.59894	28.4947
0	0.877013	0.784963	1.09523	0
16.49626038	17.38966065	15.02206606	18.21820371	14.61358
9.25378	10.57662	16.06097	19.44734	43.21342
0.109520421	0.16771869	2.619672004	4.718518	3.387421186
6.39175	7.64343	10.20993911	8.938080013	27.76881034
11.78136	12.57559	15.91518075	14.80613	19.439
11.93689211	15.64127384	9.6135766	8.854620176	3.279291744
14.53301257	14.355877	13.6052166	12.80733574	7.908459133
0	0	0	0	0
1.218046691	1.137478	3.520921448	3.943557379	0.415384
7.06038	10.38040662	8.900494	8.600069407	21.33464
214.5896022	212.90546	239.6429183	194.8350301	166.118
19.6310662	20.668835	24.55736354	26.42431055	42.29183837
19.6310662	20.668835	24.55736354	26.42431055	42.29183837
17.426339	17.191648	27.52544	28.47322	20.264808
0.926061	0.46492	0.297339	0.200962	6.563647758
4.06774	4.12291	4.25845	5.67472	6.62187
19.799539	16.396716	13.96625603	14.390863	11.37804
3.56674	4.54503	4.84574	4.32645	36.2096
11.98148662	11.68136247	14.62639501	12.86760586	34.3641
9.025729	7.20488	19.24342178	21.697297	7.51839
18.8424686	19.267157	20.3624124	19.669179	16.6792
5.359530773	6.373753324	14.347501	11.84488	232.893
40.9739	46.21	59.8534	51.7694	99.32539247
15.537427	15.017158	13.2248566	14.46188136	99.32539247
15.537427	15.017158	13.2248566	14.46188136	99.32539247
7.879126	6.991919056	5.75108	4.614796929	3.09628

136.33741	145.17861	171.15863	147.43426	84.1613
136.33741	145.17861	171.15863	147.43426	16.5192
164.588065	209.0863135	129.3489596	119.5207514	164.6936217
164.588065	209.0863135	129.3489596	119.5207514	164.6936217
66.0029	31.34742	22.02853	37.6247	9.49425
5.649775511	5.726375396	14.78374644	12.03008626	11.26823
35.20633	32.55153	23.09155	21.788685	41.748375
59.81149669	62.70490802	51.78806337	48.69729	30.090398
4.09701	4.93438	5.90153	5.21591	29.8019
21.54822295	21.71076	23.4483899	24.78409	52.86470974
0.0350792	0.0355861	0	0.0932722	0
0.177261	0	0.262387	0.238515	0
6.351074	6.53005	9.11655	6.56219	1.85059
23.96124137	22.92221902	16.11043568	18.83018803	4.389053873
0.679219434	0.44358323	2.562858664	1.556901112	1.748951486
7.1547489	6.835919719	11.70034295	12.5485017	8.032081407
27.03703	27.51078	25.90527	28.96349	44.0765
8.982409824	10.78378	16.17101	13.98254099	36.2485
8.982409824	10.78378	16.17101	13.98254099	28.20080278
14.694552	17.69837	15.283917	16.029661	23.303844
4.581708004	6.783949099	16.57397282	13.47324308	31.99809
4.581708004	6.783949099	16.57397282	13.47324308	31.99809
11.80979488	11.21919611	12.94528239	12.5772495	81.59612143
11.9947	10.0882	18.2405	16.9322	19.0051
0	0	0	0	0
0	0	0	0	0
0	0	0	0	0
215.726	267.714	495.473	355.898	783.637
0	0	0	0	0
68.97694002	68.973665	73.430562	64.04362032	67.4591
68.97694002	68.973665	73.430562	64.04362032	34.2913
12.10286365	10.11594866	25.09370003	14.70701096	4.780638542
14.89316597	11.96044846	13.13449212	8.391541934	3.550133
14.89316597	11.96044846	13.13449212	8.391541934	3.550133
118.814656	117.6627398	91.050482	81.85805427	16.30534
43.07657	24.68237	187.3554	184.5233	10.2986
5.628258	0.827333	3.78052	1.262365	1.0445
23.4828078	18.7532	31.4627198	19.209713	83.28253
10.56348594	9.941933788	10.93156569	11.42283166	11.2187
8.67682	9.36809	12.3566	10.14116	27.2371
42.76172	38.9855	53.9866	46.616	90.8415
10.748649	9.03400156	8.296545	8.557398414	6.642967782
10.748649	9.03400156	8.296545	8.557398414	6.642967782
3.923899895	3.718973	3.495561791	2.718801256	1.6146129

0	0	0	0	0
0.369943	0.401691	0.365757	0.422582	0.189643
1.027913	0.44269	4.789175008	5.058486	0.576573
1.027913	0.44269	4.789175008	5.058486	77.6447
0	0	0	0.5693	0
32.93434	36.36934	33.05846412	41.16294	62.4232
32.93434	36.36934	33.05846412	41.16294	69.879013
52.3864353	52.1294407	34.30080508	37.37050204	9.091606
51.52086289	53.60036201	76.54601392	66.48539942	121.8781
62.6956	67.5332	56.5865	52.8183	92.8989
63.32168748	52.79200361	86.20665205	90.31605283	52.41832492
2.26906714	2.7559877	3.71638702	3.8178385	6.802110003
2.26906714	2.7559877	3.71638702	3.8178385	6.802110003
12.08742	10.23191912	7.681414414	8.168972	3.680575
0.743886	0.285463	2.8778644	5.072346	4.590197
48.91301241	52.16472528	40.53879747	40.06821	10.51917
51.05241645	58.32921308	81.65380274	86.13208403	191.986
577.0205	537.7961	663.9548	538.0969	725.668
45.30407	49.29359	49.2454	50.0607	104.1231149
14.8741	15.4278	36.9107	32.2772	10.1559
472.2864	457.3042	628.1985	539.4007	13.3122
472.2864	457.3042	628.1985	539.4007	371.074
472.2864	457.3042	628.1985	539.4007	5.75389
67.24166279	71.025912	55.17603929	39.184149	27.8029574
168.086	127.544	140.384	168.226	156.247
65.786348	68.18203	62.455277	54.68015559	41.72131539
65.786348	68.18203	62.455277	54.68015559	6.974733
0	0	2.57664	6.5125	1.36599
0	0	0.749267	2.31398	0.791179
5.77401	6.5481	13.4604	13.9794	37.7155
0.16346	0.253272	0.081547	0	0
41.019213	40.481424	54.50448	38.42742	64.9219
22.33195	24.146634	20.070309	15.254642	27.1041
6.4126	6.06483	8.11739	6.0389	15.1323
0	0	0	0	0
11.48976108	11.814407	12.34012616	13.6900156	34.36438345
6.37339	5.42512	6.43556	6.76459	4.10849
64.59219	65.26252288	66.307712	72.783687	63.2841
48.2022	42.3229	16.2887	19.6326	0.951311
0	0.0499568	0	0	0
662.2278147	302.4944261	3020.149601	1314.360833	4.180608851
662.2278147	302.4944261	3020.149601	1314.360833	16.8752
65.36749228	65.63329253	73.14280689	81.18117212	38.26261074
0.199804	0	0.298496	0	0.121843

2.38957	1.326555	2.220788197	1.020740001	5.9946
34.292426	33.136716	29.022736	31.67069471	29.2038357
24.683472	26.8638011	34.98384	32.65196786	30.06866
4.627630836	3.862844389	3.712600732	4.123408	0
4.627630836	3.862844389	3.712600732	4.123408	0.225103
5.47369	9.85704	10.7627	5.87248	28.1981
46.6765	33.29155571	21.14287	27.67619	3.89219
3.850138153	3.64752217	2.685511997	2.74256595	0.597446
0	0	0	0	0
15.48826	11.6927	18.36783	12.58415	60.40211
15.48826	11.6927	18.36783	12.58415	60.40211
0	0	1.30659	3.70449	0.314214
41.2747512	38.95335624	35.94809	37.162988	26.35232419
41.2747512	38.95335624	35.94809	37.162988	26.35232419
15.93639	20.31636	20.57466	14.92268	8.9627
26.09418091	25.44680771	26.28603672	29.680021	11.28145088
36.63708615	32.51245245	22.72530816	24.42570693	8.286171152
14.8035594	14.515061	21.870452	23.066046	34.03664
97.63549	40.82948	367.82281	125.42806	0.401707
662.2278147	302.4944261	3020.149601	1314.360833	3.23012
0	0	0	0	0
0	0	0.395012	0.538638	0.263804
2.250215	2.193904	2.43731	1.70910265	0
2.250215	2.193904	2.43731	1.70910265	0.0932315
91.1592	65.6779	29.14922	38.85465	4.44193
2.517368	1.883980002	3.031717	3.41794	5.75441
162.089	150.896	15.4987	87.7291	3.7647
25.032802	24.12036986	3.09261907	6.518624829	54.17713
10.61071986	9.544205359	9.640699877	10.63084904	3.89918504
57.18334431	59.85963861	51.36145826	47.87102734	119.772286
73.715493	71.323882	68.869189	82.39609057	232.4495
16.87471	16.30167	18.17855	19.00162	46.7462
29.18919	26.34738	22.80772	23.72060738	15.4809
33.67468	42.387957	53.89897	43.85695	71.0469
21.35190802	22.05291152	14.34353754	16.18757626	31.698742
21.35190802	22.05291152	14.34353754	16.18757626	31.698742
2.823832492	0.553757069	33.453086	71.4787681	83.3496
2.823832492	0.553757069	33.453086	71.4787681	0
2.823832492	0.553757069	33.453086	71.4787681	0
0.413774	0.380492	0.00107456	0.00550043	0.000326956
0	0	0	0	0
0.761707	1.08578	1.4271	1.68733	0.374154
2.83503	2.67545	3.21561	2.45773	3.650208361
5.84387	4.266012679	3.983099	4.930027214	3.650208361

5.84387	4.266012679	3.983099	4.930027214	3.887680871
6.01978	5.74071	8.48366	6.6499	12.83170419
169.9297	170.1374	197.90026	159.2264	66.89980554
0.270804	0.596381	0.582246	0.240463	0
148.2296837	135.0735321	93.1500606	95.73519185	20.68168
37.35703	35.24071	40.25951	37.19787	40.5716
59.3295428	61.78001	62.28556018	63.71091	106.8833
1.419990825	0.49698	15.18084	10.83721525	9.354437259
1.419990825	0.49698	15.18084	10.83721525	9.354437259
4.07621	1.16999	6.87747	0	2.62533
4.374949	4.764207	5.618252	5.458778	19.39050283
0.596210543	1.12333453	1.75522582	3.171845701	0.711163
0.596210543	1.12333453	1.75522582	3.171845701	0.577835
0	0.0957615	0	0	0.114383
0	0	0	0	0.744508
3.13296	6.01308	3.30635	4.3458	0.000103203
53.4744	46.8432	108.239	154.531	32.1769
0.958179	0.54996	1.59091	0.703547	1.891942
55.32406	51.76841	37.84189	44.26274	15.11968
0.269183	0	10.1645	23.5515	28.486
0	0	0	0	0
9.458544	11.127259	17.49703	12.41162671	5.07177
59.016	55.1934	35.86512	40.6226	10.10854
9.260927	9.458756	11.38462	10.6737	24.7289
0	0	0	0	0
0.106981	0	0	0	0
2.823081831	2.52568961	3.310748159	4.33283062	0.823647
23.7236	27.6224	33.5136	29.4711	176.717
5.32672	3.38447	8.96338	12.6593	38.1539
13.74262	16.448	19.09628	14.43096	64.2408
7.882707	10.05351882	9.751374	9.53218	18.87414
7.882707	10.05351882	9.751374	9.53218	18.87414
9.5383587	10.8711	15.16426682	17.24134846	41.74679
81.59049765	69.99631171	61.46046134	69.27471329	41.629103
81.59049765	69.99631171	61.46046134	69.27471329	41.629103
81.59049765	69.99631171	61.46046134	69.27471329	41.629103
54.20889714	62.4644334	37.4455421	41.31950826	124.38996
54.20889714	62.4644334	37.4455421	41.31950826	124.38996
14.8862	15.4692	27.7724	26.4236	35.8612
36.2294	40.975	41.8266	41.4827	13.032865
36.4989318	35.42499541	25.43272011	25.40704465	7.10384
30.70098564	32.83409	20.65565184	23.399836	7.069576
8.400664048	8.669097466	10.54889005	11.59483234	22.98364
68.02829	66.95372	59.06683	63.1889	33.53767

68.02829	66.95372	59.06683	63.1889	33.53767
9.1643	7.730289	21.4185769	21.713517	22.85340925
115.9291208	109.0701104	128.0250658	93.86492589	19.61200427
2.128401	1.07604219	2.51205	2.478520249	2.54527
19.452833	20.960307	23.5549504	21.15812366	33.503753
23.74795506	25.988992	25.91005593	26.0607564	18.86058
63.53159	65.59283	44.276996	46.34713	56.88058518
24.26907	26.43276	20.34392	19.54854	30.101
69.14978253	85.94853684	44.0748732	38.58090484	4.752487353
69.14978253	85.94853684	44.0748732	38.58090484	4.752487353
68.8091	93.0682	76.5197	64.987	224.851
23.37797388	23.2623599	17.99648537	19.7886143	34.034
0.033959	0.0348526	0.0684235	0.182915	0
0	0.0405841	0.0596737	0.0532701	0
7.9917719	7.5236071	15.735229	14.65054101	36.29989
4.199182	4.14046	5.91493	4.489095706	19.6204
15.7809032	16.619462	13.55645	14.09012	9.34619
79.28580103	72.57272791	95.871647	106.4832412	29.34680747
0.351258575	0.4270014	0.414876774	0.077273841	0
12.7354	15.2274	12.3572	12.8018	39.5283
31.9101924	29.7967117	25.794502	30.031291	40.02219404
31.9101924	29.7967117	25.794502	30.031291	40.02219404
31.9101924	29.7967117	25.794502	30.031291	40.02219404
19.13535739	16.86330251	10.66107013	12.6783005	3.981373213
9.42904	11.1581	10.5905	11.606	2.88011
2.14E-05	0.0068339	1.55E-05	5.73E-06	1.099080622
67.79690617	71.610586	114.284122	110.85456	2.61471
67.79690617	71.610586	114.284122	110.85456	62.09878
0.0438665	0.0895437	1.093044	2.565513	2.885172212
94.59643	78.53514	75.66446	78.45073	63.0829
0.7189851	0.171537	0.581357	1.405099	1.818640964
17.87473307	26.12144307	54.034117	48.86193527	4.562595811
17.87473307	26.12144307	54.034117	48.86193527	0.961930342
17.87473307	26.12144307	54.034117	48.86193527	8.307749
25.23788	26.4290511	32.77601041	30.3394852	51.45432
39.44401035	49.22592409	48.88591051	39.74041463	133.2860384
0.3652695	0.801500246	0.097047497	0.086622929	101.01526
32.31399278	34.3697753	25.09417155	25.23039286	8.27543
0.65362	4.68672	0	0	305.648
2.416374	0.14284116	35.9159	80.4742	78.7882
0.300970113	0	4.440042631	8.95917	5.2628094
14.52664451	14.38072828	16.14950177	15.57822101	15.094315
878.8923	905.7044	967.3552	918.4092807	796.2383793
24.81695	24.16663	31.90988	28.39018	59.48887

24.81695	24.16663	31.90988	28.39018	40.54712559
11.487122	11.092182	7.127232462	9.333	1.732020823
11.487122	11.092182	7.127232462	9.333	1.732020823
29.84618535	36.24543262	21.568128	21.16528066	9.269077
3.591268	4.304437024	2.846451	4.790872881	3.666278125
3.591268	4.304437024	2.846451	4.790872881	3.666278125
16.93261	17.584819	20.0405666	21.31757841	34.1485
685.844	718.345	1160.561	1156.253	1479.980184
17.69161742	20.17088021	15.118457	16.9961058	39.43479
17.69161742	20.17088021	15.118457	16.9961058	39.43479
15.03050086	16.91957	17.798352	16.594066	39.58342
16.1167013	16.00655	16.84074	19.07827	25.8250014
16.5106066	17.02314654	18.97411878	21.19280909	14.71101383
16.68927534	18.39160686	13.91226389	14.61057021	6.996880111
16.68927534	18.39160686	13.91226389	14.61057021	6.996880111
20.72233491	22.31483	15.567697	16.0642	29.971764
20.72233491	22.31483	15.567697	16.0642	29.971764
37.9869	36.1363	49.6677	46.2033	58.122058
20.892753	21.98129556	21.15401408	19.708886	5.282900909
2.90048	3.55401	1.51765	2.04819	43.5168
17.64915364	19.05585838	11.69013482	13.372801	13.1529
31.8248	33.1758	33.9344	26.1619	26.5233
35.021	31.1987	41.4617	47.9979	17.1672
135.4155	135.98932	134.9089	131.3071	171.12792
0.535776605	1.163644	5.28694	10.17556	0.555146
26.3281	25.1467	24.0231	24.918	21.9691
5.237246331	5.623413358	9.118137494	7.749082545	12.9026
118.74164	118.6447168	120.4076962	109.594	151.3613089
0	0	0	0	0
0	0	0	0	0
32.5098264	33.77106573	36.57964781	34.58992176	141.8969296
147.509054	156.829062	157.3850269	153.6690235	140.2584
28.1008202	30.0900352	28.9179385	30.96602	27.77454162
28.1008202	30.0900352	28.9179385	30.96602	27.77454162
28.1008202	30.0900352	28.9179385	30.96602	27.77454162
37.00969262	37.27004338	46.405764	45.12219455	79.3962
8.54717	4.22292	8.64846	6.65818	1.7898
7.874809	8.848325	8.826215	8.593485186	24.60966
234.491718	203.881964	96.232207	111.042174	13.88997
26.46816	25.68703	17.07813009	19.711133	17.21418566
20.127569	17.72492001	14.663711	11.49108568	6.397605788
0.27585	0.055413	0.325029	0.790236	0
0.071531301	0.036226721	2.087574	2.303879	2.92768
0.725572	0.677874	4.30E-05	0.716955	0.222924

25.756063	26.638583	38.3108281	42.12862	57.63121
25.756063	26.638583	38.3108281	42.12862	57.63121
60.51163216	62.98780415	14.56363248	13.80759324	0
60.51163216	62.98780415	14.56363248	13.80759324	1.613460388
5.280482646	3.394645307	9.957992288	8.546413658	3.937777
6.402643228	5.89969	20.58030665	17.233324	16.67582
0.376108	0.0765157	2.28893	4.58553	2.98606
69.6201	68.6858	70.1334	71.4041	32.88017
12.19891	14.80794	7.06786	9.27457	18.9103
65.20178409	60.0044581	68.42090668	74.89397925	103.20506
17.441198	18.63641679	8.980801938	11.24218929	10.4508
0.636438	0.44616	1.36871	0.916846	4.5617
18.98224	17.89708	20.00681	18.58207	43.13873489
127.3406448	138.514243	131.7439182	123.7915584	338.6039704
127.3406448	138.514243	131.7439182	123.7915584	338.6039704
0.246941	0.230770591	0.364973	0.451770918	0
0.118102	0.594832	0.114318	0.308831	0.54587
0.28767	0.217768	0.461164	0.189396	0.470168
9.10825	9.57068	13.61684	10.30874	40.70323
10.50188	5.46673	8.73783	8.55283	34.358749
0.1486423	0.09144171	1.385223	2.43071	2.28439
0.0576631	6.42E-256	0.728377	0.770926	1.14107
40.068143	44.366624	41.950484	44.38574	50.93746
24.4086	20.8034	29.2875	18.4385	185.238
0.31658	0.745282	0.481142	1.93094	14.2047
17.71560255	14.67688	20.50636	21.7419	32.53425
96.87146	132.9316	101.90184	119.2677327	194.525
105.9280903	99.33170327	91.55653915	106.153268	73.91260796
0	0	0.877622	5.16011	6.93798
0.652271	0	6.39E-15	2.48055	3.882
0.652271	0	6.39E-15	2.48055	0
0	0	0	1.06406	0.508135
0	0	0	0	0
1.10E-33	2.19E-08	0.539388	3.30E-06	7.62881
98.9717	99.48732102	89.75810441	86.41438684	17.0306
98.9717	99.48732102	89.75810441	86.41438684	26.153712
98.9717	99.48732102	89.75810441	86.41438684	26.153712
35.2872	25.8199	26.4027	23.5198	7.17972
5.49429	5.266373297	9.4665	6.955319395	79.3074
38.2062	34.1425	30.3639	26.1349	6.92347
85.11091	90.0994	82.48073	83.21871	227.4017
28.2743	29.5727	65.0233	66.7336	59.847
14.7756	18.2031	10.0031	10.1788	24.357132
21.02205278	23.94294254	30.29558859	27.84155065	30.34500844

0.925137	0.4617148	1.153604057	1.081823177	0
1.91734	0	12.0235	22.4385	13.7587
8.85145	10.66474	5.9719	4.9802	4.43513
0.109803	0.16711	0.212848	0.358272	0.0646267
0.793702	5.24E-05	5.86724	16.8838	4.34637
0.246789	0.415506	8.92446	14.8233	13.06915
22.0879	22.8989	35.29955	34.62162	43.0405
3.519635	3.168042311	4.798667995	4.10214885	1.206451459
0.791010242	0.751723113	3.809849	3.197626519	1.47916
5.32410174	2.962447007	7.181627247	6.668931	1.594133873
5.84E-265	0	3.63E-302	8.40E-156	9.29E-10
0.176256	0.059962659	2.324376534	3.559236078	2.9485
11.43744675	10.748651	16.71184434	18.27343303	10.71926203
11.43744675	10.748651	16.71184434	18.27343303	10.71926203
10.0858	11.1265	13.4558	11.7361	3.45526
0.021682713	0.0431762	0.599749371	1.931706	0.427791
0	0	0	0	0
7.395186001	8.618793	8.848712214	9.39245	12.54950265
8.277014	8.71957	11.00215	10.153276	30.3238
0	0	0.000201646	1.3239	2.23884
0	0	0.000201646	1.3239	2.23884
29.42824695	40.65611849	34.34845853	40.26301356	49.00095
29.42824695	40.65611849	34.34845853	40.26301356	49.00095
16.36584	17.29317207	15.2877905	17.091792	18.18899
16.36584	17.29317207	15.2877905	17.091792	12.9062
5.316832	3.301528	6.256759	4.838190718	4.344672162
5.316832	3.301528	6.256759	4.838190718	3.45935
0	0	0.160015	0.214402	0
0.00352149	6.27E-05	0	0	2.91023
6.51643209	6.162744	9.739571451	10.67323902	18.32163
0	0	0	0	0
0	0	0	0	0
0	0	0	1.27	0
17.046781	15.24755	12.48005	15.04943	26.71771
12.00811	10.8263	18.80761	14.9393	52.2805
0	0	0	0	0
2.129580007	2.517680004	8.24316003	11.4237076	0.49174
15.8951	14.8495	22.2505	16.7801	140.7684
28.1878207	24.76563974	26.15409863	28.74437386	12.01321554
0	0	0.861606	0.591197	0
0	0	0.445643	0	0
42.41046888	41.24844231	60.87762326	54.2586432	11.32957
0.269335	0.271341	0.462319	0.352021	0.0827446
17.680722	22.47943	25.36513	32.539357	48.7701

25.8239772	27.7263728	23.5067164	23.51396974	41.53573
25.8239772	27.7263728	23.5067164	23.51396974	41.53573
0.321628	0	0	0.213336	0.189644
14.8182	12.0363	15.7734	15.9453	2.04674
20.38238601	22.7054014	13.54897565	27.34965639	9.657490001
20.38238601	22.7054014	13.54897565	27.34965639	9.657490001
6.842072	6.689069	10.293952	10.07430771	35.52035129
6.842072	6.689069	10.293952	10.07430771	35.52035129
23.21015301	26.72258407	23.620753	23.96942696	1.63057
23.21015301	26.72258407	23.620753	23.96942696	10.89821462
23.21015301	26.72258407	23.620753	23.96942696	10.89821462
23.21015301	26.72258407	23.620753	23.96942696	10.89821462
25.6474	19.275	5.97899	7.00199	0.647777
15.26604	14.62608	24.80421	22.71435	24.8137
2.4274188	1.30379516	4.158990011	3.742375	0.461396031
2.4274188	1.30379516	4.158990011	3.742375	0
153.6193698	149.5598006	150.148047	110.5192837	16.289273
153.6193698	149.5598006	150.148047	110.5192837	16.289273
36.33520145	37.30830186	41.38509555	40.12054998	30.85271207
10.2593001	9.463167884	8.388387866	8.571268029	2.201211088
10.2593001	9.463167884	8.388387866	8.571268029	2.201211088
8.20514	8.86454	10.1081	8.92803	56.6594
2.3705	4.78189	5.32504	5.63551	17.8221
0.110125	0.14780731	0.403836	0	2.0702
11.60591	11.69131	23.70162	20.12878	14.46011243
13.89340326	18.214019	13.58623007	13.76397195	6.064458505
13.89340326	18.214019	13.58623007	13.76397195	6.064458505
13.89340326	18.214019	13.58623007	13.76397195	6.064458505
37.746122	39.77136	47.251588	37.03903	56.38793
97.2266	109.8365	129.32693	123.08014	83.4379608
97.2266	109.8365	129.32693	123.08014	83.4379608
91.30219	96.84487	93.882516	88.72102806	30.14843742
11.77830755	12.03099182	13.094838	10.17268983	21.54763594
25.18280883	25.90074354	23.604259	23.65822146	39.275651
98.3407	100.34104	96.6417	100.41747	75.64707
90.8842	108.657	84.9351	87.6223	252.048
51.61441166	56.94098647	20.31534045	27.56878415	4.066361192
371.8521	338.5928	53.3391	120.7215	12.48716
1.7360562	2.116375	7.112055	6.376677352	2.880413775
3.847427224	2.60135788	3.975668	3.302147495	8.62141
1.12277	0.313341	1.74218	2.29302	0.286253
16.410788	16.661703	22.541266	17.72065412	70.79324
170.19663	153.24369	39.92171	54.60308	7.588653
170.19663	153.24369	39.92171	54.60308	7.588653

2.108825668	3.089896	4.7143205	4.372170931	6.896900589
0.14462032	0.067647093	0	0	12.10106046
50.98058	17.62718334	382.6222	227.2368788	0
0.139109	0.106026	0.0691018	0.231765	5.765348828
0.139109	0.106026	0.0691018	0.231765	5.765348828
2.9367	2.4938	0	0	206.106
2.256222	1.971175	3.479196	4.120525	4.06259
0	0	0	0	0
0	0	0	0	0
0	0	0	0	0
0.429286092	0.2558655	0.173061442	0.6103847	0.0369396
1.80756	3.40715	2.85313	1.97145	0
0	0	0	0	0
0	0.282798	0.27413	0.0923081	0
6.8771665	6.290582	2.334241378	2.186222885	0.288978
0.351692	0	0	0.446742	0
27.61313533	33.52215666	36.28084221	31.98405548	65.1962
7.420951	7.754970005	9.679661	9.163024137	20.29814
30.76538	28.00225	30.07837	34.30997	43.1537
0	0	0	0.0725325	0
0	0.051349	0.148824	0	0
42.46468	39.8331	5.67664	8.735376	0.242729
57.19551533	52.18173821	39.01907602	39.0814759	10.073881
57.19551533	52.18173821	39.01907602	39.0814759	10.073881
57.19551533	52.18173821	39.01907602	39.0814759	10.073881
0.225031632	0.457930588	0.042337931	0.150745143	111.7467768
0.225031632	0.457930588	0.042337931	0.150745143	111.7467768
745.9367096	655.0444807	152.4079689	227.730933	29.882
19.33504034	20.44282597	20.32413362	22.00570002	25.3396
0	0	0	0	0
17.960546	17.64559	21.362821	23.488679	32.312762
35.98118	36.61995	40.71671	37.7958	52.07772
51.09867503	39.50440703	27.68809675	33.01875947	8.46044
51.09867503	39.50440703	27.68809675	33.01875947	8.46044
0.0221869	0.0225559	0.862724968	2.487448	0
0.0221869	0.0225559	0.862724968	2.487448	2.081746
71.90798735	68.26657909	52.26136385	52.53148365	58.42419
0.908310351	0.560912166	2.530349411	3.027932722	1.469456544
38.21507	36.6834	27.120629	29.45864977	1.4115
45.81881115	50.72638021	30.72332892	31.57486393	72.443522
2.137468941	2.590227	4.092660035	4.557575	17.24473
327.03	301.383	447	338.715	31.12500106
327.03	301.383	447	338.715	31.12500106
48.0574	52.4341	24.4003	26.4774	7.59551

82.9325	89.7659	42.5977	50.0449	12.5635
901.4929	840.8682	894.6168	757.54359	54.781
17.90028233	20.75608017	22.045714	24.68284205	42.5078796
19.50303	15.69066	42.66298	49.30484	41.71844
11.406442	11.007925	11.397371	11.425622	16.622358
7.174064071	6.71901549	3.483710149	2.173760791	0.603731
0	0	0	0	0
22.07716618	18.58348588	15.87436665	15.53741056	5.70745
22.07716618	18.58348588	15.87436665	15.53741056	6.466481
90.38161964	80.815563	144.5505634	152.9530882	28.93563939
90.38161964	80.815563	144.5505634	152.9530882	14.0683
90.38161964	80.815563	144.5505634	152.9530882	21.43839
24.27077	22.10722	49.20397	57.3118	8.3328
0	0	0	0	0
22.849226	18.437928	13.608505	19.171121	23.972936
16.61217774	17.758947	21.98406895	29.1271125	34.06572715
1.28787	0.858731	1.25262	0.563644	0.736844
52.57778	56.01848	49.08754	38.4738	49.53473
29.0257454	29.529901	23.760372	26.2739555	31.22472
75.491	77.3248	4.56814	12.64402	0.744562955
358.8048061	329.2606008	17.79905009	80.14661039	4.021523
251.98966	282.24661	237.36349	214.01019	185.07372
251.98966	282.24661	237.36349	214.01019	185.07372
4.1938	7.09628	2.3622	4.57688	91.5462
0.974535	1.71722	0.563695	1.7259	0
4.533281814	5.807480308	8.5553916	9.014853	8.793606185
0	0	0	0	0
4.533281814	5.807480308	8.5553916	9.014853	8.793606185
0	0	0	0	0
540.41776	512.7904838	737.4489416	488.667328	22.38154
540.41776	512.7904838	737.4489416	488.667328	28.38327
12.9700662	14.45866	7.664533686	6.947474001	1.180000387
12.9700662	14.45866	7.664533686	6.947474001	1.180000387
53.92256316	50.46918571	34.9094867	32.10328428	11.06318815
53.92256316	50.46918571	34.9094867	32.10328428	11.06318815
90.70377	108.708208	137.0765105	123.09121	258.7878506
90.70377	108.708208	137.0765105	123.09121	258.7878506
0	0	0	0	0
26.90139024	20.12973	12.67822496	13.57948501	3.090710721
49.32816	44.12787	51.56462	38.20053	31.30944744
17.349219	16.33963	21.738659	20.62085262	38.19578
49.3907185	62.32321503	76.17448801	76.91010378	165.706
31.63841038	32.38847706	26.656546	28.19251903	35.1887
0.954943317	1.473669084	2.416857739	1.139537906	2.042475801

0.954943317	1.473669084	2.416857739	1.139537906	0
46.018093	44.24231164	41.83455975	41.450884	42.51927
4.246942978	4.286896662	6.339666957	5.405181402	37.545242
4.246942978	4.286896662	6.339666957	5.405181402	37.545242
43.0911	42.82623551	45.59501	48.00614	15.94271618
11.20710825	9.50264707	10.25276928	10.315299	6.073273068
11.20710825	9.50264707	10.25276928	10.315299	6.073273068
11.20710825	9.50264707	10.25276928	10.315299	6.073273068
3.54675	4.0961	5.62769	5.19811	0.758118
39.08687	51.94579	20.40799	26.87787	3.056010733
63.68320018	51.745713	25.29095278	25.051762	0.394806
0.61448	0	0	0.201044	0
47.53324109	49.182346	13.29663604	14.42691	1.430953
0.0419241	0.0422835	0.0414253	0.222794	0.205261
0	0	0.422356	1.14923	1.37837
9.63655	10.41681	13.95634	12.84961	30.9186
0	0	0	0	0
0	0	0	0	0
0	0	0.120611	0	0
0	0.0437649	0	0.028768	0
0	0	0	0	0
6.92403	7.96278	6.87331	9.05825	1.454278594
0	0	0	0	0
0.141712319	0.100978009	0.223583804	0.133336784	25.464413
0.956634004	0.783337653	1.291968	1.878878	12.83070681
0.956634004	0.783337653	1.291968	1.878878	12.83070681
2.557530868	2.686120922	4.010228	4.484481	31.1766
1.485475921	1.494463	0.078909322	0.53472796	0.995411
13.4563	14.5253	7.665661054	7.440715134	1.01864
0.0413281	0.124603	0.439667	0.754491	0.147311
0.789553	0.263923	0.247419	0.336937	0
0.403938	0.0804716	0.076675	0	0.0955253
0	0	0	0	0
83.91687	90.94695	109.52447	105.92233	33.69780812
83.91687	90.94695	109.52447	105.92233	92.8147
8.61894	7.92922	11.61124	8.63642	18.374702
446.77179	325.618918	164.642936	211.11405	14.061227
446.77179	325.618918	164.642936	211.11405	14.061227
0.0511329	0	0	0	0
163.75	124.887	57.5274	54.0298	3.21654
12.40131716	10.13159883	9.528081785	11.13708717	7.74291
12.40131716	10.13159883	9.528081785	11.13708717	7.74291
0.19311	0	0.439304	0.168644	0.157558
17.67127606	15.93511882	14.97046846	15.75644625	5.616215

1.556321742	1.7746545	1.594098	1.802264571	38.25315728
0	0	0	0	0
10.0753215	10.2841675	12.92448172	13.34064977	20.44134
139.949	166.853	88.8801	82.376	91.1963
18.73147	16.62407	24.29572	19.73177	36.8583
0.661115	0.403382	0.527437	0.702786	9.69291
701.5715	738.761	1098.66	1083.433	1229.094104
5.42812617	4.927756	8.890156938	6.837080082	3.94132
7.13693	7.66368	10.4632	13.8389	8.023622
36.2389666	31.00288539	22.06704439	24.92732017	5.484912069
11.5343009	13.20809439	9.93192197	12.39893543	8.577060649
21.6863	23.663	15.741	16.0095	3.88085
0	0	0	0	6.42247
0	0	0.26665	0	0.166055
4.32548	4.08462	2.6185	3.74241	1.147467427
0	0	0.13542	0.367384	0.517585
0	0	0	0	5.07167
130.38286	129.43555	134.4092	139.97391	115.614307
22.66832685	22.06343256	20.61486651	18.78165561	3.714
6.07895	6.90754	3.14718	3.23742	1.93535
0	0	0	0	0
16.2229854	20.736329	14.45715659	16.5145643	43.73487
34.25960799	34.0206317	26.31922	27.0399	63.9851
34.25960799	34.0206317	26.31922	27.0399	41.77179
0	0	0.592504	0.967268	0.762454
21.17605451	22.61093252	20.01722261	23.59785981	31.82133931
0	0	0	0	0
15.701609	15.370666	31.372076	24.188375	50.37028462
15.701609	15.370666	31.372076	24.188375	50.37028462
0	0.116768	0.056531	0	0.583266
0.0512614	0	0	0	1.15275
7.414944	7.122016624	11.992657	15.804789	6.330353778
7.414944	7.122016624	11.992657	15.804789	6.330353778
5343.43	2780.31	2613.27	2920.9	25.0072
21.55335546	18.78793219	12.52382059	12.58022974	3.872840702
363.255	266.208	78.6416	78.7183	3.12624
16.2622	15.26186	12.36567	12.79358	44.62945
25.3344	20.9305	16.6024	18.7701	7.250412487
15.0049	16.3673	17.9688	19.0447	24.674351
0	0	0	0	0
23.95610142	21.29976792	23.29890153	28.84067245	6.304233357
11.496034	11.51398502	11.65915	11.201058	9.72767
0	0	0	1.18323	9.58773
18.05390506	24.69538	24.69142	28.19514	31.57300669

18.05390506	24.69538	24.69142	28.19514	31.57300669
18.05390506	24.69538	24.69142	28.19514	31.57300669
0.2904361	0.320293265	0.4301469	0.7448099	0
230.593	209.522	98.3027	108.918	2.93795
7807.28	4455.419	3459.675	4571.403	58.6023
47.2037	57.961	86.137	66.9825	93.0107
10.62695	11.06595	10.55086	10.14663	37.0446
27.3913	30.0206	32.7645	27.85282	56.4726
26.56628	26.25011	9.39016	14.79622	18.70013
571.2522724	501.8752	751.3878664	670.6504678	314.8677
79.59790002	88.9161	134.5182	96.38540008	237.114
13.59676	12.21074	30.99907	29.27879	26.77344003
13.59676	12.21074	30.99907	29.27879	26.77344003
0.835026	0.240357	17.3677	30.7891	35.6809
0.818246	1.06209	0.346115	0.308442	0.984195
53.690385	53.51159208	47.97231367	47.26796	31.46911555
3.5291	3.32116	4.43418	4.8901	11.2603
29.2555	29.27087	25.81373	22.35449	32.73397581
0.905215	0.226146	10.98840102	25.5027	20.3998
341.574	163.5799	123.4903	164.6612	736.618
28.64501278	26.30221731	4.105576857	9.081981925	1.321501
0.717453	0	0	0	0
0.330988969	2.79899625	0.916996907	1.4854011	264.649
9.868162	13.2095	18.9657	21.19926	48.36269
7.783033405	8.797558104	10.4629185	10.376659	24.226527
762.0665765	759.9770099	607.5269137	505.084376	64.3023
19.75845392	23.136947	22.498595	17.9929262	34.64108
8.58948	8.728712	6.5135888	6.594398	2.16451
14.33409	14.9967	14.29142	15.48663	44.1078
151.7543	150.1664	133.9945	79.6796	131.7184
0	0.566158	0.263787	0	4.88112
1548.403431	1245.586	583.3564449	729.2964889	12.8413
1548.403431	1245.586	583.3564449	729.2964889	49.9823
1.08596	0.668419	0.253086	0.560169	0.356237
14.00742	16.22298	17.18753	18.2479	71.5274
22.78462	26.66572	25.29461	24.5495	72.4311
3.778701411	5.172313	5.838606	4.698284454	64.05532
245.386	278.18	328.353	264.932	92.6102
0	0	0.214655	1.237724	0.342847
1.7459	2.01919	2.28765	2.23964	7.76278
43.05460047	54.91250046	64.3969	54.4172	50.2114
1.5309487	0.961055	3.742021409	5.482830025	0
1.5309487	0.961055	3.742021409	5.482830025	2.43595
0	0	0	0	0

0.0543793	0	0.0527324	0	0
1.949838	2.462874	2.014178501	1.378246302	0.328283
2.271957	2.465352	2.3444237	2.669092	0.38799
0	0	0	0	0
0	0	0	0	0
0	0	0.157111	0.418087	0.593651
0	0	0	0.413273	0.172161
0.454647	0.261043	0.440733	0.76227	0.867024669
0	0	0	0	0
0.607913	0.0847352	9.522304	17.02643	12.42380076
0.125343488	0	3.246235052	4.616041335	2.631836007
0	0	1.127419	8.08479	2.296182
7.03062	8.14391	13.8195	9.90558	0.31157
0	0	0	0	0
0	0.198609	0	0	1.52849
2.192732	0.770397324	20.62066	38.30806	18.36449901
25.0961	20.02539	20.35894	25.00131	15.03086
78.92733	66.31147	41.17601	33.0501	5.461011886
78.92733	66.31147	41.17601	33.0501	5.461011886
32.09518	29.15211	20.6702	23.16496	6.8731
1.41595	1.36503	1.786571298	1.25842	7.97869
0.70404	0	0	0	0
27.72911385	30.65910972	30.59830427	28.50800414	26.54590684
14.1159704	12.67025801	16.03093484	15.11375184	25.68992
0	0.803933	0	0	0
0	0.169920658	1.802524493	5.55167	34.68668
0	0	0	0	0
0	0	0	0	0
0.818126021	1.219400218	0.0480329	0.446362194	124.865
22.29715	24.709102	12.9791203	13.406991	15.70465655
9.33982	10.6656	18.72223	21.03792	29.2278
280.3933866	264.6565862	190.2495941	217.5728745	296.12906
0	0	0.13387	0	0.333523
35.57760597	37.677925	26.537689	28.18186811	38.19281471
0.000964269	1.67E-11	3.09E-115	2.56E-09	2.95E-09
0.3739681	0.289709622	1.454167827	3.692016974	2.321732176
0.639915	0.6493181	0	0.184693	326.956
100.69206	119.3181921	51.83442	48.234623	7.703356
100.69206	119.3181921	51.83442	48.234623	42.73765
4.071072184	3.473132	3.961268666	3.177064	1.814642241
4.071072184	3.473132	3.961268666	3.177064	1.814642241
0	0	0.372499	1.56508	0.153681
10.5003	9.337314725	6.35338	7.589233	1.8970611
0	0	0	0	0.782911

0	0	0	0	0
3.58462768	3.479137358	2.697011314	3.85425601	5.83932
17.2795454	17.14993686	22.86938691	29.6267422	77.6946
0	0.425658	0	0	0
12.10437	11.89504	14.734958	13.204222	21.60545
5.18882	8.753004	9.32741	5.87839	13.2961
40.6583	45.4102	25.1582	29.4347	28.76061823
27.04568762	24.171056	34.72753768	32.58280015	92.6161985
27.04568762	24.171056	34.72753768	32.58280015	92.6161985
27.04568762	24.171056	34.72753768	32.58280015	23.554393
12.35340022	9.736300012	4.706680007	5.319640004	0
0	0	0	0	0
19.20393	18.62361	17.74829	17.89012	27.63348299
4.892631308	5.453968706	6.395181447	6.565398699	25.38910594
4.892631308	5.453968706	6.395181447	6.565398699	25.38910594
2.862101	3.985522	3.525471	3.577195	17.8152
24.61289	27.70727	28.02419	30.060372	43.67210126
0	0	0	0	0
20.1623	17.3976	14.0712	14.954	4.75568439
2.20011	0.658529	1.23234	0.563012	0
0.240196	0.0963921	0.0930953	0.187375	3.24553
0.0677337	7.28E-160	0.937413	2.52457	0.202528
0.718940403	1.518568	0.178309	0.316583	148.877
4.69064	3.80346	4.661293	3.021701	2.80609
0.335218	0.526726	1.574611	5.43649	2.9263
14.206424	15.230041	25.477346	19.429486	90.418
0.244623	1.00701	7.96E-06	0.418691	161.133
25.26971211	26.90894644	17.0258075	21.19517493	12.22354124
0.468443638	0.565617051	0.142952328	0.087547011	2.82165
1.79981	1.24627	0.329041	0.000188151	17.9314
45.7552	52.453481	29.718205	30.72170003	16.948971
0	1.10232	0	0	0.651003
58.05752	59.905444	52.130637	52.642592	107.7969
41.32636235	40.71321488	30.09221839	27.66557138	10.99791
31.66174	29.6801198	37.70191	38.61014799	58.35956
632.8800314	583.5530207	78.62530691	170.1520119	9.649600904
65.463456	51.23743312	18.32086346	16.16383032	1.1590915
15.02214	17.56924	34.4622	23.599888	56.1849
27.19158	27.61932	24.64164	22.149745	16.28547056
0	0	0.000817714	3.49988	1.68902
0	0	0	0	0
0	0.18844	0.183326	0.122539	12.094742
14.19604	14.08467	30.41173	36.41564	25.15318
2.473438568	1.555747005	5.765955	8.151768488	3.785056687

3.49667	3.18384	4.24433	3.0722	5.35909
2.41244879	2.29711453	1.881094444	2.282445212	1.0510839
0.0919069	0	0.130081	0.175904	0.0533324
0	0	0	0	0
0.0400031	0	0.552832171	0.21241944	0.361148
0.0403144	0	0	0	0
0	0	0	0	0
0.502875	0.189083	0.0605697	0.407341	0.29904
0	0	0	3.1062	4.58367
8.13569	7.0135	5.41517	4.48346	4.65023
49.74696	50.59438	40.50308	36.765656	11.15538
0.312792	0.0628454	0.0618163	0.163711	0.467899793
1.91095	0.463901	2.83544	0.6056	0.467899793
0	0	0	0.491125	0
12.27074	14.04579	9.85595	10.049277	27.81726
2.3161365	2.3197399	3.206589105	1.750418001	4.611196
0	0.0937977	0	0.24542	0
0	0	0	0	0
0.024748851	0.046993727	0	0.030194765	38.3805
0	0	0	0	0
12.15304	11.84070697	12.11214322	13.14890181	23.53672236
88.9532	78.73040628	62.78093814	62.0574	19.1771021
14.8958573	14.40176102	17.94468023	16.28220317	12.48697133
14.8958573	14.40176102	17.94468023	16.28220317	12.48697133
2013.17084	2219.4441	2278.43575	1703.398961	6.00279
2013.17084	2219.4441	2278.43575	1703.398961	6.83793
2013.17084	2219.4441	2278.43575	1703.398961	163.6095
1.284659209	1.054937	1.826032947	2.333878	1.982556
0	0	0.680688	2.38792	0.817215
0.667871	0.843077	0.654864	0	0
0.114917	0	1.02974	2.58216	1.342442
57.1787102	46.903	24.91415924	30.5369	3.06502952
0.266557	0.268832	0.765517	3.14042	1.576
0.76372154	0.471340952	0.960978214	5.441631	6.884884502
0.76372154	0.471340952	0.960978214	5.441631	6.884884502
0.109052	0.0732448	4.50811	11.5156	6.884884502
0	0	1.47055	4.26674	4.06448
0	0	1.644	6.02698	4.09441
0	0	2.625453	6.48798	5.960995
0.0528102	0.0107896	2.09956	6.18197	6.41165
31.6139339	32.0326413	30.39136404	27.754027	120.65146
98.82047224	95.7355	94.21962	85.0666768	120.65146
32.010286	34.68916	30.12357	28.53034	45.161771
0	0.0568806	0.387061	1.25538	1.25592

1.29385	1.57476	2.38145	1.21883	2.64963
0.308676516	0.249200321	0.243553397	1.006578649	0.152904
0	0	0	0	0
0	0	0	0.194999	0.221559
9.610767682	8.845657	10.59421116	8.153873528	29.663868
0.222599	0.226762	3.99837	8.82013	5.95957
11.76553784	10.92309645	9.378199767	7.789914804	39.8268
30.04117	31.91158	32.88985	32.364045	26.65490645
17.06037	17.68968	19.4874	16.78409	77.3116
1.60E-05	3.65901	1.224306	0.8440994	2.75E-06
1.60E-05	3.65901	1.224306	0.8440994	1.50335
0.0403752	0	0.0397543	0.0533713	0
0	0	0	0	0
0	0	0	0	0
45.03847	58.64849785	87.34405076	58.26966161	3.93846
45.03847	58.64849785	87.34405076	58.26966161	9.44631
0	0	1.41495	1.92406	7.96765
37.41382314	39.44391371	22.97290127	27.54858009	11.34599259
3.366296744	3.031181	6.4805822	6.350183845	0.998046
33.9845	12.71830594	5.012613613	4.920674532	0
29.4550845	27.58182184	25.703382	27.502464	10.82456824
126.4290538	122.0170127	127.4470424	138.0480375	102.3050118
195.5671576	185.5637287	129.5931927	135.4412494	66.04270453
0	0	0	0.0964705	0
0	0	0	0	0
0	0	0	0	0
12.79272706	12.46429097	8.436883	12.48253579	7.381765
0.492218	0.162749	0.152927	0	0
0.17351	0.117797	0.864888	0.308865	0
0.114635735	0	5.140625346	8.40087	7.05431
0	0	0	0	0
23.980108	21.65312	17.54556	15.4473	4.325980037
0	0	0	0	0
0	0	0	0	0
0	0	0.547593	0	0.329242
8.95395	10.0561	8.27113	5.0593	0.436388
0.706809	0.241244	0.22775	0.307766	0.268466
0	0	0	0	0
0	0	0	0	0
0	0	0	0	0
0	0	0.0765201	0	13.3075
0.20597	1.49E-06	0	0.0144937	12.27828
0.859488	0.000454124	0.00068726	1.12E-06	4.61E-05
14.15965	16.28506	22.62361	17.89877	62.68388

0.23671	0.119728	0	0	17.8421
0	0	0	0	0
44.7218217	41.0002879	23.0667	26.10726	22.71974
0.107896	0	0.474672	0.982307	0.849186727
44.1360055	40.502956	57.03056033	61.872279	169.3345
6.85028	4.63571	6.84049	6.16043	0.00083077
1.17604	0.372107	56.0249	128.746	192.398
5.254871	5.307392814	4.642349	4.577600062	1.440277
4.343830382	4.361931066	1.929307545	3.579930069	132.0448
0.55915935	3.743317	4.87415	7.96262	133.3992
0.55915935	3.743317	4.87415	7.96262	133.3992
4.343830382	4.361931066	1.929307545	3.579930069	69.4207
39.59314618	37.29065223	36.25417908	40.18029009	28.60241822
1.58033	1.04316	2.15128	1.92816	43.7821
5.41837	5.43133	14.17163	13.93448	14.83734
208.1072516	205.24236	206.31474	198.3851011	146.7630563
208.1072516	205.24236	206.31474	198.3851011	146.7630563
140.5121107	138.7412816	151.6221097	133.1208444	89.5839
60.5882	63.9587	20.613	25.7892	19.198
0.701827	0.985835285	0.923861	0.95868	2.933633359
1.2222	2.33266	0.147229	0.0495492	386.165
1.0628	0.352881	0.00181549	0.000383745	0.000188166
0.104195	0.130804	0.0254964	0.0342066	19.8858
0.133420269	0.379609033	0.087221122	0.069283723	42.7767
21.83244	27.91488	25.69788	29.43617	11.42552
0.679017934	0.0467158	3.527317	3.817150108	0
0.679017934	0.0467158	3.527317	3.817150108	1.52714
1.42146361	1.4026216	6.155578316	8.871703303	1.314873415
1.42146361	1.4026216	6.155578316	8.871703303	0.71835
1.42146361	1.4026216	6.155578316	8.871703303	3.323425
74.6333	87.3077	73.6192	66.9707	181.671
28.10882	22.2714	26.44191	28.31522	52.7814
19.86583	27.36779	38.82521	36.16579	158.3
2.10309	2.28689	4.0983813	3.273654995	6.87E-05
18.33602035	18.62054821	23.99567515	24.5727481	19.00072
0.0467677	1.07E-33	2.51E-09	1.04E-12	0.27365
18.49188	18.21113	20.68115	19.00265	21.65084
1.61E-159	1.11E-36	5.09E-94	1.02E-93	29.4575311
60.1592	63.2112	69.30228	76.7169	43.9592
4.4544174	4.680336664	7.24799549	6.17082238	16.1498
0	0	0	0	0
16.9042	18.6585	23.4235	22.7789	25.402
120.1305	116.6612	134.387	106.928	42.1263
120.1305	116.6612	134.387	106.928	43.9092

0	0	0	0	0
0	0	0	0	0.539850034
8.58E-19	0.471477	0	0.0712236	4.10E-05
0	0	2.99317	6.50699	6.79659
217.79	194.1042	8.7538	8.75571	13.9734
2.0017692	2.421452936	4.164130782	7.13296992	1.7647
1.64904	1.24108	1.37855	1.74011	1.92984
0.747080276	0.905552614	0.2477319	0.581812124	0.769579015
7.654536	8.85062	7.35032	7.13492	3.423935
10.15568	10.965926	13.85504	15.039871	17.82830469
12.5433	14.70939	20.65916	15.59712	93.3201
41.67632846	47.450683	43.715148	47.778109	43.64489521
6.90213856	7.9087141	9.60300968	8.7301685	3.034033246
6.90213856	7.9087141	9.60300968	8.7301685	3.034033246
3.285010921	5.2145987	3.59897927	5.20345019	54.07298
48.33362	43.10912	39.10917	32.72196	28.87386628
4.0392833	6.381328	11.6488	9.155766	19.171
31.01987	35.79206	31.87957	28.78351	44.20854
4.0392833	6.381328	11.6488	9.155766	99.5107
31.16242903	26.0120849	18.3996503	20.94305761	8.860971967
31.16242903	26.0120849	18.3996503	20.94305761	8.860971967
31.16242903	26.0120849	18.3996503	20.94305761	8.860971967
23.73499155	26.78411173	20.74408803	18.950351	28.66925
23.73499155	26.78411173	20.74408803	18.950351	30.48070835
4.09699	5.88788	13.2214	10.8109	2.61724
10.43742662	9.293012518	12.20686172	13.47150022	117.49504
0	0	0	0	0
0.43526	0.343781	3.77348	8.40141	11.8561
33.8886	51.892	10.3906	13.2821	5.41988
32.13480899	40.0195064	32.74400011	27.78275473	51.28590652
32.13480899	40.0195064	32.74400011	27.78275473	51.28590652
33.52984	30.36055	41.43531	50.78276	45.03374
148.8599	213.9272	234.674	212.72016	125.95936
7.64394	8.90757	16.9582	10.5046	34.097
19.8601	35.6805	19.3012	24.8935	103.942
9.65110746	9.667144801	6.793841841	5.517430383	1.331350622
0	0	0	0	0
0	0	0	0	0
0	0	0	0	0
0	0	0	0	0
0	0.0926049	0.271505	0.481456	11.2619
0.434255	1.12748	0.50989	1.1352	0.632124
5.878841105	5.413873484	6.2924332	6.196491298	7.402742
29.87076549	32.57883986	25.17706256	22.39463752	50.9613

13.953187	11.25171487	10.68457431	13.23139749	2.92E-06
183.91197	163.85277	24.397146	29.11268	3.6385311
43.719748	41.318815	47.066661	56.389655	30.2605
43.719748	41.318815	47.066661	56.389655	38.4353
55.7979	55.1455	61.0896	62.4217	150.1408
5.80725128	3.961978884	7.123432879	5.810091905	1.67919
3.33387	2.80869	4.24659	3.6638	1.00807
4.94369	3.97276	3.30694	1.93375	6.42554
5.1533599	6.762769577	10.09474871	8.660480597	30.01301
2.148709038	1.900925544	4.718445749	3.779471561	2.691583946
7.254101	8.172735	9.55644	8.966105	21.863354
23.86509	27.454902	33.68642027	27.36343	42.62976542
23.86509	27.454902	33.68642027	27.36343	42.62976542
2.159656	1.567480202	2.666199205	1.681290007	0.596291053
2.159656	1.567480202	2.666199205	1.681290007	0.596291053
458.2282	410.6964	575.5453	534.1469	449.5187
11.544763	12.74086099	23.309817	21.472264	58.3264
24.72269	36.67	22.2026686	21.3378	5.46579
24.72269	36.67	22.2026686	21.3378	0.631685
42.960261	42.7898018	57.25020291	55.05440043	81.3901
1.41301	2.06711	10.3517	1.56518	0.000695886
1.44688	0.727314	1.87384	1.42233	0.733332
2.46941	3.48418	6.09537	4.46353	4.64233
9.3032109	9.93994507	8.602902	8.381473937	9.490842546
9.3032109	9.93994507	8.602902	8.381473937	196.725
0	0	0	0.120876	4.51E-05
0.871937	0.000169111	1.08E-05	2.27E-05	0.000133811
0	0	0	0	0
0	0	0	0	0
0	0	0	0	0
0.0273291	0.0272934	0.0267222	0.144316	0
0	0	0	0.213561	0.189935
0	0	0	0.311656	0
1.354	1.42762	1.05421	1.85374	0.559806
1.279170379	0.961521248	0.692738012	0.385376726	0
0	0	0	0	0.105165
0.0398844	0	0.0396058	0.0535236	0.0483163
0	0	0.0315559	0	0
0	0.332421	0	0	0
78.92838	66.15051	84.2531	90.79575	43.4586
0.891491	0.853370268	1.063451699	2.367807926	0.532121
1264.940914	1256.526609	1759.509058	1641.386372	1088.439
18.6876	18.3221	22.1381	23.3192	30.9021
54.82728722	51.35877136	32.89984453	37.28603733	19.76700012

0.883544	0.217777	0.208049	0.834857	0.544125
14.91789006	13.68465593	13.59498742	13.67736212	71.37320005
6.242356965	6.434725072	10.55136094	10.12229493	28.91604
6.242356965	6.434725072	10.55136094	10.12229493	52.91944277
30.591694	26.671541	7.15716	8.095396	12.20043
106.782	96.7934	9.72075	51.4454	2.13947
26.17404	25.49158214	33.19416349	36.41782	21.08411
13.72704492	12.51138091	10.22719786	10.42269412	11.8986
8.65508	6.8814	11.8844	11.2935	5.75881
76.8949	93.3154	74.4747	73.108	78.0888
19.32915415	19.55314685	17.80356966	18.49357769	18.99463003
19.32915415	19.55314685	17.80356966	18.49357769	18.99463003
118.179494	111.8474	101.06224	105.44014	0
0	0	0	0	0
118.179494	111.8474	101.06224	105.44014	25.36914
118.179494	111.8474	101.06224	105.44014	7.19385
18.27494932	17.64608155	11.37675243	10.13981046	4.107799818
20.052	18.0822	9.57263	10.1859	17.3343
9.40259	9.35375	12.96467	8.66913	19.15484
2.98499	6.2492	8.01461	6.82165	27.3467
16.7496	0.000128765	1.95E-07	1.99E-07	22.5539
1.742851	4.65333	1.205556	3.68749	156.8617
73.0852	85.0053	84.533323	74.67761	76.1619
0	0	0	0	0
0	0	0	0.123079	0
0	0	0.251397	0	0
0.264926	0.0447088	0.0434309	0.11748	0.0522144
9.58847	14.59857	11.84302	14.88397	4.46434
0.44510973	0.770425313	0.063342747	0.340950724	0.077226
2.93021	2.59688	2.47623	1.31534	0.507178
0	0	0	0	0.263912
0.314469	0.12798	0.1806705	0.523346	11.1897208
9.91838	10.7963	11.0989	12.2574	126.469
1.636020585	1.545047576	5.182215318	3.768120776	17.92566717
1.636020585	1.545047576	5.182215318	3.768120776	17.92566717
36.2420013	32.40005964	21.63827251	25.48359726	1.151752
0	0	0	1.63789	1.12838
36.2420013	32.40005964	21.63827251	25.48359726	8.420431967
21.72139	24.6094	72.24143	74.6418	34.6008
0	0	0	0	0
286.4077961	235.27806	51.85975737	74.41609	4.35037
0.496494166	0.477552415	0.5587571	1.112107483	2.304723
16.52009	15.91372	16.460579	20.4206	4.67747
79.70864	75.35072592	67.51749347	71.1483023	3.413075

79.70864	75.35072592	67.51749347	71.1483023	16.70164116
45.54603057	47.91511	32.12653	35.92164	16.44320456
0	0	0	0	0
0	0	0	0	0
0	0	0	0	0
0	0	0	0	0.249552
0	0	0.368075	0.868954	0
0	0	0	0	0
0	0	0.103422	0	0
0.077634249	0	1.675091	2.15673846	2.077281
36.97736	43.1573904	33.383156	36.981889	31.37411412
23.71652	26.36696	23.01518	21.07843	46.78446
51.5439176	61.47522	54.96106454	50.90557079	26.0266
1.939157	2.180428959	2.813518	2.543286	49.2585
15.62347711	14.65912335	9.970696327	12.11152505	23.97856724
0	0	0	0	3.186960512
1.415190023	1.70187032	1.700170282	1.367090411	9.548470174
16.05336274	19.28075257	23.74164072	24.43840397	49.358
16.05336274	19.28075257	23.74164072	24.43840397	49.358
10.29411424	9.870259401	6.682880447	6.602171747	5.3437
0	0	0.805728	2.20804	0.733726
60.48852081	58.793556	56.82928788	59.44813912	76.40514
1.71846	1.31842	0.406633	0	0
19.14642	23.733282	18.364831	17.1963	40.73857
15.5597434	14.04005441	7.777132819	7.579686862	37.30320628
56.8018	65.0877	87.7484	92.0917	92.1913
3.90772	4.01309	9.00422	6.62812	30.91
40.5579557	40.9452548	26.14629373	25.85232929	10.83300724
0.105828407	0.171109342	0.0209112	0	41.82926
19.49666	20.20701	31.31829	27.791574	59.1964
0.2876405	0.0779529	3.592367	5.861218	3.961331
14.3315897	13.9764	8.831811065	10.66442502	3.2337125
43.91520105	41.20622732	21.42323059	20.24104376	8.498317851
1050.697	858.811	255.8066	317.733	15.0762
1.59379	1.05497	2.477	1.57523	1.1464
0	0	0	0	0
4.38815	5.21534	14.0964	13.3803	10.9611
0	0	9.66E-05	1.87E-05	1.03799
0	0	4.106928143	6.628170924	4.408352
0	0	4.106928143	6.628170924	4.408352
0	0	0	0.243561	0
1.04187328	0.104705194	14.66760231	26.96877763	20.28735221
2.513071016	1.764638098	4.094289741	7.429724847	1.16846
2.513071016	1.764638098	4.094289741	7.429724847	3.066768223

0	0	0	0	0.425225
0.4488227	0.1823851	0.9298033	1.009764222	6.020157732
0	0	0.165241	0	4.963844969
0.407603	9.61E-05	0.000126576	9.98E-05	8.38E-05
24.85898252	23.836533	18.89259785	20.62963577	6.34112476
0	0	0	0	0
18.15932	22.297367	25.85881	27.96513	31.63042315
23.69322054	23.55788437	24.75181831	22.14081488	66.7988
1.082169014	0.713436338	7.306083234	5.271802	1.584594723
0.303969	0.131722	0.128593	0.229616	0.10439
0.500046	0.503471	0.982162	0.872359	0.731358
0	0.158218	0.152665	0.205268	0.356848
0.0957585	0	0	0.492434	0
0.305544	0	0.0981695	0.53073	0
0	0.100308	3.203284085	7.17578403	3.582506508
0.405811	0.825887	2.81856	1.07644	1.21519
21.09054003	19.58325111	51.92912512	45.795175	44.37878075
213.807102	205.04783	15.4985268	43.003696	0
23.440651	26.75403371	11.33451106	11.14715239	5.142183
4.12618	4.12649	1.13982	1.55934	0.27694
5.367813541	6.257633358	9.013370114	7.701614941	8.032730344
52.78071208	43.63588415	402.5766001	727.1864	217.1119
52.78071208	43.63588415	402.5766001	727.1864	2.02494
0.646782057	0.660814465	1.429483	0.434179	19.29308722
1.54359	0	33.3523	118.863	2993.03
18.8107937	26.71537433	39.96841715	42.56043	17.7992
0	0.618767	0	0	0.25362
3.35184257	4.87508262	6.43947609	9.661725757	383.8854
2.170320137	0.898893633	3.386690605	3.460440435	2.645960652
0	0	0	0.350795	6.149
5.91175362	12.9570632	0.080339	0.322332519	2397.3076
4.381083	4.78397	9.154210005	7.72355	5.859295567
4.381083	4.78397	9.154210005	7.72355	5.859295567
31.16485991	29.5647011	15.98608886	19.54479791	8.425080938
31.16485991	29.5647011	15.98608886	19.54479791	8.425080938
31.16485991	29.5647011	15.98608886	19.54479791	8.425080938
0.040781141	0.5778993	0	0.054654883	38.15146
559.383	483.226	439.346	526.885	190.423
3.666913244	3.39097	0.493215165	1.4394	0.211952
0	0	0	0	0
0.305183373	0.923029442	1.35726601	1.307317	0.169548
0.147518	0	0.287915	0	0.18132
54.3906	49.2565	98.8692	68.676	98.254
0	3.38E-05	0.832281	3.13942	1.5692

7.554223352	6.380573	2.78082579	3.457558962	0.910066424
20.1300578	19.65212373	11.78095878	10.8923279	2.420767
20.1300578	19.65212373	11.78095878	10.8923279	2.420767
24.76788	22.10464	19.04802	21.98571	5.577382
18.5291	18.0817	65.416	53.1271	10.684
0	0	0	0	0
19.92513	19.651	11.95178	11.96343	2.876706
13.8798	10.1003	7.14081	5.53316	1.3899
39.78079362	35.48326	24.50491537	25.5920956	6.94771
46.5596	40.00284153	47.41033	41.5184	74.47700607
46.5596	40.00284153	47.41033	41.5184	74.47700607
46.5596	40.00284153	47.41033	41.5184	74.47700607
46.5596	40.00284153	47.41033	41.5184	74.47700607
1.96057	0.947076	2.40261	4.58639	3.67722
0	1.10408	0.479138	2.02869	1.30116
4.587177128	4.493890617	12.0469029	7.568614009	1.45919
0.308006	0	0.594825	0.20186	0
0	0.144914	0	0	0.175041
736.359	754.718	65.6283	170.487	19.4255
3.87527	16.8365	13.6606	71.4175	66.7489
137.03217	185.955596	265.997501	899.724566	115.362
0.160705115	0.054227926	0.691651	1.091472	0.366249
66.1604	55.8681	58.55270045	67.90910187	35.06790047
6.94064757	6.820813078	8.696198	7.51154523	6.265640325
3.112145837	2.749488625	7.19162955	7.664778238	10.15511511
2.55E-06	1.293691	3.3722	5.959025078	32.8046
1.84217922	2.073926808	8.57204174	8.435316749	3.849316416
1.84217922	2.073926808	8.57204174	8.435316749	3.849316416
0.330844418	0.318321735	1.399121046	1.177592636	0.713980408
1.935699251	2.525905236	0.917258385	0.851972202	0.466367
1.680607311	1.968587264	1.768011731	3.151046	9.177622709
1.680607311	1.968587264	1.768011731	3.151046	9.177622709
4.453682739	5.202557376	7.241430122	5.73528339	4.166599016
4.83187	5.40478	8.205774	6.317287601	3.935975
0	0	0	0	0
0	0	0	0	0
12.83246	13.60959	6.0871	7.4161	0.234834
9.724079627	12.127288	5.912339	9.282330719	18.60678837
9.724079627	12.127288	5.912339	9.282330719	18.60678837
9.724079627	12.127288	5.912339	9.282330719	18.60678837
9.668542	11.335405	14.494631	8.6719	13.4989
0	0	2.10352	2.74787	2.63493
10.138776	11.03852	15.44577	12.863338	25.555477
13.05343	12.81175	10.23518	9.94007	3.626808

5.15114	5.041645	8.391212	11.484584	7.61899
5.15114	5.041645	8.391212	11.484584	17.9736
0	0	0.341724	0	0
0.0774395	0	0.0510122	0	0
0	0.492691	0.771465	0.424982	0.376966
0	0	2.58145	26.7317	455.514
0	0	14.3151	22.9489	169.109
0	0	11.4745	50.9856	465.873
13.1959	8.43202	13.6447	12.6916	26.5002
0	0	0	0	0.0911964
247.062153	256.4670042	443.489894	397.1130049	1035.399
14.01835	14.31945	16.863294	16.209988	19.4916
14.01835	14.31945	16.863294	16.209988	20.8462
1.07994	1.00872	1.35182	1.5524	0.181322
9.01014	5.7152	16.3281	11.4912	26.2112001
0	0	0	0	2.62331
0	0	0	0	0.102403
0	0.127118	0.122339	0.0832648	0.073734
0.176245	0	0.432584	0	0.107641
1.028278988	0.923184	1.279340025	0.646513201	0.171914
0.129744	0.197741	0.578463	0.25985	0
28.67597008	28.417814	36.010077	38.42047558	13.42856276
28.67597008	28.417814	36.010077	38.42047558	13.42856276
0	0	0	0	0
0	0	0	0	0
0.0719886	0	0	0.0961394	0
0	0	0.225898	0	0
0.597984	0.710976	0.197105	0.930384	0
0	0	0	0	0
0	0	0	0.407402	0
0	0.176874	0.516369	0.231267	0.22219
0	0	0	0	0
71.9951603	67.57436199	48.22764003	50.19425201	6.00914
0	0.103842	0.0506622	0.206642	0.122338
0	0	0	0	0
0.11021	0.798494	0.166129	0.609564	0.169335
0	0	1.47399	4.22001	1.42043
1.16E-05	0.372055	0.196818	0	0.000553123
0	0	0	0	0
0	0	0.618866	1.7508	0
0	0	0	0	0
7.17772	8.33733	12.7396	9.97888	28.1054
1.10158	0	1.87486	2.51203	0.643434
0.145187767	0	0.610818751	0.825856	2.027446493

0.145187767	0	0.610818751	0.825856	2.027446493
133.9430323	136.7226853	108.5331032	101.952569	231.1950515
133.9430323	136.7226853	108.5331032	101.952569	231.1950515
133.9430323	136.7226853	108.5331032	101.952569	16.61236
9.62132	9.06616	11.2284	11.1218	15.1189
822.438	812.871	139.7599	522.6769	35.6887
1.97636	2.09918	1.79044	2.42769	13.680213
19.0421	9.21064	36.7615	34.3788	26.556
14.667	18.8376	31.128	27.6255	57.1023
362.4228703	360.5463564	319.2448223	306.2257536	4.80652
362.4228703	360.5463564	319.2448223	306.2257536	12.3711
362.4228703	360.5463564	319.2448223	306.2257536	38.78330016
8.25962	8.30069	11.0842	15.5605	17.3194
5.78911	5.2543	2.99148	6.75019	5.66467
4.077406	3.599350953	6.646935932	8.406738	11.38505
24.40761429	32.29312796	39.83575563	29.08647026	11.58112
24.40761429	32.29312796	39.83575563	29.08647026	49.0842
2.85E-06	5.50E-06	0.646149	1.67332	0.000549016
0.118925	0.059127	0.0574001	0.076342	0.138646
0	0.497755	0	0	0.585618
7.90701768	4.777417602	11.995271	11.08119515	39.13390397
7.90701768	4.777417602	11.995271	11.08119515	39.13390397
1.380464315	1.020825019	2.462527592	2.916925863	35.12002
11.9678	16.40080014	23.65609	30.0468	0.000885693
0.177175369	0.064070301	2.872507	4.98338	2.856854
138.934	122.5703	10.85158	33.47924	1.560117534
34.62737	31.123414	30.171662	27.102894	9.59552
0.291754	0.2570511	2.231398	1.728118	0.803980636
6.56899	9.65264	7.90556	6.78826	2.84621
0	0	0	0	0
2.08198	2.87578	0.741724	3.0296	0
0	0	0	0	0
0.101841	0.305557	0.0986578	0	0
0	0	0	0	0.436695
3.26743	1.79651	2.34029	5.92E-06	0.00663594
21.06072948	15.51900959	5.858646902	7.947089008	1.464554472
0.111528157	0	1.671572471	3.747654907	2.218744678
0.111528157	0	1.671572471	3.747654907	2.218744678
2.67627	0	3.10642	0	0
43.51693	46.92640574	40.8397981	40.42957582	62.95202
115.76653	87.02054	53.79912	45.83558	45.26572
2.62E-09	4.69E-07	4.34E-05	7.20E-05	7.03E-08
0	0	0	0.30407	0
56.1137	28.2543	102.195	77.8497	5.16686

0.982178	0.833793	2.02559	2.11625	0.281287
67.84700003	76.99084009	74.87454439	78.51688	74.45706
67.84700003	76.99084009	74.87454439	78.51688	74.45706
77.57916813	70.55235541	76.55882682	60.84011421	53.00720304
338.5526267	420.67099	258.6765601	239.9578245	489.2220266
0.7356358	2.05447	5.79695	3.105188	1.24127
23.72862933	19.21858636	28.73728084	24.37530259	18.21727
206.78639	218.72391	27.53513467	39.55548753	21.02391
26.8083	28.2281	33.8992	26.4899	56.7636
0.777957	7.42922	2.94844	1.65428	65.3916
8.921060001	5.10410557	3.55774	4.18332	27.7146
25.0155	24.6324	40.2205	36.7084	28.5386
73.67552	67.466779	73.68030103	78.965137	67.95750305
73.67552	67.466779	73.68030103	78.965137	67.95750305
73.67552	67.466779	73.68030103	78.965137	31.6249
22.6371	26.723	36.5626	34.1662	93.9706
0	0	0	0	0
0	0	0	0	0
1.46141144	1.366720281	3.666072854	3.126453559	1.816608757
1.46141144	1.366720281	3.666072854	3.126453559	1.816608757
33.32866551	32.419657	37.78687051	46.34377468	69.487913
33.32866551	32.419657	37.78687051	46.34377468	38.57742604
743.262	286.2133	718.058	785.912	16.60599
0	0	0	0	0.241532
0	0	0	0	0
0	0	0	0	1.28389
0	0	0	0	0.506367
0	0	0	1.10041	0
0	0	1.10423	0	0
0.260604	0	0.0835679	0.332894	4.42E-05
0	0	0	0	0.100548
0.733879	1.30577	5.04123	7.75793	257.2805
0.720618	0.364687	2.07643	3.09959	21.3619
3.16727	1.749993788	9.96829	10.485912	454.837
0	0	0	0	0.990204
0	0	0.171755	0.460997	0
0	0	0	0	0.397391
0	0	0.139374	0	0
0	0	0	0	0
0	0	0	0	0
0	0	0	0	0
0	0	0	0	0
0	0	0	0	0
0	0.0757917	0	0	0.0925303

0	0.119072	0	0.309628	0
0	0.142309	0	0	0
0	0	0	0	0
0.210309	0.214573	0.125632	1.01174	0.255779
78.95260912	74.56064039	61.65656	52.35171052	13.38113234
0.231481	0.0591946	5.36815	11.5878	18.5512
0	0	0	0	0
0	0	0	0	0
0	0	0	0	0
0	0	0.533382	0.945862	6.19616
4.593126	5.114732916	5.34894235	6.540653	9.086147124
3.577748095	6.10638	19.61759	36.05789	14.7009
8.9601408	11.53900714	3.14866	4.447613651	2.88772158
4.13893483	5.453666417	10.90514797	12.70177475	45.15953567
4.13893483	5.453666417	10.90514797	12.70177475	45.15953567
0	3.01267	0	0	8.175120658
6.42928017	5.95839674	6.529280011	7.003818	14.22394343
0.744814	0	0.368636	0.740378	1.54491
40.52024242	36.69976706	27.3224791	27.72688161	4.474061
40.52024242	36.69976706	27.3224791	27.72688161	4.474061
40.52024242	36.69976706	27.3224791	27.72688161	1.539981407
70.9567	72.7691	45.89382723	54.4831	87.81370865
70.9567	72.7691	45.89382723	54.4831	488.1835
40.39718	40.30137	25.9656547	26.88940298	3.69698
0.544292	0.748847	0.435248	1.75E-06	0.99442
8.466015334	10.3048076	1.210024979	2.3271418	0.395839
2.553085001	2.095167424	1.651571667	2.131731674	2.144889
0.597285632	0.357027297	1.486261426	3.47737494	0.554959
42.6762	39.409	42.8325	37.9213	20.2204
1.99965	1.11004	2.86178	2.14814	1.15695
34.91911	43.21105	45.36453	45.19223	96.631
0.077587697	0	4.912091	4.97193	4.964800015
0.077587697	0	4.912091	4.97193	4.964800015
0.590492	0	5.28107	8.38096	7.39673
25.8142538	27.3236	29.24516	20.812911	7.69584
25.8142538	27.3236	29.24516	20.812911	70.6415
22.55148	16.747681	21.97885	19.52213	70.6415
22.55148	16.747681	21.97885	19.52213	6.87263
3.133591	3.159233358	5.315731	7.13601363	17.07465
57.91856	58.42653	54.73096	58.27497	56.24502
17.2722	10.7374	48.30530001	44.7001	56.001225
6.00066	5.65924	8.17655	9.21194	29.4684
9.39001	8.32206	2.266029	1.842012	0.0646383
23.84968661	27.56010885	33.40806001	25.12971382	36.6827272

29.619927	29.90777	30.410032	29.2661	32.94450041
0.721174	0.245498	1.61845	3.52823	0.848463
10.61254594	10.7958385	6.933327412	8.456147036	333.4936
93.312636	98.70078627	135.114461	89.012751	15.594
26.50607	25.44601	15.557157	14.90031904	24.09324754
26.50607	25.44601	15.557157	14.90031904	24.09324754
7.674241	7.853218	9.37977	1.4871	18.99068
7.634372	7.281036721	1.738741	2.057275055	0.127398
14.983109	5.726193665	7.437266	6.286665541	17.036709
14.983109	5.726193665	7.437266	6.286665541	17.036709
36.444397	34.776661	28.563159	29.069107	19.957426
0.0297148	0.0197256	0.0336261	0.154612	0.0599785
0.0227388	0.0226849	0.0441604	0	0
0.0185888	0	0.0914171	0.244505	0.0413961
0.594674	0.4212418	0.899404	1.839557	0.173128
0.594674	0.4212418	0.899404	1.839557	0.601428
23.73417359	26.20968134	28.05900651	28.27200888	36.62141892
23.73417359	26.20968134	28.05900651	28.27200888	36.62141892
26.362857	28.38227787	27.09443953	29.87827707	9.57066
17.5095	20.9654	24.0796	21.7792	38.2615
20.696198	22.073484	29.77336659	28.0547309	68.49055
184.562	197.798	186.475	142.777	103.437
9.41753	9.94812	13.615655	10.912662	22.71585675
1123.67761	1206.36333	2002.48146	2022.8241	1557.94785
16.740338	18.41472027	20.49860621	16.73477156	33.25823818
16.740338	18.41472027	20.49860621	16.73477156	33.25823818
16.61129	15.56994	23.14095	20.26081	31.19777
42.72637	42.72203	31.45387	29.72346	37.74461957
8.233966	0.865156	11.42036	10.80809	14.608
1.36952	1.6167	2.63465	3.5417	4.58319
48.478185	47.30709	26.80292	27.115	15.52351
48.478185	47.30709	26.80292	27.115	15.52351
7.23561	8.81261	12.0154	10.4943	49.0686
58.46309	60.65402	144.6289	141.25387	83.06599
3.19184	2.086123062	4.59858	4.7231	62.70609
2.194258899	2.237630607	4.256391623	5.450725765	44.37650332
9.043157093	9.761568233	7.951783	7.977810706	5.718289129
20.937161	18.180849	14.79657101	15.93029	1.54928
20.937161	18.180849	14.79657101	15.93029	3.51154
2.545688	4.35701	4.80865	5.74592	15.76385
0	0	0	0	0.107237
0	0	0	0	0
7.551348057	5.9785189	2.782970213	4.5573109	0.792018577
7.551348057	5.9785189	2.782970213	4.5573109	2.717634587

101.289	105.907	66.9062	70.5649	86.8844
225.4884977	228.0211766	74.38855979	94.722216	56.16749
225.4884977	228.0211766	74.38855979	94.722216	1.805782
45.296216	44.039454	63.39808	50.39301	91.2391
2.677329918	2.551376693	6.895853773	5.414038478	3.98385
35.484101	34.075082	20.8352005	22.2207003	1.37906
2.38133	2.65887	2.25903	2.88202	7.84922
2.38133	2.65887	2.25903	2.88202	16.797
0	0	0	0	0
69.64	50.3149	22.8862	27.4793	0.970667
0.474677	0.358492	0.115098	0.155795	0.146953
125.9854287	129.3170513	117.150304	121.5965328	12.23311208
125.9854287	129.3170513	117.150304	121.5965328	12.23311208
125.9854287	129.3170513	117.150304	121.5965328	12.23311208
0.102327	0.101199	0	0	0.366645
0	0	0.50683	2.02216	1.41146
5.96E-05	0.259627	6.69E-06	0.0650413	17.075
33.5117122	31.4630039	38.630141	32.026782	10.4035
4.277855436	4.048336064	3.042158907	3.769126	3.30806
31.5306	40.7062	54.8782	32.4489	1.29199
3.52261	4.82149	7.6962	6.12943	16.13876948
25.89261006	23.72863001	21.06594058	22.50316	16.13876948
25.89261006	23.72863001	21.06594058	22.50316	16.13876948
21.48735	22.17247	22.45476	19.49732	56.24150731
55.3003	60.2584	43.5071	47.3636	34.0333
238.4270168	265.6554152	193.4795083	146.8611264	210.383
238.4270168	265.6554152	193.4795083	146.8611264	18.62121
0.313376271	0.056310746	1.62608341	2.65692932	0.609653
14.86882789	12.56481086	1.726278	5.265464448	1.184847
203.4104779	174.0780684	106.272257	117.688015	21.11450175
203.4104779	174.0780684	106.272257	117.688015	2.31195
8.536557	7.102421	35.04608663	40.7494233	60.9776
6.051691	6.034533	13.85014617	10.9076176	84.6545
20.94483239	22.725268	29.46171543	26.41780188	40.47239
4.75909	8.08378	6.19631	6.78956	8.55931
0.241179	0	0	0	0
0	0	0.0993534	0.269531	0
16.3353	16.2267	10.0754	10.5785	7.21886
35.42045	36.39296712	31.039574	29.19527717	8.719852038
47.48981	40.79304	27.64211	28.368781	6.764530522
16.0172676	17.7603021	16.644909	16.209746	12.311761
0	0.101035	0	0	0.124777
10.8714604	11.54746071	14.03649148	13.87864823	28.815809
151.6663269	177.0357507	276.3579291	234.73132	133.2538

78.09239	82.01905	81.87745	59.477364	145.4674
0.5609737	0.185333029	6.591300019	11.850061	6.49925
0.799641902	0.901792664	1.060537628	0.905602623	34.670231
90.05777526	85.40444722	69.0432012	78.37634066	77.21990535
12.64584269	11.81403741	8.6913171	10.74289674	7.33244
29.2338399	33.76698	5.279632575	10.67627641	0.79093839
29.2338399	33.76698	5.279632575	10.67627641	0.79093839
0	0	0	0	0
0.16741	0.0861618	0	0.281342	0
0	0	0	0	1.37297
0	0	0	0	0.896772
4.891490356	5.07627	4.088154	3.925006483	1.702984709
0	0	0	0	0
0.533346	0.160789	1.63493	3.21172	2.74793
24.21881144	21.2333	21.16172	20.50626	0.773973
24.21881144	21.2333	21.16172	20.50626	8.40227578
22.97354134	22.58181664	16.69456494	18.42376294	3.529847614
70.66840087	70.15194749	63.23788932	62.3833315	13.21021023
0	0	0	0	0
181.6139	194.8425	526.144	543.948	829.989
3.965897546	3.345905	6.5927381	3.2401554	18.1541
0.0568566	0	0	0	0
4.074410031	3.866824408	9.2473108	11.23614569	4.042994
11.619862	11.36646301	12.38322502	10.76552406	6.624156
49.6261	45.086	100.5298	122.8309	40.50324
11.98157824	12.3079539	12.67170575	12.57970691	25.34333037
0.345102	0.116744	0.555396	0	0
0	0	0	0	0.544514
23.207808	24.71215	24.000201	26.36417	30.367843
9.111019	8.144613842	5.417776015	6.362566261	0.895864
0	0	0	0	0
45.987133	43.492718	61.886568	55.68305	70.8832
10.16398	10.93204	14.218075	18.29915	47.87656
9.98E-21	0.543175	1.10E-23	1.79E-58	47.87656
226.56923	325.6839	236.53181	187.0317556	386.6157198
66.85408647	57.17319768	80.76988876	88.23275	2.76483
66.85408647	57.17319768	80.76988876	88.23275	83.21673093
66.85408647	57.17319768	80.76988876	88.23275	83.21673093
37.35822207	33.45184113	27.62970787	29.22284979	11.902
37.27819	37.44827	26.7896	25.29882	41.03044
13.71178	13.66164	8.62521	7.04408	3.02639
0	0	0	0	0
34.279574	32.77047	37.914874	40.853118	43.568207
5.98654091	7.790851	18.20456	18.57408	8.46234

18.80155	18.18562	13.20871	14.10872	18.972822
29.96431255	31.5417729	23.76148164	27.198312	22.45306906
29.96431255	31.5417729	23.76148164	27.198312	22.45306906
29.96431255	31.5417729	23.76148164	27.198312	22.45306906
15.678393	15.600304	9.25228	8.862291453	4.028161825
15.678393	15.600304	9.25228	8.862291453	4.028161825
3.930906	4.15166	4.39037	4.58905	24.5124
40.6381	34.83012	50.1136	53.96086	119.5812957
0.421856	1.40577	0.3025	0.305611	184.342
29.298306	28.910209	50.690523	48.158794	15.85157686
12.98538	11.78691	16.073418	13.942432	29.55182068
68.18097	69.84668	58.4492	53.2595	49.811292
698.4002825	697.9343287	620.6970511	607.656636	225.9073
0	0.0589468	0.40452	0.778207	0.282053
31.088848	36.15615	44.59809	40.00497	86.0476392
32.57610442	31.68770712	26.42310774	27.61571221	56.68286
11.832723	14.168987	13.1469724	12.38703346	40.2158579
78.3563	45.0408	26.5759	43.6582	1.80876
264.265	247.174	341.535	293.929	174.858
0	0	0	0	0.122585
24.20174855	24.0483902	22.14107041	25.222713	56.30565431
19.08974	17.26502927	25.76653	22.78810803	36.40732
26.93577901	27.68438662	26.42652979	23.8979569	17.17066
33.3943397	33.89772769	32.15024647	32.71675112	28.75226237
96.70737378	78.37695205	120.4691727	126.0416433	125.56304
37.02088	36.62064	32.20614	30.15096	15.65792
14.343778	15.51182	11.82065	12.4255	14.027861
14.71814306	18.43814045	15.579506	15.905194	86.85544195
14.71814306	18.43814045	15.579506	15.905194	24.9769
14.71814306	18.43814045	15.579506	15.905194	10.6794
16.77324026	18.4803794	28.64300607	29.77803111	54.69972
1.5263781	1.506918436	2.845508438	3.465752741	23.37043
1.5263781	1.506918436	2.845508438	3.465752741	0
19.57253989	18.27022905	28.48920242	26.791068	73.8821
0	0	0	0	0
0	0	0	0	0
0.0403433	0.338253	0.475004	0.346848	5.56135
3.2123	3.70473	5.91796	5.91087	9.71965
0.911714	0.186165	1.58824	1.67362	6.62183
8.243453222	4.773606927	1.42253	1.335972133	0.790603
8.243453222	4.773606927	1.42253	1.335972133	0.790603
14.47894184	16.71119229	19.05301807	20.42113082	33.80994526
31.157	32.510311	30.461079	29.3207	22.93451194
6.639767	7.314702	6.717376	9.106127	3.033160401

5.81169	6.07642	10.3958	7.81484	13.52154
40.81945212	47.88106123	39.92861	37.17186003	35.47433273
40.81945212	47.88106123	39.92861	37.17186003	35.47433273
81.60109	87.04499	136.735783	146.8728767	116.40151
33.75386	37.3858944	46.91011492	41.7997106	88.36559144
33.75386	37.3858944	46.91011492	41.7997106	29.6345
22.35020735	22.63285341	22.47506691	27.27546	31.10285368
22.35020735	22.63285341	22.47506691	27.27546	31.10285368
21.956827	23.841437	41.87284	21.607798	5.1073013
0	0	1.09985	0	0
65.8453	68.888644	60.3847	54.8546	67.83843908
0.0139749	0.00697078	0.0406549	0.0727191	4.959960001
1.62879	1.17872	1.87068	2.24001	4.959960001
39.69159	39.25193	43.04921	40.55924	35.39021
11.9788	11.2606	14.5023	14.0695	9.653300013
0	0	0	0	0
1.667739	1.256097	3.373579	2.26536	19.4543
8.037744	7.48256	13.172243	14.27166541	78.1293
0.355296	0.700001	0	0	0.8733
15.8536	16.10357	28.489	31.4242	73.813
80.788559	64.722266	48.51675	56.80681947	17.477608
894.62701	945.86387	767.76609	558.95974	198.7493231
0	0	0	0	0
3.36554	3.4023	0.355596	1.95299	0.921145
20.36935	27.05242	18.37694	14.11925	55.5239
20.36935	27.05242	18.37694	14.11925	2.51489
1.926751542	1.019395254	1.587438413	2.30539893	46.56163893
16.65011942	19.323259	19.07712	17.091787	27.5032
145.5876	140.74896	51.13655	70.12236	21.112075
2.21846	0.00213235	3.97761	1.78962	40.4892
47.04269	45.729444	45.8119637	49.18863	33.999041
47.04269	45.729444	45.8119637	49.18863	33.999041
17.46088508	21.8927985	3.938681572	5.508553752	3.1071552
18.76874	19.17356	18.23976	16.521949	21.34961
8.8924	10.276486	6.69546	7.627791	17.54112001
16.92207922	17.65998546	17.99980196	19.77946446	53.107325
26.09709	29.68823	25.81983	26.92051	43.3055
0	0	0	0	0
63.3706	68.3193	58.7435	53.4315	88.21550919
3.621317079	3.920843839	5.544203064	5.770598598	3.31671
0	0	0.0470394	0	0
45.67608	43.83277	31.051515	36.67824	12.80383001
61.77075	69.94808	23.087042	19.914692	4.161543013
61.77075	69.94808	23.087042	19.914692	3.271222926

0.0891375	0.616827	0.469374	0.743593823	0
22.88575	20.68759	36.4879	28.3161	96.3365
18.39831928	17.82676103	43.3133515	35.16393623	8.508134
18.39831928	17.82676103	43.3133515	35.16393623	13.22403011
18.39831928	17.82676103	43.3133515	35.16393623	13.22403011
18.39831928	17.82676103	43.3133515	35.16393623	0.821904
19.46886315	21.67762814	17.82951173	17.81771402	25.23552456
82.29374	98.390559	34.568305	44.19524	22.84232123
82.29374	98.390559	34.568305	44.19524	22.84232123
3.6835	4.190873	4.172827	4.692262	3.792473
17.21145152	15.213789	20.363059	18.76590808	2.95E-05
17.21145152	15.213789	20.363059	18.76590808	51.8265
4.097073709	5.19173203	8.007415006	6.74460863	27.71571
31.73148903	33.53103344	28.94970104	31.37087428	33.04091928
[truncated: 262,001 more chars]
